# Multi-omics of the gut microbial ecosystem in inflammatory bowel diseases

**DOI:** 10.1038/s41586-019-1237-9

**Published:** 2019-05-29

**Authors:** Jason Lloyd-Price, Cesar Arze, Ashwin N. Ananthakrishnan, Melanie Schirmer, Julian Avila-Pacheco, Tiffany W. Poon, Elizabeth Andrews, Nadim J. Ajami, Kevin S. Bonham, Colin J. Brislawn, David Casero, Holly Courtney, Antonio Gonzalez, Thomas G. Graeber, A. Brantley Hall, Kathleen Lake, Carol J. Landers, Himel Mallick, Damian R. Plichta, Mahadev Prasad, Gholamali Rahnavard, Jenny Sauk, Dmitry Shungin, Yoshiki Vázquez-Baeza, Richard A. White, IBDMDB Investigators, Jason Bishai, Kevin Bullock, Amy Deik, Courtney Dennis, Jess L. Kaplan, Hamed Khalili, Lauren J. McIver, Christopher J. Moran, Long Nguyen, Kerry A. Pierce, Randall Schwager, Alexandra Sirota-Madi, Betsy W. Stevens, William Tan, Johanna J. ten Hoeve, George Weingart, Robin G. Wilson, Vijay Yajnik, Jonathan Braun, Lee A. Denson, Janet K. Jansson, Rob Knight, Subra Kugathasan, Dermot P. B. McGovern, Joseph F. Petrosino, Thaddeus S. Stappenbeck, Harland S. Winter, Clary B. Clish, Eric A. Franzosa, Hera Vlamakis, Ramnik J. Xavier, Curtis Huttenhower

**Affiliations:** 1 Infectious Disease and Microbiome Program, Broad Institute of MIT and Harvard, Cambridge, MA USA; 2 Department of Biostatistics, Harvard T. H. Chan School of Public Health, Boston, MA USA; 3 Gastroenterology, Massachusetts General Hospital, Boston, MA USA; 4 Metabolomics Platform, Broad Institute of MIT and Harvard, Cambridge, MA USA; 5 Molecular Virology and Microbiology, Baylor College of Medicine, Houston, TX USA; 6 Earth and Biological Sciences Directorate, Pacific Northwest National Lab, Richland, WA USA; 7 Department of Pathology and Laboratory Medicine, David Geffen School of Medicine, University of California Los Angeles, Los Angeles, CA USA; 8 Department of Pediatrics, University of California San Diego, La Jolla, CA USA; 9 Molecular and Medical Pharmacology, University of California Los Angeles, Los Angeles, CA USA; 10 Department of Pediatrics, Cincinnati Children’s Hospital Medical Center, Cincinnati, OH USA; 11 F. Widjaja Foundation Inflammatory Bowel and Immunobiology Research Institute, Cedars-Sinai Medical Center, Los Angeles, CA USA; 12 Department of Pediatrics, Emory University, Atlanta, GA USA; 13 Vatche and Tamar Manoukian Division of Digestive Diseases, University of California Los Angeles, Los Angeles, CA USA; 14 Department of Odontology, Umeå University, Umeå, Sweden; 15 Jacobs School of Engineering, University of California San Diego, La Jolla, CA USA; 16 Center for Microbiome Innovation, University of California San Diego, La Jolla, CA USA; 18 Department of Pediatrics, University of Cincinnati College of Medicine, Cincinnati, OH USA; 19 Department of Computer Science and Engineering, University of California San Diego, La Jolla, CA USA; 20 Department of Pathology & Immunology, Washington University, St. Louis, MO USA; 21 Department of Pediatrics, MassGeneral Hospital for Children, Boston, MA USA; 22 Department of Pediatrics, Harvard Medical School, Boston, MA USA; 23 Center for Microbiome Informatics and Therapeutics, Massachusetts Institute of Technology, Cambridge, MA USA

**Keywords:** Ulcerative colitis, Microbiome, Systems analysis, Crohn's disease

## Abstract

Inflammatory bowel diseases, which include Crohn’s disease and ulcerative colitis, affect several million individuals worldwide. Crohn’s disease and ulcerative colitis are complex diseases that are heterogeneous at the clinical, immunological, molecular, genetic, and microbial levels. Individual contributing factors have been the focus of extensive research. As part of the Integrative Human Microbiome Project (HMP2 or iHMP), we followed 132 subjects for one year each to generate integrated longitudinal molecular profiles of host and microbial activity during disease (up to 24 time points each; in total 2,965 stool, biopsy, and blood specimens). Here we present the results, which provide a comprehensive view of functional dysbiosis in the gut microbiome during inflammatory bowel disease activity. We demonstrate a characteristic increase in facultative anaerobes at the expense of obligate anaerobes, as well as molecular disruptions in microbial transcription (for example, among clostridia), metabolite pools (acylcarnitines, bile acids, and short-chain fatty acids), and levels of antibodies in host serum. Periods of disease activity were also marked by increases in temporal variability, with characteristic taxonomic, functional, and biochemical shifts. Finally, integrative analysis identified microbial, biochemical, and host factors central to this dysregulation. The study’s infrastructure resources, results, and data, which are available through the Inflammatory Bowel Disease Multi’omics Database (http://ibdmdb.org), provide the most comprehensive description to date of host and microbial activities in inflammatory bowel diseases.

## Abstract (web summary)

The Inflammatory Bowel Disease Multi’omics Database includes longitudinal data encompassing a multitude of analyses of stool, blood and biopsies of more than 100 individuals, and provides a comprehensive description of host and microbial activities in inflammatory bowel diseases.

## Main

Inflammatory bowel diseases (IBD) affect more than 3.5 million people, and their incidence is increasing worldwide^1^. These diseases, the most prevalent forms of which are Crohn’s disease (CD) and ulcerative colitis (UC), are characterized by debilitating and chronic relapsing and remitting inflammation of the gastrointestinal tract (for CD) or the colon (in UC). These conditions result from a complex interplay among host^2,3^, microbial^4–6^, and environmental^7^ factors. Drivers of IBD in the human genome include more than 200 risk variants, many of which are responsible for host–microbe interactions^3^. Common changes in the gut microbiome in individuals with IBD include an increase in facultative anaerobes, including *Escherichia coli*^8^, and a decrease in obligately anaerobic producers of short-chain fatty acids (SCFAs)^4^. Here, to support a systems-level understanding of the aetiology of the IBD-associated gut microbiome that goes beyond previously reported metagenomic profiles, we introduce the IBDMDB, as part of the Integrative Human Microbiome Project.

**Fig. 1:**
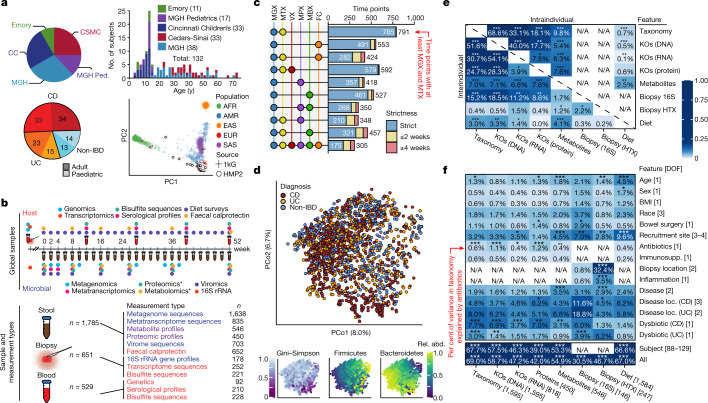
Multi-omics of the IBD microbiome in the IBDMDB study. **a**, Overview of cohort characteristics. We followed 132 participants (with CD, with UC, or without IBD (control)) for one year each. Principal component analysis (PCA) of SNP profiles shows that the resulting IBDMDB cohort is mostly of European ancestry as compared to the 1000 Genomes (1kG) reference (see Methods). **b**, Sampling strategy. The study yielded host and microbial data from colon biopsy (baseline), blood (approximately quarterly), and stool (every two weeks), assessing global time points for all subjects and dense time courses for a subset. Raw, non-quality-controlled sample counts are shown. **c**, Overlap of multi-omic measurements from the same sample (strict) or from near-concordant time points (with differences of up to 2 or 4 weeks; see Methods). **d**, Principal coordinates analysis (PCoA) based on species-level Bray–Curtis dissimilarity; most variation is driven by a tradeoff between phylum Bacteroidetes versus Firmicutes. Samples from individuals with IBD (CD in particular) had weakly lower Gini–Simpson alpha diversity (Wald test *P* = 0.26 and 0.014 for UC and CD compared with non-IBD, respectively). **e**, Mantel tests quantifying variance explained (square of Mantel statistic) between measurement type pairs, with differences across subjects (inter-individual) or within subjects over time (intra-individual; see Methods); results show tight coupling across measurement types. Sample sizes in **f**. **f**, PERMANOVA shows that inter-individual variation is largest for all measurement types, with even relatively large effects (for example, antibiotics or IBD phenotype) capturing less variation (see Methods). Stratified tests (CD/UC) consider only samples within the indicated phenotype (note that sample counts decrease for these, resulting in larger expected covariation by chance). Stars show FDR-corrected statistical significance (FDR *P ≤ 0.05, **P ≤ 0.01, ***P ≤ 0.001). Variance is estimated for each feature independently (Methods). ‘All’ refers to a model with all metadata. Total *n* for each measurement type is shown in square brackets, distributed across up to 132 subjects (Extended Data Fig. 1a, see Methods).

**Extended Data Fig. 1:**
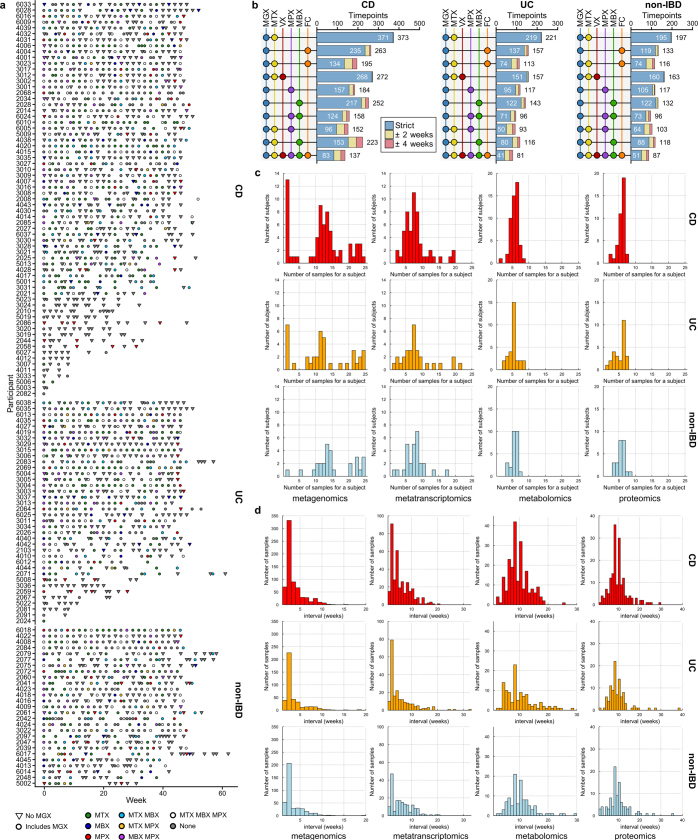
Distribution of sample and measurement types and timing. **a**, Measurements available over time for each IBDMDB participant. **b**, Number of identical or closely-aligned time points available for which each measurement type has been generated (see Methods). **c**, Distributions of the number of processed samples per subject, stratified by disease and measurement type. **d**, Distributions of time intervals between consecutive samples for each measurement type.

We recruited 132 participants from five academic medical centres (three paediatric sub-cohorts: Cincinnati Children’s Hospital, Massachusetts General Hospital (MGH) Pediatrics, and Emory University Hospital; and two adult cohorts: MGH and Cedars-Sinai Medical Center; Fig. 1a, Extended Data Table 1, see Methods). Individuals not diagnosed with IBD on the basis of initial endoscopic and histopathologic findings were classified as ‘non-IBD’ controls. We analysed 651 biopsies (baseline) and 529 blood samples (approximately quarterly), which were collected in the clinic, and 1,785 stool samples, which were collected every two weeks using a home shipment protocol for one year (Fig. 1b). The latter yielded primarily microbially focused profiles: metagenomes (MGX), metatranscriptomes (MTX), proteomes (MPX), metabolomes (MBX), and viromes (VX) at several ‘global’ time points across all subjects (Fig. 1b), as well as denser, more intensive sampling from individuals with more variable disease activity (see Methods, Extended Data Fig. 1a–d). We generated multiple measurement types from many individual stool specimens, including 305 samples that yielded all stool-derived measurements, and 791 MGX–MTX pairs (Fig. 1c, Extended Data Fig. 1b). Biopsies yielded host- and microbe-targeted human RNA sequencing (RNA-seq (HTX)), epigenetic reduced representation bisulfite sequencing (RRBS), and 16S rRNA gene amplicon sequencing (16S), which were matched with human exome sequencing, serological profiles, and RRBS from blood. All data are available at https://ibdmdb.org/.

## Multi-omic gut microbiome changes in IBD

**Extended Data Fig. 2:**
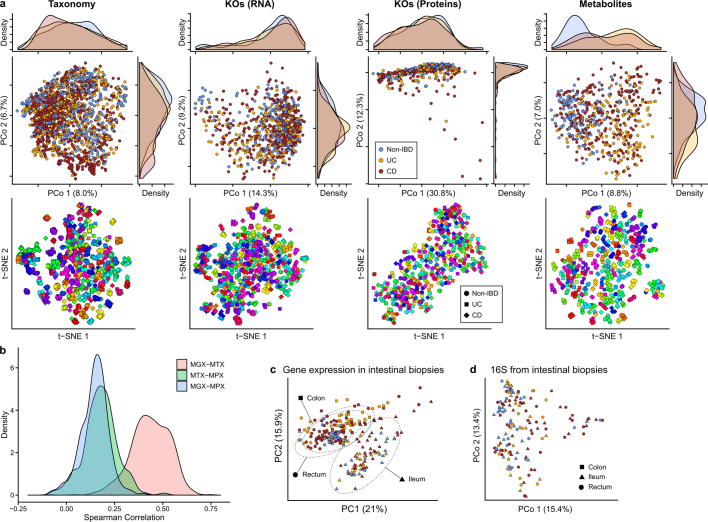
Within-individual stability is a major driver of microbiome differences across measurement types. **a**, PCoA and t-SNE embeddings based on Bray–Curtis dissimilarity matrices from stool species abundances, transcripts, proteins, and metabolites. Marginal densities are shown for the PCoAs that show disease separation for some measurements. In the t-SNEs, each subject has been assigned a different hue, showing that small clusters generally represent individuals’ time courses, as inter-individual differences are the greatest driver of microbiome variation across measurement types (Fig. 1f). Sample counts are shown in Fig. 1b. **b**, Distributions of correlations between functional profiles, captured as UniRef90^100^ gene family abundances^73^, measured from paired metagenomes, metatranscriptomes, and metaproteomes (see Methods). **c**, Human transcriptional expression was mostly determined by biopsy location rather than IBD phenotype and inflammation (*n* = 249 samples from 91 subjects). Ordination shows PCA on gene expression levels normalized by library sizes and represented as CPM. Ellipses indicate 95% confidence regions for the indicated sample types. **d**, Principal coordinates plot (Bray–Curtis on OTU profiles) of community profiles from biopsy samples shows that mucosal microbial communities do not differ significantly by biopsy location (shape), unlike human gene expression in epithelial tissue (Fig. 4b).

Consistent with prior studies^4,5^, although subsets of IBD (CD in particular) contributed to the second axis of taxonomy-based principal coordinates (Fig. 1d, Extended Data Fig. 2a), inter-individual variation accounted for the majority of variance for all measurement types^5,9,10^ (Fig. 1e, f, Extended Data Fig. 2a). Even relatively large effects, such as disease status or physiological and technical factors, explained a smaller proportion of variation (Fig. 1f); this was true across measurement types, although these captured distinct aspects of IBD dysbiosis (see below).

Most measurement types captured correlated changes among and within subjects, cross-sectionally and longitudinally (Fig. 1e). Functional profiles, measured from MGX, MTX, and MPX, were the most tightly coupled (Fig. 1e), although some individual feature-wise correlations were weak (Spearman’s correlation MGX–MTX 0.44 ± 0.10 (mean ± s.d.), MGX–MPX 0.14 ± 0.083, and MTX–MPX 0.18 ± 0.096l; Extended Data Fig. 2b). Unexpectedly, characterized enzymes tended to be only weakly correlated with their known substrates or products (Supplementary Fig. 1). Although our dietary characterization was obtained through a very broad-level food frequency questionnaire, it provides an initial characterization of longitudinal diet–microbiome coupling in a substantial population over many months; diet accounted for a small but significant 3% (false discovery rate (FDR) *P* = 7.4 × 10^−4^) of taxonomic variation between subjects, and 0.7% (FDR *P* = 4.3 × 10^−4^) of variation longitudinally.

**Fig. 2:**
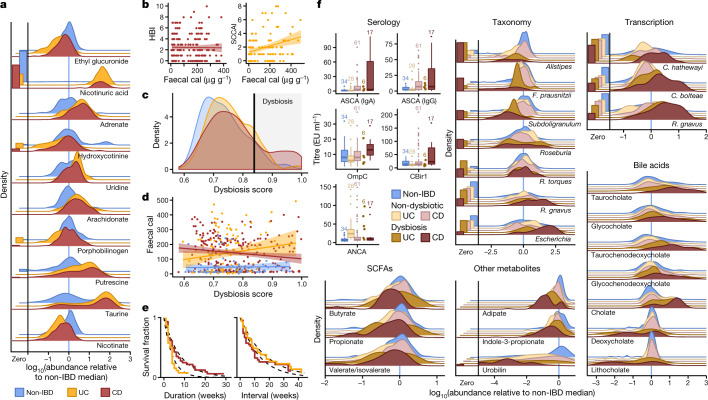
Metagenomic, metatranscriptomic, and stool metabolomic profiles are disrupted during IBD activity. **a**, Relative abundance distributions for ten of the most cross-sectionally significantly differentially abundant metabolites in samples from individuals with IBD, as a ratio to the median relative abundance in individuals without IBD (Wald test; all FDR *P* < 0.003; see Methods; Supplementary Tables 1–14). Left, fraction of samples below detection limit (see Methods). *n* = 546 samples from 106 subjects. **b**, Relationships between two measures of disease activity: patient-reported (Harvey–Bradshaw index (HBI) in CD, *n* = 680 samples from 65 subjects; simple clinical colitis activity index (SCCAI) in UC, *n* = 429 samples from 38 subjects) and host molecular (faecal calprotectin (cal)^43^, *n* = 652 samples from 98 subjects). Linear regression shown with 95% confidence bound. **c**, **d**, Distribution of microbial dysbiosis scores as a measure of disease activity (**c**, median Bray–Curtis dissimilarity between a sample and non-IBD samples; see Methods) and its relationship with calprotectin (**d**, *n* = 652 samples from 98 subjects). Linear regression with 95% confidence. **e**, Kaplan–Meier curves for the distributions of the durations of (left) and intervals between (right) dysbiotic episodes in UC and CD. Both are approximately exponential (fits in dashed lines), with means of 4.1 and 17.2 weeks, respectively, for UC, and 7.8 and 12.8 weeks for CD (see Methods). **f**, Relative abundance distributions of significantly different metagenomic species (*n* = 1,595 samples from 130 subjects), metabolites (*n* = 546 samples from 106 subjects), and microbial transcribers (*n* = 818 samples from 106 subjects) in dysbiotic samples compared to non-dysbiotic samples from the same disease group (Wald test; all FDR *P* < 0.05; full results in Supplementary Tables 15–28). Also shown are antibody titres for ANCA, ASCA (IgG or IgA), anti-OmpC, and anti-CBir1 antibodies (*n* = 146 samples from 61 subjects). Boxplots show median and lower/upper quartiles; whiskers show inner fences; sample sizes above boxes.

Simple cross-sectional differences between individuals with IBD and those without (Supplementary Tables 1–14) were most apparent in the metabolome (Figs. 1f, 2a, Extended Data Fig. 2a, c, d, see Methods). Overall, metabolite pools were less diverse in individuals with IBD, paralleling previous observations for microbial diversity (Supplementary Table 2); this might be caused by poor nutrient absorption, greater water or blood content in the bowels, and shorter bowel transit times in individuals with active IBD^11^. The smaller number of compounds that were more abundant in patients with IBD included polyunsaturated fatty acids such as adrenate and arachidonate. Pantothenate and nicotinate (vitamins B5 and B3, respectively) were particularly depleted in the gut during IBD; this is notable because these are not typically among the B vitamins that are deficient in the serum of patients with IBD^12^, although low nicotinate levels have been detected during active CD^13^. Both vitamins are required to produce cofactors used in lipid metabolism^14^, and nicotinate has anti-inflammatory and anti-apoptotic functions in the gut^15^. Notably, nicotinuric acid, a metabolite of nicotinate^16^, was found almost exclusively in the stool of patients with IBD. Faecal calprotectin and the Harvey–Bradshaw Index (HBI), two measures of disease severity in CD, showed no significant correlation, whereas the Simple Clinical Colitis Activity Index^17^ (SCCAI) in UC did correlate weakly with faecal calprotectin levels (Fig. 2b).

**Extended Data Fig. 3:**
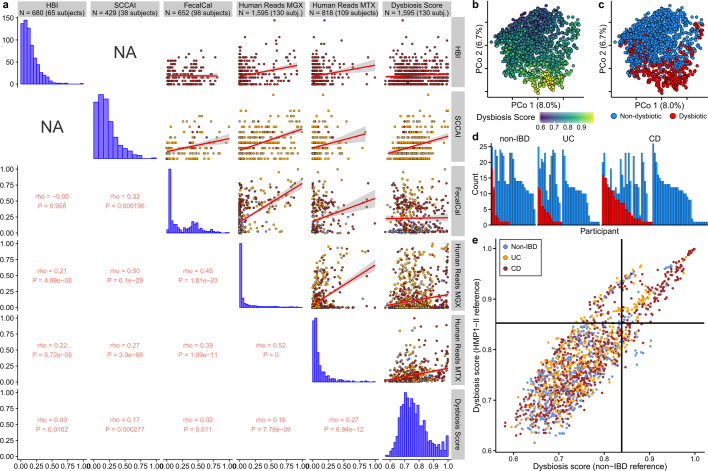
Patient-reported, molecular, and microbial disease activity measures. **a**, Relationships between six measures of disease activity: the patient-reported HBI in CD, patient-reported SCCAI in UC, faecal calprotectin, the fractions of human reads from stool MGX and MTX, and a dysbiosis score defined here as departures from control population microbiome configurations (Fig. 2c). Rho values are Spearman correlations with ties broken randomly. Linear regression is shown (red line) with 95% confidence bound (shaded). Sample counts are presented in the title bar, though sample counts for a particular correlation may be less as samples must be paired. **b**, **c**, PCoA based on metagenomic species-level Bray–Curtis dissimilarities (*n* = 1,595 samples from 130 subjects), indicating dysbiosis score (**b**) and whether the sample was defined as dysbiotic (**c**). **d**, Number of dysbiotic samples per participant. Colour scheme as in **c**. **e**, Relationship between the dysbiosis score, when using the HMP1-II gut data set as reference (*n* = 553 from 249 subjects), compared to the non-IBD data set. The threshold for the dysbiosis classification is also shown (black lines). The two scores are highly correlated (Pearson *ρ* = 0.86; two-sided *P* < 2.2 × 10^−16^), as are the resulting dysbiosis classifications (odds ratio of 56; Fisher’s exact test *P* < 2.2 × 10^−16^). *n* = 1,595 samples from 130 subjects.

Notably, no metagenomic species were significantly different between samples from individuals with IBD and those from control individuals after correction for multiple hypothesis testing (Supplementary Table 1), in contrast with previous work^4,5,18^. We hypothesized this was due to the differentiation of study participants into two subsets, one with relatively inactive IBD (due to remission or recent onset) and the other with greater activity. This differentiation has been observed in several cohorts of patients with IBD^5,18^, but it is more pronounced here because we did not take samples specifically from subjects selected for active disease. We therefore classified samples with taxonomic compositions highly unlike those of non-IBD control samples as ‘dysbiotic’ (Fig. 2c, Extended Data Fig. 3a–e, see Methods). Dysbiotic excursions in this cohort did not correspond with disease location (for example, ileal CD; *F*-test *P* = 0.11, see Methods), and occurred longitudinally within subjects; they were weakly correlated with patient-reported and molecular measures of disease activity (Fig. 2d, Extended Data Fig. 3a). In total, 272 dysbiotic samples were taken during 78 full periods of dysbiosis and 9 censored periods (that is, subjects who were dysbiotic at the end of the time series, see Methods), or 17.1% of all samples (*n* = 178 (24.3%) in CD and *n* = 51 (11.6%) in UC). Plots of the durations of and times between dysbiotic periods were approximately exponential, suggesting that transitions are triggered, at least in part, by events with constant probability over time (and are thus potentially stochastic; Fig. 2e).

**Extended Data Fig. 4:**
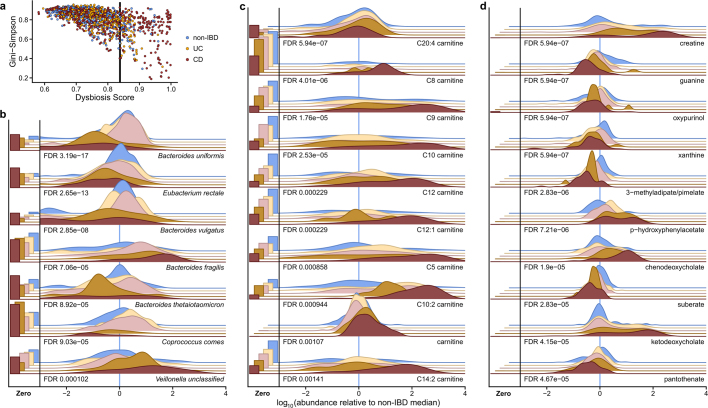
Significant microbial and metabolic perturbations during taxonomic dysbioses. **a**, Alpha diversity (Gini–Simpson) as a function of the dysbiosis score for the sample (Pearson correlation –0.60; *P* < 2.2 × 10^−16^). *n* = 1,595 samples from 130 subjects. **b**, Seven (chosen for space constraints) most differentially abundant species not shown elsewhere in this manuscript (*n* = 1,595 samples from 130 subjects; Wald test; see Methods; full results in Supplementary Table 15). **c**, Top 10 differentially abundant acylcarnitines in dysbiosis (*n* = 546 samples from 106 subjects; Wald test). Carnitine and acylcarnitines are more abundant in dysbiotic CD, whereas C20:4 carnitine is significantly depleted (Supplementary Table 16). **d**, Top 10 differentially abundant metabolites during dysbiosis not shown elsewhere in this manuscript (*n* = 546 samples from 106 subjects; Wald test; full results in Supplementary Table 16).

Using the resulting definition of dysbiosis, dysbiotic periods corresponded to a larger fraction of variation in all measurement types than did overall IBD phenotype (Fig. 1f, Supplementary Tables 15–28); this is likely to reflect a clearer delineation between active and less active disease states within extremely heterogeneous subjects over time. Though it is unclear which aspects of dysbiosis are causes or consequences of IBD, characterization of these changes will lead to greater understanding of microbial dynamics in disease. As in previous cross-sectional studies of established disease^4^, differences between dysbiotic and non-dysbiotic samples from individuals with CD were more pronounced than in those from individuals with UC (Fig. 1f). Notably, dysbiosis also distinguished between independent host measures, such as individuals with high and low ASCA (anti-*Saccharomyces cerevisiae* antibodies), ANCA (anti-neutrophil cytoplasm antibodies), OmpC (outer membrane protein C), and CBir1 (anti-flagellin) antibody titres in serological profiles (Fig. 2f; Fisher’s combined probability test *P* = 0.00044 from Wilcoxon tests between dysbiotic and non-dysbiotic CD). Dysbiosis was not significantly associated with demographics or medication (logistic regression with subject as random effect, all FDR *P* > 0.05). Dysbiosis recapitulated a known decrease in alpha diversity in active disease, but we also identified numerous communities with normal complexity as dysbiotic (Extended Data Fig. 4a). Notably, taxonomic perturbations during dysbiosis mirrored those previously observed cross-sectionally in IBD^6^, such as the depletion of obligate anaerobes including *Faecalibacterium prausnitzii* and *Roseburia hominis* in CD and the enrichment of facultative anaerobes such as *E. coli* (Fig. 2f, Extended Data Fig. 4b). *Ruminococcus torques* and *Ruminococcus gnavus*, two prominent species in IBD^19^, were also differentially abundant in dysbiotic CD and UC, respectively (FDR *P* = 0.041 and 0.0087. A smaller subset of species also increased significantly in transcriptional activity (mean total transcript relative abundance relative to genomic abundance; see Methods) as well as showing differences in abundance, including *Clostridium hathewayi*, *Clostridium bolteae*, and *R. gnavus* (Fig. 2f). All had significantly increased expression during dysbiosis (all FDR *P* < 0.07), and thus their roles in IBD may be more pronounced than suggested solely by their differences in genomic abundance.

In the metabolome, SCFAs were generally reduced in dysbiosis (Fig. 2f). The reduction in butyrate in particular is consistent with the previously observed depletion of butyrate producers^6^ such as *F. prausnitzii* and *R. hominis*, which was also observed here (Fig. 2f). We also detected enrichment of the primary bile acid cholate and its glycine and taurine conjugates (glycocholate *q* = 5.2 × 10^−5^, taurocholate *q* = 1.3 × 10^−5^) in dysbiotic samples from participants with CD, when compared with non-dysbiotic samples. Similarly, glycochenodeoxycholate (*q* = 1.1 × 10^−4^) was also enriched. By contrast, the secondary bile acids lithocholate and deoxycholate (*q* = 5 × 10^−7^ and *q* = 1.8 × 10^−4^, respectively) were reduced in dysbiosis, suggesting that secondary bile-acid producing bacteria are depleted in IBD-related dysbiosis, or that transit time through the colon is too short for these compounds to be metabolized^20,21^. These significant metabolomic differences during microbial dysbiosis, which were concordant with changes expected during disease, provide further evidence that the dysbiosis measure is specifically relevant in IBD.

We also observed several previously undescribed biochemical differences during dysbiosis, such as large changes in acylcarnitine levels. Many acylcarnitines were significantly enriched in dysbiosis (all FDR *P* < 0.05; see Extended Data Fig. 4c), whereas levels of base metabolites were typically reduced (Fig. 2f, Extended Data Fig. 4d). Of note, however, arachidonoyl carnitine (C20:4 carnitine) was reduced, and free arachidonate, a precursor of prostaglandins involved in inflammation, was increased (Fig. 2a). Like bile acids, carnitines are microbially modified compounds that can have competing phenotypic effects depending on the precise modifications: l-carnitine, for example, tends to be anti-inflammatory, whereas fatty acid-conjugated carnitine does not act uniformly on gut inflammation^22^. These opposing changes in biochemically related metabolites further suggest that the differences seen during dysbiosis do not stem simply from the wholesale dilution of stool. Numerous other metabolites were also significantly altered in individuals with dysbiotic IBD (117 of 548 tested known metabolites with FDR *P* < 0.05; Extended Data Fig. 4d, Supplementary Table 16), showing large-scale dysregulation of metabolite pools in tandem with host- and microbiome-specific taxonomic and molecular features (Fig. 2f). Finally, although we found only a single, poorly characterized bacteriophage to be differentially prevalent in both IBD and dysbiosis (notably with reduced prevalence in IBD; Supplementary Tables 3, 17), we note that several participants showed a spike in viral load before a dysbiotic period (Supplementary Fig. 2).

## Decreased gut microbiome stability in IBD

**Fig. 3:**
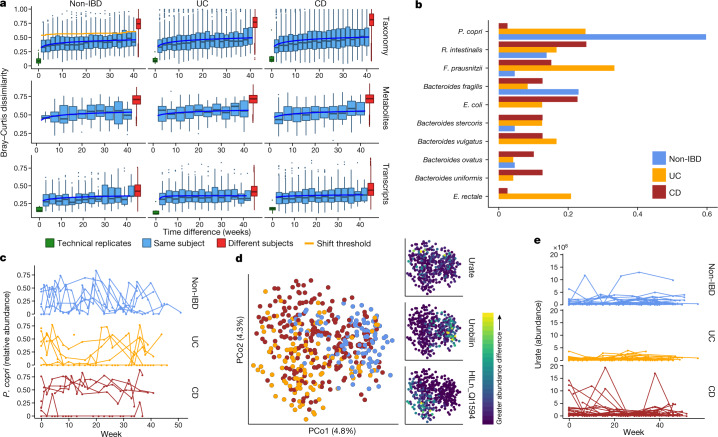
Temporal shifts in the microbiome are more frequent and more extreme in IBD. **a**, Bray–Curtis dissimilarities within subjects as a function of intervening time difference, as compared to different people or technical replicates; calculated for metagenomic taxonomic profiles (species; *n* = 1,595 samples from 130 subjects), metabolomics (*n* = 546 samples from 106 subjects), and functional profiles (KO^30^ gene families; *n* = 818 samples from 106 subjects). Boxplots show median and lower/upper quartiles; whiskers show inner fences. Blue, least-squares power-law fits; orange, thresholds for microbiome shifts (see Methods). Proteomics and species-level transcripts in Extended Data Fig. 5a. Within-subject changes are significantly more extreme in UC and CD than in non-IBD for taxonomic profiles (*F*-test *P* = 3.9 × 10^−10^ and 1.2 × 10^−18^, respectively) and transcripts (*P* = 0.00016 and 1.7 × 10^−5^), with mixed differences for metabolites (*P* = 0.012 and 0.23). Technical replicates shown (when possible) at 0 weeks. **b**, Shift frequencies for the top 10 species with greatest change during shifts, ranked by number of shifts as primary contributor, stratified by disease phenotype(s) (full table Supplementary Table 29). **c**, *P. copri* is of interest in arthritis^23^ and international populations^44^, and it alone retained stable abundances in CD but bloom-relaxation dynamics in controls (two-tailed Wilcoxon test of absolute differences between consecutive time points *P* = 4.2 × 10^−6^ between non-IBD and UC, and 1.1 × 10^−4^ between non-IBD and CD). Plot shows 22 subjects with at least one time point with more than 10% differential abundance (*n* = 267 samples). **d**, Ordination of temporally adjacent samples within individual, based on metabolomics (Bray–Curtis principal coordinates on normalized absolute abundance differences). Disease groups separate significantly (*n* = 440 sample pairs from 106 subjects; PERMANOVA *R*^2^ = 2.8%, *P* < 10^−4^). Urobilin, urate, and an unidentified untargeted feature that segregates with disease groups in the PCoA are shown (right); HILn_QI1594 (HILIC-neg method *m*/*z* = 152.0354, RT = 4.16 min). **e**, As in **c**, but for urate (two-tailed Wilcoxon test *P* = 0.0012 non-IBD–UC, *P* = 0.044 non-IBD–CD; *n* = 546 samples from 106 subjects).

**Extended Data Fig. 5:**
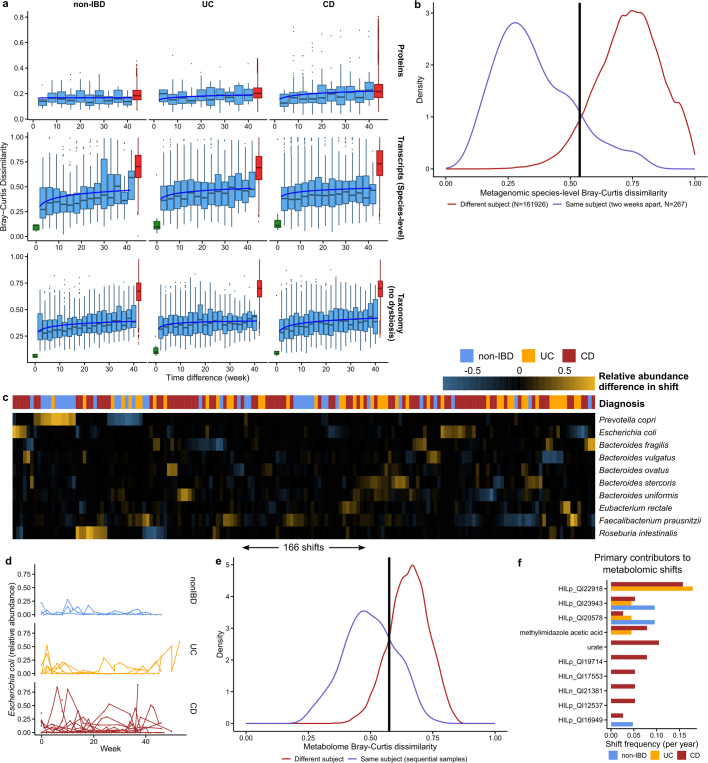
Detecting shifts in longitudinal microbiome multi-omics. **a**, Distributions of Bray–Curtis dissimilarities as a function of time difference between samples for protein profiles, species-level transcriptional activity (see Methods), and species-level taxonomy (though excluding subjects with dysbiosis at any time point), otherwise as in Fig. 3a. Removing subjects with dysbiotic samples removes the extreme dissimilarities (near 1) observed in IBD subjects. Boxplots show median and lower/upper quartiles; whiskers show inner fences. **b**, Distribution of Bray–Curtis dissimilarities between samples from the same subject, two weeks apart versus those from different individuals, allowing us to define a ‘shift’ in the microbiome as a change more likely to have been drawn from the between-subject distribution than within-subject distances (corresponding to Bray–Curtis > 0.54). **c**, Relative abundance differences of the top ten microorganisms that contributed to each of the 183 detected taxonomic shifts among any two within-subject subsequent time points. Shifts are typically reciprocal (that is, losing a microorganism and regaining it later, or vice versa), and microorganism with frequent high-abundance shifts generally correspond to frequent contributors in Fig. 3b. Sample ordering is from a hierarchical clustering using average linkage followed by optimal leaf ordering^101^. **d**, As in Fig. 3c, but for *E. coli* (*n* = 322 samples from 24 subjects; two-tailed Wilcoxon test of the absolute differences in relative abundances between consecutive time points *P* = 2.2 × 10^−4^ for non-IBD to UC, and *P* = 0.029 for non-IBD to CD), which is frequently implicated in gut inflammation. **e**, As in **b**, but showing Bray–Curtis dissimilarities of metabolomic profiles. Here, 22% (96 out of 440) of sample pairs exceed the shift threshold, whereas 13% (183 out of 1,413) exceed the threshold in **b**. If metagenomic profiles are sub-sampled to match the metabolomics samples, this increases to 14% (57 out of 398) of sample pairs, showing that if we increased the sampling rate, this measurement type would be likely to shift more than the metagenomes. **f**, As in Fig. 3b, but showing the primary contributors to metabolomic shifts, that is, the metabolite with the largest change in relative abundance during a shift. Note that other metabolites may still experience large changes in abundance (for example, for this reason, urate was not a primary contributor to any non-IBD shifts, though large changes are visible for one non-IBD individual in Fig. 3e). The full table of detected metabolomic shifts is given in Supplementary Table 30. Violin plot shows the density of points around that intake frequency; bandwidth chosen automatically by Silverman’s method^102^.

Our dense time series for stool-derived multi-omics from many subjects enabled us to carry out in-depth longitudinal analysis, integrating multiple measurements of the microbiome. Each subject’s microbiome tended to diverge more from the baseline over time for metagenomic, metatranscriptomic, and metabolomic profiles (Fig. 3a; *F*-test power law fit *P* < 10^−24^; see Methods). These changes were most pronounced for the taxonomic profiles of individuals with CD and UC (*F*-test difference in power law fits *P* < 10^−9^), where a the microbiome of an individual may have almost no species in common with itself at an earlier time point (dissimilarity of 1; Fig. 3a), consistent with previous observations^9^. Transcripts summarized within species (Extended Data Fig. 5a) showed similar trends (all *F*-test *P* < 8 × 10^−4^) to metagenomic species abundances. Meanwhile, gene family transcripts (Kyoto Encyclopedia of Genes and Genomes (KEGG) Orthologues (KOs)), metabolites (Fig. 3a), and proteins (KOs, Extended Data Fig. 5a) varied much more rapidly, with essentially as much change after around two weeks as over longer time periods (increasing trends less or not significant: non-IBD, UC and CD *F*-test *P* = 0.0006, 0.001, and 0.04, respectively for transcripts; 0.02, 0.06, and 0.003 for metabolites; and 0.5, 0.15, and 0.06 for proteomics). This indicates that these features vary rapidly in the guts of individuals with and without IBD and lack additional, more extreme excursions during disease.

We further characterized large-scale temporal differences by searching for ‘shifts’ in the microbiome between consecutive time points, defined as Bray–Curtis dissimilarities more similar to those between different people than within one person (Fig. 3a, Extended Data Fig. 5b, see Methods). First, considering only metagenomic taxonomic profiles, we found 166 such shifts, with 39 in individuals without IBD (of 382 total possible), 44 in individuals with UC (of 381), and 83 in individuals with CD (of 650) (Supplementary Table 29). Owing to differences in total observation times, the rate of shifts was only marginally higher in individuals with CD or UC than in non-IBD participants (2.09 and 1.83 shifts per year, respectively, compared with 1.79), and these were generally confined to a subpopulation of dysbiotic individuals (Fig. 3a). However, the species with the greatest changes in relative abundance differed markedly (Fig. 3b). Shifts in individuals without IBD occurred primarily in individuals with high abundances of *Prevotella copri*, which underwent repeated expansion and relaxation cycles over the course of weeks to months (Fig. 3c). This organism is of particular interest owing to its behaviour as a population-scale outgroup and its enrichment during new-onset rheumatoid arthritis^23^. The lack of shifts due to *P. copri* in participants with IBD was not due to an absence of *P. copri* in these individuals or an overabundance in those without IBD (6 of 27 non-IBD subjects had at least one time point with more than 10% *P. copri*, consistent with healthy populations^10,24^). Instead, the relative abundances that were present remained more stable in the population with IBD (Fig. 3c). Taxonomic shifts in participants with IBD mirrored earlier observations of relative reductions in obligate anaerobes and overgrowth of facultative anaerobes (Fig. 3b, Extended Data Fig. 5c), and frequently corresponded with entry into and exit from dysbiosis (28 and 23 shifts marked entries and exits in IBD, respectively, accounting for 40% of shifts in IBD). *E. coli* in particular contributed to a large number of shifts in IBD, although there was no clear pattern in which species it traded abundance with (Extended Data Fig. 5c, d).

When we define shifts in a similar manner for metabolomics profiles (Extended Data Fig. 5e), the rate of shifts is approximately half that seen for the metagenome (1.05 shifts per year in participants without IBD, 0.99 shifts per year in UC and 1.36 shifts per year in CD), although these data were strongly affected by the availability of fewer metabolomics samples (Extended Data Fig. 5e). We examined differences in metabolite profiles between adjacent samples from the same subjects and found significant separation by diagnosis (Fig. 3d; PERMANOVA *P* < 10^−4^). These differences were largely driven by unknown compounds, emphasizing the need for further compound annotation efforts and follow up to determine the significance of these compounds in IBD. Features with the greatest differences included urobilin (which showed larger differences in individuals without IBD), urate (largely in patients with CD), and a feature with an *m*/*z* of 152.0354 and retention time (RT) of 4.16 min (potentially the formic acid adduct of pyridinaldehyde), which accounted for differences largely specific to UC. The primary contributors to shifts were largely unidentified compounds (Extended Data Fig. 5f, Supplementary Table 30). HILp_QI22918, an unknown feature with *m*/*z* of 648.43067 and RT of 5.03 min, contributed the most (ten) shifts exclusively in individuals with IBD. Among known compounds, methylimidazole acetic acid and urate were the primary contributors to the most shifts (four shifts each; Fig. 3e).

## Microbiome-associated host factors

When we incorporated host molecular measurements, primarily from intestinal biopsies taken colonoscopically at baseline, into our analysis of the microbiome in IBD, the main influences on population variability were strikingly different from those that affected the microbiota alone. In particular, tissue location was a major driver of intestinal epithelial gene expression (Extended Data Fig. 2c) even in the face of microbial variation^25^ (Extended Data Fig. 2d). We therefore performed microbiome and phenotypic association analyses independently for each standardized biopsy location (see Methods).

**Fig. 4:**
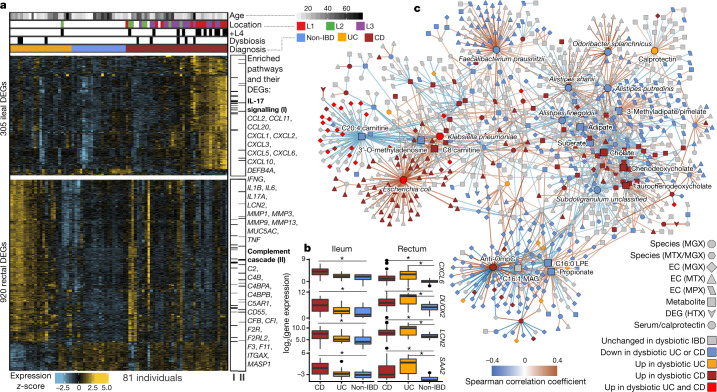
Colonic epithelial molecular processes perturbed during IBD and in tandem with multi-omic host–microbe interactions. **a**, Human DEGs (negative binomial FDR *P* < 0.05, minimum fold change 1.5; Supplementary Table 31) from 81 subjects with paired ileal and rectal biopsies. Ordering by diagnosis, clustering within diagnosis. IL-17 signalling (I) showed strongest enrichment in ileal DEGs (FDR *P* = 8.2 × 10^−12^)^31^, while the complement cascade (II)^45^ was enriched in rectal DEGs from patients with UC (FDR *P* = 5.2 × 10^−8^; KEGG^30^ gene sets, Supplementary Table 32). Example DEGs shown with I and II. **b**, Expression of four genes involved in host–microbe interactions^26–29^. Inflamed biopsy samples are shown for CD from ileum (left, *n* = 20, 23, 39 independent samples for non-IBD, UC, CD respectively); for CD and UC in rectum (right column; *n* = 22, 25, 41 independent samples for non-IBD, UC, CD); non-IBD samples were non-inflamed. Asterisks indicate significant differential expression compared to non-IBD (Fisher’s exact test, FDR *P* < 0.05; *P* values in Supplementary Table 31). Boxplots show median and lower/upper quartiles; whiskers show inner fences. **c**, Significant associations among 10 aspects of host–microbiome interactions: metagenomic species, species-level transcription ratios, functional profiles captured as EC gene families (MGX, MTX and MPX), metabolites, host transcription (rectum and ileum), serology, and calprotectin (sample counts in Fig. 1b, c). Network shows top 300 significant correlations (FDR *P* < 0.05) between each pair of measurement types (for serology, FDR *P* < 0.25). Nodes coloured by disease group in which they are ‘high’, edges by sign and strength of association. Spearman correlations use residuals of a mixed-effects model with subjects as random effects (or a simple linear model when only baseline samples were used (biopsies)) after covariate adjustment (see Methods). Time points approximately matched with maximum separation 4 weeks (see Methods). Singletons pruned for visualization (Extended Data Fig. 8). Hubs (nodes with at least 20 connections) emphasized.

**Extended Data Fig. 6:**
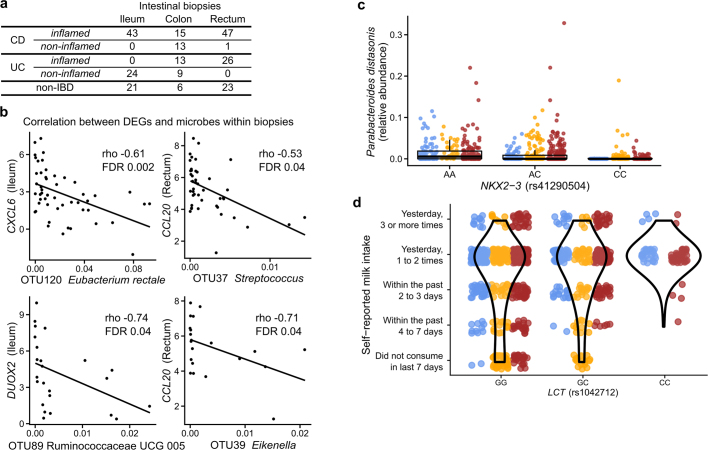
Mucosal communities and human genetics. **a**, Number of biopsy samples available for each biopsy location and inflammation status. **b**, DEGs (Fig. 4a) with newly identified significant correlations with OTU abundances in biopsies (partial Spearman correlation conditioned on disease status, BMI, age at consent and sex; FDR *P* < 0.05; *n* = 54 in ileum and *n* = 52 independent 16S–RNA-seq pairs; full table in Supplementary Table 33). **c**, A limited subset of the microbiome trended with genetic variants in targeted testing, including the strongest trend shown here of *Parabacteroides distasonis* with genotypes of *NKX2-3* (a known IBD-associated locus^103^; boxplots show median and lower/upper quartiles; whiskers show inner fences). This is the most significant association by *P* value among all tested associations between metagenomic taxa and five known IBD loci (nominal significance *P* = 0.006; no associations passed FDR *P* < 0.05, mixed effect model with age, sex, antibiotic and immunosuppressant use and first 20 genetic principal components as covariates while specifying subjects as random effects; Wald test; *n* = 84 subjects of European ancestry with exomes and 960 metagenomes; full results in Supplementary Table 34). **d**, Association between rs1042712 SNP in the *LCT* locus and self-reported milk intake from dietary recall. Self-reported short-term milk intake (from dietary recalls accompanying stool samples) was significantly associated with the count of C alleles (29.8% allele frequency) at rs1042712 in the *LCT* gene locus using a linear mixed effect model accounting for age, sex, first 20 genetic principal components and with subjects as random effects (*P* = 0.028, linear mixed effect regression with Wald test, see Methods). All available data are plotted for unique subjects of European ancestry with exome data (per-genotype subject count (GG/GC/CC):50/26/8). Differences between IBD and non-IBD groups are not statistically significant (odds ratio 0.27; 95% CI 0.05–1.33; *P* = 0.10; *n* = 84 subjects of European ancestry with exomes and 960 dietary surveys; model: IBD (yes|no) ~ intercept + SNP + sex + age + PC1–PC20).

We identified genes that were significantly differentially expressed (DEGs) in patient biopsies taken in inflamed locations of the ileum (from individuals with CD) and rectum (both CD and UC) compared to individuals without IBD (Extended Data Fig. 6a). This analysis identified 305 and 920 genes, genes that were differentially expressed (primarily overexpressed) in the ileum and rectum, respectively, for further analysis (together representing 1,008 unique genes, negative binomial model FDR *P* < 0.05 and fold-change >1.5; Fig. 4a, Supplementary Table 31). These included genes that can affect commensal microorganisms directly, such as the antimicrobial *CXCL6* (a cell membrane disruptor^26^) and *SAA2* (which inhibits growth of Gram-negative bacteria^27^), as well as indirect microbial modulators such as *DUOX2* (which produces reactive oxygen species^28^) and *LCN2* (which induces microbial iron starvation through sequestration^29^; Fig. 4b). Enrichment analysis testing for overrepresentation of KEGG^30^ pathways among DEGs also confirmed strong representation of immune-related pathways (one-sided hypergeometric test, FDR *P* < 0.05). In particular, the IL-17 signalling pathway, components of which have been previously identified in gene expression studies of ileal biopsies from patients with CD^31,32^, was enriched in upregulated DEGs in both ileum and rectum (FDR *P* = 2.8 × 10^−14^; Fig. 4a, Supplementary Table 32). Among upregulated DEGs in rectal biopsies from patients with UC, we found further enrichment of the complement cascade (FDR *P* = 4.4 × 10^−10^), a component of innate immunity^33^ that has been implicated in IBD^25,34,35^.

To identify the components of the microbiome that were most associated with these changes, we tested for transcripts that covaried with the relative abundance of microorganisms measured directly from the same specimens using 16S amplicon sequencing. We identified 31 and 106 significant gene–operational taxonomic unit (OTU) pairs in the ileum and rectum, respectively, with no overlap between the two sites, consistent with the different overall gene expression patterns that separate them (partial Spearman correlation FDR *P* < 0.05; see Methods, Extended Data Fig. 6b, Supplementary Table 33). The genes involved included known IBD-associated host–microbial interaction factors, including *DUOX2* and its maturation factor *DUOXA2*^31,36^, both of which were negatively associated with the abundance of Ruminococcaceae UCG 005 (OTU 89) in the ileum. The expression of several chemokine genes, some of which have reported antimicrobial properties^37^ (*CXCL6*, *CCL20*), were negatively correlated with the relative abundance of *Eubacterium rectale* (OTU 120) in the ileum, and *Streptococcus* (OTU 37) and *Eikenella* (OTU 39) in the rectum, suggesting that these species are the most susceptible to the activity of these chemokines. Finally, although this cohort was not designed for genetic association discovery (Supplementary Discussion, Extended Data Fig. 6c, d, Supplementary Table 34), we also provide exome sequencing for 92 subjects, which may be integrated with larger populations in the future.

## Dynamic, multi-omic microbiome interactions

We next searched for host and microbial molecular interactions that might underlie disease activity in IBD by constructing a large-scale cross-measurement type association network that incorporated ten microbiome measurements: metagenomic species, species-level transcription ratios, functional profiles captured as Enzyme Commission (EC) gene families (MGX, MTX and MPX), metabolites, host transcription (rectum and ileum separately), serology, and faecal calprotectin. To identify co-variation between components of the microbiome above and beyond those linked strictly to inflammation and disease state, each measurement type was first residualized using the same mixed-effects model (or linear model when appropriate) used to determine differential abundance (‘adjusted’ network; see Methods). This residualization uses longitudinal measurements to minimize any inter-individual variation (including IBD status), as well as dysbiotic excursions as drivers of the detected associations, and thus highlights within-person associations over time. The resulting network contained 53,161 total significant edges (FDR *P* < 0.05) and 2,916 nodes spanning features from all measurement types (Supplementary Table 35). We constructed a filtered subnetwork for visualization from the top 300 edges (by *P* value) per measurement type in which at least one connected node was dysbiosis-associated (Fig. 4c).

**Extended Data Fig. 7:**
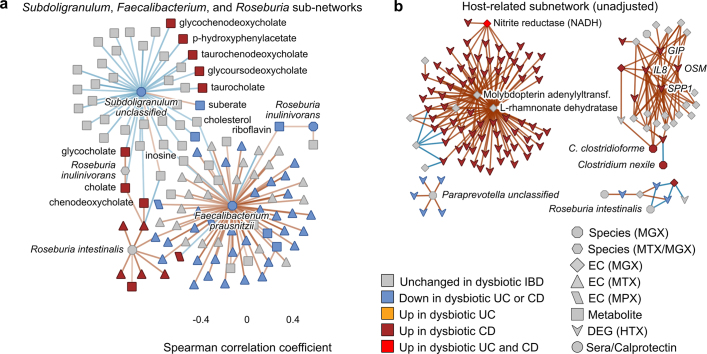
Microbial and host-related subsets of the multi-omic association networks. **a**, Subset of the network in Fig. 4c showing metagenomic abundances (octagons) and expression levels (hexagons) of *Subdoligranulum*, *Roseburia* spp. and *F. prausnitzii* and their neighbours (three functionally associated microbial hubs selected for further investigation based on anti-inflammatory associations in the literature, see text). **b**, The host expression-related subnetwork of the ‘unadjusted’ association network (Extended Data Fig. 9, Supplementary Discussion). Sample counts in Fig. 1b, c.

Representatives from the five stool-derived measurements occurred as hubs (defined as nodes with at least 20 connections) in this network, all of which were identified as differentially abundant in dysbiosis. Particularly connected taxonomic features (from metagenomes and metatranscriptomes) included the abundances of *F. prausnitzii* and unclassified clades related to *Subdoligranulum*^38^, which are closely phylogenetically related, although the only molecular features common to both organisms covaried with the abundances of cholesterol and inosine (Extended Data Fig. 7a). *F. prausnitzii* accounted for some of the strongest associations overall, including the expression of numerous ECs that were downregulated in dysbiosis. On the other hand, *E. coli* (and to lesser extent *Haemophilus parainfluenzae*) accounted for a large fraction of upregulated ECs. Members of the *Roseburia* genus were also associated, metatranscriptionally as well as metagenomically, with bile acids and a number of acylcarnitines, suggesting that *Roseburia* (together with *Subdoligranulum*) are involved in the carnitine and bile acid dysregulation observed in IBD.

**Extended Data Fig. 8:**
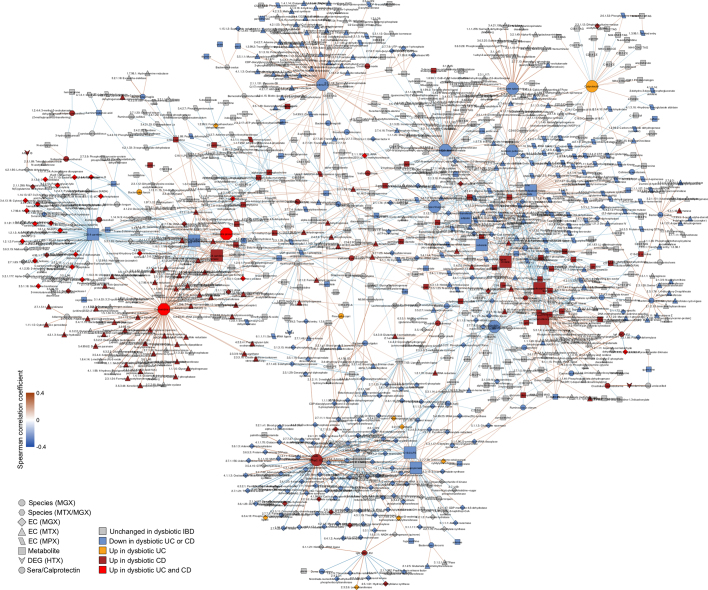
Significant covariation among multi-omic components of the gut microbiome and host interactors in IBD (adjusted). Detailed labelling of the association network in Fig. 4c (intended for magnification). The network was constructed from ten data sets: metagenomic species, species-level transcription ratios, functional profiles at the EC levels (MGX, MTX and MPX), metabolites, host transcription (rectal and ileal separately), serology and faecal calprotectin. As in Fig. 4c, measurement types were approximately matched in time with a maximum separation between paired samples of four weeks. The top 300 significant correlations (FDR *P* < 0.05) among correlations between features that were differentially abundant in dysbiosis were used to construct the network visualized here (for serology, a threshold of FDR *P* < 0.25 was used). Nodes are coloured by the disease group in which they are ‘high’, and edges are coloured by the sign and strength of the correlation. For this adjusted network, Spearman correlations were calculated using HAllA from the residuals of a mixed-effects model with subjects as random effects (or a simple linear model without the random effects when only baseline samples were used) after adjusting for age, sex, diagnosis, dysbiosis status, recruitment site, and antibiotics (see Methods). Appropriate normalization and/or transformation for each measurement type was performed independently before the model fitting (see Methods). Singleton node pairs were pruned from the network. Source associations are in Supplementary Table 35, sample counts in Fig. 1b, c.

Acylcarnitines and bile acids as overall chemical classes featured prominently in the network, related in part to their changes during dysbiosis. Acylcarnitines were associated with numerous dysbiosis-associated species including *R. hominis* (nine acylcarnitines, FDR *P* < 0.05; Supplementary Table 35), *Klebsiella pneumoniae* (three), and *H. parainfluenzae* (three), as well as expression of *C. bolteae* (three), suggesting that multiple scales of regulation, including long-term growth-based and short-term transcriptional, are involved. Particularly notable biochemical hubs in the network included C8 carnitine, another acylcarnitine that was significantly increased in dysbiotic CD, cholate, chenodeoxycholate, and taurochenodeoxycholate, which together accounted for 107 edges (6%; Fig. 4c). Other prominent metabolite associations included several long-chain lipid hubs and the SCFA propionate; antibodies against OmpC were strongly associated with these, as well as with the metagenomic abundances of the numerous ECs involved in the system’s biosynthesis or as interactors. Calprotectin, as the sole feature in its own measurement type, was weakly associated with a number of metabolites that were not differentially abundant in dysbiosis, as well as with the metagenomic abundance of several dysbiosis-associated ECs. Three host genes appeared in this high-significance subnetwork: ileal expression of *GIP*, *NXPE4*, and *ANXA10*. Expression of RNA polymerase was also a prominent node in the network, though not a hub, that was upregulated in dysbiosis (Extended Data Fig. 8). The regulation of this essential enzyme class is growth-rate-dependent^39^, suggesting that microbial communities as a whole are more often in higher growth conditions in dysbiotic IBD.

**Extended Data Fig. 9:**
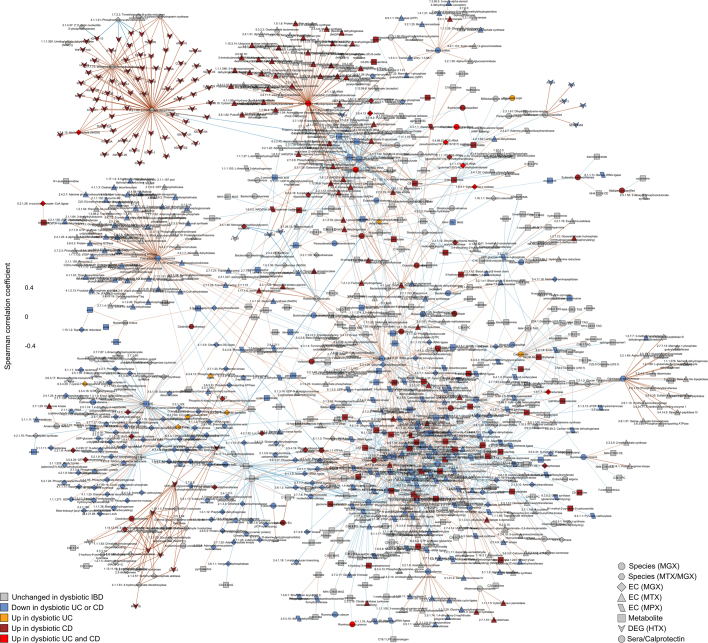
Significant covariation among multi-omic components of the gut microbiome and host interactors in IBD (unadjusted). The network was constructed from ten data sets: metagenomic species, species-level transcription ratios, functional profiles at the EC levels (MGX, MTX and MPX), metabolites, host transcription (rectal and ileal separately), serology and faecal calprotectin. As in Fig. 4c, measurement types were approximately matched in time with a maximum separation between paired samples of four weeks. The top 300 significant correlations (FDR *P* < 0.05) among correlations between features that were differentially abundant in dysbiosis were used to construct the network visualized here (for serology, a threshold of FDR *P* < 0.25 was used). Nodes are coloured by the disease group in which they are ‘high’, and edges are coloured by the sign and strength of the correlation. For this unadjusted network, Spearman correlations were calculated using HAllA from the residuals of the same model as in Extended Data Fig. 8, though without adjusting for dysbiosis (see Methods). Appropriate normalization and/or transformation for each measurement type was performed independently before the model fitting (see Methods). Singleton node pairs were pruned from the network. Source associations are in Supplementary Table 36, sample counts in Fig. 1b, c.

Finally, we also identified associations among features in the microbiome that took dysbiosis into account, resulting in a second network using the same methodology but without adjusting for dysbiosis (‘unadjusted’; Supplementary Discussion, Extended Data Figs. 7b, 9, Supplementary Table 36). Together, these networks contextualize the multiple types of microbiome disruption that are observed in IBD, with associations among many molecular feature types that represent potential targets for follow-up studies on the mechanisms that underlie IBD and gastrointestinal inflammation.

## Conclusions

As part of the HMP2, we have developed the IBDMDB, one of the first integrated studies of multiple molecular features of the gut microbiome that have been implicated in IBD dynamics. While overall population structure was comparable among measurements of the microbiome—metagenomic, metatranscriptomic, metabolomic, and others—each measurement identified complementary molecular components of longitudinal dysbioses in CD and UC. Some, such as taxonomic shifts in favour of aerotolerant, pro-inflammatory clades, have been captured by previous studies; others, such as greater gene expression by clostridia during disease, were discovered by the use of new measurements (metatranscriptomes). The temporal stability of multiple microbiome measurements likewise differed across IBD phenotypes and disease activity, with distinct effects on molecular components of the microbiome (including unexpected stability of the relative abundance of *P. copri* in individuals with IBD). Our data provide a catalogue of new relationships between multi-omic features identified as potentially central during IBD, in addition to data, protocols, and relevant bioinformatic approaches to enable future research.

By leveraging a multi-omic view on the microbiome, our results single out a number of host and microbial features for follow-up characterization. An unclassified *Subdoligranulum* species, recently shown to form a complex of new species-level clades^38^, was both markedly reduced in IBD and central to the functional network, associating with a wide range of IBD-linked metabolites both identifiable (for example, bile acids and polyunsaturated fatty acids) and unidentifiable. The clade is likely to contain at least seven species that are closely related to the *Subdoligranulum*, *Gemmiger*, and *Faecalibacterium* genera, typically butyrate producers that are considered to be beneficial, particularly in IBD^40^. Therefore, the isolation and characterization of additional species—especially in tandem with these associated metabolites—is likely to reveal these clades’ physiological and immunological interactions and the consequences of their depletion in IBD. More generally, strain-level profiling of implicated microorganisms remains to be carried out, particularly in direct association with host epithelium and corresponding molecular changes. This profiling is feasible with existing data from this study, and will serve to pinpoint the specific organisms responsible for IBD-associated accumulation of primary unconjugated bile acids and depletion of secondary bile acids^41^. Only very few, low-abundance species are currently known to be capable of secondary bile acid metabolism^42^, and expanding the range of strains known to carry appropriate metabolic cassettes will indicate potential new targets for therapeutic restoration. Beyond short-chain fatty acids and bile acids, the large-scale acylcarnitine dysbiosis observed here may also provide a promising new target for IBD, particularly after determining whether this shift in metabolite pools is host- or microbiome-driven.

We stress that it has not yet been determined whether these multi-omic features of the microbiome can predict disease events before their occurrence and that the disease-relevant time scales of distinct molecular events have not been identified (for example, static host genetics, relatively slow epigenetics or microbial growth, rapid host and microbial transcriptional changes). It may also be fruitful to seek out the earliest departures from a subject-specific baseline state that, while themselves still ‘eubiotic’, may predict the subsequent onset of dysbiosis or disease symptoms. Some such characterization may be possible in data from this study, although other causal analysis may be better carried out at finer-grained time scales or using interventional study designs. It will be most important to take these molecular results back to the clinic, in the form of better predictive biomarkers of IBD progression and outcome, and as a set of new host–microbe interaction targets for which treatments to ameliorate the disease may be developed.

## Methods

### Recruitment and specimen collection

#### Recruitment

Five medical centres participated in the IBDMDB: Cincinnati Children’s Hospital, Emory University Hospital, Massachusetts General Hospital, Massachusetts General Hospital for Children, and Cedars-Sinai Medical Center. Patients were approached for potential recruitment upon presentation for routine age-related colorectal cancer screening, work up of other gastrointestinal (GI) symptoms, or suspected IBD, either with positive imaging (for example, colonic wall thickening or ileal inflammation) or symptoms of chronic diarrhoea or rectal bleeding. Participants could not have had a prior screening or diagnostic colonoscopy. Potential participants were excluded if they were unable to or did not consent to provide tissue, blood, or stool, were pregnant, had a known bleeding disorder or an acute gastrointestinal infection, were actively being treated for a malignancy with chemotherapy, were diagnosed with indeterminate colitis, or had undergone a prior, major gastrointestinal surgery such as an ileal/colonic diversion or j-pouch. Upon enrolment, an initial colonoscopy was performed to determine study strata. Subjects not diagnosed with IBD based on endoscopic and histopathologic findings were classified as ‘non-IBD’ controls, including the aforementioned healthy individuals presenting for routine screening, and those with more benign or non-specific symptoms. This creates a control group that, while not completely ‘healthy’, differs from the IBD cohorts specifically by clinical IBD status. Differences observed between these groups are therefore more likely to constitute differences specific to IBD, and not differences attributable to general GI distress. In total, 132 subjects took part in the study (Extended Data Table 1).

#### Regulatory compliance

The study was reviewed by the Institutional Review Boards at each sampling site: overall Partners Data Coordination (IRB #2013P002215); MGH Adult cohort (IRB #2004P001067); MGH Paediatrics (IRB #2014P001115); Emory (IRB #IRB00071468); Cincinnati Children’s Hospital Medical Center (2013-7586); and Cedars-Sinai Medical Center (3358/CR00011696). All study participants gave written informed consent before providing samples. Each IRB has a federal wide assurance and follows the regulations established at 45 CFR Part 46. The study was conducted in accordance with the ethical principles expressed in the Declaration of Helsinki and the requirements of applicable federal regulations.

#### Specimen collection and storage

Specimens for research (biopsies, blood draws, and stool samples) were collected during the screening colonoscopy, at up to five quarterly follow-up visits at the clinic (termed ‘baseline’, visit 2, and so on, occurring at months 0, 3, 6, 9, and 12), and every two weeks by mail.

#### Biopsies

Biopsies were primarily gathered during the initial screening colonoscopy, where approximately four to fourteen biopsies were collected for each subject. For each location sampled (at least ileum and 10 cm from rectum, plus discretionary sites of inflammation), one biopsy was collected for standard histopathology at the sampling institution, two biopsies were collected and stored in RNAlater for molecular data generation (host and microbial, stored at –20 °C), and one biopsy was collected and placed in a sterile tube with 5% glycerol (stored at –80 °C). If possible, additional biopsies from inflamed tissue and nearby non-inflamed tissue were taken from participants with CD or UC. For adults, a second set of biopsies was also collected from each location (rectum and ileum) for epithelial cell culture (for detailed protocols see http://ibdmdb.org/protocols). All biopsies were stored for up to two months at the collection site, and shipped overnight on dry ice to Washington University for epithelial cell culture or to the Broad Institute for molecular profiling.

#### Blood samples

Blood samples (whole blood and serum) were taken at the quarterly clinical visits. For whole blood, 1 ml of blood was collected and stored at –80 °C. For serum, blood was drawn into a 5-ml SST tube, and left at room temperature for 40 min. This was centrifuged for 15 min at 3,000 r.p.m. and 0.5-ml portions were immediately aliquoted into 2-ml microtubes. Tubes were stored at –80 °C.

#### Stool samples

Stool specimens were collected both at the clinical visits and every two weeks by mail using a home collection kit developed for the project (http://ibdmdb.org/protocols) and previously validated^46^. Participants first deposited stool into a collection bowl suspended over a commode. They then collected two aliquots using a scoop to transfer stool into two Sarstedt 80.623 tubes: one with approximately 5 ml molecular biology grade 100% ethanol, and one with no preservative. Stool samples were then sent from each participant by FedEx to the Broad Institute where they were processed immediately before storage at –80 °C. The ethanol tube was centrifuged to pellet stool, which was subaliquotted, and the supernatant was transferred to a new tube for metabolomic analysis. Stool from ethanol was aliquoted into 2-ml cryovials in ~100–200-mg aliquots, prioritizing specimens for meta’omic sequencing, metabolomics, and viromics in that order. Any remaining stool was stored in additional aliquot tubes. One hundred milligrams of the non-ethanol stool was stored for assaying faecal calprotectin and the remainder was saved in a second tube. All samples were stored at –80 °C after receipt before processing. This home-collection method was shown previously to produce reproducible results compared to flash-frozen samples^46^, consistent with previous observations across data types^47–49^. Note that an accurate estimate of the stool water content could not be obtained, as samples were collected by subjects and preserved in ethanol at room temperature until aliquots were generated for the different data generation platforms.

#### Participant and sample metadata

Descriptions of each participant and specimen were captured at baseline and accompanying each specimen collection, respectively. At baseline (that is, during or before the screening colonoscopy), subjects completed a Reported Symptoms Questionnaire, the Short Inflammatory Bowel Disease Questionnaire^50^, a Food Frequency Questionnaire, and an Environmental Questionnaire, and the Simple Endoscopic Score^51^ for CD subjects or Baron’s Score^52^ for UC subjects was assessed.

During both follow-up visits and paired with mailed stool samples, subjects completed an Activity Index and Dietary Recall Questionnaire to assess their disease activity index (HBI for CD or SCCAI for UC) and provide a retrospective recall of their recent diet. All questionnaires, as well as detailed protocols (including product numbers), can be found on the IBDMDB data portal at http://ibdmdb.org/protocols. Responses and metadata are available at http://ibdmdb.org/results, and summaries of phenotypes for samples and subjects are provided (Supplementary Fig. 3) along with summaries of the final time series for each subject (Supplementary Fig. 2).

### Stool specimen processing

#### Sample selection

Sample selection proceeded in two phases, with an initial round of data generation producing a pilot metagenomics and metatranscriptomics data set, which was analysed separately^53^. This pilot sample selection included at least one sample per participant that was enrolled in the study at that time, two long time courses per disease group (CD, UC, non-IBD), and multiple shorter time courses, resulting in 300 samples. For a subset of 78 samples, metatranscriptomic data were generated. Samples were chosen on the basis of sample mass, preferentially selecting samples that could be re-sequenced if needed during the later data generation.

For the second, larger phase of data generation, stool samples were selected for different assays with the goal of generating data covering as many aspects of the cohort as possible, including per-subject time courses, cross-subject global time points, and samples from all patients, phenotypes, age ranges, clinical centres, and so forth (Fig. 1b). The subset of measurements performed for each sample was determined in large part by aliquot requirements (in particular, mass requirements for the assay relative to how much the patient provided) and cost.

For proteomics and metabolomics, six global time points were equally distributed over the year-long time series for as many subjects as possible. Restrictions such as available sample mass and missing samples were incorporated by selecting the nearest suitable sample in time, resulting in slight irregularities in the sampling pattern. In total, 546 metabolite profiles and 450 proteomics profiles were generated. From among these samples, 768 were selected for metagenomics, metatranscriptomics, and viromics, corresponding to 8 plates of 96 samples each. Samples already selected for proteomics or metabolomics were prioritised to facilitate integrated data analysis (316 samples had sufficient mass), resulting in six global time points for all subjects. In cases where the respective sample was not available for a subject, the nearest suitable sample in time was selected. Subjects with greater fluctuations in their HBI or SCCAI scores were then prioritized for denser sampling, resulting in 12 long time courses for 5 participants with CD, 4 with UC, and 3 without IBD. The selection also included 23 technical replicates for metagenomics, metatranscriptomics and viromics.

Finally, 576 additional samples were selected specifically for metagenomic sequencing (6 plates) resulting in a total of 1,344 metagenomic samples. Samples at previously selected global time points and long time courses that had been restricted by available mass for other measurement types were prioritized. An additional four global time points were added by this process, as well as 15 long time courses (representing 10 participants with CD, 10 with UC, and 7 without IBD), and 22 samples that had been previously sequenced for the pilot data and represented additional technical replicates. Lastly, 522 samples were selected for faecal calprotectin measurements, prioritizing samples that were selected for any other multi-omics data generation and representing a broad overview of the cohort. Of a total of 2,653 collected stool samples, 1,785 generated at least one measurement type (Fig. 1b).

Sample selection for RNA-seq and 16S sequencing from biopsies, and host genotyping from blood draws, aimed to cover the 95 subjects who contributed at least 14 stool samples, as permitted by the availability of biopsies and blood draws for each assay. Sample selection from biopsies additionally aimed to cover biopsies from inflamed and non-inflamed sites. In total, 254 biopsies were selected for RNA-seq, covering 43 participants with CD, 25 with UC, and 22 without IBD, and distributed across biopsy sites and inflammation statuses (Extended Data Fig. 6A); and 161 biopsies were selected for 16S sequencing, covering 36 participants with CD, 21 with UC, and 22 without IBD. Exome sequencing was performed for 46 participants with CD, 24 with UC, and 22 without IBD.

Sample selection for remaining sample types (RRBS, blood serology) included all samples with a suitable sample available.

### Sequencing assays

#### DNA and RNA isolation for metagenomics and metatranscriptomics

Total nucleic acid was extracted from one aliquot of each assayed stool sample via the Chemagic MSM I with the Chemagic DNA Blood Kit-96 from Perkin Elmer. This kit combines chemical and mechanical lysis with magnetic bead-based purification. Prior to extraction on the MSM-I, TE buffer, lysozyme, proteinase K, and RLT buffer with beta-mercaptoethanol were added to each stool sample. The stool lysate solution was vortexed to mix.

Samples were then placed on the MSM I unit to automate the following steps: M-PVA magnetic beads were added to the stool lysate solution and vortexed to mix. The bead-bound total nucleic acid was then removed from solution using a 96-rod magnetic head and washed in three ethanol-based wash buffers. The beads were then washed in a final water wash buffer. Finally, the beads were dipped in elution buffer to resuspend the DNA sample in solution. The beads were then removed from solution, leaving purified total nucleic acid eluate. The eluate was then split into two equal volumes: one for DNA and the other for RNA. SUPERase-IN solution was added to the DNA samples, and the reaction was cleaned up using AMPure XP SPRI beads. DNase was added to the RNA samples, and the reaction was cleaned up using AMPure XP SPRI beads.

DNA samples were quantified using a fluorescence-based PicoGreen assay. RNA samples were quantified using a fluorescence-based RiboGreen assay. RNA quality was assessed via smear analysis on the Caliper LabChip GX.

#### Metagenome sequencing

Metagenomes were generated from the resulting DNA for 1,638 stool samples, selected to obtain both a broad overview of targeted, aligned time points for all subjects (Fig. 1b), complemented by a dense sampling of subjects which tended to have greater disease activity, as determined by their HBI or SCCAI scores.

Whole-genome fragment libraries were prepared as follows. Metagenomic DNA samples were quantified by Quant-iT PicoGreen dsDNA Assay (Life Technologies) and normalized to a concentration of 50 pg/ul. Illumina sequencing libraries were prepared from 100–250 pg DNA using the Nextera XT DNA Library Preparation kit (Illumina) according to the manufacturer’s recommended protocol, with reaction volumes scaled accordingly. Prior to sequencing, libraries were pooled by collecting equal volumes (200 nl) of each library from batches of 96 samples. Insert sizes and concentrations for each pooled library were determined using an Agilent Bioanalyzer DNA 1000 kit (Agilent Technologies). Libraries were sequenced on HiSeq2000 or 2500 2x101 to yield ~10 million paired end reads. Post-sequencing de-multiplexing and generation of BAM and FASTQ files were generated using the Picard suite (https://broadinstitute.github.io/picard).

#### Metatranscriptome sequencing

Metatranscriptomes were generated for 855 stool samples, subsampled from metagenomic selections as above. Illumina cDNA libraries were generated using a modified version of the RNAtag-seq protocol^54^. In brief, 500 ng–1 μg of total RNA was fragmented, depleted of genomic DNA, dephosphorylated, and ligated to DNA adapters carrying 5′-AN8-3′ barcodes of known sequence with a 5′ phosphate and a 3′ blocking group. Barcoded RNAs were pooled and depleted of rRNA using the RiboZero rRNA depletion kit (Epicentre). Pools of barcoded RNAs were converted to Illumina cDNA libraries in two main steps: (i) reverse transcription of the RNA using a primer designed to the constant region of the barcoded adaptor with addition of an adaptor to the 3′ end of the cDNA by template switching using SMARTScribe (Clontech) as described^55^; (ii) PCR amplification using primers whose 5′ ends target the constant regions of the 3′ or 5′ adaptors and whose 3′ ends contain the full Illumina P5 or P7 sequences. cDNA libraries were sequenced on the Illumina HiSeq2500 platform to generate ~13 million paired end reads.

#### Viromics

We selected 703 stool samples for viral profiling, following the sample selection used for metatranscriptomics and adjusted slightly only when aliquots were unavailable (Fig. 1c). Viral nucleic acids were extracted using the MagMax Viral RNA Isolation Kit (AM1939, Thermo Fisher Scientific). Viral RNA was reverse transcribed using SuperScript II RT (18064014, Thermo Fisher) and random hexamers. After short molecule and random hexamer removal using ChargeSwitch (CS12000, Thermo Fisher), molecules were amplified and tagged with a BC12-V8A2 construct^56^ using AccuPrimeTM Taq polymerase and cleaned with ChargeSwitch kit.

The resulting viral amplicons were normalized, pooled, and made into an Illumina library without shearing. The library (150–600 bp) was loaded into an Illumina HiSeq 2000 (Illumina, Carlsbad, CA) and sequenced using the 2 × 100 bp chemistry. Reads were demultiplexed into a sample bin using the barcode prefixing read-1 and read-2, allowing zero mismatches. Demultiplexed reads were further processed by trimming off barcodes, semi-random primer sequences, and Illumina adapters. This process used a custom demultiplexer and the BBDuk algorithm included in BBMap (http://sourceforge.net/projects/bbmap). The resulting trimmed data set was analysed using a pipeline created at the Alkek Center for Metagenomics and Microbiome Research at Baylor College of Medicine^57^. In brief, the viral analysis pipeline uses a clustering algorithm creates putative viral genomes using a mapping assembly strategy that leverages nucleotide and translated nucleotide alignment information. Viral taxonomies were assigned using a scoring system that incorporates nucleotide and translated nucleotide alignment results in a per-base fashion and optimizes for the highest resolution taxonomic rank.

### Metabolomics

#### Sample selection, receipt, and storage

Sample selection for metabolomics aimed to obtain only a broad sampling of many subjects. In total, 546 stool samples were selected for profiling (Fig. 1b). A portion of each selected stool sample (40–100 mg) and the entire volume of originating ethanol preservative were stored in 15-ml centrifuge tubes at –80 °C until all samples were collected.

#### Sample processing

Samples were thawed on ice and then centrifuged (4 °C, 5,000*g*) for 5 min. Ethanol was evaporated using a gentle stream of nitrogen gas using a nitrogen evaporator (TurboVap LV; Biotage) and stored at –80 °C until all samples in the study had been dried. Aqueous homogenates were generated by sonicating each sample in 900 μl H_2_O using an ultrasonic probe homogenizer (Branson Sonifier 250) set to a duty cycle of 25% and output control of 2 for 3 min. Samples were kept on ice during the homogenization process. The homogenate for each sample was aliquoted into two 10-µl and two 30-µl samples in 1.5-ml centrifuge tubes for LC–MS sample preparation and 30 µl of homogenate from each sample was transferred into a 50-ml conical tube on ice to create a pooled reference sample. The pooled reference mixture was mixed by vortexing and then aliquoted (100 µl per aliquot) into 1.5-ml centrifuge tubes. Aliquots and reference sample aliquots were stored at –80 °C until LC–MS analyses were conducted.

#### LC–MS analyses

A combination of four LC–MS methods were used to profile metabolites in the faecal homogenates, as previously published^58^; two methods that measure polar metabolites, a method that measures metabolites of intermediate polarity (for example, fatty acids and bile acids), and a lipid profiling method. For the analysis queue in each method, subjects were randomized and longitudinal samples from each subject were randomized and analysed as a group. Additionally, pairs of pooled reference samples were inserted into the queue at intervals of approximately 20 samples for quality control and data standardization. Samples were prepared for each method using extraction procedures that are matched for use with the chromatography conditions. Data were acquired using LC–MS systems comprised of Nexera X2 U-HPLC systems (Shimadzu Scientific Instruments) coupled to Q Exactive/Exactive Plus orbitrap mass spectrometers (Thermo Fisher Scientific). The method details are summarized below.

LC–MS Method 1: HILIC-pos (positive ion mode MS analyses of polar metabolites). LC–MS samples were prepared from stool homogenates (10 µl) by protein precipitation with the addition of nine volumes of 74.9:24.9:0.2 v/v/v acetonitrile/methanol/formic acid containing stable isotope-labelled internal standards (valine-d8, Isotec; and phenylalanine-d8, Cambridge Isotope Laboratories). The samples were centrifuged (10 min, 9,000*g*, 4 °C), and the supernatants injected directly onto a 150 × 2-mm Atlantis HILIC column (Waters). The column was eluted isocratically at a flow rate of 250 µl/min with 5% mobile phase A (10 mM ammonium formate and 0.1% formic acid in water) for 1 min followed by a linear gradient to 40% mobile phase B (acetonitrile with 0.1% formic acid) over 10 min. MS analyses were carried out using electrospray ionization in the positive ion mode using full scan analysis over *m*/*z* 70–800 at 70,000 resolution and 3-Hz data acquisition rate. Additional MS settings are: ion spray voltage, 3.5 kV; capillary temperature, 350 °C; probe heater temperature, 300 °C; sheath gas, 40; auxiliary gas, 15; and S-lens RF level 40.

LC–MS Method 2: HILIC-neg (negative ion mode MS analysis of polar metabolites). LC–MS samples were prepared from stool homogenates (30 µl) by protein precipitation with the addition of four volumes of 80% methanol containing inosine-15N4, thymine-d4 and glycocholate-d4 internal standards (Cambridge Isotope Laboratories). The samples were centrifuged (10 min, 9,000*g*, 4 °C) and the supernatants were injected directly onto a 150 × 2.0-mm Luna NH2 column (Phenomenex). The column was eluted at a flow rate of 400 µl/min with initial conditions of 10% mobile phase A (20 mM ammonium acetate and 20 mM ammonium hydroxide in water) and 90% mobile phase B (10 mM ammonium hydroxide in 75:25 v/v acetonitrile/methanol) followed by a 10-min linear gradient to 100% mobile phase A. MS analyses were carried out using electrospray ionization in the negative ion mode using full scan analysis over *m*/*z* 60–750 at 70,000 resolution and 3 Hz data acquisition rate. Additional MS settings are: ion spray voltage, –3.0 kV; capillary temperature, 350 °C; probe heater temperature, 325 °C; sheath gas, 55; auxiliary gas, 10; and S-lens RF level 40.

LC–MS Method 3: C18-neg (negative ion mode analysis of metabolites of intermediate polarity; for example, bile acids and free fatty acids). Stool homogenates (30 µl) were extracted using 90 µl methanol containing PGE2-d4 as an internal standard (Cayman Chemical Co.) and centrifuged (10 min, 9,000*g*, 4 °C). The supernatants (10 µl) were injected onto a 150 × 2.1-mm ACQUITY BEH C18 column (Waters). The column was eluted isocratically at a flow rate of 450 µl/min with 20% mobile phase A (0.01% formic acid in water) for 3 min followed by a linear gradient to 100% mobile phase B (0.01% acetic acid in acetonitrile) over 12 min. MS analyses were carried out using electrospray ionization in the negative ion mode using full scan analysis over *m*/*z* 70–850 at 70,000 resolution and 3 Hz data acquisition rate. Additional MS settings are: ion spray voltage, –3.5 kV; capillary temperature, 320 °C; probe heater temperature, 300 °C; sheath gas, 45; auxiliary gas, 10; and S-lens RF level 60.

LC-MS Method 4: C8-pos. Lipids (polar and nonpolar) were extracted from stool homogenates (10 µl) using 190 µl isopropanol containing 1-dodecanoyl-2-tridecanoyl-*sn*-glycero-3-phosphocholine as an internal standard (Avanti Polar Lipids; Alabaster, AL). After centrifugation (10 min, 9,000*g*, ambient temperature), supernatants (10 µl) were injected directly onto a 100 × 2.1-mm ACQUITY BEH C8 column (1.7 µm; Waters). The column was eluted at a flow rate of 450 µl/min isocratically for 1 min at 80% mobile phase A (95:5:0.1 v/v/vl 10 mM ammonium acetate/methanol/acetic acid), followed by a linear gradient to 80% mobile phase B (99.9:0.1 v/v methanol/acetic acid) over 2 min, a linear gradient to 100% mobile phase B over 7 min, and then 3 min at 100% mobile phase B. MS analyses were carried out using electrospray ionization in the positive ion mode using full scan analysis over *m*/*z* 200–1,100 at 70,000 resolution and 3 Hz data acquisition rate. Additional MS settings are: ion spray voltage, 3.0 kV; capillary temperature, 300 °C; probe heater temperature, 300 °C; sheath gas, 50; auxiliary gas, 15; and S-lens RF level 60.

#### Metabolomics data processing

Raw LC–MS data were acquired to the data acquisition computer interfaced to each LC–MS system and then stored on a robust and redundant file storage system (Isilon Systems) accessed via the internal network at the Broad Institute. Nontargeted data were processed using Progenesis QIsoftware (v 2.0, Nonlinear Dynamics) to detect and de-isotope peaks, perform chromatographic retention time alignment, and integrate peak areas. Peaks of unknown ID were tracked by method, *m*/*z* and retention time. Identification of nontargeted metabolite LC–MS peaks was conducted by: i) matching measured retention times and masses to mixtures of reference metabolites analysed in each batch; and ii) matching an internal database of >600 compounds that have been characterized using the Broad Institute methods. Temporal drift was monitored and normalized with the intensities of features measured in the pooled reference samples.

### Proteomics

#### Sample selection and LC–MS/MS

Sample selection for proteomics largely followed sample selection for metabolomics (Fig. 1b, c), with slight adjustments when aliquots were unavailable. In total, 447 stool samples were targeted for profiling. From the selected samples, proteins were proteolytically digested using trypsin, and each digest was subjected to automated offline high-pH reversed-phase fractionation with fraction concatenation. LC–MS/MS analysis for each fraction was performed using a Thermo Scientific Q-Exactive Orbitrap mass spectrometer at UCLA, outfitted with a custom-built nano-ESI interface. Samples were loaded onto an in-house packed capillary LC column (70 cm × 75 μm, 3-μm particle size), and data were acquired for 120 min. Precursor MS spectra were collected over 400–2,000 *m*/*z*, followed by data-dependent MS/MS spectra of the twelve most abundant ions, using a collision energy of 30%. A dynamic exclusion time of 30 s was used to discriminate against previously analysed ions.

#### Peptide identification and protein data roll-up

Mass spectra from the resulting analyses were evaluated using the MSGF^+^ software^59^ v10072 using the HMP 1 gut reference genomes (HMP_Refgenome-gut_2015-06-18). In brief, after conversion of the metagenomic assemblies into predicted open reading frames (for example, predicted proteins), libraries were created using the forward and reverse direction to allow determination of FDR. The reverse decoy database allows measurement of the rate of detection of false hits, which in turn allows calculation of FDR and appropriate filtering of the data to maximize real peptide identifications while minimizing spurious ones. MSGF^+^ was then used to search the experimental mass spectra data against both the forward and reverse decoy databases. Cut-offs for data included: MSGF^+^ spectra probability (>1 × 10^10^, equivalent to a BLAST *e* value), mass accuracy (± 20 p.p.m.), protein level FDR of 1% and one unique peptide per protein identification.

### Faecal calprotectin

Faecal calprotectin was quantified for 652 stool samples, which were stored at –80 °C without preservative before processing. Sample selection focused on obtaining a broad survey of all subjects rather than detailed time series (Fig. 1b). Calprotectin was quantified using QUANTA Lite Calprotectin ELISA (Inova Diagnostics 704770) following the manufacturer’s protocol. Between 80 and 120 mg of stool was used for input. Incubation time before stopping the reaction was adjusted to obtain OD_405_ values in the suggested range for assay.

### Biopsy specimen processing

#### Co-isolation of DNA and RNA from frozen tissue

DNA and RNA were extracted from RNA-later-preserved biopsies using the AllPrep DNA/RNA Universal Kit from Qiagen. Biological samples were cut into 20–25-mg pieces on a dry ice batch, then placed in tubes with a steel bead for mechanical homogenization and a highly denaturing guanidine isothiocyanate-containing buffer, which immediately inactivates DNases and RNases to ensure isolation of intact DNA and RNA. After homogenization, the lysate was passed through an AllPrep DNA Mini spin column. This column, in combination with the high-salt buffer, allows selective and efficient binding of genomic DNA. On-column proteinase K digestion in optimized buffer conditions allows purification of high DNA yields from all sample types. The column was then washed and DNA was eluted in TE buffer. Flow-through from the AllPrep DNA Mini spin column was digested by proteinase K in the presence of ethanol. This optimized digestion, together with the subsequent addition of further ethanol, allowed for appropriate binding of total RNA, including miRNA, to the RNeasy Mini spin column. Samples were then digested with DNase I to ensure high-yields of DNA-free RNA. Contaminants were efficiently washed away and RNA was eluted in water.

#### 16S rRNA gene profiling

We selected 178 biopsies for 16S amplicon-based taxonomic profiling. The 16S rRNA gene-sequencing protocol was adapted from the Earth Microbiome Project^60^ and the Human Microbiome Project^61–63^. In brief, bacterial genomic DNA was extracted from the total mass of the biopsied specimens using the MoBIO PowerLyzer Tissue and Cells DNA isolation kit and sterile spatulas for tissue transfer. The 16S rDNA V4 region was amplified from the extracted DNA by PCR and sequenced in the MiSeq platform (Illumina) using the 2 × 250 bp paired-end protocol, yielding pair-end reads that overlapped almost completely. The primers used contained adapters for MiSeq sequencing and single-index barcodes such that PCR products may be pooled and sequenced directly^61^, targeting at least 10,000 reads per sample.

Read pairs were demultiplexed and merged using USEARCH v7.0.1090^64^. Sequences were clustered into OTUs at a similarity threshold of 97% using the UPARSE algorithm^65^. OTUs were subsequently mapped to a subset of the SILVA database^66^ containing only sequences from the V4 region of the 16S rRNA gene to determine taxonomies. Abundances were then recovered by mapping the demultiplexed reads to the UPARSE OTUs, producing the final taxonomic profiles. The 150 samples with ≥1,000 mapped reads were used in downstream analyses.

### Host RNA-seq

#### cDNA library construction

In total, 252 biopsies were selected for transcriptional profiling. Total RNA was quantified using the Quant-iT RiboGreen RNA Assay Kit and normalized to 5 ng/μl. Following plating, 2 μl of ERCC controls (using a 1:1,000 dilution) were spiked into each sample. An aliquot of 200 ng for each sample was transferred into library preparation, which was an automated variant of the Illumina TruSeq Stranded mRNA Sample Preparation Kit. This method preserves strand orientation of the RNA transcript. It uses oligo dT beads to select mRNA from the total RNA sample. It is followed by heat fragmentation and cDNA synthesis from the RNA template. The resultant 500-bp cDNA then goes through library preparation (end repair, base ‘A’ addition, adaptor ligation, and enrichment) using Broad Institute designed indexed adapters substituted in for multiplexing. After enrichment the libraries were quantified using Quant-iT PicoGreen (1:200 dilution). After normalizing samples to 5 ng/μl, the set was pooled and quantified using the KAPA Library Quantification Kit for Illumina Sequencing Platforms. The entire process is in 96-well format and all pipetting is done by either Agilent Bravo or Hamilton Starlet.

#### Illumina sequencing

Pooled libraries were normalized to 2 nM and denatured using 0.1 N NaOH before sequencing. Flowcell cluster amplification and sequencing were performed according to the manufacturer’s protocols using either the HiSeq 2000 or HiSeq 2500. Each run was a 101-bp paired-end with an eight-base index barcode read. Data were organized using the Broad Institute Picard Pipeline which includes de-multiplexing and lane aggregation.

### Blood specimen processing

#### Serological analysis

We analysed 210 serum samples for expression of ANCA, ASCA, anti-OmpC, and anti-CBir1 by ELISA as previously described^67,68^. Antibody levels were determined and the results expressed as ELISA units (EU/ml), which are relative to laboratory standards consisting of pooled, antigen-reactive sera from of patients with well-characterized disease.

#### DNA isolation from whole blood

DNA was extracted using Chemagic MSM I with the Chemagic DNA Blood Kit-96 from Perkin Elmer. The kit combines chemical and mechanical lysis with magnetic bead-based purification. Whole blood samples were incubated at 37 °C for 5–10 min to thaw. The blood was then transferred to a deep well plate with protease and placed on the Chemagic MSM I. The following steps were automated on the MSM I.

M-PVA magnetic beads were added to the blood and protease solution. Lysis buffer was added to the solution and vortexed to mix. The bead-bound DNA was then removed from solution using a 96-rod magnetic head and washed in three ethanol-based wash buffers to eliminate cell debris and protein residue. The beads were then washed in a final water wash buffer. Finally, the beads were dipped in elution buffer to resuspend the DNA. The beads were then removed from solution, leaving purified DNA eluate. The resulting DNA samples were quantified using a fluorescence-based PicoGreen assay.

### Host exome sequencing

Ninety-two host exomes were sequenced from DNA extracts using previously published methods^69^. Whole-exome libraries were constructed and sequenced on an Illumina HiSeq 4000 sequencer with 151-bp paired-end reads. Output from Illumina software was processed by the Picard pipeline to yield BAM files containing calibrated, aligned reads.

#### Library construction

Library construction was performed as described^69^ with some slight modifications. Initial genomic DNA input into shearing was reduced from 3 µg to 50 ng in 10 µl solution and enzymatically sheared. In addition, for adaptor ligation, dual-indexed Illumina paired end adapters were replaced with palindromic forked adapters with unique 8-base index sequences embedded within the adaptor and added to each end.

#### In-solution hybrid selection for exome enrichment

In-solution hybrid selection was performed using the Illumina Rapid Capture Exome enrichment kit with 38 Mb target territory (29 Mb baited). The targeted region includes 98.3% of the intervals in the Refseq exome database. Dual-indexed libraries were pooled into groups of up to 96 samples before hybridization. The liquid handling was automated on a Hamilton Starlet. The enriched library pools were quantified using PicoGreen after elution from streptavadin beads and then normalized to a range compatible with sequencing template denature protocols.

#### Preparation of libraries for cluster amplification and sequencing

Following sample preparation, the libraries prepared using forked, indexed adapters were quantified using quantitative PCR (purchased from KAPA biosystems), normalized to 2 nM using the Hamilton Starlet Liquid Handling system, and pooled by equal volume using the Hamilton Starlet Liquid Handling system. Pools were then denatured using 0.1 N NaOH. Denatured samples were diluted into strip tubes using the Hamilton Starlet Liquid Handling system.

#### Cluster amplification and sequencing

Cluster amplification of the templates was performed according to the manufacturer’s protocol (Illumina) using the Illumina cBot. Flow cells were sequenced on HiSeq 4000 Sequencing-by-Synthesis Kits, then analysed using RTA2.7.3

#### Host genetic data processing

Host genetic exome sequence data were processed using the Broad Institute sequencing pipeline by the Data Sciences Platform (Broad Institute). This was done in three steps: pre-processing (including reads mapping, alignment to a reference genome and data cleanup), variant discovery (including per-sample variant calling and joint genotyping), and variant filtering to produce callset ready for downstream genetic analysis, using Genome Analysis Toolkit (GATK) (detailed documentation at https://software.broadinstitute.org/gatk/documentation/).

### Reduced representation bisulfite sequencing

Reduced representation bisulfite sequencing (RRBS) libraries were prepared for 221 biopsies and 228 blood samples as described previously^70^ with modifications detailed below. In brief, genomic DNA samples were quantified using a Quant-It dsDNA high sensitivity kit (ThermoFisher, Q33120) and normalized to a concentration of 10 ng/μl. A total of 100 ng of normalized genomic DNA was digested with MspI in a 20-μl reaction containing 1 μl MspI (20 U/μl) (NEB, R0106L) and 2 μl of 10× CutSmart Buffer (NEB, B7204S). MspI digestion reactions were then incubated at 37 °C for 2 h followed by a 15 min incubation at 65 °C.

Next, A-tailing reactions were performed by adding 1 μl dNTP mix (containing 10 mM dATP, 1 mM dCTP and 1 mM dGTP) (NEB, N0446S), 1 μl Klenow 3′-5′ exo^-^ (NEB, M0212L) and 1 μl 10× CutSmart Buffer in a total reaction volume of 30 μl. A-tailing reactions were then incubated at 30 °C for 20 min, followed by 37 °C for 20 min, followed by 65 °C for 15 min.

Methylated Illumina sequencing adapters^70^ were then ligated to the A-tailed material (30 μl) by adding 1 μl 10× CutSmart Buffer, 5 μl 10 mM ATP (NEB, P0756S), 1 μl T4 DNA Ligase (2,000,000 U/ml) (NEB, M0202M) and 2 μl methylated adapters in a total reaction volume of 40 μl. Adaptor ligation reactions were then incubated at 16 °C overnight (16–20 h) followed by incubation at 65 °C for 15 min. Adaptor ligated material was purified using 1.2× volumes of Ampure XP according to the manufacturer’s recommended protocol (Beckman Coulter, A63881).

Following adaptor ligation, bisulfite conversion and subsequent sample purification were performed using the QIAGEN EpiTect kit according to the manufacturer’s recommended protocol designated for DNA extracted from FFPE tissues (QIAGEN, 59104). Two rounds of bisulfite conversion were performed yielding a total of 40 μl bisulfite-converted DNA.

In order to determine the minimum number of PCR cycles required for final library amplification, 50 μl PCR reactions containing 3 μl bisulfite-converted DNA, 5 μl 10× PfuTurbo Cx hotstart DNA polymerase buffer, 0.5 μl 100 mM dNTP (25 mM each dNTP) (Agilent, 200415), 0.5 μl Illumina TruSeq PCR primers (25 μM each primer)^70^ and 1 μl PfuTurbo Cx hotstart DNA polymerase (Agilent, 600412) were prepared. Reactions were then split equally into four separate tubes and thermocycled using the following conditions: denature at 95 °C for 2 min followed by *X* cycles of 95 °C for 30 s, 65 °C for 30 s, 72 °C for 45 s (where *X* number of cycles = 11, 13, 15 and 17), followed by a final extension at 72 °C for 7 min. PCR products were purified using 1.2× volumes of Ampure XP and analysed on an Agilent Bioanalyzer using a High Sensitivity DNA kit (Agilent, 5067-4626). Once the optimal number of PCR cycles was determined, 200-μl PCR reactions were prepared using 24 μl bisulfite-converted DNA, 20 μl 10× PfuTurbo Cx hotstart DNA polymerase buffer, 2 μl 100 mM dNTPs (25 mM each), 2 μl Illumina TruSeq PCR primers (25 μM each) and 4 μl PfuTurbo Cx hotstart DNA polymerase with the thermal cycling conditions listed above. PCR reactions were purified using 1.2× volumes of Ampure XP according to the manufacturer’s recommended protocol and analysed on an Agilent Bioanalyzer using a High Sensitivity DNA kit.

RRBS sequencing produced an average of 15.0M reads (s.d. 4.0M reads) over all 504 samples, with 448 (88.9%) samples exceeding 10M reads. Samples were analysed with Picard 2.9.4 using default parameters, resulting in a mean alignment rate to the human genome hg19 of 95.1%. Mean CpG coverage was 8.9× (s.d. 2.1%). As expected, 99.9% (s.d. 0.02%) of non-CpG bases and 49.8% (s.d. 2.8%) of CpG bases were converted.

### Data handling

#### Informatics for microbial community sequencing data

For metagenomes and metatranscriptomes, sequencing reads from each sample in a pool were demultiplexed based on their associated barcode sequence using custom scripts. Up to one mismatch in the barcode was allowed provided it did not make assignment of the read to a different barcode possible. Barcode sequences were removed from the first read as were terminal Gs from the second read that may have been added by SMARTScribe during template switching.

Taxonomic and functional profiles were generated with the bioBakery meta’omics workflow^71^ v0.9.0 (http://huttenhower.sph.harvard.edu/biobakery_workflows). In brief, reads mapping to the human genome were first filtered out using KneadData 0.7.0. Taxonomic profiles of shotgun metagenomes were generated using MetaPhlAn2^72^ v2.6.0, which uses a library of clade-specific markers to provide pan-microbial (bacterial, archaeal, viral, and eukaryotic) profiling (http://huttenhower.sph.harvard.edu/metaphlan2). Functional profiling was performed by HUMAnN2^73^ v0.11.0 (http://huttenhower.sph.harvard.edu/humann2). HUMAnN2 constructs a sample-specific reference database from the pangenomes of the subset of species detected in the sample by MetaPhlAn2 (pangenomes are precomputed representations of the open reading frames of a given species^74^). Sample reads are mapped against this database to quantify gene presence and abundance on a per-species basis. A translated search is then performed against a UniRef-based protein sequence catalogue^75^ (UniRef release 2014_07) for all reads that fail to map at the nucleotide level. The result are abundance profiles of gene families (UniRef90s), for both metagenomics and metatranscriptomics, stratified by each species contributing those genes, and which can then be summarized to higher-level gene groupings such as ECs or KOs.

Sample counts in Fig. 1b represent the numbers of raw products available. To ensure a reasonable read depth in each sample, only samples (metagenomes and metatranscriptomes) with at least 1 million reads (after human filtering) and at least one non-zero microbial abundance detected by MetaPhlAn2 were used in downstream analyses (Fig. 1c and later). In total, this was 1,595 metagenomic and 818 metatranscriptomic samples. Principal coordinates plots were generated with the cmdscale function in the R package stats. Visualizations were principally generated using ggplot2^76^.

#### Species-level meta’omic functional profile summaries

Functional profiles per clade (typically species) were further quantified by summing the total sum-normalized stratified abundance attributed to each organism in the HUMAnN2 functional profiles from both metatranscriptomics and metagenomics. For metatranscriptomic analyses, the expression ratio for the species was then also defined as the ratio between these sums.

#### Metaproteomic gene family functional profiles

Gene family profiles were generated from metaproteomic peptides using UniRef90 identifiers by mapping peptide sequences to the Diamond-annotated reference in HUMAnN2 v0.11.0 with v0.8.22.84^77^. Each peptide was mapped to the UniRef90 with the highest per cent identity (minimum 90% match).

### Statistical methods and association testing

#### PERMANOVA and Mantel tests

Omnibus testing was performed on Bray–Curtis dissimilarity matrices from MGX, MTX, MPX, and biopsy measurements. Functional profiles (MGX, MTX, and MPX) were first summarized to the KO level using HUMAnN2. Profiles were first normalized before calculation of dissimilarities. Dietary distance matrices were calculated by ordering the dietary intake frequencies from less to more frequent, assigning integers to these levels, and calculating the Manhattan distance.

Quantifications of covariation between measurement types in Fig. 1e were done using Mantel tests. To quantify cross-sectional (‘inter-individual’) covariation, we first produced an average profile for each subject by taking the feature-wise mean over all samples from the subject. Subject-subject dissimilarity matrices were then generated and compared using the mantel.rtest function in the R package ape4. To quantify longitudinal covariation, we first generated the complete sample–sample dissimilarity matrix, but only calculate the Mantel test statistic (the Pearson correlation between distances) from distances between samples from the same subject. Significance in this case was assessed using a permutation test with permutations limited within-subject.

Quantifications of variance explained in Fig. 1f were calculated using PERMANOVA with the adonis function in the R package Vegan^78^. Apart from the All row in Fig. 1f, the total variance explained by each variable was calculated independently of other variables (that is, as the sole variable in the model) to avoid issues related to variable ordering, and should thus be considered the total variance explainable by that variable. To account for the repeated measures present for all measurement types tested, relevant permutations were performed blocked within subject for variables that change over time (medication, biopsy location, inflammation status, and dysbiosis). Meanwhile, variables that were constant (or change slowly enough to be considered constant) across samples from the same subject (age, sex, body mass index (BMI), race, recruitment site, diagnosis, and disease location) were first permuted across subjects and samples were relabelled with the variable from their permuted subject. To determine the significance of models including inter-individual variance (the Subject and All rows), permutations were performed freely. For subjects with incomplete records for BMI, we imputed the mean BMI of the remaining population. The All row is the total variance explained when including all other variables in the model.

#### Differential microbiome feature abundance

Differential abundance (DA) analysis of all microbial measurement types (except for viruses, which were modelled as presence/absence binary features) were tested as follows. First, an appropriate transformation/normalization method was applied: arcsine square-root transformation for microbial taxonomic and functional relative abundances, log transformation (with pseudo count 1 for zero values) for metabolite profiles and protein abundances, and log transform with no pseudocount for expression ratios (non-finite values removed). Transformed abundances were then fit with the following per-feature linear mixed-effects model:

$$\begin{array}{l}\,{\rm{feature}} \sim \left({\rm{intercept}}\right)+{\rm{diagnosis}}+{\rm{diagnosis}}/{\rm{dysbiosis}}+{\rm{antibiotic}}\;{\rm{use}}\\ \,+{\rm{consent}}\;{\rm{age}}+\left(1| {\rm{recruitment}}\;{\rm{site}}\right)+\left(1| {\rm{subject}}\right)\end{array}$$

That is, in each per-feature multivariable model, recruitment sites and subjects were included as random effects to account for the correlations in the repeated measures (denoted by (1 | recruitment site) and (1 | subject), respectively) and the transformed abundance of each feature was modelled as a function of diagnosis (a categorical variable with non-IBD as the reference group) and dysbiosis state as a nested binary variable (with non-dysbiotic as reference) within each IBD phenotype (UC, CD, and non-IBD), while adjusting for consent age as a continuous covariate, and antibiotics as as binary covariate. Pearson’s residual values from the above linear mixed effects models were retained for use in subsequent analyses (see ‘Cross-measurement type interaction testing’).

Fitting was performed with the nlme package in R^79^ (using the lme function), where significance of the association was assessed using Wald’s test (except for viruses, where a logistic random effects model was considered with the glmer function from the lmer R package). Nominal *P* values were adjusted for multiple hypothesis testing with a target FDR of 0.25. In order to reduce the effect of zero-inflation in microbiome data, features with no variance or with >90% zeros were removed before fitting linear models. In addition, a variance filtering step was applied to remove features with very low variance. To further remove the effect of redundancy in KO gene family abundances (explainable by at most a single taxon), only features (both DNA and RNA) with low correlation with individual microbial abundances (Spearman correlation coefficient <0.6) were retained.

#### Differential host gene expression

Differentially expressed human genes between disease groups were quantified using a quasi-likelihood negative binomial generalized log-linear model (glmQLFit), implemented in the edgeR package in R^80,81^. Analysis was performed separately for each section of the intestine on genes with at least 2 CPM (counts per million) in 10 or more samples, with significance threshold FDR *P* < 0.05 and >1.5 log-fold change. Gene enrichment analysis was performed on differentially expressed genes against the KEGG database^82^ using a one-sided hypergeometric test in the package limma^83^.

#### Associations with host gene expression

Associations between host gene expression and biopsy taxonomic profiles were assessed using partial Spearman correlation, accounting for BMI, age at consent, sex and diagnosis. Association testing was performed for each biopsy location independently, as biopsy location was shown to heavily influence expression profiles (Figs. 1f, 4a, b). This simpler method was used rather than the more complex procedure outlined above for microbial measurement types since host gene expression, once filtered by biopsy location, do not have the same repeat measures problem as the microbial measurement types, allowing a simpler test.

#### Genetic associations

Genetic principal components for IBDMDB subjects as well as 1,000 Genomes subjects^84^ were calculated for a set of independent SNPs overlapping between the two data sets and pruned on the basis of linkage disequilibrium (LD). Pruning was first performed in HMP2 using the –indep-pairwise 1500 150 0.1 command in PLINK^85^ by calculating LD (*r*^2^) for each pair of SNPs within a window of 1,500 SNPs, removing one of a pair of SNPs if *r*^2^ > 0.1 and repeating this procedure by shifting the window 150 SNPs forward. We then used the 1,000 Genomes reference phase 3 version 5a data for 2,504 participants (http://bochet.gcc.biostat.washington.edu/beagle/1000_Genomes_phase3_v5a) to merge with the HMP2 pruned data, resulting in 7,227 overlapping independent SNPs. Using these, we performed genome-wide estimation of identity-by-descent allele sharing on the combined data set using the –genome function in PLINK, followed by calculation of genetic principal components using the –cluster–mds-plot function for the first two principal components (Fig. 1a).

For association analyses, we used first 20 genetic principal components as covariates, obtained from the same identity-by-descent sharing matrix using the –cluster–pca 20 function. We targeted associations in five loci that had strong previously reported associations with IBD and/or have been implicated in microbial interactions^86–88^. To avoid confounding by ancestry, we restricted the analysis to subjects of European ancestry, excluding eight subjects with exomes available from other ancestral backgrounds. When available, we used reported SNPs that had minor allele frequency of at least 5% and Hardy–Weinberg equilibrium *P* < 5 × 10^−5^. If not, we used close proxies (LD *r*^2^ < 0.8 in CEU population using 1,000 genomes phase 3 version 5 reference via http://analysistools.nci.nih.gov/LDlink (*MST1*, *FUT2*, *IRGM*, *NKX2-3*) or SNPs from the gnomAD browser at http://gnomad.broadinstitute.org (*PTGER4*)).

We used the following linear mixed effect model with the SNP as a predictor variable, coded with an additive genetic model with the outcome as the arcsine-square root transformed microbial relative abundance measured from stool metagenomes. Age, sex, antibiotic and immunosuppressant use, and the first 20 genetic principal components (PCs) were fitted as covariates with subjects as the random effect:

$$\begin{array}{l}{\rm{taxon}} \sim {\rm{intercept}}+{\rm{SNP}}+{\rm{antibiotic}}\;{\rm{use}}+{\rm{sex}}+{\rm{age}}\\ \,+{\rm{recruitment}}\;{\rm{site}}+{\rm{PC}}1-{\rm{PC}}20+\left(1| {\rm{subject}}\right)\end{array}$$

Optimization was performed using the lme function (from the nlme R package), with *P* values calculated using the Wald test.

Associations between the rs1042712 SNP of the *LCT* locus^89^ and self-reported milk intake from dietary recall forms were tested using the same mixed effect model. Reported dairy intake options were assigned the following numeric values for regression: 1 (‘yesterday, 3 or more times’), 2 (‘yesterday, 1 to 2 times’); 3 (‘within the past 2 to 3 days’), 4 (‘within the past 4 to 7 days’), and 5 (‘did not consume in last 7 days’).

#### Density ridgeline plots of differentially abundant features

To visualize the abundances of features that showed significantly different abundances by one of the tests above (Fig. 2, Extended Data Fig. 4), the bandwidth for kernel density estimation was selected independently for the portion of each feature above the detection limit (non-‘zero’) using the Sheather and Jones method^90^. Density estimates were scaled such that the maximum density for the plot spanned the distance between baselines for a given disease group. Samples below the detection limit are represented as barplots on the left, where a bar that spans the distance between baselines for a disease group represents 100% zeros. Density estimates were then additionally scaled by the fraction of non-zero samples such that relative differences in densities between groups with differing fractions of zeros are accurately represented. For both density estimates and fraction of zeros, samples were weighted by the inverse of the number of samples obtained from that subject, to avoid biasing estimates towards subjects with more densely sampled time series.

### Dysbiosis analyses

#### Dysbiosis score

To identify samples with highly divergent (dysbiotic) metagenomic microbial compositions, as a complement to baseline disease diagnosis, we defined a dysbiosis score based on Bray–Curtis dissimilarities to non-IBD metagenomes. First, a ‘reference set’ of samples was constructed from non-IBD subjects by taking all samples after the 20th week after the subject’s first stool sample. This was chosen because a subset of the non-IBD subjects at the start of their respective time series may not yet have overcome any gastrointestinal symptoms that triggered the initial visit to a doctor, though these were ultimately not caused by IBD. The dysbiosis score of a given sample was then defined as the median Bray–Curtis dissimilarity to this reference sample set, excluding samples that came from the same subject (Fig. 2c).

To identify samples that were highly divergent from the reference set, we thresholded the dysbiosis score at the 90th percentile of this score for non-IBD samples. This therefore identifies samples with a feature configuration that has a less than 10% probability of occurring in a participant without IBD. By this measure, 272 metagenomes were classified as dysbiotic. Samples from participants with CD or UC were overrepresented in the dysbiotic set, with 24.3% and 11.6% of their samples classified as dysbiotic, respectively. As expected, these samples also tended to locate in the extremes of the taxonomic ordination based on metagenomes (Extended Data Fig. 3b, c). Dysbiosis was unevenly distributed among subjects (Extended Data Fig. 3d), with some subjects remaining dysbiotic for all or most of their time series, while others remained non-dysbiotic for their entire time series.

To lend additional support to the definition of dysbiosis (that is, as outliers by one type of microbiome profile), we tested the concordance between dysbiosis classifications made using the same statistical definition, but applied to metabolomic rather than taxonomic profiles. That is, we defined a metabolomic dysbiosis score as the median Bray–Curtis dissimilarity of one metabolomic profile to the non-IBD metabolomic profiles (after the 20th week), and defined the dysbiosis threshold as the 90th percentile of this distribution among non-IBD metabolomic profiles. We then compared these dysbiosis classifications with those from the nearest metagenomic sample (up to two weeks, see ‘Cross-measurement type temporal matching’) and found that dysbiotic samples identified by metagenomics were 4.6 times more likely to be dysbiotic by metabolomics (Fisher’s exact *P* = 5.9 × 10^−9^), showing that dysbiosis measurements are highly consistent across measurement types.

To test the sensitivity of the dysbiosis classification to the choice of reference data set, we also performed the dysbiosis classification using the HMP1-II stool samples^10^ as the reference sample set instead of the non-IBD samples. The resulting dysbiosis scores (Extended Data Fig. 3e) were highly concordant (Spearman *ρ* = 0.86; *P* < 2.2 × 10^−16^), as were the dysbiosis classifications (odds ratio of 56; Fisher’s exact *P* < 2.2 × 10^−16^). This shows that, despite the inclusion of subjects with other conditions in the non-IBD group here, as well as large differences in measurement technologies between the data sets, the dysbiosis classification is highly robust. Furthermore, 43 out of 426 (10.1%) of non-IBD samples were classified as dysbiotic using the HMP1-II samples as reference, falling remarkably close to the 10% expected by the definition and showing that the enrichment of IBD samples in the dysbiotic set is not simply a consequence of the definition.

#### Dysbiosis durations and intervals

Samples of the dysbiosis durations and intervals were obtained by taking the difference in time between stool metagenomes in which the dysbiosis state changes, that is, the time from the first dysbiotic sample in an excursion into dysbiosis until the next non-dysbiotic sample was taken as one sample of the dysbiosis duration distribution. If the subject’s time series ended before this transition occurred, this resulted in a ‘censored’ duration or interval (Fig. 2e). Estimates of the durations of and time between dysbioses were then obtained from a censored maximum likelihood estimator for the mean of an exponential distribution. This incorporates the censored durations and intervals into the estimate to avoid underestimating these durations owing to limited observation times.

#### Association of dysbiosis with disease location

We tested for a relationship between the Montreal disease location classification in CD and periods of dysbiosis to ensure that dysbiosis was not simply detecting different disease locations. For this, an *F*-test of no significance was used with a Kenward–Roger approximation of degrees of freedom^91^ in a logistic random effects regression that models dysbiosis as the binary outcome with subjects as a random effect and disease location as covariate, as implemented in the function glmer in the R package lme4. Only individuals with CD were considered.

### Temporal analyses

#### Power-law fits to Bray–Curtis dissimilarities

Power-law fits to species-level metagenomic, metatranscriptomic, and metabolite Bray–Curtis dissimilarities (Fig. 3a, Extended Data Fig. 5a) were performed by fitting a power-law curve with free intercept by least-squares using the neldermead function from the R package nloptr. Significance was assessed using an *F*-test to compare the fit model with a flat line. Significance of the difference of the fit between disease groups was also assessed using an *F*-test, comparing a model jointly fit to both disease groups with separate fits to each group.

#### Microbiome shifts

A microbiome ‘shift’ was defined as having occurred between two consecutive time points from the same person if the Bray–Curtis dissimilarity between their profiles was more likely to have come from a comparison between samples from different people rather than from the same person (Extended Data Fig. 5b). As an individual’s microbial profile naturally changes over time^10,92^, the Bray–Curtis threshold at which this occurs will increase with the time difference between samples. To determine these thresholds, kernel density estimates were generated for the distribution of Bray–Curtis dissimilarities between profiles from different individuals without IBD and between samples from the individuals without IBD at a range of time differences, using the density function in R. The point at which the inter-individual density estimate exceeded the intra-individual density estimate was then taken as the threshold to define a ‘shift’, with the additional constraint that this must be a monotonically increasing function of the time difference between samples (Fig. 3a). Metabolomic shifts were defined similarly, although owing to the more sparse temporal sampling of the metabolomics data (Extended Data Fig. 1) and lack of a strong upward trend in Bray–Curtis dissimilarities with time difference (Fig. 3a), only a single threshold was used based on the distribution of Bray–Curtis dissimilarities from comparisons within-subject over time in participants without IBD. Heatmaps of shift differences were generated using the R package pheatmap 1.0.10^93^.

#### Longitudinal multi-omic study design

Owing to the large variation in microbial profiles between people (Supplementary Fig. 2), with relatively smaller variation within subjects over time (Fig. 3a), longitudinal study designs have the potential to be higher-powered than purely cross-sectional studies, particularly in their ability to self-control individuals and to capture transitions between phenotypes (or after interventions) of interest^94^. Here, although some subjects remained in a dysbiotic state far longer than others (Extended Data Fig. 3d), the heterogeneity observed was enough to discover differences in measurement types other than where dysbiosis was defined. For example, subjects who had unusual, disease-associated microbiome taxonomic profiles also proved to have generally shifted serological and/or metabolomic profiles at corresponding time points. Noting these dysbiotic time points offered a complementary set of differences to what was visible cross-sectionally (Fig. 2).

Among microbially related measurements, metabolomics provided the most robust separation between disease and dysbiosis groups, possibly because it integrated a combination of host, microbial, and dietary differences (Fig. 1e, f). Thus, despite the challenges presented by untargeted metabolomics, such as unknown compound identification, the presence of redundant and background signals, and the complexity of the stool matrix, this measurement type often provides a robust characterization of subjects, their disease state, and individual small molecules that interface between host and microbiome. Conversely, the current state of viromics assays and reference databases makes this more challenging to work with, although the importance of the virome in microbial community dynamics^95^ will make this an extremely interesting feature space going forward.

All longitudinal microbial measurement types showed significant variation within two weeks (Fig. 3a, Extended Data Fig. 5a). This suggests that even higher sampling rates may be needed to catch relevant microbial variation, particularly before the onset of more severe clinical symptoms. Our sampling protocol also did not account for other potential sources of within-subject variation, such as transit time or the precise portion of each whole stool that was sampled. In this data set, we thus cannot distinguish between temporal and technical variation within subjects. To mitigate the higher costs associated with processing additional samples, a future study aiming to achieve higher temporal resolution might proceed in two phases, thanks to the coupling between data types (Fig. 1e): first, collect samples at a higher frequency, and process these with metagenomic or 16S sequencing. Then proceed with more expensive and detailed data generation only for samples taken specifically around periods of interest, such as periods of dysbiosis identified during the first stage. To this end, the sampling rate can also be tuned to target a particular probability of missing a dysbiosis period.

### Integrative analyses

#### Lenient cross-measurement type temporal matching

For comparison between multiple measurement types, we first constructed sets of samples corresponding to the same biosample across data sets. However, exact matches were not always possible, for example, owing to specimen limitations during sample selection (see ‘Sample selection’) or to samples that failed quality control. In these cases, matching sample sets across data sets were created using nearby samples. During this process, a degree of leniency was allowed in the matching, allowing samples up to a given time difference (two or four weeks) apart to be ‘matched’. To perform this matching (sample numbers in Fig. 1c and Extended Data Fig. 1b), we used the following algorithm.

For a given set of measurement types to be matched for a particular subject, find the first time window in which all measurement types have at least one produced sample. Next, within this window, find the time point with the most measurement types produced; in the event of a tie, select earlier time points. Finally, for each data set, select the nearest sample to this target time point that is within the time window, breaking ties towards earlier time points. This set of selected samples comprises one ‘matched’ sample. For each data set, all samples up to and including the later of the selected sample or the target time point are then disregarded from future consideration (and thus any sample will be included in at most only one matched time point). This process is repeated for each subject until no such window exists.

#### Cross-measurement type interaction testing

Significant associations between features from multiple measurement types were identified using two different models: an ‘unadjusted’ model of associations that are mainly due to dysbiosis, and an ‘adjusted’ model that emphasizes associations in addition to those that are dysbiosis-linked. Associations in both cases incorporated features from ten data sets: metagenomic species, species-level transcription ratios, functional profiles at the EC level (MGX, MTX and MPX), metabolites, host transcription (rectal and ileal separately), serology and faecal calprotectin.

To detect adjusted associations, we first obtained residuals of features from the above data types fit to a mixed-effects model including subjects as random effects as above for the differential abundance testing (or a simple linear model without the random effects when only baseline samples were used) and adjusting for age, sex, diagnosis, dysbiosis status, antibiotic and immunosuppressant use, and bowel surgery status. Residuals from subjects with fewer than four samples in their time series for the measurement type were ignored, and for measurements with no longitudinal samples (for example, serology and host transcriptomics measurements), the residualization was repeated with the first available samples using a simple linear model without random effects. This allowed the identification of significant (FDR *P* < 0.05 for most measurement types, *P* < 0.25 for serology) Spearman associations using HAllA 0.8.17 (hierarchical all-against-all association testing, http://huttenhower.sph.harvard.edu/halla, Supplementary Table 35). As subject-specific random effects and covariate effects were removed from these residuals, the resulting correlations are likely to be independent of all sources of inter-individual variation as well as confounding effects due to the covariates. See Fig. 4c and Extended Data Figs. 7–9 for summary visualizations of these results. Similarly, unadjusted associations were identified using the same procedure, but without including dysbiosis as a covariate (Supplementary Table 36). Network visualization was done using Cytoscape^96^ 3.6.0.

### Reporting summary

Further information on research design is available in the Nature Research Reporting Summary linked to this paper.

## Online content

Any methods, additional references, Nature Research reporting summaries, source data, statements of data availability and associated accession codes are available at 10.1038/s41586-019-1237-9.

## Supplementary information

Supplementary Discussion This file contains Supplementary Discussion and associated references.

Reporting Summary

Supplementary Figure 1: Abundances of enzymes and their associated metabolites The relationship between the abundances of ECs in metagenomes (MGX), metatranscriptomes (MTX), or proteomes (MPX) are shown against each other and with the metabolites which the EC’s associated reaction either consumes or produces based on the MetaCyc database. The ratio between transcript relative abundance and genomic relative abundance (MTX/MGX) is also shown. MetaCyc unique IDs for the reaction and for the metabolite are shown in brackets. Only features for which MBX and at least one other measurement type have at least 10% prevalence are shown.

Supplementary Figure 2: Per-subject summaries of data available in the IBDMDB Summaries are shown for the 111 subjects for which at least two metagenomes are available. On the left, short-term dietary information collected with each stool sample is shown, along with information on medication use, hospitalization, disease severity scores including calprotectin, HBI/SCCAI, and the dysbiosis score, antibody titers measured from sera, and final reads gathered from metagenomic and metatranscriptomic sequencing (along with the fraction of human reads filtered out during QC). Middle panels provide summaries of the data gathered from microbial measurements, and include profiles for metagenomic and metatranscriptomic taxa, their aerotolerance, viruses, transcribed KOs, metabolites, and proteins. Right panels display the locations of the subject’s samples in the Principal Coordinates Plots from Extended Data Fig. 2. Samples are colored according to their dysbiosis classification (red dysbiotic, blue non-dysbiotic), and connected in sequence by lines. All profiles are available through the IBDMDB at http://ibdmdb.org.

Supplementary Figure 3: Distributions of subject-associated metadata The distribution of each associated metadatum is shown across subjects (“per Participant ID”), biosamples (“per site_sub_coll”), and generated profiles (“per row”). Numeric fields are displayed as histograms.

Supplementary Tables This zipped folder contains Supplementary Tables 1-36 and legends.

## Figures and Tables

**Extended Data Table 1:**
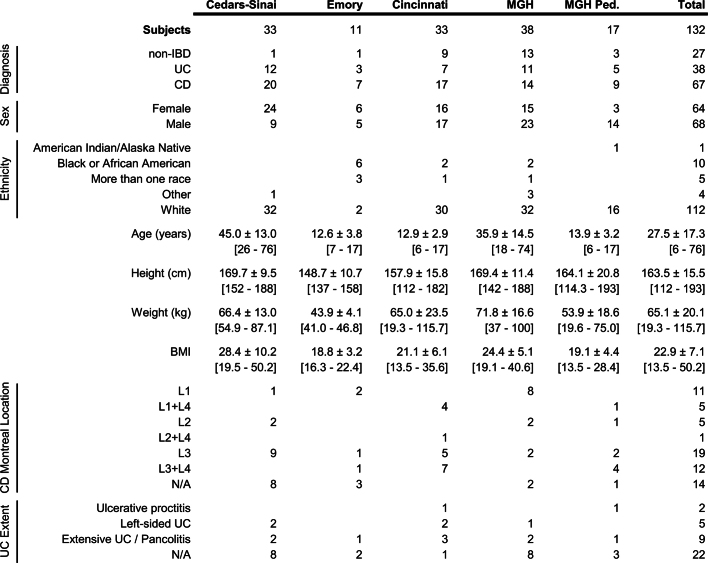
IBDMDB cohort characteristics

Breakdown of the cohort by diagnosis, sex, ethnicity, physical characteristics, and disease location for each clinical site. Numeric data are summarized as mean ± s.d., with the range given by [min–max], and with blank cells for NA (not applicable). Subject counts for disease locations are subsets of the respective diseases.

## Data Availability

Protocols and data (both raw and summarized to data type-dependent profiles) are available at the IBDMDB website (https://ibdmdb.org), the HMP DACC web portal (https://www.hmpdacc.org/ihmp/), and Qiita^97^ (https://qiita.ucsd.edu/). Sequence data are available from SRA BioProject PRJNA398089. Expression data have been deposited in the NCBI Gene Expression Omnibus^98^ and is accessible through GEO Series accession number GSE111889. Metabolomics data are available at the NIH Common Fund’s Metabolomics Data Repository and Coordinating Center (supported by NIH grant U01-DK097430) website, the Metabolomics Workbench (http://www.metabolomicsworkbench.org), where it has been assigned Project ID PR000639. Mass spectrometry proteomics data have been deposited to the ProteomeXchange Consortium via the PRIDE^99^ partner repository with the data set identifiers PXD008675 and 10.6019/PXD008675. Reprints and permissions information is available at www.nature.com/reprints.

## Code availability

Bioinformatics workflows for metagenomics and metatranscriptomics data are available at https://bitbucket.org/biobakery/hmp2_workflows. Analysis scripts are available at https://bitbucket.org/biobakery/hmp2_analysis.
