# Supplementary material for: Multi-omics of the gut microbial ecosystem in inflammatory bowel diseases
Source: Nature. 2019 May 29;569(7758):655–62. doi: 10.1038/s41586-019-1237-9 (PMC6650278; doi:10.1038/s41586-019-1237-9)
Supplement: Supplementary file 1 — This file contains Supplementary Discussion and associated references. [file 41586_2019_1237_MOESM1_ESM.pdf]

nature

SUPPLEMENTARY INFORMATION

https://doi.org/10.1038/s41586-019-1237-9

In the format provided by the authors and unedited.

Multi-omics of the gut microbial ecosystem in inflammatory bowel diseases

Jason Lloyd-Price[1,2], Cesar Arze[2], Ashwin N. Ananthakrishnan[3], Melanie Schirmer[1,3], Julian Avila-Pacheco[4], Tiffany W. Poon[1], Elizabeth Andrews[3], Nadim J. Ajami[5], Kevin S. Bonham[1,2], Colin J. Brislawn[6], David Casero[7], Holly Courtney[3], Antonio Gonzalez[8], Thomas G. Graeber[9], A. Brantley Hall[1], Kathleen Lake[10], Carol J. Landers[11], Himel Mallick[1,2], Damian R. Plichta[1], Mahadev Prasad[12], Gholamali Rahnavard[1,2], Jenny Sauk[13], Dmitry Shungin[1,14], Yoshiki Vázquez-Baeza[15,16], Richard A. White III[6], IBDMDB Investigators[17], Jonathan Braun[7], Lee A. Denson[10,18], Janet K. Jansson[6], Rob Knight[8,16,19], Subra Kugathasan[12], Dermot P. B. McGovern[11], Joseph F. Petrosino[5], Thaddeus S. Stappenbeck[20], Harland S. Winter[21,22], Clary B. Clish[4], Eric A. Franzosa[2], Hera Vlamakis[1], Ramnik J. Xavier[1,3,23,24] & Curtis Huttenhower[1,2,24]*

[1]Infectious Disease and Microbiome Program, Broad Institute of MIT and Harvard, Cambridge, MA, USA. [2]Department of Biostatistics, Harvard T. H. Chan School of Public Health, Boston, MA, USA. [3]Gastroenterology, Massachusetts General Hospital, Boston, MA, USA. [4]Metabolomics Platform, Broad Institute of MIT and Harvard, Cambridge, MA, USA. [5]Molecular Virology and Microbiology, Baylor College of Medicine, Houston, TX, USA. [6]Earth and Biological Sciences Directorate, Pacific Northwest National Lab, Richland, WA, USA. [7]Department of Pathology and Laboratory Medicine, David Geffen School of Medicine, University of California Los Angeles, Los Angeles, CA, USA. [8]Department of Pediatrics, University of California San Diego, La Jolla, CA, USA. [9]Molecular and Medical Pharmacology, University of California Los Angeles, Los Angeles, CA, USA. [10]Department of Pediatrics, Cincinnati Children's Hospital Medical Center, Cincinnati, OH, USA. [11]F. Widjaja Foundation Inflammatory Bowel and Immunobiology Research Institute, Cedars-Sinai Medical Center, Los Angeles, CA, USA. [12]Department of Pediatrics, Emory University, Atlanta, GA, USA. [13]Vatche and Tamar Manoukian Division of Digestive Diseases, University of California Los Angeles, Los Angeles, CA, USA. [14]Department of Odontology, Umeå University, Umeå, Sweden. [15]Jacobs School of Engineering, University of California San Diego, La Jolla, CA, USA. [16]Center for Microbiome Innovation, University of California San Diego, La Jolla, CA, USA. [17]A list of participants and their affiliations appears at the end of the paper. [18]Department of Pediatrics, University of Cincinnati College of Medicine, Cincinnati, OH, USA. [19]Department of Computer Science and Engineering, University of California San Diego, La Jolla, CA, USA. [20]Department of Pathology & Immunology, Washington University, St. Louis, MO, USA. [21]Department of Pediatrics, MassGeneral Hospital for Children, Boston, MA, USA. [22]Department of Pediatrics, Harvard Medical School, Boston, MA, USA. [23]Center for Microbiome Informatics and Therapeutics, Massachusetts Institute of Technology, Cambridge, MA, USA. [24]These authors jointly supervised this work: Ramnik J. Xavier & Curtis Huttenhower. *e-mail: chuttenh@hsph.harvard.edu

Supplementary Discussion for "Multi'omics Detail the Gut Microbial Ecosystem in Inflammatory Bowel Disease"

Genetic associations in the IBDMDB

While this cohort was not designed for genetic association discovery and is not powered for this task, exome sequencing of 92 subjects provides the opportunity to integrate with larger populations in the future. To validate these data, we examined the influence of 5 known IBD-related loci on the microbiome (*MST1, NKX2-3, FUT2, IRGM* and *PTGER4*[1-3]). As expected, no associations between these loci and metagenomic species abundances were significant after multiple hypothesis correction (linear mixed effect regression with Wald test, FDR $p<0.05$, **Supplementary Table S34**, **Methods**). However, the strongest association found (nominal $p=0.002$) was between *Parabacteroides distasonis*, an obligate anaerobe that is reduced in dysbiotic CD, and a *NKX2-3* locus, reported to control intestinal epithelial cells differentiation and lymphocyte migration[4,5] (**Extended Data Fig. 6C**). As a positive control for robustness of the genetic data for future use, we further replicated the association between the rs1042712 SNP at the *LCT* locus[6] and self-reported dietary recall (accompanying biweekly stool samples) of milk intake ($p=0.028$; **Extended Data Fig. 6D**).

"Unadjusted" network analysis

We identified associations among features in the microbiome that did take dysbiosis into account, resulting in a second network using the same methodology but without adjusting for dysbiosis ("unadjusted"). This network included fewer associations and features compared to the adjusted network, totaling 44,159 edges among 3,001 nodes (**Extended Data Fig. 9**, **Supplementary Table S36**), though it contained additional connections especially with biopsy-derived host gene expression (**Extended Data Fig. 7B**). This component was highly associated with metagenomic functional profiles: human transcripts correlated with 27 metagenomic ECs, in addition to 4 taxonomic abundances. Highly-connected host genes included interleukin 8 (*IL8*), a key mediator associated with inflammation, and *OSM* and *SPP1*, both of which are involved in the regulation of other interleukins (specifically, *IL6* and *IL12*)[7]. As these networks regress out as many confounding effects as we have access to, their associations are arguably much closer to "mechanistic" relationships than a typical non-interventional study, particularly given that longitudinal sampling means these associations must exist over time within-subject. Hub features thus represent an additional prioritization of features to target to either correct IBD-associated dysbiosis, or to at least alleviate symptoms.

References

1 Jostins, L. *et al.* Host-microbe interactions have shaped the genetic architecture of inflammatory bowel disease. *Nature* **491**, 119-124, doi:10.1038/nature11582 (2012).

2 Liu, J. Z. *et al.* Association analyses identify 38 susceptibility loci for inflammatory bowel disease and highlight shared genetic risk across populations. *Nat Genet* **47**, 979-986, doi:10.1038/ng.3359 (2015).

3 Hall, A. B., Tolonen, A. C. & Xavier, R. J. Human genetic variation and the gut microbiome in disease. *Nat Rev Genet*, doi:10.1038/nrg.2017.63 (2017).

4 Pabst, O., Forster, R., Lipp, M., Engel, H. & Arnold, H. H. NKX2.3 is required for MAdCAM-1 expression and homing of lymphocytes in spleen and mucosa-associated lymphoid tissue. *EMBO J* **19**, 2015-2023, doi:10.1093/emboj/19.9.2015 (2000).

5 Wellcome Trust Case Control Consortium. Genome-wide association study of 14,000 cases of seven common diseases and 3,000 shared controls. *Nature* **447**, 661-678, doi:10.1038/nature05911 (2007).

6 Enattah, N. S. *et al.* Identification of a variant associated with adult-type hypolactasia. *Nat Genet* **30**, 233-237, doi:10.1038/ng826 (2002).

7 Turner, M. D., Nedjai, B., Hurst, T. & Pennington, D. J. Cytokines and chemokines: At the crossroads of cell signalling and inflammatory disease. *Biochim Biophys Acta* **1843**, 2563-2582, doi:10.1016/j.bbamcr.2014.05.014 (2014).
