# Supplementary material for: Multi-omics of the gut microbial ecosystem in inflammatory bowel diseases
Source: Nature. 2019 May 29;569(7758):655–62. doi: 10.1038/s41586-019-1237-9 (PMC6650278; doi:10.1038/s41586-019-1237-9)
Supplement: Supplementary file 2 — Reporting Summary [file 41586_2019_1237_MOESM2_ESM.pdf]

nature research

Corresponding author(s): Curtis Huttenhower

Life Sciences Reporting Summary

Nature Research wishes to improve the reproducibility of the work that we publish. This form is intended for publication with all accepted life science papers and provides structure for consistency and transparency in reporting. Every life science submission will use this form; some list items might not apply to an individual manuscript, but all fields must be completed for clarity.

For further information on the points included in this form, see Reporting Life Sciences Research. For further information on Nature Research policies, including our data availability policy, see Authors & Referees and the Editorial Policy Checklist.

Please do not complete any field with "not applicable" or n/a. Refer to the help text for what text to use if an item is not relevant to your study. For final submission: please carefully check your responses for accuracy; you will not be able to make changes later.

Experimental design

1. Sample size

Describe how sample size was determined.

The target sample size calculated for at least n=72 subjects with repeated measures was designed to have power of 0.9 to detect 1) between-group differences in taxon abundance (repeated measures ANOVA, group $F > 0.4$), 2) differentially expressed transcripts (Edland's test for a linear mixed model with random slope, $d > 0.07$), and 3) multi'omic correlations (Pearson correlation, $r > 0.6$). Power calculations incorporated conservative Bonferroni p-value correction, with numbers of post-QC microbial features and within-sample correlations estimated from previous microbiome studies.

2. Data exclusions

Describe any data exclusions.

Potential subjects were excluded from the study if they were unable or did not consent to provide tissue, blood, or stool, were pregnant, had a known bleeding disorder or an acute gastrointestinal infection, were actively being treated for a malignancy with chemotherapy, were diagnosed with indeterminate colitis, or had a prior, major gastrointestinal surgery such as an ileal/colonic diversion or j-pouch. These criteria were established prior to the study start. Samples were filtered based on data type-specific quality control measures. For metagenomes and metatrascriptomes, samples were required to have >1M reads and at least one species detected by MetaPhlAn2.

3. Replication

Describe the measures taken to verify the reproducibility of the experimental findings.

The study was a large-scale clinical cohort and we did not attempt to replicate all aspects of sample collection and data generation. However, data and source code for computational tools used are available to the public and therefore all of our analysis can be reproduced using our methods or re-analyzed using other methods. When possible, we refer to existing literature that supports our findings. Multiple pilot studies as well as technical replicates covering a subset of samples are also available, and these data were successfully integrated into subsequent multi-batch analyses, ensuring that data generation methods produced reproducible results.

4. Randomization

Describe how samples/organisms/participants were allocated into experimental groups.

Experimental groups could not be randomized as they depended on diagnosis. Participants were recruited into the three disease groups as available from each of the recruitment sites. Upon enrollment, an initial colonoscopy was performed to determine study strata. Subjects not diagnosed with IBD based on endoscopic and histopathologic findings were classified as "non-IBD" controls, including the aforementioned healthy individuals presenting for routine screening, and those with more benign or non-specific symptoms. This creates a control group that, while not completely "healthy", differs from the IBD cohorts specifically by clinical IBD status.

5. Blinding

Describe whether the investigators were blinded to group allocation during data collection and/or analysis.

Samples were collected by clinical staff who were not blinded as they needed to examine patients to determine which experimental group they should be allocated to. All data were generated by investigators that were blinded to the metadata. Once data were generated, computational analysis was performed with all of the necessary clinical information to test between groups.

Note: all in vivo studies must report how sample size was determined and whether blinding and randomization were used.

6. Statistical parameters

For all figures and tables that use statistical methods, confirm that the following items are present in relevant figure legends (or in the Methods section if additional space is needed).

n/a	Confirmed	
☐	☒	The exact sample size (*n*) for each experimental group/condition, given as a discrete number and unit of measurement (animals, litters, cultures, etc.)
☐	☒	A description of how samples were collected, noting whether measurements were taken from distinct samples or whether the same sample was measured repeatedly
☒	☐	A statement indicating how many times each experiment was replicated
☐	☒	The statistical test(s) used and whether they are one- or two-sided<br>*Only common tests should be described solely by name; describe more complex techniques in the Methods section.*
☐	☒	A description of any assumptions or corrections, such as an adjustment for multiple comparisons
☐	☒	Test values indicating whether an effect is present<br>*Provide confidence intervals or give results of significance tests (e.g. P values) as exact values whenever appropriate and with effect sizes noted.*
☐	☒	A clear description of statistics including central tendency (e.g. median, mean) and variation (e.g. standard deviation, interquartile range)
☐	☒	Clearly defined error bars in all relevant figure captions (with explicit mention of central tendency and variation)

See the web collection on statistics for biologists for further resources and guidance.

Software

Policy information about availability of computer code

7. Software

Describe the software used to analyze the data in this study.	Analysis of mass spectra: MSGF+ software v10072<br>Sequence processing: Picard 2.9.4<br>Metagenome and metatranscriptome profiles generated using bioBakery meta'omics workflow v0.9.0 (Preprocessing: KneadData 0.7.0; Taxonomic profiles: MetaPhlAn2 v2.6.0; Functional profiles: HUMAnN2 v0.11.0 with UniRef release 2014_07, Diamond v0.8.22.84)<br>Viral profiles: VirMAP<br>16S analysis: USEARCH v7.0.1090<br>Data analysis and plotting: R (main packages: edgeR, nlme, ggplot2, ggridges, pheatmap, vegan, tsne), Julia plots package<br>Network construction and visualization: HAllA 0.8.17, Cytoscape 3.6.0

For manuscripts utilizing custom algorithms or software that are central to the paper but not yet described in the published literature, software must be made available to editors and reviewers upon request. We strongly encourage code deposition in a community repository (e.g. GitHub). *Nature Methods* guidance for providing algorithms and software for publication provides further information on this topic.

Materials and reagents

Policy information about availability of materials

8. Materials availability

Indicate whether there are restrictions on availability of unique materials or if these materials are only available for distribution by a third party.	Remaining biospecimen aliquots from the project are available by request from the corresponding author.

9. Antibodies

Describe the antibodies used and how they were validated for use in the system under study (i.e. assay and species).	Antibodies were used for serology and the ELISAs we performed are not commercially available as kits or reagents (except for secondary antibodies).<br>The primary antibodies are patient sera (validation not applicable).<br>Conjugated polyclonal secondary antibodies are utilized, clone not applicable:<br>Jackson ImmunoResearch Laboratories goat anti-human IgG-alkaline phosphatase; Cat # 109-056-098; 1:1000 dilution<br>Jackson ImmunoResearch Laboratories goat anti-human IgA-alkaline phosphatase; Cat # 109-055-011; 1:1000 dilution

10. Eukaryotic cell lines

a. State the source of each eukaryotic cell line used.	No eukaryotic cell lines were used.
b. Describe the method of cell line authentication used.	No eukaryotic cell lines were used.
c. Report whether the cell lines were tested for mycoplasma contamination.	No eukaryotic cell lines were used.
d. If any of the cell lines used are listed in the database of commonly misidentified cell lines maintained by ICLAC, provide a scientific rationale for their use.	No commonly misidentified cell lines were used.

Animals and human research participants

Policy information about studies involving animals; when reporting animal research, follow the ARRIVE guidelines

11. Description of research animals

Provide all relevant details on animals and/or animal-derived materials used in the study.	No animals were used.

Policy information about studies involving human research participants

12. Description of human research participants

Describe the covariate-relevant population characteristics of the human research participants.	Population characteristics are presented in Extended Data Table 1.
