# Supplementary material for: Multi-omics of the gut microbial ecosystem in inflammatory bowel diseases
Source: Nature. 2019 May 29;569(7758):655–62. doi: 10.1038/s41586-019-1237-9 (PMC6650278; doi:10.1038/s41586-019-1237-9)
Supplement: Supplementary file 3 — The relationship between the abundances of ECs in metagenomes (MGX), metatranscriptomes (MTX), or proteomes (MPX) are shown against each other and with the metabolites which the EC’s associated reaction either consumes or produces based on the MetaCyc database. The ratio between transcript relative abundance and genomic relative abundance (MTX/MGX) is also shown. MetaCyc unique IDs for the reaction and for the metabolite are shown in brackets. Only features for which MBX and at least one other measurement type have at least 10% prevalence are shown. [file 41586_2019_1237_MOESM3_ESM.pdf]

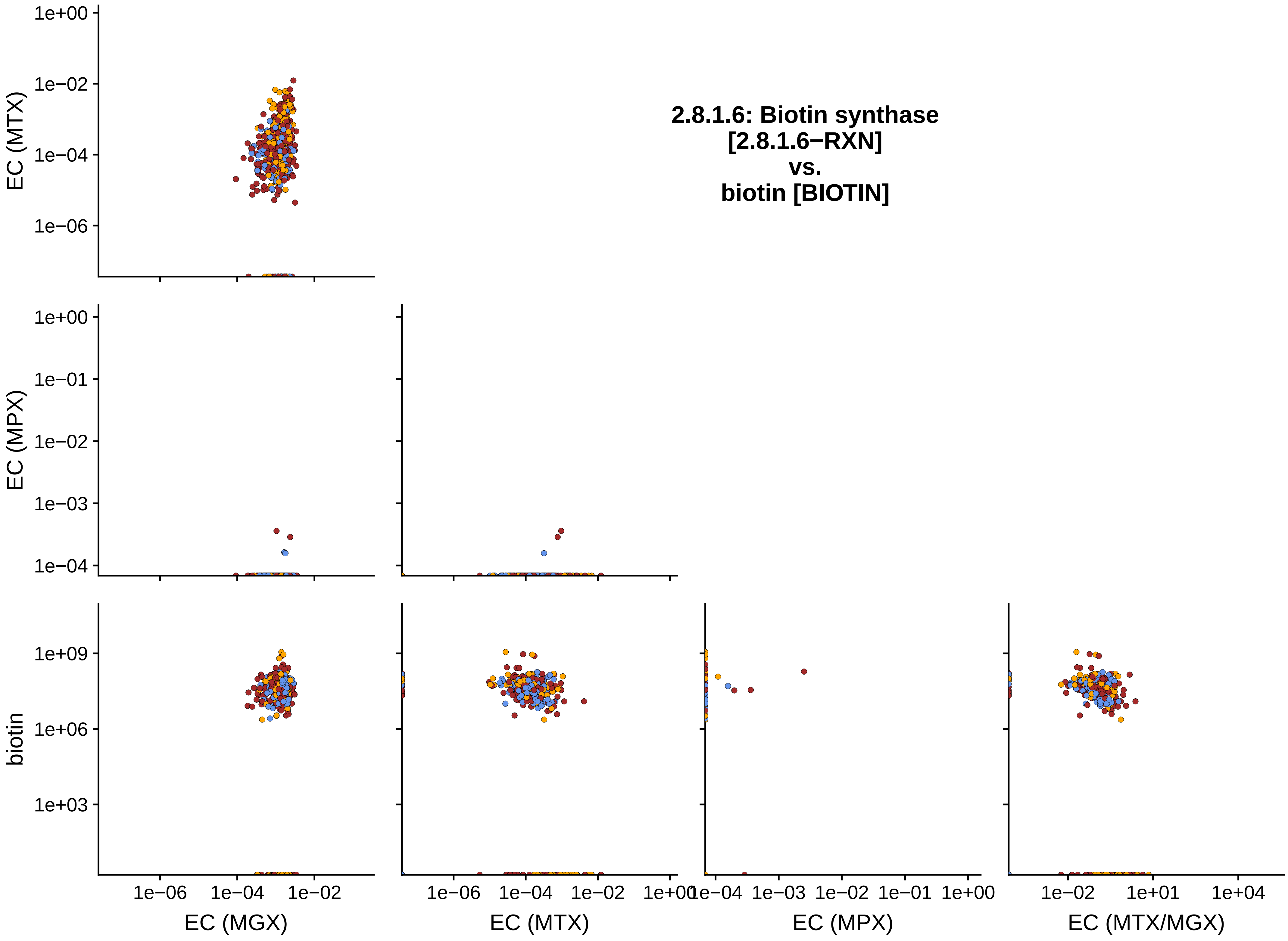

2.8.1.6: Biotin synthase
[2.8.1.6−RXN]
vs.
biotin [BIOTIN]
EC (MTX)
EC (MPX)
biotin
EC (MGX)
EC (MTX)
EC (MPX)
EC (MTX/MGX)
1e+00
1e−02
1e−04
1e−06
1e+00
1e−01
1e−02
1e−03
1e−04
1e+09
1e+06
1e+03
1e−06
1e−04
1e−02
1e+00
1e−03
1e−02
1e−01
1e+01
1e+04

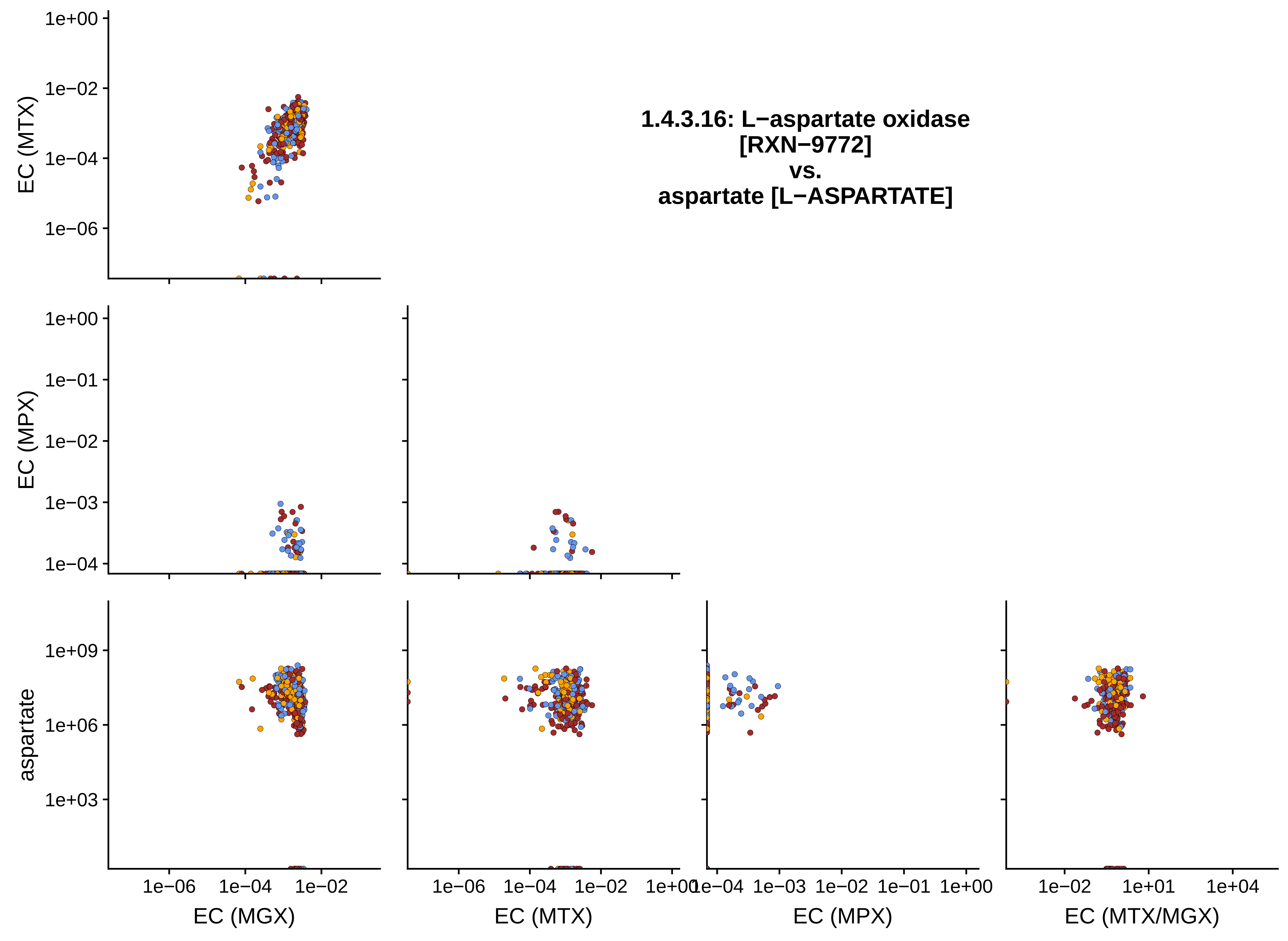

1.4.3.16: L−aspartate oxidase
[RXN−9772]
vs.
aspartate [L−ASPARTATE]
EC (MTX)
EC (MPX)
aspartate
EC (MGX)
EC (MTX)
EC (MPX)
EC (MTX/MGX)
1e+00
1e−02
1e−04
1e−06
1e−01
1e−03
1e+09
1e+06
1e+03
1e+04
1e+01

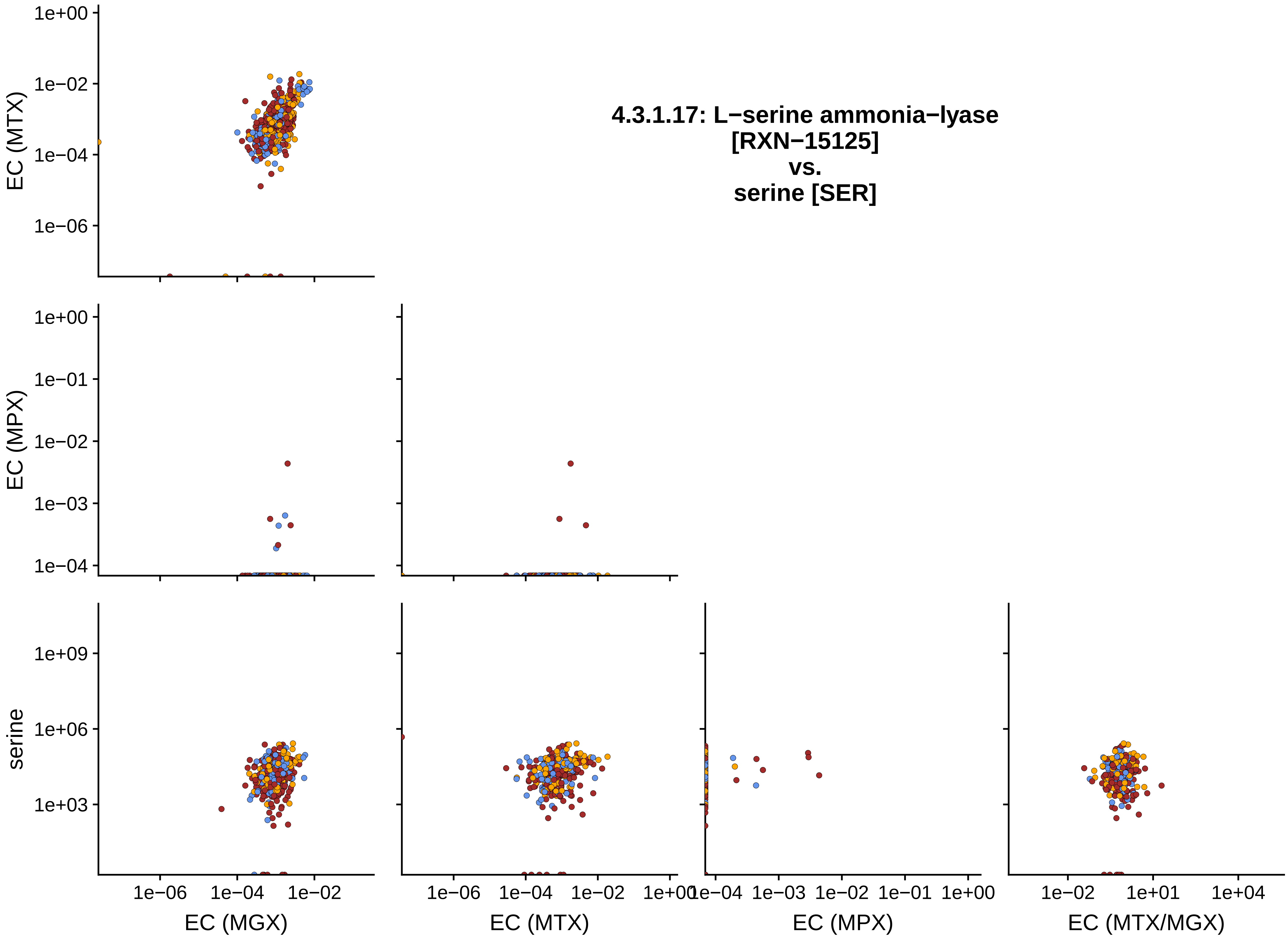

4.3.1.17: L−serine ammonia−lyase
[RXN−15125]
vs.
serine [SER]
EC (MTX)
EC (MPX)
serine
EC (MGX)
EC (MTX)
EC (MPX)
EC (MTX/MGX)
1e+00
1e−02
1e−04
1e−06
1e+00
1e−01
1e−02
1e−03
1e−04
1e+09
1e+06
1e+03
1e−06
1e−04
1e−02
1e−06
1e−04
1e−02
1e+00
1e−04
1e−03
1e−02
1e−01
1e+00
1e−02
1e+01
1e+04

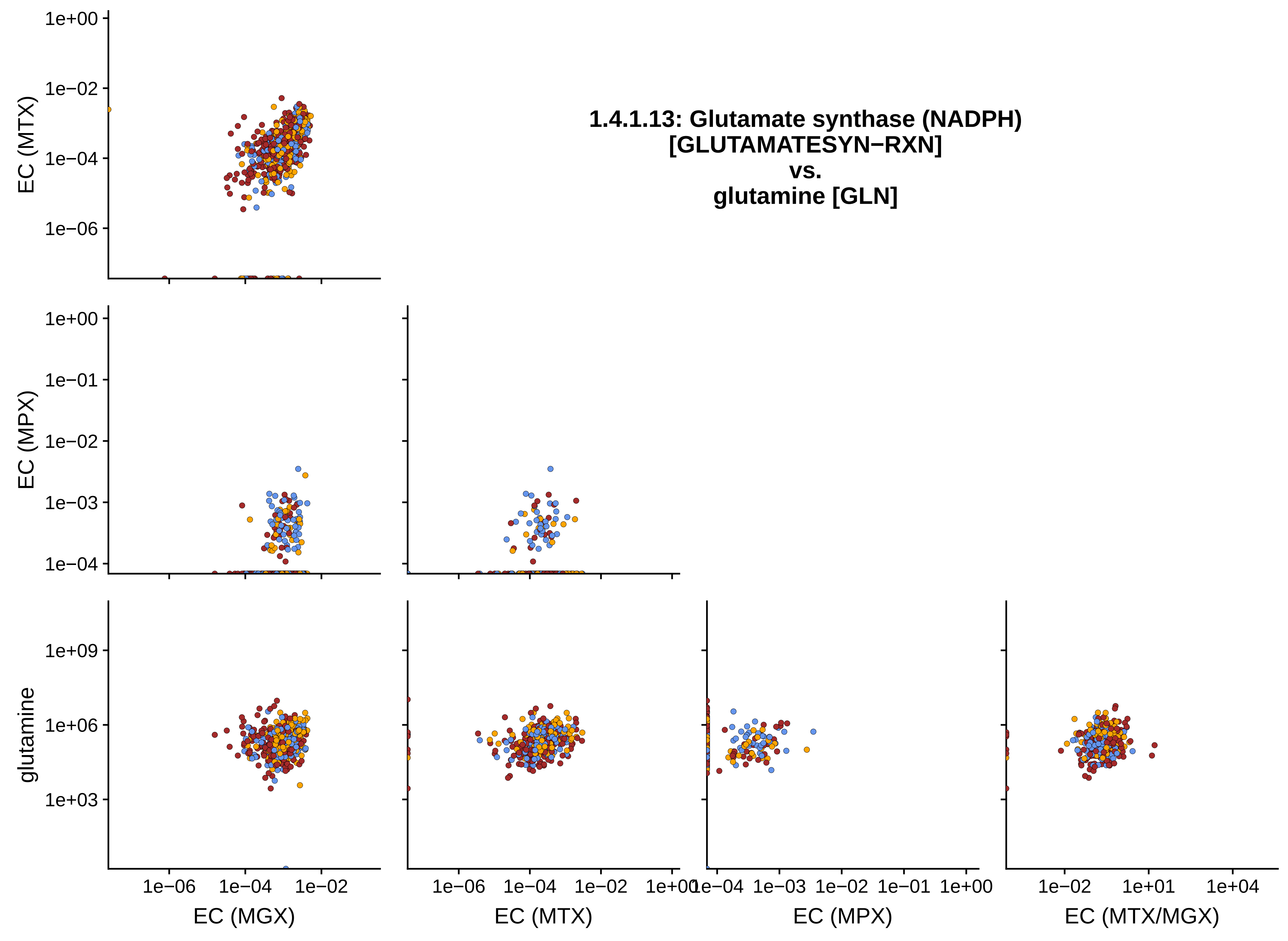
1.4.1.13: Glutamate synthase (NADPH)
[GLUTAMATESYN−RXN]
vs.
glutamine [GLN]
EC (MTX)
EC (MPX)
glutamine
EC (MGX)
EC (MTX)
EC (MPX)
EC (MTX/MGX)
1e+00
1e−02
1e−04
1e−06
1e−01
1e−03
1e+09
1e+06
1e+03
1e+04
1e+01

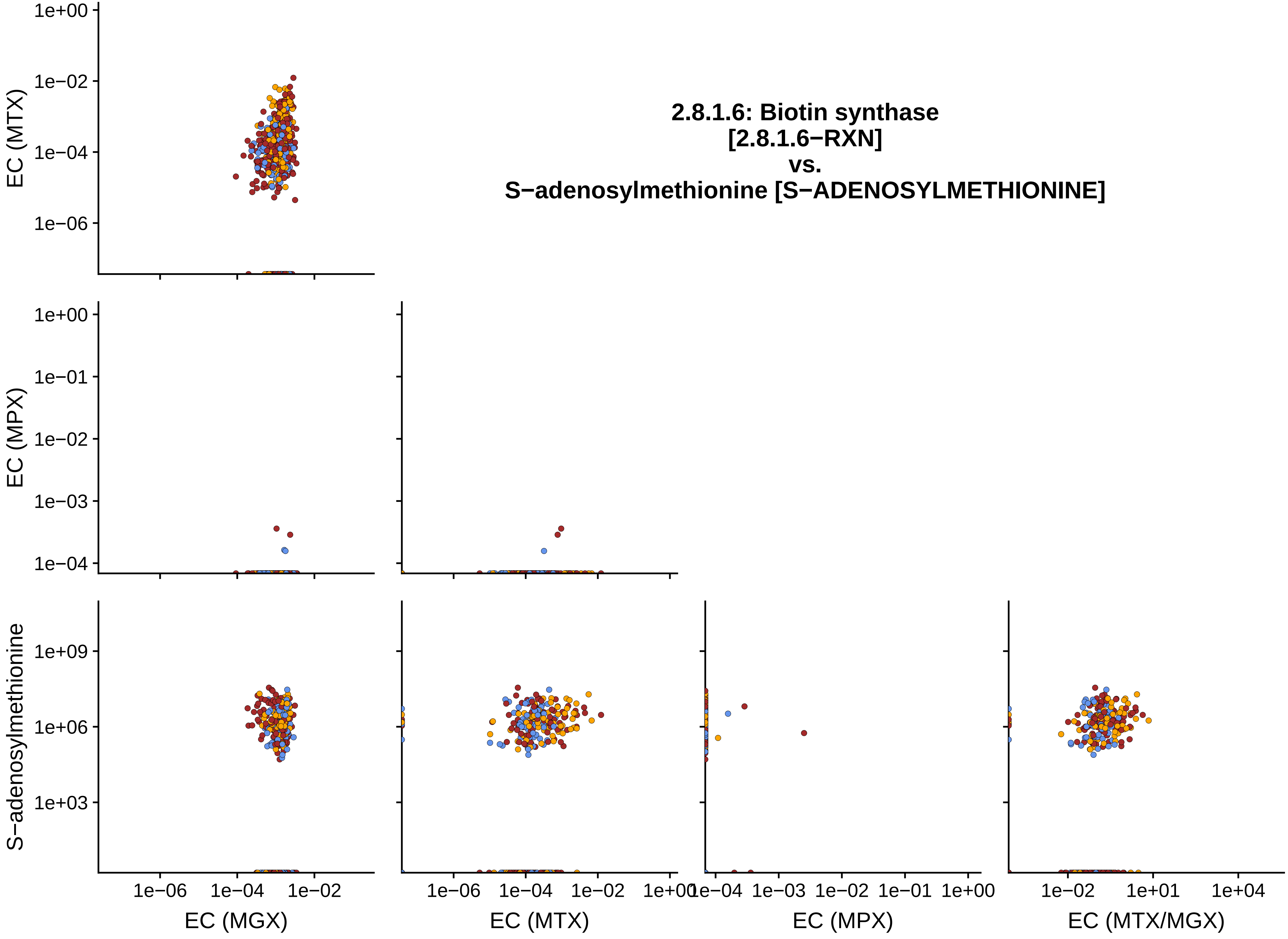

2.8.1.6: Biotin synthase
[2.8.1.6−RXN]
vs.
S−adenosylmethionine [S−ADENOSYLMETHIONINE]
EC (MTX)
EC (MPX)
S−adenosylmethionine
EC (MGX)
EC (MTX)
EC (MPX)
EC (MTX/MGX)
1e+00
1e−02
1e−04
1e−06
1e−01
1e−03
1e+09
1e+06
1e+03
1e+01
1e+04

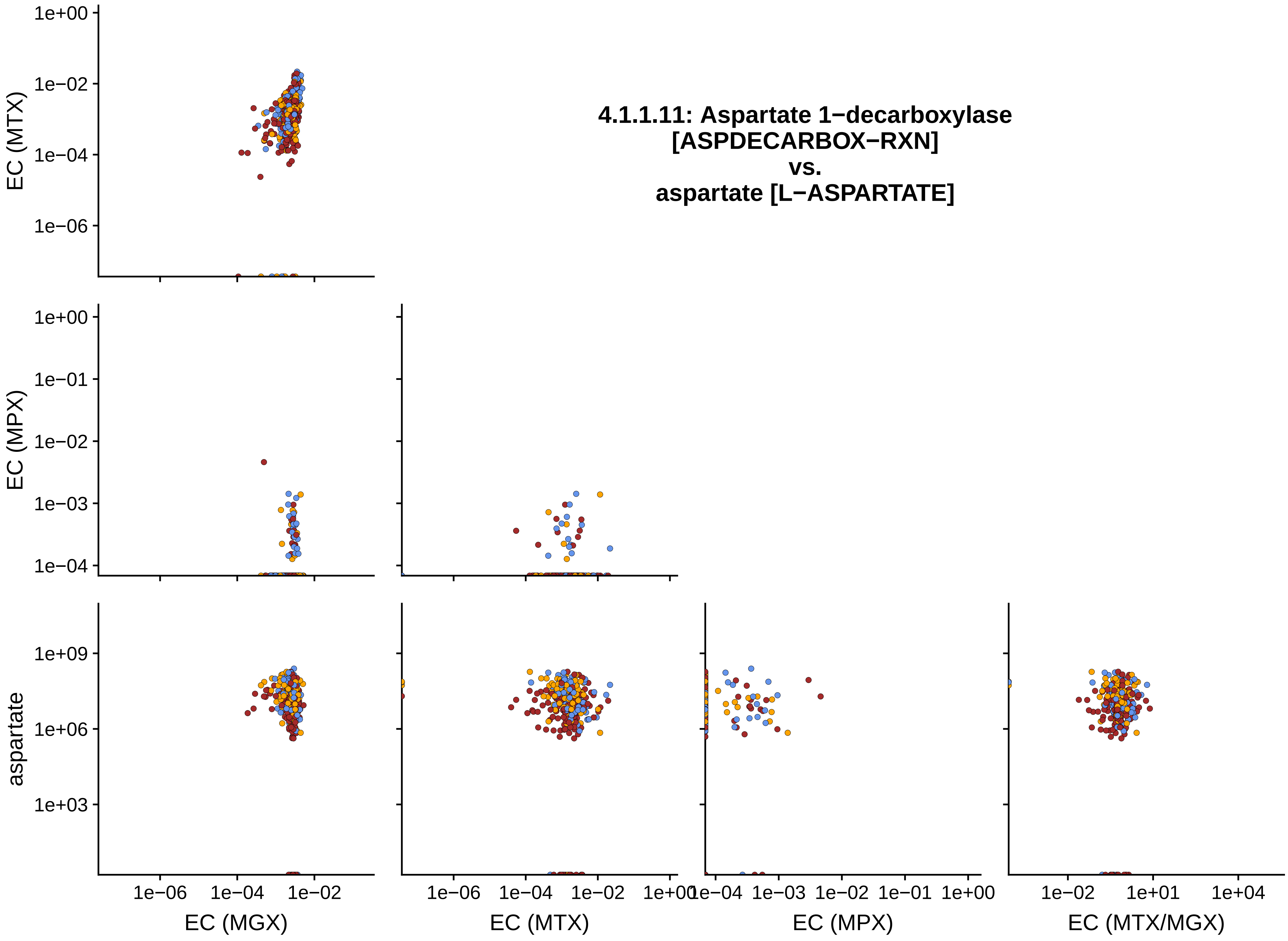
4.1.1.11: Aspartate 1−decarboxylase
[ASPDECARBOX−RXN]
vs.
aspartate [L−ASPARTATE]
EC (MTX)
EC (MPX)
aspartate
EC (MGX)
EC (MTX)
EC (MPX)
EC (MTX/MGX)
1e+00
1e−02
1e−04
1e−06
1e−01
1e−03
1e+09
1e+06
1e+03
1e+01
1e+04

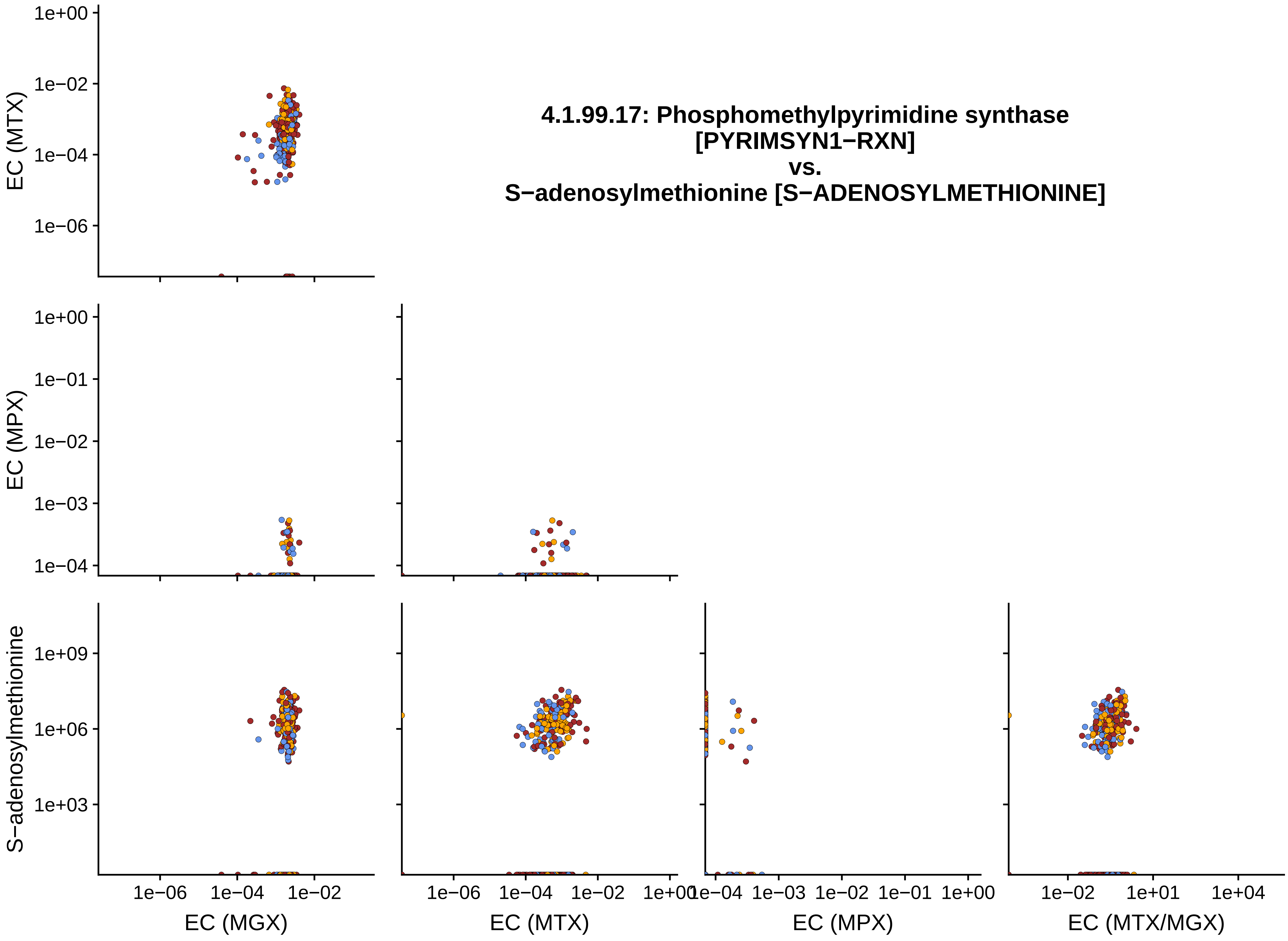

4.1.99.17: Phosphomethylpyrimidine synthase
[PYRIMSYN1−RXN]
vs.
S−adenosylmethionine [S−ADENOSYLMETHIONINE]
EC (MTX)
EC (MPX)
S−adenosylmethionine
EC (MGX)
EC (MTX)
EC (MPX)
EC (MTX/MGX)
1e+00
1e−02
1e−04
1e−06
1e+00
1e−01
1e−02
1e−03
1e−04
1e+09
1e+06
1e+03
1e−06
1e−04
1e−02
1e−06
1e−04
1e−02
1e+00
1e−04
1e−03
1e−02
1e−01
1e+00
1e−02
1e+01
1e+04

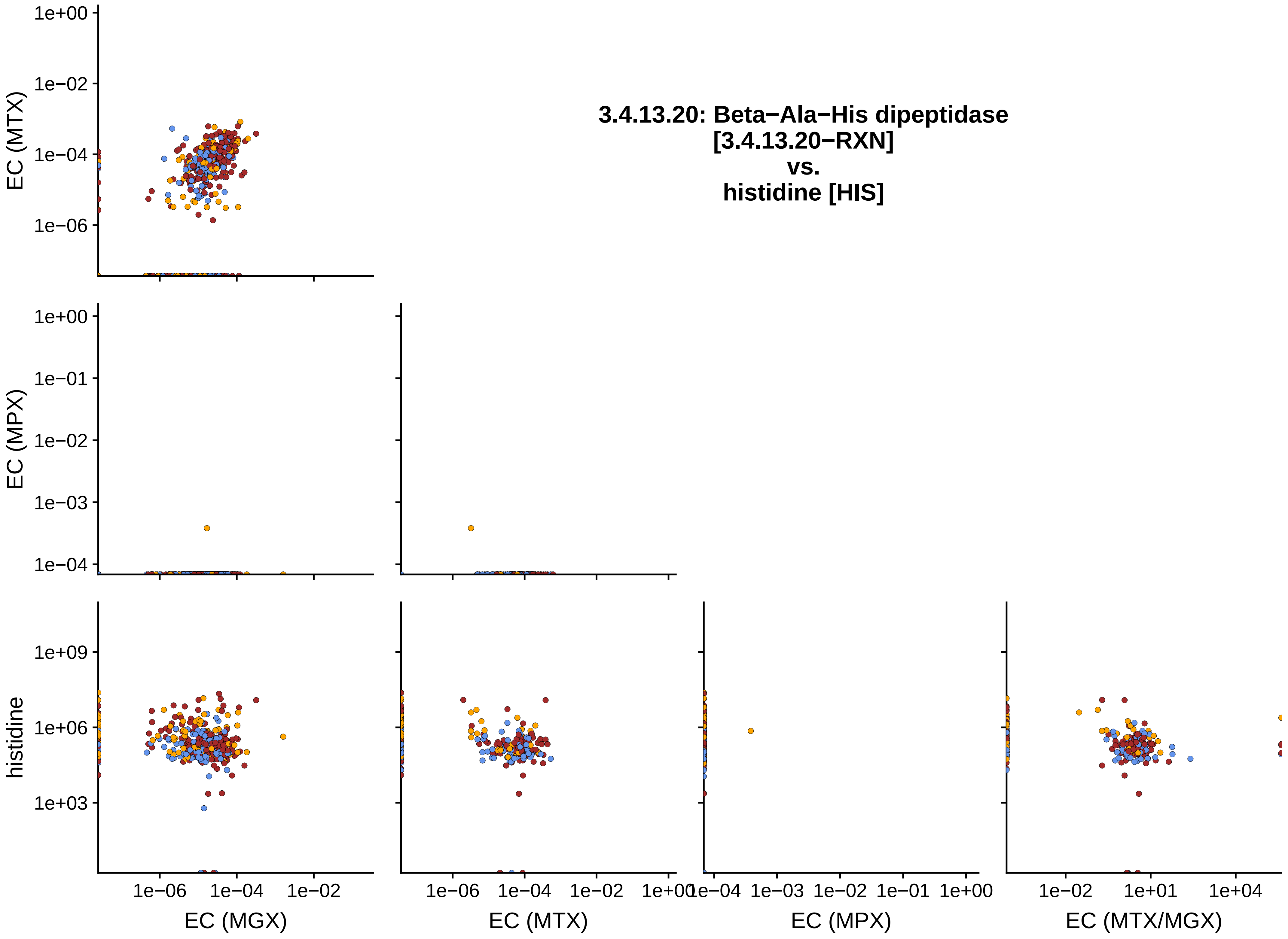
3.4.13.20: Beta−Ala−His dipeptidase
[3.4.13.20−RXN]
vs.
histidine [HIS]
EC (MTX)
EC (MPX)
histidine
EC (MGX)
EC (MTX)
EC (MPX)
EC (MTX/MGX)
1e+00
1e−02
1e−04
1e−06
1e−01
1e−03
1e+09
1e+06
1e+03
1e+01
1e+04

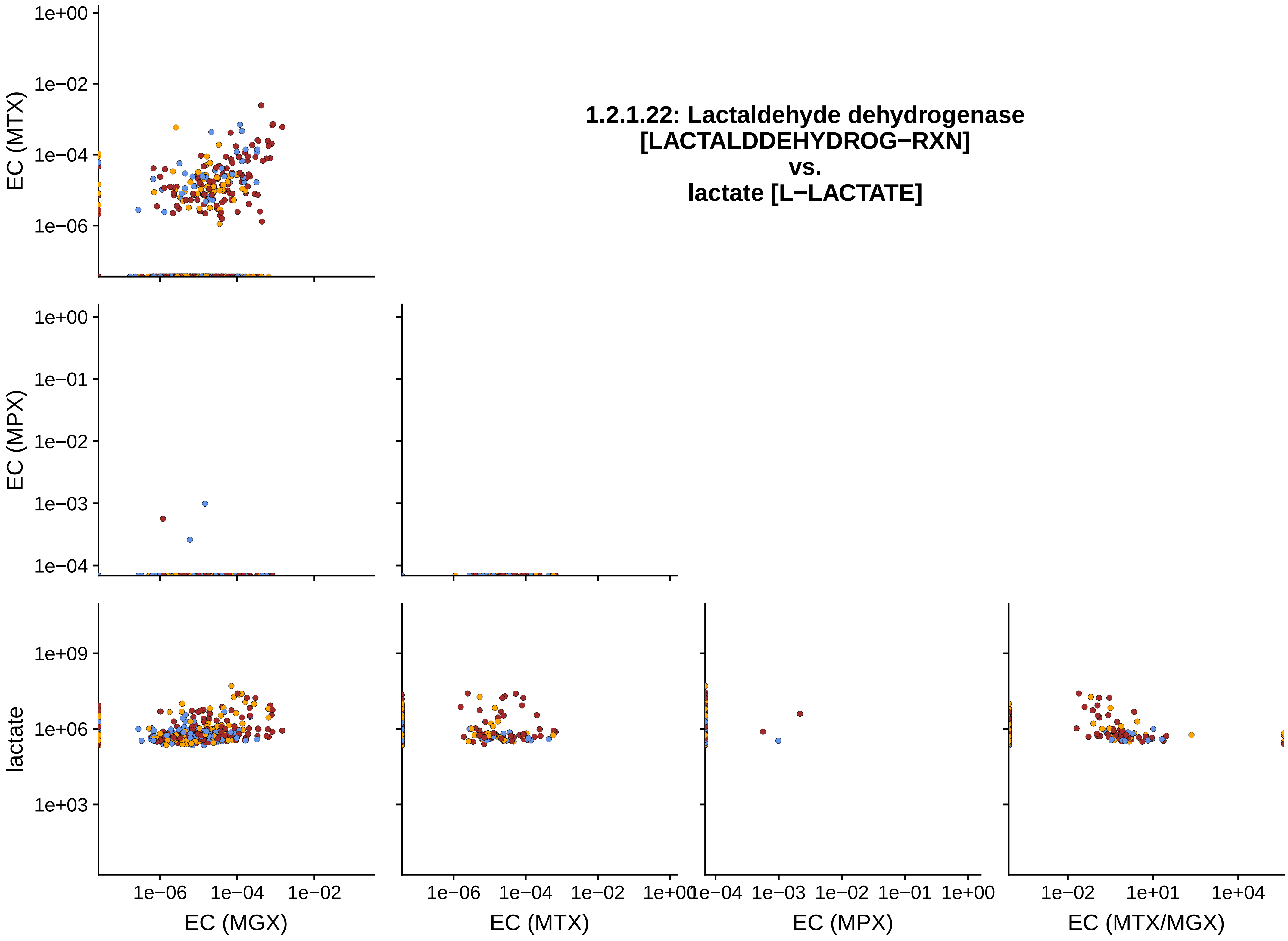

1.2.1.22: Lactaldehyde dehydrogenase
[LACTALDDEHYDROG−RXN]
vs.
lactate [L−LACTATE]
EC (MTX)
1e+00
1e−02
1e−04
1e−06
EC (MPX)
1e+00
1e−01
1e−02
1e−03
1e−04
lactate
1e+09
1e+06
1e+03
EC (MGX)
1e−06
1e−04
1e−02
EC (MTX)
1e−06
1e−04
1e−02
1e+00
EC (MPX)
1e−04
1e−03
1e−02
1e−01
1e+00
EC (MTX/MGX)
1e−02
1e+01
1e+04

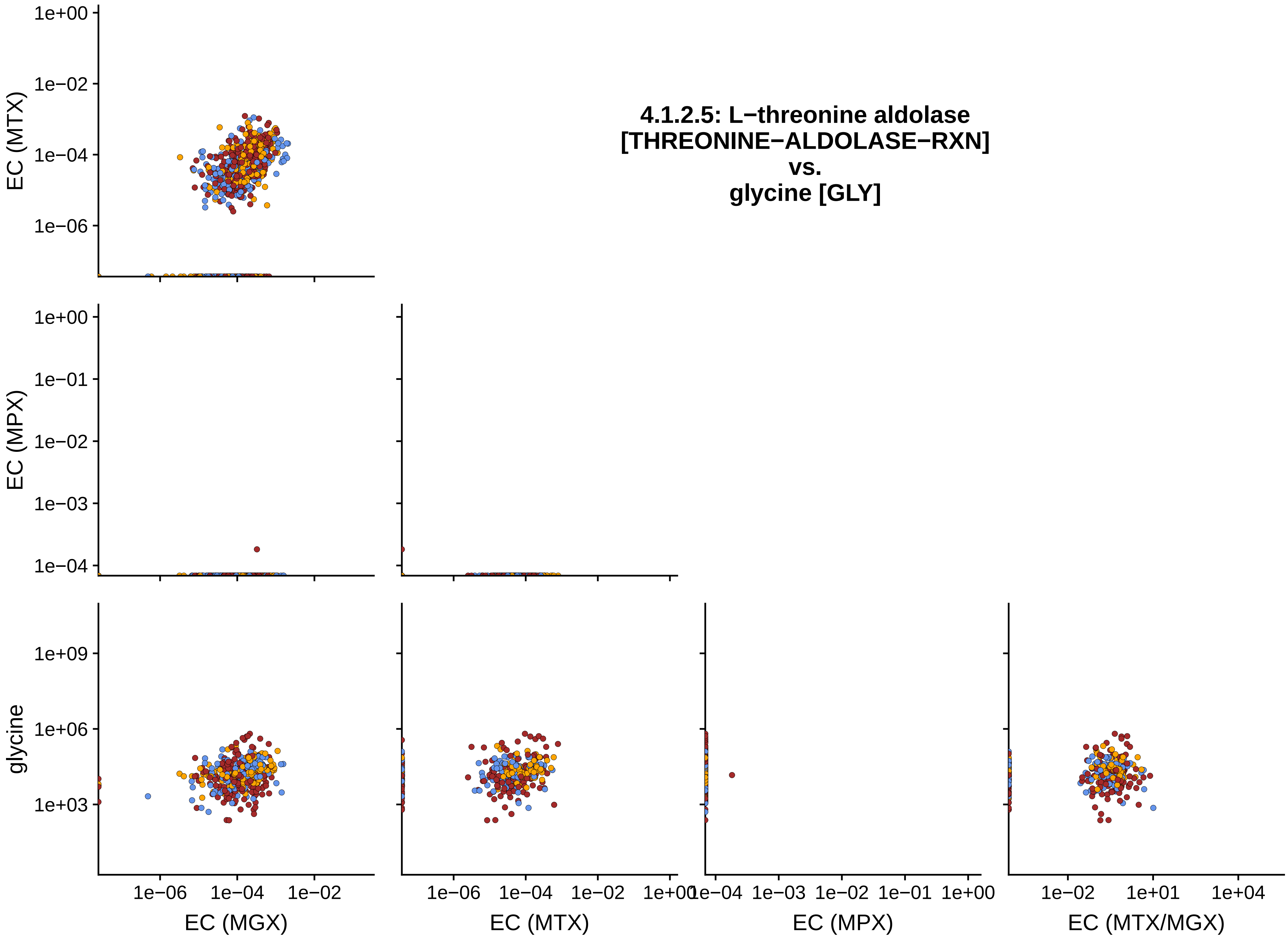
4.1.2.5: L−threonine aldolase
[THREONINE−ALDOLASE−RXN]
vs.
glycine [GLY]
EC (MTX)
EC (MPX)
glycine
EC (MGX)
EC (MTX)
EC (MPX)
EC (MTX/MGX)
1e+00
1e−02
1e−04
1e−06
1e−01
1e−03
1e+09
1e+06
1e+03
1e+01
1e+04

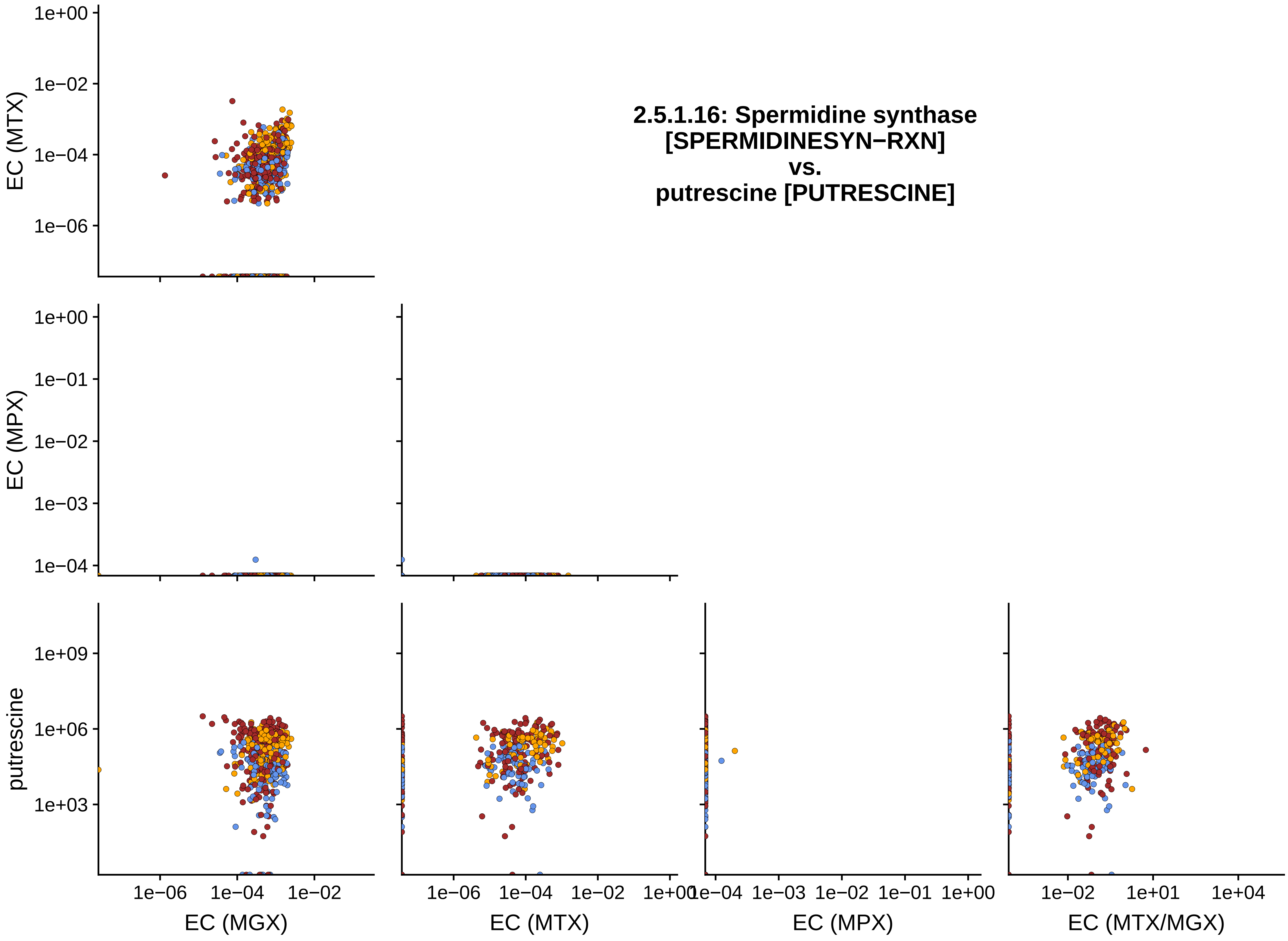
2.5.1.16: Spermidine synthase
[SPERMIDINESYN−RXN]
vs.
putrescine [PUTRESCINE]
EC (MTX)
EC (MPX)
putrescine
EC (MGX)
EC (MTX)
EC (MPX)
EC (MTX/MGX)
1e+00
1e−02
1e−04
1e−06
1e−01
1e−03
1e+09
1e+06
1e+03
1e+01
1e+04

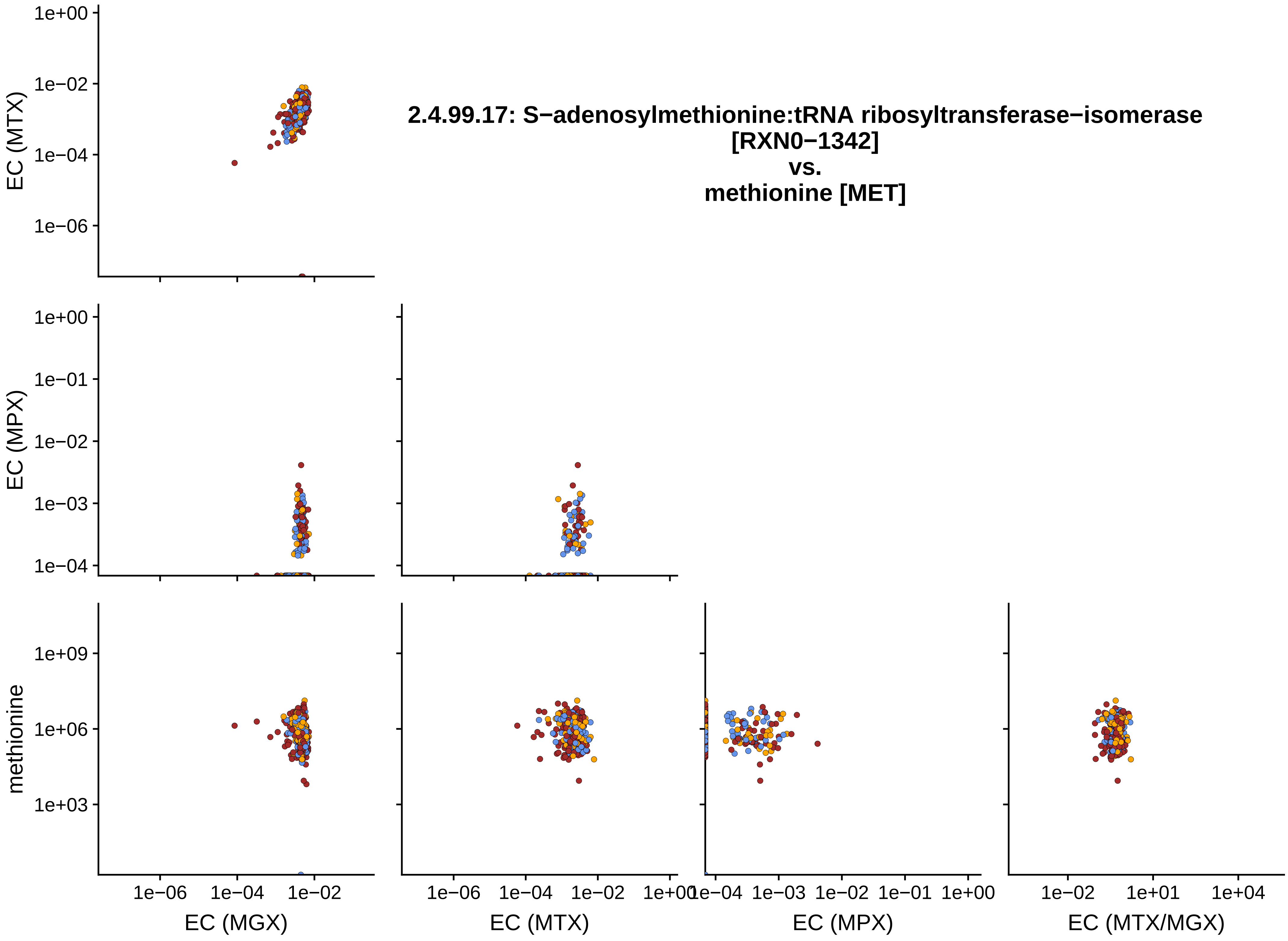

2.4.99.17: S−adenosylmethionine:tRNA ribosyltransferase−isomerase
[RXN0−1342]
vs.
methionine [MET]
EC (MTX)
EC (MPX)
methionine
EC (MGX)
EC (MTX)
EC (MPX)
EC (MTX/MGX)
1e+00
1e−02
1e−04
1e−06
1e−01
1e−03
1e+09
1e+06
1e+03
1e+01
1e+04

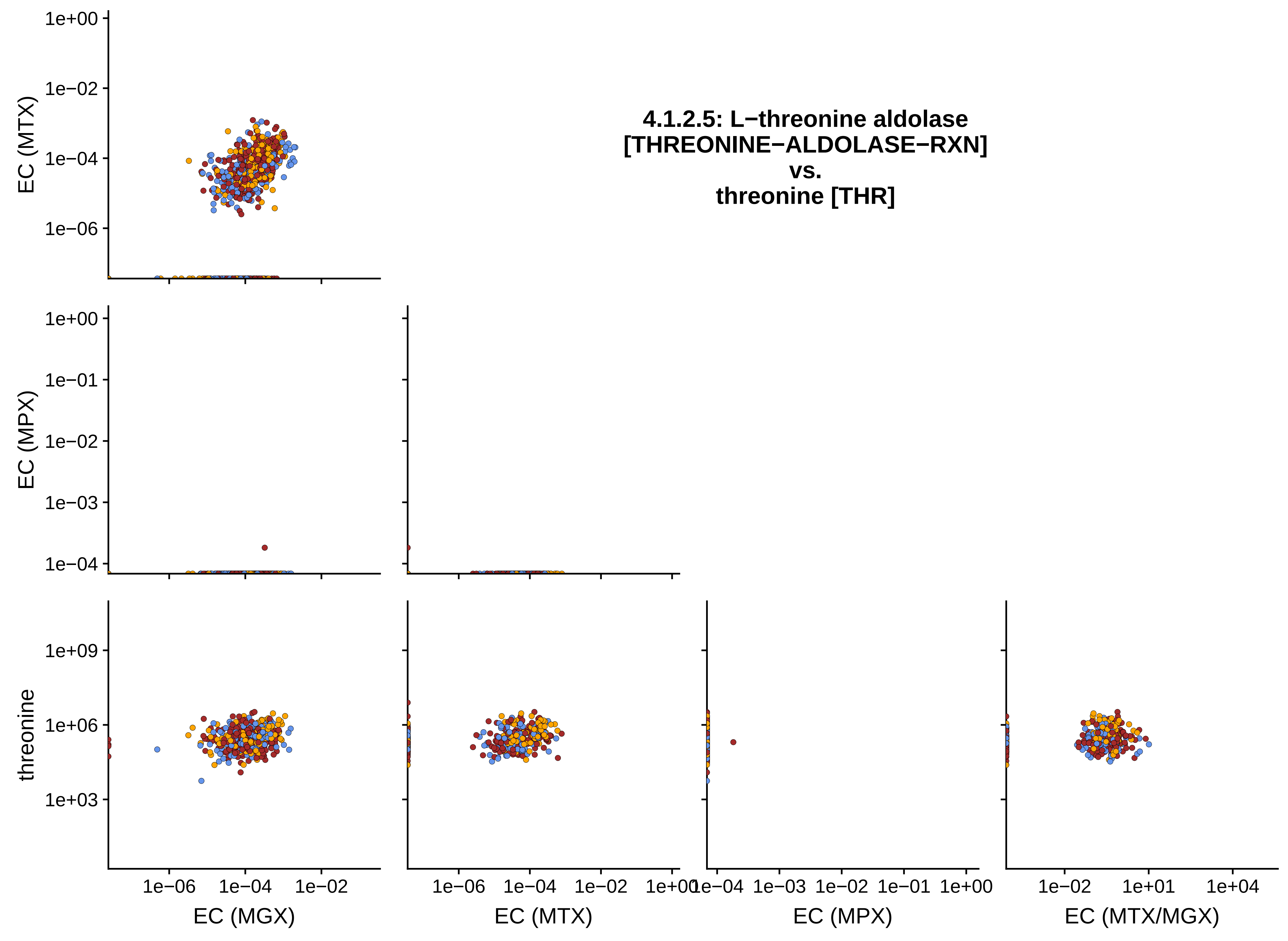
4.1.2.5: L−threonine aldolase
[THREONINE−ALDOLASE−RXN]
vs.
threonine [THR]
EC (MTX)
EC (MPX)
threonine
EC (MGX)
EC (MTX)
EC (MPX)
EC (MTX/MGX)
1e+00
1e−02
1e−04
1e−06
1e−01
1e−03
1e+09
1e+06
1e+03
1e+01
1e+04

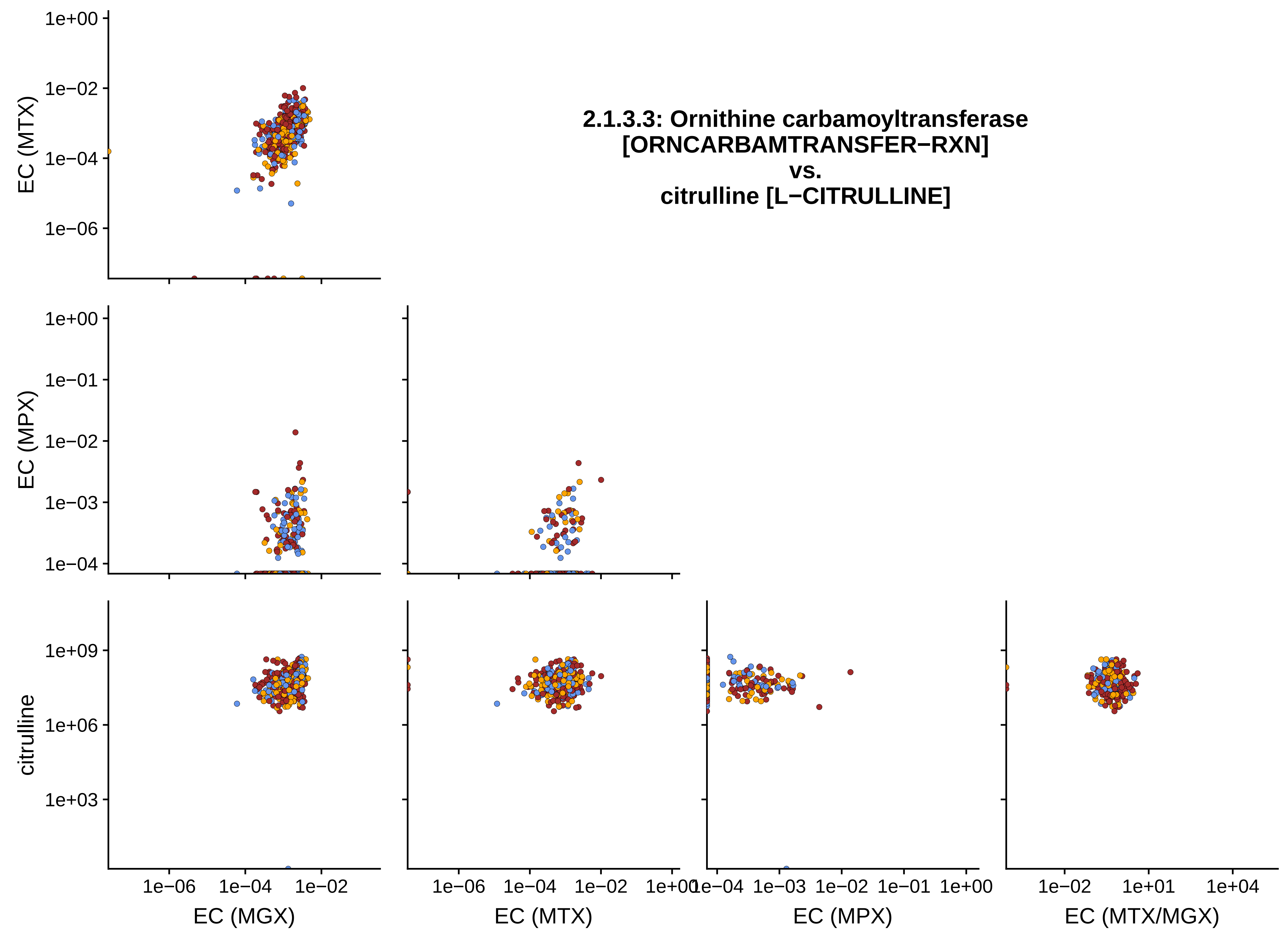

2.1.3.3: Ornithine carbamoyltransferase
[ORNCARBAMTRANSFER−RXN]
vs.
citrulline [L−CITRULLINE]
EC (MTX)
EC (MPX)
citrulline
EC (MGX)
EC (MTX)
EC (MPX)
EC (MTX/MGX)
1e+00
1e−02
1e−04
1e−06
1e−01
1e−03
1e+09
1e+06
1e+03
1e+01
1e+04

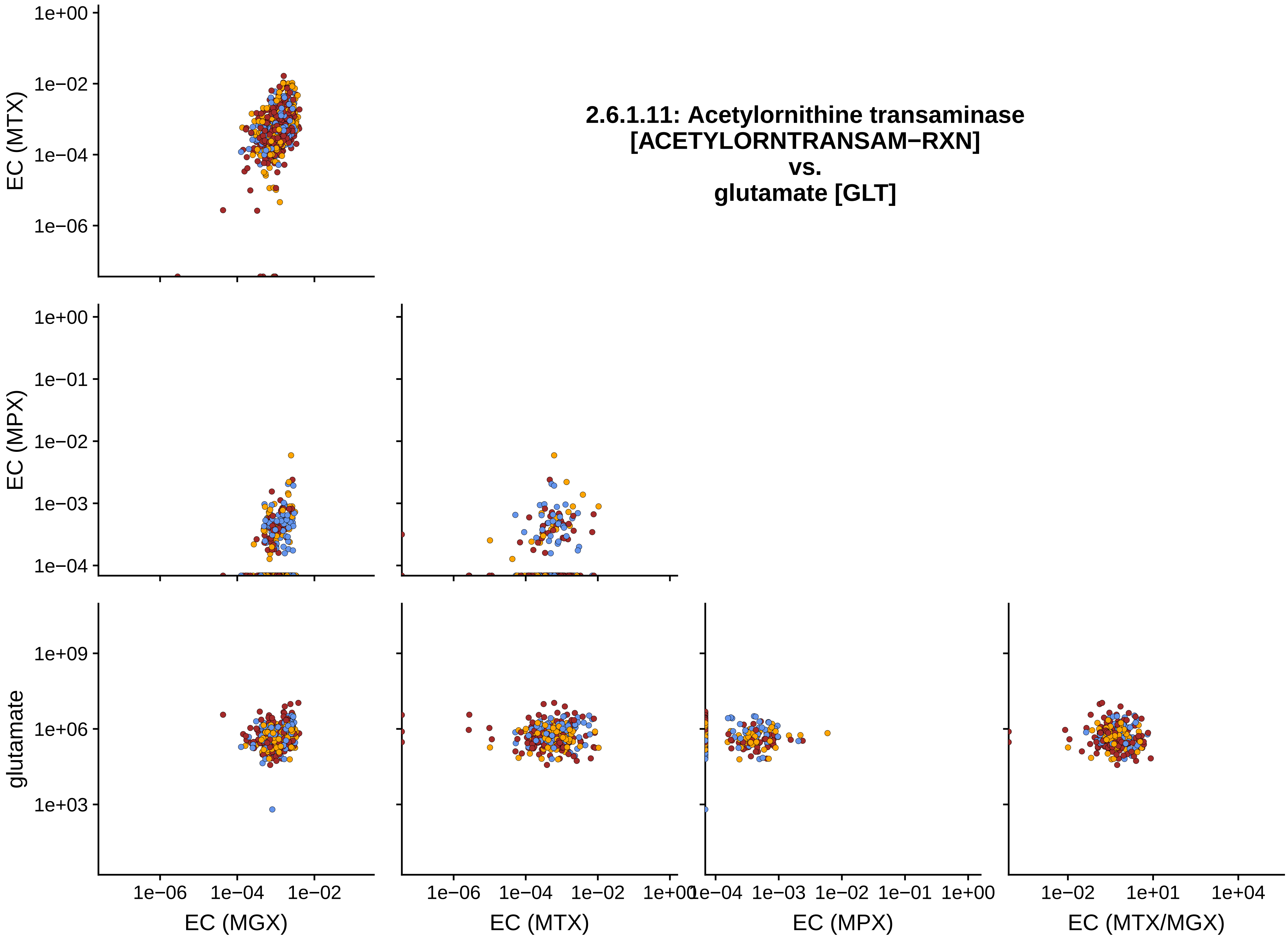
2.6.1.11: Acetylornithine transaminase
[ACETYLORNTRANSAM−RXN]
vs.
glutamate [GLT]
EC (MTX)
EC (MPX)
glutamate
EC (MGX)
EC (MTX)
EC (MPX)
EC (MTX/MGX)
1e+00
1e−02
1e−04
1e−06
1e−01
1e−03
1e+09
1e+06
1e+03
1e+04
1e+01

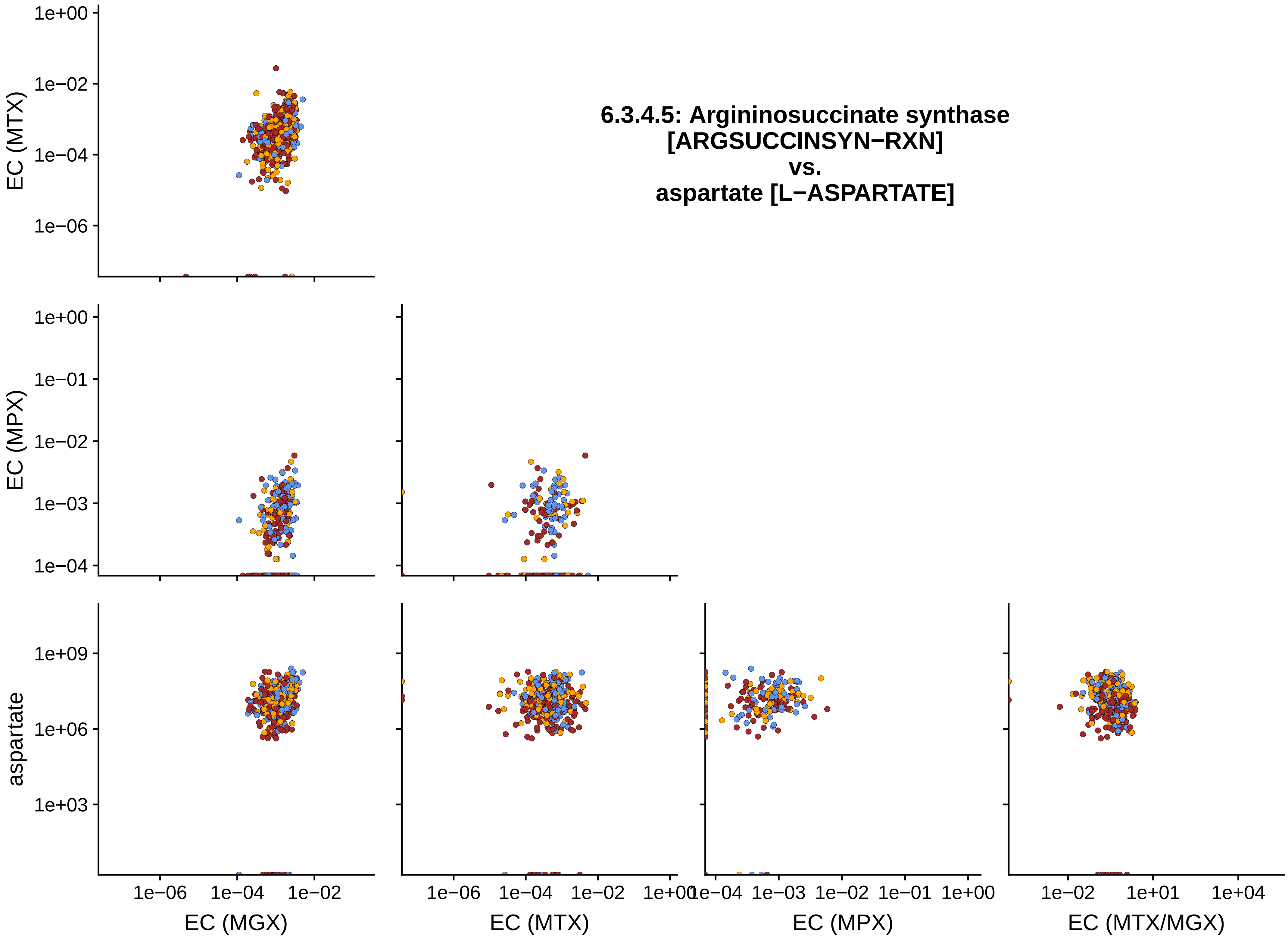

6.3.4.5: Argininosuccinate synthase
[ARGSUCCINSYN−RXN]
vs.
aspartate [L−ASPARTATE]
EC (MTX)
EC (MPX)
aspartate
EC (MGX)
EC (MTX)
EC (MPX)
EC (MTX/MGX)
1e+00
1e−02
1e−04
1e−06
1e−01
1e−03
1e+09
1e+06
1e+03
1e+01
1e+04

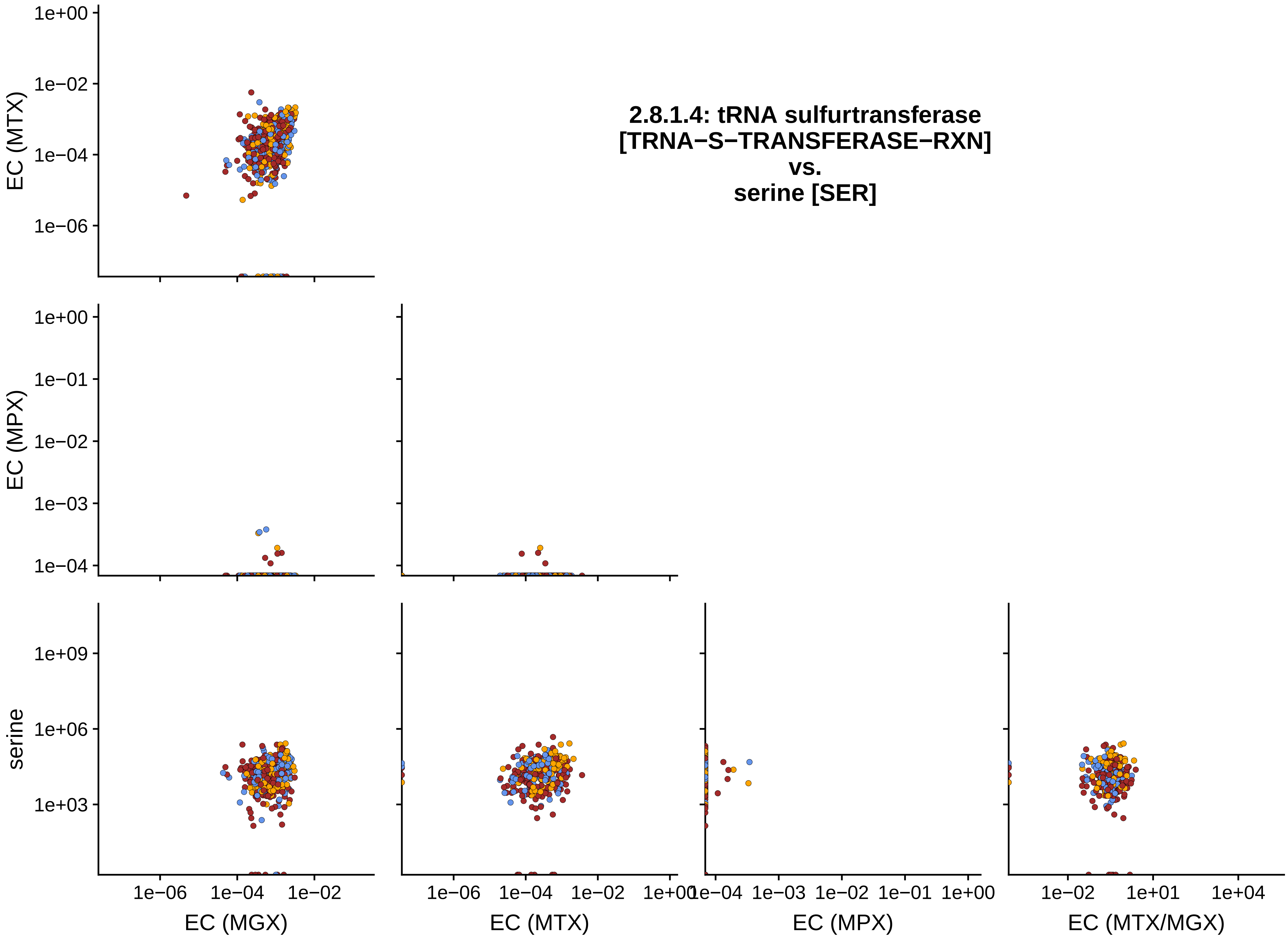

2.8.1.4: tRNA sulfurtransferase
[TRNA−S−TRANSFERASE−RXN]
vs.
serine [SER]
EC (MTX)
EC (MPX)
serine
EC (MGX)
EC (MTX)
EC (MPX)
EC (MTX/MGX)
1e+00
1e−02
1e−04
1e−06
1e−01
1e−03
1e+09
1e+06
1e+03
1e+01
1e+04

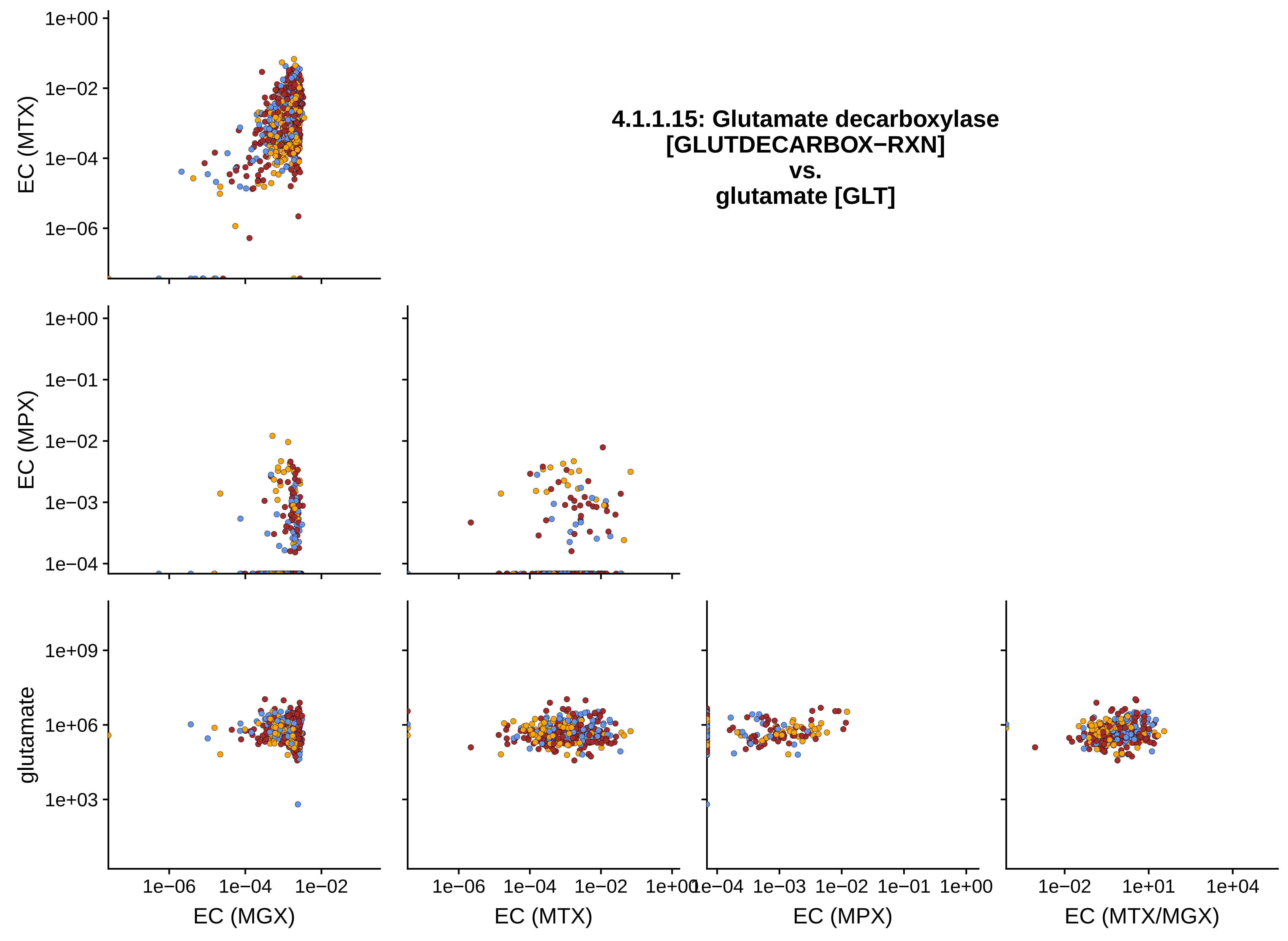
4.1.1.15: Glutamate decarboxylase
[GLUTDECARBOX−RXN]
vs.
glutamate [GLT]
EC (MTX)
EC (MPX)
glutamate
EC (MGX)
EC (MTX)
EC (MPX)
EC (MTX/MGX)
1e+00
1e−02
1e−04
1e−06
1e−01
1e−02
1e−03
1e−04
1e+09
1e+06
1e+03
1e−06
1e−04
1e−02
1e+00
1e−04
1e−03
1e−02
1e−01
1e+00
1e−02
1e+01
1e+04

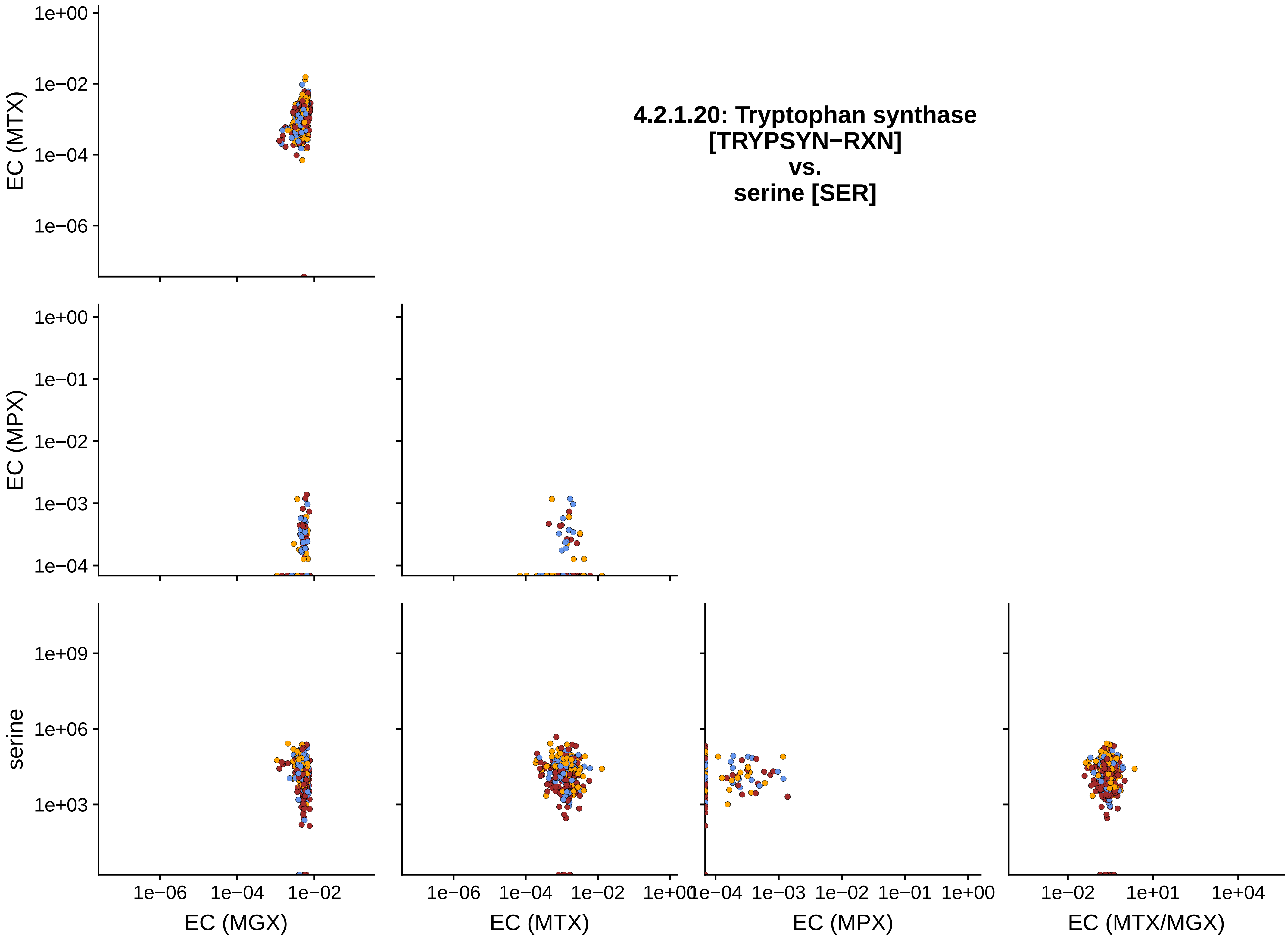
4.2.1.20: Tryptophan synthase
[TRYPSYN−RXN]
vs.
serine [SER]
EC (MTX)
EC (MPX)
serine
EC (MGX)
EC (MTX)
EC (MPX)
EC (MTX/MGX)
1e+00
1e−02
1e−04
1e−06
1e+00
1e−01
1e−02
1e−03
1e−04
1e+09
1e+06
1e+03
1e−06
1e−04
1e−02
1e+00
1e−03
1e−01
1e+01
1e+04

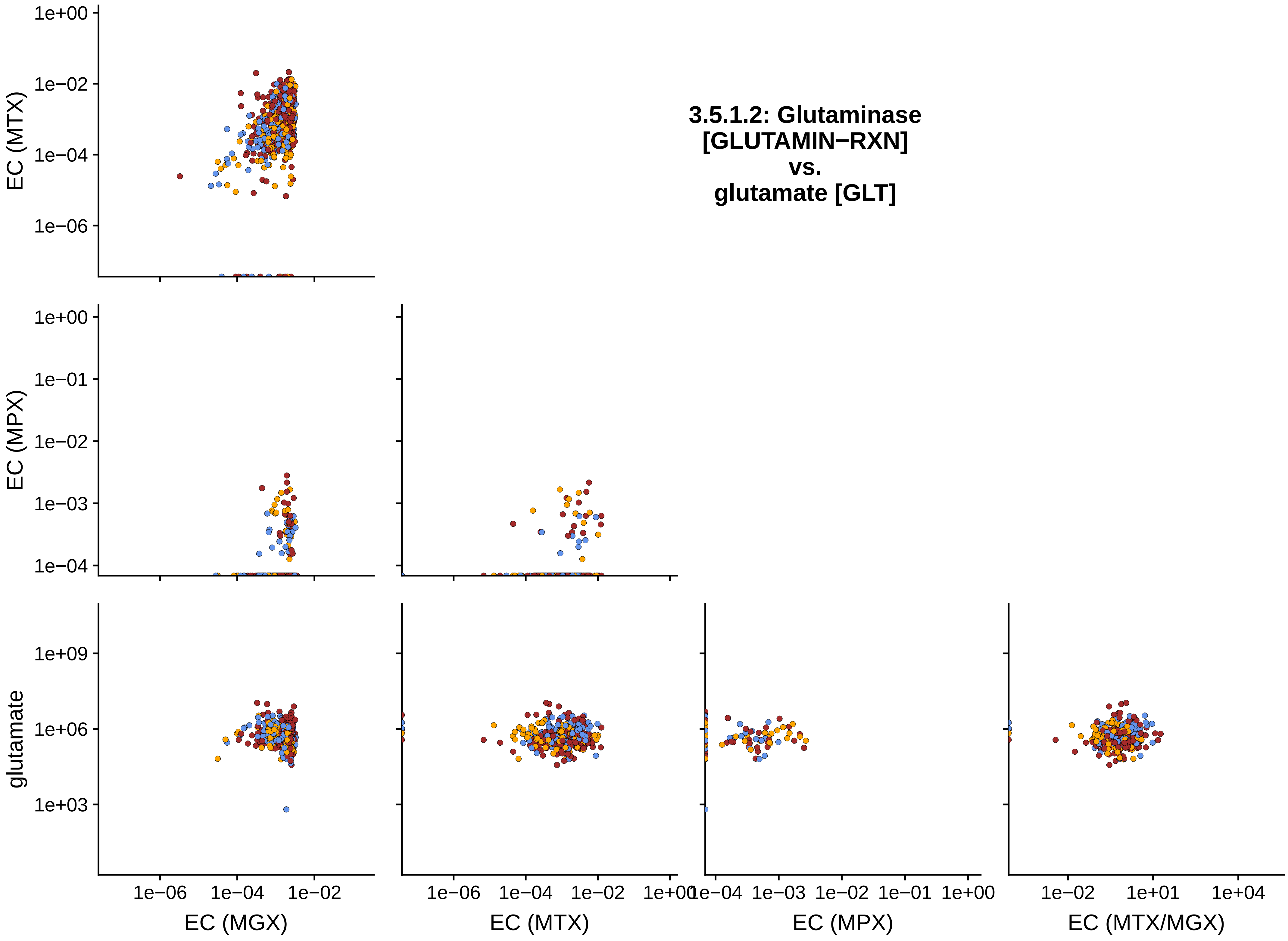
3.5.1.2: Glutaminase
[GLUTAMIN−RXN]
vs.
glutamate [GLT]
EC (MTX)
EC (MPX)
glutamate
EC (MGX)
EC (MTX)
EC (MPX)
EC (MTX/MGX)
1e+00
1e−02
1e−04
1e−06
1e−01
1e−03
1e+09
1e+06
1e+03
1e+01
1e+04

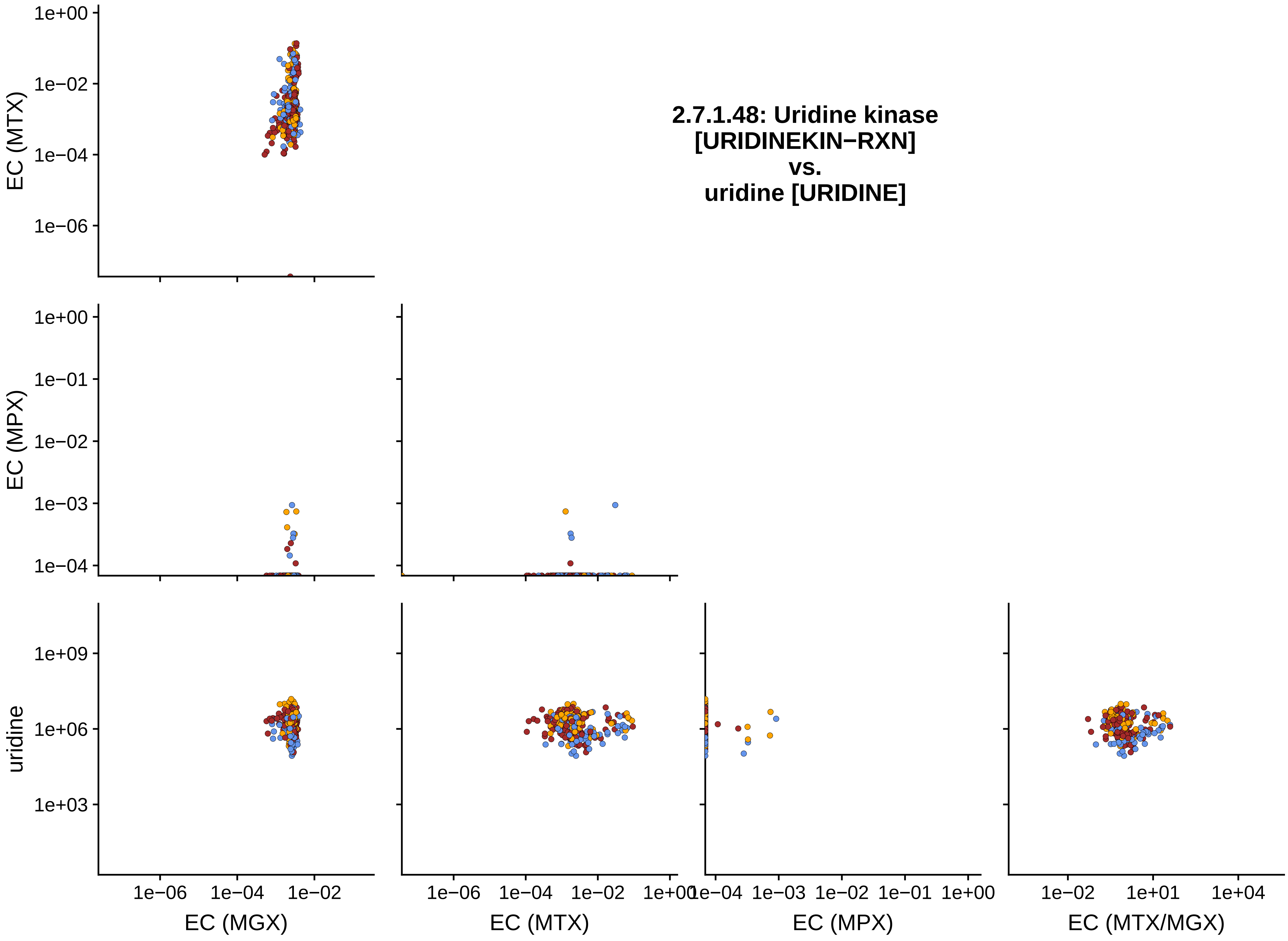
2.7.1.48: Uridine kinase
[URIDINEKIN−RXN]
vs.
uridine [URIDINE]
EC (MTX)
EC (MPX)
uridine
EC (MGX)
EC (MTX)
EC (MPX)
EC (MTX/MGX)
1e+00
1e−02
1e−04
1e−06
1e−01
1e−03
1e+09
1e+06
1e+03
1e+01
1e+04

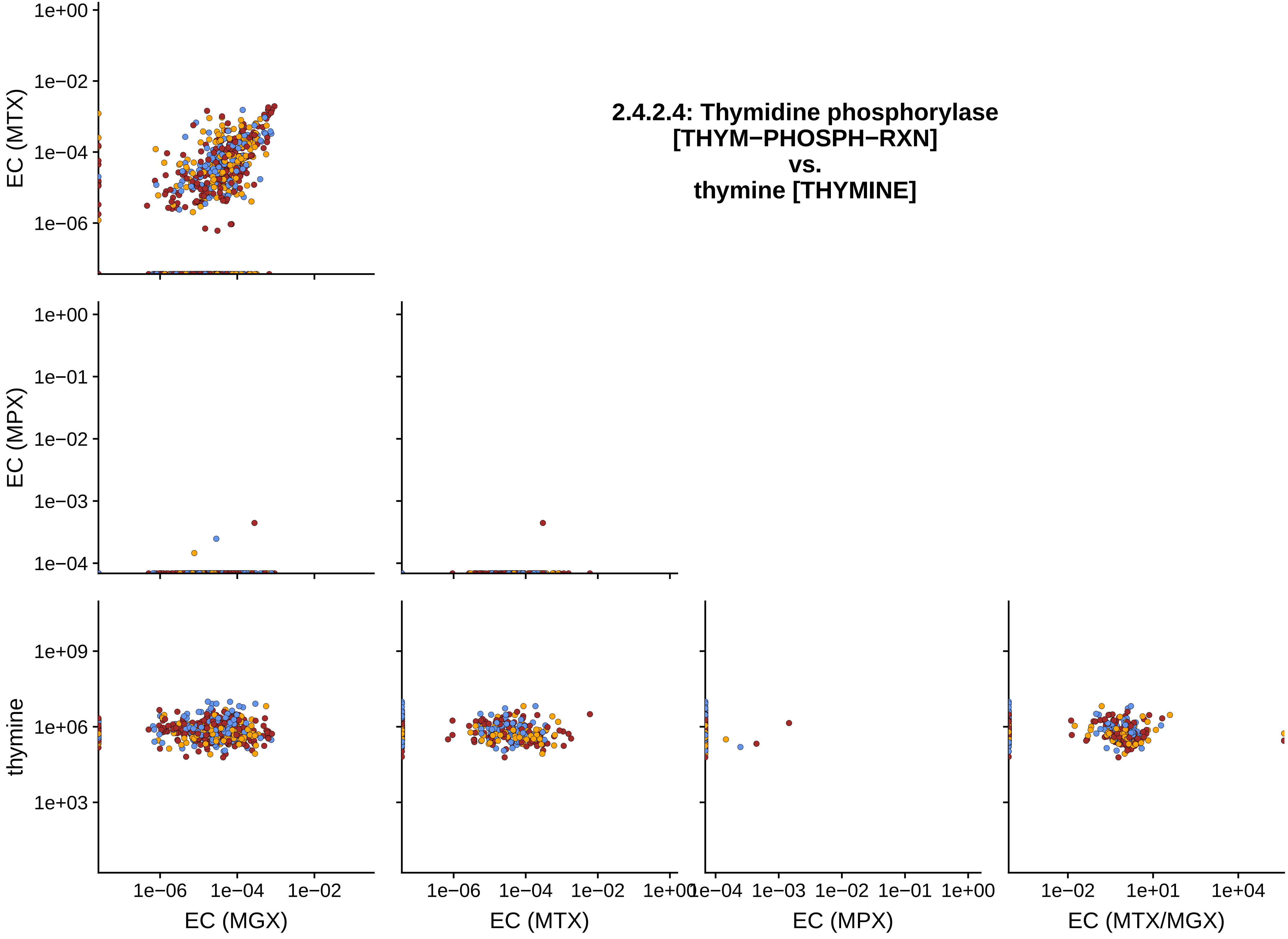

2.4.2.4: Thymidine phosphorylase
[THYM−PHOSPH−RXN]
vs.
thymine [THYMINE]
EC (MTX)
EC (MPX)
thymine
EC (MGX)
EC (MTX)
EC (MPX)
EC (MTX/MGX)
1e+00
1e−02
1e−04
1e−06
1e−01
1e−03
1e+09
1e+06
1e+03
1e+04
1e+01

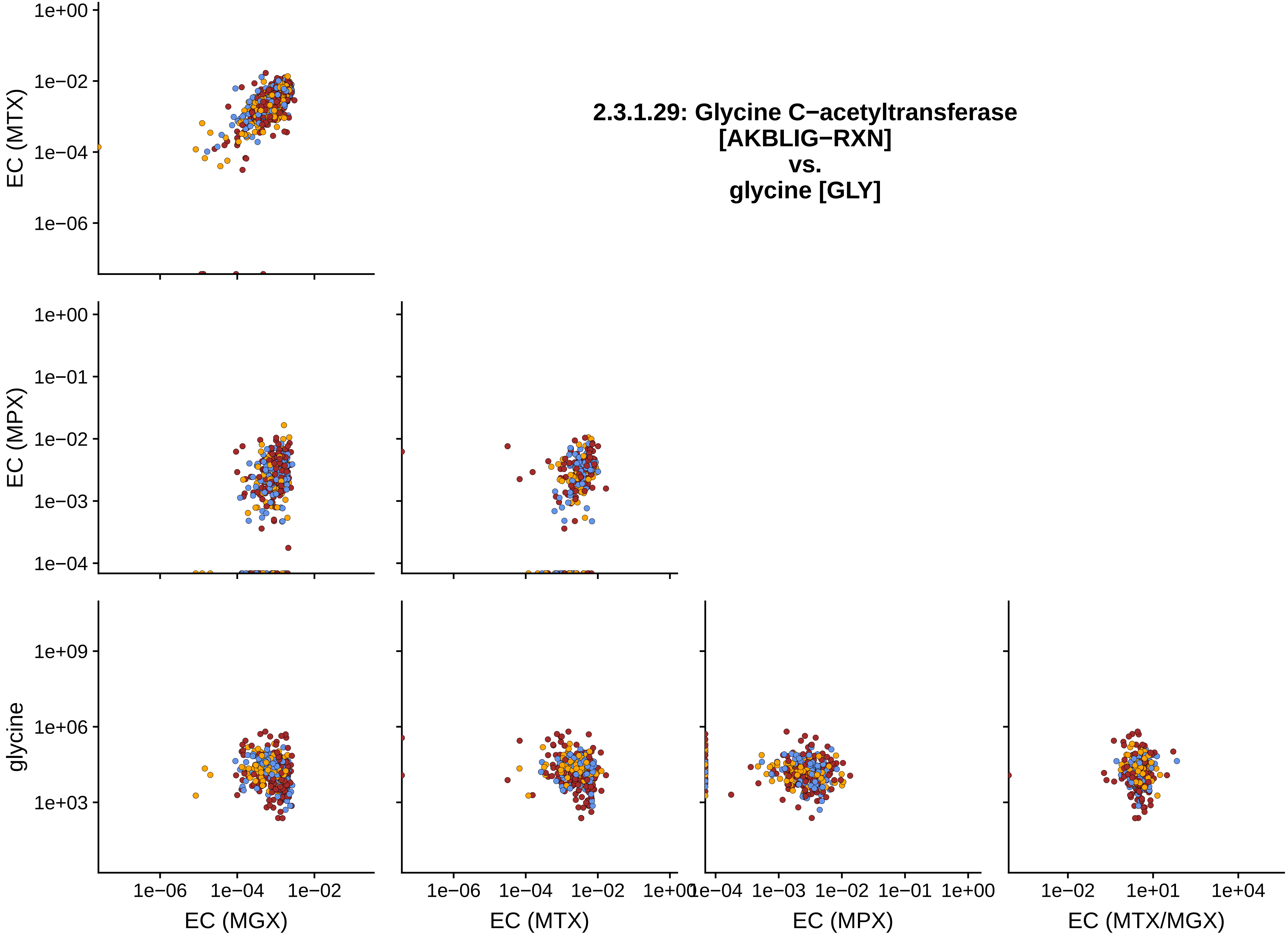

2.3.1.29: Glycine C−acetyltransferase
[AKBLIG−RXN]
vs.
glycine [GLY]
EC (MTX)
EC (MPX)
glycine
EC (MGX)
EC (MTX)
EC (MPX)
EC (MTX/MGX)
1e+00
1e−02
1e−04
1e−06
1e+00
1e−01
1e−02
1e−03
1e−04
1e+09
1e+06
1e+03
1e−06
1e−04
1e−02
1e−06
1e−04
1e−02
1e+00
1e−04
1e−03
1e−02
1e−01
1e+00
1e−02
1e+01
1e+04

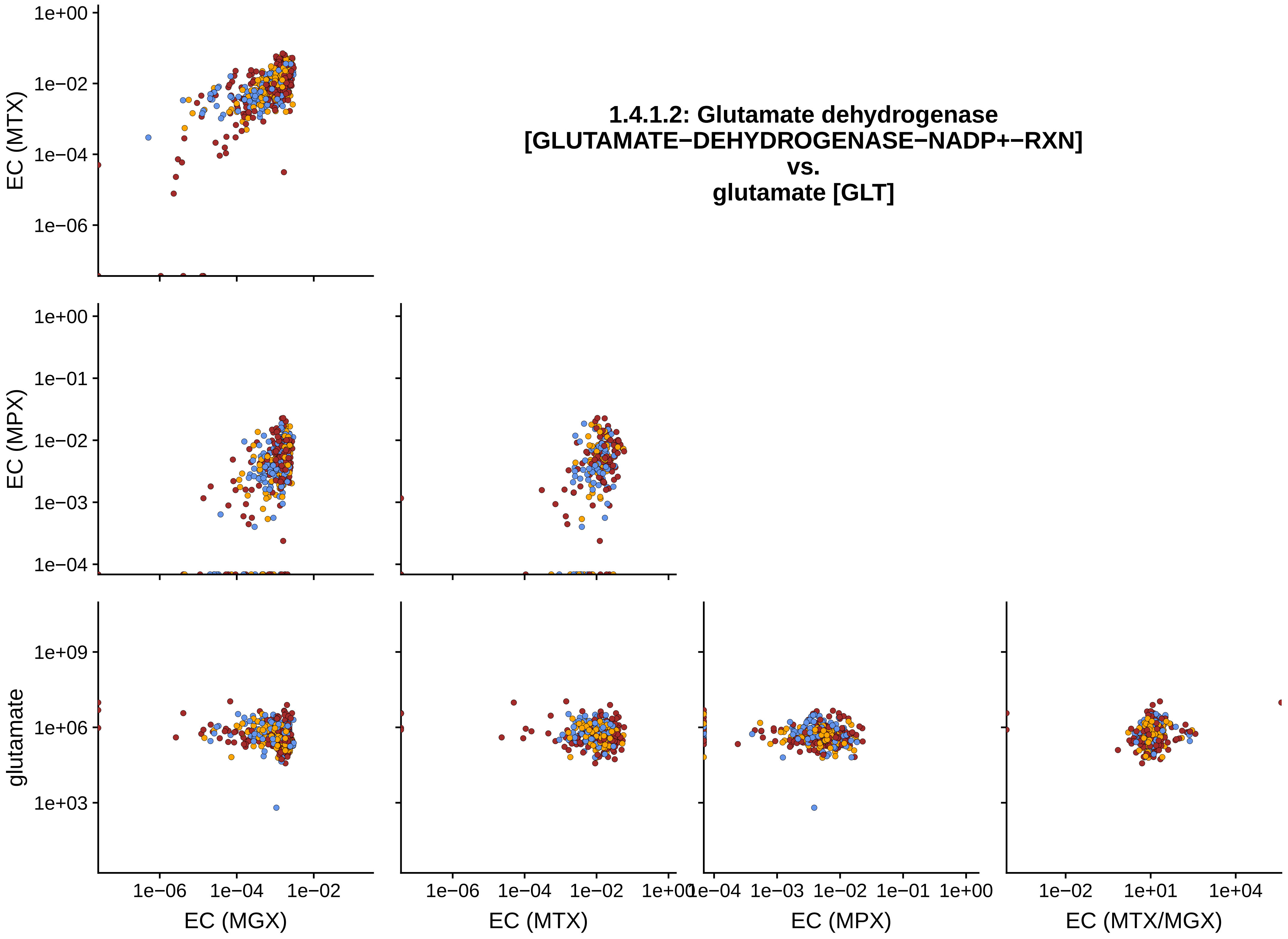
1.4.1.2: Glutamate dehydrogenase
[GLUTAMATE−DEHYDROGENASE−NADP+−RXN]
vs.
glutamate [GLT]
EC (MTX)
EC (MPX)
glutamate
EC (MGX)
EC (MTX)
EC (MPX)
EC (MTX/MGX)
1e+00
1e−02
1e−04
1e−06
1e+00
1e−01
1e−02
1e−03
1e−04
1e+09
1e+06
1e+03
1e−06
1e−04
1e−02
1e−06
1e−04
1e−02
1e+00
1e−04
1e−03
1e−02
1e−01
1e+00
1e−02
1e+01
1e+04

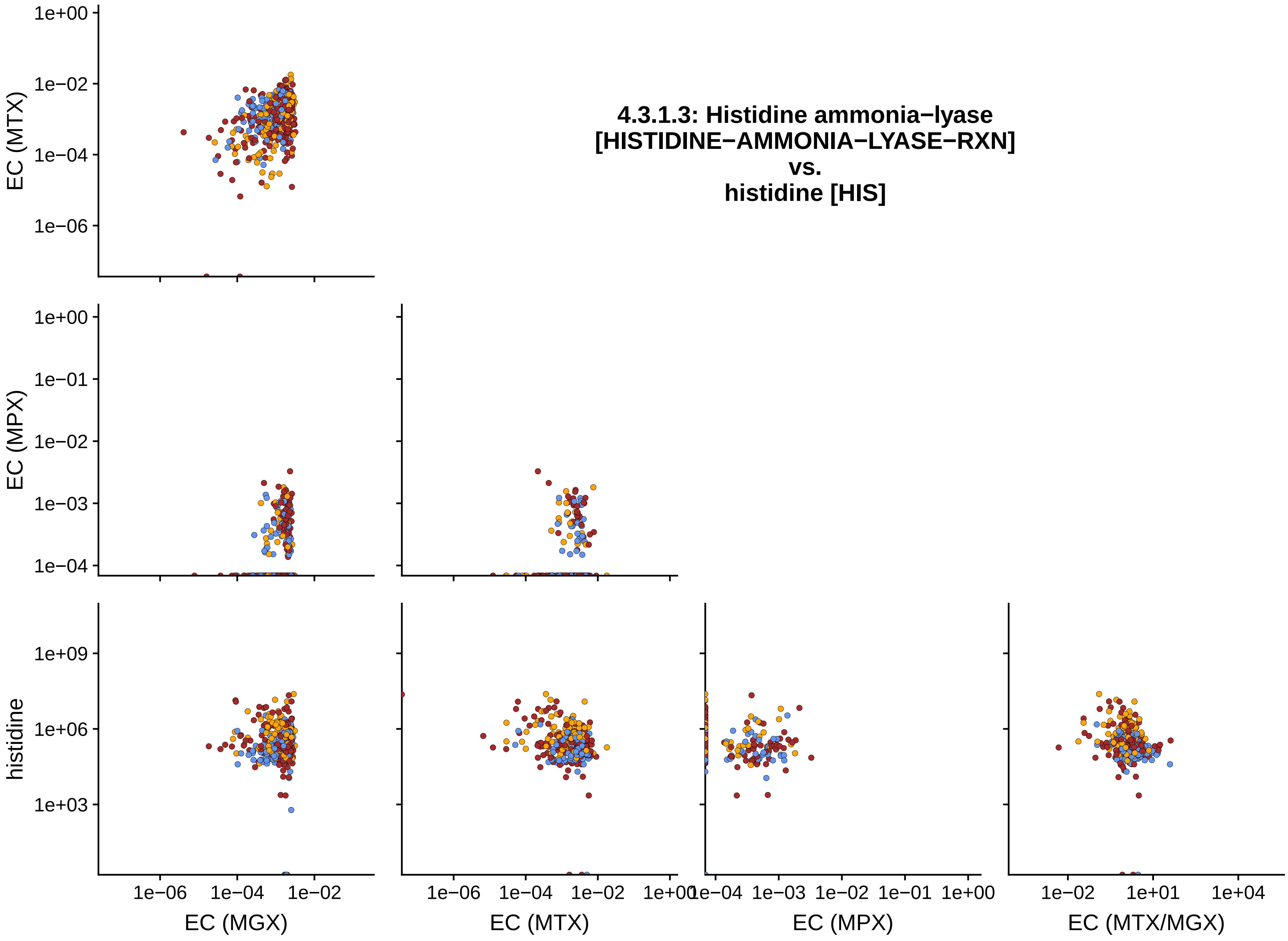

4.3.1.3: Histidine ammonia−lyase
[HISTIDINE−AMMONIA−LYASE−RXN]
vs.
histidine [HIS]
EC (MTX)
EC (MPX)
histidine
EC (MGX)
EC (MTX)
EC (MPX)
EC (MTX/MGX)
1e+00
1e−02
1e−04
1e−06
1e+00
1e−01
1e−02
1e−03
1e−04
1e+09
1e+06
1e+03
1e−06
1e−04
1e−02
1e−06
1e−04
1e−02
1e+00
1e−04
1e−03
1e−02
1e−01
1e+00
1e−02
1e+01
1e+04

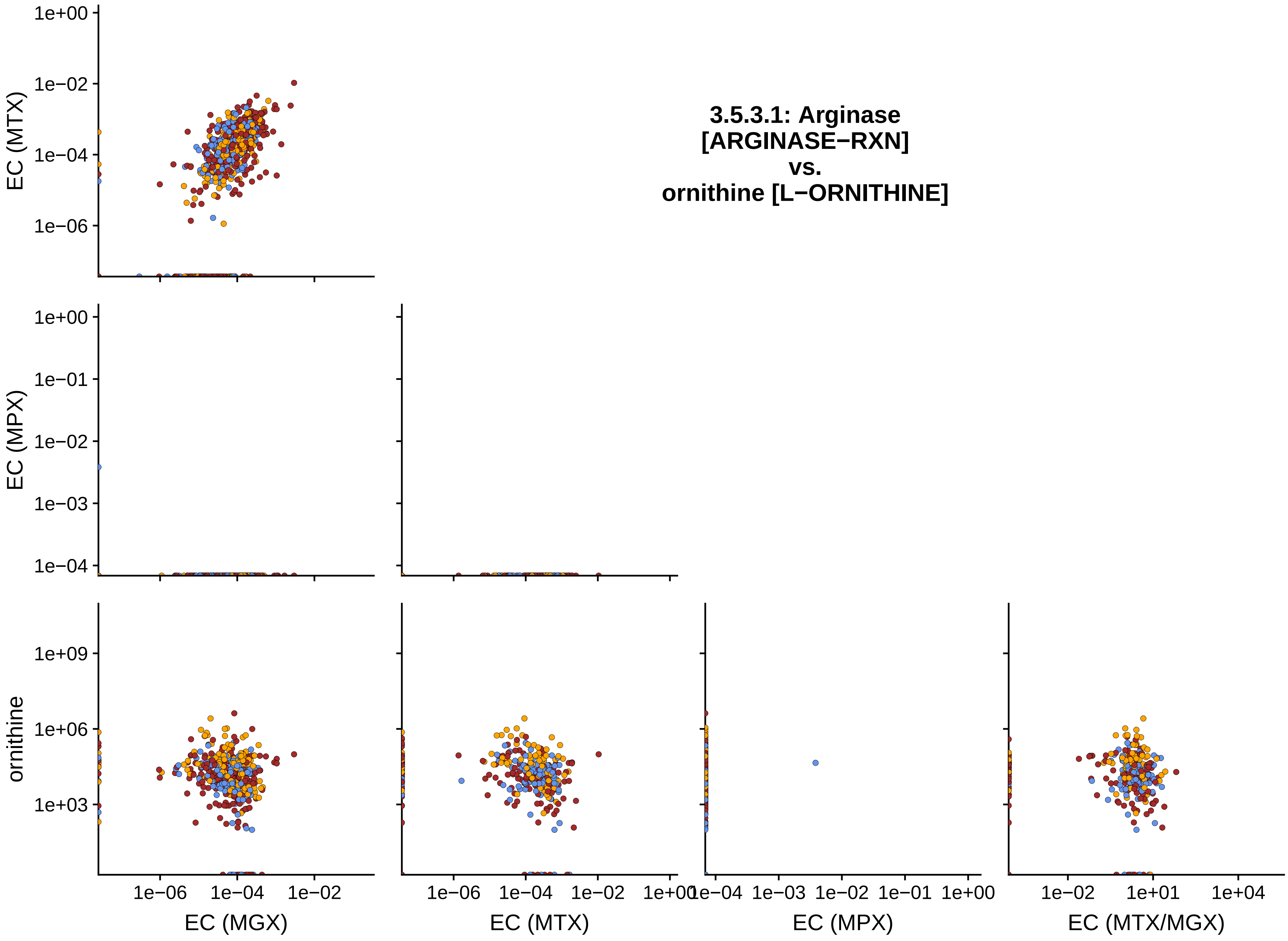
3.5.3.1: Arginase
[ARGINASE−RXN]
vs.
ornithine [L−ORNITHINE]
EC (MTX)
EC (MPX)
ornithine
EC (MGX)
EC (MTX)
EC (MPX)
EC (MTX/MGX)
1e+00
1e−02
1e−04
1e−06
1e−01
1e−03
1e+09
1e+06
1e+03
1e+01
1e+04

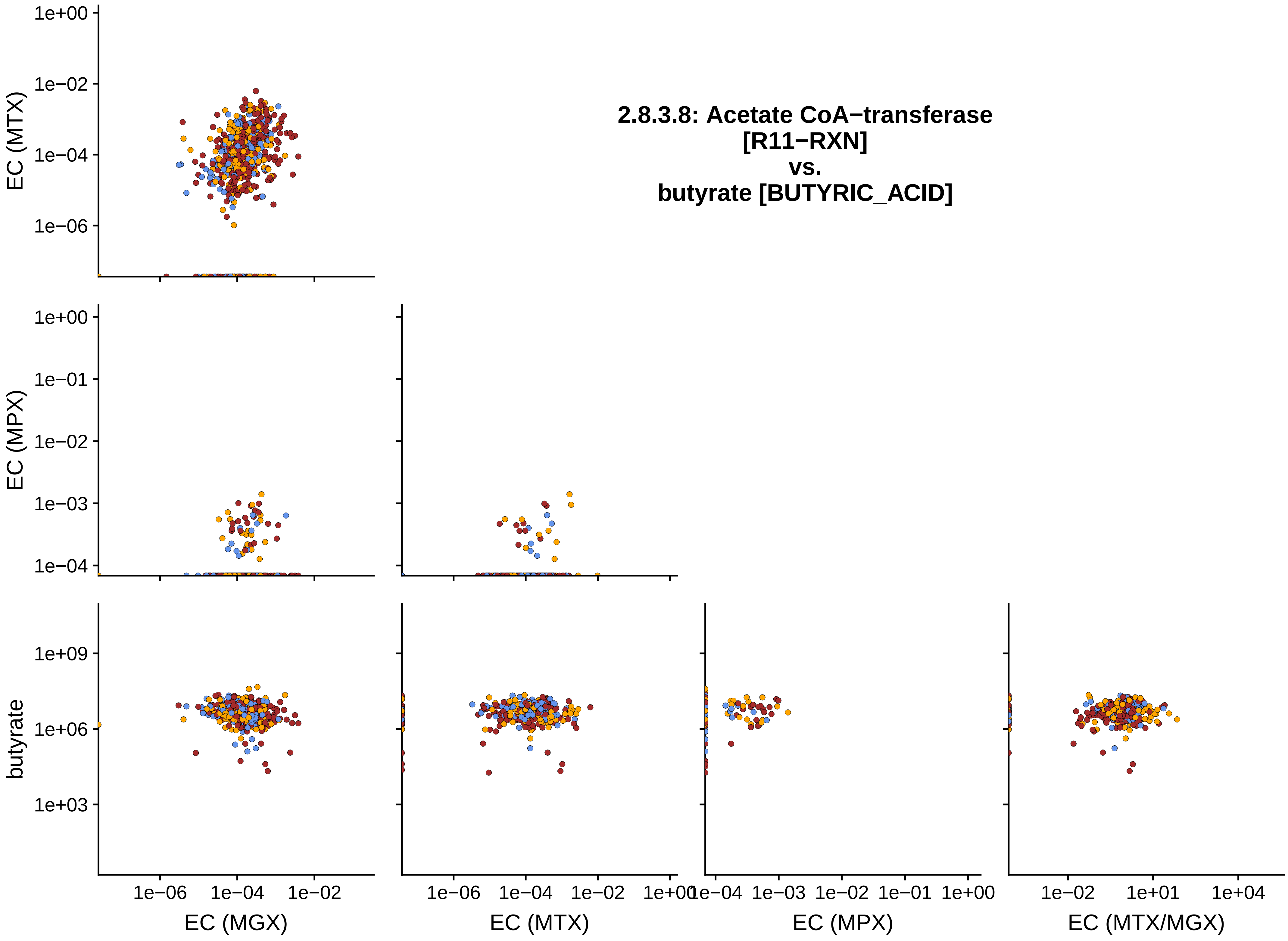
2.8.3.8: Acetate CoA−transferase
[R11−RXN]
vs.
butyrate [BUTYRIC_ACID]
EC (MTX)
EC (MPX)
butyrate
EC (MGX)
EC (MTX)
EC (MPX)
EC (MTX/MGX)
1e+00
1e−02
1e−04
1e−06
1e−01
1e−03
1e+09
1e+06
1e+03
1e−06
1e−04
1e−02
1e+00
1e−01
1e+01
1e+04

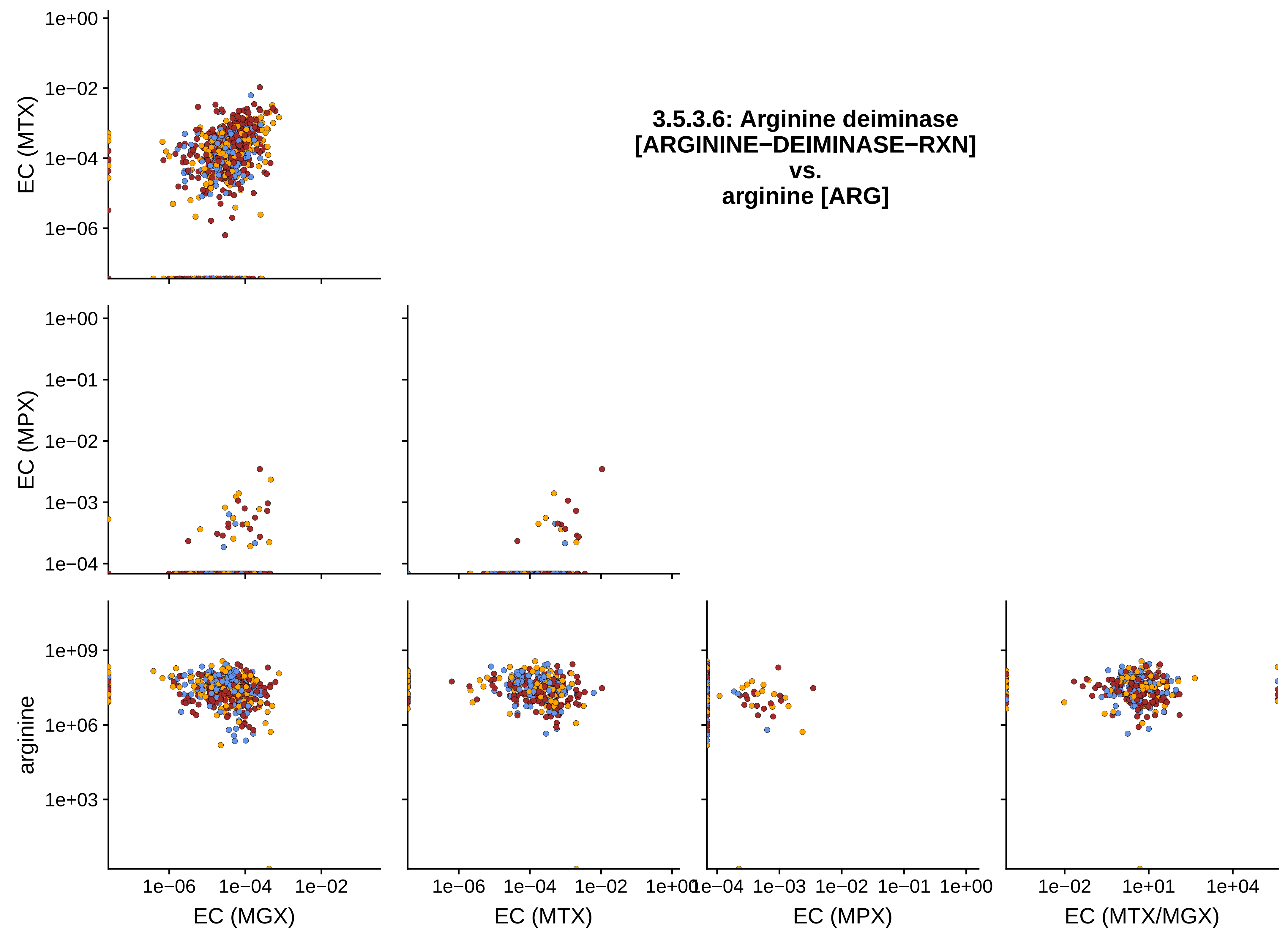

3.5.3.6: Arginine deiminase
[ARGININE−DEIMINASE−RXN]
vs.
arginine [ARG]
EC (MTX)
EC (MPX)
arginine
EC (MGX)
EC (MTX)
EC (MPX)
EC (MTX/MGX)
1e+00
1e−02
1e−04
1e−06
1e−01
1e−03
1e+09
1e+06
1e+03
1e+04
1e+01

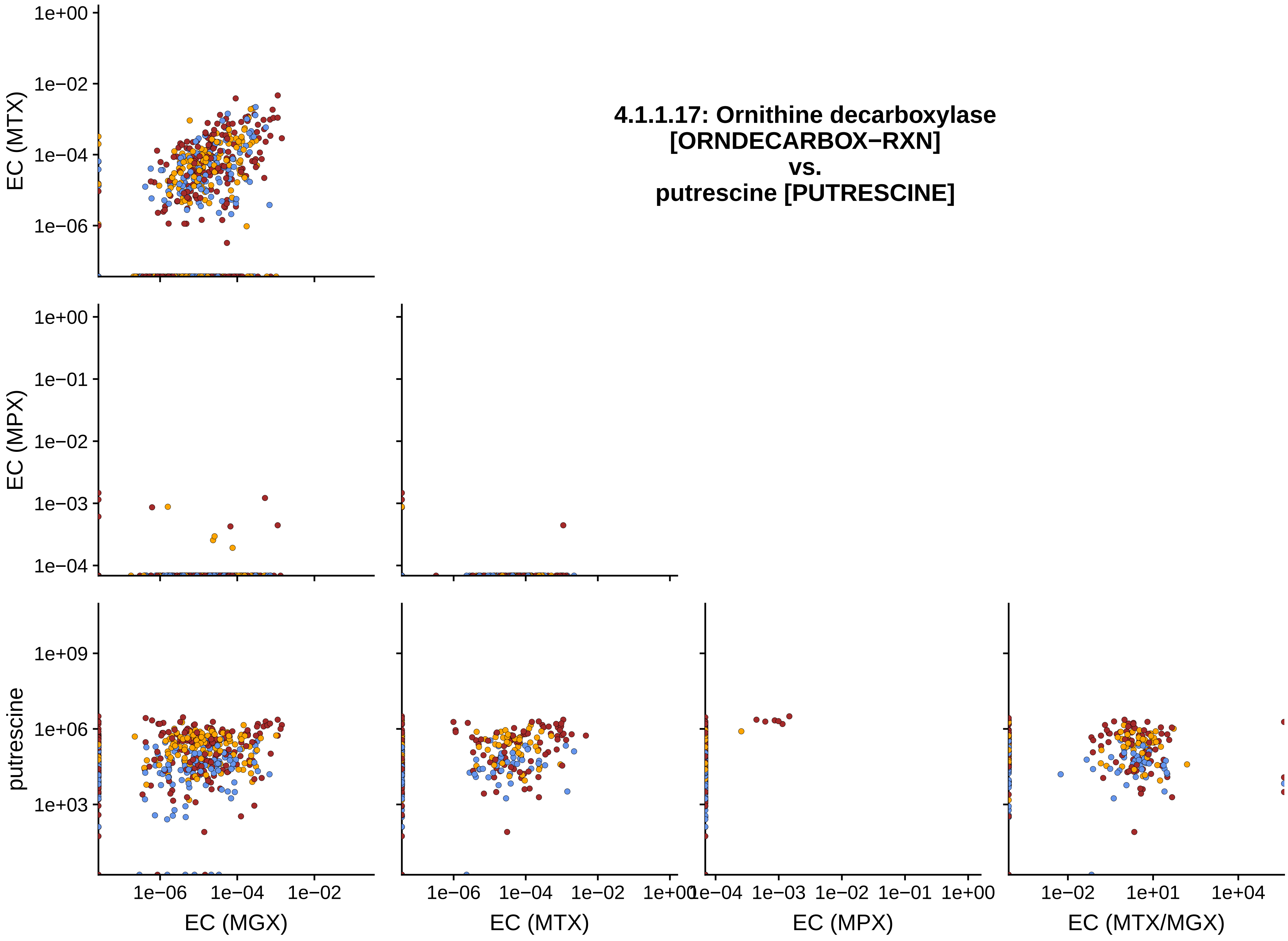
4.1.1.17: Ornithine decarboxylase
[ORNDECARBOX−RXN]
vs.
putrescine [PUTRESCINE]
EC (MTX)
EC (MPX)
putrescine
EC (MGX)
EC (MTX)
EC (MPX)
EC (MTX/MGX)
1e+00
1e−02
1e−04
1e−06
1e−01
1e−03
1e+09
1e+06
1e+03
1e+01
1e+04

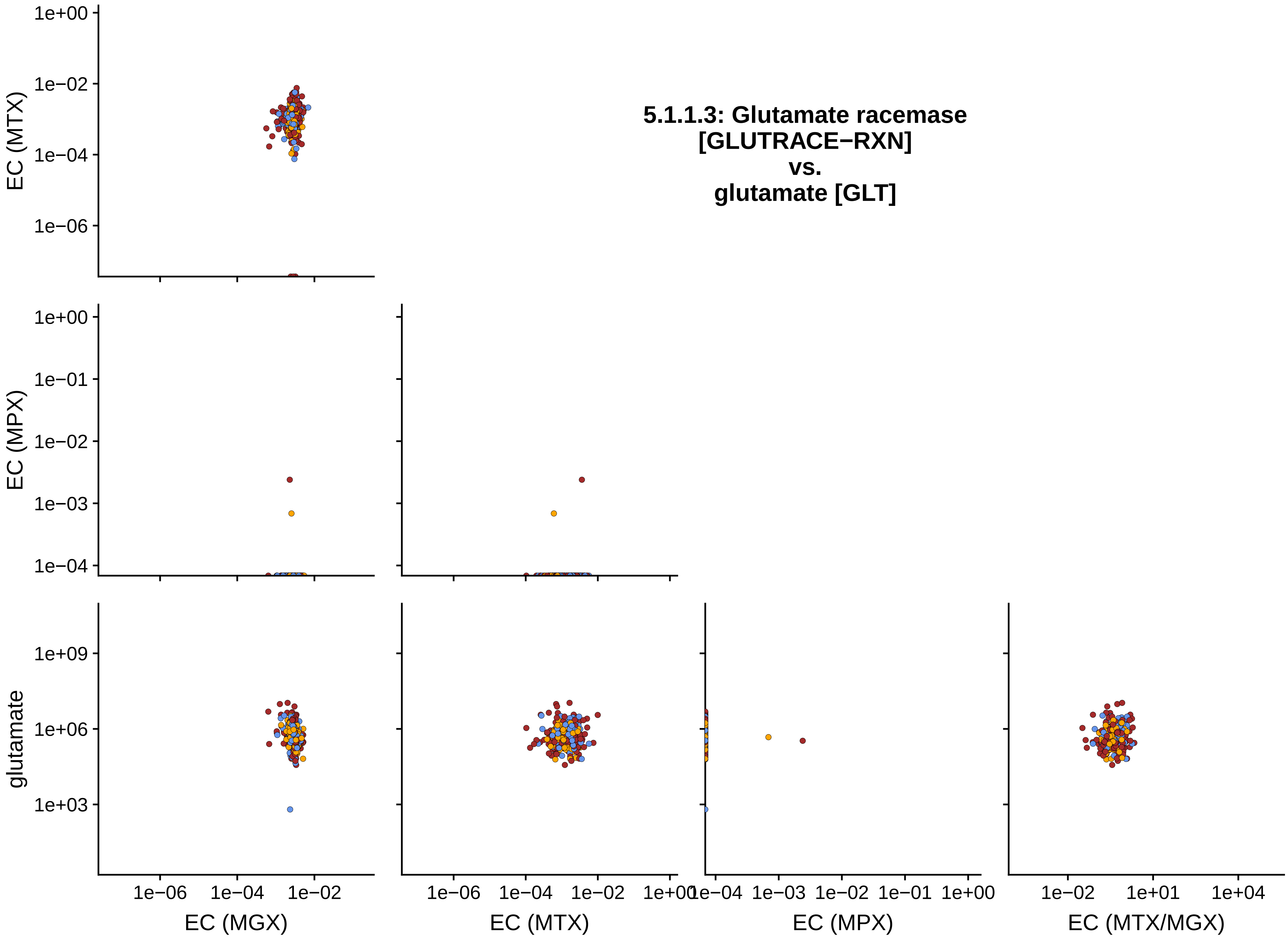

5.1.1.3: Glutamate racemase
[GLUTRACE−RXN]
vs.
glutamate [GLT]
EC (MTX)
EC (MPX)
glutamate
EC (MGX)
EC (MTX)
EC (MPX)
EC (MTX/MGX)
1e+00
1e−02
1e−04
1e−06
1e−01
1e−03
1e+09
1e+06
1e+03
1e+01
1e+04

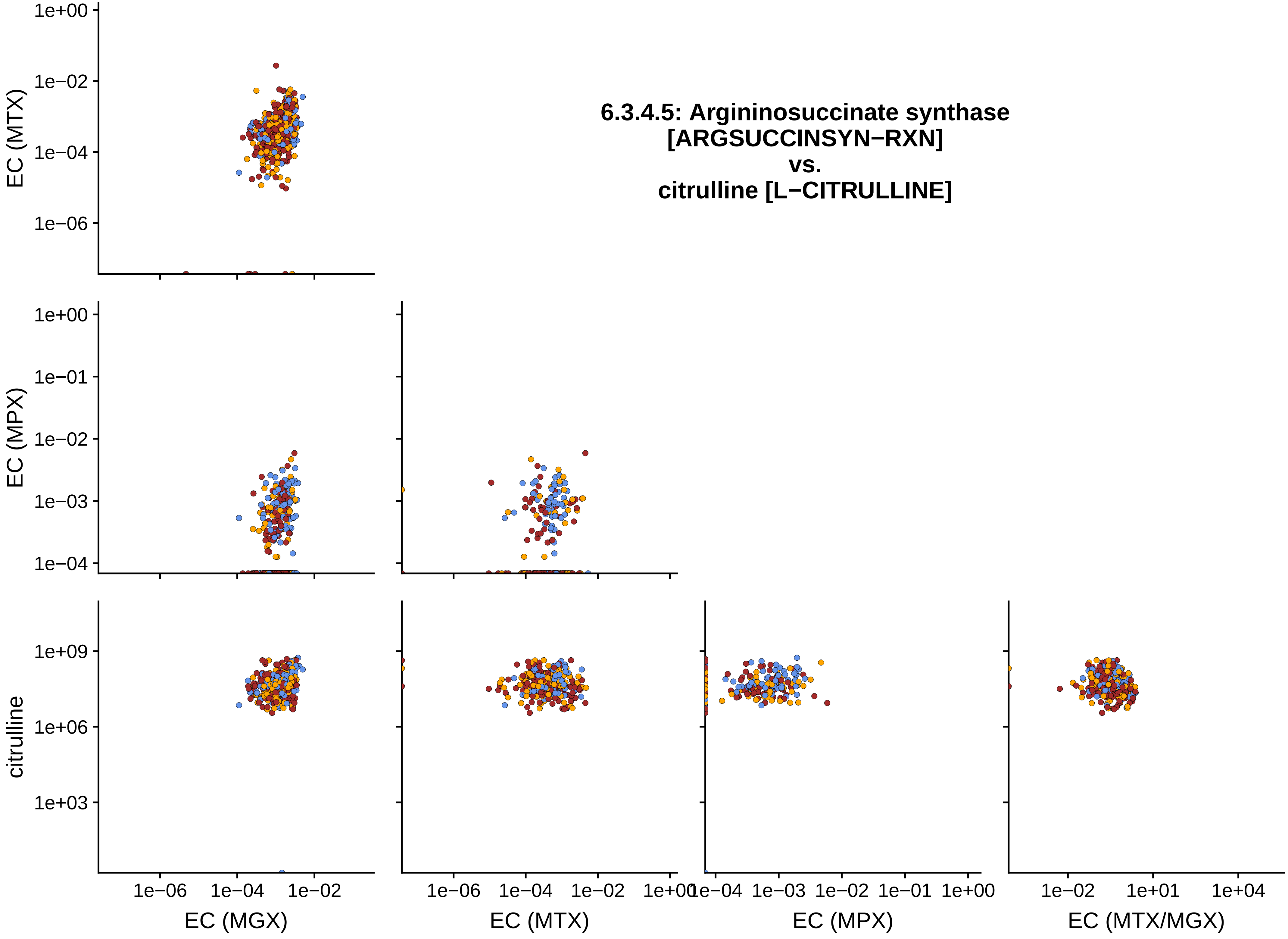

6.3.4.5: Argininosuccinate synthase
[ARGSUCCINSYN−RXN]
vs.
citrulline [L−CITRULLINE]
EC (MTX)
EC (MPX)
citrulline
EC (MGX)
EC (MTX)
EC (MPX)
EC (MTX/MGX)
1e+00
1e−02
1e−04
1e−06
1e−01
1e−03
1e+09
1e+06
1e+03
1e+01
1e+04

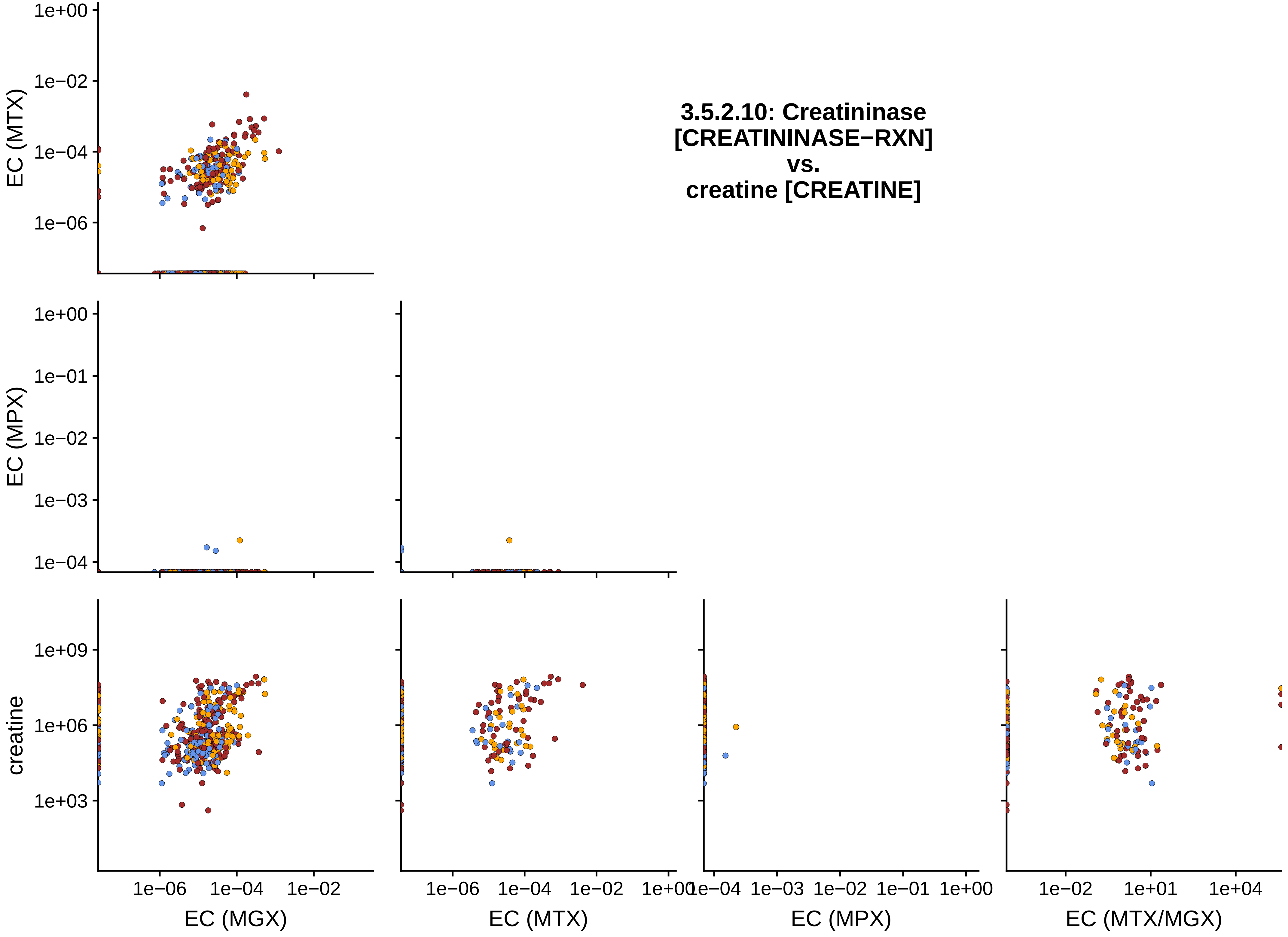
3.5.2.10: Creatininase
[CREATININASE−RXN]
vs.
creatine [CREATINE]
EC (MTX)
EC (MPX)
creatine
EC (MGX)
EC (MTX)
EC (MPX)
EC (MTX/MGX)
1e+00
1e−02
1e−04
1e−06
1e−01
1e−03
1e+09
1e+06
1e+03
1e+04
1e+01

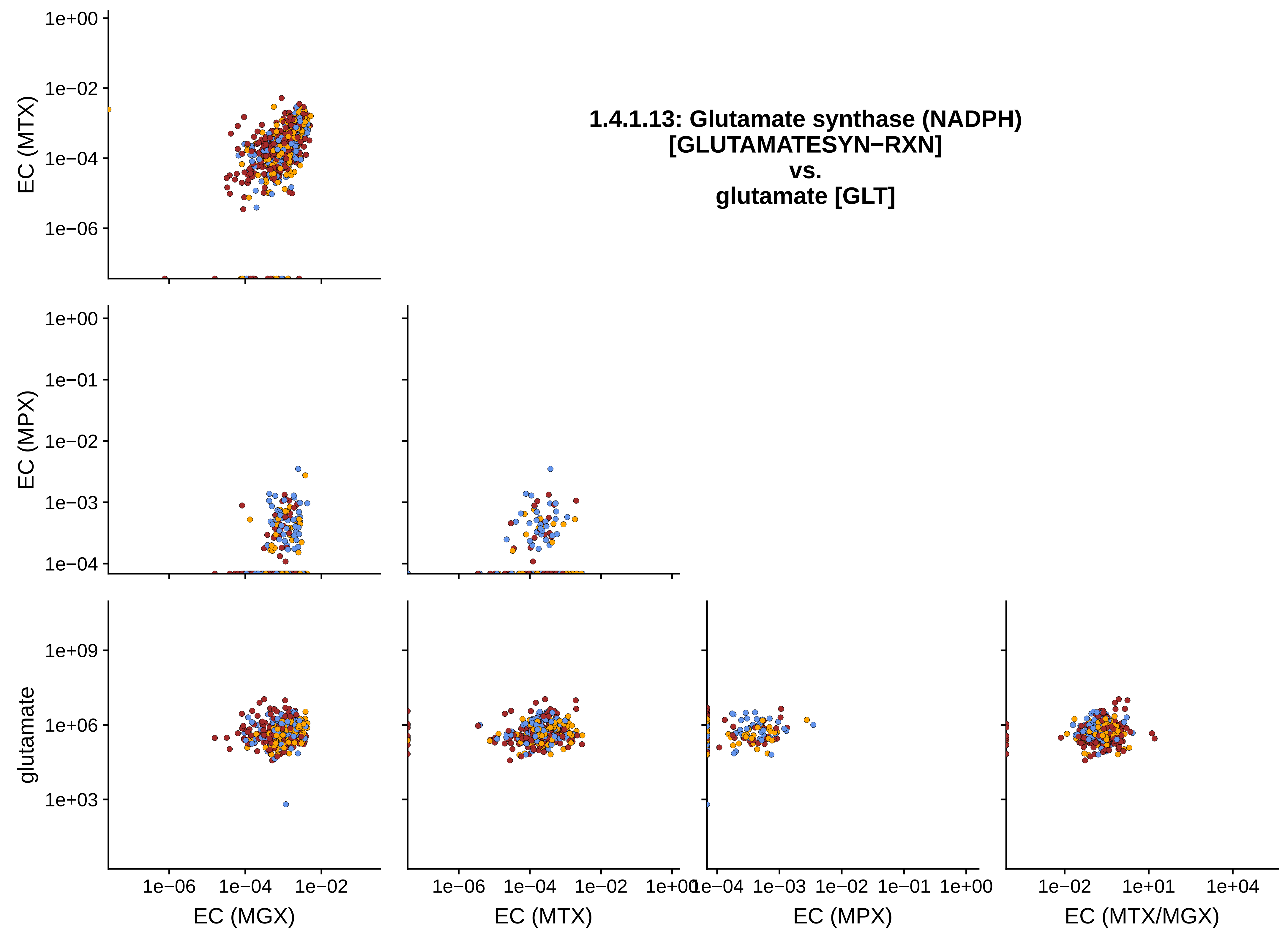

1.4.1.13: Glutamate synthase (NADPH)
[GLUTAMATESYN−RXN]
vs.
glutamate [GLT]
EC (MTX)
EC (MPX)
glutamate
EC (MGX)
EC (MTX)
EC (MPX)
EC (MTX/MGX)
1e+00
1e−02
1e−04
1e−06
1e−01
1e−03
1e+09
1e+06
1e+03
1e+01
1e+04

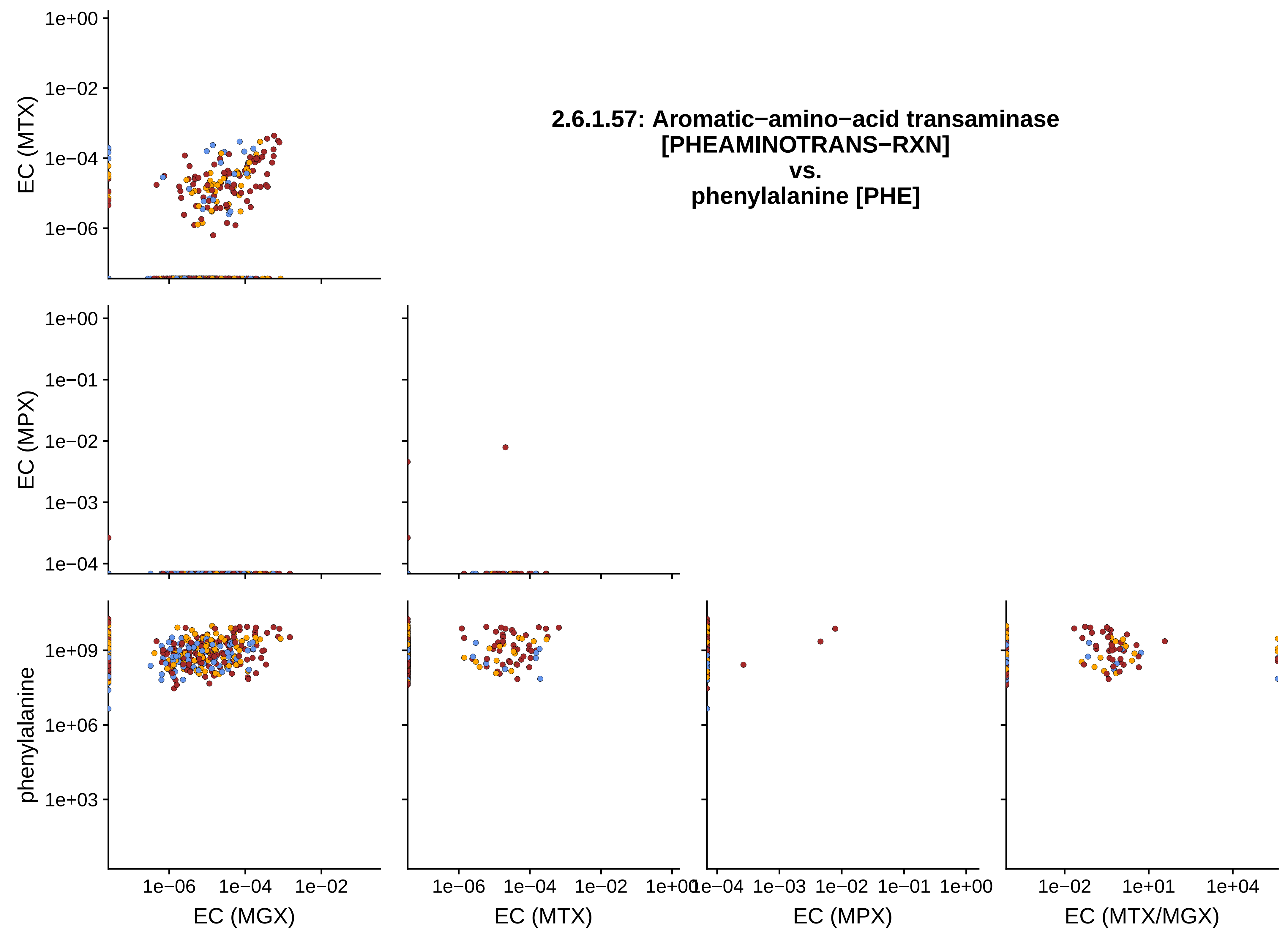

2.6.1.57: Aromatic−amino−acid transaminase
[PHEAMINOTRANS−RXN]
vs.
phenylalanine [PHE]
EC (MTX)
EC (MPX)
phenylalanine
EC (MGX)
EC (MTX)
EC (MPX)
EC (MTX/MGX)
1e+00
1e−02
1e−04
1e−06
1e−01
1e−03
1e+09
1e+06
1e+03
1e+01
1e+04

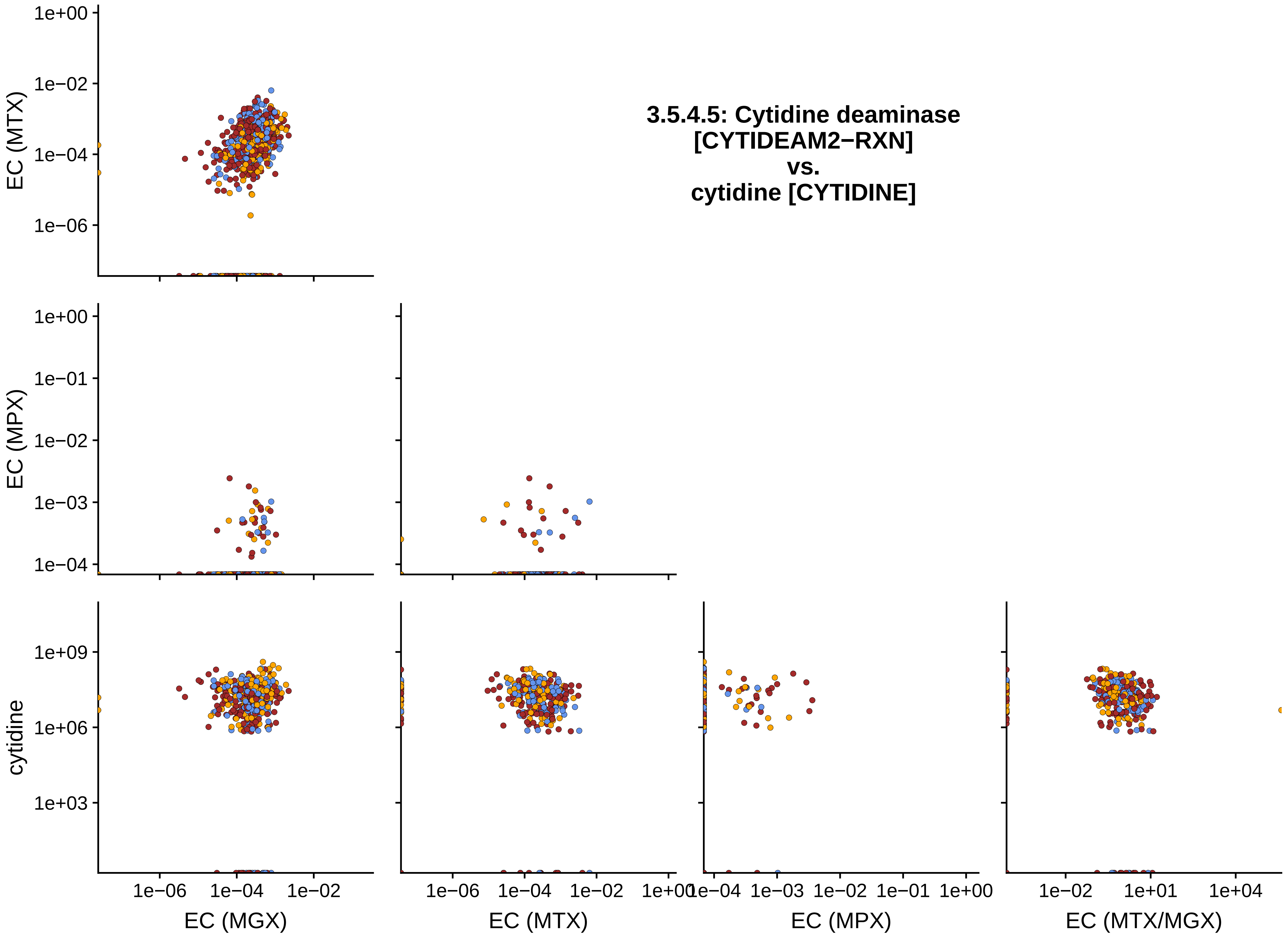
3.5.4.5: Cytidine deaminase
[CYTIDEAM2−RXN]
vs.
cytidine [CYTIDINE]
EC (MTX)
EC (MPX)
cytidine
EC (MGX)
EC (MTX)
EC (MPX)
EC (MTX/MGX)
1e+00
1e−02
1e−04
1e−06
1e−01
1e−03
1e+09
1e+06
1e+03
1e+01
1e+04

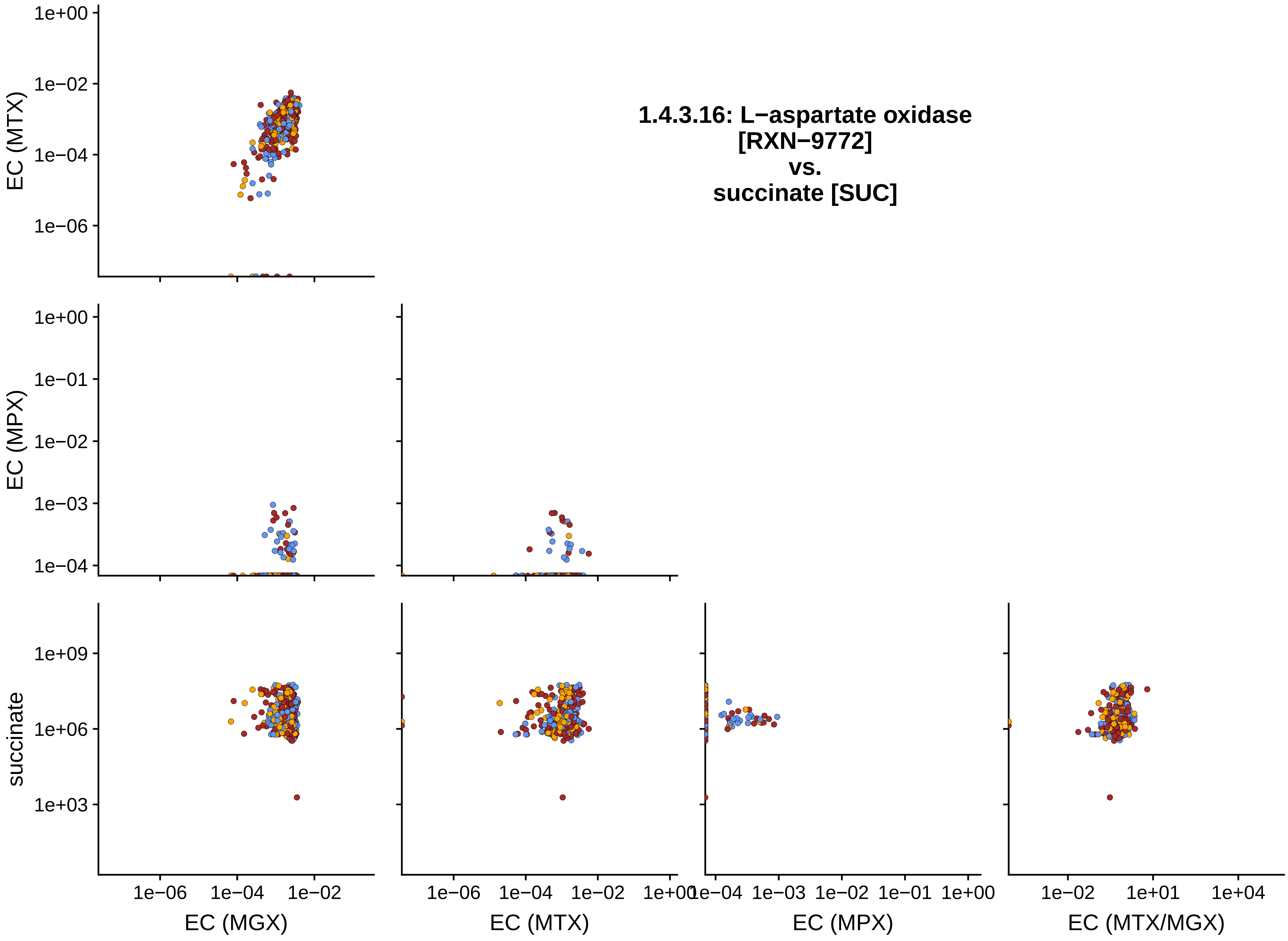
1.4.3.16: L−aspartate oxidase
[RXN−9772]
vs.
succinate [SUC]
EC (MTX)
EC (MPX)
succinate
EC (MGX)
EC (MTX)
EC (MPX)
EC (MTX/MGX)
1e+00
1e−02
1e−04
1e−06
1e−01
1e−03
1e+09
1e+06
1e+03
1e+01
1e+04

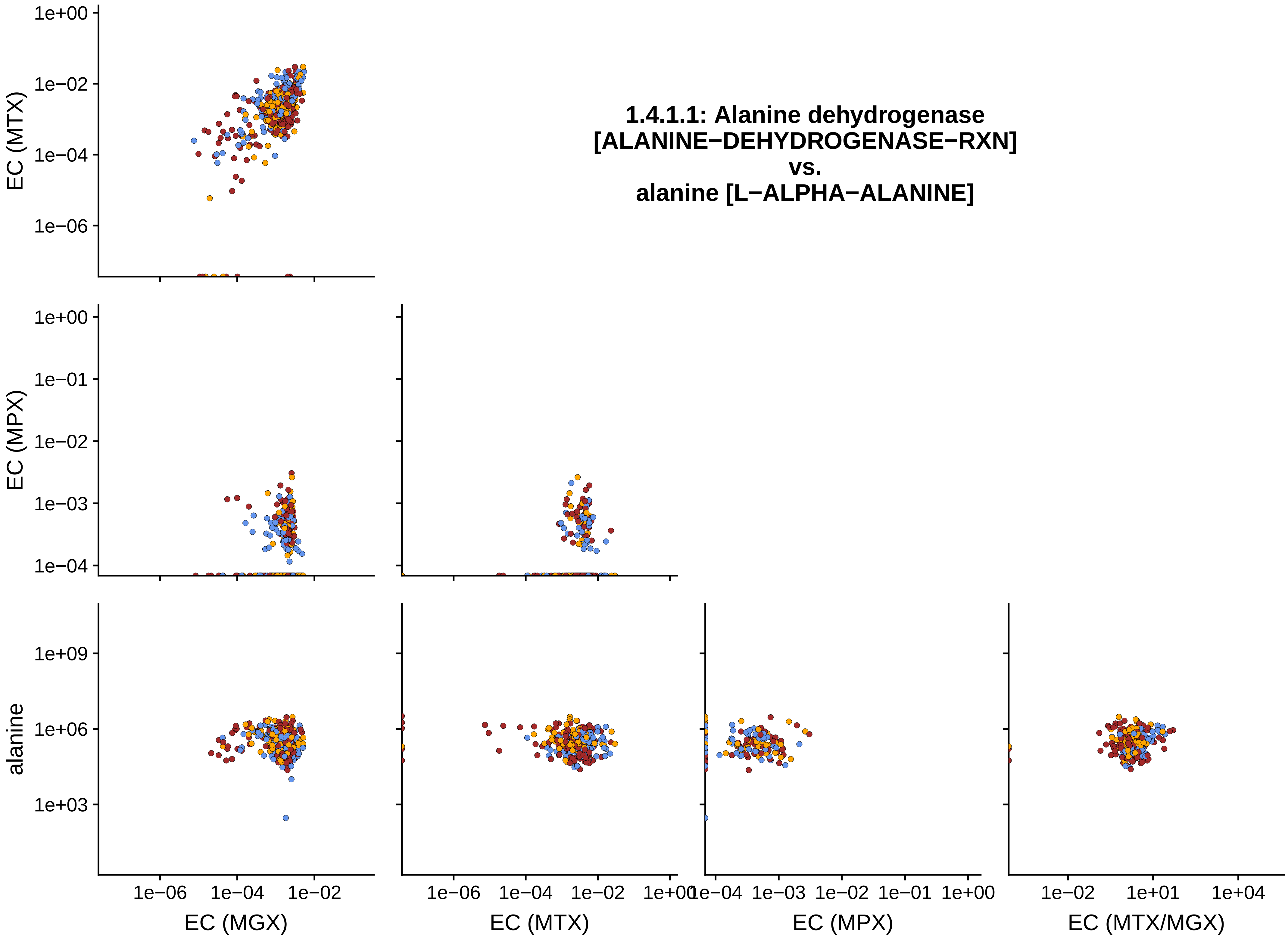
1.4.1.1: Alanine dehydrogenase
[ALANINE−DEHYDROGENASE−RXN]
vs.
alanine [L−ALPHA−ALANINE]
EC (MTX)
EC (MPX)
alanine
EC (MGX)
EC (MTX)
EC (MPX)
EC (MTX/MGX)
1e+00
1e−02
1e−04
1e−06
1e−01
1e−03
1e+09
1e+06
1e+03
1e+01
1e+04

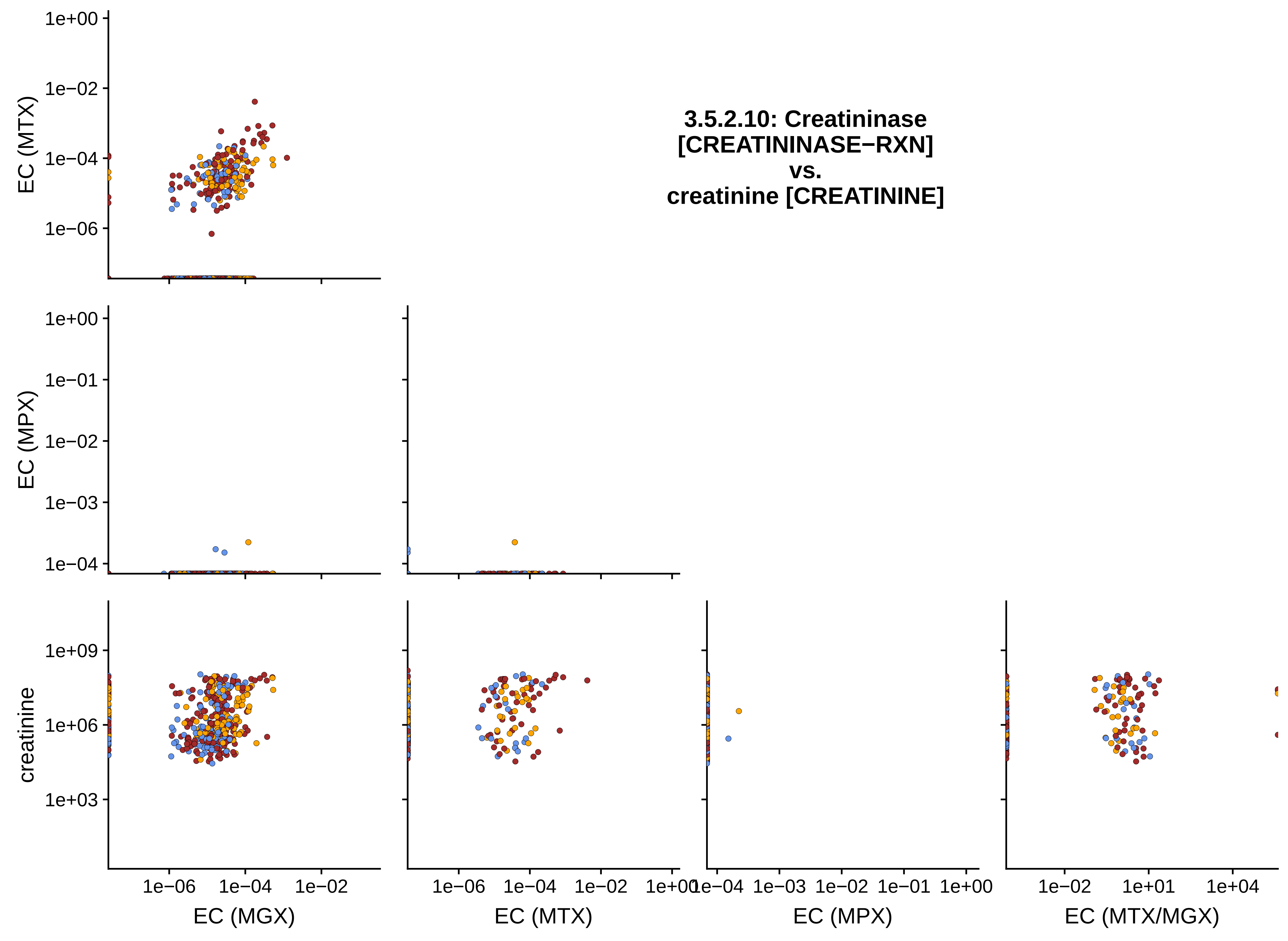
3.5.2.10: Creatininase
[CREATININASE−RXN]
vs.
creatinine [CREATININE]
EC (MTX)
EC (MPX)
creatinine
EC (MGX)
EC (MTX)
EC (MPX)
EC (MTX/MGX)
1e+00
1e−02
1e−04
1e−06
1e−01
1e−03
1e+09
1e+06
1e+03
1e+04

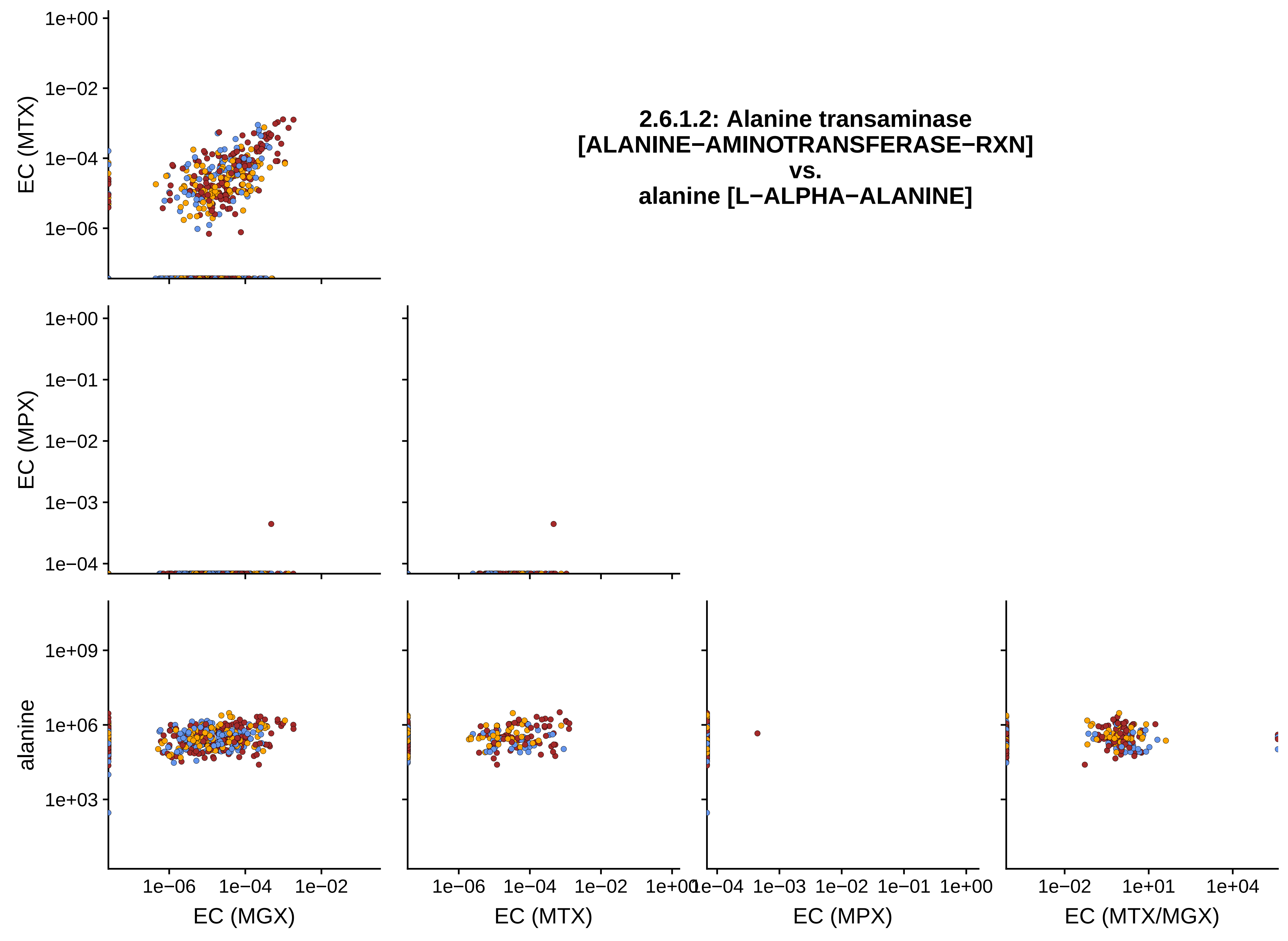
2.6.1.2: Alanine transaminase
[ALANINE−AMINOTRANSFERASE−RXN]
vs.
alanine [L−ALPHA−ALANINE]
EC (MTX)
EC (MPX)
alanine
EC (MGX)
EC (MTX)
EC (MPX)
EC (MTX/MGX)
1e+00
1e−02
1e−04
1e−06
1e−01
1e−03
1e+09
1e+06
1e+03
1e+01
1e+04

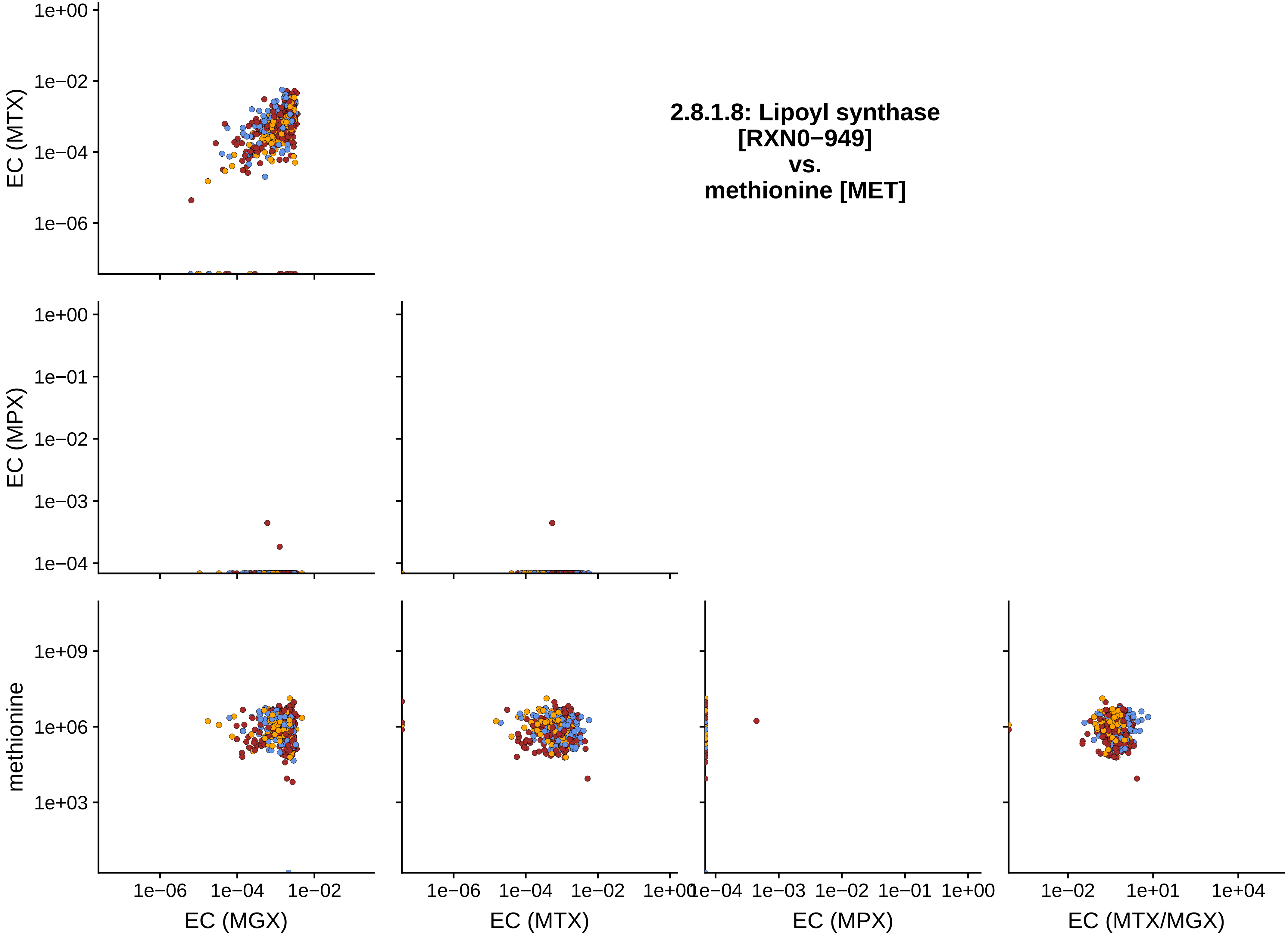
2.8.1.8: Lipoyl synthase
[RXN0−949]
vs.
methionine [MET]
EC (MTX)
EC (MPX)
methionine
EC (MGX)
EC (MTX)
EC (MPX)
EC (MTX/MGX)
1e+00
1e−02
1e−04
1e−06
1e−01
1e−03
1e+09
1e+06
1e+03
1e+01
1e+04

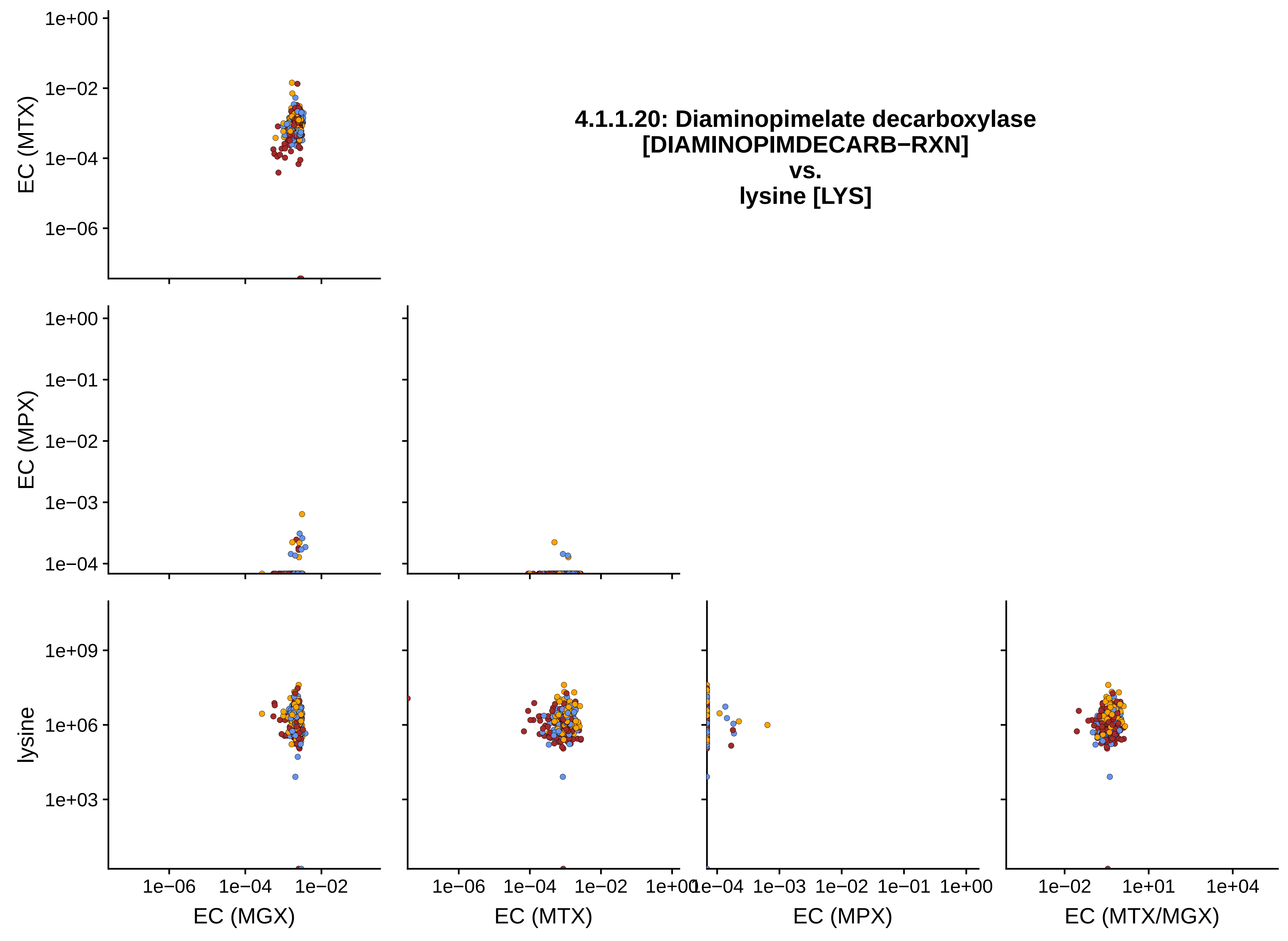
4.1.1.20: Diaminopimelate decarboxylase
[DIAMINOPIMDECARB−RXN]
vs.
lysine [LYS]
EC (MTX)
EC (MPX)
lysine
EC (MGX)
EC (MTX)
EC (MPX)
EC (MTX/MGX)
1e+00
1e−02
1e−04
1e−06
1e+00
1e−01
1e−02
1e−03
1e−04
1e+09
1e+06
1e+03
1e−06
1e−04
1e−02
1e+00
1e−01
1e+04
1e+01

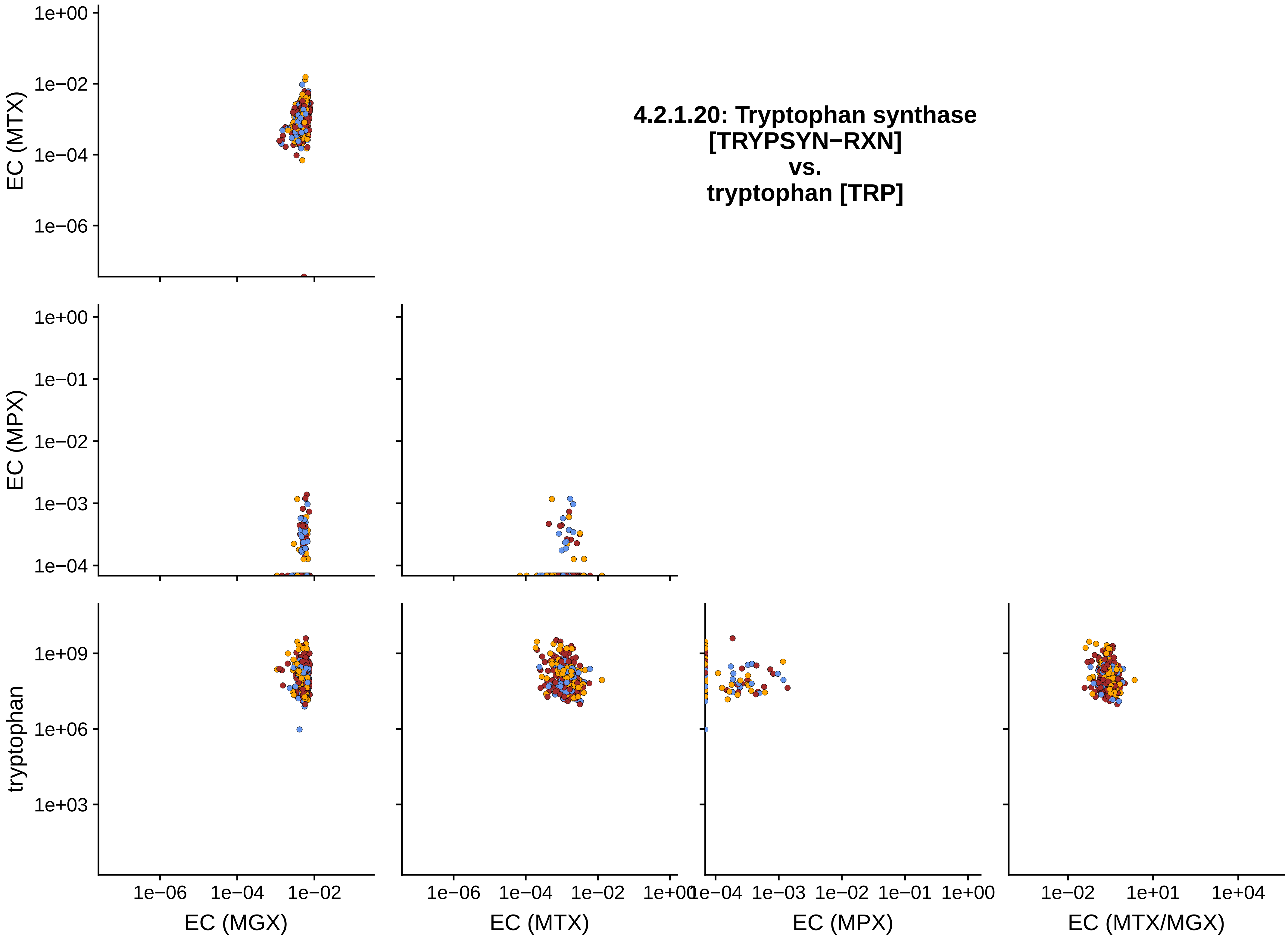

4.2.1.20: Tryptophan synthase
[TRYPSYN−RXN]
vs.
tryptophan [TRP]
EC (MTX)
EC (MPX)
tryptophan
EC (MGX)
EC (MTX)
EC (MPX)
EC (MTX/MGX)
1e+00
1e−02
1e−04
1e−06
1e+00
1e−01
1e−02
1e−03
1e−04
1e+09
1e+06
1e+03
1e−06
1e−04
1e−02
1e−06
1e−04
1e−02
1e+00
1e−04
1e−03
1e−02
1e−01
1e+00
1e−02
1e+01
1e+04

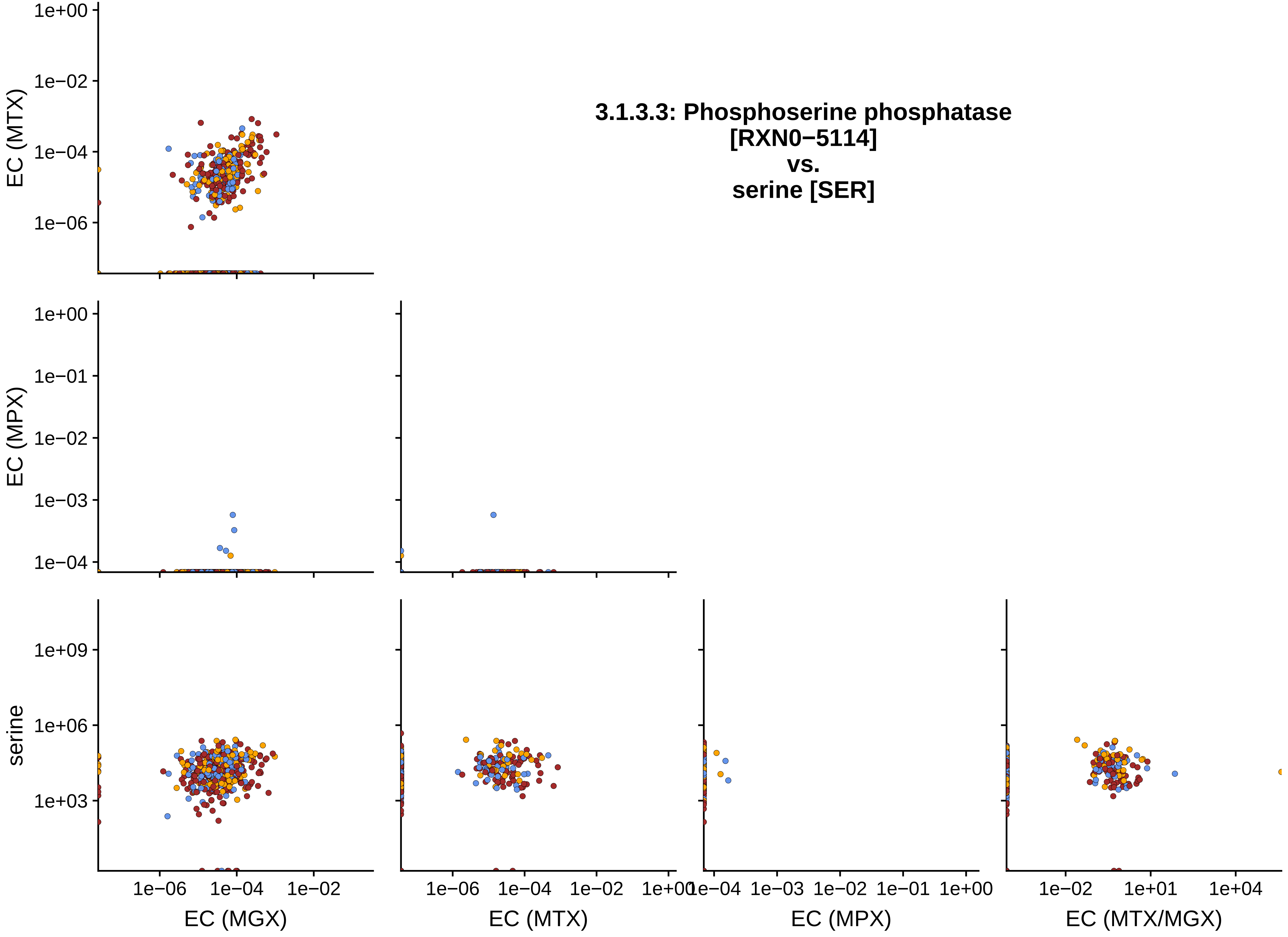
3.1.3.3: Phosphoserine phosphatase
[RXN0−5114]
vs.
serine [SER]
EC (MTX)
EC (MPX)
serine
EC (MGX)
EC (MTX)
EC (MPX)
EC (MTX/MGX)
1e+00
1e−02
1e−04
1e−06
1e−01
1e−03
1e+09
1e+06
1e+03
1e+04
1e+01

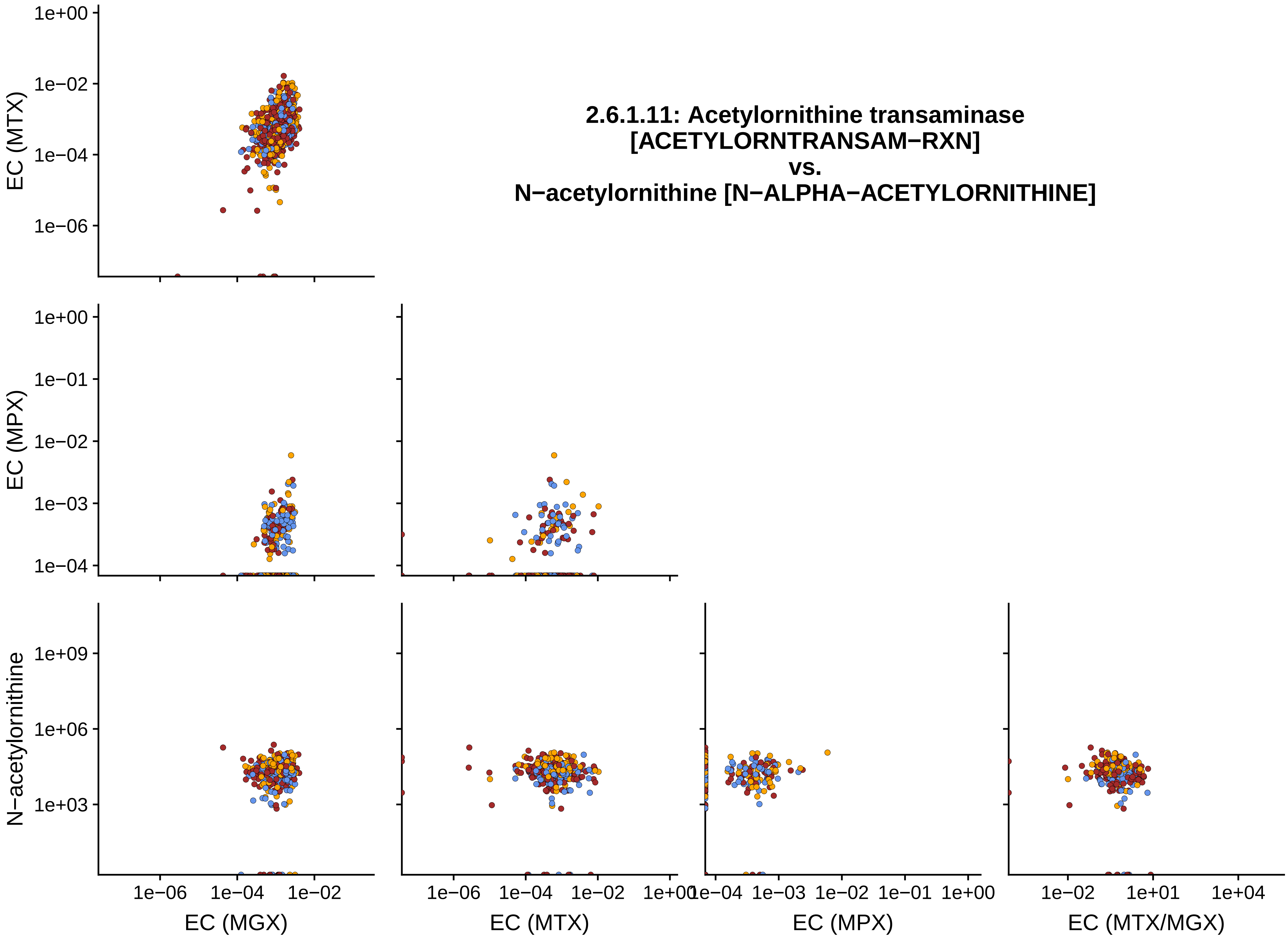
2.6.1.11: Acetylornithine transaminase
[ACETYLORNTRANSAM−RXN]
vs.
N−acetylornithine [N−ALPHA−ACETYLORNITHINE]
EC (MTX)
EC (MPX)
N−acetylornithine
EC (MGX)
EC (MTX)
EC (MPX)
EC (MTX/MGX)
1e+00
1e−02
1e−04
1e−06
1e−01
1e−03
1e+09
1e+06
1e+03
1e+01
1e+04

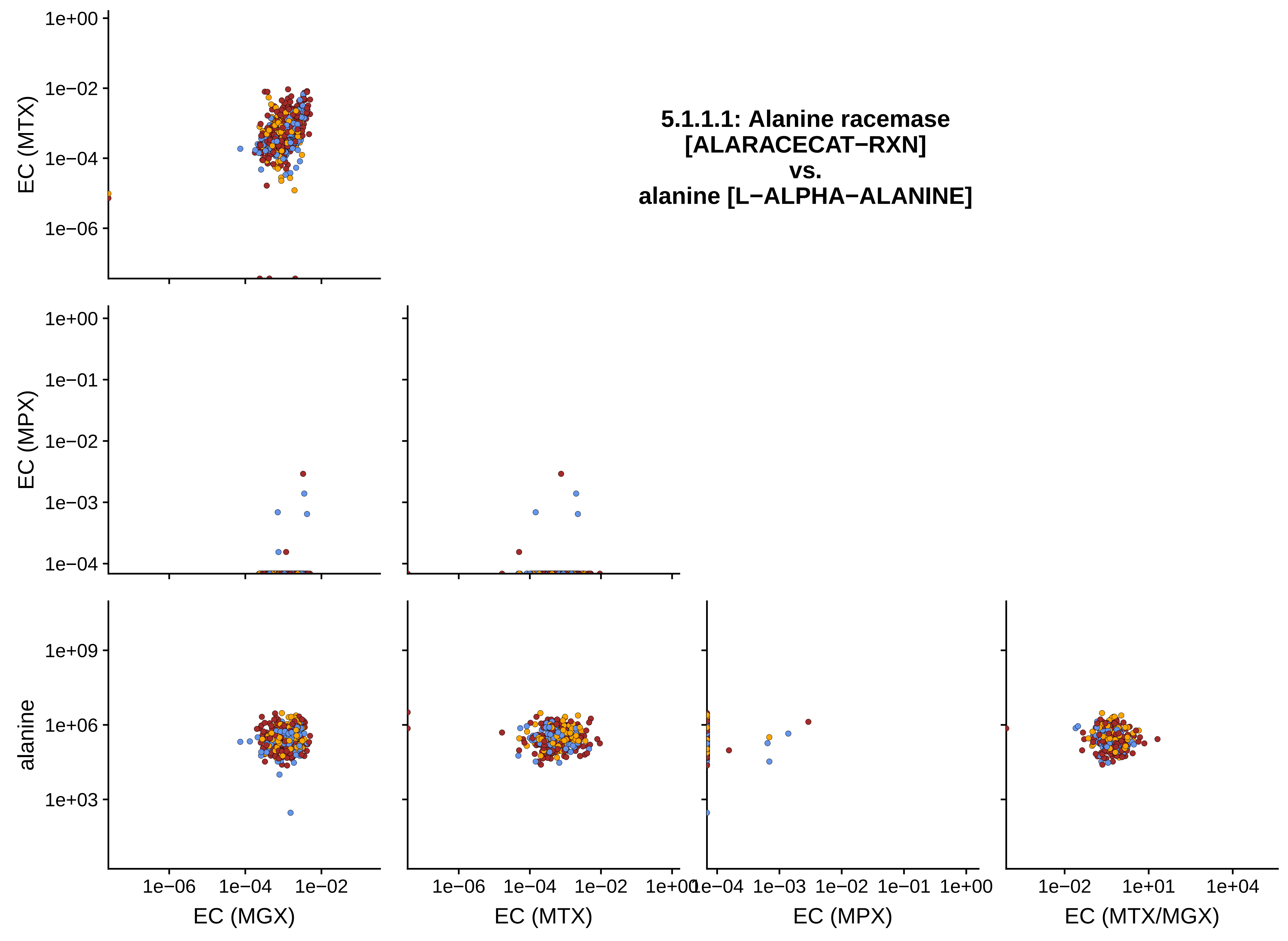

5.1.1.1: Alanine racemase
[ALARACECAT−RXN]
vs.
alanine [L−ALPHA−ALANINE]
EC (MTX)
EC (MPX)
alanine
EC (MGX)
EC (MTX)
EC (MPX)
EC (MTX/MGX)
1e+00
1e−02
1e−04
1e−06
1e−01
1e−03
1e+09
1e+06
1e+03
1e+01
1e+04

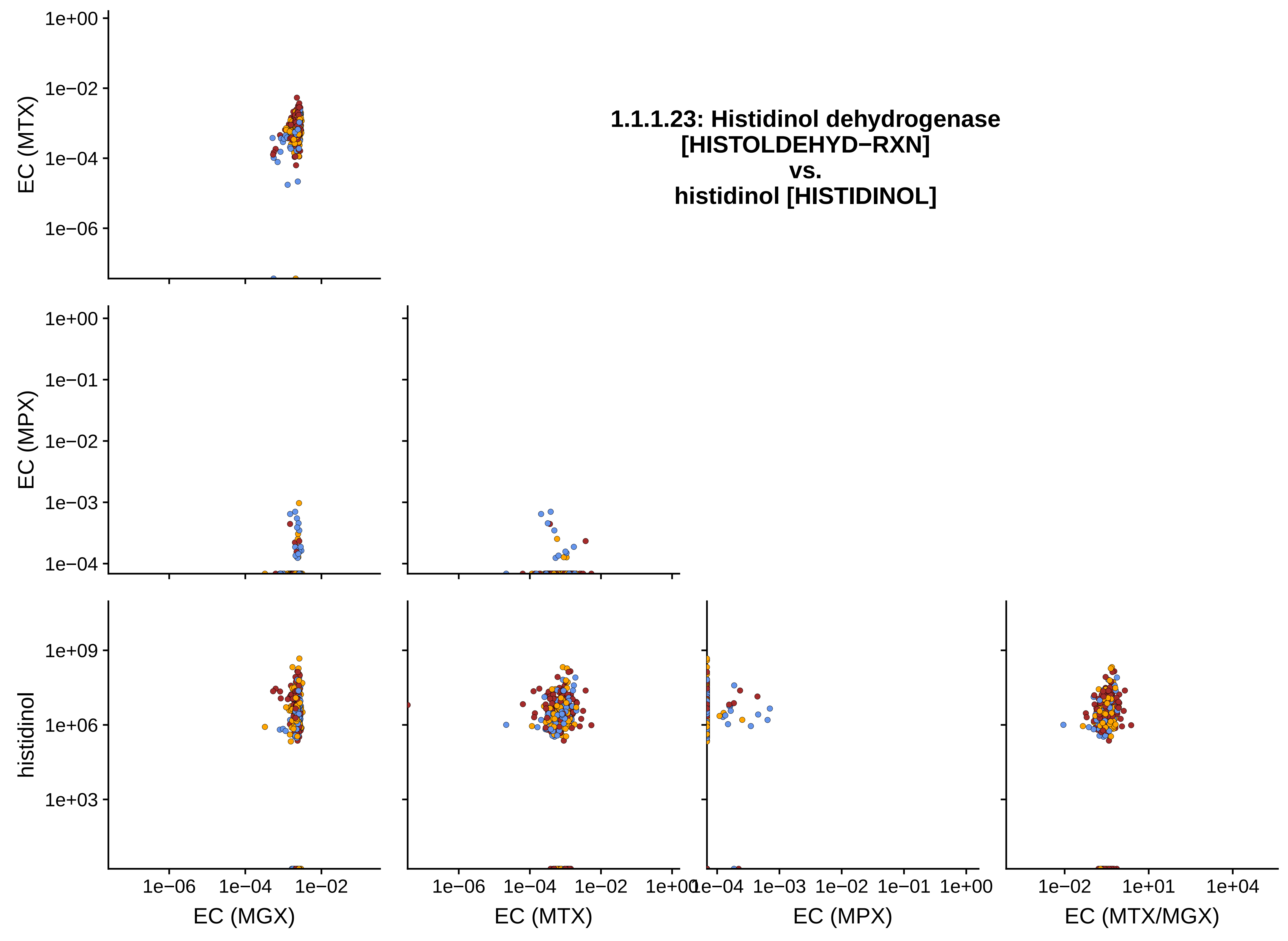
1.1.1.23: Histidinol dehydrogenase
[HISTOLDEHYD−RXN]
vs.
histidinol [HISTIDINOL]
EC (MTX)
EC (MPX)
histidinol
EC (MGX)
EC (MTX)
EC (MPX)
EC (MTX/MGX)
1e+00
1e−02
1e−04
1e−06
1e+00
1e−01
1e−02
1e−03
1e−04
1e+09
1e+06
1e+03
1e−06
1e−04
1e−02
1e−06
1e−04
1e−02
1e+00
1e−04
1e−03
1e−02
1e−01
1e+00
1e−02
1e+01
1e+04

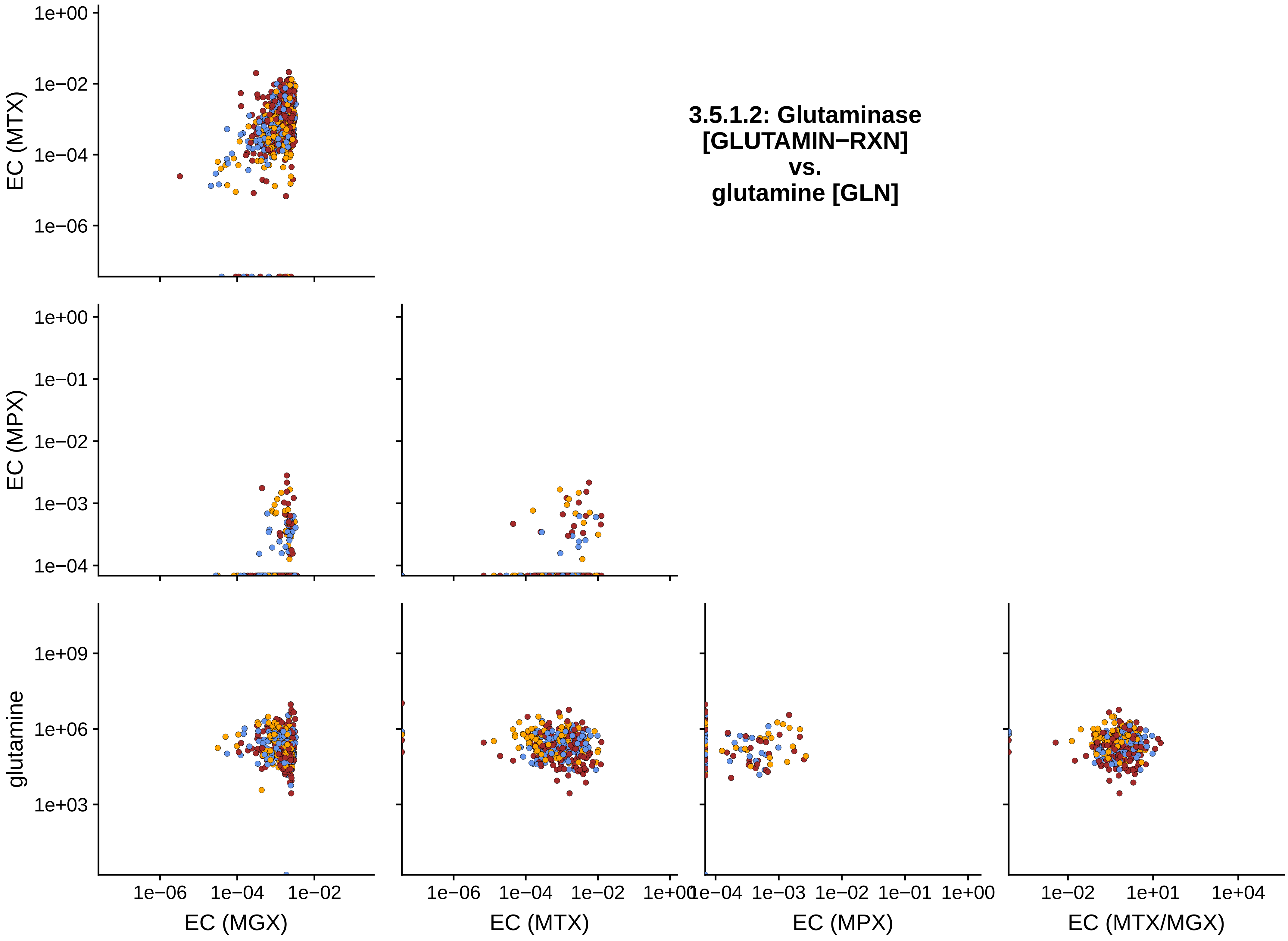

3.5.1.2: Glutaminase
[GLUTAMIN−RXN]
vs.
glutamine [GLN]
EC (MTX)
EC (MPX)
glutamine
EC (MGX)
EC (MTX)
EC (MPX)
EC (MTX/MGX)
1e+00
1e−02
1e−04
1e−06
1e−01
1e−03
1e+09
1e+06
1e+03
1e+01
1e+04

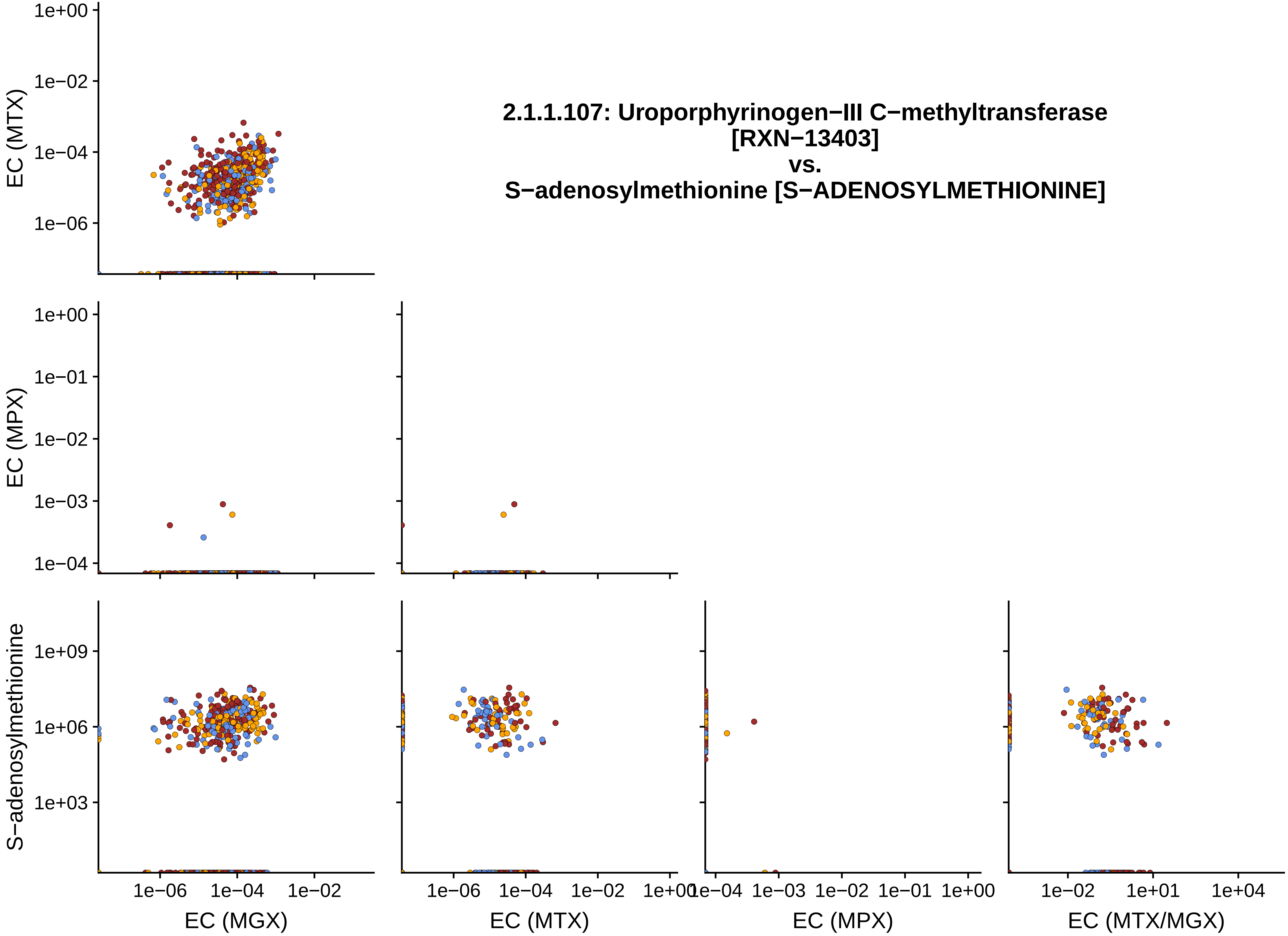

2.1.1.107: Uroporphyrinogen−III C−methyltransferase
[RXN−13403]
vs.
S−adenosylmethionine [S−ADENOSYLMETHIONINE]
EC (MTX)
EC (MPX)
S−adenosylmethionine
EC (MGX)
EC (MTX)
EC (MPX)
EC (MTX/MGX)
1e+00
1e−02
1e−04
1e−06
1e−01
1e−03
1e+09
1e+06
1e+03
1e+01
1e+04

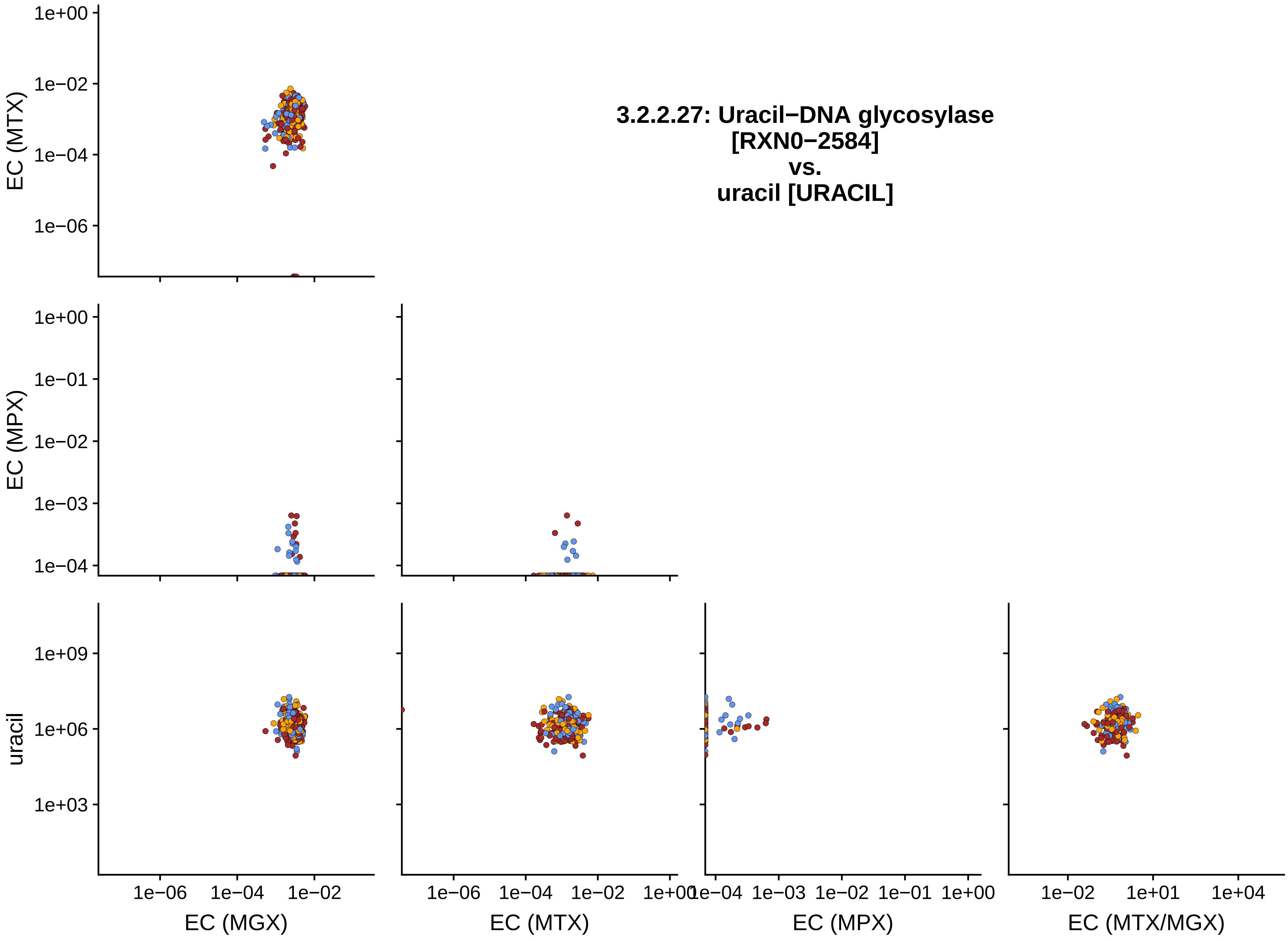

3.2.2.27: Uracil−DNA glycosylase
[RXN0−2584]
vs.
uracil [URACIL]
EC (MTX)
EC (MPX)
uracil
EC (MGX)
EC (MTX)
EC (MPX)
EC (MTX/MGX)
1e+00
1e−02
1e−04
1e−06
1e−01
1e−03
1e+09
1e+06
1e+03
1e+01
1e+04

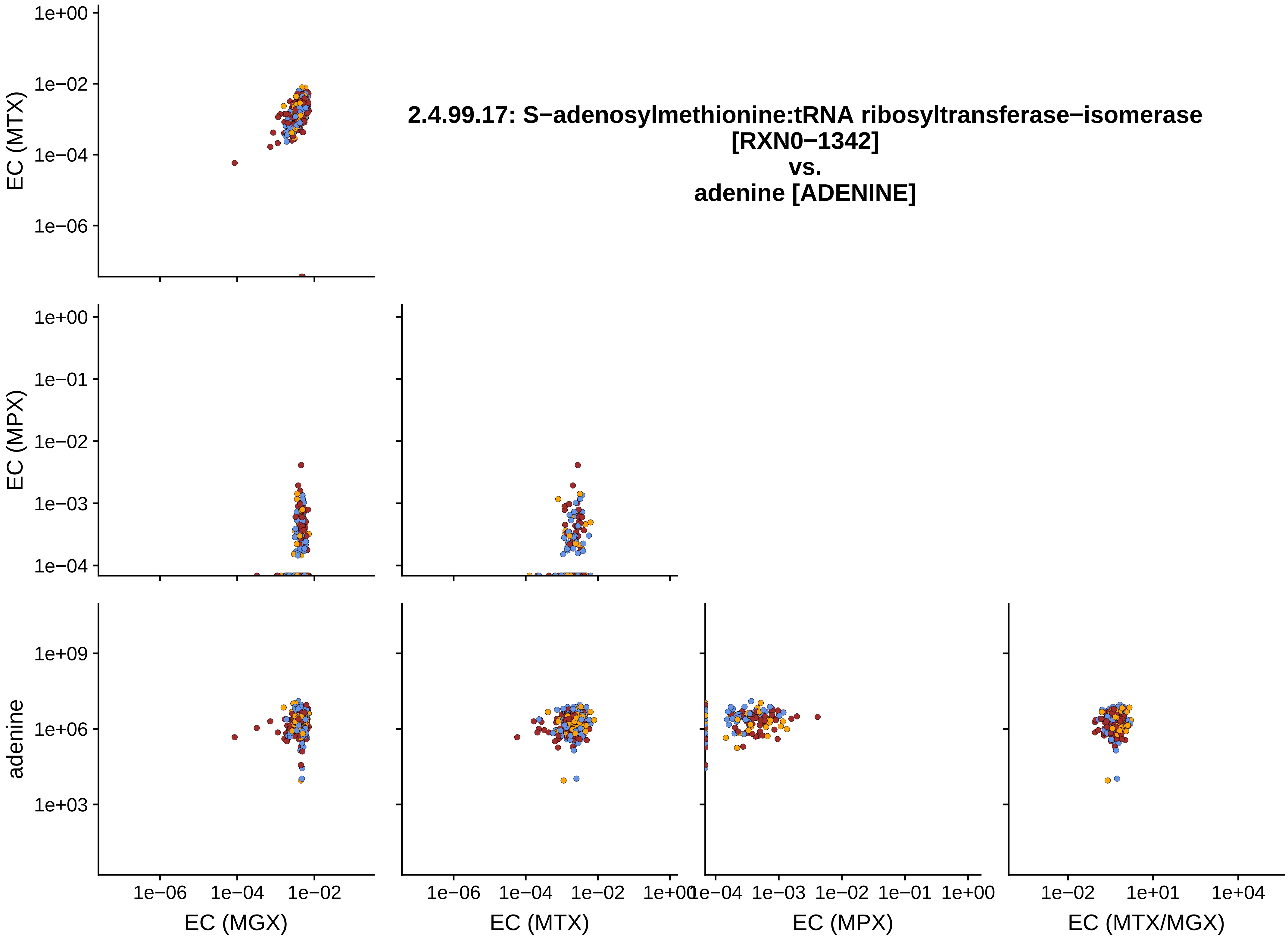
2.4.99.17: S−adenosylmethionine:tRNA ribosyltransferase−isomerase
[RXN0−1342]
vs.
adenine [ADENINE]
EC (MTX)
EC (MPX)
adenine
EC (MGX)
EC (MTX)
EC (MPX)
EC (MTX/MGX)
1e+00
1e−02
1e−04
1e−06
1e+00
1e−01
1e−02
1e−03
1e−04
1e+09
1e+06
1e+03
1e−06
1e−04
1e−02
1e+00
1e−01
1e+01
1e+04

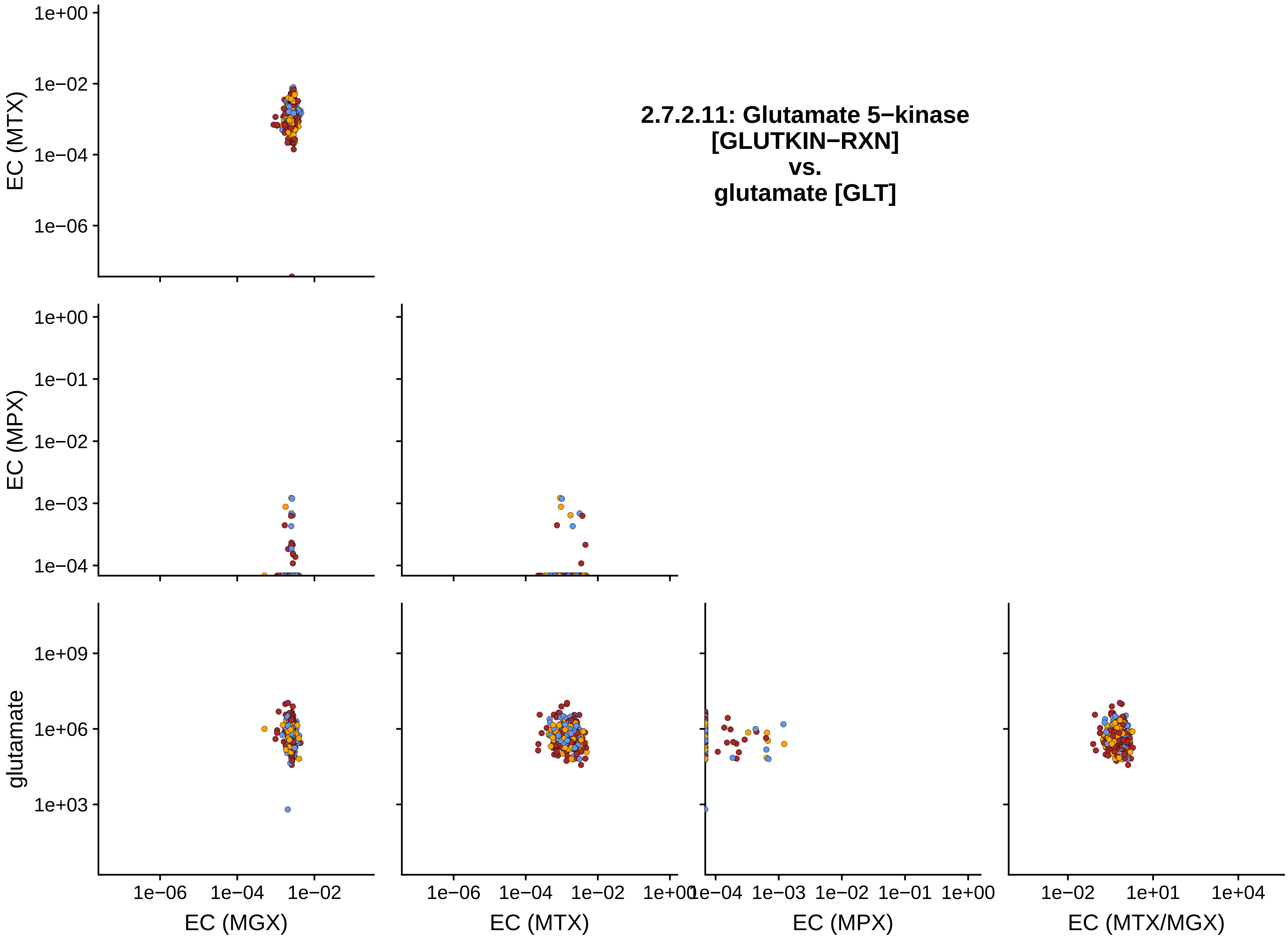
2.7.2.11: Glutamate 5−kinase
[GLUTKIN−RXN]
vs.
glutamate [GLT]
EC (MTX)
EC (MPX)
glutamate
EC (MGX)
EC (MTX)
EC (MPX)
EC (MTX/MGX)
1e+00
1e−02
1e−04
1e−06
1e−01
1e−03
1e+09
1e+06
1e+03
1e+01
1e+04

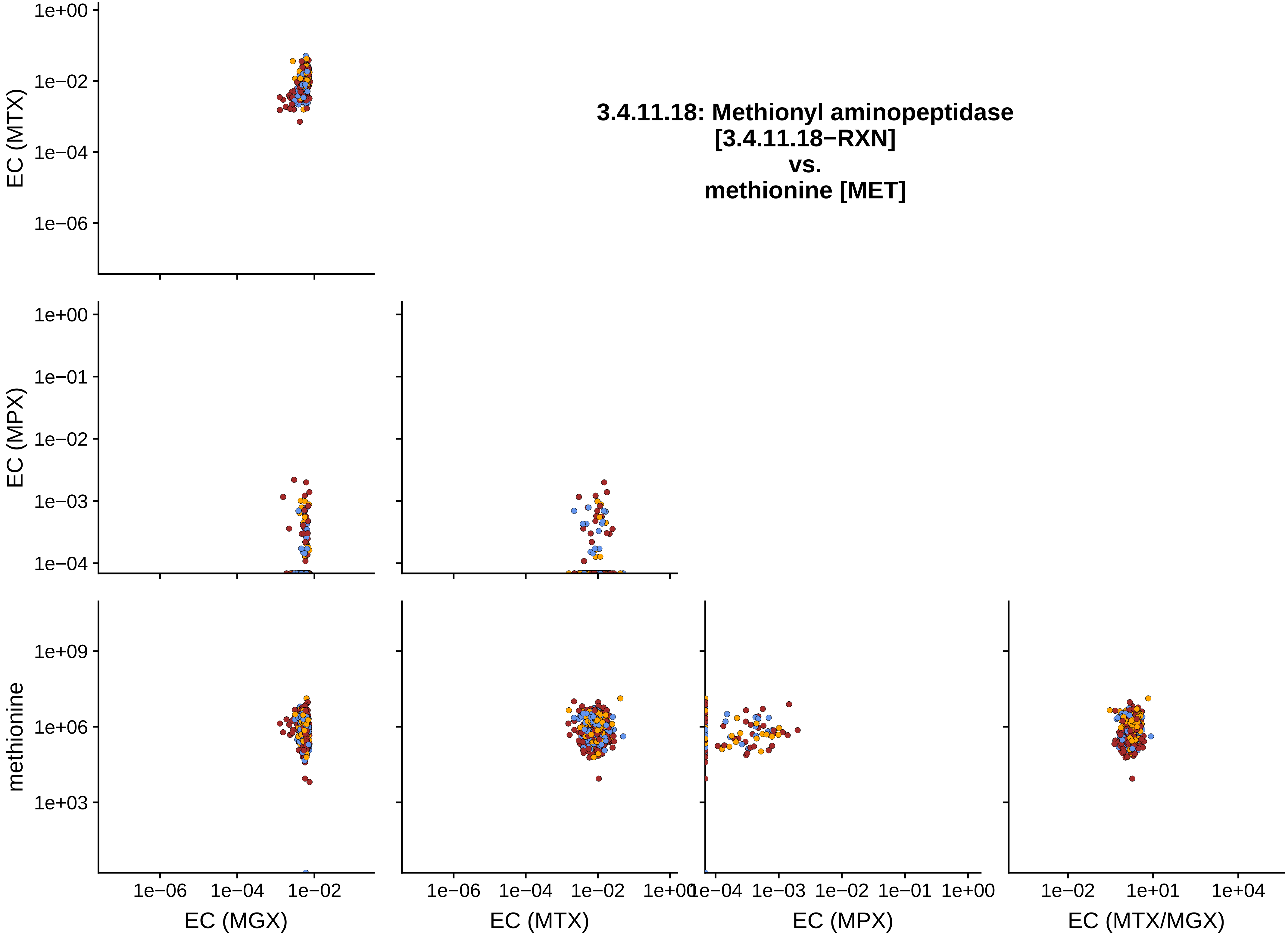
3.4.11.18: Methionyl aminopeptidase
[3.4.11.18−RXN]
vs.
methionine [MET]
EC (MTX)
EC (MPX)
methionine
EC (MGX)
EC (MTX)
EC (MPX)
EC (MTX/MGX)
1e+00
1e−02
1e−04
1e−06
1e−01
1e−03
1e+09
1e+06
1e+03
1e+01
1e+04

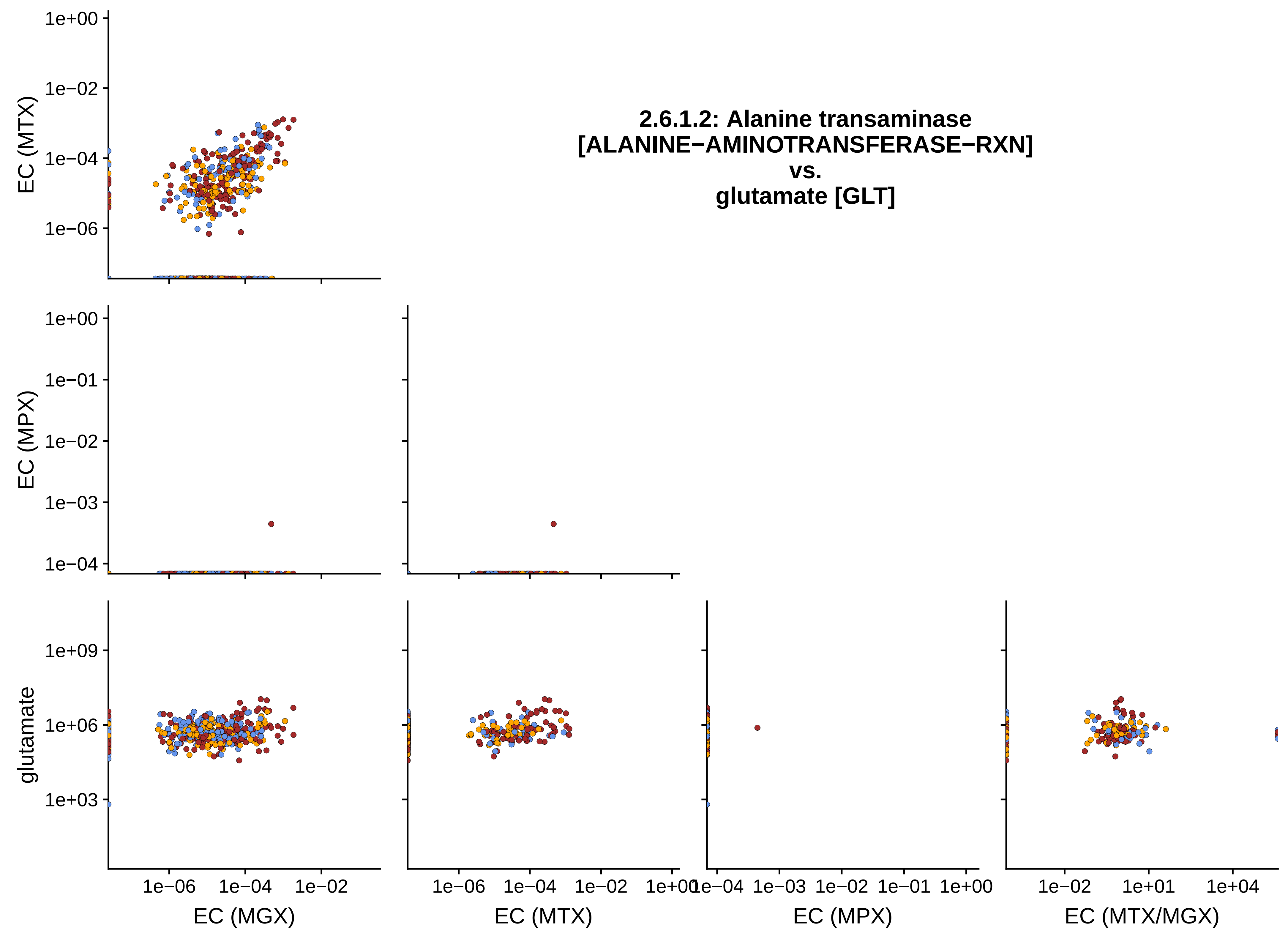

2.6.1.2: Alanine transaminase
[ALANINE−AMINOTRANSFERASE−RXN]
vs.
glutamate [GLT]
EC (MTX)
EC (MPX)
glutamate
EC (MGX)
EC (MTX)
EC (MPX)
EC (MTX/MGX)
1e+00
1e−02
1e−04
1e−06
1e+00
1e−01
1e−02
1e−03
1e−04
1e+09
1e+06
1e+03
1e−06
1e−04
1e−02
1e−06
1e−04
1e−02
1e+00
1e−04
1e−03
1e−02
1e−01
1e+00
1e−02
1e+01
1e+04

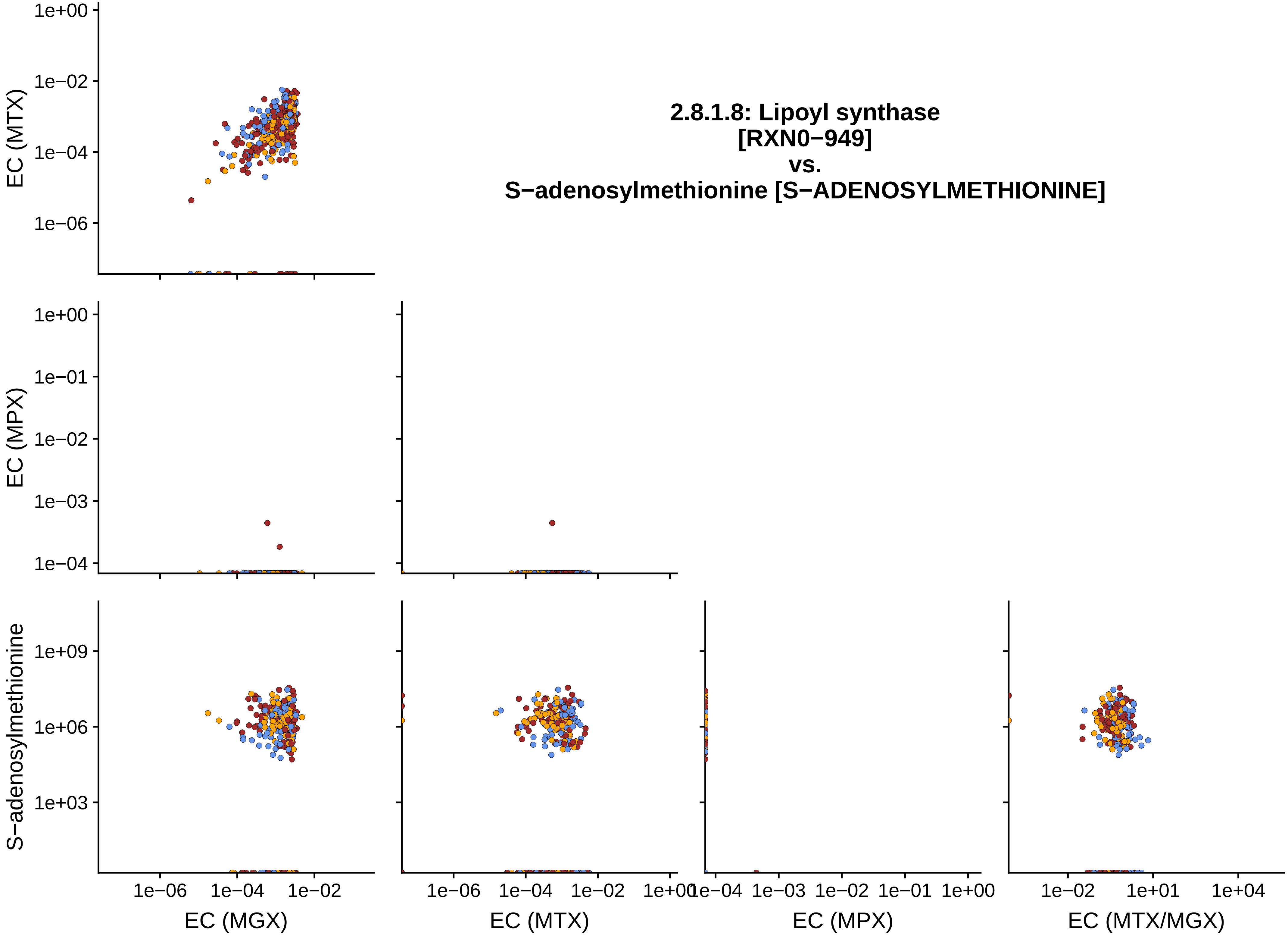
2.8.1.8: Lipoyl synthase
[RXN0−949]
vs.
S−adenosylmethionine [S−ADENOSYLMETHIONINE]
EC (MTX)
EC (MPX)
S−adenosylmethionine
EC (MGX)
EC (MTX)
EC (MPX)
EC (MTX/MGX)
1e+00
1e−02
1e−04
1e−06
1e−01
1e−03
1e+09
1e+06
1e+03
1e+01
1e+04

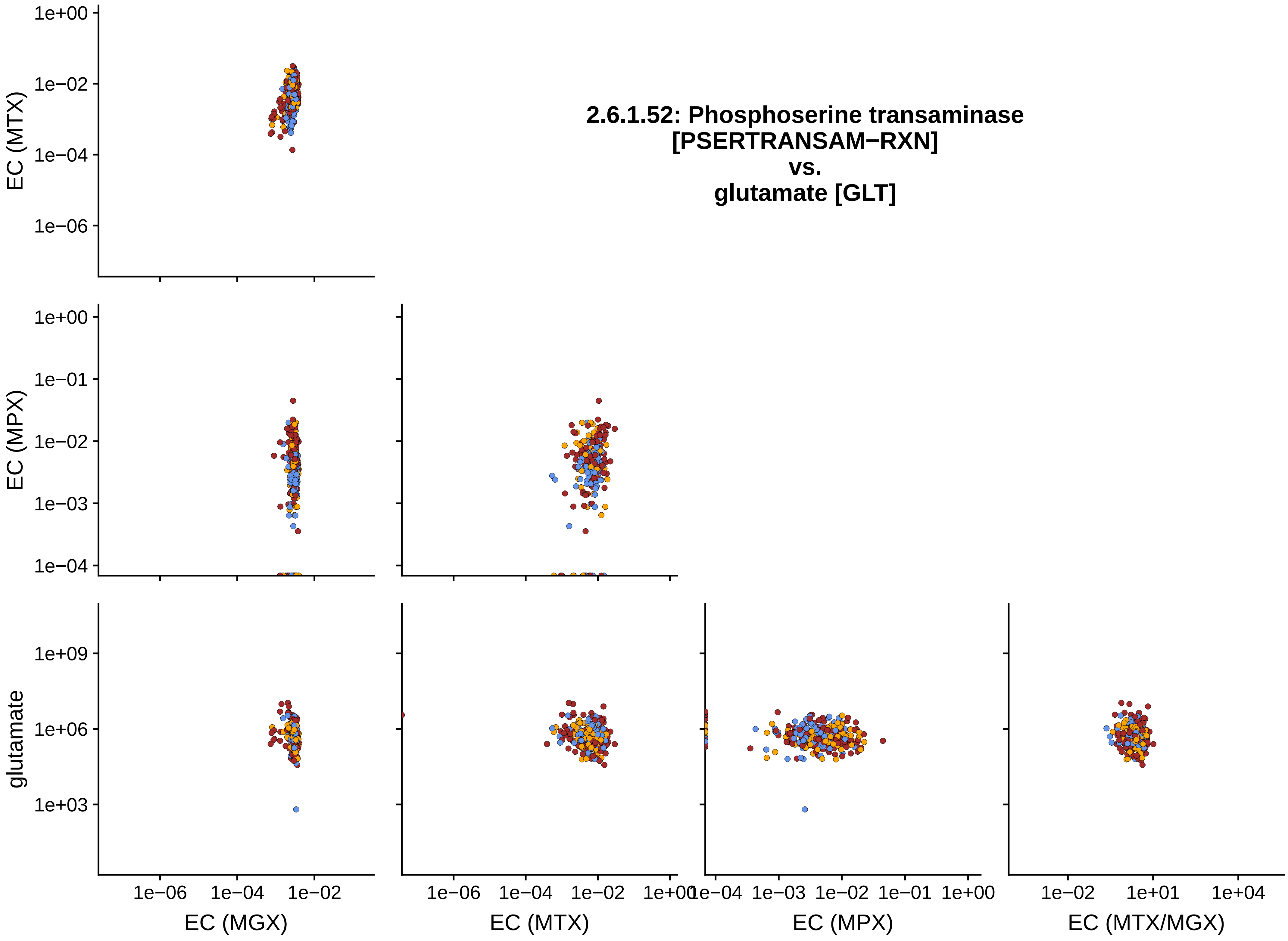
2.6.1.52: Phosphoserine transaminase
[PSERTRANSAM−RXN]
vs.
glutamate [GLT]
EC (MTX)
EC (MPX)
glutamate
EC (MGX)
EC (MTX)
EC (MPX)
EC (MTX/MGX)
1e+00
1e−02
1e−04
1e−06
1e−01
1e−03
1e+09
1e+06
1e+03
1e+04
1e+01

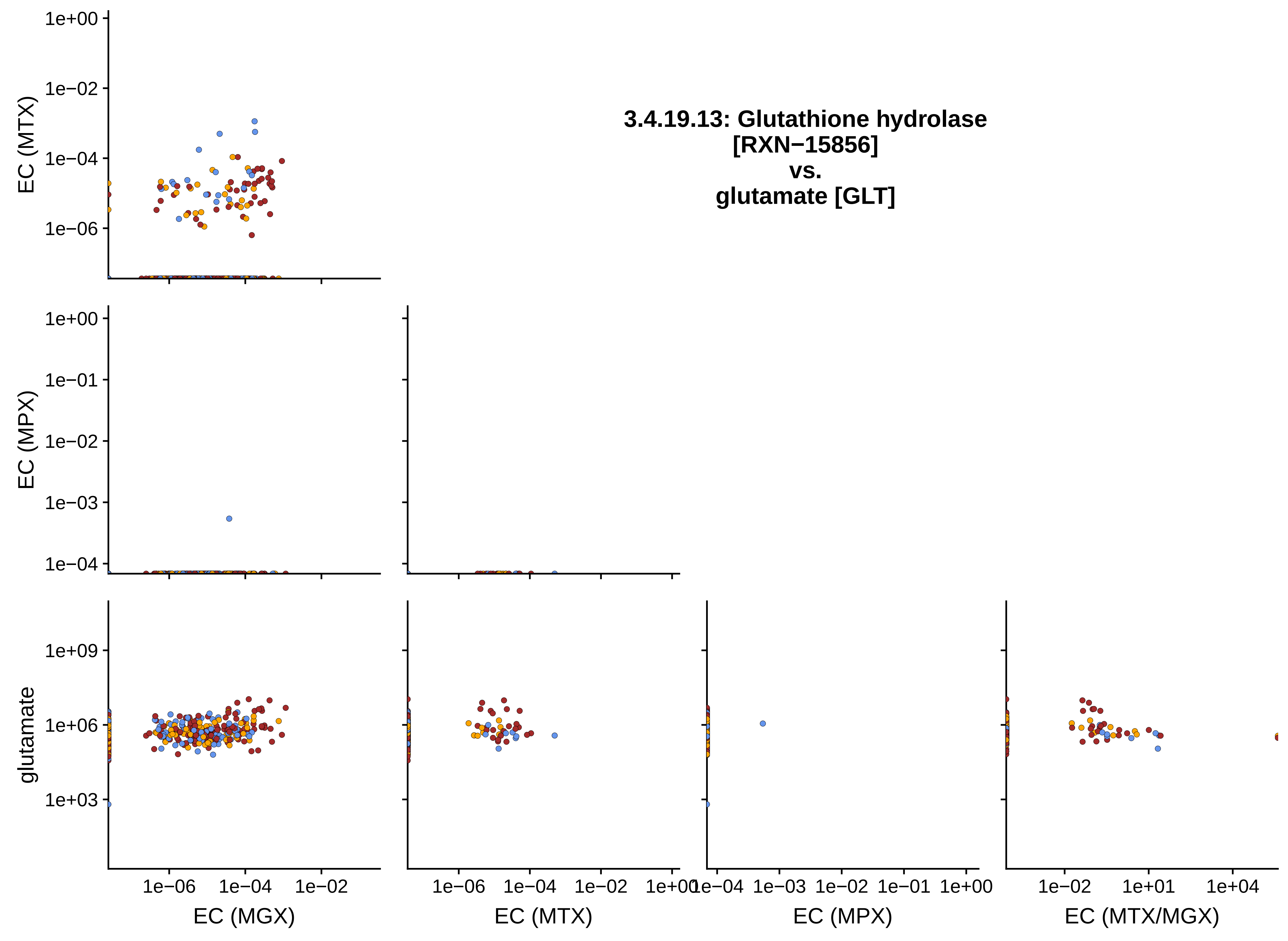

3.4.19.13: Glutathione hydrolase
[RXN−15856]
vs.
glutamate [GLT]
EC (MTX)
EC (MPX)
glutamate
EC (MGX)
EC (MTX)
EC (MPX)
EC (MTX/MGX)
1e+00
1e−02
1e−04
1e−06
1e−01
1e−03
1e+09
1e+06
1e+03
1e+01
1e+04

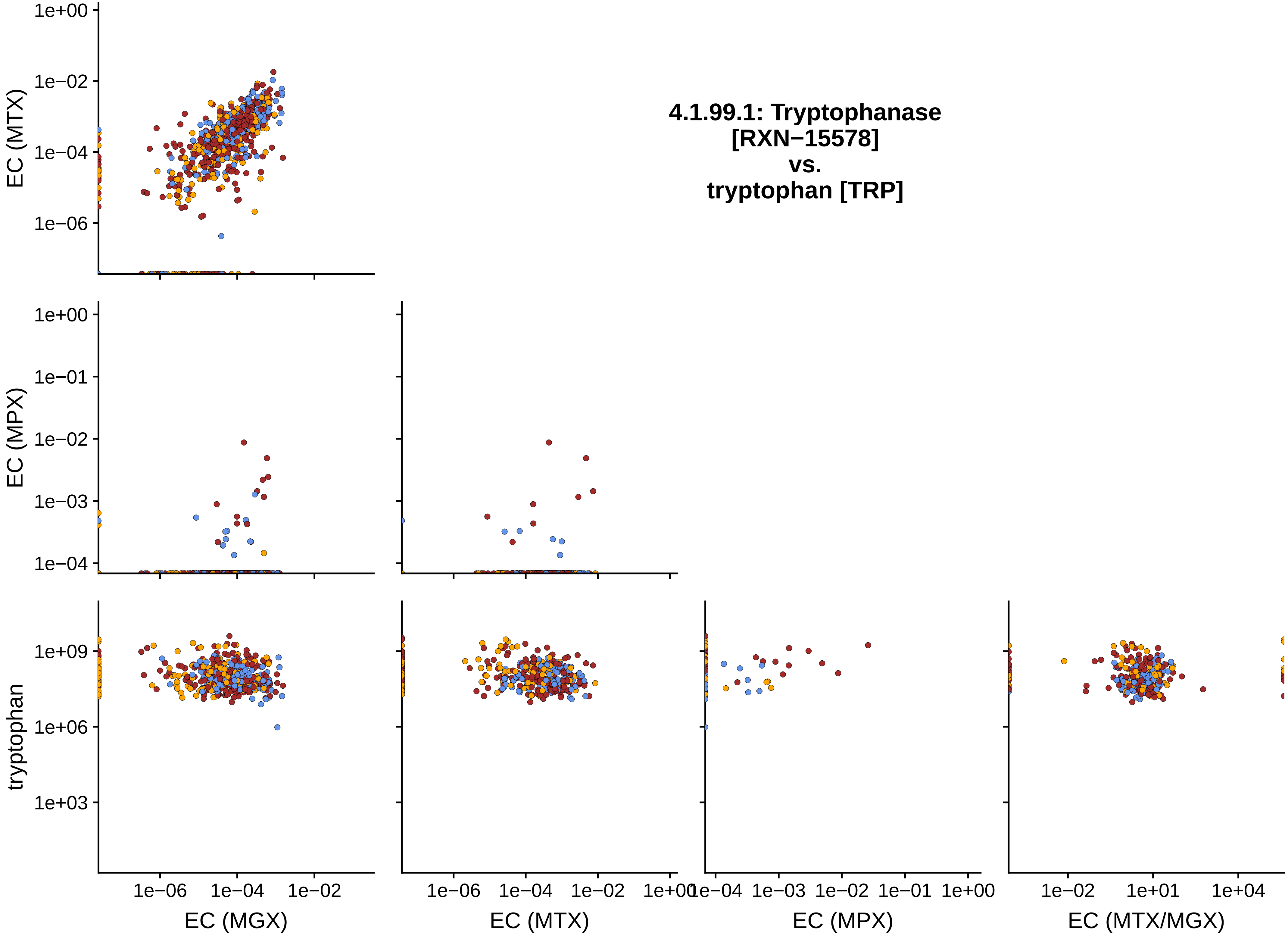
4.1.99.1: Tryptophanase
[RXN−15578]
vs.
tryptophan [TRP]
EC (MTX)
EC (MPX)
tryptophan
EC (MGX)
EC (MTX)
EC (MPX)
EC (MTX/MGX)
1e+00
1e−02
1e−04
1e−06
1e−01
1e−03
1e+09
1e+06
1e+03
1e+04
1e+01

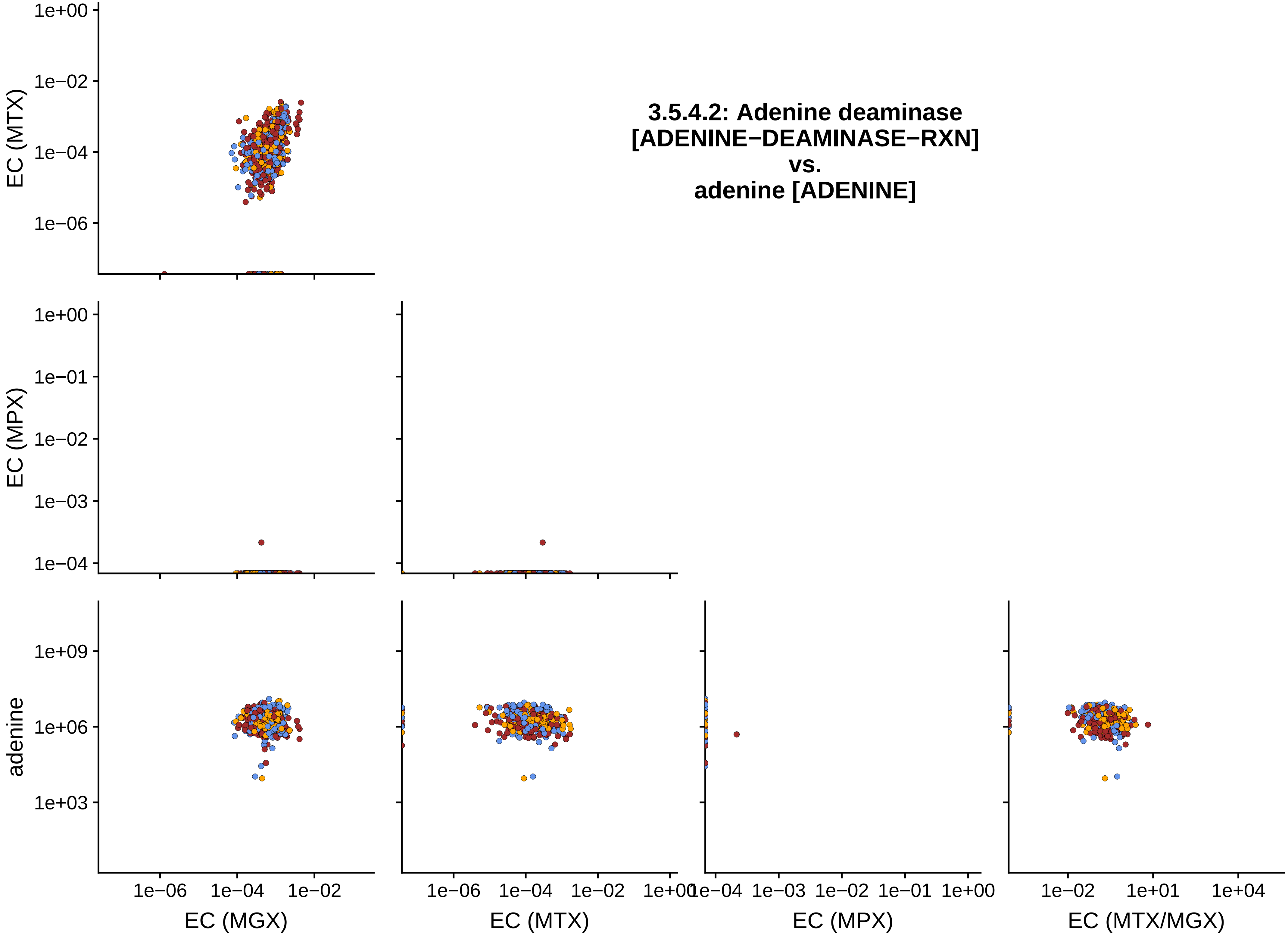

3.5.4.2: Adenine deaminase
[ADENINE−DEAMINASE−RXN]
vs.
adenine [ADENINE]
EC (MTX)
EC (MPX)
adenine
EC (MGX)
EC (MTX)
EC (MPX)
EC (MTX/MGX)
1e+00
1e−02
1e−04
1e−06
1e−01
1e−03
1e+09
1e+06
1e+03
1e+01
1e+04

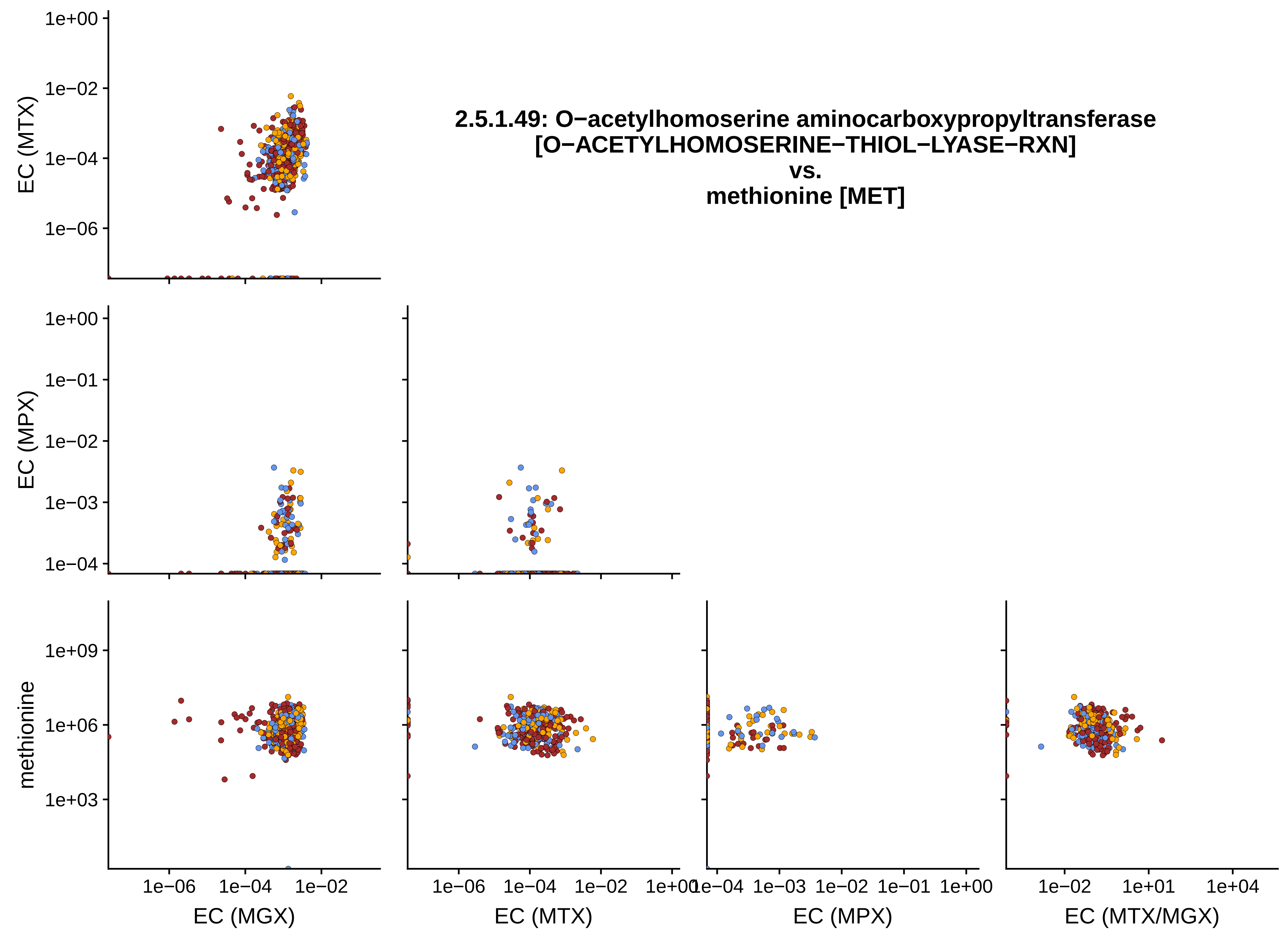
2.5.1.49: O−acetylhomoserine aminocarboxypropyltransferase
[O−ACETYLHOMOSERINE−THIOL−LYASE−RXN]
vs.
methionine [MET]
EC (MTX)
EC (MPX)
methionine
EC (MGX)
EC (MTX)
EC (MPX)
EC (MTX/MGX)
1e+00
1e−02
1e−04
1e−06
1e−01
1e−03
1e+09
1e+06
1e+03
1e+01
1e+04

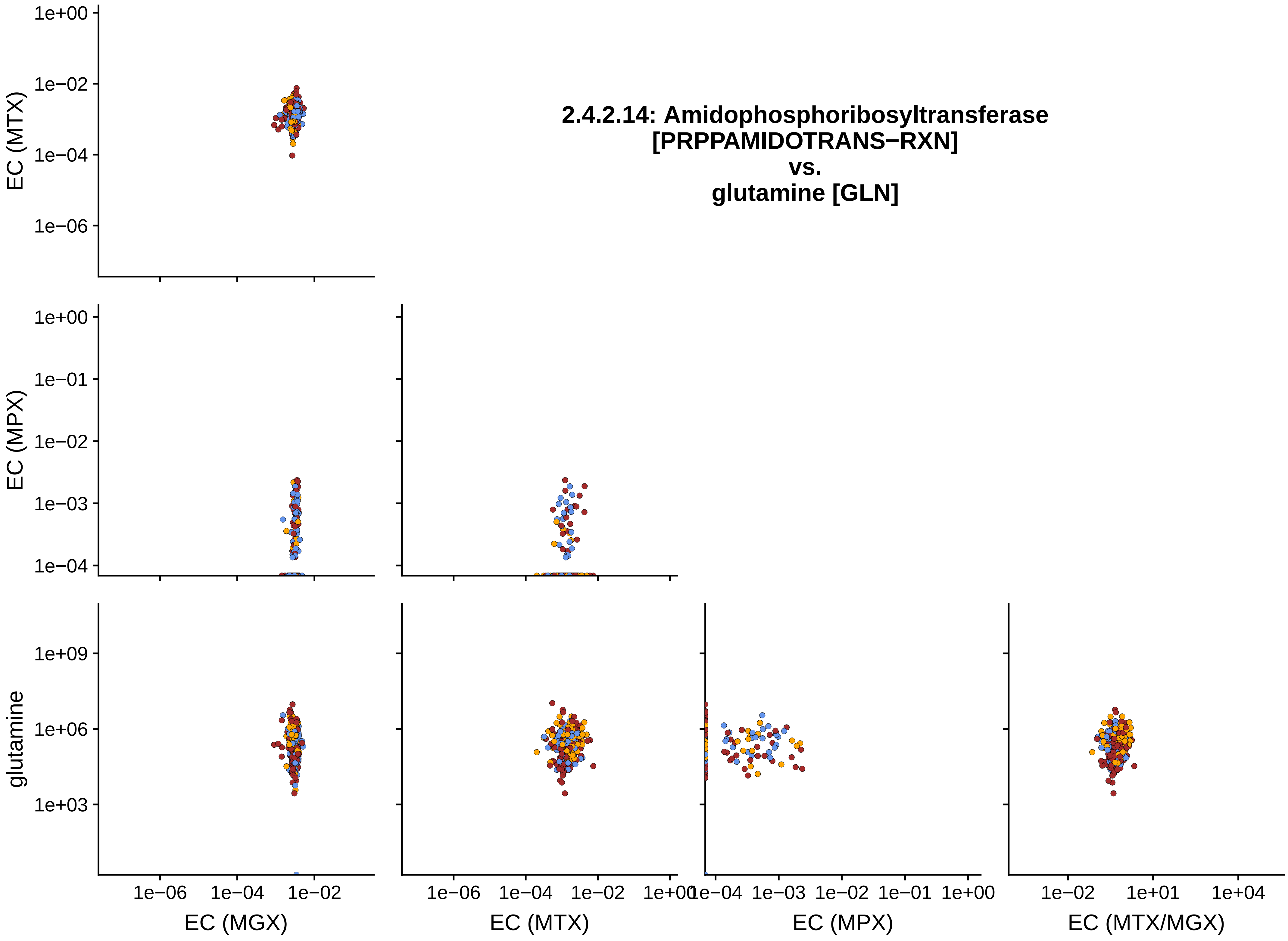
2.4.2.14: Amidophosphoribosyltransferase
[PRPPAMIDOTRANS−RXN]
vs.
glutamine [GLN]
EC (MTX)
EC (MPX)
glutamine
EC (MGX)
EC (MTX)
EC (MPX)
EC (MTX/MGX)
1e+00
1e−02
1e−04
1e−06
1e−01
1e−03
1e+09
1e+06
1e+03
1e+01
1e+04

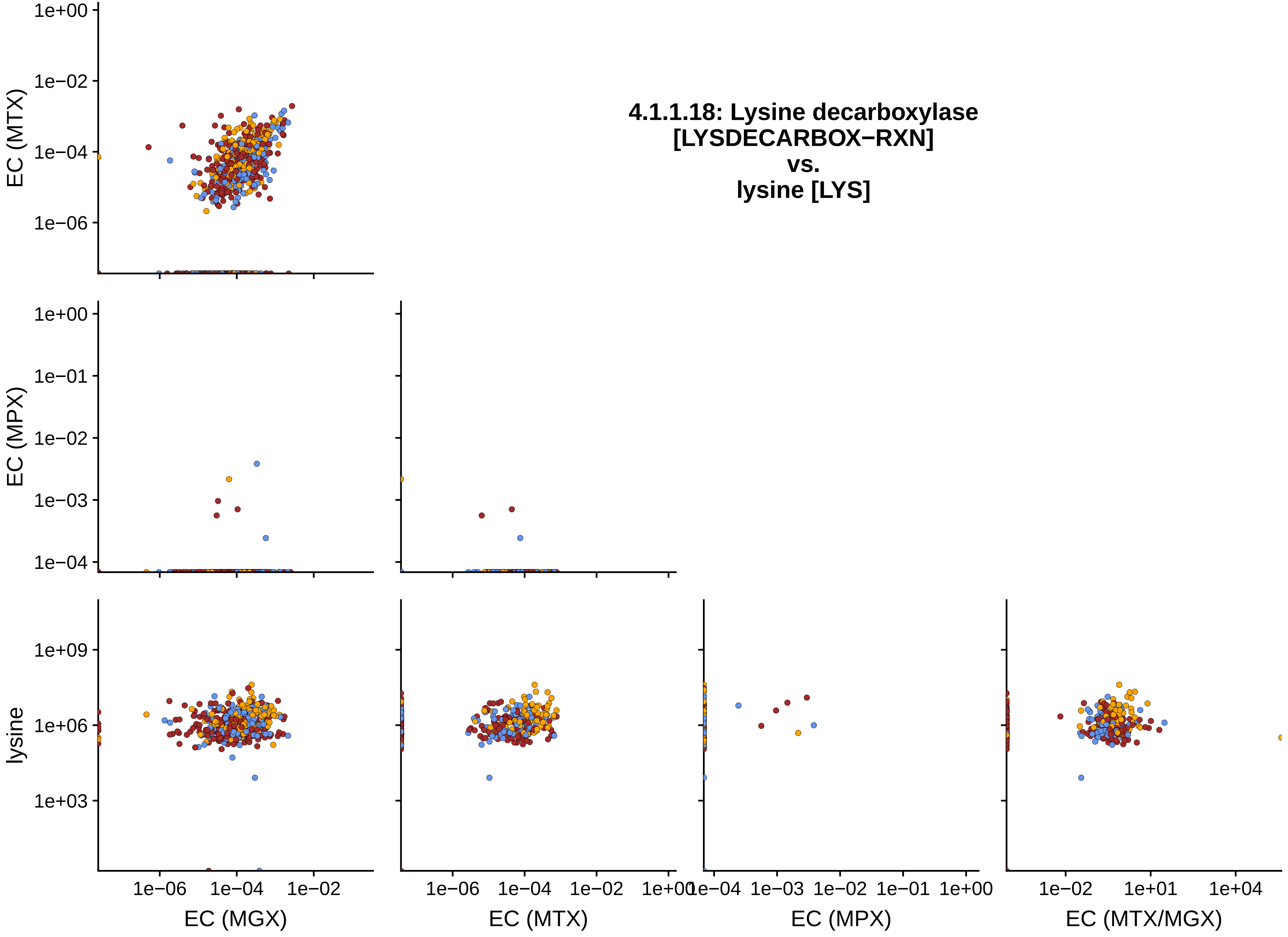

4.1.1.18: Lysine decarboxylase
[LYSDECARBOX−RXN]
vs.
lysine [LYS]
EC (MTX)
EC (MPX)
lysine
EC (MGX)
EC (MTX)
EC (MPX)
EC (MTX/MGX)
1e+00
1e−02
1e−04
1e−06
1e−01
1e−03
1e+09
1e+06
1e+03
1e+01
1e+04

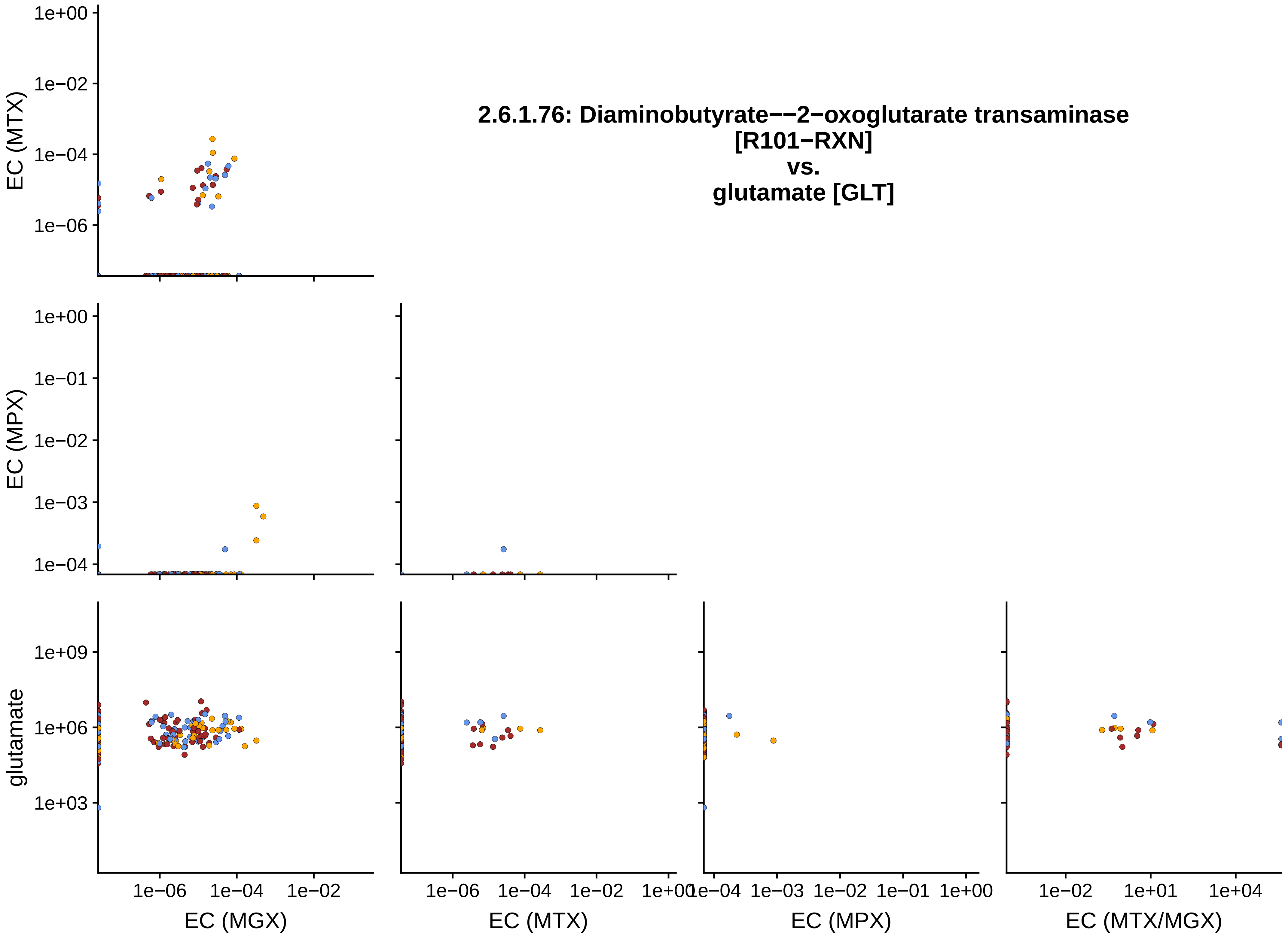

2.6.1.76: Diaminobutyrate−−2−oxoglutarate transaminase
[R101−RXN]
vs.
glutamate [GLT]
EC (MTX)
EC (MPX)
glutamate
EC (MGX)
EC (MTX)
EC (MPX)
EC (MTX/MGX)
1e+00
1e−02
1e−04
1e−06
1e−01
1e−03
1e+09
1e+06
1e+03
1e+01
1e+04

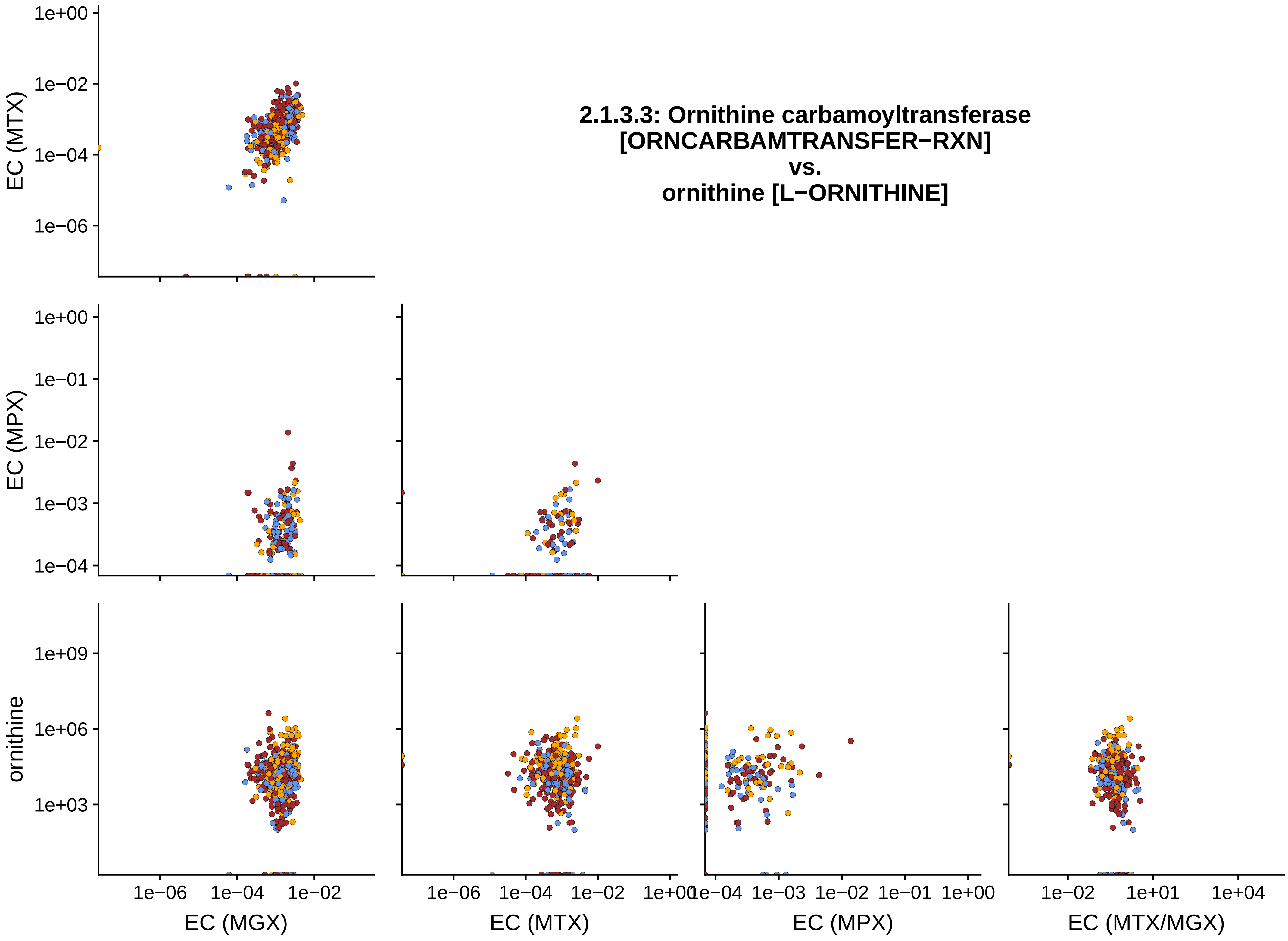

2.1.3.3: Ornithine carbamoyltransferase
[ORNCARBAMTRANSFER−RXN]
vs.
ornithine [L−ORNITHINE]
EC (MTX)
EC (MPX)
ornithine
EC (MGX)
EC (MTX)
EC (MPX)
EC (MTX/MGX)
1e+00
1e−02
1e−04
1e−06
1e−01
1e−03
1e+09
1e+06
1e+03
1e+01
1e+04

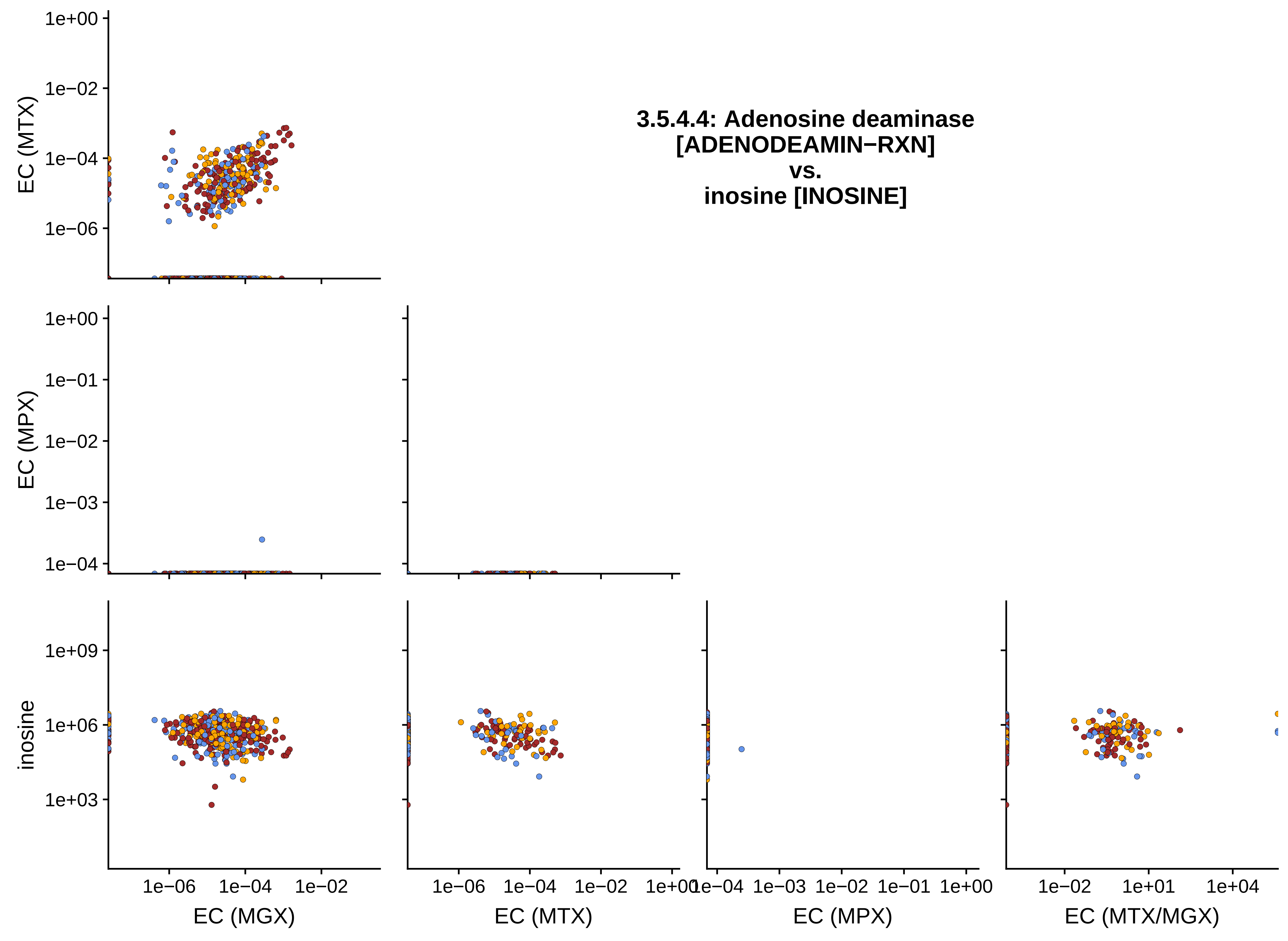

3.5.4.4: Adenosine deaminase
[ADENODEAMIN−RXN]
vs.
inosine [INOSINE]
EC (MTX)
EC (MPX)
inosine
EC (MGX)
EC (MTX)
EC (MPX)
EC (MTX/MGX)
1e+00
1e−02
1e−04
1e−06
1e−01
1e−03
1e+09
1e+06
1e+03
1e+01
1e+04

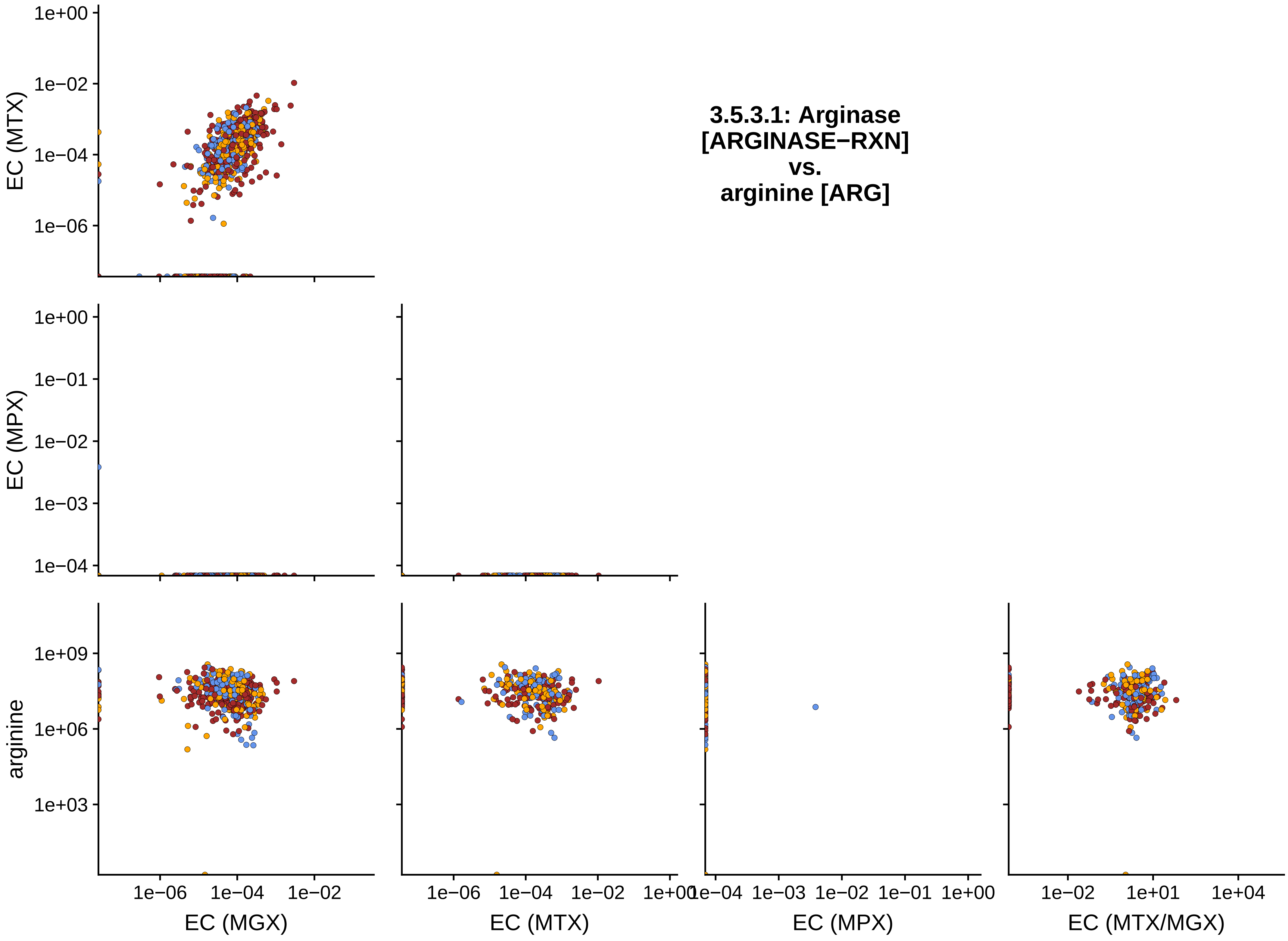

3.5.3.1: Arginase
[ARGINASE−RXN]
vs.
arginine [ARG]
EC (MTX)
EC (MPX)
arginine
EC (MGX)
EC (MTX)
EC (MPX)
EC (MTX/MGX)
1e+00
1e−02
1e−04
1e−06
1e−01
1e−03
1e+09
1e+06
1e+03
1e+01
1e+04

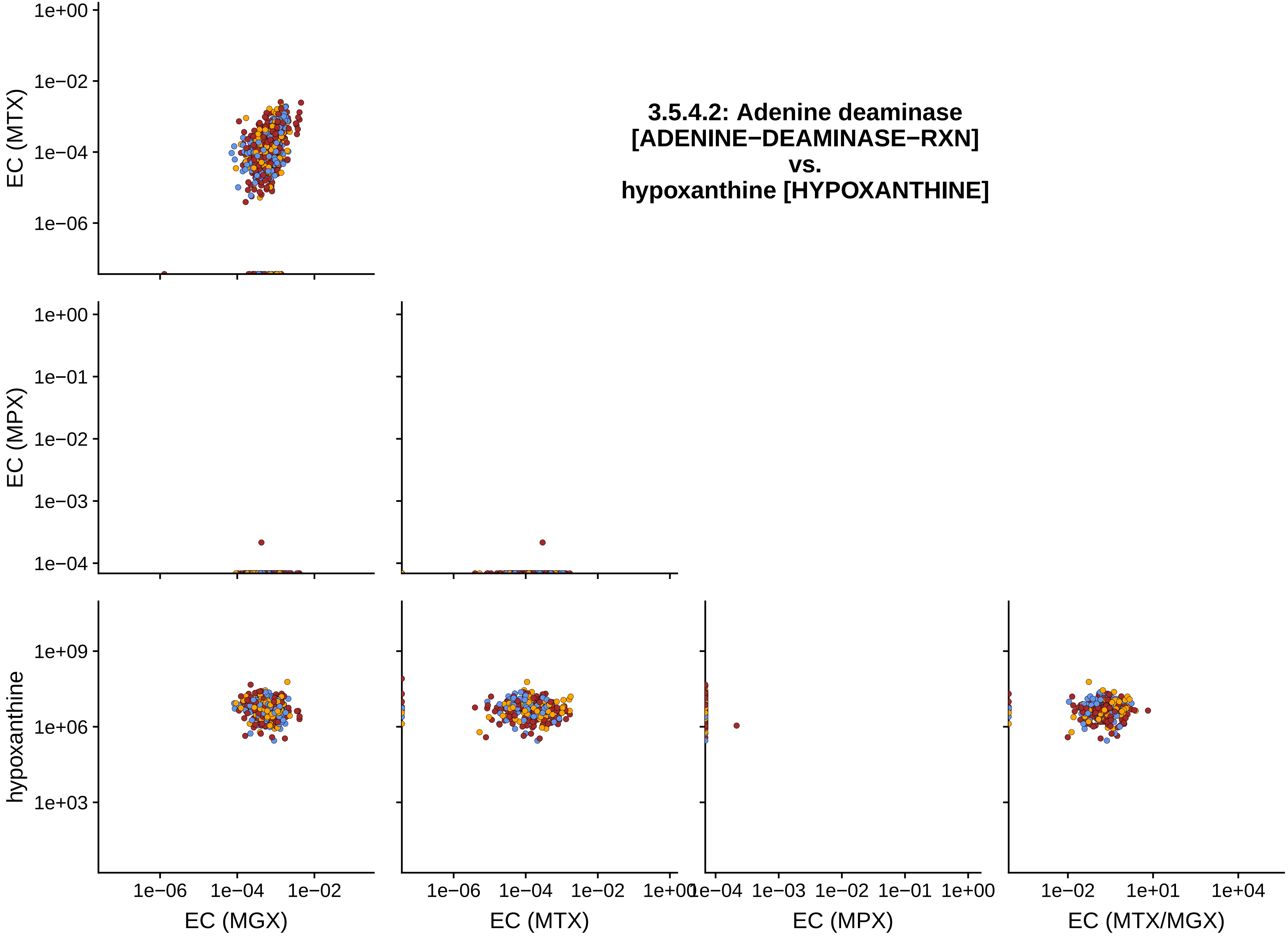
3.5.4.2: Adenine deaminase
[ADENINE−DEAMINASE−RXN]
vs.
hypoxanthine [HYPOXANTHINE]
EC (MTX)
EC (MPX)
hypoxanthine
EC (MGX)
EC (MTX)
EC (MPX)
EC (MTX/MGX)
1e+00
1e−02
1e−04
1e−06
1e−01
1e−03
1e+09
1e+06
1e+03
1e+01
1e+04

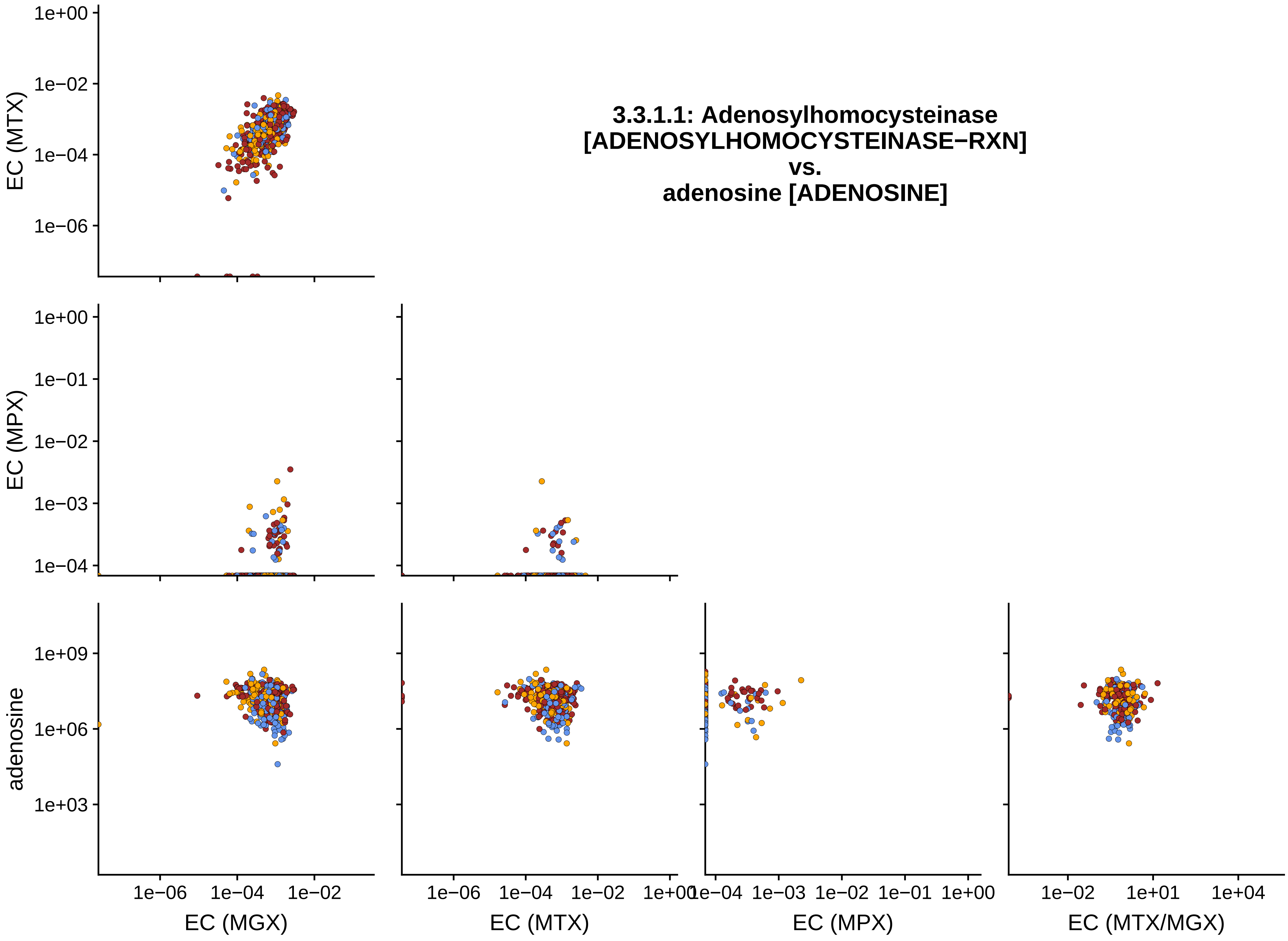
3.3.1.1: Adenosylhomocysteinase
[ADENOSYLHOMOCYSTEINASE−RXN]
vs.
adenosine [ADENOSINE]
EC (MTX)
EC (MPX)
adenosine
EC (MGX)
EC (MTX)
EC (MPX)
EC (MTX/MGX)
1e+00
1e−02
1e−04
1e−06
1e−01
1e−03
1e+09
1e+06
1e+03
1e+01
1e+04

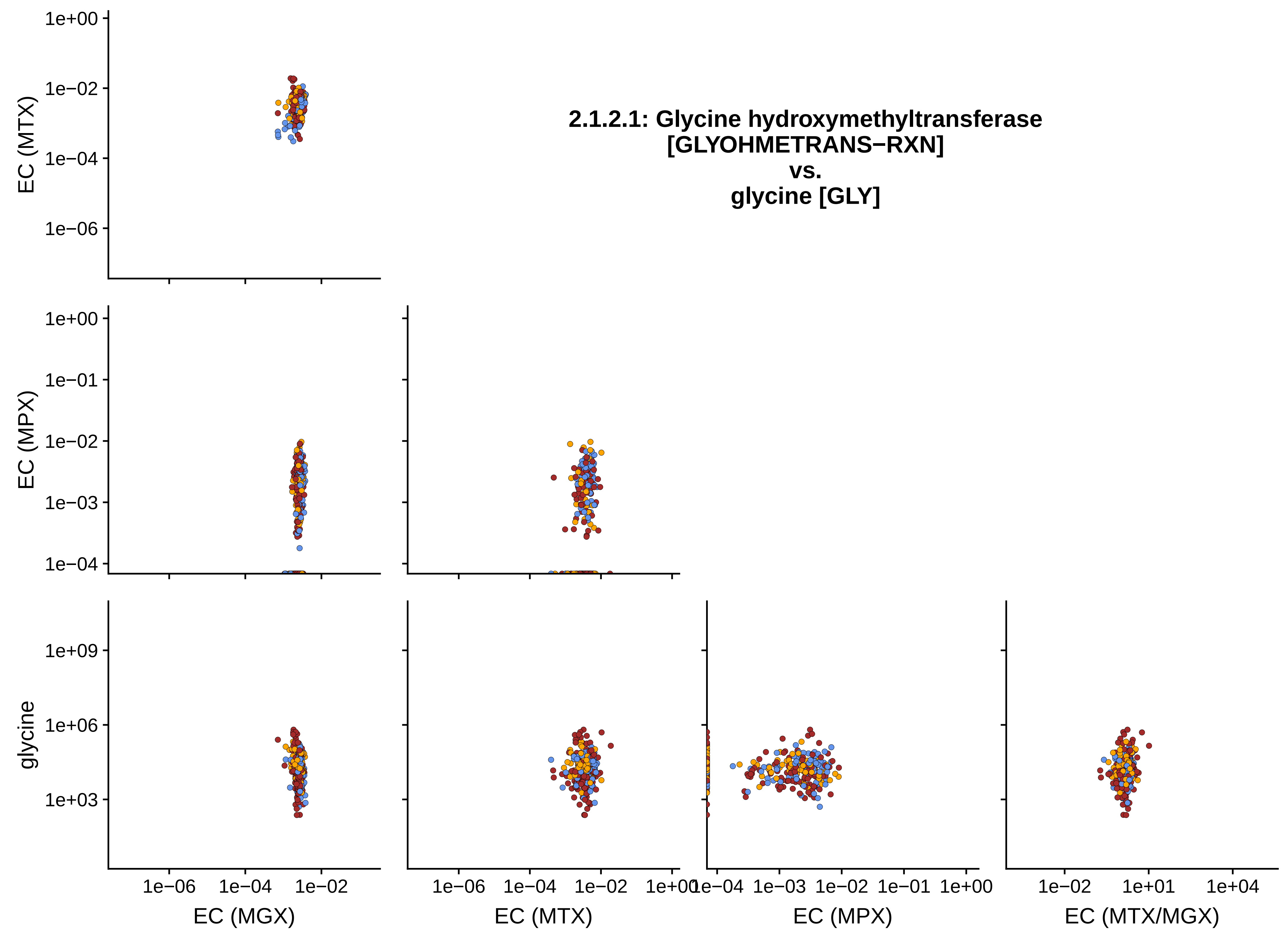

2.1.2.1: Glycine hydroxymethyltransferase
[GLYOHMETRANS−RXN]
vs.
glycine [GLY]
EC (MTX)
EC (MPX)
glycine
EC (MGX)
EC (MTX)
EC (MPX)
EC (MTX/MGX)
1e+00
1e−02
1e−04
1e−06
1e−01
1e−03
1e+09
1e+06
1e+03
1e+01
1e+04

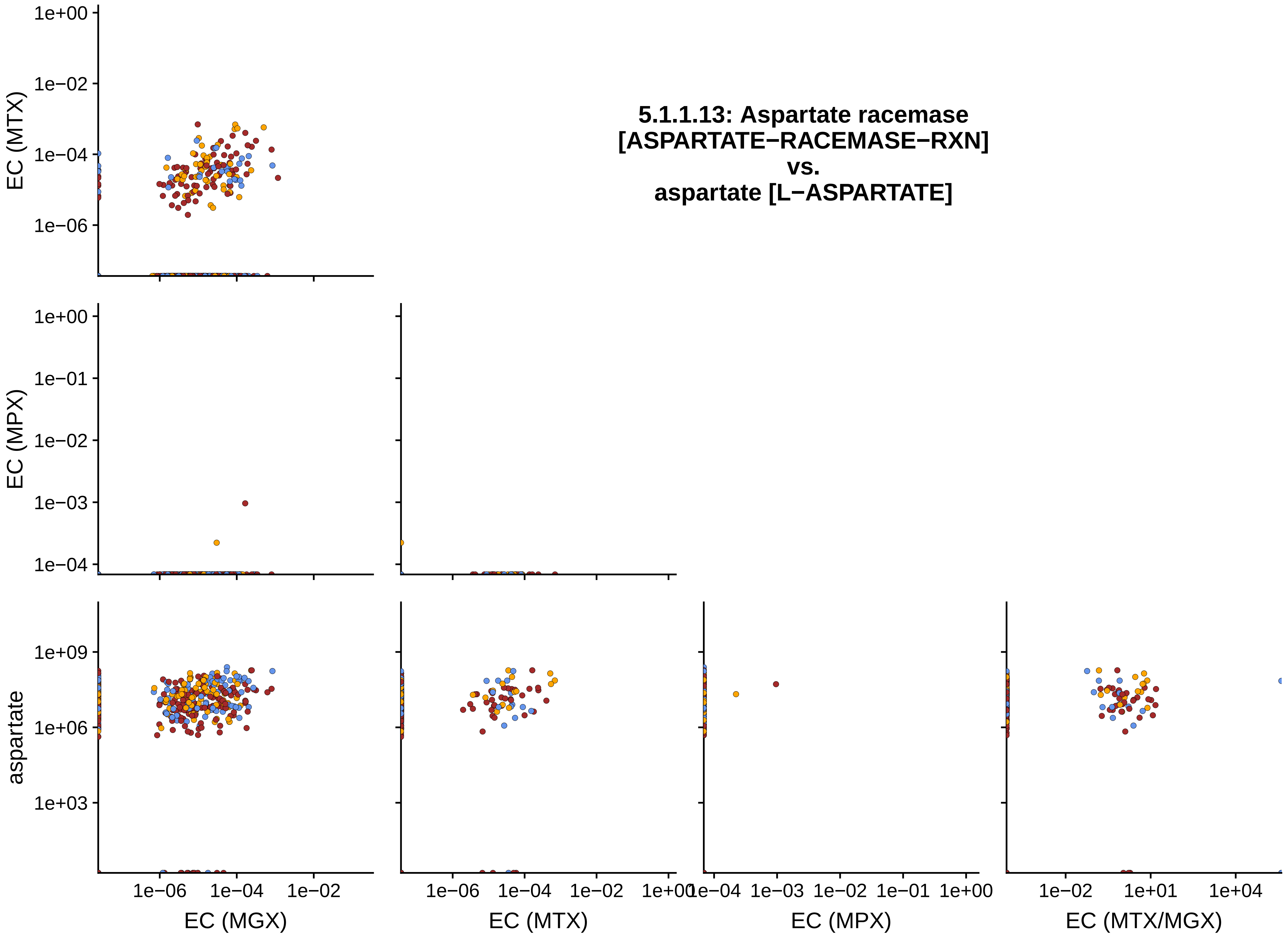

5.1.1.13: Aspartate racemase
[ASPARTATE−RACEMASE−RXN]
vs.
aspartate [L−ASPARTATE]
EC (MTX)
EC (MPX)
aspartate
EC (MGX)
EC (MTX)
EC (MPX)
EC (MTX/MGX)
1e+00
1e−02
1e−04
1e−06
1e+00
1e−01
1e−02
1e−03
1e−04
1e+09
1e+06
1e+03
1e−06
1e−04
1e−02
1e−06
1e−04
1e−02
1e+00
1e−04
1e−03
1e−02
1e−01
1e+00
1e−02
1e+01
1e+04

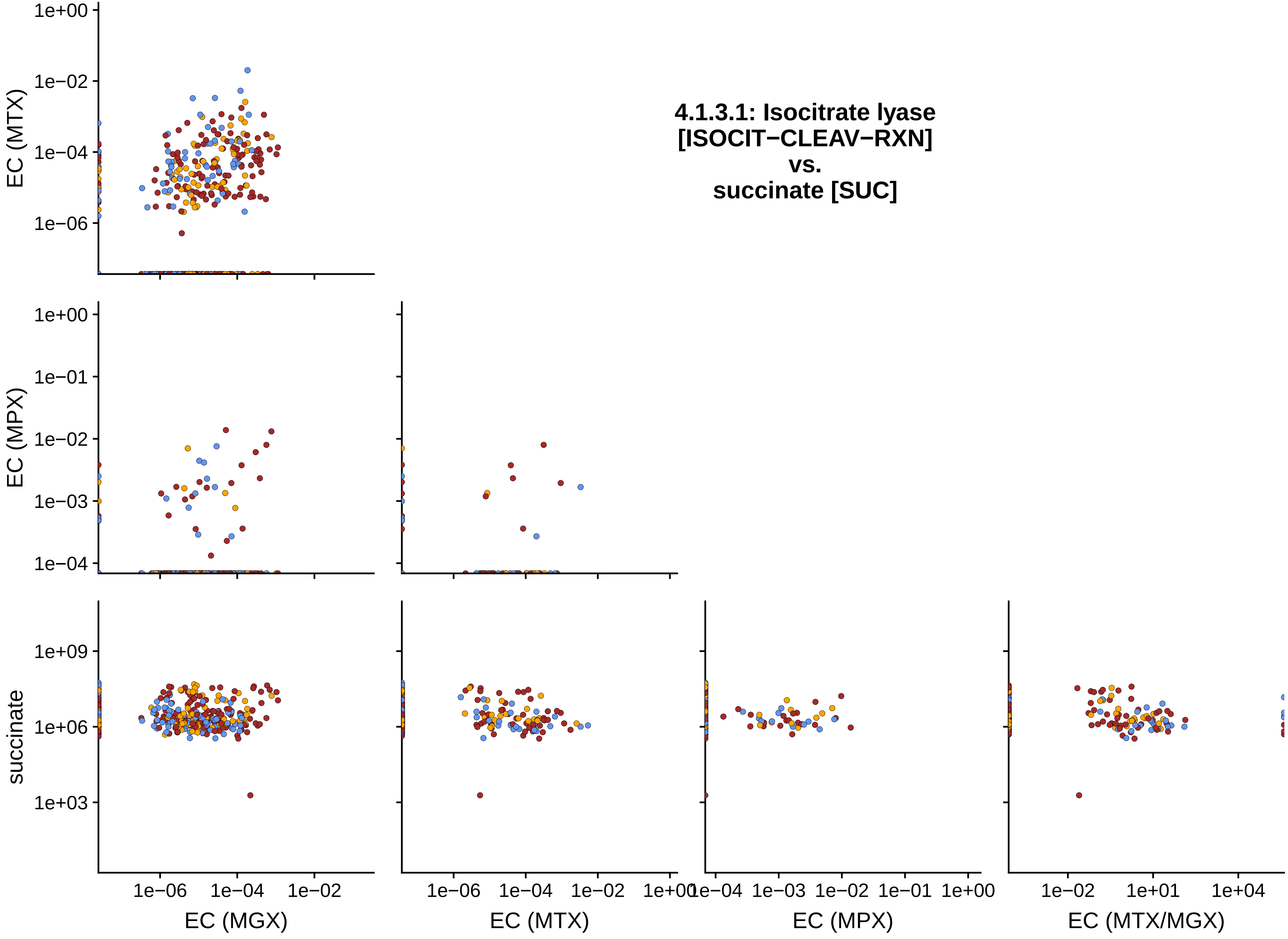
4.1.3.1: Isocitrate lyase
[ISOCIT−CLEAV−RXN]
vs.
succinate [SUC]
EC (MTX)
EC (MPX)
succinate
EC (MGX)
EC (MTX)
EC (MPX)
EC (MTX/MGX)
1e+00
1e−02
1e−04
1e−06
1e−01
1e−03
1e+09
1e+06
1e+03
1e+04
1e+01

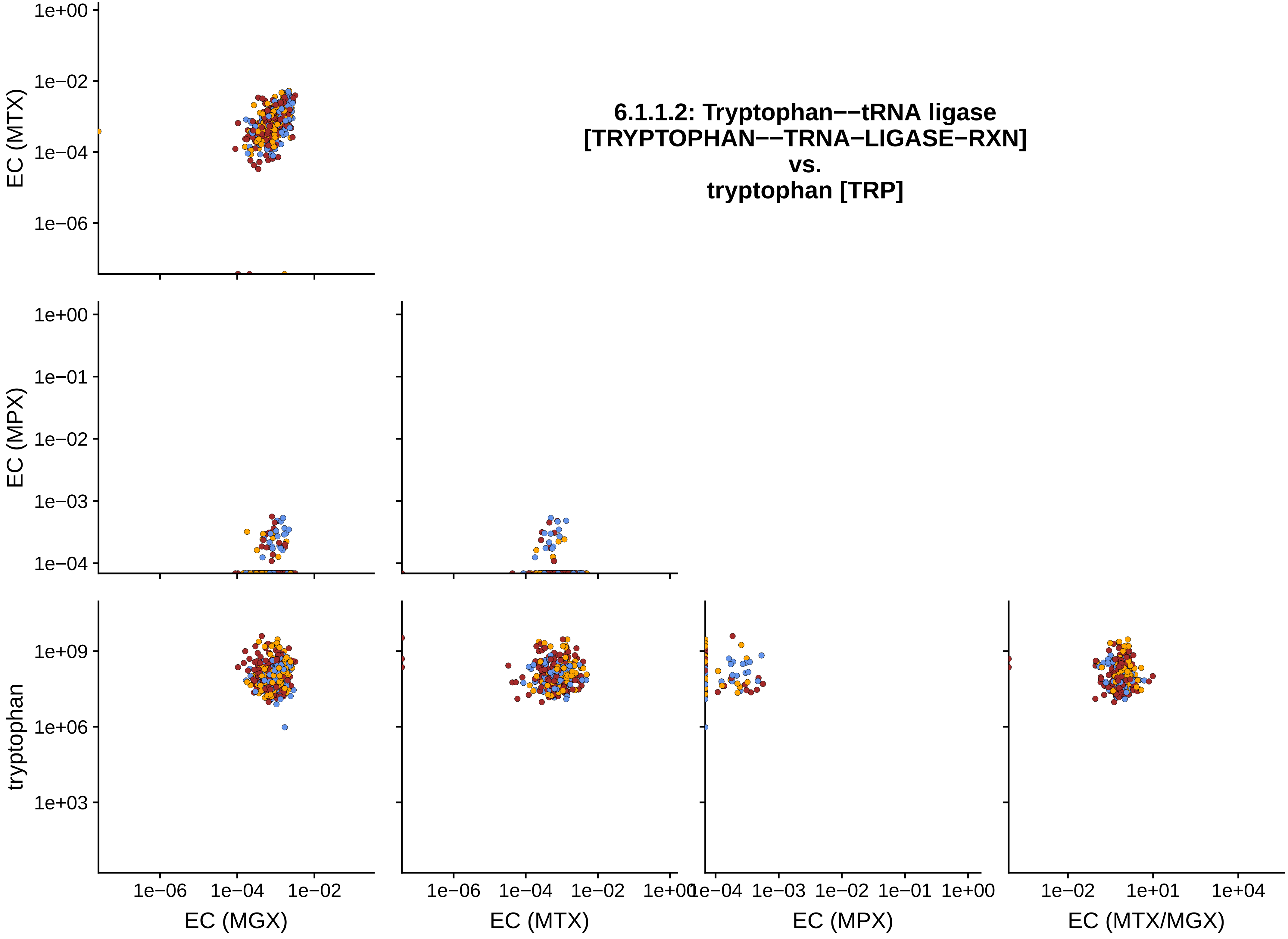
6.1.1.2: Tryptophan−−tRNA ligase
[TRYPTOPHAN−−TRNA−LIGASE−RXN]
vs.
tryptophan [TRP]
EC (MTX)
EC (MPX)
tryptophan
EC (MGX)
EC (MTX)
EC (MPX)
EC (MTX/MGX)
1e+00
1e−02
1e−04
1e−06
1e−01
1e−03
1e+09
1e+06
1e+03
1e+01
1e+04

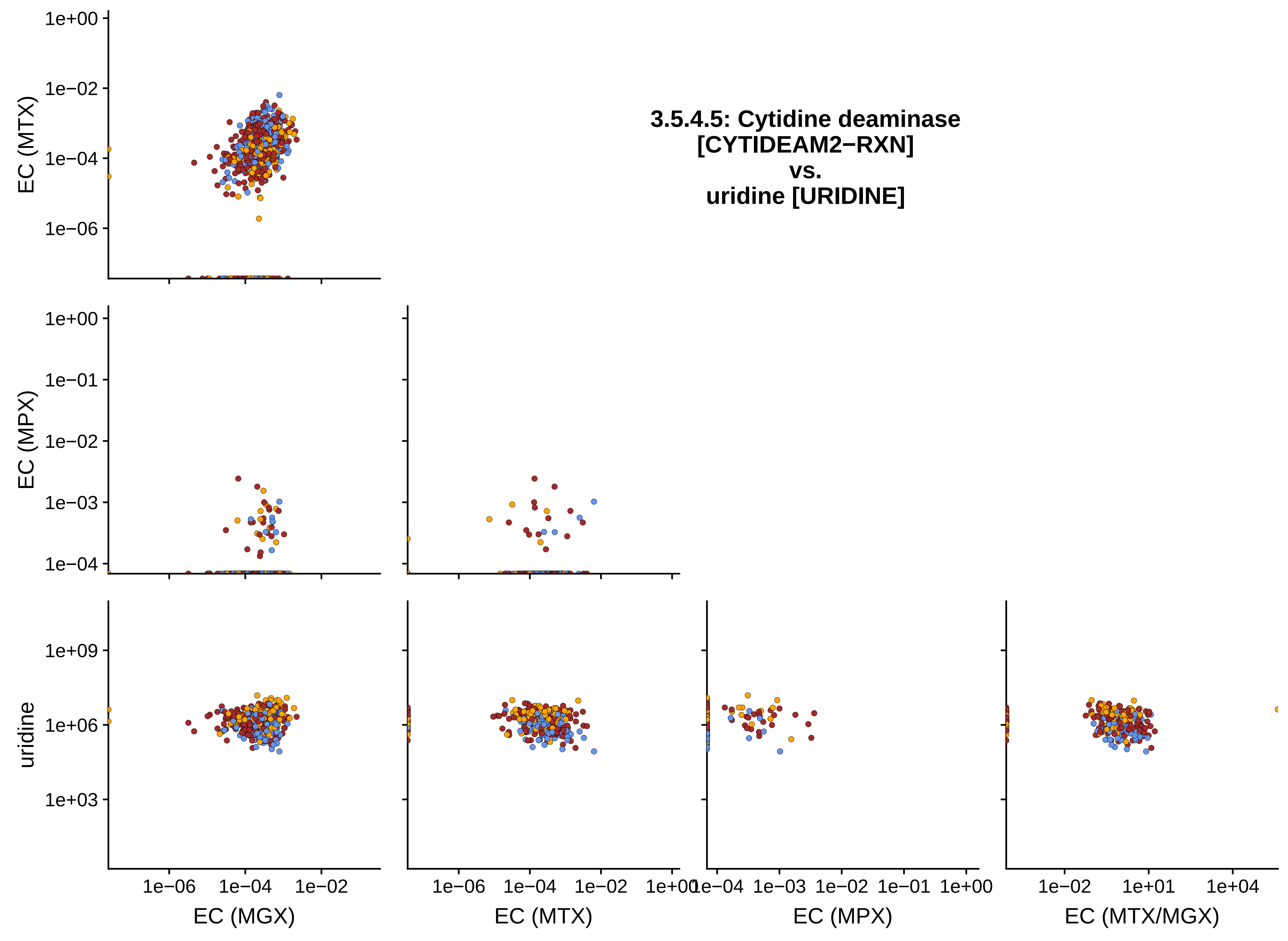

3.5.4.5: Cytidine deaminase
[CYTIDEAM2−RXN]
vs.
uridine [URIDINE]
EC (MTX)
EC (MPX)
uridine
EC (MGX)
EC (MTX)
EC (MPX)
EC (MTX/MGX)
1e+00
1e−02
1e−04
1e−06
1e−01
1e−02
1e−03
1e−04
1e+09
1e+06
1e+03
1e−06
1e−04
1e−02
1e+00
1e−04
1e−03
1e−02
1e−01
1e+00
1e−02
1e+01
1e+04

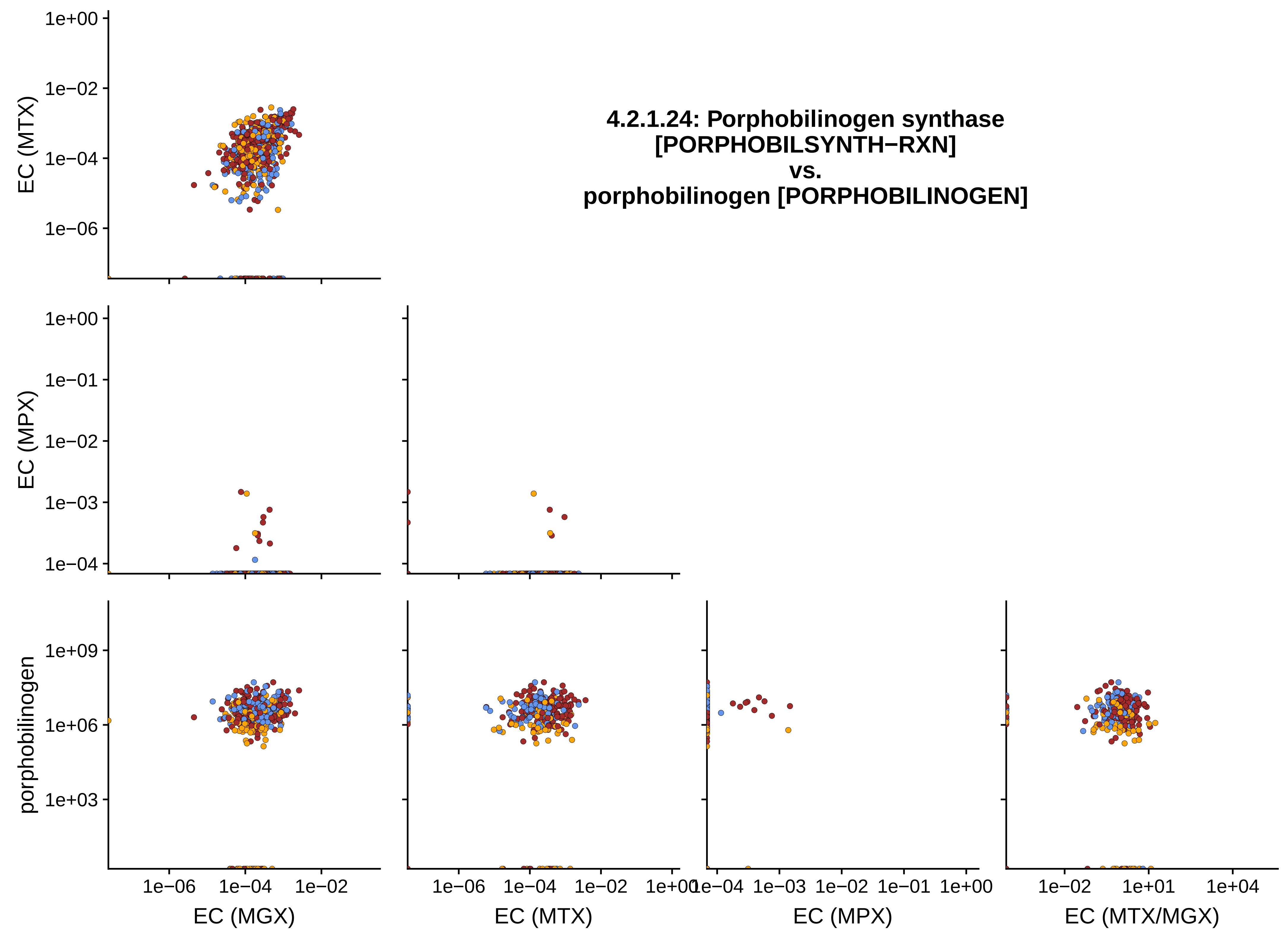

4.2.1.24: Porphobilinogen synthase
[PORPHOBILSYNTH−RXN]
vs.
porphobilinogen [PORPHOBILINOGEN]
EC (MTX)
EC (MPX)
porphobilinogen
EC (MGX)
EC (MTX)
EC (MPX)
EC (MTX/MGX)
1e+00
1e−02
1e−04
1e−06
1e−01
1e−03
1e+09
1e+06
1e+03
1e+01
1e+04

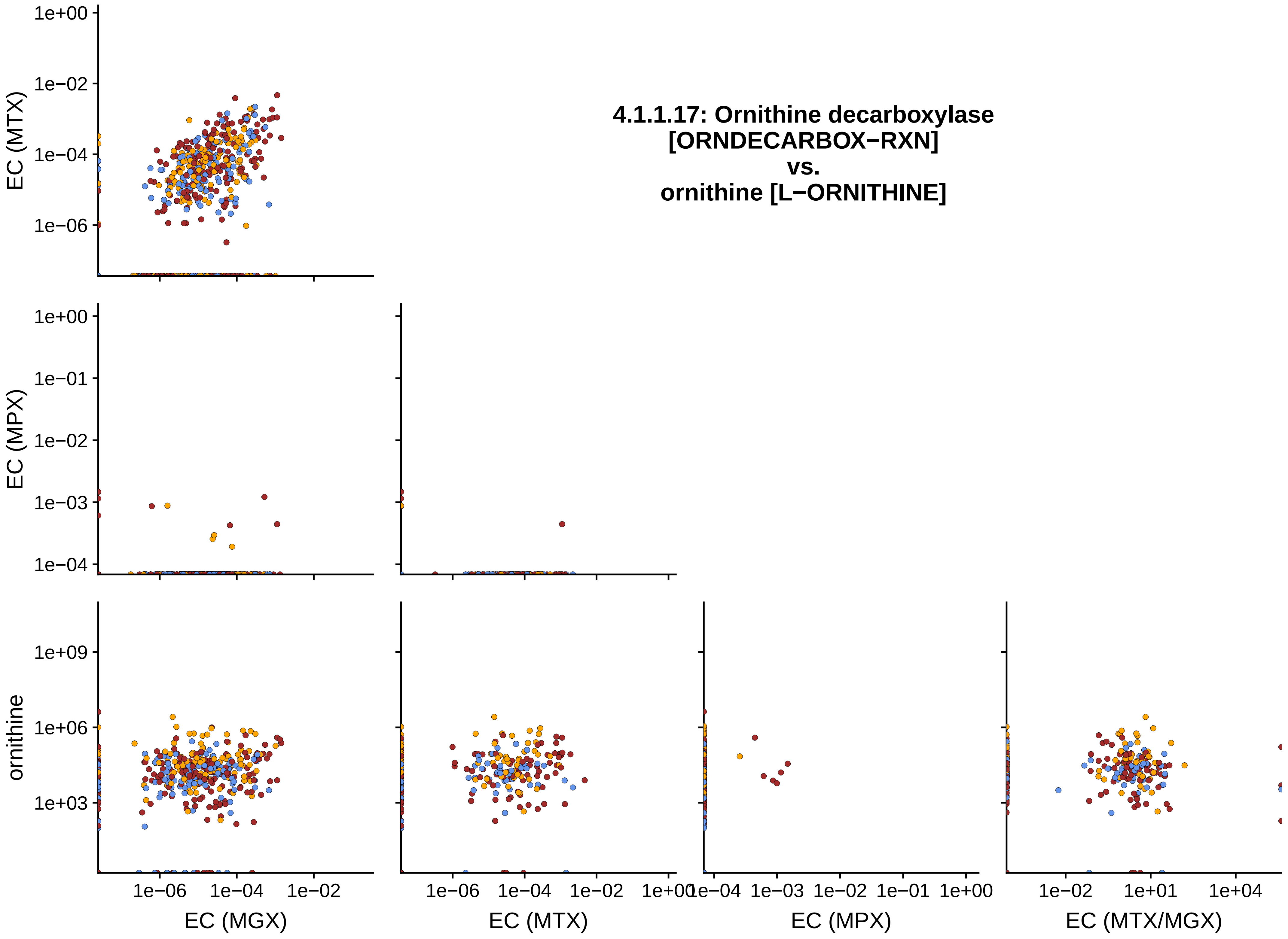
4.1.1.17: Ornithine decarboxylase
[ORNDECARBOX−RXN]
vs.
ornithine [L−ORNITHINE]
EC (MTX)
EC (MPX)
ornithine
EC (MGX)
EC (MTX)
EC (MPX)
EC (MTX/MGX)
1e+00
1e−02
1e−04
1e−06
1e−01
1e−03
1e+09
1e+06
1e+03
1e+04
1e+01

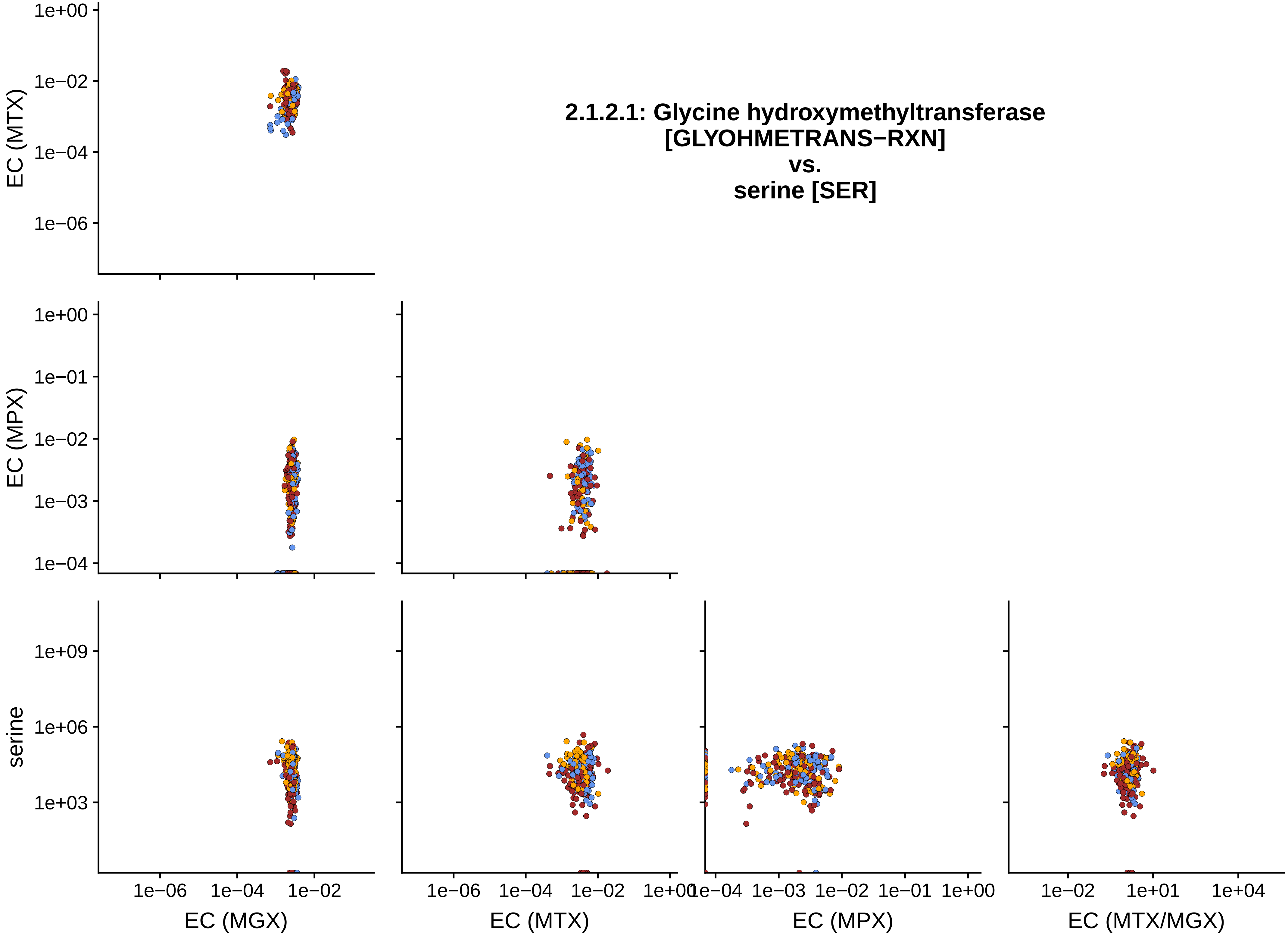
2.1.2.1: Glycine hydroxymethyltransferase
[GLYOHMETRANS−RXN]
vs.
serine [SER]
EC (MTX)
EC (MPX)
serine
EC (MGX)
EC (MTX)
EC (MPX)
EC (MTX/MGX)
1e+00
1e−02
1e−04
1e−06
1e−01
1e−03
1e+09
1e+06
1e+03
1e+01
1e+04

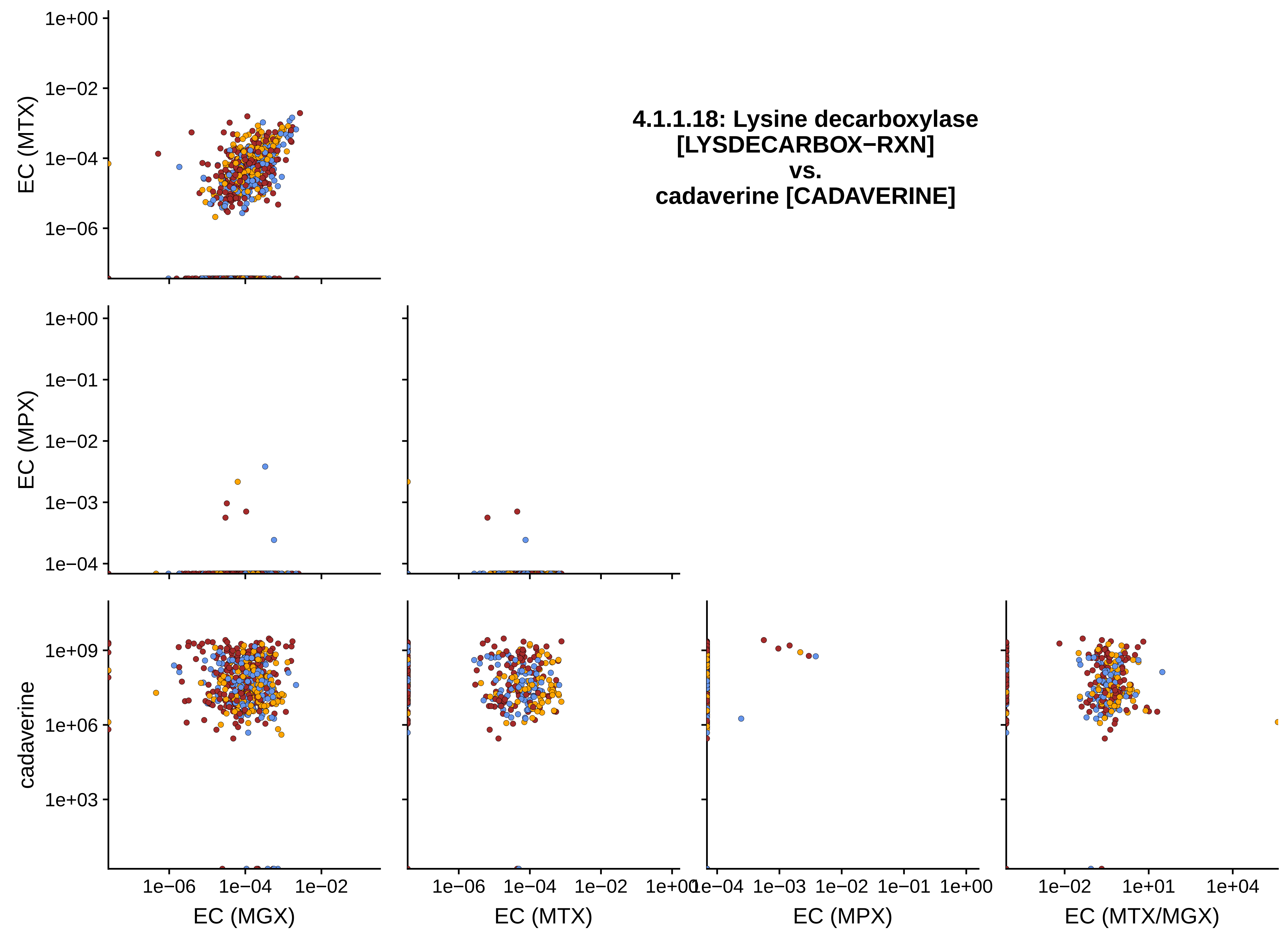
4.1.1.18: Lysine decarboxylase
[LYSDECARBOX−RXN]
vs.
cadaverine [CADAVERINE]
EC (MTX)
EC (MPX)
cadaverine
EC (MGX)
EC (MTX)
EC (MPX)
EC (MTX/MGX)
1e+00
1e−02
1e−04
1e−06
1e−01
1e−03
1e+09
1e+06
1e+03
1e+01
1e+04

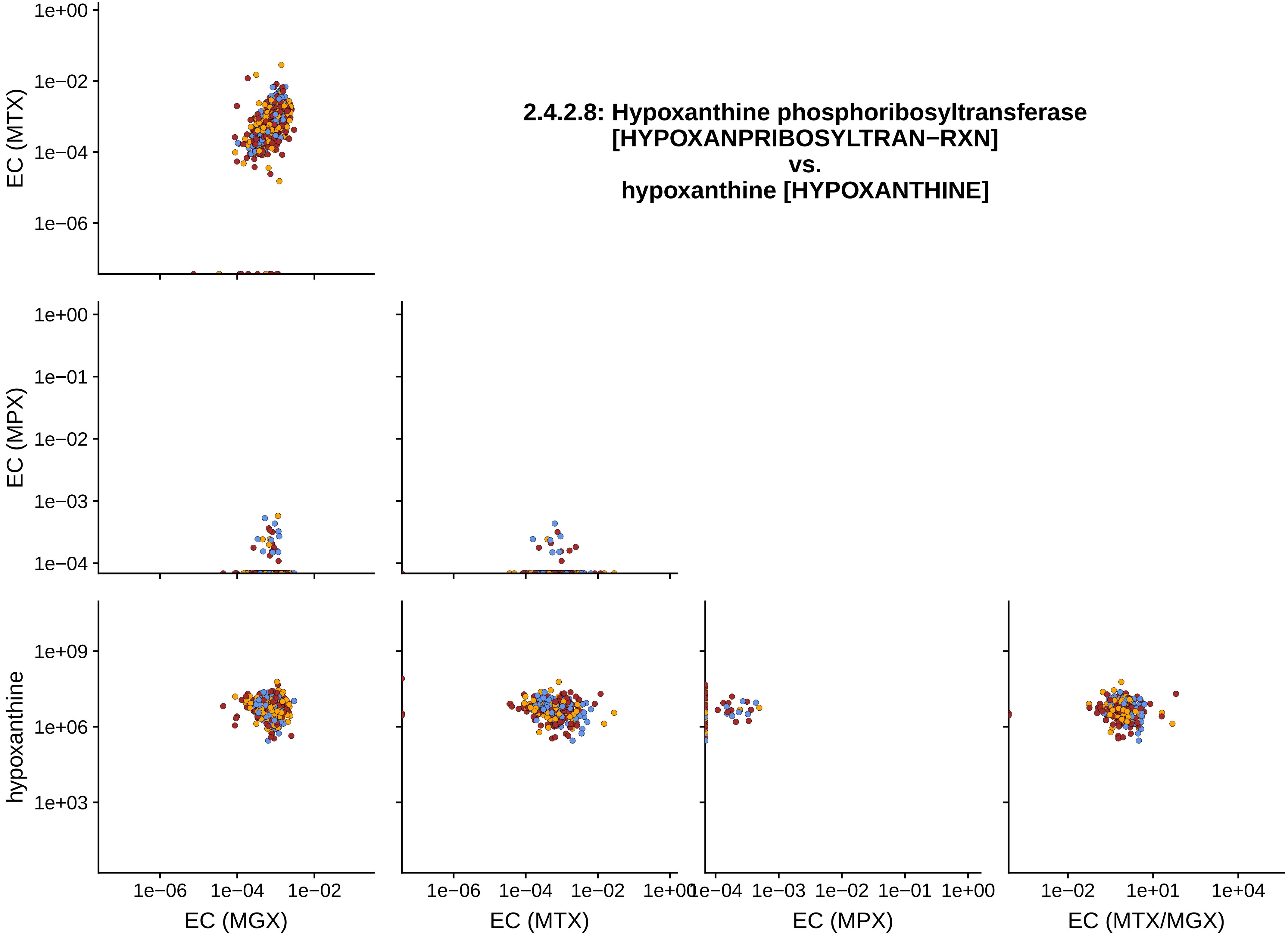

2.4.2.8: Hypoxanthine phosphoribosyltransferase
[HYPOXANPRIBOSYLTRAN−RXN]
vs.
hypoxanthine [HYPOXANTHINE]
EC (MTX)
EC (MPX)
hypoxanthine
EC (MGX)
EC (MTX)
EC (MPX)
EC (MTX/MGX)
1e+00
1e−02
1e−04
1e−06
1e−01
1e−03
1e+09
1e+06
1e+03
1e+01
1e+04

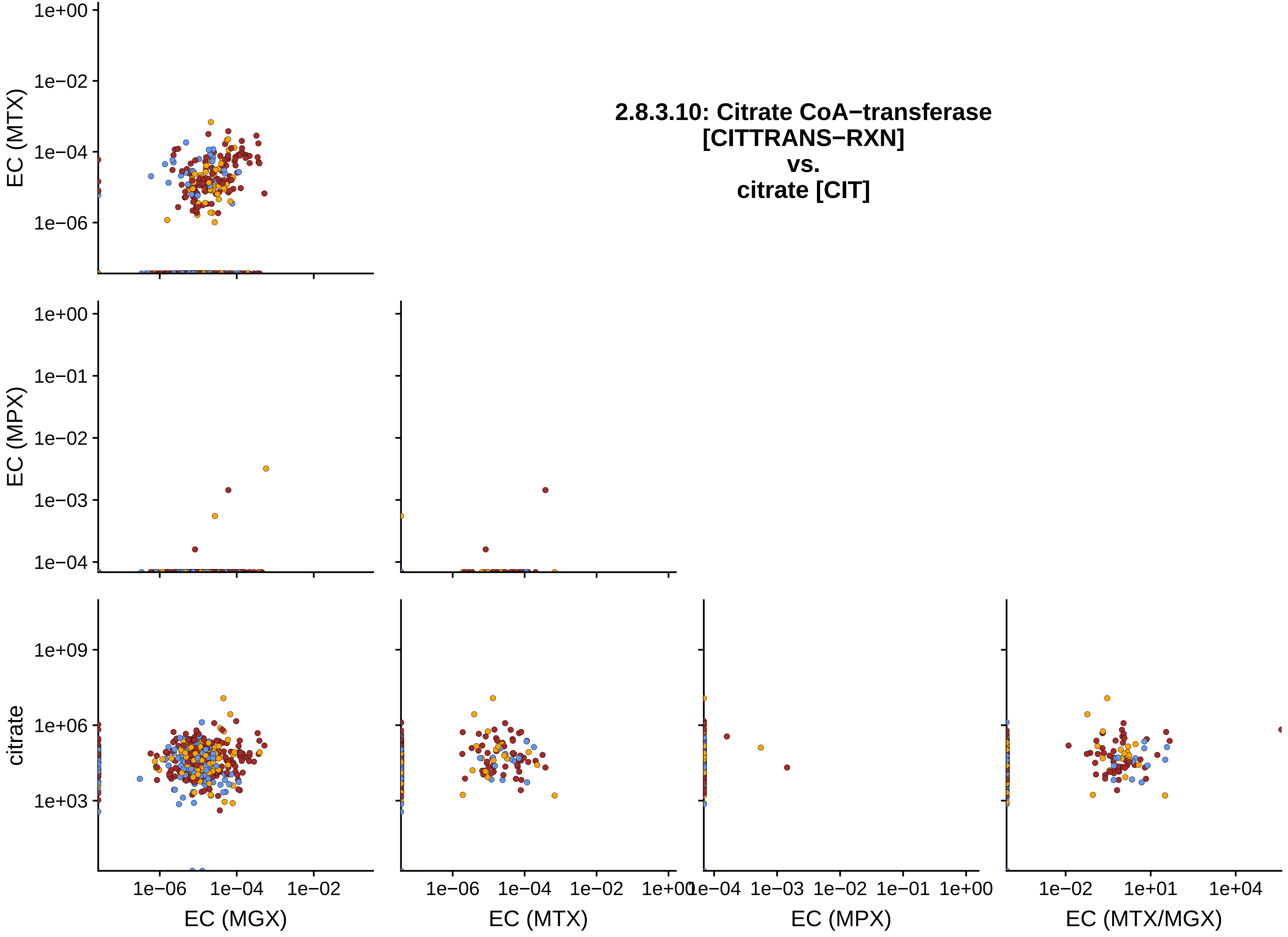

2.8.3.10: Citrate CoA−transferase
[CITTRANS−RXN]
vs.
citrate [CIT]
EC (MTX)
EC (MPX)
citrate
EC (MGX)
EC (MTX)
EC (MPX)
EC (MTX/MGX)
1e+00
1e−02
1e−04
1e−06
1e−01
1e−03
1e+09
1e+06
1e+03
1e+01
1e+04

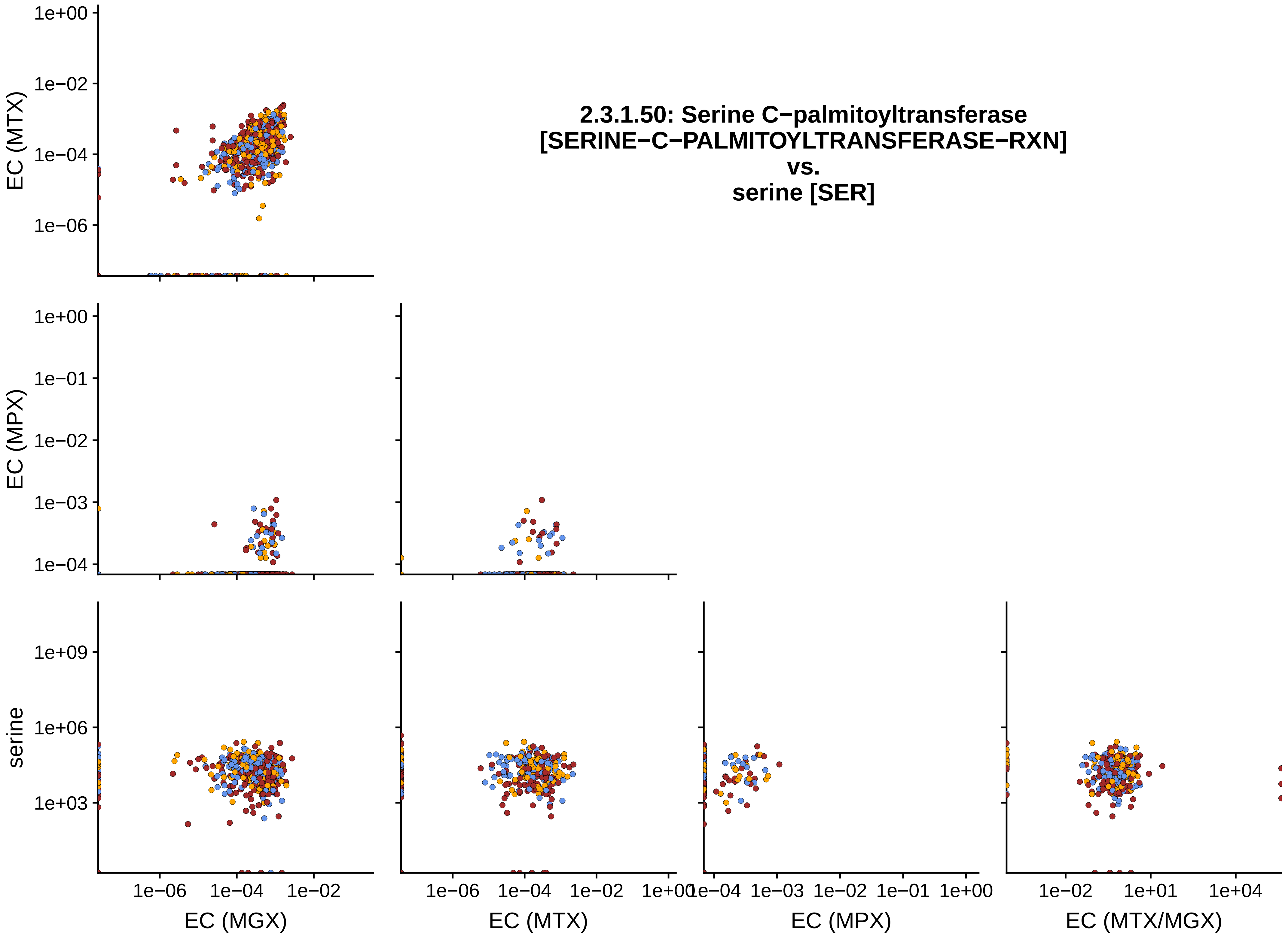
2.3.1.50: Serine C−palmitoyltransferase
[SERINE−C−PALMITOYLTRANSFERASE−RXN]
vs.
serine [SER]
EC (MTX)
EC (MPX)
serine
EC (MGX)
EC (MTX)
EC (MPX)
EC (MTX/MGX)
1e+00
1e−02
1e−04
1e−06
1e−01
1e−03
1e+09
1e+06
1e+03
1e+01
1e+04

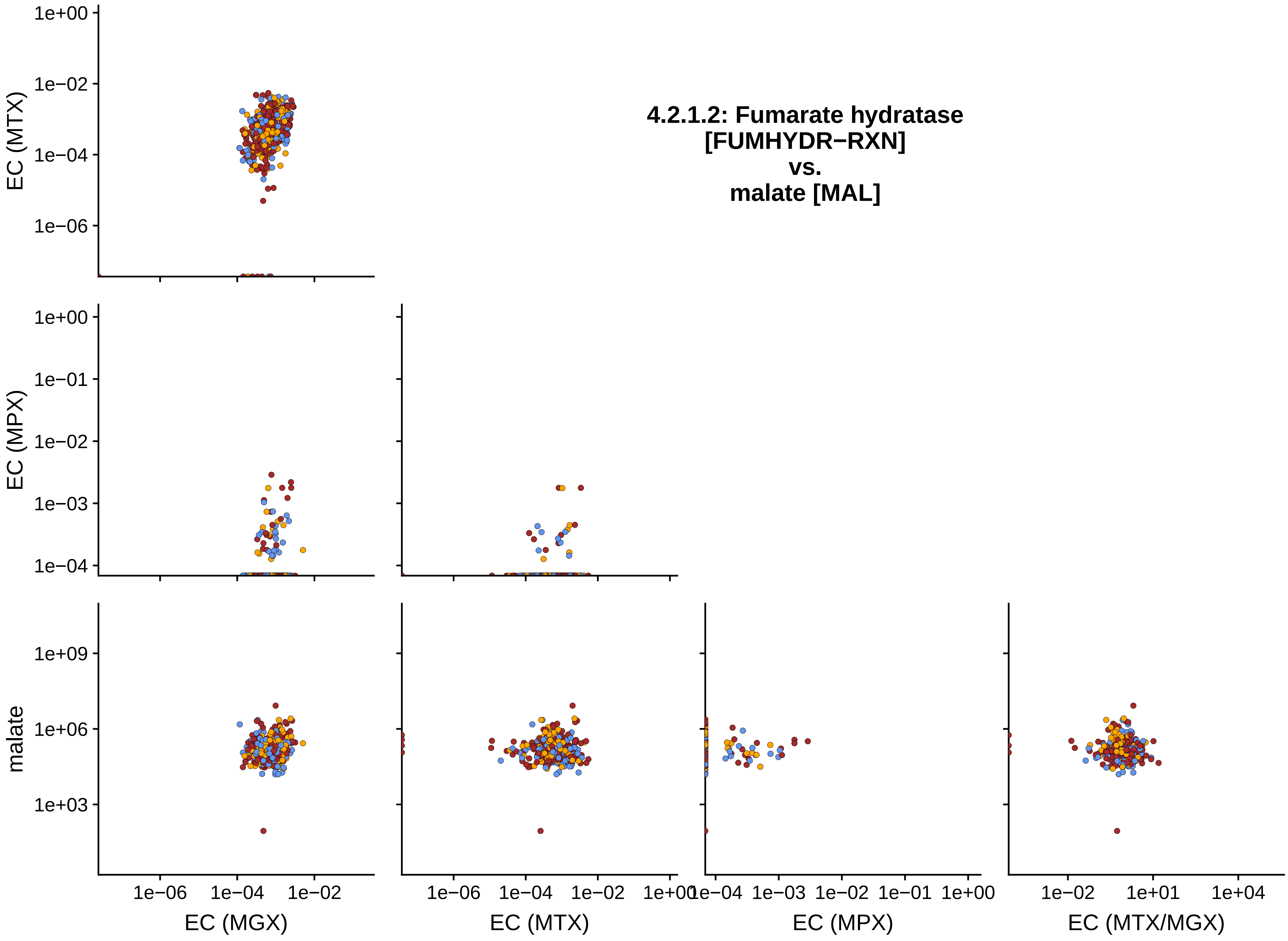
4.2.1.2: Fumarate hydratase
[FUMHYDR−RXN]
vs.
malate [MAL]
EC (MTX)
EC (MPX)
malate
EC (MGX)
EC (MTX)
EC (MPX)
EC (MTX/MGX)
1e+00
1e−02
1e−04
1e−06
1e−01
1e−03
1e+09
1e+06
1e+03
1e+01
1e+04

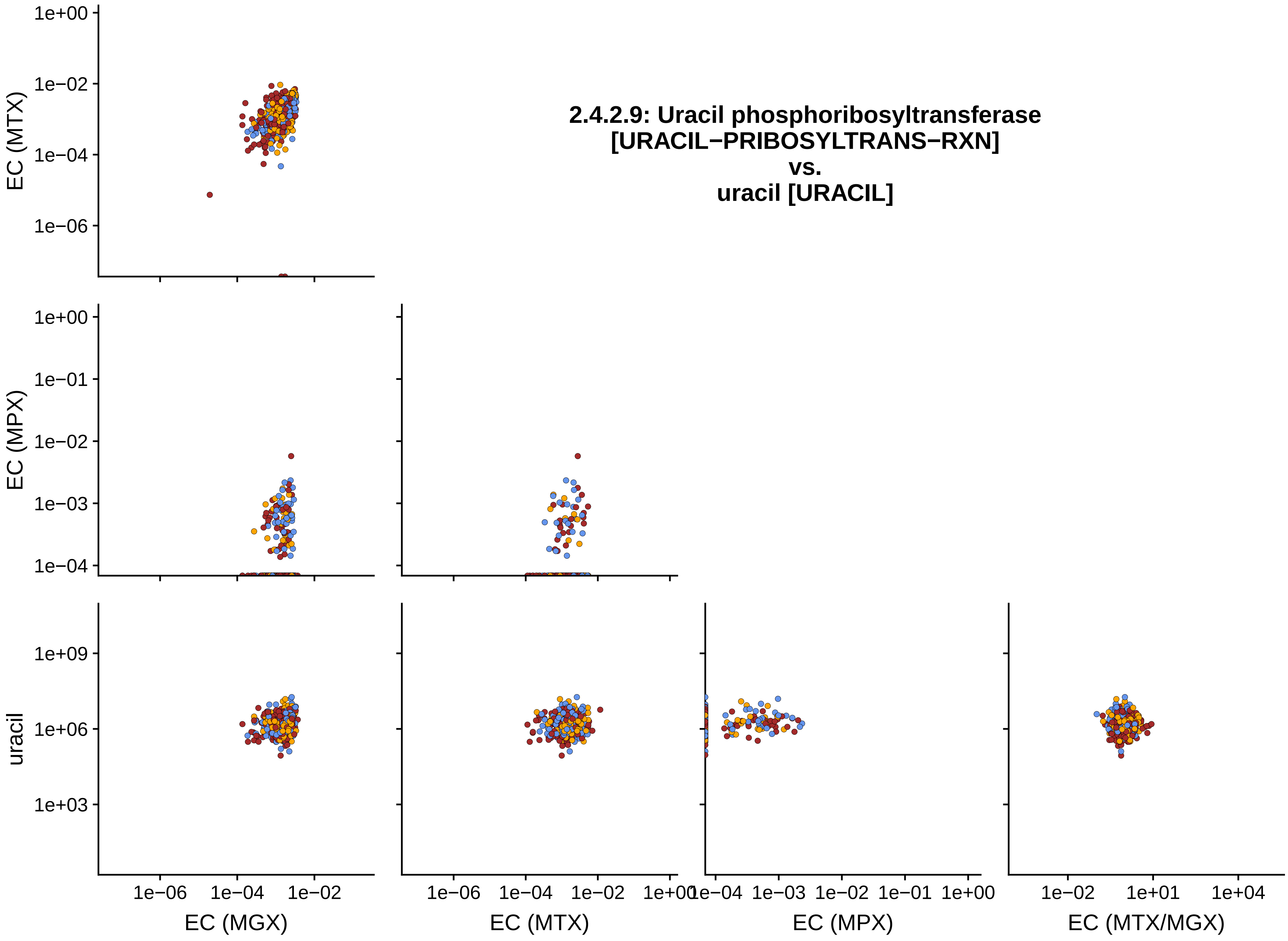
2.4.2.9: Uracil phosphoribosyltransferase
[URACIL−PRIBOSYLTRANS−RXN]
vs.
uracil [URACIL]
EC (MTX)
EC (MPX)
uracil
EC (MGX)
EC (MTX)
EC (MPX)
EC (MTX/MGX)
1e+00
1e−02
1e−04
1e−06
1e−01
1e−03
1e+09
1e+06
1e+03
1e+01
1e+04

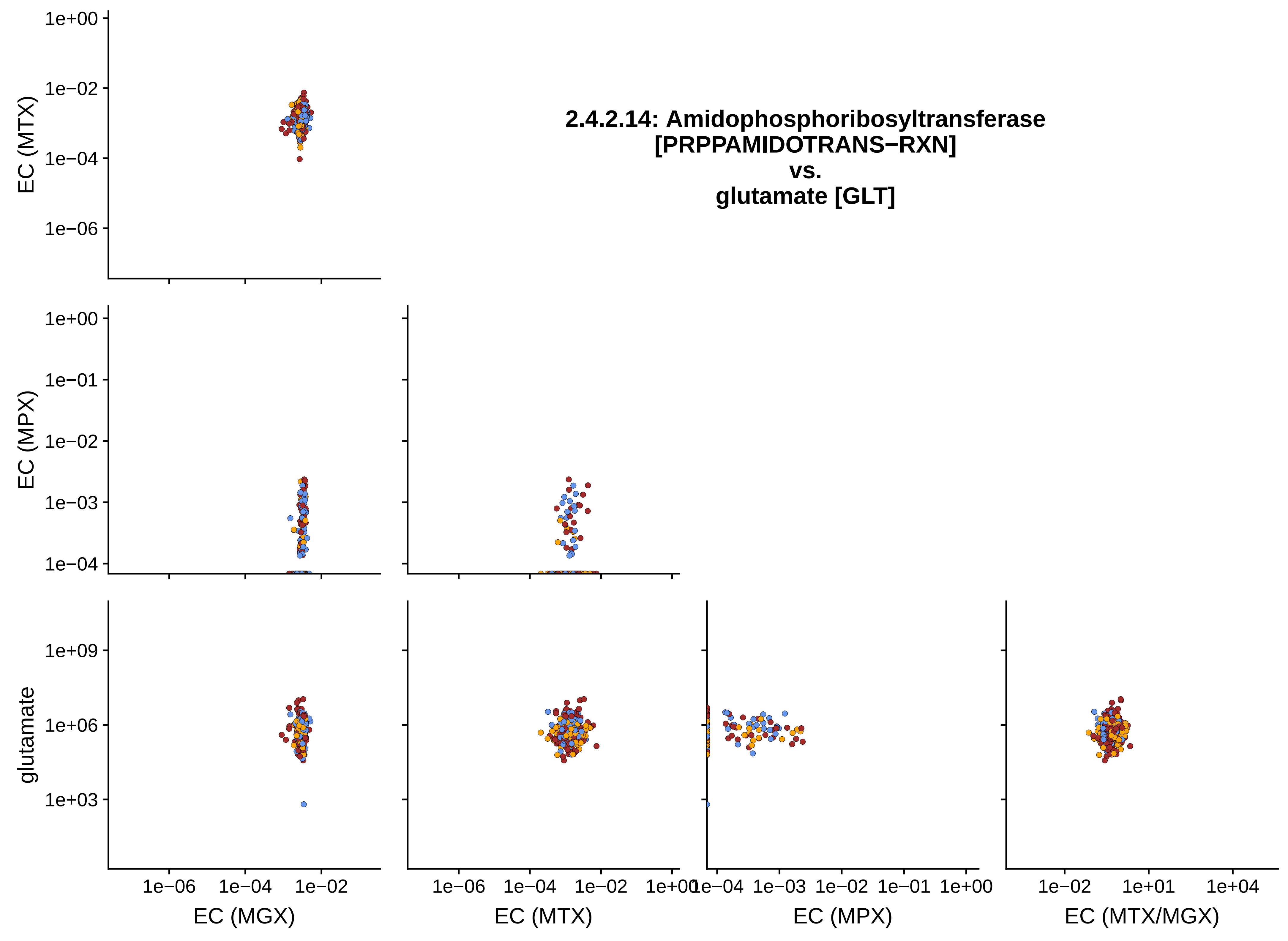

2.4.2.14: Amidophosphoribosyltransferase
[PRPPAMIDOTRANS−RXN]
vs.
glutamate [GLT]
EC (MTX)
EC (MPX)
glutamate
EC (MGX)
EC (MTX)
EC (MPX)
EC (MTX/MGX)
1e+00
1e−02
1e−04
1e−06
1e−01
1e−03
1e+09
1e+06
1e+03
1e+01
1e+04

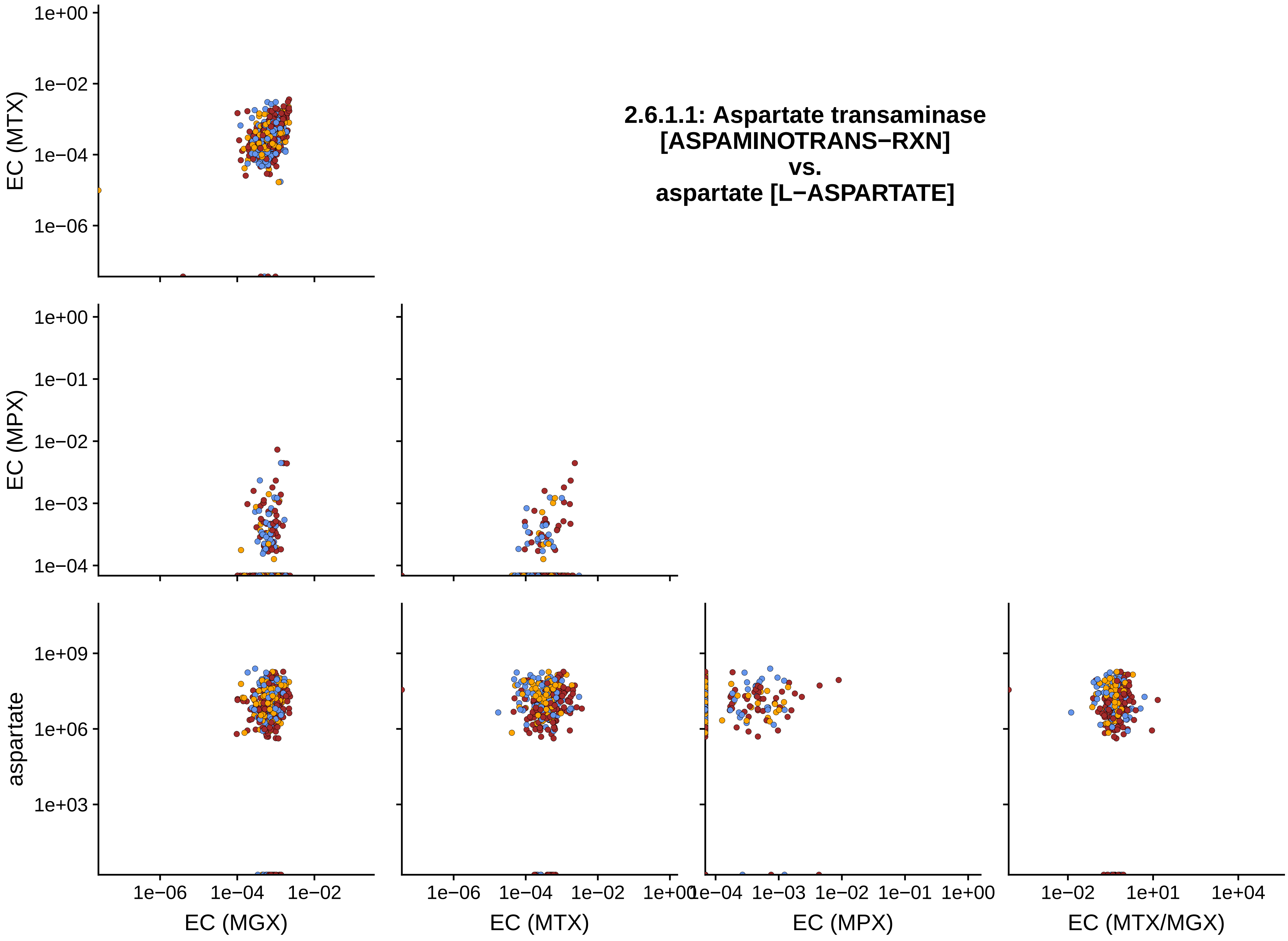

2.6.1.1: Aspartate transaminase
[ASPAMINOTRANS−RXN]
vs.
aspartate [L−ASPARTATE]
EC (MTX)
EC (MPX)
aspartate
EC (MGX)
EC (MTX)
EC (MPX)
EC (MTX/MGX)
1e+00
1e−02
1e−04
1e−06
1e−01
1e−03
1e+09
1e+06
1e+03
1e+01
1e+04

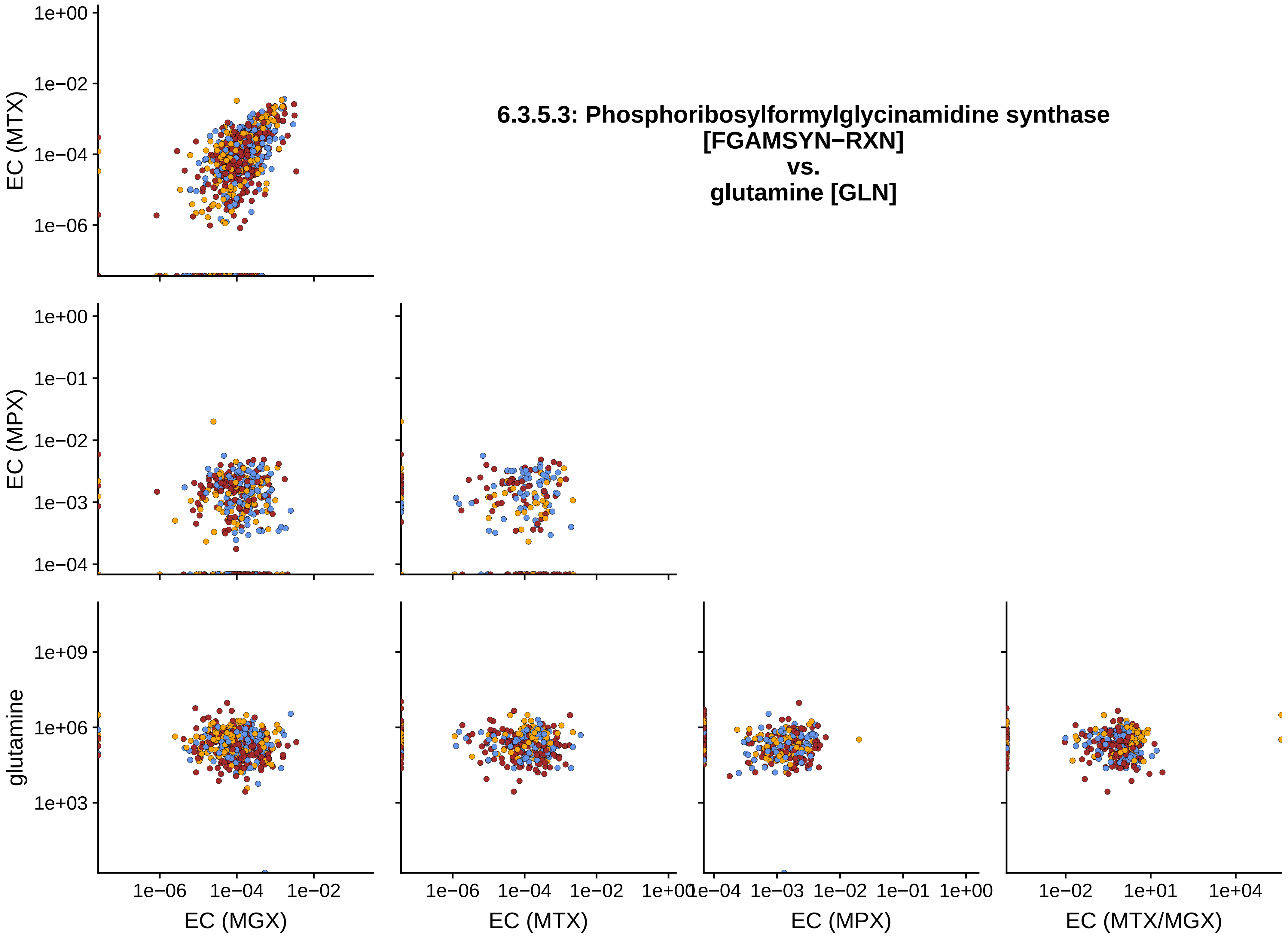

6.3.5.3: Phosphoribosylformylglycinamidine synthase
[FGAMSYN−RXN]
vs.
glutamine [GLN]
EC (MTX)
EC (MPX)
glutamine
EC (MGX)
EC (MTX)
EC (MPX)
EC (MTX/MGX)
1e+00
1e−02
1e−04
1e−06
1e−01
1e−03
1e+09
1e+06
1e+03
1e+01
1e+04

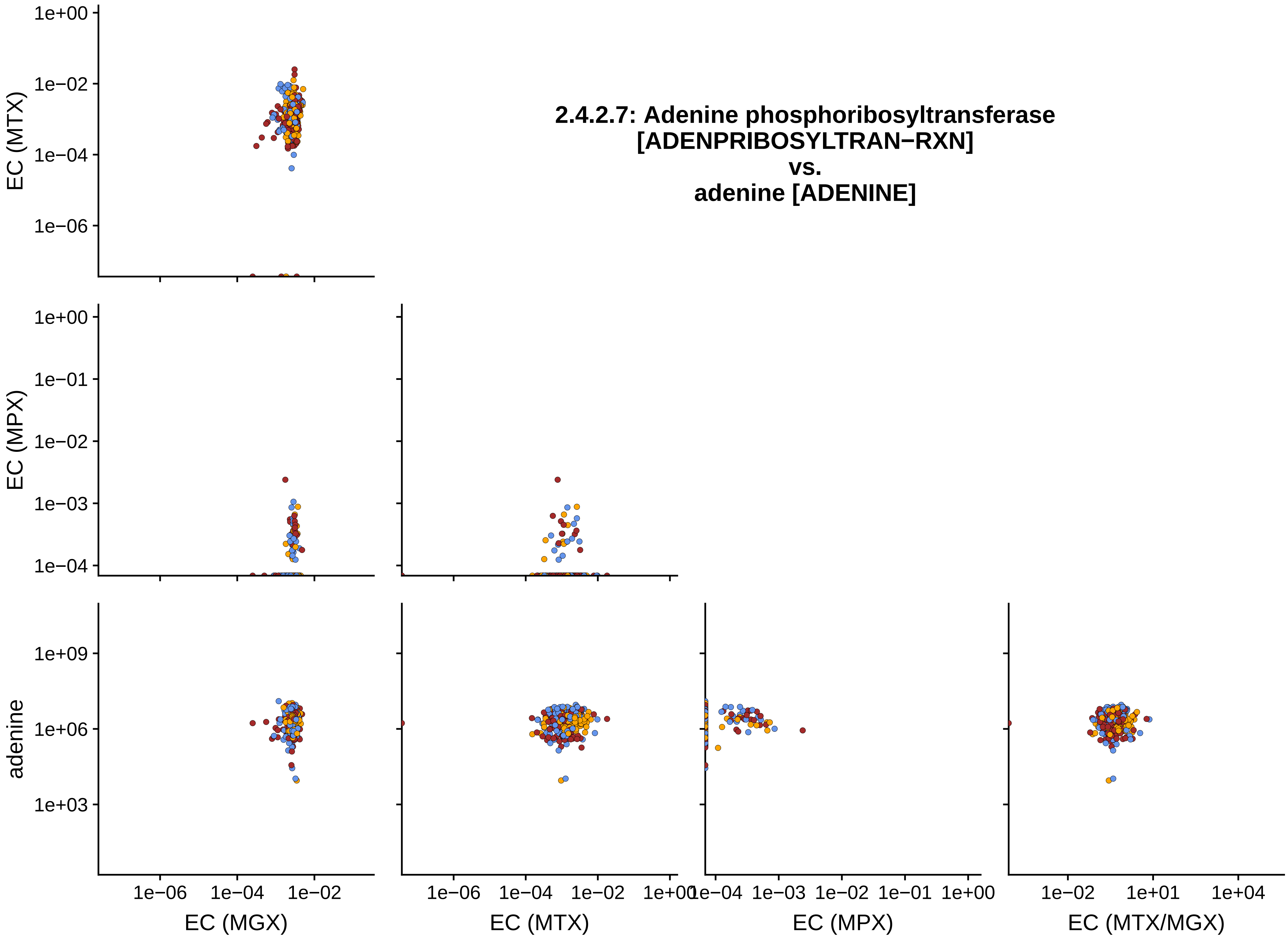

2.4.2.7: Adenine phosphoribosyltransferase
[ADENPRIBOSYLTRAN−RXN]
vs.
adenine [ADENINE]
EC (MTX)
EC (MPX)
adenine
EC (MGX)
EC (MTX)
EC (MPX)
EC (MTX/MGX)
1e+00
1e−02
1e−04
1e−06
1e−01
1e−03
1e+09
1e+06
1e+03
1e+01
1e+04

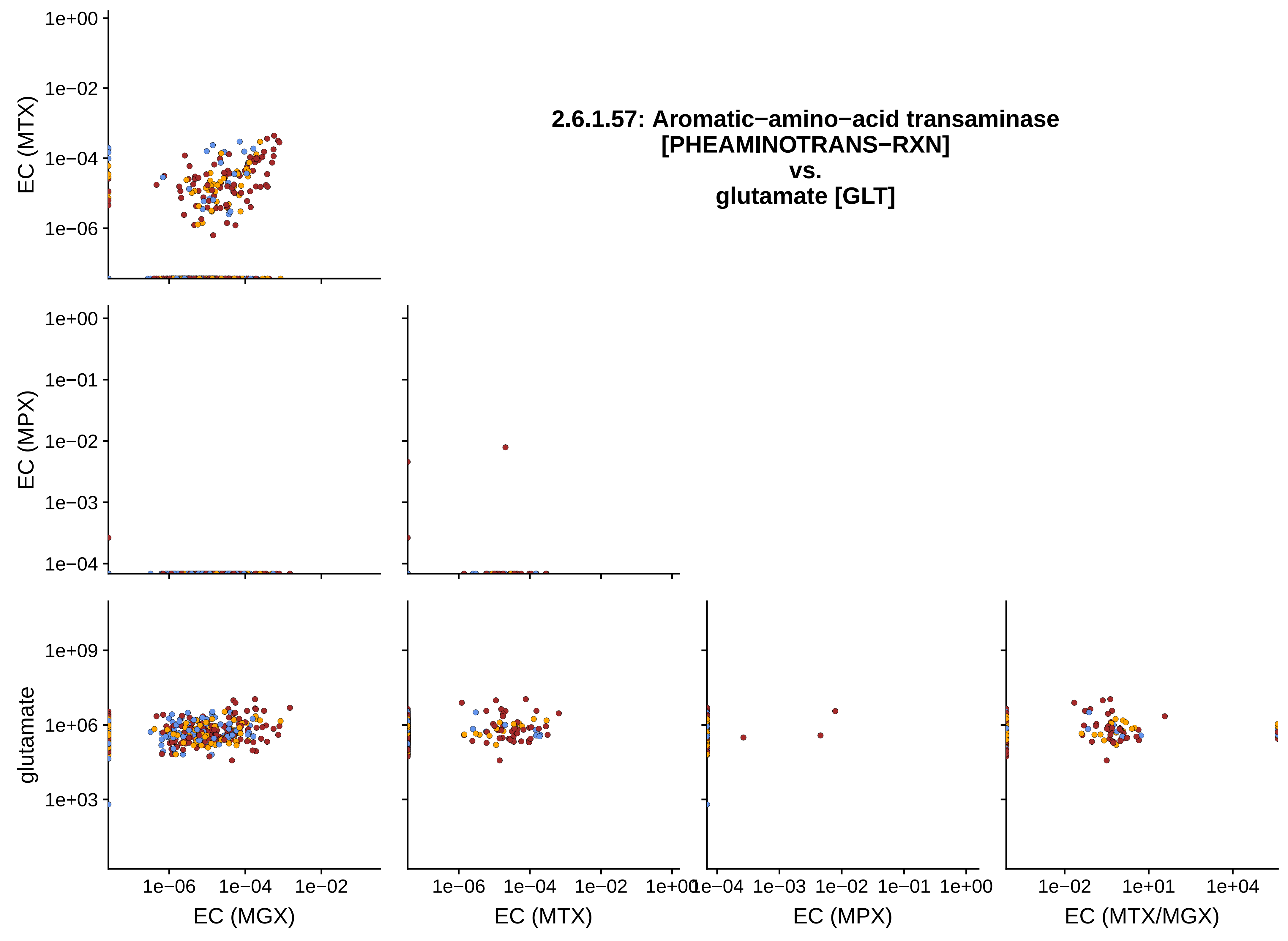
2.6.1.57: Aromatic−amino−acid transaminase
[PHEAMINOTRANS−RXN]
vs.
glutamate [GLT]
EC (MTX)
EC (MPX)
glutamate
EC (MGX)
EC (MTX)
EC (MPX)
EC (MTX/MGX)
1e+00
1e−02
1e−04
1e−06
1e−01
1e−03
1e+09
1e+06
1e+03
1e+01
1e+04

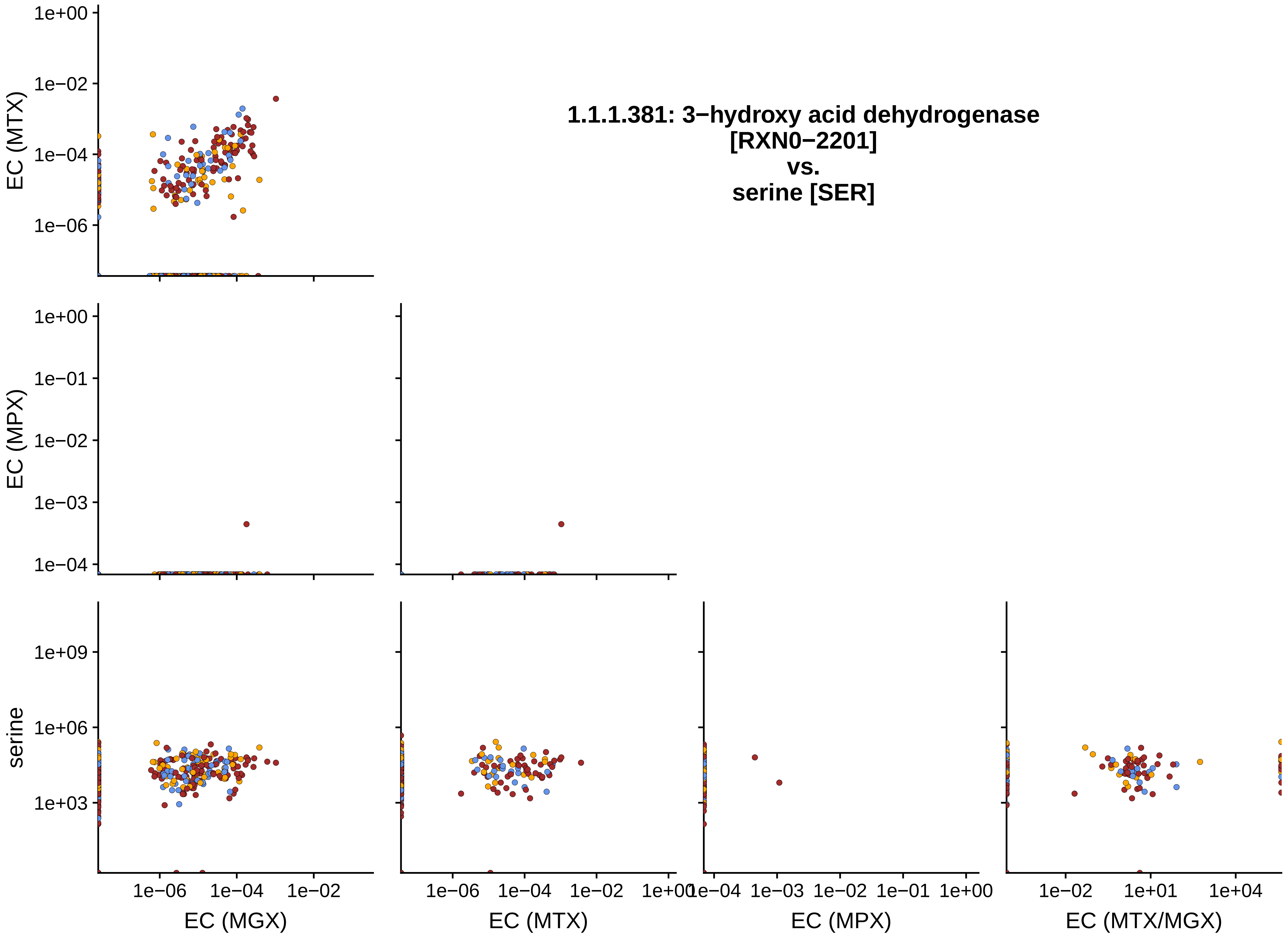
1.1.1.381: 3−hydroxy acid dehydrogenase
[RXN0−2201]
vs.
serine [SER]
EC (MTX)
EC (MPX)
serine
EC (MGX)
EC (MTX)
EC (MPX)
EC (MTX/MGX)
1e+00
1e−02
1e−04
1e−06
1e−01
1e−03
1e+09
1e+06
1e+03
1e+01
1e+04

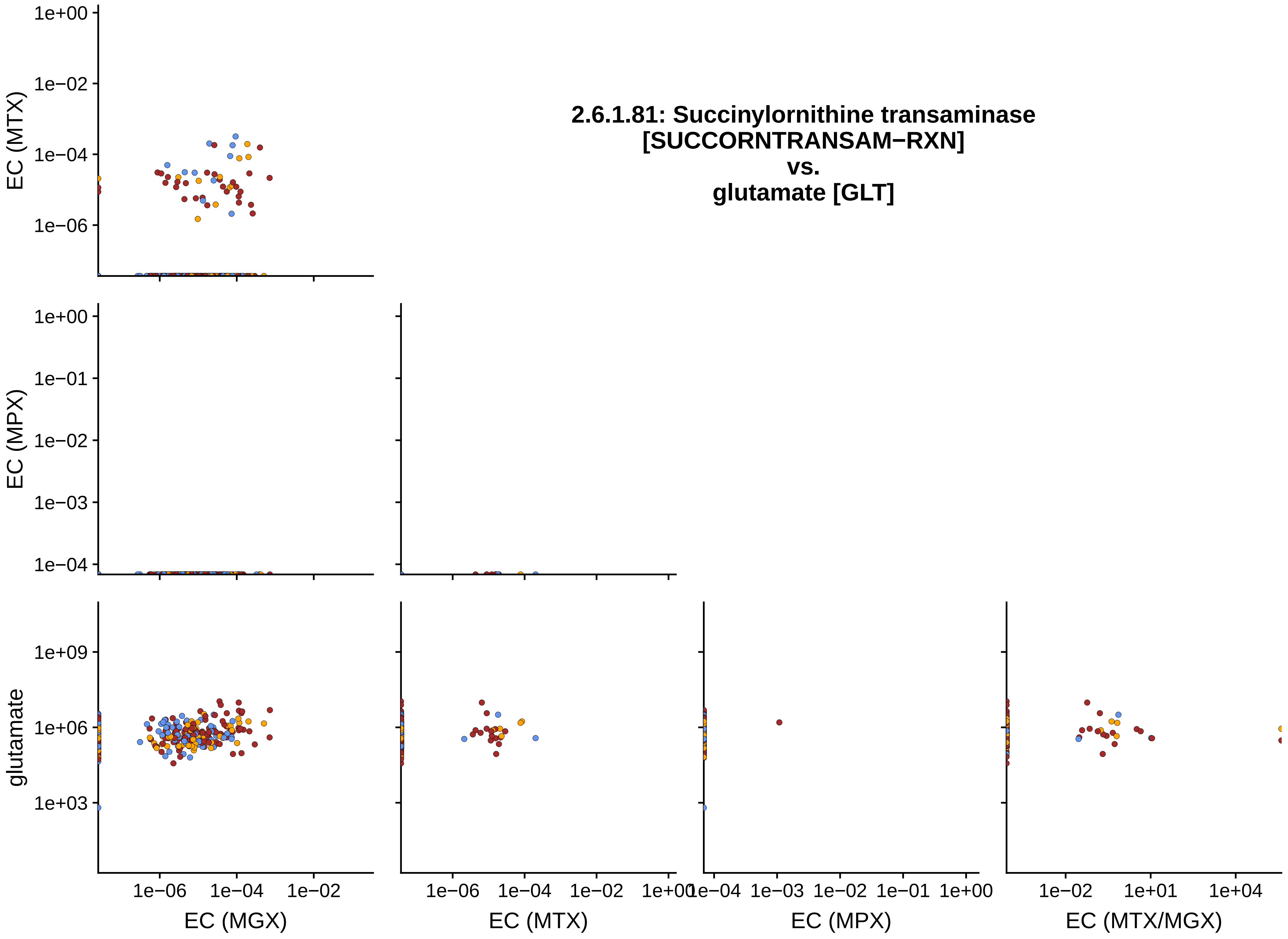
2.6.1.81: Succinylornithine transaminase
[SUCCORNTRANSAM−RXN]
vs.
glutamate [GLT]
EC (MTX)
EC (MPX)
glutamate
EC (MGX)
EC (MTX)
EC (MPX)
EC (MTX/MGX)
1e+00
1e−02
1e−04
1e−06
1e−01
1e−03
1e+09
1e+06
1e+03
1e+01
1e+04

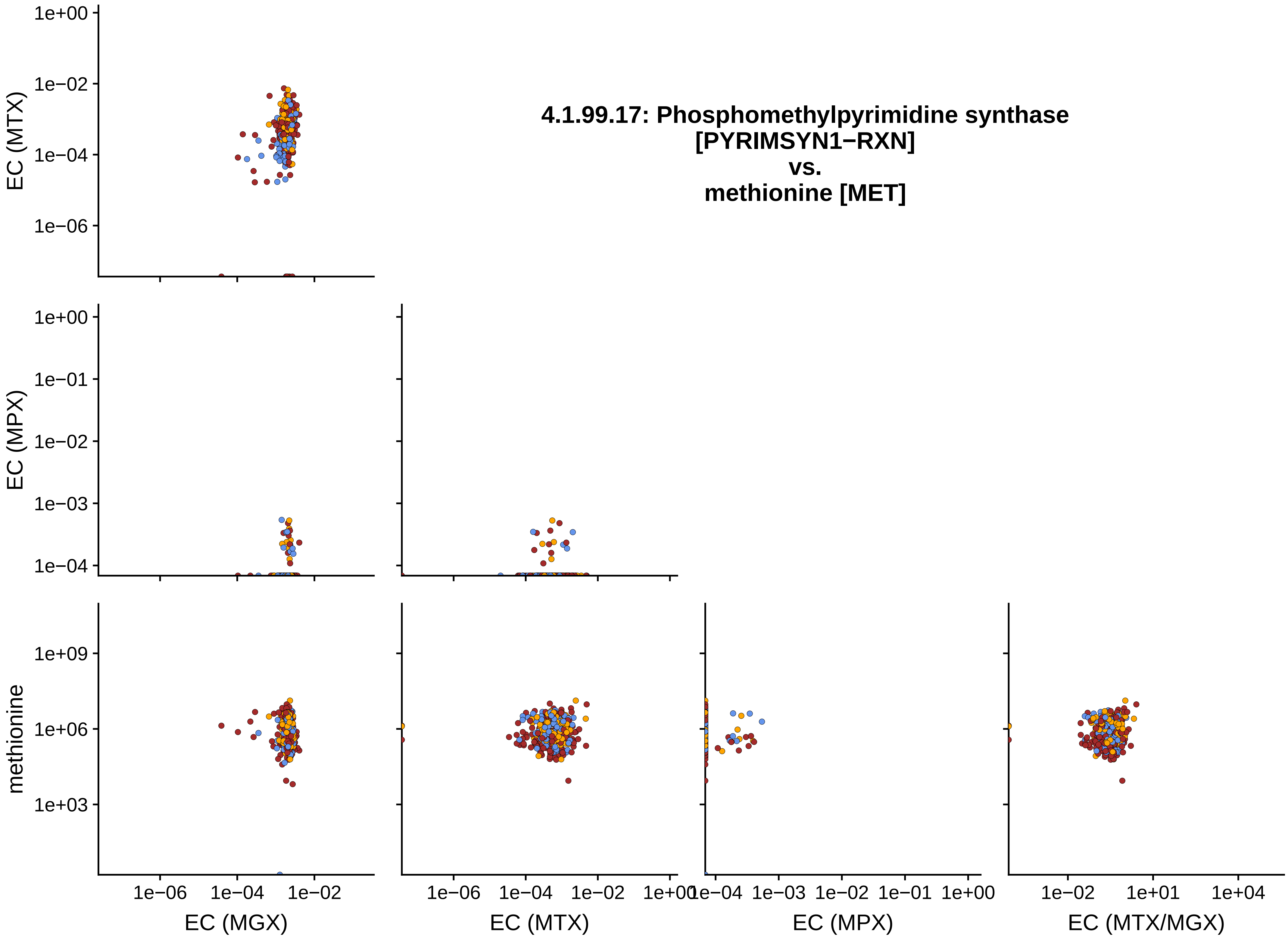
4.1.99.17: Phosphomethylpyrimidine synthase
[PYRIMSYN1−RXN]
vs.
methionine [MET]
EC (MTX)
EC (MPX)
methionine
EC (MGX)
EC (MTX)
EC (MPX)
EC (MTX/MGX)
1e+00
1e−02
1e−04
1e−06
1e+00
1e−01
1e−02
1e−03
1e−04
1e+09
1e+06
1e+03
1e−06
1e−04
1e−02
1e−06
1e−04
1e−02
1e+00
1e−04
1e−03
1e−02
1e−01
1e+00
1e−02
1e+01
1e+04

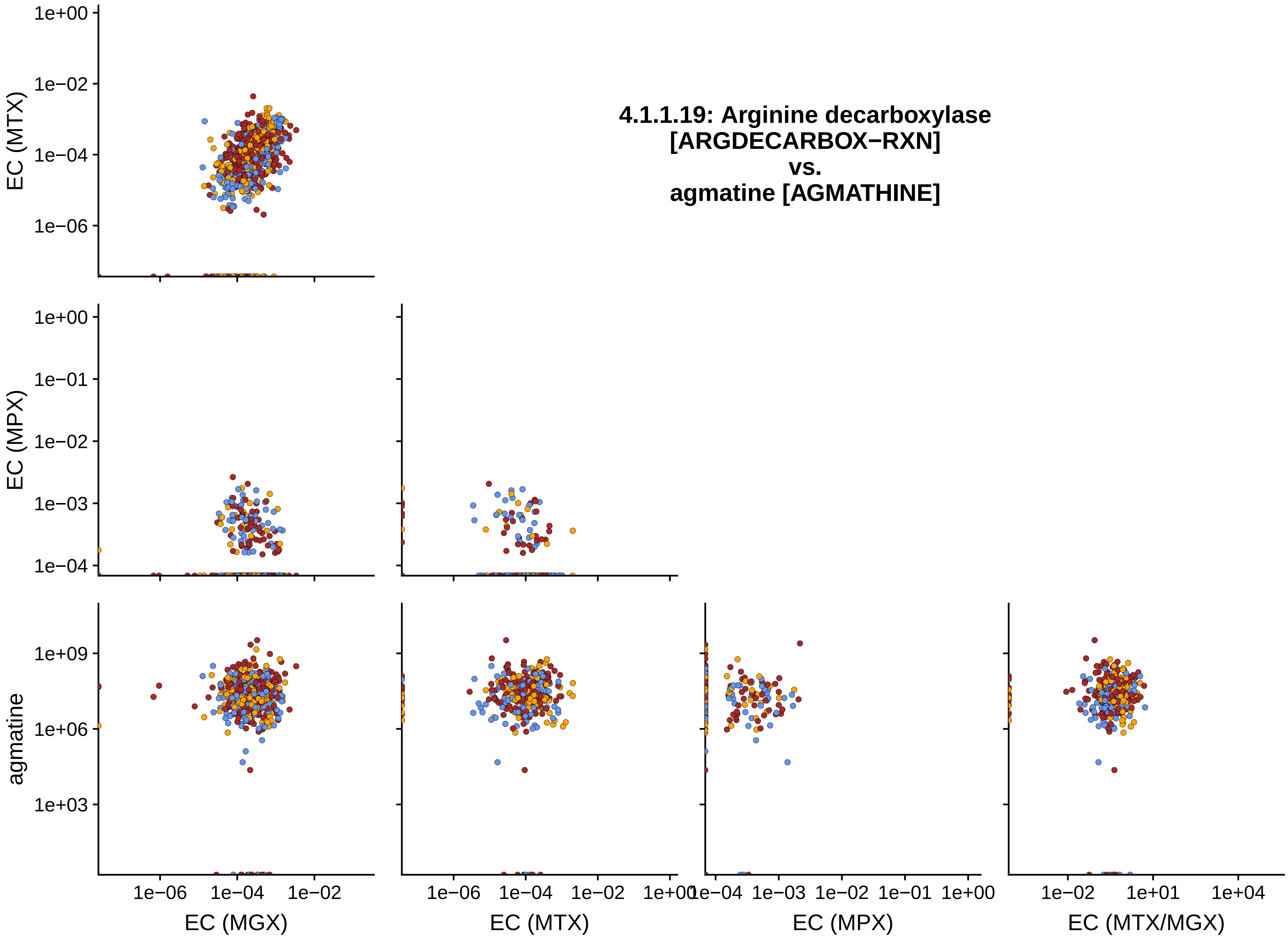

4.1.1.19: Arginine decarboxylase
[ARGDECARBOX−RXN]
vs.
agmatine [AGMATHINE]
EC (MTX)
EC (MPX)
agmatine
EC (MGX)
EC (MTX)
EC (MPX)
EC (MTX/MGX)
1e+00
1e−02
1e−04
1e−06
1e−01
1e−03
1e+09
1e+06
1e+03
1e+01
1e+04

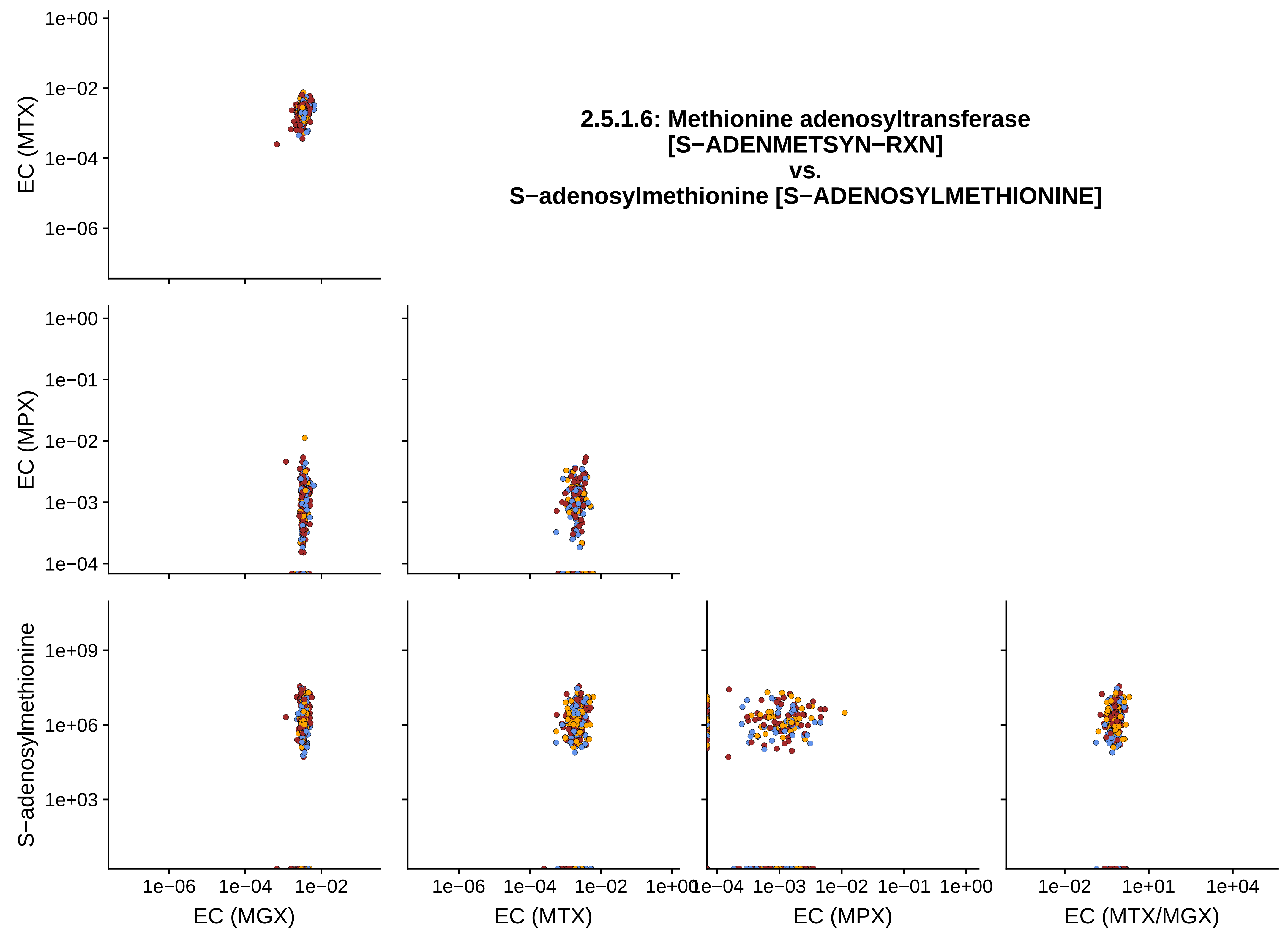
2.5.1.6: Methionine adenosyltransferase
[S−ADENMETSYN−RXN]
vs.
S−adenosylmethionine [S−ADENOSYLMETHIONINE]
EC (MTX)
EC (MPX)
S−adenosylmethionine
EC (MGX)
EC (MTX)
EC (MPX)
EC (MTX/MGX)
1e+00
1e−02
1e−04
1e−06
1e−01
1e−03
1e+09
1e+06
1e+03
1e+01
1e+04

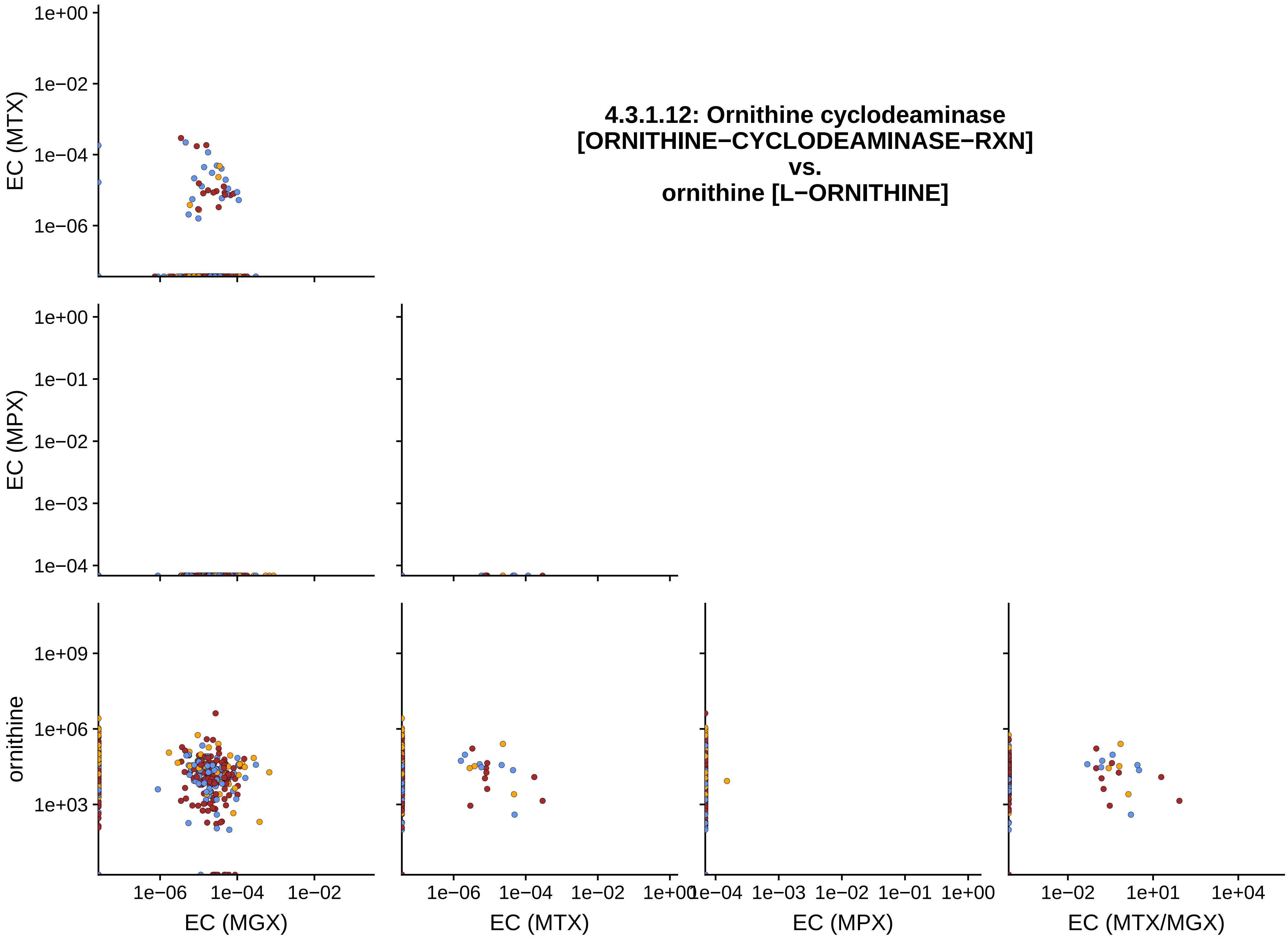
4.3.1.12: Ornithine cyclodeaminase
[ORNITHINE−CYCLODEAMINASE−RXN]
vs.
ornithine [L−ORNITHINE]
EC (MTX)
EC (MPX)
ornithine
EC (MGX)
EC (MTX)
EC (MPX)
EC (MTX/MGX)
1e+00
1e−02
1e−04
1e−06
1e−01
1e−03
1e+09
1e+06
1e+03
1e+01
1e+04

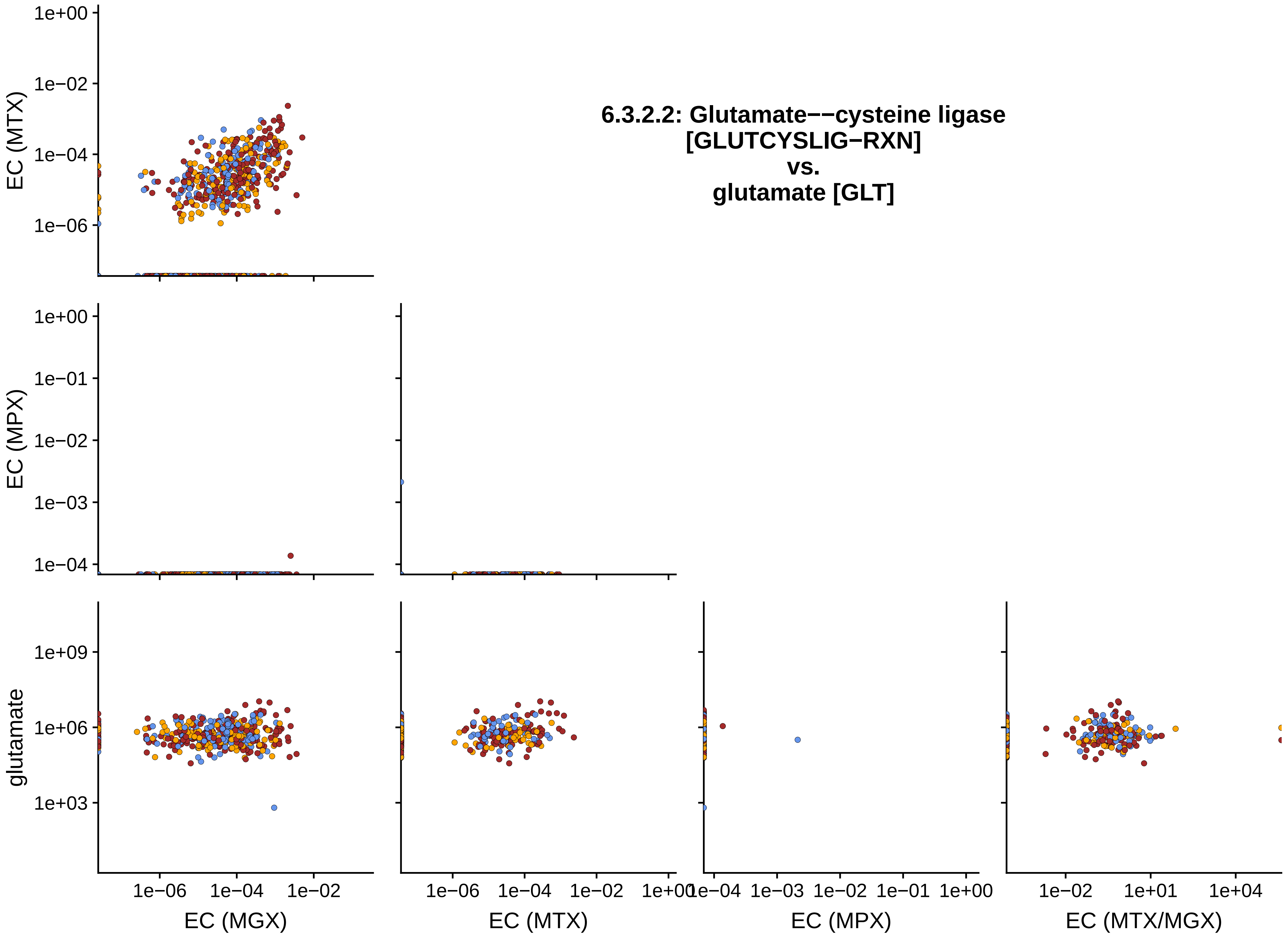

6.3.2.2: Glutamate−−cysteine ligase
[GLUTCYSLIG−RXN]
vs.
glutamate [GLT]
EC (MTX)
EC (MPX)
glutamate
EC (MGX)
EC (MTX)
EC (MPX)
EC (MTX/MGX)
1e+00
1e−02
1e−04
1e−06
1e−01
1e−03
1e+09
1e+06
1e+03
1e+04
1e+01

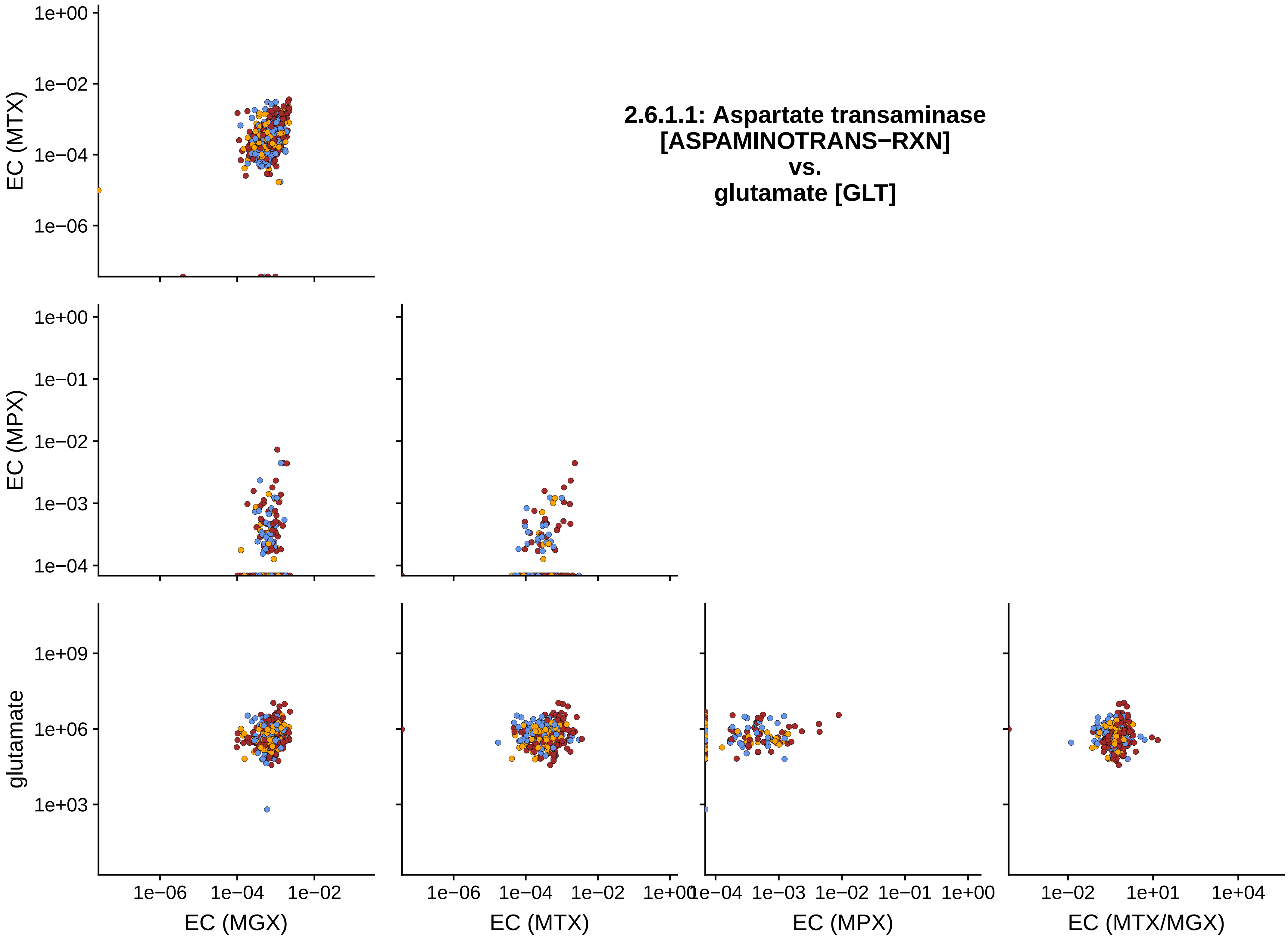

2.6.1.1: Aspartate transaminase
[ASPAMINOTRANS−RXN]
vs.
glutamate [GLT]
EC (MTX)
EC (MPX)
glutamate
EC (MGX)
EC (MTX)
EC (MPX)
EC (MTX/MGX)
1e+00
1e−02
1e−04
1e−06
1e−01
1e−03
1e+09
1e+06
1e+03
1e+01
1e+04

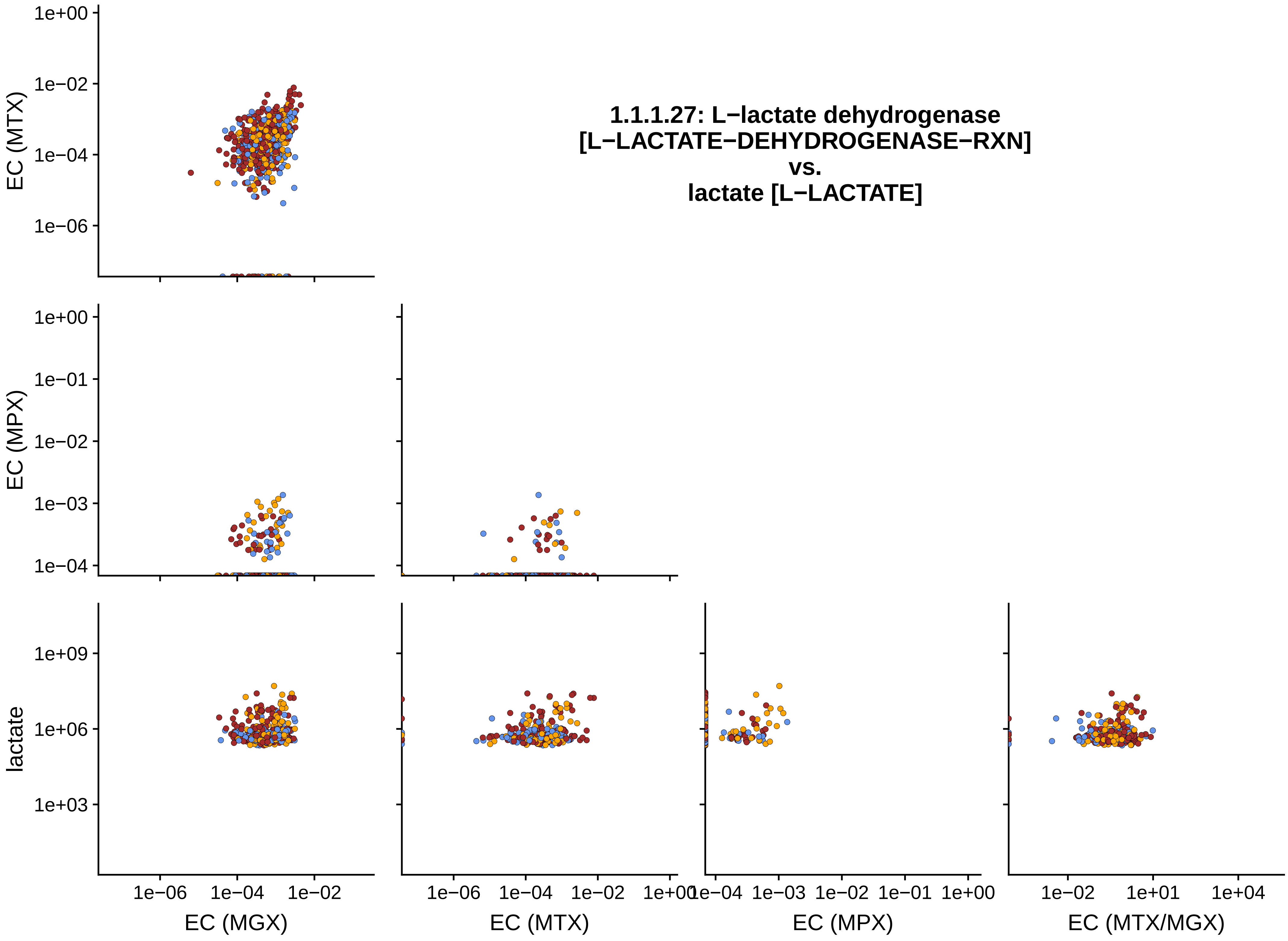

1.1.1.27: L−lactate dehydrogenase
[L−LACTATE−DEHYDROGENASE−RXN]
vs.
lactate [L−LACTATE]
EC (MTX)
EC (MPX)
lactate
EC (MGX)
EC (MTX)
EC (MPX)
EC (MTX/MGX)
1e+00
1e−02
1e−04
1e−06
1e−01
1e−03
1e+09
1e+06
1e+03
1e+01
1e+04

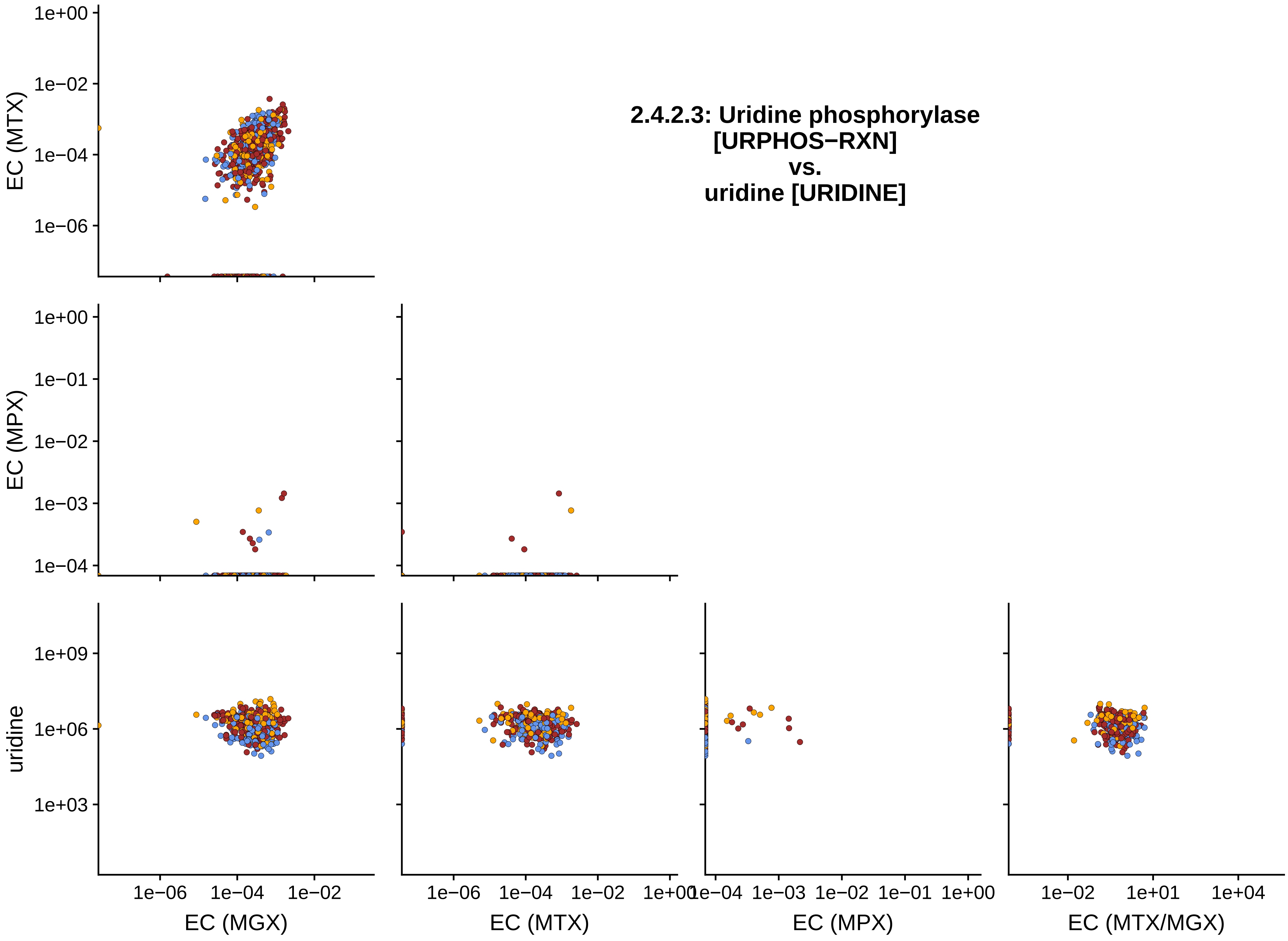
2.4.2.3: Uridine phosphorylase
[URPHOS−RXN]
vs.
uridine [URIDINE]
EC (MTX)
EC (MPX)
uridine
EC (MGX)
EC (MTX)
EC (MPX)
EC (MTX/MGX)
1e+00
1e−02
1e−04
1e−06
1e−01
1e−03
1e+09
1e+06
1e+03
1e+01
1e+04

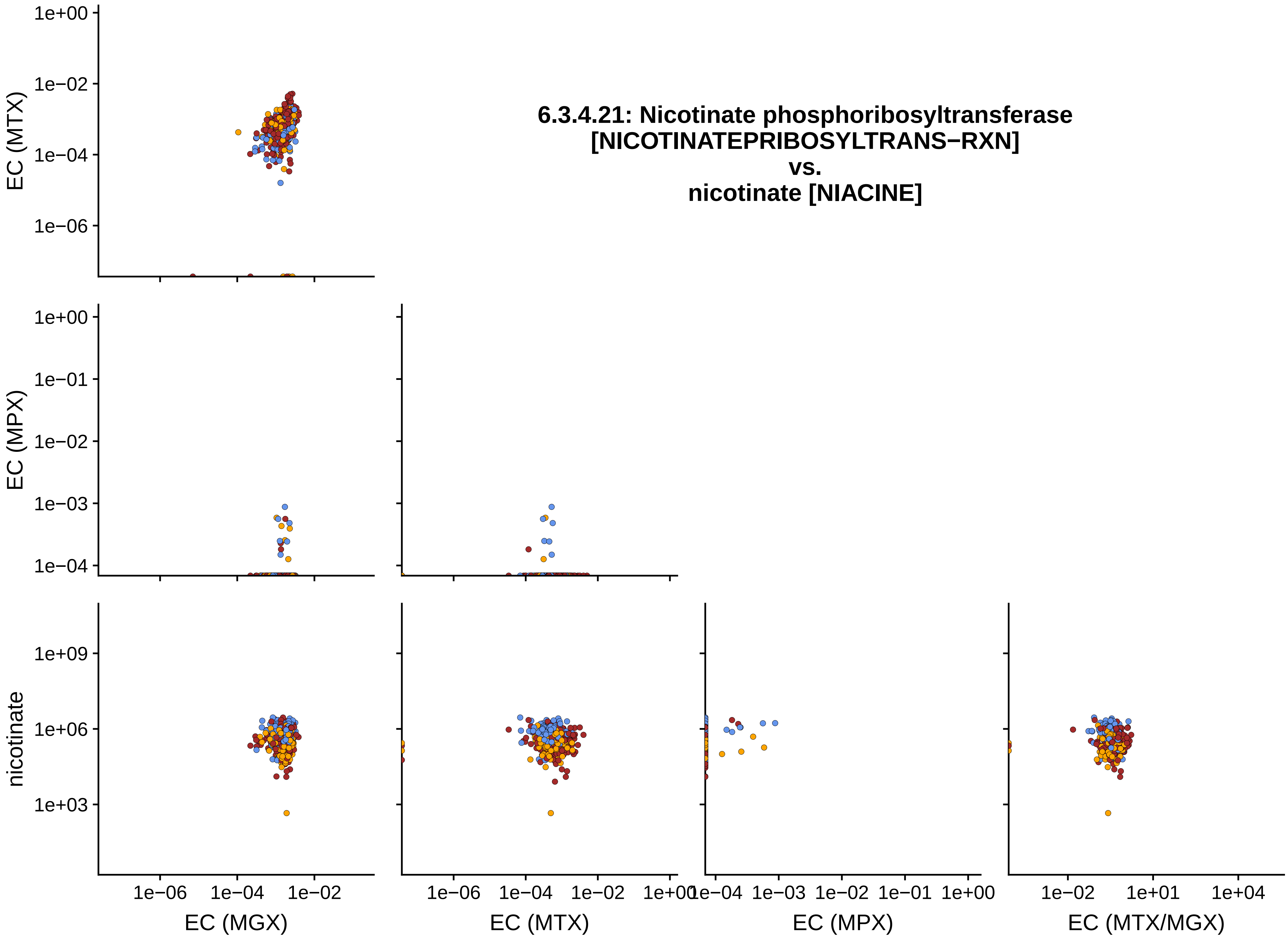

6.3.4.21: Nicotinate phosphoribosyltransferase
[NICOTINATEPRIBOSYLTRANS−RXN]
vs.
nicotinate [NIACINE]
EC (MTX)
EC (MPX)
nicotinate
EC (MGX)
EC (MTX)
EC (MPX)
EC (MTX/MGX)
1e+00
1e−02
1e−04
1e−06
1e−01
1e−03
1e+09
1e+06
1e+03
1e+01
1e+04

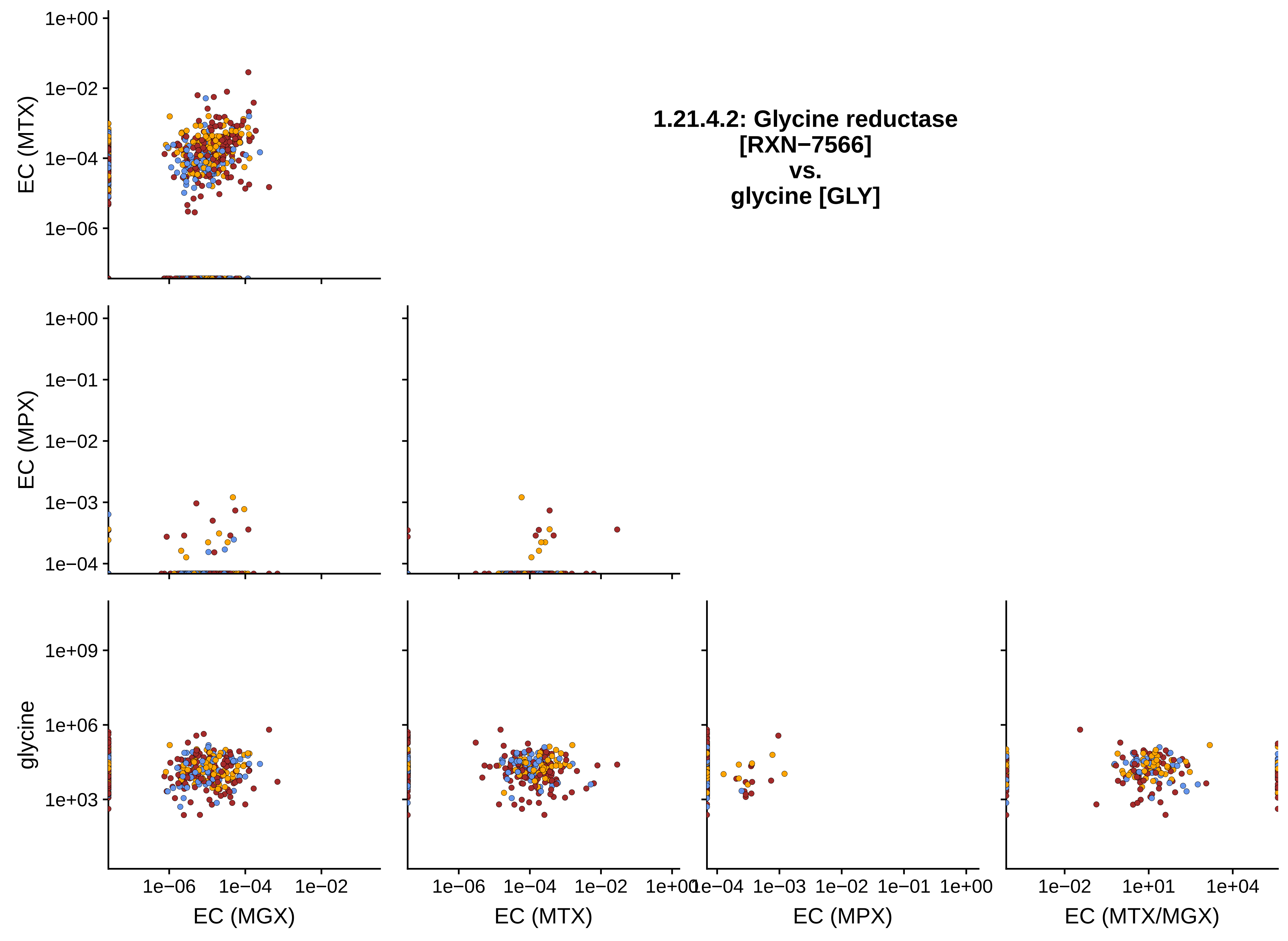

1.21.4.2: Glycine reductase
[RXN−7566]
vs.
glycine [GLY]
EC (MTX)
EC (MPX)
glycine
EC (MGX)
EC (MTX)
EC (MPX)
EC (MTX/MGX)
1e+00
1e−02
1e−04
1e−06
1e−01
1e−03
1e+09
1e+06
1e+03
1e+01
1e+04

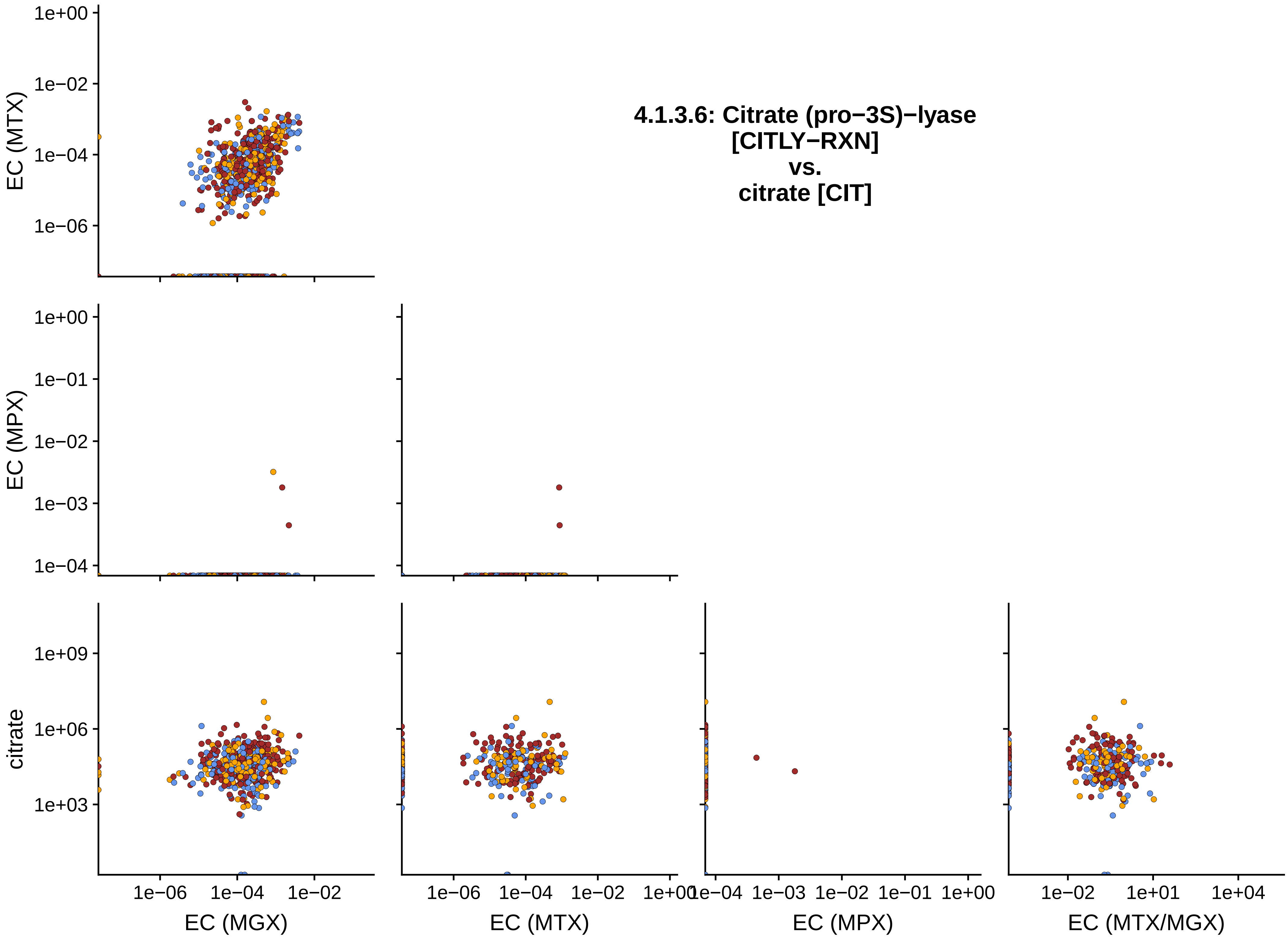
4.1.3.6: Citrate (pro−3S)−lyase
[CITLY−RXN]
vs.
citrate [CIT]
EC (MTX)
EC (MPX)
citrate
EC (MGX)
EC (MTX)
EC (MPX)
EC (MTX/MGX)
1e+00
1e−02
1e−04
1e−06
1e−01
1e−03
1e+09
1e+06
1e+03
1e+01
1e+04

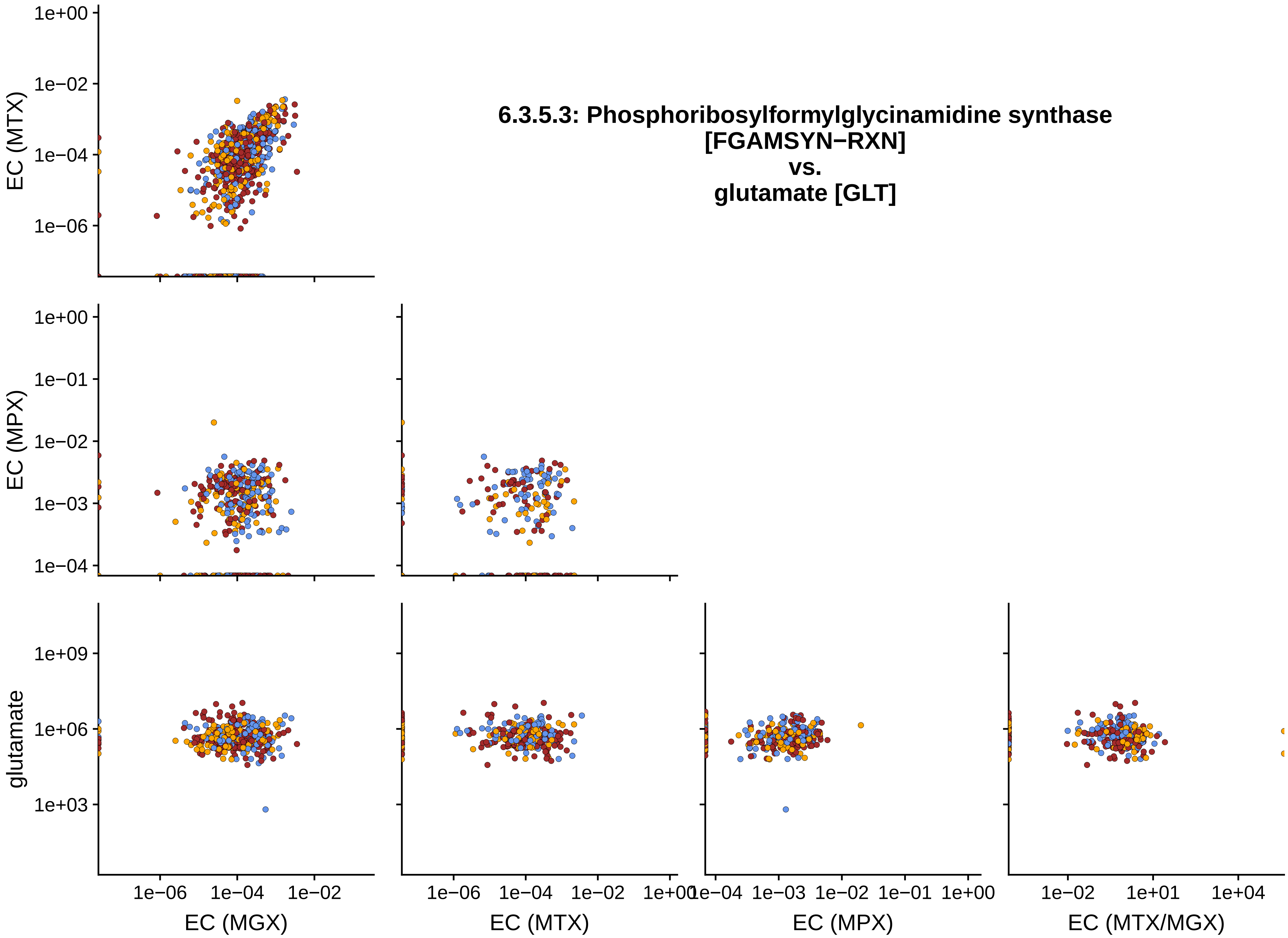

6.3.5.3: Phosphoribosylformylglycinamidine synthase
[FGAMSYN−RXN]
vs.
glutamate [GLT]
EC (MTX)
EC (MPX)
glutamate
EC (MGX)
EC (MTX)
EC (MPX)
EC (MTX/MGX)
1e+00
1e−02
1e−04
1e−06
1e−01
1e−03
1e+09
1e+06
1e+03
1e+01
1e+04

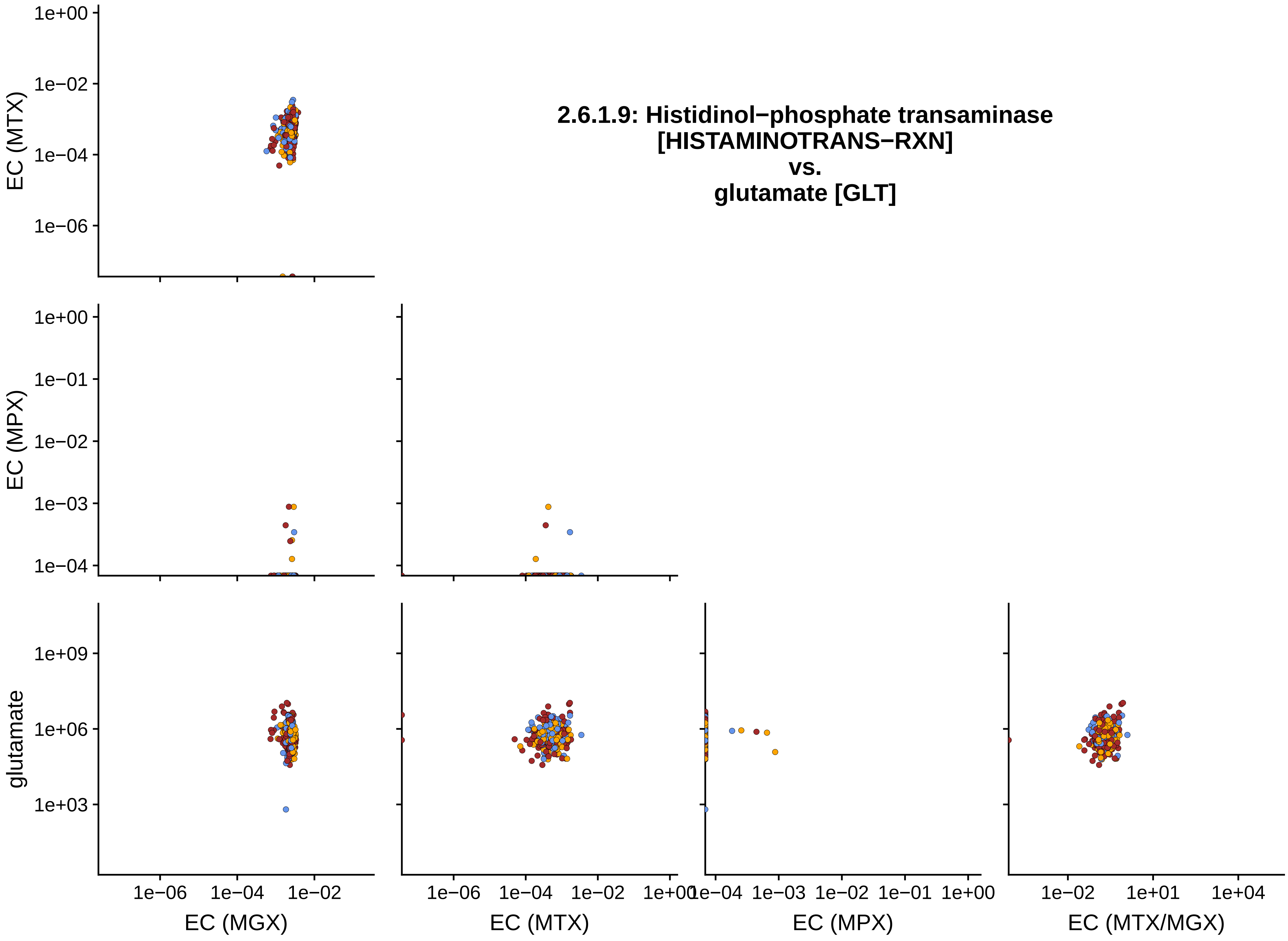

2.6.1.9: Histidinol−phosphate transaminase
[HISTAMINOTRANS−RXN]
vs.
glutamate [GLT]
EC (MTX)
EC (MPX)
glutamate
EC (MGX)
EC (MTX)
EC (MPX)
EC (MTX/MGX)
1e+00
1e−02
1e−04
1e−06
1e−01
1e−03
1e+09
1e+06
1e+03
1e+01
1e+04

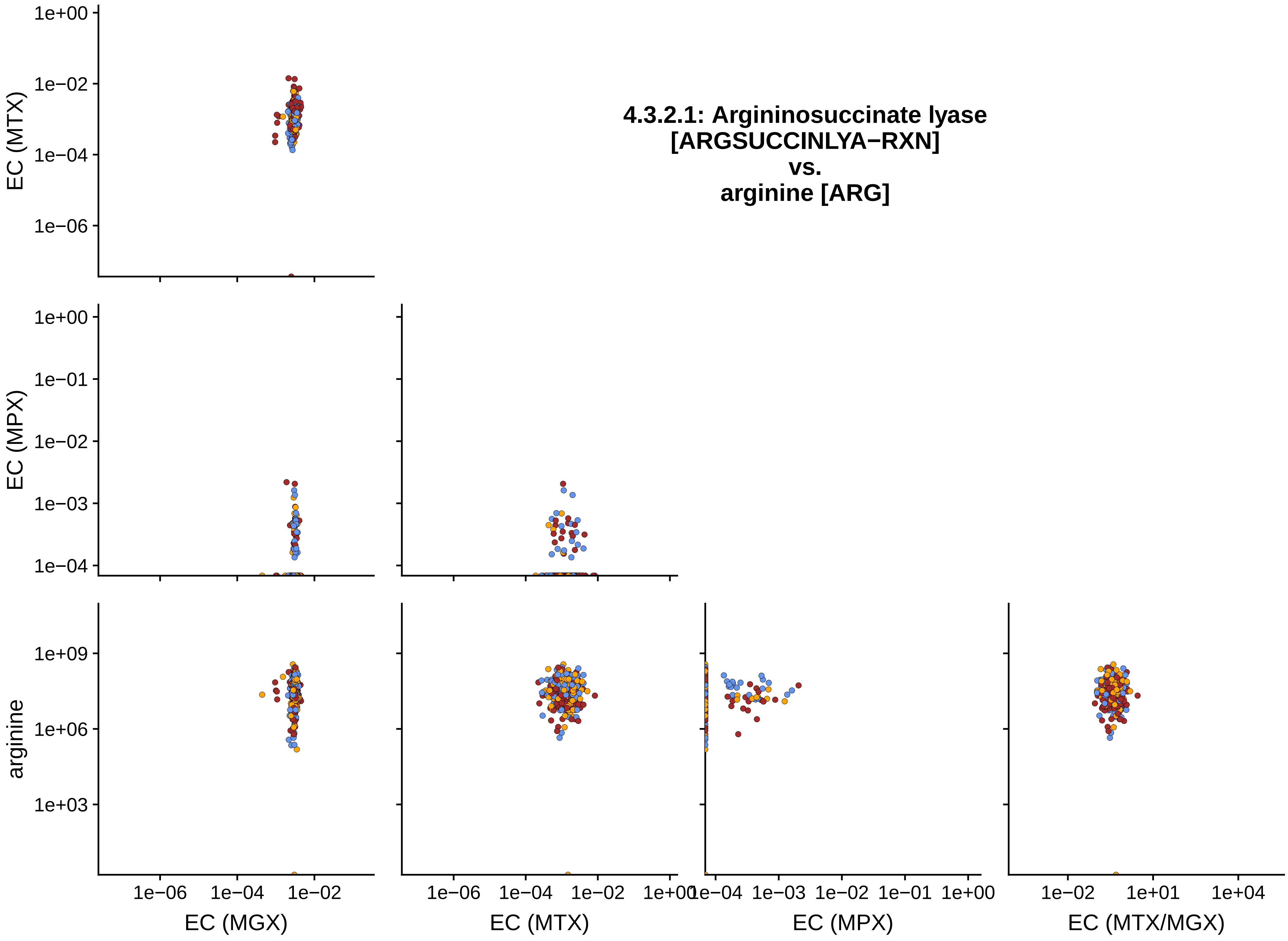
4.3.2.1: Argininosuccinate lyase
[ARGSUCCINLYA−RXN]
vs.
arginine [ARG]
EC (MTX)
EC (MPX)
arginine
EC (MGX)
EC (MTX)
EC (MPX)
EC (MTX/MGX)
1e+00
1e−02
1e−04
1e−06
1e−01
1e−03
1e+09
1e+06
1e+03
1e+01
1e+04

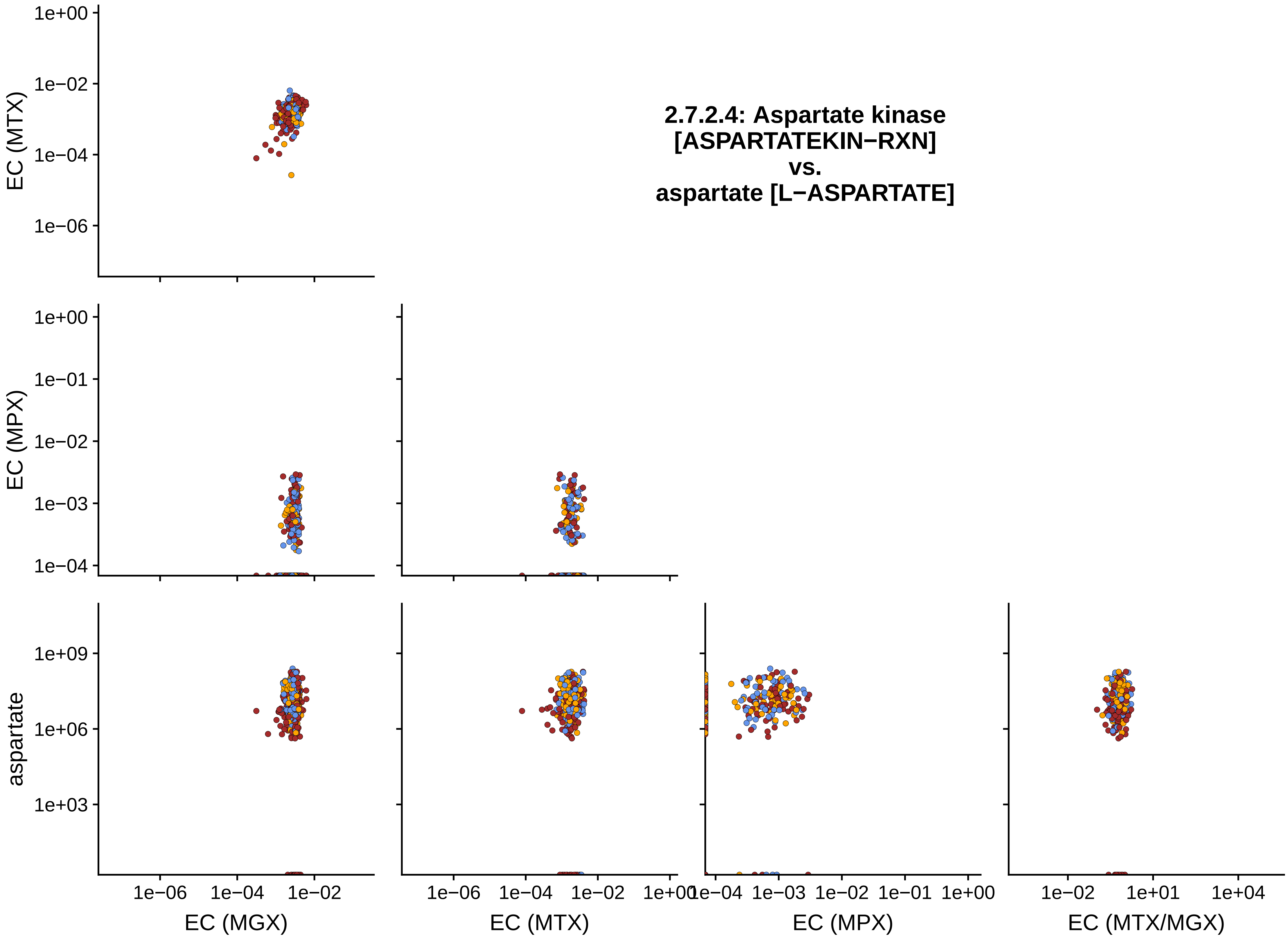

2.7.2.4: Aspartate kinase
[ASPARTATEKIN−RXN]
vs.
aspartate [L−ASPARTATE]
EC (MTX)
EC (MPX)
aspartate
EC (MGX)
EC (MTX)
EC (MPX)
EC (MTX/MGX)
1e+00
1e−02
1e−04
1e−06
1e−01
1e−03
1e+09
1e+06
1e+03
1e+01
1e+04

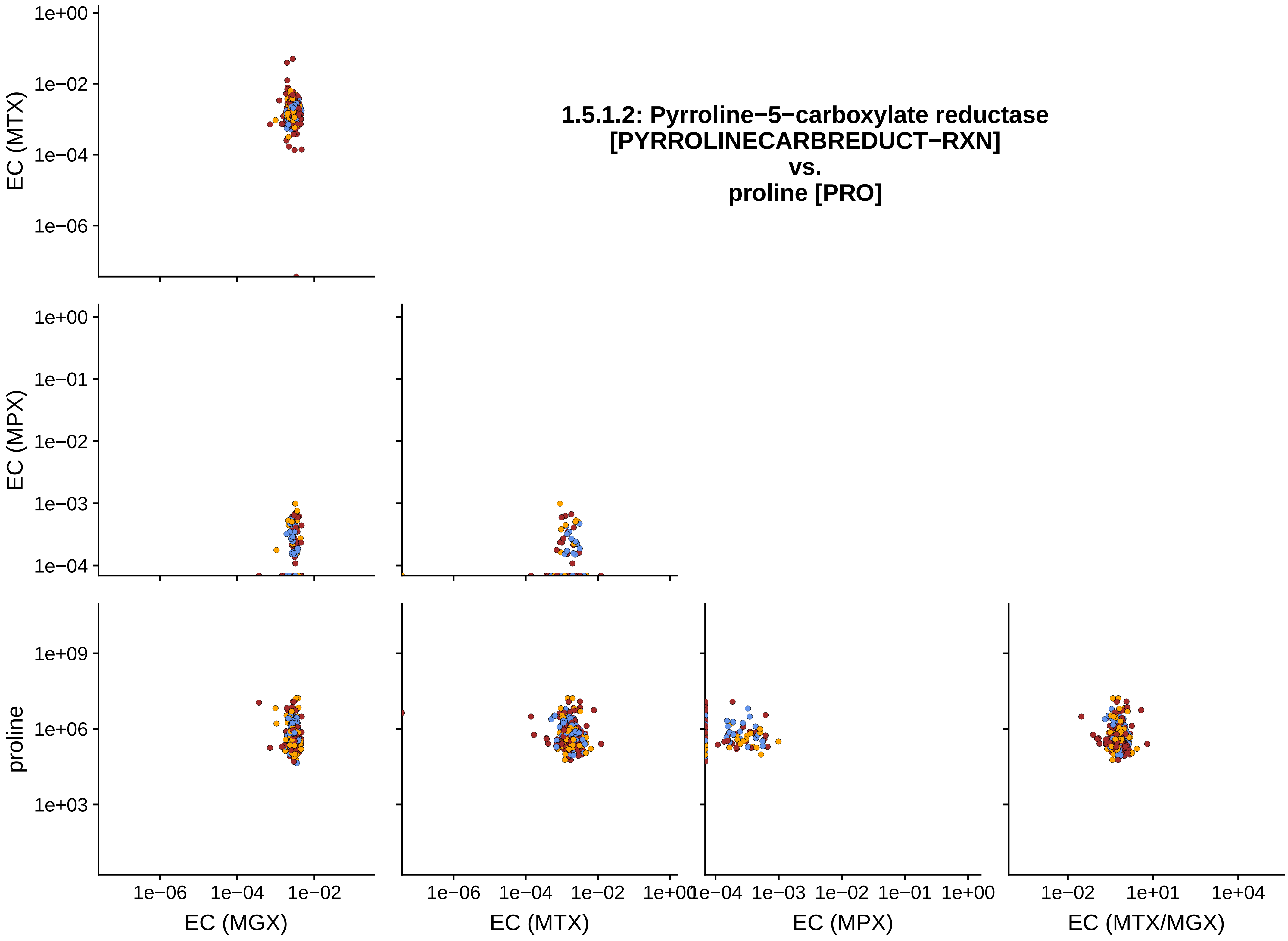

1.5.1.2: Pyrroline−5−carboxylate reductase
[PYRROLINECARBREDUCT−RXN]
vs.
proline [PRO]
EC (MTX)
EC (MPX)
proline
EC (MGX)
EC (MTX)
EC (MPX)
EC (MTX/MGX)
1e+00
1e−02
1e−04
1e−06
1e−01
1e−03
1e+09
1e+06
1e+03
1e+01
1e+04

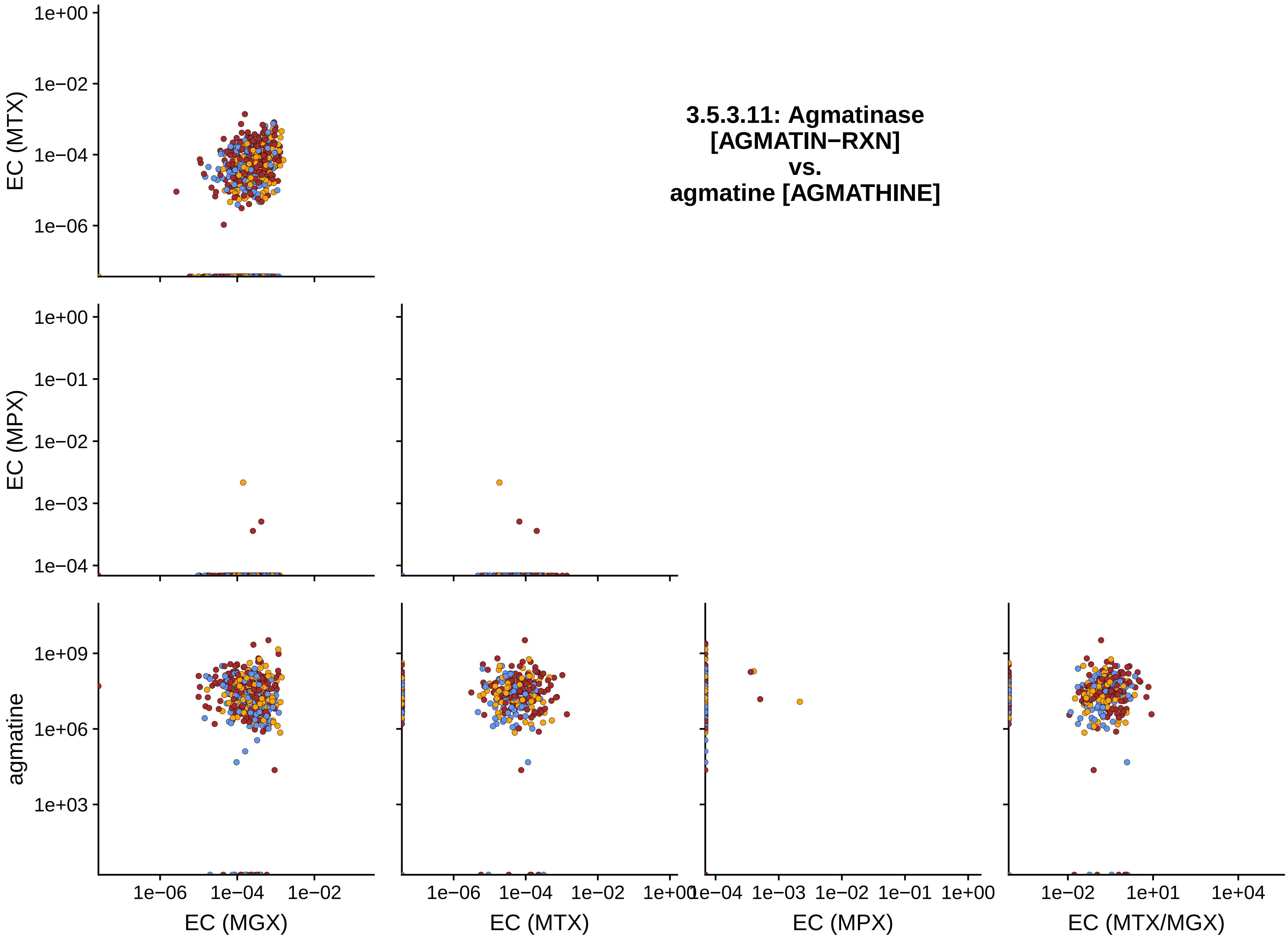

3.5.3.11: Agmatinase
[AGMATIN−RXN]
vs.
agmatine [AGMATHINE]
EC (MTX)
EC (MPX)
agmatine
EC (MGX)
EC (MTX)
EC (MPX)
EC (MTX/MGX)
1e+00
1e−02
1e−04
1e−06
1e+00
1e−01
1e−02
1e−03
1e−04
1e+09
1e+06
1e+03
1e−06
1e−04
1e−02
1e−06
1e−04
1e−02
1e+00
1e−04
1e−03
1e−02
1e−01
1e+00
1e−02
1e+01
1e+04

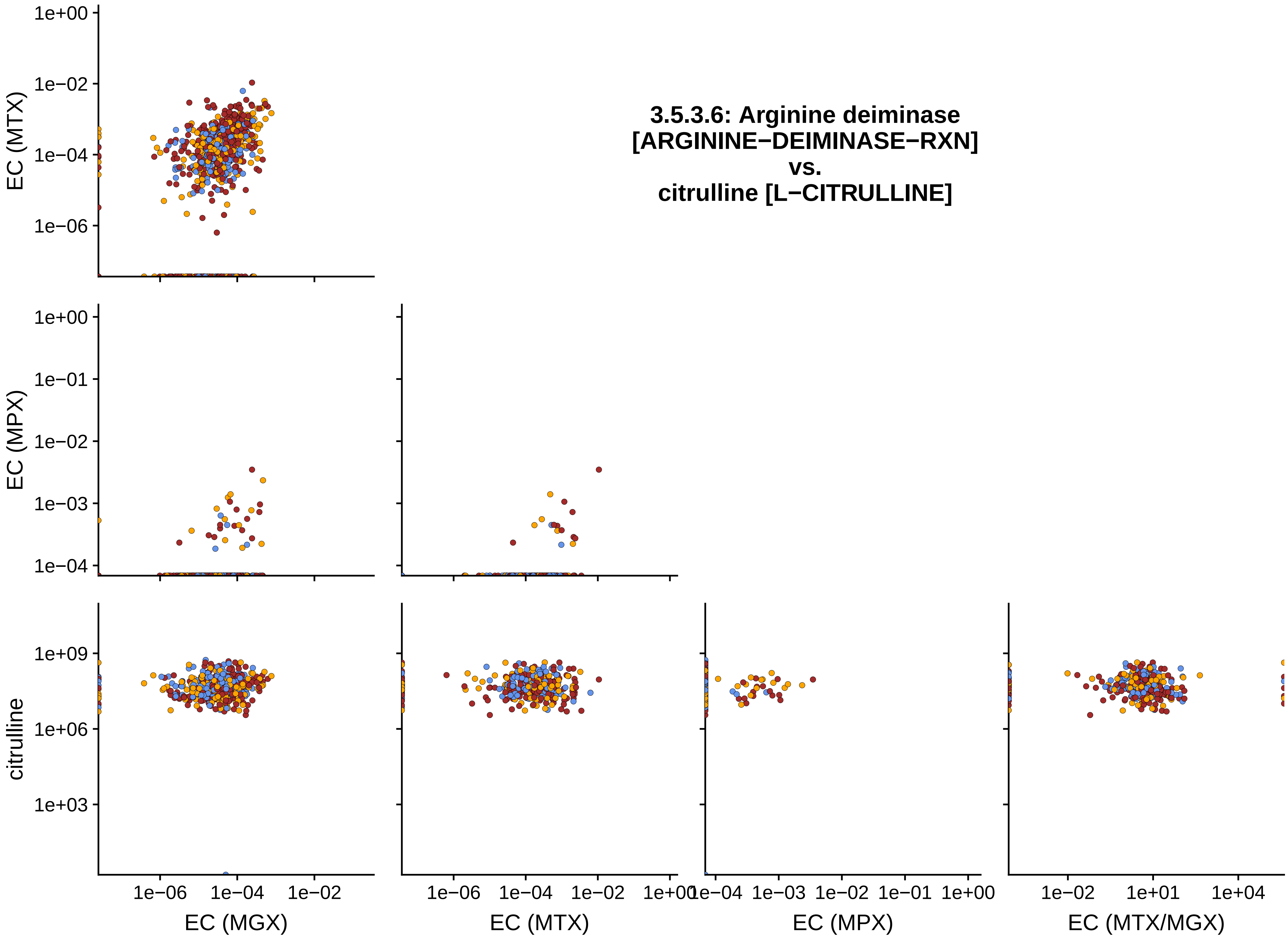

3.5.3.6: Arginine deiminase
[ARGININE−DEIMINASE−RXN]
vs.
citrulline [L−CITRULLINE]
EC (MTX)
EC (MPX)
citrulline
EC (MGX)
EC (MTX)
EC (MPX)
EC (MTX/MGX)
1e+00
1e−02
1e−04
1e−06
1e−01
1e−03
1e+09
1e+06
1e+03
1e+01
1e+04

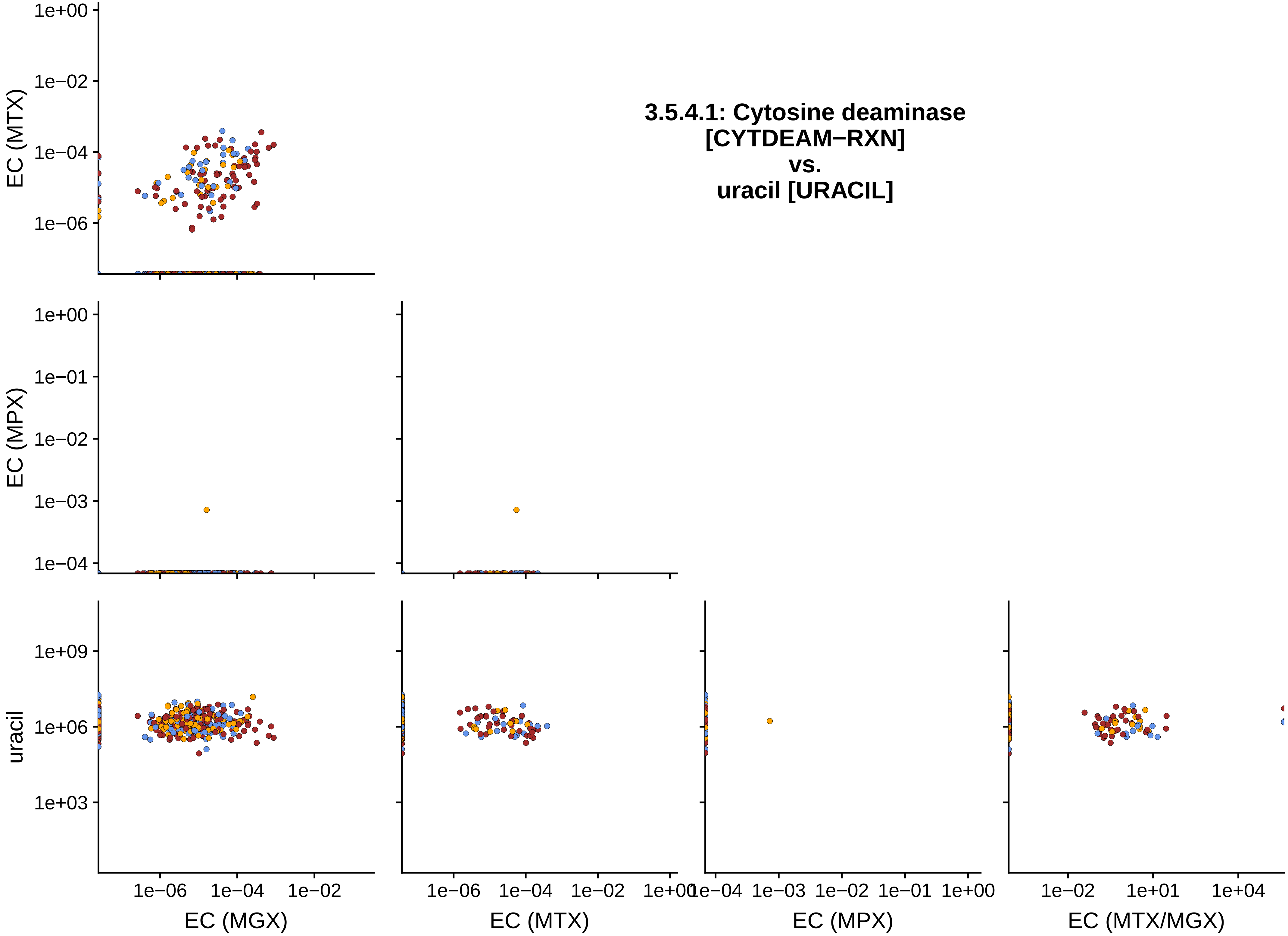

3.5.4.1: Cytosine deaminase
[CYTDEAM−RXN]
vs.
uracil [URACIL]
EC (MTX)
EC (MPX)
uracil
EC (MGX)
EC (MTX)
EC (MPX)
EC (MTX/MGX)
1e+00
1e−02
1e−04
1e−06
1e−01
1e−03
1e+09
1e+06
1e+03
1e+01
1e+04

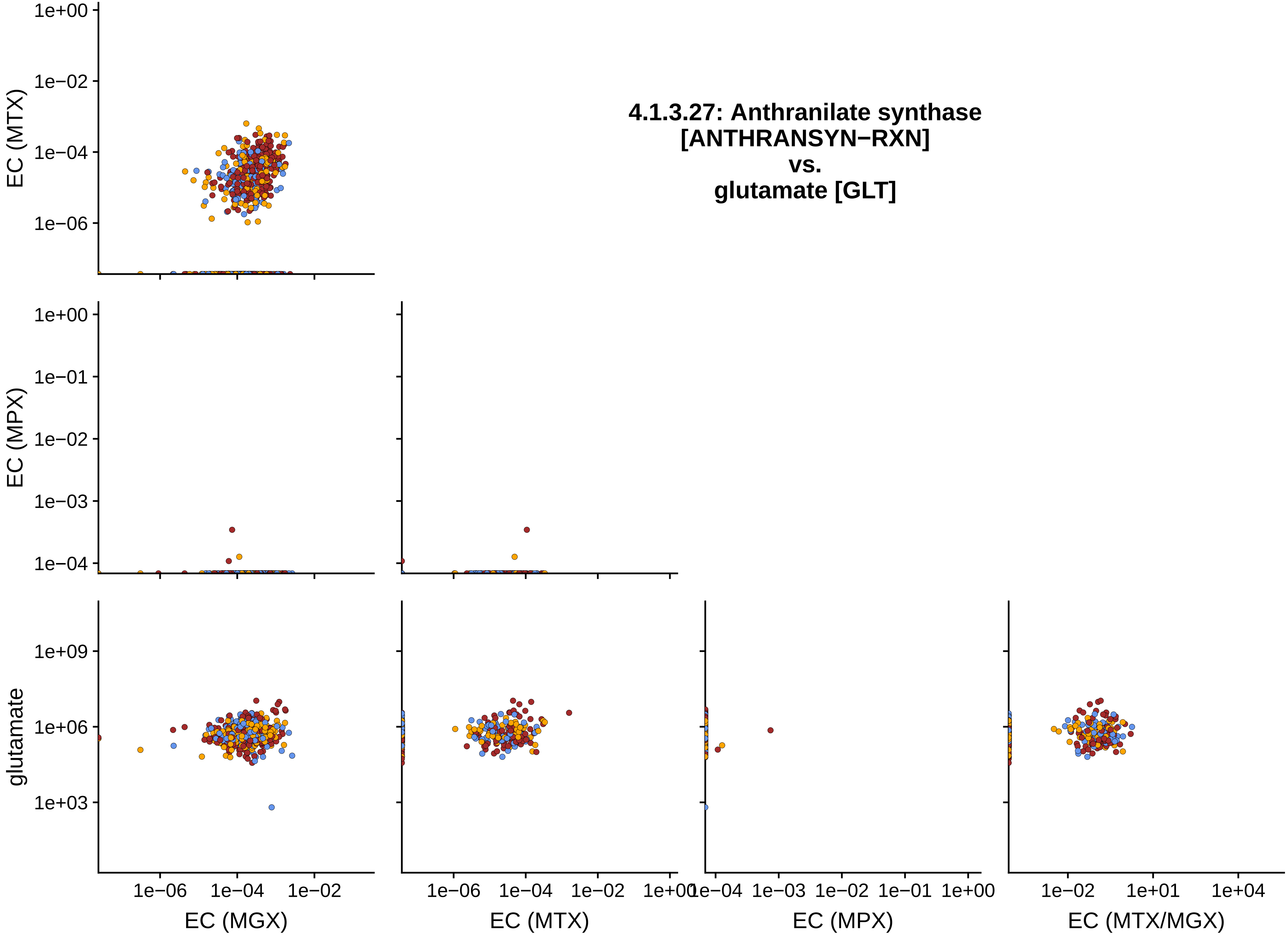

4.1.3.27: Anthranilate synthase
[ANTHRANSYN−RXN]
vs.
glutamate [GLT]
EC (MTX)
EC (MPX)
glutamate
EC (MGX)
EC (MTX)
EC (MPX)
EC (MTX/MGX)
1e+00
1e−02
1e−04
1e−06
1e−01
1e−03
1e+09
1e+06
1e+03
1e+01
1e+04

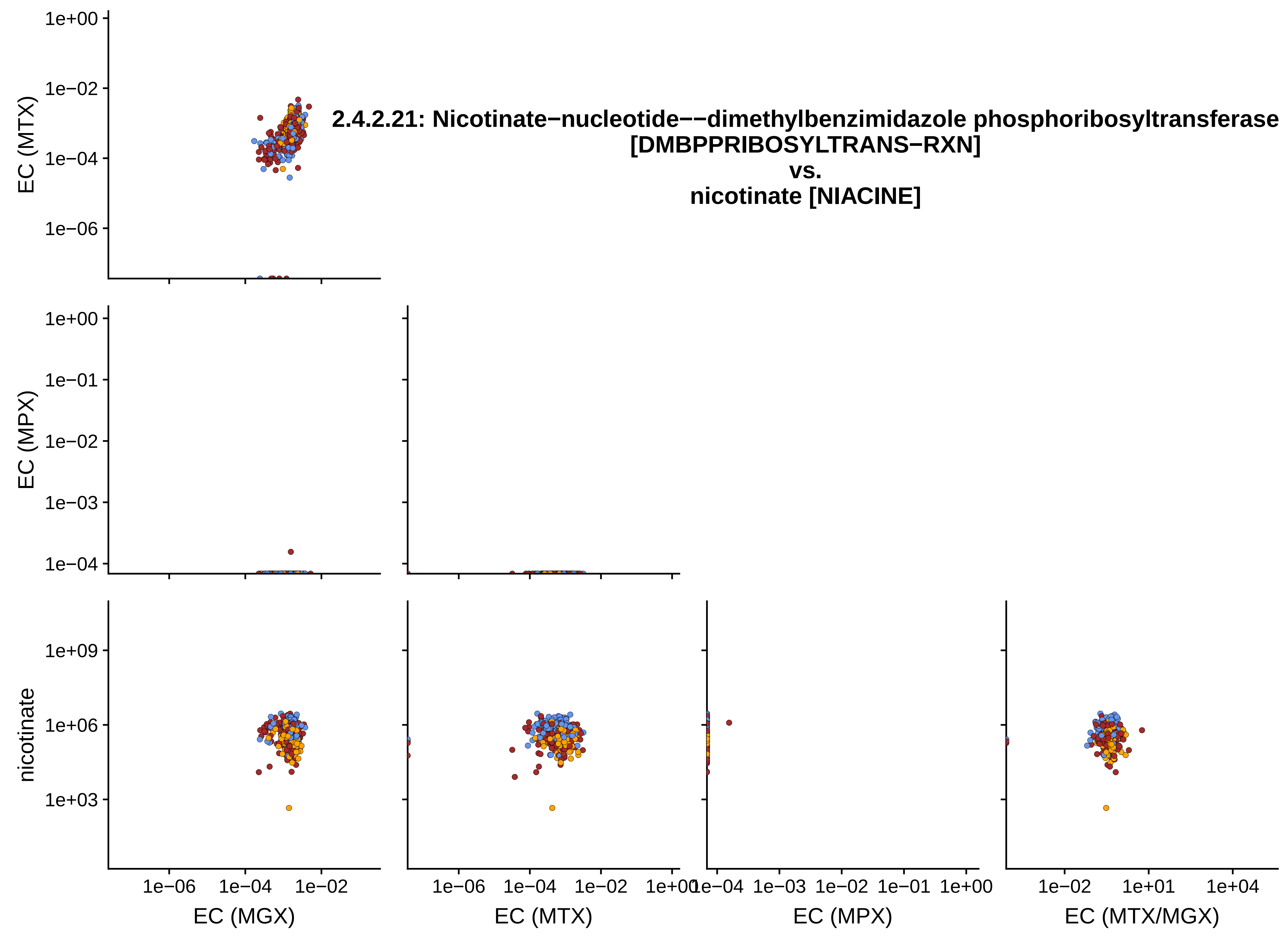

2.4.2.21: Nicotinate−nucleotide−−dimethylbenzimidazole phosphoribosyltransferase
[DMBPPRIBOSYLTRANS−RXN]
vs.
nicotinate [NIACINE]
EC (MTX)
EC (MPX)
nicotinate
EC (MGX)
EC (MTX)
EC (MPX)
EC (MTX/MGX)
1e+00
1e−02
1e−04
1e−06
1e−01
1e−03
1e+09
1e+06
1e+03
1e+01
1e+04

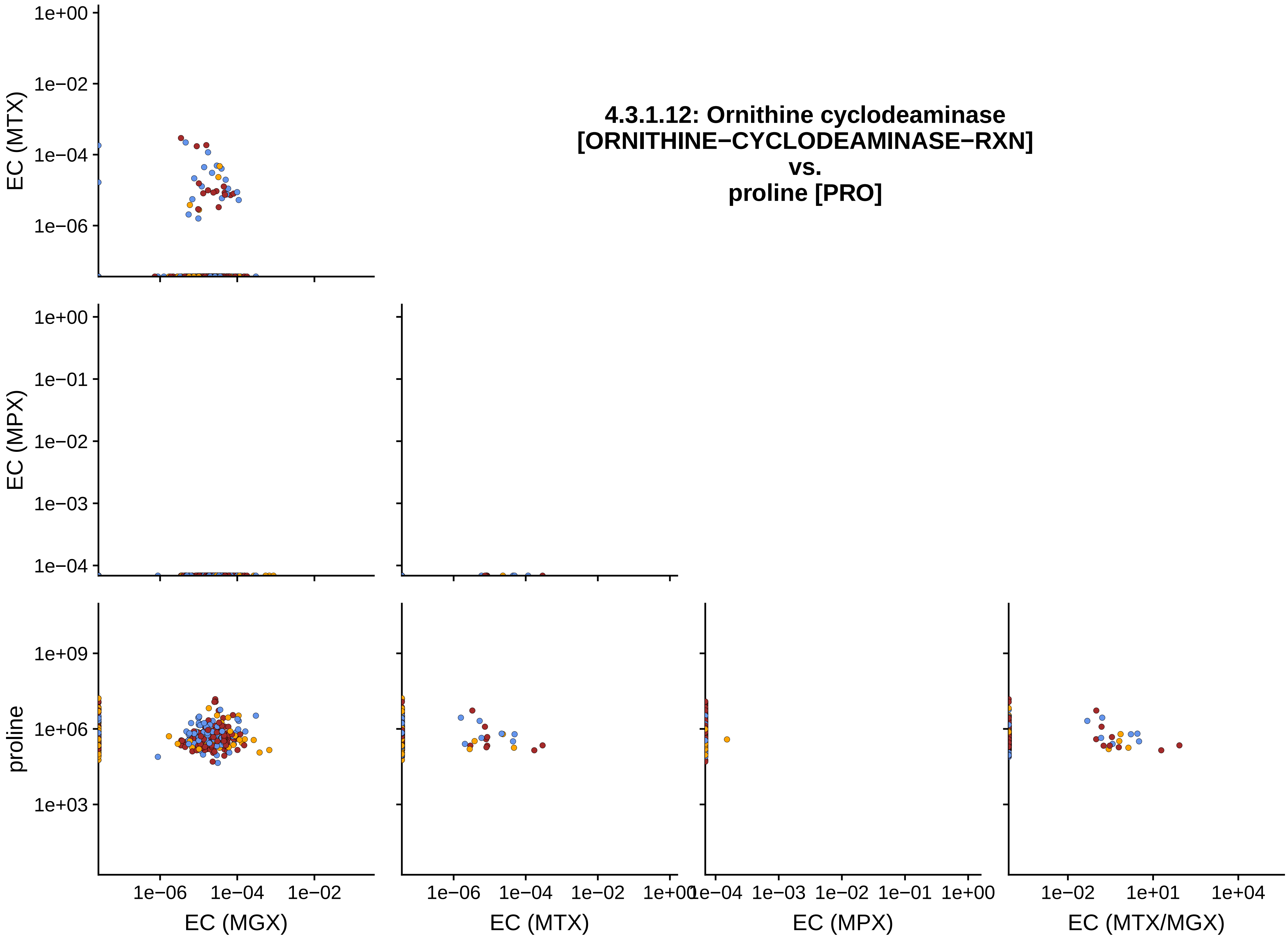

4.3.1.12: Ornithine cyclodeaminase
[ORNITHINE−CYCLODEAMINASE−RXN]
vs.
proline [PRO]
EC (MTX)
EC (MPX)
proline
EC (MGX)
EC (MTX)
EC (MPX)
EC (MTX/MGX)
1e+00
1e−02
1e−04
1e−06
1e−01
1e−03
1e+09
1e+06
1e+03
1e+01
1e+04

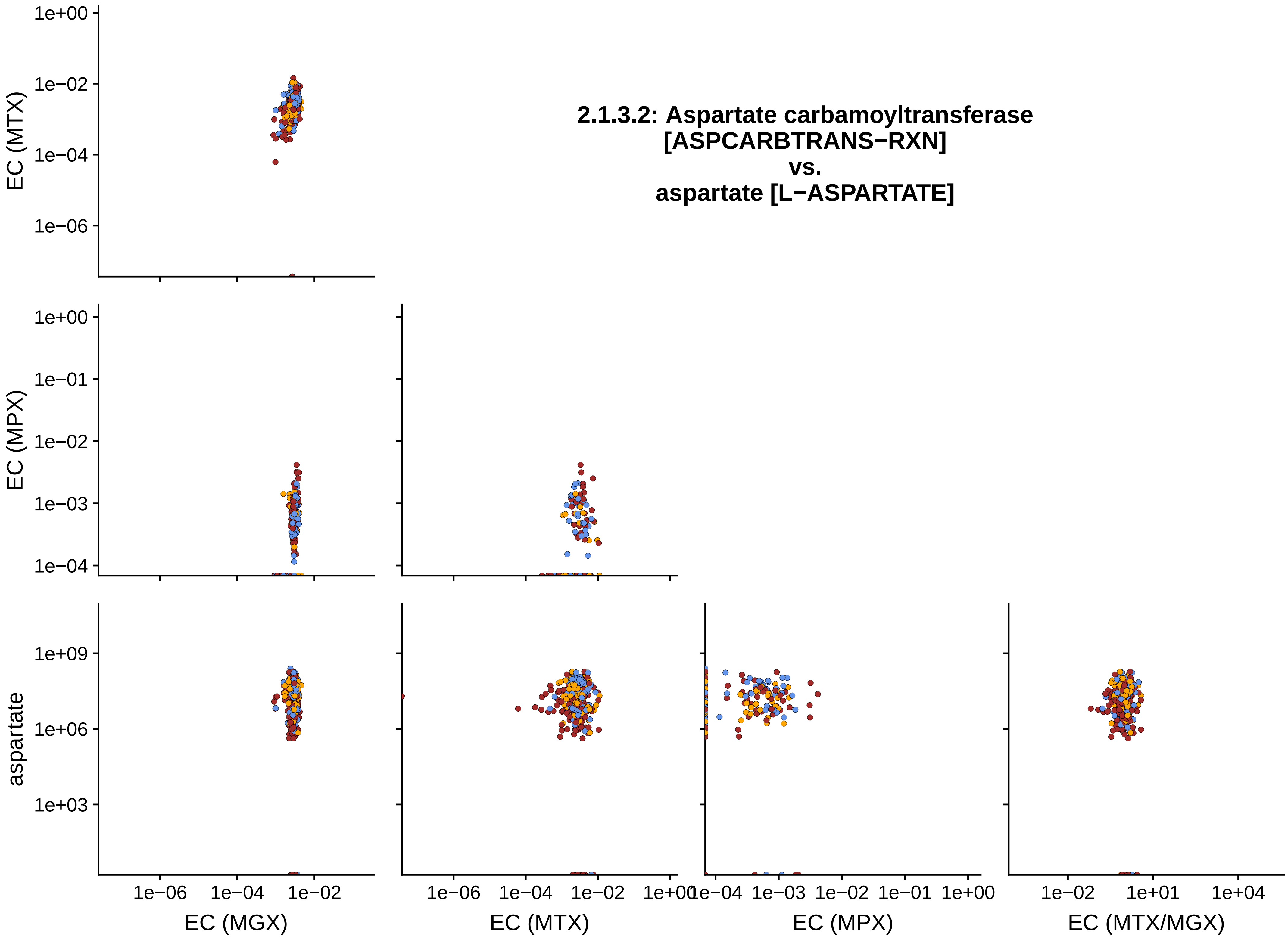
2.1.3.2: Aspartate carbamoyltransferase
[ASPCARBTRANS−RXN]
vs.
aspartate [L−ASPARTATE]
EC (MTX)
EC (MPX)
aspartate
EC (MGX)
EC (MTX)
EC (MPX)
EC (MTX/MGX)
1e+00
1e−02
1e−04
1e−06
1e−01
1e−03
1e+09
1e+06
1e+03
1e+01
1e+04

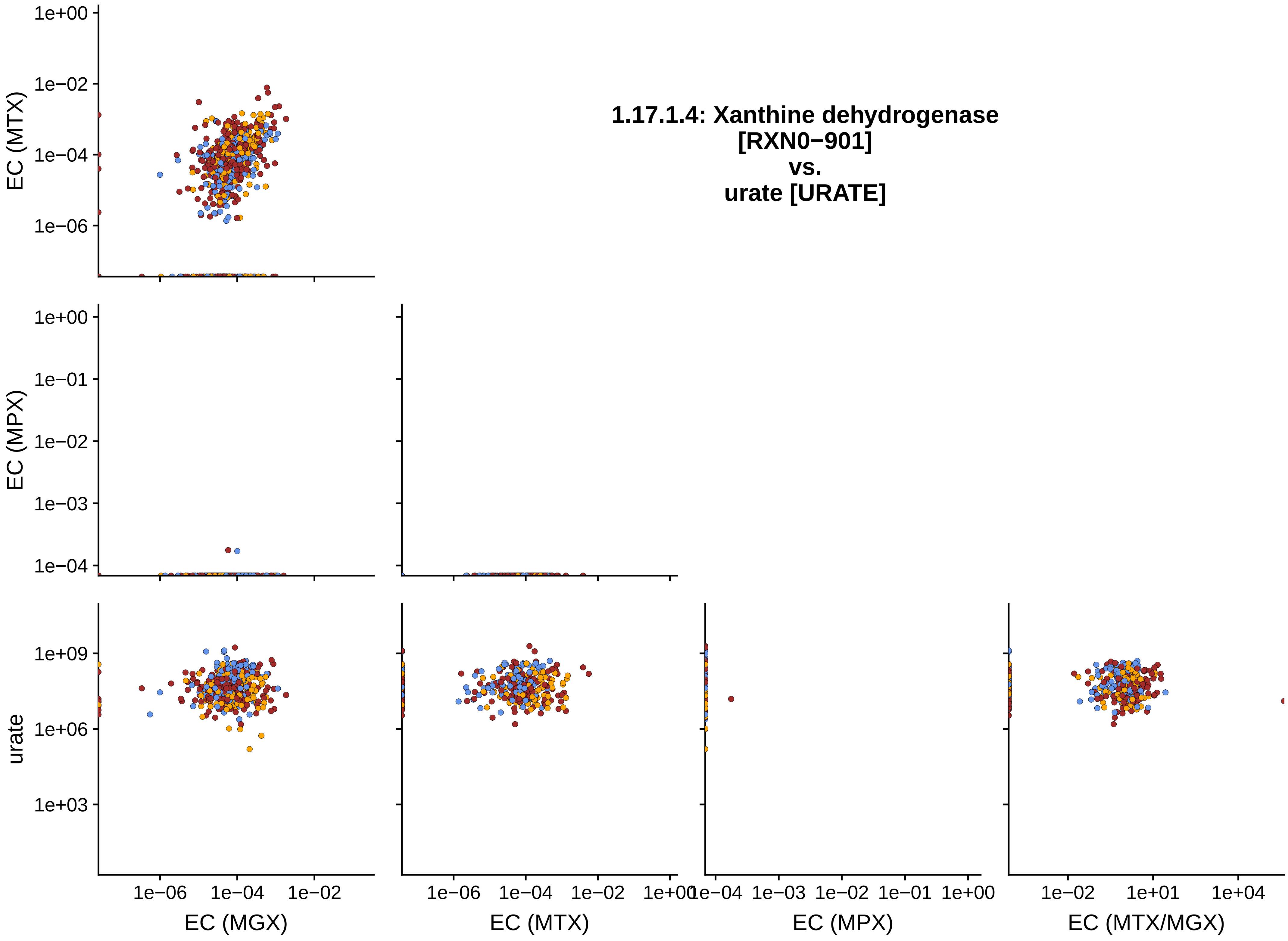

1.17.1.4: Xanthine dehydrogenase
[RXN0−901]
vs.
urate [URATE]
EC (MTX)
EC (MPX)
urate
EC (MGX)
EC (MTX)
EC (MPX)
EC (MTX/MGX)
1e+00
1e−02
1e−04
1e−06
1e−01
1e−03
1e+09
1e+06
1e+03
1e+04
1e+01

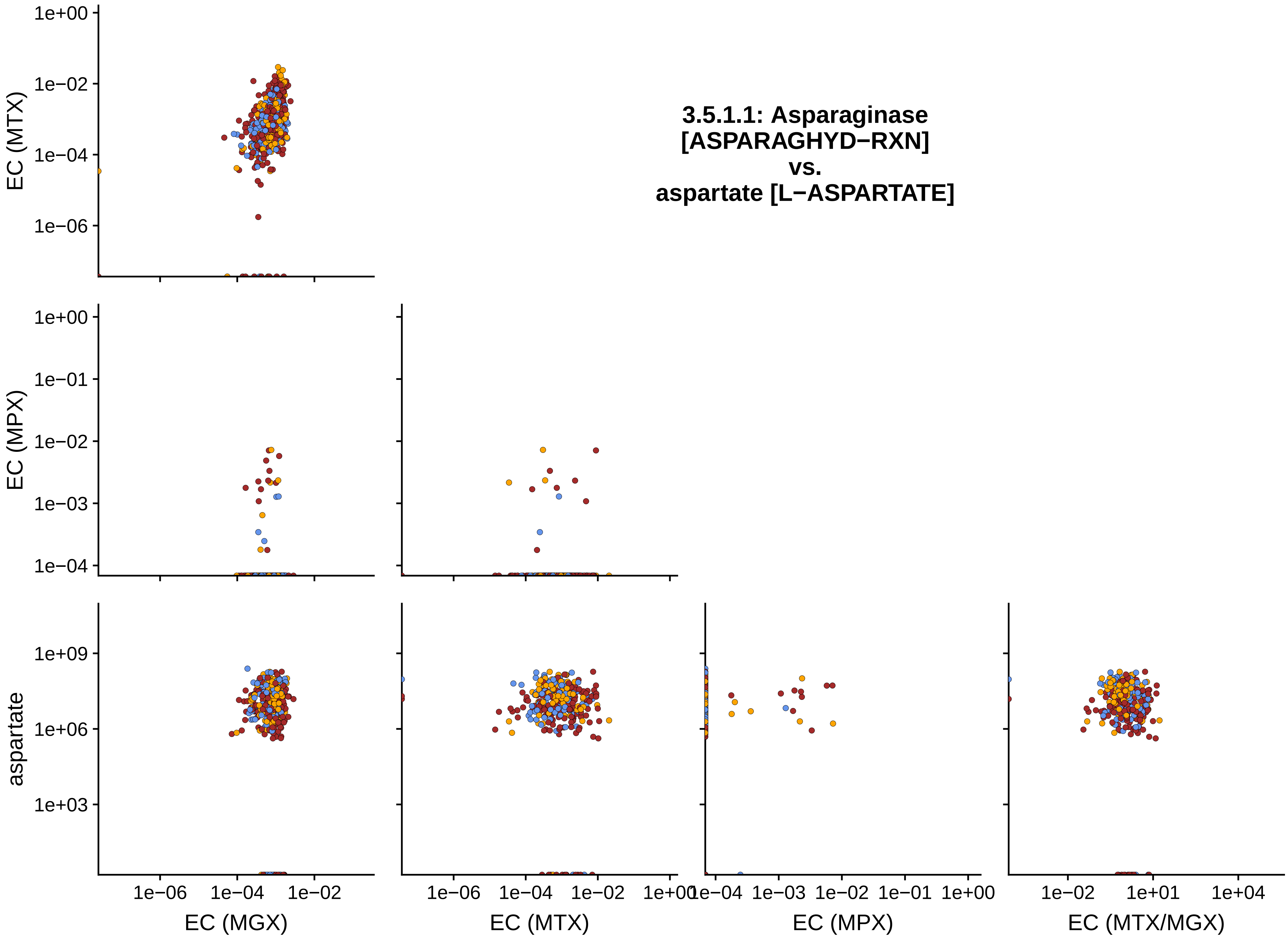

3.5.1.1: Asparaginase
[ASPARAGHYD−RXN]
vs.
aspartate [L−ASPARTATE]
EC (MTX)
EC (MPX)
aspartate
EC (MGX)
EC (MTX)
EC (MPX)
EC (MTX/MGX)
1e+00
1e−02
1e−04
1e−06
1e+00
1e−01
1e−02
1e−03
1e−04
1e+09
1e+06
1e+03
1e−06
1e−04
1e−02
1e−06
1e−04
1e−02
1e+00
1e−04
1e−03
1e−02
1e−01
1e+00
1e−02
1e+01
1e+04

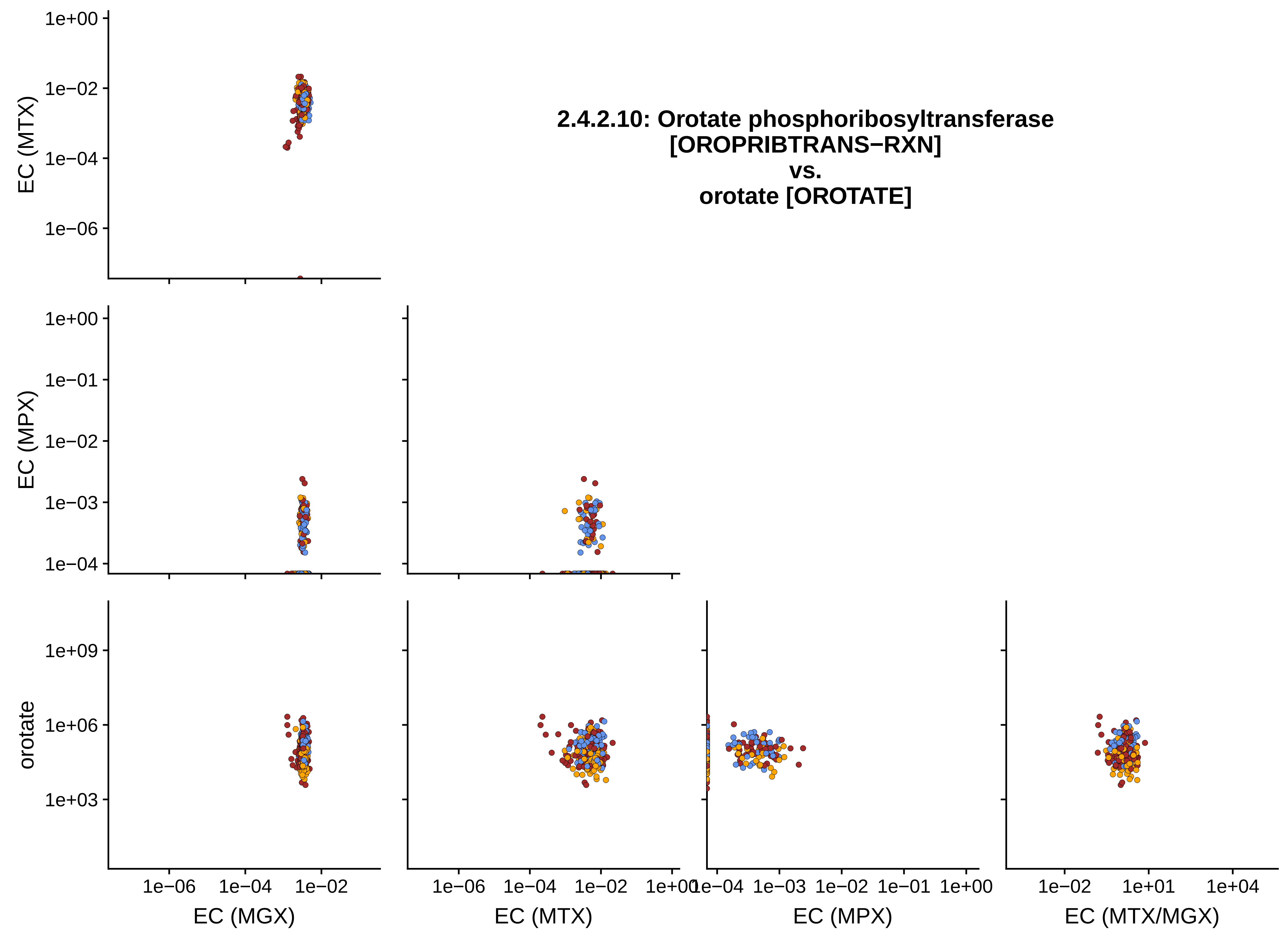
2.4.2.10: Orotate phosphoribosyltransferase
[OROPRIBTRANS−RXN]
vs.
orotate [OROTATE]
EC (MTX)
EC (MPX)
orotate
EC (MGX)
EC (MTX)
EC (MPX)
EC (MTX/MGX)
1e+00
1e−02
1e−04
1e−06
1e−01
1e−03
1e+09
1e+06
1e+03
1e+01
1e+04

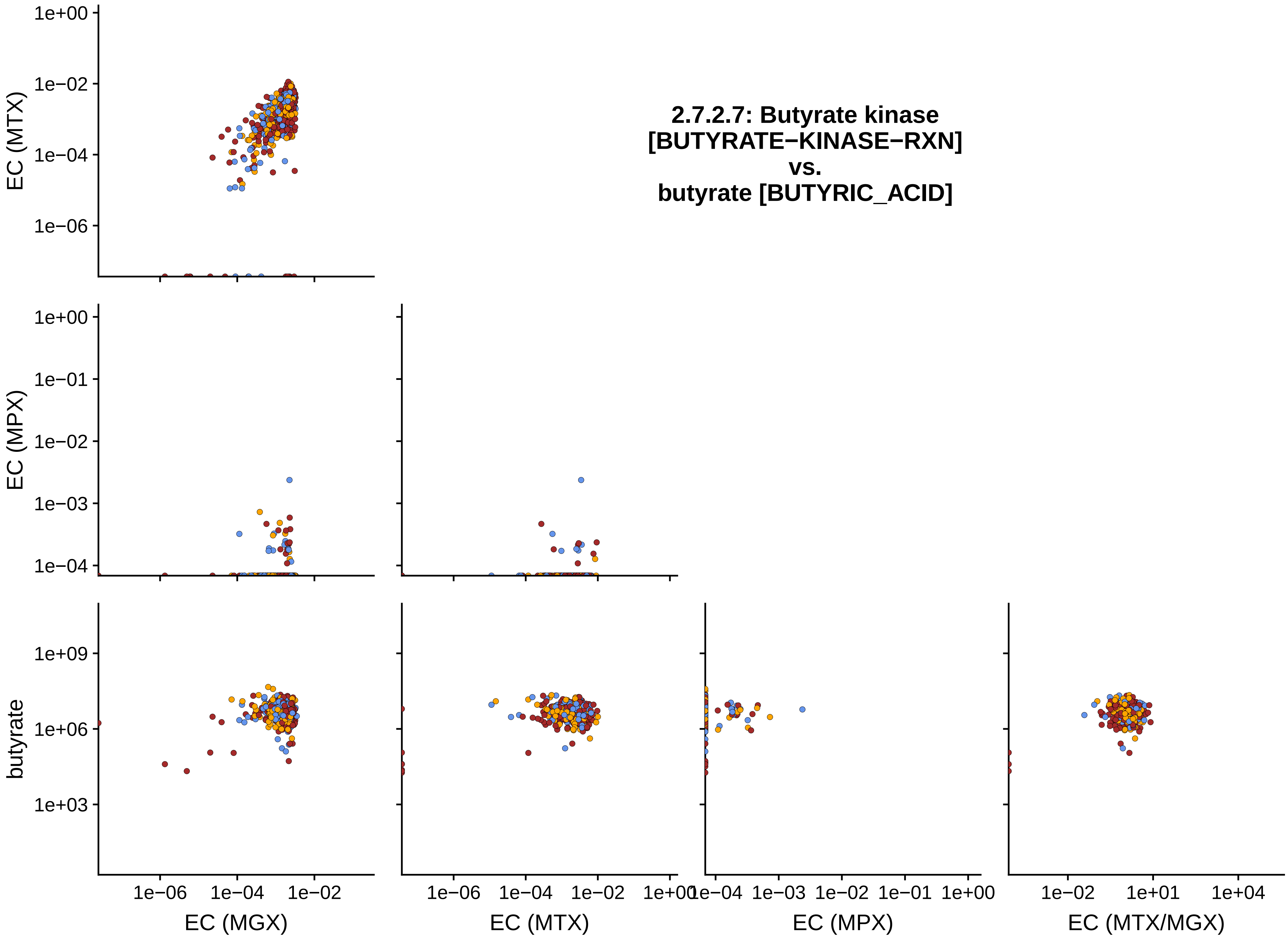
2.7.2.7: Butyrate kinase
[BUTYRATE−KINASE−RXN]
vs.
butyrate [BUTYRIC_ACID]
EC (MTX)
EC (MPX)
butyrate
EC (MGX)
EC (MTX)
EC (MPX)
EC (MTX/MGX)
1e+00
1e−02
1e−04
1e−06
1e−01
1e−03
1e+09
1e+06
1e+03
1e+01
1e+04

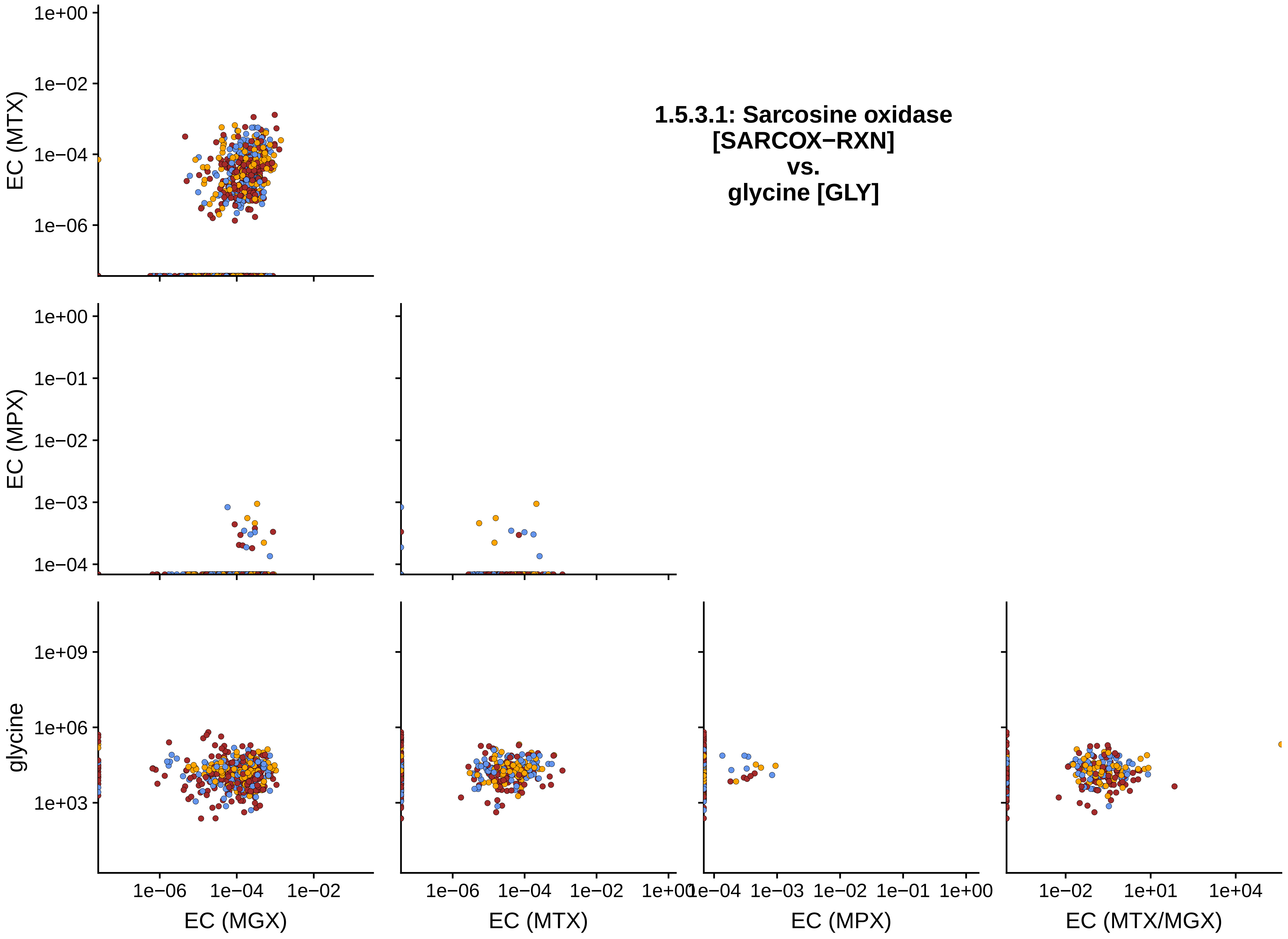
1.5.3.1: Sarcosine oxidase
[SARCOX−RXN]
vs.
glycine [GLY]
EC (MTX)
EC (MPX)
glycine
EC (MGX)
EC (MTX)
EC (MPX)
EC (MTX/MGX)
1e+00
1e−02
1e−04
1e−06
1e−01
1e−03
1e+09
1e+06
1e+03
1e+01
1e+04

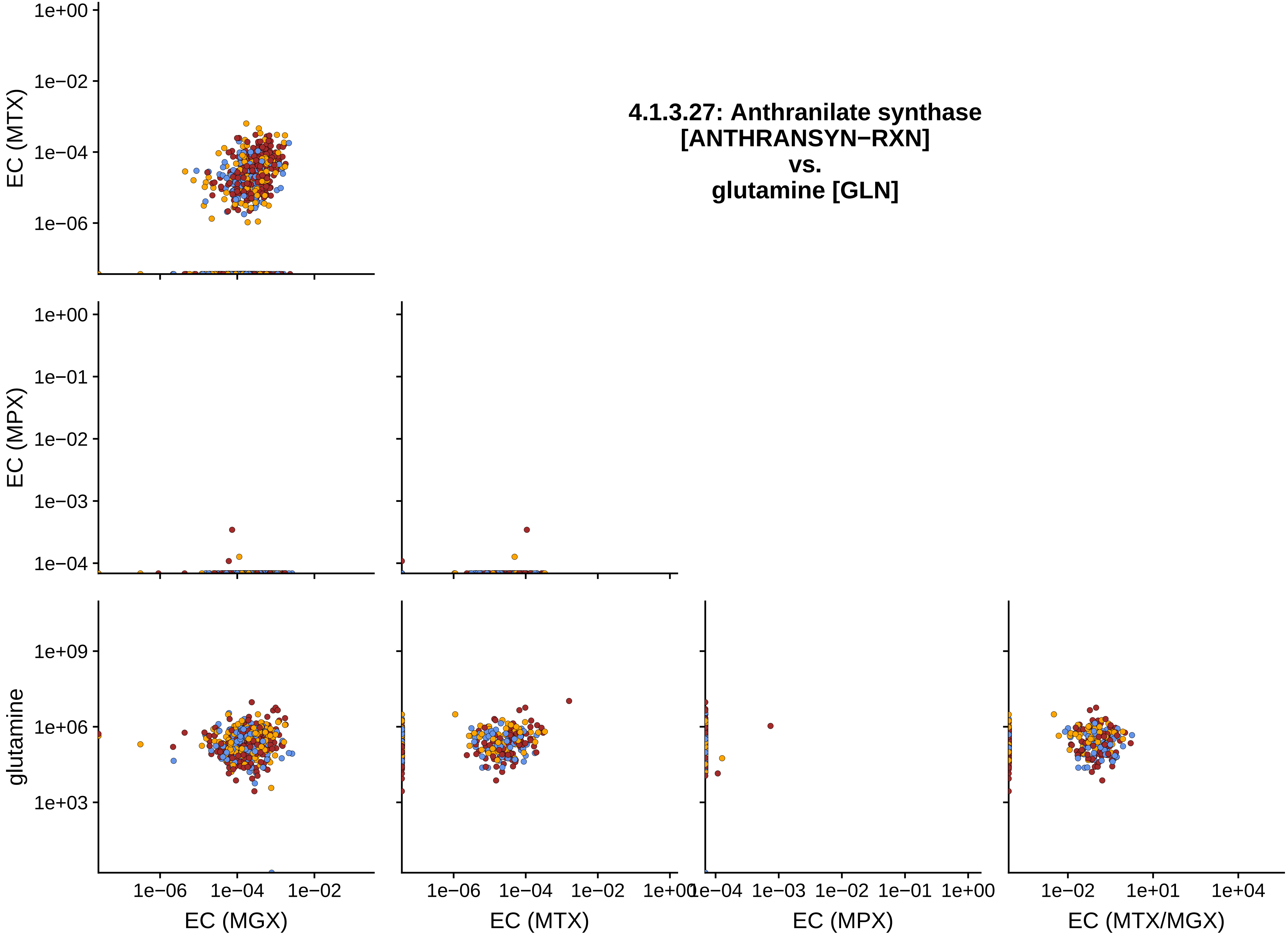

4.1.3.27: Anthranilate synthase
[ANTHRANSYN−RXN]
vs.
glutamine [GLN]
EC (MTX)
EC (MPX)
glutamine
EC (MGX)
EC (MTX)
EC (MPX)
EC (MTX/MGX)
1e+00
1e−02
1e−04
1e−06
1e−01
1e−03
1e+09
1e+06
1e+03
1e+01
1e+04

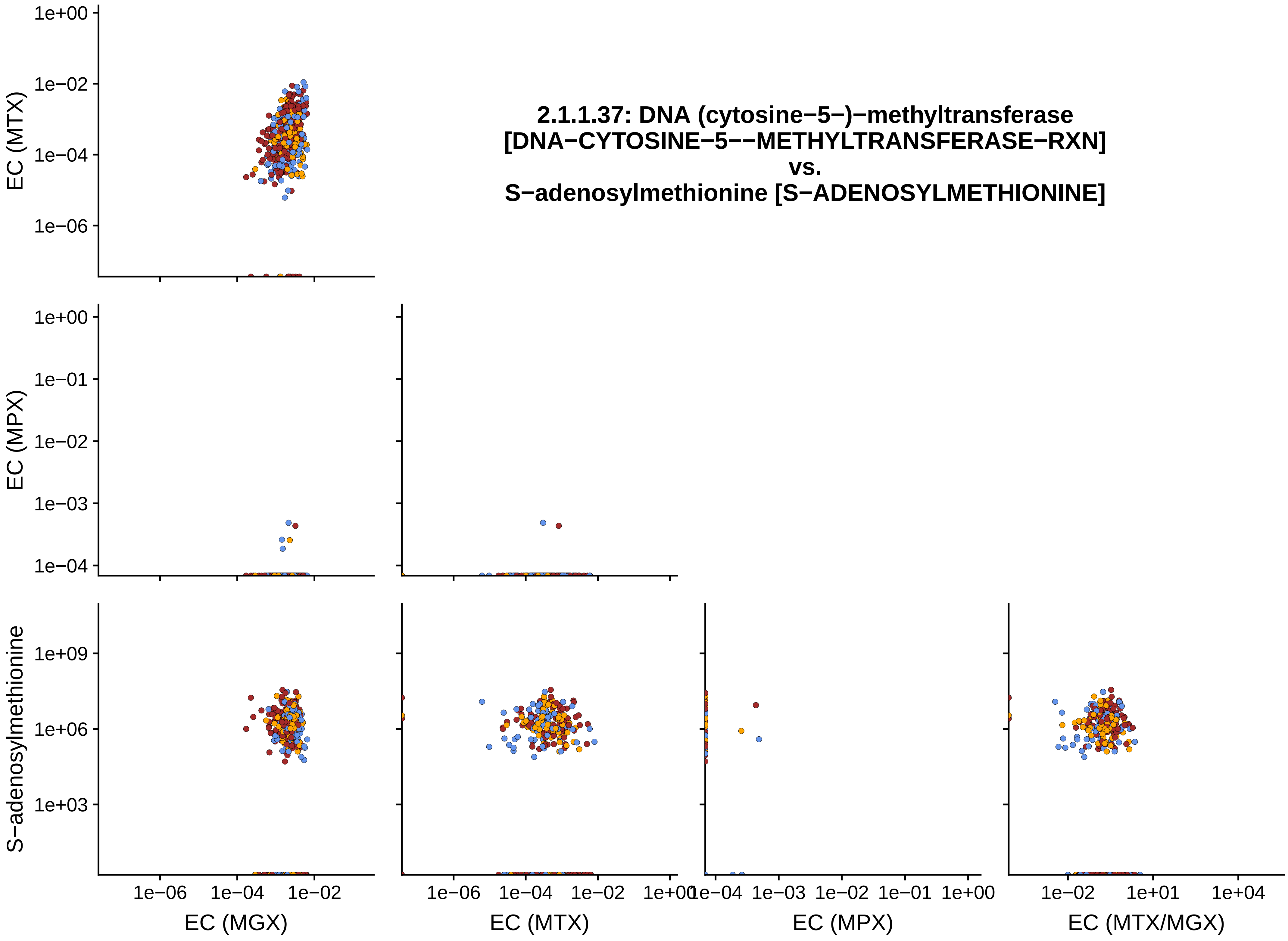

2.1.1.37: DNA (cytosine−5−)−methyltransferase
[DNA−CYTOSINE−5−−METHYLTRANSFERASE−RXN]
vs.
S−adenosylmethionine [S−ADENOSYLMETHIONINE]
EC (MTX)
EC (MPX)
S−adenosylmethionine
EC (MGX)
EC (MTX)
EC (MPX)
EC (MTX/MGX)
1e+00
1e−02
1e−04
1e−06
1e−01
1e−03
1e+09
1e+06
1e+03
1e+01
1e+04

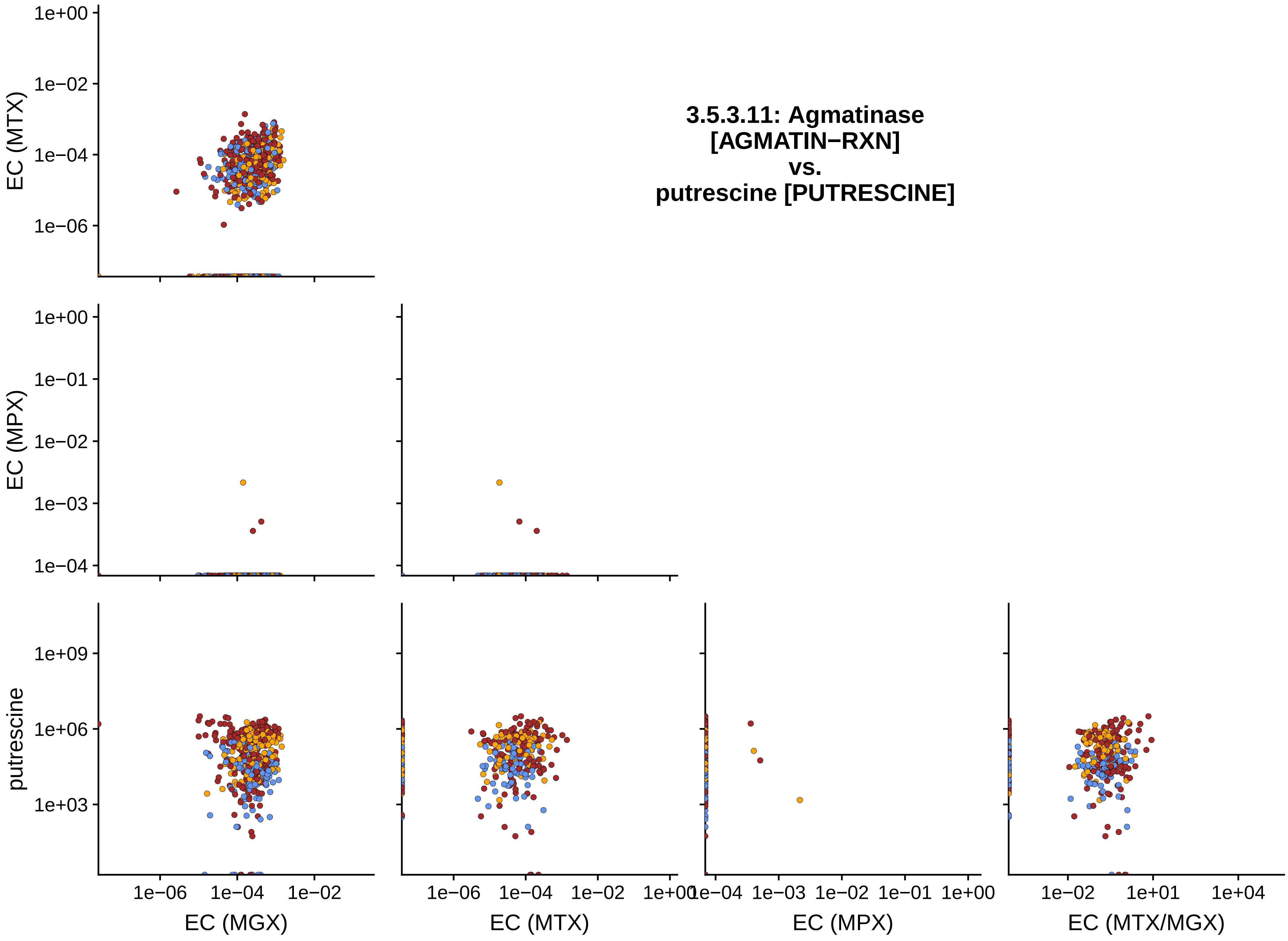

3.5.3.11: Agmatinase
[AGMATIN−RXN]
vs.
putrescine [PUTRESCINE]
EC (MTX)
EC (MPX)
putrescine
EC (MGX)
EC (MTX)
EC (MPX)
EC (MTX/MGX)
1e+00
1e−02
1e−04
1e−06
1e−01
1e−03
1e+09
1e+06
1e+03
1e+01
1e+04

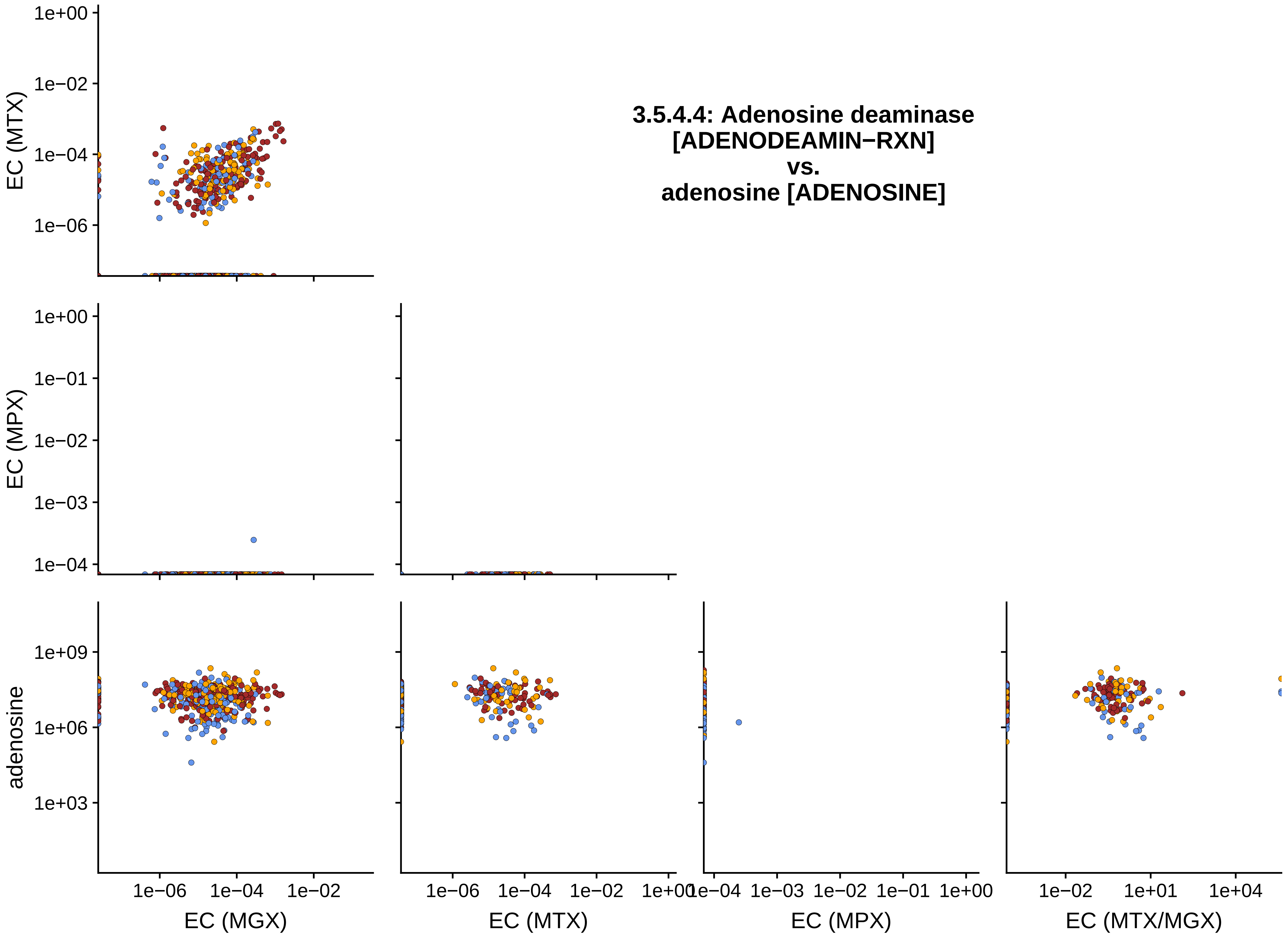

3.5.4.4: Adenosine deaminase
[ADENODEAMIN−RXN]
vs.
adenosine [ADENOSINE]
EC (MTX)
EC (MPX)
adenosine
EC (MGX)
EC (MTX)
EC (MPX)
EC (MTX/MGX)
1e+00
1e−02
1e−04
1e−06
1e−01
1e−03
1e+09
1e+06
1e+03
1e+01
1e+04

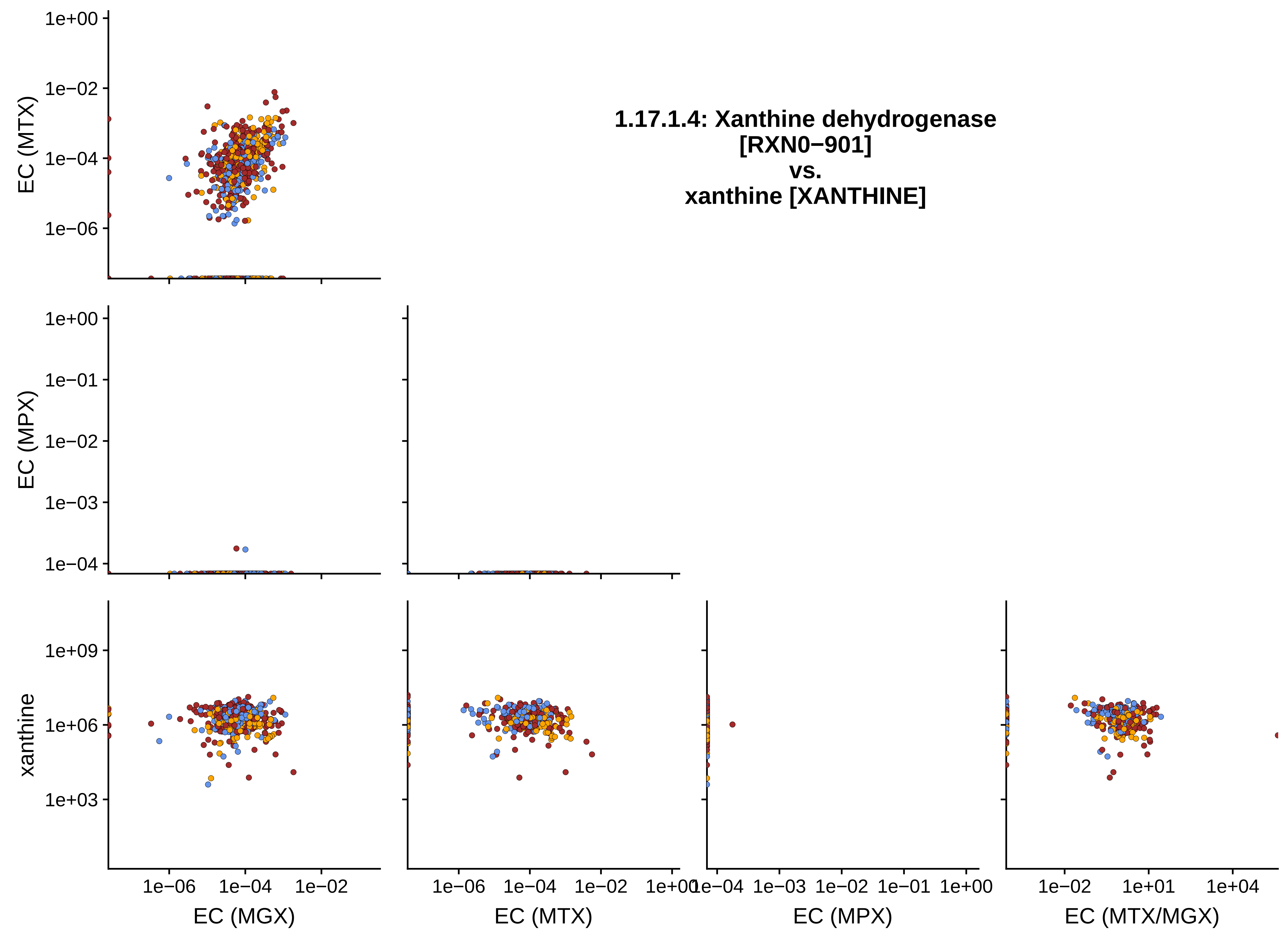

1.17.1.4: Xanthine dehydrogenase
[RXN0−901]
vs.
xanthine [XANTHINE]
EC (MTX)
EC (MPX)
xanthine
EC (MGX)
EC (MTX)
EC (MPX)
EC (MTX/MGX)
1e+00
1e−02
1e−04
1e−06
1e−01
1e−03
1e+09
1e+06
1e+03
1e+01
1e+04

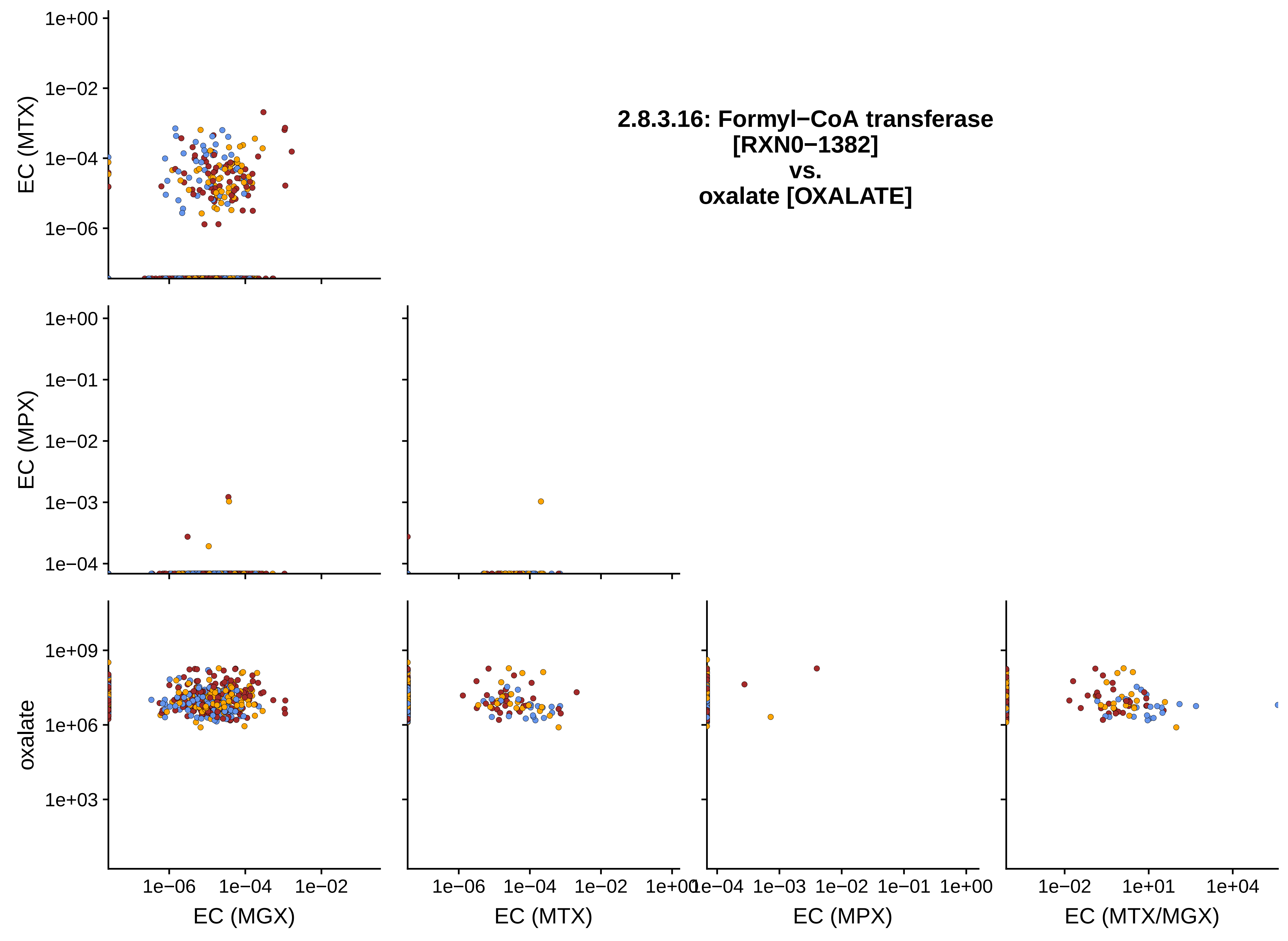

2.8.3.16: Formyl−CoA transferase
[RXN0−1382]
vs.
oxalate [OXALATE]
EC (MTX)
EC (MPX)
oxalate
EC (MGX)
EC (MTX)
EC (MPX)
EC (MTX/MGX)
1e+00
1e−02
1e−04
1e−06
1e−01
1e−03
1e+09
1e+06
1e+03
1e+01
1e+04

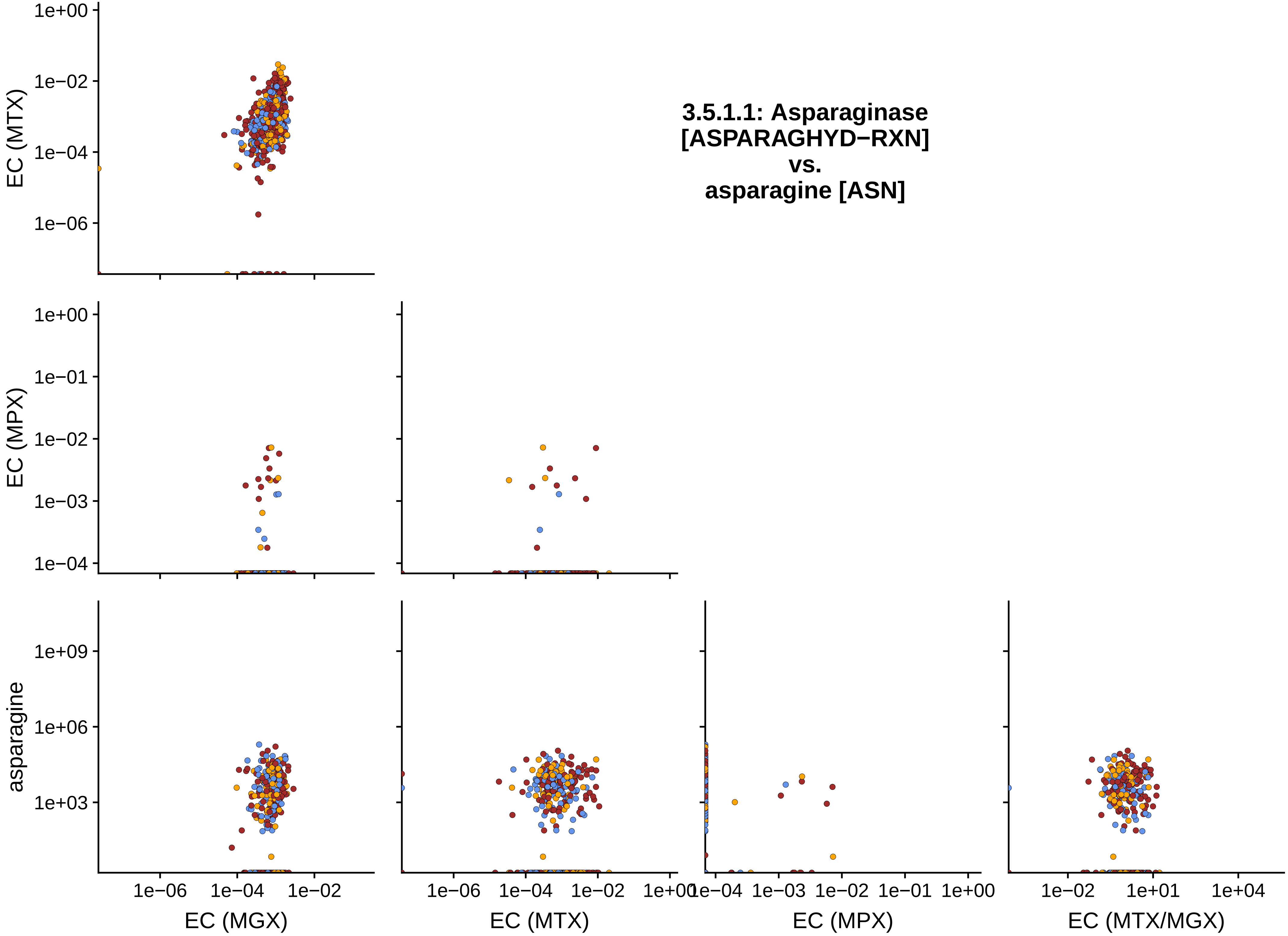

3.5.1.1: Asparaginase
[ASPARAGHYD−RXN]
vs.
asparagine [ASN]
EC (MTX)
EC (MPX)
asparagine
EC (MGX)
EC (MTX)
EC (MPX)
EC (MTX/MGX)
1e+00
1e−02
1e−04
1e−06
1e−01
1e−03
1e+09
1e+06
1e+03
1e+01
1e+04

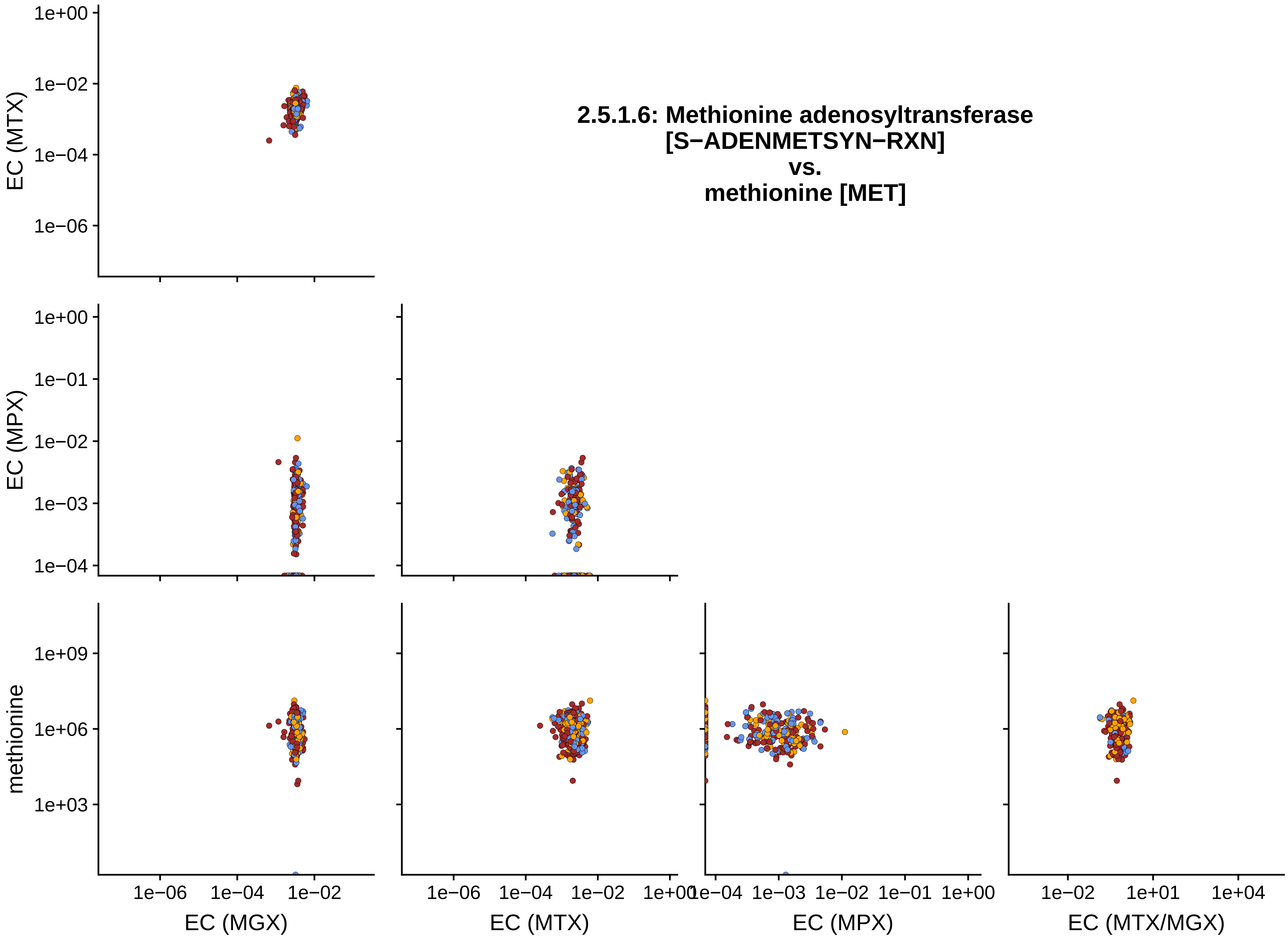

2.5.1.6: Methionine adenosyltransferase
[S−ADENMETSYN−RXN]
vs.
methionine [MET]
EC (MTX)
EC (MPX)
methionine
EC (MGX)
EC (MTX)
EC (MPX)
EC (MTX/MGX)
1e+00
1e−02
1e−04
1e−06
1e−01
1e−03
1e+09
1e+06
1e+03
1e+01
1e+04

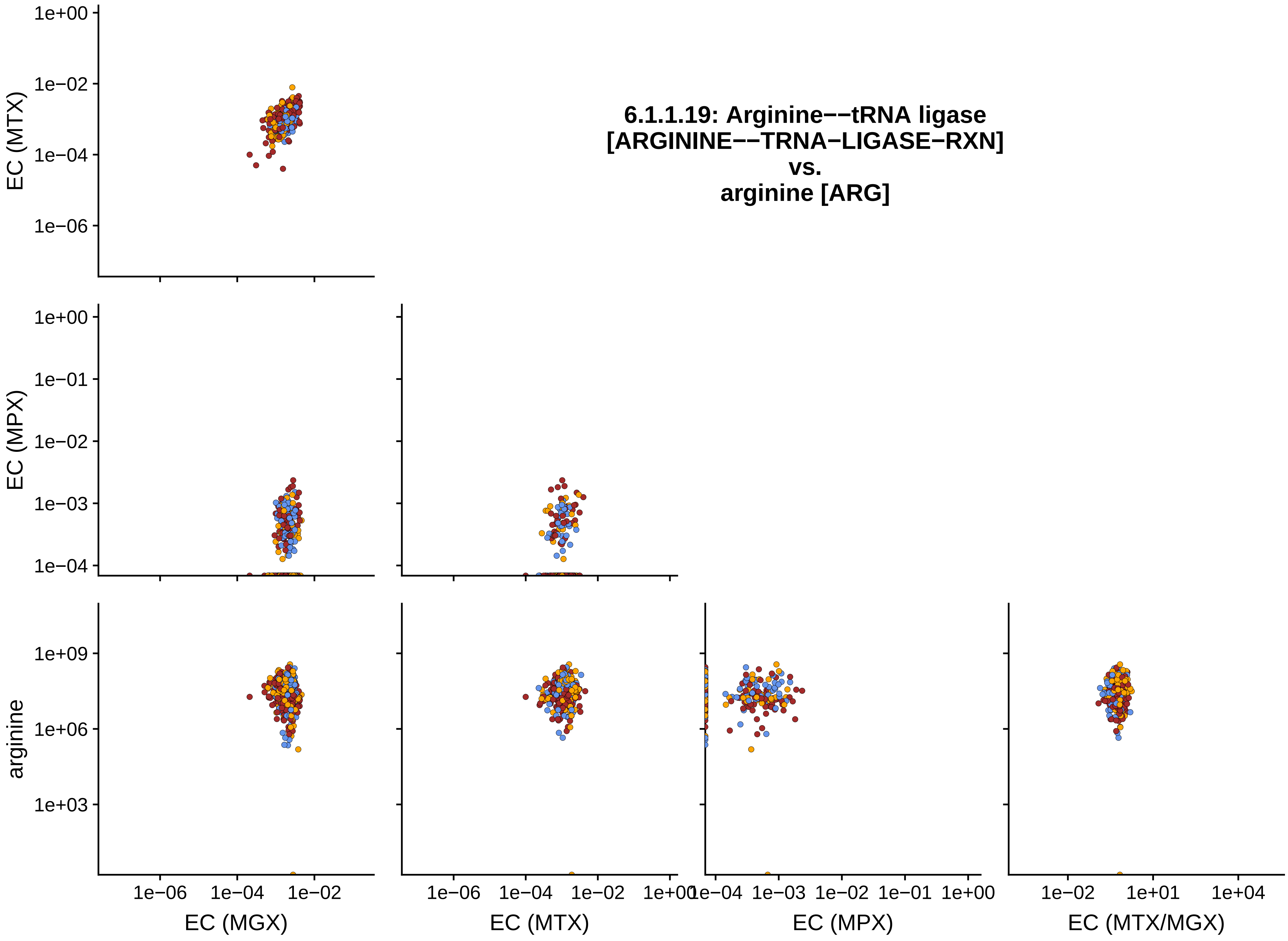

6.1.1.19: Arginine−−tRNA ligase
[ARGININE−−TRNA−LIGASE−RXN]
vs.
arginine [ARG]
EC (MTX)
EC (MPX)
arginine
EC (MGX)
EC (MTX)
EC (MPX)
EC (MTX/MGX)
1e+00
1e−02
1e−04
1e−06
1e+00
1e−01
1e−02
1e−03
1e−04
1e+09
1e+06
1e+03
1e−06
1e−04
1e−02
1e−06
1e−04
1e−02
1e+00
1e−04
1e−03
1e−02
1e−01
1e+00
1e−02
1e+01
1e+04

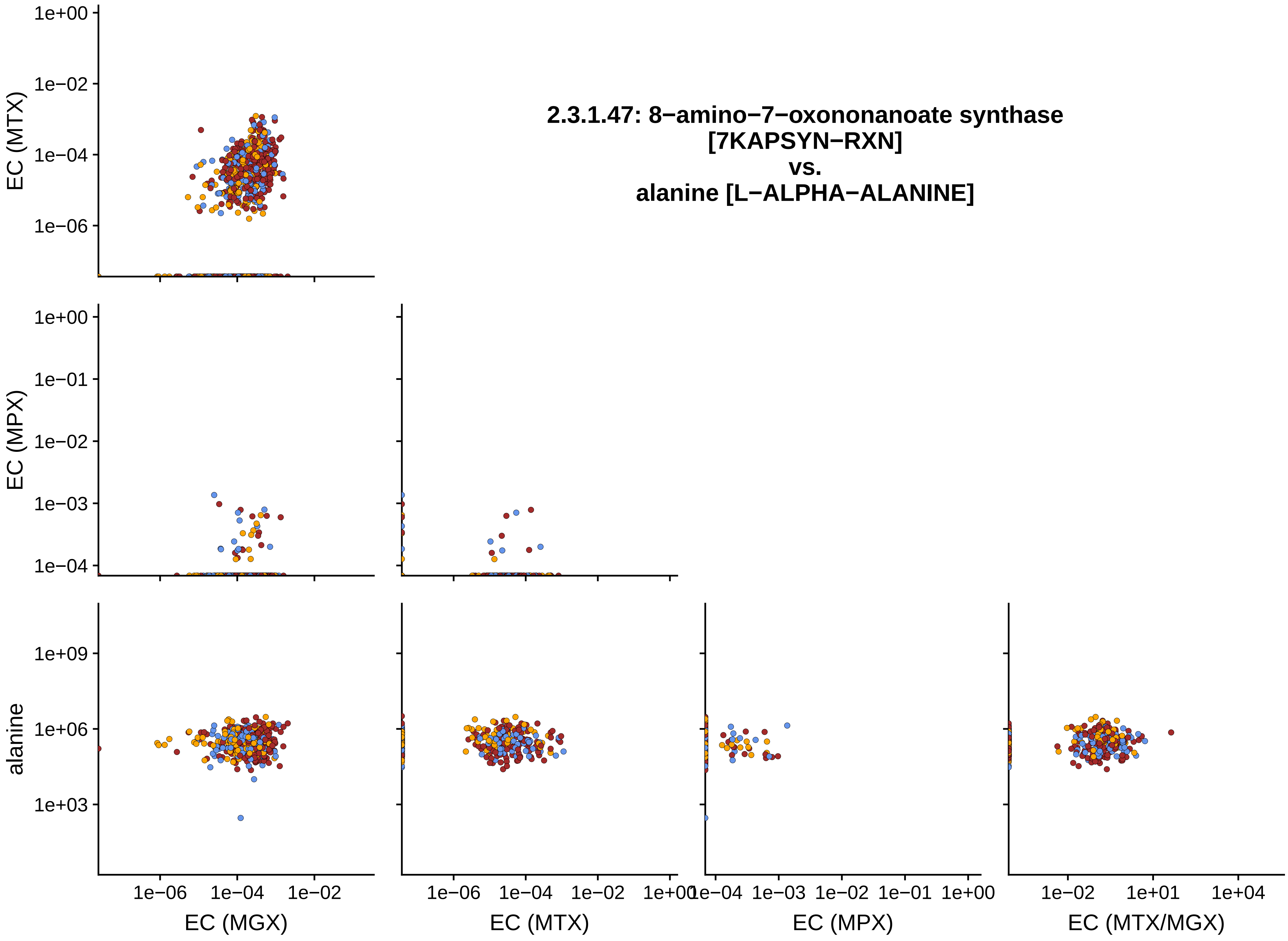

2.3.1.47: 8−amino−7−oxononanoate synthase
[7KAPSYN−RXN]
vs.
alanine [L−ALPHA−ALANINE]
EC (MTX)
EC (MPX)
alanine
EC (MGX)
EC (MTX)
EC (MPX)
EC (MTX/MGX)
1e+00
1e−02
1e−04
1e−06
1e−01
1e−03
1e+09
1e+06
1e+03
1e+01
1e+04

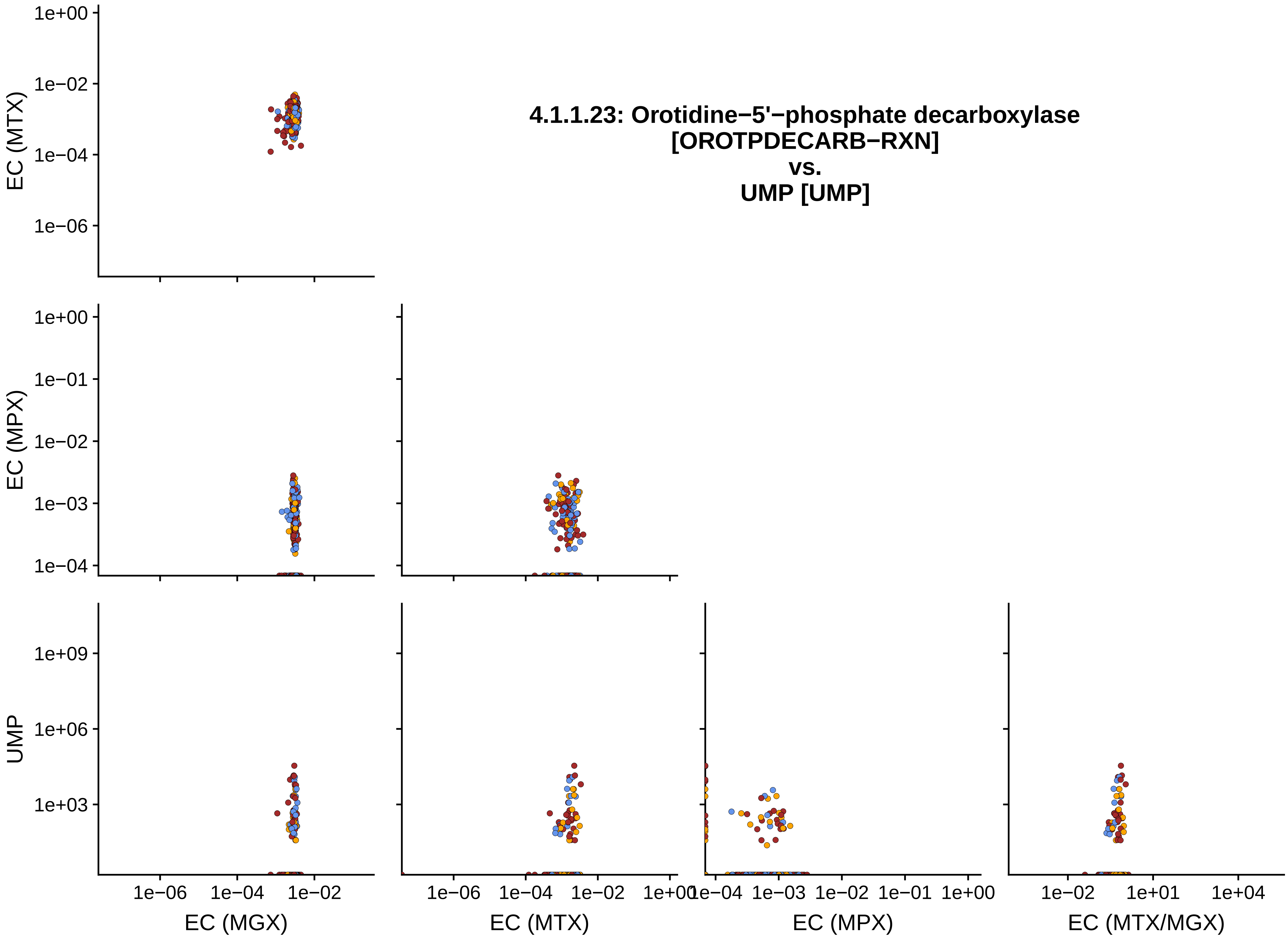

4.1.1.23: Orotidine−5'−phosphate decarboxylase
[OROTPDECARB−RXN]
vs.
UMP [UMP]
EC (MTX)
EC (MPX)
UMP
EC (MGX)
EC (MTX)
EC (MPX)
EC (MTX/MGX)
1e+00
1e−02
1e−04
1e−06
1e−01
1e−03
1e+09
1e+06
1e+03
1e+01
1e+04

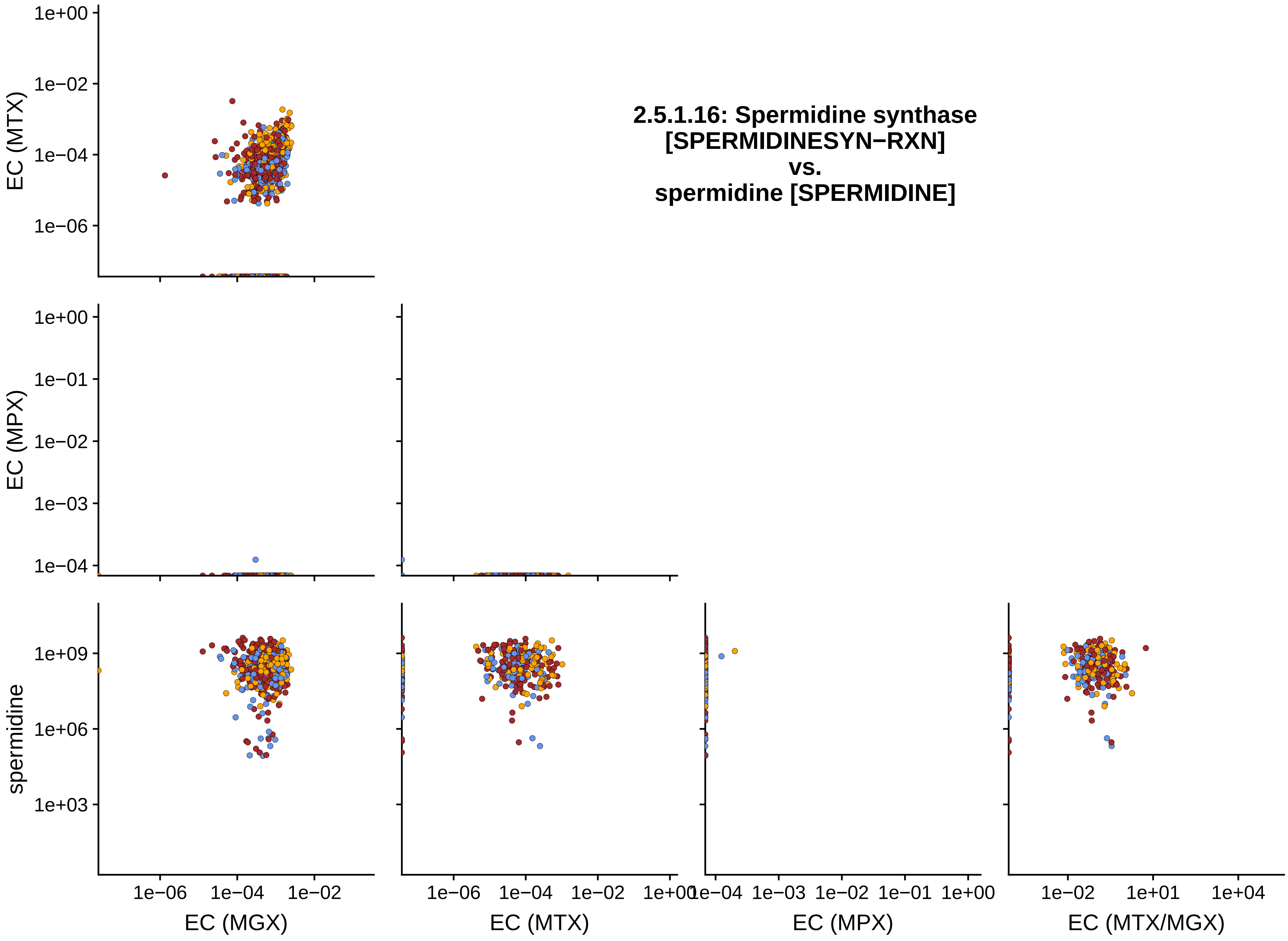
2.5.1.16: Spermidine synthase
[SPERMIDINESYN−RXN]
vs.
spermidine [SPERMIDINE]
EC (MTX)
EC (MPX)
spermidine
EC (MGX)
EC (MTX)
EC (MPX)
EC (MTX/MGX)
1e+00
1e−02
1e−04
1e−06
1e−01
1e−03
1e+09
1e+06
1e+03
1e+01
1e+04

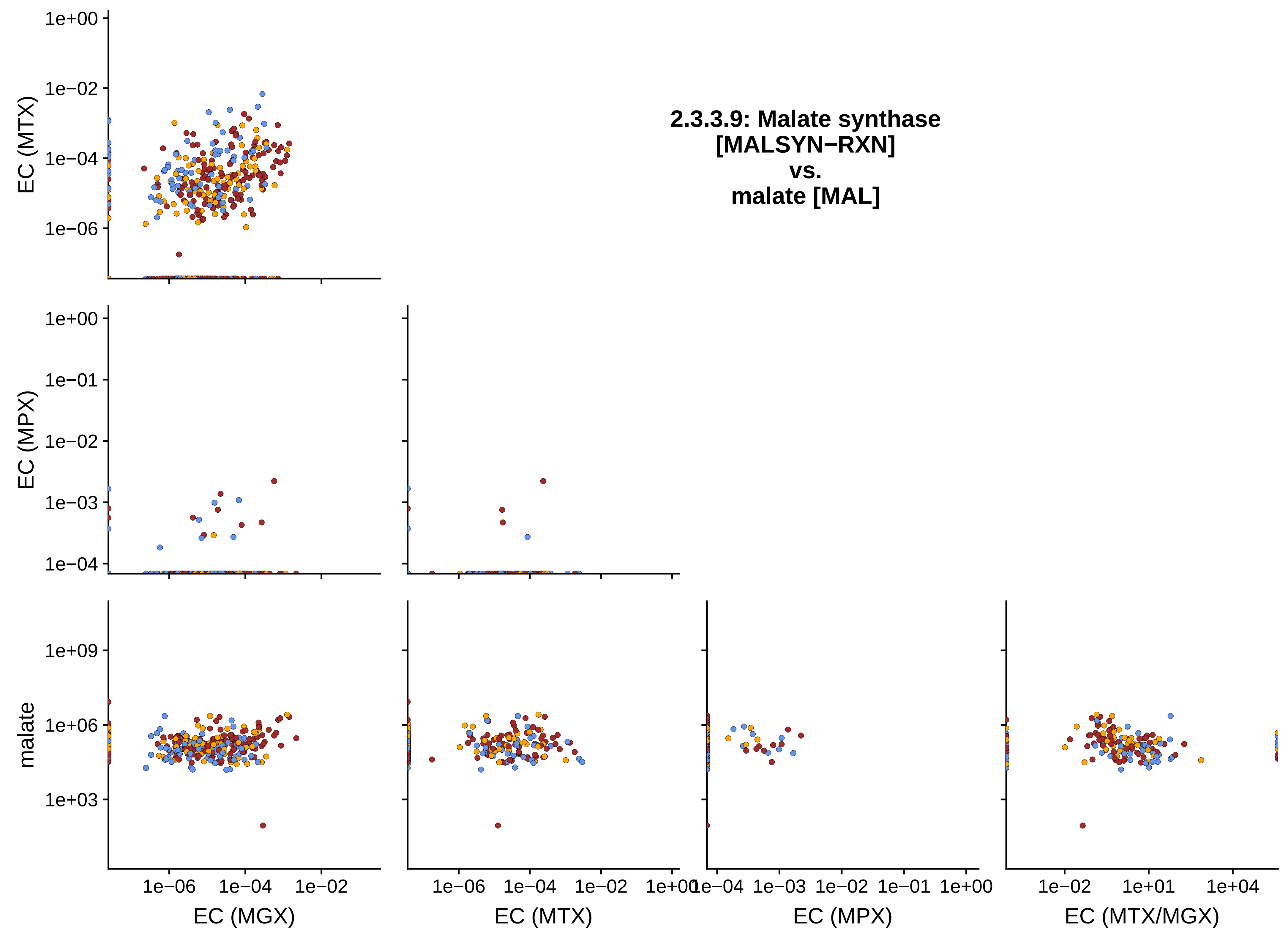

2.3.3.9: Malate synthase
[MALSYN−RXN]
vs.
malate [MAL]
EC (MTX)
EC (MPX)
malate
EC (MGX)
EC (MTX)
EC (MPX)
EC (MTX/MGX)
1e+00
1e−02
1e−04
1e−06
1e−01
1e−03
1e+09
1e+06
1e+03
1e+01
1e+04

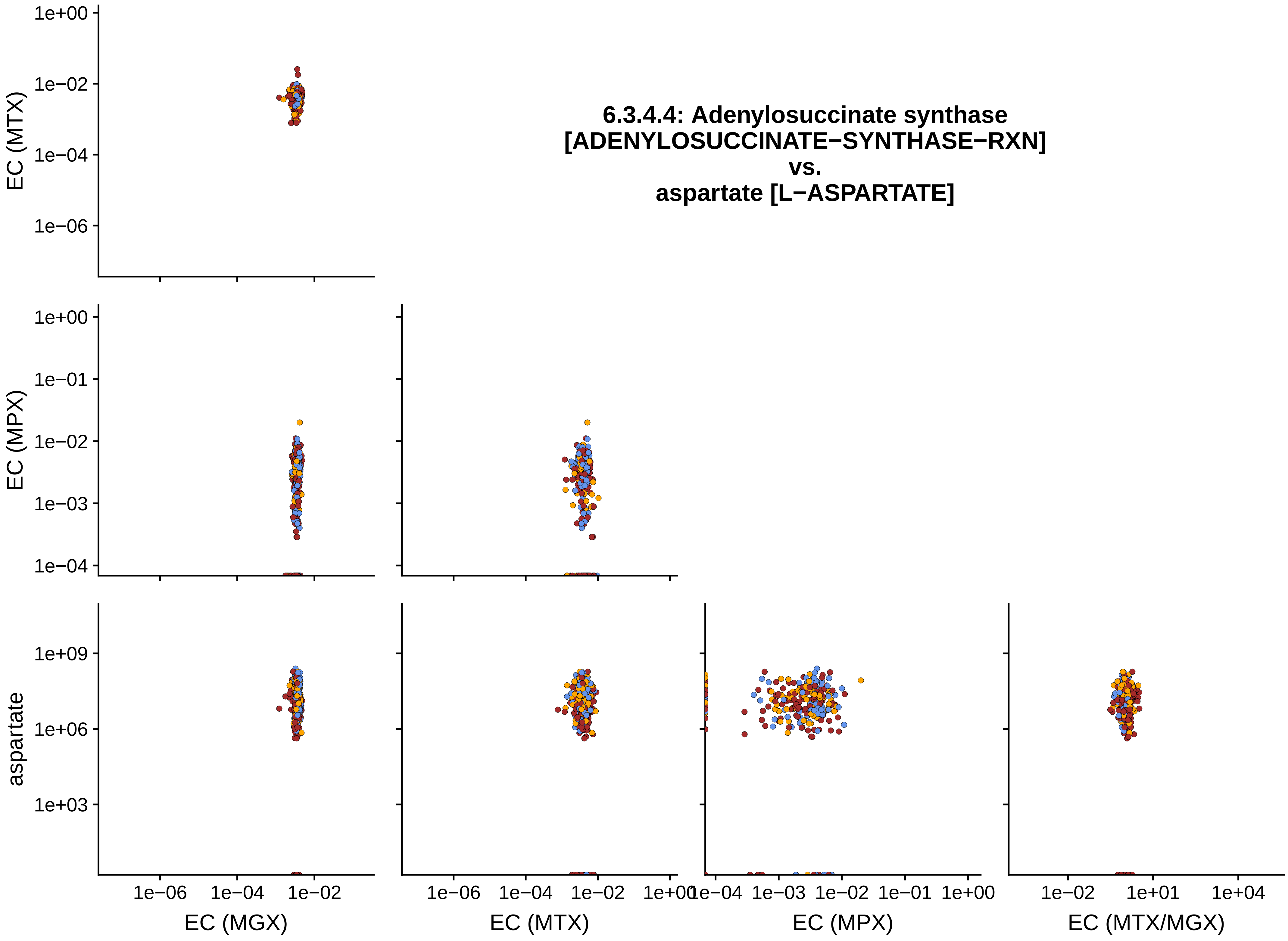

6.3.4.4: Adenylosuccinate synthase
[ADENYLOSUCCINATE−SYNTHASE−RXN]
vs.
aspartate [L−ASPARTATE]
EC (MTX)
EC (MPX)
aspartate
EC (MGX)
EC (MTX)
EC (MPX)
EC (MTX/MGX)
1e+00
1e−02
1e−04
1e−06
1e−01
1e−03
1e+09
1e+06
1e+03
1e+01
1e+04

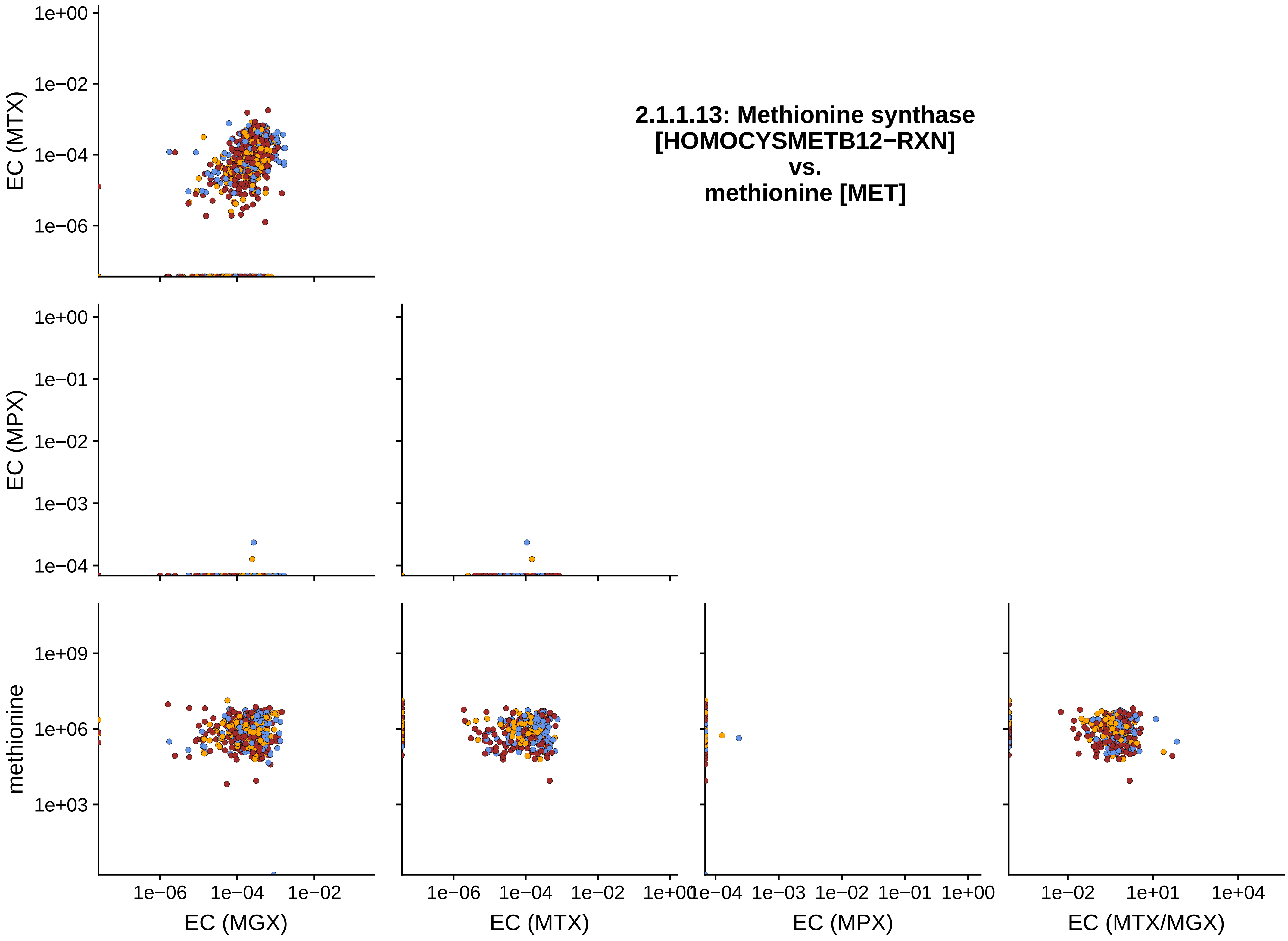
2.1.1.13: Methionine synthase
[HOMOCYSMETB12−RXN]
vs.
methionine [MET]
EC (MTX)
EC (MPX)
methionine
EC (MGX)
EC (MTX)
EC (MPX)
EC (MTX/MGX)
1e+00
1e−02
1e−04
1e−06
1e−01
1e−03
1e+09
1e+06
1e+03
1e+01
1e+04

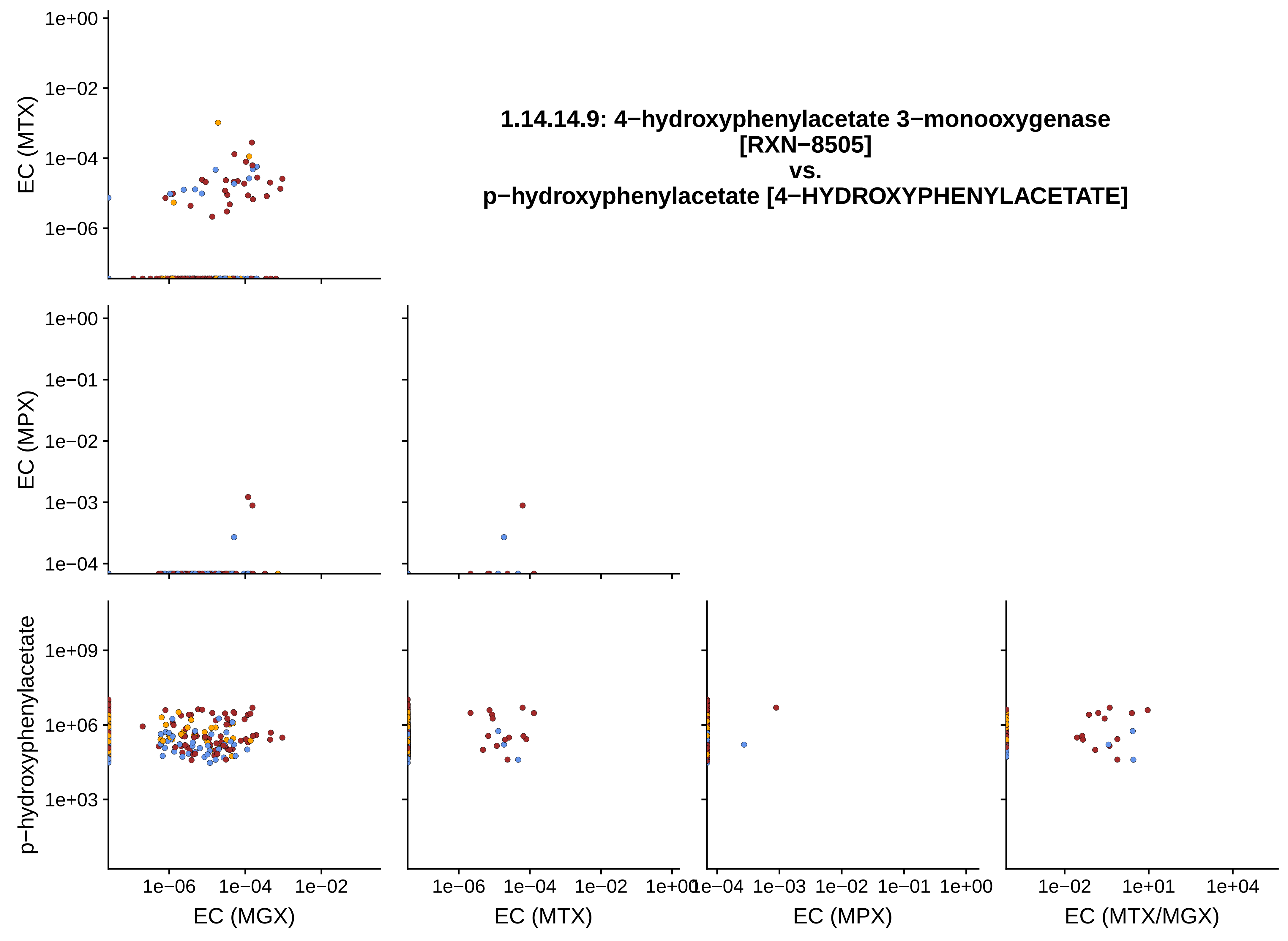

1.14.14.9: 4−hydroxyphenylacetate 3−monooxygenase
[RXN−8505]
vs.
p−hydroxyphenylacetate [4−HYDROXYPHENYLACETATE]
EC (MTX)
1e+00
1e−02
1e−04
1e−06
EC (MPX)
1e+00
1e−01
1e−02
1e−03
1e−04
p−hydroxyphenylacetate
1e+09
1e+06
1e+03
1e−06
1e−04
1e−02
EC (MGX)
1e−06
1e−04
1e−02
1e+00
EC (MTX)
1e−04
1e−03
1e−02
1e−01
1e+00
EC (MPX)
1e−02
1e+01
1e+04
EC (MTX/MGX)

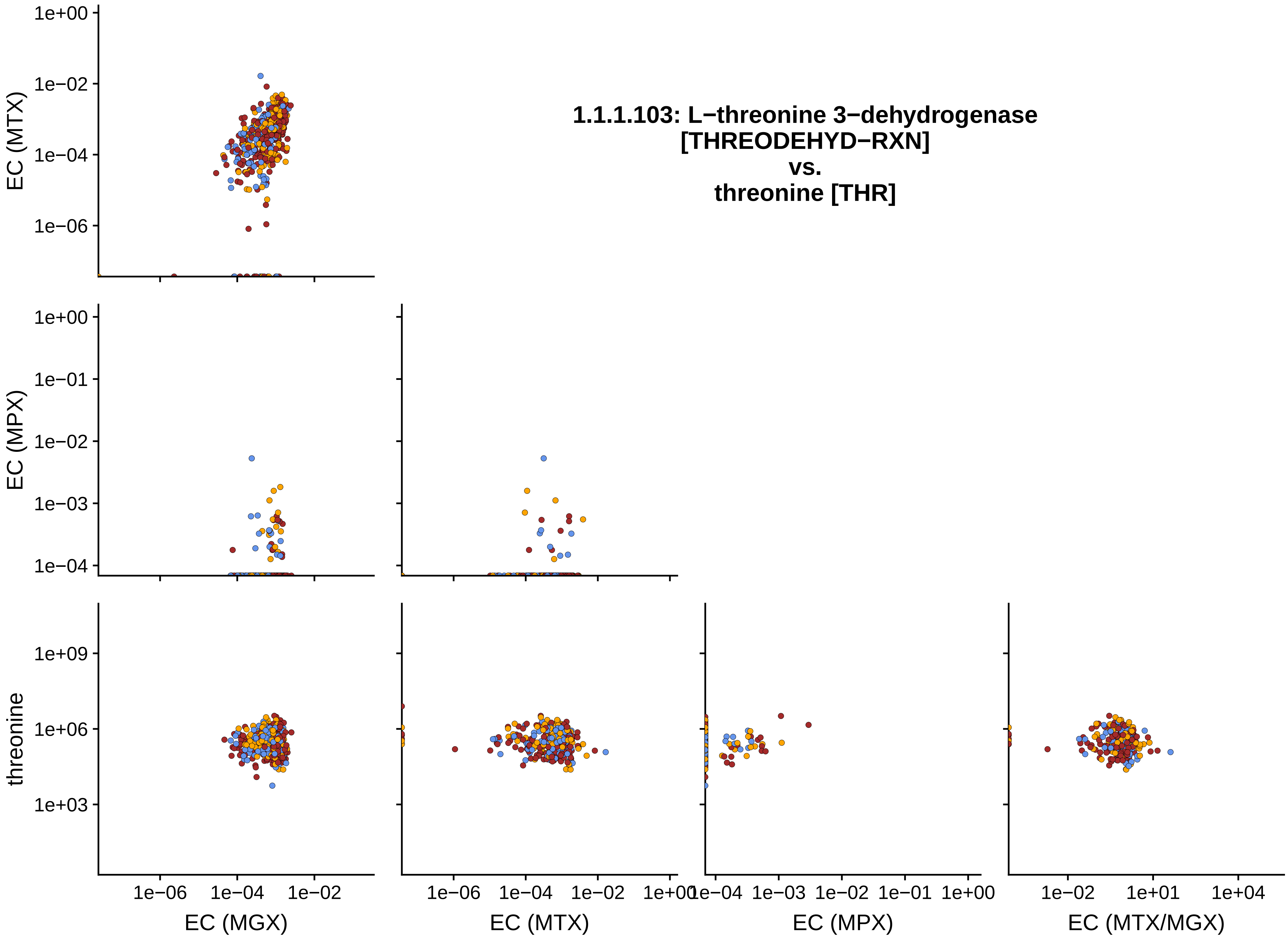
1.1.1.103: L−threonine 3−dehydrogenase
[THREODEHYD−RXN]
vs.
threonine [THR]
EC (MTX)
EC (MPX)
threonine
EC (MGX)
EC (MTX)
EC (MPX)
EC (MTX/MGX)
1e+00
1e−02
1e−04
1e−06
1e−01
1e−03
1e+09
1e+06
1e+03
1e+04
1e+01

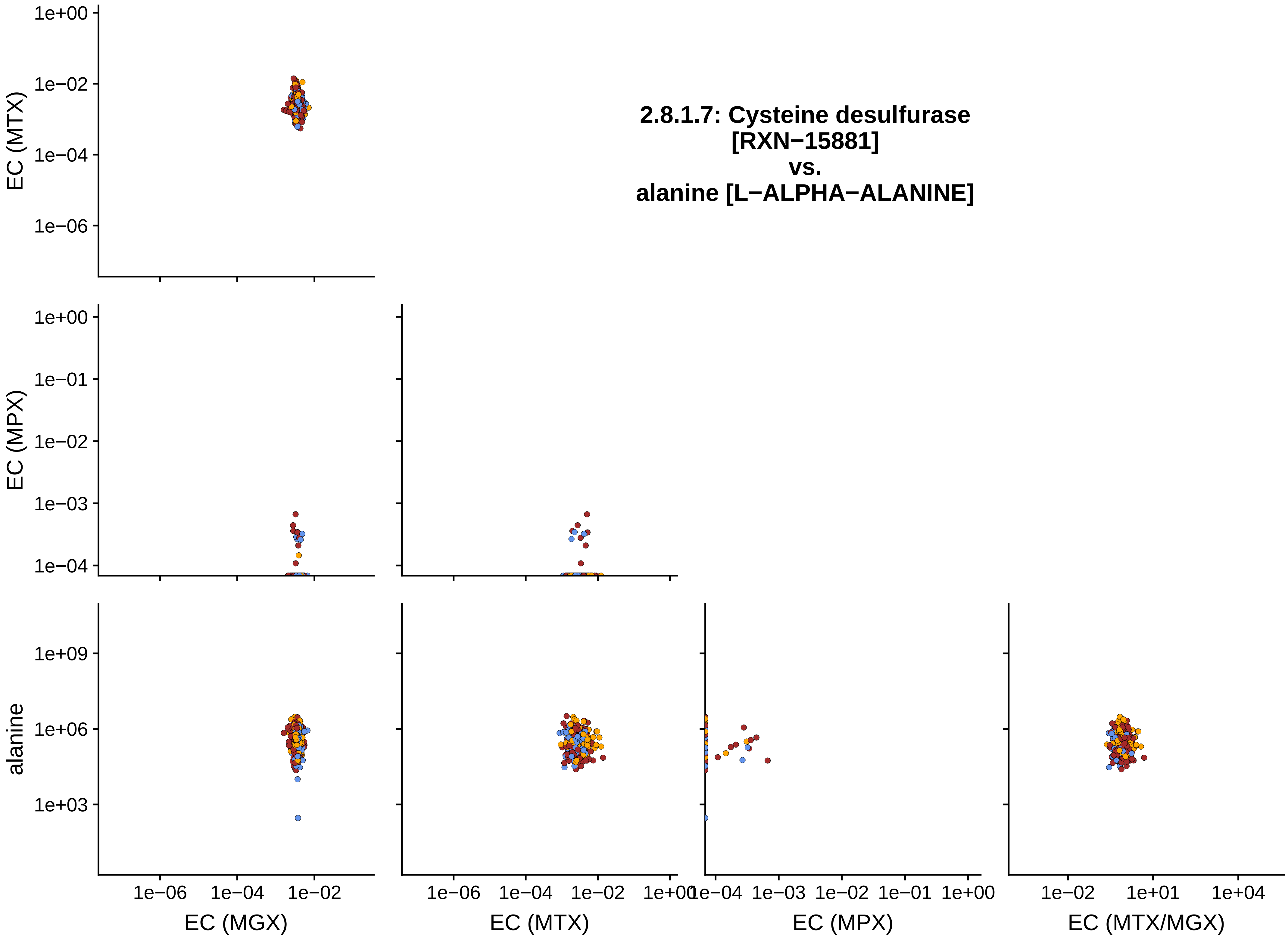

2.8.1.7: Cysteine desulfurase
[RXN−15881]
vs.
alanine [L−ALPHA−ALANINE]
EC (MTX)
EC (MPX)
alanine
EC (MGX)
EC (MTX)
EC (MPX)
EC (MTX/MGX)
1e+00
1e−02
1e−04
1e−06
1e−01
1e−03
1e+09
1e+06
1e+03
1e+01
1e+04

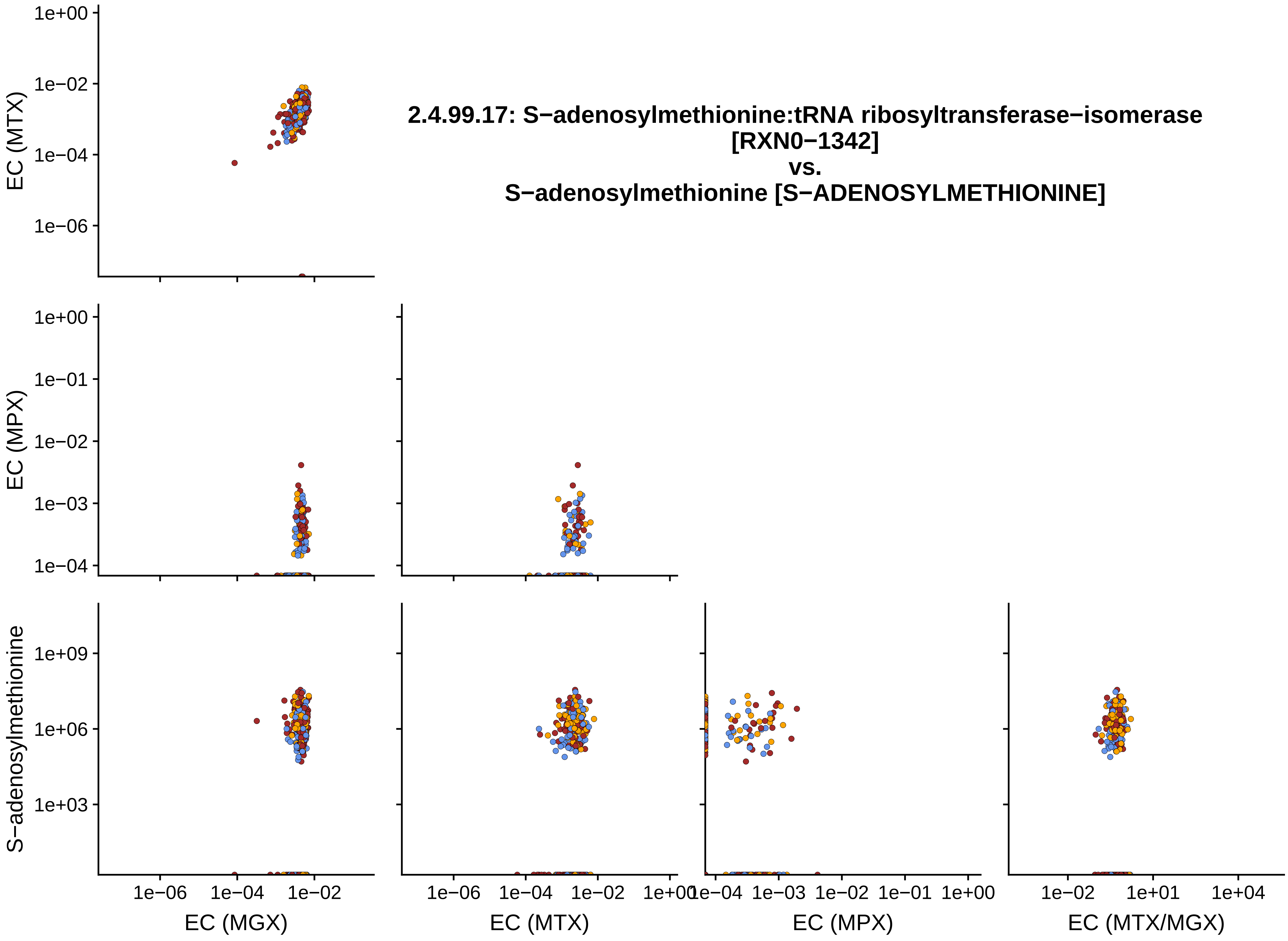
2.4.99.17: S−adenosylmethionine:tRNA ribosyltransferase−isomerase
[RXN0−1342]
vs.
S−adenosylmethionine [S−ADENOSYLMETHIONINE]
EC (MTX)
EC (MPX)
S−adenosylmethionine
EC (MGX)
EC (MTX)
EC (MPX)
EC (MTX/MGX)
1e+00
1e−02
1e−04
1e−06
1e−01
1e−03
1e+09
1e+06
1e+03
1e+01
1e+04

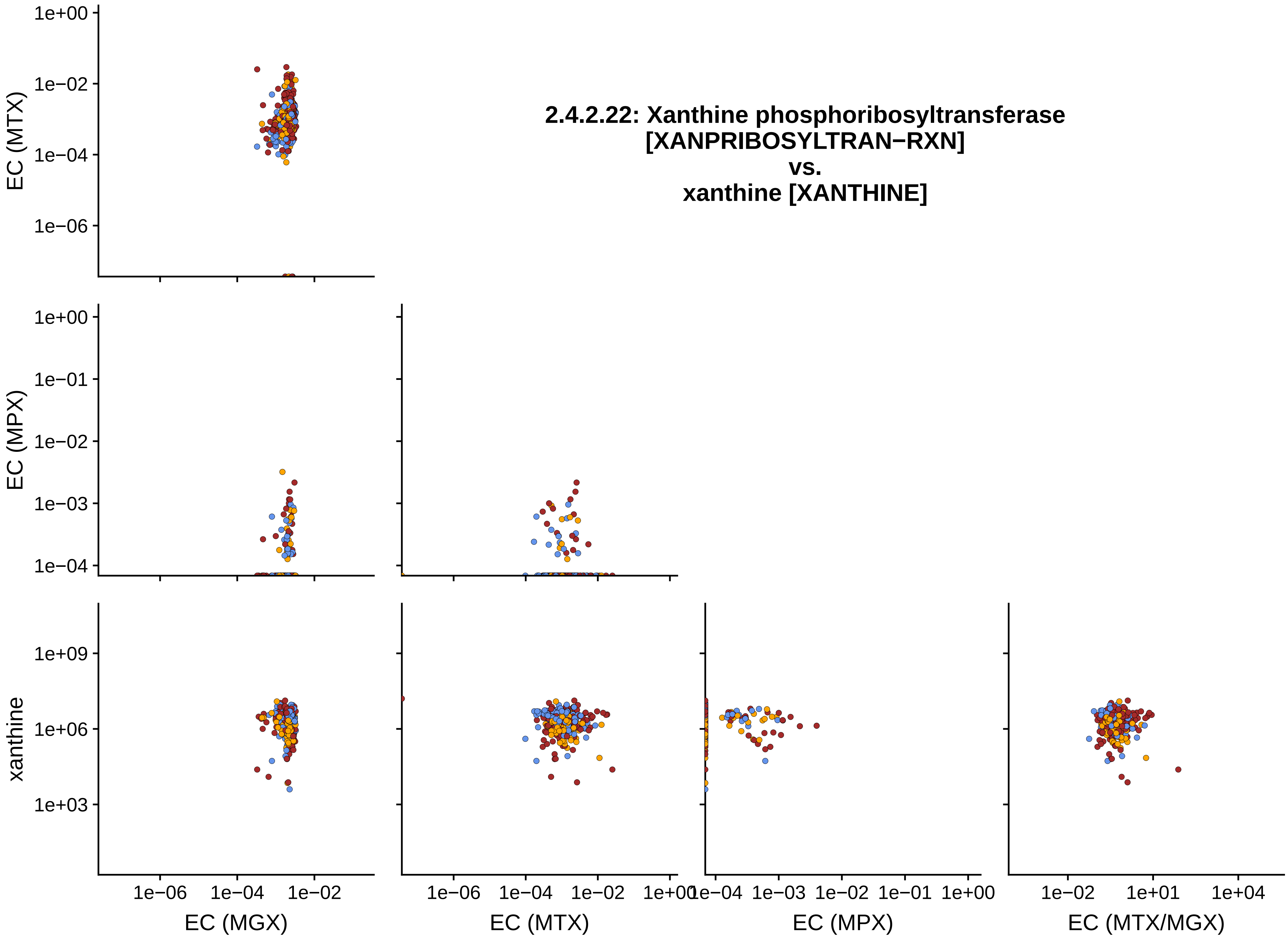

2.4.2.22: Xanthine phosphoribosyltransferase
[XANPRIBOSYLTRAN−RXN]
vs.
xanthine [XANTHINE]
EC (MTX)
EC (MPX)
xanthine
EC (MGX)
EC (MTX)
EC (MPX)
EC (MTX/MGX)
1e+00
1e−02
1e−04
1e−06
1e−01
1e−03
1e+09
1e+06
1e+03
1e+01
1e+04

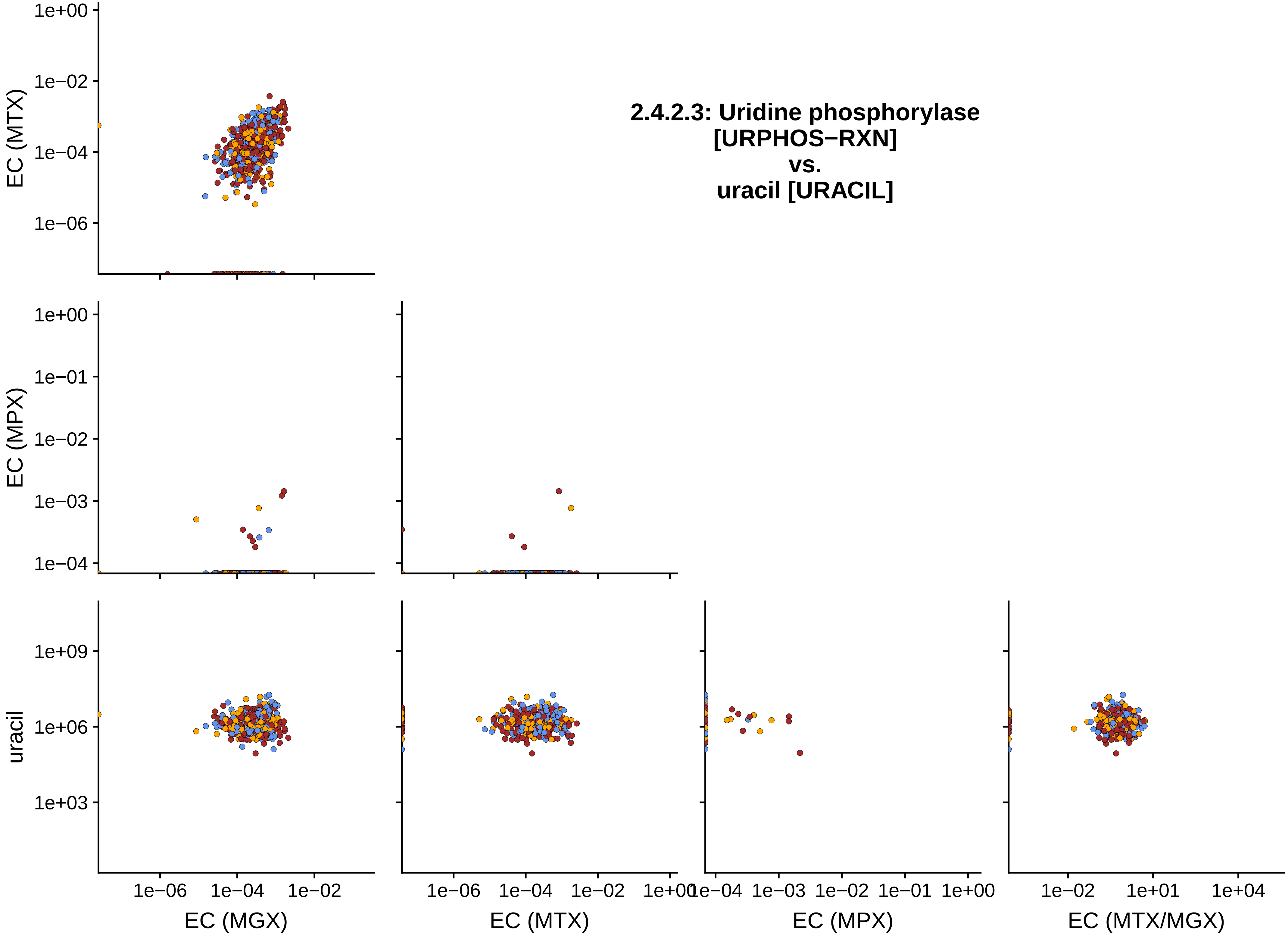

2.4.2.3: Uridine phosphorylase
[URPHOS−RXN]
vs.
uracil [URACIL]
EC (MTX)
EC (MPX)
uracil
EC (MGX)
EC (MTX)
EC (MPX)
EC (MTX/MGX)
1e+00
1e−02
1e−04
1e−06
1e−01
1e−03
1e+09
1e+06
1e+03
1e+01
1e+04

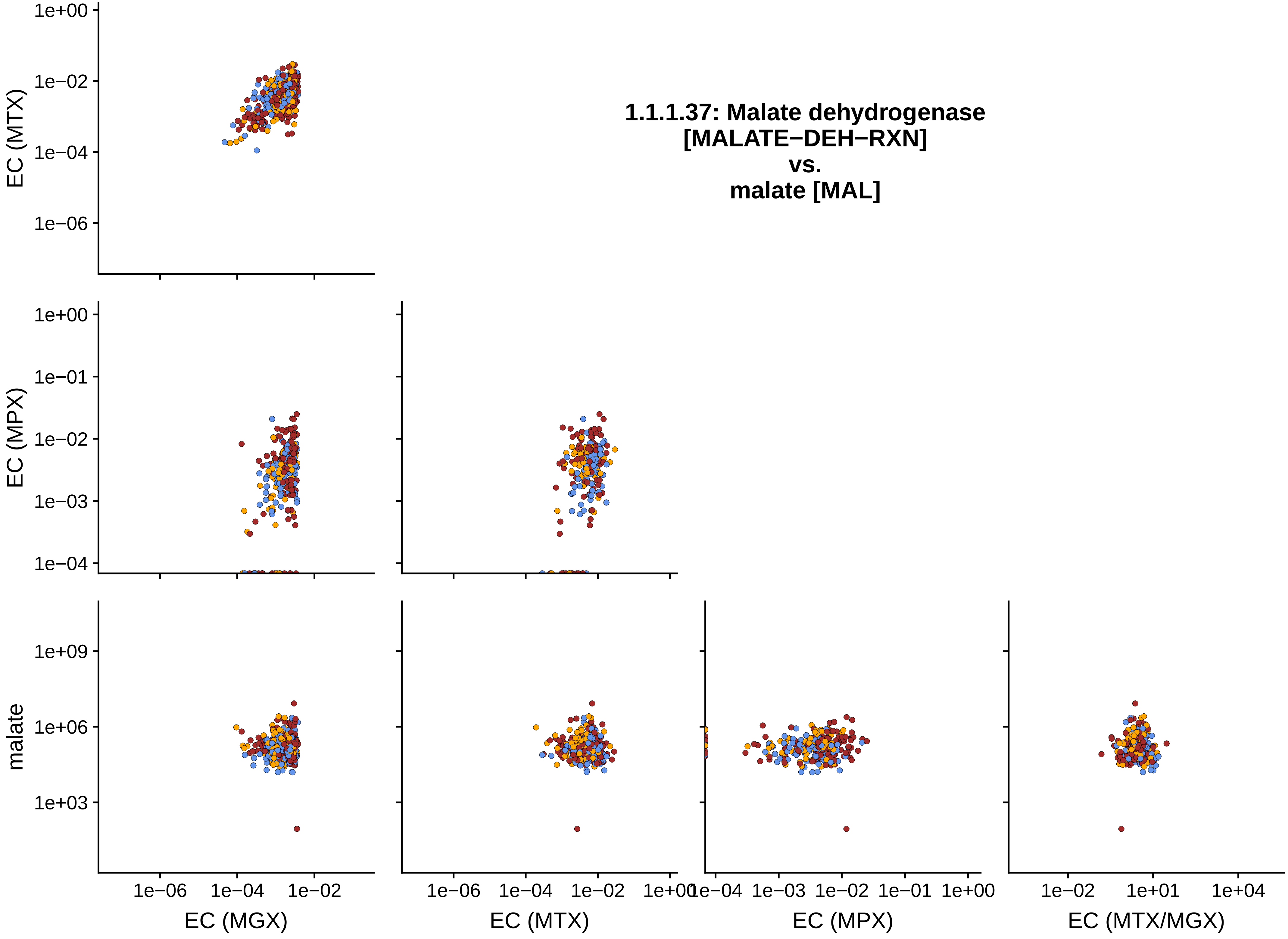
1.1.1.37: Malate dehydrogenase
[MALATE−DEH−RXN]
vs.
malate [MAL]
EC (MTX)
EC (MPX)
malate
EC (MGX)
EC (MTX)
EC (MPX)
EC (MTX/MGX)
1e+00
1e−02
1e−04
1e−06
1e−01
1e−03
1e+09
1e+06
1e+03
1e+01
1e+04

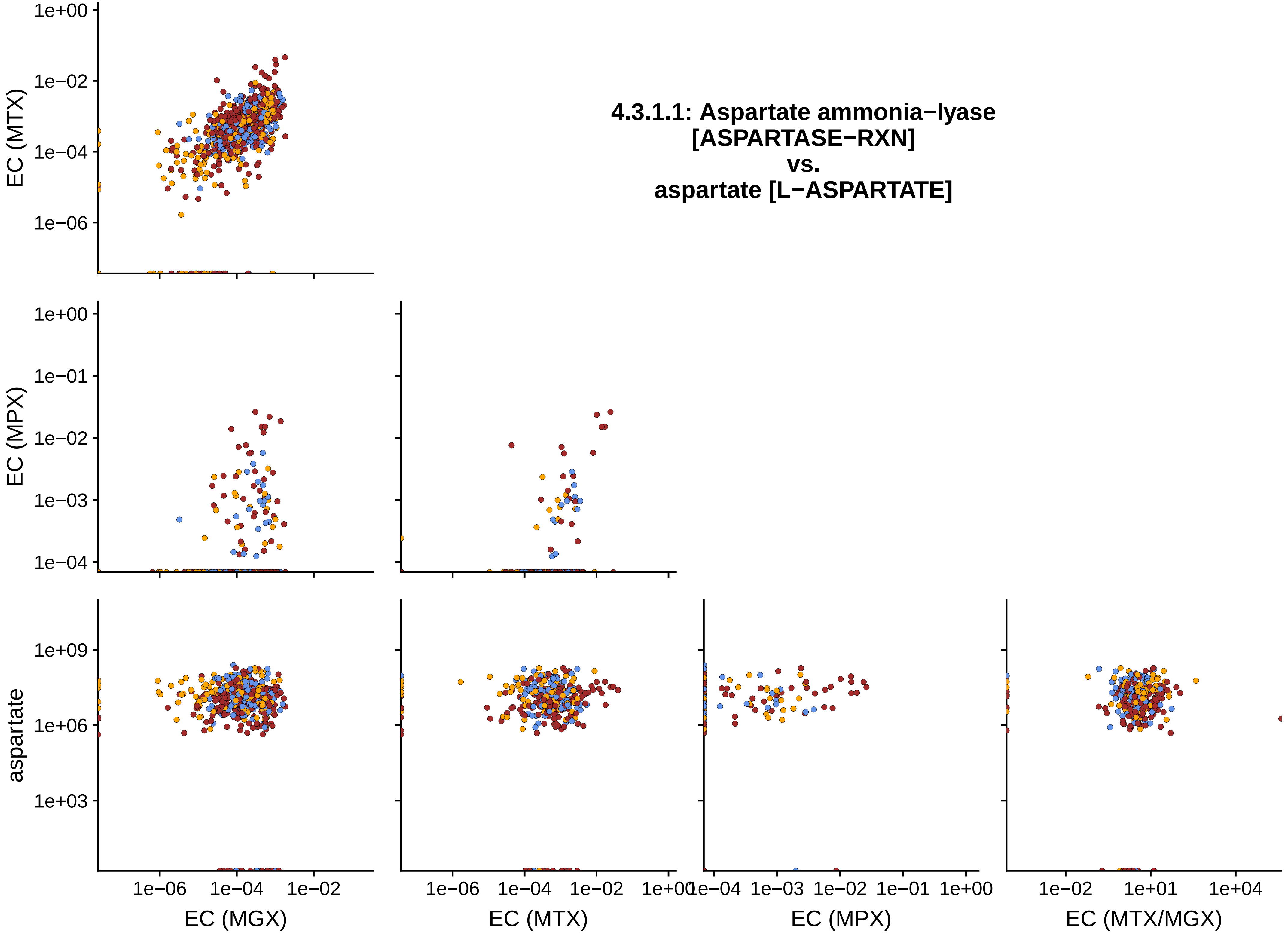

4.3.1.1: Aspartate ammonia−lyase
[ASPARTASE−RXN]
vs.
aspartate [L−ASPARTATE]
EC (MTX)
EC (MPX)
aspartate
EC (MGX)
EC (MTX)
EC (MPX)
EC (MTX/MGX)
1e+00
1e−02
1e−04
1e−06
1e−01
1e−03
1e+09
1e+06
1e+03
1e+01
1e+04

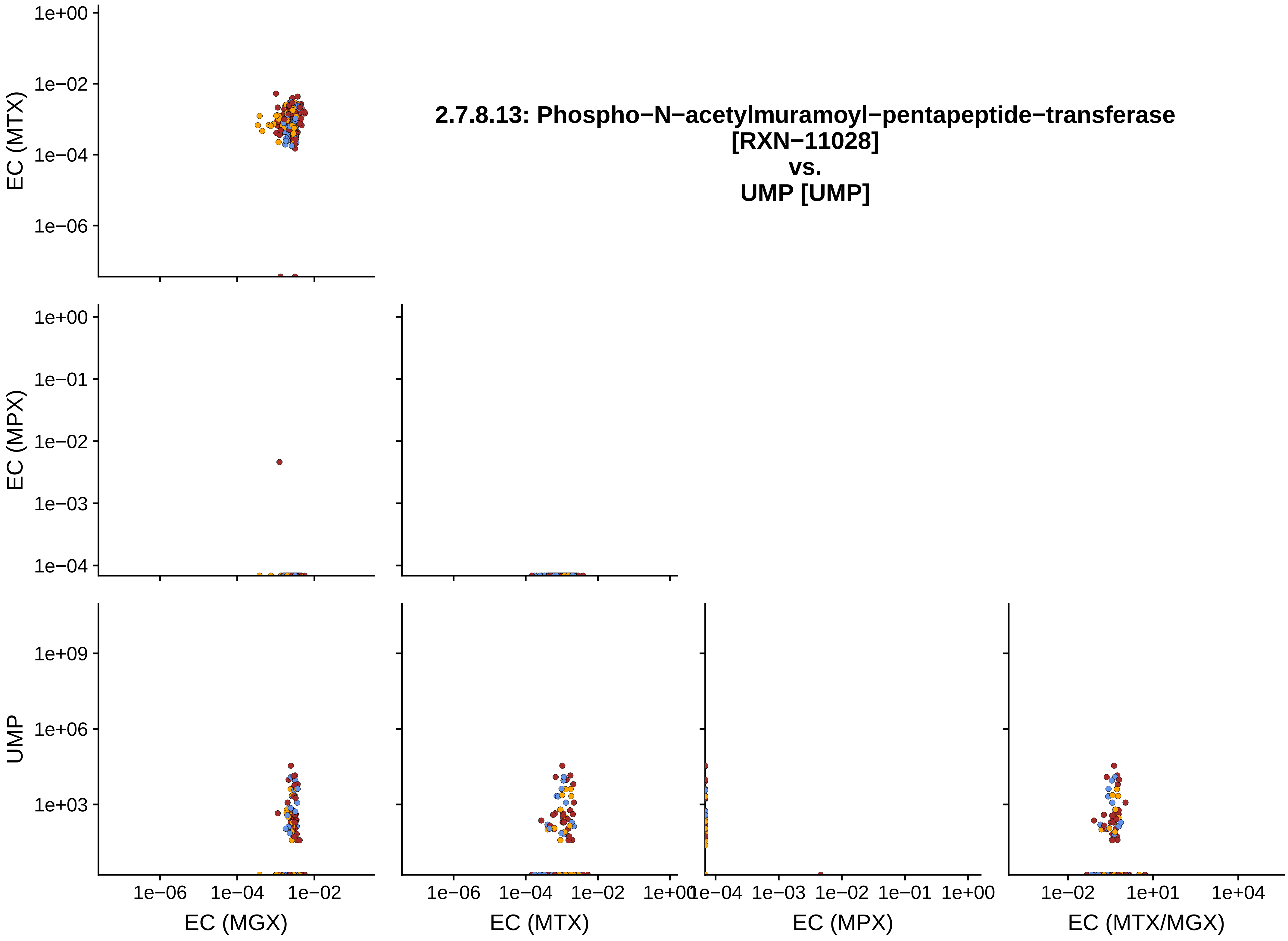
2.7.8.13: Phospho−N−acetylmuramoyl−pentapeptide−transferase
[RXN−11028]
vs.
UMP [UMP]
EC (MTX)
EC (MPX)
UMP
EC (MGX)
EC (MTX)
EC (MPX)
EC (MTX/MGX)
1e+00
1e−02
1e−04
1e−06
1e−01
1e−03
1e+09
1e+06
1e+03
1e+01
1e+04

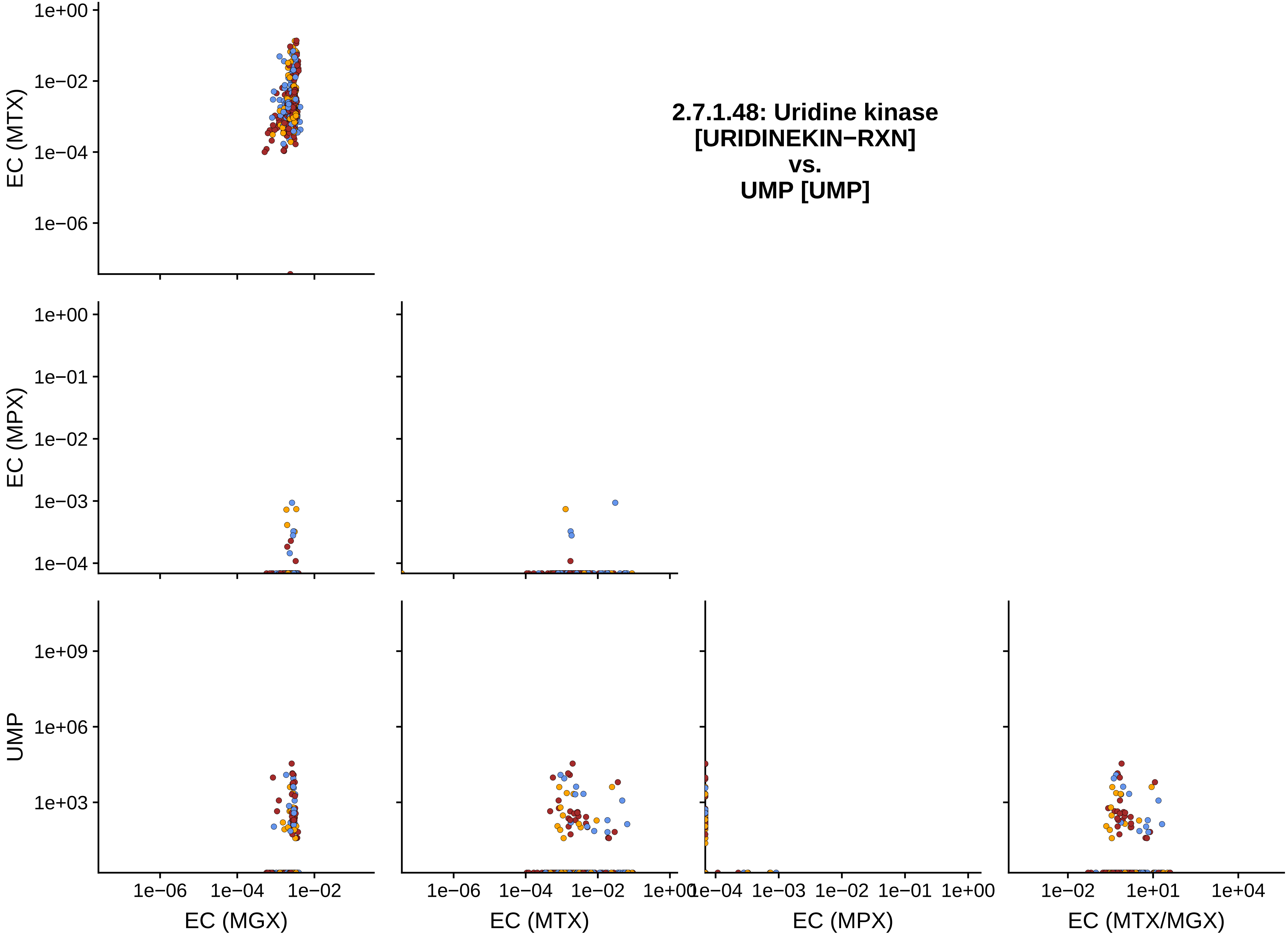

2.7.1.48: Uridine kinase
[URIDINEKIN−RXN]
vs.
UMP [UMP]
EC (MTX)
EC (MPX)
UMP
EC (MGX)
EC (MTX)
EC (MPX)
EC (MTX/MGX)
1e+00
1e−02
1e−04
1e−06
1e−01
1e−03
1e+09
1e+06
1e+03
1e+01
1e+04

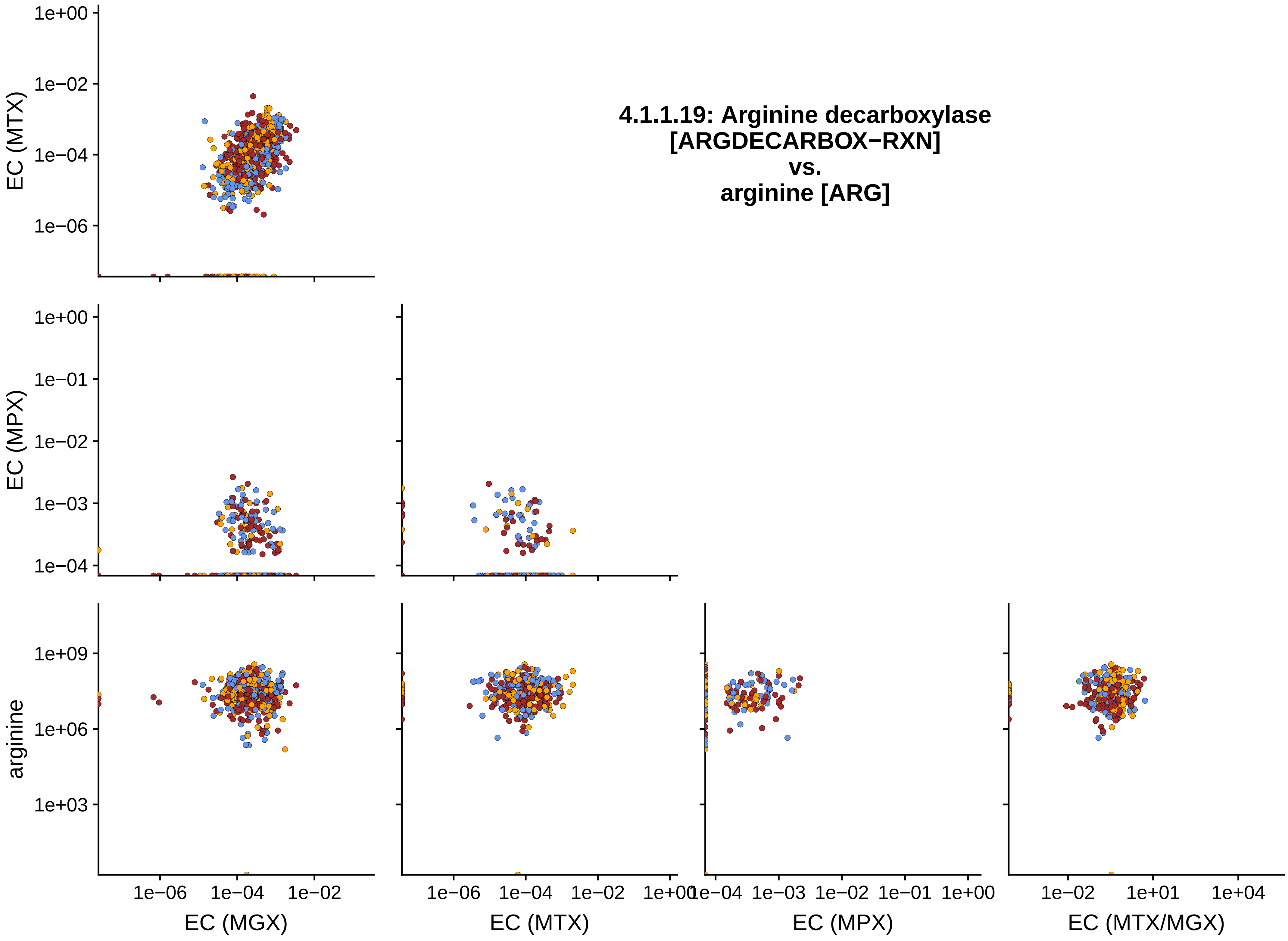

4.1.1.19: Arginine decarboxylase
[ARGDECARBOX−RXN]
vs.
arginine [ARG]
EC (MTX)
EC (MPX)
arginine
EC (MGX)
EC (MTX)
EC (MPX)
EC (MTX/MGX)
1e+00
1e−02
1e−04
1e−06
1e+00
1e−01
1e−02
1e−03
1e−04
1e+09
1e+06
1e+03
1e−06
1e−04
1e−02
1e−06
1e−04
1e−02
1e+00
1e−04
1e−03
1e−02
1e−01
1e+00
1e−02
1e+01
1e+04

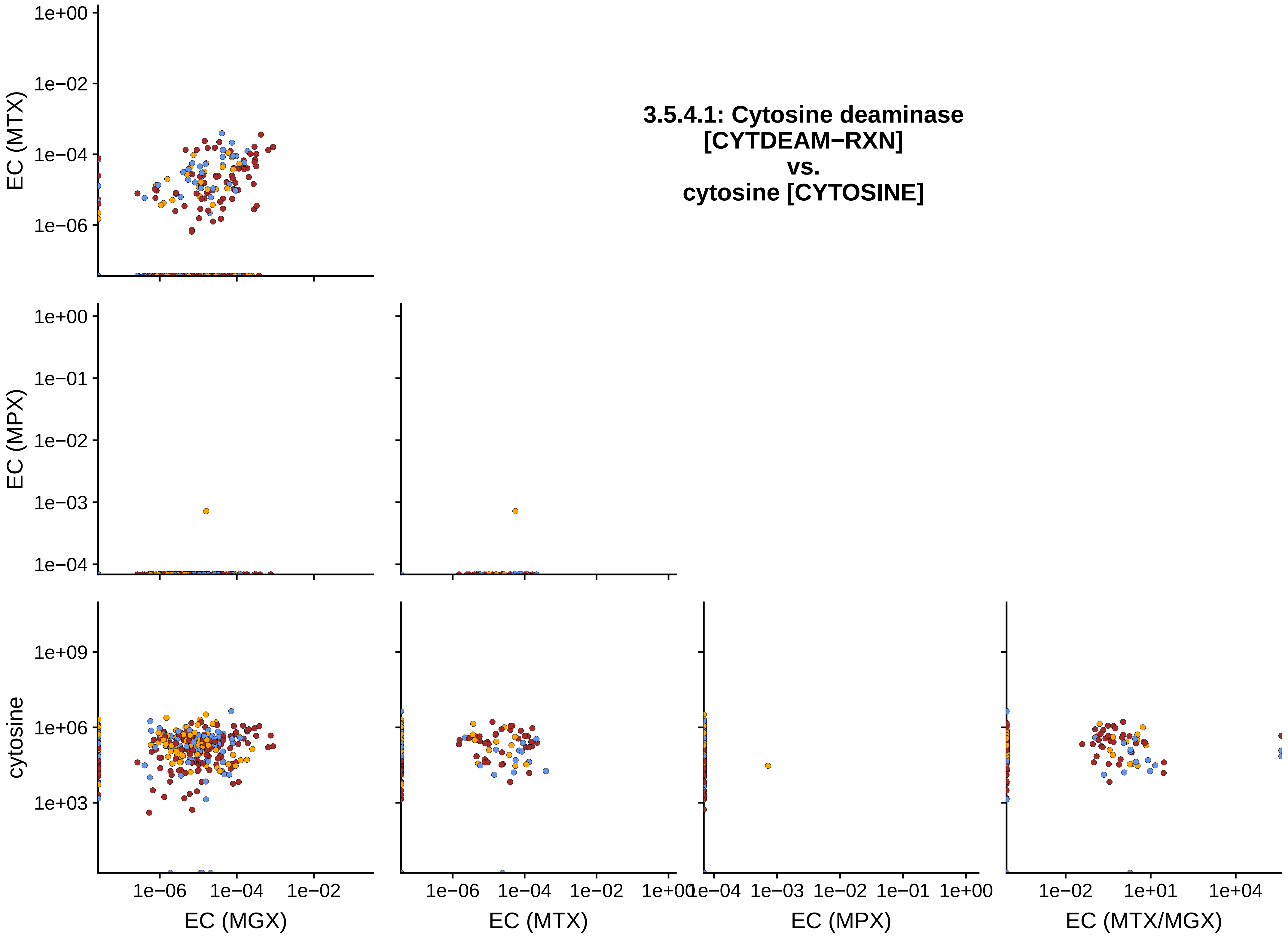

3.5.4.1: Cytosine deaminase
[CYTDEAM−RXN]
vs.
cytosine [CYTOSINE]
EC (MTX)
EC (MPX)
cytosine
EC (MGX)
EC (MTX)
EC (MPX)
EC (MTX/MGX)
1e+00
1e−02
1e−04
1e−06
1e−01
1e−03
1e+09
1e+06
1e+03
1e+01
1e+04

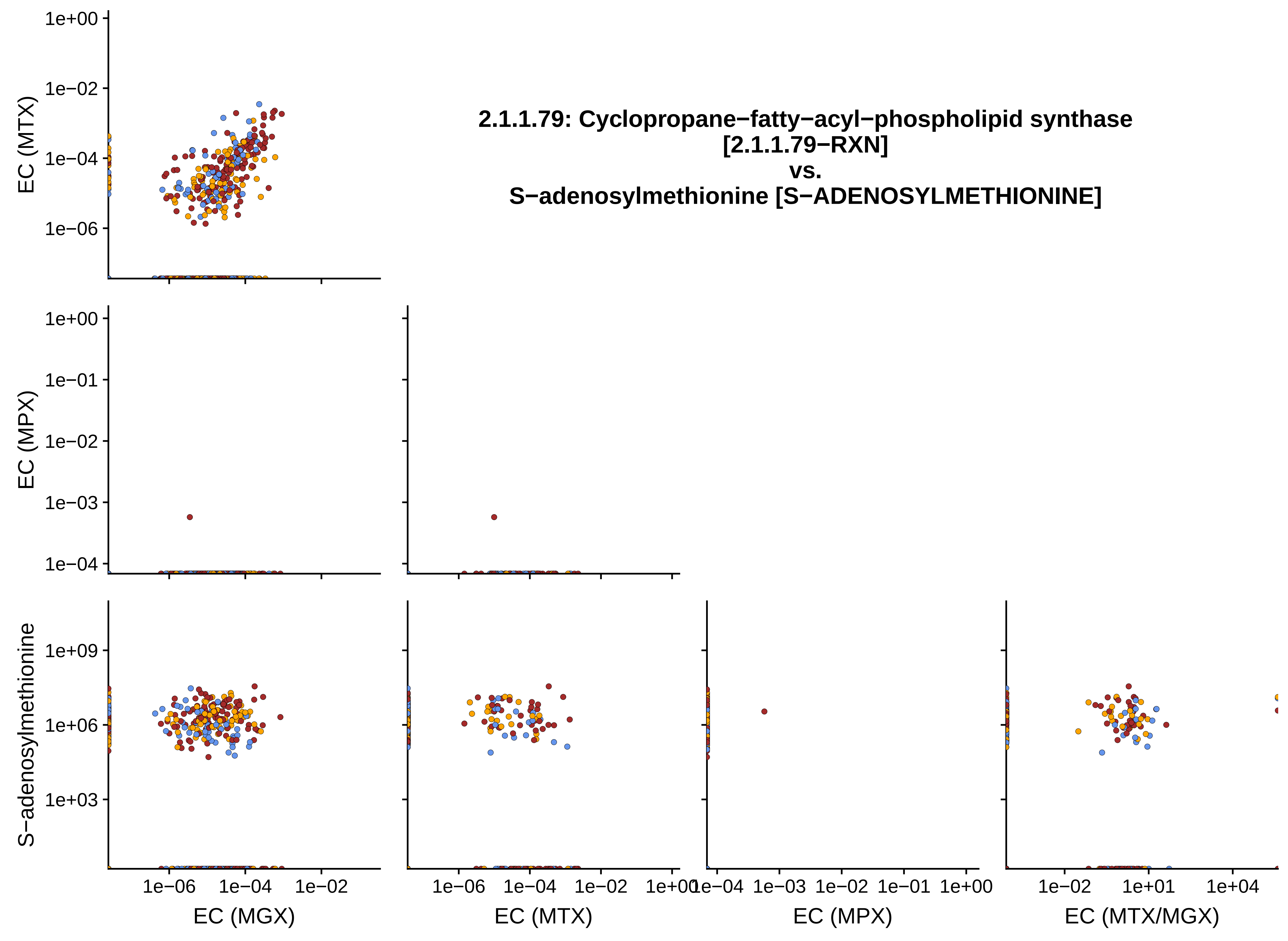

2.1.1.79: Cyclopropane−fatty−acyl−phospholipid synthase
[2.1.1.79−RXN]
vs.
S−adenosylmethionine [S−ADENOSYLMETHIONINE]
EC (MTX)
EC (MPX)
S−adenosylmethionine
EC (MGX)
EC (MTX)
EC (MPX)
EC (MTX/MGX)
1e+00
1e−02
1e−04
1e−06
1e−01
1e−03
1e+09
1e+06
1e+03
1e+01
1e+04

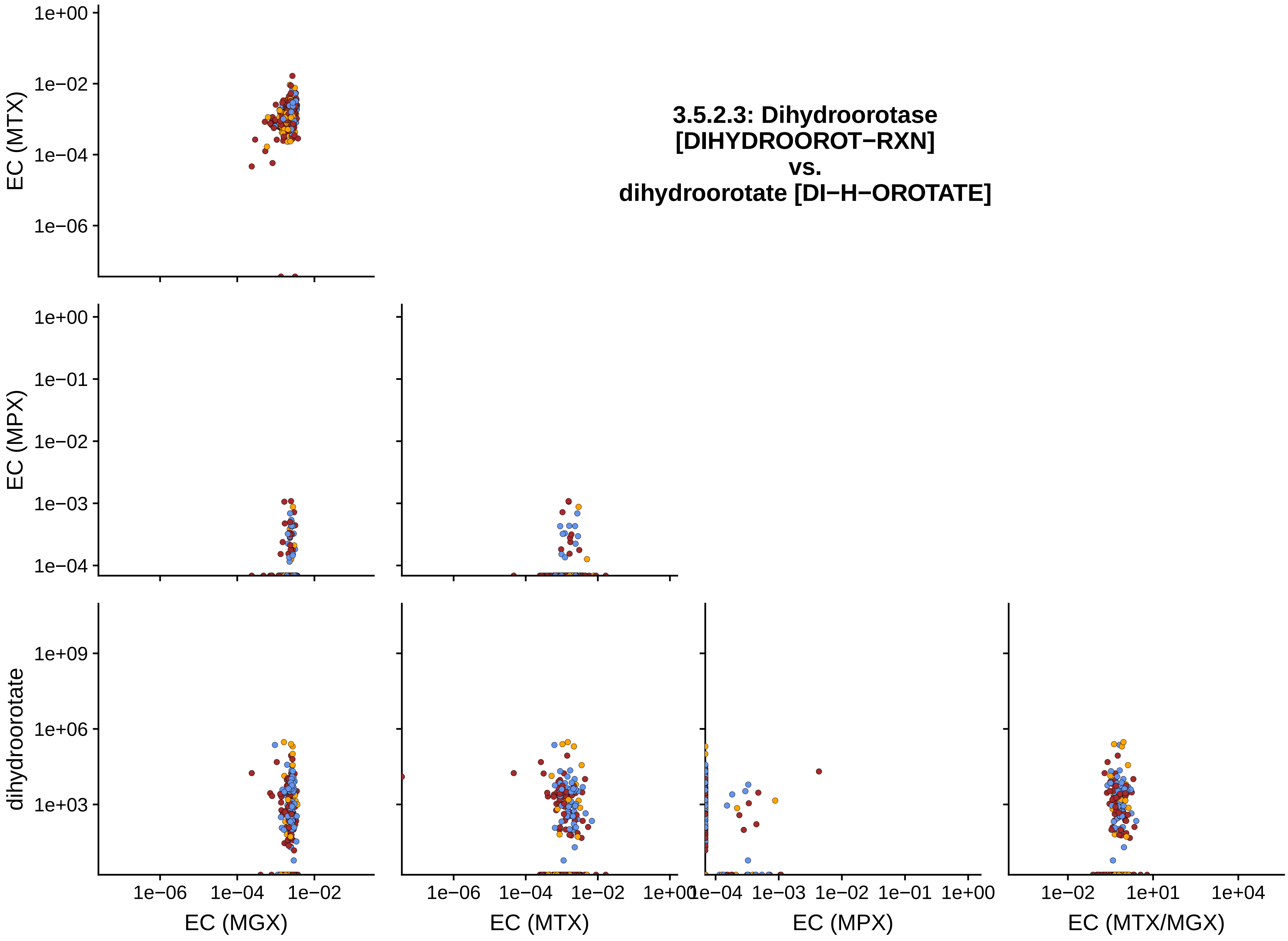

3.5.2.3: Dihydroorotase
[DIHYDROOROT−RXN]
vs.
dihydroorotate [DI−H−OROTATE]
EC (MTX)
EC (MPX)
dihydroorotate
EC (MGX)
EC (MTX)
EC (MPX)
EC (MTX/MGX)
1e+00
1e−02
1e−04
1e−06
1e−01
1e−03
1e+09
1e+06
1e+03
1e+01
1e+04

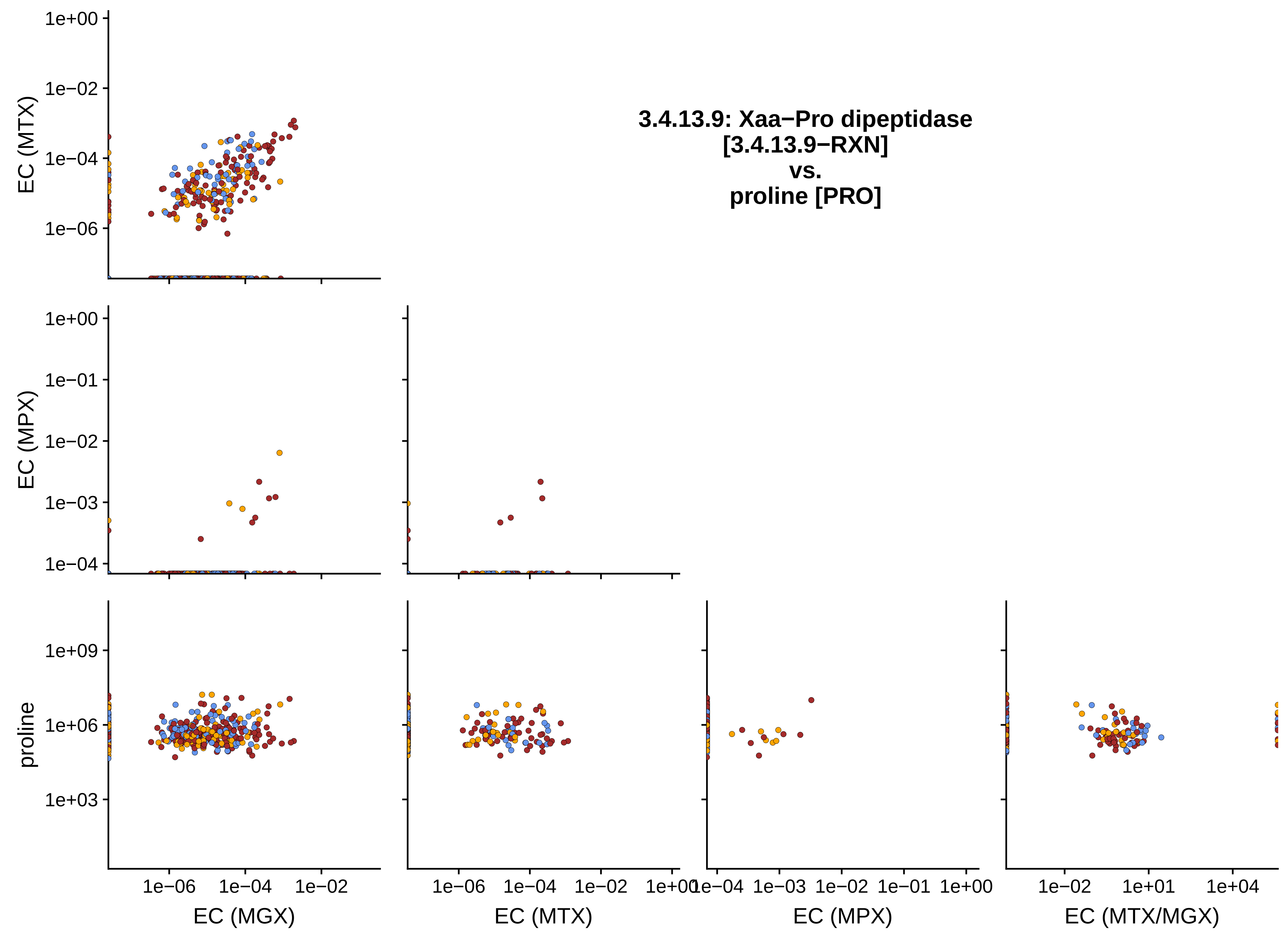

3.4.13.9: Xaa−Pro dipeptidase
[3.4.13.9−RXN]
vs.
proline [PRO]
EC (MTX)
EC (MPX)
proline
EC (MGX)
EC (MTX)
EC (MPX)
EC (MTX/MGX)
1e+00
1e−02
1e−04
1e−06
1e−01
1e−03
1e+09
1e+06
1e+03
1e+01
1e+04

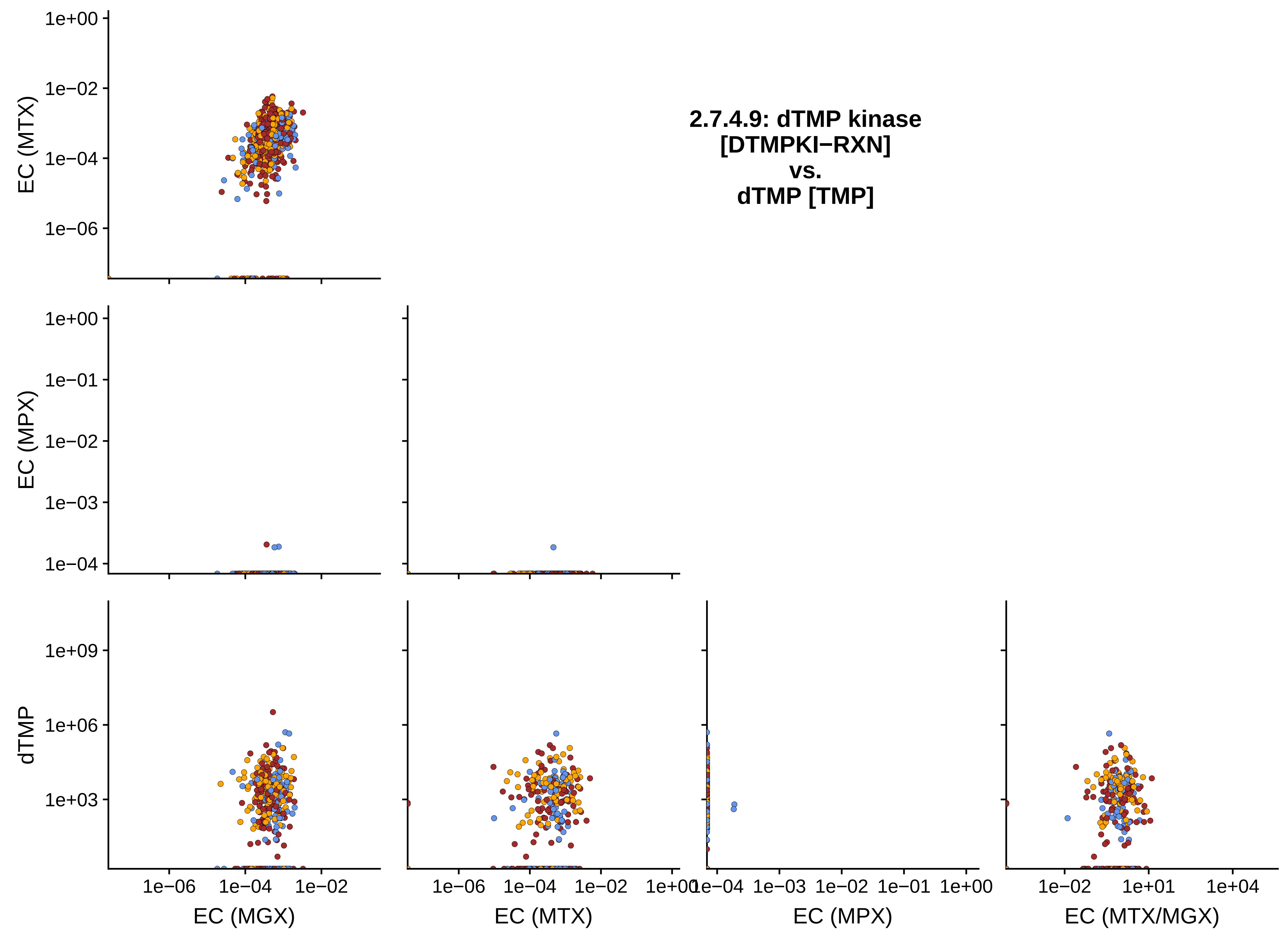
2.7.4.9: dTMP kinase
[DTMPKI−RXN]
vs.
dTMP [TMP]
EC (MTX)
EC (MPX)
dTMP
EC (MGX)
EC (MTX)
EC (MPX)
EC (MTX/MGX)
1e+00
1e−02
1e−04
1e−06
1e−01
1e−03
1e+09
1e+06
1e+03
1e+01
1e+04

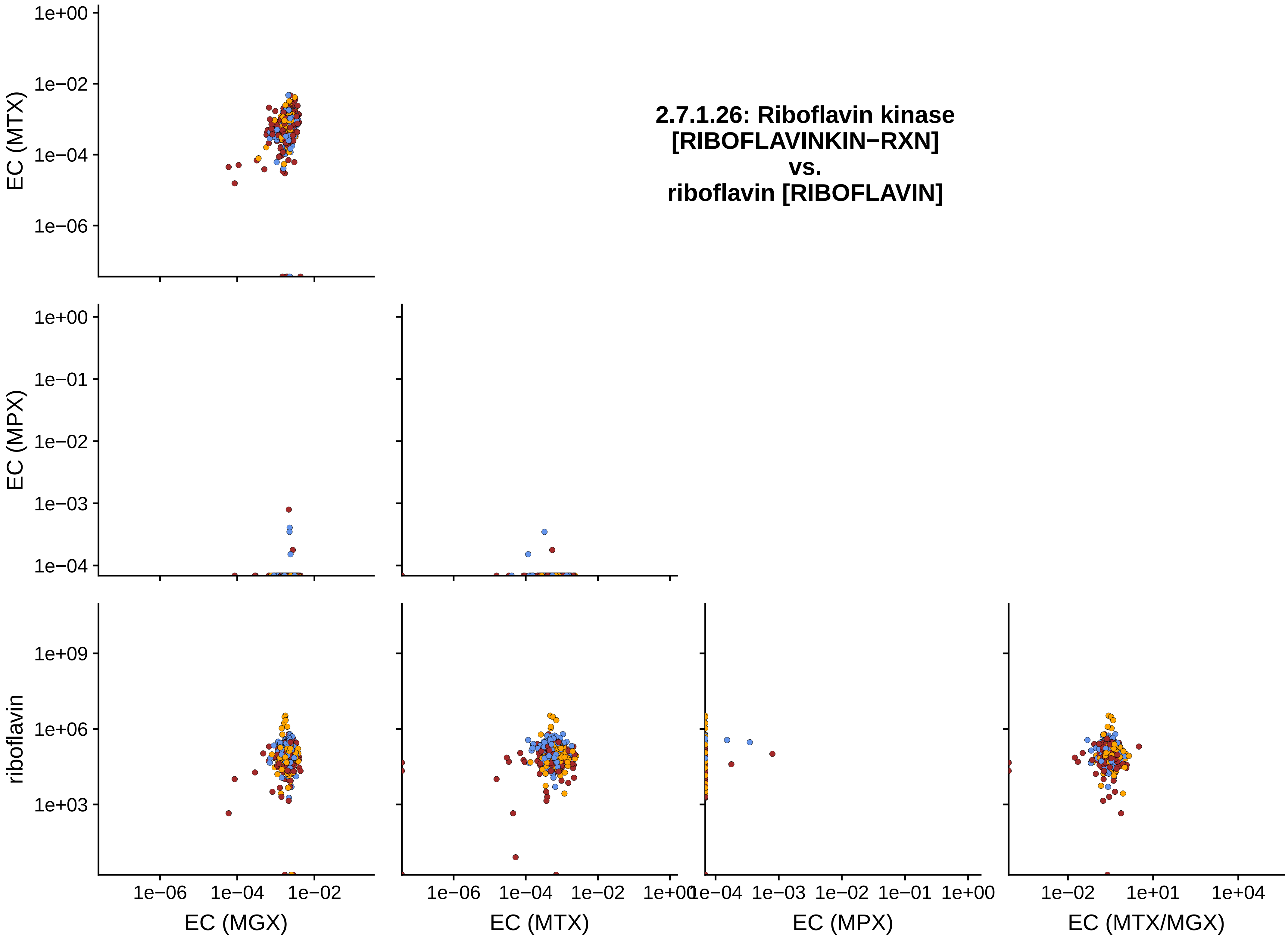
2.7.1.26: Riboflavin kinase
[RIBOFLAVINKIN−RXN]
vs.
riboflavin [RIBOFLAVIN]
EC (MTX)
EC (MPX)
riboflavin
EC (MGX)
EC (MTX)
EC (MPX)
EC (MTX/MGX)
1e+00
1e−02
1e−04
1e−06
1e−01
1e−03
1e+09
1e+06
1e+03
1e+01
1e+04

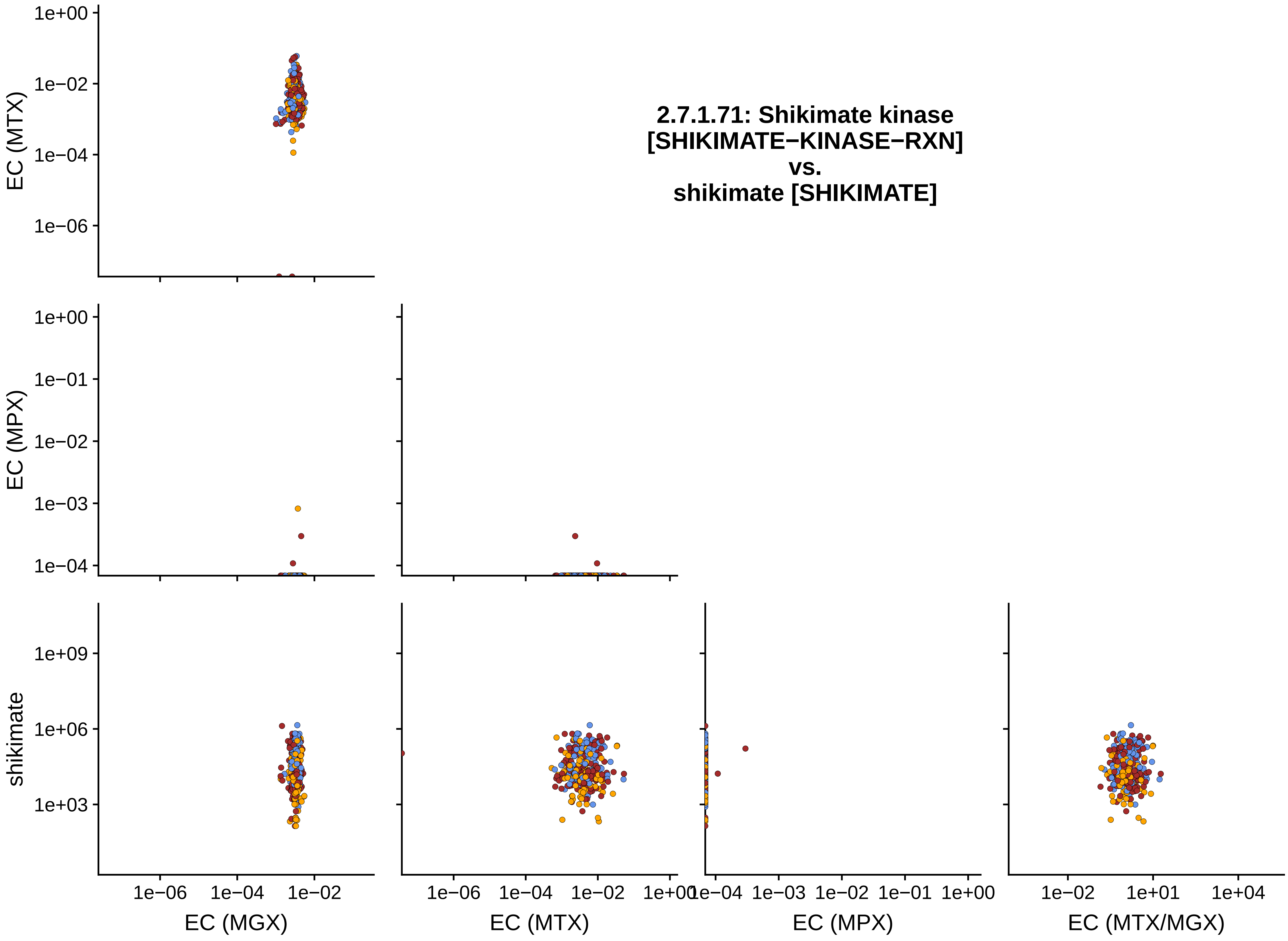

2.7.1.71: Shikimate kinase
[SHIKIMATE−KINASE−RXN]
vs.
shikimate [SHIKIMATE]
EC (MTX)
EC (MPX)
shikimate
EC (MGX)
EC (MTX)
EC (MPX)
EC (MTX/MGX)
1e+00
1e−02
1e−04
1e−06
1e+00
1e−01
1e−02
1e−03
1e−04
1e+09
1e+06
1e+03
1e−06
1e−04
1e−02
1e−06
1e−04
1e−02
1e+00
1e−04
1e−03
1e−02
1e−01
1e+00
1e−02
1e+01
1e+04

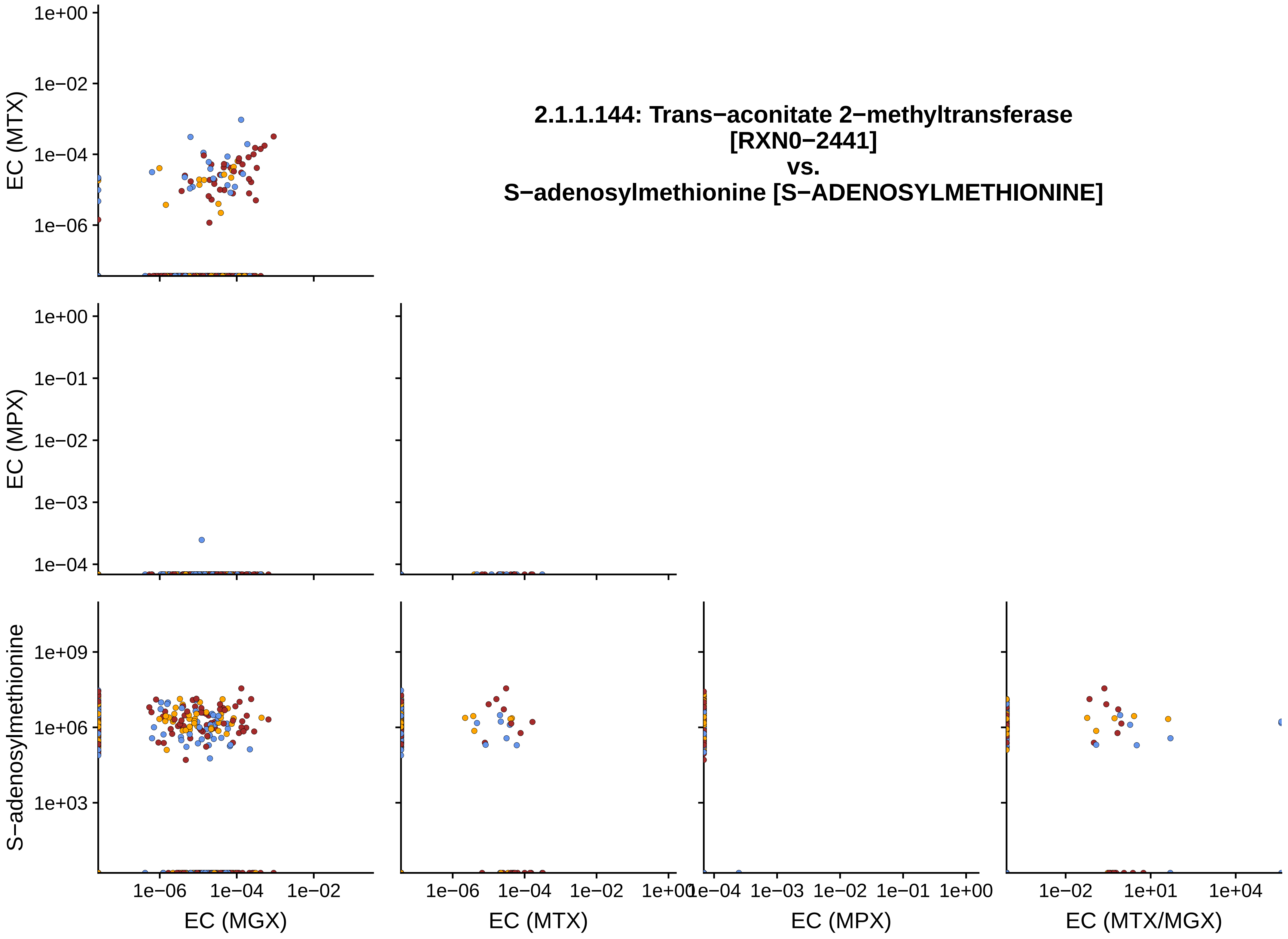

2.1.1.144: Trans−aconitate 2−methyltransferase
[RXN0−2441]
vs.
S−adenosylmethionine [S−ADENOSYLMETHIONINE]
EC (MTX)
EC (MPX)
S−adenosylmethionine
EC (MGX)
EC (MTX)
EC (MPX)
EC (MTX/MGX)
1e+00
1e−02
1e−04
1e−06
1e−01
1e−03
1e+09
1e+06
1e+03
1e+01
1e+04

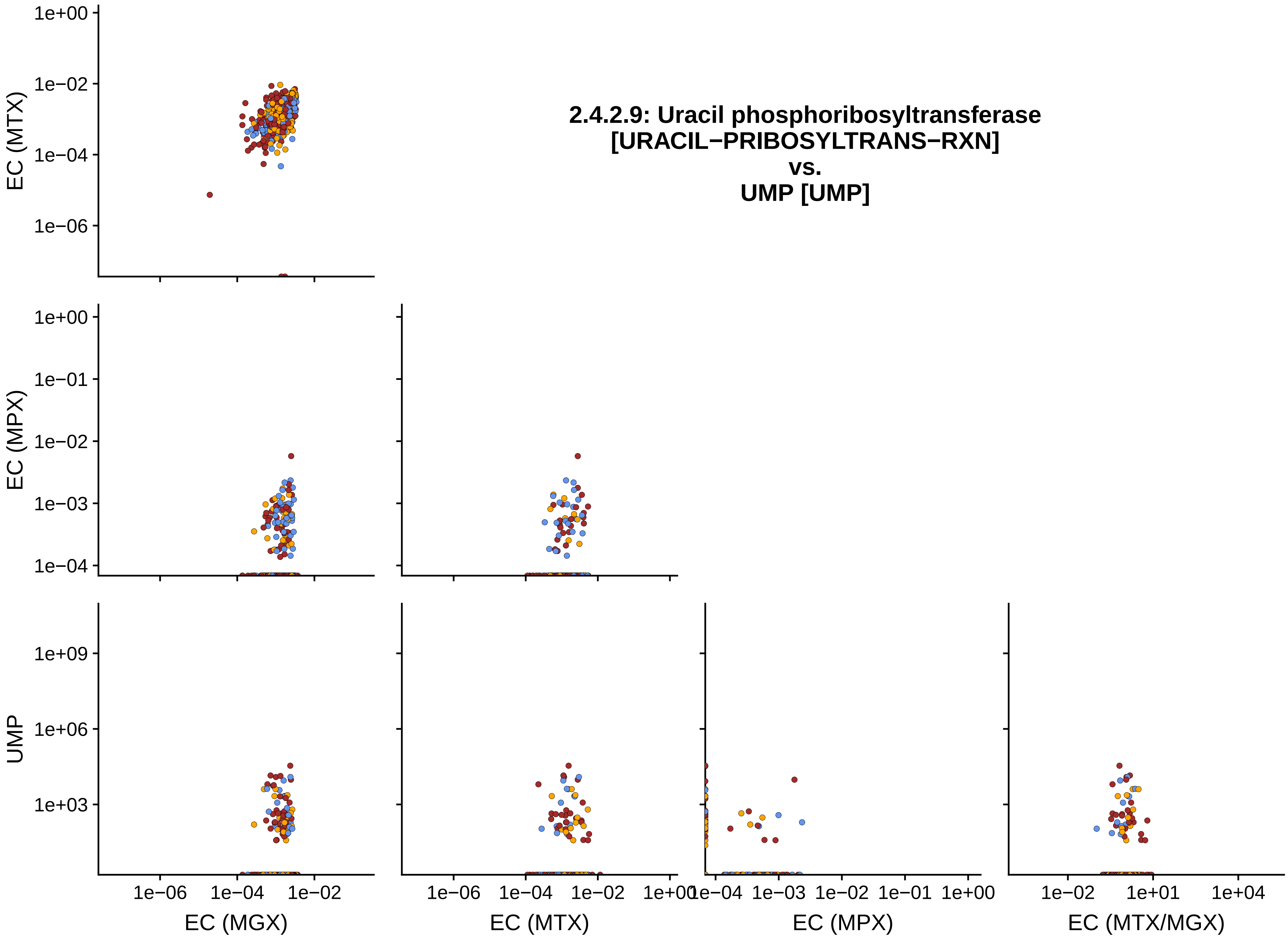

2.4.2.9: Uracil phosphoribosyltransferase
[URACIL−PRIBOSYLTRANS−RXN]
vs.
UMP [UMP]
EC (MTX)
EC (MPX)
UMP
EC (MGX)
EC (MTX)
EC (MPX)
EC (MTX/MGX)
1e+00
1e−02
1e−04
1e−06
1e+00
1e−01
1e−02
1e−03
1e−04
1e+09
1e+06
1e+03
1e−06
1e−04
1e−02
1e+00
1e−04
1e−03
1e−02
1e−01
1e+00
1e−02
1e+01
1e+04

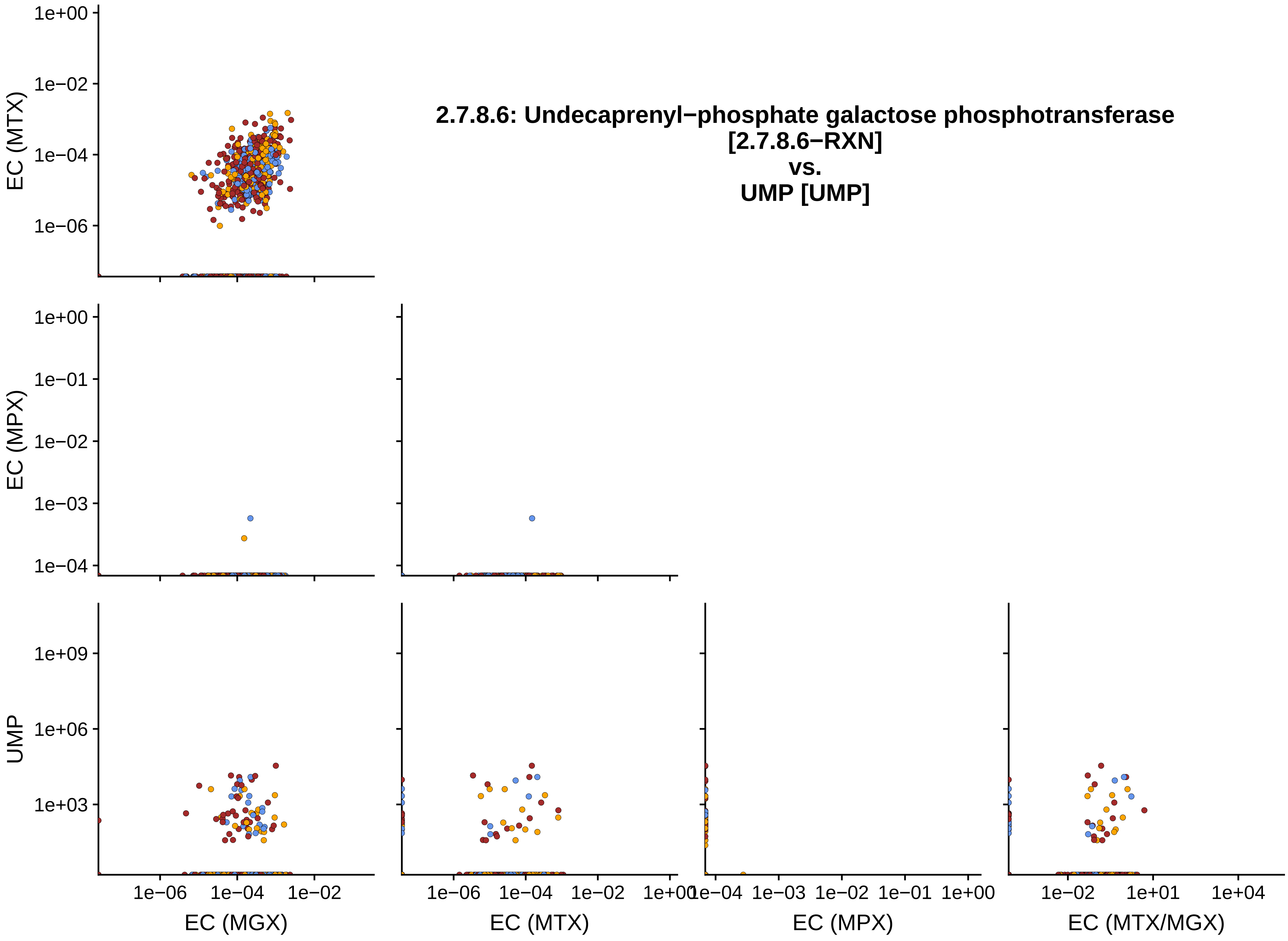
2.7.8.6: Undecaprenyl−phosphate galactose phosphotransferase
[2.7.8.6−RXN]
vs.
UMP [UMP]
EC (MTX)
EC (MPX)
UMP
EC (MGX)
EC (MTX)
EC (MPX)
EC (MTX/MGX)
1e+00
1e−02
1e−04
1e−06
1e−01
1e−03
1e+09
1e+06
1e+03
1e+01
1e+04

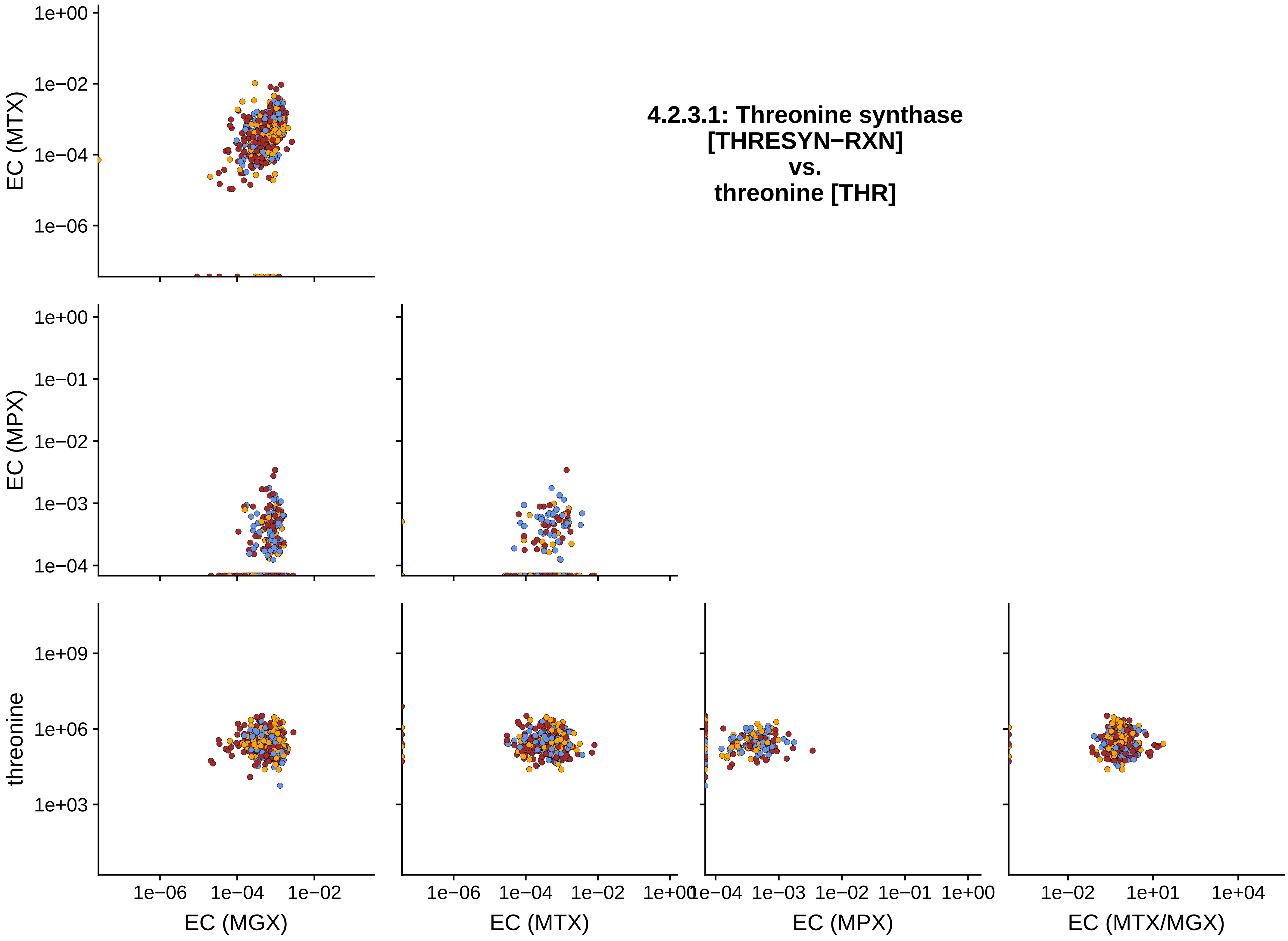
4.2.3.1: Threonine synthase
[THRESYN−RXN]
vs.
threonine [THR]
EC (MTX)
EC (MPX)
threonine
EC (MGX)
EC (MTX)
EC (MPX)
EC (MTX/MGX)
1e+00
1e−02
1e−04
1e−06
1e−01
1e−03
1e+09
1e+06
1e+03
1e+01
1e+04

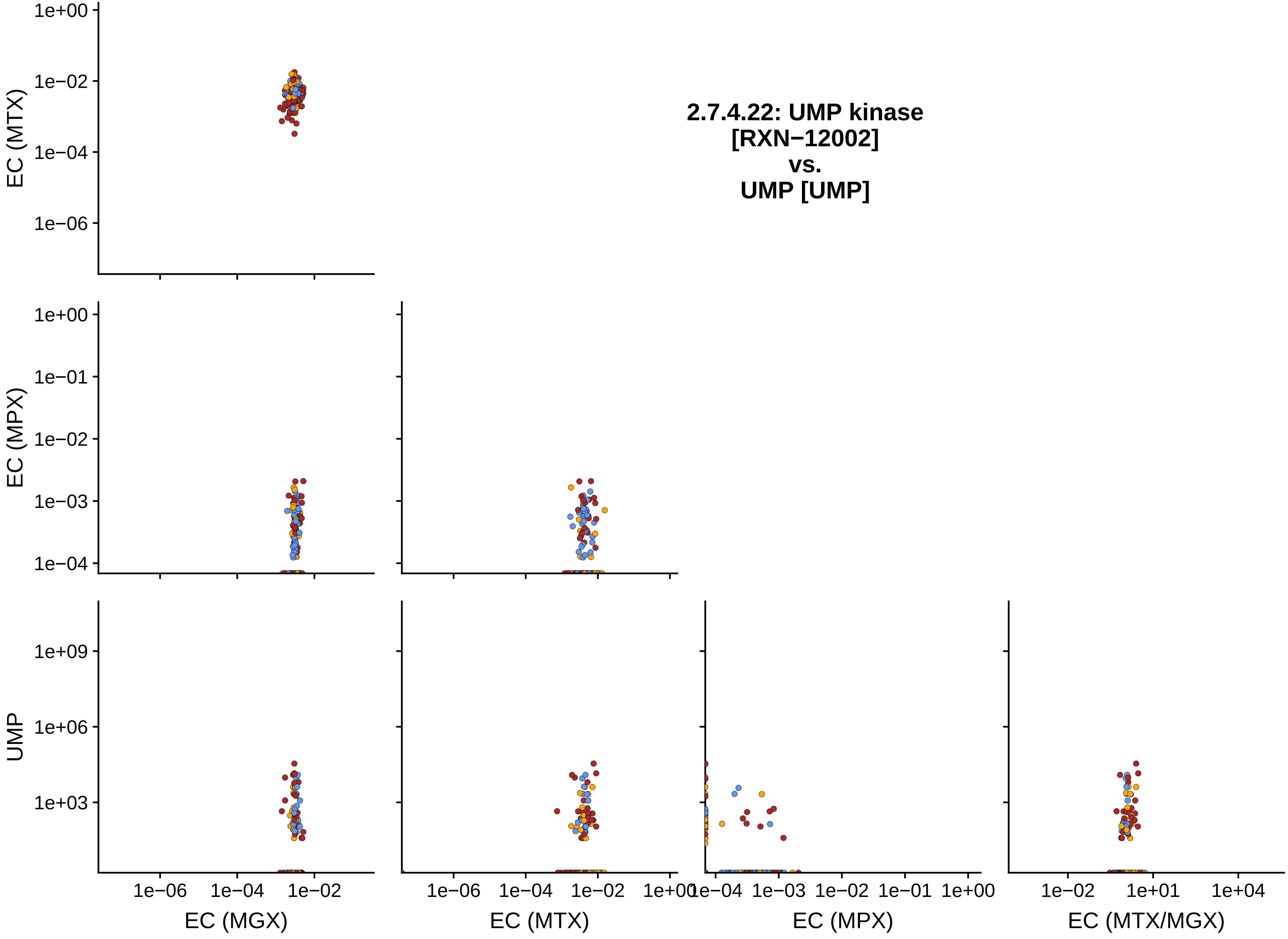
2.7.4.22: UMP kinase
[RXN−12002]
vs.
UMP [UMP]
EC (MTX)
EC (MPX)
UMP
EC (MGX)
EC (MTX)
EC (MPX)
EC (MTX/MGX)
1e+00
1e−02
1e−04
1e−06
1e+00
1e−01
1e−02
1e−03
1e−04
1e+09
1e+06
1e+03
1e−06
1e−04
1e−02
1e−06
1e−04
1e−02
1e+00
1e−04
1e−03
1e−02
1e−01
1e+00
1e−02
1e+01
1e+04

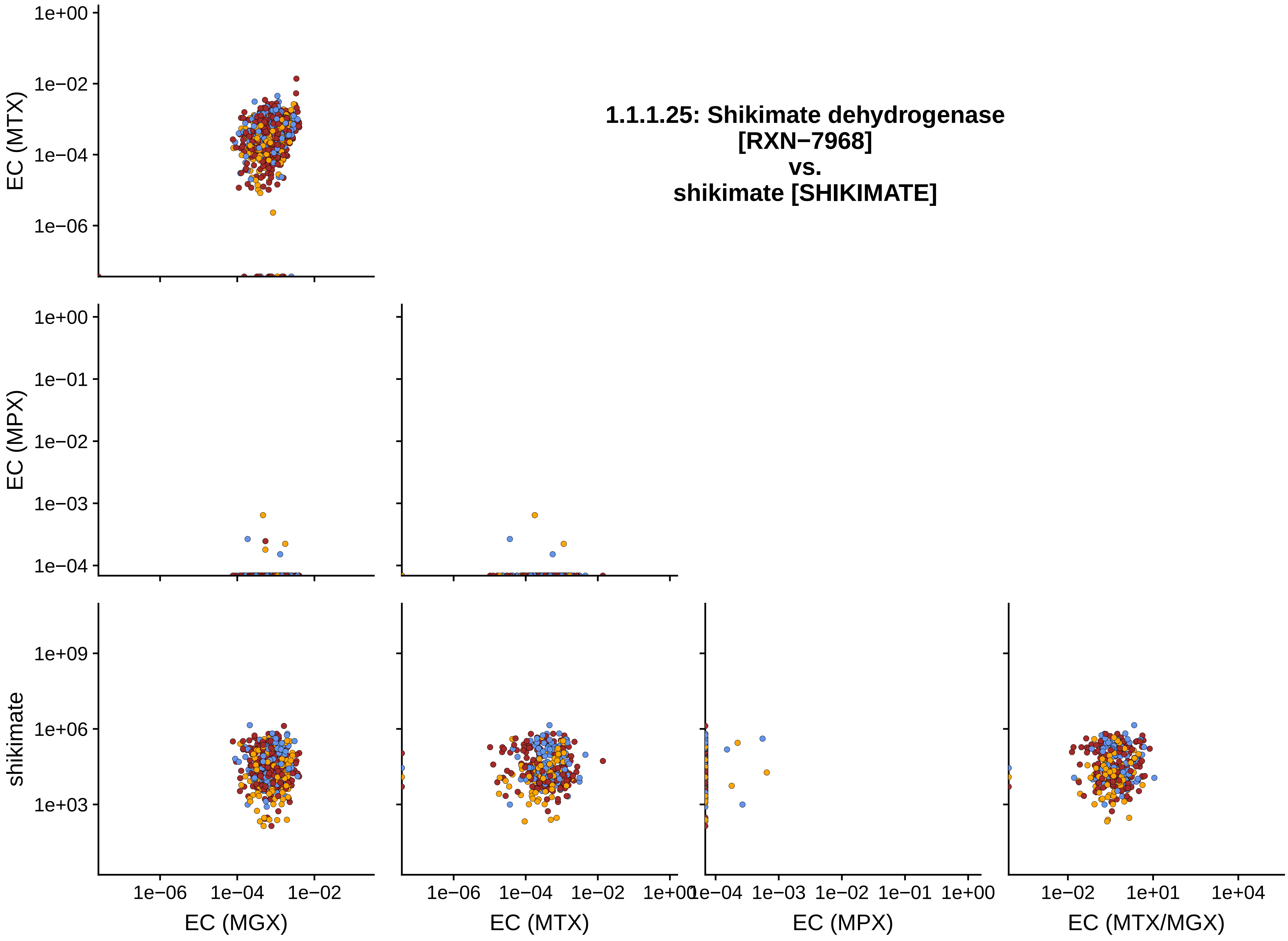

1.1.1.25: Shikimate dehydrogenase
[RXN−7968]
vs.
shikimate [SHIKIMATE]
EC (MTX)
EC (MPX)
shikimate
EC (MGX)
EC (MTX)
EC (MPX)
EC (MTX/MGX)
1e+00
1e−02
1e−04
1e−06
1e−01
1e−03
1e+09
1e+06
1e+03
1e+01
1e+04

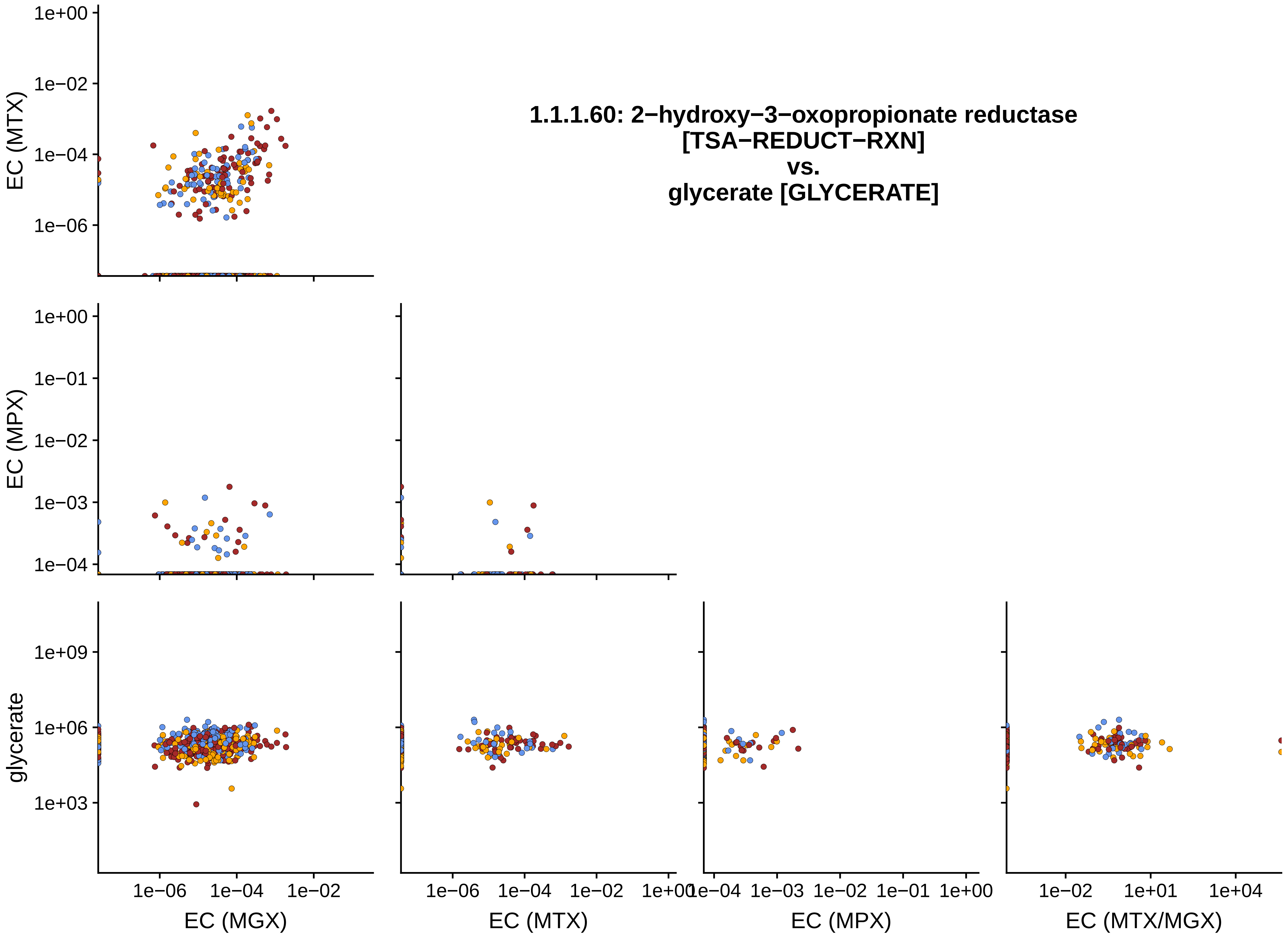
1.1.1.60: 2−hydroxy−3−oxopropionate reductase
[TSA−REDUCT−RXN]
vs.
glycerate [GLYCERATE]
EC (MTX)
EC (MPX)
glycerate
EC (MGX)
EC (MTX)
EC (MPX)
EC (MTX/MGX)
1e+00
1e−02
1e−04
1e−06
1e−01
1e−03
1e+09
1e+06
1e+03
1e+01
1e+04

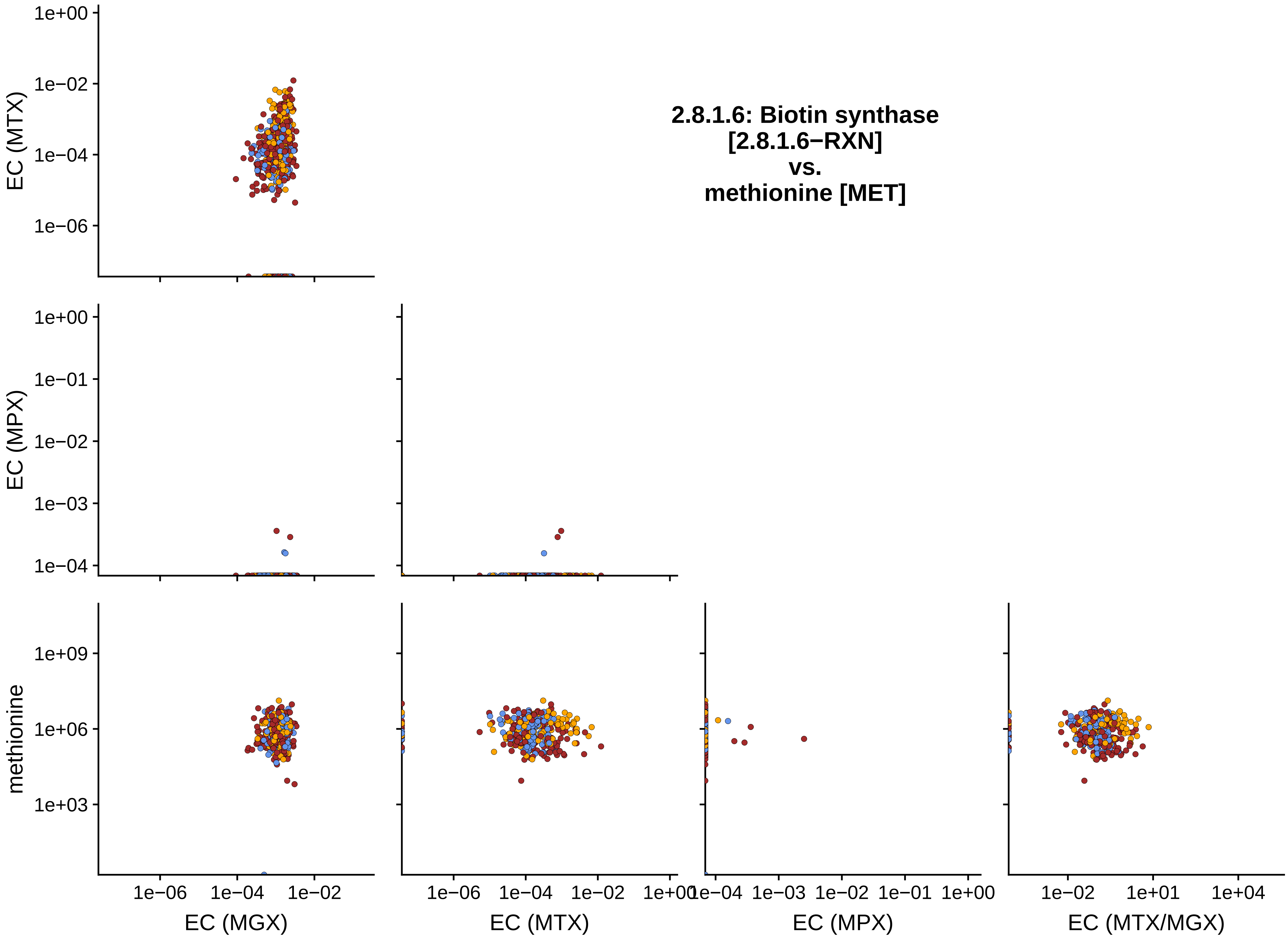

2.8.1.6: Biotin synthase
[2.8.1.6−RXN]
vs.
methionine [MET]
EC (MTX)
EC (MPX)
methionine
EC (MGX)
EC (MTX)
EC (MPX)
EC (MTX/MGX)
1e+00
1e−02
1e−04
1e−06
1e−01
1e−03
1e+09
1e+06
1e+03
1e+01
1e+04

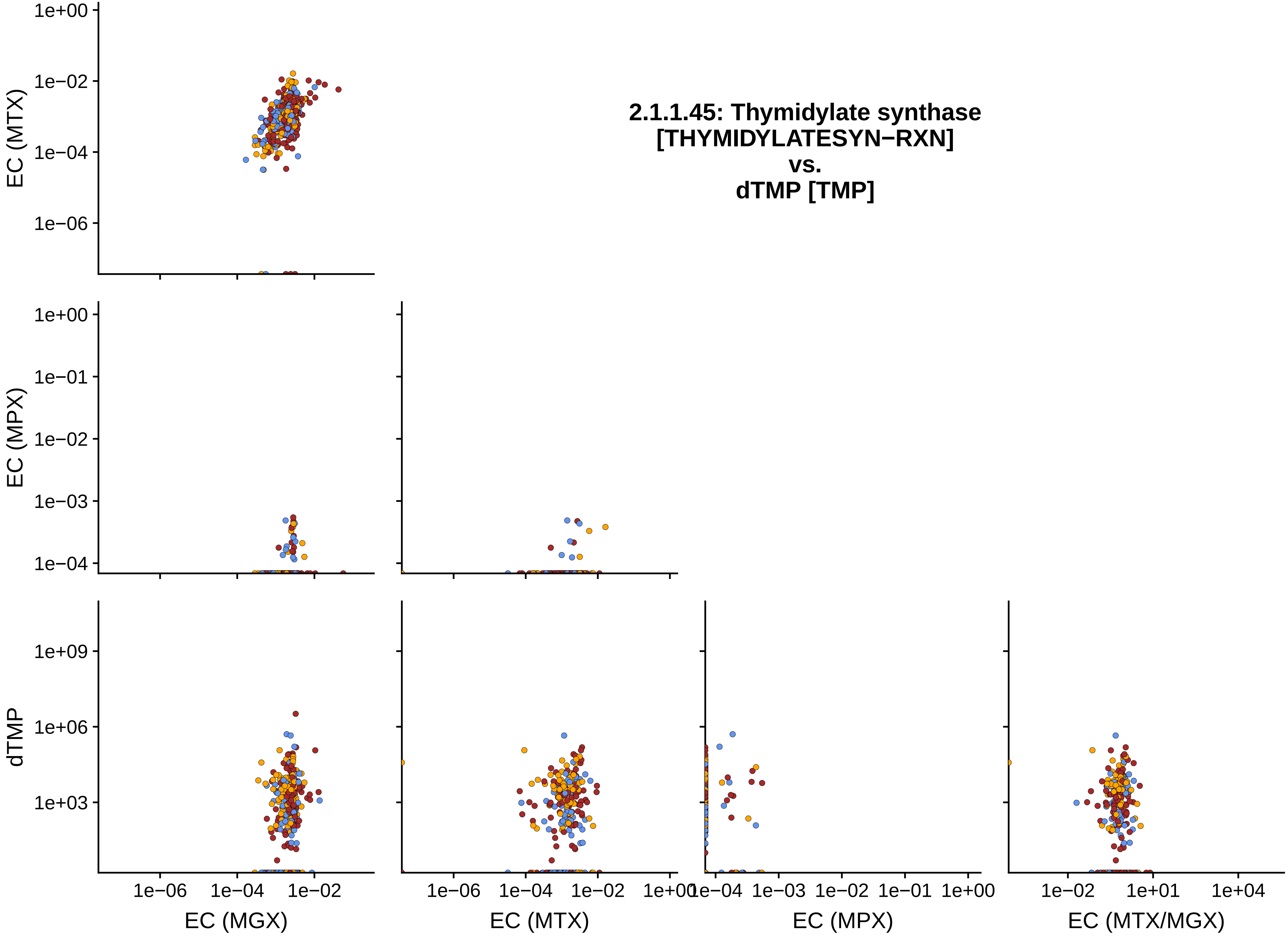

2.1.1.45: Thymidylate synthase
[THYMIDYLATESYN−RXN]
vs.
dTMP [TMP]
EC (MTX)
EC (MPX)
dTMP
EC (MGX)
EC (MTX)
EC (MPX)
EC (MTX/MGX)
1e+00
1e−02
1e−04
1e−06
1e−01
1e−03
1e+09
1e+06
1e+03
1e+01
1e+04

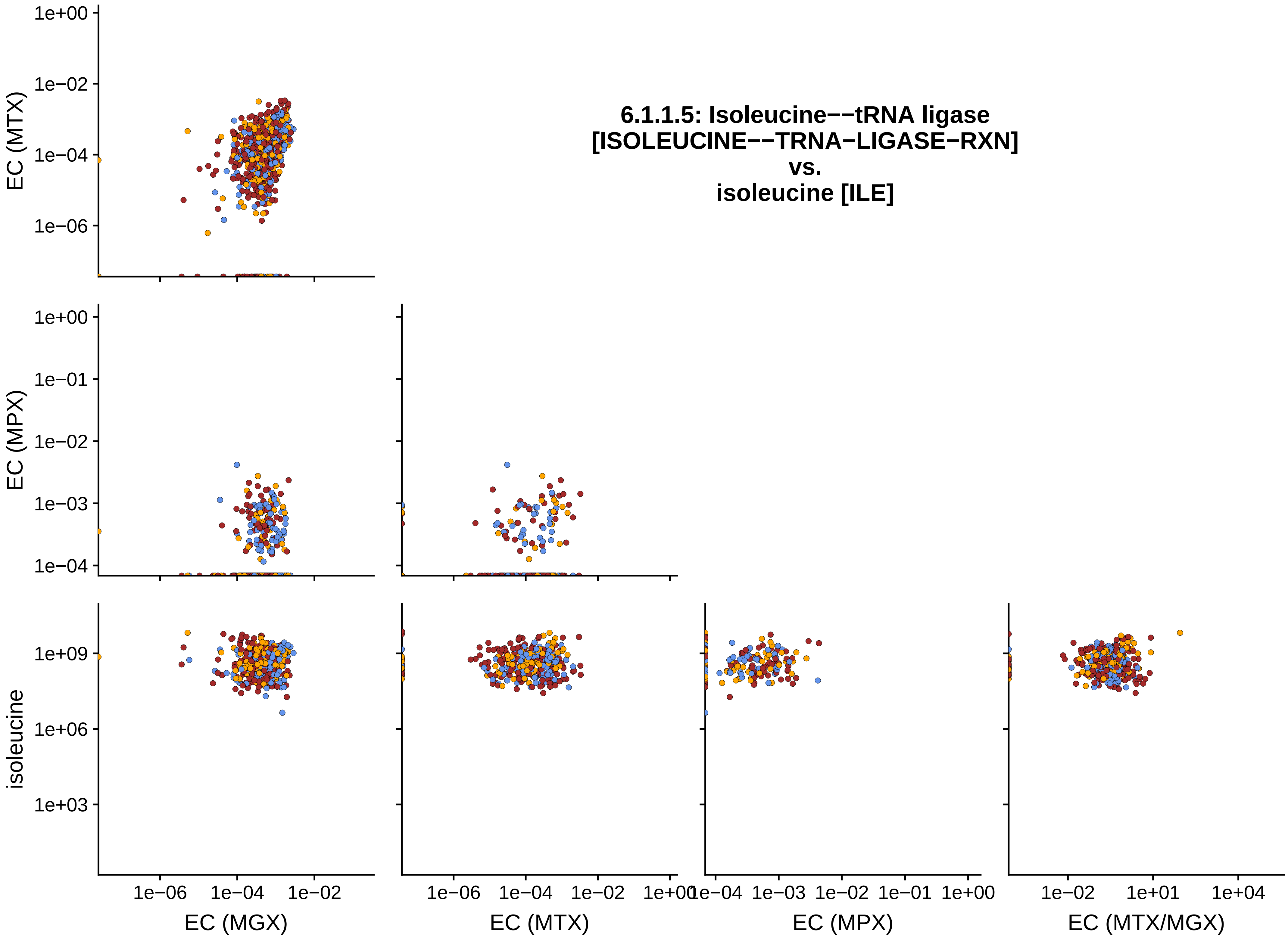

6.1.1.5: Isoleucine−−tRNA ligase
[ISOLEUCINE−−TRNA−LIGASE−RXN]
vs.
isoleucine [ILE]
EC (MTX)
EC (MPX)
isoleucine
EC (MGX)
EC (MTX)
EC (MPX)
EC (MTX/MGX)

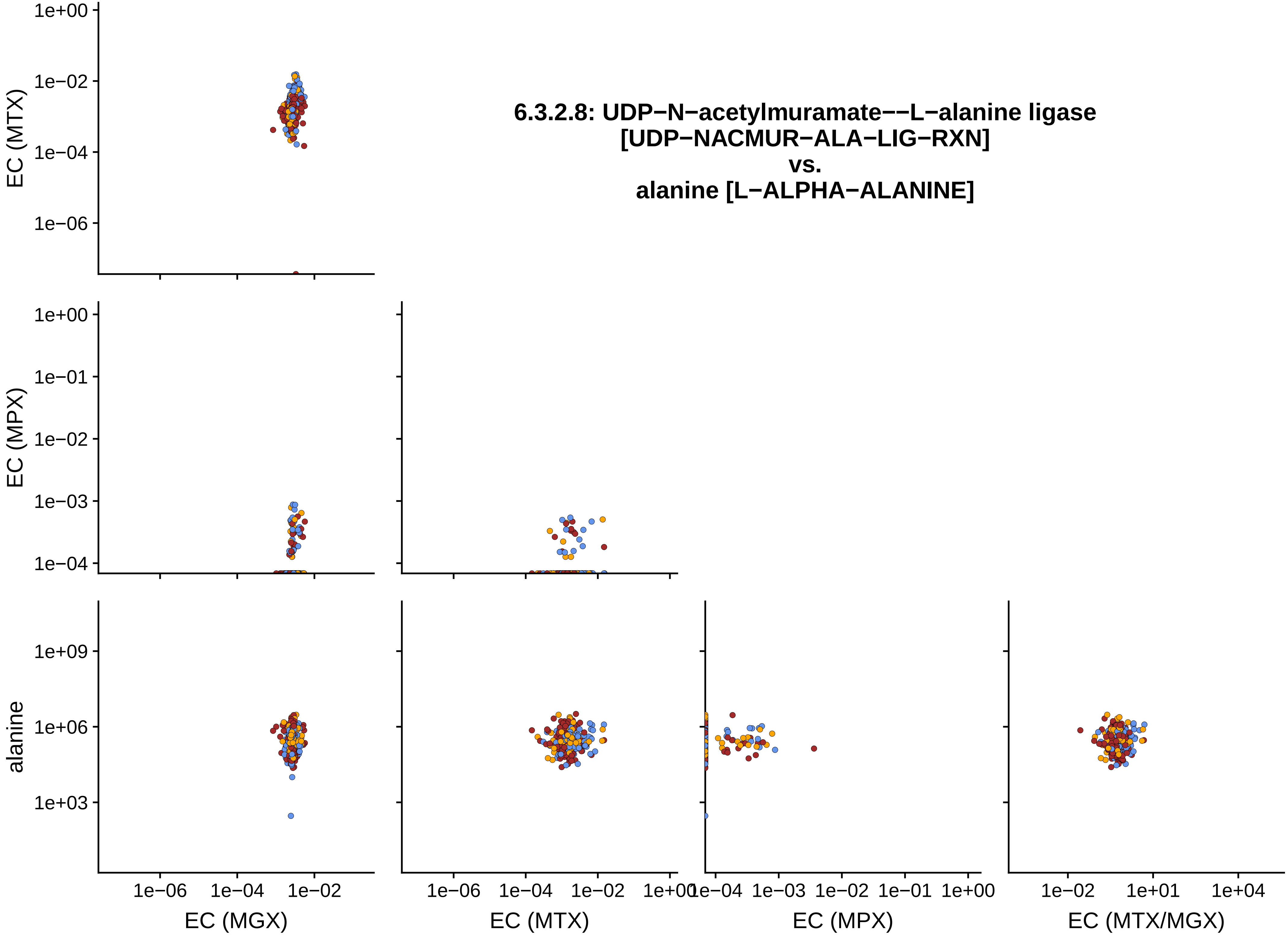
6.3.2.8: UDP−N−acetylmuramate−−L−alanine ligase
[UDP−NACMUR−ALA−LIG−RXN]
vs.
alanine [L−ALPHA−ALANINE]
EC (MTX)
EC (MPX)
alanine
EC (MGX)
EC (MTX)
EC (MPX)
EC (MTX/MGX)
1e+00
1e−02
1e−04
1e−06
1e−01
1e−03
1e+09
1e+06
1e+03
1e+01
1e+04

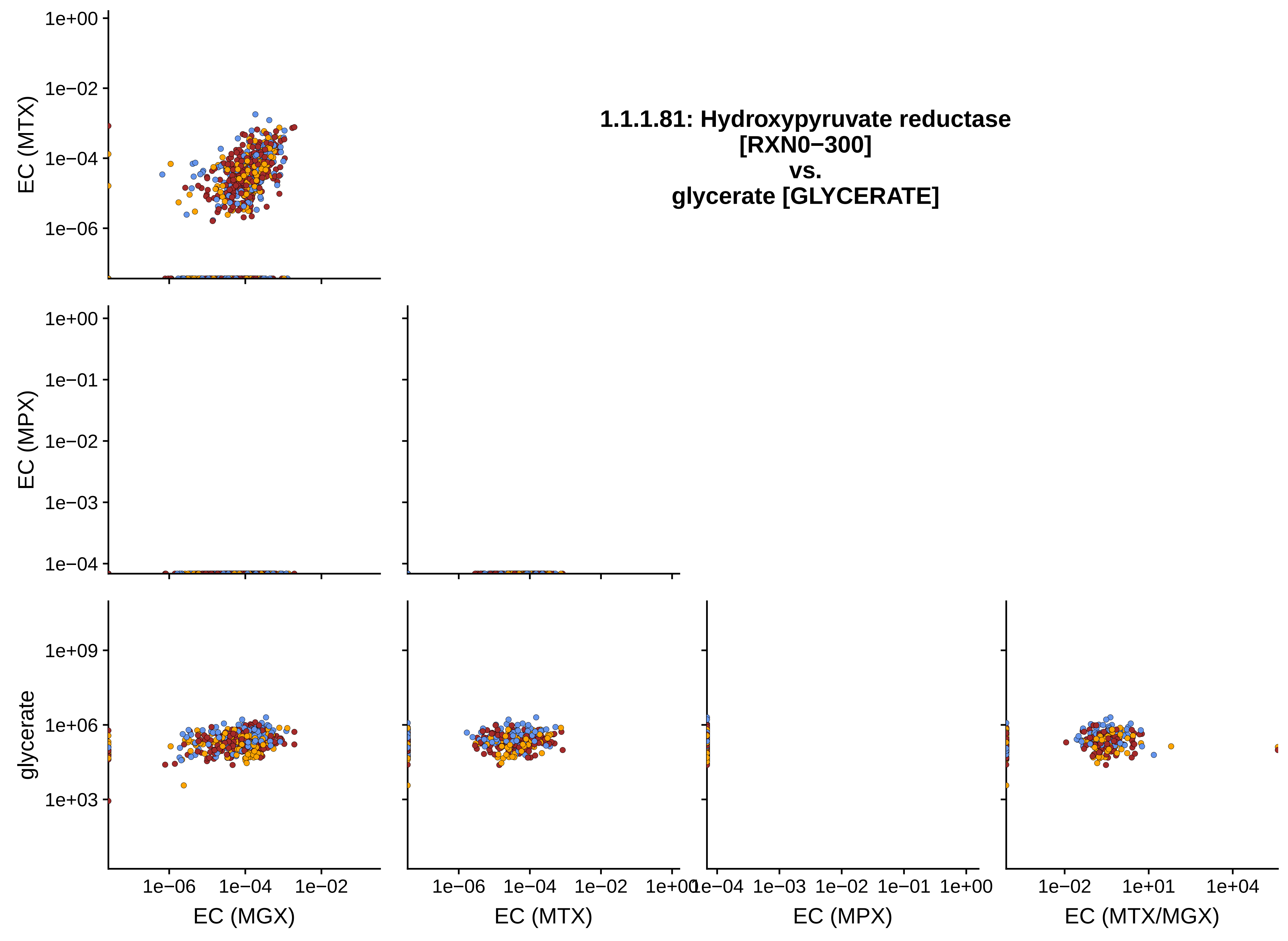
1.1.1.81: Hydroxypyruvate reductase
[RXN0−300]
vs.
glycerate [GLYCERATE]
EC (MTX)
EC (MPX)
glycerate
EC (MGX)
EC (MTX)
EC (MPX)
EC (MTX/MGX)
1e+00
1e−02
1e−04
1e−06
1e−01
1e−03
1e+09
1e+06
1e+03
1e+01
1e+04

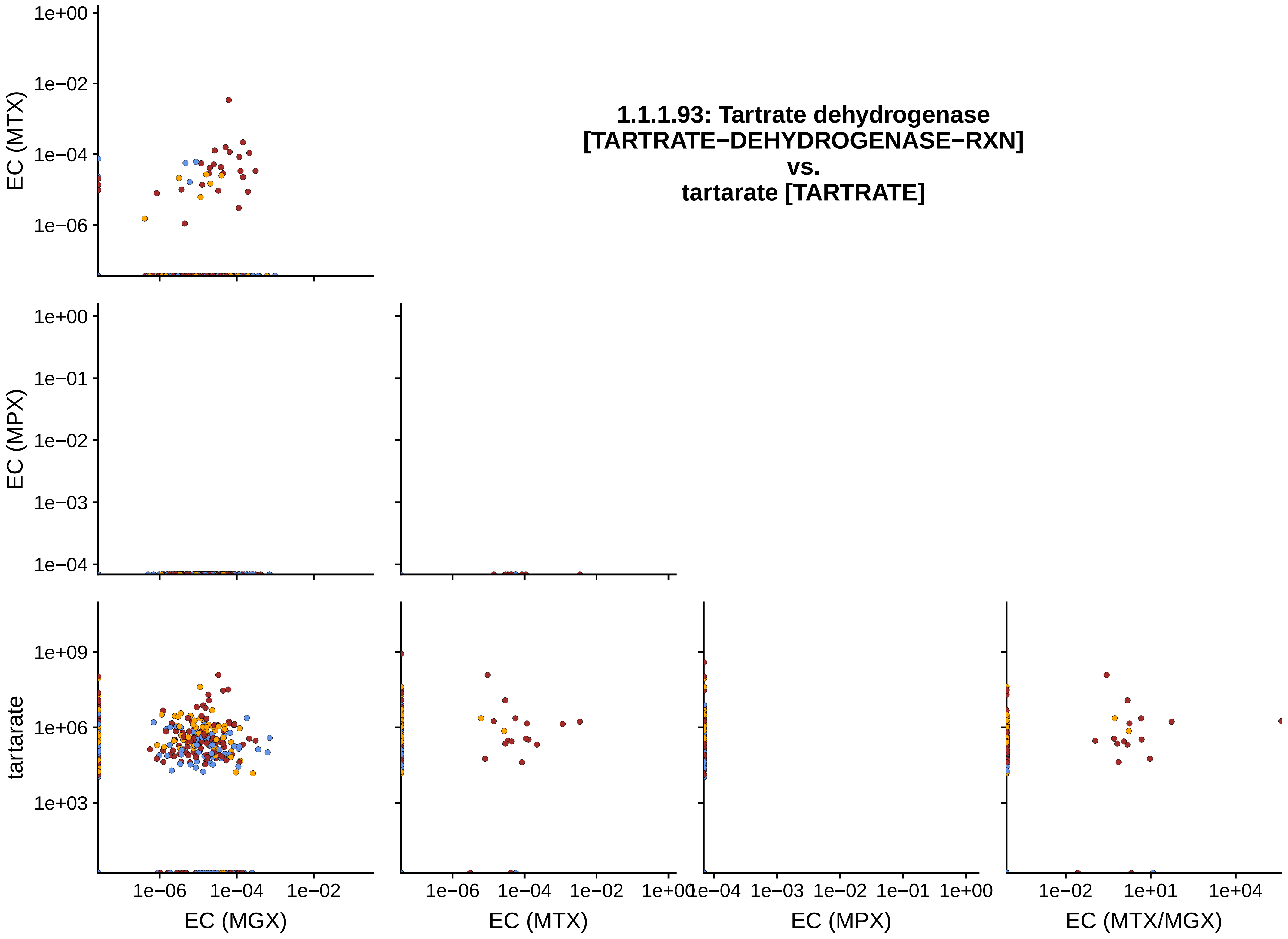

1.1.1.93: Tartrate dehydrogenase
[TARTRATE−DEHYDROGENASE−RXN]
vs.
tartarate [TARTRATE]
EC (MTX)
EC (MPX)
tartarate
EC (MGX)
EC (MTX)
EC (MPX)
EC (MTX/MGX)
1e+00
1e−02
1e−04
1e−06
1e−01
1e−03
1e+09
1e+06
1e+03
1e+01
1e+04

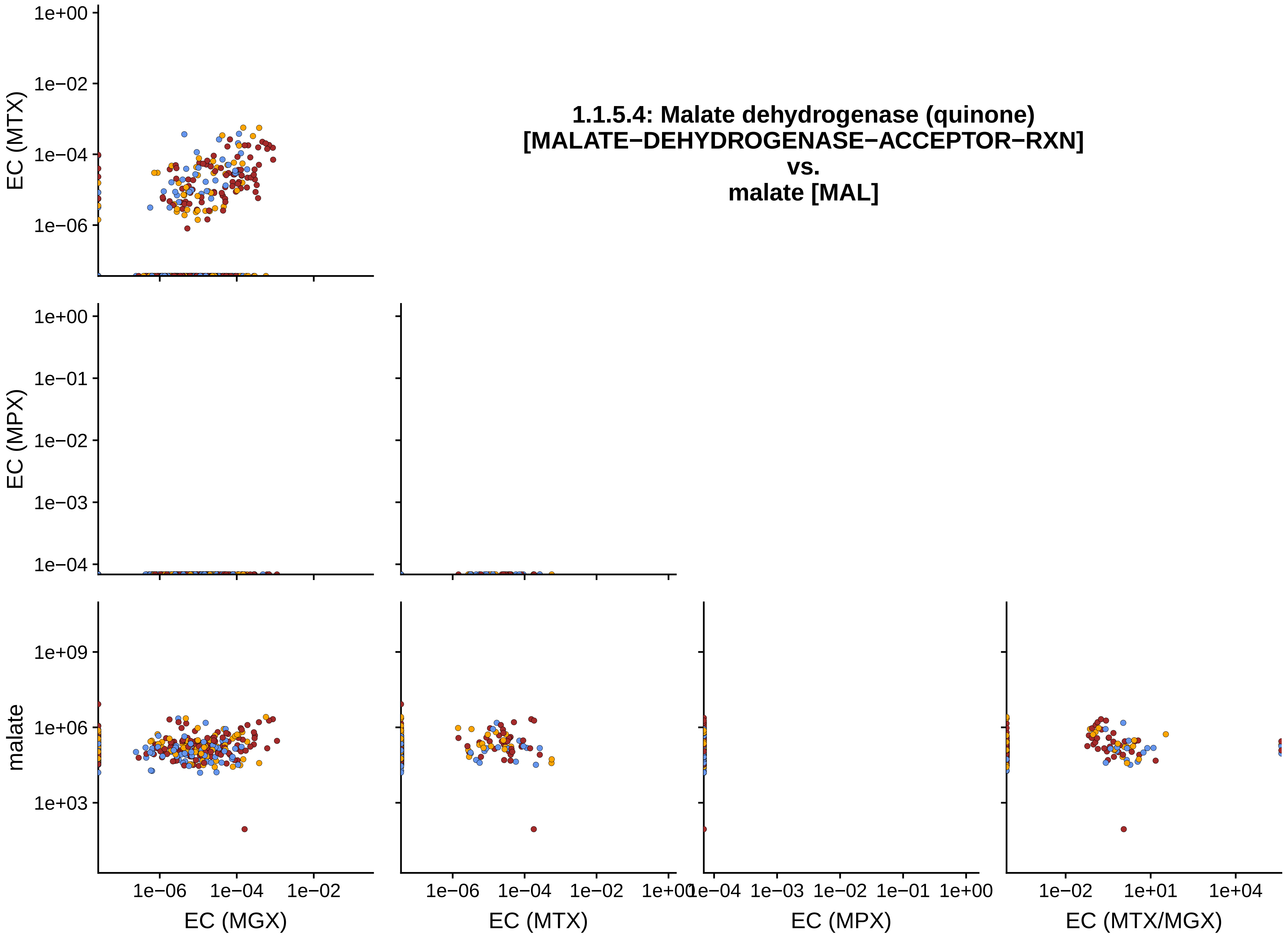
1.1.5.4: Malate dehydrogenase (quinone)
[MALATE−DEHYDROGENASE−ACCEPTOR−RXN]
vs.
malate [MAL]
EC (MTX)
EC (MPX)
malate
EC (MGX)
EC (MTX)
EC (MPX)
EC (MTX/MGX)
1e+00
1e−02
1e−04
1e−06
1e−01
1e−03
1e+09
1e+06
1e+03
1e+01
1e+04

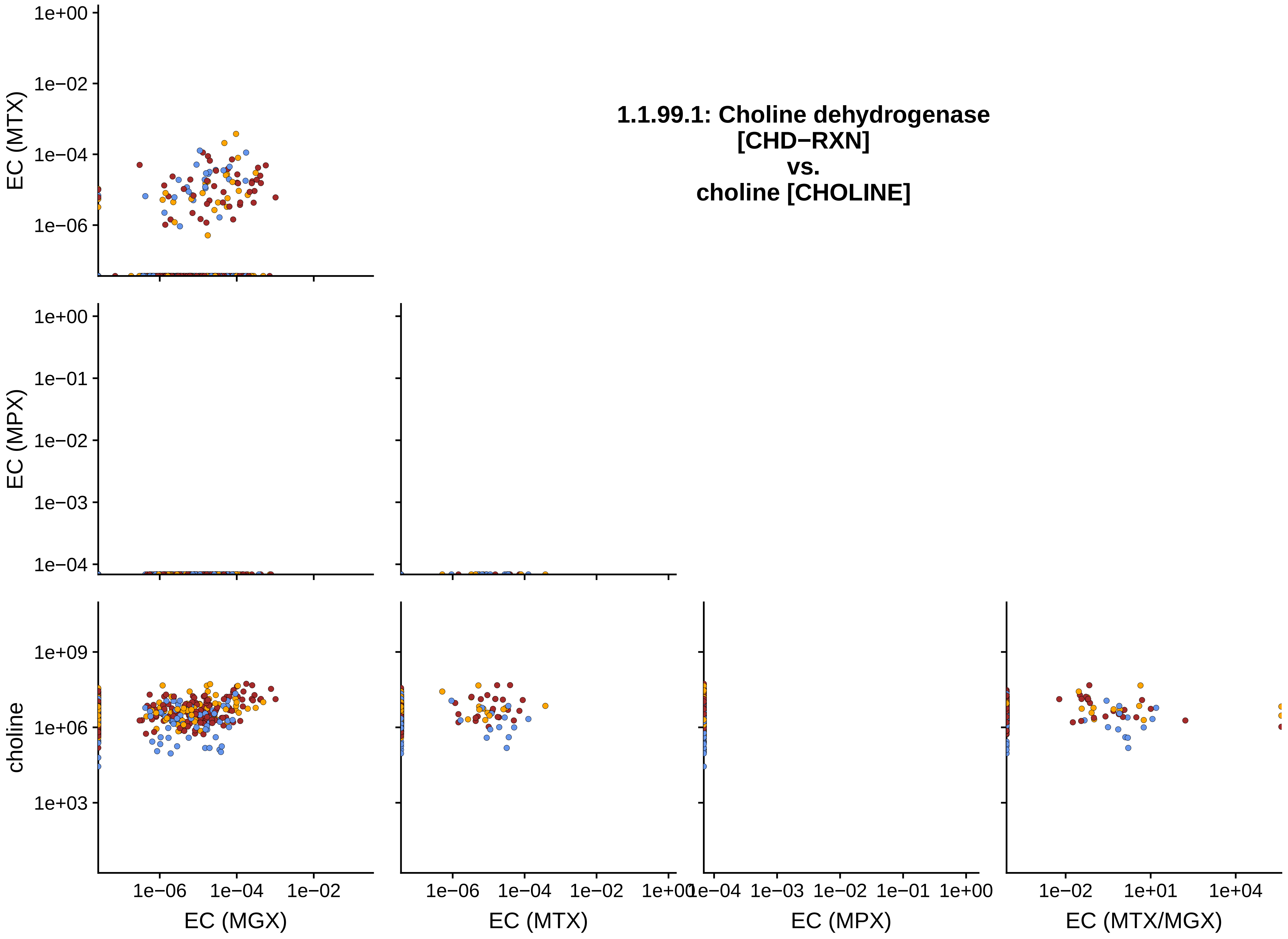

1.1.99.1: Choline dehydrogenase
[CHD−RXN]
vs.
choline [CHOLINE]
EC (MTX)
EC (MPX)
choline
EC (MGX)
EC (MTX)
EC (MPX)
EC (MTX/MGX)
1e+00
1e−02
1e−04
1e−06
1e−01
1e−03
1e+09
1e+06
1e+03
1e+01
1e+04

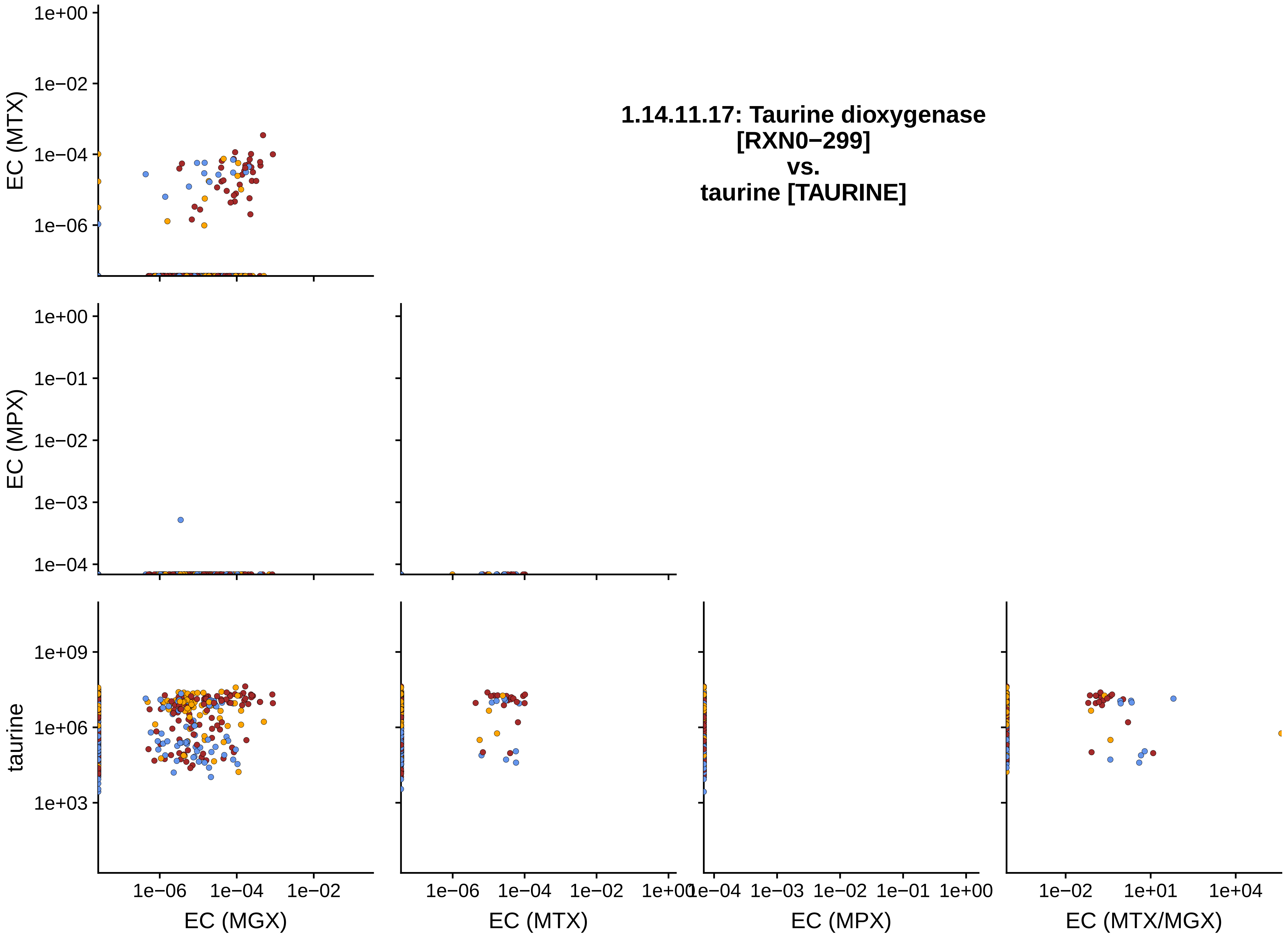

1.14.11.17: Taurine dioxygenase
[RXN0−299]
vs.
taurine [TAURINE]
EC (MTX)
EC (MPX)
taurine
EC (MGX)
EC (MTX)
EC (MPX)
EC (MTX/MGX)
1e+00
1e−02
1e−04
1e−06
1e−01
1e−03
1e+09
1e+06
1e+03
1e+01
1e+04

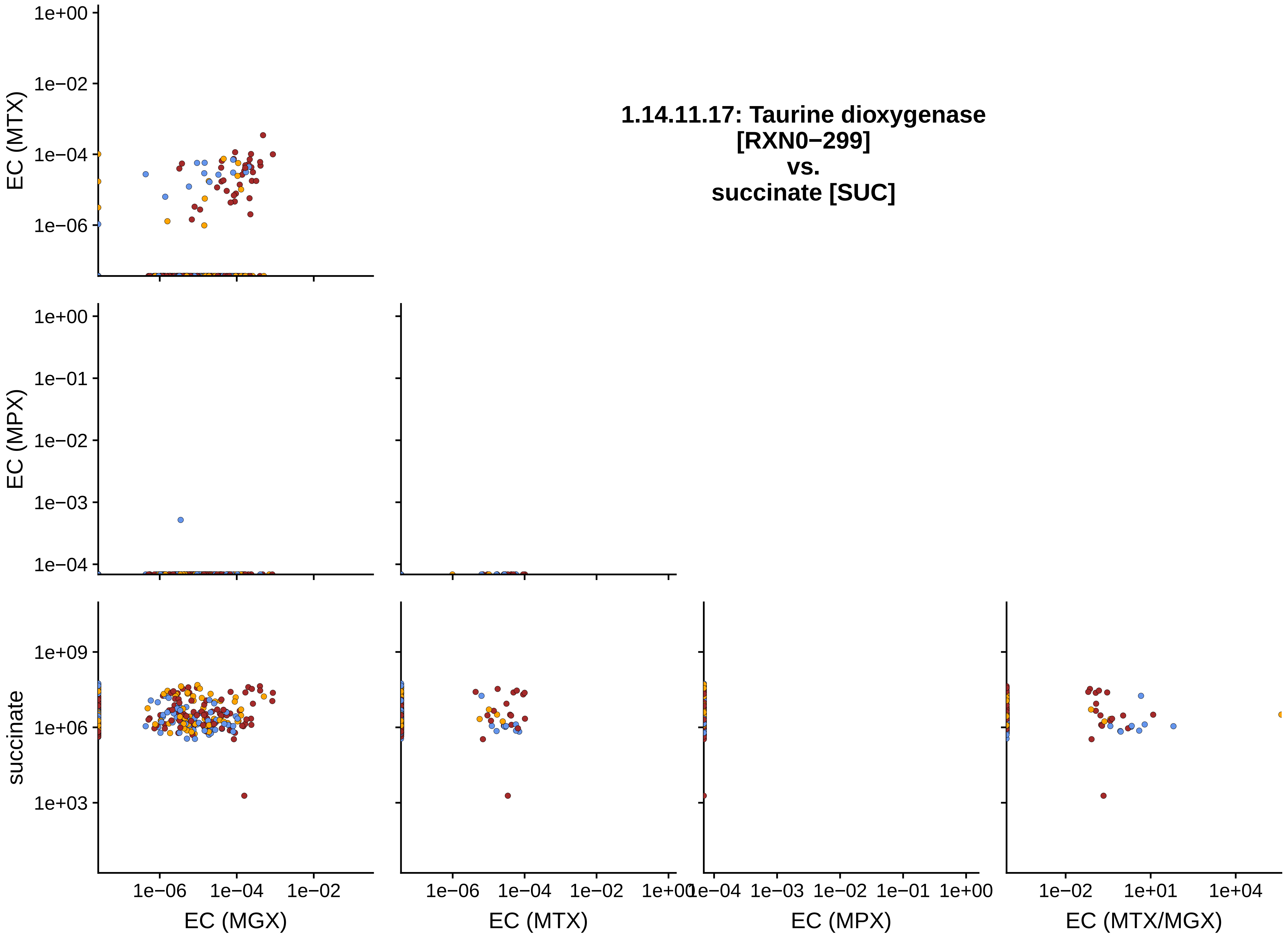
1.14.11.17: Taurine dioxygenase
[RXN0−299]
vs.
succinate [SUC]
EC (MTX)
EC (MPX)
succinate
EC (MGX)
EC (MTX)
EC (MPX)
EC (MTX/MGX)
1e+00
1e−02
1e−04
1e−06
1e−01
1e−03
1e+09
1e+06
1e+03
1e+01
1e+04

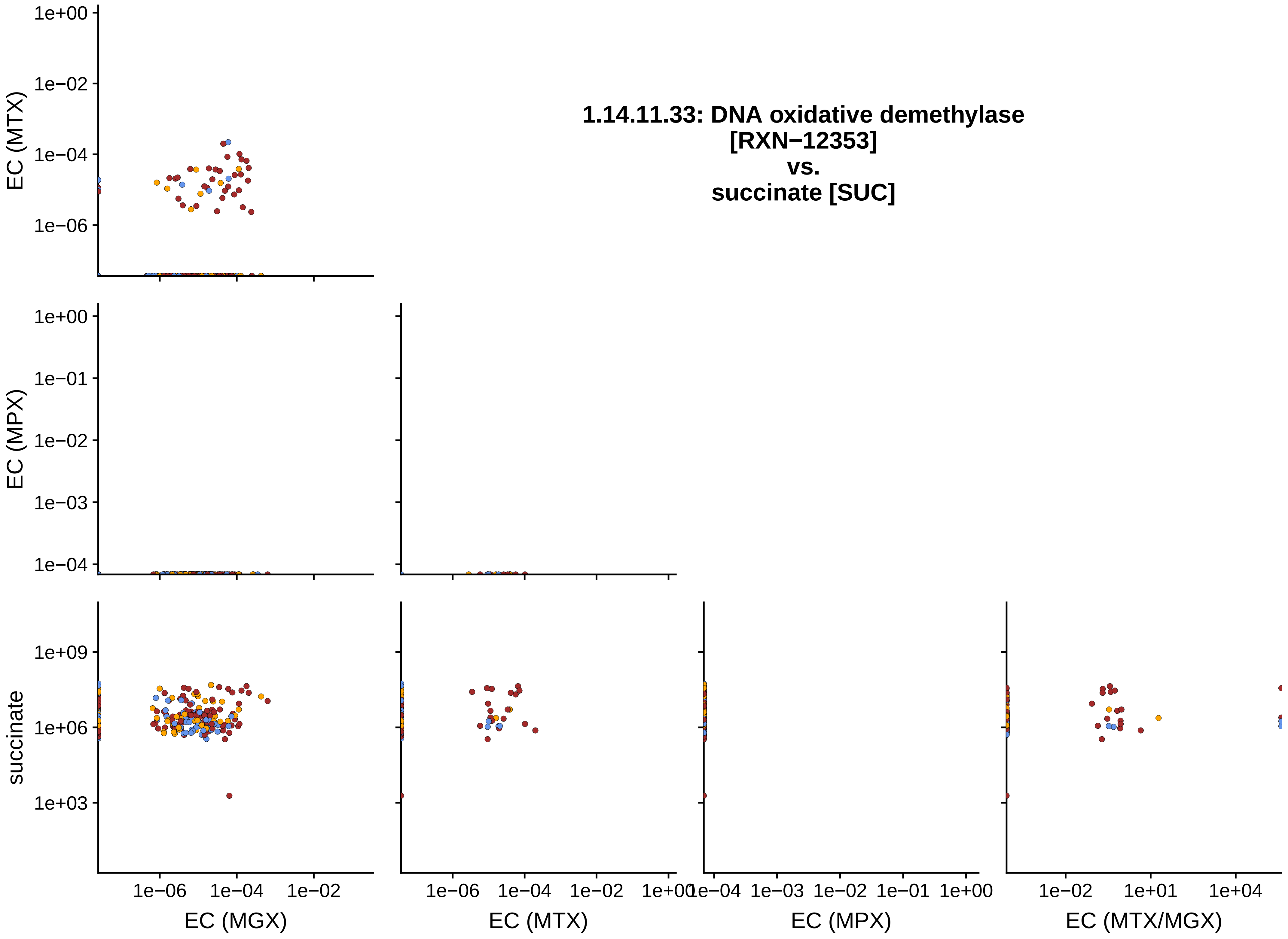

1.14.11.33: DNA oxidative demethylase
[RXN−12353]
vs.
succinate [SUC]
EC (MTX)
1e+00
1e−02
1e−04
1e−06
EC (MPX)
1e+00
1e−01
1e−02
1e−03
1e−04
succinate
1e+09
1e+06
1e+03
1e−06
1e−04
1e−02
EC (MGX)
1e−06
1e−04
1e−02
1e+00
EC (MTX)
1e−04
1e−03
1e−02
1e−01
1e+00
EC (MPX)
1e−02
1e+01
1e+04
EC (MTX/MGX)

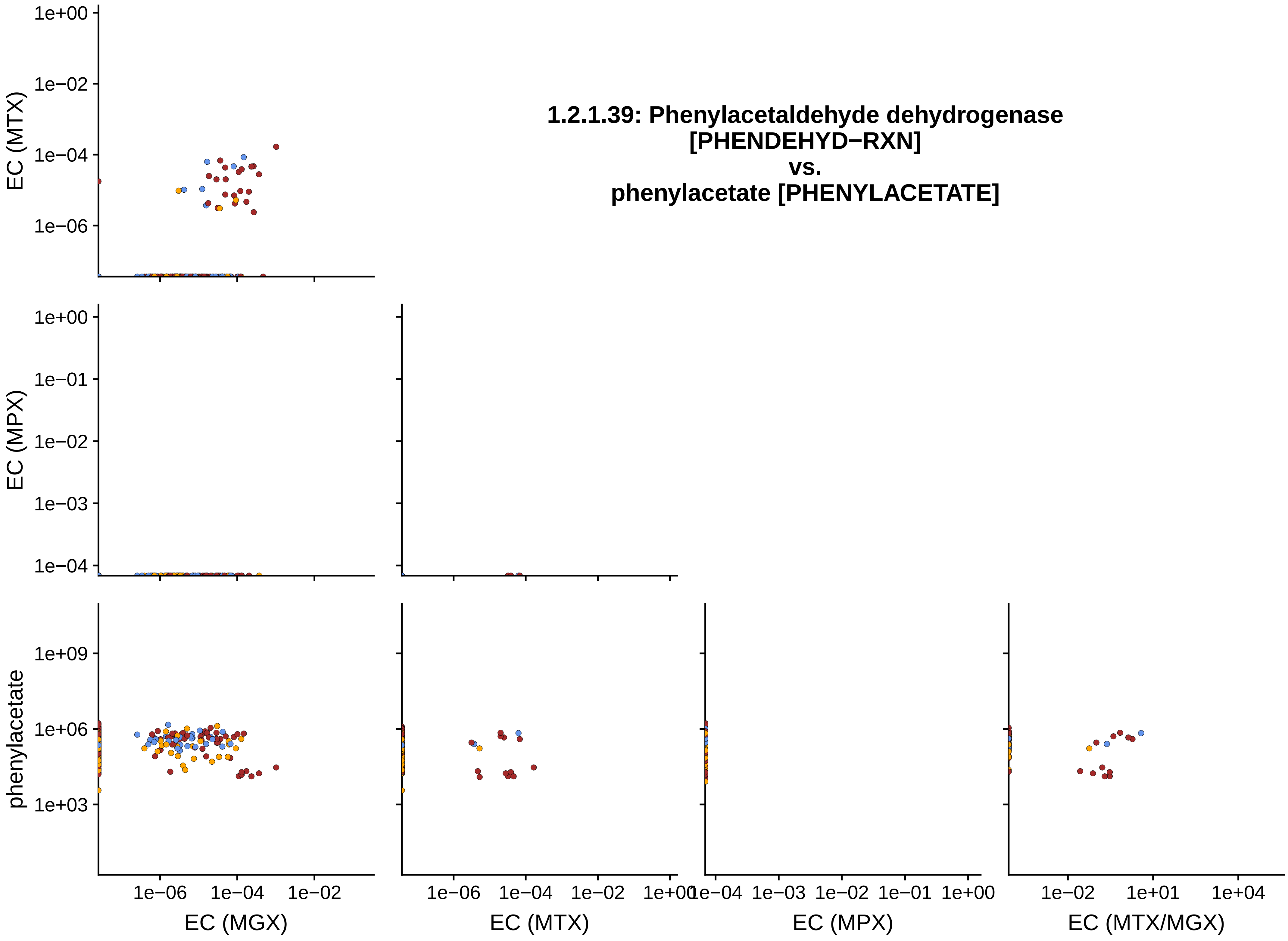

1.2.1.39: Phenylacetaldehyde dehydrogenase
[PHENDEHYD−RXN]
vs.
phenylacetate [PHENYLACETATE]
EC (MTX)
EC (MPX)
phenylacetate
EC (MGX)
EC (MTX)
EC (MPX)
EC (MTX/MGX)
1e+00
1e−02
1e−04
1e−06
1e−01
1e−03
1e+09
1e+06
1e+03
1e+01
1e+04

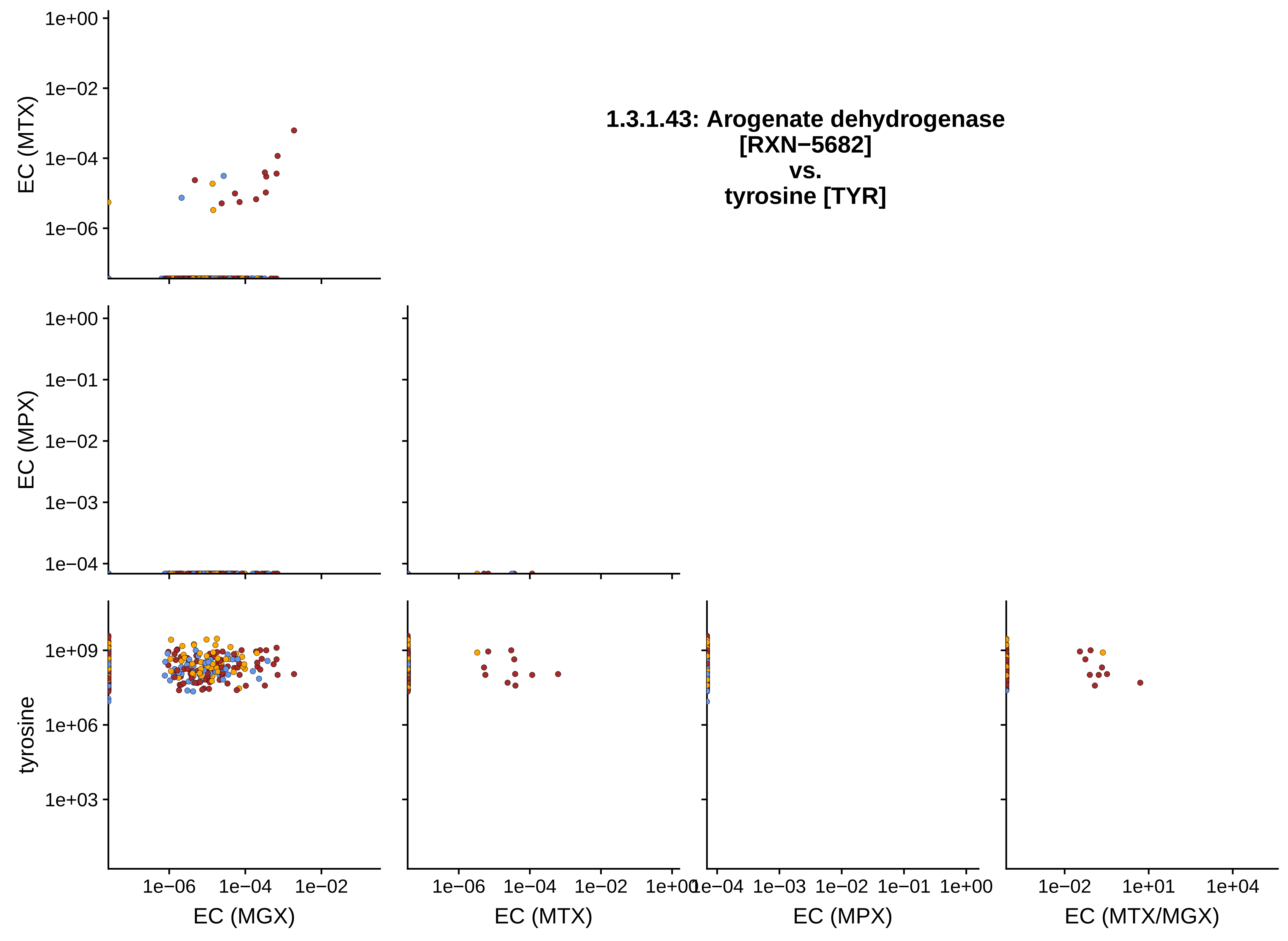
1.3.1.43: Arogenate dehydrogenase
[RXN−5682]
vs.
tyrosine [TYR]
EC (MTX)
EC (MPX)
tyrosine
EC (MGX)
EC (MTX)
EC (MPX)
EC (MTX/MGX)
1e+00
1e−02
1e−04
1e−06
1e−01
1e−03
1e+09
1e+06
1e+03
1e+01
1e+04

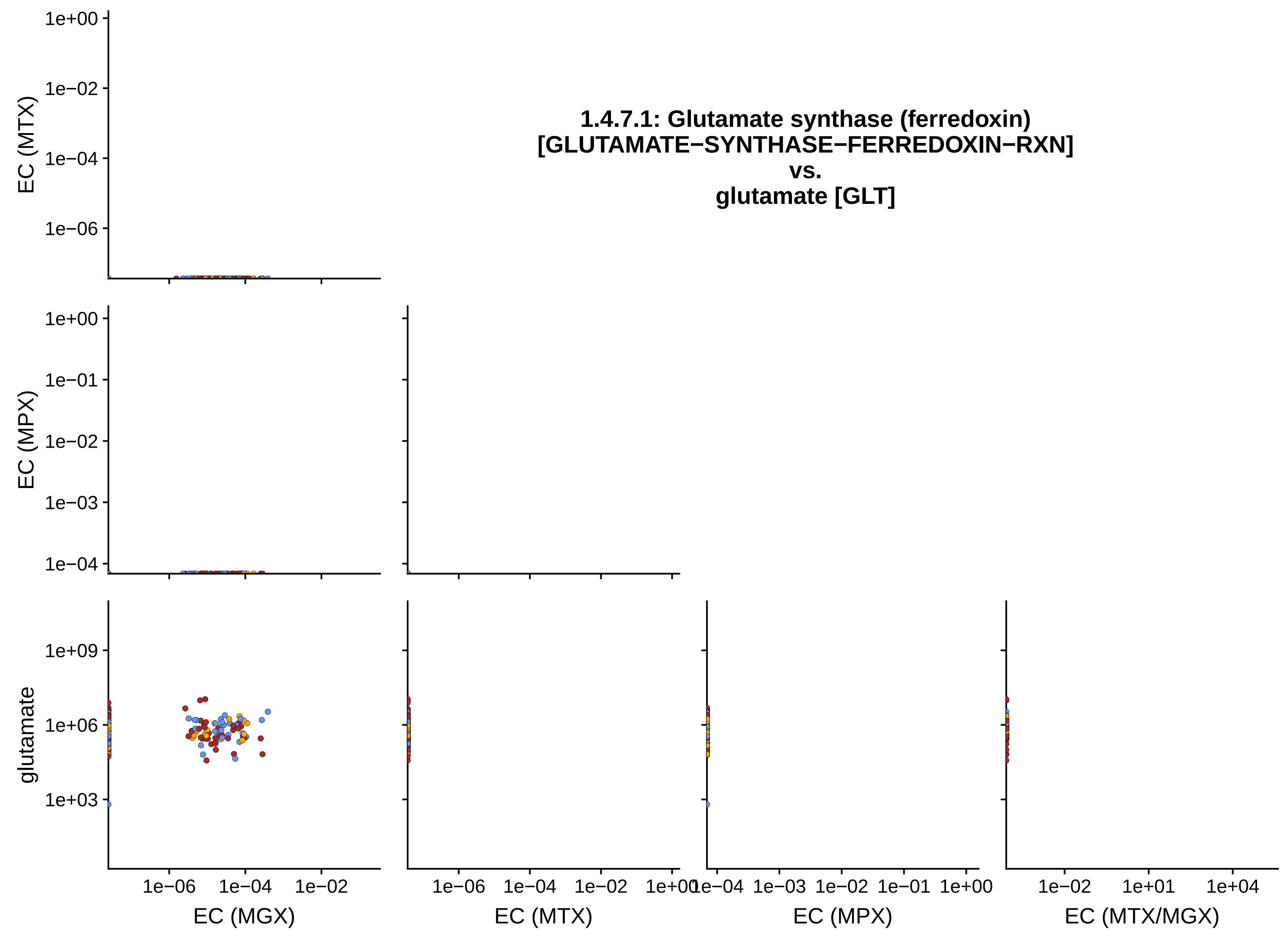

1.4.7.1: Glutamate synthase (ferredoxin)
[GLUTAMATE−SYNTHASE−FERREDOXIN−RXN]
vs.
glutamate [GLT]
EC (MTX)
EC (MPX)
glutamate
EC (MGX)
EC (MTX)
EC (MPX)
EC (MTX/MGX)
1e+00
1e−02
1e−04
1e−06
1e+00
1e−01
1e−02
1e−03
1e−04
1e+09
1e+06
1e+03
1e−06
1e−04
1e−02
1e−06
1e−04
1e−02
1e+00
1e−04
1e−03
1e−02
1e−01
1e+00
1e−02
1e+01
1e+04

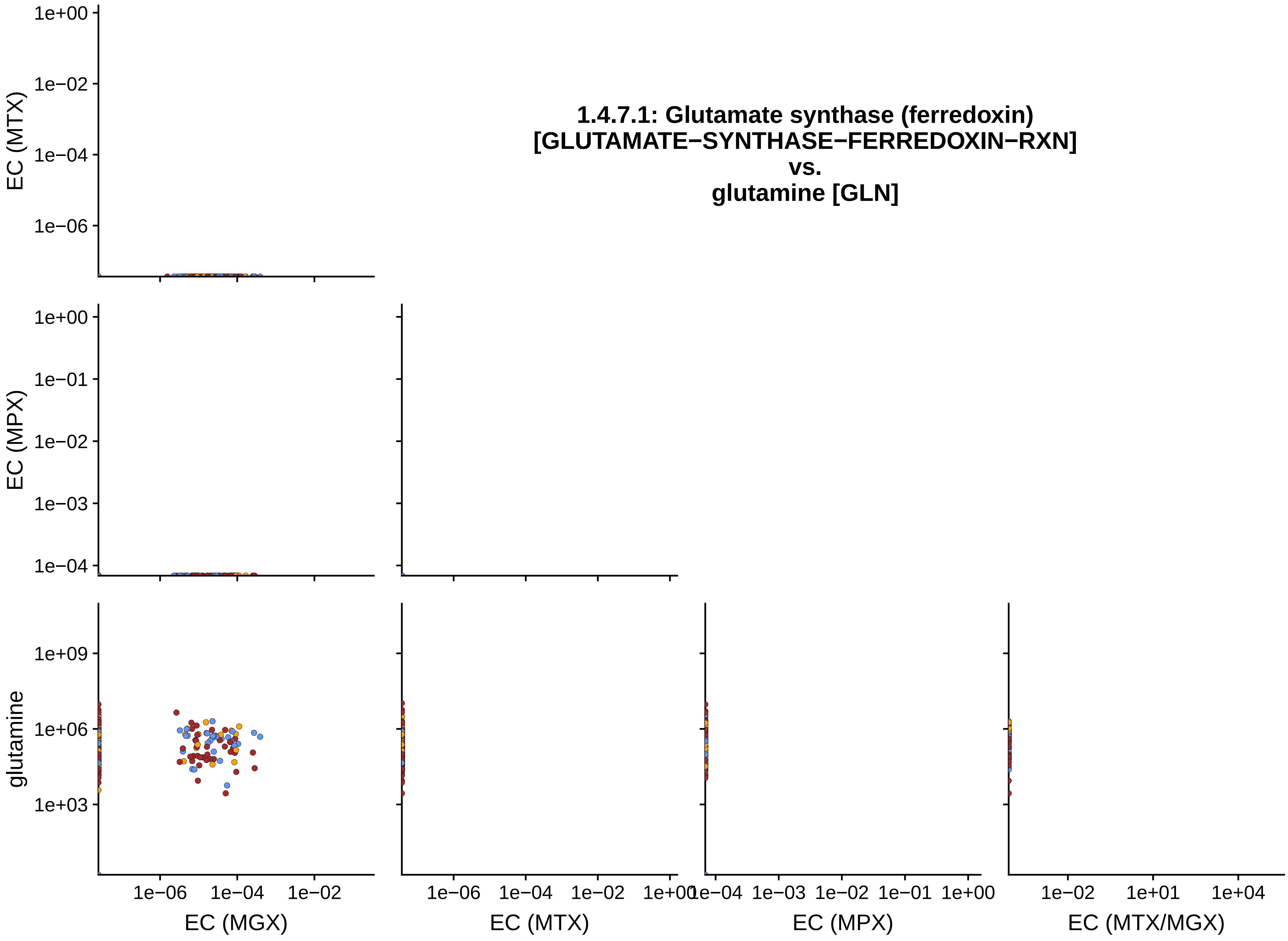

1.4.7.1: Glutamate synthase (ferredoxin)
[GLUTAMATE−SYNTHASE−FERREDOXIN−RXN]
vs.
glutamine [GLN]
EC (MTX)
EC (MPX)
glutamine
EC (MGX)
EC (MTX)
EC (MPX)
EC (MTX/MGX)
1e+00
1e−02
1e−04
1e−06
1e+00
1e−01
1e−02
1e−03
1e−04
1e+09
1e+06
1e+03
1e−06
1e−04
1e−02
1e−06
1e−04
1e−02
1e+00
1e−04
1e−03
1e−02
1e−01
1e+00
1e−02
1e+01
1e+04

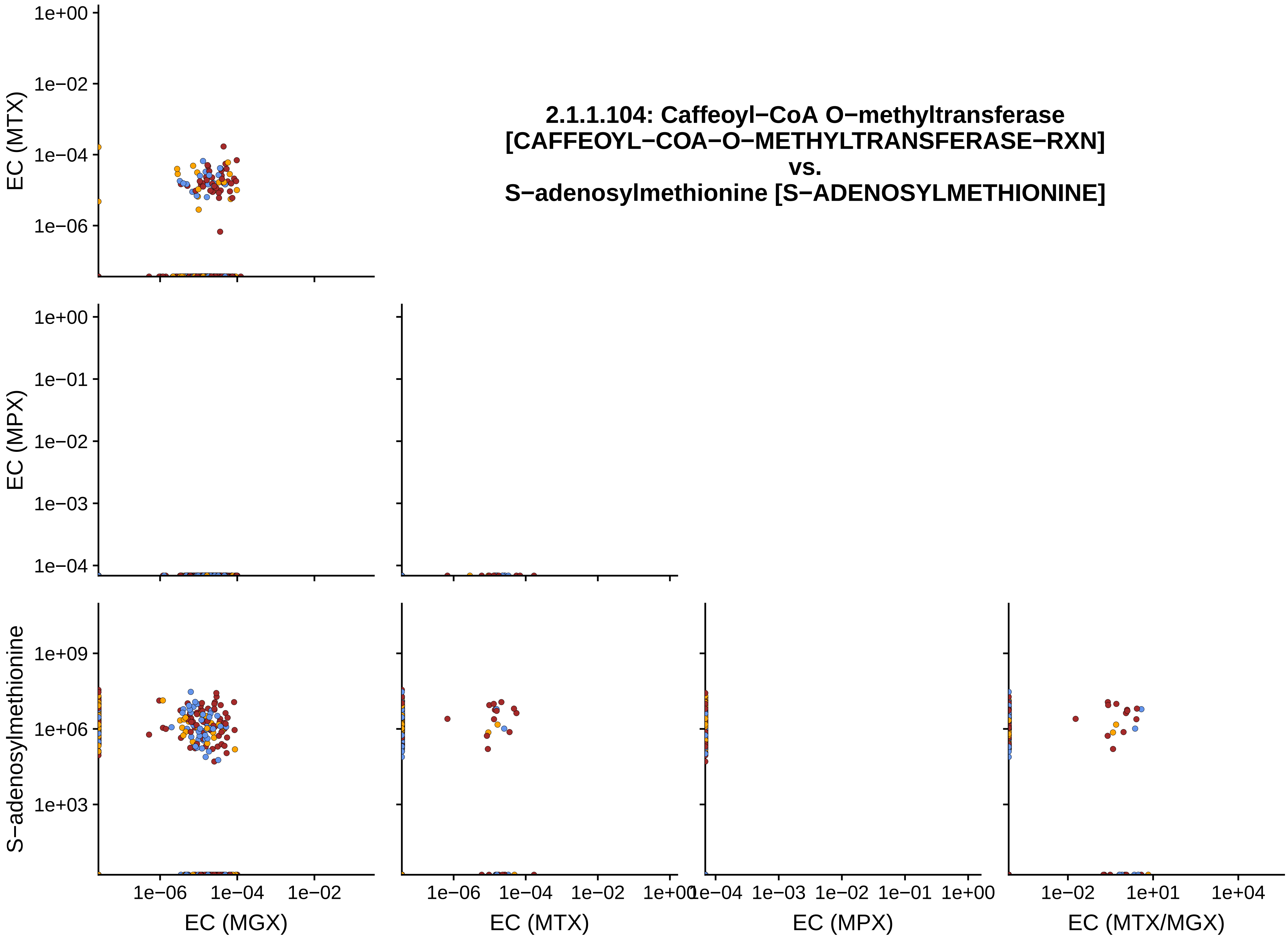
2.1.1.104: Caffeoyl−CoA O−methyltransferase
[CAFFEOYL−COA−O−METHYLTRANSFERASE−RXN]
vs.
S−adenosylmethionine [S−ADENOSYLMETHIONINE]
EC (MTX)
EC (MPX)
S−adenosylmethionine
EC (MGX)
EC (MTX)
EC (MPX)
EC (MTX/MGX)
1e+00
1e−02
1e−04
1e−06
1e−01
1e−03
1e+09
1e+06
1e+03
1e+01
1e+04

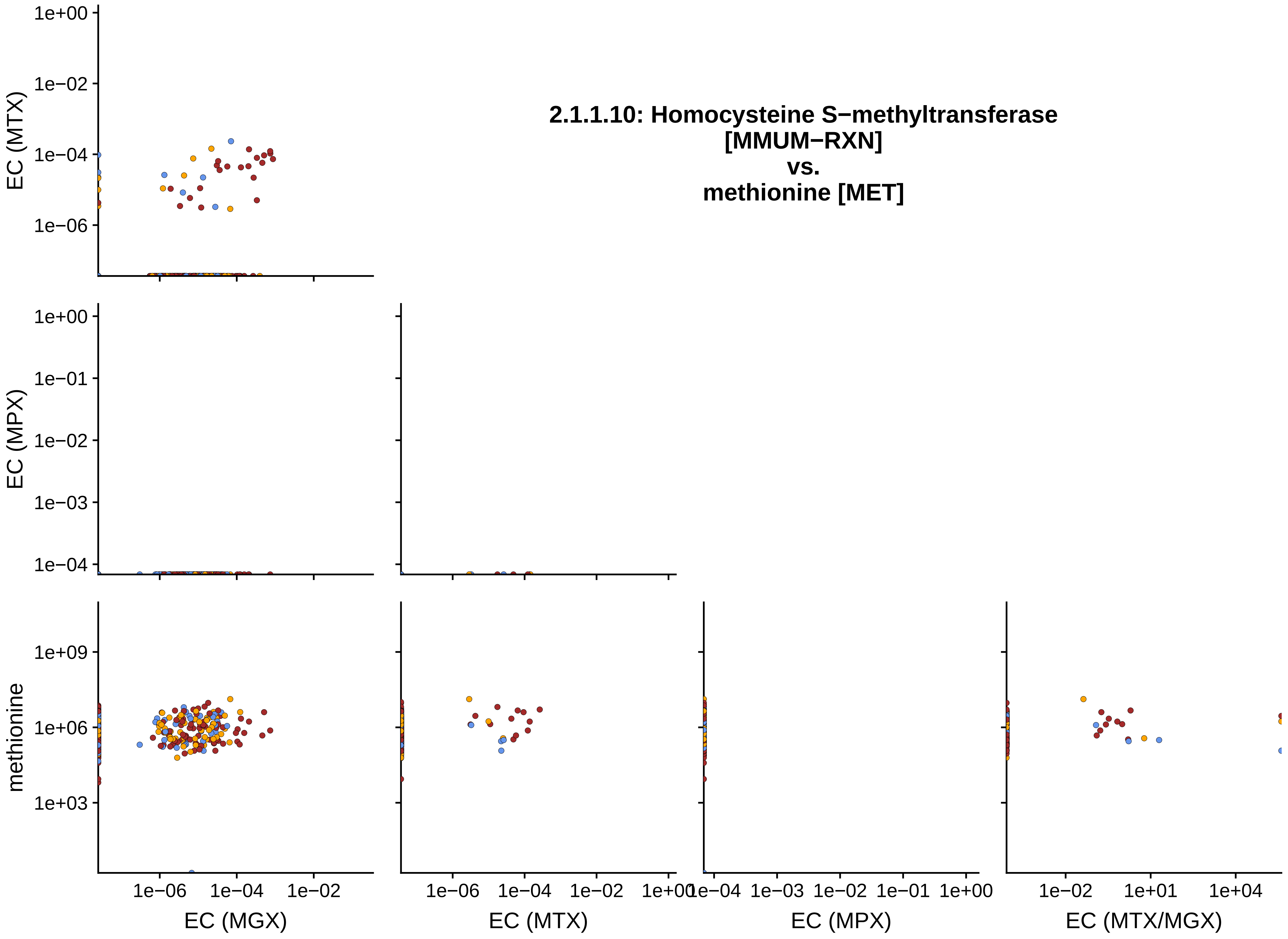
2.1.1.10: Homocysteine S−methyltransferase
[MMUM−RXN]
vs.
methionine [MET]
EC (MTX)
EC (MPX)
methionine
EC (MGX)
EC (MTX)
EC (MPX)
EC (MTX/MGX)
1e+00
1e−02
1e−04
1e−06
1e−01
1e−03
1e+09
1e+06
1e+03
1e+01
1e+04

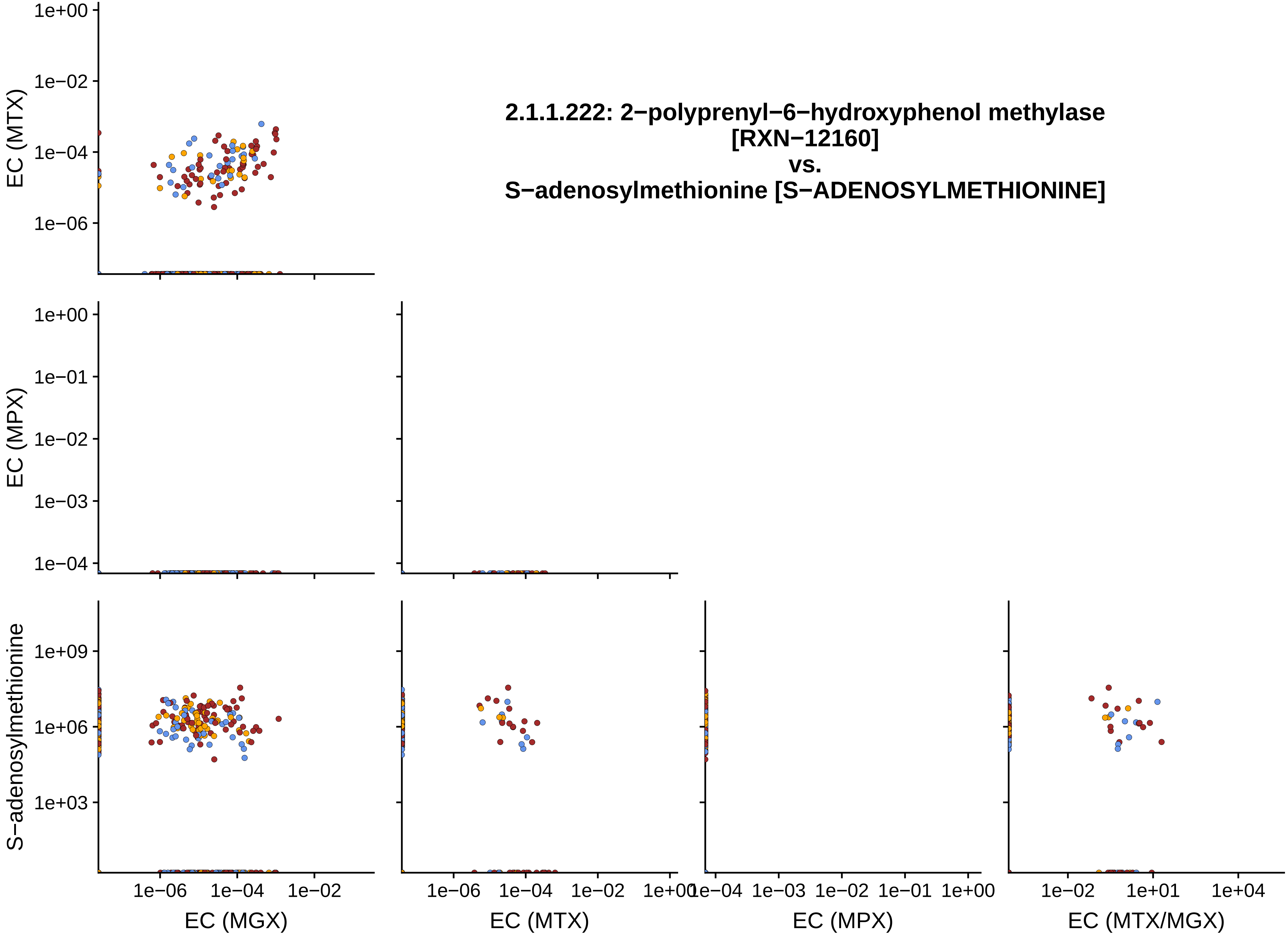
2.1.1.222: 2−polyprenyl−6−hydroxyphenol methylase
[RXN−12160]
vs.
S−adenosylmethionine [S−ADENOSYLMETHIONINE]
EC (MTX)
EC (MPX)
S−adenosylmethionine
EC (MGX)
EC (MTX)
EC (MPX)
EC (MTX/MGX)
1e+00
1e−02
1e−04
1e−06
1e−01
1e−03
1e+09
1e+06
1e+03
1e+01
1e+04

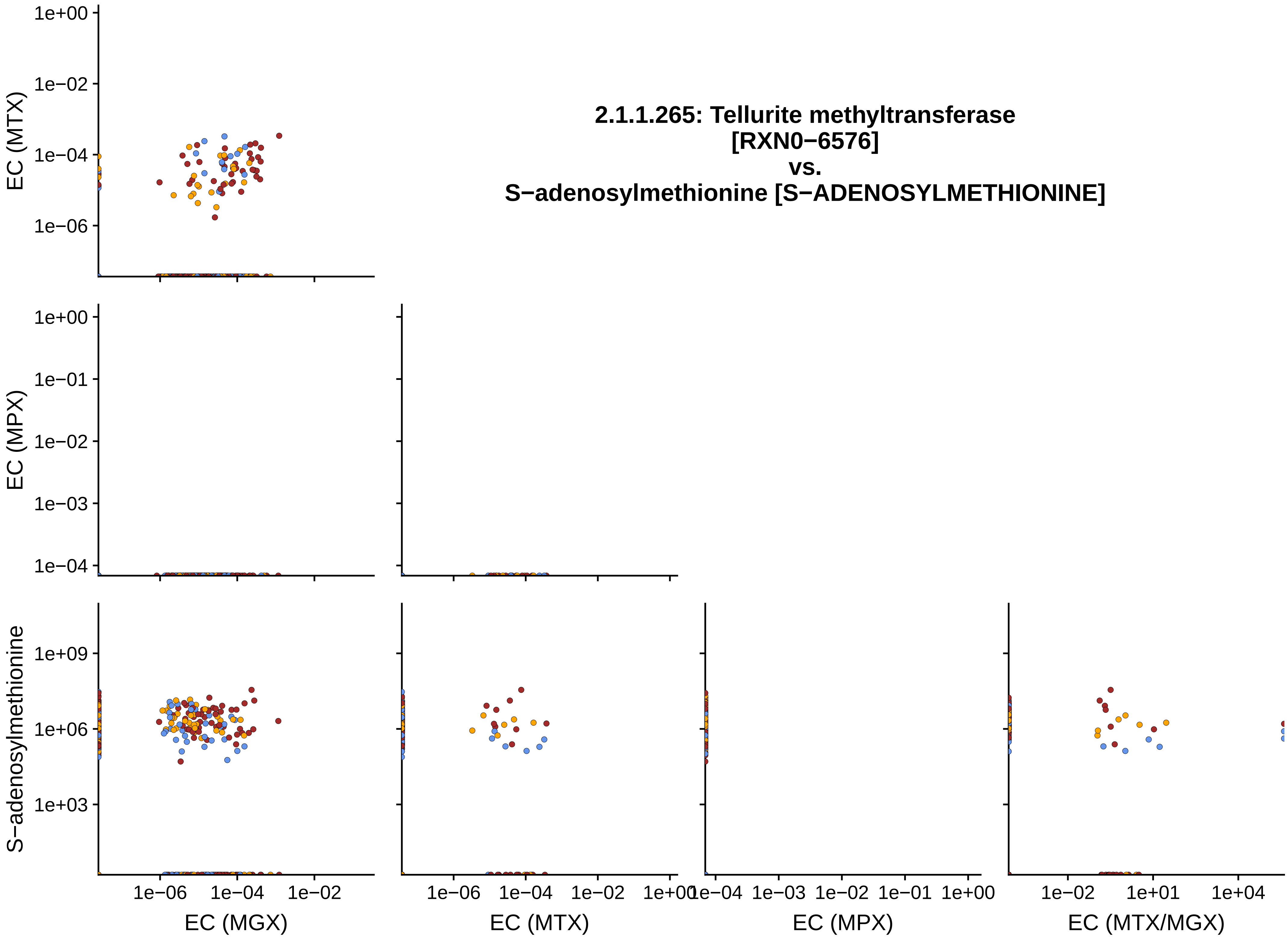

2.1.1.265: Tellurite methyltransferase
[RXN0−6576]
vs.
S−adenosylmethionine [S−ADENOSYLMETHIONINE]
EC (MTX)
EC (MPX)
S−adenosylmethionine
EC (MGX)
EC (MTX)
EC (MPX)
EC (MTX/MGX)
1e+00
1e−02
1e−04
1e−06
1e−01
1e−03
1e+09
1e+06
1e+03
1e−01
1e+01
1e+04

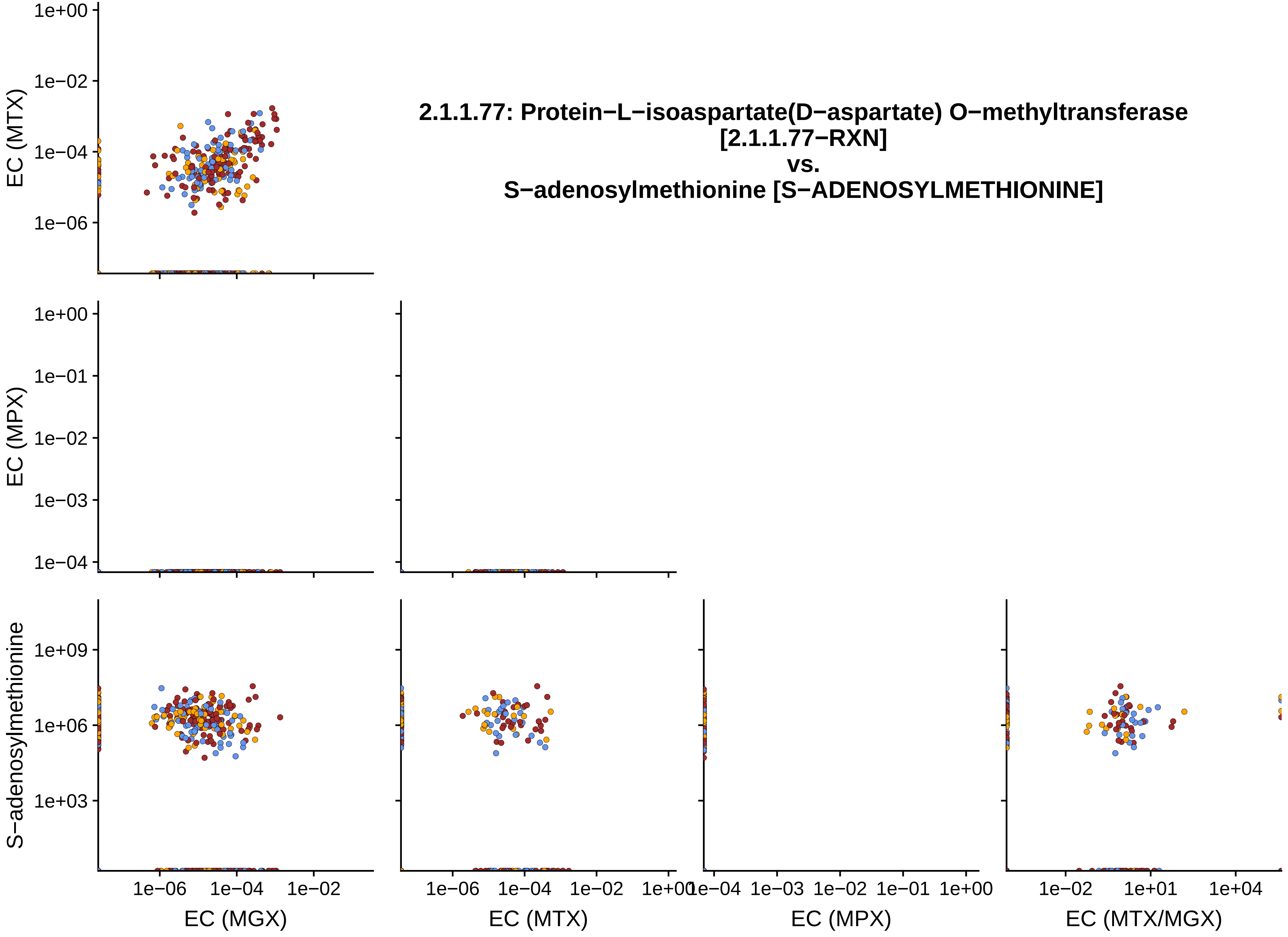
2.1.1.77: Protein−L−isoaspartate(D−aspartate) O−methyltransferase
[2.1.1.77−RXN]
vs.
S−adenosylmethionine [S−ADENOSYLMETHIONINE]
EC (MTX)
EC (MPX)
S−adenosylmethionine
EC (MGX)
EC (MTX)
EC (MPX)
EC (MTX/MGX)
1e+00
1e−02
1e−04
1e−06
1e−01
1e−03
1e+09
1e+06
1e+03
1e+01
1e+04

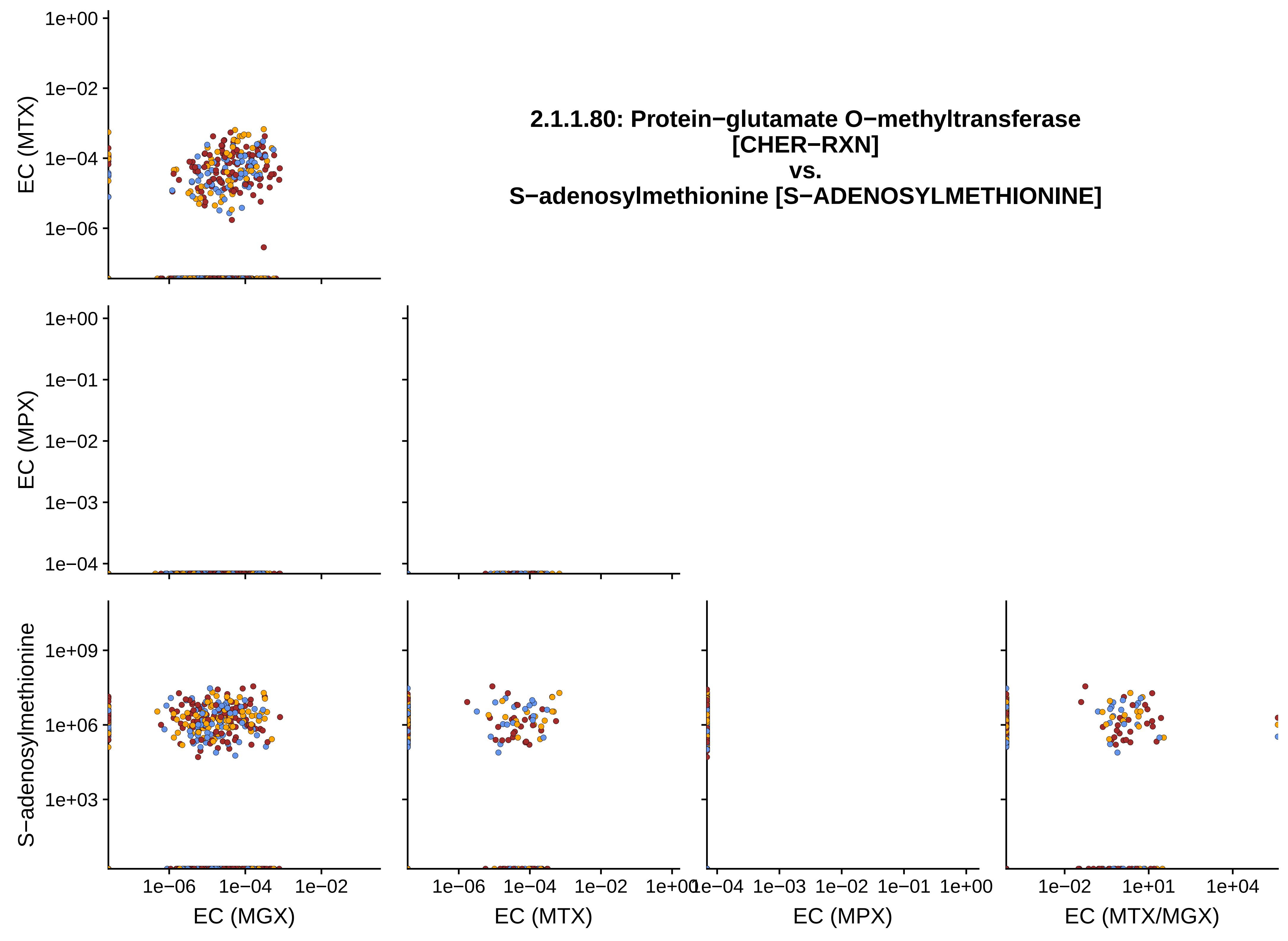

2.1.1.80: Protein−glutamate O−methyltransferase
[CHER−RXN]
vs.
S−adenosylmethionine [S−ADENOSYLMETHIONINE]
EC (MTX)
EC (MPX)
S−adenosylmethionine
EC (MGX)
EC (MTX)
EC (MPX)
EC (MTX/MGX)
1e+00
1e−02
1e−04
1e−06
1e−01
1e−03
1e+09
1e+06
1e+03
1e+01
1e+04

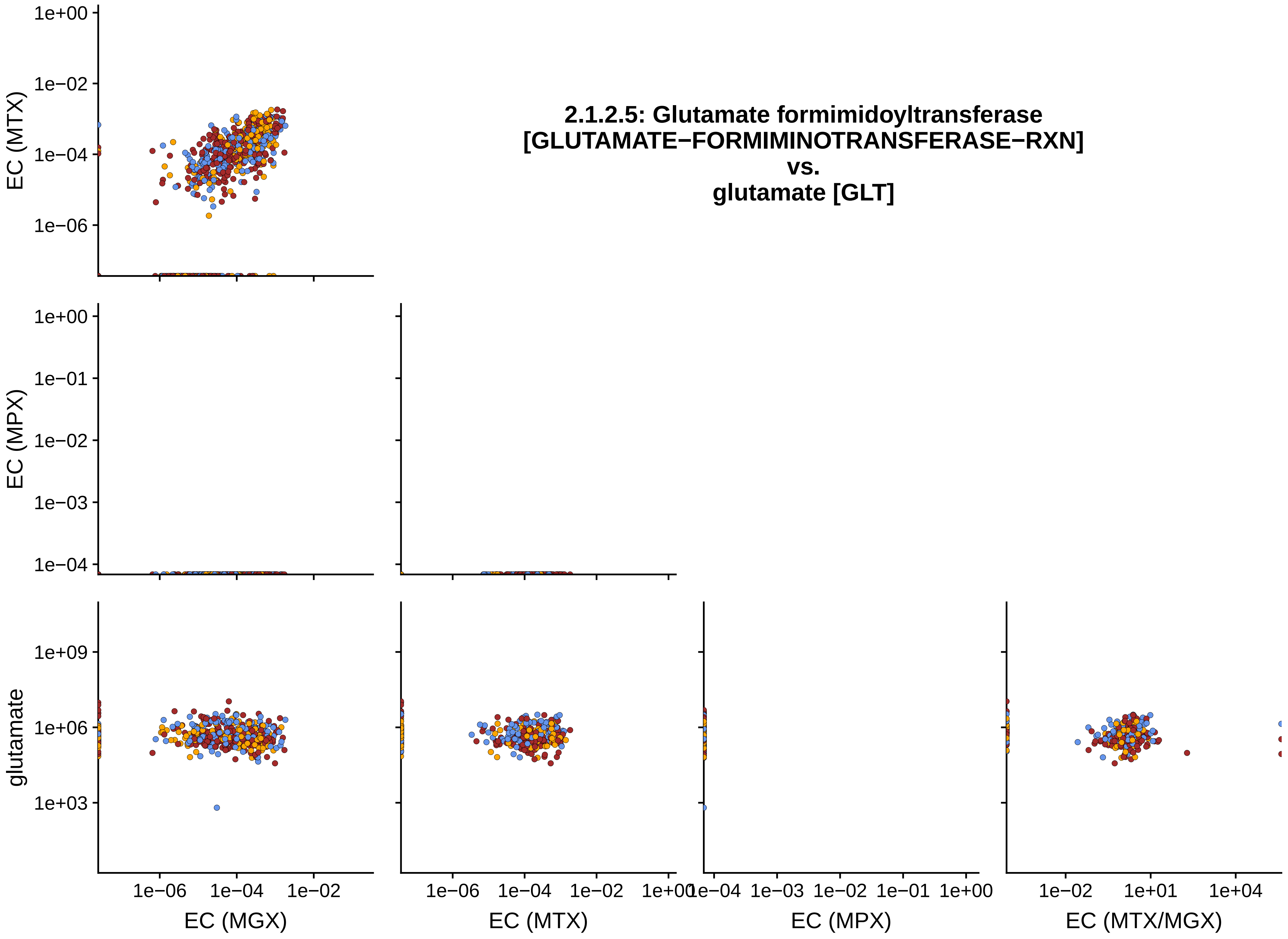
2.1.2.5: Glutamate formimidoyltransferase
[GLUTAMATE−FORMIMINOTRANSFERASE−RXN]
vs.
glutamate [GLT]
EC (MTX)
EC (MPX)
glutamate
EC (MGX)
EC (MTX)
EC (MPX)
EC (MTX/MGX)
1e+00
1e−02
1e−04
1e−06
1e−01
1e−03
1e+09
1e+06
1e+03
1e+04
1e+01

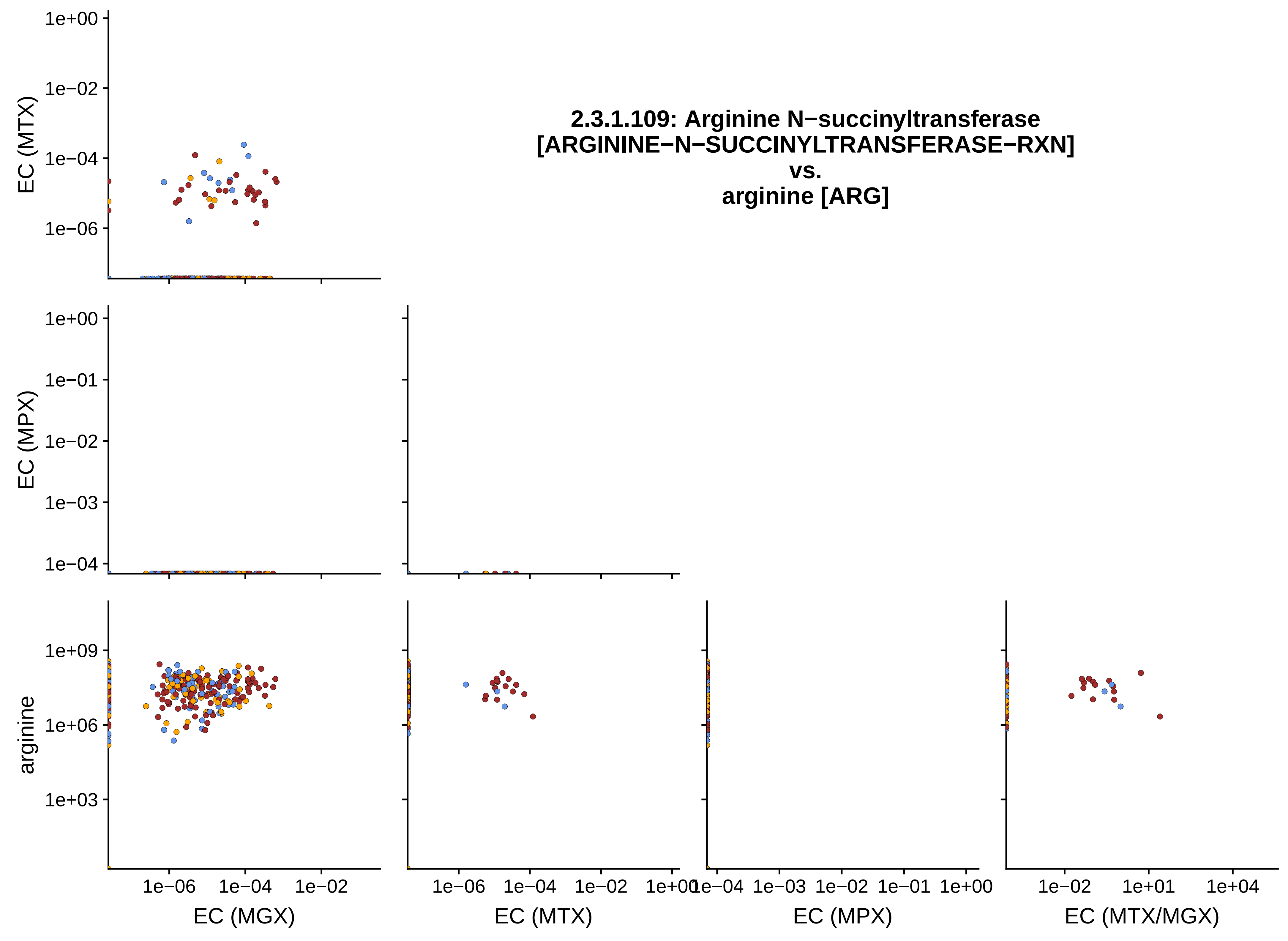
2.3.1.109: Arginine N−succinyltransferase
[ARGININE−N−SUCCINYLTRANSFERASE−RXN]
vs.
arginine [ARG]
EC (MTX)
EC (MPX)
arginine
EC (MGX)
EC (MTX)
EC (MPX)
EC (MTX/MGX)
1e+00
1e−02
1e−04
1e−06
1e−01
1e−03
1e+09
1e+06
1e+03
1e+01
1e+04

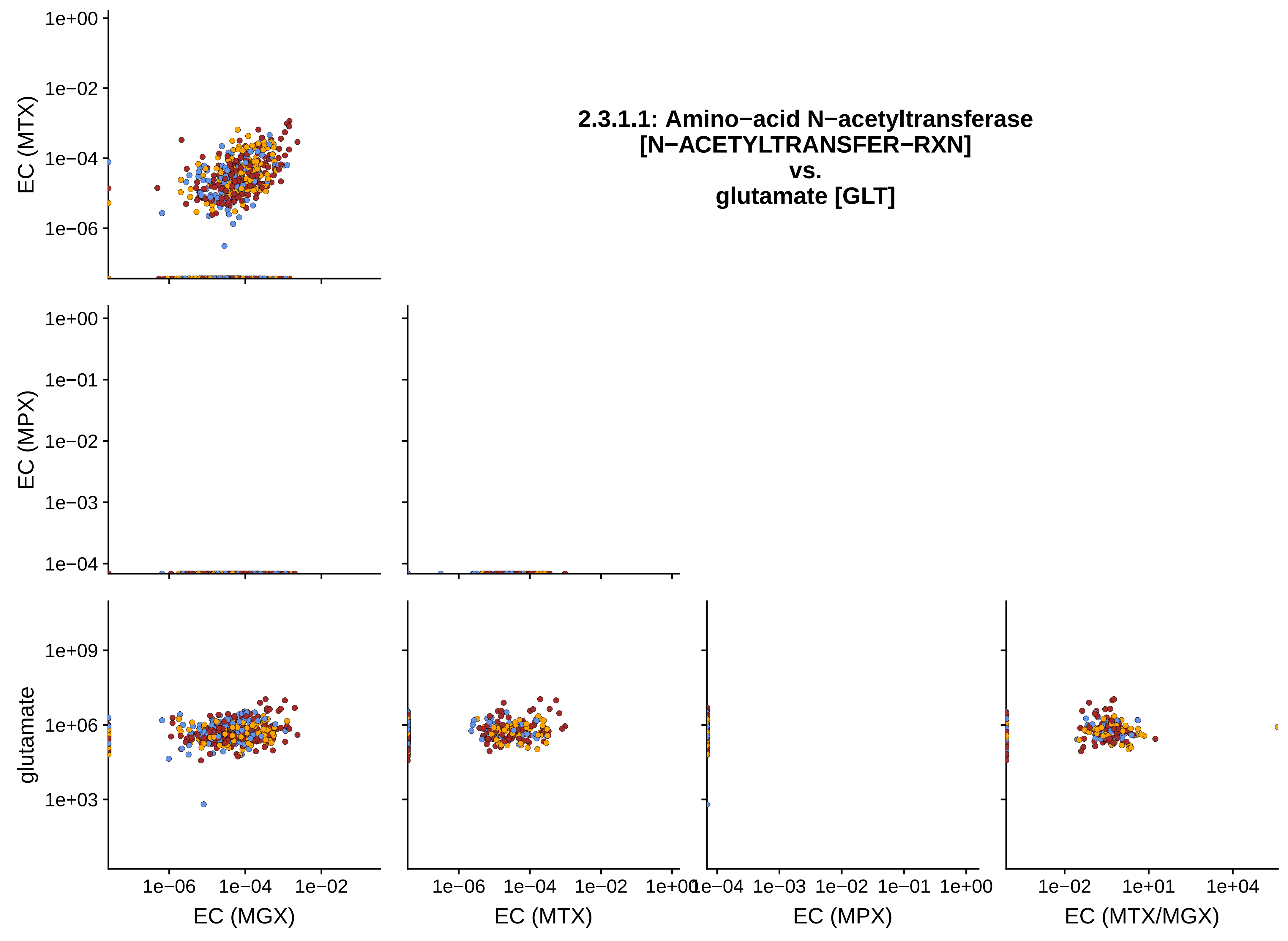
2.3.1.1: Amino−acid N−acetyltransferase
[N−ACETYLTRANSFER−RXN]
vs.
glutamate [GLT]
EC (MTX)
1e+00
1e−02
1e−04
1e−06
EC (MPX)
1e+00
1e−01
1e−02
1e−03
1e−04
glutamate
1e+09
1e+06
1e+03
1e−06
1e−04
1e−02
EC (MGX)
1e−06
1e−04
1e−02
1e+00
EC (MTX)
1e−04
1e−03
1e−02
1e−01
1e+00
EC (MPX)
1e−02
1e+01
1e+04
EC (MTX/MGX)

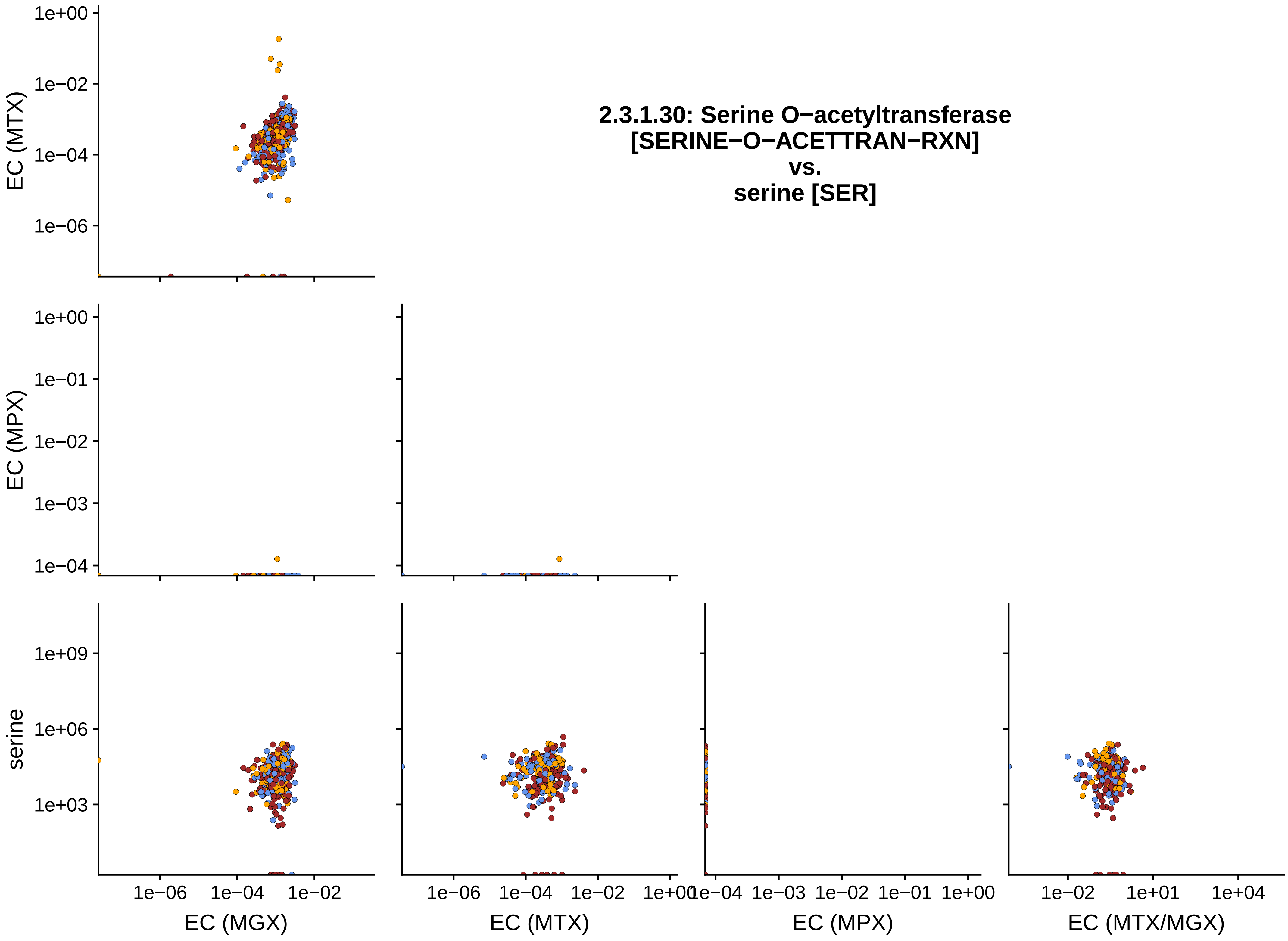

2.3.1.30: Serine O−acetyltransferase
[SERINE−O−ACETTRAN−RXN]
vs.
serine [SER]
EC (MTX)
EC (MPX)
serine
EC (MGX)
EC (MTX)
EC (MPX)
EC (MTX/MGX)
1e+00
1e−02
1e−04
1e−06
1e−01
1e−03
1e+09
1e+06
1e+03
1e+01
1e+04

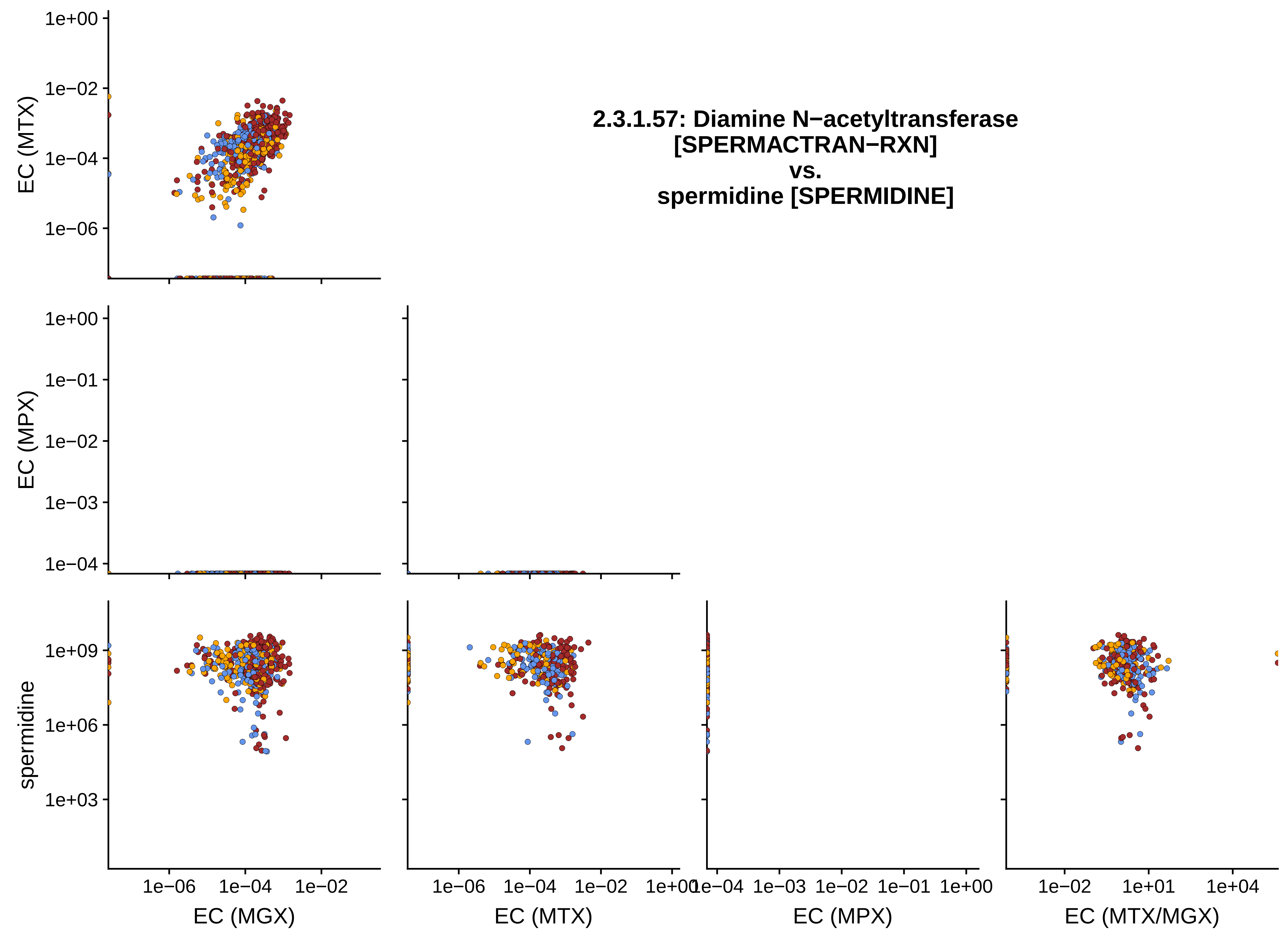

2.3.1.57: Diamine N−acetyltransferase
[SPERMACTRAN−RXN]
vs.
spermidine [SPERMIDINE]
EC (MTX)
EC (MPX)
spermidine
EC (MGX)
EC (MTX)
EC (MPX)
EC (MTX/MGX)
1e+00
1e−02
1e−04
1e−06
1e−01
1e−03
1e+09
1e+06
1e+03
1e+01
1e+04

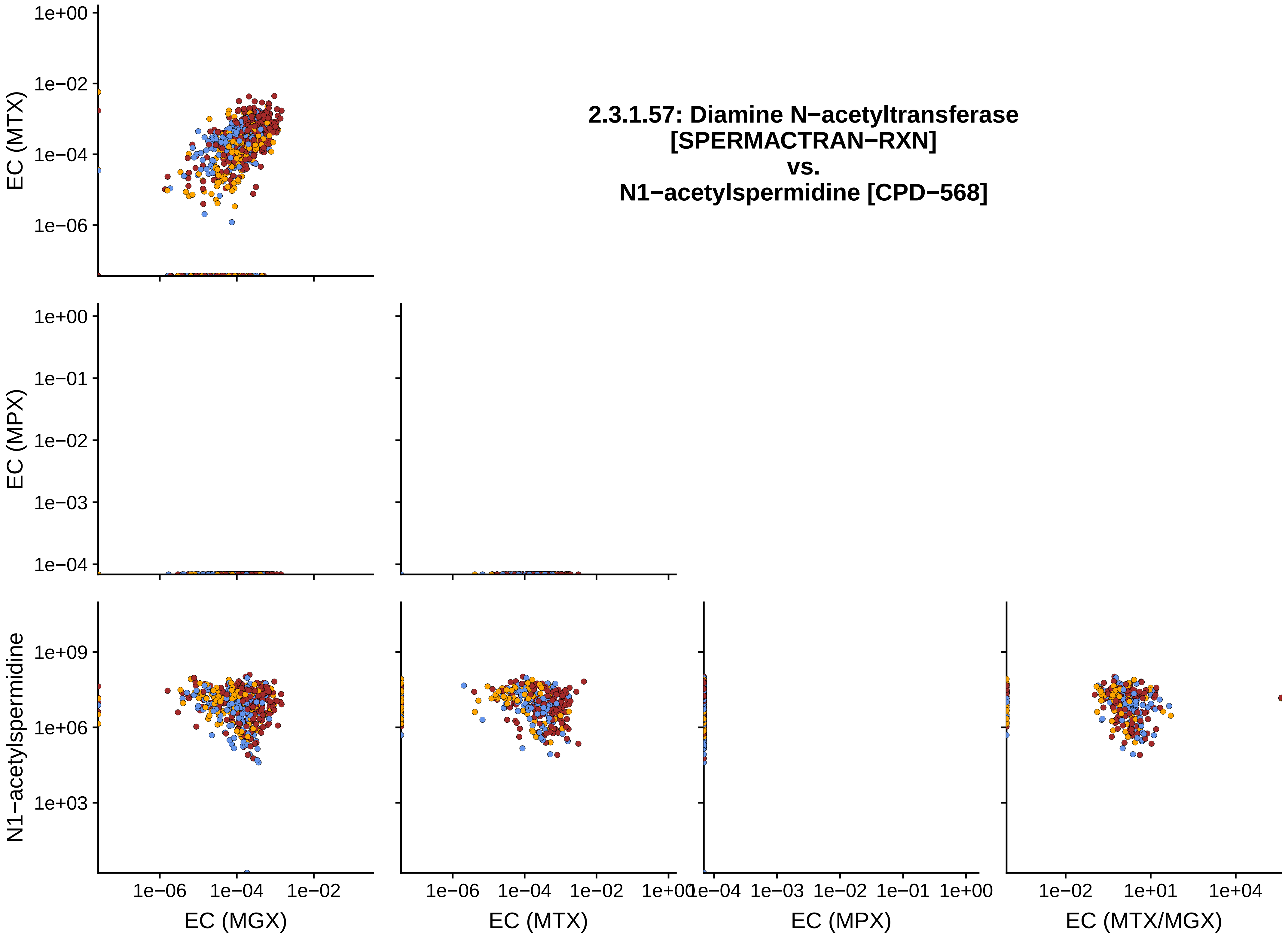
2.3.1.57: Diamine N−acetyltransferase
[SPERMACTRAN−RXN]
vs.
N1−acetylspermidine [CPD−568]
EC (MTX)
EC (MPX)
N1−acetylspermidine
EC (MGX)
EC (MTX)
EC (MPX)
EC (MTX/MGX)
1e+00
1e−02
1e−04
1e−06
1e−01
1e−03
1e+09
1e+06
1e+03
1e+01
1e+04

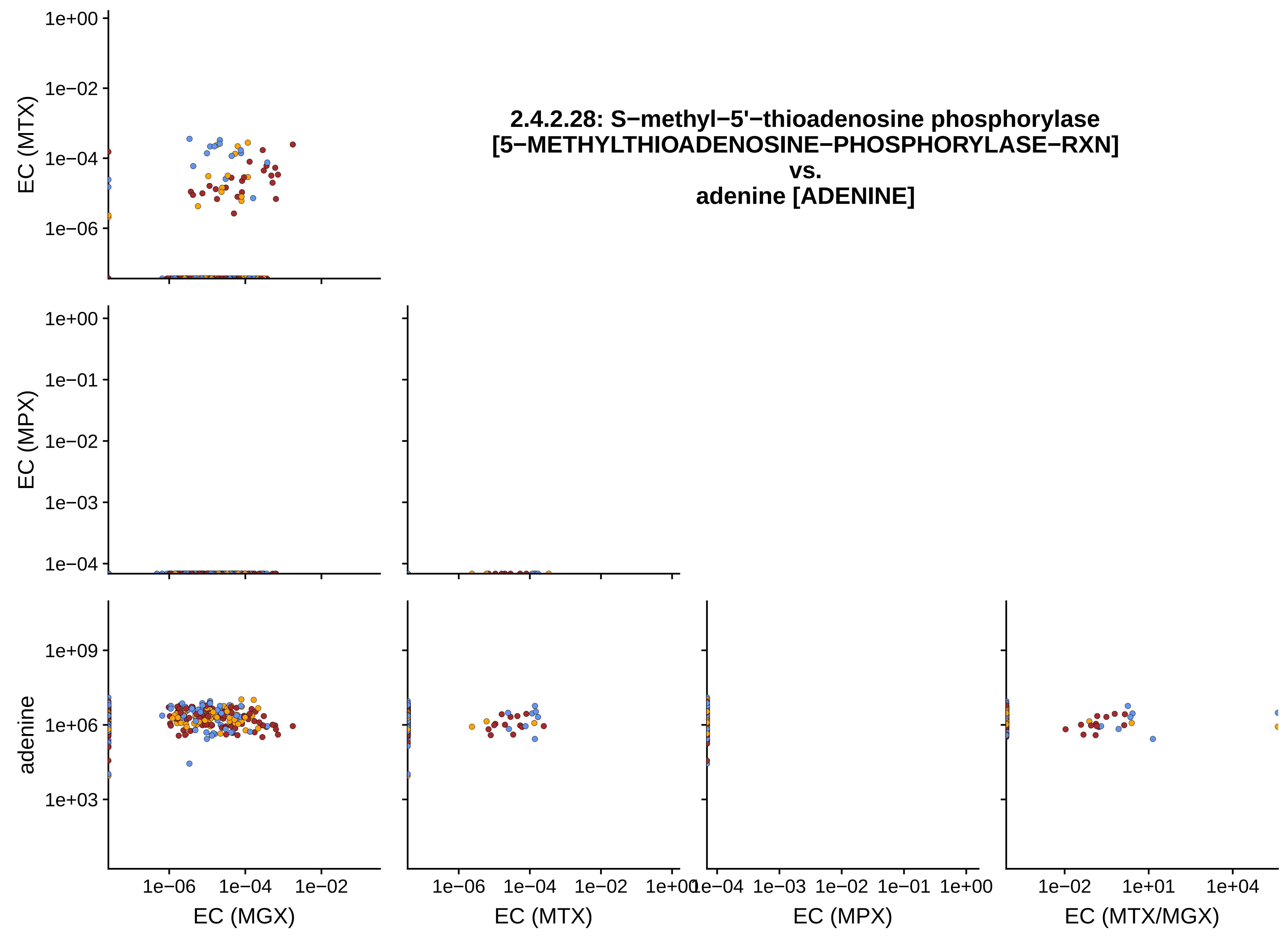
2.4.2.28: S−methyl−5'−thioadenosine phosphorylase
[5−METHYLTHIOADENOSINE−PHOSPHORYLASE−RXN]
vs.
adenine [ADENINE]
EC (MTX)
EC (MPX)
adenine
EC (MGX)
EC (MTX)
EC (MPX)
EC (MTX/MGX)
1e+00
1e−02
1e−04
1e−06
1e−01
1e−03
1e+09
1e+06
1e+03
1e+01
1e+04

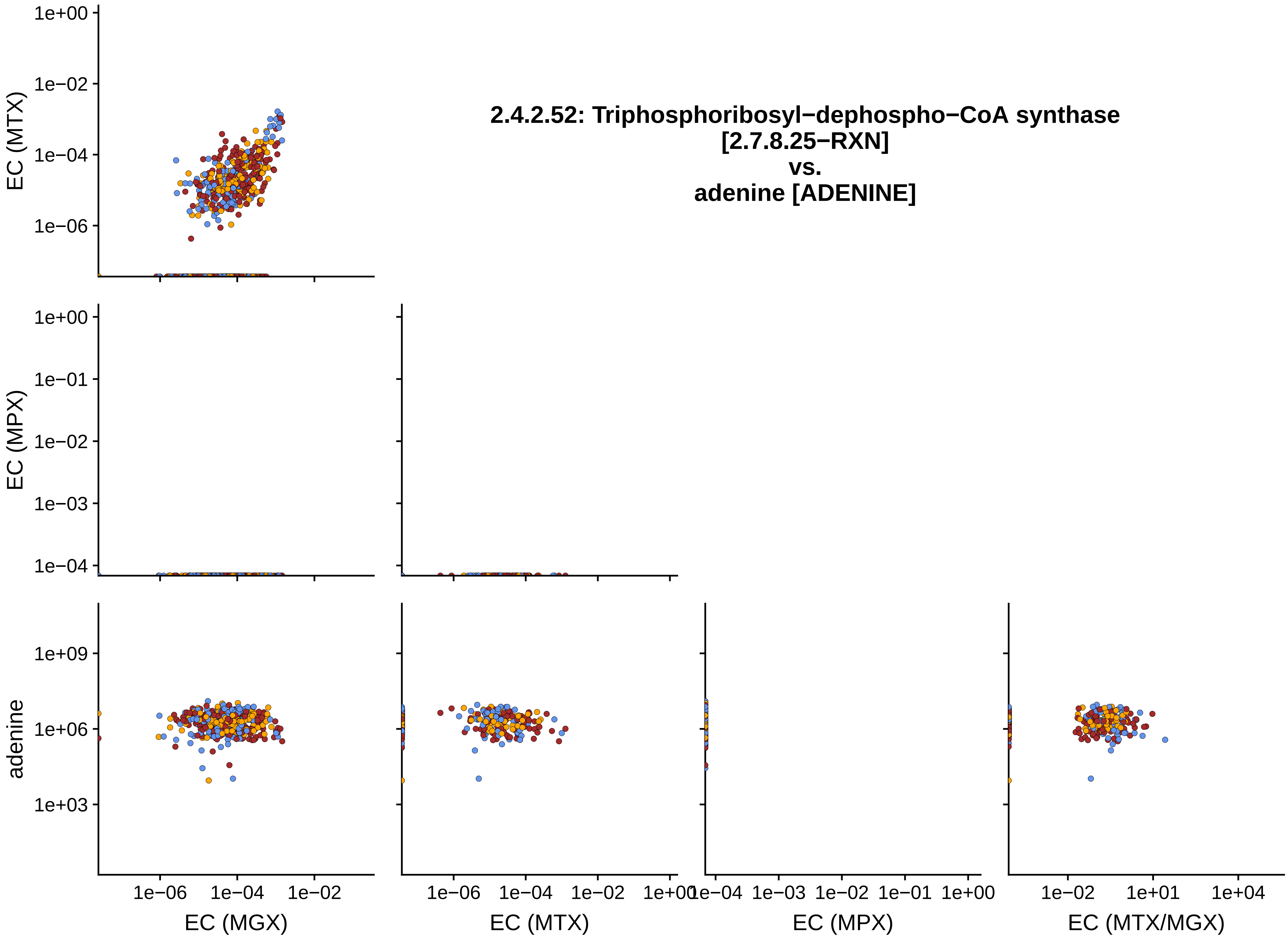

2.4.2.52: Triphosphoribosyl−dephospho−CoA synthase
[2.7.8.25−RXN]
vs.
adenine [ADENINE]
EC (MTX)
EC (MPX)
adenine
EC (MGX)
EC (MTX)
EC (MPX)
EC (MTX/MGX)
1e+00
1e−02
1e−04
1e−06
1e+00
1e−01
1e−02
1e−03
1e−04
1e+09
1e+06
1e+03
1e−06
1e−04
1e−02
1e−06
1e−04
1e−02
1e+00
1e−04
1e−03
1e−02
1e−01
1e+00
1e−02
1e+01
1e+04

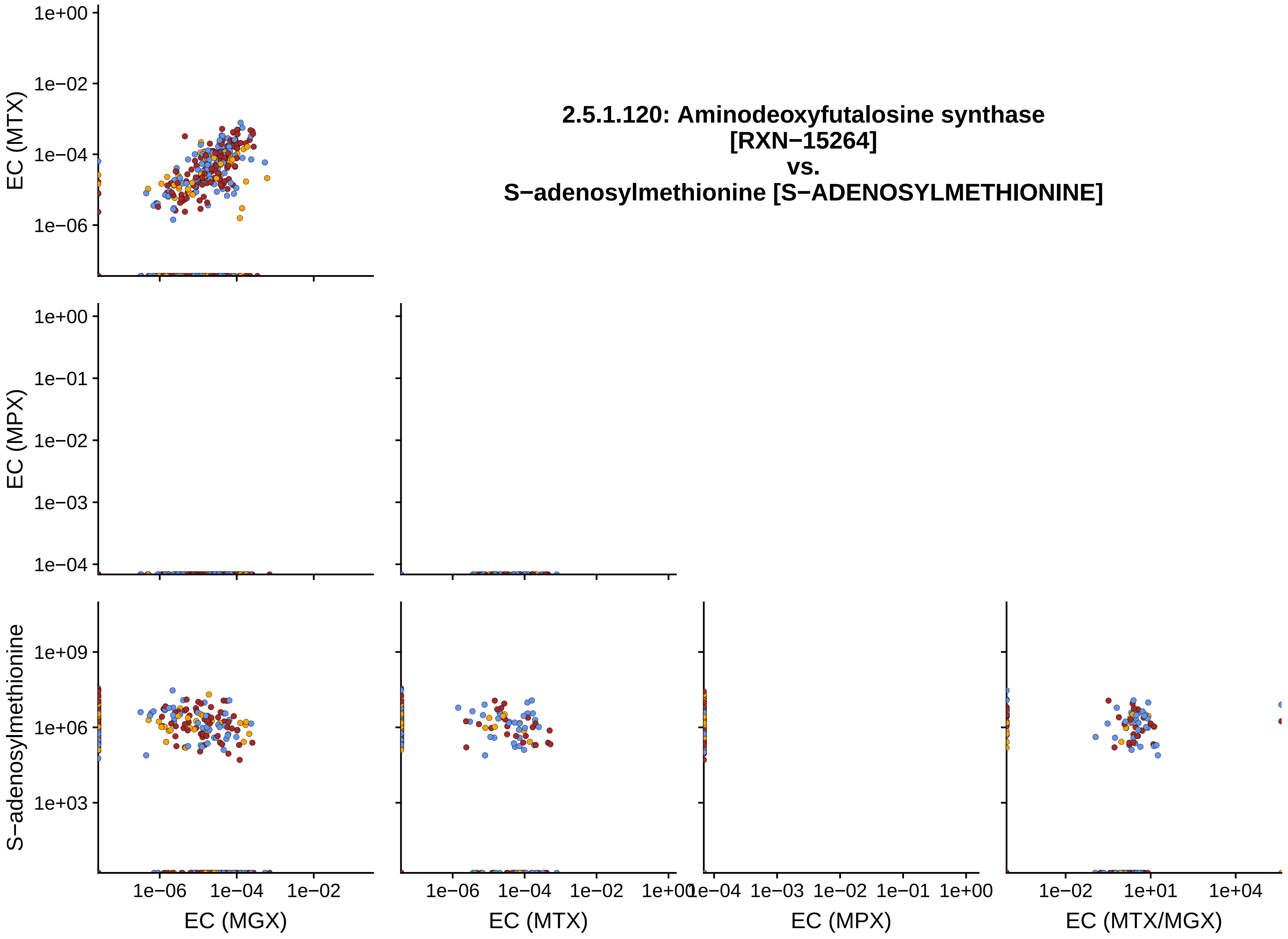

2.5.1.120: Aminodeoxyfutalosine synthase
[RXN−15264]
vs.
S−adenosylmethionine [S−ADENOSYLMETHIONINE]
EC (MTX)
EC (MPX)
S−adenosylmethionine
EC (MGX)
EC (MTX)
EC (MPX)
EC (MTX/MGX)
1e+00
1e−02
1e−04
1e−06
1e−01
1e−03
1e+09
1e+06
1e+03
1e+01
1e+04

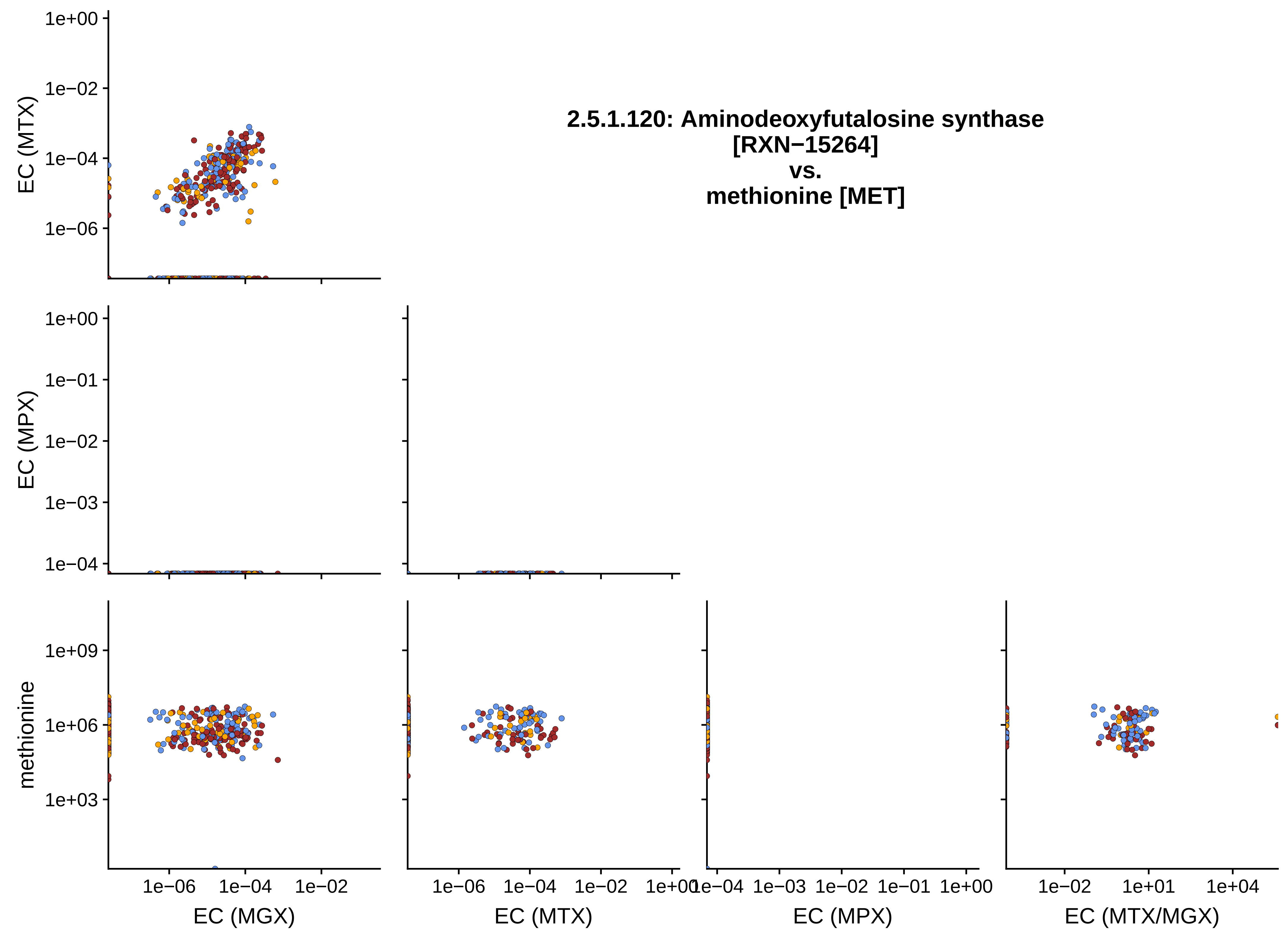
2.5.1.120: Aminodeoxyfutalosine synthase
[RXN−15264]
vs.
methionine [MET]
EC (MTX)
EC (MPX)
methionine
EC (MGX)
EC (MTX)
EC (MPX)
EC (MTX/MGX)
1e+00
1e−02
1e−04
1e−06
1e−01
1e−03
1e+09
1e+06
1e+03
1e+01
1e+04

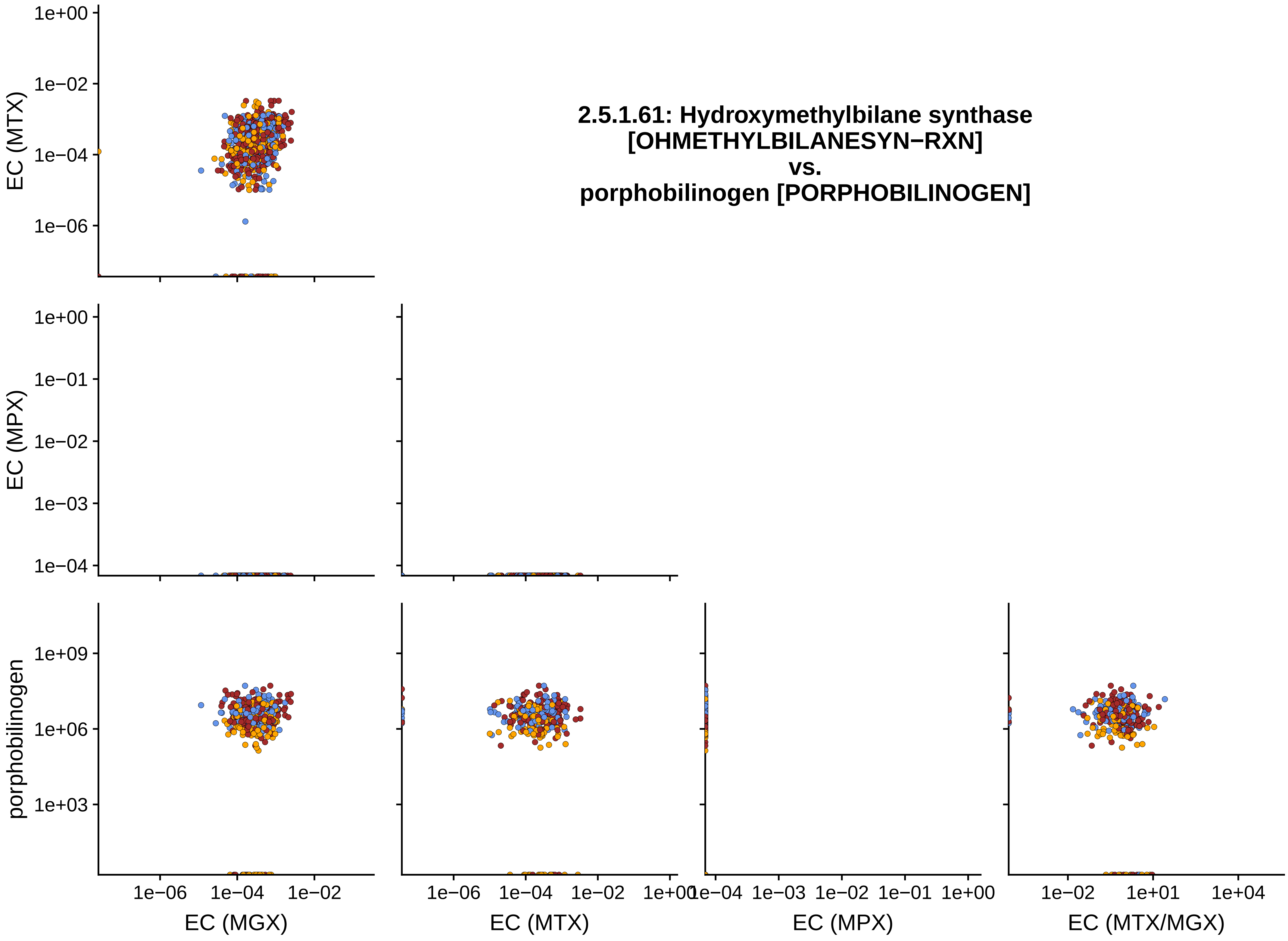

2.5.1.61: Hydroxymethylbilane synthase
[OHMETHYLBILANESYN−RXN]
vs.
porphobilinogen [PORPHOBILINOGEN]
EC (MTX)
EC (MPX)
porphobilinogen
EC (MGX)
EC (MTX)
EC (MPX)
EC (MTX/MGX)
1e+00
1e−02
1e−04
1e−06
1e−01
1e−03
1e+09
1e+06
1e+03
1e+01
1e+04

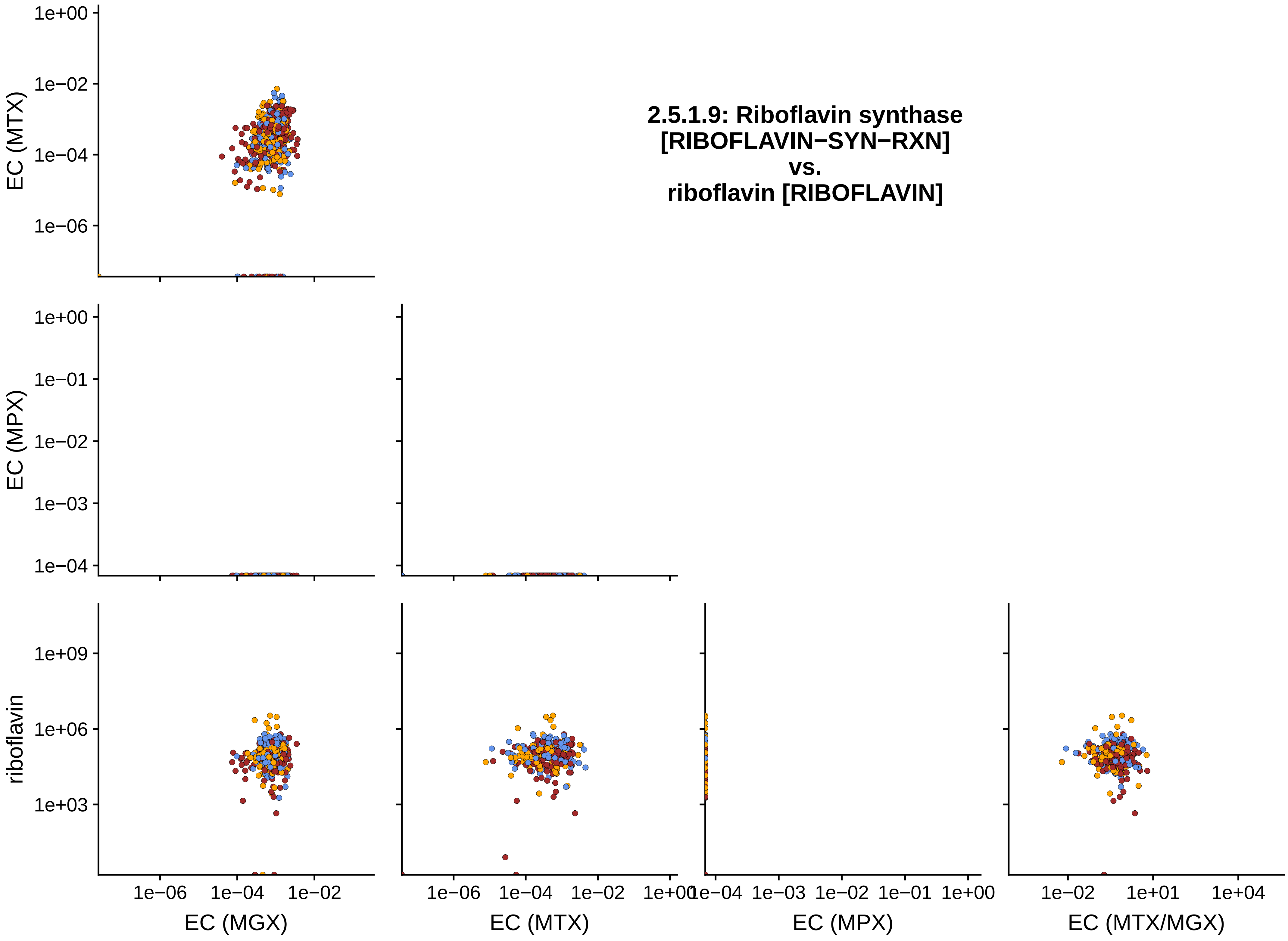
2.5.1.9: Riboflavin synthase
[RIBOFLAVIN−SYN−RXN]
vs.
riboflavin [RIBOFLAVIN]
EC (MTX)
EC (MPX)
riboflavin
EC (MGX)
EC (MTX)
EC (MPX)
EC (MTX/MGX)
1e+00
1e−02
1e−04
1e−06
1e−01
1e−03
1e+09
1e+06
1e+03
1e+01
1e+04

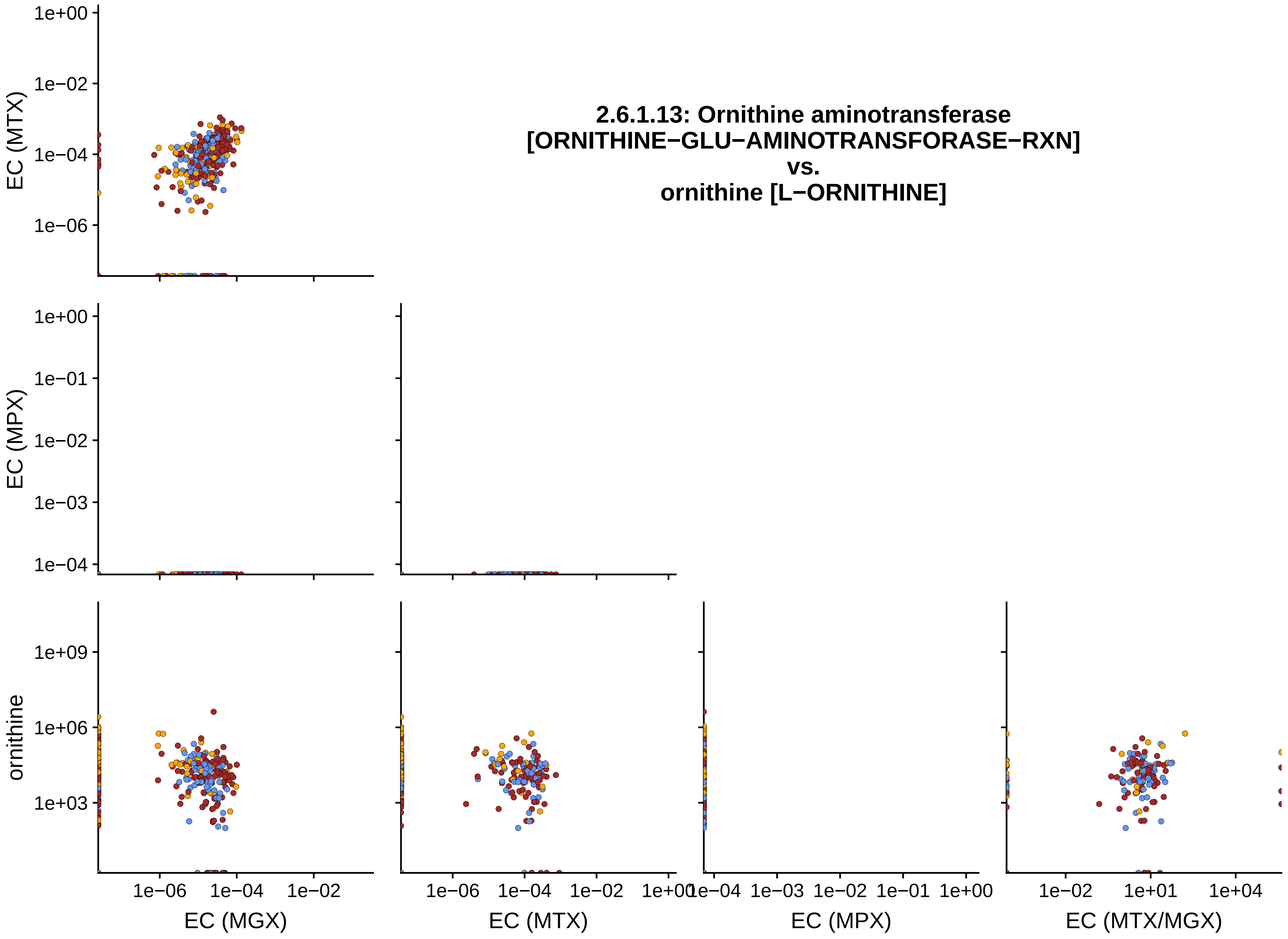
2.6.1.13: Ornithine aminotransferase
[ORNITHINE−GLU−AMINOTRANSFORASE−RXN]
vs.
ornithine [L−ORNITHINE]
EC (MTX)
EC (MPX)
ornithine
EC (MGX)
EC (MTX)
EC (MPX)
EC (MTX/MGX)
1e+00
1e−02
1e−04
1e−06
1e−01
1e−03
1e+09
1e+06
1e+03
1e+01
1e+04

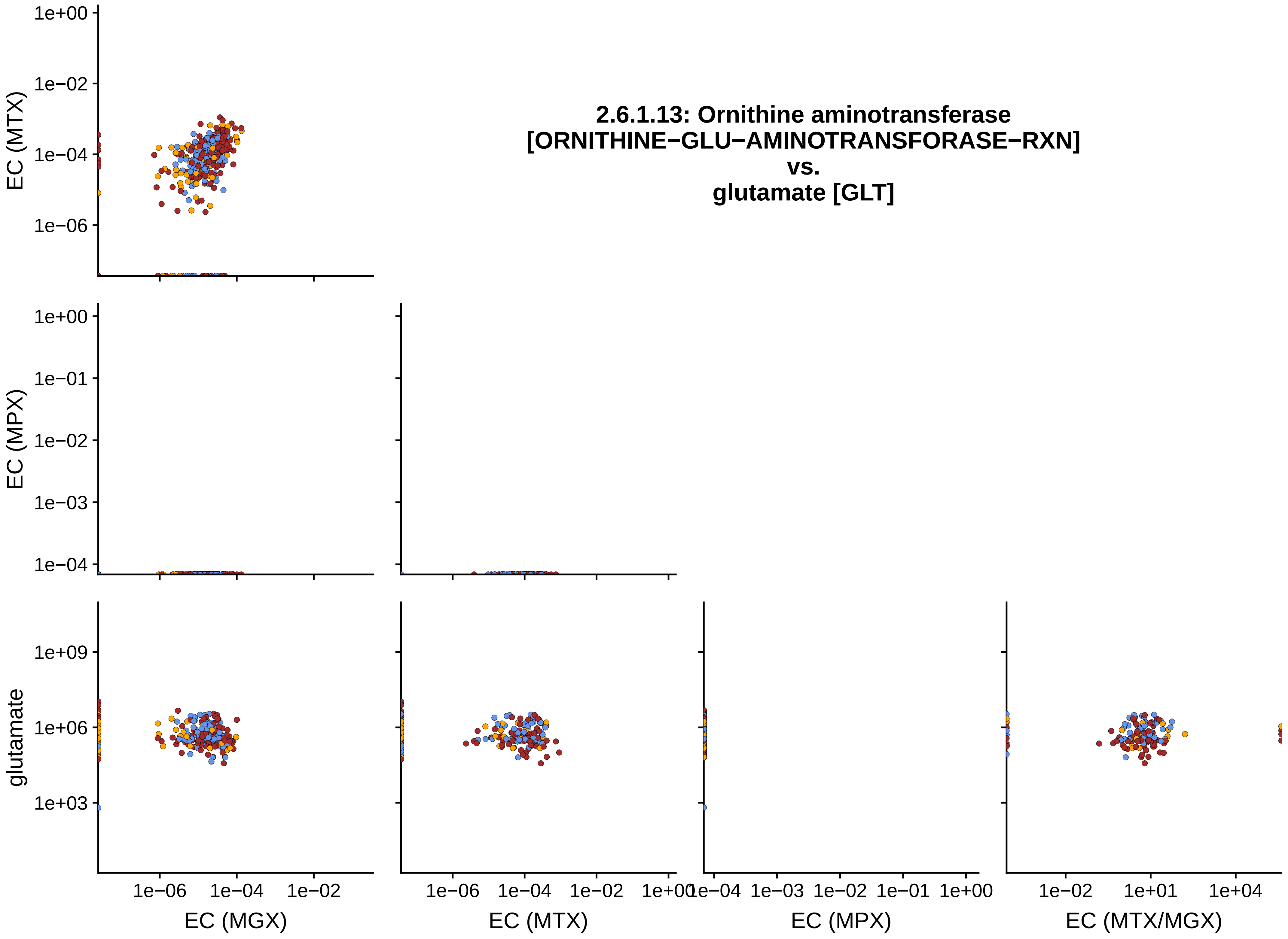
2.6.1.13: Ornithine aminotransferase
[ORNITHINE−GLU−AMINOTRANSFORASE−RXN]
vs.
glutamate [GLT]
EC (MTX)
EC (MPX)
glutamate
EC (MGX)
EC (MTX)
EC (MPX)
EC (MTX/MGX)
1e+00
1e−02
1e−04
1e−06
1e−01
1e−03
1e+09
1e+06
1e+03
1e+01
1e+04

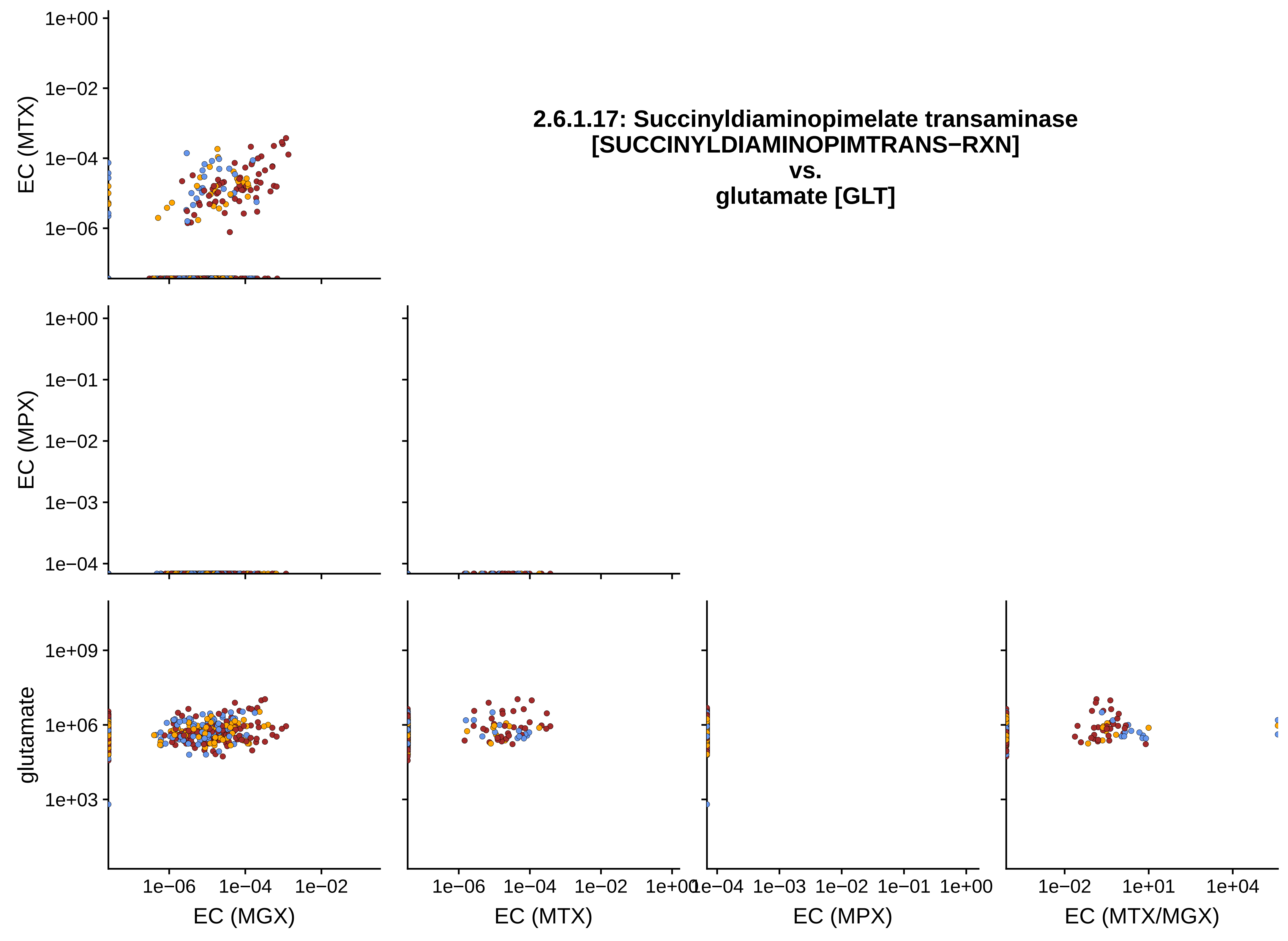
2.6.1.17: Succinyldiaminopimelate transaminase
[SUCCINYLDIAMINOPIMTRANS−RXN]
vs.
glutamate [GLT]
EC (MTX)
EC (MPX)
glutamate
EC (MGX)
EC (MTX)
EC (MPX)
EC (MTX/MGX)
1e+00
1e−02
1e−04
1e−06
1e−01
1e−03
1e+09
1e+06
1e+03
1e+01
1e+04

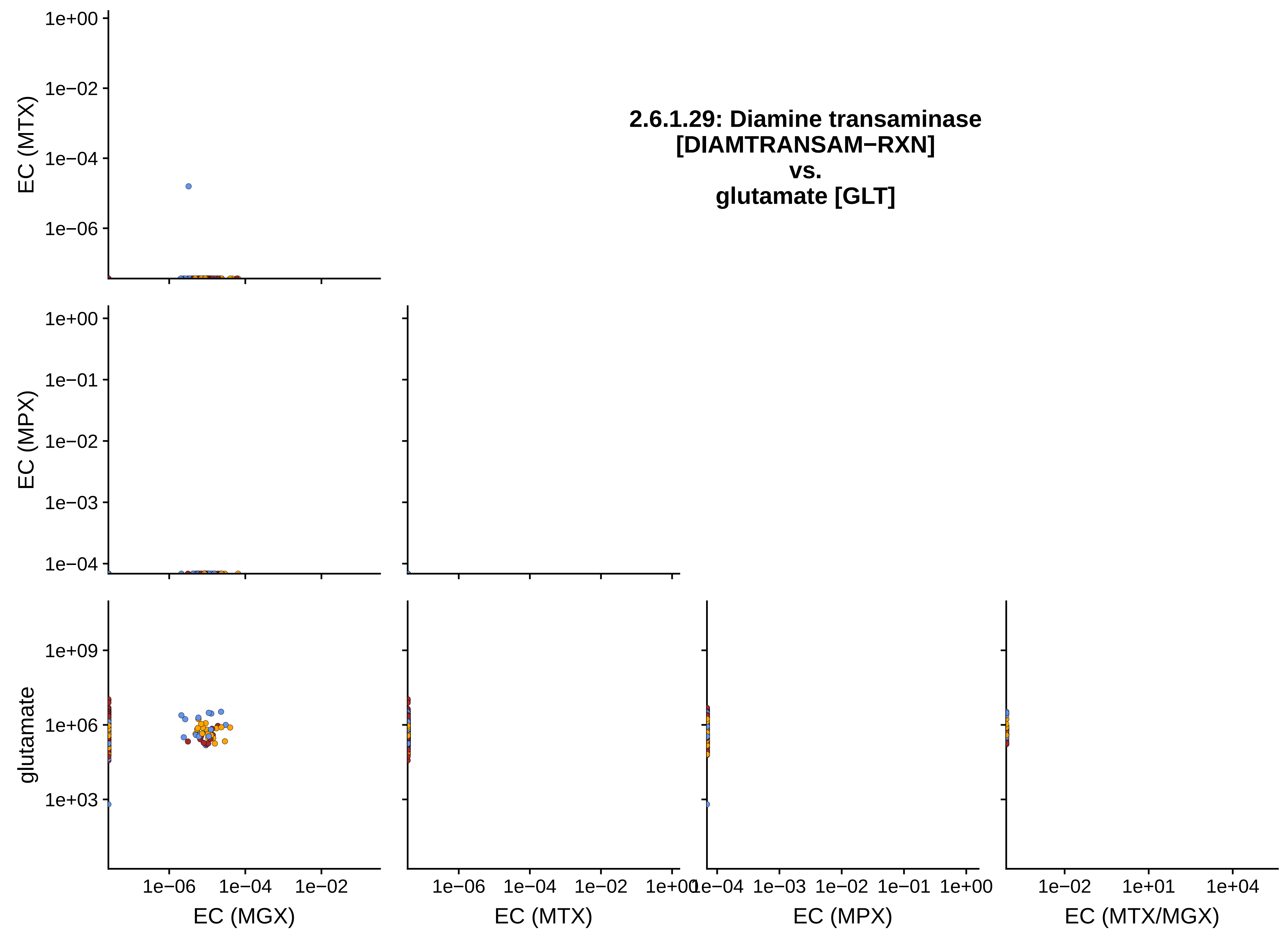

2.6.1.29: Diamine transaminase
[DIAMTRANSAM−RXN]
vs.
glutamate [GLT]
EC (MTX)
EC (MPX)
glutamate
EC (MGX)
EC (MTX)
EC (MPX)
EC (MTX/MGX)
1e+00
1e−02
1e−04
1e−06
1e−01
1e−03
1e+09
1e+06
1e+03
1e+01
1e+04

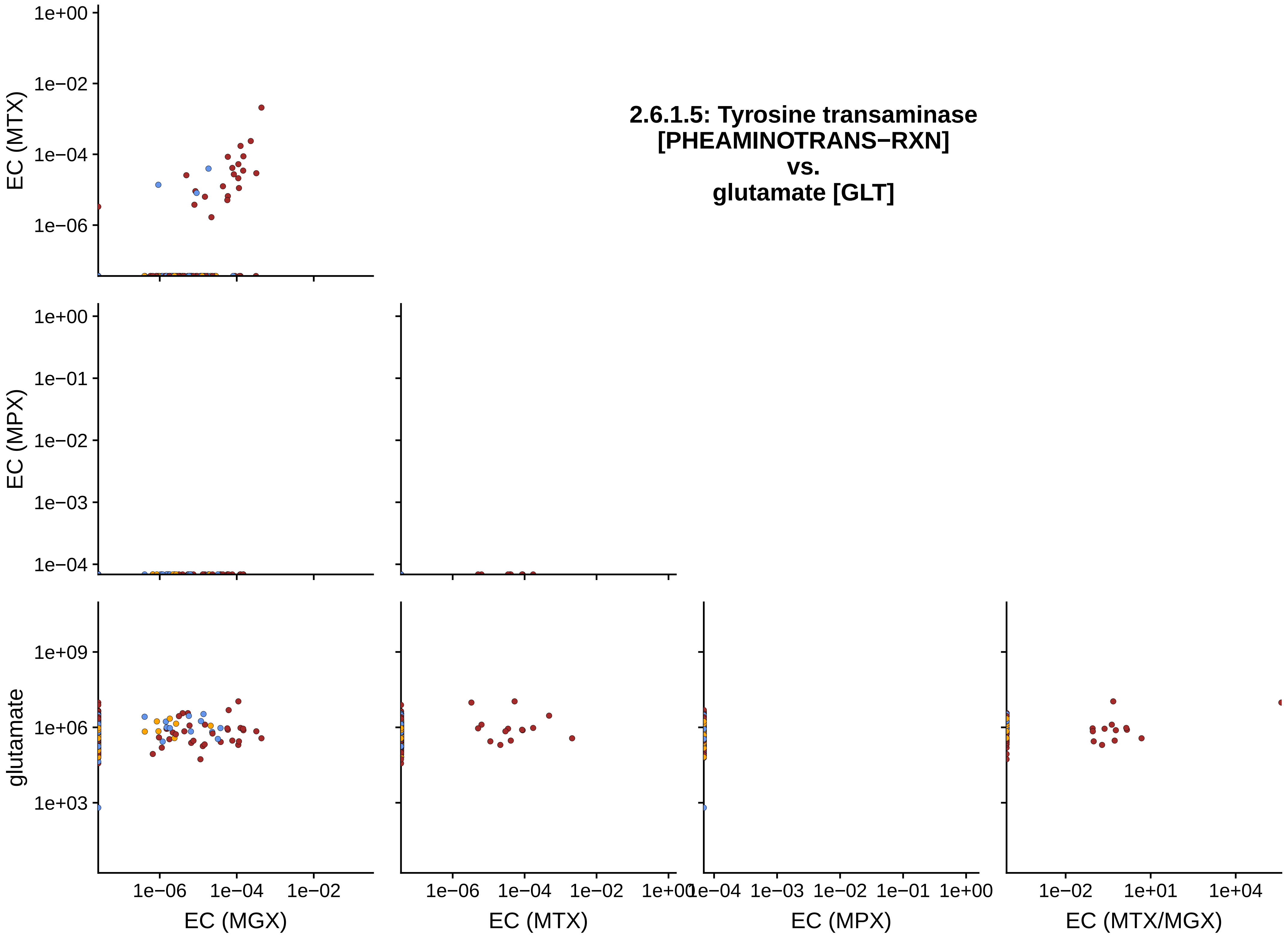

2.6.1.5: Tyrosine transaminase
[PHEAMINOTRANS−RXN]
vs.
glutamate [GLT]
EC (MTX)
EC (MPX)
glutamate
EC (MGX)
EC (MTX)
EC (MPX)
EC (MTX/MGX)
1e+00
1e−02
1e−04
1e−06
1e+00
1e−01
1e−02
1e−03
1e−04
1e+09
1e+06
1e+03
1e−06
1e−04
1e−02
1e−06
1e−04
1e−02
1e+00
1e−04
1e−03
1e−02
1e−01
1e+00
1e−02
1e+01
1e+04

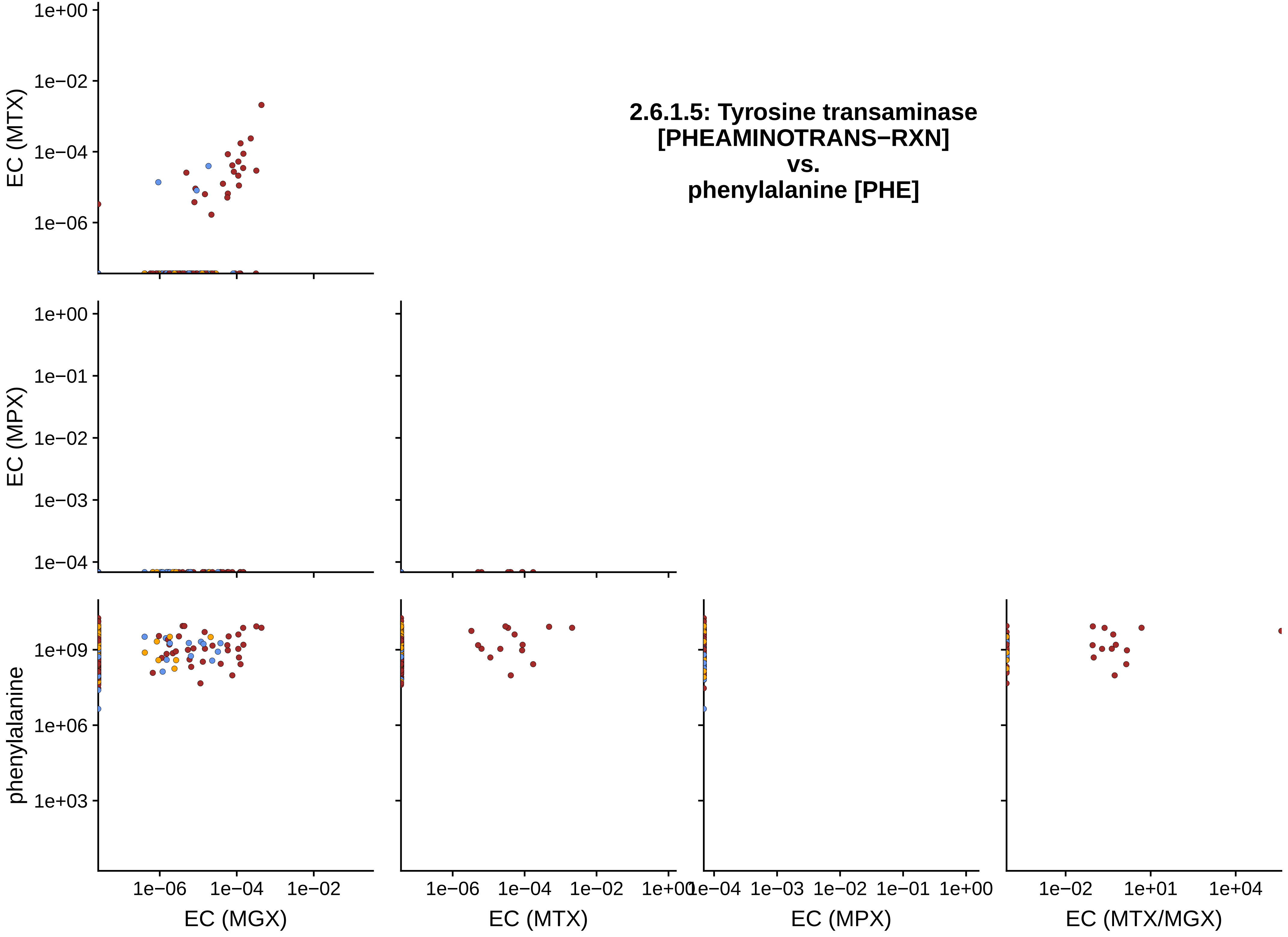
2.6.1.5: Tyrosine transaminase
[PHEAMINOTRANS−RXN]
vs.
phenylalanine [PHE]
EC (MTX)
EC (MPX)
phenylalanine
EC (MGX)
EC (MTX)
EC (MPX)
EC (MTX/MGX)
1e+00
1e−02
1e−04
1e−06
1e+00
1e−01
1e−02
1e−03
1e−04
1e+09
1e+06
1e+03
1e−06
1e−04
1e−02
1e−06
1e−04
1e−02
1e+00
1e−04
1e−03
1e−02
1e−01
1e+00
1e−02
1e+01
1e+04

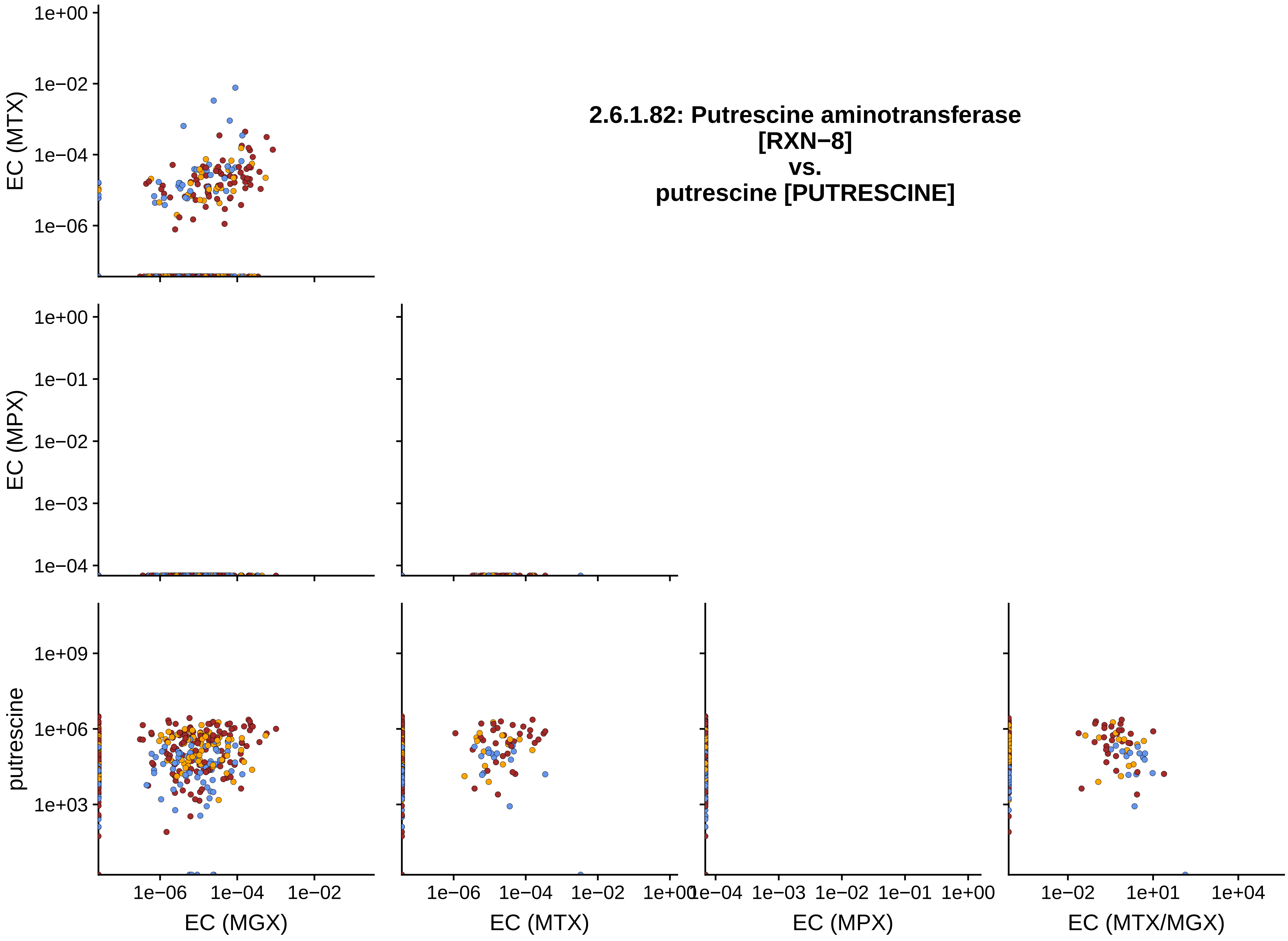

2.6.1.82: Putrescine aminotransferase
[RXN−8]
vs.
putrescine [PUTRESCINE]
EC (MTX)
EC (MPX)
putrescine
EC (MGX)
EC (MTX)
EC (MPX)
EC (MTX/MGX)
1e+00
1e−02
1e−04
1e−06
1e−01
1e−03
1e+09
1e+06
1e+03
1e+04
1e+01

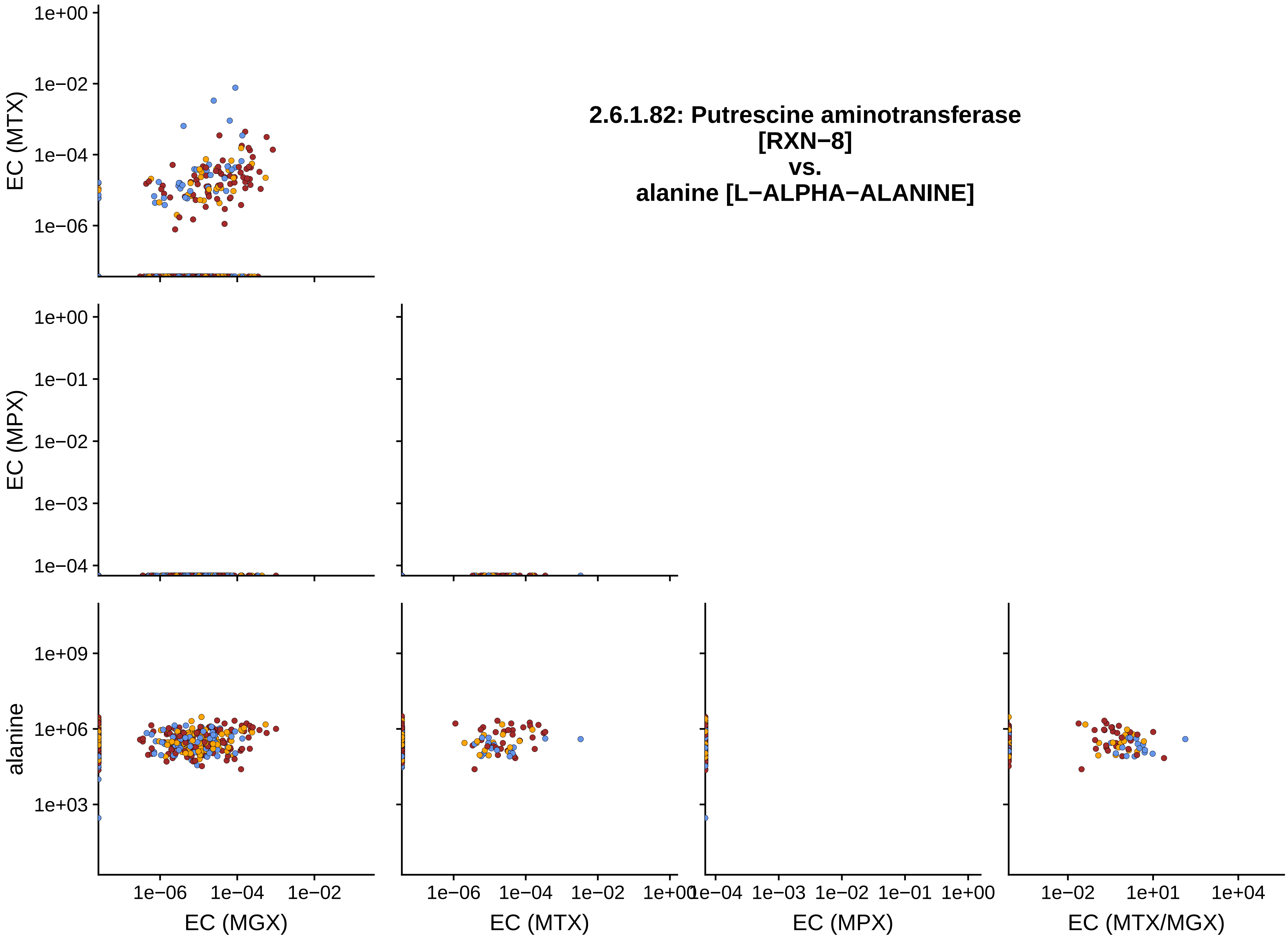

2.6.1.82: Putrescine aminotransferase
[RXN−8]
vs.
alanine [L−ALPHA−ALANINE]
EC (MTX)
EC (MPX)
alanine
EC (MGX)
EC (MTX)
EC (MPX)
EC (MTX/MGX)
1e+00
1e−02
1e−04
1e−06
1e−01
1e−03
1e+09
1e+06
1e+03
1e+01
1e+04

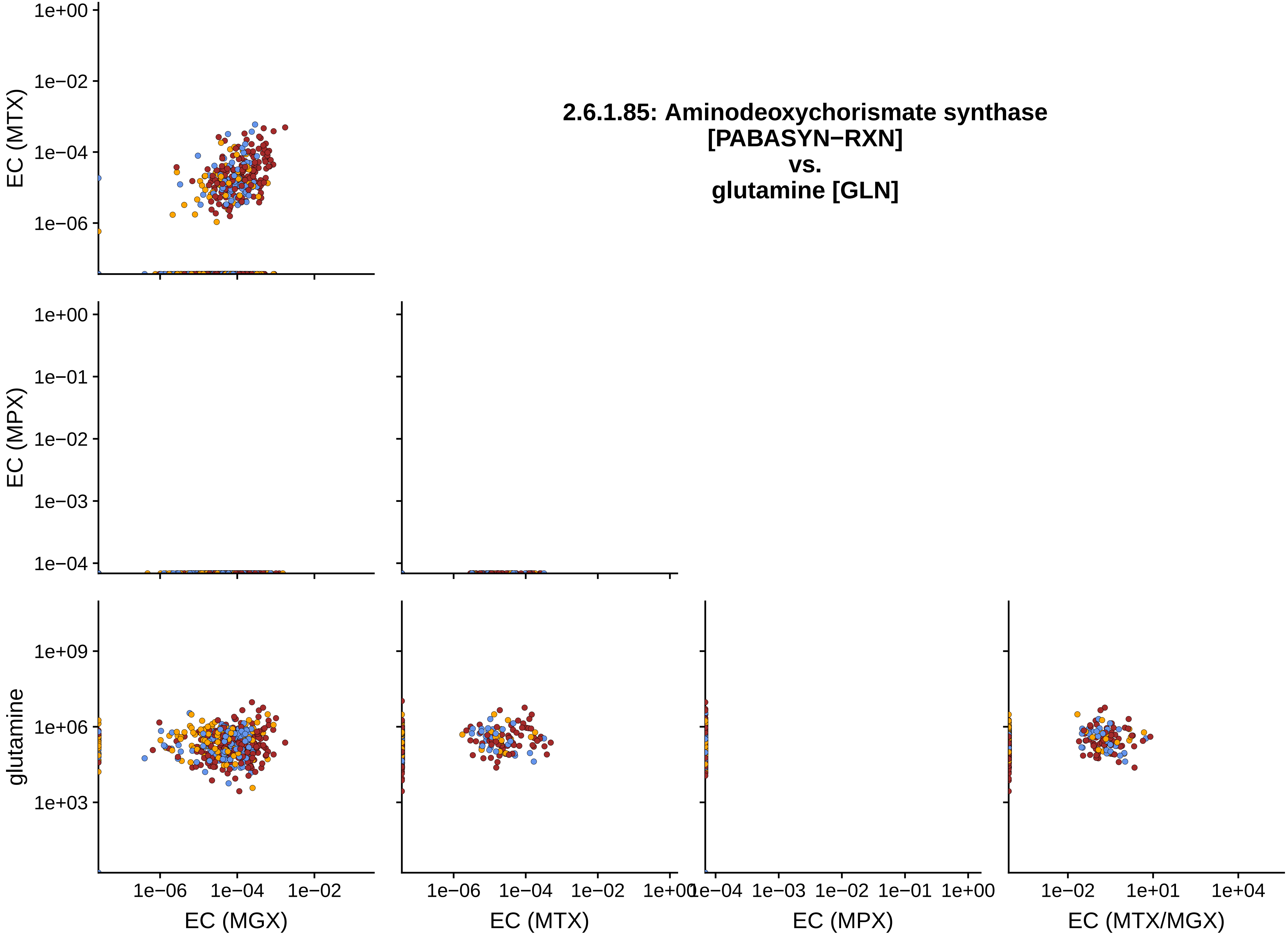
2.6.1.85: Aminodeoxychorismate synthase
[PABASYN−RXN]
vs.
glutamine [GLN]
EC (MTX)
EC (MPX)
glutamine
EC (MGX)
EC (MTX)
EC (MPX)
EC (MTX/MGX)
1e+00
1e−02
1e−04
1e−06
1e−01
1e−03
1e+09
1e+06
1e+03
1e−06
1e−04
1e−02
1e+00
1e−03
1e−01
1e+01
1e+04

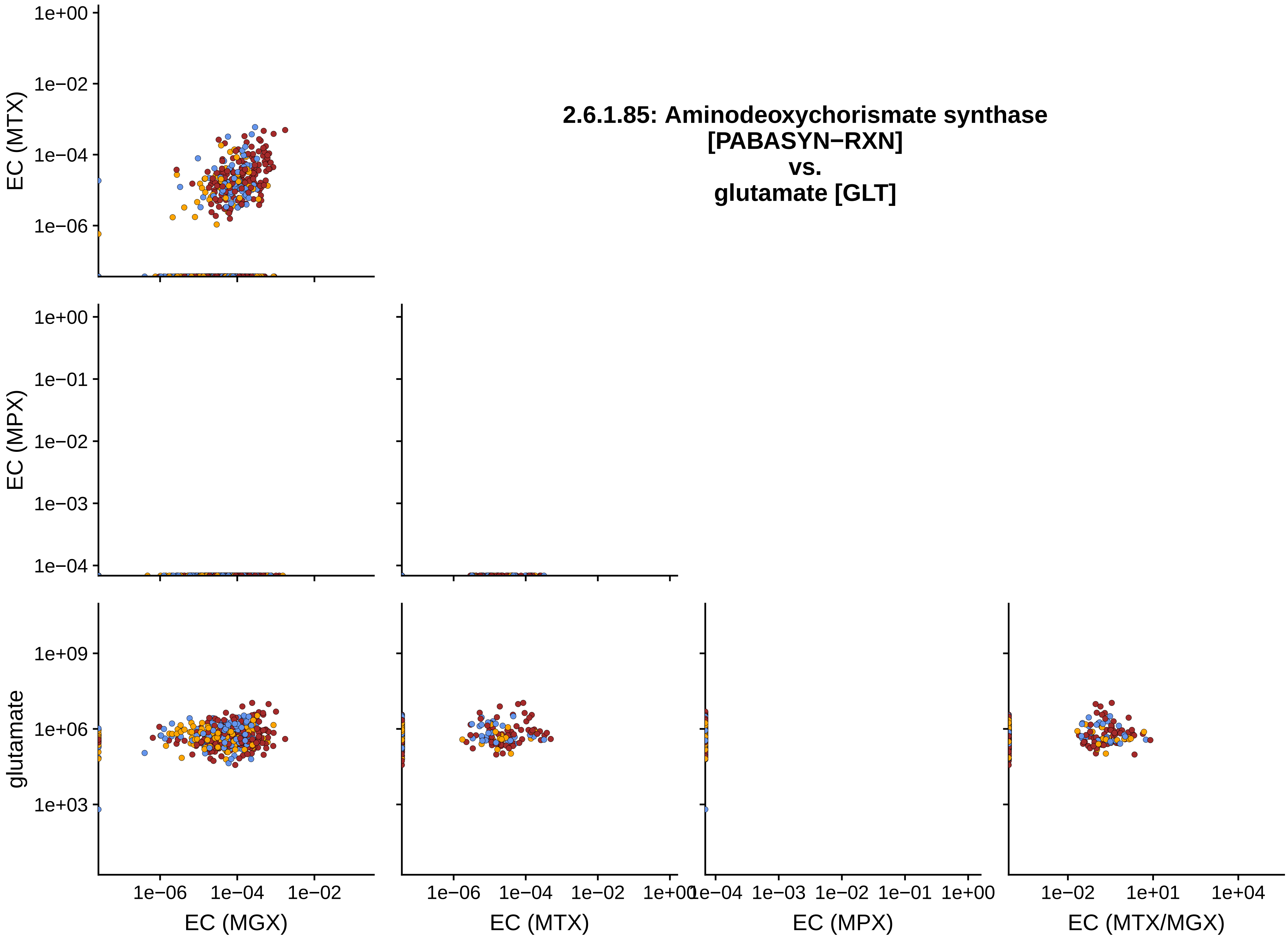

2.6.1.85: Aminodeoxychorismate synthase
[PABASYN−RXN]
vs.
glutamate [GLT]
EC (MTX)
EC (MPX)
glutamate
EC (MGX)
EC (MTX)
EC (MPX)
EC (MTX/MGX)
1e+00
1e−02
1e−04
1e−06
1e−01
1e−03
1e+09
1e+06
1e+03
1e+01
1e+04

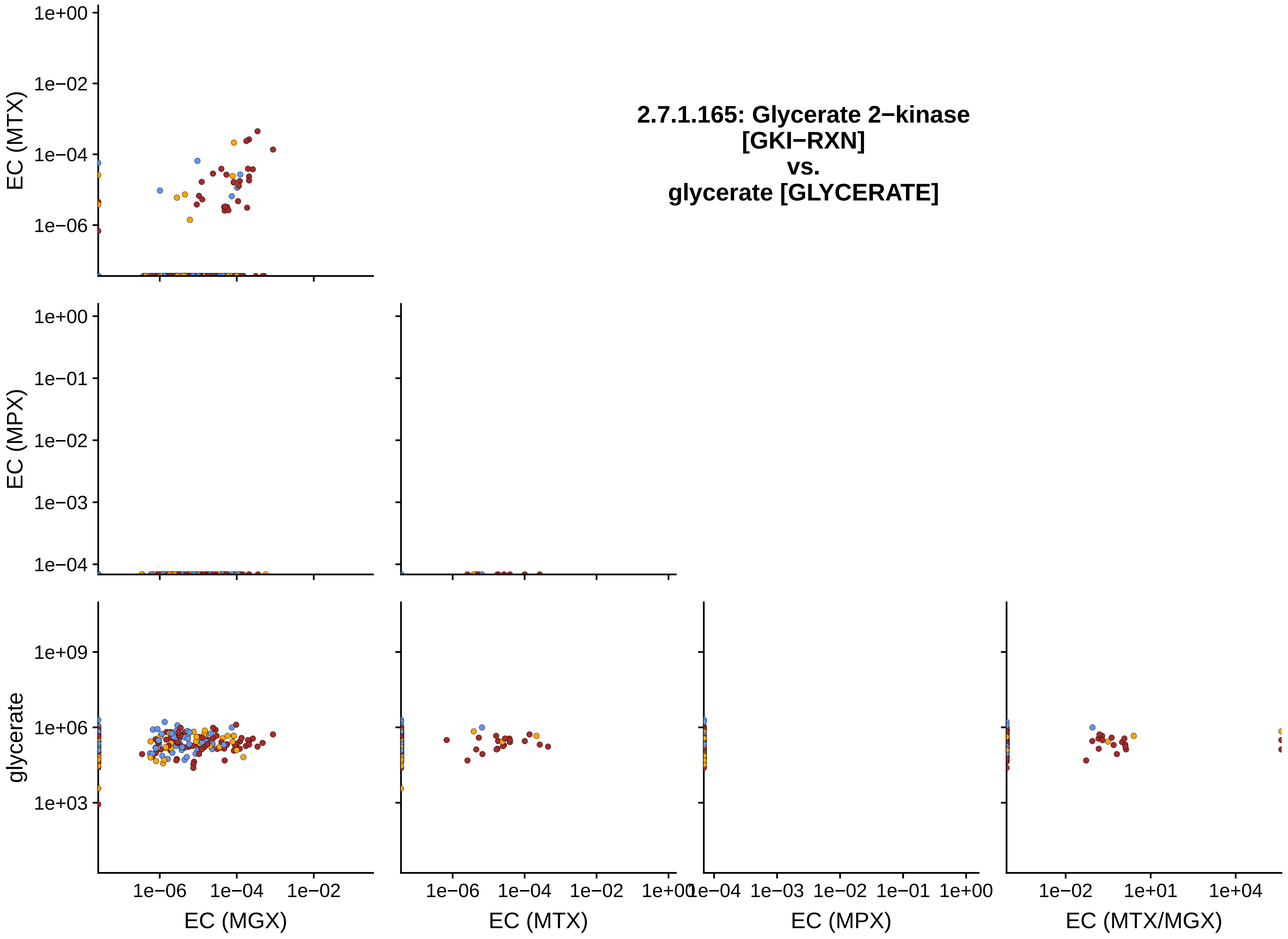
2.7.1.165: Glycerate 2−kinase
[GKI−RXN]
vs.
glycerate [GLYCERATE]
EC (MTX)
EC (MPX)
glycerate
EC (MGX)
EC (MTX)
EC (MPX)
EC (MTX/MGX)
1e+00
1e−02
1e−04
1e−06
1e−01
1e−03
1e+09
1e+06
1e+03
1e+01
1e+04

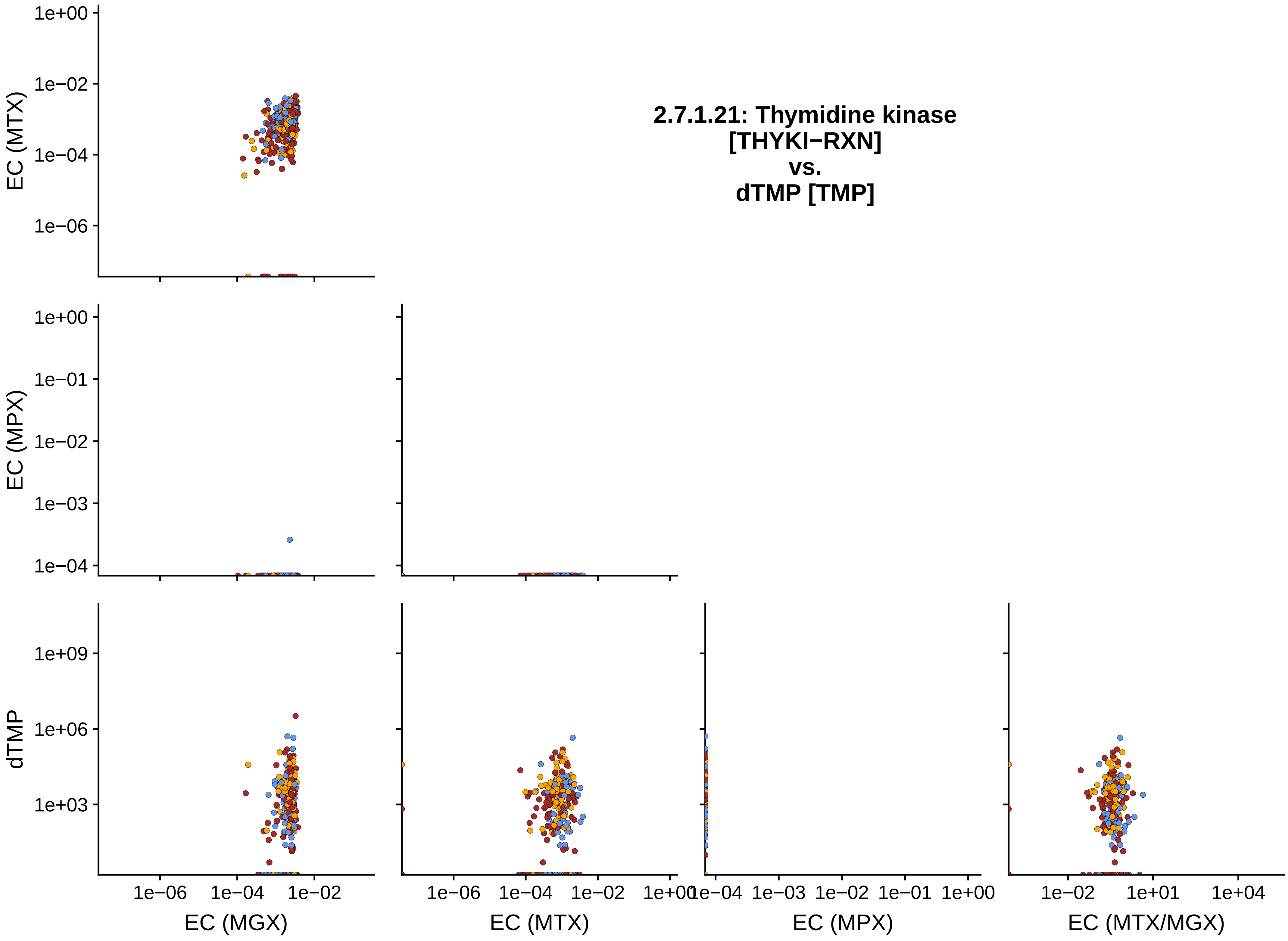
2.7.1.21: Thymidine kinase
[THYKI−RXN]
vs.
dTMP [TMP]
EC (MTX)
EC (MPX)
dTMP
EC (MGX)
EC (MTX)
EC (MPX)
EC (MTX/MGX)
1e+00
1e−02
1e−04
1e−06
1e−01
1e−03
1e+09
1e+06
1e+03
1e+01
1e+04

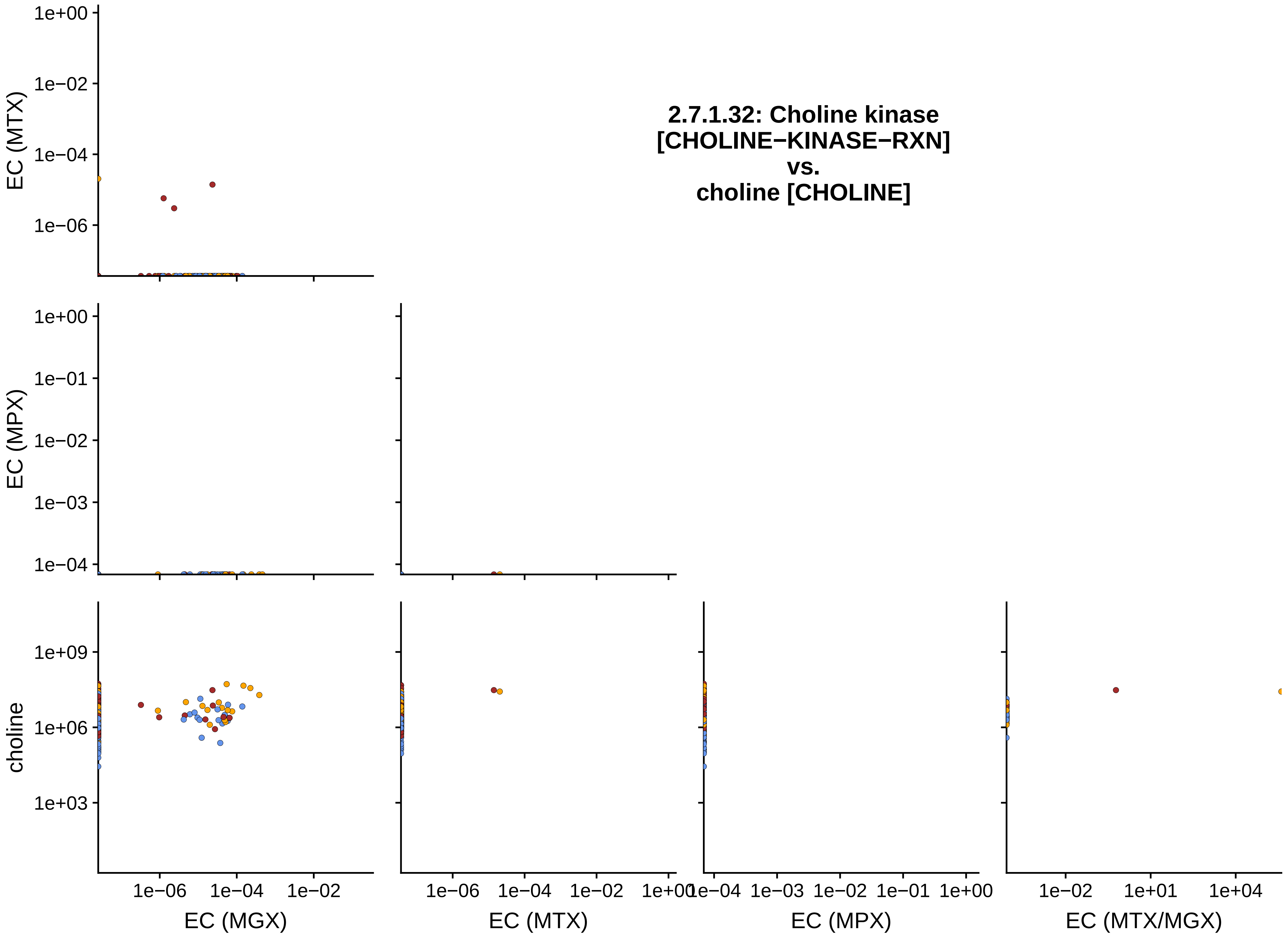
2.7.1.32: Choline kinase
[CHOLINE−KINASE−RXN]
vs.
choline [CHOLINE]
EC (MTX)
EC (MPX)
choline
EC (MGX)
EC (MTX)
EC (MPX)
EC (MTX/MGX)
1e+00
1e−02
1e−04
1e−06
1e−01
1e−03
1e+09
1e+06
1e+03
1e+01
1e+04

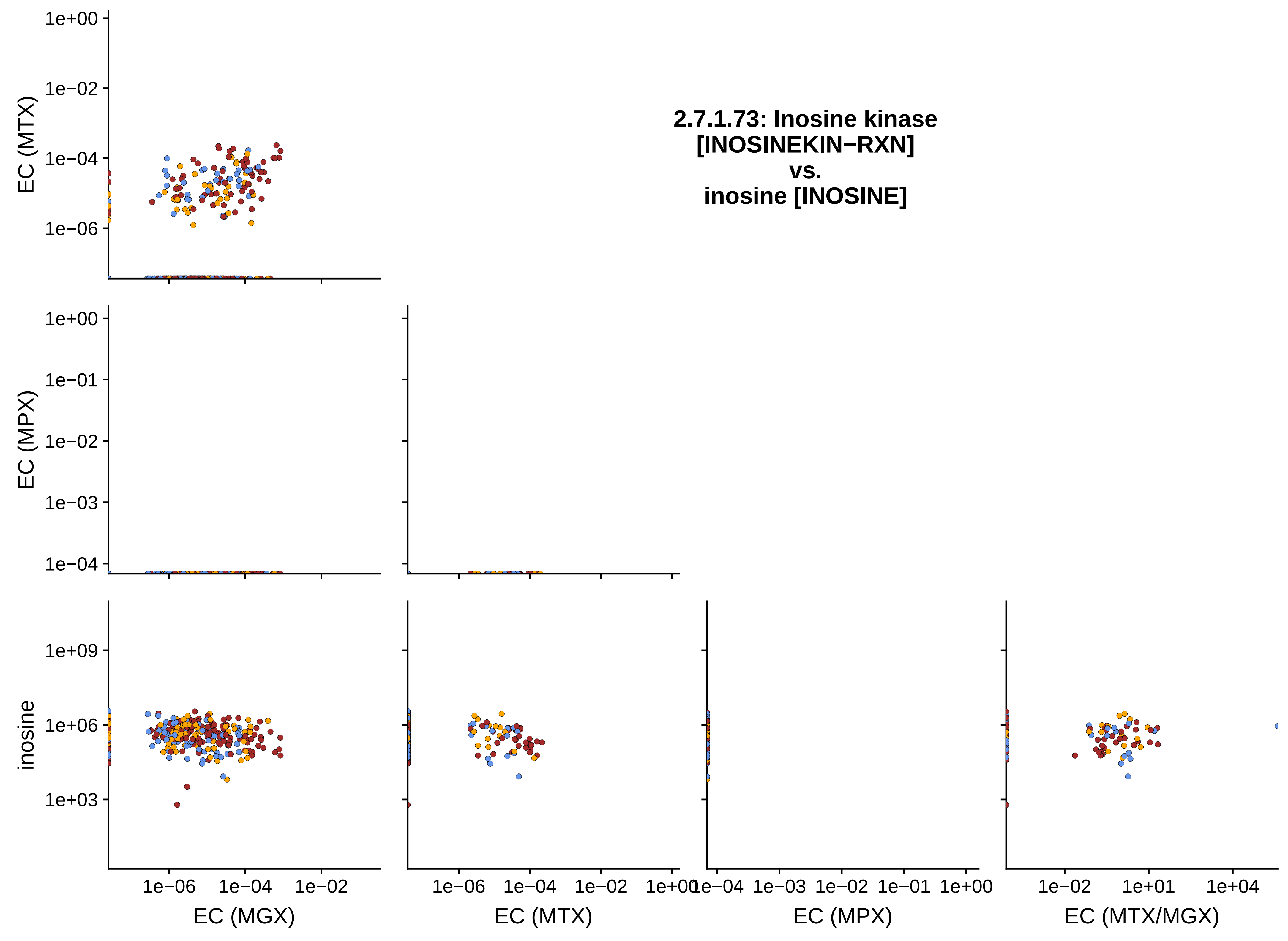
2.7.1.73: Inosine kinase
[INOSINEKIN−RXN]
vs.
inosine [INOSINE]
EC (MTX)
EC (MPX)
inosine
EC (MGX)
EC (MTX)
EC (MPX)
EC (MTX/MGX)
1e+00
1e−02
1e−04
1e−06
1e−01
1e−03
1e+09
1e+06
1e+03
1e+04
1e+01

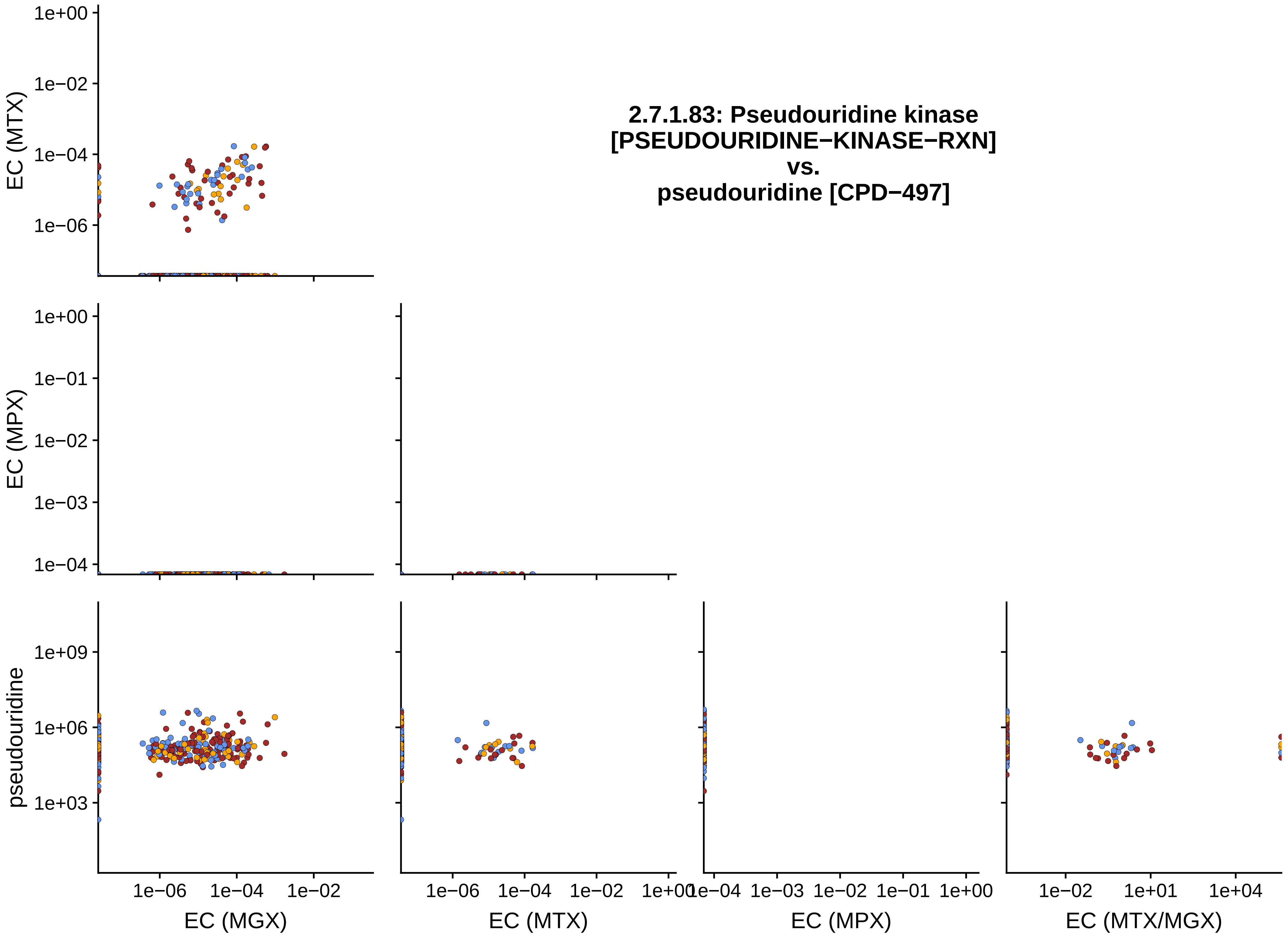
2.7.1.83: Pseudouridine kinase
[PSEUDOURIDINE−KINASE−RXN]
vs.
pseudouridine [CPD−497]
EC (MTX)
EC (MPX)
pseudouridine
EC (MGX)
EC (MTX)
EC (MPX)
EC (MTX/MGX)
1e+00
1e−02
1e−04
1e−06
1e+00
1e−01
1e−02
1e−03
1e−04
1e+09
1e+06
1e+03
1e−06
1e−04
1e−02
1e−06
1e−04
1e−02
1e+00
1e−04
1e−03
1e−02
1e−01
1e+00
1e−02
1e+01
1e+04

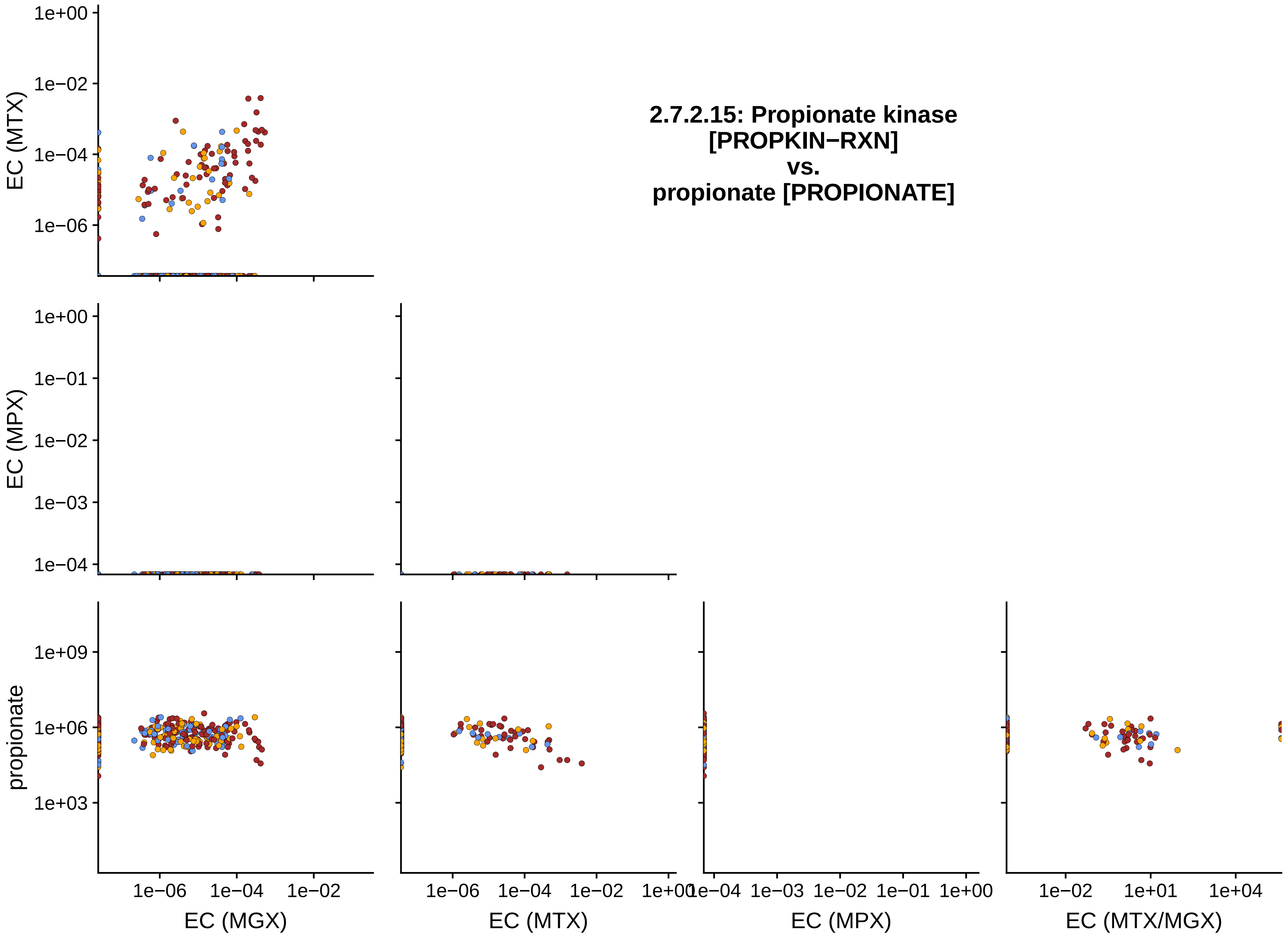

2.7.2.15: Propionate kinase
[PROPKIN−RXN]
vs.
propionate [PROPIONATE]
EC (MTX)
EC (MPX)
propionate
EC (MGX)
EC (MTX)
EC (MPX)
EC (MTX/MGX)
1e+00
1e−02
1e−04
1e−06
1e−01
1e−03
1e+09
1e+06
1e+03
1e+01
1e+04

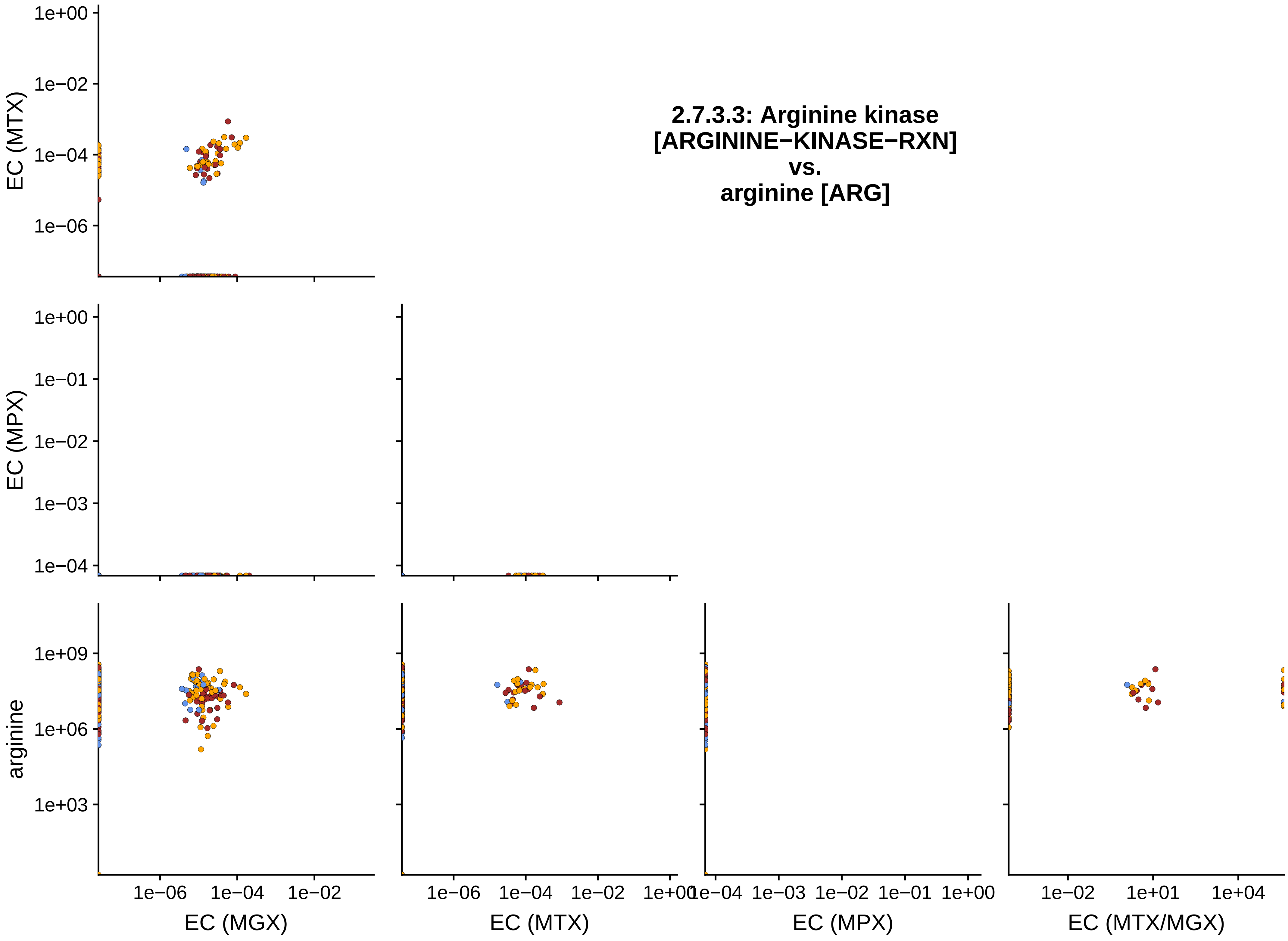

2.7.3.3: Arginine kinase
[ARGININE−KINASE−RXN]
vs.
arginine [ARG]
EC (MTX)
EC (MPX)
arginine
EC (MGX)
EC (MTX)
EC (MPX)
EC (MTX/MGX)
1e+00
1e−02
1e−04
1e−06
1e−01
1e−03
1e+09
1e+06
1e+03
1e+01
1e+04

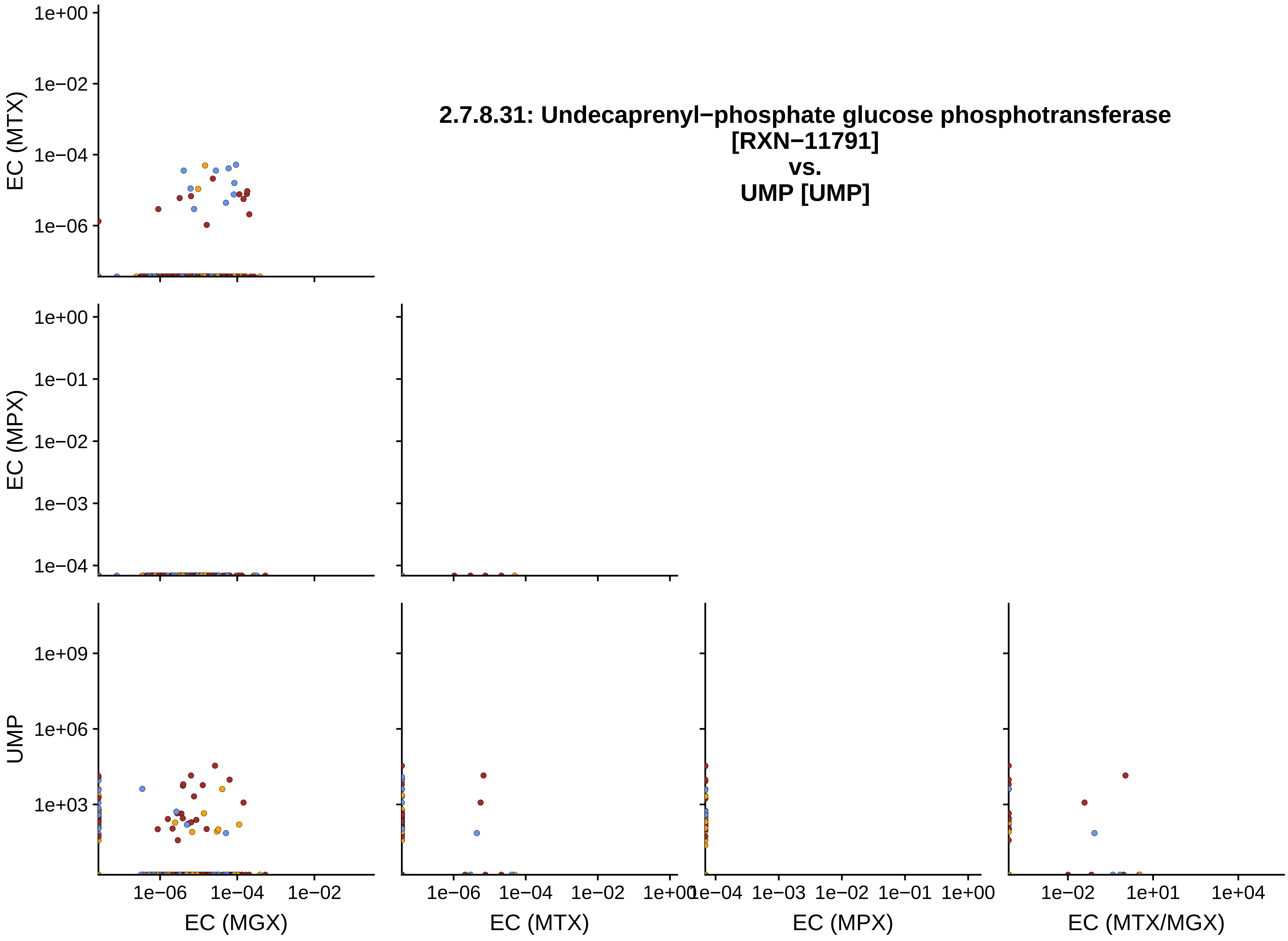

2.7.8.31: Undecaprenyl−phosphate glucose phosphotransferase
[RXN−11791]
vs.
UMP [UMP]
EC (MTX)
EC (MPX)
UMP
EC (MGX)
EC (MTX)
EC (MPX)
EC (MTX/MGX)
1e+00
1e−02
1e−04
1e−06
1e+00
1e−01
1e−02
1e−03
1e−04
1e+09
1e+06
1e+03
1e−06
1e−04
1e−02
1e−06
1e−04
1e−02
1e+00
1e−04
1e−03
1e−02
1e−01
1e+00
1e−02
1e+01
1e+04

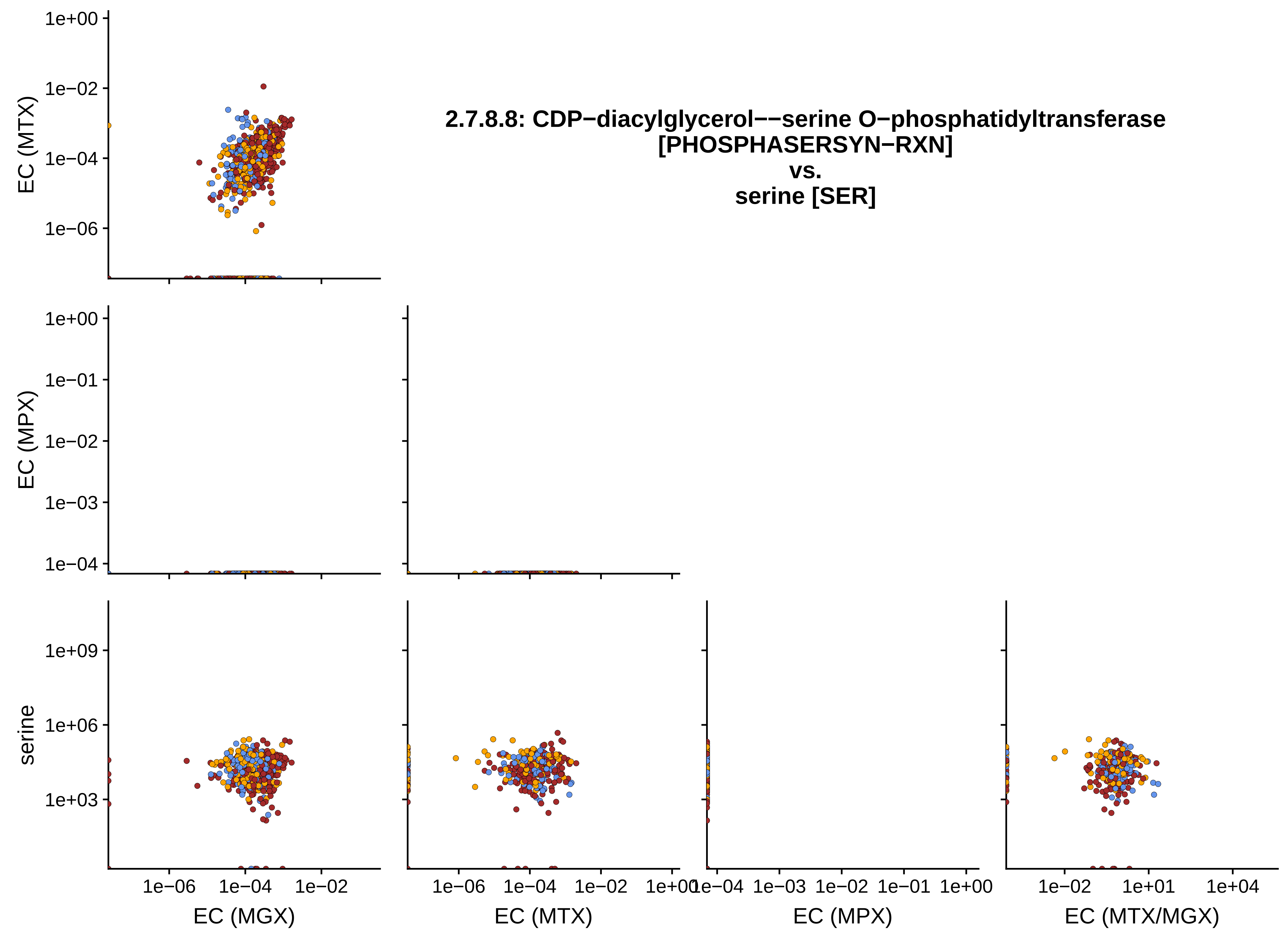
2.7.8.8: CDP−diacylglycerol−−serine O−phosphatidyltransferase
[PHOSPHASERSYN−RXN]
vs.
serine [SER]
EC (MTX)
EC (MPX)
serine
EC (MGX)
EC (MTX)
EC (MPX)
EC (MTX/MGX)
1e+00
1e−02
1e−04
1e−06
1e+00
1e−01
1e−02
1e−03
1e−04
1e+09
1e+06
1e+03
1e−06
1e−04
1e−02
1e−06
1e−04
1e−02
1e+00
1e−04
1e−03
1e−02
1e−01
1e+00
1e−02
1e+01
1e+04

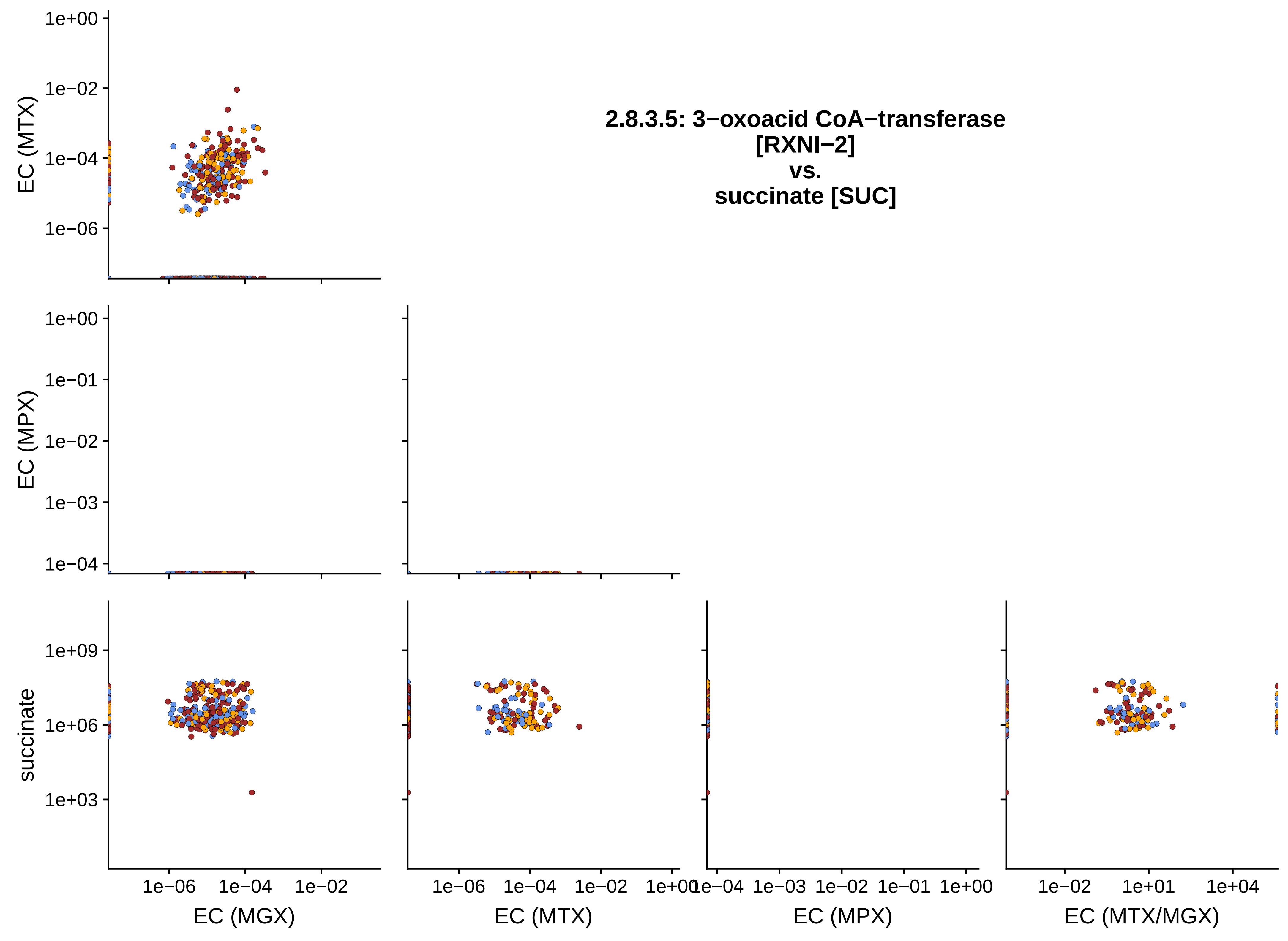

2.8.3.5: 3−oxoacid CoA−transferase
[RXNI−2]
vs.
succinate [SUC]
EC (MTX)
EC (MPX)
succinate
EC (MGX)
EC (MTX)
EC (MPX)
EC (MTX/MGX)
1e+00
1e−02
1e−04
1e−06
1e−01
1e−03
1e+09
1e+06
1e+03
1e+01
1e+04

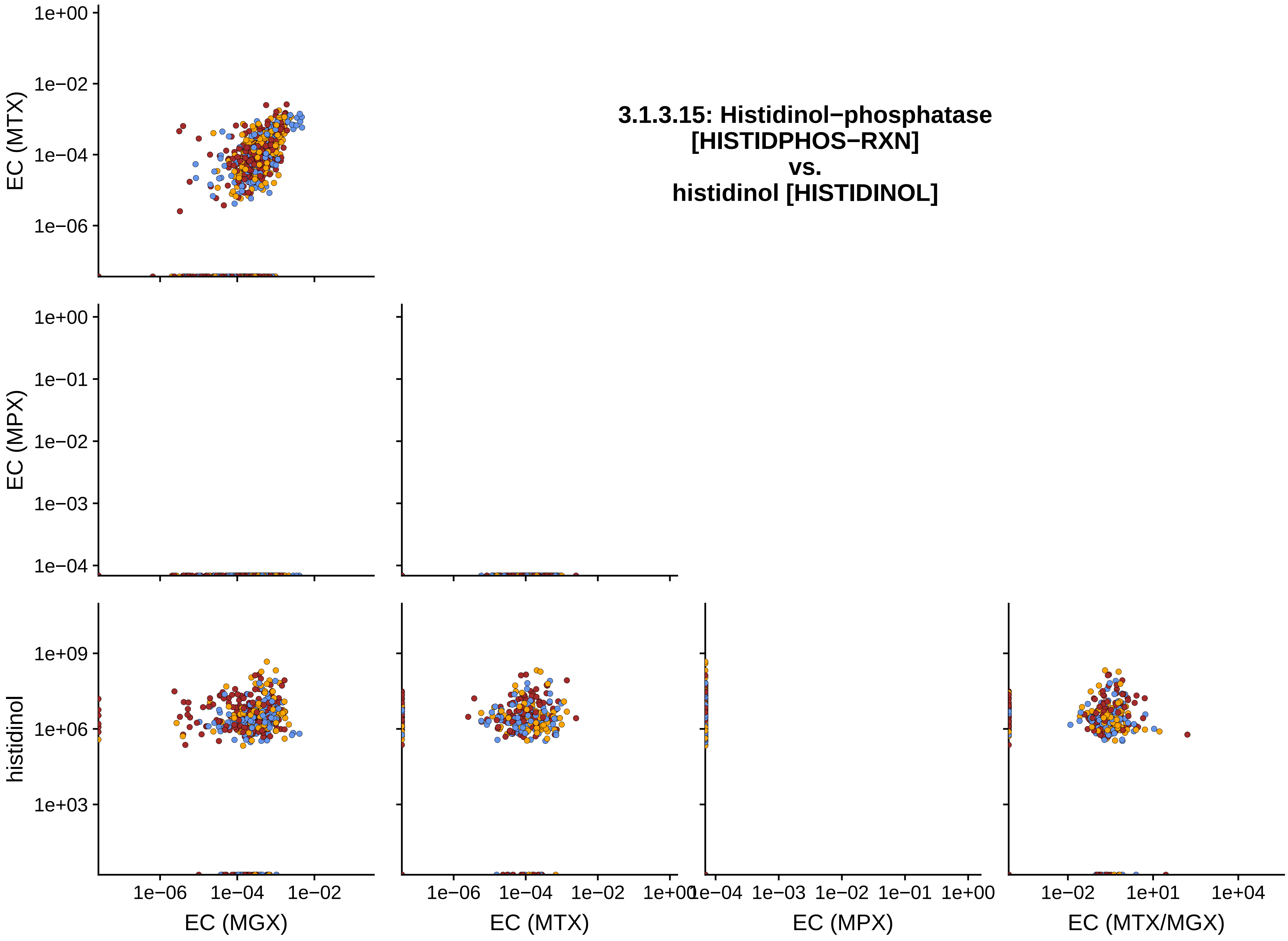
3.1.3.15: Histidinol−phosphatase
[HISTIDPHOS−RXN]
vs.
histidinol [HISTIDINOL]
EC (MTX)
EC (MPX)
histidinol
EC (MGX)
EC (MTX)
EC (MPX)
EC (MTX/MGX)
1e+00
1e−02
1e−04
1e−06
1e−01
1e−03
1e+09
1e+06
1e+03
1e+01
1e+04

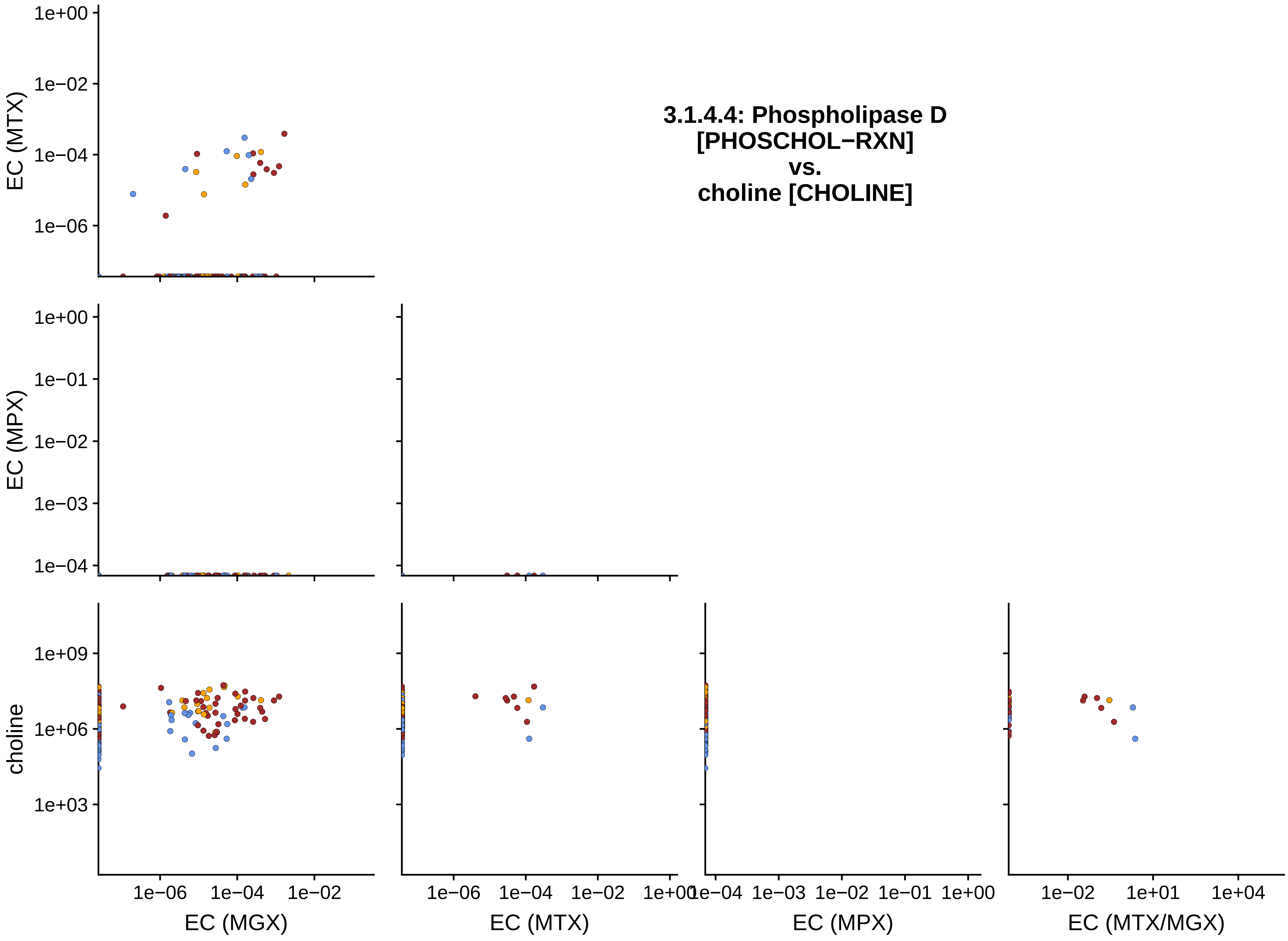

3.1.4.4: Phospholipase D
[PHOSCHOL−RXN]
vs.
choline [CHOLINE]
EC (MTX)
EC (MPX)
choline
EC (MGX)
EC (MTX)
EC (MPX)
EC (MTX/MGX)
1e+00
1e−02
1e−04
1e−06
1e−01
1e−03
1e+09
1e+06
1e+03
1e+01
1e+04

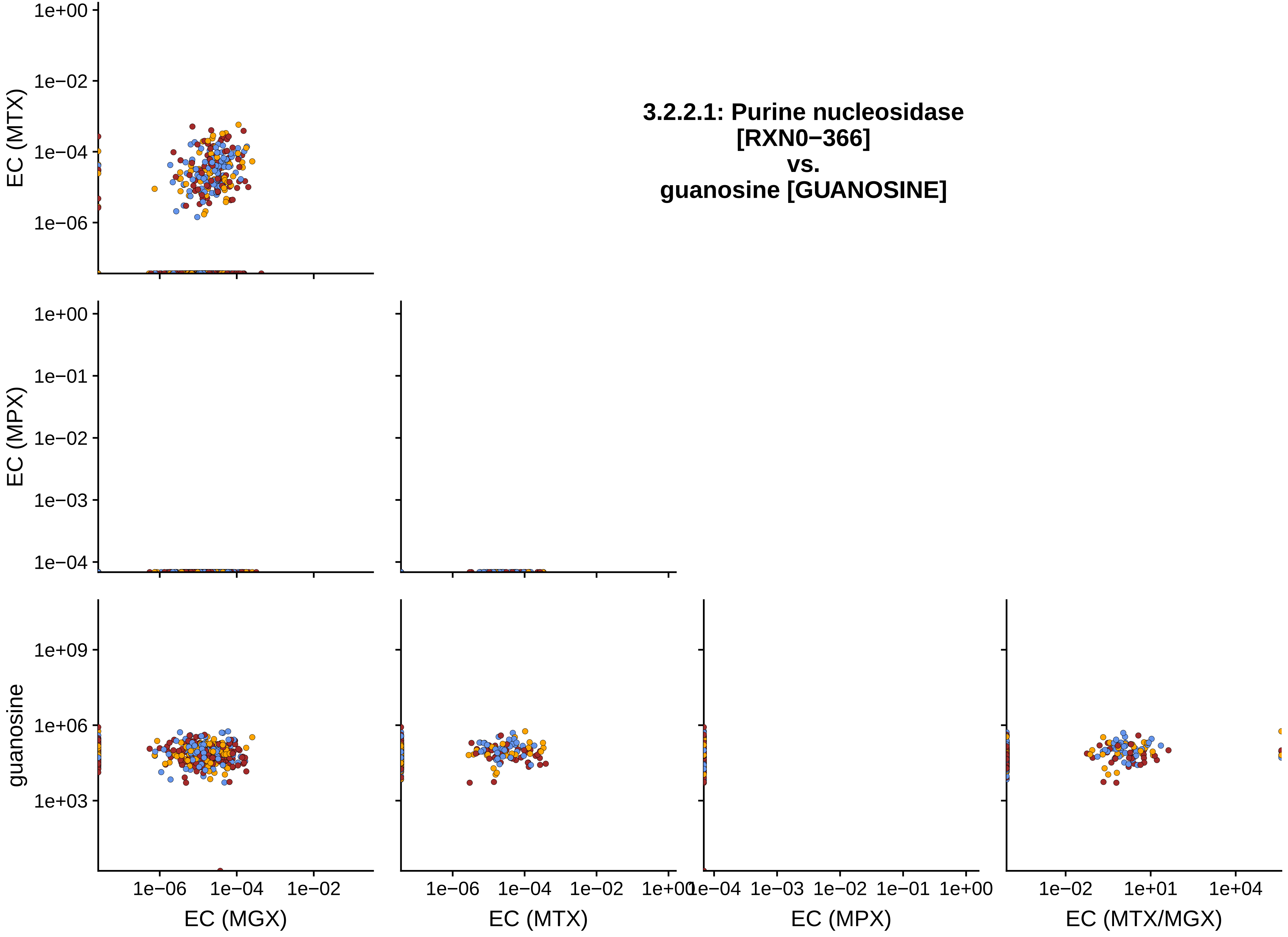

3.2.2.1: Purine nucleosidase
[RXN0−366]
vs.
guanosine [GUANOSINE]
EC (MTX)
EC (MPX)
guanosine
EC (MGX)
EC (MTX)
EC (MPX)
EC (MTX/MGX)
1e+00
1e−02
1e−04
1e−06
1e−01
1e−03
1e+09
1e+06
1e+03
1e+01
1e+04

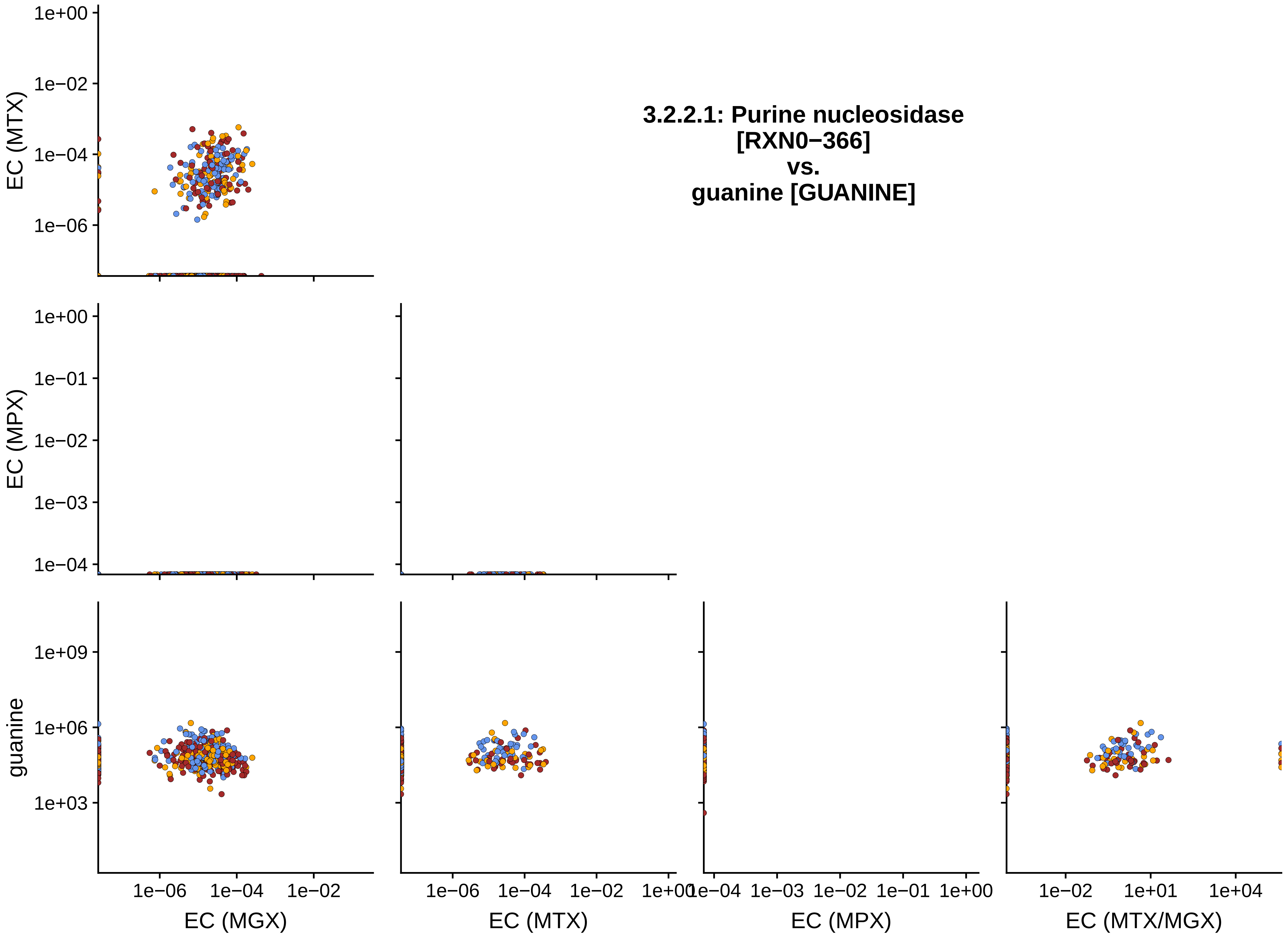
3.2.2.1: Purine nucleosidase
[RXN0−366]
vs.
guanine [GUANINE]
EC (MTX)
EC (MPX)
guanine
EC (MGX)
EC (MTX)
EC (MPX)
EC (MTX/MGX)
1e+00
1e−02
1e−04
1e−06
1e−01
1e−03
1e+09
1e+06
1e+03
1e+01
1e+04

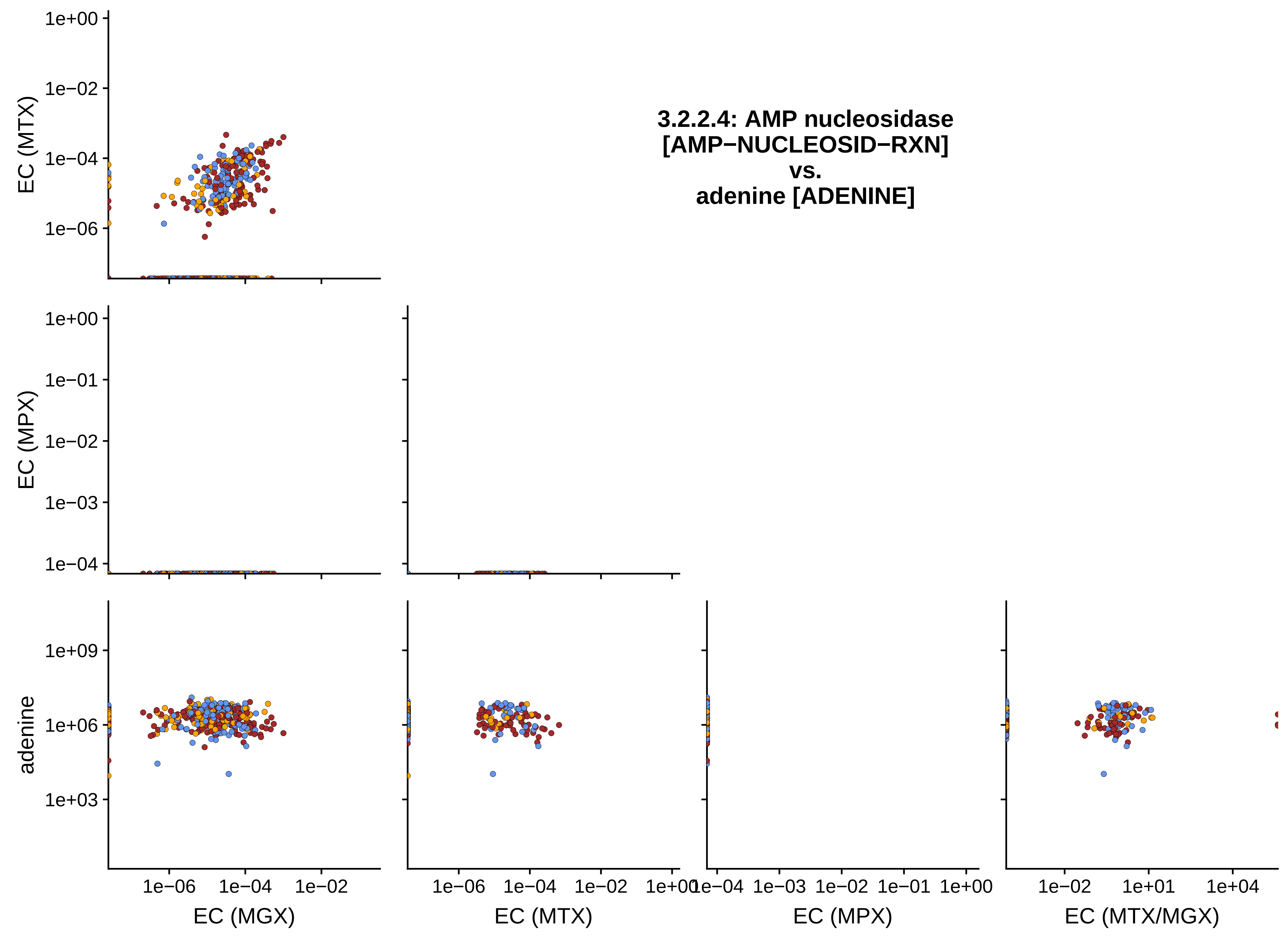
3.2.2.4: AMP nucleosidase
[AMP−NUCLEOSID−RXN]
vs.
adenine [ADENINE]
EC (MTX)
EC (MPX)
adenine
EC (MGX)
EC (MTX)
EC (MPX)
EC (MTX/MGX)
1e+00
1e−02
1e−04
1e−06
1e−01
1e−03
1e+09
1e+06
1e+03
1e+01
1e+04

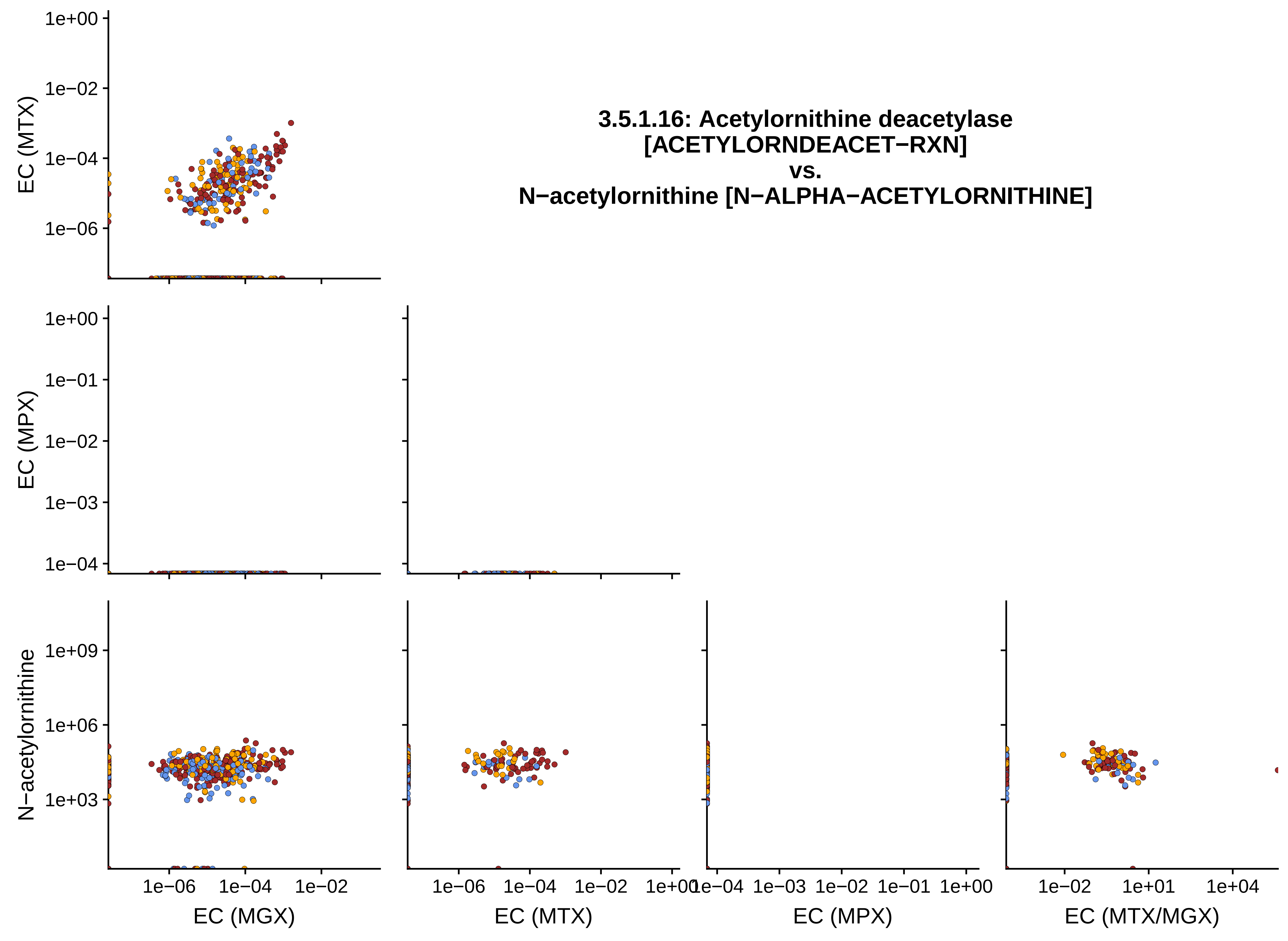
3.5.1.16: Acetylornithine deacetylase
[ACETYLORNDEACET−RXN]
vs.
N−acetylornithine [N−ALPHA−ACETYLORNITHINE]
EC (MTX)
EC (MPX)
N−acetylornithine
EC (MGX)
EC (MTX)
EC (MPX)
EC (MTX/MGX)
1e+00
1e−02
1e−04
1e−06
1e−01
1e−03
1e+09
1e+06
1e+03
1e+01
1e+04

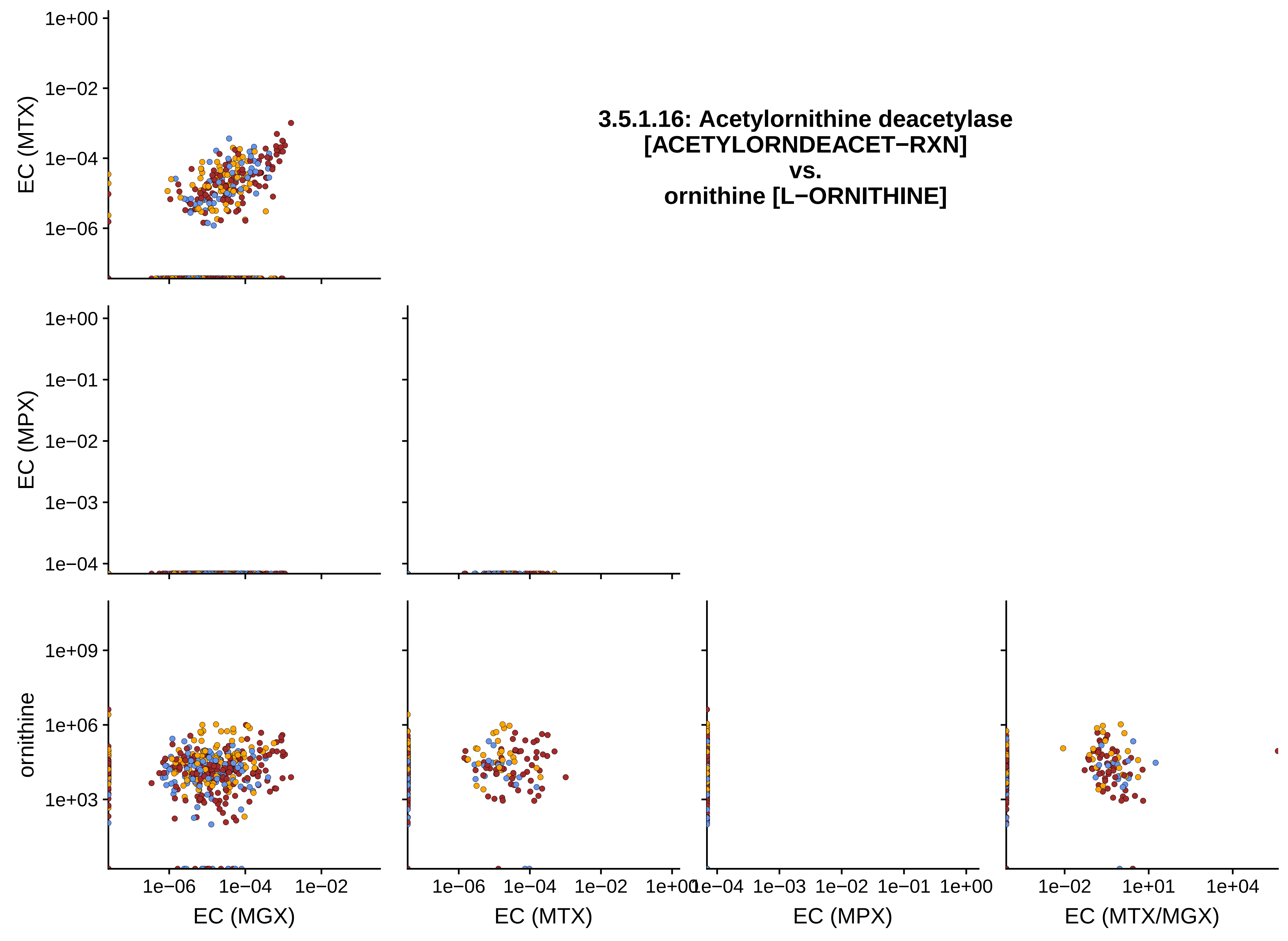

3.5.1.16: Acetylornithine deacetylase
[ACETYLORNDEACET−RXN]
vs.
ornithine [L−ORNITHINE]
EC (MTX)
EC (MPX)
ornithine
EC (MGX)
EC (MTX)
EC (MPX)
EC (MTX/MGX)
1e+00
1e−02
1e−04
1e−06
1e−01
1e−03
1e+09
1e+06
1e+03
1e+01
1e+04

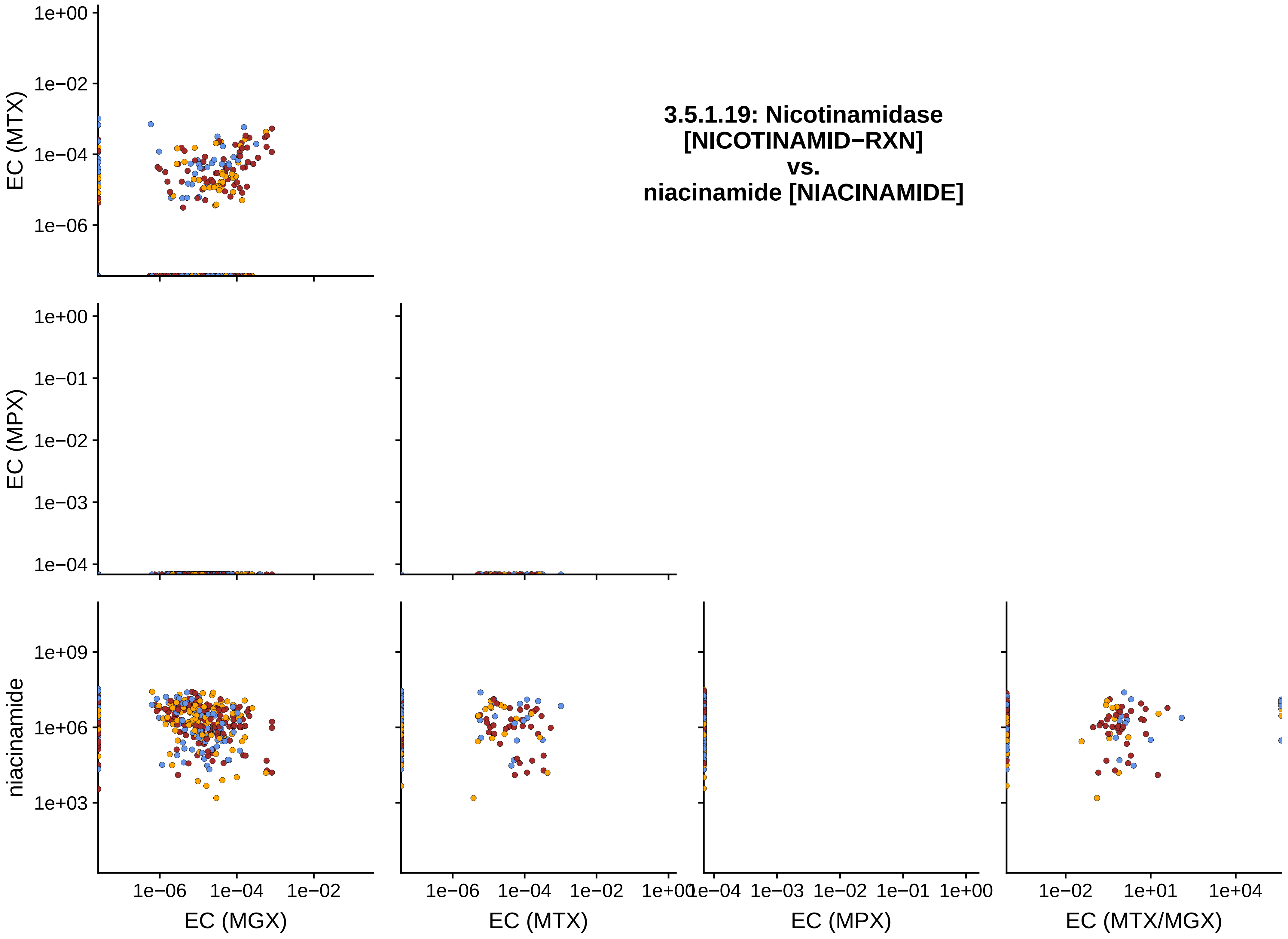

3.5.1.19: Nicotinamidase
[NICOTINAMID−RXN]
vs.
niacinamide [NIACINAMIDE]
EC (MTX)
EC (MPX)
niacinamide
EC (MGX)
EC (MTX)
EC (MPX)
EC (MTX/MGX)
1e+00
1e−02
1e−04
1e−06
1e+00
1e−01
1e−02
1e−03
1e−04
1e+09
1e+06
1e+03
1e−06
1e−04
1e−02
1e−06
1e−04
1e−02
1e+00
1e−04
1e−03
1e−02
1e−01
1e+00
1e−02
1e+01
1e+04

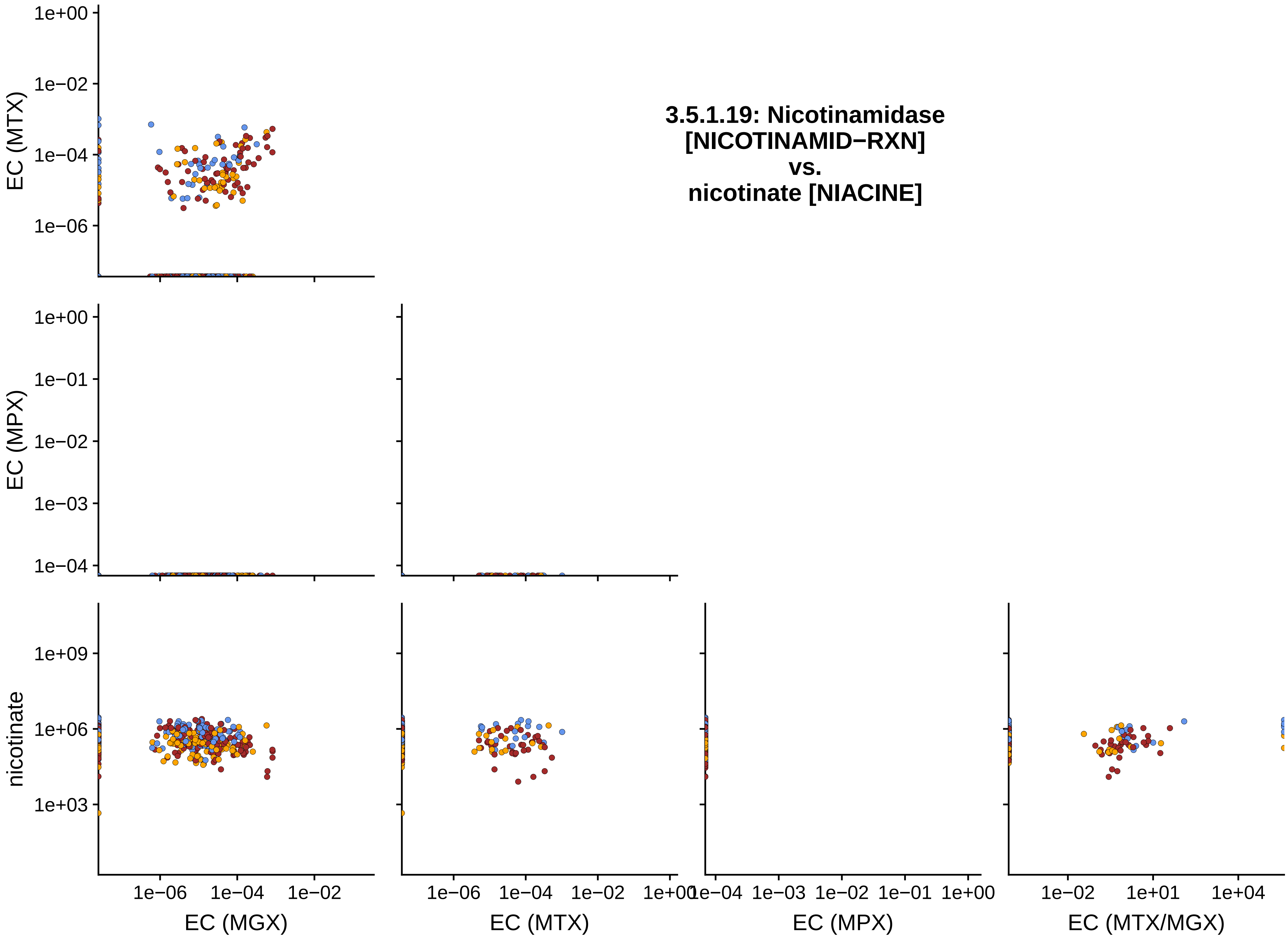
3.5.1.19: Nicotinamidase
[NICOTINAMID−RXN]
vs.
nicotinate [NIACINE]
EC (MTX)
EC (MPX)
nicotinate
EC (MGX)
EC (MTX)
EC (MPX)
EC (MTX/MGX)
1e+00
1e−02
1e−04
1e−06
1e−01
1e−03
1e+09
1e+06
1e+03
1e+01
1e+04

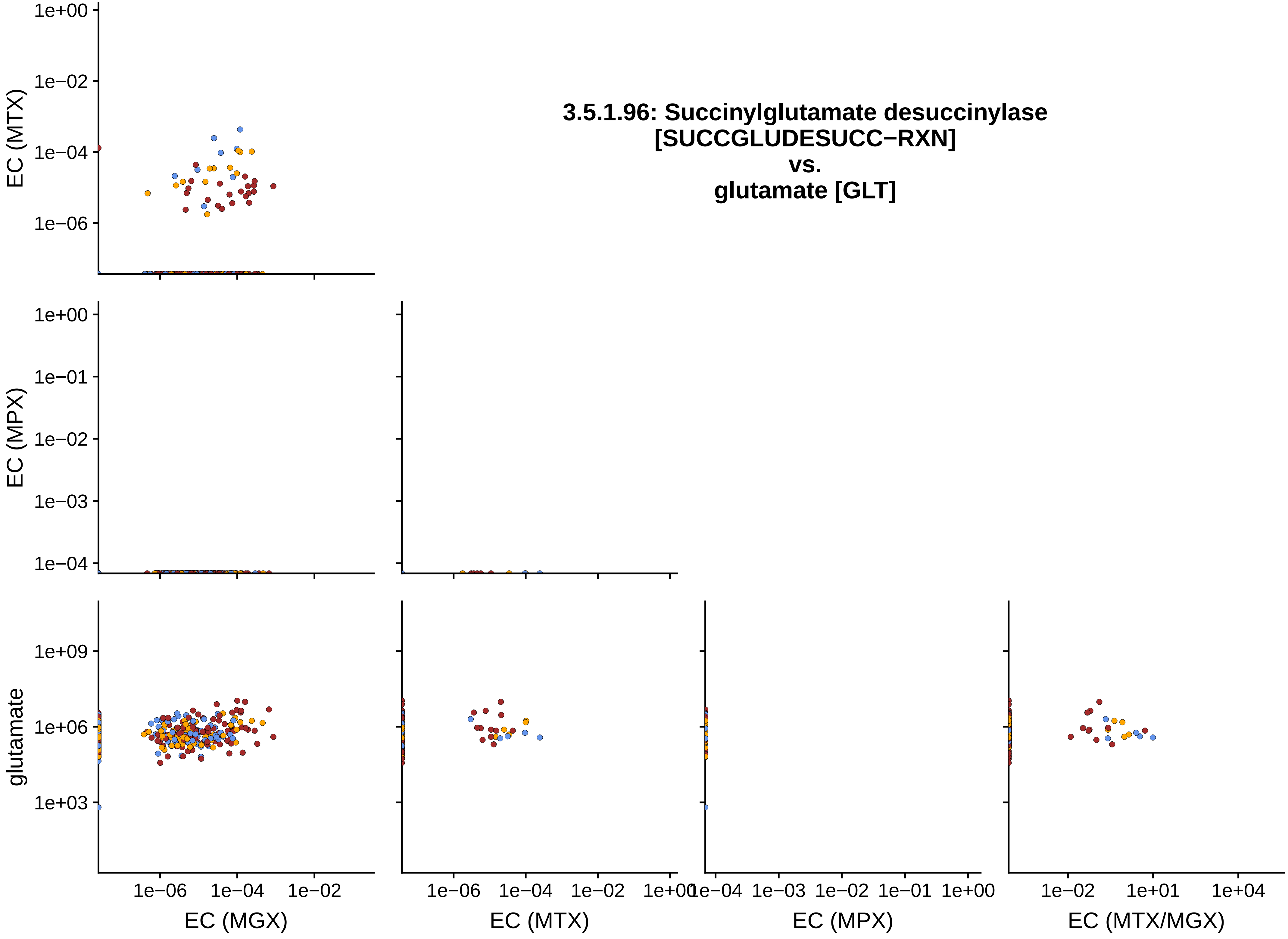

3.5.1.96: Succinylglutamate desuccinylase
[SUCCGLUDESUCC−RXN]
vs.
glutamate [GLT]
EC (MTX)
EC (MPX)
glutamate
EC (MGX)
EC (MTX)
EC (MPX)
EC (MTX/MGX)
1e+00
1e−02
1e−04
1e−06
1e−01
1e−03
1e+09
1e+06
1e+03
1e+01
1e+04

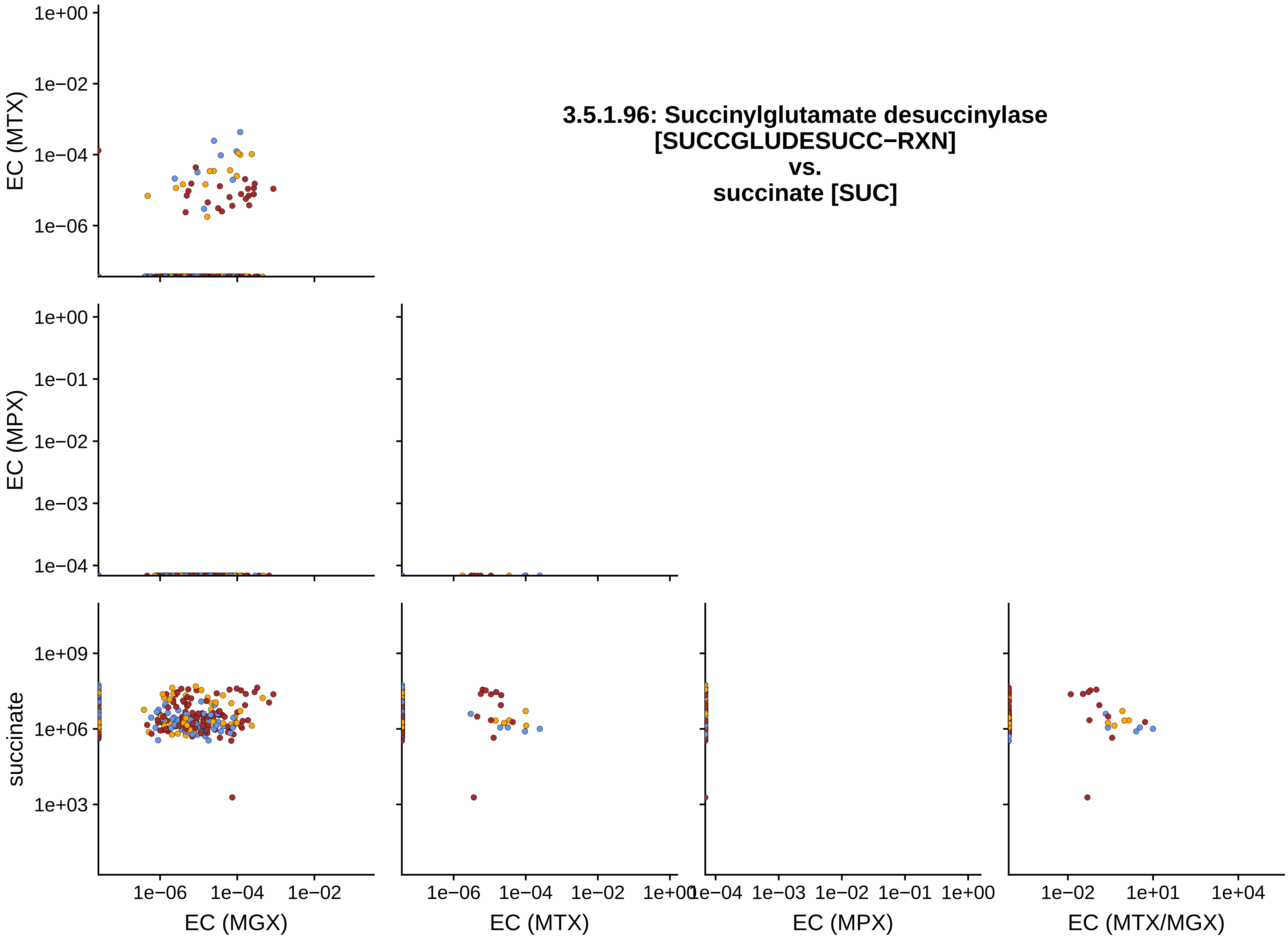
3.5.1.96: Succinylglutamate desuccinylase
[SUCCGLUDESUCC−RXN]
vs.
succinate [SUC]
EC (MTX)
EC (MPX)
succinate
EC (MGX)
EC (MTX)
EC (MPX)
EC (MTX/MGX)
1e+00
1e−02
1e−04
1e−06
1e−01
1e−03
1e+09
1e+06
1e+03
1e+01
1e+04

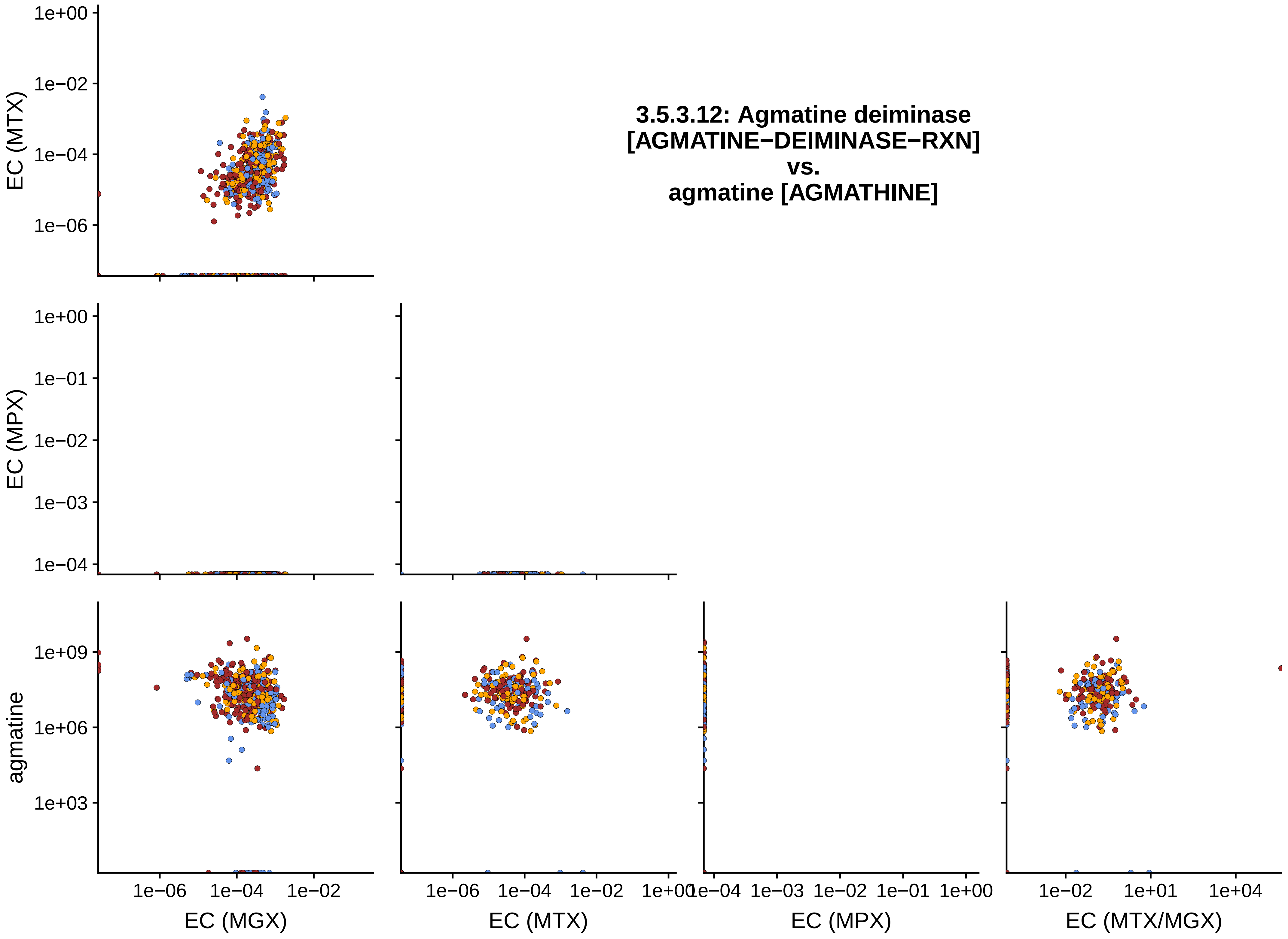

3.5.3.12: Agmatine deiminase
[AGMATINE−DEIMINASE−RXN]
vs.
agmatine [AGMATHINE]
EC (MTX)
EC (MPX)
agmatine
EC (MGX)
EC (MTX)
EC (MPX)
EC (MTX/MGX)
1e+00
1e−02
1e−04
1e−06
1e−01
1e−03
1e+09
1e+06
1e+03
1e+01
1e+04

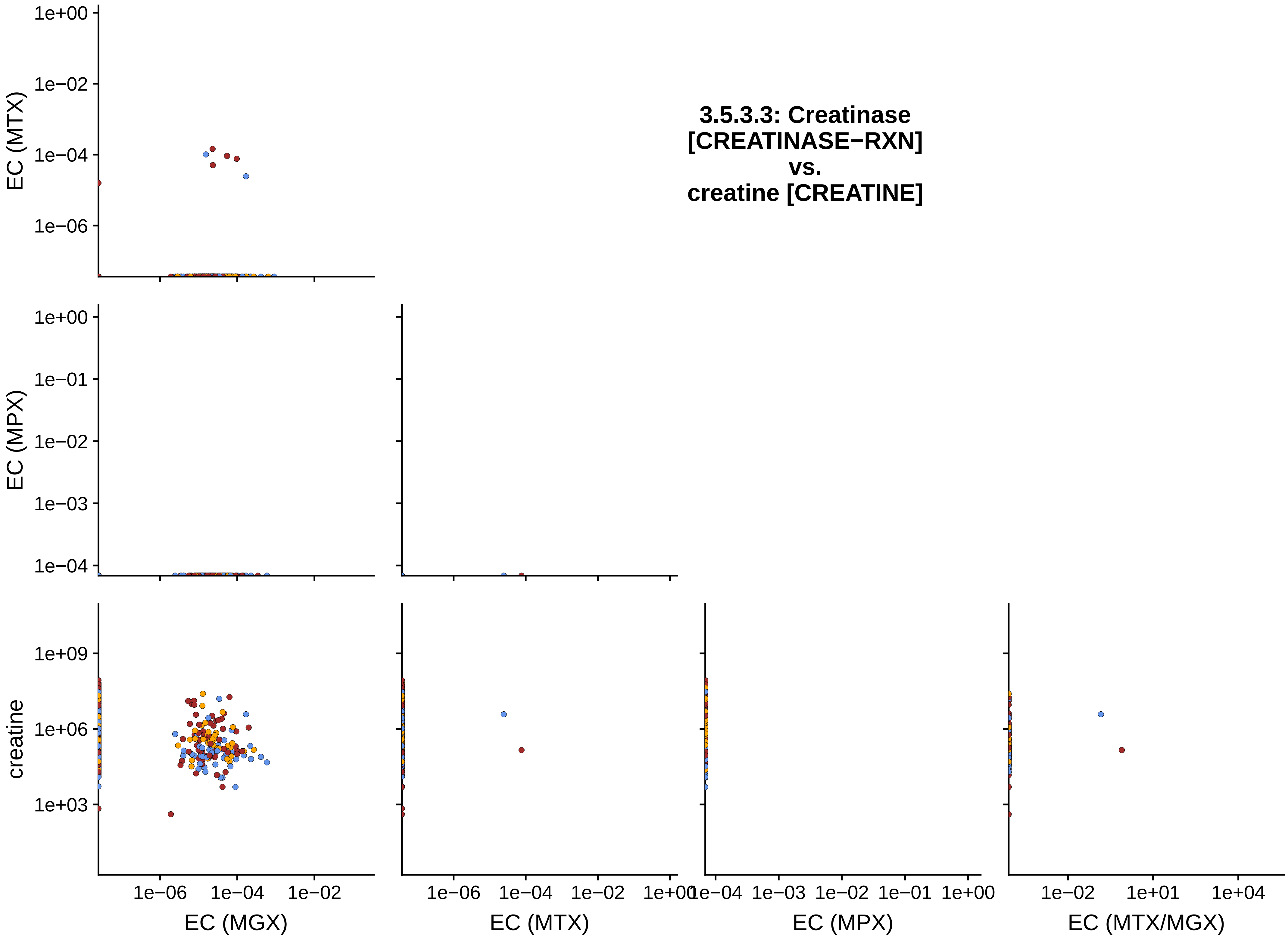

3.5.3.3: Creatinase
[CREATINASE−RXN]
vs.
creatine [CREATINE]
EC (MTX)
EC (MPX)
creatine
EC (MGX)
EC (MTX)
EC (MPX)
EC (MTX/MGX)
1e+00
1e−02
1e−04
1e−06
1e−01
1e−03
1e+09
1e+06
1e+03
1e−06
1e−04
1e−02
1e+00
1e−04
1e−03
1e−02
1e−01
1e+00
1e−02
1e+01
1e+04

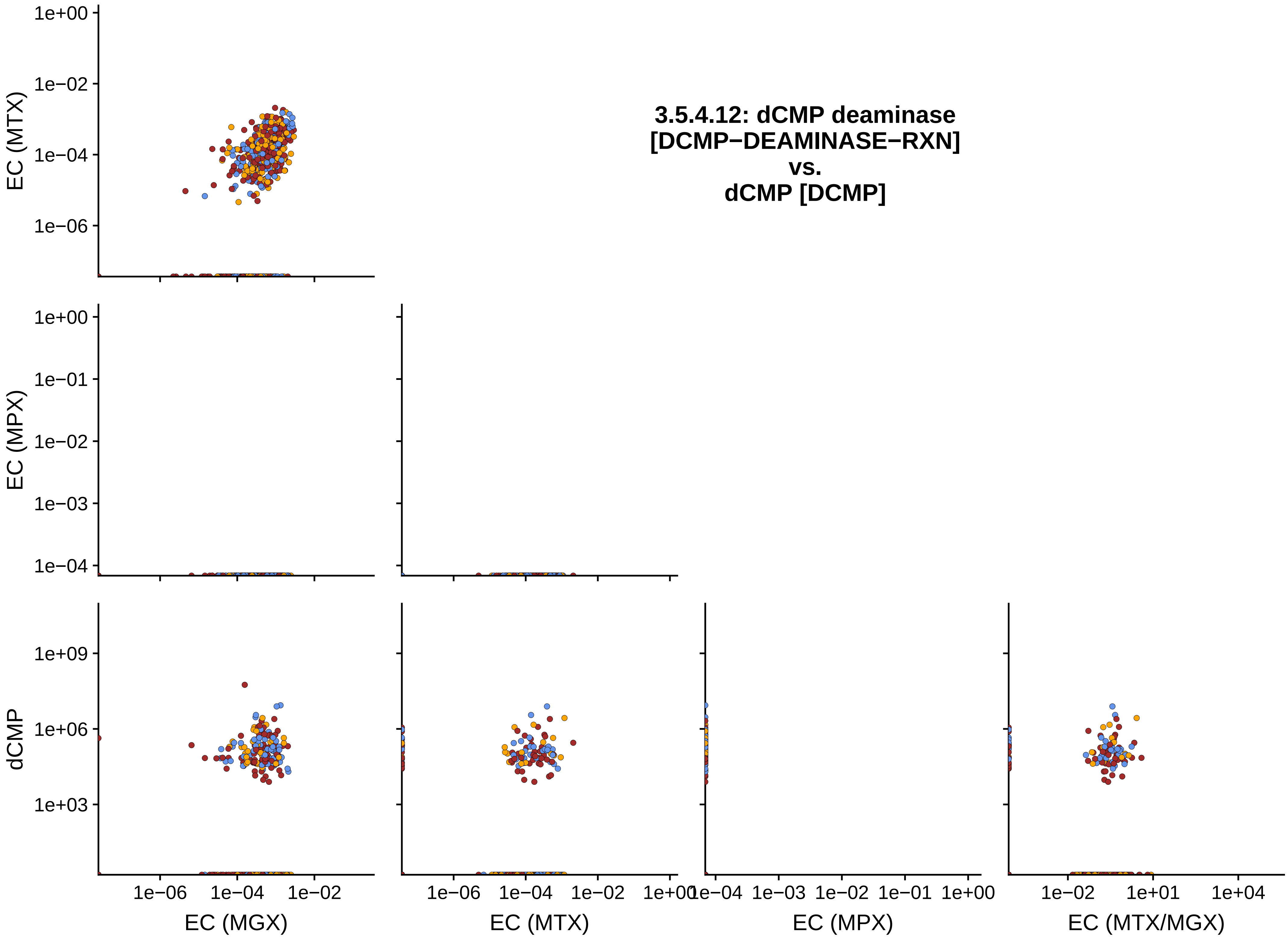

3.5.4.12: dCMP deaminase
[DCMP−DEAMINASE−RXN]
vs.
dCMP [DCMP]
EC (MTX)
1e+00
1e−02
1e−04
1e−06
EC (MPX)
1e+00
1e−01
1e−02
1e−03
1e−04
dCMP
1e+09
1e+06
1e+03
1e−06
1e−04
1e−02
1e+00
1e−04
1e−03
1e−02
1e−01
1e+00
1e−02
1e+01
1e+04
EC (MGX)
EC (MTX)
EC (MPX)
EC (MTX/MGX)

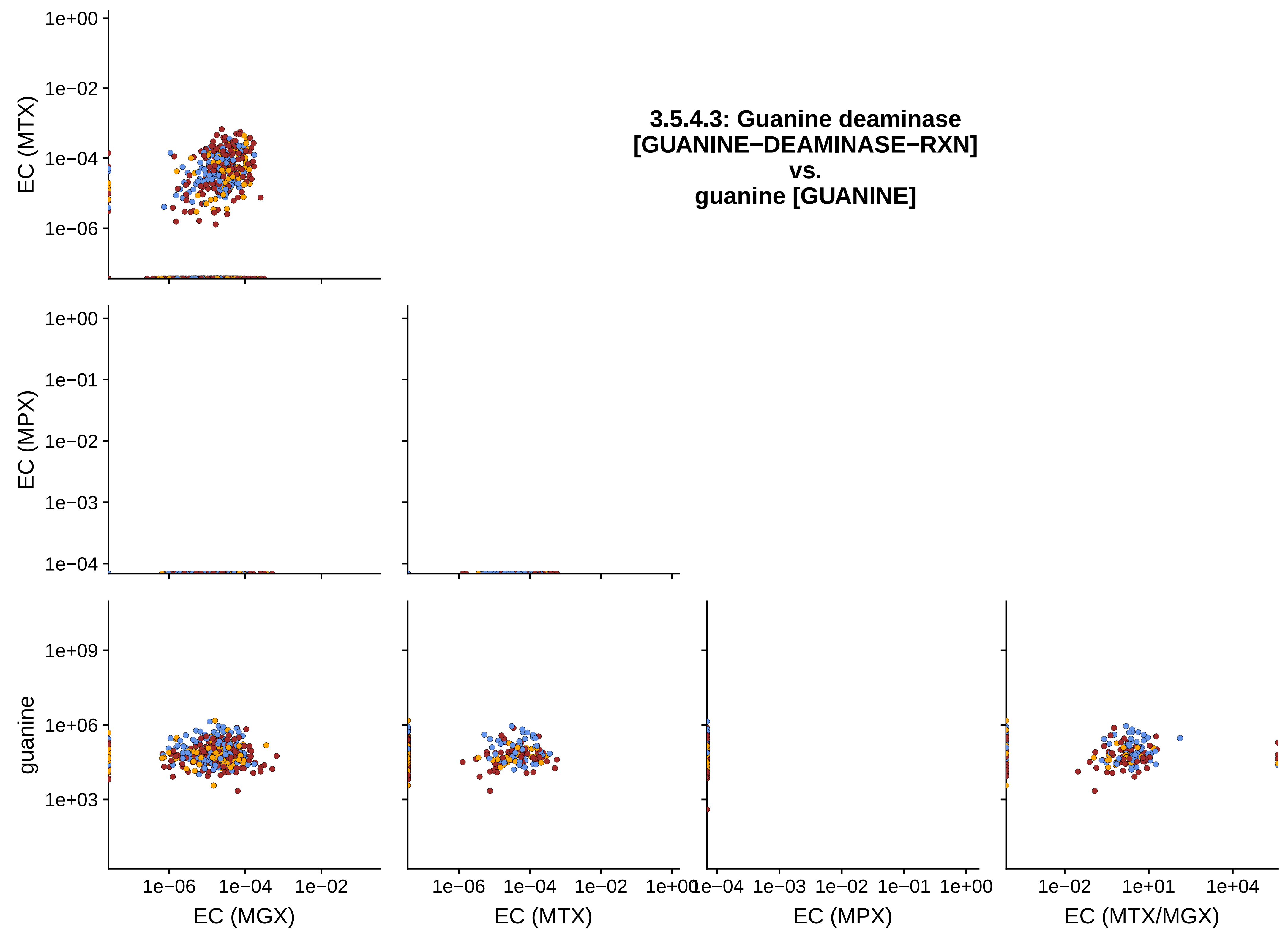

3.5.4.3: Guanine deaminase
[GUANINE−DEAMINASE−RXN]
vs.
guanine [GUANINE]
EC (MTX)
EC (MPX)
guanine
EC (MGX)
EC (MTX)
EC (MPX)
EC (MTX/MGX)
1e+00
1e−02
1e−04
1e−06
1e−01
1e−03
1e+09
1e+06
1e+03
1e+04
1e+01

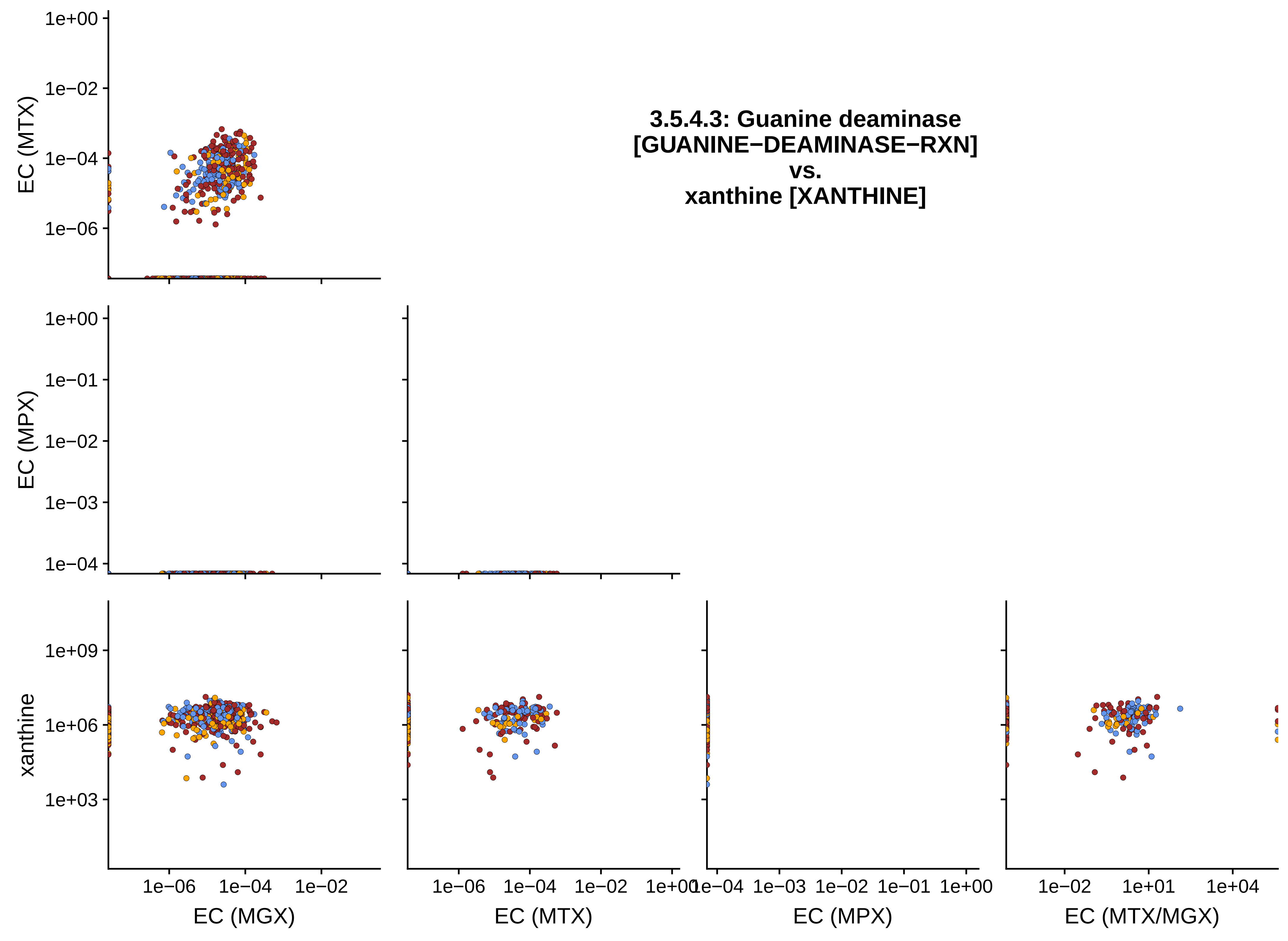

3.5.4.3: Guanine deaminase
[GUANINE−DEAMINASE−RXN]
vs.
xanthine [XANTHINE]
EC (MTX)
EC (MPX)
xanthine
EC (MGX)
EC (MTX)
EC (MPX)
EC (MTX/MGX)
1e+00
1e−02
1e−04
1e−06
1e−01
1e−03
1e+09
1e+06
1e+03
1e−06
1e−04
1e−02
1e−04
1e−03
1e−02
1e−01
1e+00
1e−02
1e+01
1e+04

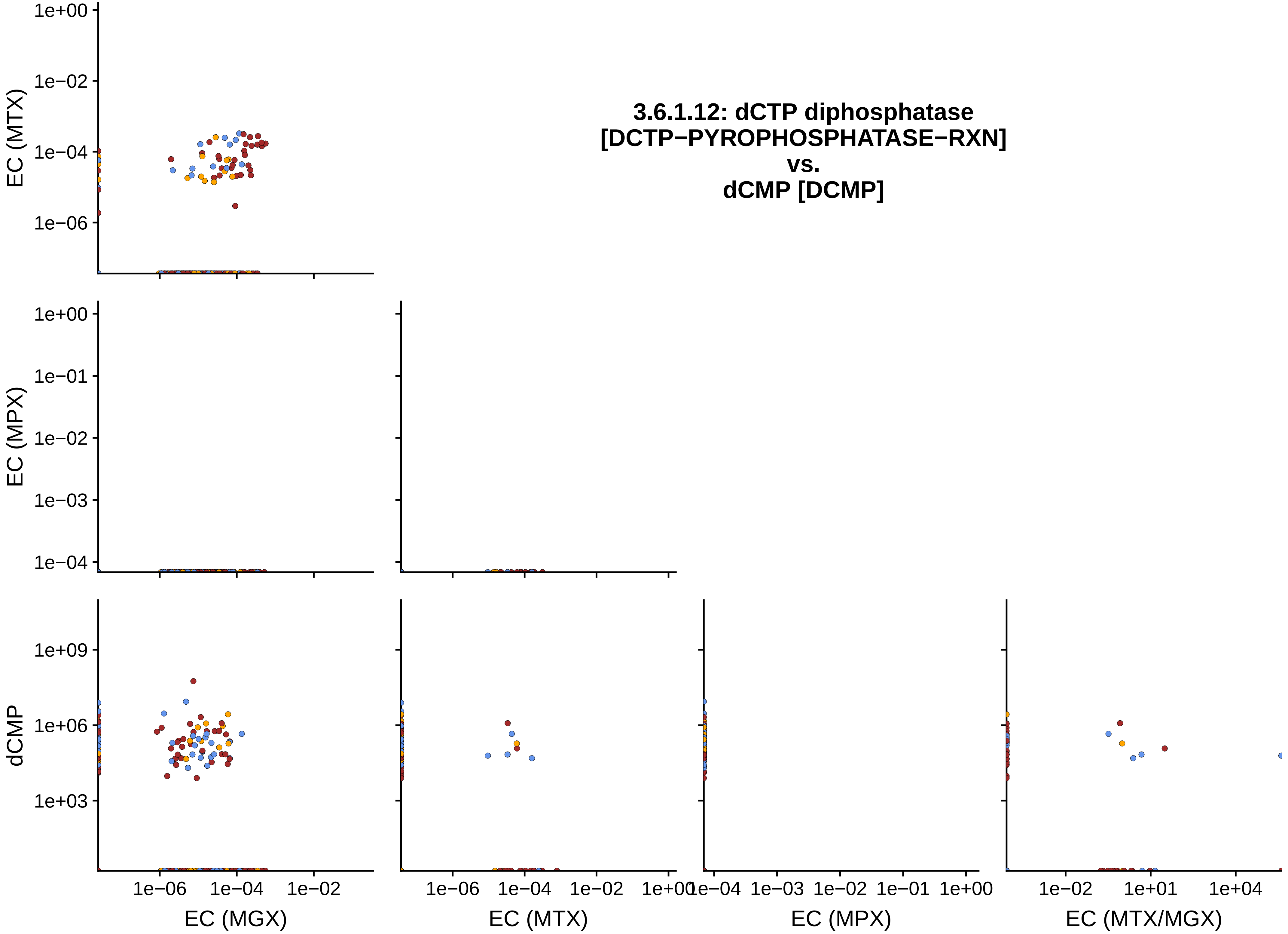

3.6.1.12: dCTP diphosphatase
[DCTP−PYROPHOSPHATASE−RXN]
vs.
dCMP [DCMP]
EC (MTX)
EC (MPX)
dCMP
EC (MGX)
EC (MTX)
EC (MPX)
EC (MTX/MGX)

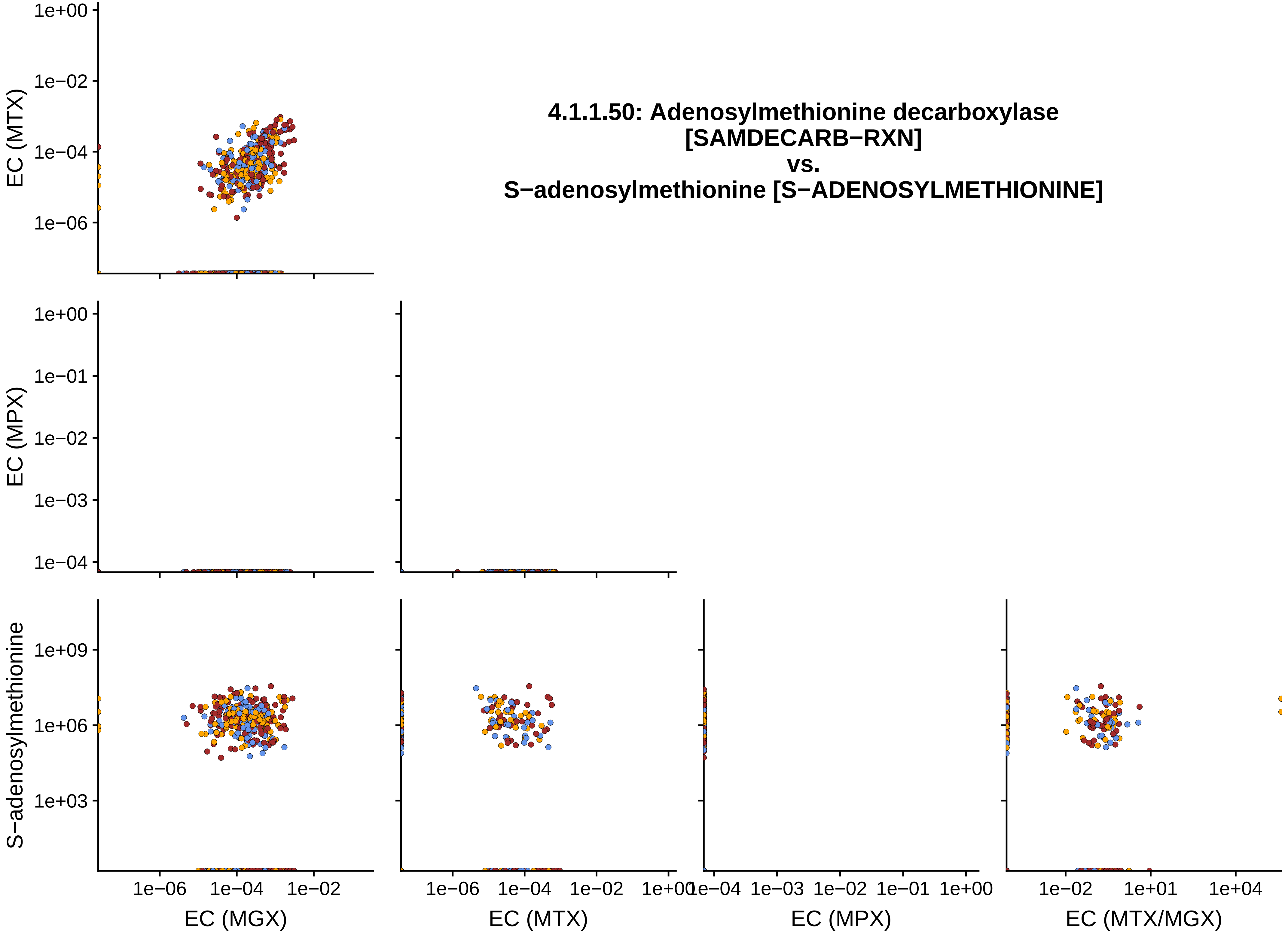
4.1.1.50: Adenosylmethionine decarboxylase
[SAMDECARB−RXN]
vs.
S−adenosylmethionine [S−ADENOSYLMETHIONINE]
EC (MTX)
EC (MPX)
S−adenosylmethionine
EC (MGX)
EC (MTX)
EC (MPX)
EC (MTX/MGX)
1e+00
1e−02
1e−04
1e−06
1e−01
1e−03
1e+09
1e+06
1e+03
1e+01
1e+04

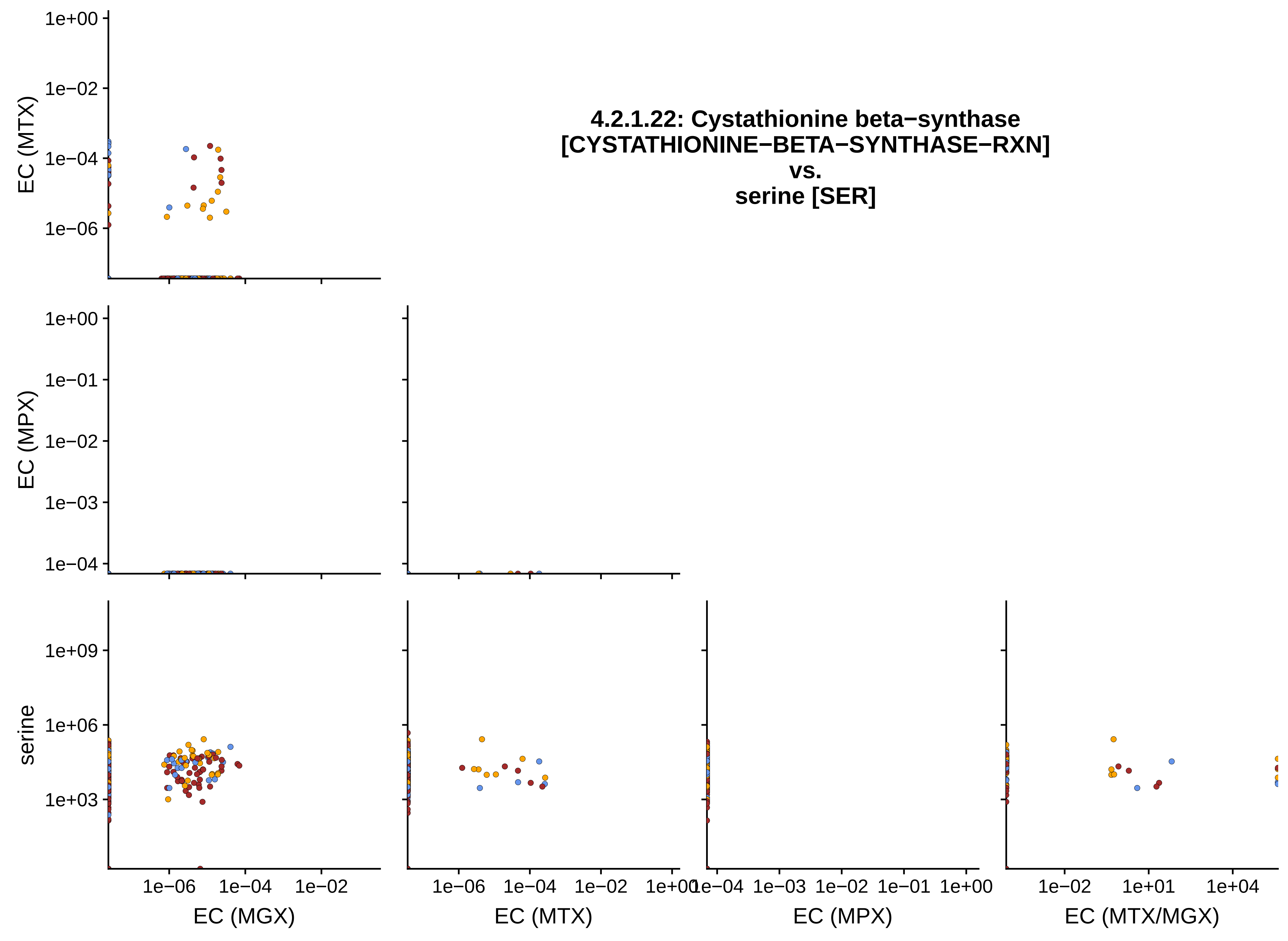

4.2.1.22: Cystathionine beta−synthase
[CYSTATHIONINE−BETA−SYNTHASE−RXN]
vs.
serine [SER]
EC (MTX)
EC (MPX)
serine
EC (MGX)
EC (MTX)
EC (MPX)
EC (MTX/MGX)
1e+00
1e−02
1e−04
1e−06
1e−01
1e−03
1e+09
1e+06
1e+03
1e+01
1e+04

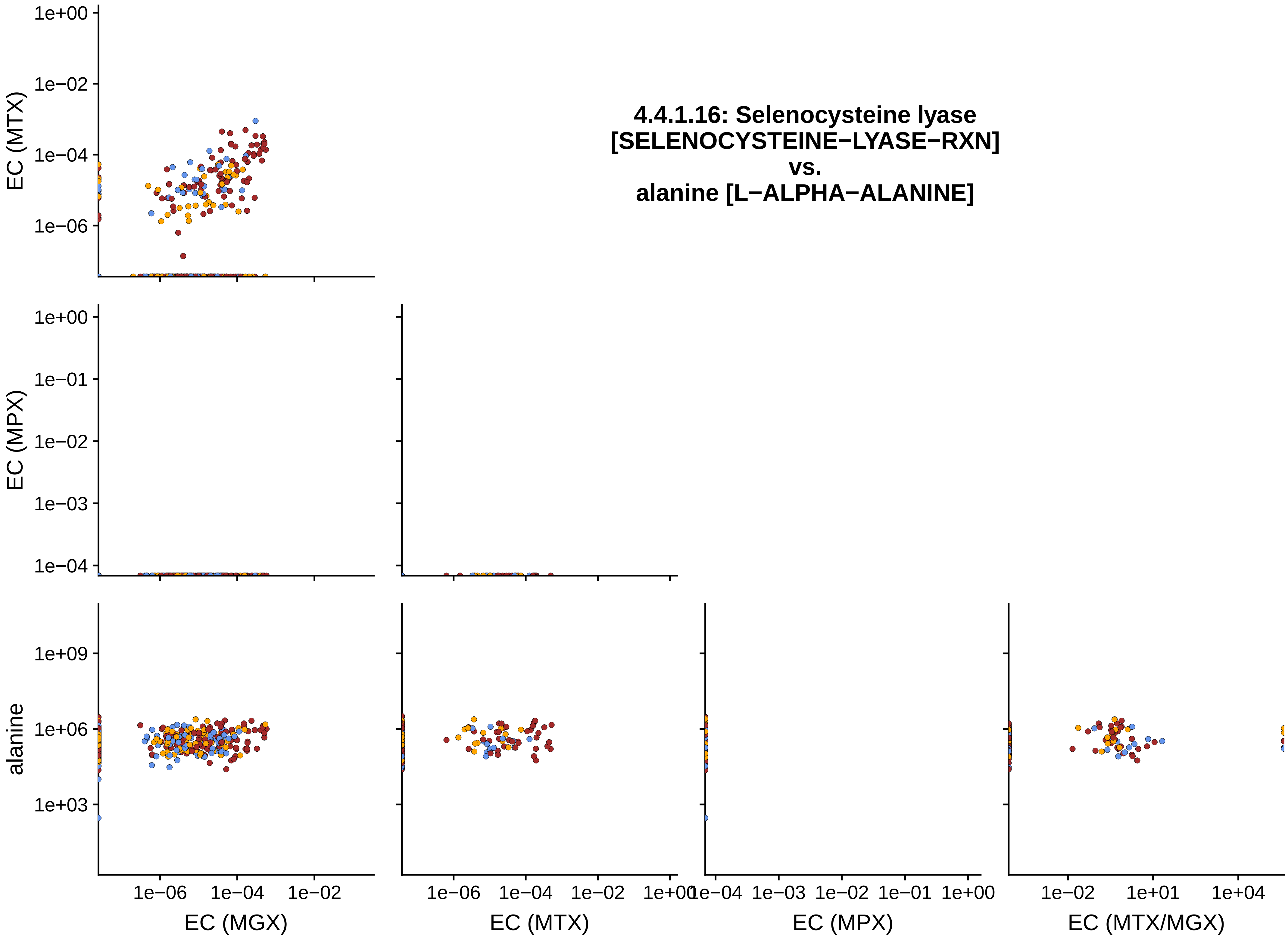
4.4.1.16: Selenocysteine lyase
[SELENOCYSTEINE−LYASE−RXN]
vs.
alanine [L−ALPHA−ALANINE]
EC (MTX)
EC (MPX)
alanine
EC (MGX)
EC (MTX)
EC (MPX)
EC (MTX/MGX)
1e+00
1e−02
1e−04
1e−06
1e−01
1e−03
1e+09
1e+06
1e+03
1e+01
1e+04

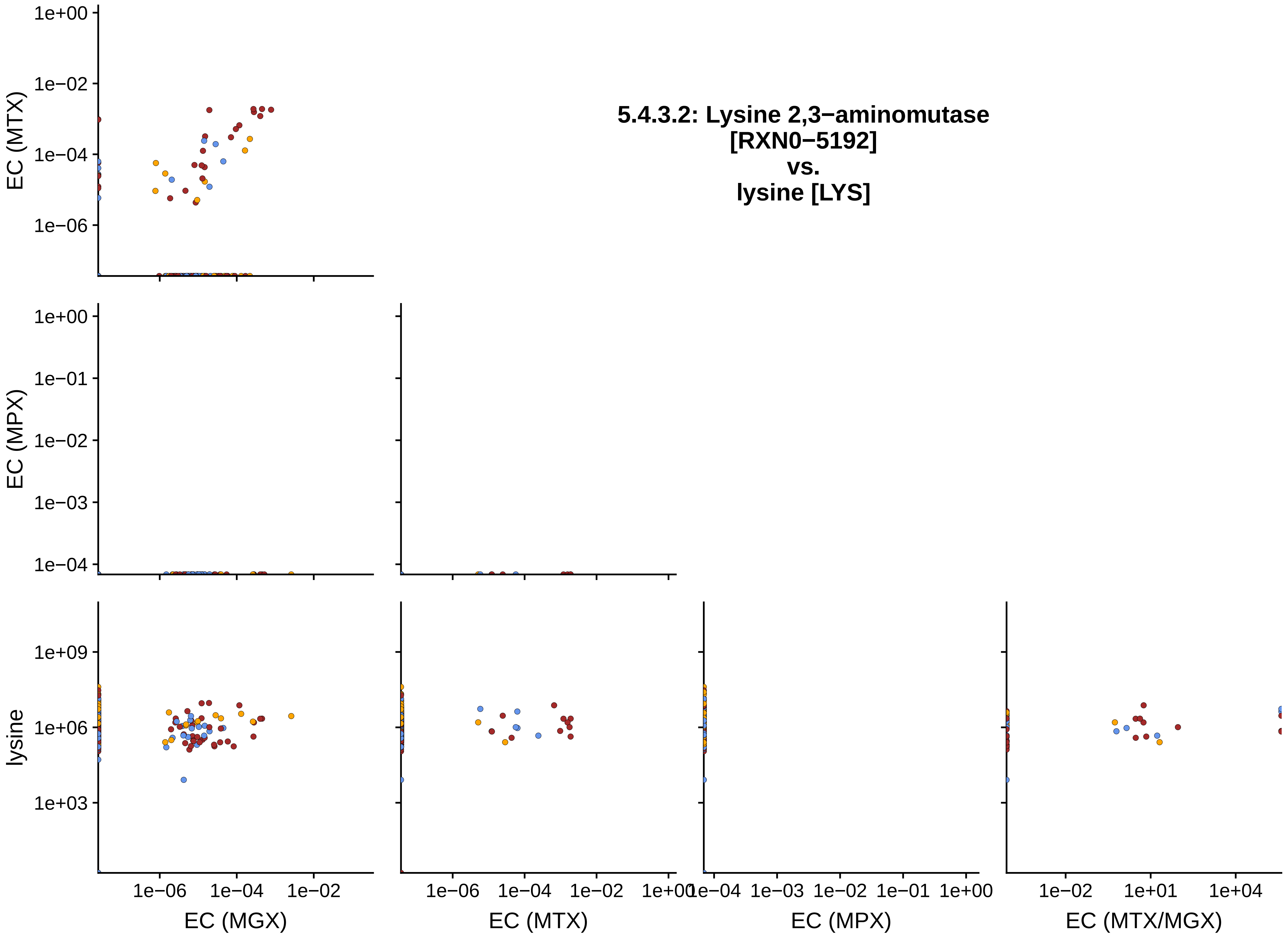
5.4.3.2: Lysine 2,3−aminomutase
[RXN0−5192]
vs.
lysine [LYS]
EC (MTX)
EC (MPX)
lysine
EC (MGX)
EC (MTX)
EC (MPX)
EC (MTX/MGX)
1e+00
1e−02
1e−04
1e−06
1e−01
1e−03
1e+09
1e+06
1e+03
1e+01
1e+04

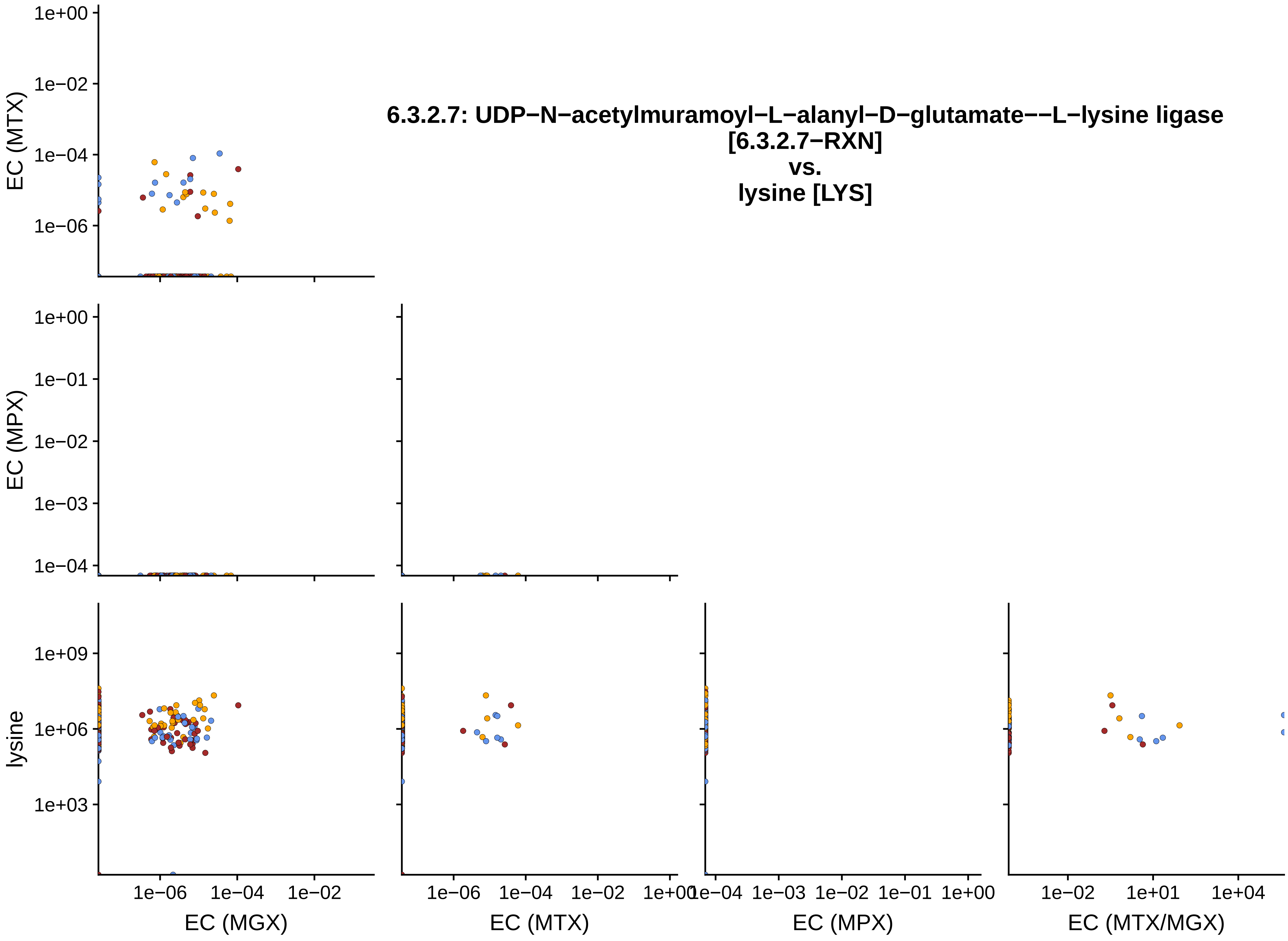

6.3.2.7: UDP−N−acetylmuramoyl−L−alanyl−D−glutamate−−L−lysine ligase
[6.3.2.7−RXN]
vs.
lysine [LYS]
EC (MTX)
EC (MPX)
lysine
EC (MGX)
EC (MTX)
EC (MPX)
EC (MTX/MGX)
1e+00
1e−02
1e−04
1e−06
1e−01
1e−03
1e+09
1e+06
1e+03
1e+01
1e+04
