# Supplementary material for: Multi-omics of the gut microbial ecosystem in inflammatory bowel diseases
Source: Nature. 2019 May 29;569(7758):655–62. doi: 10.1038/s41586-019-1237-9 (PMC6650278; doi:10.1038/s41586-019-1237-9)
Supplement: Supplementary file 4 — Summaries are shown for the 111 subjects for which at least two metagenomes are available. On the left, short-term dietary information collected with each stool sample is shown, along with information on medication use, hospitalization, disease severity scores including calprotectin, HBI/SCCAI, and the dysbiosis score, antibody titers measured from sera, and final reads gathered from metagenomic and metatranscriptomic sequencing (along with the fraction of human reads filtered out during QC). Middle panels provide summaries of the data gathered from microbial measurements, and include profiles for metagenomic and metatranscriptomic taxa, their aerotolerance, viruses, transcribed KOs, metabolites, and proteins. Right panels display the locations of the subject’s samples in the Principal Coordinates Plots from Extended Data Fig. 2. Samples are colored according to their dysbiosis classification (red dysbiotic, blue non-dysbiotic), and connected in sequence by lines. All profiles are available through the IBDMDB at http://ibdmdb.org. [file 41586_2019_1237_MOESM4_ESM.pdf]

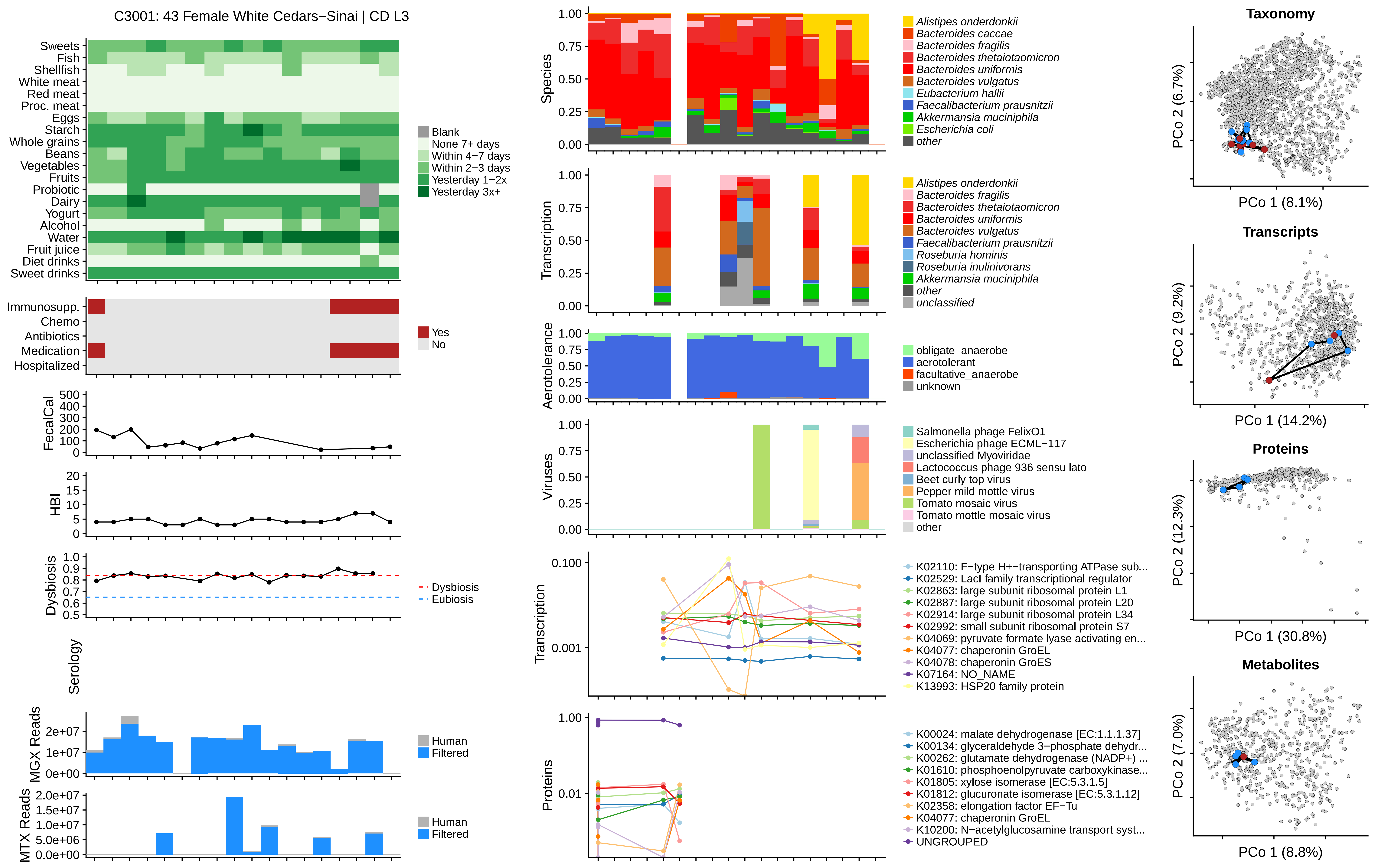
C3001: 43 Female White Cedars−Sinai | CD L3
Sweets
Fish
Shellfish
White meat
Red meat
Proc. meat
Eggs
Starch
Whole grains
Beans
Vegetables
Fruits
Probiotic
Dairy
Yogurt
Alcohol
Water
Fruit juice
Diet drinks
Sweet drinks
Blank
None 7+ days
Within 4−7 days
Within 2−3 days
Yesterday 1−2x
Yesterday 3x+
Immunosupp.
Chemo
Antibiotics
Medication
Hospitalized
Yes
No
FecalCal
HBI
Dysbiosis
Dysbiosis
Eubiosis
Serology
MGX Reads
Human
Filtered
MTX Reads
Human
Filtered
Species
Alistipes onderdonkii
Bacteroides caccae
Bacteroides fragilis
Bacteroides thetaiotaomicron
Bacteroides uniformis
Bacteroides vulgatus
Eubacterium hallii
Faecalibacterium prausnitzii
Akkermansia muciniphila
Escherichia coli
other
Transcription
Alistipes onderdonkii
Bacteroides fragilis
Bacteroides thetaiotaomicron
Bacteroides uniformis
Bacteroides vulgatus
Faecalibacterium prausnitzii
Roseburia hominis
Roseburia inulinivorans
Akkermansia muciniphila
other
unclassified
Aerotolerance
obligate_anaerobe
aerotolerant
facultative_anaerobe
unknown
Viruses
Salmonella phage FelixO1
Escherichia phage ECML−117
unclassified Myoviridae
Lactococcus phage 936 sensu lato
Beet curly top virus
Pepper mild mottle virus
Tomato mosaic virus
Tomato mottle mosaic virus
other
Transcription
K02110: F−type H+−transporting ATPase sub...
K02529: LacI family transcriptional regulator
K02863: large subunit ribosomal protein L1
K02887: large subunit ribosomal protein L20
K02914: large subunit ribosomal protein L34
K02992: small subunit ribosomal protein S7
K04069: pyruvate formate lyase activating en...
K04077: chaperonin GroEL
K04078: chaperonin GroES
K07164: NO_NAME
K13993: HSP20 family protein
Proteins
K00024: malate dehydrogenase [EC:1.1.1.37]
K00134: glyceraldehyde 3−phosphate dehydr...
K00262: glutamate dehydrogenase (NADP+) ...
K01610: phosphoenolpyruvate carboxykinase...
K01805: xylose isomerase [EC:5.3.1.5]
K01812: glucuronate isomerase [EC:5.3.1.12]
K02358: elongation factor EF−Tu
K04077: chaperonin GroEL
K10200: N−acetylglucosamine transport syst...
UNGROUPED
Taxonomy
PCo 1 (8.1%)
PCo 2 (6.7%)
Transcripts
PCo 1 (14.2%)
PCo 2 (9.2%)
Proteins
PCo 1 (30.8%)
PCo 2 (12.3%)
Metabolites
PCo 1 (8.8%)
PCo 2 (7.0%)

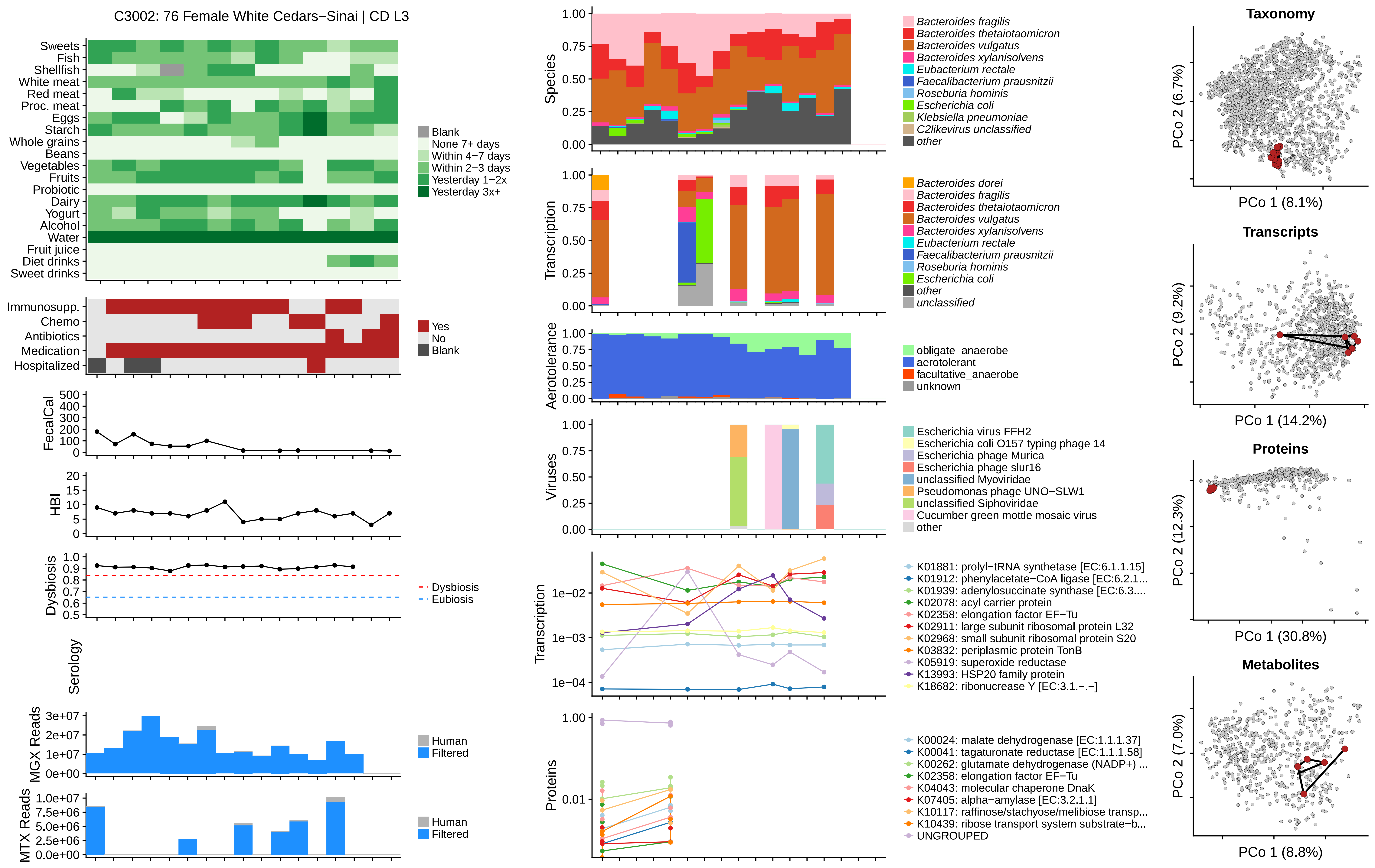
C3002: 76 Female White Cedars−Sinai | CD L3
Sweets
Fish
Shellfish
White meat
Red meat
Proc. meat
Eggs
Starch
Whole grains
Beans
Vegetables
Fruits
Probiotic
Dairy
Yogurt
Alcohol
Water
Fruit juice
Diet drinks
Sweet drinks
Blank
None 7+ days
Within 4−7 days
Within 2−3 days
Yesterday 1−2x
Yesterday 3x+
Immunosupp.
Chemo
Antibiotics
Medication
Hospitalized
Yes
No
Blank
FecalCal
HBI
Dysbiosis
Dysbiosis
Eubiosis
Serology
MGX Reads
Human
Filtered
MTX Reads
Human
Filtered
Species
Bacteroides fragilis
Bacteroides thetaiotaomicron
Bacteroides vulgatus
Bacteroides xylanisolvens
Eubacterium rectale
Faecalibacterium prausnitzii
Roseburia hominis
Escherichia coli
Klebsiella pneumoniae
C2likevirus unclassified
other
Transcription
Bacteroides dorei
Bacteroides fragilis
Bacteroides thetaiotaomicron
Bacteroides vulgatus
Bacteroides xylanisolvens
Eubacterium rectale
Faecalibacterium prausnitzii
Roseburia hominis
Escherichia coli
other
unclassified
Aerotolerance
obligate_anaerobe
aerotolerant
facultative_anaerobe
unknown
Viruses
Escherichia virus FFH2
Escherichia coli O157 typing phage 14
Escherichia phage Murica
Escherichia phage slur16
unclassified Myoviridae
Pseudomonas phage UNO−SLW1
unclassified Siphoviridae
Cucumber green mottle mosaic virus
other
Transcription
K01881: prolyl−tRNA synthetase [EC:6.1.1.15]
K01912: phenylacetate−CoA ligase [EC:6.2.1...
K01939: adenylosuccinate synthase [EC:6.3....
K02078: acyl carrier protein
K02358: elongation factor EF−Tu
K02911: large subunit ribosomal protein L32
K02968: small subunit ribosomal protein S20
K03832: periplasmic protein TonB
K05919: superoxide reductase
K13993: HSP20 family protein
K18682: ribonucrease Y [EC:3.1.−.−]
Proteins
K00024: malate dehydrogenase [EC:1.1.1.37]
K00041: tagaturonate reductase [EC:1.1.1.58]
K00262: glutamate dehydrogenase (NADP+) ...
K02358: elongation factor EF−Tu
K04043: molecular chaperone DnaK
K07405: alpha−amylase [EC:3.2.1.1]
K10117: raffinose/stachyose/melibiose transp...
K10439: ribose transport system substrate−b...
UNGROUPED
Taxonomy
PCo 1 (8.1%)
PCo 2 (6.7%)
Transcripts
PCo 1 (14.2%)
PCo 2 (9.2%)
Proteins
PCo 1 (30.8%)
PCo 2 (12.3%)
Metabolites
PCo 1 (8.8%)
PCo 2 (7.0%)

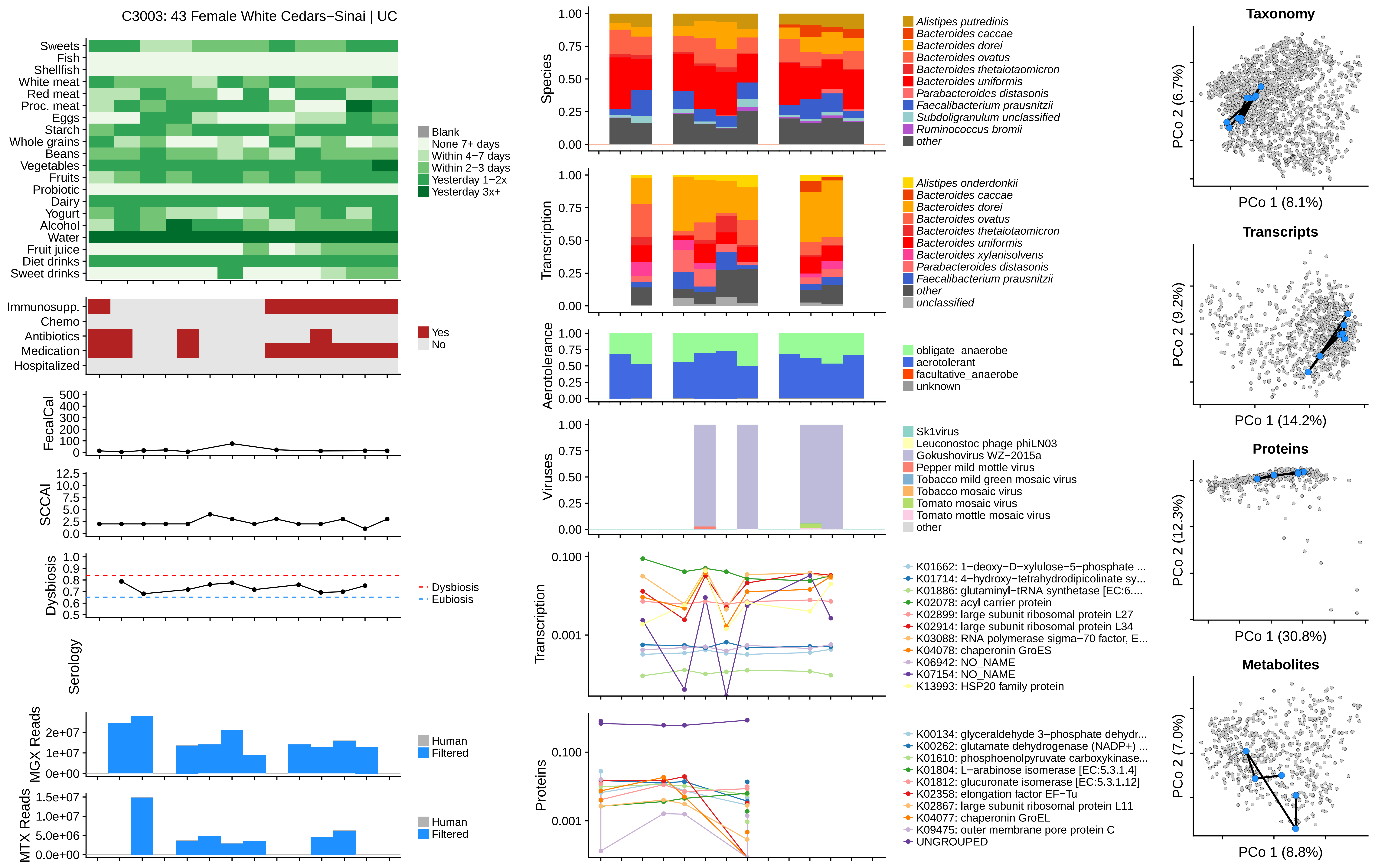

C3003: 43 Female White Cedars−Sinai | UC
Sweets
Fish
Shellfish
White meat
Red meat
Proc. meat
Eggs
Starch
Whole grains
Beans
Vegetables
Fruits
Probiotic
Dairy
Yogurt
Alcohol
Water
Fruit juice
Diet drinks
Sweet drinks
Blank
None 7+ days
Within 4−7 days
Within 2−3 days
Yesterday 1−2x
Yesterday 3x+
Immunosupp.
Chemo
Antibiotics
Medication
Hospitalized
Yes
No
FecalCal
SCCAI
Dysbiosis
Dysbiosis
Eubiosis
Serology
MGX Reads
MTX Reads
Human
Filtered
Human
Filtered
Species
Alistipes putredinis
Bacteroides caccae
Bacteroides dorei
Bacteroides ovatus
Bacteroides thetaiotaomicron
Bacteroides uniformis
Parabacteroides distasonis
Faecalibacterium prausnitzii
Subdoligranulum unclassified
Ruminococcus bromii
other
Transcription
Alistipes onderdonkii
Bacteroides caccae
Bacteroides dorei
Bacteroides ovatus
Bacteroides thetaiotaomicron
Bacteroides uniformis
Bacteroides xylanisolvens
Parabacteroides distasonis
Faecalibacterium prausnitzii
other
unclassified
Aerotolerance
obligate_anaerobe
aerotolerant
facultative_anaerobe
unknown
Viruses
Sk1virus
Leuconostoc phage phiLN03
Gokushovirus WZ−2015a
Pepper mild mottle virus
Tobacco mild green mosaic virus
Tobacco mosaic virus
Tomato mosaic virus
Tomato mottle mosaic virus
other
Transcription
K01662: 1−deoxy−D−xylulose−5−phosphate ...
K01714: 4−hydroxy−tetrahydrodipicolinate sy...
K01886: glutaminyl−tRNA synthetase [EC:6....
K02078: acyl carrier protein
K02899: large subunit ribosomal protein L27
K02914: large subunit ribosomal protein L34
K03088: RNA polymerase sigma−70 factor, E...
K04078: chaperonin GroES
K06942: NO_NAME
K07154: NO_NAME
K13993: HSP20 family protein
Proteins
K00134: glyceraldehyde 3−phosphate dehydr...
K00262: glutamate dehydrogenase (NADP+) ...
K01610: phosphoenolpyruvate carboxykinase...
K01804: L−arabinose isomerase [EC:5.3.1.4]
K01812: glucuronate isomerase [EC:5.3.1.12]
K02358: elongation factor EF−Tu
K02867: large subunit ribosomal protein L11
K04077: chaperonin GroEL
K09475: outer membrane pore protein C
UNGROUPED
Taxonomy
PCo 1 (8.1%)
PCo 2 (6.7%)
Transcripts
PCo 1 (14.2%)
PCo 2 (9.2%)
Proteins
PCo 1 (30.8%)
PCo 2 (12.3%)
Metabolites
PCo 1 (8.8%)
PCo 2 (7.0%)

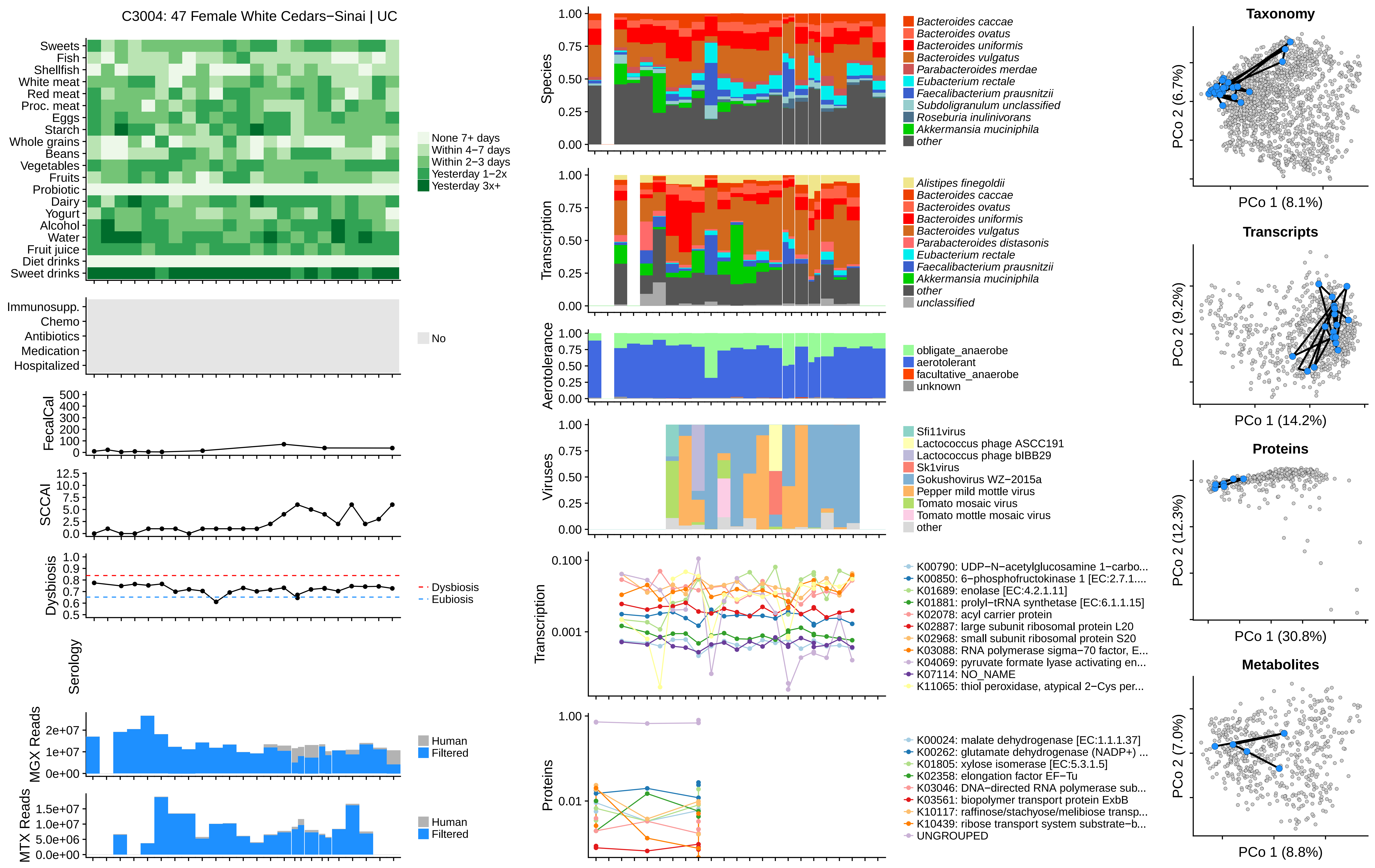

C3004: 47 Female White Cedars−Sinai | UC
Sweets
Fish
Shellfish
White meat
Red meat
Proc. meat
Eggs
Starch
Whole grains
Beans
Vegetables
Fruits
Probiotic
Dairy
Yogurt
Alcohol
Water
Fruit juice
Diet drinks
Sweet drinks
None 7+ days
Within 4−7 days
Within 2−3 days
Yesterday 1−2x
Yesterday 3x+
Immunosupp.
Chemo
Antibiotics
Medication
Hospitalized
No
FecalCal
SCCAI
Dysbiosis
Dysbiosis
Eubiosis
Serology
MGX Reads
Human
Filtered
MTX Reads
Human
Filtered
Species
Bacteroides caccae
Bacteroides ovatus
Bacteroides uniformis
Bacteroides vulgatus
Parabacteroides merdae
Eubacterium rectale
Faecalibacterium prausnitzii
Subdoligranulum unclassified
Roseburia inulinivorans
Akkermansia muciniphila
other
Transcription
Alistipes finegoldii
Bacteroides caccae
Bacteroides ovatus
Bacteroides uniformis
Bacteroides vulgatus
Parabacteroides distasonis
Eubacterium rectale
Faecalibacterium prausnitzii
Akkermansia muciniphila
other
unclassified
Aerotolerance
obligate_anaerobe
aerotolerant
facultative_anaerobe
unknown
Viruses
Sfi11virus
Lactococcus phage ASCC191
Lactococcus phage bIBB29
Sk1virus
Gokushovirus WZ−2015a
Pepper mild mottle virus
Tomato mosaic virus
Tomato mottle mosaic virus
other
Transcription
K00790: UDP−N−acetylglucosamine 1−carbo...
K00850: 6−phosphofructokinase 1 [EC:2.7.1....
K01689: enolase [EC:4.2.1.11]
K01881: prolyl−tRNA synthetase [EC:6.1.1.15]
K02078: acyl carrier protein
K02887: large subunit ribosomal protein L20
K02968: small subunit ribosomal protein S20
K03088: RNA polymerase sigma−70 factor, E...
K04069: pyruvate formate lyase activating en...
K07114: NO_NAME
K11065: thiol peroxidase, atypical 2−Cys per...
Proteins
K00024: malate dehydrogenase [EC:1.1.1.37]
K00262: glutamate dehydrogenase (NADP+) ...
K01805: xylose isomerase [EC:5.3.1.5]
K02358: elongation factor EF−Tu
K03046: DNA−directed RNA polymerase sub...
K03561: biopolymer transport protein ExbB
K10117: raffinose/stachyose/melibiose transp...
K10439: ribose transport system substrate−b...
UNGROUPED
Taxonomy
PCo 1 (8.1%)
PCo 2 (6.7%)
Transcripts
PCo 1 (14.2%)
PCo 2 (9.2%)
Proteins
PCo 1 (30.8%)
PCo 2 (12.3%)
Metabolites
PCo 1 (8.8%)
PCo 2 (7.0%)

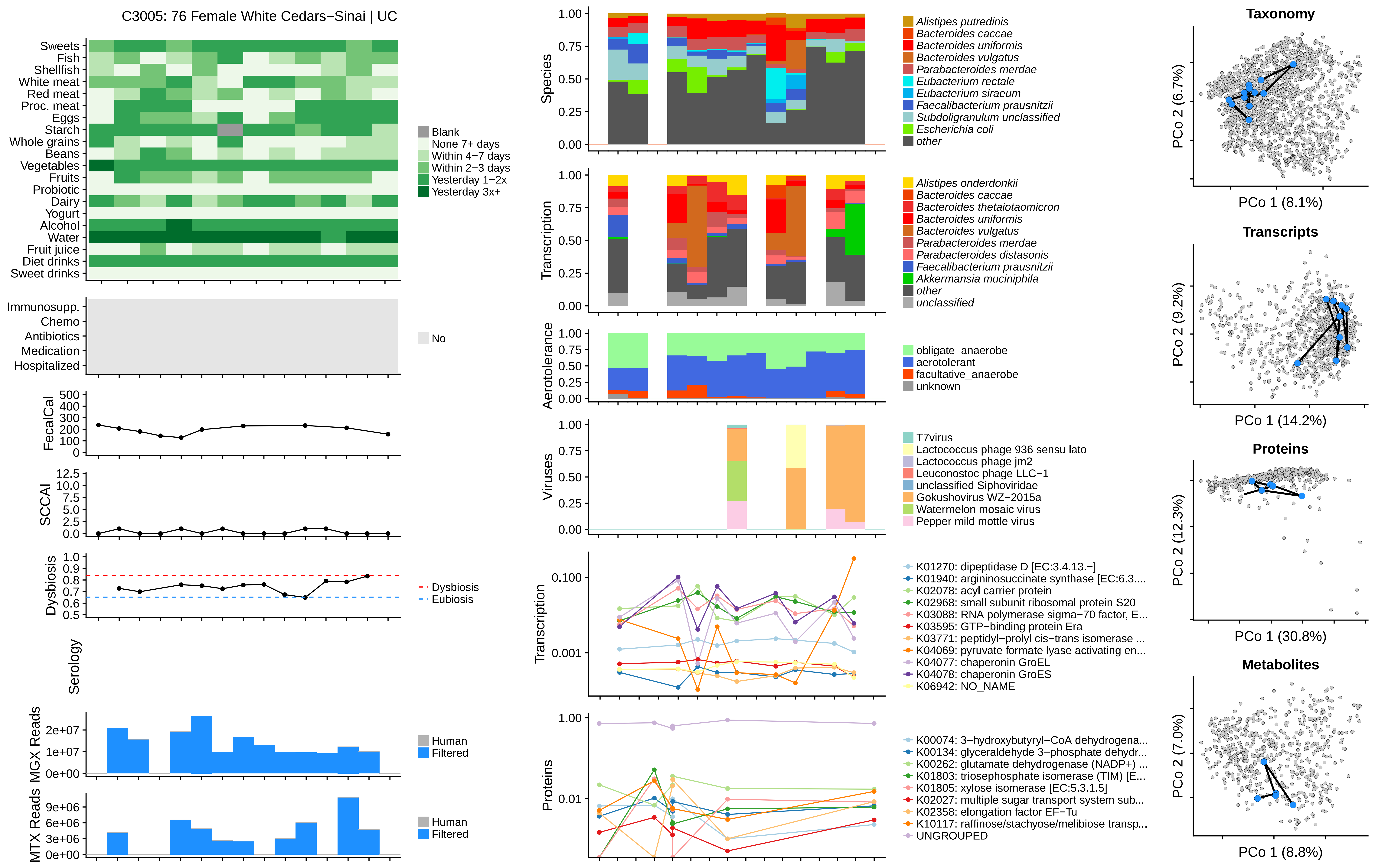

C3005: 76 Female White Cedars−Sinai | UC
Sweets
Fish
Shellfish
White meat
Red meat
Proc. meat
Eggs
Starch
Whole grains
Beans
Vegetables
Fruits
Probiotic
Dairy
Yogurt
Alcohol
Water
Fruit juice
Diet drinks
Sweet drinks
Blank
None 7+ days
Within 4−7 days
Within 2−3 days
Yesterday 1−2x
Yesterday 3x+
Immunosupp.
Chemo
Antibiotics
Medication
Hospitalized
No
FecalCal
SCCAI
Dysbiosis
Dysbiosis
Eubiosis
Serology
MGX Reads
MTX Reads
Human
Filtered
Species
Alistipes putredinis
Bacteroides caccae
Bacteroides uniformis
Bacteroides vulgatus
Parabacteroides merdae
Eubacterium rectale
Eubacterium siraeum
Faecalibacterium prausnitzii
Subdoligranulum unclassified
Escherichia coli
other
Transcription
Alistipes onderdonkii
Bacteroides caccae
Bacteroides thetaiotaomicron
Bacteroides uniformis
Bacteroides vulgatus
Parabacteroides merdae
Parabacteroides distasonis
Faecalibacterium prausnitzii
Akkermansia muciniphila
other
unclassified
Aerotolerance
obligate_anaerobe
aerotolerant
facultative_anaerobe
unknown
Viruses
T7virus
Lactococcus phage 936 sensu lato
Lactococcus phage jm2
Leuconostoc phage LLC−1
unclassified Siphoviridae
Gokushovirus WZ−2015a
Watermelon mosaic virus
Pepper mild mottle virus
Transcription
K01270: dipeptidase D [EC:3.4.13.−]
K01940: argininosuccinate synthase [EC:6.3....
K02078: acyl carrier protein
K02968: small subunit ribosomal protein S20
K03088: RNA polymerase sigma−70 factor, E...
K03595: GTP−binding protein Era
K03771: peptidyl−prolyl cis−trans isomerase ...
K04069: pyruvate formate lyase activating en...
K04077: chaperonin GroEL
K04078: chaperonin GroES
K06942: NO_NAME
Proteins
K00074: 3−hydroxybutyryl−CoA dehydrogena...
K00134: glyceraldehyde 3−phosphate dehydr...
K00262: glutamate dehydrogenase (NADP+) ...
K01803: triosephosphate isomerase (TIM) [E...
K01805: xylose isomerase [EC:5.3.1.5]
K02027: multiple sugar transport system sub...
K02358: elongation factor EF−Tu
K10117: raffinose/stachyose/melibiose transp...
UNGROUPED
Taxonomy
PCo 1 (8.1%)
PCo 2 (6.7%)
Transcripts
PCo 1 (14.2%)
PCo 2 (9.2%)
Proteins
PCo 1 (30.8%)
PCo 2 (12.3%)
Metabolites
PCo 1 (8.8%)
PCo 2 (7.0%)

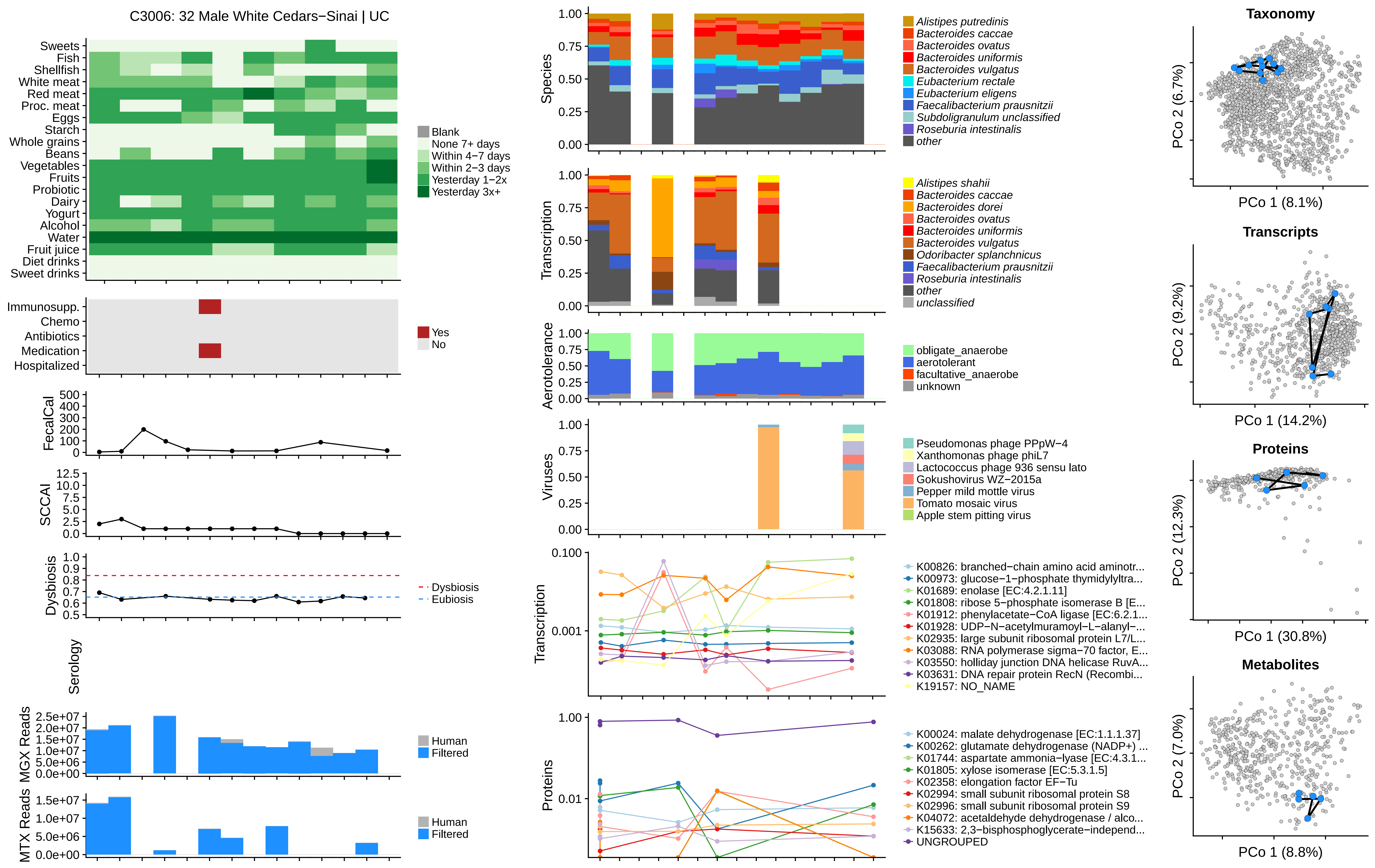

C3006: 32 Male White Cedars−Sinai | UC
Sweets
Fish
Shellfish
White meat
Red meat
Proc. meat
Eggs
Starch
Whole grains
Beans
Vegetables
Fruits
Probiotic
Dairy
Yogurt
Alcohol
Water
Fruit juice
Diet drinks
Sweet drinks
Blank
None 7+ days
Within 4−7 days
Within 2−3 days
Yesterday 1−2x
Yesterday 3x+
Immunosupp.
Chemo
Antibiotics
Medication
Hospitalized
Yes
No
FecalCal
SCCAI
Dysbiosis
Dysbiosis
Eubiosis
Serology
MGX Reads
MTX Reads
Human
Filtered
Species
Alistipes putredinis
Bacteroides caccae
Bacteroides ovatus
Bacteroides uniformis
Bacteroides vulgatus
Eubacterium rectale
Eubacterium eligens
Faecalibacterium prausnitzii
Subdoligranulum unclassified
Roseburia intestinalis
other
Transcription
Alistipes shahii
Bacteroides caccae
Bacteroides dorei
Bacteroides ovatus
Bacteroides uniformis
Bacteroides vulgatus
Odoribacter splanchnicus
Faecalibacterium prausnitzii
Roseburia intestinalis
other
unclassified
Aerotolerance
obligate_anaerobe
aerotolerant
facultative_anaerobe
unknown
Viruses
Pseudomonas phage PPpW−4
Xanthomonas phage philL7
Lactococcus phage 936 sensu lato
Gokushovirus WZ−2015a
Pepper mild mottle virus
Tomato mosaic virus
Apple stem pitting virus
Transcription
K00826: branched−chain amino acid aminotr...
K00973: glucose−1−phosphate thymidylyltra...
K01689: enolase [EC:4.2.1.11]
K01808: ribose 5−phosphate isomerase B [E...
K01912: phenylacetate−CoA ligase [EC:6.2.1...
K01928: UDP−N−acetylmuramoyl−L−alanyl−...
K02935: large subunit ribosomal protein L7/L...
K03088: RNA polymerase sigma−70 factor, E...
K03550: holliday junction DNA helicase RuvA...
K03631: DNA repair protein RecN (Recombi...
K19157: NO_NAME
Proteins
K00024: malate dehydrogenase [EC:1.1.1.37]
K00262: glutamate dehydrogenase (NADP+) ...
K01744: aspartate ammonia−lyase [EC:4.3.1...
K01805: xylose isomerase [EC:5.3.1.5]
K02358: elongation factor EF−Tu
K02994: small subunit ribosomal protein S8
K02996: small subunit ribosomal protein S9
K04072: acetaldehyde dehydrogenase / alco...
K15633: 2,3−bisphosphoglycerate−independ...
UNGROUPED
Taxonomy
PCo 1 (8.1%)
PCo 2 (6.7%)
Transcripts
PCo 1 (14.2%)
PCo 2 (9.2%)
Proteins
PCo 1 (30.8%)
PCo 2 (12.3%)
Metabolites
PCo 1 (8.8%)
PCo 2 (7.0%)

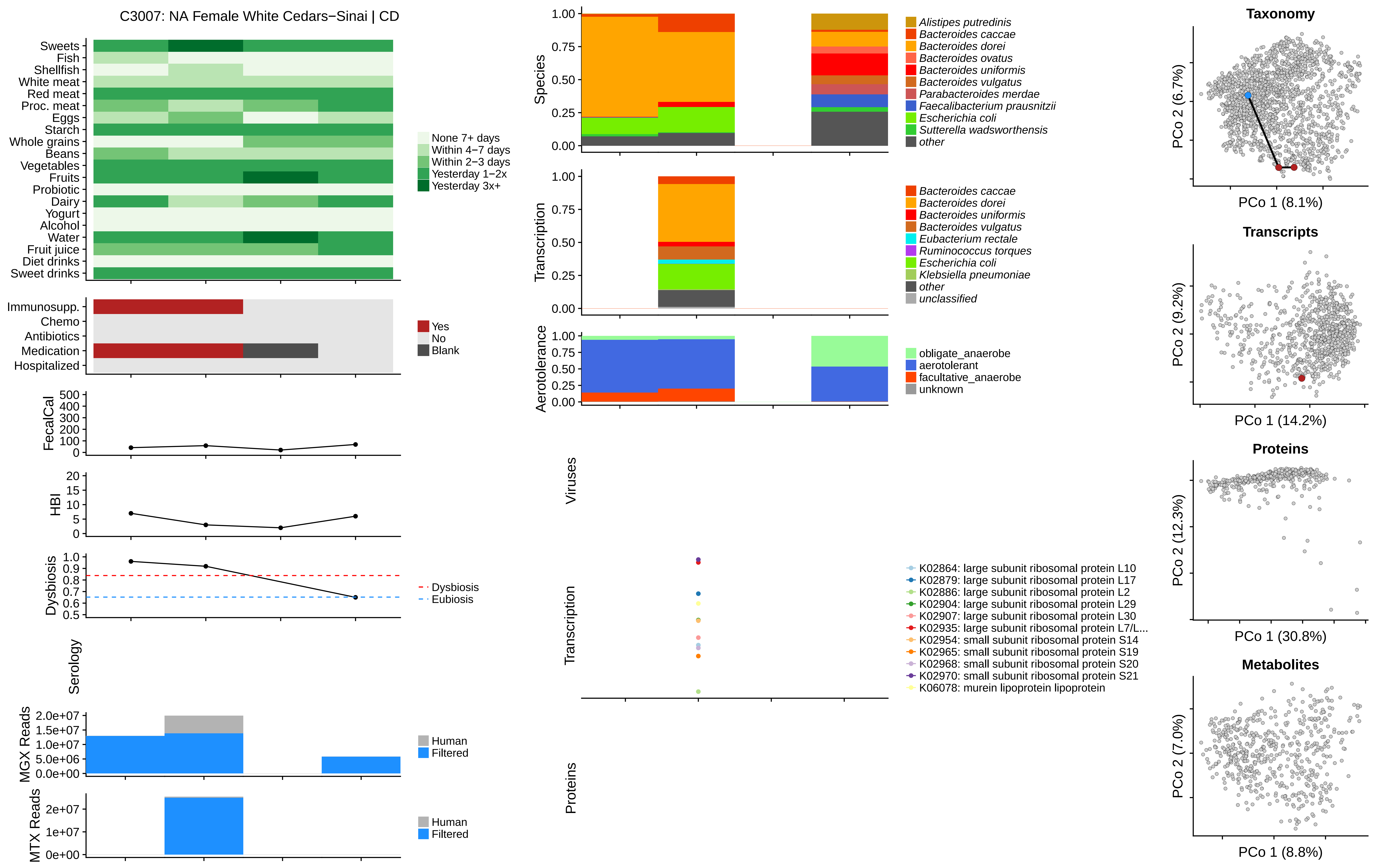

C3007: NA Female White Cedars−Sinai | CD
Sweets
Fish
Shellfish
White meat
Red meat
Proc. meat
Eggs
Starch
Whole grains
Beans
Vegetables
Fruits
Probiotic
Dairy
Yogurt
Alcohol
Water
Fruit juice
Diet drinks
Sweet drinks
None 7+ days
Within 4−7 days
Within 2−3 days
Yesterday 1−2x
Yesterday 3x+
Immunosupp.
Chemo
Antibiotics
Medication
Hospitalized
Yes
No
Blank
FecalCal
HBI
Dysbiosis
Dysbiosis
Eubiosis
Serology
MGX Reads
Human
Filtered
MTX Reads
Human
Filtered
Species
Alistipes putredinis
Bacteroides caccae
Bacteroides dorei
Bacteroides ovatus
Bacteroides uniformis
Bacteroides vulgatus
Parabacteroides merdae
Faecalibacterium prausnitzii
Escherichia coli
Sutterella wadsworthensis
other
Transcription
Bacteroides caccae
Bacteroides dorei
Bacteroides uniformis
Bacteroides vulgatus
Eubacterium rectale
Ruminococcus torques
Escherichia coli
Klebsiella pneumoniae
other
unclassified
Aerotolerance
obligate_anaerobe
aerotolerant
facultative_anaerobe
unknown
Viruses
Transcription
K02864: large subunit ribosomal protein L10
K02879: large subunit ribosomal protein L17
K02886: large subunit ribosomal protein L2
K02904: large subunit ribosomal protein L29
K02907: large subunit ribosomal protein L30
K02935: large subunit ribosomal protein L7/L...
K02954: small subunit ribosomal protein S14
K02965: small subunit ribosomal protein S19
K02968: small subunit ribosomal protein S20
K02970: small subunit ribosomal protein S21
K06078: murein lipoprotein lipoprotein
Proteins
Taxonomy
PCo 1 (8.1%)
PCo 2 (6.7%)
Transcripts
PCo 1 (14.2%)
PCo 2 (9.2%)
Proteins
PCo 1 (30.8%)
PCo 2 (12.3%)
Metabolites
PCo 1 (8.8%)
PCo 2 (7.0%)

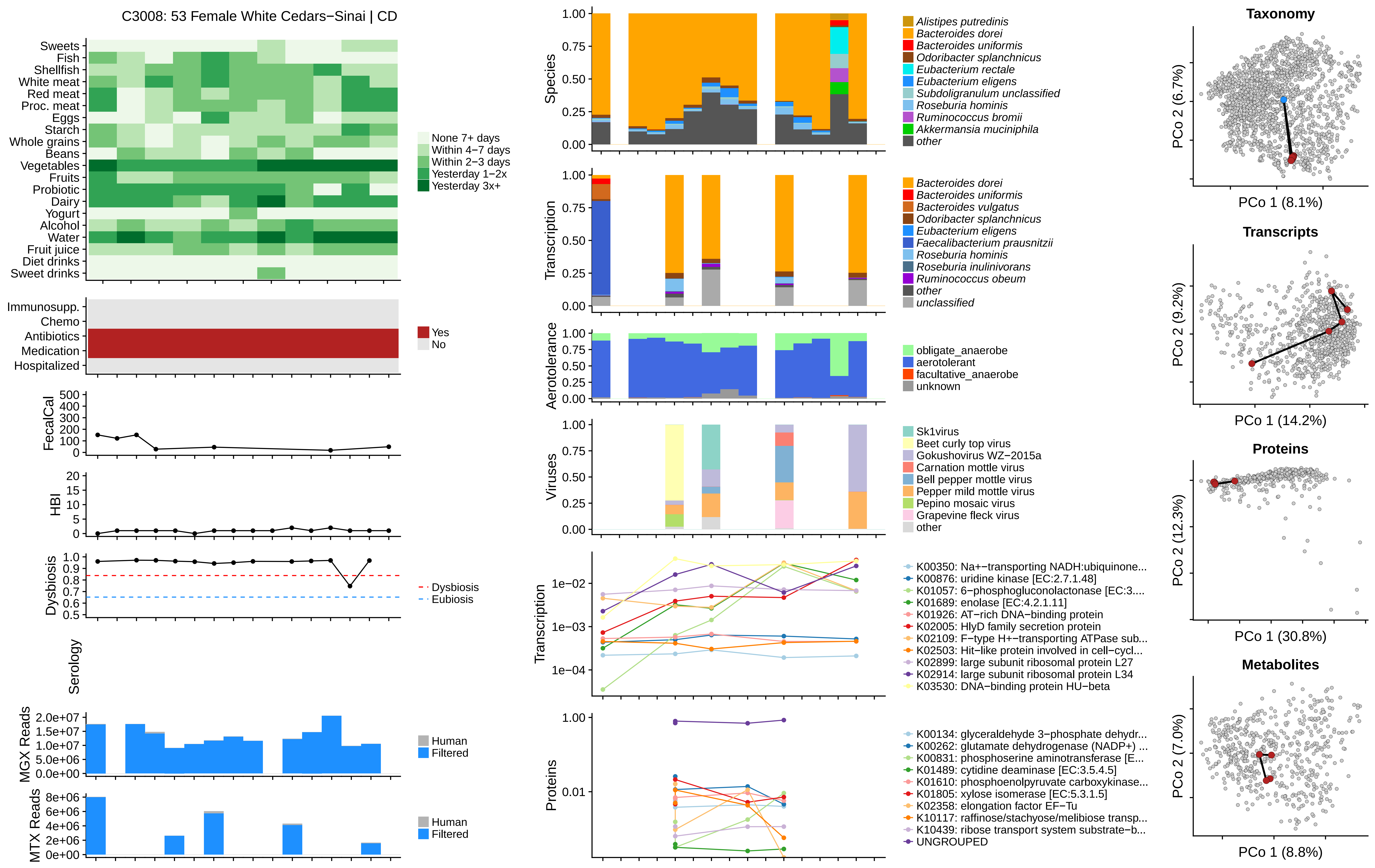

C3008: 53 Female White Cedars−Sinai | CD
Sweets
Fish
Shellfish
White meat
Red meat
Proc. meat
Eggs
Starch
Whole grains
Beans
Vegetables
Fruits
Probiotic
Dairy
Yogurt
Alcohol
Water
Fruit juice
Diet drinks
Sweet drinks
None 7+ days
Within 4−7 days
Within 2−3 days
Yesterday 1−2x
Yesterday 3x+
Immunosupp.
Chemo
Antibiotics
Medication
Hospitalized
Yes
No
FecalCal
HBI
Dysbiosis
Dysbiosis
Eubiosis
Serology
MGX Reads
Human
Filtered
MTX Reads
Human
Filtered
Species
Alistipes putredinis
Bacteroides dorei
Bacteroides uniformis
Odoribacter splanchnicus
Eubacterium rectale
Eubacterium eligens
Subdoligranulum unclassified
Roseburia hominis
Ruminococcus bromii
Akkermansia muciniphila
other
Transcription
Bacteroides dorei
Bacteroides uniformis
Bacteroides vulgatus
Odoribacter splanchnicus
Eubacterium eligens
Faecalibacterium prausnitzii
Roseburia hominis
Roseburia inulinivorans
Ruminococcus obeum
other
unclassified
Aerotolerance
obligate_anaerobe
aerotolerant
facultative_anaerobe
unknown
Viruses
Sk1virus
Beet curly top virus
Gokushovirus WZ−2015a
Carnation mottle virus
Bell pepper mottle virus
Pepper mild mottle virus
Pepino mosaic virus
Grapevine fleck virus
other
Transcription
K00350: Na+−transporting NADH:ubiquinone...
K00876: uridine kinase [EC:2.7.1.48]
K01057: 6−phosphogluconolactonase [EC:3....
K01689: enolase [EC:4.2.1.11]
K01926: AT−rich DNA−binding protein
K02005: HlyD family secretion protein
K02109: F−type H+−transporting ATPase sub...
K02503: Hit−like protein involved in cell−cycl...
K02899: large subunit ribosomal protein L27
K02914: large subunit ribosomal protein L34
K03530: DNA−binding protein HU−beta
Proteins
K00134: glyceraldehyde 3−phosphate dehydr...
K00262: glutamate dehydrogenase (NADP+) ...
K00831: phosphoserine aminotransferase [E...
K01489: cytidine deaminase [EC:3.5.4.5]
K01610: phosphoenolpyruvate carboxykinase...
K01805: xylose isomerase [EC:5.3.1.5]
K02358: elongation factor EF−Tu
K10117: raffinose/stachyose/melibiose transp...
K10439: ribose transport system substrate−b...
UNGROUPED
Taxonomy
PCo 1 (8.1%)
PCo 2 (6.7%)
Transcripts
PCo 1 (14.2%)
PCo 2 (9.2%)
Proteins
PCo 1 (30.8%)
PCo 2 (12.3%)
Metabolites
PCo 1 (8.8%)
PCo 2 (7.0%)

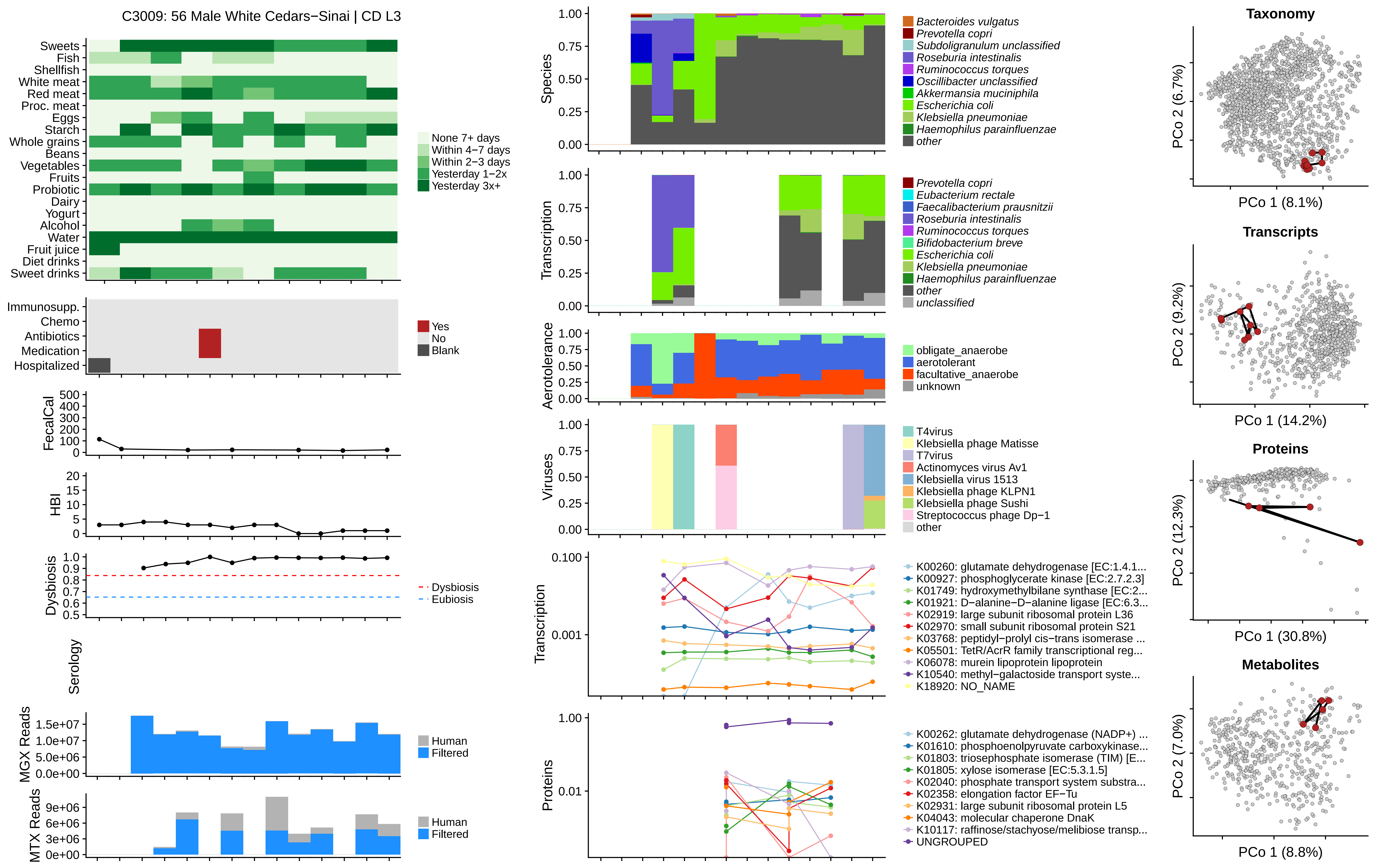
C3009: 56 Male White Cedars−Sinai | CD L3
Sweets
Fish
Shellfish
White meat
Red meat
Proc. meat
Eggs
Starch
Whole grains
Beans
Vegetables
Fruits
Probiotic
Dairy
Yogurt
Alcohol
Water
Fruit juice
Diet drinks
Sweet drinks
None 7+ days
Within 4−7 days
Within 2−3 days
Yesterday 1−2x
Yesterday 3x+
Immunosupp.
Chemo
Antibiotics
Medication
Hospitalized
Yes
No
Blank
FecalCal
500
400
300
200
100
0
HBI
20
15
10
5
0
Dysbiosis
1.0
0.9
0.8
0.7
0.6
0.5
Dysbiosis
Eubiosis
Serology
MGX Reads
1.5e+07
1.0e+07
5.0e+06
0.0e+00
Human
Filtered
MTX Reads
9e+06
6e+06
3e+06
0e+00
Human
Filtered
Species
1.00
0.75
0.50
0.25
0.00
Bacteroides vulgatus
Prevotella copri
Subdoligranulum unclassified
Roseburia intestinalis
Ruminococcus torques
Oscillibacter unclassified
Akkermansia muciniphila
Escherichia coli
Klebsiella pneumoniae
Haemophilus parainfluenzae
other
Transcription
Prevotella copri
Eubacterium rectale
Faecalibacterium prausnitzii
Roseburia intestinalis
Ruminococcus torques
Bifidobacterium breve
Escherichia coli
Klebsiella pneumoniae
Haemophilus parainfluenzae
other
unclassified
Aerotolerance
obligate_anaerobe
aerotolerant
facultative_anaerobe
unknown
Viruses
T4virus
Klebsiella phage Matisse
T7virus
Actinomyces virus Av1
Klebsiella virus 1513
Klebsiella phage KLPN1
Klebsiella phage Sushi
Streptococcus phage Dp−1
other
Transcription
0.100
0.001
K00260: glutamate dehydrogenase [EC:1.4.1...
K00927: phosphoglycerate kinase [EC:2.7.2.3]
K01749: hydroxymethylbilane synthase [EC:2...
K01921: D−alanine−D−alanine ligase [EC:6.3...
K02919: large subunit ribosomal protein L36
K02970: small subunit ribosomal protein S21
K03768: peptidyl−prolyl cis−trans isomerase ...
K05501: TetR/AcrR family transcriptional reg...
K06078: murein lipoprotein lipoprotein
K10540: methyl−galactoside transport syste...
K18920: NO_NAME
Proteins
1.00
0.01
K00262: glutamate dehydrogenase (NADP+) ...
K01610: phosphoenolpyruvate carboxykinase...
K01803: triosephosphate isomerase (TIM) [E...
K01805: xylose isomerase [EC:5.3.1.5]
K02040: phosphate transport system substra...
K02358: elongation factor EF−Tu
K02931: large subunit ribosomal protein L5
K04043: molecular chaperone DnaK
K10117: raffinose/stachyose/melibiose transp...
UNGROUPED
Taxonomy
PCo 1 (8.1%)
PCo 2 (6.7%)
Transcripts
PCo 1 (14.2%)
PCo 2 (9.2%)
Proteins
PCo 1 (30.8%)
PCo 2 (12.3%)
Metabolites
PCo 1 (8.8%)
PCo 2 (7.0%)

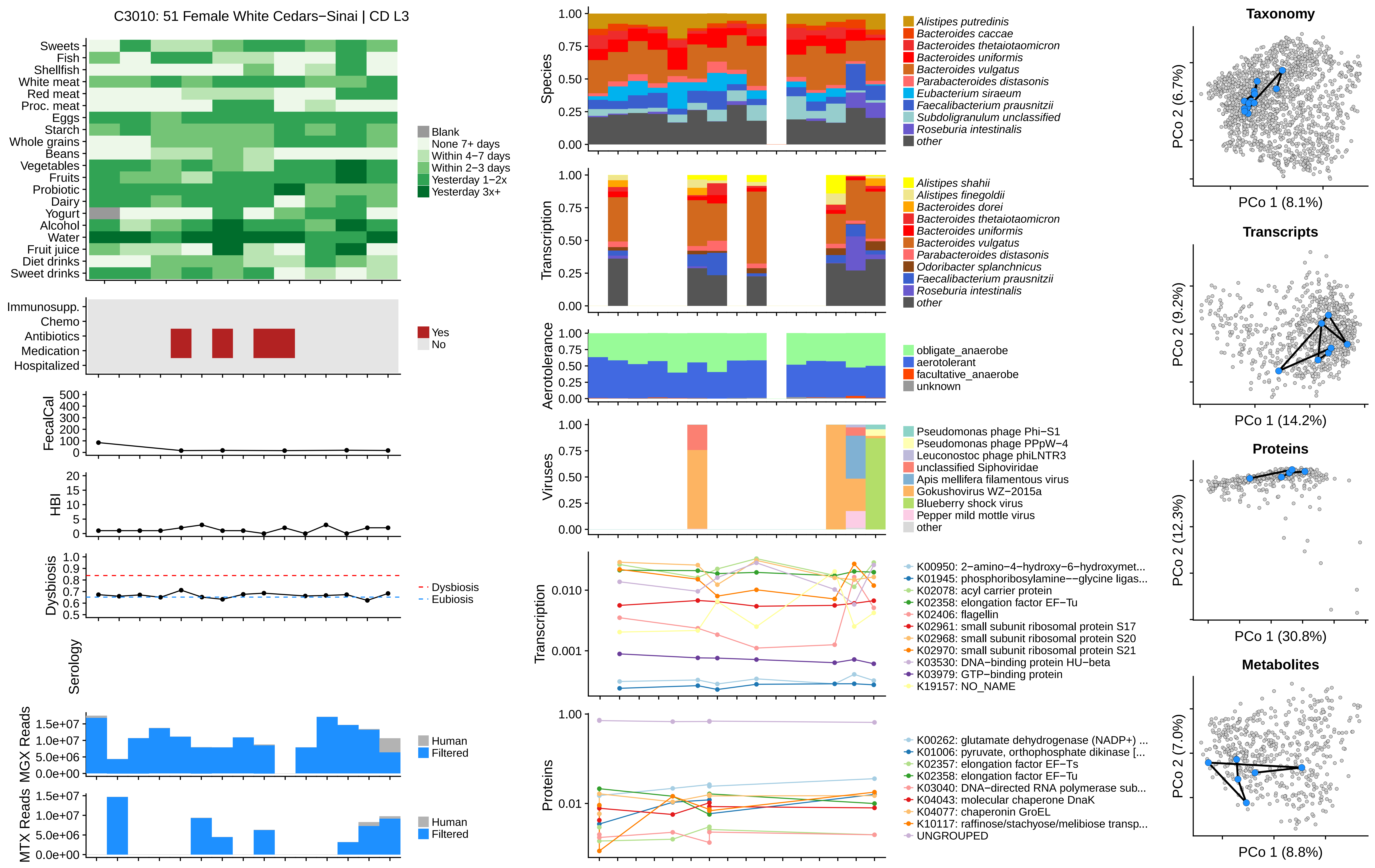
C3010: 51 Female White Cedars−Sinai | CD L3
Sweets
Fish
Shellfish
White meat
Red meat
Proc. meat
Eggs
Starch
Whole grains
Beans
Vegetables
Fruits
Probiotic
Dairy
Yogurt
Alcohol
Water
Fruit juice
Diet drinks
Sweet drinks
Blank
None 7+ days
Within 4−7 days
Within 2−3 days
Yesterday 1−2x
Yesterday 3x+
Immunosupp.
Chemo
Antibiotics
Medication
Hospitalized
Yes
No
FecalCal
HBI
Dysbiosis
Dysbiosis
Eubiosis
Serology
MGX Reads
Human
Filtered
MTX Reads
Human
Filtered
Species
Alistipes putredinis
Bacteroides caccae
Bacteroides thetaiotaomicron
Bacteroides uniformis
Bacteroides vulgatus
Parabacteroides distasonis
Eubacterium siraeum
Faecalibacterium prausnitzii
Subdoligranulum unclassified
Roseburia intestinalis
other
Transcription
Alistipes shahii
Alistipes finegoldii
Bacteroides dorei
Bacteroides thetaiotaomicron
Bacteroides uniformis
Bacteroides vulgatus
Parabacteroides distasonis
Odoribacter splanchnicus
Faecalibacterium prausnitzii
Roseburia intestinalis
other
Aerotolerance
obligate_anaerobe
aerotolerant
facultative_anaerobe
unknown
Viruses
Pseudomonas phage Phi−S1
Pseudomonas phage PPpW−4
Leuconostoc phage philNTR3
unclassified Siphoviridae
Apis mellifera filamentous virus
Gokushovirus WZ−2015a
Blueberry shock virus
Pepper mild mottle virus
other
Transcription
K00950: 2−amino−4−hydroxy−6−hydroxymet...
K01945: phosphoribosylamine−−glycine ligas...
K02078: acyl carrier protein
K02358: elongation factor EF−Tu
K02406: flagellin
K02961: small subunit ribosomal protein S17
K02968: small subunit ribosomal protein S20
K02970: small subunit ribosomal protein S21
K03530: DNA−binding protein HU−beta
K03979: GTP−binding protein
K19157: NO_NAME
Proteins
K00262: glutamate dehydrogenase (NADP+) ...
K01006: pyruvate, orthophosphate dikinase [...
K02357: elongation factor EF−Ts
K02358: elongation factor EF−Tu
K03040: DNA−directed RNA polymerase sub...
K04043: molecular chaperone DnaK
K04077: chaperonin GroEL
K10117: raffinose/stachyose/melibiose transp...
UNGROUPED
Taxonomy
PCo 1 (8.1%)
PCo 2 (6.7%)
Transcripts
PCo 1 (14.2%)
PCo 2 (9.2%)
Proteins
PCo 1 (30.8%)
PCo 2 (12.3%)
Metabolites
PCo 1 (8.8%)
PCo 2 (7.0%)

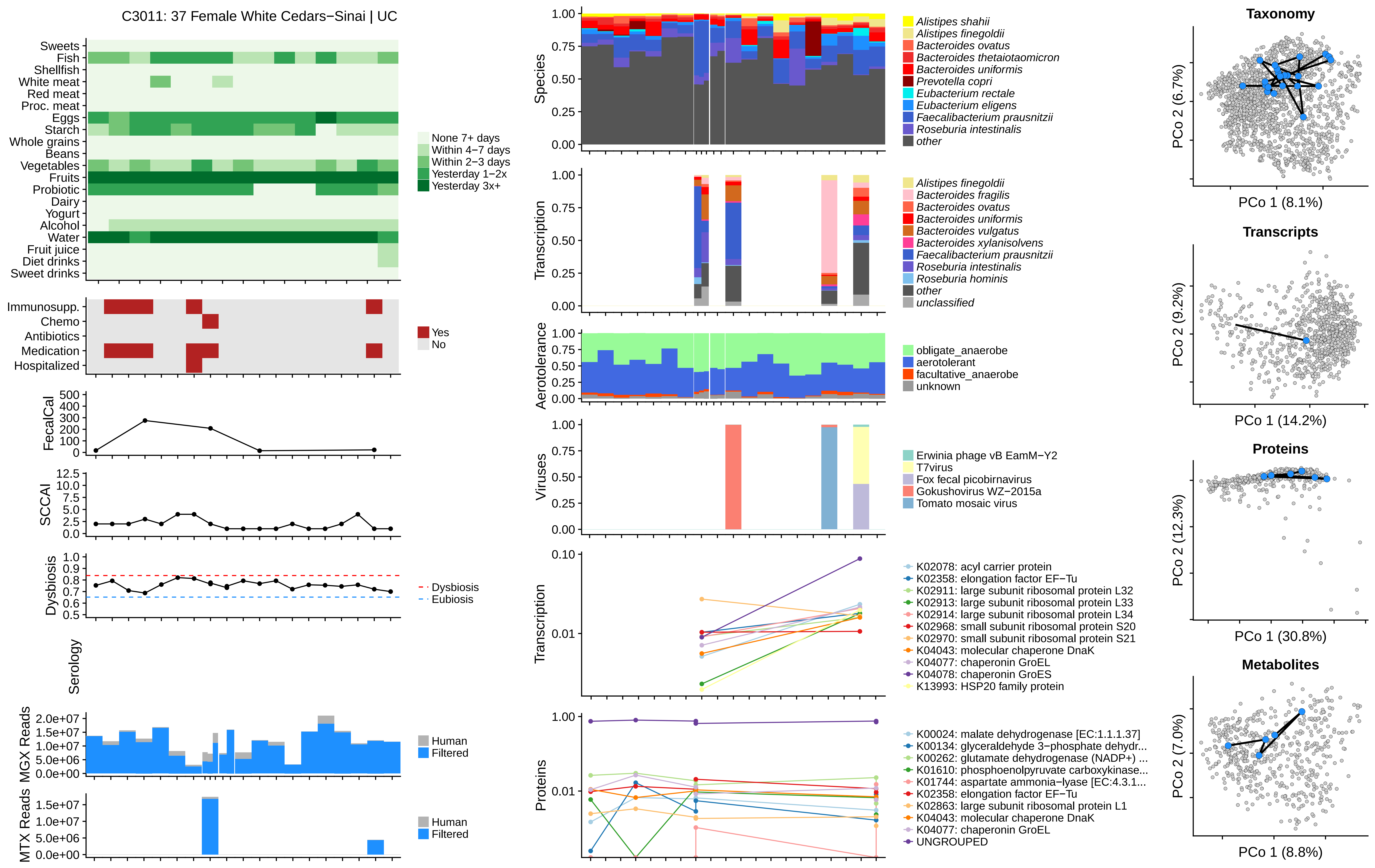
C3011: 37 Female White Cedars−Sinai | UC
Sweets
Fish
Shellfish
White meat
Red meat
Proc. meat
Eggs
Starch
Whole grains
Beans
Vegetables
Fruits
Probiotic
Dairy
Yogurt
Alcohol
Water
Fruit juice
Diet drinks
Sweet drinks
None 7+ days
Within 4−7 days
Within 2−3 days
Yesterday 1−2x
Yesterday 3x+
Immunosupp.
Chemo
Antibiotics
Medication
Hospitalized
Yes
No
FecalCal
SCCAI
Dysbiosis
Dysbiosis
Eubiosis
Serology
MGX Reads
Human
Filtered
MTX Reads
Human
Filtered
Species
Alistipes shahii
Alistipes finegoldii
Bacteroides ovatus
Bacteroides thetaiotaomicron
Bacteroides uniformis
Prevotella copri
Eubacterium rectale
Eubacterium eligens
Faecalibacterium prausnitzii
Roseburia intestinalis
other
Transcription
Alistipes finegoldii
Bacteroides fragilis
Bacteroides ovatus
Bacteroides uniformis
Bacteroides vulgatus
Bacteroides xylanisolvens
Faecalibacterium prausnitzii
Roseburia intestinalis
Roseburia hominis
other
unclassified
Aerotolerance
obligate_anaerobe
aerotolerant
facultative_anaerobe
unknown
Viruses
Erwinia phage vB EamM−Y2
T7virus
Fox fecal picobirnavirus
Gokushovirus WZ−2015a
Tomato mosaic virus
Transcription
K02078: acyl carrier protein
K02358: elongation factor EF−Tu
K02911: large subunit ribosomal protein L32
K02913: large subunit ribosomal protein L33
K02914: large subunit ribosomal protein L34
K02968: small subunit ribosomal protein S20
K02970: small subunit ribosomal protein S21
K04043: molecular chaperone DnaK
K04077: chaperonin GroEL
K04078: chaperonin GroES
K13993: HSP20 family protein
Proteins
K00024: malate dehydrogenase [EC:1.1.1.37]
K00134: glyceraldehyde 3−phosphate dehydr...
K00262: glutamate dehydrogenase (NADP+) ...
K01610: phosphoenolpyruvate carboxykinase...
K01744: aspartate ammonia−lyase [EC:4.3.1...
K02358: elongation factor EF−Tu
K02863: large subunit ribosomal protein L1
K04043: molecular chaperone DnaK
K04077: chaperonin GroEL
UNGROUPED
Taxonomy
PCo 1 (8.1%)
PCo 2 (6.7%)
Transcripts
PCo 1 (14.2%)
PCo 2 (9.2%)
Proteins
PCo 1 (30.8%)
PCo 2 (12.3%)
Metabolites
PCo 1 (8.8%)
PCo 2 (7.0%)

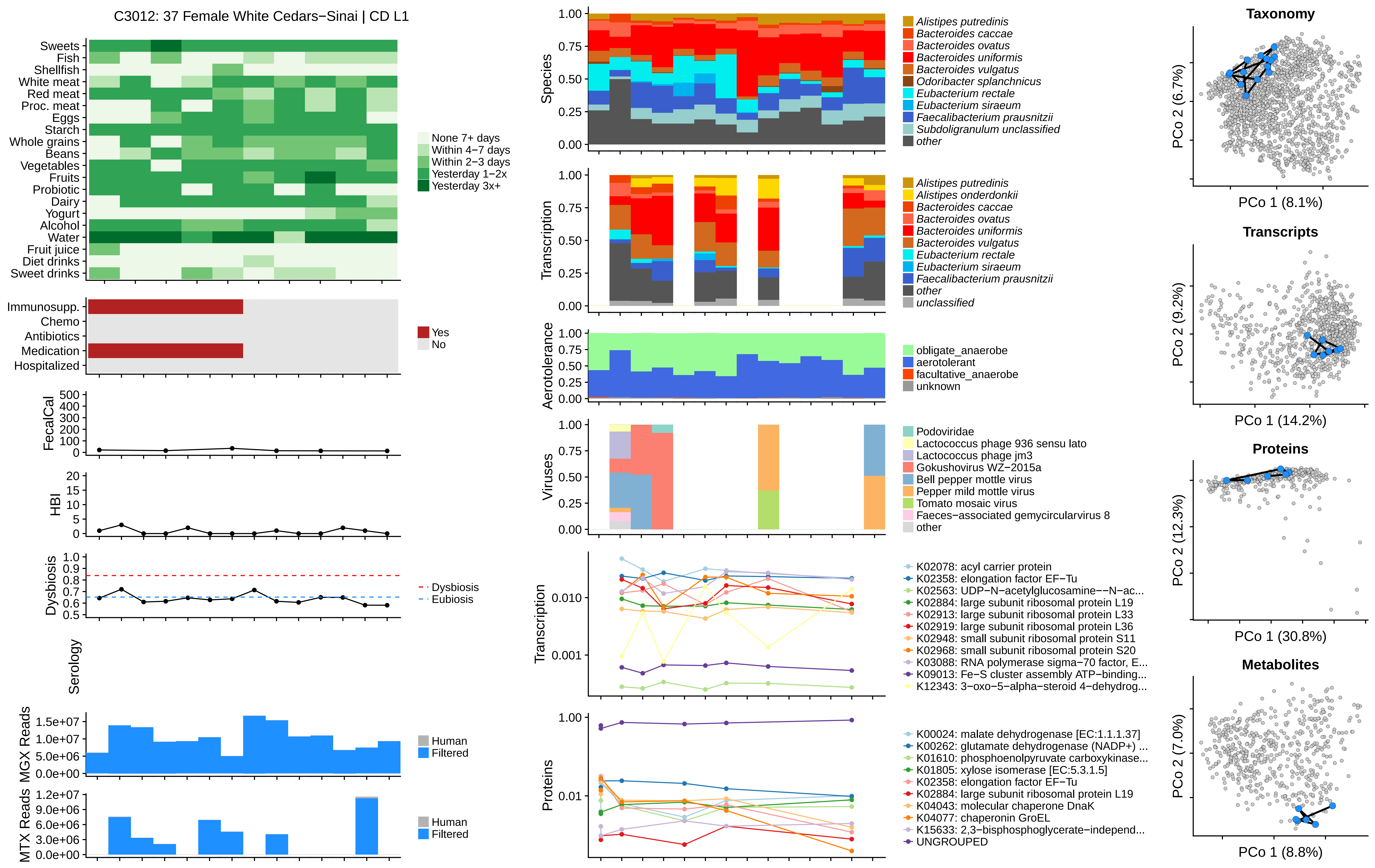
C3012: 37 Female White Cedars−Sinai | CD L1
Sweets
Fish
Shellfish
White meat
Red meat
Proc. meat
Eggs
Starch
Whole grains
Beans
Vegetables
Fruits
Probiotic
Dairy
Yogurt
Alcohol
Water
Fruit juice
Diet drinks
Sweet drinks
None 7+ days
Within 4−7 days
Within 2−3 days
Yesterday 1−2x
Yesterday 3x+
Immunosupp.
Chemo
Antibiotics
Medication
Hospitalized
Yes
No
FecalCal
HBI
Dysbiosis
Dysbiosis
Eubiosis
Serology
MGX Reads
Human
Filtered
MTX Reads
Human
Filtered
Species
Alistipes putredinis
Bacteroides caccae
Bacteroides ovatus
Bacteroides uniformis
Bacteroides vulgatus
Odoribacter splanchnicus
Eubacterium rectale
Eubacterium siraeum
Faecalibacterium prausnitzii
Subdoligranulum unclassified
other
Transcription
Alistipes putredinis
Alistipes onderdonkii
Bacteroides caccae
Bacteroides ovatus
Bacteroides uniformis
Bacteroides vulgatus
Eubacterium rectale
Eubacterium siraeum
Faecalibacterium prausnitzii
other
unclassified
Aerotolerance
obligate_anaerobe
aerotolerant
facultative_anaerobe
unknown
Viruses
Podoviridae
Lactococcus phage 936 sensu lato
Lactococcus phage jm3
Gokushovirus WZ−2015a
Bell pepper mottle virus
Pepper mild mottle virus
Tomato mosaic virus
Faeces−associated gemycircularvirus 8
other
Transcription
K02078: acyl carrier protein
K02358: elongation factor EF−Tu
K02563: UDP−N−acetylglucosamine−−N−ac...
K02884: large subunit ribosomal protein L19
K02913: large subunit ribosomal protein L33
K02919: large subunit ribosomal protein L36
K02948: small subunit ribosomal protein S11
K02968: small subunit ribosomal protein S20
K03088: RNA polymerase sigma−70 factor, E...
K09013: Fe−S cluster assembly ATP−binding...
K12343: 3−oxo−5−alpha−steroid 4−dehydrog...
Proteins
K00024: malate dehydrogenase [EC:1.1.1.37]
K00262: glutamate dehydrogenase (NADP+) ...
K01610: phosphoenolpyruvate carboxykinase...
K01805: xylose isomerase [EC:5.3.1.5]
K02358: elongation factor EF−Tu
K02884: large subunit ribosomal protein L19
K04043: molecular chaperone DnaK
K04077: chaperonin GroEL
K15633: 2,3−bisphosphoglycerate−independ...
UNGROUPED
Taxonomy
PCo 1 (8.1%)
PCo 2 (6.7%)
Transcripts
PCo 1 (14.2%)
PCo 2 (9.2%)
Proteins
PCo 1 (30.8%)
PCo 2 (12.3%)
Metabolites
PCo 1 (8.8%)
PCo 2 (7.0%)

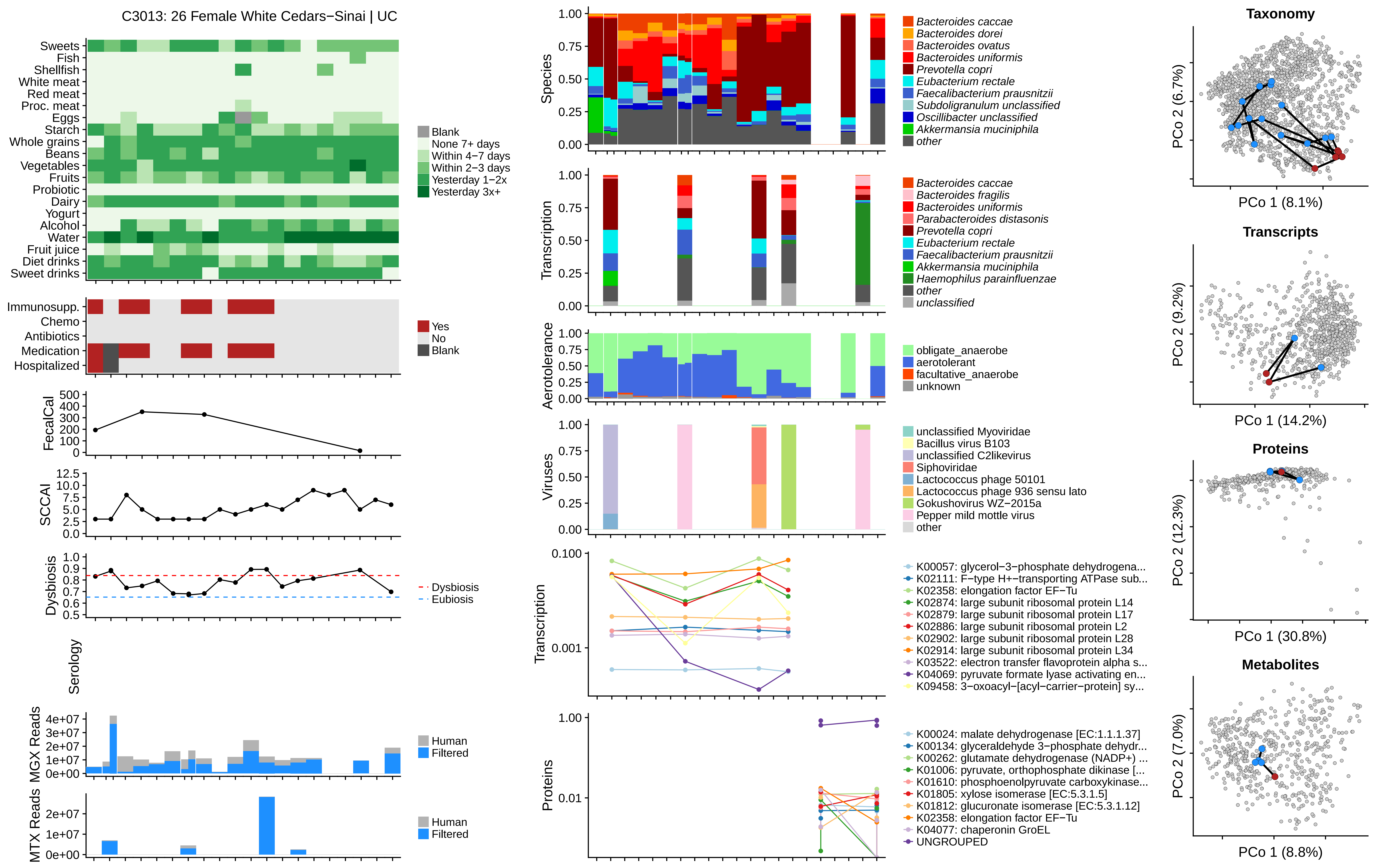

C3013: 26 Female White Cedars−Sinai | UC
Sweets
Fish
Shellfish
White meat
Red meat
Proc. meat
Eggs
Starch
Whole grains
Beans
Vegetables
Fruits
Probiotic
Dairy
Yogurt
Alcohol
Water
Fruit juice
Diet drinks
Sweet drinks
Blank
None 7+ days
Within 4−7 days
Within 2−3 days
Yesterday 1−2x
Yesterday 3x+
Immunosupp.
Chemo
Antibiotics
Medication
Hospitalized
Yes
No
Blank
FecalCal
SCCAI
Dysbiosis
Dysbiosis
Eubiosis
Serology
MGX Reads
Human
Filtered
MTX Reads
Human
Filtered
Species
Bacteroides caccae
Bacteroides dorei
Bacteroides ovatus
Bacteroides uniformis
Prevotella copri
Eubacterium rectale
Faecalibacterium prausnitzii
Subdoligranulum unclassified
Oscillibacter unclassified
Akkermansia muciniphila
other
Transcription
Bacteroides caccae
Bacteroides fragilis
Bacteroides uniformis
Parabacteroides distasonis
Prevotella copri
Eubacterium rectale
Faecalibacterium prausnitzii
Akkermansia muciniphila
Haemophilus parainfluenzae
other
unclassified
Aerotolerance
obligate_anaerobe
aerotolerant
facultative_anaerobe
unknown
Viruses
unclassified Myoviridae
Bacillus virus B103
unclassified C2likevirus
Siphoviridae
Lactococcus phage 50101
Lactococcus phage 936 sensu lato
Gokushovirus WZ−2015a
Pepper mild mottle virus
other
Transcription
K00057: glycerol−3−phosphate dehydrogena...
K02111: F−type H+−transporting ATPase sub...
K02358: elongation factor EF−Tu
K02874: large subunit ribosomal protein L14
K02879: large subunit ribosomal protein L17
K02886: large subunit ribosomal protein L2
K02902: large subunit ribosomal protein L28
K02914: large subunit ribosomal protein L34
K03522: electron transfer flavoprotein alpha s...
K04069: pyruvate formate lyase activating en...
K09458: 3−oxoacyl−[acyl−carrier−protein] sy...
Proteins
K00024: malate dehydrogenase [EC:1.1.1.37]
K00134: glyceraldehyde 3−phosphate dehydr...
K00262: glutamate dehydrogenase (NADP+) ...
K01006: pyruvate, orthophosphate dikinase [...
K01610: phosphoenolpyruvate carboxykinase...
K01805: xylose isomerase [EC:5.3.1.5]
K01812: glucuronate isomerase [EC:5.3.1.12]
K02358: elongation factor EF−Tu
K04077: chaperonin GroEL
UNGROUPED
Taxonomy
PCo 1 (8.1%)
PCo 2 (6.7%)
Transcripts
PCo 1 (14.2%)
PCo 2 (9.2%)
Proteins
PCo 1 (30.8%)
PCo 2 (12.3%)
Metabolites
PCo 1 (8.8%)
PCo 2 (7.0%)

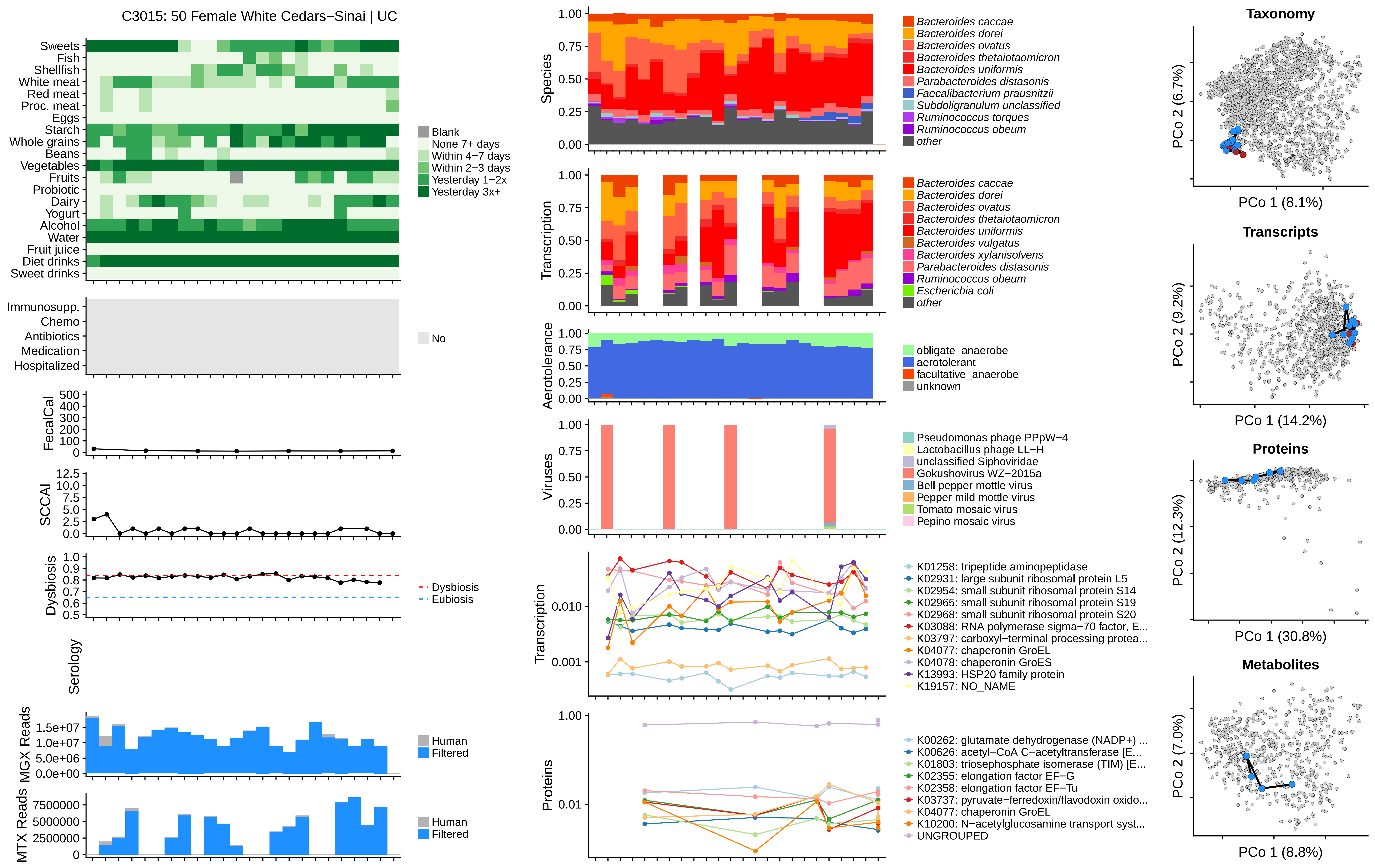
C3015: 50 Female White Cedars−Sinai | UC
Sweets
Fish
Shellfish
White meat
Red meat
Proc. meat
Eggs
Starch
Whole grains
Beans
Vegetables
Fruits
Probiotic
Dairy
Yogurt
Alcohol
Water
Fruit juice
Diet drinks
Sweet drinks
Blank
None 7+ days
Within 4−7 days
Within 2−3 days
Yesterday 1−2x
Yesterday 3x+
Immunosupp.
Chemo
Antibiotics
Medication
Hospitalized
No
FecalCal
SCCAI
Dysbiosis
Dysbiosis
Eubiosis
Serology
MGX Reads
Human
Filtered
MTX Reads
Human
Filtered
Species
Bacteroides caccae
Bacteroides dorei
Bacteroides ovatus
Bacteroides thetaiotaomicron
Bacteroides uniformis
Parabacteroides distasonis
Faecalibacterium prausnitzii
Subdoligranulum unclassified
Ruminococcus torques
Ruminococcus obeum
other
Transcription
Bacteroides caccae
Bacteroides dorei
Bacteroides ovatus
Bacteroides thetaiotaomicron
Bacteroides uniformis
Bacteroides vulgatus
Bacteroides xylanisolvens
Parabacteroides distasonis
Ruminococcus obeum
Escherichia coli
other
Aerotolerance
obligate_anaerobe
aerotolerant
facultative_anaerobe
unknown
Viruses
Pseudomonas phage PPpW−4
Lactobacillus phage LL−H
unclassified Siphoviridae
Gokushovirus WZ−2015a
Bell pepper mottle virus
Pepper mild mottle virus
Tomato mosaic virus
Pepino mosaic virus
Transcription
K01258: tripeptide aminopeptidase
K02931: large subunit ribosomal protein L5
K02954: small subunit ribosomal protein S14
K02965: small subunit ribosomal protein S19
K02968: small subunit ribosomal protein S20
K03088: RNA polymerase sigma−70 factor, E...
K03797: carboxyl−terminal processing protea...
K04077: chaperonin GroEL
K04078: chaperonin GroES
K13993: HSP20 family protein
K19157: NO_NAME
Proteins
K00262: glutamate dehydrogenase (NADP+) ...
K00626: acetyl−CoA C−acetyltransferase [E...
K01803: triosephosphate isomerase (TIM) [E...
K02355: elongation factor EF−G
K02358: elongation factor EF−Tu
K03737: pyruvate−ferredoxin/flavodoxin oxido...
K04077: chaperonin GroEL
K10200: N−acetylglucosamine transport syst...
UNGROUPED
Taxonomy
PCo 1 (8.1%)
PCo 2 (6.7%)
Transcripts
PCo 1 (14.2%)
PCo 2 (9.2%)
Proteins
PCo 1 (30.8%)
PCo 2 (12.3%)
Metabolites
PCo 1 (8.8%)
PCo 2 (7.0%)

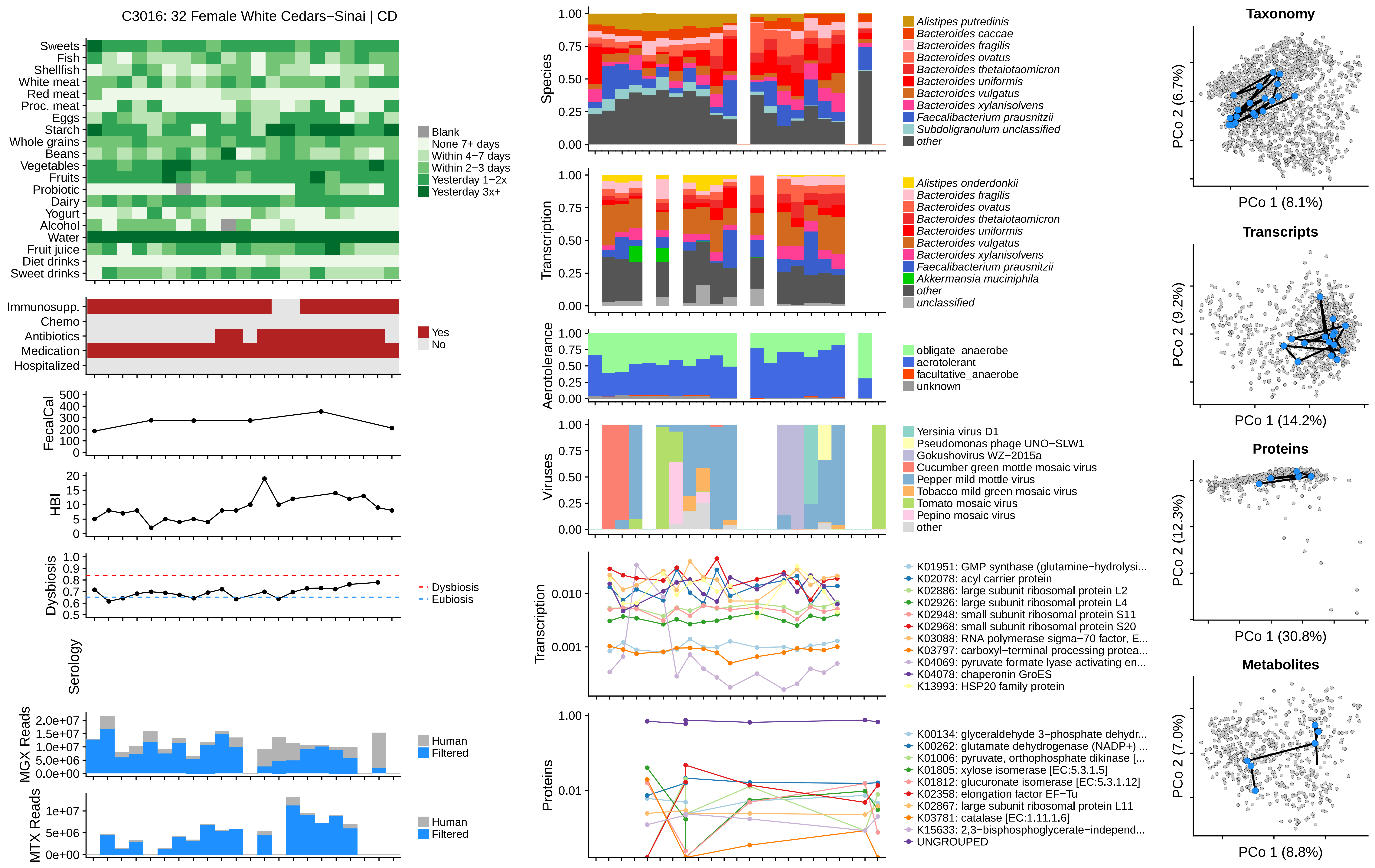
C3016: 32 Female White Cedars−Sinai | CD
Sweets
Fish
Shellfish
White meat
Red meat
Proc. meat
Eggs
Starch
Whole grains
Beans
Vegetables
Fruits
Probiotic
Dairy
Yogurt
Alcohol
Water
Fruit juice
Diet drinks
Sweet drinks
Blank
None 7+ days
Within 4−7 days
Within 2−3 days
Yesterday 1−2x
Yesterday 3x+
Immunosupp.
Chemo
Antibiotics
Medication
Hospitalized
Yes
No
FecalCal
HBI
Dysbiosis
Dysbiosis
Eubiosis
Serology
MGX Reads
Human
Filtered
MTX Reads
Human
Filtered
Species
Alistipes putredinis
Bacteroides caccae
Bacteroides fragilis
Bacteroides ovatus
Bacteroides thetaiotaomicron
Bacteroides uniformis
Bacteroides vulgatus
Bacteroides xylanisolvens
Faecalibacterium prausnitzii
Subdoligranulum unclassified
other
Transcription
Alistipes onderdonkii
Bacteroides fragilis
Bacteroides ovatus
Bacteroides thetaiotaomicron
Bacteroides uniformis
Bacteroides vulgatus
Bacteroides xylanisolvens
Faecalibacterium prausnitzii
Akkermansia muciniphila
other
unclassified
Aerotolerance
obligate_anaerobe
aerotolerant
facultative_anaerobe
unknown
Viruses
Yersinia virus D1
Pseudomonas phage UNO−SLW1
Gokushovirus WZ−2015a
Cucumber green mottle mosaic virus
Pepper mild mottle virus
Tobacco mild green mosaic virus
Tomato mosaic virus
Pepino mosaic virus
other
Transcription
K01951: GMP synthase (glutamine−hydrolysi...
K02078: acyl carrier protein
K02886: large subunit ribosomal protein L2
K02926: large subunit ribosomal protein L4
K02948: small subunit ribosomal protein S11
K02968: small subunit ribosomal protein S20
K03088: RNA polymerase sigma−70 factor, E...
K03797: carboxyl−terminal processing protea...
K04069: pyruvate formate lyase activating en...
K04078: chaperonin GroES
K13993: HSP20 family protein
Proteins
K00134: glyceraldehyde 3−phosphate dehydr...
K00262: glutamate dehydrogenase (NADP+) ...
K01006: pyruvate, orthophosphate dikinase [...
K01805: xylose isomerase [EC:5.3.1.5]
K01812: glucuronate isomerase [EC:5.3.1.12]
K02358: elongation factor EF−Tu
K02867: large subunit ribosomal protein L11
K03781: catalase [EC:1.11.1.6]
K15633: 2,3−bisphosphoglycerate−independ...
UNGROUPED
Taxonomy
PCo 1 (8.1%)
PCo 2 (6.7%)
Transcripts
PCo 1 (14.2%)
PCo 2 (9.2%)
Proteins
PCo 1 (30.8%)
PCo 2 (12.3%)
Metabolites
PCo 1 (8.8%)
PCo 2 (7.0%)

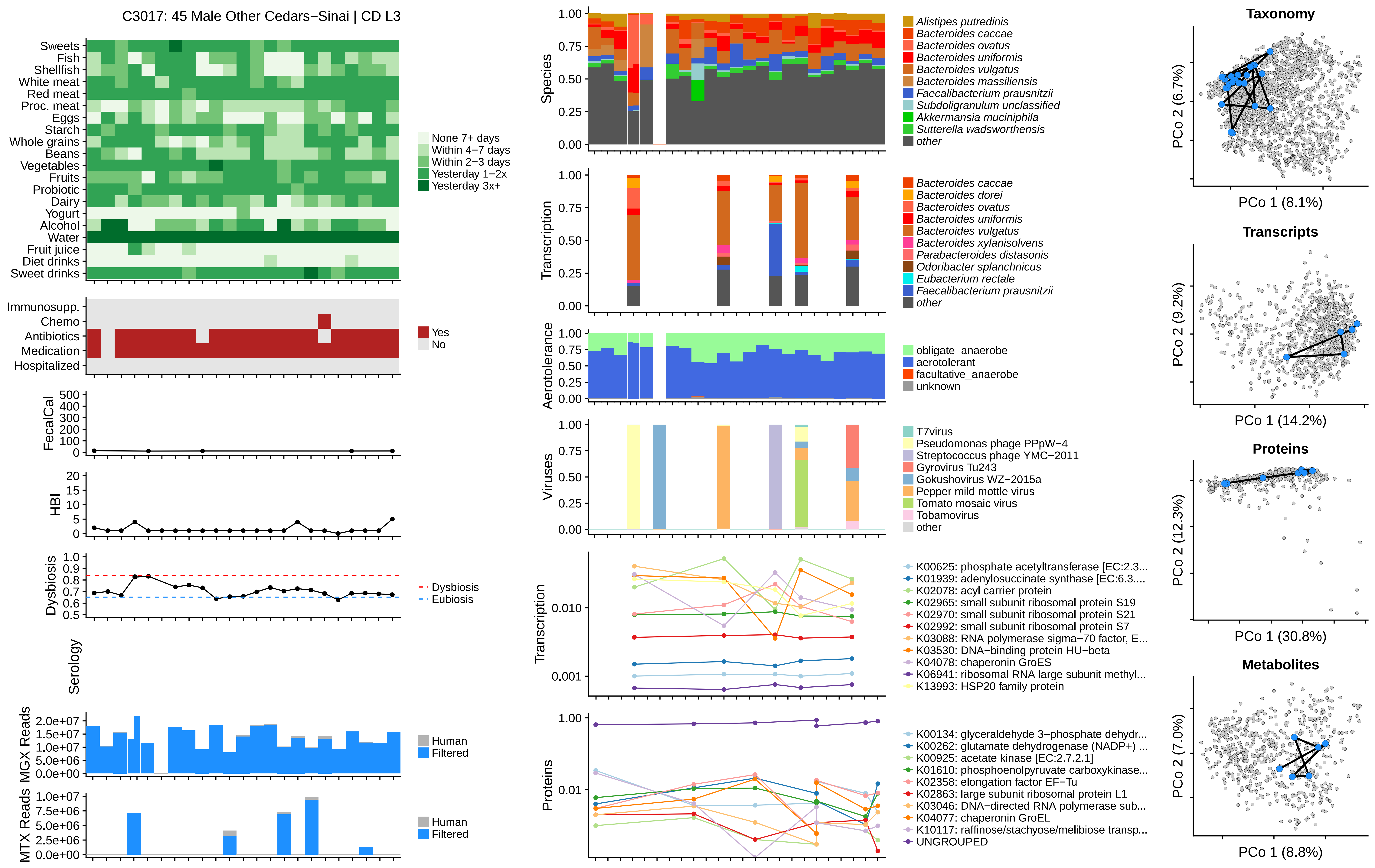
C3017: 45 Male Other Cedars−Sinai | CD L3
Sweets
Fish
Shellfish
White meat
Red meat
Proc. meat
Eggs
Starch
Whole grains
Beans
Vegetables
Fruits
Probiotic
Dairy
Yogurt
Alcohol
Water
Fruit juice
Diet drinks
Sweet drinks
None 7+ days
Within 4−7 days
Within 2−3 days
Yesterday 1−2x
Yesterday 3x+
Immunosupp.
Chemo
Antibiotics
Medication
Hospitalized
Yes
No
FecalCal
HBI
Dysbiosis
Dysbiosis
Eubiosis
Serology
MGX Reads
Human
Filtered
MTX Reads
Human
Filtered
Species
Alistipes putredinis
Bacteroides caccae
Bacteroides ovatus
Bacteroides uniformis
Bacteroides vulgatus
Bacteroides massiliensis
Faecalibacterium prausnitzii
Subdoligranulum unclassified
Akkermansia muciniphila
Sutterella wadsworthensis
other
Transcription
Bacteroides caccae
Bacteroides dorei
Bacteroides ovatus
Bacteroides uniformis
Bacteroides vulgatus
Bacteroides xylanisolvens
Parabacteroides distasonis
Odoribacter splanchnicus
Eubacterium rectale
Faecalibacterium prausnitzii
other
Aerotolerance
obligate_anaerobe
aerotolerant
facultative_anaerobe
unknown
Viruses
T7virus
Pseudomonas phage PPpW−4
Streptococcus phage YMC−2011
Gyrovirus Tu243
Gokushovirus WZ−2015a
Pepper mild mottle virus
Tomato mosaic virus
Tobamovirus
other
Transcription
K00625: phosphate acetyltransferase [EC:2.3...
K01939: adenylosuccinate synthase [EC:6.3....
K02078: acyl carrier protein
K02965: small subunit ribosomal protein S19
K02970: small subunit ribosomal protein S21
K02992: small subunit ribosomal protein S7
K03088: RNA polymerase sigma−70 factor, E...
K03530: DNA−binding protein HU−beta
K04078: chaperonin GroES
K06941: ribosomal RNA large subunit methyl...
K13993: HSP20 family protein
Proteins
K00134: glyceraldehyde 3−phosphate dehydr...
K00262: glutamate dehydrogenase (NADP+) ...
K00925: acetate kinase [EC:2.7.2.1]
K01610: phosphoenolpyruvate carboxykinase...
K02358: elongation factor EF−Tu
K02863: large subunit ribosomal protein L1
K03046: DNA−directed RNA polymerase sub...
K04077: chaperonin GroEL
K10117: raffinose/stachyose/melibiose transp...
UNGROUPED
Taxonomy
PCo 1 (8.1%)
PCo 2 (6.7%)
Transcripts
PCo 1 (14.2%)
PCo 2 (9.2%)
Proteins
PCo 1 (30.8%)
PCo 2 (12.3%)
Metabolites
PCo 1 (8.8%)
PCo 2 (7.0%)

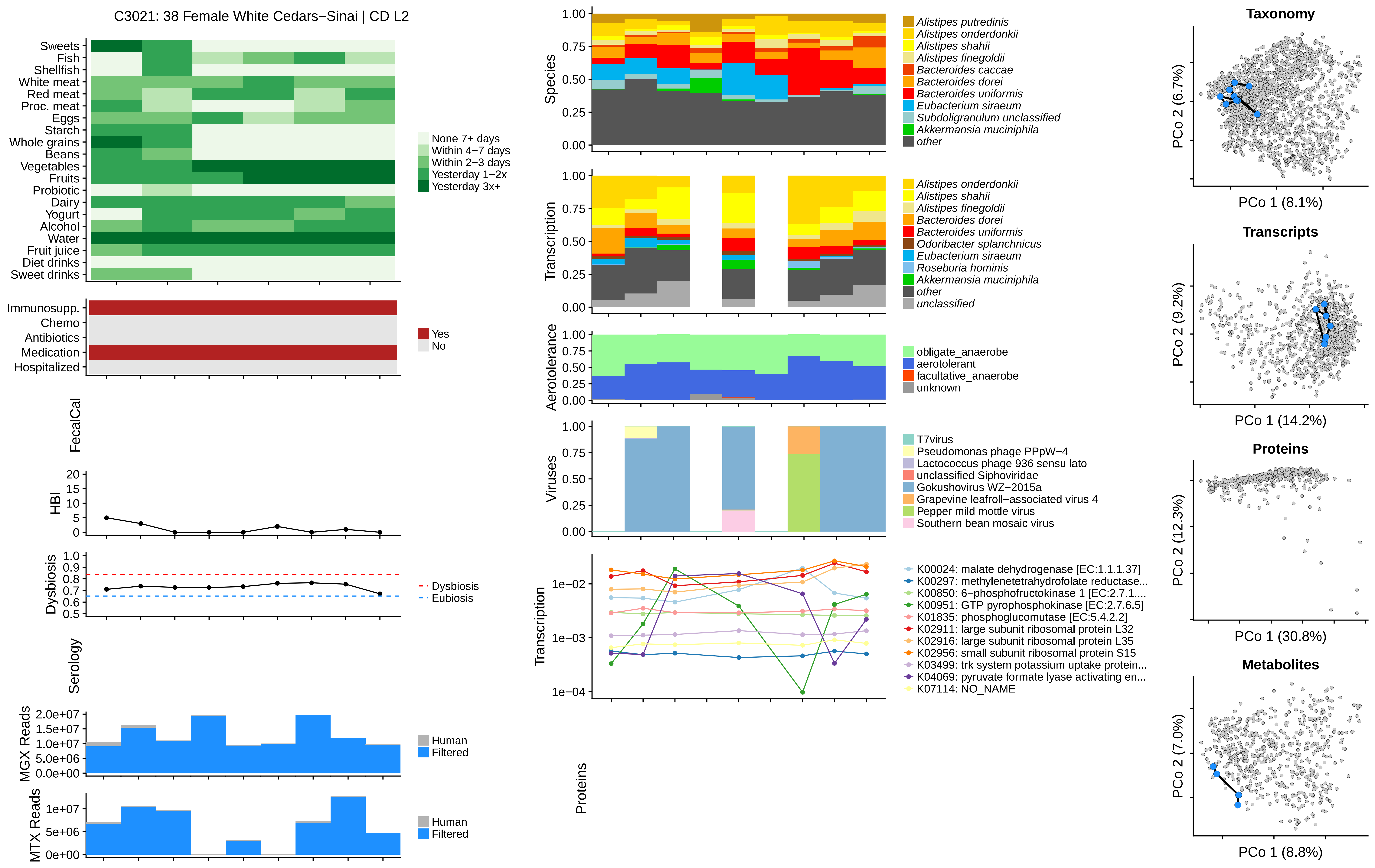

C3021: 38 Female White Cedars−Sinai | CD L2
Sweets
Fish
Shellfish
White meat
Red meat
Proc. meat
Eggs
Starch
Whole grains
Beans
Vegetables
Fruits
Probiotic
Dairy
Yogurt
Alcohol
Water
Fruit juice
Diet drinks
Sweet drinks
None 7+ days
Within 4−7 days
Within 2−3 days
Yesterday 1−2x
Yesterday 3x+
Immunosupp.
Chemo
Antibiotics
Medication
Hospitalized
Yes
No
FecalCal
HBI
Dysbiosis
Dysbiosis
Eubiosis
Serology
MGX Reads
Human
Filtered
MTX Reads
Human
Filtered
Species
Alistipes putredinis
Alistipes onderdonkii
Alistipes shahii
Alistipes finegoldii
Bacteroides caccae
Bacteroides dorei
Bacteroides uniformis
Eubacterium siraeum
Subdoligranulum unclassified
Akkermansia muciniphila
other
Transcription
Alistipes onderdonkii
Alistipes shahii
Alistipes finegoldii
Bacteroides dorei
Bacteroides uniformis
Odoribacter splanchnicus
Eubacterium siraeum
Roseburia hominis
Akkermansia muciniphila
other
unclassified
Aerotolerance
obligate_anaerobe
aerotolerant
facultative_anaerobe
unknown
Viruses
T7virus
Pseudomonas phage PPpW−4
Lactococcus phage 936 sensu lato
unclassified Siphoviridae
Gokushovirus WZ−2015a
Grapevine leafroll−associated virus 4
Pepper mild mottle virus
Southern bean mosaic virus
Transcription
K00024: malate dehydrogenase [EC:1.1.1.37]
K00297: methylenetetrahydrofolate reductase...
K00850: 6−phosphofructokinase 1 [EC:2.7.1....
K00951: GTP pyrophosphokinase [EC:2.7.6.5]
K01835: phosphoglucomutase [EC:5.4.2.2]
K02911: large subunit ribosomal protein L32
K02916: large subunit ribosomal protein L35
K02956: small subunit ribosomal protein S15
K03499: trk system potassium uptake protein...
K04069: pyruvate formate lyase activating en...
K07114: NO_NAME
Proteins
Taxonomy
PCo 1 (8.1%)
PCo 2 (6.7%)
Transcripts
PCo 1 (14.2%)
PCo 2 (9.2%)
Proteins
PCo 1 (30.8%)
PCo 2 (12.3%)
Metabolites
PCo 1 (8.8%)
PCo 2 (7.0%)

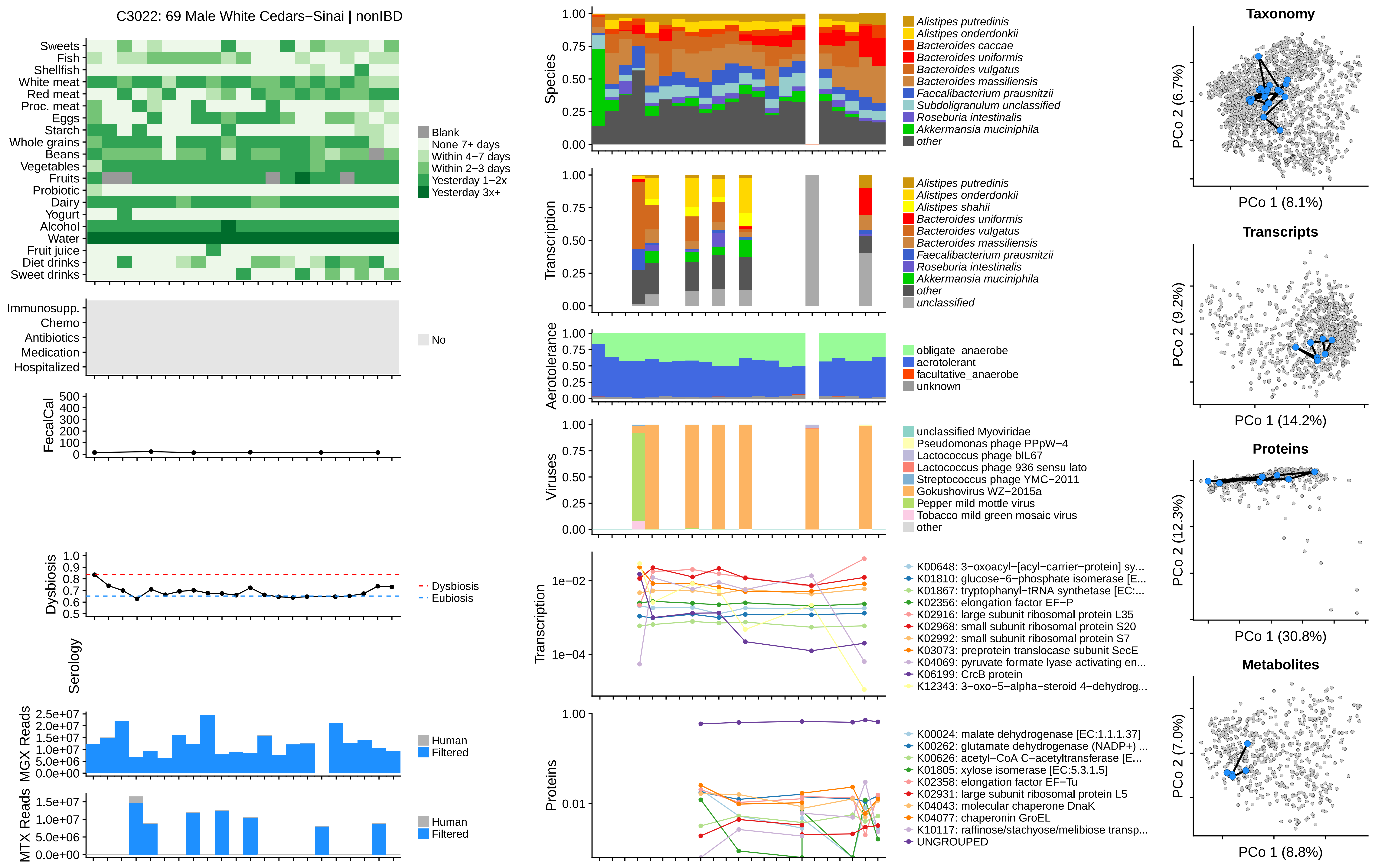
C3022: 69 Male White Cedars−Sinai | nonIBD
Sweets
Fish
Shellfish
White meat
Red meat
Proc. meat
Eggs
Starch
Whole grains
Beans
Vegetables
Fruits
Probiotic
Dairy
Yogurt
Alcohol
Water
Fruit juice
Diet drinks
Sweet drinks
Blank
None 7+ days
Within 4−7 days
Within 2−3 days
Yesterday 1−2x
Yesterday 3x+
Immunosupp.
Chemo
Antibiotics
Medication
Hospitalized
No
FecalCal
Dysbiosis
Dysbiosis
Eubiosis
Serology
MGX Reads
Human
Filtered
MTX Reads
Human
Filtered
Species
Alistipes putredinis
Alistipes onderdonkii
Bacteroides caccae
Bacteroides uniformis
Bacteroides vulgatus
Bacteroides massiliensis
Faecalibacterium prausnitzii
Subdoligranulum unclassified
Roseburia intestinalis
Akkermansia muciniphila
other
Transcription
Alistipes putredinis
Alistipes onderdonkii
Alistipes shahii
Bacteroides uniformis
Bacteroides vulgatus
Bacteroides massiliensis
Faecalibacterium prausnitzii
Roseburia intestinalis
Akkermansia muciniphila
other
unclassified
Aerotolerance
obligate_anaerobe
aerotolerant
facultative_anaerobe
unknown
Viruses
unclassified Myoviridae
Pseudomonas phage PPpW−4
Lactococcus phage bIL67
Lactococcus phage 936 sensu lato
Streptococcus phage YMC−2011
Gokushovirus WZ−2015a
Pepper mild mottle virus
Tobacco mild green mosaic virus
other
Transcription
K00648: 3−oxoacyl−[acyl−carrier−protein] sy...
K01810: glucose−6−phosphate isomerase [E...
K01867: tryptophanyl−tRNA synthetase [EC:...
K02356: elongation factor EF−P
K02916: large subunit ribosomal protein L35
K02968: small subunit ribosomal protein S20
K02992: small subunit ribosomal protein S7
K03073: preprotein translocase subunit SecE
K04069: pyruvate formate lyase activating en...
K06199: CrcB protein
K12343: 3−oxo−5−alpha−steroid 4−dehydrog...
Proteins
K00024: malate dehydrogenase [EC:1.1.1.37]
K00262: glutamate dehydrogenase (NADP+) ...
K00626: acetyl−CoA C−acetyltransferase [E...
K01805: xylose isomerase [EC:5.3.1.5]
K02358: elongation factor EF−Tu
K02931: large subunit ribosomal protein L5
K04043: molecular chaperone DnaK
K04077: chaperonin GroEL
K10117: raffinose/stachyose/melibiose transp...
UNGROUPED
Taxonomy
PCo 1 (8.1%)
PCo 2 (6.7%)
Transcripts
PCo 1 (14.2%)
PCo 2 (9.2%)
Proteins
PCo 1 (30.8%)
PCo 2 (12.3%)
Metabolites
PCo 1 (8.8%)
PCo 2 (7.0%)

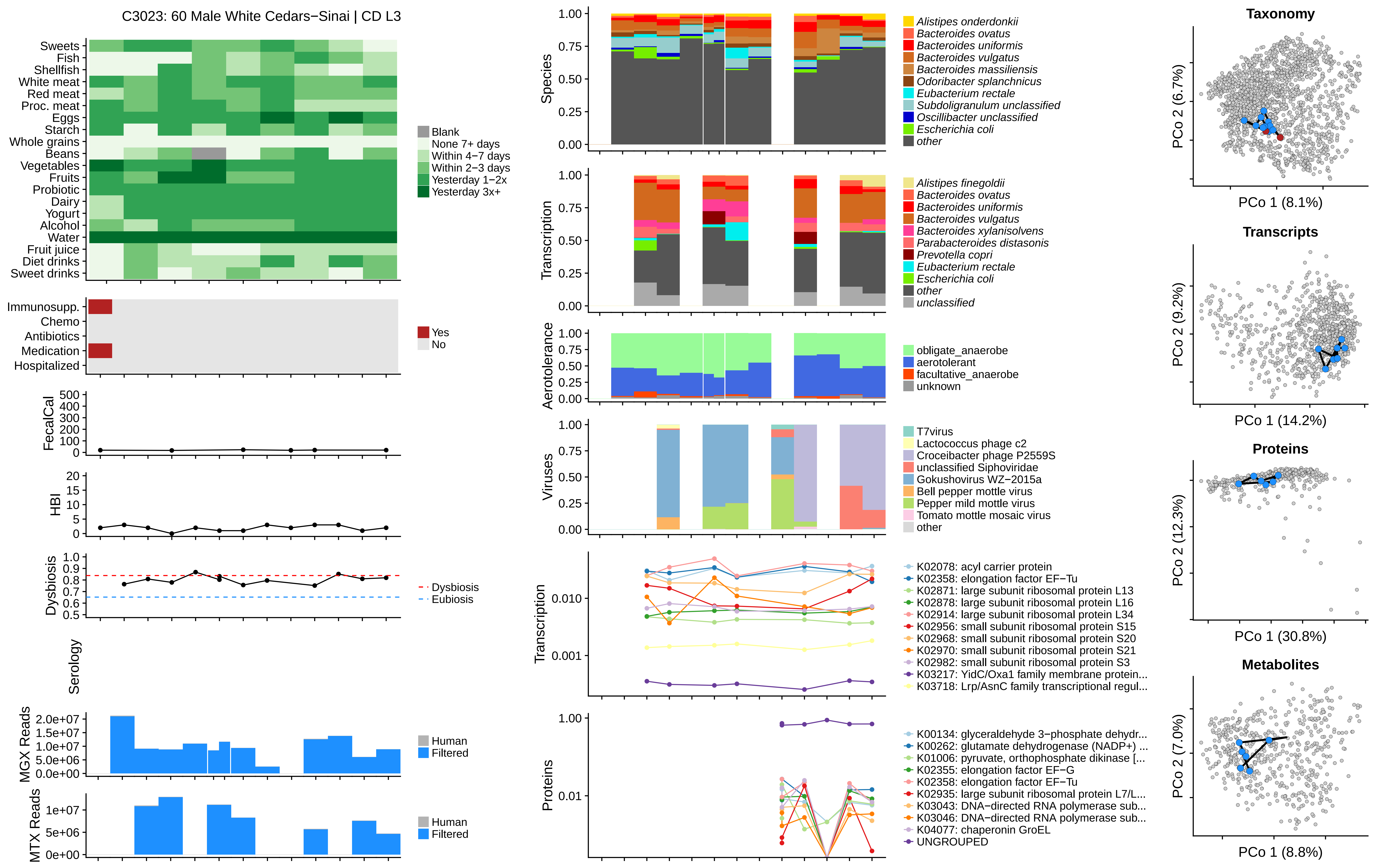

C3023: 60 Male White Cedars−Sinai | CD L3
Sweets
Fish
Shellfish
White meat
Red meat
Proc. meat
Eggs
Starch
Whole grains
Beans
Vegetables
Fruits
Probiotic
Dairy
Yogurt
Alcohol
Water
Fruit juice
Diet drinks
Sweet drinks
Blank
None 7+ days
Within 4−7 days
Within 2−3 days
Yesterday 1−2x
Yesterday 3x+
Immunosupp.
Chemo
Antibiotics
Medication
Hospitalized
Yes
No
FecalCal
HBI
Dysbiosis
Dysbiosis
Eubiosis
Serology
MGX Reads
Human
Filtered
MTX Reads
Human
Filtered
Species
Alistipes onderdonkii
Bacteroides ovatus
Bacteroides uniformis
Bacteroides vulgatus
Bacteroides massiliensis
Odoribacter splanchnicus
Eubacterium rectale
Subdoligranulum unclassified
Oscillibacter unclassified
Escherichia coli
other
Transcription
Alistipes finegoldii
Bacteroides ovatus
Bacteroides uniformis
Bacteroides vulgatus
Bacteroides xylanisolvens
Parabacteroides distasonis
Prevotella copri
Eubacterium rectale
Escherichia coli
other
unclassified
Aerotolerance
obligate_anaerobe
aerotolerant
facultative_anaerobe
unknown
Viruses
T7virus
Lactococcus phage c2
Croceibacter phage P2559S
unclassified Siphoviridae
Gokushovirus WZ−2015a
Bell pepper mottle virus
Pepper mild mottle virus
Tomato mottle mosaic virus
other
Transcription
K02078: acyl carrier protein
K02358: elongation factor EF−Tu
K02871: large subunit ribosomal protein L13
K02878: large subunit ribosomal protein L16
K02914: large subunit ribosomal protein L34
K02956: small subunit ribosomal protein S15
K02968: small subunit ribosomal protein S20
K02970: small subunit ribosomal protein S21
K02982: small subunit ribosomal protein S3
K03217: YidC/Oxa1 family membrane protein...
K03718: Lrp/AsnC family transcriptional regul...
Proteins
K00134: glyceraldehyde 3−phosphate dehydr...
K00262: glutamate dehydrogenase (NADP+) ...
K01006: pyruvate, orthophosphate dikinase [...
K02355: elongation factor EF−G
K02358: elongation factor EF−Tu
K02935: large subunit ribosomal protein L7/L...
K03043: DNA−directed RNA polymerase sub...
K03046: DNA−directed RNA polymerase sub...
K04077: chaperonin GroEL
UNGROUPED
Taxonomy
PCo 1 (8.1%)
PCo 2 (6.7%)
Transcripts
PCo 1 (14.2%)
PCo 2 (9.2%)
Proteins
PCo 1 (30.8%)
PCo 2 (12.3%)
Metabolites
PCo 1 (8.8%)
PCo 2 (7.0%)

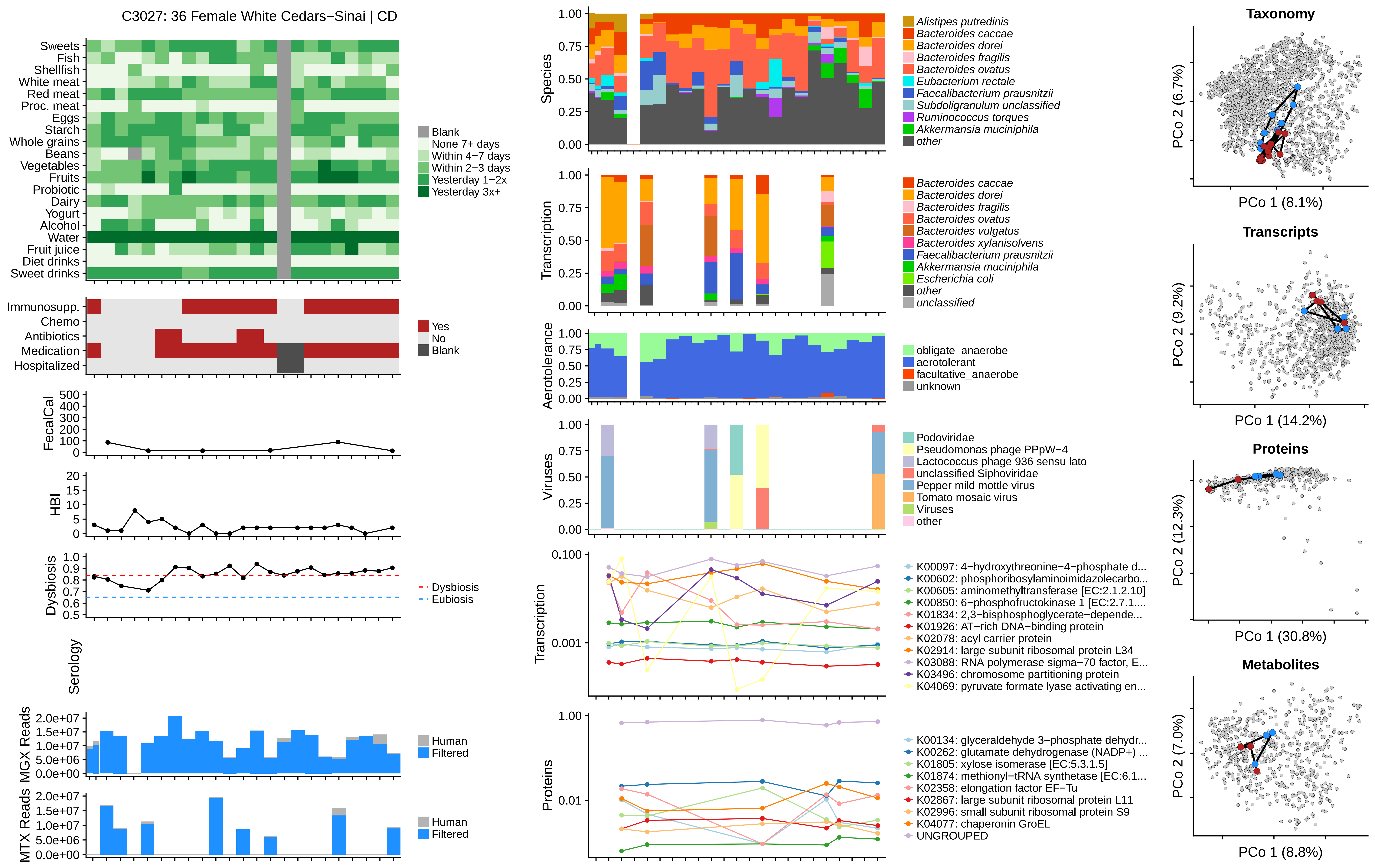
C3027: 36 Female White Cedars−Sinai | CD
Sweets
Fish
Shellfish
White meat
Red meat
Proc. meat
Eggs
Starch
Whole grains
Beans
Vegetables
Fruits
Probiotic
Dairy
Yogurt
Alcohol
Water
Fruit juice
Diet drinks
Sweet drinks
Blank
None 7+ days
Within 4−7 days
Within 2−3 days
Yesterday 1−2x
Yesterday 3x+
Immunosupp.
Chemo
Antibiotics
Medication
Hospitalized
Yes
No
Blank
FecalCal
500
400
300
200
100
0
HBI
20
15
10
5
0
Dysbiosis
1.0
0.9
0.8
0.7
0.6
0.5
Dysbiosis
Eubiosis
Serology
MGX Reads
2.0e+07
1.5e+07
1.0e+07
5.0e+06
0.0e+00
Human
Filtered
MTX Reads
2.0e+07
1.5e+07
1.0e+07
5.0e+06
0.0e+00
Human
Filtered
Species
1.00
0.75
0.50
0.25
0.00
Alistipes putredinis
Bacteroides caccae
Bacteroides dorei
Bacteroides fragilis
Bacteroides ovatus
Eubacterium rectale
Faecalibacterium prausnitzii
Subdoligranulum unclassified
Ruminococcus torques
Akkermansia muciniphila
other
Transcription
1.00
0.75
0.50
0.25
0.00
Bacteroides caccae
Bacteroides dorei
Bacteroides fragilis
Bacteroides ovatus
Bacteroides vulgatus
Bacteroides xylanisolvens
Faecalibacterium prausnitzii
Akkermansia muciniphila
Escherichia coli
other
unclassified
Aerotolerance
1.00
0.75
0.50
0.25
0.00
obligate_anaerobe
aerotolerant
facultative_anaerobe
unknown
Viruses
1.00
0.75
0.50
0.25
0.00
Podoviridae
Pseudomonas phage PPpW−4
Lactococcus phage 936 sensu lato
unclassified Siphoviridae
Pepper mild mottle virus
Tomato mosaic virus
Viruses
other
Transcription
0.100
0.001
K00097: 4−hydroxythreonine−4−phosphate d...
K00602: phosphoribosylaminoimidazolecarbo...
K00605: aminomethyltransferase [EC:2.1.2.10]
K00850: 6−phosphofructokinase 1 [EC:2.7.1....
K01834: 2,3−bisphosphoglycerate−depende...
K01926: AT−rich DNA−binding protein
K02078: acyl carrier protein
K02914: large subunit ribosomal protein L34
K03088: RNA polymerase sigma−70 factor, E...
K03496: chromosome partitioning protein
K04069: pyruvate formate lyase activating en...
Proteins
1.00
0.01
K00134: glyceraldehyde 3−phosphate dehydr...
K00262: glutamate dehydrogenase (NADP+) ...
K01805: xylose isomerase [EC:5.3.1.5]
K01874: methionyl−tRNA synthetase [EC:6.1...
K02358: elongation factor EF−Tu
K02867: large subunit ribosomal protein L11
K02996: small subunit ribosomal protein S9
K04077: chaperonin GroEL
UNGROUPED
Taxonomy
PCo 2 (6.7%)
PCo 1 (8.1%)
Transcripts
PCo 2 (9.2%)
PCo 1 (14.2%)
Proteins
PCo 2 (12.3%)
PCo 1 (30.8%)
Metabolites
PCo 2 (7.0%)
PCo 1 (8.8%)

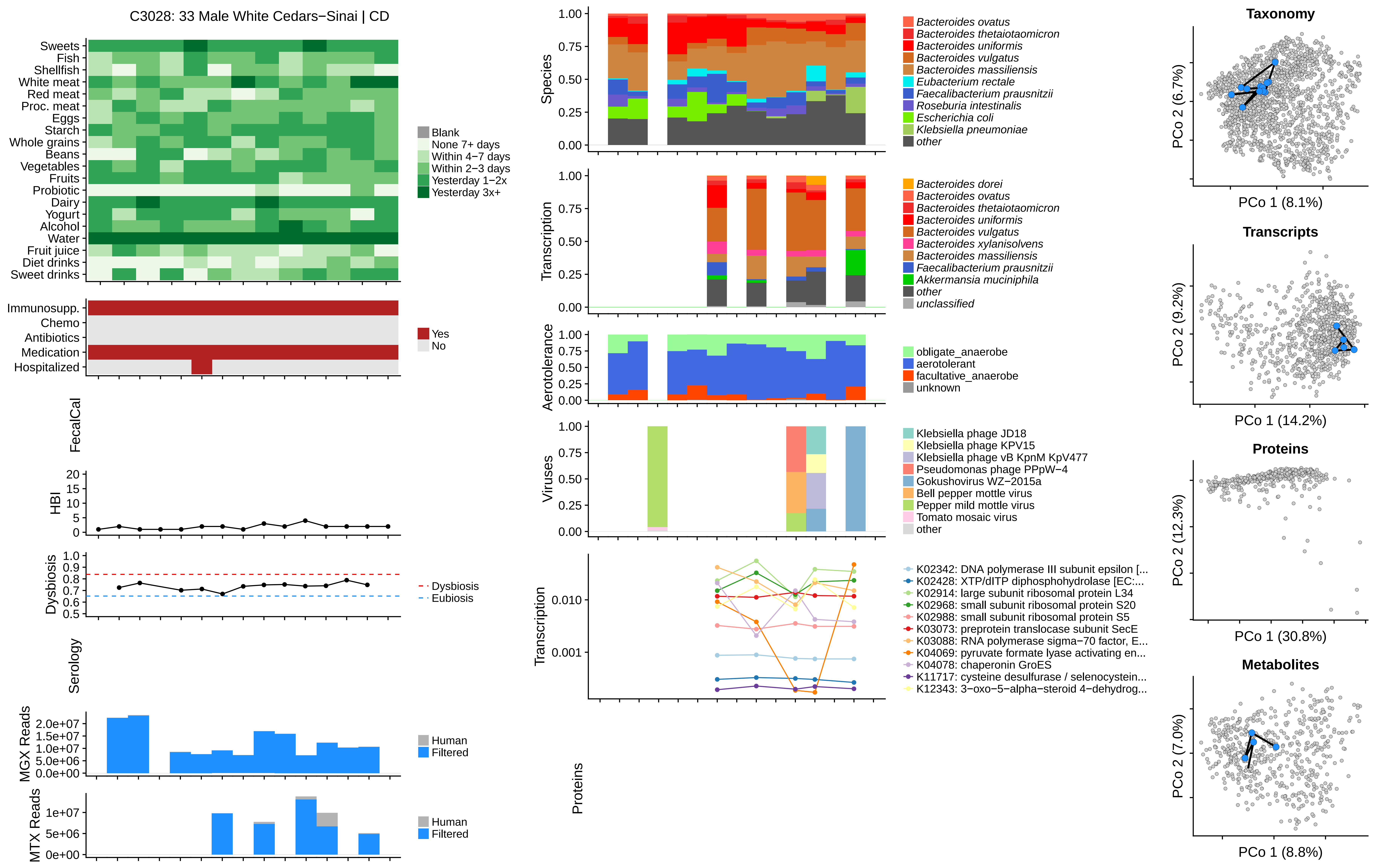
C3028: 33 Male White Cedars−Sinai | CD
Sweets
Fish
Shellfish
White meat
Red meat
Proc. meat
Eggs
Starch
Whole grains
Beans
Vegetables
Fruits
Probiotic
Dairy
Yogurt
Alcohol
Water
Fruit juice
Diet drinks
Sweet drinks
Blank
None 7+ days
Within 4−7 days
Within 2−3 days
Yesterday 1−2x
Yesterday 3x+
Immunosupp.
Chemo
Antibiotics
Medication
Hospitalized
Yes
No
FecalCal
HBI
Dysbiosis
Dysbiosis
Eubiosis
Serology
MGX Reads
Human
Filtered
MTX Reads
Human
Filtered
Species
Bacteroides ovatus
Bacteroides thetaiotaomicron
Bacteroides uniformis
Bacteroides vulgatus
Bacteroides massiliensis
Eubacterium rectale
Faecalibacterium prausnitzii
Roseburia intestinalis
Escherichia coli
Klebsiella pneumoniae
other
Transcription
Bacteroides dorei
Bacteroides ovatus
Bacteroides thetaiotaomicron
Bacteroides uniformis
Bacteroides vulgatus
Bacteroides xylanisolvens
Bacteroides massiliensis
Faecalibacterium prausnitzii
Akkermansia muciniphila
other
unclassified
Aerotolerance
obligate_anaerobe
aerotolerant
facultative_anaerobe
unknown
Viruses
Klebsiella phage JD18
Klebsiella phage KPV15
Klebsiella phage vB KpnM KpV477
Pseudomonas phage PPpW−4
Gokushovirus WZ−2015a
Bell pepper mottle virus
Pepper mild mottle virus
Tomato mosaic virus
other
Transcription
K02342: DNA polymerase III subunit epsilon [...
K02428: XTP/dITP diphosphohydrolase [EC:...
K02914: large subunit ribosomal protein L34
K02968: small subunit ribosomal protein S20
K02988: small subunit ribosomal protein S5
K03073: preprotein translocase subunit SecE
K03088: RNA polymerase sigma−70 factor, E...
K04069: pyruvate formate lyase activating en...
K04078: chaperonin GroES
K11717: cysteine desulfurase / selenocystein...
K12343: 3−oxo−5−alpha−steroid 4−dehydrog...
Proteins
Taxonomy
PCo 1 (8.1%)
PCo 2 (6.7%)
Transcripts
PCo 1 (14.2%)
PCo 2 (9.2%)
Proteins
PCo 1 (30.8%)
PCo 2 (12.3%)
Metabolites
PCo 1 (8.8%)
PCo 2 (7.0%)

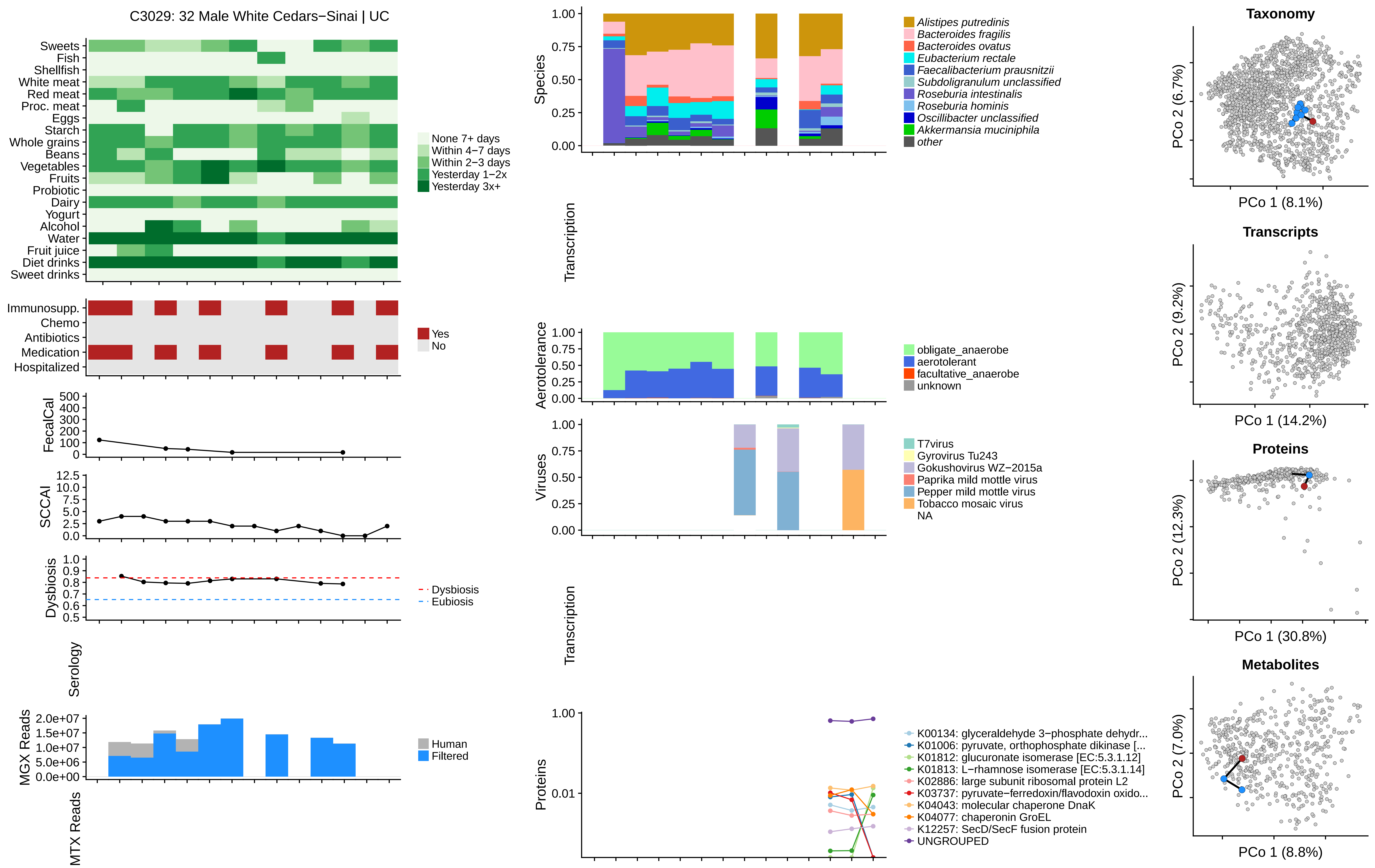
C3029: 32 Male White Cedars−Sinai | UC
Sweets
Fish
Shellfish
White meat
Red meat
Proc. meat
Eggs
Starch
Whole grains
Beans
Vegetables
Fruits
Probiotic
Dairy
Yogurt
Alcohol
Water
Fruit juice
Diet drinks
Sweet drinks
None 7+ days
Within 4−7 days
Within 2−3 days
Yesterday 1−2x
Yesterday 3x+
Immunosupp.
Chemo
Antibiotics
Medication
Hospitalized
Yes
No
FecalCal
SCCAI
Dysbiosis
Dysbiosis
Eubiosis
Serology
MGX Reads
Human
Filtered
MTX Reads
Species
Alistipes putredinis
Bacteroides fragilis
Bacteroides ovatus
Eubacterium rectale
Faecalibacterium prausnitzii
Subdoligranulum unclassified
Roseburia intestinalis
Roseburia hominis
Oscillibacter unclassified
Akkermansia muciniphila
other
Transcription
Aerotolerance
obligate_anaerobe
aerotolerant
facultative_anaerobe
unknown
Viruses
T7virus
Gyrovirus Tu243
Gokushovirus WZ−2015a
Paprika mild mottle virus
Pepper mild mottle virus
Tobacco mosaic virus
NA
Transcription
Proteins
K00134: glyceraldehyde 3−phosphate dehydr...
K01006: pyruvate, orthophosphate dikinase [...
K01812: glucuronate isomerase [EC:5.3.1.12]
K01813: L−rhamnose isomerase [EC:5.3.1.14]
K02886: large subunit ribosomal protein L2
K03737: pyruvate−ferredoxin/flavodoxin oxido...
K04043: molecular chaperone DnaK
K04077: chaperonin GroEL
K12257: SecD/SecF fusion protein
UNGROUPED
Taxonomy
PCo 1 (8.1%)
PCo 2 (6.7%)
Transcripts
PCo 1 (14.2%)
PCo 2 (9.2%)
Proteins
PCo 1 (30.8%)
PCo 2 (12.3%)
Metabolites
PCo 1 (8.8%)
PCo 2 (7.0%)

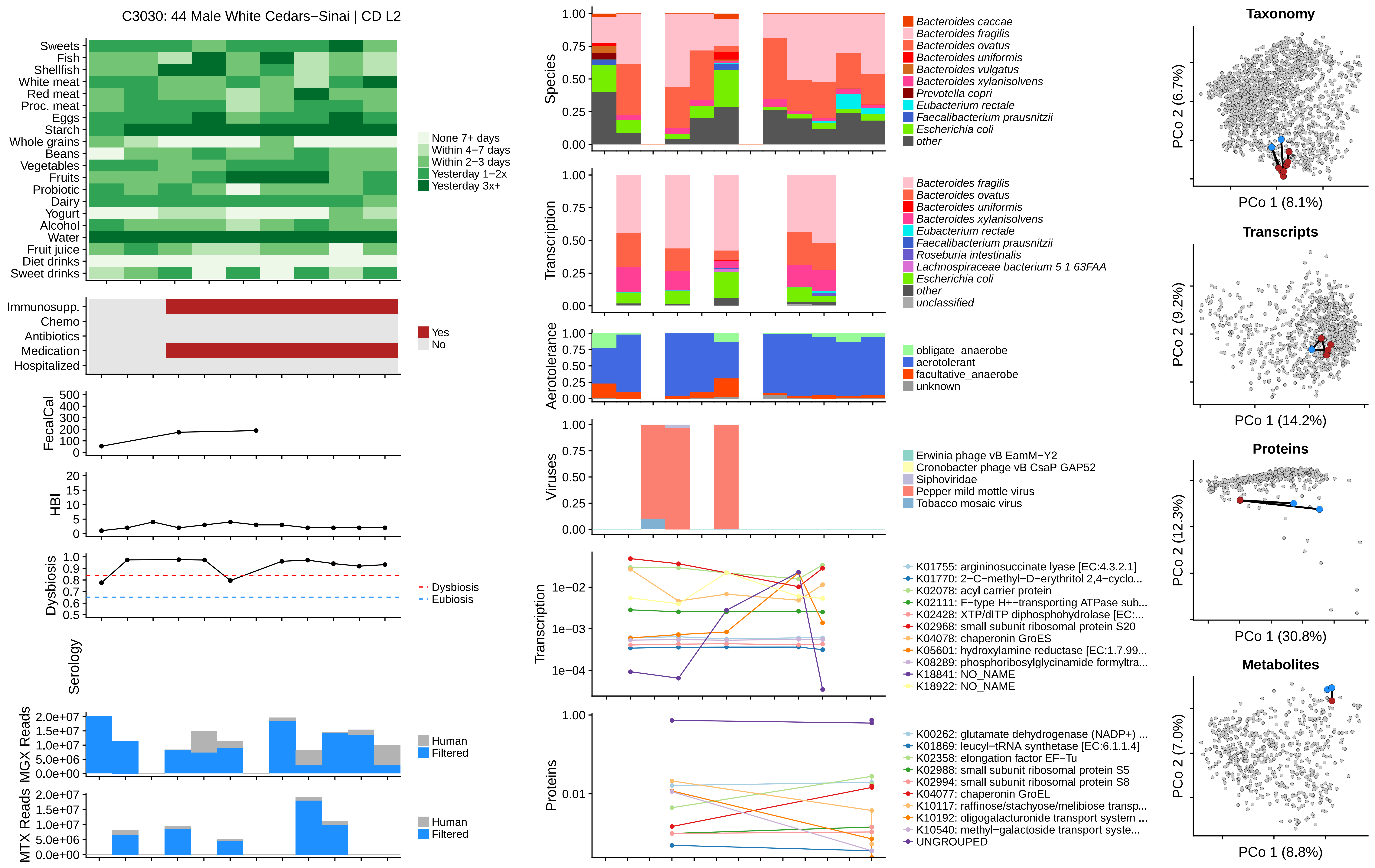
C3030: 44 Male White Cedars−Sinai | CD L2
Sweets
Fish
Shellfish
White meat
Red meat
Proc. meat
Eggs
Starch
Whole grains
Beans
Vegetables
Fruits
Probiotic
Dairy
Yogurt
Alcohol
Water
Fruit juice
Diet drinks
Sweet drinks
None 7+ days
Within 4−7 days
Within 2−3 days
Yesterday 1−2x
Yesterday 3x+
Immunosupp.
Chemo
Antibiotics
Medication
Hospitalized
Yes
No
FecalCal
HBI
Dysbiosis
Dysbiosis
Eubiosis
Serology
MGX Reads
MTX Reads
Human
Filtered
Species
Bacteroides caccae
Bacteroides fragilis
Bacteroides ovatus
Bacteroides uniformis
Bacteroides vulgatus
Bacteroides xylanisolvens
Prevotella copri
Eubacterium rectale
Faecalibacterium prausnitzii
Escherichia coli
other
Transcription
Roseburia intestinalis
Lachnospiraceae bacterium 5 1 63FAA
unclassified
Aerotolerance
obligate_anaerobe
aerotolerant
facultative_anaerobe
unknown
Viruses
Erwinia phage vB EamM−Y2
Cronobacter phage vB CsaP GAP52
Siphoviridae
Pepper mild mottle virus
Tobacco mosaic virus
K01755: argininosuccinate lyase [EC:4.3.2.1]
K01770: 2−C−methyl−D−erythritol 2,4−cyclo...
K02078: acyl carrier protein
K02111: F−type H+−transporting ATPase sub...
K02428: XTP/dITP diphosphohydrolase [EC:...
K02968: small subunit ribosomal protein S20
K04078: chaperonin GroES
K05601: hydroxylamine reductase [EC:1.7.99...
K08289: phosphoribosylglycinamide formyltra...
K18841: NO_NAME
K18922: NO_NAME
Proteins
K00262: glutamate dehydrogenase (NADP+) ...
K01869: leucyl−tRNA synthetase [EC:6.1.1.4]
K02358: elongation factor EF−Tu
K02988: small subunit ribosomal protein S5
K02994: small subunit ribosomal protein S8
K04077: chaperonin GroEL
K10117: raffinose/stachyose/melibiose transp...
K10192: oligogalacturonide transport system ...
K10540: methyl−galactoside transport syste...
UNGROUPED
Taxonomy
PCo 1 (8.1%)
PCo 2 (6.7%)
Transcripts
PCo 1 (14.2%)
PCo 2 (9.2%)
Proteins
PCo 1 (30.8%)
PCo 2 (12.3%)
Metabolites
PCo 1 (8.8%)
PCo 2 (7.0%)

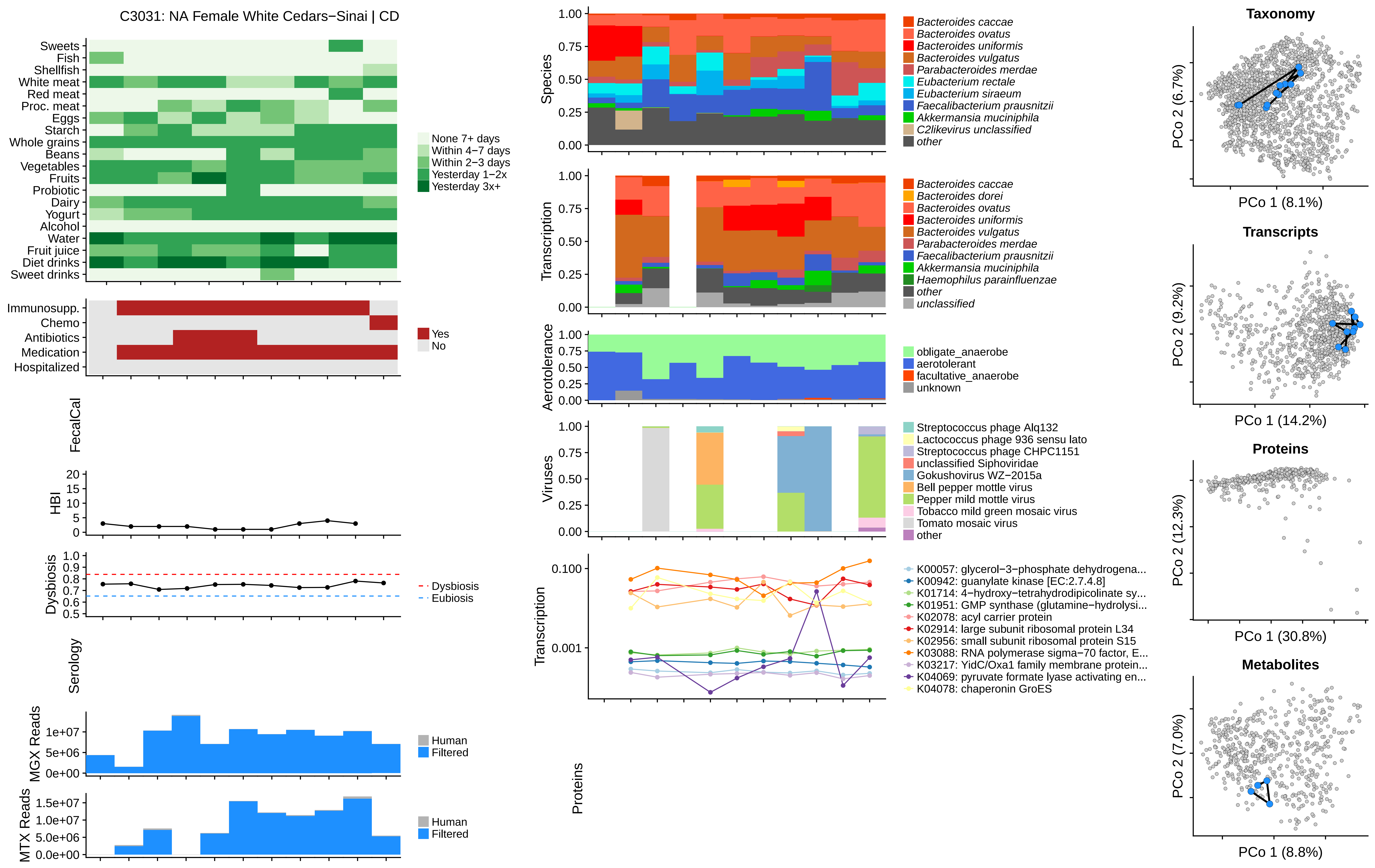

C3031: NA Female White Cedars−Sinai | CD
Sweets
Fish
Shellfish
White meat
Red meat
Proc. meat
Eggs
Starch
Whole grains
Beans
Vegetables
Fruits
Probiotic
Dairy
Yogurt
Alcohol
Water
Fruit juice
Diet drinks
Sweet drinks
None 7+ days
Within 4−7 days
Within 2−3 days
Yesterday 1−2x
Yesterday 3x+
Immunosupp.
Chemo
Antibiotics
Medication
Hospitalized
Yes
No
FecalCal
HBI
Dysbiosis
Dysbiosis
Eubiosis
Serology
MGX Reads
Human
Filtered
MTX Reads
Human
Filtered
Species
Bacteroides caccae
Bacteroides ovatus
Bacteroides uniformis
Bacteroides vulgatus
Parabacteroides merdae
Eubacterium rectale
Eubacterium siraeum
Faecalibacterium prausnitzii
Akkermansia muciniphila
C2likevirus unclassified
other
Transcription
Bacteroides caccae
Bacteroides dorei
Bacteroides ovatus
Bacteroides uniformis
Bacteroides vulgatus
Parabacteroides merdae
Faecalibacterium prausnitzii
Akkermansia muciniphila
Haemophilus parainfluenzae
other
unclassified
Aerotolerance
obligate_anaerobe
aerotolerant
facultative_anaerobe
unknown
Viruses
Streptococcus phage Alq132
Lactococcus phage 936 sensu lato
Streptococcus phage CHPC1151
unclassified Siphoviridae
Gokushovirus WZ−2015a
Bell pepper mottle virus
Pepper mild mottle virus
Tobacco mild green mosaic virus
Tomato mosaic virus
other
Transcription
K00057: glycerol−3−phosphate dehydrogena...
K00942: guanylate kinase [EC:2.7.4.8]
K01714: 4−hydroxy−tetrahydrodipicolinate sy...
K01951: GMP synthase (glutamine−hydrolysi...
K02078: acyl carrier protein
K02914: large subunit ribosomal protein L34
K02956: small subunit ribosomal protein S15
K03088: RNA polymerase sigma−70 factor, E...
K03217: YidC/Oxa1 family membrane protein...
K04069: pyruvate formate lyase activating en...
K04078: chaperonin GroES
Proteins
Taxonomy
PCo 1 (8.1%)
PCo 2 (6.7%)
Transcripts
PCo 1 (14.2%)
PCo 2 (9.2%)
Proteins
PCo 1 (30.8%)
PCo 2 (12.3%)
Metabolites
PCo 1 (8.8%)
PCo 2 (7.0%)

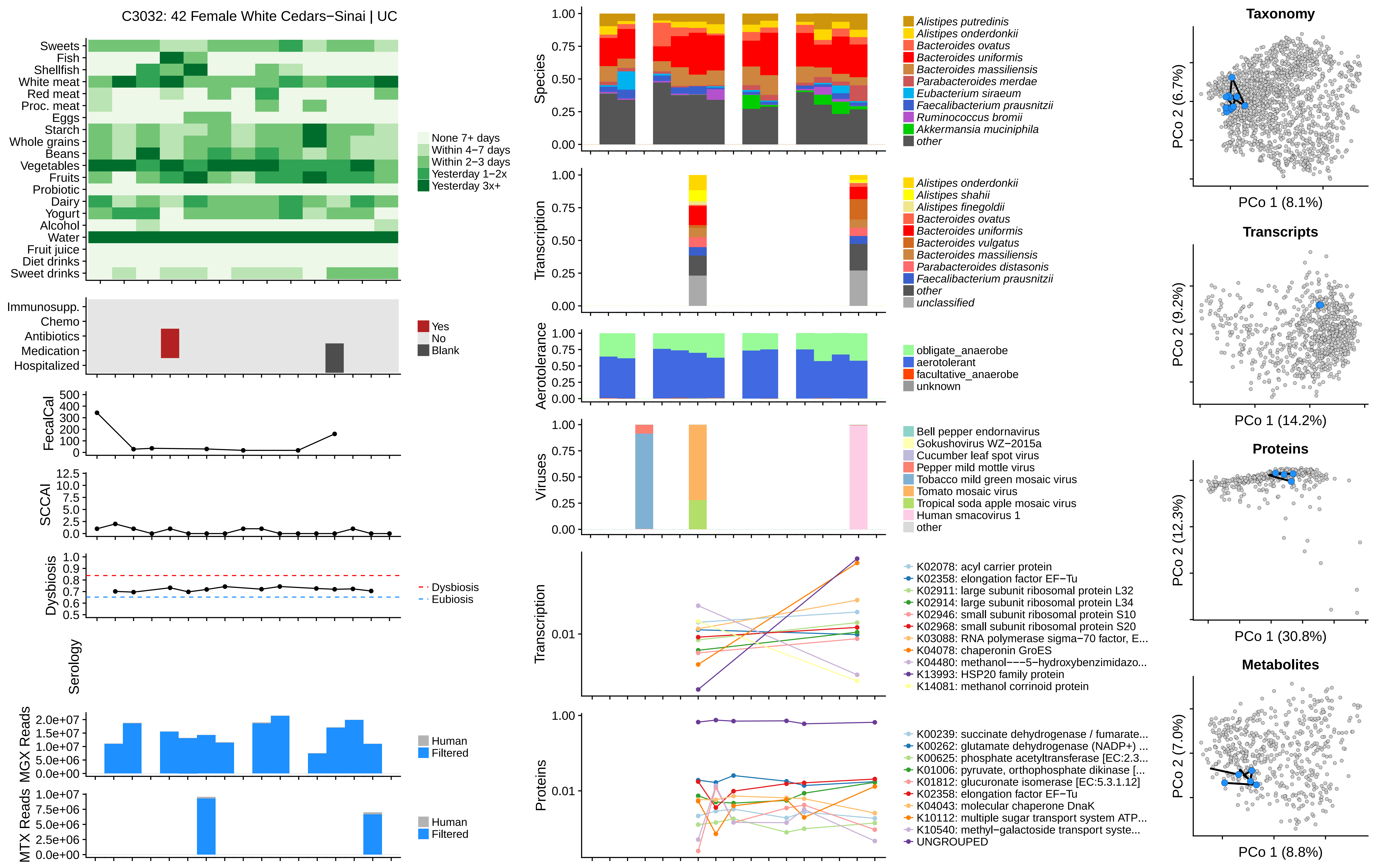
C3032: 42 Female White Cedars−Sinai | UC
Sweets
Fish
Shellfish
White meat
Red meat
Proc. meat
Eggs
Starch
Whole grains
Beans
Vegetables
Fruits
Probiotic
Dairy
Yogurt
Alcohol
Water
Fruit juice
Diet drinks
Sweet drinks
None 7+ days
Within 4−7 days
Within 2−3 days
Yesterday 1−2x
Yesterday 3x+
Immunosupp.
Chemo
Antibiotics
Medication
Hospitalized
Yes
No
Blank
FecalCal
SCCAI
Dysbiosis
Dysbiosis
Eubiosis
Serology
MGX Reads
Human
Filtered
MTX Reads
Human
Filtered
Species
Alistipes putredinis
Alistipes onderdonkii
Bacteroides ovatus
Bacteroides uniformis
Bacteroides massiliensis
Parabacteroides merdae
Eubacterium siraeum
Faecalibacterium prausnitzii
Ruminococcus bromii
Akkermansia muciniphila
other
Transcription
Alistipes onderdonkii
Alistipes shahii
Alistipes finegoldii
Bacteroides ovatus
Bacteroides uniformis
Bacteroides vulgatus
Bacteroides massiliensis
Parabacteroides distasonis
Faecalibacterium prausnitzii
other
unclassified
Aerotolerance
obligate_anaerobe
aerotolerant
facultative_anaerobe
unknown
Viruses
Bell pepper endornavirus
Gokushovirus WZ−2015a
Cucumber leaf spot virus
Pepper mild mottle virus
Tobacco mild green mosaic virus
Tomato mosaic virus
Tropical soda apple mosaic virus
Human smacovirus 1
other
Transcription
K02078: acyl carrier protein
K02358: elongation factor EF−Tu
K02911: large subunit ribosomal protein L32
K02914: large subunit ribosomal protein L34
K02946: small subunit ribosomal protein S10
K02968: small subunit ribosomal protein S20
K03088: RNA polymerase sigma−70 factor, E...
K04078: chaperonin GroES
K04480: methanol−−−5−hydroxybenzimidazo...
K13993: HSP20 family protein
K14081: methanol corrinoid protein
Proteins
K00239: succinate dehydrogenase / fumarate...
K00262: glutamate dehydrogenase (NADP+) ...
K00625: phosphate acetyltransferase [EC:2.3...
K01006: pyruvate, orthophosphate dikinase [...
K01812: glucuronate isomerase [EC:5.3.1.12]
K02358: elongation factor EF−Tu
K04043: molecular chaperone DnaK
K10112: multiple sugar transport system ATP...
K10540: methyl−galactoside transport syste...
UNGROUPED
Taxonomy
PCo 2 (6.7%)
PCo 1 (8.1%)
Transcripts
PCo 2 (9.2%)
PCo 1 (14.2%)
Proteins
PCo 2 (12.3%)
PCo 1 (30.8%)
Metabolites
PCo 2 (7.0%)
PCo 1 (8.8%)

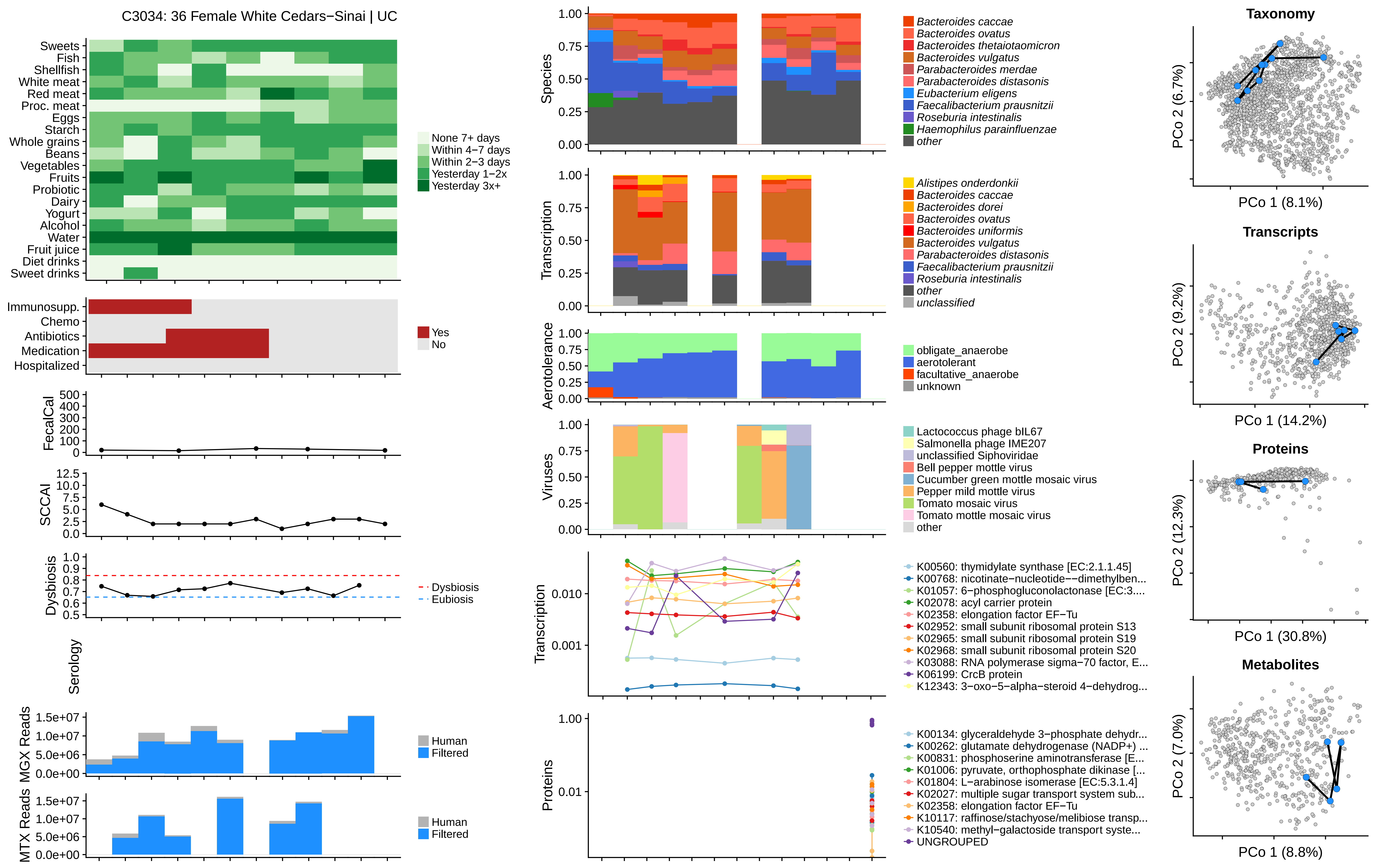

C3034: 36 Female White Cedars−Sinai | UC
Sweets
Fish
Shellfish
White meat
Red meat
Proc. meat
Eggs
Starch
Whole grains
Beans
Vegetables
Fruits
Probiotic
Dairy
Yogurt
Alcohol
Water
Fruit juice
Diet drinks
Sweet drinks
None 7+ days
Within 4−7 days
Within 2−3 days
Yesterday 1−2x
Yesterday 3x+
Immunosupp.
Chemo
Antibiotics
Medication
Hospitalized
Yes
No
FecalCal
SCCAI
Dysbiosis
Dysbiosis
Eubiosis
Serology
MGX Reads
Human
Filtered
MTX Reads
Human
Filtered
Species
Bacteroides caccae
Bacteroides ovatus
Bacteroides thetaiotaomicron
Bacteroides vulgatus
Parabacteroides merdae
Parabacteroides distasonis
Eubacterium eligens
Faecalibacterium prausnitzii
Roseburia intestinalis
Haemophilus parainfluenzae
other
Transcription
Alistipes onderdonkii
Bacteroides caccae
Bacteroides dorei
Bacteroides ovatus
Bacteroides uniformis
Bacteroides vulgatus
Parabacteroides distasonis
Faecalibacterium prausnitzii
Roseburia intestinalis
other
unclassified
Aerotolerance
obligate_anaerobe
aerotolerant
facultative_anaerobe
unknown
Viruses
Lactococcus phage bIL67
Salmonella phage IME207
unclassified Siphoviridae
Bell pepper mottle virus
Cucumber green mottle mosaic virus
Pepper mild mottle virus
Tomato mosaic virus
Tomato mottle mosaic virus
other
Transcription
K00560: thymidylate synthase [EC:2.1.1.45]
K00768: nicotinate−−dimethylben...
K01057: 6−phosphogluconolactonase [EC:3....
K02078: acyl carrier protein
K02358: elongation factor EF−Tu
K02952: small subunit ribosomal protein S13
K02965: small subunit ribosomal protein S19
K02968: small subunit ribosomal protein S20
K03088: RNA polymerase sigma−70 factor, E...
K06199: CrcB protein
K12343: 3−oxo−5−alpha−steroid 4−dehydrog...
Proteins
K00134: glyceraldehyde 3−phosphate dehydr...
K00262: glutamate dehydrogenase (NADP+) ...
K00831: phosphoserine aminotransferase [E...
K01006: pyruvate, orthophosphate dikinase [...
K01804: L−arabinose isomerase [EC:5.3.1.4]
K02027: multiple sugar transport system sub...
K02358: elongation factor EF−Tu
K10117: raffinose/stachyose/melibiose transp...
K10540: methyl−galactoside transport syste...
UNGROUPED
Taxonomy
PCo 1 (8.1%)
PCo 2 (6.7%)
Transcripts
PCo 1 (14.2%)
PCo 2 (9.2%)
Proteins
PCo 1 (30.8%)
PCo 2 (12.3%)
Metabolites
PCo 1 (8.8%)
PCo 2 (7.0%)

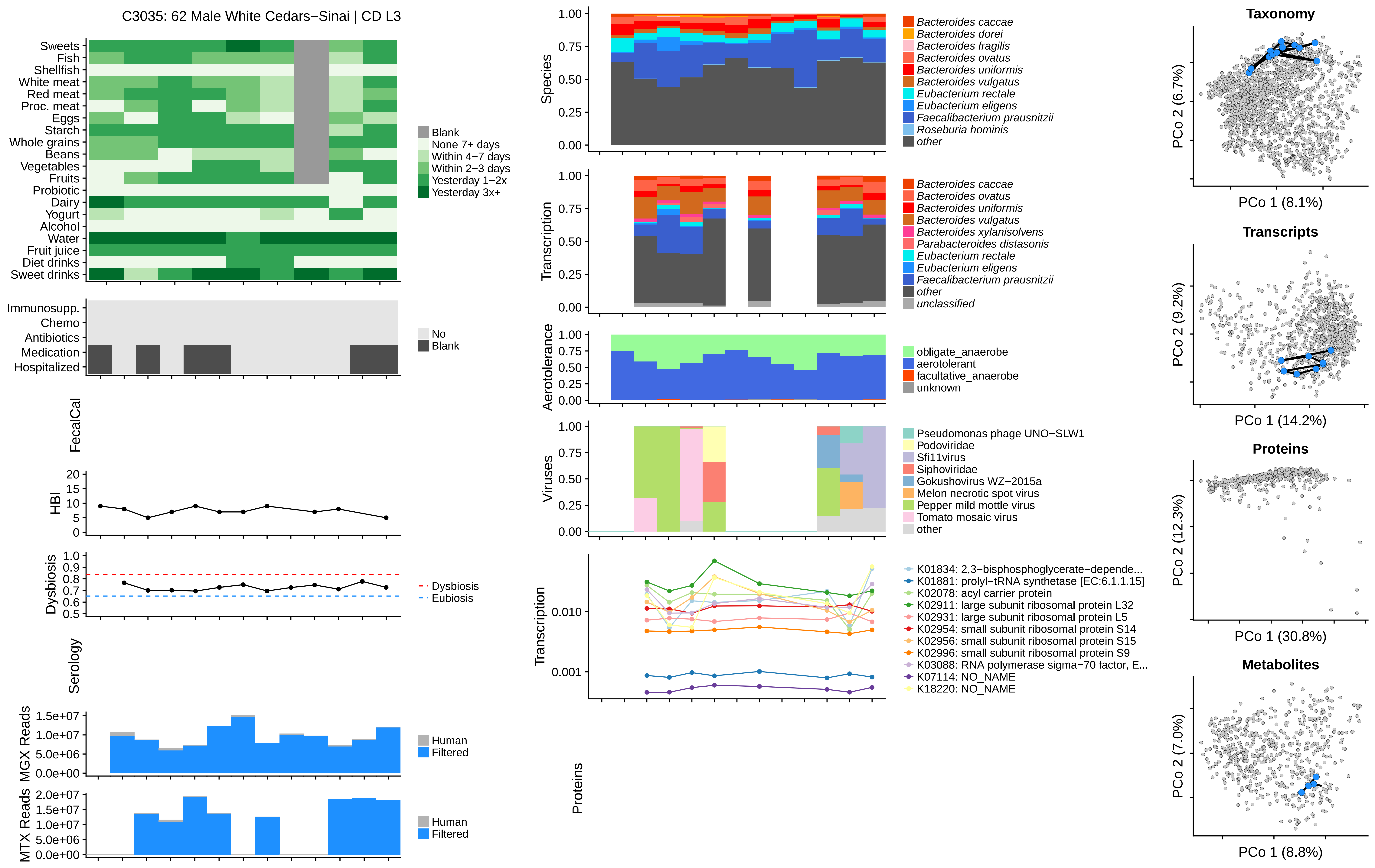
C3035: 62 Male White Cedars−Sinai | CD L3
Sweets
Fish
Shellfish
White meat
Red meat
Proc. meat
Eggs
Starch
Whole grains
Beans
Vegetables
Fruits
Probiotic
Dairy
Yogurt
Alcohol
Water
Fruit juice
Diet drinks
Sweet drinks
Blank
None 7+ days
Within 4−7 days
Within 2−3 days
Yesterday 1−2x
Yesterday 3x+
Immunosupp.
Chemo
Antibiotics
Medication
Hospitalized
No
Blank
FecalCal
HBI
Dysbiosis
Dysbiosis
Eubiosis
Serology
MGX Reads
Human
Filtered
MTX Reads
Human
Filtered
Species
Bacteroides caccae
Bacteroides dorei
Bacteroides fragilis
Bacteroides ovatus
Bacteroides uniformis
Bacteroides vulgatus
Eubacterium rectale
Eubacterium eligens
Faecalibacterium prausnitzii
Roseburia hominis
other
Transcription
Bacteroides caccae
Bacteroides ovatus
Bacteroides uniformis
Bacteroides vulgatus
Bacteroides xylanisolvens
Parabacteroides distasonis
Eubacterium rectale
Eubacterium eligens
Faecalibacterium prausnitzii
other
unclassified
Aerotolerance
obligate_anaerobe
aerotolerant
facultative_anaerobe
unknown
Viruses
Pseudomonas phage UNO−SLW1
Podoviridae
Sfi11virus
Siphoviridae
Gokushovirus WZ−2015a
Melon necrotic spot virus
Pepper mild mottle virus
Tomato mosaic virus
other
Transcription
K01834: 2,3−bisphosphoglycerate−depende...
K01881: prolyl−tRNA synthetase [EC:6.1.1.15]
K02078: acyl carrier protein
K02911: large subunit ribosomal protein L32
K02931: large subunit ribosomal protein L5
K02954: small subunit ribosomal protein S14
K02956: small subunit ribosomal protein S15
K02996: small subunit ribosomal protein S9
K03088: RNA polymerase sigma−70 factor, E...
K07114: NO_NAME
K18220: NO_NAME
Proteins
Taxonomy
PCo 1 (8.1%)
PCo 2 (6.7%)
Transcripts
PCo 1 (14.2%)
PCo 2 (9.2%)
Proteins
PCo 1 (30.8%)
PCo 2 (12.3%)
Metabolites
PCo 1 (8.8%)
PCo 2 (7.0%)

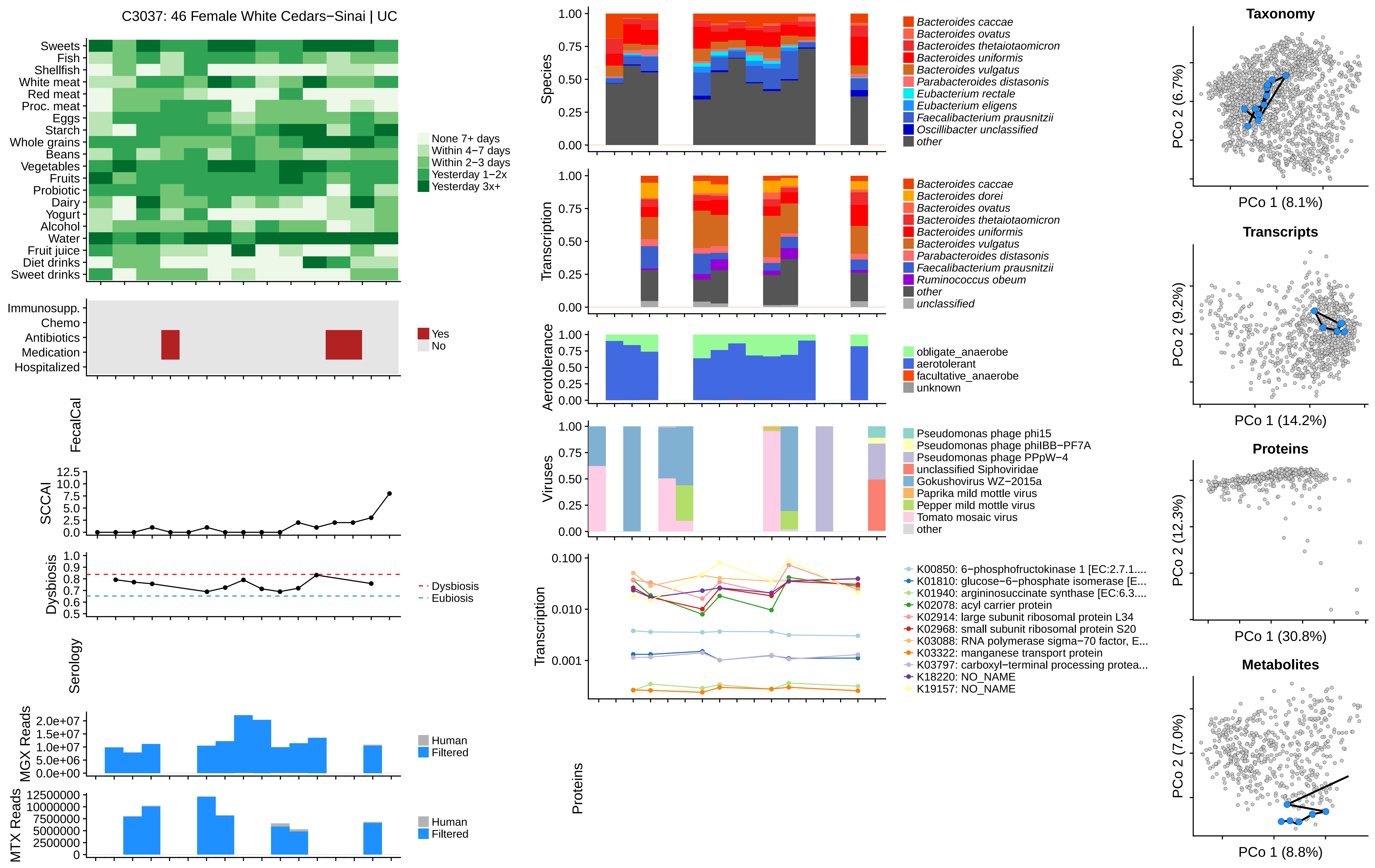

C3037: 46 Female White Cedars−Sinai | UC
Sweets
Fish
Shellfish
White meat
Red meat
Proc. meat
Eggs
Starch
Whole grains
Beans
Vegetables
Fruits
Probiotic
Dairy
Yogurt
Alcohol
Water
Fruit juice
Diet drinks
Sweet drinks
None 7+ days
Within 4−7 days
Within 2−3 days
Yesterday 1−2x
Yesterday 3x+
Immunosupp.
Chemo
Antibiotics
Medication
Hospitalized
Yes
No
FecalCal
SCCAI
Dysbiosis
Dysbiosis
Eubiosis
Serology
MGX Reads
Human
Filtered
MTX Reads
Human
Filtered
Species
Bacteroides caccae
Bacteroides ovatus
Bacteroides thetaiotaomicron
Bacteroides uniformis
Bacteroides vulgatus
Parabacteroides distasonis
Eubacterium rectale
Eubacterium eligens
Faecalibacterium prausnitzii
Oscillibacter unclassified
other
Transcription
Bacteroides caccae
Bacteroides dorei
Bacteroides ovatus
Bacteroides thetaiotaomicron
Bacteroides uniformis
Bacteroides vulgatus
Parabacteroides distasonis
Faecalibacterium prausnitzii
Ruminococcus obeum
other
unclassified
Aerotolerance
obligate_anaerobe
aerotolerant
facultative_anaerobe
unknown
Viruses
Pseudomonas phage phi15
Pseudomonas phage philBB−PF7A
Pseudomonas phage PPpW−4
unclassified Siphoviridae
Gokushovirus WZ−2015a
Paprika mild mottle virus
Pepper mild mottle virus
Tomato mosaic virus
other
Transcription
K00850: 6−phosphofructokinase 1 [EC:2.7.1....
K01810: glucose−6−phosphate isomerase [E...
K01940: argininosuccinate synthase [EC:6.3....
K02078: acyl carrier protein
K02914: large subunit ribosomal protein L34
K02968: small subunit ribosomal protein S20
K03088: RNA polymerase sigma−70 factor, E...
K03322: manganese transport protein
K03797: carboxyl−terminal processing protea...
K18220: NO_NAME
K19157: NO_NAME
Proteins
Taxonomy
PCo 1 (8.1%)
PCo 2 (6.7%)
Transcripts
PCo 1 (14.2%)
PCo 2 (9.2%)
Proteins
PCo 1 (30.8%)
PCo 2 (12.3%)
Metabolites
PCo 1 (8.8%)
PCo 2 (7.0%)

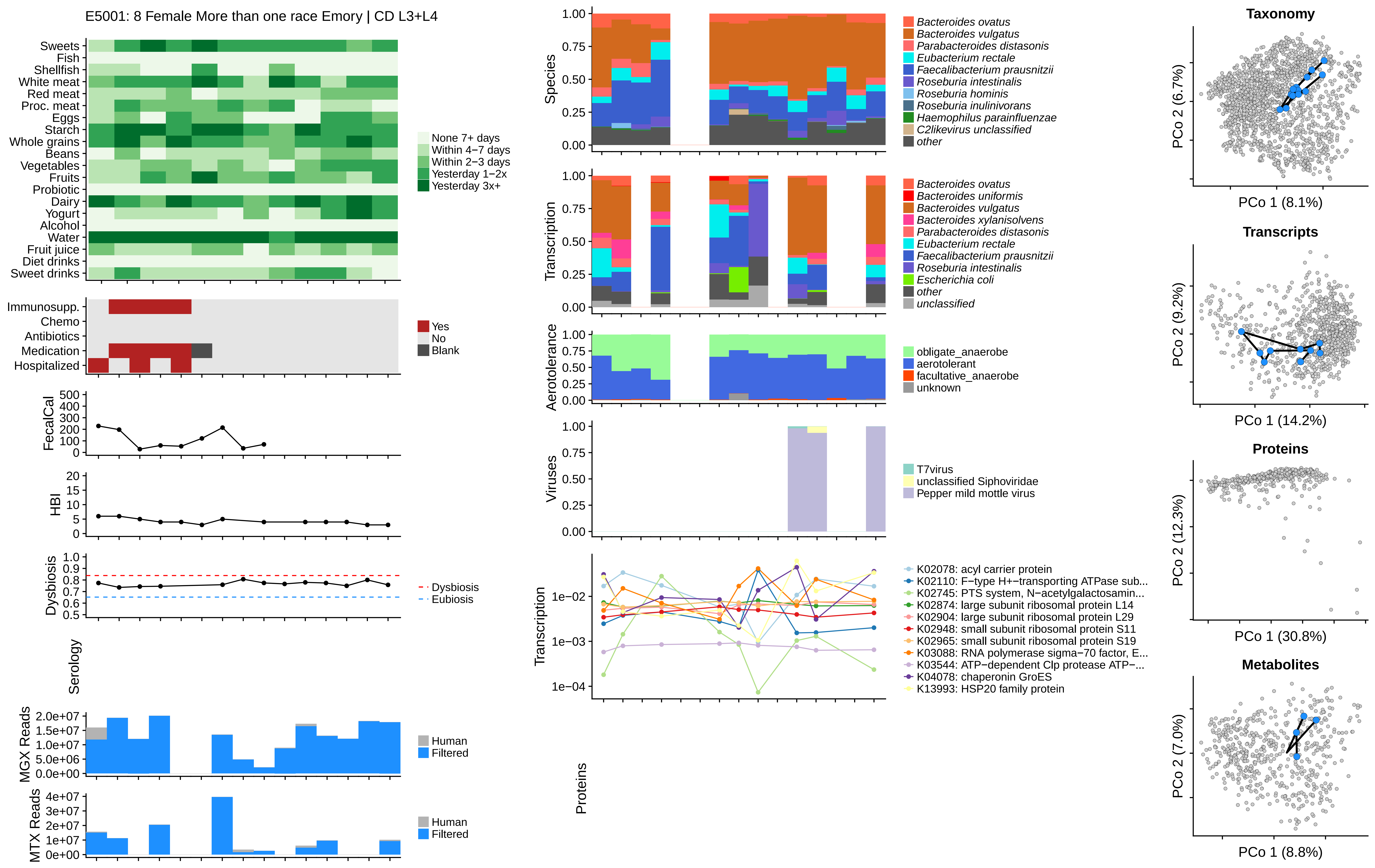

E5001: 8 Female More than one race Emory | CD L3+L4
Sweets
Fish
Shellfish
White meat
Red meat
Proc. meat
Eggs
Starch
Whole grains
Beans
Vegetables
Fruits
Probiotic
Dairy
Yogurt
Alcohol
Water
Fruit juice
Diet drinks
Sweet drinks
None 7+ days
Within 4−7 days
Within 2−3 days
Yesterday 1−2x
Yesterday 3x+
Immunosupp.
Chemo
Antibiotics
Medication
Hospitalized
Yes
No
Blank
FecalCal
HBI
Dysbiosis
Dysbiosis
Eubiosis
Serology
MGX Reads
Human
Filtered
MTX Reads
Human
Filtered
Species
Bacteroides ovatus
Bacteroides vulgatus
Parabacteroides distasonis
Eubacterium rectale
Faecalibacterium prausnitzii
Roseburia intestinalis
Roseburia hominis
Roseburia inulinivorans
Haemophilus parainfluenzae
C2likevirus unclassified
other
Transcription
Bacteroides ovatus
Bacteroides uniformis
Bacteroides vulgatus
Bacteroides xylanisolvens
Parabacteroides distasonis
Eubacterium rectale
Faecalibacterium prausnitzii
Roseburia intestinalis
Escherichia coli
other
unclassified
Aerotolerance
obligate_anaerobe
aerotolerant
facultative_anaerobe
unknown
Viruses
T7virus
unclassified Siphoviridae
Pepper mild mottle virus
Transcription
K02078: acyl carrier protein
K02110: F−type H+−transporting ATPase sub...
K02745: PTS system, N−acetylgalactosamin...
K02874: large subunit ribosomal protein L14
K02904: large subunit ribosomal protein L29
K02948: small subunit ribosomal protein S11
K02965: small subunit ribosomal protein S19
K03088: RNA polymerase sigma−70 factor, E...
K03544: ATP−dependent Clp protease ATP−...
K04078: chaperonin GroES
K13993: HSP20 family protein
Proteins
Taxonomy
PCo 1 (8.1%)
PCo 2 (6.7%)
Transcripts
PCo 1 (14.2%)
PCo 2 (9.2%)
Proteins
PCo 1 (30.8%)
PCo 2 (12.3%)
Metabolites
PCo 1 (8.8%)
PCo 2 (7.0%)

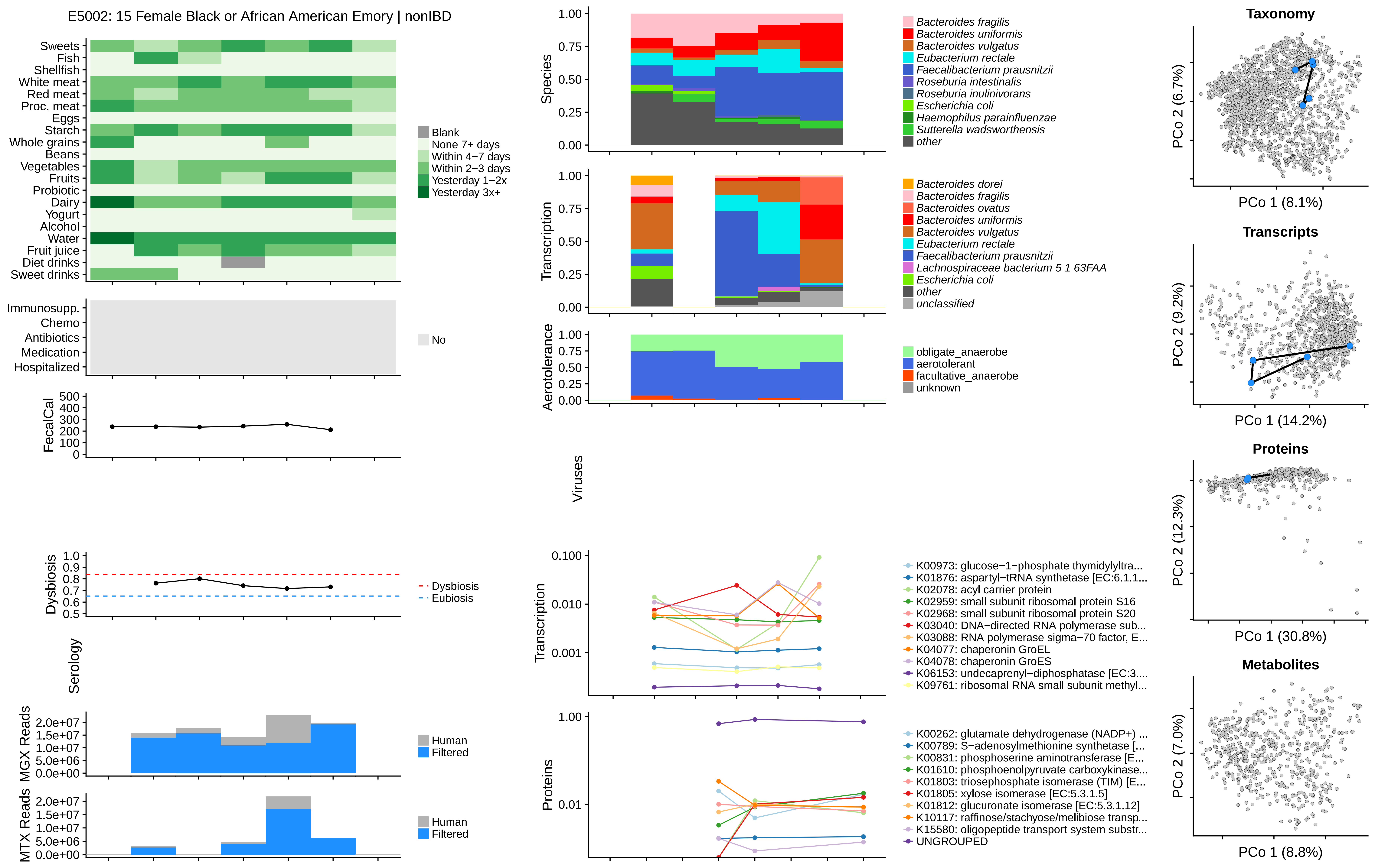

E5002: 15 Female Black or African American Emory | nonIBD
Sweets
Fish
Shellfish
White meat
Red meat
Proc. meat
Eggs
Starch
Whole grains
Beans
Vegetables
Fruits
Probiotic
Dairy
Yogurt
Alcohol
Water
Fruit juice
Diet drinks
Sweet drinks
Blank
None 7+ days
Within 4−7 days
Within 2−3 days
Yesterday 1−2x
Yesterday 3x+
Immunosupp.
Chemo
Antibiotics
Medication
Hospitalized
No
FecalCal
Dysbiosis
Dysbiosis
Eubiosis
Serology
MGX Reads
Human
Filtered
MTX Reads
Human
Filtered
Species
Bacteroides fragilis
Bacteroides uniformis
Bacteroides vulgatus
Eubacterium rectale
Faecalibacterium prausnitzii
Roseburia intestinalis
Roseburia inulinivorans
Escherichia coli
Haemophilus parainfluenzae
Sutterella wadsworthensis
other
Transcription
Bacteroides dorei
Bacteroides fragilis
Bacteroides ovatus
Bacteroides uniformis
Bacteroides vulgatus
Eubacterium rectale
Faecalibacterium prausnitzii
Lachnospiraceae bacterium 5 1 63FAA
Escherichia coli
other
unclassified
Aerotolerance
obligate_anaerobe
aerotolerant
facultative_anaerobe
unknown
Viruses
Transcription
K00973: glucose−1−phosphate thymidylyltra...
K01876: aspartyl−tRNA synthetase [EC:6.1.1...
K02078: acyl carrier protein
K02959: small subunit ribosomal protein S16
K02968: small subunit ribosomal protein S20
K03040: DNA−directed RNA polymerase sub...
K03088: RNA polymerase sigma−70 factor, E...
K04077: chaperonin GroEL
K04078: chaperonin GroES
K06153: undecaprenyl−diphosphatase [EC:3....
K09761: ribosomal RNA small subunit methyl...
Proteins
K00262: glutamate dehydrogenase (NADP+) ...
K00789: S−adenosylmethionine synthetase [...
K00831: phosphoserine aminotransferase [E...
K01610: phosphoenolpyruvate carboxykinase...
K01803: triosephosphate isomerase (TIM) [E...
K01805: xylose isomerase [EC:5.3.1.5]
K01812: glucuronate isomerase [EC:5.3.1.12]
K10117: raffinose/stachyose/melibiose transp...
K15580: oligopeptide transport system substr...
UNGROUPED
Taxonomy
PCo 1 (8.1%)
PCo 2 (6.7%)
Transcripts
PCo 1 (14.2%)
PCo 2 (9.2%)
Proteins
PCo 1 (30.8%)
PCo 2 (12.3%)
Metabolites
PCo 1 (8.8%)
PCo 2 (7.0%)

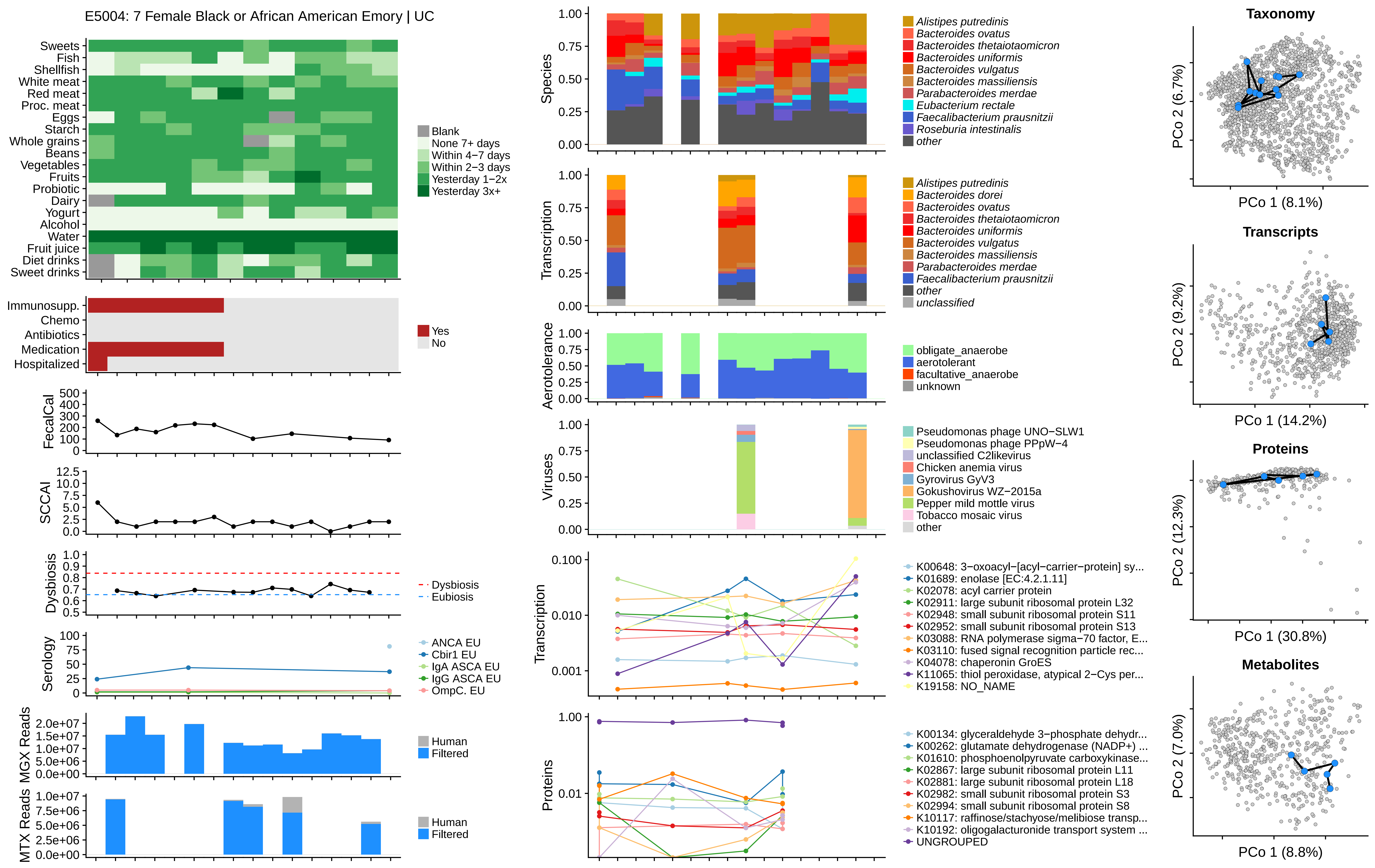
E5004: 7 Female Black or African American Emory | UC
Sweets
Fish
Shellfish
White meat
Red meat
Proc. meat
Eggs
Starch
Whole grains
Beans
Vegetables
Fruits
Probiotic
Dairy
Yogurt
Alcohol
Water
Fruit juice
Diet drinks
Sweet drinks
Blank
None 7+ days
Within 4−7 days
Within 2−3 days
Yesterday 1−2x
Yesterday 3x+
Immunosupp.
Chemo
Antibiotics
Medication
Hospitalized
Yes
No
FecalCal
SCCAI
Dysbiosis
Dysbiosis
Eubiosis
Serology
ANCA EU
Cbir1 EU
IgA ASCA EU
IgG ASCA EU
OmpC. EU
MGX Reads
Human
Filtered
MTX Reads
Human
Filtered
Species
Alistipes putredinis
Bacteroides ovatus
Bacteroides thetaiotaomicron
Bacteroides uniformis
Bacteroides vulgatus
Bacteroides massiliensis
Parabacteroides merdae
Eubacterium rectale
Faecalibacterium prausnitzii
Roseburia intestinalis
other
Transcription
Alistipes putredinis
Bacteroides dorei
Bacteroides ovatus
Bacteroides thetaiotaomicron
Bacteroides uniformis
Bacteroides vulgatus
Bacteroides massiliensis
Parabacteroides merdae
Faecalibacterium prausnitzii
other
unclassified
Aerotolerance
obligate_anaerobe
aerotolerant
facultative_anaerobe
unknown
Viruses
Pseudomonas phage UNO−SLW1
Pseudomonas phage PPpW−4
unclassified C2likevirus
Chicken anemia virus
Gyrovirus GyV3
Gokushovirus WZ−2015a
Pepper mild mottle virus
Tobacco mosaic virus
other
Transcription
K00648: 3−oxoacyl−[acyl−carrier−protein] sy...
K01689: enolase [EC:4.2.1.11]
K02078: acyl carrier protein
K02911: large subunit ribosomal protein L32
K02948: small subunit ribosomal protein S11
K02952: small subunit ribosomal protein S13
K03088: RNA polymerase sigma−70 factor, E...
K03110: fused signal recognition particle rec...
K04078: chaperonin GroES
K11065: thiol peroxidase, atypical 2−Cys per...
K19158: NO_NAME
Proteins
K00134: glyceraldehyde 3−phosphate dehydr...
K00262: glutamate dehydrogenase (NADP+) ...
K01610: phosphoenolpyruvate carboxykinase...
K02867: large subunit ribosomal protein L11
K02881: large subunit ribosomal protein L18
K02982: small subunit ribosomal protein S3
K02994: small subunit ribosomal protein S8
K10117: raffinose/stachyose/melibiose transp...
K10192: oligogalacturonide transport system ...
UNGROUPED
Taxonomy
PCo 1 (8.1%)
PCo 2 (6.7%)
Transcripts
PCo 1 (14.2%)
PCo 2 (9.2%)
Proteins
PCo 1 (30.8%)
PCo 2 (12.3%)
Metabolites
PCo 1 (8.8%)
PCo 2 (7.0%)

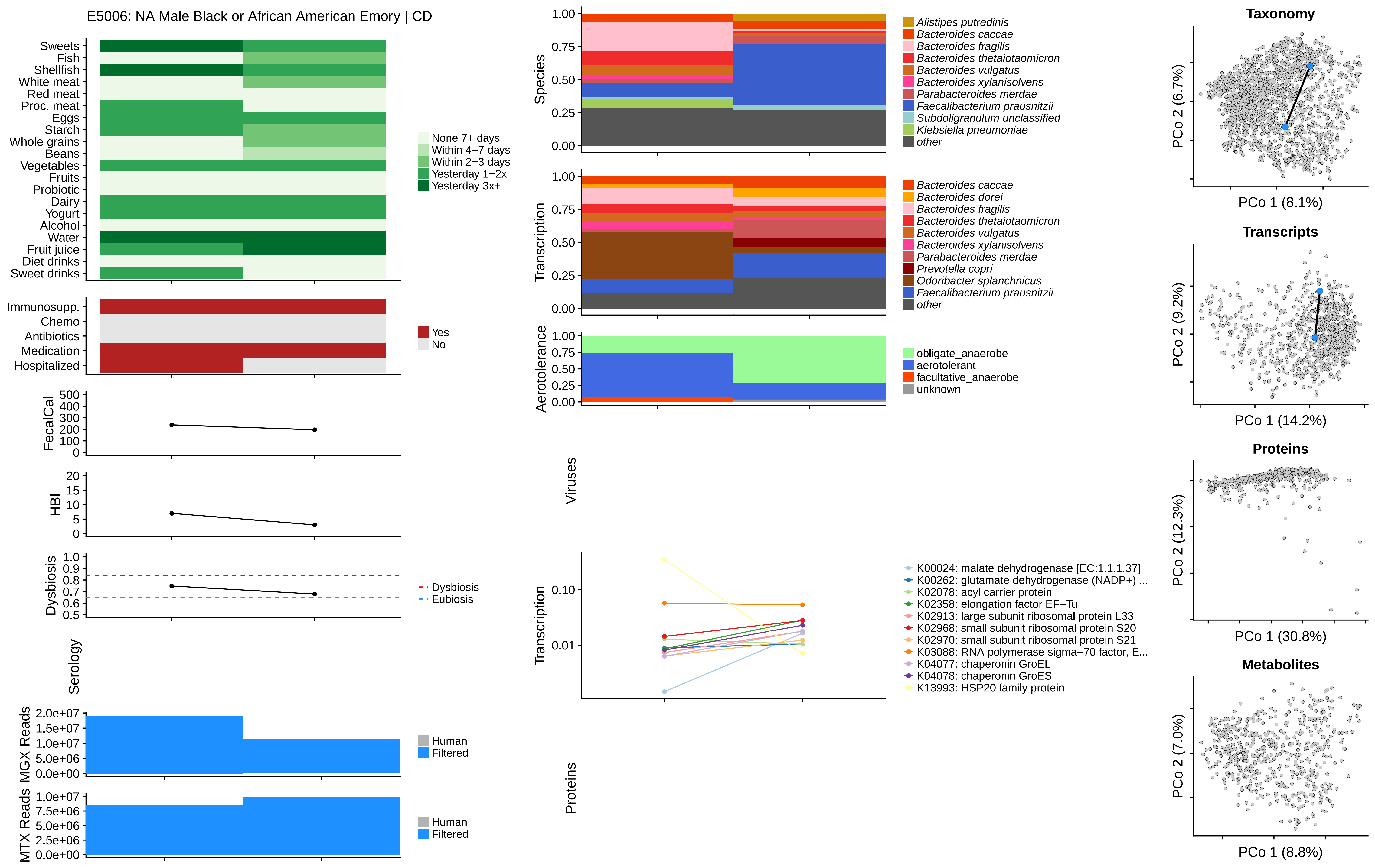

E5006: NA Male Black or African American Emory | CD
Sweets
Fish
Shellfish
White meat
Red meat
Proc. meat
Eggs
Starch
Whole grains
Beans
Vegetables
Fruits
Probiotic
Dairy
Yogurt
Alcohol
Water
Fruit juice
Diet drinks
Sweet drinks
None 7+ days
Within 4−7 days
Within 2−3 days
Yesterday 1−2x
Yesterday 3x+
Immunosupp.
Chemo
Antibiotics
Medication
Hospitalized
Yes
No
FecalCal
0
100
200
300
400
500
HBI
0
5
10
15
20
Dysbiosis
0.5
0.6
0.7
0.8
0.9
1.0
Dysbiosis
Eubiosis
Serology
MGX Reads
0.0e+00
5.0e+06
1.0e+07
1.5e+07
2.0e+07
Human
Filtered
MTX Reads
0.0e+00
2.5e+06
5.0e+06
7.5e+06
1.0e+07
Human
Filtered
Species
0.00
0.25
0.50
0.75
1.00
Alistipes putredinis
Bacteroides caccae
Bacteroides fragilis
Bacteroides thetaiotaomicron
Bacteroides vulgatus
Bacteroides xylanisolvens
Parabacteroides merdae
Faecalibacterium prausnitzii
Subdoligranulum unclassified
Klebsiella pneumoniae
other
Transcription
Bacteroides caccae
Bacteroides dorei
Bacteroides fragilis
Bacteroides thetaiotaomicron
Bacteroides vulgatus
Bacteroides xylanisolvens
Parabacteroides merdae
Prevotella copri
Odoribacter splanchnicus
Faecalibacterium prausnitzii
other
Aerotolerance
obligate_anaerobe
aerotolerant
facultative_anaerobe
unknown
Viruses
Transcription
0.01
0.10
K00024: malate dehydrogenase [EC:1.1.1.37]
K00262: glutamate dehydrogenase (NADP+) ...
K02078: acyl carrier protein
K02358: elongation factor EF−Tu
K02913: large subunit ribosomal protein L33
K02968: small subunit ribosomal protein S20
K02970: small subunit ribosomal protein S21
K03088: RNA polymerase sigma−70 factor, E...
K04077: chaperonin GroEL
K04078: chaperonin GroES
K13993: HSP20 family protein
Proteins
Taxonomy
PCo 1 (8.1%)
PCo 2 (6.7%)
Transcripts
PCo 1 (14.2%)
PCo 2 (9.2%)
Proteins
PCo 1 (30.8%)
PCo 2 (12.3%)
Metabolites
PCo 1 (8.8%)
PCo 2 (7.0%)

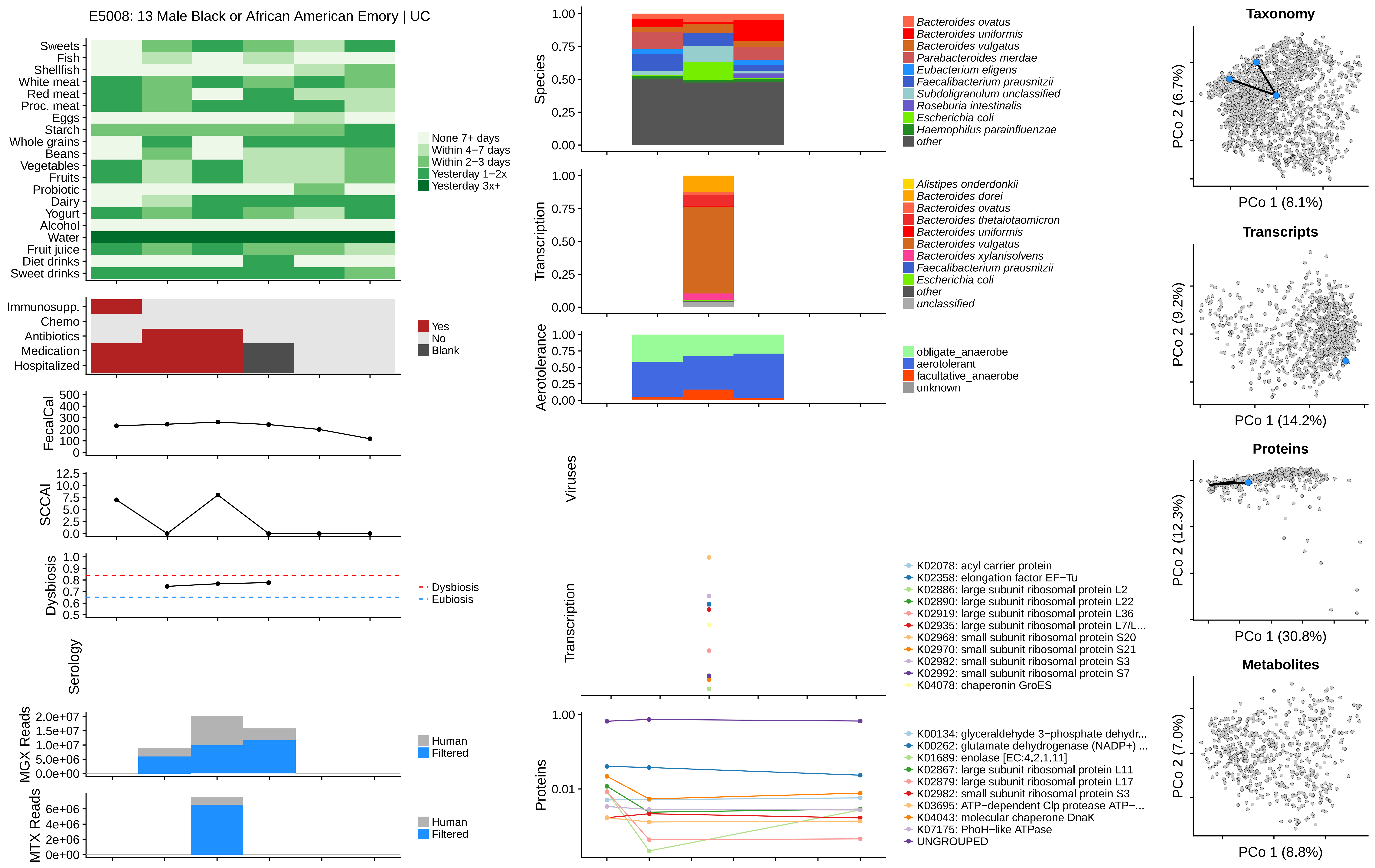

E5008: 13 Male Black or African American Emory | UC
Sweets
Fish
Shellfish
White meat
Red meat
Proc. meat
Eggs
Starch
Whole grains
Beans
Vegetables
Fruits
Probiotic
Dairy
Yogurt
Alcohol
Water
Fruit juice
Diet drinks
Sweet drinks
None 7+ days
Within 4−7 days
Within 2−3 days
Yesterday 1−2x
Yesterday 3x+
Immunosupp.
Chemo
Antibiotics
Medication
Hospitalized
Yes
No
Blank
FecalCal
SCCAI
Dysbiosis
Dysbiosis
Eubiosis
Serology
MGX Reads
Human
Filtered
MTX Reads
Human
Filtered
Species
Bacteroides ovatus
Bacteroides uniformis
Bacteroides vulgatus
Parabacteroides merdae
Eubacterium eligens
Faecalibacterium prausnitzii
Subdoligranulum unclassified
Roseburia intestinalis
Escherichia coli
Haemophilus parainfluenzae
other
Transcription
Alistipes onderdonkii
Bacteroides dorei
Bacteroides ovatus
Bacteroides thetaiotaomicron
Bacteroides uniformis
Bacteroides vulgatus
Bacteroides xylanisolvens
Faecalibacterium prausnitzii
Escherichia coli
other
unclassified
Aerotolerance
obligate_anaerobe
aerotolerant
facultative_anaerobe
unknown
Viruses
Transcription
K02078: acyl carrier protein
K02358: elongation factor EF−Tu
K02886: large subunit ribosomal protein L2
K02890: large subunit ribosomal protein L22
K02919: large subunit ribosomal protein L36
K02935: large subunit ribosomal protein L7/L...
K02968: small subunit ribosomal protein S20
K02970: small subunit ribosomal protein S21
K02982: small subunit ribosomal protein S3
K02992: small subunit ribosomal protein S7
K04078: chaperonin GroES
Proteins
K00134: glyceraldehyde 3−phosphate dehydr...
K00262: glutamate dehydrogenase (NADP+) ...
K01689: enolase [EC:4.2.1.11]
K02867: large subunit ribosomal protein L11
K02879: large subunit ribosomal protein L17
K02982: small subunit ribosomal protein S3
K03695: ATP−dependent Clp protease ATP−...
K04043: molecular chaperone DnaK
K07175: PhoH−like ATPase
UNGROUPED
Taxonomy
PCo 1 (8.1%)
PCo 2 (6.7%)
Transcripts
PCo 1 (14.2%)
PCo 2 (9.2%)
Proteins
PCo 1 (30.8%)
PCo 2 (12.3%)
Metabolites
PCo 1 (8.8%)
PCo 2 (7.0%)

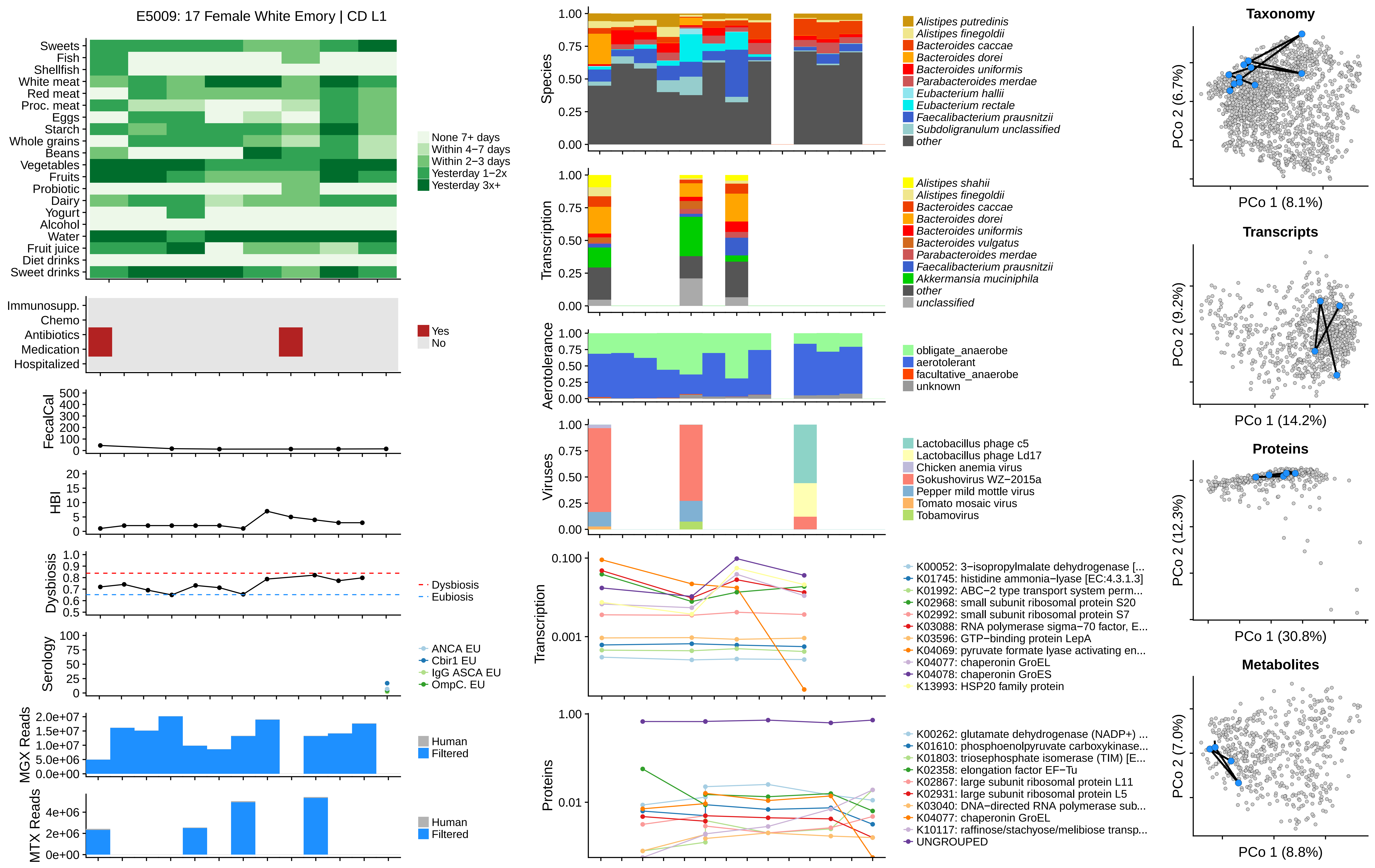
E5009: 17 Female White Emory | CD L1
Sweets
Fish
Shellfish
White meat
Red meat
Proc. meat
Eggs
Starch
Whole grains
Beans
Vegetables
Fruits
Probiotic
Dairy
Yogurt
Alcohol
Water
Fruit juice
Diet drinks
Sweet drinks
None 7+ days
Within 4−7 days
Within 2−3 days
Yesterday 1−2x
Yesterday 3x+
Immunosupp.
Chemo
Antibiotics
Medication
Hospitalized
Yes
No
FecalCal
HBI
Dysbiosis
Dysbiosis
Eubiosis
Serology
ANCA EU
Cbir1 EU
IgG ASCA EU
OmpC. EU
MGX Reads
Human
Filtered
MTX Reads
Human
Filtered
Species
Alistipes putredinis
Alistipes finegoldii
Bacteroides caccae
Bacteroides dorei
Bacteroides uniformis
Parabacteroides merdae
Eubacterium hallii
Eubacterium rectale
Faecalibacterium prausnitzii
Subdoligranulum unclassified
other
Transcription
Alistipes shahii
Alistipes finegoldii
Bacteroides caccae
Bacteroides dorei
Bacteroides uniformis
Bacteroides vulgatus
Parabacteroides merdae
Faecalibacterium prausnitzii
Akkermansia muciniphila
other
unclassified
Aerotolerance
obligate_anaerobe
aerotolerant
facultative_anaerobe
unknown
Viruses
Lactobacillus phage c5
Lactobacillus phage Ld17
Chicken anemia virus
Gokushovirus WZ−2015a
Pepper mild mottle virus
Tomato mosaic virus
Tobamovirus
Transcription
K00052: 3−isopropylmalate dehydrogenase [...
K01745: histidine ammonia−lyase [EC:4.3.1.3]
K01992: ABC−2 type transport system perm...
K02968: small subunit ribosomal protein S20
K02992: small subunit ribosomal protein S7
K03088: RNA polymerase sigma−70 factor, E...
K03596: GTP−binding protein LepA
K04069: pyruvate formate lyase activating en...
K04077: chaperonin GroEL
K04078: chaperonin GroES
K13993: HSP20 family protein
Proteins
K00262: glutamate dehydrogenase (NADP+) ...
K01610: phosphoenolpyruvate carboxykinase...
K01803: triosephosphate isomerase (TIM) [E...
K02358: elongation factor EF−Tu
K02867: large subunit ribosomal protein L11
K02931: large subunit ribosomal protein L5
K03040: DNA−directed RNA polymerase sub...
K04077: chaperonin GroEL
K10117: raffinose/stachyose/melibiose transp...
UNGROUPED
Taxonomy
PCo 1 (8.1%)
PCo 2 (6.7%)
Transcripts
PCo 1 (14.2%)
PCo 2 (9.2%)
Proteins
PCo 1 (30.8%)
PCo 2 (12.3%)
Metabolites
PCo 1 (8.8%)
PCo 2 (7.0%)

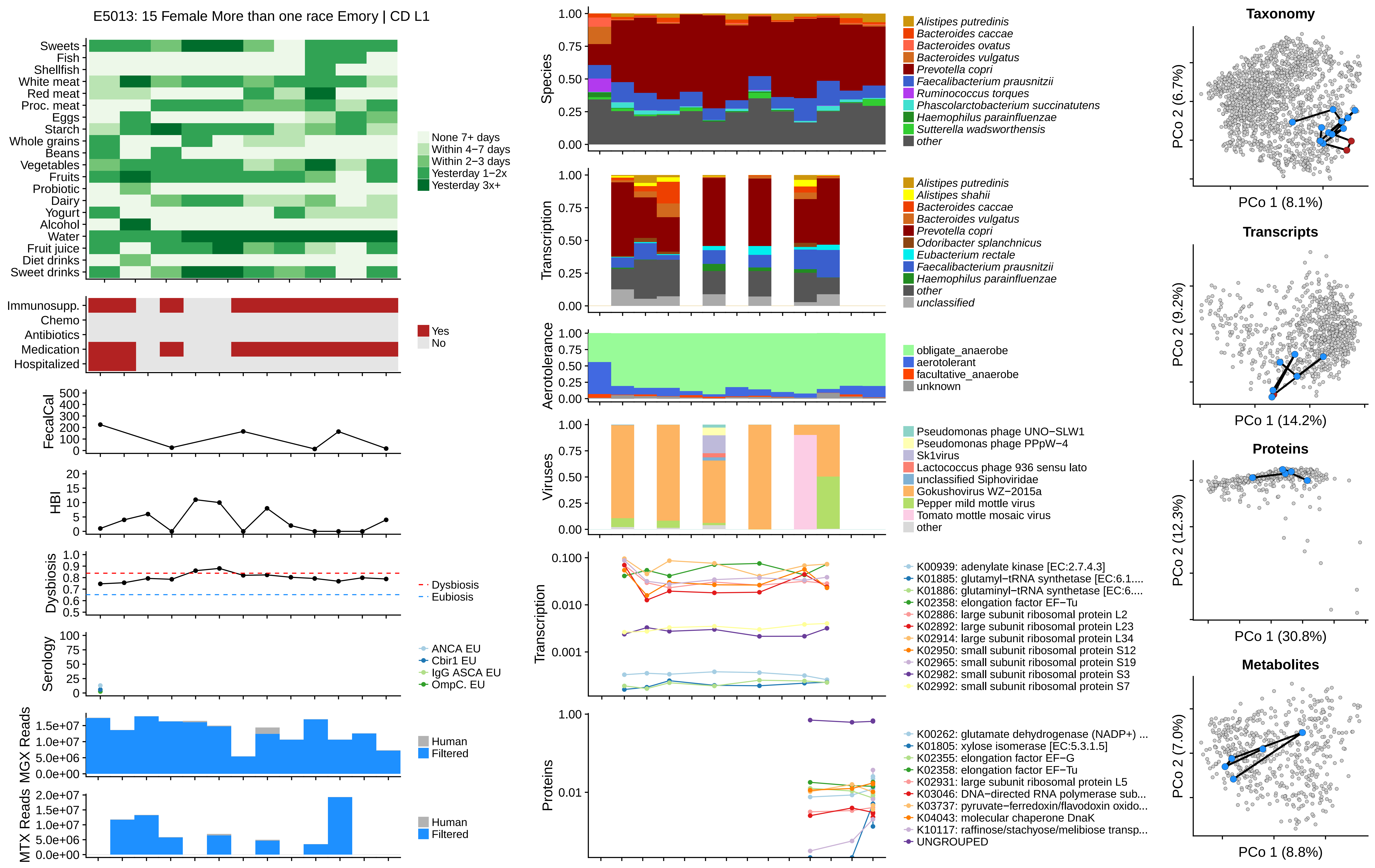
E5013: 15 Female More than one race Emory | CD L1
Sweets
Fish
Shellfish
White meat
Red meat
Proc. meat
Eggs
Starch
Whole grains
Beans
Vegetables
Fruits
Probiotic
Dairy
Yogurt
Alcohol
Water
Fruit juice
Diet drinks
Sweet drinks
None 7+ days
Within 4−7 days
Within 2−3 days
Yesterday 1−2x
Yesterday 3x+
Immunosupp.
Chemo
Antibiotics
Medication
Hospitalized
Yes
No
FecalCal
HBI
Dysbiosis
Dysbiosis
Eubiosis
Serology
ANCA EU
Cbir1 EU
IgG ASCA EU
OmpC. EU
MGX Reads
Human
Filtered
MTX Reads
Human
Filtered
Species
Alistipes putredinis
Bacteroides caccae
Bacteroides ovatus
Bacteroides vulgatus
Prevotella copri
Faecalibacterium prausnitzii
Ruminococcus torques
Phascolarctobacterium succinatutens
Haemophilus parainfluenzae
Sutterella wadsworthensis
other
Transcription
Alistipes putredinis
Alistipes shahii
Bacteroides caccae
Bacteroides vulgatus
Prevotella copri
Odoribacter splanchnicus
Eubacterium rectale
Faecalibacterium prausnitzii
Haemophilus parainfluenzae
other
unclassified
Aerotolerance
obligate_anaerobe
aerotolerant
facultative_anaerobe
unknown
Viruses
Pseudomonas phage UNO−SLW1
Pseudomonas phage PPpW−4
Sk1virus
Lactococcus phage 936 sensu lato
unclassified Siphoviridae
Gokushovirus WZ−2015a
Pepper mild mottle virus
Tomato mottle mosaic virus
other
Transcription
K00939: adenylate kinase [EC:2.7.4.3]
K01885: glutamyl−tRNA synthetase [EC:6.1....
K01886: glutaminyl−tRNA synthetase [EC:6....
K02358: elongation factor EF−Tu
K02886: large subunit ribosomal protein L2
K02892: large subunit ribosomal protein L23
K02914: large subunit ribosomal protein L34
K02950: small subunit ribosomal protein S12
K02965: small subunit ribosomal protein S19
K02982: small subunit ribosomal protein S3
K02992: small subunit ribosomal protein S7
Proteins
K00262: glutamate dehydrogenase (NADP+) ...
K01805: xylose isomerase [EC:5.3.1.5]
K02355: elongation factor EF−G
K02358: elongation factor EF−Tu
K02931: large subunit ribosomal protein L5
K03046: DNA−directed RNA polymerase sub...
K03737: pyruvate−ferredoxin/flavodoxin oxido...
K04043: molecular chaperone DnaK
K10117: raffinose/stachyose/melibiose transp...
UNGROUPED
Taxonomy
PCo 1 (8.1%)
PCo 2 (6.7%)
Transcripts
PCo 1 (14.2%)
PCo 2 (9.2%)
Proteins
PCo 1 (30.8%)
PCo 2 (12.3%)
Metabolites
PCo 1 (8.8%)
PCo 2 (7.0%)

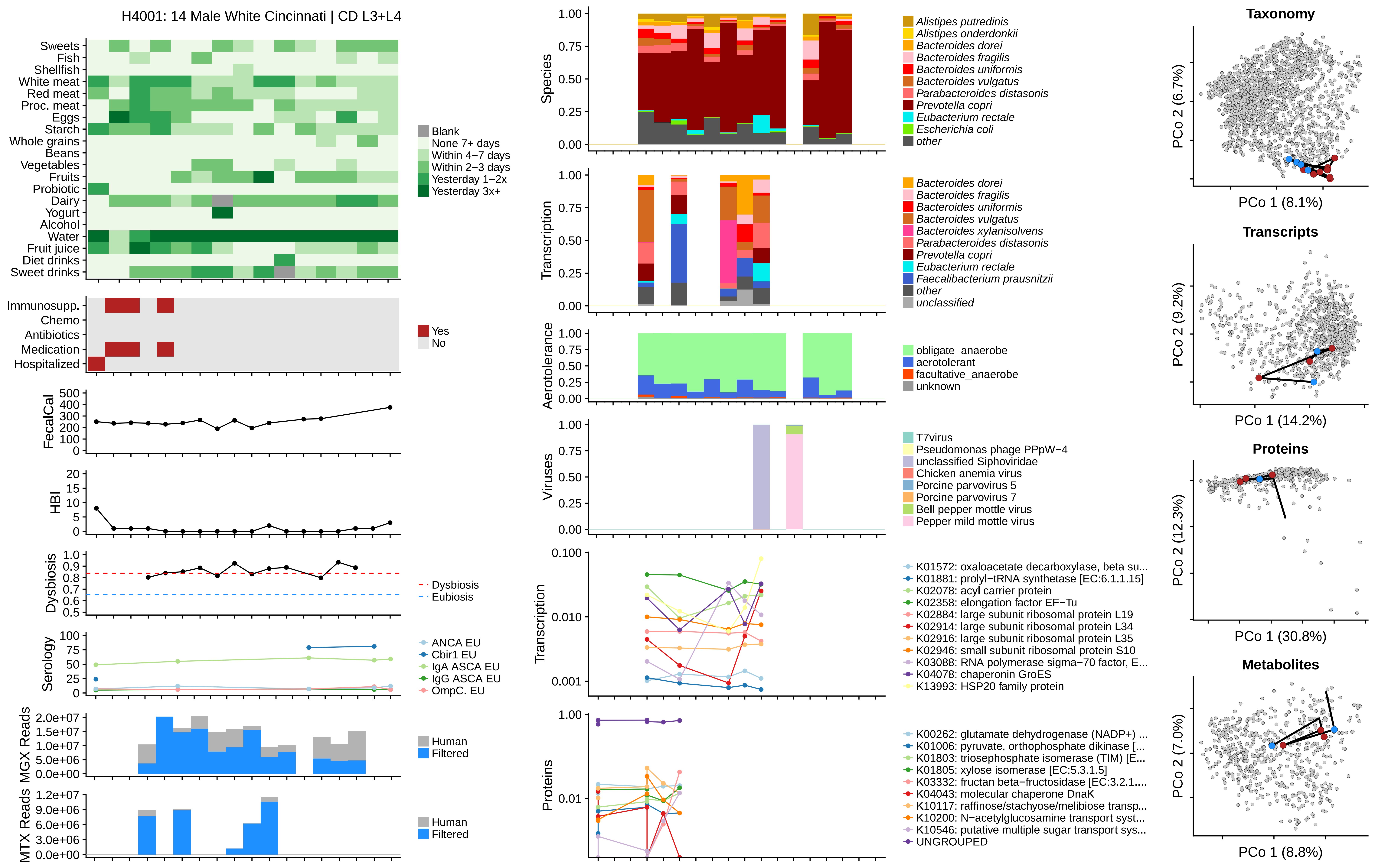

H4001: 14 Male White Cincinnati | CD L3+L4
Sweets
Fish
Shellfish
White meat
Red meat
Proc. meat
Eggs
Starch
Whole grains
Beans
Vegetables
Fruits
Probiotic
Dairy
Yogurt
Alcohol
Water
Fruit juice
Diet drinks
Sweet drinks
Blank
None 7+ days
Within 4−7 days
Within 2−3 days
Yesterday 1−2x
Yesterday 3x+
Immunosupp.
Chemo
Antibiotics
Medication
Hospitalized
Yes
No
FecalCal
HBI
Dysbiosis
Dysbiosis
Eubiosis
Serology
ANCA EU
Cbir1 EU
IgA ASCA EU
IgG ASCA EU
OmpC. EU
MGX Reads
Human
Filtered
MTX Reads
Human
Filtered
Species
Alistipes putredinis
Alistipes onderdonkii
Bacteroides dorei
Bacteroides fragilis
Bacteroides uniformis
Bacteroides vulgatus
Parabacteroides distasonis
Prevotella copri
Eubacterium rectale
Escherichia coli
other
Transcription
Bacteroides dorei
Bacteroides fragilis
Bacteroides uniformis
Bacteroides vulgatus
Bacteroides xylanisolvens
Parabacteroides distasonis
Prevotella copri
Eubacterium rectale
Faecalibacterium prausnitzii
other
unclassified
Aerotolerance
obligate_anaerobe
aerotolerant
facultative_anaerobe
unknown
Viruses
T7virus
Pseudomonas phage PPpW−4
unclassified Siphoviridae
Chicken anemia virus
Porcine parvovirus 5
Porcine parvovirus 7
Bell pepper mottle virus
Pepper mild mottle virus
Transcription
K01572: oxaloacetate decarboxylase, beta su...
K01881: prolyl−tRNA synthetase [EC:6.1.1.15]
K02078: acyl carrier protein
K02358: elongation factor EF−Tu
K02884: large subunit ribosomal protein L19
K02914: large subunit ribosomal protein L34
K02916: large subunit ribosomal protein L35
K02946: small subunit ribosomal protein S10
K03088: RNA polymerase sigma−70 factor, E...
K04078: chaperonin GroES
K13993: HSP20 family protein
Proteins
K00262: glutamate dehydrogenase (NADP+) ...
K01006: pyruvate, orthophosphate dikinase [...
K01803: triosephosphate isomerase (TIM) [E...
K01805: xylose isomerase [EC:5.3.1.5]
K03332: fructan beta−fructosidase [EC:3.2.1....
K04043: molecular chaperone DnaK
K10117: raffinose/stachyose/melibiose transp...
K10200: N−acetylglucosamine transport syst...
K10546: putative multiple sugar transport sys...
UNGROUPED
Taxonomy
PCo 1 (8.1%)
PCo 2 (6.7%)
Transcripts
PCo 1 (14.2%)
PCo 2 (9.2%)
Proteins
PCo 1 (30.8%)
PCo 2 (12.3%)
Metabolites
PCo 1 (8.8%)
PCo 2 (7.0%)

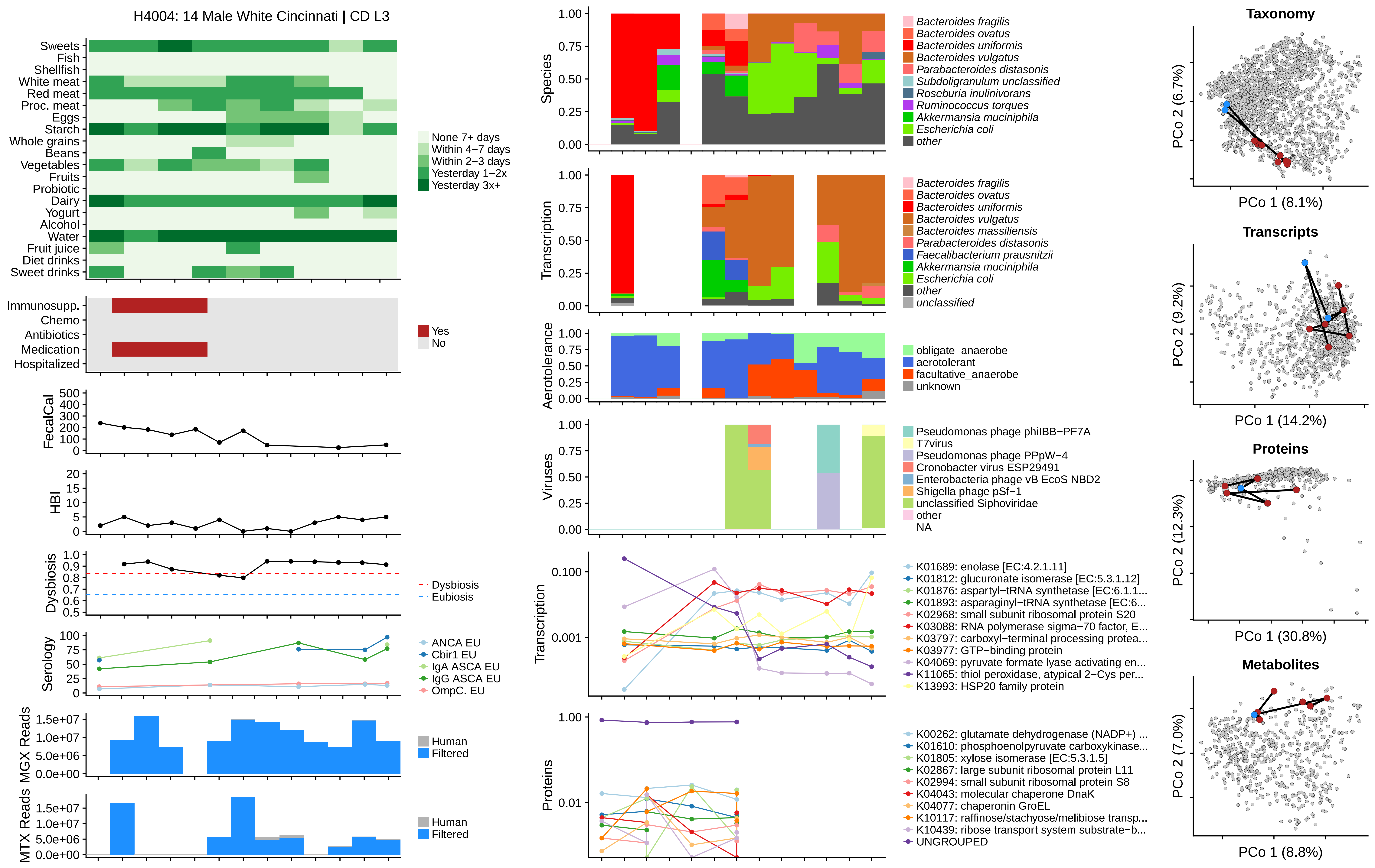

H4004: 14 Male White Cincinnati | CD L3
Sweets
Fish
Shellfish
White meat
Red meat
Proc. meat
Eggs
Starch
Whole grains
Beans
Vegetables
Fruits
Probiotic
Dairy
Yogurt
Alcohol
Water
Fruit juice
Diet drinks
Sweet drinks
None 7+ days
Within 4−7 days
Within 2−3 days
Yesterday 1−2x
Yesterday 3x+
Immunosupp.
Chemo
Antibiotics
Medication
Hospitalized
Yes
No
FecalCal
HBI
Dysbiosis
Dysbiosis
Eubiosis
Serology
ANCA EU
Cbir1 EU
IgA ASCA EU
IgG ASCA EU
OmpC. EU
MGX Reads
Human
Filtered
MTX Reads
Human
Filtered
Species
Bacteroides fragilis
Bacteroides ovatus
Bacteroides uniformis
Bacteroides vulgatus
Parabacteroides distasonis
Subdoligranulum unclassified
Roseburia inulinivorans
Ruminococcus torques
Akkermansia muciniphila
Escherichia coli
other
Transcription
Bacteroides fragilis
Bacteroides ovatus
Bacteroides uniformis
Bacteroides vulgatus
Bacteroides massiliensis
Parabacteroides distasonis
Faecalibacterium prausnitzii
Akkermansia muciniphila
Escherichia coli
other
unclassified
Aerotolerance
obligate_anaerobe
aerotolerant
facultative_anaerobe
unknown
Viruses
Pseudomonas phage philBB−PF7A
T7virus
Pseudomonas phage PPpW−4
Cronobacter virus ESP29491
Enterobacteria phage vB EcoS NBD2
Shigella phage pSf−1
unclassified Siphoviridae
other
NA
Transcription
K01689: enolase [EC:4.2.1.11]
K01812: glucuronate isomerase [EC:5.3.1.12]
K01876: aspartyl−tRNA synthetase [EC:6.1.1...
K01893: asparaginyl−tRNA synthetase [EC:6...
K02968: small subunit ribosomal protein S20
K03088: RNA polymerase sigma−70 factor, E...
K03797: carboxyl−terminal processing protea...
K03977: GTP−binding protein
K04069: pyruvate formate lyase activating en...
K11065: thiol peroxidase, atypical 2−Cys per...
K13993: HSP20 family protein
Proteins
K00262: glutamate dehydrogenase (NADP+) ...
K01610: phosphoenolpyruvate carboxykinase...
K01805: xylose isomerase [EC:5.3.1.5]
K02867: large subunit ribosomal protein L11
K02994: small subunit ribosomal protein S8
K04043: molecular chaperone DnaK
K04077: chaperonin GroEL
K10117: raffinose/stachyose/melibiose transp...
K10439: ribose transport system substrate−b...
UNGROUPED
Taxonomy
PCo 1 (8.1%)
PCo 2 (6.7%)
Transcripts
PCo 1 (14.2%)
PCo 2 (9.2%)
Proteins
PCo 1 (30.8%)
PCo 2 (12.3%)
Metabolites
PCo 1 (8.8%)
PCo 2 (7.0%)

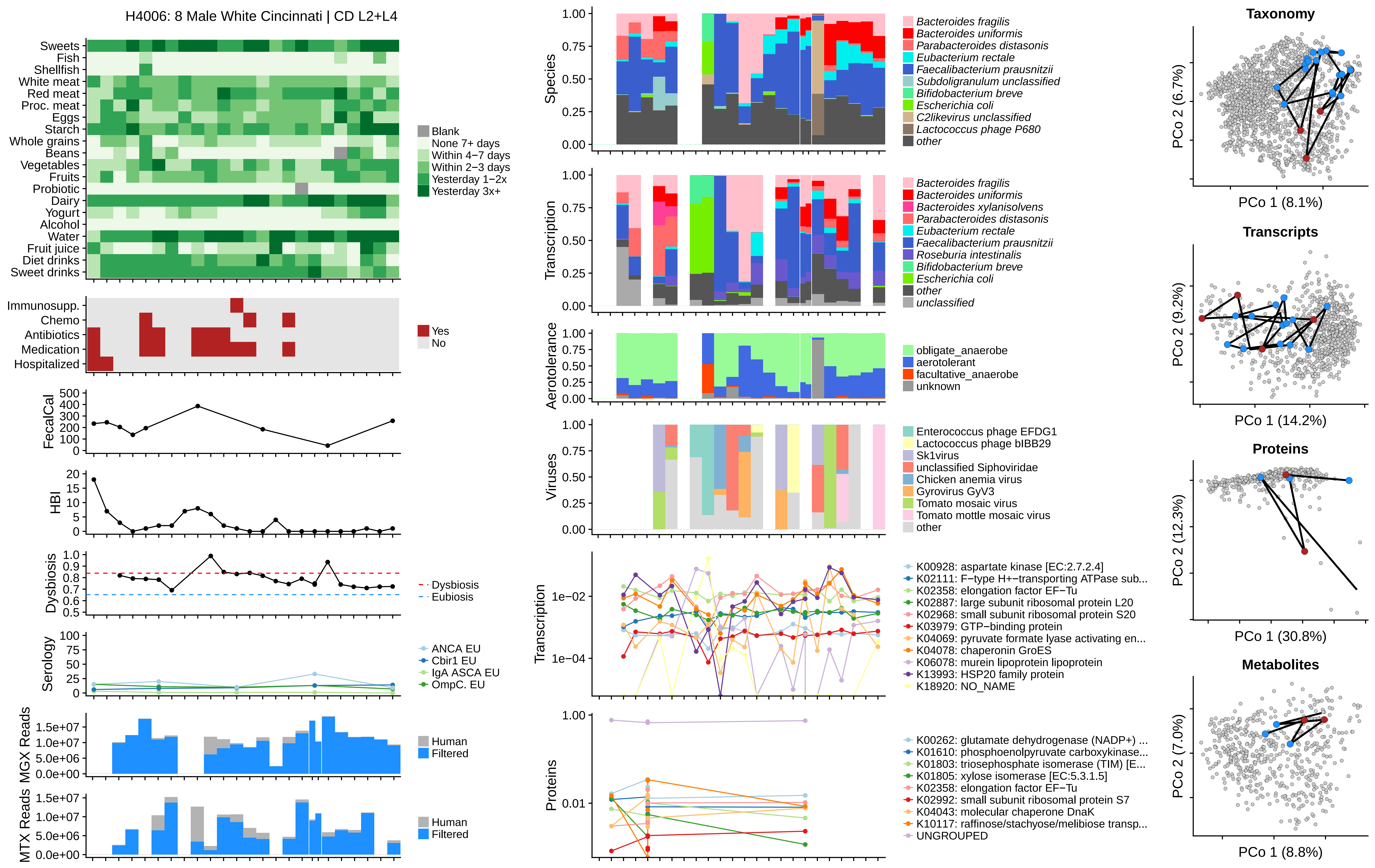

H4006: 8 Male White Cincinnati | CD L2+L4
Sweets
Fish
Shellfish
White meat
Red meat
Proc. meat
Eggs
Starch
Whole grains
Beans
Vegetables
Fruits
Probiotic
Dairy
Yogurt
Alcohol
Water
Fruit juice
Diet drinks
Sweet drinks
Blank
None 7+ days
Within 4−7 days
Within 2−3 days
Yesterday 1−2x
Yesterday 3x+
Immunosupp.
Chemo
Antibiotics
Medication
Hospitalized
Yes
No
FecalCal
HBI
Dysbiosis
Dysbiosis
Eubiosis
Serology
ANCA EU
Cbir1 EU
IgA ASCA EU
OmpC. EU
MGX Reads
Human
Filtered
MTX Reads
Human
Filtered
Species
Bacteroides fragilis
Bacteroides uniformis
Parabacteroides distasonis
Eubacterium rectale
Faecalibacterium prausnitzii
Subdoligranulum unclassified
Bifidobacterium breve
Escherichia coli
C2likevirus unclassified
Lactococcus phage P680
other
Transcription
Bacteroides fragilis
Bacteroides uniformis
Bacteroides xylanisolvens
Parabacteroides distasonis
Eubacterium rectale
Faecalibacterium prausnitzii
Roseburia intestinalis
Bifidobacterium breve
Escherichia coli
other
unclassified
Aerotolerance
obligate_anaerobe
aerotolerant
facultative_anaerobe
unknown
Viruses
Enterococcus phage EFDG1
Lactococcus phage bIBB29
Sk1virus
unclassified Siphoviridae
Chicken anemia virus
Gyrovirus GyV3
Tomato mosaic virus
Tomato mottle mosaic virus
other
Transcription
K00928: aspartate kinase [EC:2.7.2.4]
K02111: F−type H+−transporting ATPase sub...
K02358: elongation factor EF−Tu
K02887: large subunit ribosomal protein L20
K02968: small subunit ribosomal protein S20
K03979: GTP−binding protein
K04069: pyruvate formate lyase activating en...
K04078: chaperonin GroES
K06078: murein lipoprotein lipoprotein
K13993: HSP20 family protein
K18920: NO_NAME
Proteins
K00262: glutamate dehydrogenase (NADP+) ...
K01610: phosphoenolpyruvate carboxykinase...
K01803: triosephosphate isomerase (TIM) [E...
K01805: xylose isomerase [EC:5.3.1.5]
K02358: elongation factor EF−Tu
K02992: small subunit ribosomal protein S7
K04043: molecular chaperone DnaK
K10117: raffinose/stachyose/melibiose transp...
UNGROUPED
Taxonomy
PCo 1 (8.1%)
PCo 2 (6.7%)
Transcripts
PCo 1 (14.2%)
PCo 2 (9.2%)
Proteins
PCo 1 (30.8%)
PCo 2 (12.3%)
Metabolites
PCo 1 (8.8%)
PCo 2 (7.0%)

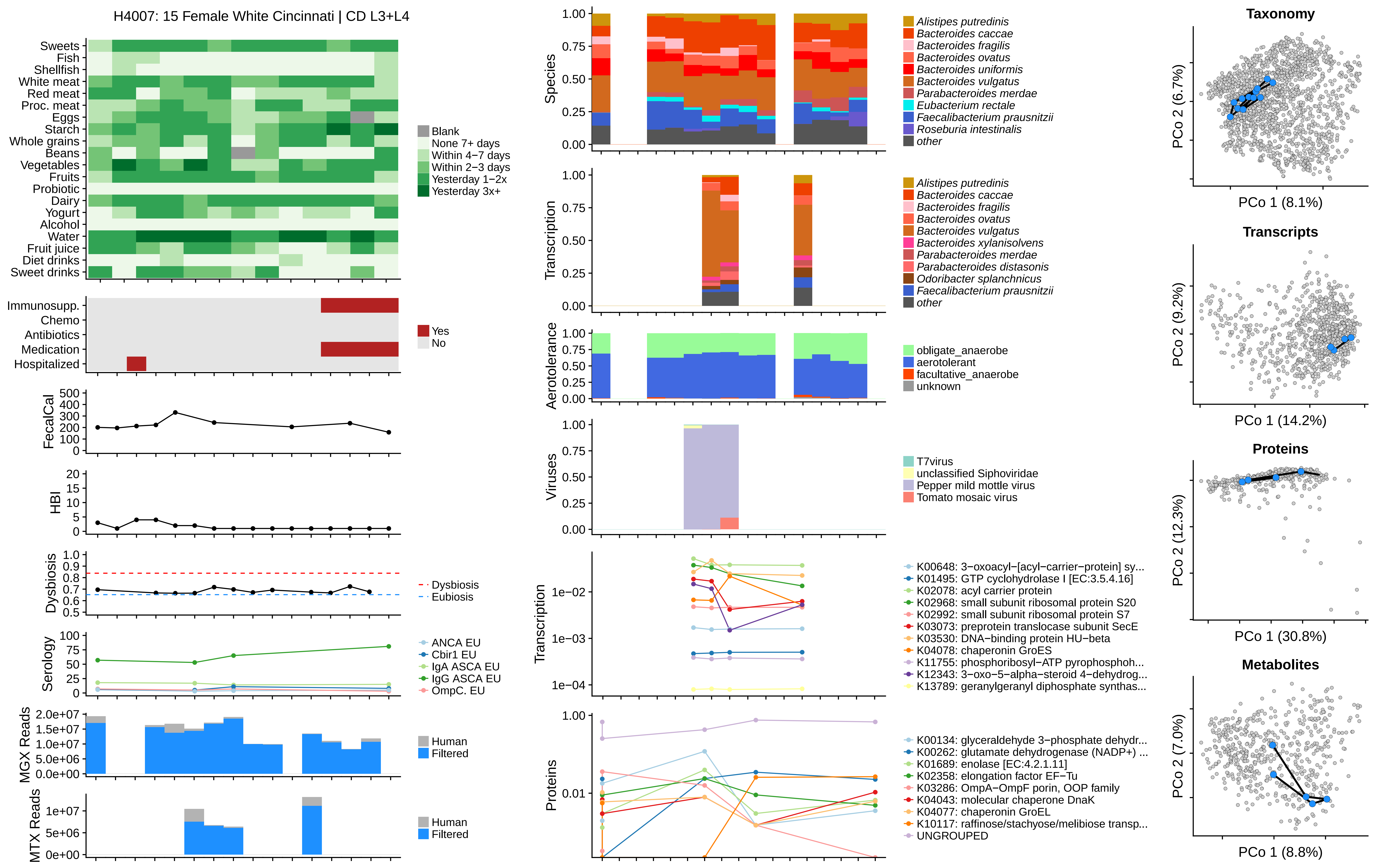

H4007: 15 Female White Cincinnati | CD L3+L4
Sweets
Fish
Shellfish
White meat
Red meat
Proc. meat
Eggs
Starch
Whole grains
Beans
Vegetables
Fruits
Probiotic
Dairy
Yogurt
Alcohol
Water
Fruit juice
Diet drinks
Sweet drinks
Blank
None 7+ days
Within 4−7 days
Within 2−3 days
Yesterday 1−2x
Yesterday 3x+
Immunosupp.
Chemo
Antibiotics
Medication
Hospitalized
Yes
No
FecalCal
HBI
Dysbiosis
Dysbiosis
Eubiosis
Serology
ANCA EU
Cbir1 EU
IgA ASCA EU
IgG ASCA EU
OmpC. EU
MGX Reads
Human
Filtered
MTX Reads
Human
Filtered
Species
Alistipes putredinis
Bacteroides caccae
Bacteroides fragilis
Bacteroides ovatus
Bacteroides uniformis
Bacteroides vulgatus
Parabacteroides merdae
Eubacterium rectale
Faecalibacterium prausnitzii
Roseburia intestinalis
other
Transcription
Alistipes putredinis
Bacteroides caccae
Bacteroides fragilis
Bacteroides ovatus
Bacteroides vulgatus
Bacteroides xylanisolvens
Parabacteroides merdae
Parabacteroides distasonis
Odoribacter splanchnicus
Faecalibacterium prausnitzii
other
Aerotolerance
obligate_anaerobe
aerotolerant
facultative_anaerobe
unknown
Viruses
T7virus
unclassified Siphoviridae
Pepper mild mottle virus
Tomato mosaic virus
Transcription
K00648: 3−oxoacyl−[acyl−carrier−protein] sy...
K01495: GTP cyclohydrolase I [EC:3.5.4.16]
K02078: acyl carrier protein
K02968: small subunit ribosomal protein S20
K02992: small subunit ribosomal protein S7
K03073: preprotein translocase subunit SecE
K03530: DNA−binding protein HU−beta
K04078: chaperonin GroES
K11755: phosphoribosyl−ATP pyrophosphoh...
K12343: 3−oxo−5−alpha−steroid 4−dehydrog...
K13789: geranylgeranyl diphosphate synthas...
Proteins
K00134: glyceraldehyde 3−phosphate dehydr...
K00262: glutamate dehydrogenase (NADP+) ...
K01689: enolase [EC:4.2.1.11]
K02358: elongation factor EF−Tu
K03286: OmpA−OmpF porin, OOP family
K04043: molecular chaperone DnaK
K04077: chaperonin GroEL
K10117: raffinose/stachyose/melibiose transp...
UNGROUPED
Taxonomy
PCo 1 (8.1%)
PCo 2 (6.7%)
Transcripts
PCo 1 (14.2%)
PCo 2 (9.2%)
Proteins
PCo 1 (30.8%)
PCo 2 (12.3%)
Metabolites
PCo 1 (8.8%)
PCo 2 (7.0%)

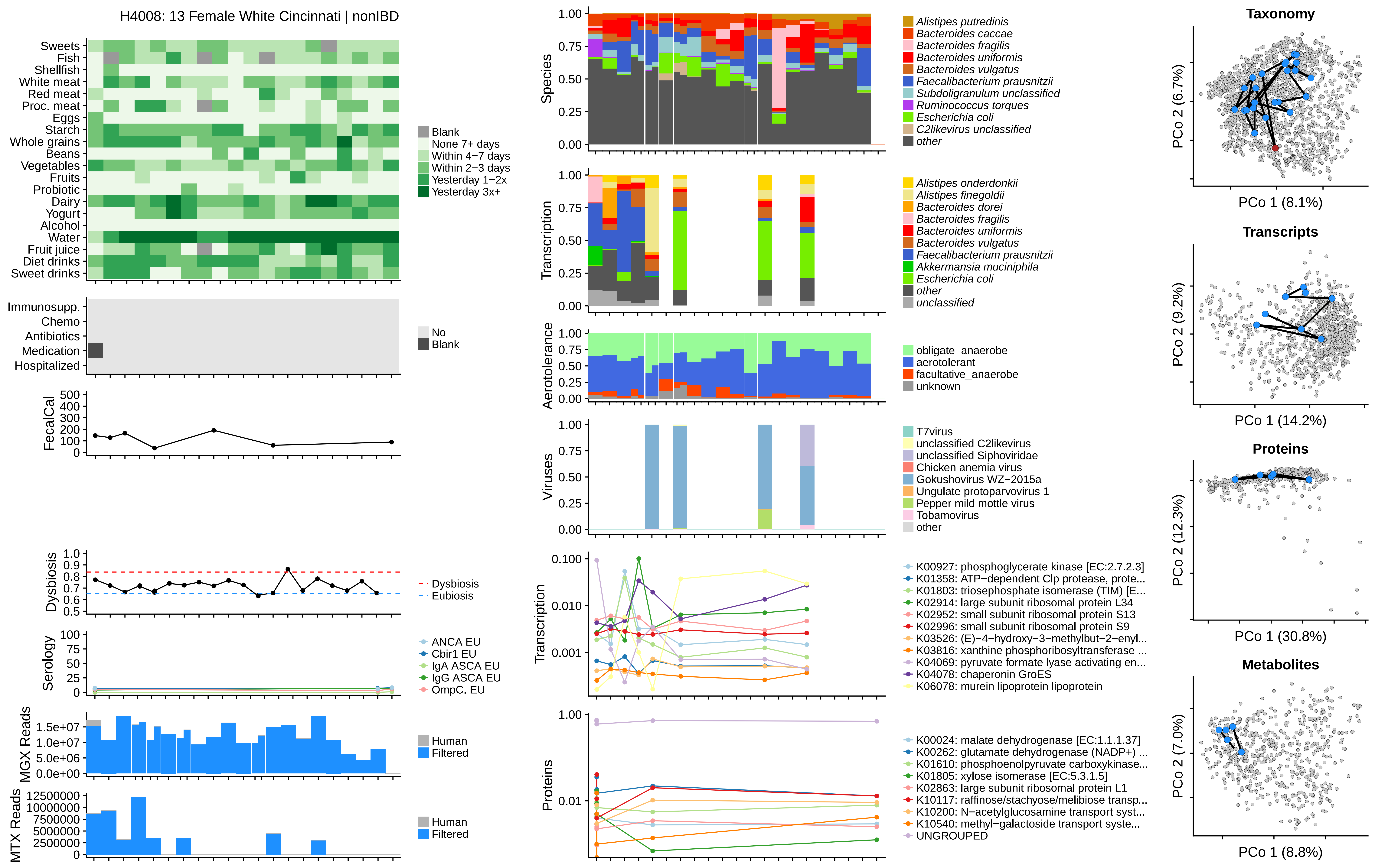
H4008: 13 Female White Cincinnati | nonIBD
Sweets
Fish
Shellfish
White meat
Red meat
Proc. meat
Eggs
Starch
Whole grains
Beans
Vegetables
Fruits
Probiotic
Dairy
Yogurt
Alcohol
Water
Fruit juice
Diet drinks
Sweet drinks
Blank
None 7+ days
Within 4−7 days
Within 2−3 days
Yesterday 1−2x
Yesterday 3x+
Immunosupp.
Chemo
Antibiotics
Medication
Hospitalized
No
Blank
FecalCal
Dysbiosis
Dysbiosis
Eubiosis
Serology
ANCA EU
Cbir1 EU
IgA ASCA EU
IgG ASCA EU
OmpC. EU
MGX Reads
Human
Filtered
MTX Reads
Human
Filtered
Species
Alistipes putredinis
Bacteroides caccae
Bacteroides fragilis
Bacteroides uniformis
Bacteroides vulgatus
Faecalibacterium prausnitzii
Subdoligranulum unclassified
Ruminococcus torques
Escherichia coli
C2likevirus unclassified
other
Transcription
Alistipes onderdonkii
Alistipes finegoldii
Bacteroides dorei
Bacteroides fragilis
Bacteroides uniformis
Bacteroides vulgatus
Faecalibacterium prausnitzii
Akkermansia muciniphila
Escherichia coli
other
unclassified
Aerotolerance
obligate_anaerobe
aerotolerant
facultative_anaerobe
unknown
Viruses
T7virus
unclassified C2likevirus
unclassified Siphoviridae
Chicken anemia virus
Gokushovirus WZ−2015a
Ungulate protoparvovirus 1
Pepper mild mottle virus
Tobamovirus
other
Transcription
K00927: phosphoglycerate kinase [EC:2.7.2.3]
K01358: ATP−dependent Clp protease, prote...
K01803: triosephosphate isomerase (TIM) [E...
K02914: large subunit ribosomal protein L34
K02952: small subunit ribosomal protein S13
K02996: small subunit ribosomal protein S9
K03526: (E)−4−hydroxy−3−methylbut−2−enyl...
K03816: xanthine phosphoribosyltransferase ...
K04069: pyruvate formate lyase activating en...
K04078: chaperonin GroES
K06078: murein lipoprotein lipoprotein
Proteins
K00024: malate dehydrogenase [EC:1.1.1.37]
K00262: glutamate dehydrogenase (NADP+) ...
K01610: phosphoenolpyruvate carboxykinase...
K01805: xylose isomerase [EC:5.3.1.5]
K02863: large subunit ribosomal protein L1
K10117: raffinose/stachyose/melibiose transp...
K10200: N−acetylglucosamine transport syst...
K10540: methyl−galactoside transport syste...
UNGROUPED
Taxonomy
PCo 1 (8.1%)
PCo 2 (6.7%)
Transcripts
PCo 1 (14.2%)
PCo 2 (9.2%)
Proteins
PCo 1 (30.8%)
PCo 2 (12.3%)
Metabolites
PCo 1 (8.8%)
PCo 2 (7.0%)

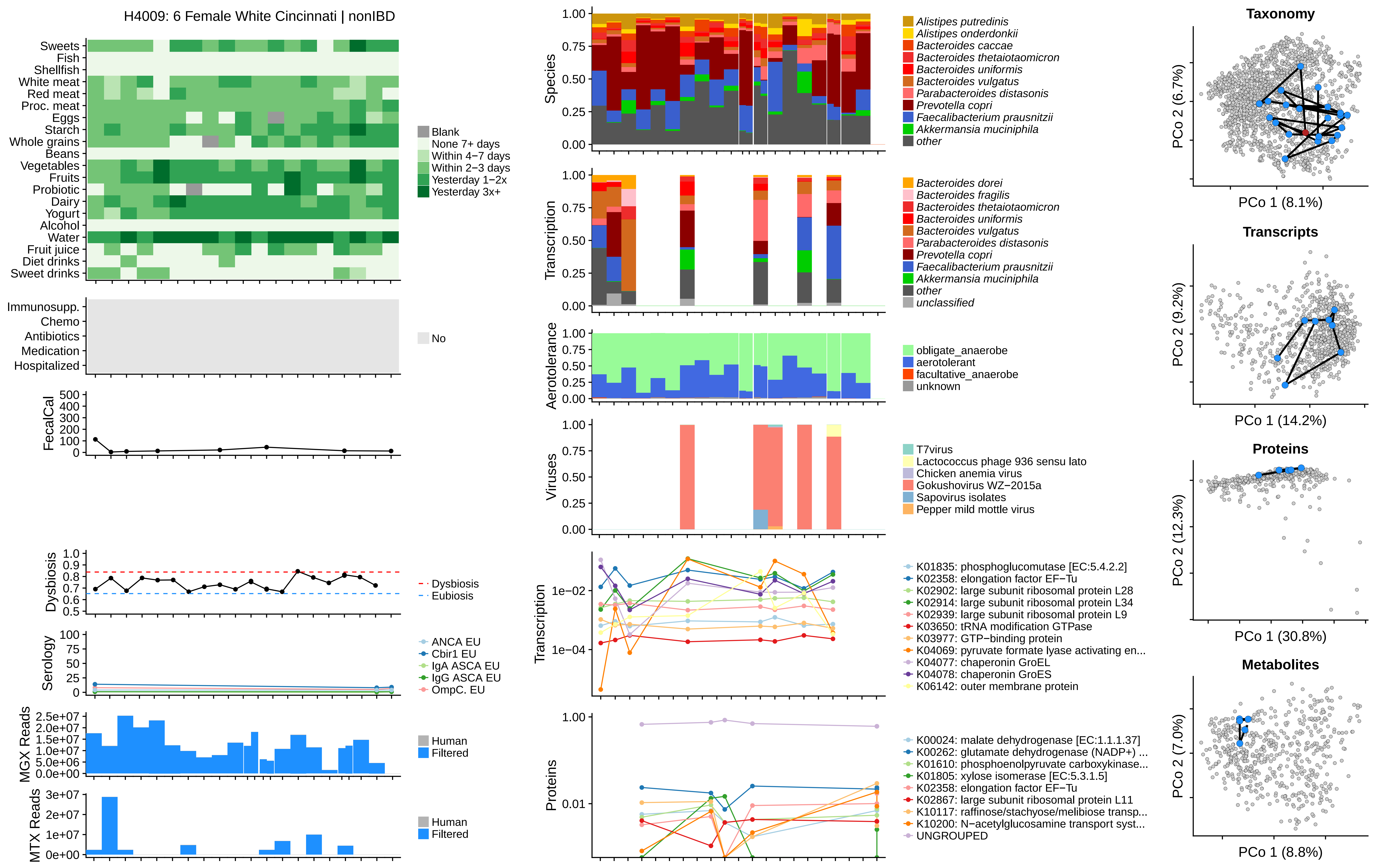
H4009: 6 Female White Cincinnati | nonIBD
Sweets
Fish
Shellfish
White meat
Red meat
Proc. meat
Eggs
Starch
Whole grains
Beans
Vegetables
Fruits
Probiotic
Dairy
Yogurt
Alcohol
Water
Fruit juice
Diet drinks
Sweet drinks
Blank
None 7+ days
Within 4−7 days
Within 2−3 days
Yesterday 1−2x
Yesterday 3x+
Immunosupp.
Chemo
Antibiotics
Medication
Hospitalized
No
FecalCal
Dysbiosis
Dysbiosis
Eubiosis
Serology
ANCA EU
Cbir1 EU
IgA ASCA EU
IgG ASCA EU
OmpC. EU
MGX Reads
Human
Filtered
MTX Reads
Human
Filtered
Species
Alistipes putredinis
Alistipes onderdonkii
Bacteroides caccae
Bacteroides thetaiotaomicron
Bacteroides uniformis
Bacteroides vulgatus
Parabacteroides distasonis
Prevotella copri
Faecalibacterium prausnitzii
Akkermansia muciniphila
other
Transcription
Bacteroides dorei
Bacteroides fragilis
Bacteroides thetaiotaomicron
Bacteroides uniformis
Bacteroides vulgatus
Parabacteroides distasonis
Prevotella copri
Faecalibacterium prausnitzii
Akkermansia muciniphila
other
unclassified
Aerotolerance
obligate_anaerobe
aerotolerant
facultative_anaerobe
unknown
Viruses
T7virus
Lactococcus phage 936 sensu lato
Chicken anemia virus
Gokushovirus WZ−2015a
Sapovirus isolates
Pepper mild mottle virus
Transcription
K01835: phosphoglucomutase [EC:5.4.2.2]
K02358: elongation factor EF−Tu
K02902: large subunit ribosomal protein L28
K02914: large subunit ribosomal protein L34
K02939: large subunit ribosomal protein L9
K03650: tRNA modification GTPase
K03977: GTP−binding protein
K04069: pyruvate formate lyase activating en...
K04077: chaperonin GroEL
K04078: chaperonin GroES
K06142: outer membrane protein
Proteins
K00024: malate dehydrogenase [EC:1.1.1.37]
K00262: glutamate dehydrogenase (NADP+) ...
K01610: phosphoenolpyruvate carboxykinase...
K01805: xylose isomerase [EC:5.3.1.5]
K02358: elongation factor EF−Tu
K02867: large subunit ribosomal protein L11
K10117: raffinose/stachyose/melibiose transp...
K10200: N−acetylglucosamine transport syst...
UNGROUPED
Taxonomy
PCo 1 (8.1%)
PCo 2 (6.7%)
Transcripts
PCo 1 (14.2%)
PCo 2 (9.2%)
Proteins
PCo 1 (30.8%)
PCo 2 (12.3%)
Metabolites
PCo 1 (8.8%)
PCo 2 (7.0%)

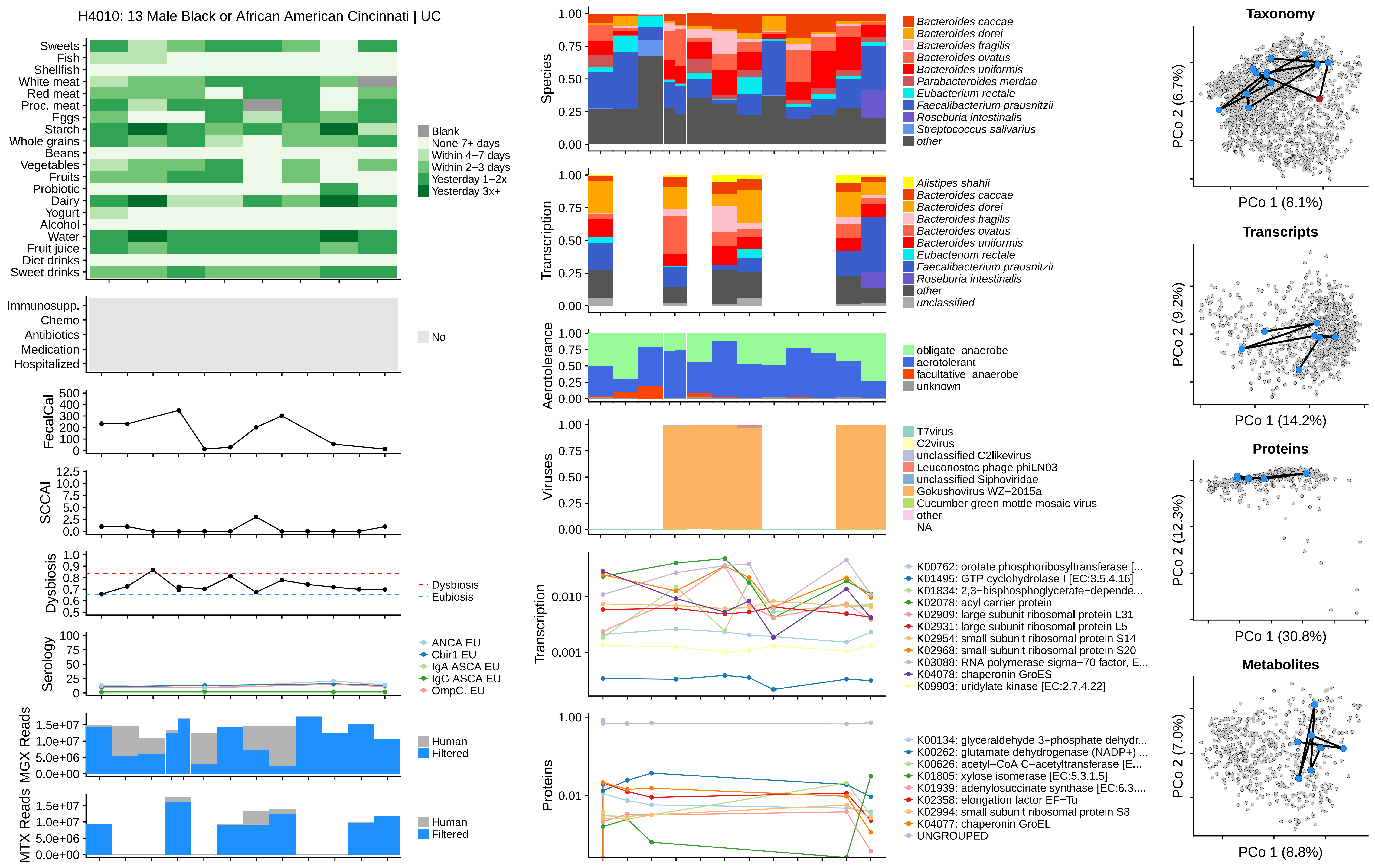
H4010: 13 Male Black or African American Cincinnati | UC
Sweets
Fish
Shellfish
White meat
Red meat
Proc. meat
Eggs
Starch
Whole grains
Beans
Vegetables
Fruits
Probiotic
Dairy
Yogurt
Alcohol
Water
Fruit juice
Diet drinks
Sweet drinks
Blank
None 7+ days
Within 4−7 days
Within 2−3 days
Yesterday 1−2x
Yesterday 3x+
Immunosupp.
Chemo
Antibiotics
Medication
Hospitalized
No
FecalCal
SCCAI
Dysbiosis
Dysbiosis
Eubiosis
Serology
ANCA EU
Cbir1 EU
IgA ASCA EU
IgG ASCA EU
OmpC. EU
MGX Reads
Human
Filtered
MTX Reads
Human
Filtered
Species
Bacteroides caccae
Bacteroides dorei
Bacteroides fragilis
Bacteroides ovatus
Bacteroides uniformis
Parabacteroides merdae
Eubacterium rectale
Faecalibacterium prausnitzii
Roseburia intestinalis
Streptococcus salivarius
other
Transcription
Alistipes shahii
Bacteroides caccae
Bacteroides dorei
Bacteroides fragilis
Bacteroides ovatus
Bacteroides uniformis
Eubacterium rectale
Faecalibacterium prausnitzii
Roseburia intestinalis
other
unclassified
Aerotolerance
obligate_anaerobe
aerotolerant
facultative_anaerobe
unknown
Viruses
T7virus
C2virus
unclassified C2likevirus
Leuconostoc phage phiLN03
unclassified Siphoviridae
Gokushovirus WZ−2015a
Cucumber green mottle mosaic virus
other
NA
Transcription
K00762: orotate phosphoribosyltransferase [...
K01495: GTP cyclohydrolase I [EC:3.5.4.16]
K01834: 2,3−bisphosphoglycerate−depende...
K02078: acyl carrier protein
K02909: large subunit ribosomal protein L31
K02931: large subunit ribosomal protein L5
K02954: small subunit ribosomal protein S14
K02968: small subunit ribosomal protein S20
K03088: RNA polymerase sigma−70 factor, E...
K04078: chaperonin GroES
K09903: uridylate kinase [EC:2.7.4.22]
Proteins
K00134: glyceraldehyde 3−phosphate dehydr...
K00262: glutamate dehydrogenase (NADP+) ...
K00626: acetyl−CoA C−acetyltransferase [E...
K01805: xylose isomerase [EC:5.3.1.5]
K01939: adenylosuccinate synthase [EC:6.3....
K02358: elongation factor EF−Tu
K02994: small subunit ribosomal protein S8
K04077: chaperonin GroEL
UNGROUPED
Taxonomy
PCo 1 (8.1%)
PCo 2 (6.7%)
Transcripts
PCo 1 (14.2%)
PCo 2 (9.2%)
Proteins
PCo 1 (30.8%)
PCo 2 (12.3%)
Metabolites
PCo 1 (8.8%)
PCo 2 (7.0%)

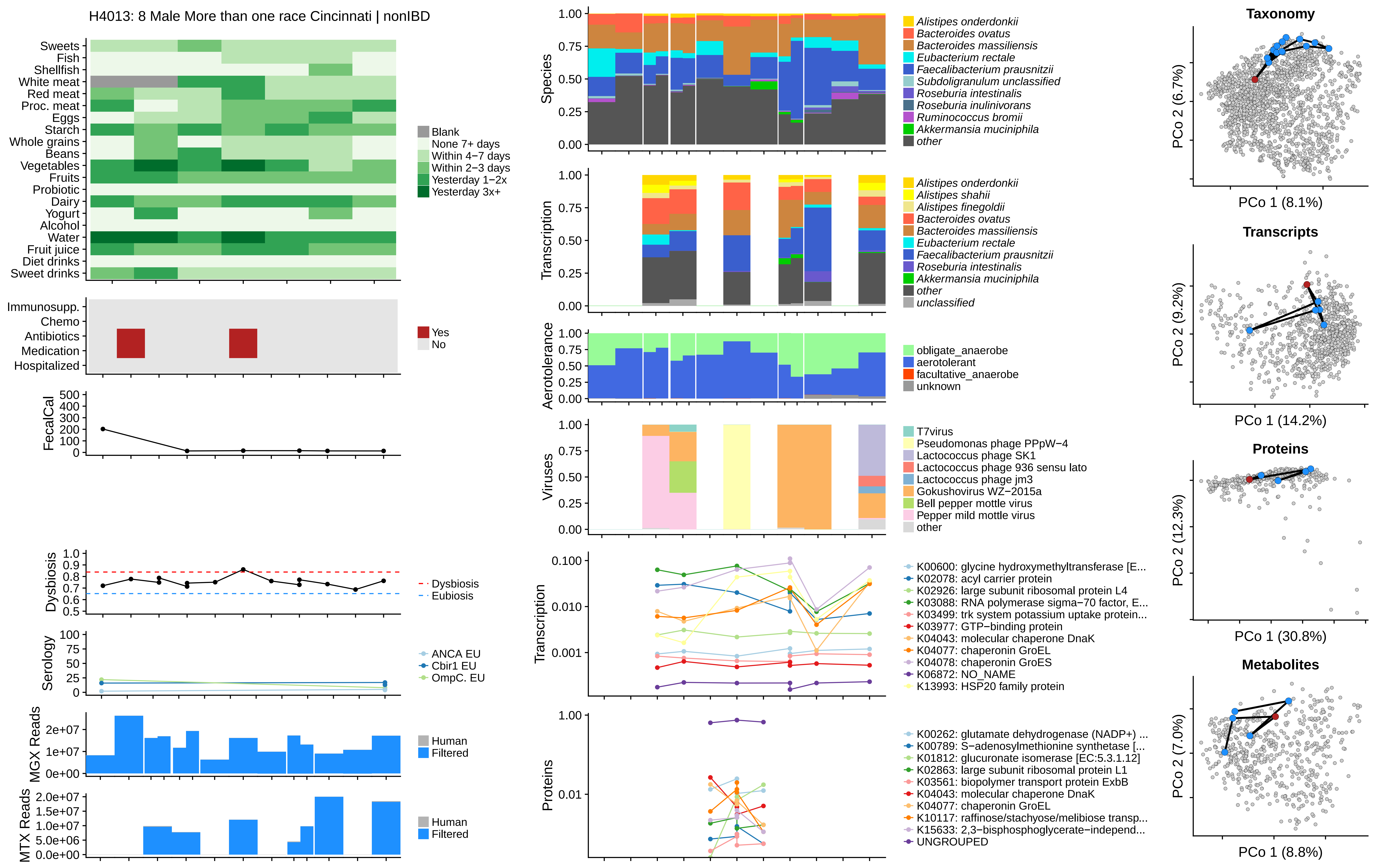
H4013: 8 Male More than one race Cincinnati | nonIBD
Sweets
Fish
Shellfish
White meat
Red meat
Proc. meat
Eggs
Starch
Whole grains
Beans
Vegetables
Fruits
Probiotic
Dairy
Yogurt
Alcohol
Water
Fruit juice
Diet drinks
Sweet drinks
Blank
None 7+ days
Within 4−7 days
Within 2−3 days
Yesterday 1−2x
Yesterday 3x+
Immunosupp.
Chemo
Antibiotics
Medication
Hospitalized
Yes
No
FecalCal
Dysbiosis
Dysbiosis
Eubiosis
Serology
ANCA EU
Cbir1 EU
OmpC. EU
MGX Reads
Human
Filtered
MTX Reads
Human
Filtered
Species
Alistipes onderdonkii
Bacteroides ovatus
Bacteroides massiliensis
Eubacterium rectale
Faecalibacterium prausnitzii
Subdoligranulum unclassified
Roseburia intestinalis
Roseburia inulinivorans
Ruminococcus bromii
Akkermansia muciniphila
other
Transcription
Alistipes onderdonkii
Alistipes shahii
Alistipes finegoldii
Bacteroides ovatus
Bacteroides massiliensis
Eubacterium rectale
Faecalibacterium prausnitzii
Roseburia intestinalis
Akkermansia muciniphila
other
unclassified
Aerotolerance
obligate_anaerobe
aerotolerant
facultative_anaerobe
unknown
Viruses
T7virus
Pseudomonas phage PPpW−4
Lactococcus phage SK1
Lactococcus phage 936 sensu lato
Lactococcus phage jm3
Gokushovirus WZ−2015a
Bell pepper mottle virus
Pepper mild mottle virus
other
Transcription
K00600: glycine hydroxymethyltransferase [E...
K02078: acyl carrier protein
K02926: large subunit ribosomal protein L4
K03088: RNA polymerase sigma−70 factor, E...
K03499: trk system potassium uptake protein...
K03977: GTP−binding protein
K04043: molecular chaperone DnaK
K04077: chaperonin GroEL
K04078: chaperonin GroES
K06872: NO_NAME
K13993: HSP20 family protein
Proteins
K00262: glutamate dehydrogenase (NADP+) ...
K00789: S−adenosylmethionine synthetase [...
K01812: glucuronate isomerase [EC:5.3.1.12]
K02863: large subunit ribosomal protein L1
K03561: biopolymer transport protein ExbB
K04043: molecular chaperone DnaK
K04077: chaperonin GroEL
K10117: raffinose/stachyose/melibiose transp...
K15633: 2,3−bisphosphoglycerate−independ...
UNGROUPED
Taxonomy
PCo 1 (8.1%)
PCo 2 (6.7%)
Transcripts
PCo 1 (14.2%)
PCo 2 (9.2%)
Proteins
PCo 1 (30.8%)
PCo 2 (12.3%)
Metabolites
PCo 1 (8.8%)
PCo 2 (7.0%)

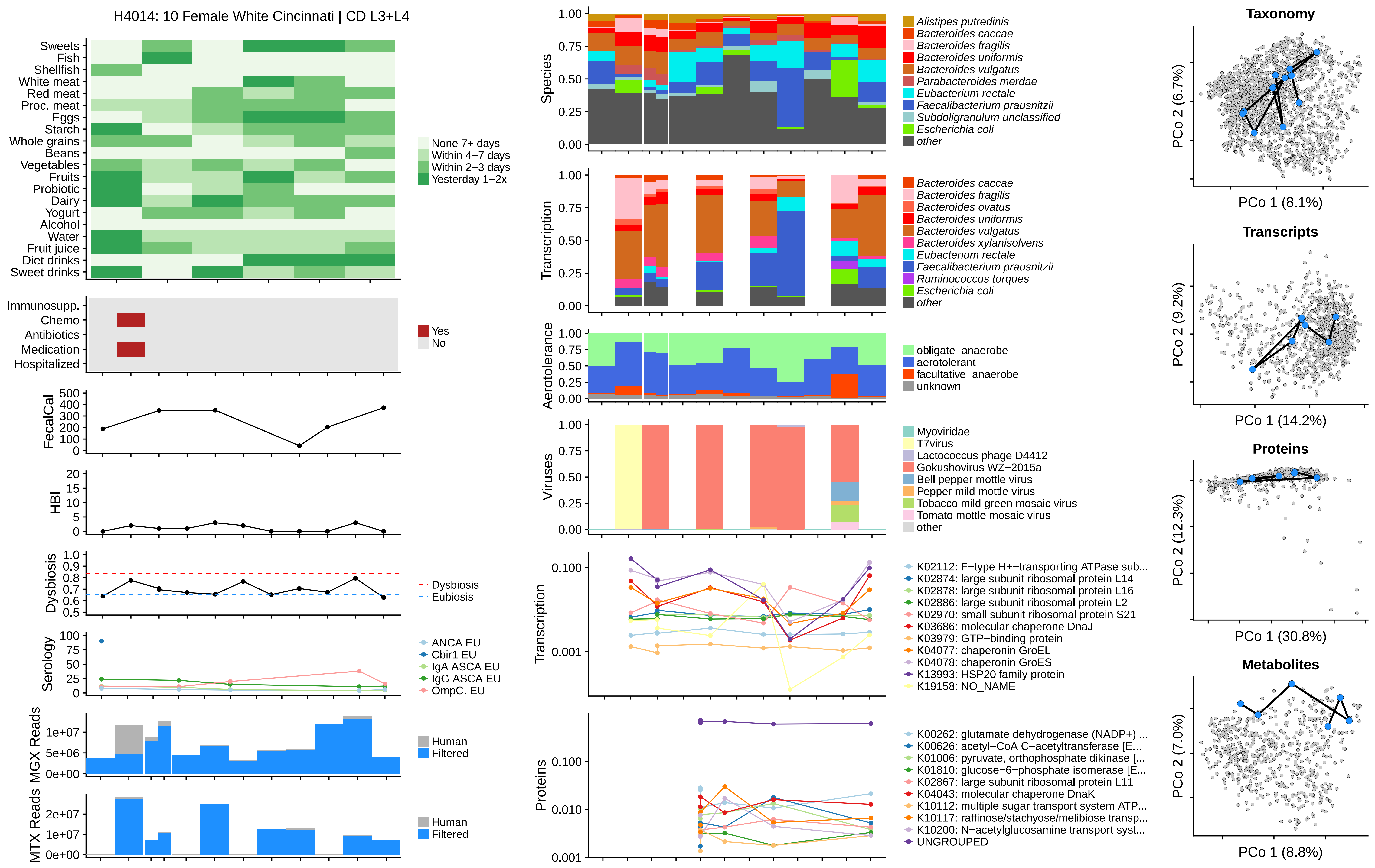
H4014: 10 Female White Cincinnati | CD L3+L4
Sweets
Fish
Shellfish
White meat
Red meat
Proc. meat
Eggs
Starch
Whole grains
Beans
Vegetables
Fruits
Probiotic
Dairy
Yogurt
Alcohol
Water
Fruit juice
Diet drinks
Sweet drinks
None 7+ days
Within 4−7 days
Within 2−3 days
Yesterday 1−2x
Immunosupp.
Chemo
Antibiotics
Medication
Hospitalized
Yes
No
FecalCal
HBI
Dysbiosis
Dysbiosis
Eubiosis
Serology
ANCA EU
Cbir1 EU
IgA ASCA EU
IgG ASCA EU
OmpC. EU
MGX Reads
Human
Filtered
MTX Reads
Human
Filtered
Species
Alistipes putredinis
Bacteroides caccae
Bacteroides fragilis
Bacteroides uniformis
Bacteroides vulgatus
Parabacteroides merdae
Eubacterium rectale
Faecalibacterium prausnitzii
Subdoligranulum unclassified
Escherichia coli
other
Transcription
Bacteroides caccae
Bacteroides fragilis
Bacteroides ovatus
Bacteroides uniformis
Bacteroides vulgatus
Bacteroides xylanisolvens
Eubacterium rectale
Faecalibacterium prausnitzii
Ruminococcus torques
Escherichia coli
other
Aerotolerance
obligate_anaerobe
aerotolerant
facultative_anaerobe
unknown
Viruses
Myoviridae
T7virus
Lactococcus phage D4412
Gokushovirus WZ−2015a
Bell pepper mottle virus
Pepper mild mottle virus
Tobacco mild green mosaic virus
Tomato mottle mosaic virus
other
Transcription
K02112: F−type H+−transporting ATPase sub...
K02874: large subunit ribosomal protein L14
K02878: large subunit ribosomal protein L16
K02886: large subunit ribosomal protein L2
K02970: small subunit ribosomal protein S21
K03686: molecular chaperone DnaJ
K03979: GTP−binding protein
K04077: chaperonin GroEL
K04078: chaperonin GroES
K13993: HSP20 family protein
K19158: NO_NAME
Proteins
K00262: glutamate dehydrogenase (NADP+) ...
K00626: acetyl−CoA C−acetyltransferase [E...
K01006: pyruvate, orthophosphate dikinase [...
K01810: glucose−6−phosphate isomerase [E...
K02867: large subunit ribosomal protein L11
K04043: molecular chaperone DnaK
K10112: multiple sugar transport system ATP...
K10117: raffinose/stachyose/melibiose transp...
K10200: N−acetylglucosamine transport syst...
UNGROUPED
Taxonomy
PCo 1 (8.1%)
PCo 2 (6.7%)
Transcripts
PCo 1 (14.2%)
PCo 2 (9.2%)
Proteins
PCo 1 (30.8%)
PCo 2 (12.3%)
Metabolites
PCo 1 (8.8%)
PCo 2 (7.0%)

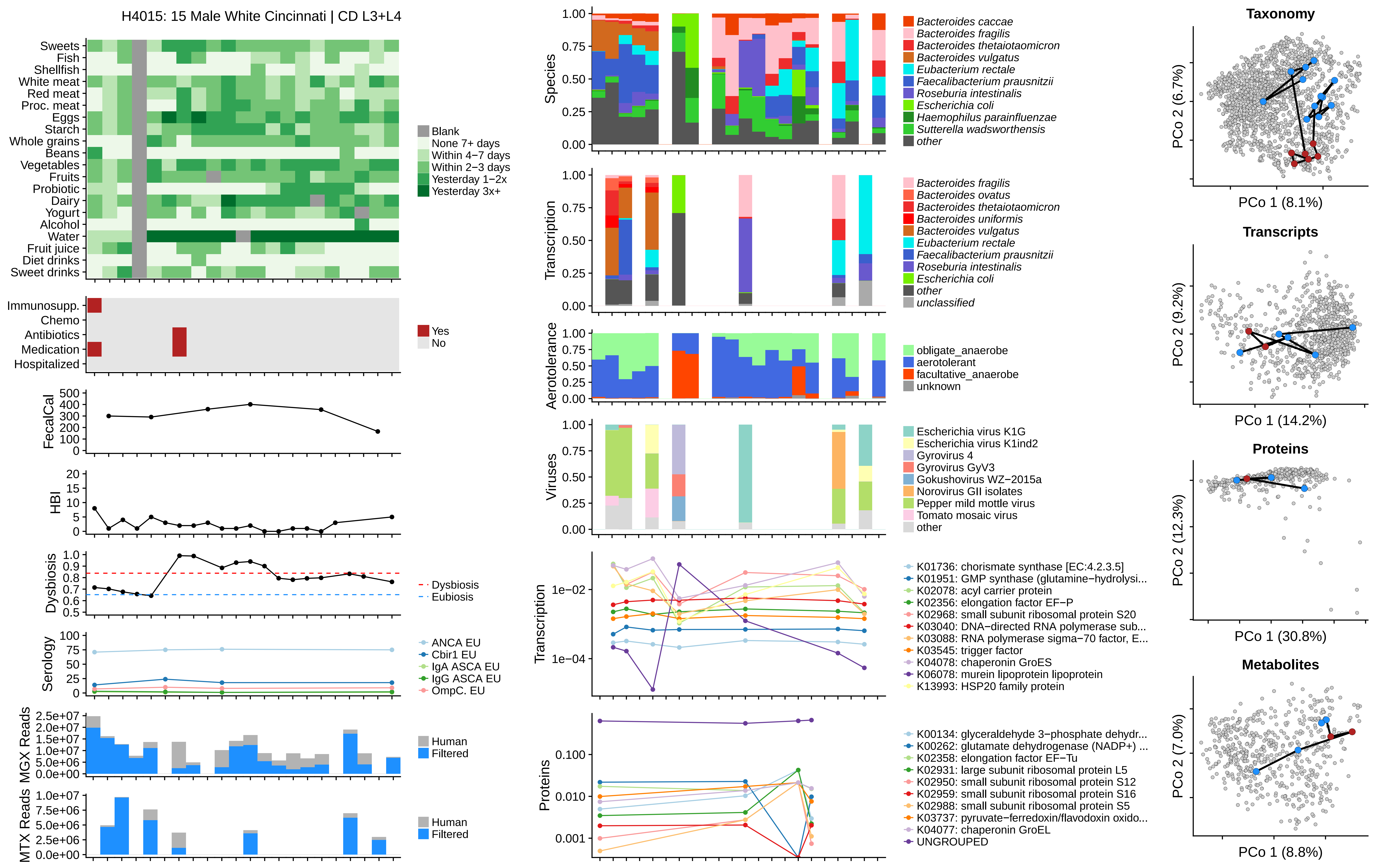
H4015: 15 Male White Cincinnati | CD L3+L4
Sweets
Fish
Shellfish
White meat
Red meat
Proc. meat
Eggs
Starch
Whole grains
Beans
Vegetables
Fruits
Probiotic
Dairy
Yogurt
Alcohol
Water
Fruit juice
Diet drinks
Sweet drinks
Blank
None 7+ days
Within 4−7 days
Within 2−3 days
Yesterday 1−2x
Yesterday 3x+
Immunosupp.
Chemo
Antibiotics
Medication
Hospitalized
Yes
No
FecalCal
HBI
Dysbiosis
Dysbiosis
Eubiosis
Serology
ANCA EU
Cbir1 EU
IgA ASCA EU
IgG ASCA EU
OmpC. EU
MGX Reads
Human
Filtered
MTX Reads
Human
Filtered
Species
Bacteroides caccae
Bacteroides fragilis
Bacteroides thetaiotaomicron
Bacteroides vulgatus
Eubacterium rectale
Faecalibacterium prausnitzii
Roseburia intestinalis
Escherichia coli
Haemophilus parainfluenzae
Sutterella wadsworthensis
other
Transcription
Bacteroides fragilis
Bacteroides ovatus
Bacteroides thetaiotaomicron
Bacteroides uniformis
Bacteroides vulgatus
Eubacterium rectale
Faecalibacterium prausnitzii
Roseburia intestinalis
Escherichia coli
other
unclassified
Aerotolerance
obligate_anaerobe
aerotolerant
facultative_anaerobe
unknown
Viruses
Escherichia virus K1G
Escherichia virus K1ind2
Gyrovirus 4
Gyrovirus GyV3
Gokushovirus WZ−2015a
Norovirus GII isolates
Pepper mild mottle virus
Tomato mosaic virus
other
Transcription
K01736: chorismate synthase [EC:4.2.3.5]
K01951: GMP synthase (glutamine−hydrolysi...
K02078: acyl carrier protein
K02356: elongation factor EF−P
K02968: small subunit ribosomal protein S20
K03040: DNA−directed RNA polymerase sub...
K03088: RNA polymerase sigma−70 factor, E...
K03545: trigger factor
K04078: chaperonin GroES
K06078: murein lipoprotein lipoprotein
K13993: HSP20 family protein
Proteins
K00134: glyceraldehyde 3−phosphate dehydr...
K00262: glutamate dehydrogenase (NADP+) ...
K02358: elongation factor EF−Tu
K02931: large subunit ribosomal protein L5
K02950: small subunit ribosomal protein S12
K02959: small subunit ribosomal protein S16
K02988: small subunit ribosomal protein S5
K03737: pyruvate−ferredoxin/flavodoxin oxido...
K04077: chaperonin GroEL
UNGROUPED
Taxonomy
PCo 1 (8.1%)
PCo 2 (6.7%)
Transcripts
PCo 1 (14.2%)
PCo 2 (9.2%)
Proteins
PCo 1 (30.8%)
PCo 2 (12.3%)
Metabolites
PCo 1 (8.8%)
PCo 2 (7.0%)

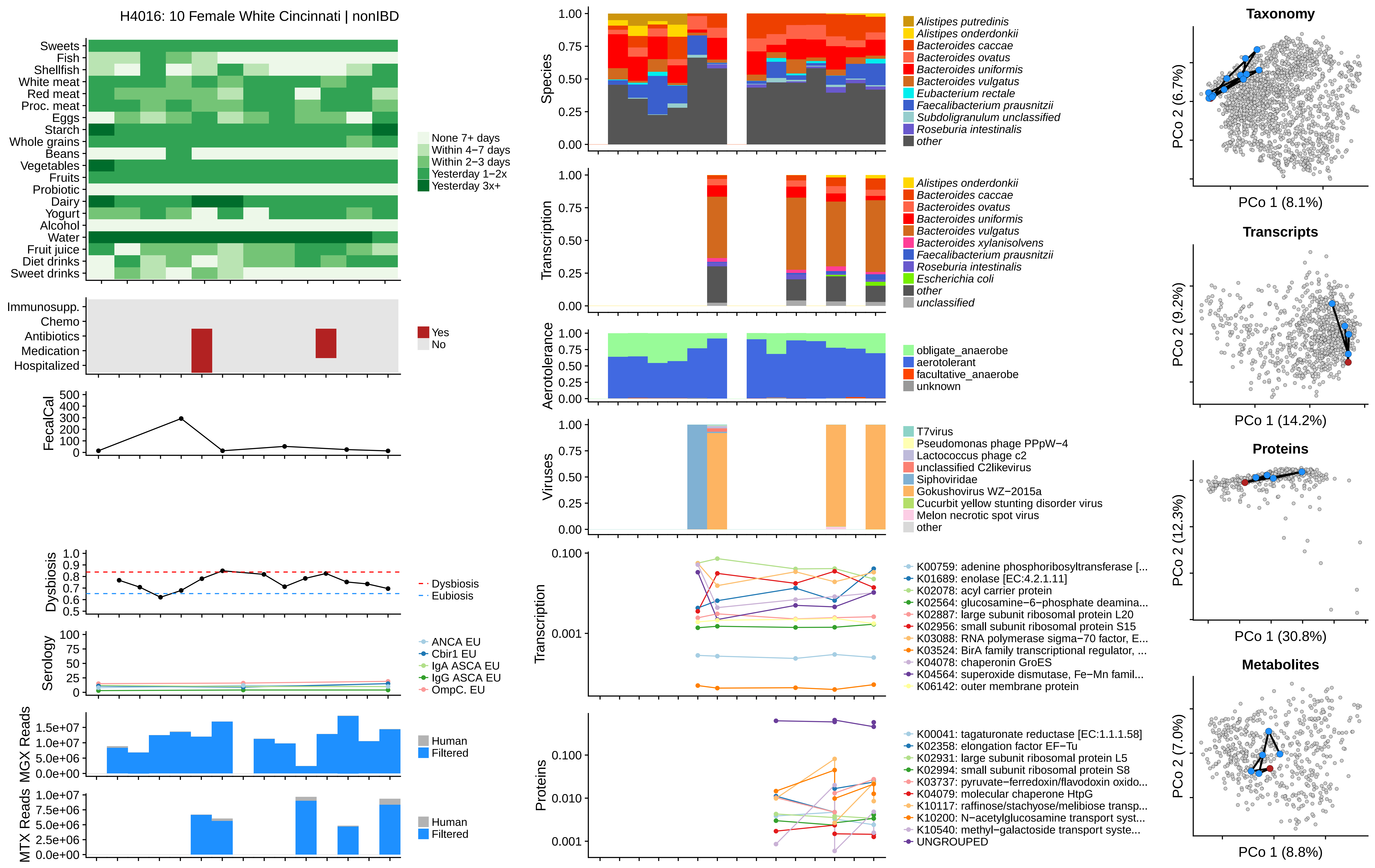

H4016: 10 Female White Cincinnati | nonIBD
Sweets
Fish
Shellfish
White meat
Red meat
Proc. meat
Eggs
Starch
Whole grains
Beans
Vegetables
Fruits
Probiotic
Dairy
Yogurt
Alcohol
Water
Fruit juice
Diet drinks
Sweet drinks
None 7+ days
Within 4−7 days
Within 2−3 days
Yesterday 1−2x
Yesterday 3x+
Immunosupp.
Chemo
Antibiotics
Medication
Hospitalized
Yes
No
FecalCal
Dysbiosis
Dysbiosis
Eubiosis
Serology
ANCA EU
Cbir1 EU
IgA ASCA EU
IgG ASCA EU
OmpC. EU
MGX Reads
Human
Filtered
MTX Reads
Human
Filtered
Species
Alistipes putredinis
Alistipes onderdonkii
Bacteroides caccae
Bacteroides ovatus
Bacteroides uniformis
Bacteroides vulgatus
Eubacterium rectale
Faecalibacterium prausnitzii
Subdoligranulum unclassified
Roseburia intestinalis
other
Transcription
Alistipes onderdonkii
Bacteroides caccae
Bacteroides ovatus
Bacteroides uniformis
Bacteroides vulgatus
Bacteroides xylanisolvens
Faecalibacterium prausnitzii
Roseburia intestinalis
Escherichia coli
other
unclassified
Aerotolerance
obligate_anaerobe
aerotolerant
facultative_anaerobe
unknown
Viruses
T7virus
Pseudomonas phage PPpW−4
Lactococcus phage c2
unclassified C2likevirus
Siphoviridae
Gokushovirus WZ−2015a
Cucurbit yellow stunting disorder virus
Melon necrotic spot virus
other
Transcription
K00759: adenine phosphoribosyltransferase [...
K01689: enolase [EC:4.2.1.11]
K02078: acyl carrier protein
K02564: glucosamine−6−phosphate deamina...
K02887: large subunit ribosomal protein L20
K02956: small subunit ribosomal protein S15
K03088: RNA polymerase sigma−70 factor, E...
K03524: BirA family transcriptional regulator, ...
K04078: chaperonin GroES
K04564: superoxide dismutase, Fe−Mn famil...
K06142: outer membrane protein
Proteins
K00041: tagaturonate reductase [EC:1.1.1.58]
K02358: elongation factor EF−Tu
K02931: large subunit ribosomal protein L5
K02994: small subunit ribosomal protein S8
K03737: pyruvate−ferredoxin/flavodoxin oxido...
K04079: molecular chaperone HtpG
K10117: raffinose/stachyose/melibiose transp...
K10200: N−acetylglucosamine transport syst...
K10540: methyl−galactoside transport syste...
UNGROUPED
Taxonomy
PCo 1 (8.1%)
PCo 2 (6.7%)
Transcripts
PCo 1 (14.2%)
PCo 2 (9.2%)
Proteins
PCo 1 (30.8%)
PCo 2 (12.3%)
Metabolites
PCo 1 (8.8%)
PCo 2 (7.0%)

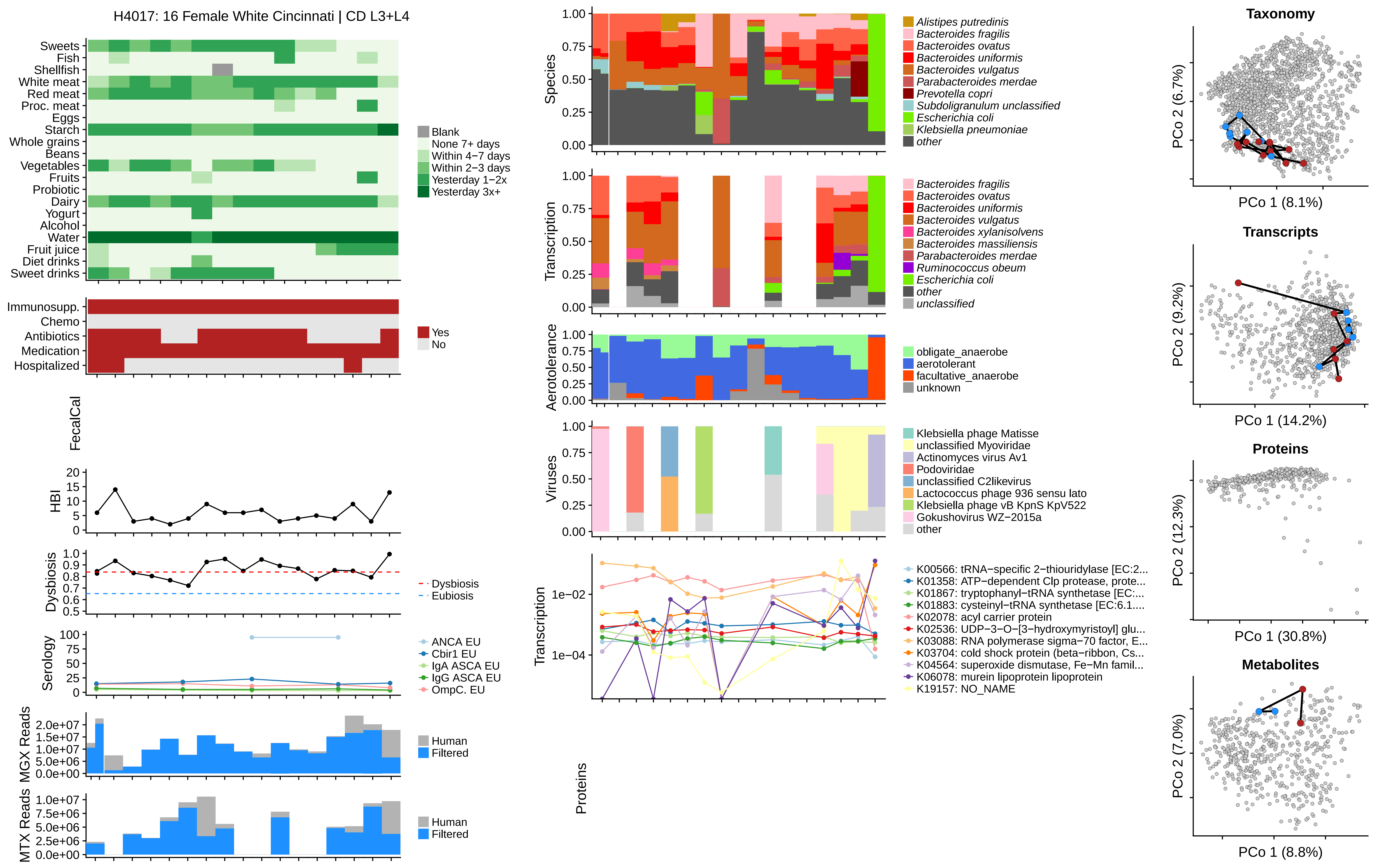
H4017: 16 Female White Cincinnati | CD L3+L4
Sweets
Fish
Shellfish
White meat
Red meat
Proc. meat
Eggs
Starch
Whole grains
Beans
Vegetables
Fruits
Probiotic
Dairy
Yogurt
Alcohol
Water
Fruit juice
Diet drinks
Sweet drinks
Blank
None 7+ days
Within 4−7 days
Within 2−3 days
Yesterday 1−2x
Yesterday 3x+
Immunosupp.
Chemo
Antibiotics
Medication
Hospitalized
Yes
No
FecalCal
HBI
Dysbiosis
Dysbiosis
Eubiosis
Serology
ANCA EU
Cbir1 EU
IgA ASCA EU
IgG ASCA EU
OmpC. EU
MGX Reads
Human
Filtered
MTX Reads
Human
Filtered
Species
Alistipes putredinis
Bacteroides fragilis
Bacteroides ovatus
Bacteroides uniformis
Bacteroides vulgatus
Parabacteroides merdae
Prevotella copri
Subdoligranulum unclassified
Escherichia coli
Klebsiella pneumoniae
other
Transcription
Bacteroides fragilis
Bacteroides ovatus
Bacteroides uniformis
Bacteroides vulgatus
Bacteroides xylanisolvens
Bacteroides massiliensis
Parabacteroides merdae
Ruminococcus obeum
Escherichia coli
other
unclassified
Aerotolerance
obligate_anaerobe
aerotolerant
facultative_anaerobe
unknown
Viruses
Klebsiella phage Matisse
unclassified Myoviridae
Actinomyces virus Av1
Podoviridae
unclassified C2likevirus
Lactococcus phage 936 sensu lato
Klebsiella phage vB KpnS KpV522
Gokushovirus WZ−2015a
other
Transcription
K00566: tRNA−specific 2−thiouridylase [EC:2...
K01358: ATP−dependent Clp protease, prote...
K01867: tryptophanyl−tRNA synthetase [EC:...
K01883: cysteinyl−tRNA synthetase [EC:6.1....
K02078: acyl carrier protein
K02536: UDP−3−O−[3−hydroxymyristoyl] glu...
K03088: RNA polymerase sigma−70 factor, E...
K03704: cold shock protein (beta−ribbon, Cs...
K04564: superoxide dismutase, Fe−Mn famil...
K06078: murein lipoprotein lipoprotein
K19157: NO_NAME
Proteins
Taxonomy
PCo 1 (8.1%)
PCo 2 (6.7%)
Transcripts
PCo 1 (14.2%)
PCo 2 (9.2%)
Proteins
PCo 1 (30.8%)
PCo 2 (12.3%)
Metabolites
PCo 1 (8.8%)
PCo 2 (7.0%)

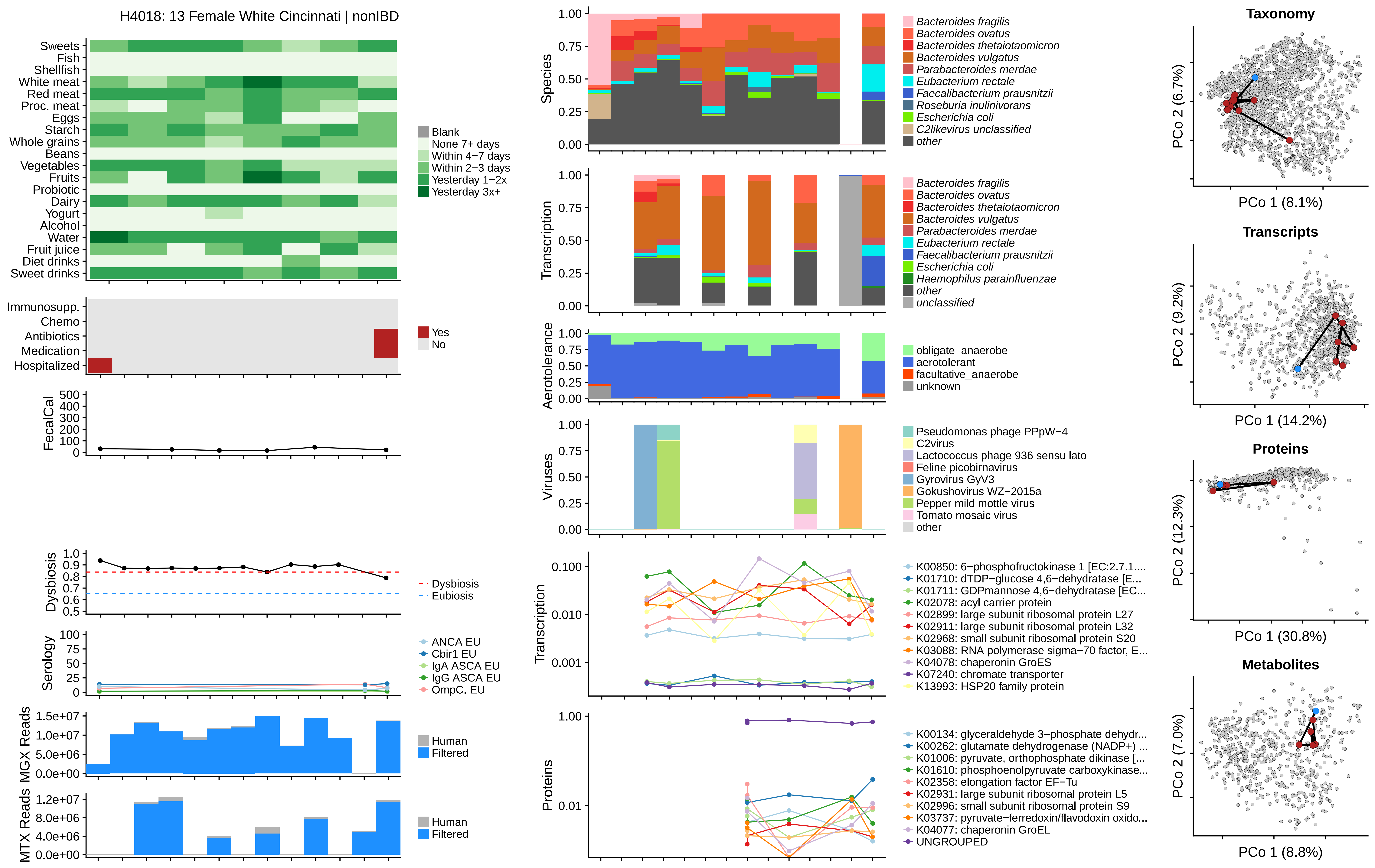

H4018: 13 Female White Cincinnati | nonIBD
Sweets
Fish
Shellfish
White meat
Red meat
Proc. meat
Eggs
Starch
Whole grains
Beans
Vegetables
Fruits
Probiotic
Dairy
Yogurt
Alcohol
Water
Fruit juice
Diet drinks
Sweet drinks
Blank
None 7+ days
Within 4−7 days
Within 2−3 days
Yesterday 1−2x
Yesterday 3x+
Immunosupp.
Chemo
Antibiotics
Medication
Hospitalized
Yes
No
FecalCal
Dysbiosis
Dysbiosis
Eubiosis
Serology
ANCA EU
Cbir1 EU
IgA ASCA EU
IgG ASCA EU
OmpC. EU
MGX Reads
Human
Filtered
MTX Reads
Human
Filtered
Species
Bacteroides fragilis
Bacteroides ovatus
Bacteroides thetaiotaomicron
Bacteroides vulgatus
Parabacteroides merdae
Eubacterium rectale
Faecalibacterium prausnitzii
Roseburia inulinivorans
Escherichia coli
C2likevirus unclassified
other
Transcription
Bacteroides fragilis
Bacteroides ovatus
Bacteroides thetaiotaomicron
Bacteroides vulgatus
Parabacteroides merdae
Eubacterium rectale
Faecalibacterium prausnitzii
Escherichia coli
Haemophilus parainfluenzae
other
unclassified
Aerotolerance
obligate_anaerobe
aerotolerant
facultative_anaerobe
unknown
Viruses
Pseudomonas phage PPpW−4
C2virus
Lactococcus phage 936 sensu lato
Feline picobirnavirus
Gyrovirus GyV3
Gokushovirus WZ−2015a
Pepper mild mottle virus
Tomato mosaic virus
other
Transcription
K00850: 6−phosphofructokinase 1 [EC:2.7.1....
K01710: dTDP−glucose 4,6−dehydratase [E...
K01711: GDPmannose 4,6−dehydratase [EC...
K02078: acyl carrier protein
K02899: large subunit ribosomal protein L27
K02911: large subunit ribosomal protein L32
K02968: small subunit ribosomal protein S20
K03088: RNA polymerase sigma−70 factor, E...
K04078: chaperonin GroES
K07240: chromate transporter
K13993: HSP20 family protein
Proteins
K00134: glyceraldehyde 3−phosphate dehydr...
K00262: glutamate dehydrogenase (NADP+) ...
K01006: pyruvate, orthophosphate dikinase [...
K01610: phosphoenolpyruvate carboxykinase...
K02358: elongation factor EF−Tu
K02931: large subunit ribosomal protein L5
K02996: small subunit ribosomal protein S9
K03737: pyruvate−ferredoxin/flavodoxin oxido...
K04077: chaperonin GroEL
UNGROUPED
Taxonomy
PCo 1 (8.1%)
PCo 2 (6.7%)
Transcripts
PCo 1 (14.2%)
PCo 2 (9.2%)
Proteins
PCo 1 (30.8%)
PCo 2 (12.3%)
Metabolites
PCo 1 (8.8%)
PCo 2 (7.0%)

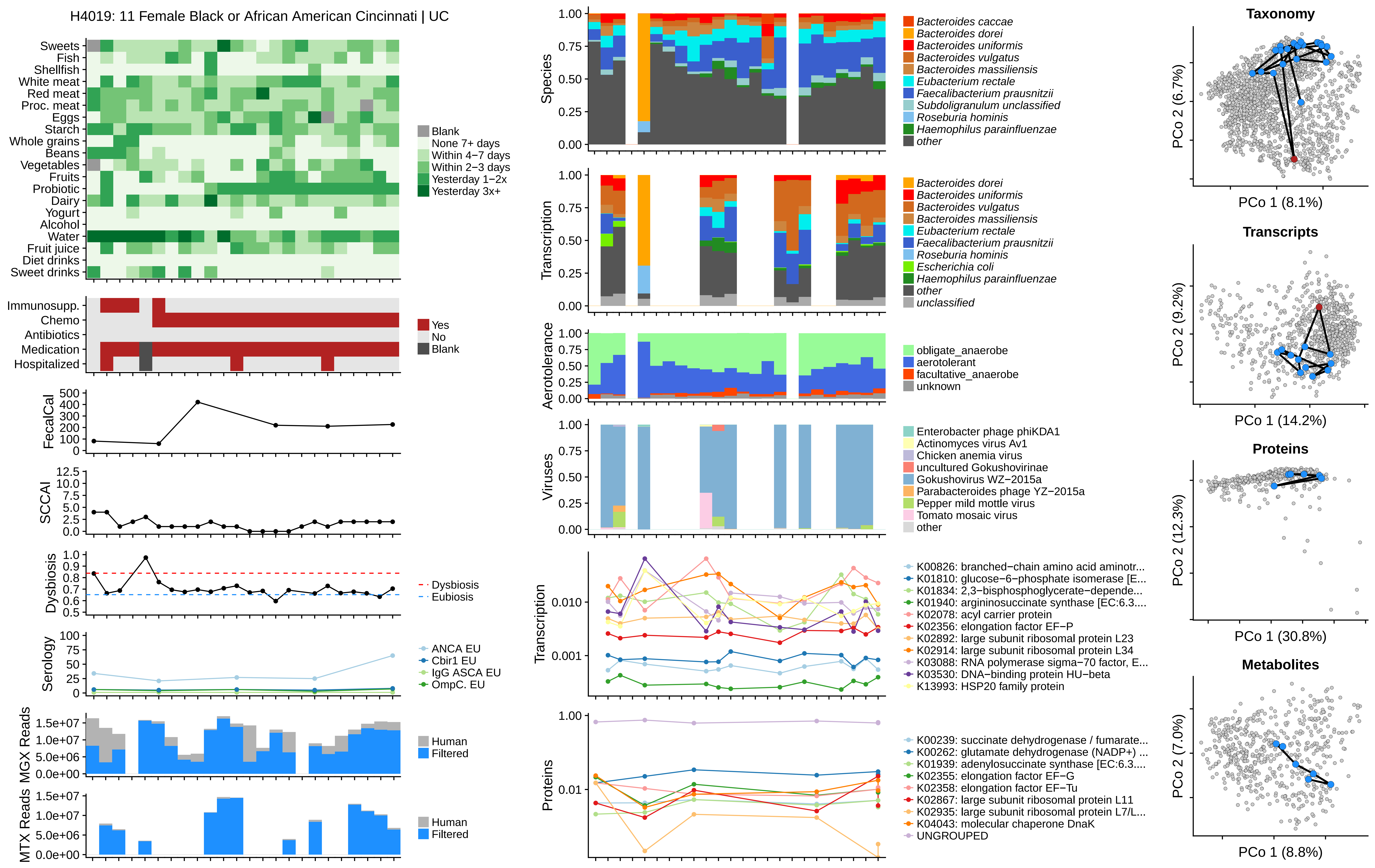
H4019: 11 Female Black or African American Cincinnati | UC
Sweets
Fish
Shellfish
White meat
Red meat
Proc. meat
Eggs
Starch
Whole grains
Beans
Vegetables
Fruits
Probiotic
Dairy
Yogurt
Alcohol
Water
Fruit juice
Diet drinks
Sweet drinks
Blank
None 7+ days
Within 4−7 days
Within 2−3 days
Yesterday 1−2x
Yesterday 3x+
Immunosupp.
Chemo
Antibiotics
Medication
Hospitalized
Yes
No
Blank
FecalCal
SCCAI
Dysbiosis
Dysbiosis
Eubiosis
Serology
ANCA EU
Cbir1 EU
IgG ASCA EU
OmpC. EU
MGX Reads
Human
Filtered
MTX Reads
Human
Filtered
Species
Bacteroides caccae
Bacteroides dorei
Bacteroides uniformis
Bacteroides vulgatus
Bacteroides massiliensis
Eubacterium rectale
Faecalibacterium prausnitzii
Subdoligranulum unclassified
Roseburia hominis
Haemophilus parainfluenzae
other
Transcription
Bacteroides dorei
Bacteroides uniformis
Bacteroides vulgatus
Bacteroides massiliensis
Eubacterium rectale
Faecalibacterium prausnitzii
Roseburia hominis
Escherichia coli
Haemophilus parainfluenzae
other
unclassified
Aerotolerance
obligate_anaerobe
aerotolerant
facultative_anaerobe
unknown
Viruses
Enterobacter phage phiKDA1
Actinomyces virus Av1
Chicken anemia virus
uncultured Gokushovirinae
Gokushovirus WZ−2015a
Parabacteroides phage YZ−2015a
Pepper mild mottle virus
Tomato mosaic virus
other
Transcription
K00826: branched−chain amino acid aminotr...
K01810: glucose−6−phosphate isomerase [E...
K01834: 2,3−bisphosphoglycerate−depende...
K01940: argininosuccinate synthase [EC:6.3....
K02078: acyl carrier protein
K02356: elongation factor EF−P
K02892: large subunit ribosomal protein L23
K02914: large subunit ribosomal protein L34
K03088: RNA polymerase sigma−70 factor, E...
K03530: DNA−binding protein HU−beta
K13993: HSP20 family protein
Proteins
K00239: succinate dehydrogenase / fumarate...
K00262: glutamate dehydrogenase (NADP+) ...
K01939: adenylosuccinate synthase [EC:6.3....
K02355: elongation factor EF−G
K02358: elongation factor EF−Tu
K02867: large subunit ribosomal protein L11
K02935: large subunit ribosomal protein L7/L...
K04043: molecular chaperone DnaK
UNGROUPED
Taxonomy
PCo 1 (8.1%)
PCo 2 (6.7%)
Transcripts
PCo 1 (14.2%)
PCo 2 (9.2%)
Proteins
PCo 1 (30.8%)
PCo 2 (12.3%)
Metabolites
PCo 1 (8.8%)
PCo 2 (7.0%)

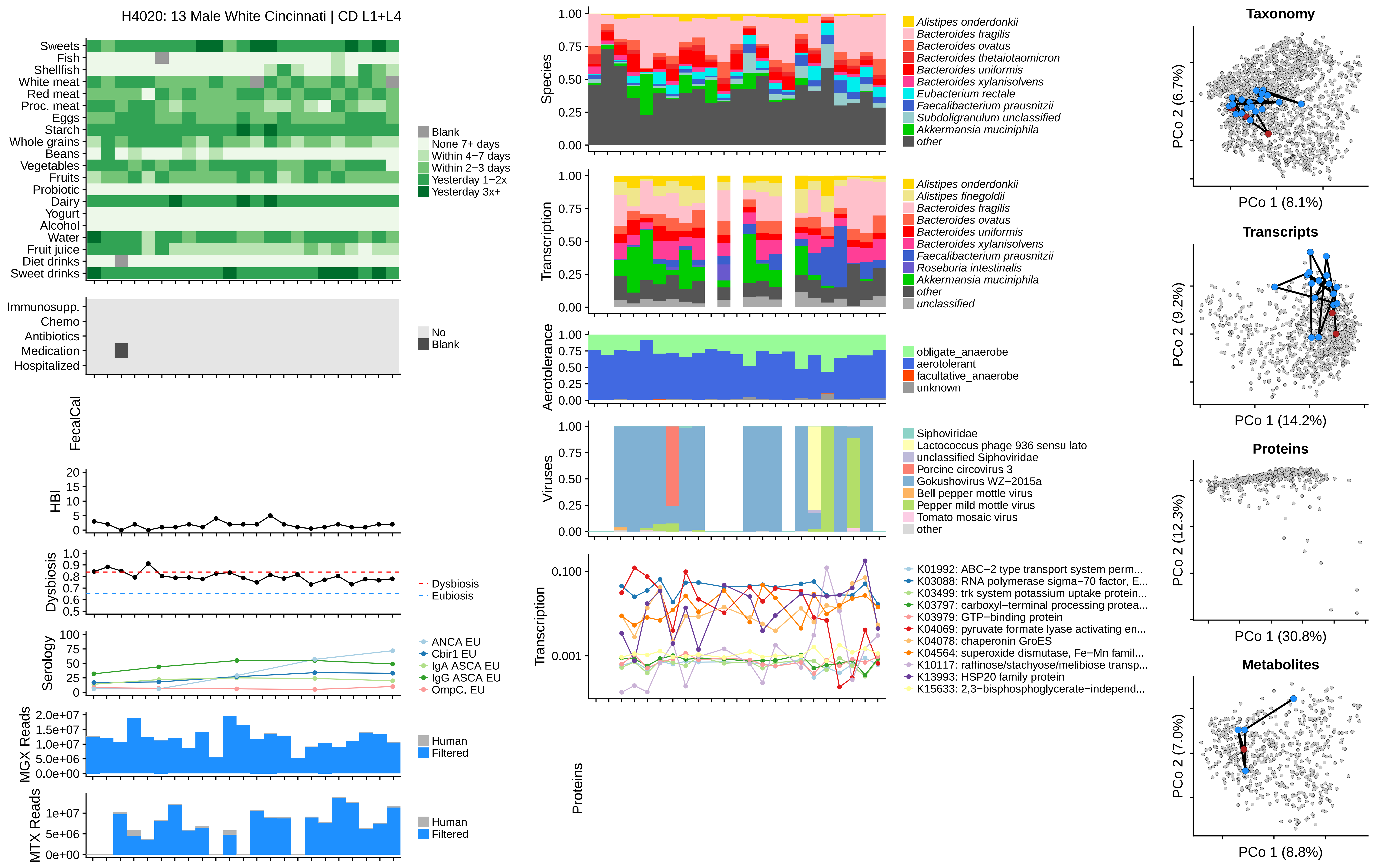

H4020: 13 Male White Cincinnati | CD L1+L4
Sweets
Fish
Shellfish
White meat
Red meat
Proc. meat
Eggs
Starch
Whole grains
Beans
Vegetables
Fruits
Probiotic
Dairy
Yogurt
Alcohol
Water
Fruit juice
Diet drinks
Sweet drinks
Blank
None 7+ days
Within 4−7 days
Within 2−3 days
Yesterday 1−2x
Yesterday 3x+
Immunosupp.
Chemo
Antibiotics
Medication
Hospitalized
No
Blank
FecalCal
HBI
Dysbiosis
Dysbiosis
Eubiosis
Serology
ANCA EU
Cbir1 EU
IgA ASCA EU
IgG ASCA EU
OmpC. EU
MGX Reads
Human
Filtered
MTX Reads
Human
Filtered
Species
Alistipes onderdonkii
Bacteroides fragilis
Bacteroides ovatus
Bacteroides thetaiotaomicron
Bacteroides uniformis
Bacteroides xylanisolvens
Eubacterium rectale
Faecalibacterium prausnitzii
Subdoligranulum unclassified
Akkermansia muciniphila
other
Transcription
Alistipes onderdonkii
Alistipes finegoldii
Bacteroides fragilis
Bacteroides ovatus
Bacteroides uniformis
Bacteroides xylanisolvens
Faecalibacterium prausnitzii
Roseburia intestinalis
Akkermansia muciniphila
other
unclassified
Aerotolerance
obligate_anaerobe
aerotolerant
facultative_anaerobe
unknown
Viruses
Siphoviridae
Lactococcus phage 936 sensu lato
unclassified Siphoviridae
Porcine circovirus 3
Gokushovirus WZ−2015a
Bell pepper mottle virus
Pepper mild mottle virus
Tomato mosaic virus
other
Transcription
K01992: ABC−2 type transport system perm...
K03088: RNA polymerase sigma−70 factor, E...
K03499: trk system potassium uptake protein...
K03797: carboxyl−terminal processing protea...
K03979: GTP−binding protein
K04069: pyruvate formate lyase activating en...
K04078: chaperonin GroES
K04564: superoxide dismutase, Fe−Mn famil...
K10117: raffinose/stachyose/melibiose transp...
K13993: HSP20 family protein
K15633: 2,3−bisphosphoglycerate−independ...
Proteins
Taxonomy
PCo 1 (8.1%)
PCo 2 (6.7%)
Transcripts
PCo 1 (14.2%)
PCo 2 (9.2%)
Proteins
PCo 1 (30.8%)
PCo 2 (12.3%)
Metabolites
PCo 1 (8.8%)
PCo 2 (7.0%)

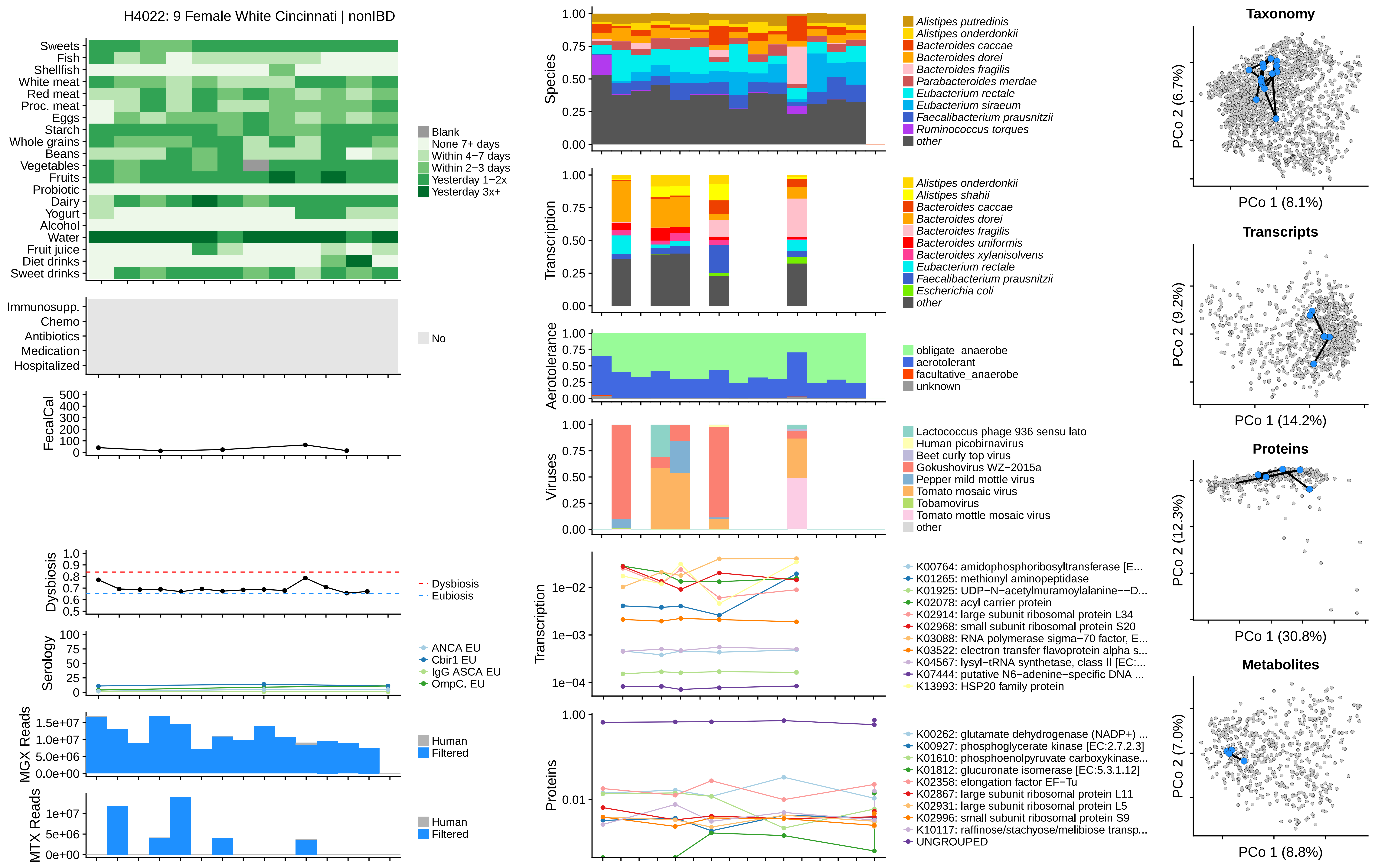
H4022: 9 Female White Cincinnati | nonIBD
Sweets
Fish
Shellfish
White meat
Red meat
Proc. meat
Eggs
Starch
Whole grains
Beans
Vegetables
Fruits
Probiotic
Dairy
Yogurt
Alcohol
Water
Fruit juice
Diet drinks
Sweet drinks
Blank
None 7+ days
Within 4−7 days
Within 2−3 days
Yesterday 1−2x
Yesterday 3x+
Immunosupp.
Chemo
Antibiotics
Medication
Hospitalized
No
FecalCal
Dysbiosis
Dysbiosis
Eubiosis
Serology
ANCA EU
Cbir1 EU
IgG ASCA EU
OmpC. EU
MGX Reads
Human
Filtered
MTX Reads
Human
Filtered
Species
Alistipes putredinis
Alistipes onderdonkii
Bacteroides caccae
Bacteroides dorei
Bacteroides fragilis
Parabacteroides merdae
Eubacterium rectale
Eubacterium siraeum
Faecalibacterium prausnitzii
Ruminococcus torques
other
Transcription
Alistipes onderdonkii
Alistipes shahii
Bacteroides caccae
Bacteroides dorei
Bacteroides fragilis
Bacteroides uniformis
Bacteroides xylanisolvens
Eubacterium rectale
Faecalibacterium prausnitzii
Escherichia coli
other
Aerotolerance
obligate_anaerobe
aerotolerant
facultative_anaerobe
unknown
Viruses
Lactococcus phage 936 sensu lato
Human picobirnavirus
Beet curly top virus
Gokushovirus WZ−2015a
Pepper mild mottle virus
Tomato mosaic virus
Tobamovirus
Tomato mottle mosaic virus
other
Transcription
K00764: amidophosphoribosyltransferase [E...
K01265: methionyl aminopeptidase
K01925: UDP−N−acetylmuramoylalanine−−D...
K02078: acyl carrier protein
K02914: large subunit ribosomal protein L34
K02968: small subunit ribosomal protein S20
K03088: RNA polymerase sigma−70 factor, E...
K03522: electron transfer flavoprotein alpha s...
K04567: lysyl−tRNA synthetase, class II [EC:...
K07444: putative N6−adenine−specific DNA ...
K13993: HSP20 family protein
Proteins
K00262: glutamate dehydrogenase (NADP+) ...
K00927: phosphoglycerate kinase [EC:2.7.2.3]
K01610: phosphoenolpyruvate carboxykinase...
K01812: glucuronate isomerase [EC:5.3.1.12]
K02358: elongation factor EF−Tu
K02867: large subunit ribosomal protein L11
K02931: large subunit ribosomal protein L5
K02996: small subunit ribosomal protein S9
K10117: raffinose/stachyose/melibiose transp...
UNGROUPED
Taxonomy
PCo 1 (8.1%)
PCo 2 (6.7%)
Transcripts
PCo 1 (14.2%)
PCo 2 (9.2%)
Proteins
PCo 1 (30.8%)
PCo 2 (12.3%)
Metabolites
PCo 1 (8.8%)
PCo 2 (7.0%)

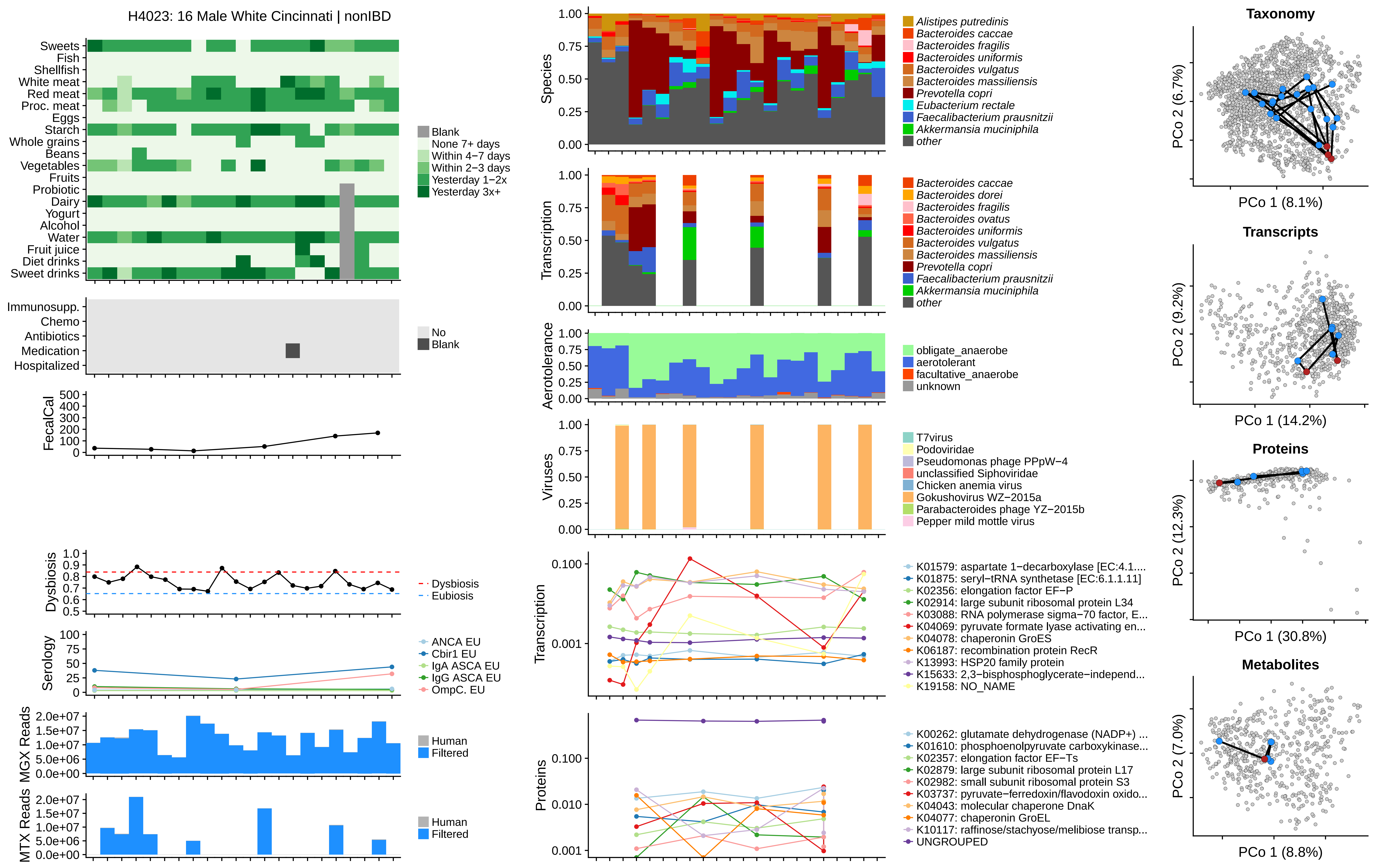
H4023: 16 Male White Cincinnati | nonIBD
Sweets
Fish
Shellfish
White meat
Red meat
Proc. meat
Eggs
Starch
Whole grains
Beans
Vegetables
Fruits
Probiotic
Dairy
Yogurt
Alcohol
Water
Fruit juice
Diet drinks
Sweet drinks
Blank
None 7+ days
Within 4−7 days
Within 2−3 days
Yesterday 1−2x
Yesterday 3x+
Immunosupp.
Chemo
Antibiotics
Medication
Hospitalized
No
Blank
FecalCal
Dysbiosis
Eubiosis
Serology
ANCA EU
Cbir1 EU
IgA ASCA EU
IgG ASCA EU
OmpC. EU
MGX Reads
MTX Reads
Human
Filtered
Species
Alistipes putredinis
Bacteroides caccae
Bacteroides fragilis
Bacteroides uniformis
Bacteroides vulgatus
Bacteroides massiliensis
Prevotella copri
Eubacterium rectale
Faecalibacterium prausnitzii
Akkermansia muciniphila
other
Transcription
Bacteroides dorei
Bacteroides ovatus
Aerotolerance
obligate_anaerobe
aerotolerant
facultative_anaerobe
unknown
Viruses
T7virus
Podoviridae
Pseudomonas phage PPpW−4
unclassified Siphoviridae
Chicken anemia virus
Gokushovirus WZ−2015a
Parabacteroides phage YZ−2015b
Pepper mild mottle virus
K01579: aspartate 1−decarboxylase [EC:4.1....
K01875: seryl−tRNA synthetase [EC:6.1.1.11]
K02356: elongation factor EF−P
K02914: large subunit ribosomal protein L34
K03088: RNA polymerase sigma−70 factor, E...
K04069: pyruvate formate lyase activating en...
K04078: chaperonin GroES
K06187: recombination protein RecR
K13993: HSP20 family protein
K15633: 2,3−bisphosphoglycerate−independ...
K19158: NO_NAME
Proteins
K00262: glutamate dehydrogenase (NADP+) ...
K01610: phosphoenolpyruvate carboxykinase...
K02357: elongation factor EF−Ts
K02879: large subunit ribosomal protein L17
K02982: small subunit ribosomal protein S3
K03737: pyruvate−ferredoxin/flavodoxin oxido...
K04043: molecular chaperone DnaK
K04077: chaperonin GroEL
K10117: raffinose/stachyose/melibiose transp...
UNGROUPED
Taxonomy
PCo 1 (8.1%)
PCo 2 (6.7%)
Transcripts
PCo 1 (14.2%)
PCo 2 (9.2%)
Proteins
PCo 1 (30.8%)
PCo 2 (12.3%)
Metabolites
PCo 1 (8.8%)
PCo 2 (7.0%)

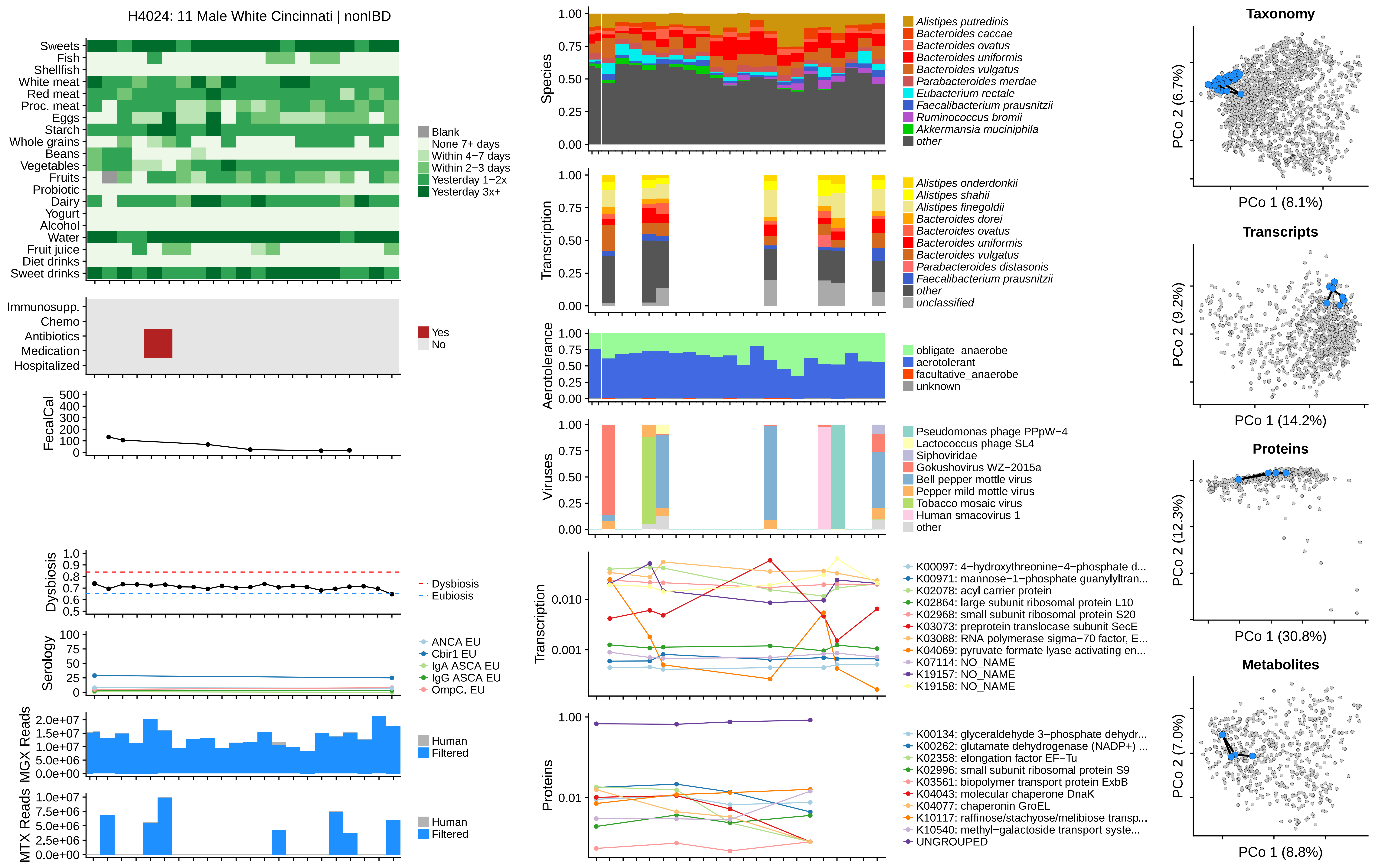
H4024: 11 Male White Cincinnati | nonIBD
Sweets
Fish
Shellfish
White meat
Red meat
Proc. meat
Eggs
Starch
Whole grains
Beans
Vegetables
Fruits
Probiotic
Dairy
Yogurt
Alcohol
Water
Fruit juice
Diet drinks
Sweet drinks
Blank
None 7+ days
Within 4−7 days
Within 2−3 days
Yesterday 1−2x
Yesterday 3x+
Immunosupp.
Chemo
Antibiotics
Medication
Hospitalized
Yes
No
FecalCal
Dysbiosis
Dysbiosis
Eubiosis
Serology
ANCA EU
Cbir1 EU
IgA ASCA EU
IgG ASCA EU
OmpC. EU
MGX Reads
Human
Filtered
MTX Reads
Human
Filtered
Species
Alistipes putredinis
Bacteroides caccae
Bacteroides ovatus
Bacteroides uniformis
Bacteroides vulgatus
Parabacteroides merdae
Eubacterium rectale
Faecalibacterium prausnitzii
Ruminococcus bromii
Akkermansia muciniphila
other
Transcription
Alistipes onderdonkii
Alistipes shahii
Alistipes finegoldii
Bacteroides dorei
Bacteroides ovatus
Bacteroides uniformis
Bacteroides vulgatus
Parabacteroides distasonis
Faecalibacterium prausnitzii
other
unclassified
Aerotolerance
obligate_anaerobe
aerotolerant
facultative_anaerobe
unknown
Viruses
Pseudomonas phage PPpW−4
Lactococcus phage SL4
Siphoviridae
Gokushovirus WZ−2015a
Bell pepper mottle virus
Pepper mild mottle virus
Tobacco mosaic virus
Human smacovirus 1
other
Transcription
K00097: 4−hydroxythreonine−4−phosphate d...
K00971: mannose−1−phosphate guanylyltran...
K02078: acyl carrier protein
K02864: large subunit ribosomal protein L10
K02968: small subunit ribosomal protein S20
K03073: preprotein translocase subunit SecE
K03088: RNA polymerase sigma−70 factor, E...
K04069: pyruvate formate lyase activating en...
K07114: NO_NAME
K19157: NO_NAME
K19158: NO_NAME
Proteins
K00134: glyceraldehyde 3−phosphate dehydr...
K00262: glutamate dehydrogenase (NADP+) ...
K02358: elongation factor EF−Tu
K02996: small subunit ribosomal protein S9
K03561: biopolymer transport protein ExbB
K04043: molecular chaperone DnaK
K04077: chaperonin GroEL
K10117: raffinose/stachyose/melibiose transp...
K10540: methyl−galactoside transport syste...
UNGROUPED
Taxonomy
PCo 1 (8.1%)
PCo 2 (6.7%)
Transcripts
PCo 1 (14.2%)
PCo 2 (9.2%)
Proteins
PCo 1 (30.8%)
PCo 2 (12.3%)
Metabolites
PCo 1 (8.8%)
PCo 2 (7.0%)

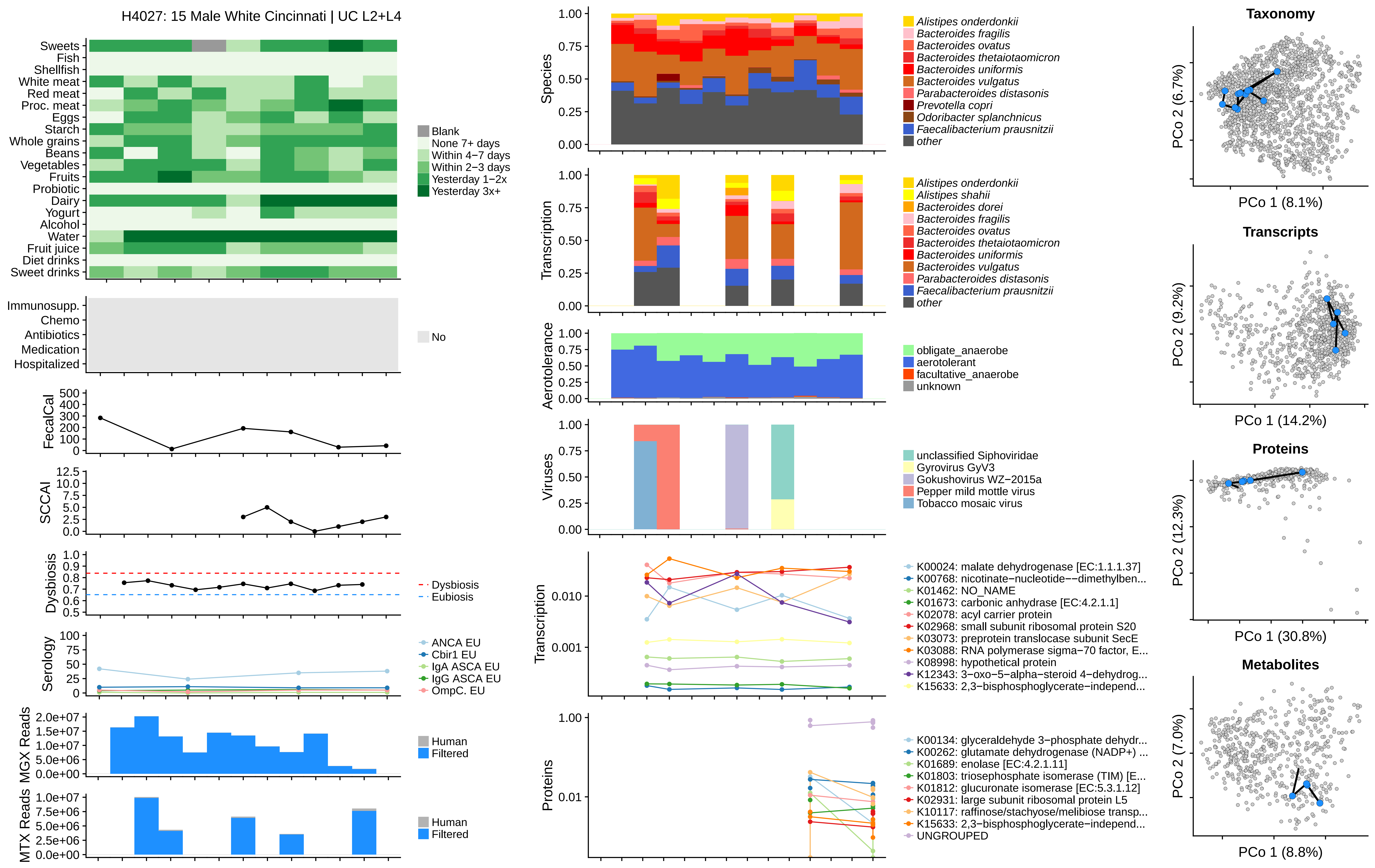

H4027: 15 Male White Cincinnati | UC L2+L4
Sweets
Fish
Shellfish
White meat
Red meat
Proc. meat
Eggs
Starch
Whole grains
Beans
Vegetables
Fruits
Probiotic
Dairy
Yogurt
Alcohol
Water
Fruit juice
Diet drinks
Sweet drinks
Blank
None 7+ days
Within 4−7 days
Within 2−3 days
Yesterday 1−2x
Yesterday 3x+
Immunosupp.
Chemo
Antibiotics
Medication
Hospitalized
No
FecalCal
SCCAI
Dysbiosis
Dysbiosis
Eubiosis
Serology
ANCA EU
Cbir1 EU
IgA ASCA EU
IgG ASCA EU
OmpC. EU
MGX Reads
Human
Filtered
MTX Reads
Human
Filtered
Species
Alistipes onderdonkii
Bacteroides fragilis
Bacteroides ovatus
Bacteroides thetaiotaomicron
Bacteroides uniformis
Bacteroides vulgatus
Parabacteroides distasonis
Prevotella copri
Odoribacter splanchnicus
Faecalibacterium prausnitzii
other
Transcription
Alistipes onderdonkii
Alistipes shahii
Bacteroides dorei
Bacteroides fragilis
Bacteroides ovatus
Bacteroides thetaiotaomicron
Bacteroides uniformis
Bacteroides vulgatus
Parabacteroides distasonis
Faecalibacterium prausnitzii
other
Aerotolerance
obligate_anaerobe
aerotolerant
facultative_anaerobe
unknown
Viruses
unclassified Siphoviridae
Gyrovirus GyV3
Gokushovirus WZ−2015a
Pepper mild mottle virus
Tobacco mosaic virus
Transcription
K00024: malate dehydrogenase [EC:1.1.1.37]
K00768: nicotinate−nucleotide−−dimethylben...
K01462: NO_NAME
K01673: carbonic anhydrase [EC:4.2.1.1]
K02078: acyl carrier protein
K02968: small subunit ribosomal protein S20
K03073: preprotein translocase subunit SecE
K03088: RNA polymerase sigma−70 factor, E...
K08998: hypothetical protein
K12343: 3−oxo−5−alpha−steroid 4−dehydrog...
K15633: 2,3−bisphosphoglycerate−independ...
Proteins
K00134: glyceraldehyde 3−phosphate dehydr...
K00262: glutamate dehydrogenase (NADP+) ...
K01689: enolase [EC:4.2.1.11]
K01803: triosephosphate isomerase (TIM) [E...
K01812: glucuronate isomerase [EC:5.3.1.12]
K02931: large subunit ribosomal protein L5
K10117: raffinose/stachyose/melibiose transp...
K15633: 2,3−bisphosphoglycerate−independ...
UNGROUPED
Taxonomy
PCo 1 (8.1%)
PCo 2 (6.7%)
Transcripts
PCo 1 (14.2%)
PCo 2 (9.2%)
Proteins
PCo 1 (30.8%)
PCo 2 (12.3%)
Metabolites
PCo 1 (8.8%)
PCo 2 (7.0%)

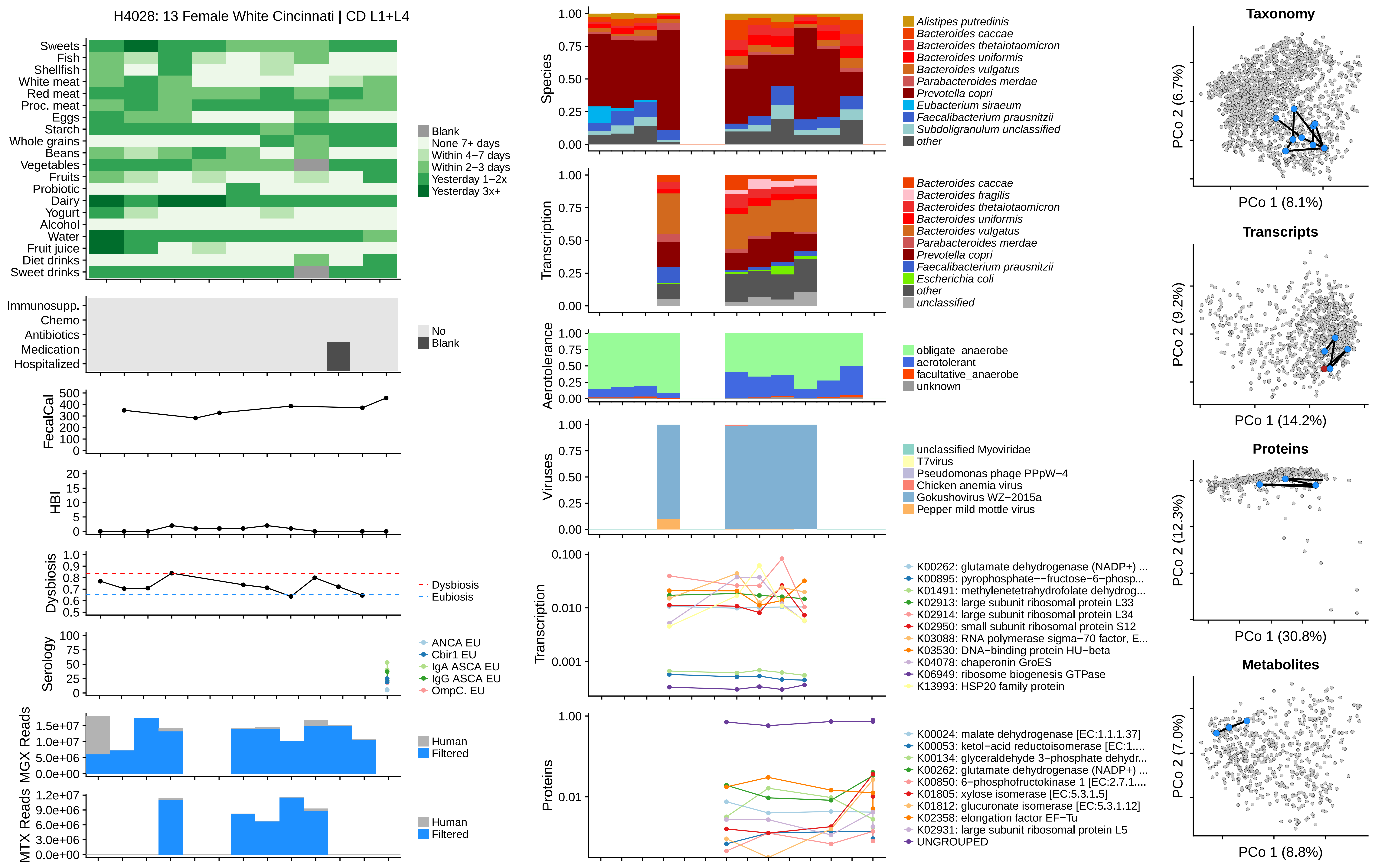

H4028: 13 Female White Cincinnati | CD L1+L4
Sweets
Fish
Shellfish
White meat
Red meat
Proc. meat
Eggs
Starch
Whole grains
Beans
Vegetables
Fruits
Probiotic
Dairy
Yogurt
Alcohol
Water
Fruit juice
Diet drinks
Sweet drinks
Blank
None 7+ days
Within 4−7 days
Within 2−3 days
Yesterday 1−2x
Yesterday 3x+
Immunosupp.
Chemo
Antibiotics
Medication
Hospitalized
No
Blank
FecalCal
HBI
Dysbiosis
Dysbiosis
Eubiosis
Serology
ANCA EU
Cbir1 EU
IgA ASCA EU
IgG ASCA EU
OmpC. EU
MGX Reads
Human
Filtered
MTX Reads
Human
Filtered
Species
Alistipes putredinis
Bacteroides caccae
Bacteroides thetaiotaomicron
Bacteroides uniformis
Bacteroides vulgatus
Parabacteroides merdae
Prevotella copri
Eubacterium siraeum
Faecalibacterium prausnitzii
Subdoligranulum unclassified
other
Transcription
Bacteroides caccae
Bacteroides fragilis
Bacteroides thetaiotaomicron
Bacteroides uniformis
Bacteroides vulgatus
Parabacteroides merdae
Prevotella copri
Faecalibacterium prausnitzii
Escherichia coli
other
unclassified
Aerotolerance
obligate_anaerobe
aerotolerant
facultative_anaerobe
unknown
Viruses
unclassified Myoviridae
T7virus
Pseudomonas phage PPpW−4
Chicken anemia virus
Gokushovirus WZ−2015a
Pepper mild mottle virus
Transcription
K00262: glutamate dehydrogenase (NADP+) ...
K00895: pyrophosphate−−fructose−6−phosp...
K01491: methylenetetrahydrofolate dehydrog...
K02913: large subunit ribosomal protein L33
K02914: large subunit ribosomal protein L34
K02950: small subunit ribosomal protein S12
K03088: RNA polymerase sigma−70 factor, E...
K03530: DNA−binding protein HU−beta
K04078: chaperonin GroES
K06949: ribosome biogenesis GTPase
K13993: HSP20 family protein
Proteins
K00024: malate dehydrogenase [EC:1.1.1.37]
K00053: ketol−acid reductoisomerase [EC:1....
K00134: glyceraldehyde 3−phosphate dehydr...
K00262: glutamate dehydrogenase (NADP+) ...
K00850: 6−phosphofructokinase 1 [EC:2.7.1....
K01805: xylose isomerase [EC:5.3.1.5]
K01812: glucuronate isomerase [EC:5.3.1.12]
K02358: elongation factor EF−Tu
K02931: large subunit ribosomal protein L5
UNGROUPED
Taxonomy
PCo 1 (8.1%)
PCo 2 (6.7%)
Transcripts
PCo 1 (14.2%)
PCo 2 (9.2%)
Proteins
PCo 1 (30.8%)
PCo 2 (12.3%)
Metabolites
PCo 1 (8.8%)
PCo 2 (7.0%)

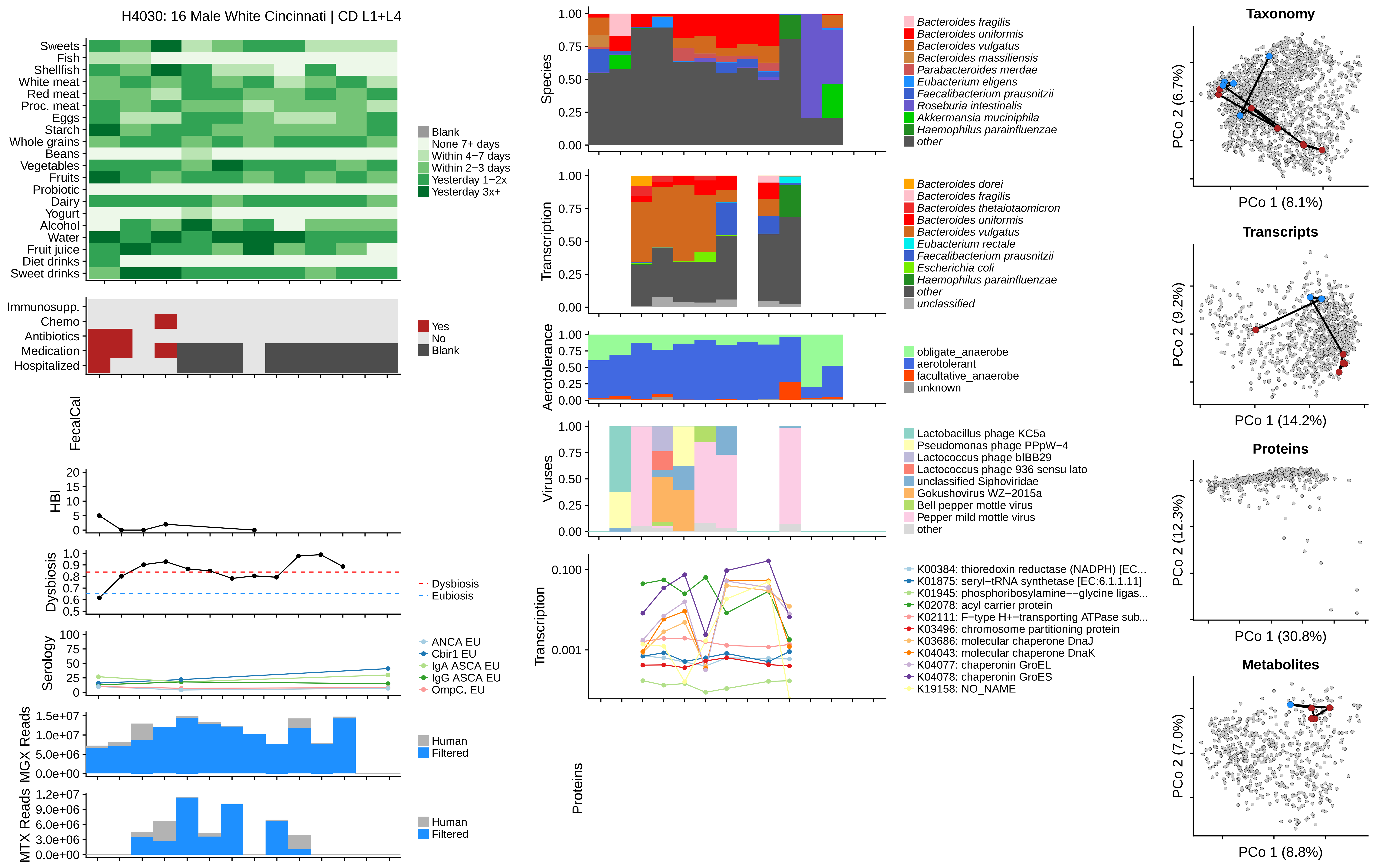

H4030: 16 Male White Cincinnati | CD L1+L4
Sweets
Fish
Shellfish
White meat
Red meat
Proc. meat
Eggs
Starch
Whole grains
Beans
Vegetables
Fruits
Probiotic
Dairy
Yogurt
Alcohol
Water
Fruit juice
Diet drinks
Sweet drinks
Blank
None 7+ days
Within 4−7 days
Within 2−3 days
Yesterday 1−2x
Yesterday 3x+
Immunosupp.
Chemo
Antibiotics
Medication
Hospitalized
Yes
No
Blank
FecalCal
HBI
Dysbiosis
Dysbiosis
Eubiosis
Serology
ANCA EU
Cbir1 EU
IgA ASCA EU
IgG ASCA EU
OmpC. EU
MGX Reads
Human
Filtered
MTX Reads
Human
Filtered
Species
Bacteroides fragilis
Bacteroides uniformis
Bacteroides vulgatus
Bacteroides massiliensis
Parabacteroides merdae
Eubacterium eligens
Faecalibacterium prausnitzii
Roseburia intestinalis
Akkermansia muciniphila
Haemophilus parainfluenzae
other
Transcription
Bacteroides dorei
Bacteroides fragilis
Bacteroides thetaiotaomicron
Bacteroides uniformis
Bacteroides vulgatus
Eubacterium rectale
Faecalibacterium prausnitzii
Escherichia coli
Haemophilus parainfluenzae
other
unclassified
Aerotolerance
obligate_anaerobe
aerotolerant
facultative_anaerobe
unknown
Viruses
Lactobacillus phage KC5a
Pseudomonas phage PPpW−4
Lactococcus phage bIBB29
Lactococcus phage 936 sensu lato
unclassified Siphoviridae
Gokushovirus WZ−2015a
Bell pepper mottle virus
Pepper mild mottle virus
other
Transcription
K00384: thioredoxin reductase (NADPH) [EC...
K01875: seryl−tRNA synthetase [EC:6.1.1.11]
K01945: phosphoribosylamine−−glycine ligas...
K02078: acyl carrier protein
K02111: F−type H+−transporting ATPase sub...
K03496: chromosome partitioning protein
K03686: molecular chaperone DnaJ
K04043: molecular chaperone DnaK
K04077: chaperonin GroEL
K04078: chaperonin GroES
K19158: NO_NAME
Proteins
Taxonomy
PCo 1 (8.1%)
PCo 2 (6.7%)
Transcripts
PCo 1 (14.2%)
PCo 2 (9.2%)
Proteins
PCo 1 (30.8%)
PCo 2 (12.3%)
Metabolites
PCo 1 (8.8%)
PCo 2 (7.0%)

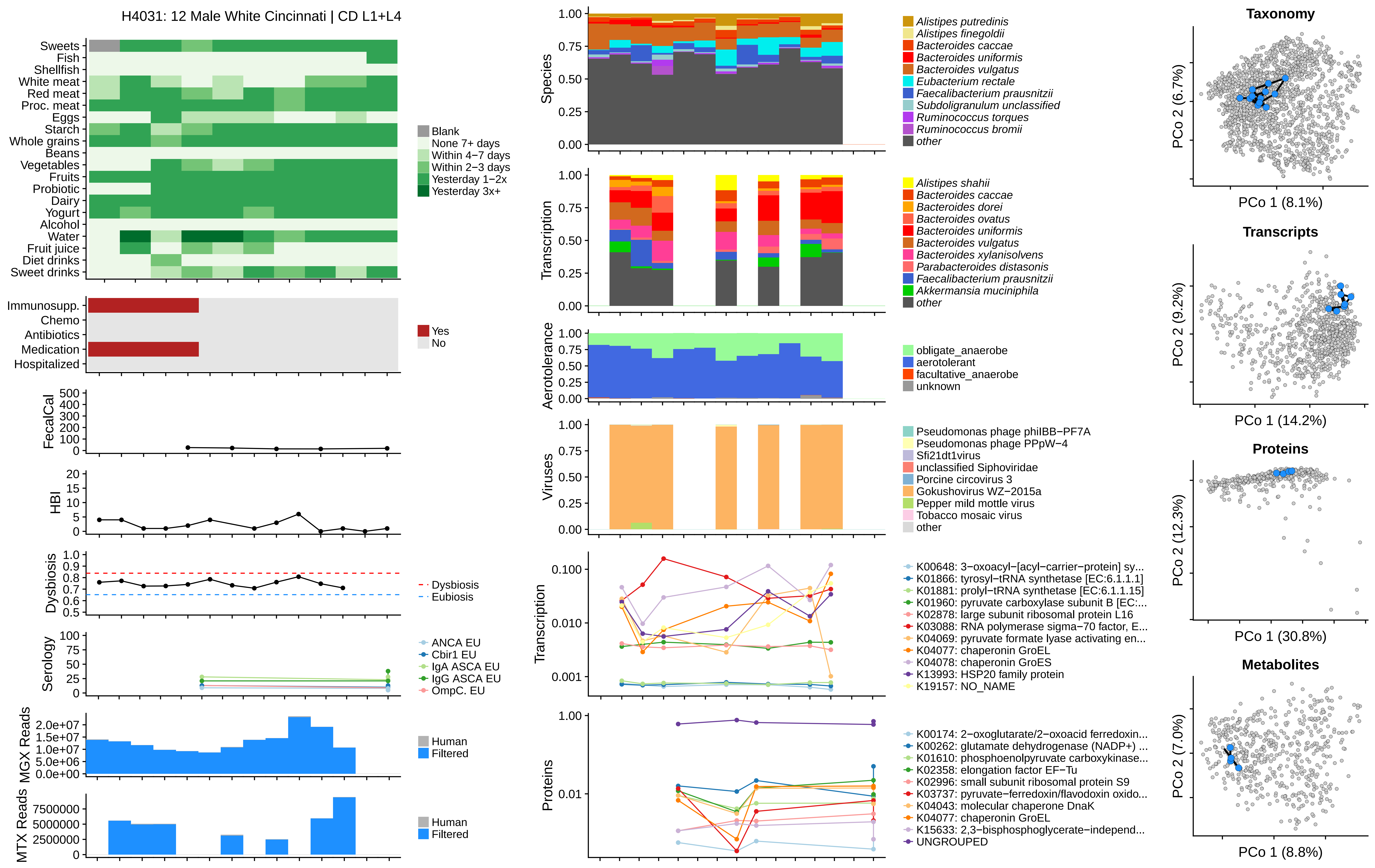
H4031: 12 Male White Cincinnati | CD L1+L4
Sweets
Fish
Shellfish
White meat
Red meat
Proc. meat
Eggs
Starch
Whole grains
Beans
Vegetables
Fruits
Probiotic
Dairy
Yogurt
Alcohol
Water
Fruit juice
Diet drinks
Sweet drinks
Blank
None 7+ days
Within 4−7 days
Within 2−3 days
Yesterday 1−2x
Yesterday 3x+
Immunosupp.
Chemo
Antibiotics
Medication
Hospitalized
Yes
No
FecalCal
HBI
Dysbiosis
Dysbiosis
Eubiosis
Serology
ANCA EU
Cbir1 EU
IgA ASCA EU
IgG ASCA EU
OmpC. EU
MGX Reads
Human
Filtered
MTX Reads
Human
Filtered
Species
Alistipes putredinis
Alistipes finegoldii
Bacteroides caccae
Bacteroides uniformis
Bacteroides vulgatus
Eubacterium rectale
Faecalibacterium prausnitzii
Subdoligranulum unclassified
Ruminococcus torques
Ruminococcus bromii
other
Transcription
Alistipes shahii
Bacteroides caccae
Bacteroides dorei
Bacteroides ovatus
Bacteroides uniformis
Bacteroides vulgatus
Bacteroides xylanisolvens
Parabacteroides distasonis
Faecalibacterium prausnitzii
Akkermansia muciniphila
other
Aerotolerance
obligate_anaerobe
aerotolerant
facultative_anaerobe
unknown
Viruses
Pseudomonas phage philBB−PF7A
Pseudomonas phage PPpW−4
Sfi21dt1virus
unclassified Siphoviridae
Porcine circovirus 3
Gokushovirus WZ−2015a
Pepper mild mottle virus
Tobacco mosaic virus
other
Transcription
K00648: 3−oxoacyl−[acyl−carrier−protein] sy...
K01866: tyrosyl−tRNA synthetase [EC:6.1.1.1]
K01881: prolyl−tRNA synthetase [EC:6.1.1.15]
K01960: pyruvate carboxylase subunit B [EC:...
K02878: large subunit ribosomal protein L16
K03088: RNA polymerase sigma−70 factor, E...
K04069: pyruvate formate lyase activating en...
K04077: chaperonin GroEL
K04078: chaperonin GroES
K13993: HSP20 family protein
K19157: NO_NAME
Proteins
K00174: 2−oxoglutarate/2−oxoacid ferredoxin...
K00262: glutamate dehydrogenase (NADP+) ...
K01610: phosphoenolpyruvate carboxykinase...
K02358: elongation factor EF−Tu
K02996: small subunit ribosomal protein S9
K03737: pyruvate−ferredoxin/flavodoxin oxido...
K04043: molecular chaperone DnaK
K04077: chaperonin GroEL
K15633: 2,3−bisphosphoglycerate−independ...
UNGROUPED
Taxonomy
PCo 2 (6.7%)
PCo 1 (8.1%)
Transcripts
PCo 2 (9.2%)
PCo 1 (14.2%)
Proteins
PCo 2 (12.3%)
PCo 1 (30.8%)
Metabolites
PCo 2 (7.0%)
PCo 1 (8.8%)

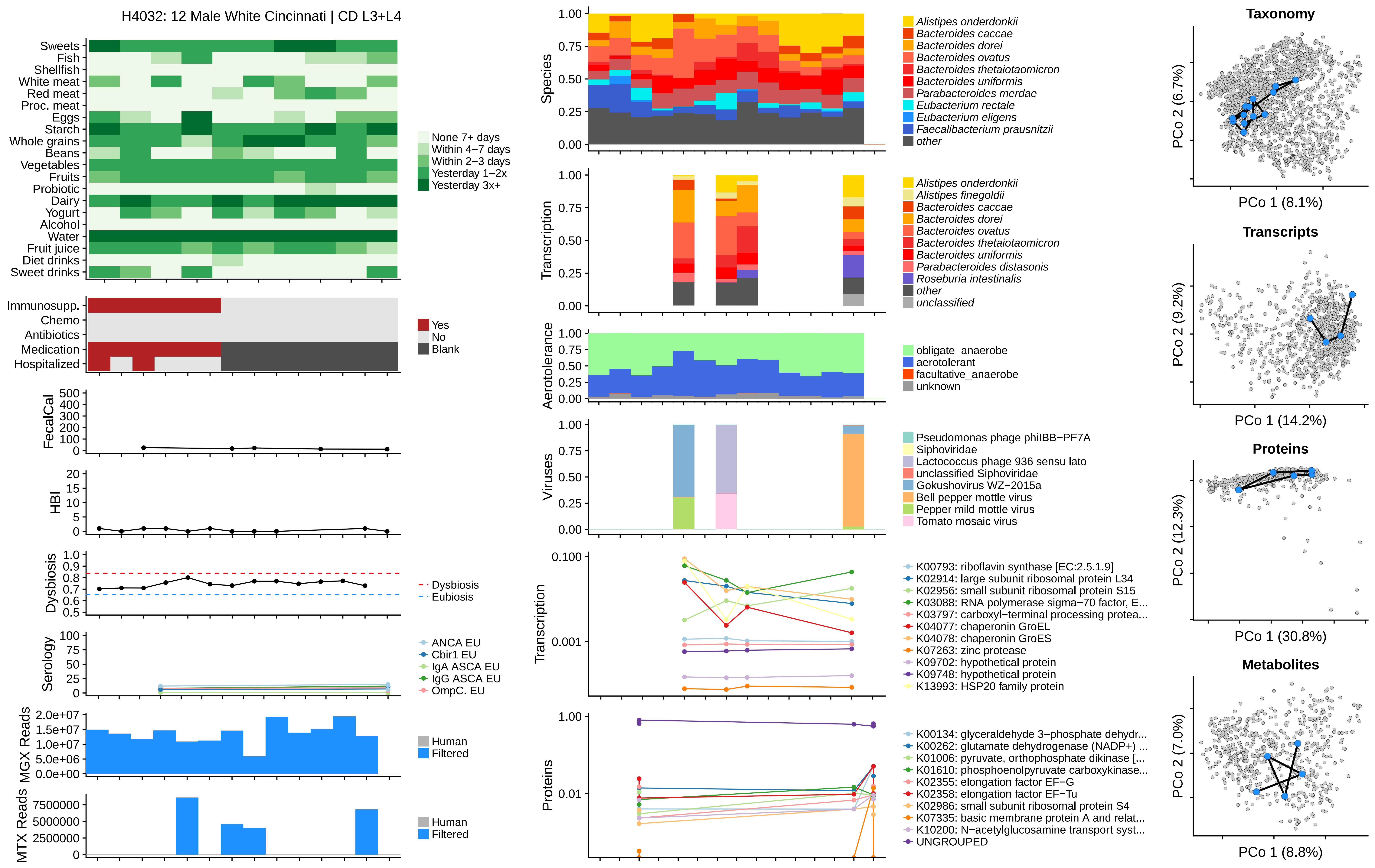
H4032: 12 Male White Cincinnati | CD L3+L4
Sweets
Fish
Shellfish
White meat
Red meat
Proc. meat
Eggs
Starch
Whole grains
Beans
Vegetables
Fruits
Probiotic
Dairy
Yogurt
Alcohol
Water
Fruit juice
Diet drinks
Sweet drinks
None 7+ days
Within 4−7 days
Within 2−3 days
Yesterday 1−2x
Yesterday 3x+
Immunosupp.
Chemo
Antibiotics
Medication
Hospitalized
Yes
No
Blank
FecalCal
HBI
Dysbiosis
Dysbiosis
Eubiosis
Serology
ANCA EU
Cbir1 EU
IgA ASCA EU
IgG ASCA EU
OmpC. EU
MGX Reads
Human
Filtered
MTX Reads
Human
Filtered
Species
Alistipes onderdonkii
Bacteroides caccae
Bacteroides dorei
Bacteroides ovatus
Bacteroides thetaiotaomicron
Bacteroides uniformis
Parabacteroides merdae
Eubacterium rectale
Eubacterium eligens
Faecalibacterium prausnitzii
other
Transcription
Alistipes onderdonkii
Alistipes finegoldii
Bacteroides caccae
Bacteroides dorei
Bacteroides ovatus
Bacteroides thetaiotaomicron
Bacteroides uniformis
Parabacteroides distasonis
Roseburia intestinalis
other
unclassified
Aerotolerance
obligate_anaerobe
aerotolerant
facultative_anaerobe
unknown
Viruses
Pseudomonas phage phiIBB−PF7A
Siphoviridae
Lactococcus phage 936 sensu lato
unclassified Siphoviridae
Gokushovirus WZ−2015a
Bell pepper mottle virus
Pepper mild mottle virus
Tomato mosaic virus
Transcription
K00793: riboflavin synthase [EC:2.5.1.9]
K02914: large subunit ribosomal protein L34
K02956: small subunit ribosomal protein S15
K03088: RNA polymerase sigma−70 factor, E...
K03797: carboxyl−terminal processing protea...
K04077: chaperonin GroEL
K04078: chaperonin GroES
K07263: zinc protease
K09702: hypothetical protein
K09748: hypothetical protein
K13993: HSP20 family protein
Proteins
K00134: glyceraldehyde 3−phosphate dehydr...
K00262: glutamate dehydrogenase (NADP+) ...
K01006: pyruvate, orthophosphate dikinase [...
K01610: phosphoenolpyruvate carboxykinase...
K02355: elongation factor EF−G
K02358: elongation factor EF−Tu
K02986: small subunit ribosomal protein S4
K07335: basic membrane protein A and relat...
K10200: N−acetylglucosamine transport syst...
UNGROUPED
Taxonomy
PCo 1 (8.1%)
PCo 2 (6.7%)
Transcripts
PCo 1 (14.2%)
PCo 2 (9.2%)
Proteins
PCo 1 (30.8%)
PCo 2 (12.3%)
Metabolites
PCo 1 (8.8%)
PCo 2 (7.0%)

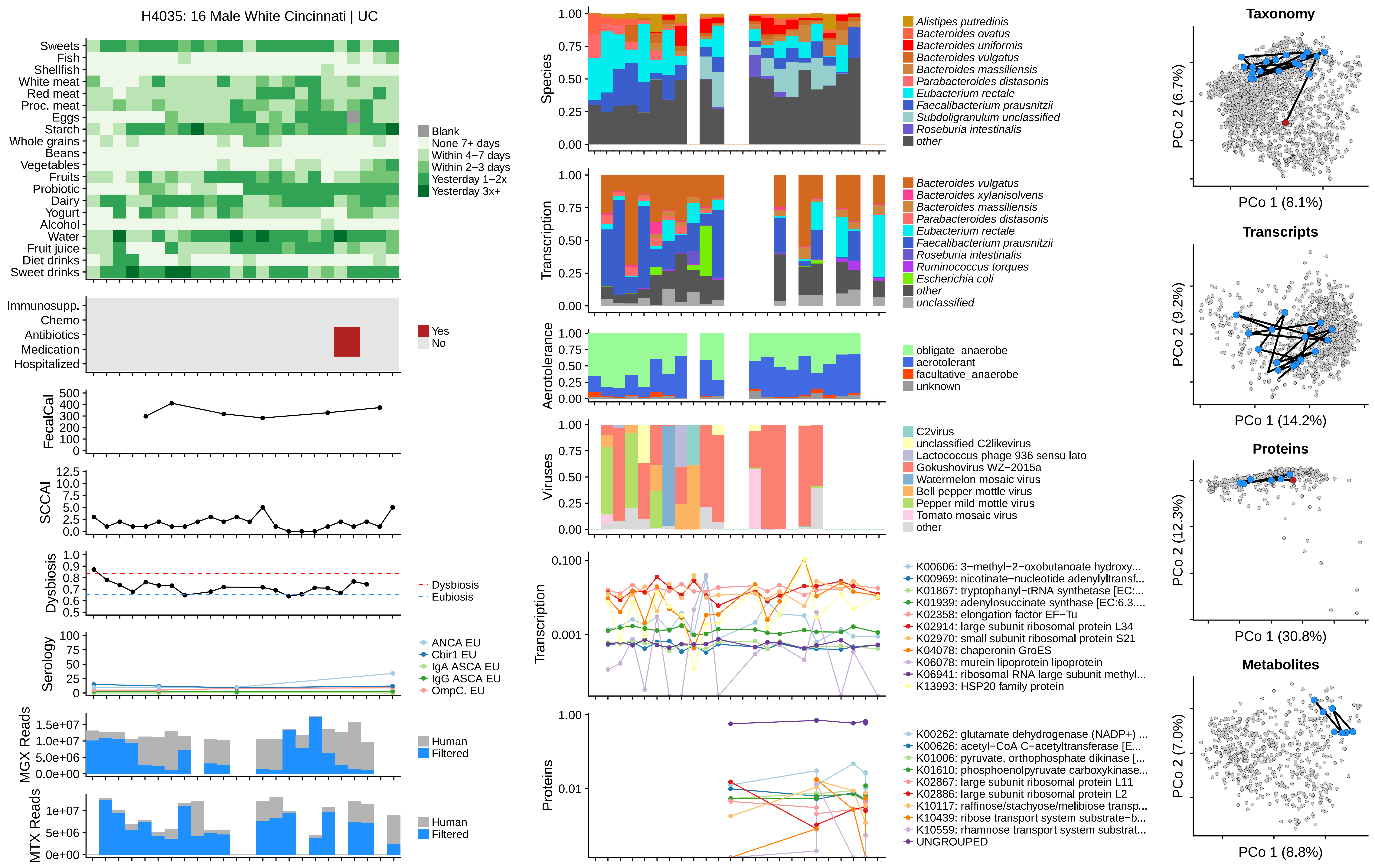
H4035: 16 Male White Cincinnati | UC
Sweets
Fish
Shellfish
White meat
Red meat
Proc. meat
Eggs
Starch
Whole grains
Beans
Vegetables
Fruits
Probiotic
Dairy
Yogurt
Alcohol
Water
Fruit juice
Diet drinks
Sweet drinks
Blank
None 7+ days
Within 4−7 days
Within 2−3 days
Yesterday 1−2x
Yesterday 3x+
Immunosupp.
Chemo
Antibiotics
Medication
Hospitalized
Yes
No
FecalCal
SCCAI
Dysbiosis
Dysbiosis
Eubiosis
Serology
ANCA EU
Cbir1 EU
IgA ASCA EU
IgG ASCA EU
OmpC. EU
MGX Reads
Human
Filtered
MTX Reads
Human
Filtered
Species
Alistipes putredinis
Bacteroides ovatus
Bacteroides uniformis
Bacteroides vulgatus
Bacteroides massiliensis
Parabacteroides distasonis
Eubacterium rectale
Faecalibacterium prausnitzii
Subdoligranulum unclassified
Roseburia intestinalis
other
Transcription
Bacteroides vulgatus
Bacteroides xylanisolvens
Bacteroides massiliensis
Parabacteroides distasonis
Eubacterium rectale
Faecalibacterium prausnitzii
Roseburia intestinalis
Ruminococcus torques
Escherichia coli
other
unclassified
Aerotolerance
obligate_anaerobe
aerotolerant
facultative_anaerobe
unknown
Viruses
C2virus
unclassified C2likevirus
Lactococcus phage 936 sensu lato
Gokushovirus WZ−2015a
Watermelon mosaic virus
Bell pepper mottle virus
Pepper mild mottle virus
Tomato mosaic virus
other
Transcription
K00606: 3−methyl−2−oxobutanoate hydroxy...
K00969: nicotinate−nucleotide adenylyltransf...
K01867: tryptophanyl−tRNA synthetase [EC:...
K01939: adenylosuccinate synthase [EC:6.3....
K02358: elongation factor EF−Tu
K02914: large subunit ribosomal protein L34
K02970: small subunit ribosomal protein S21
K04078: chaperonin GroES
K06078: murein lipoprotein lipoprotein
K06941: ribosomal RNA large subunit methyl...
K13993: HSP20 family protein
Proteins
K00262: glutamate dehydrogenase (NADP+) ...
K00626: acetyl−CoA C−acetyltransferase [E...
K01006: pyruvate, orthophosphate dikinase [...
K01610: phosphoenolpyruvate carboxykinase...
K02867: large subunit ribosomal protein L11
K02886: large subunit ribosomal protein L2
K10117: raffinose/stachyose/melibiose transp...
K10439: ribose transport system substrate−b...
K10559: rhamnose transport system substrat...
UNGROUPED
Taxonomy
PCo 1 (8.1%)
PCo 2 (6.7%)
Transcripts
PCo 1 (14.2%)
PCo 2 (9.2%)
Proteins
PCo 1 (30.8%)
PCo 2 (12.3%)
Metabolites
PCo 1 (8.8%)
PCo 2 (7.0%)

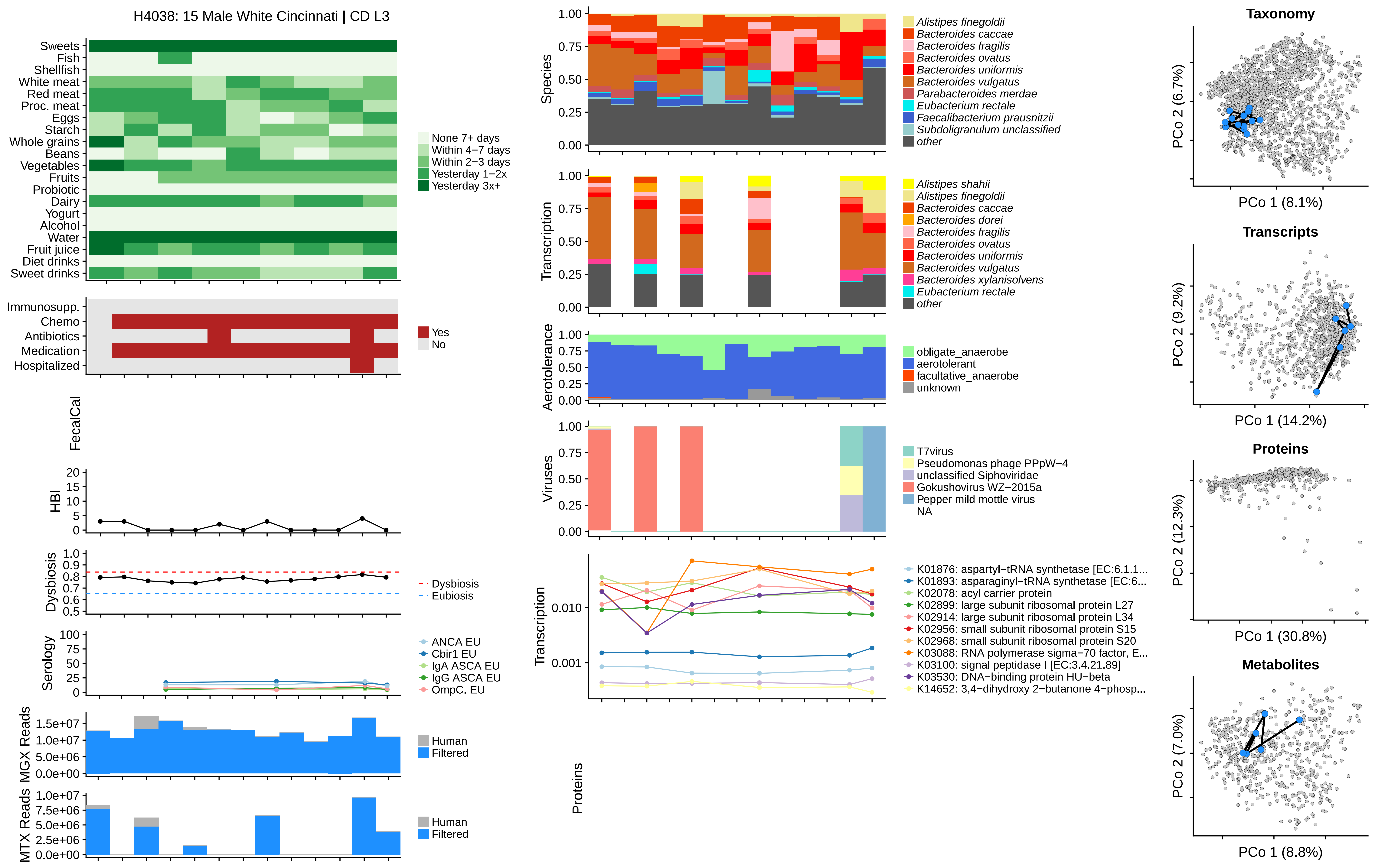
H4038: 15 Male White Cincinnati | CD L3
Sweets
Fish
Shellfish
White meat
Red meat
Proc. meat
Eggs
Starch
Whole grains
Beans
Vegetables
Fruits
Probiotic
Dairy
Yogurt
Alcohol
Water
Fruit juice
Diet drinks
Sweet drinks
None 7+ days
Within 4−7 days
Within 2−3 days
Yesterday 1−2x
Yesterday 3x+
Immunosupp.
Chemo
Antibiotics
Medication
Hospitalized
Yes
No
FecalCal
HBI
Dysbiosis
Dysbiosis
Eubiosis
Serology
ANCA EU
Cbir1 EU
IgA ASCA EU
IgG ASCA EU
OmpC. EU
MGX Reads
Human
Filtered
MTX Reads
Human
Filtered
Species
Alistipes finegoldii
Bacteroides caccae
Bacteroides fragilis
Bacteroides ovatus
Bacteroides uniformis
Bacteroides vulgatus
Parabacteroides merdae
Eubacterium rectale
Faecalibacterium prausnitzii
Subdoligranulum unclassified
other
Transcription
Alistipes shahii
Alistipes finegoldii
Bacteroides caccae
Bacteroides dorei
Bacteroides fragilis
Bacteroides ovatus
Bacteroides uniformis
Bacteroides vulgatus
Bacteroides xylanisolvens
Eubacterium rectale
other
Aerotolerance
obligate_anaerobe
aerotolerant
facultative_anaerobe
unknown
Viruses
T7virus
Pseudomonas phage PPpW−4
unclassified Siphoviridae
Gokushovirus WZ−2015a
Pepper mild mottle virus
NA
Transcription
K01876: aspartyl−tRNA synthetase [EC:6.1.1...
K01893: asparaginyl−tRNA synthetase [EC:6...
K02078: acyl carrier protein
K02899: large subunit ribosomal protein L27
K02914: large subunit ribosomal protein L34
K02956: small subunit ribosomal protein S15
K02968: small subunit ribosomal protein S20
K03088: RNA polymerase sigma−70 factor, E...
K03100: signal peptidase I [EC:3.4.21.89]
K03530: DNA−binding protein HU−beta
K14652: 3,4−dihydroxy 2−butanone 4−phosp...
Proteins
Taxonomy
PCo 1 (8.1%)
PCo 2 (6.7%)
Transcripts
PCo 1 (14.2%)
PCo 2 (9.2%)
Proteins
PCo 1 (30.8%)
PCo 2 (12.3%)
Metabolites
PCo 1 (8.8%)
PCo 2 (7.0%)

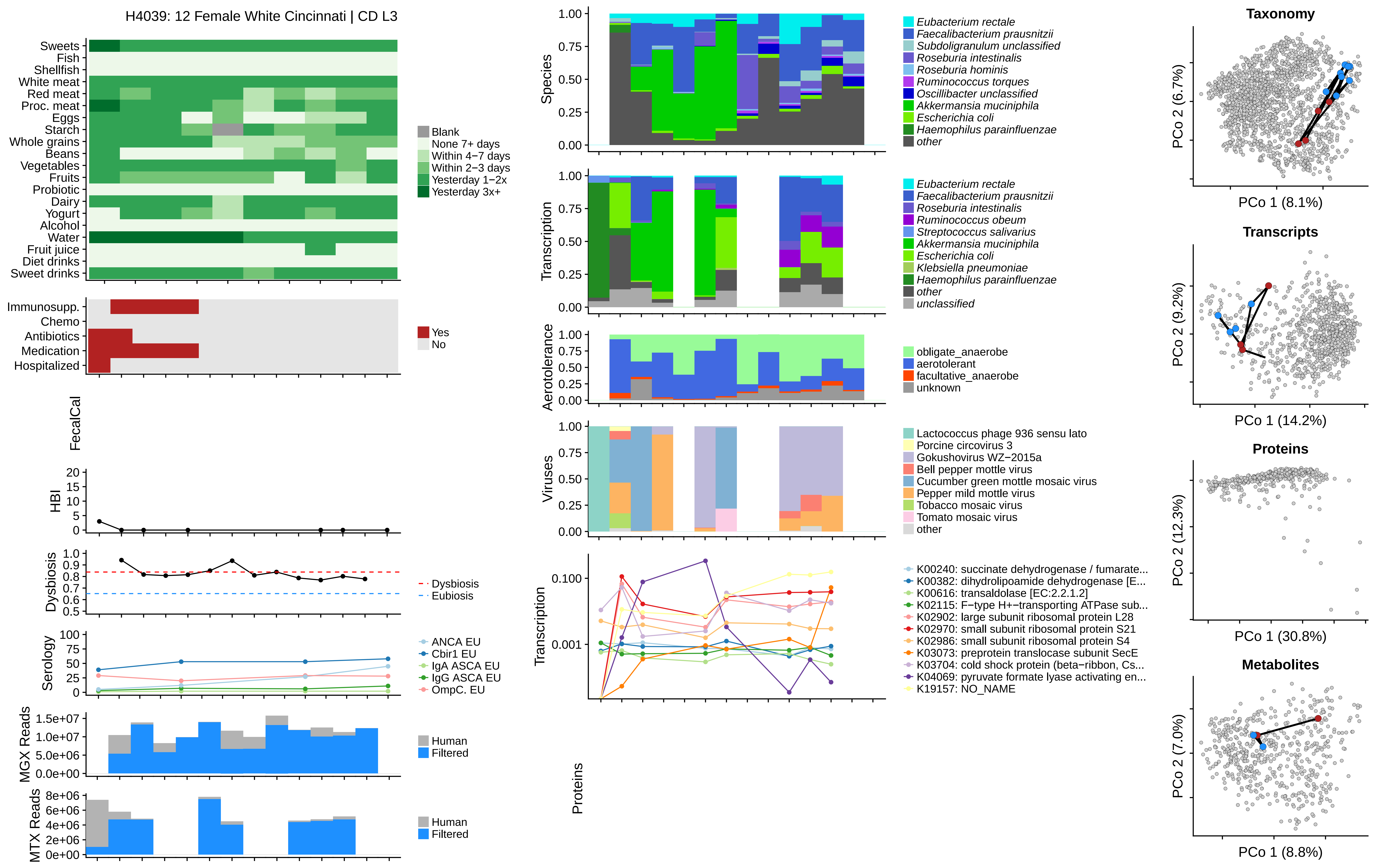
H4039: 12 Female White Cincinnati | CD L3
Sweets
Fish
Shellfish
White meat
Red meat
Proc. meat
Eggs
Starch
Whole grains
Beans
Vegetables
Fruits
Probiotic
Dairy
Yogurt
Alcohol
Water
Fruit juice
Diet drinks
Sweet drinks
Blank
None 7+ days
Within 4−7 days
Within 2−3 days
Yesterday 1−2x
Yesterday 3x+
Immunosupp.
Chemo
Antibiotics
Medication
Hospitalized
Yes
No
FecalCal
HBI
Dysbiosis
Dysbiosis
Eubiosis
Serology
ANCA EU
Cbir1 EU
IgA ASCA EU
IgG ASCA EU
OmpC. EU
MGX Reads
Human
Filtered
MTX Reads
Human
Filtered
Species
Eubacterium rectale
Faecalibacterium prausnitzii
Subdoligranulum unclassified
Roseburia intestinalis
Roseburia hominis
Ruminococcus torques
Oscillibacter unclassified
Akkermansia muciniphila
Escherichia coli
Haemophilus parainfluenzae
other
Transcription
Eubacterium rectale
Faecalibacterium prausnitzii
Roseburia intestinalis
Ruminococcus obeum
Streptococcus salivarius
Akkermansia muciniphila
Escherichia coli
Klebsiella pneumoniae
Haemophilus parainfluenzae
other
unclassified
Aerotolerance
obligate_anaerobe
aerotolerant
facultative_anaerobe
unknown
Viruses
Lactococcus phage 936 sensu lato
Porcine circovirus 3
Gokushovirus WZ−2015a
Bell pepper mottle virus
Cucumber green mottle mosaic virus
Pepper mild mottle virus
Tobacco mosaic virus
Tomato mosaic virus
other
Transcription
K00240: succinate dehydrogenase / fumarate...
K00382: dihydrolipoamide dehydrogenase [E...
K00616: transaldolase [EC:2.2.1.2]
K02115: F−type H+−transporting ATPase sub...
K02902: large subunit ribosomal protein L28
K02970: small subunit ribosomal protein S21
K02986: small subunit ribosomal protein S4
K03073: preprotein translocase subunit SecE
K03704: cold shock protein (beta−ribbon, Cs...
K04069: pyruvate formate lyase activating en...
K19157: NO_NAME
Proteins
Taxonomy
PCo 1 (8.1%)
PCo 2 (6.7%)
Transcripts
PCo 1 (14.2%)
PCo 2 (9.2%)
Proteins
PCo 1 (30.8%)
PCo 2 (12.3%)
Metabolites
PCo 1 (8.8%)
PCo 2 (7.0%)

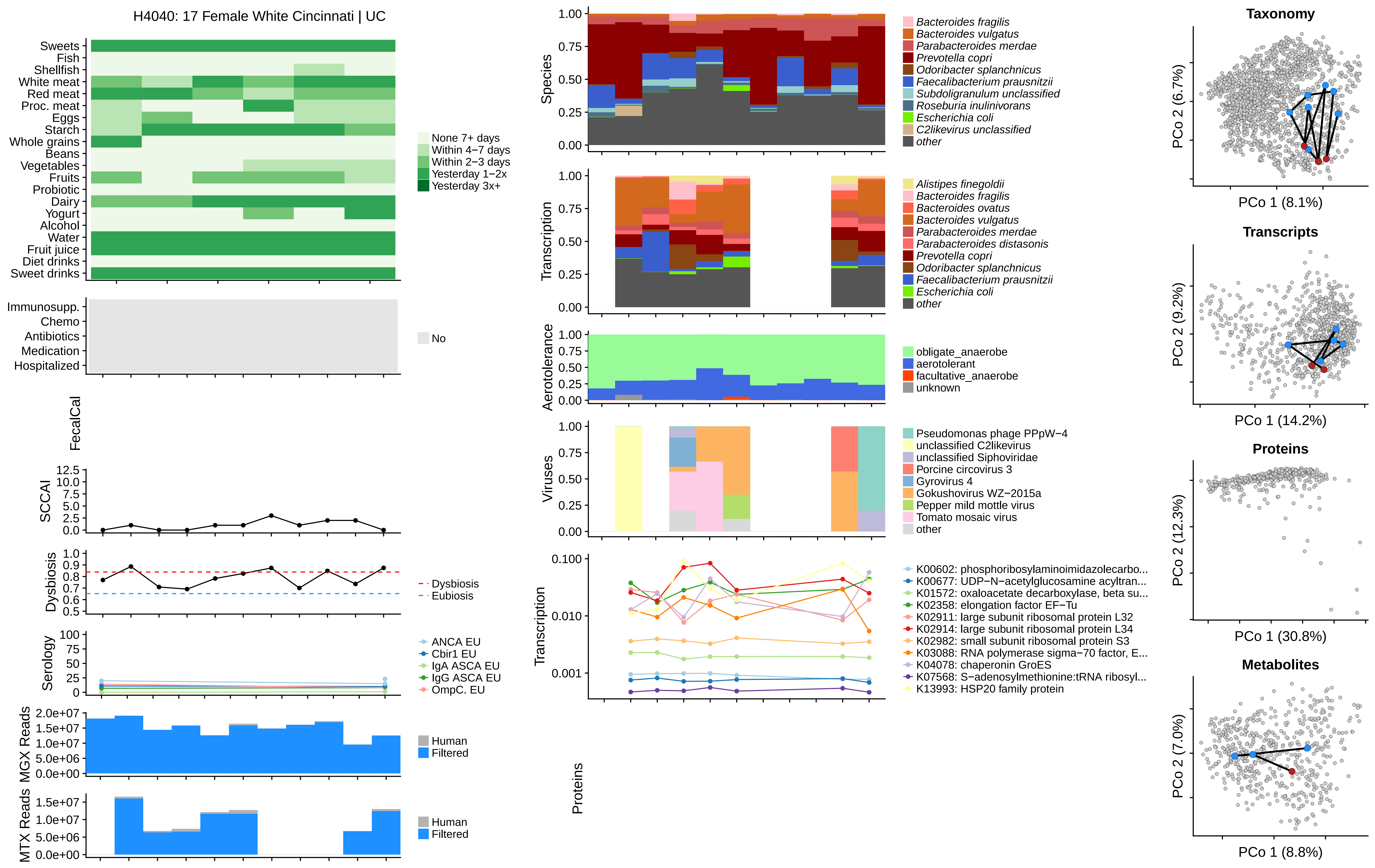

H4040: 17 Female White Cincinnati | UC
Sweets
Fish
Shellfish
White meat
Red meat
Proc. meat
Eggs
Starch
Whole grains
Beans
Vegetables
Fruits
Probiotic
Dairy
Yogurt
Alcohol
Water
Fruit juice
Diet drinks
Sweet drinks
None 7+ days
Within 4−7 days
Within 2−3 days
Yesterday 1−2x
Yesterday 3x+
Immunosupp.
Chemo
Antibiotics
Medication
Hospitalized
No
FecalCal
SCCAI
Dysbiosis
Dysbiosis
Eubiosis
Serology
ANCA EU
Cbir1 EU
IgA ASCA EU
IgG ASCA EU
OmpC. EU
MGX Reads
Human
Filtered
MTX Reads
Human
Filtered
Species
Bacteroides fragilis
Bacteroides vulgatus
Parabacteroides merdae
Prevotella copri
Odoribacter splanchnicus
Faecalibacterium prausnitzii
Subdoligranulum unclassified
Roseburia inulinivorans
Escherichia coli
C2likevirus unclassified
other
Transcription
Alistipes finegoldii
Bacteroides fragilis
Bacteroides ovatus
Bacteroides vulgatus
Parabacteroides merdae
Parabacteroides distasonis
Prevotella copri
Odoribacter splanchnicus
Faecalibacterium prausnitzii
Escherichia coli
other
Aerotolerance
obligate_anaerobe
aerotolerant
facultative_anaerobe
unknown
Viruses
Pseudomonas phage PPpW−4
unclassified C2likevirus
unclassified Siphoviridae
Porcine circovirus 3
Gyrovirus 4
Gokushovirus WZ−2015a
Pepper mild mottle virus
Tomato mosaic virus
other
Transcription
K00602: phosphoribosylaminoimidazolecarbo...
K00677: UDP−N−acetylglucosamine acyltran...
K01572: oxaloacetate decarboxylase, beta su...
K02358: elongation factor EF−Tu
K02911: large subunit ribosomal protein L32
K02914: large subunit ribosomal protein L34
K02982: small subunit ribosomal protein S3
K03088: RNA polymerase sigma−70 factor, E...
K04078: chaperonin GroES
K07568: S−adenosylmethionine:tRNA ribosyl...
K13993: HSP20 family protein
Proteins
Taxonomy
PCo 1 (8.1%)
PCo 2 (6.7%)
Transcripts
PCo 1 (14.2%)
PCo 2 (9.2%)
Proteins
PCo 1 (30.8%)
PCo 2 (12.3%)
Metabolites
PCo 1 (8.8%)
PCo 2 (7.0%)

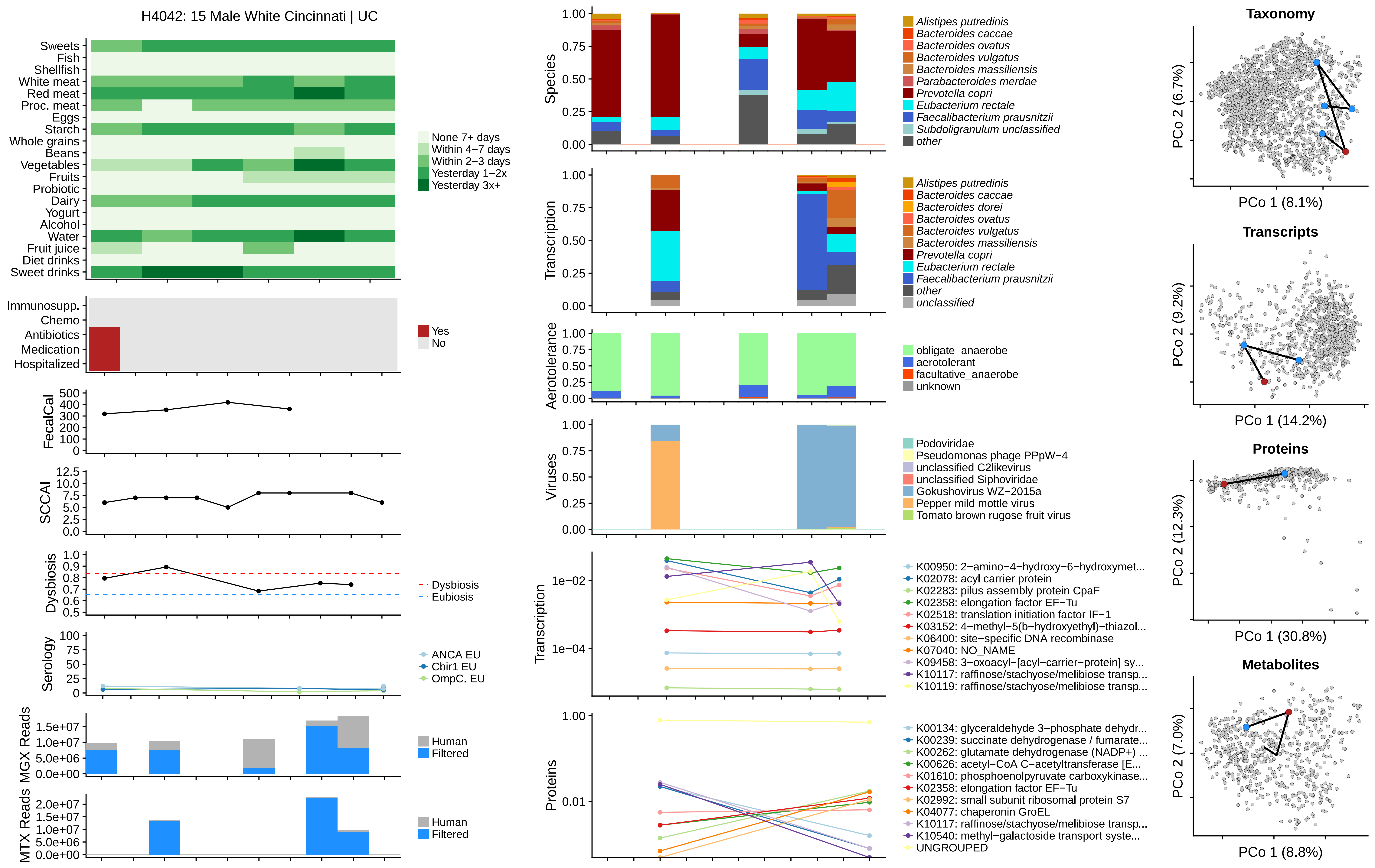
H4042: 15 Male White Cincinnati | UC
Sweets
Fish
Shellfish
White meat
Red meat
Proc. meat
Eggs
Starch
Whole grains
Beans
Vegetables
Fruits
Probiotic
Dairy
Yogurt
Alcohol
Water
Fruit juice
Diet drinks
Sweet drinks
None 7+ days
Within 4−7 days
Within 2−3 days
Yesterday 1−2x
Yesterday 3x+
Immunosupp.
Chemo
Antibiotics
Medication
Hospitalized
Yes
No
FecalCal
500
400
300
200
100
0
SCCAI
12.5
10.0
7.5
5.0
2.5
0.0
Dysbiosis
1.0
0.9
0.8
0.7
0.6
0.5
Dysbiosis
Eubiosis
Serology
100
75
50
25
0
ANCA EU
Cbir1 EU
OmpC. EU
MGX Reads
1.5e+07
1.0e+07
5.0e+06
0.0e+00
Human
Filtered
MTX Reads
2.0e+07
1.5e+07
1.0e+07
5.0e+06
0.0e+00
Human
Filtered
Species
1.00
0.75
0.50
0.25
0.00
Alistipes putredinis
Bacteroides caccae
Bacteroides ovatus
Bacteroides vulgatus
Bacteroides massiliensis
Parabacteroides merdae
Prevotella copri
Eubacterium rectale
Faecalibacterium prausnitzii
Subdoligranulum unclassified
other
Transcription
Alistipes putredinis
Bacteroides caccae
Bacteroides dorei
Bacteroides ovatus
Bacteroides vulgatus
Bacteroides massiliensis
Prevotella copri
Eubacterium rectale
Faecalibacterium prausnitzii
other
unclassified
Aerotolerance
obligate_anaerobe
aerotolerant
facultative_anaerobe
unknown
Viruses
Podoviridae
Pseudomonas phage PPpW−4
unclassified C2likevirus
unclassified Siphoviridae
Gokushovirus WZ−2015a
Pepper mild mottle virus
Tomato brown rugose fruit virus
Transcription
1e−02
1e−04
K00950: 2−amino−4−hydroxy−6−hydroxymet...
K02078: acyl carrier protein
K02283: pilus assembly protein CpaF
K02358: elongation factor EF−Tu
K02518: translation initiation factor IF−1
K03152: 4−methyl−5(b−hydroxyethyl)−thiazol...
K06400: site−specific DNA recombinase
K07040: NO_NAME
K09458: 3−oxoacyl−[acyl−carrier−protein] sy...
K10117: raffinose/stachyose/melibiose transp...
K10119: raffinose/stachyose/melibiose transp...
Proteins
1.00
0.01
K00134: glyceraldehyde 3−phosphate dehydr...
K00239: succinate dehydrogenase / fumarate...
K00262: glutamate dehydrogenase (NADP+) ...
K00626: acetyl−CoA C−acetyltransferase [E...
K01610: phosphoenolpyruvate carboxykinase...
K02358: elongation factor EF−Tu
K02992: small subunit ribosomal protein S7
K04077: chaperonin GroEL
K10117: raffinose/stachyose/melibiose transp...
K10540: methyl−galactoside transport syste...
UNGROUPED
Taxonomy
PCo 2 (6.7%)
PCo 1 (8.1%)
Transcripts
PCo 2 (9.2%)
PCo 1 (14.2%)
Proteins
PCo 2 (12.3%)
PCo 1 (30.8%)
Metabolites
PCo 2 (7.0%)
PCo 1 (8.8%)

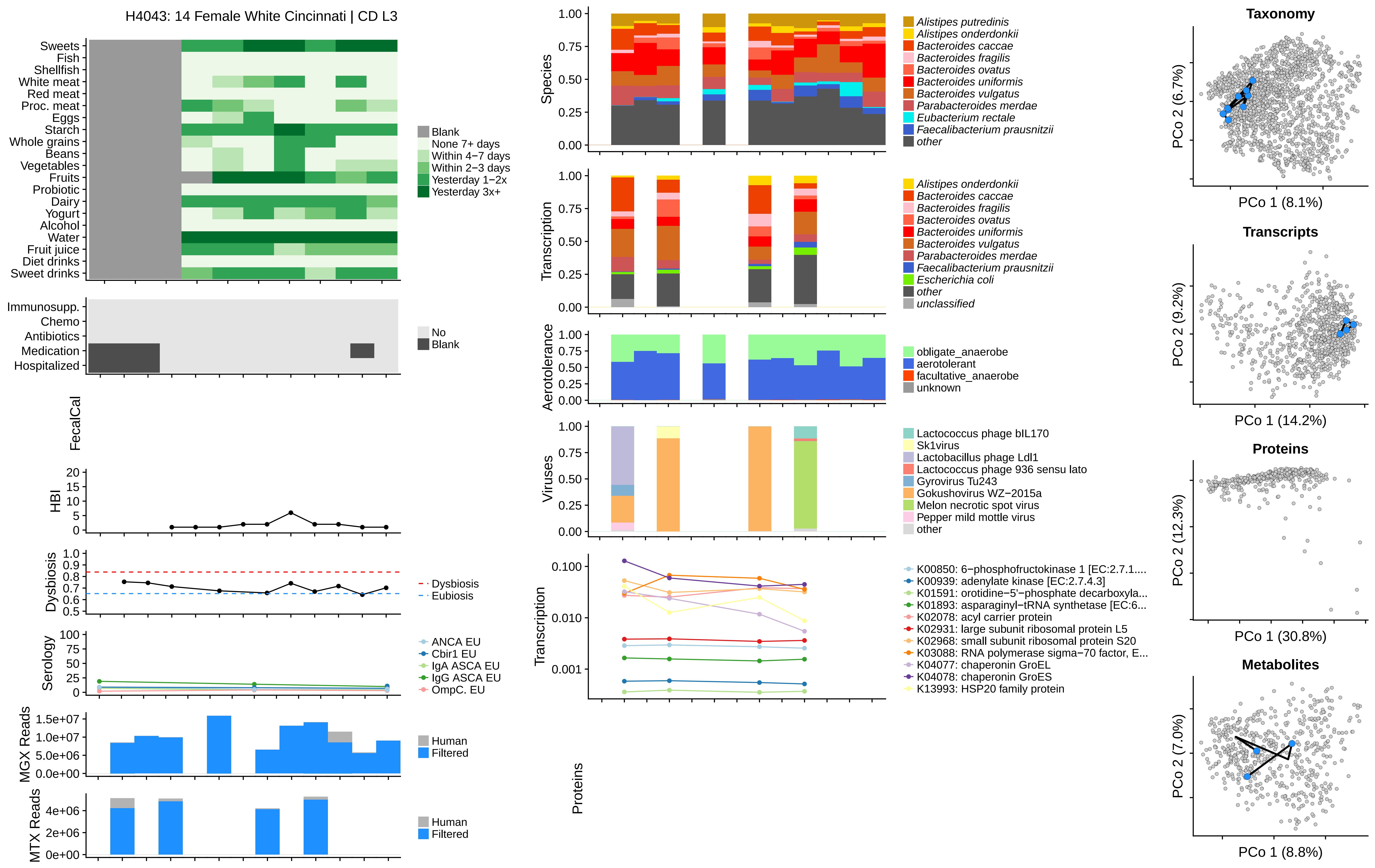

H4043: 14 Female White Cincinnati | CD L3
Sweets
Fish
Shellfish
White meat
Red meat
Proc. meat
Eggs
Starch
Whole grains
Beans
Vegetables
Fruits
Probiotic
Dairy
Yogurt
Alcohol
Water
Fruit juice
Diet drinks
Sweet drinks
Blank
None 7+ days
Within 4−7 days
Within 2−3 days
Yesterday 1−2x
Yesterday 3x+
Immunosupp.
Chemo
Antibiotics
Medication
Hospitalized
No
Blank
FecalCal
HBI
Dysbiosis
Dysbiosis
Eubiosis
Serology
ANCA EU
Cbir1 EU
IgA ASCA EU
IgG ASCA EU
OmpC. EU
MGX Reads
Human
Filtered
MTX Reads
Human
Filtered
Species
Alistipes putredinis
Alistipes onderdonkii
Bacteroides caccae
Bacteroides fragilis
Bacteroides ovatus
Bacteroides uniformis
Bacteroides vulgatus
Parabacteroides merdae
Eubacterium rectale
Faecalibacterium prausnitzii
other
Transcription
Alistipes onderdonkii
Bacteroides caccae
Bacteroides fragilis
Bacteroides ovatus
Bacteroides uniformis
Bacteroides vulgatus
Parabacteroides merdae
Faecalibacterium prausnitzii
Escherichia coli
other
unclassified
Aerotolerance
obligate_anaerobe
aerotolerant
facultative_anaerobe
unknown
Viruses
Lactococcus phage bIL170
Sk1virus
Lactobacillus phage Ldl1
Lactococcus phage 936 sensu lato
Gyrovirus Tu243
Gokushovirus WZ−2015a
Melon necrotic spot virus
Pepper mild mottle virus
other
Transcription
K00850: 6−phosphofructokinase 1 [EC:2.7.1....
K00939: adenylate kinase [EC:2.7.4.3]
K01591: orotidine−5'−phosphate decarboxyla...
K01893: asparaginyl−tRNA synthetase [EC:6...
K02078: acyl carrier protein
K02931: large subunit ribosomal protein L5
K02968: small subunit ribosomal protein S20
K03088: RNA polymerase sigma−70 factor, E...
K04077: chaperonin GroEL
K04078: chaperonin GroES
K13993: HSP20 family protein
Proteins
Taxonomy
PCo 1 (8.1%)
PCo 2 (6.7%)
Transcripts
PCo 1 (14.2%)
PCo 2 (9.2%)
Proteins
PCo 1 (30.8%)
PCo 2 (12.3%)
Metabolites
PCo 1 (8.8%)
PCo 2 (7.0%)

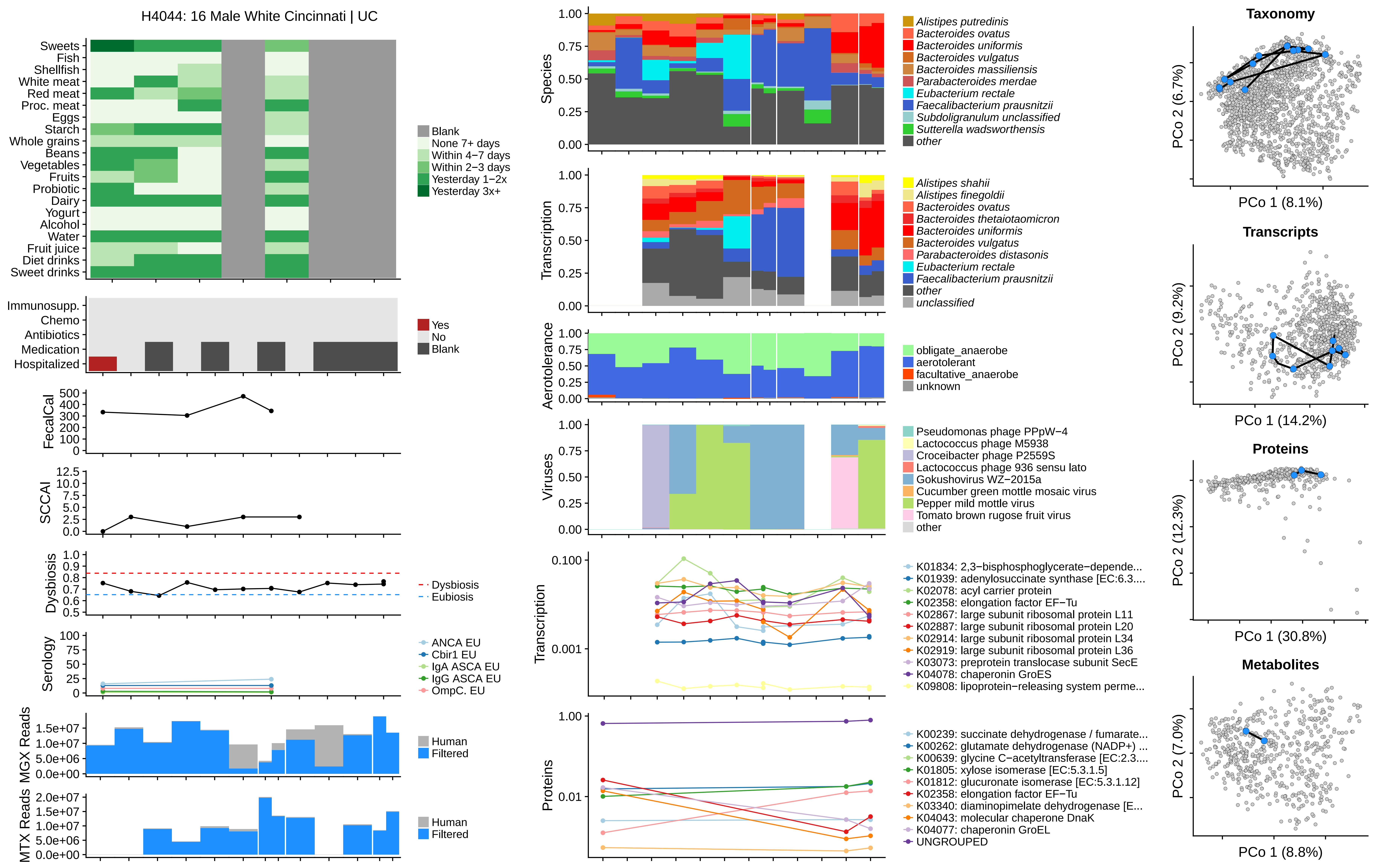
H4044: 16 Male White Cincinnati | UC
Sweets
Fish
Shellfish
White meat
Red meat
Proc. meat
Eggs
Starch
Whole grains
Beans
Vegetables
Fruits
Probiotic
Dairy
Yogurt
Alcohol
Water
Fruit juice
Diet drinks
Sweet drinks
Blank
None 7+ days
Within 4−7 days
Within 2−3 days
Yesterday 1−2x
Yesterday 3x+
Immunosupp.
Chemo
Antibiotics
Medication
Hospitalized
Yes
No
Blank
FecalCal
SCCAI
Dysbiosis
Dysbiosis
Eubiosis
Serology
ANCA EU
Cbir1 EU
IgA ASCA EU
IgG ASCA EU
OmpC. EU
MGX Reads
Human
Filtered
MTX Reads
Human
Filtered
Species
Alistipes putredinis
Bacteroides ovatus
Bacteroides uniformis
Bacteroides vulgatus
Bacteroides massiliensis
Parabacteroides merdae
Eubacterium rectale
Faecalibacterium prausnitzii
Subdoligranulum unclassified
Sutterella wadsworthensis
other
Transcription
Alistipes shahii
Alistipes finegoldii
Bacteroides ovatus
Bacteroides thetaiotaomicron
Bacteroides uniformis
Bacteroides vulgatus
Parabacteroides distasonis
Eubacterium rectale
Faecalibacterium prausnitzii
other
unclassified
Aerotolerance
obligate_anaerobe
aerotolerant
facultative_anaerobe
unknown
Viruses
Pseudomonas phage PPpW−4
Lactococcus phage M5938
Croceibacter phage P2559S
Lactococcus phage 936 sensu lato
Gokushovirus WZ−2015a
Cucumber green mottle mosaic virus
Pepper mild mottle virus
Tomato brown rugose fruit virus
other
Transcription
K01834: 2,3−bisphosphoglycerate−depende...
K01939: adenylosuccinate synthase [EC:6.3....
K02078: acyl carrier protein
K02358: elongation factor EF−Tu
K02867: large subunit ribosomal protein L11
K02887: large subunit ribosomal protein L20
K02914: large subunit ribosomal protein L34
K02919: large subunit ribosomal protein L36
K03073: preprotein translocase subunit SecE
K04078: chaperonin GroES
K09808: lipoprotein−releasing system perme...
Proteins
K00239: succinate dehydrogenase / fumarate...
K00262: glutamate dehydrogenase (NADP+) ...
K00639: glycine C−acetyltransferase [EC:2.3....
K01805: xylose isomerase [EC:5.3.1.5]
K01812: glucuronate isomerase [EC:5.3.1.12]
K02358: elongation factor EF−Tu
K03340: diaminopimelate dehydrogenase [E...
K04043: molecular chaperone DnaK
K04077: chaperonin GroEL
UNGROUPED
Taxonomy
PCo 1 (8.1%)
PCo 2 (6.7%)
Transcripts
PCo 1 (14.2%)
PCo 2 (9.2%)
Proteins
PCo 1 (30.8%)
PCo 2 (12.3%)
Metabolites
PCo 1 (8.8%)
PCo 2 (7.0%)

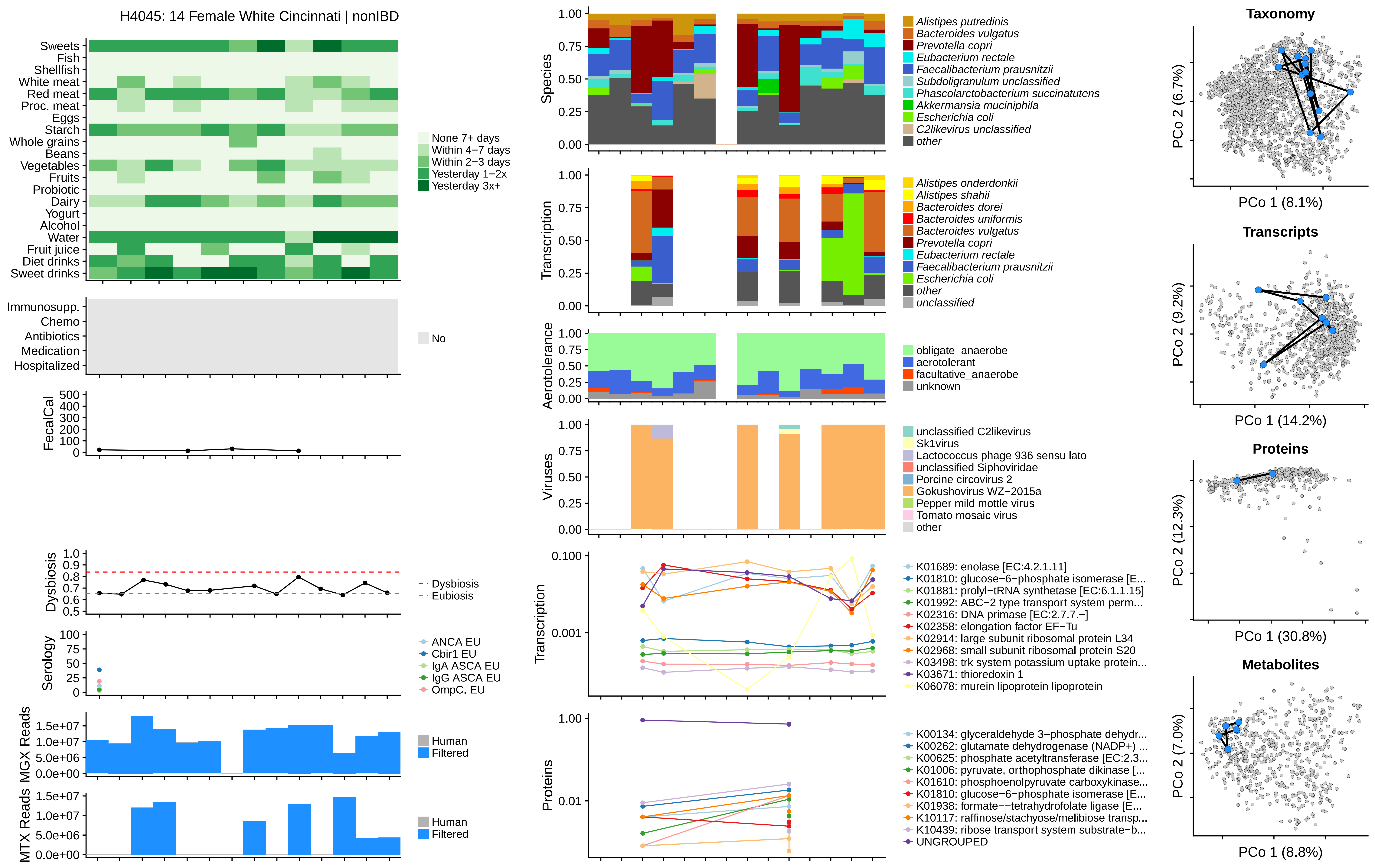
H4045: 14 Female White Cincinnati | nonIBD
Sweets
Fish
Shellfish
White meat
Red meat
Proc. meat
Eggs
Starch
Whole grains
Beans
Vegetables
Fruits
Probiotic
Dairy
Yogurt
Alcohol
Water
Fruit juice
Diet drinks
Sweet drinks
None 7+ days
Within 4−7 days
Within 2−3 days
Yesterday 1−2x
Yesterday 3x+
Immunosupp.
Chemo
Antibiotics
Medication
Hospitalized
No
FecalCal
Dysbiosis
Dysbiosis
Eubiosis
Serology
ANCA EU
Cbir1 EU
IgA ASCA EU
IgG ASCA EU
OmpC. EU
MGX Reads
Human
Filtered
MTX Reads
Human
Filtered
Species
Alistipes putredinis
Bacteroides vulgatus
Prevotella copri
Eubacterium rectale
Faecalibacterium prausnitzii
Subdoligranulum unclassified
Phascolarctobacterium succinatutens
Akkermansia muciniphila
Escherichia coli
C2likevirus unclassified
other
Transcription
Alistipes onderdonkii
Alistipes shahii
Bacteroides dorei
Bacteroides uniformis
Bacteroides vulgatus
Prevotella copri
Eubacterium rectale
Faecalibacterium prausnitzii
Escherichia coli
other
unclassified
Aerotolerance
obligate_anaerobe
aerotolerant
facultative_anaerobe
unknown
Viruses
unclassified C2likevirus
Sk1virus
Lactococcus phage 936 sensu lato
unclassified Siphoviridae
Porcine circovirus 2
Gokushovirus WZ−2015a
Pepper mild mottle virus
Tomato mosaic virus
other
Transcription
K01689: enolase [EC:4.2.1.11]
K01810: glucose−6−phosphate isomerase [E...
K01881: prolyl−tRNA synthetase [EC:6.1.1.15]
K01992: ABC−2 type transport system perm...
K02316: DNA primase [EC:2.7.7.−]
K02358: elongation factor EF−Tu
K02914: large subunit ribosomal protein L34
K02968: small subunit ribosomal protein S20
K03498: trk system potassium uptake protein...
K03671: thioredoxin 1
K06078: murein lipoprotein lipoprotein
Proteins
K00134: glyceraldehyde 3−phosphate dehydr...
K00262: glutamate dehydrogenase (NADP+) ...
K00625: phosphate acetyltransferase [EC:2.3...
K01006: pyruvate, orthophosphate dikinase [...
K01610: phosphoenolpyruvate carboxykinase...
K01810: glucose−6−phosphate isomerase [E...
K01938: formate−−tetrahydrofolate ligase [E...
K10117: raffinose/stachyose/melibiose transp...
K10439: ribose transport system substrate−b...
UNGROUPED
Taxonomy
PCo 1 (8.1%)
PCo 2 (6.7%)
Transcripts
PCo 1 (14.2%)
PCo 2 (9.2%)
Proteins
PCo 1 (30.8%)
PCo 2 (12.3%)
Metabolites
PCo 1 (8.8%)
PCo 2 (7.0%)

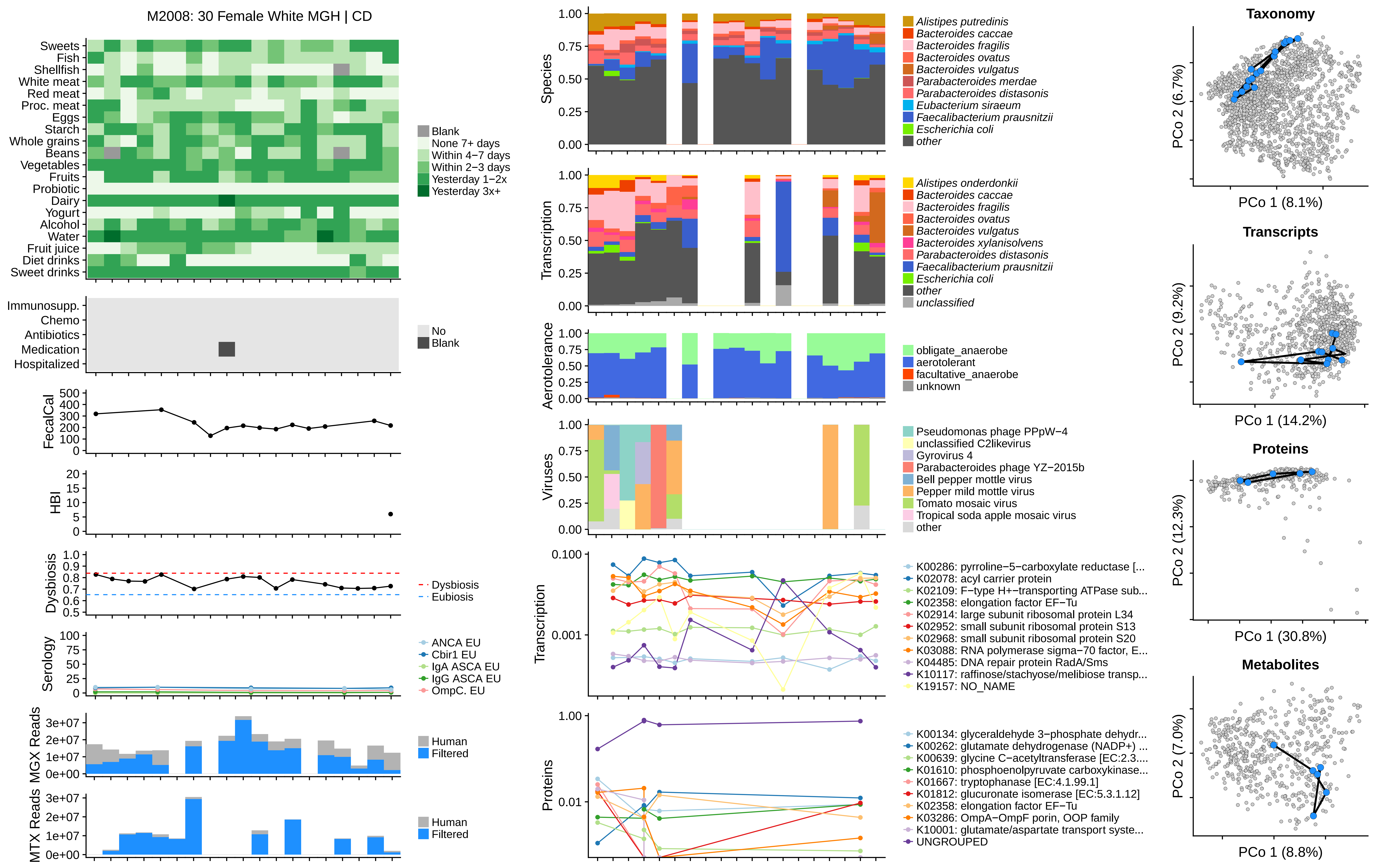

M2008: 30 Female White MGH | CD
Sweets
Fish
Shellfish
White meat
Red meat
Proc. meat
Eggs
Starch
Whole grains
Beans
Vegetables
Fruits
Probiotic
Dairy
Yogurt
Alcohol
Water
Fruit juice
Diet drinks
Sweet drinks
Blank
None 7+ days
Within 4−7 days
Within 2−3 days
Yesterday 1−2x
Yesterday 3x+
Immunosupp.
Chemo
Antibiotics
Medication
Hospitalized
No
Blank
FecalCal
HBI
Dysbiosis
Dysbiosis
Eubiosis
Serology
ANCA EU
Cbir1 EU
IgA ASCA EU
IgG ASCA EU
OmpC. EU
MGX Reads
Human
Filtered
MTX Reads
Human
Filtered
Species
Alistipes putredinis
Bacteroides caccae
Bacteroides fragilis
Bacteroides ovatus
Bacteroides vulgatus
Parabacteroides merdae
Parabacteroides distasonis
Eubacterium siraeum
Faecalibacterium prausnitzii
Escherichia coli
other
Transcription
Alistipes onderdonkii
Bacteroides caccae
Bacteroides fragilis
Bacteroides ovatus
Bacteroides vulgatus
Bacteroides xylanisolvens
Parabacteroides distasonis
Faecalibacterium prausnitzii
Escherichia coli
other
unclassified
Aerotolerance
obligate_anaerobe
aerotolerant
facultative_anaerobe
unknown
Viruses
Pseudomonas phage PPpW−4
unclassified C2likevirus
Gyrovirus 4
Parabacteroides phage YZ−2015b
Bell pepper mottle virus
Pepper mild mottle virus
Tomato mosaic virus
Tropical soda apple mosaic virus
other
Transcription
K00286: pyrroline−5−carboxylate reductase [...
K02078: acyl carrier protein
K02109: F−type H+−transporting ATPase sub...
K02358: elongation factor EF−Tu
K02914: large subunit ribosomal protein L34
K02952: small subunit ribosomal protein S13
K02968: small subunit ribosomal protein S20
K03088: RNA polymerase sigma−70 factor, E...
K04485: DNA repair protein RadA/Sms
K10117: raffinose/stachyose/melibiose transp...
K19157: NO_NAME
Proteins
K00134: glyceraldehyde 3−phosphate dehydr...
K00262: glutamate dehydrogenase (NADP+) ...
K00639: glycine C−acetyltransferase [EC:2.3....
K01610: phosphoenolpyruvate carboxykinase...
K01667: tryptophanase [EC:4.1.99.1]
K01812: glucuronate isomerase [EC:5.3.1.12]
K02358: elongation factor EF−Tu
K03286: OmpA−OmpF porin, OOP family
K10001: glutamate/aspartate transport syste...
UNGROUPED
Taxonomy
PCo 2 (6.7%)
PCo 1 (8.1%)
Transcripts
PCo 2 (9.2%)
PCo 1 (14.2%)
Proteins
PCo 2 (12.3%)
PCo 1 (30.8%)
Metabolites
PCo 2 (7.0%)
PCo 1 (8.8%)

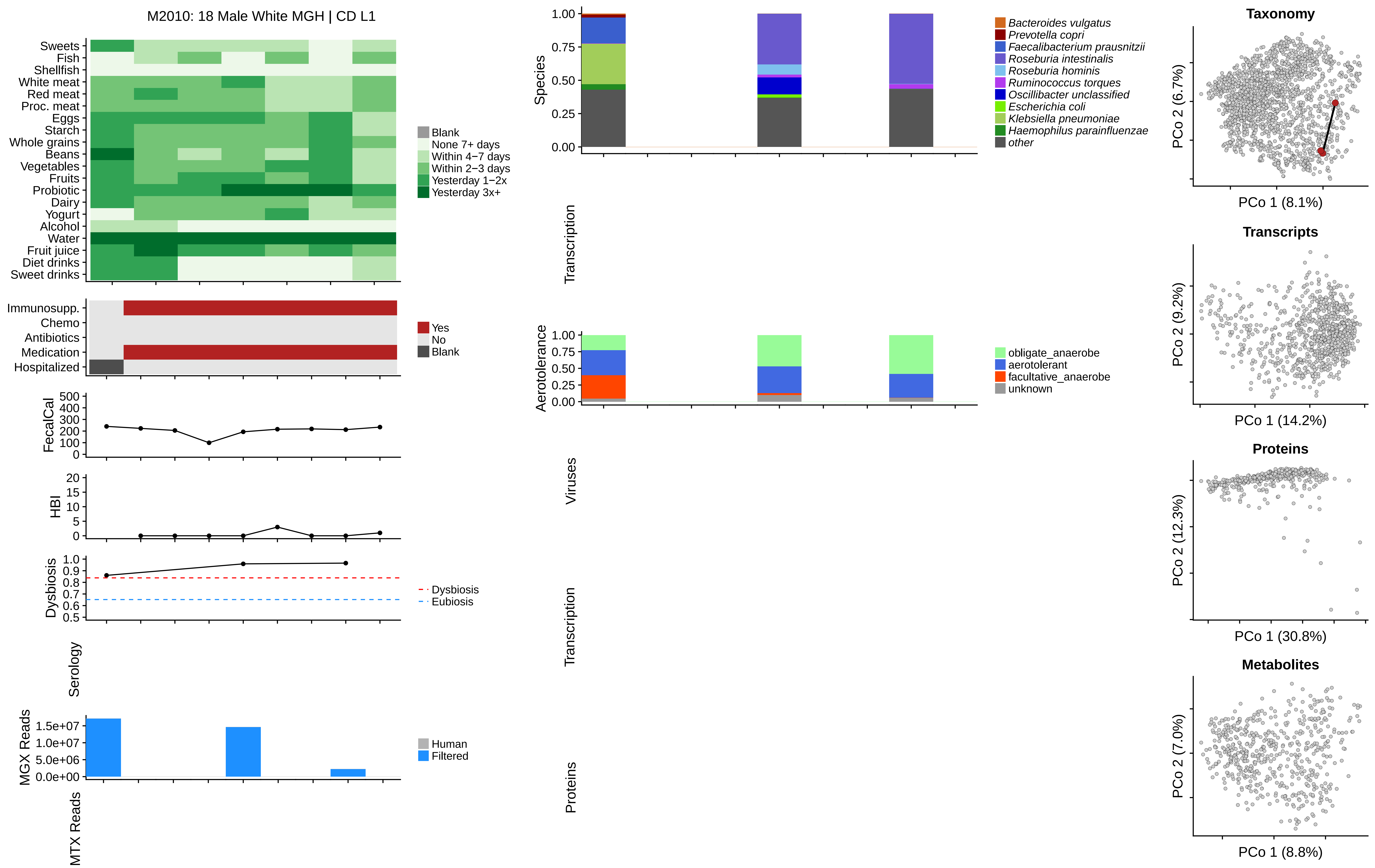

M2010: 18 Male White MGH | CD L1
Sweets
Fish
Shellfish
White meat
Red meat
Proc. meat
Eggs
Starch
Whole grains
Beans
Vegetables
Fruits
Probiotic
Dairy
Yogurt
Alcohol
Water
Fruit juice
Diet drinks
Sweet drinks
Blank
None 7+ days
Within 4−7 days
Within 2−3 days
Yesterday 1−2x
Yesterday 3x+
Immunosupp.
Chemo
Antibiotics
Medication
Hospitalized
Yes
No
Blank
FecalCal
HBI
Dysbiosis
Dysbiosis
Eubiosis
Serology
MGX Reads
Human
Filtered
MTX Reads
Species
Bacteroides vulgatus
Prevotella copri
Faecalibacterium prausnitzii
Roseburia intestinalis
Roseburia hominis
Ruminococcus torques
Oscillibacter unclassified
Escherichia coli
Klebsiella pneumoniae
Haemophilus parainfluenzae
other
Transcription
Aerotolerance
obligate_anaerobe
aerotolerant
facultative_anaerobe
unknown
Viruses
Transcription
Proteins
Taxonomy
PCo 1 (8.1%)
PCo 2 (6.7%)
Transcripts
PCo 1 (14.2%)
PCo 2 (9.2%)
Proteins
PCo 1 (30.8%)
PCo 2 (12.3%)
Metabolites
PCo 1 (8.8%)
PCo 2 (7.0%)

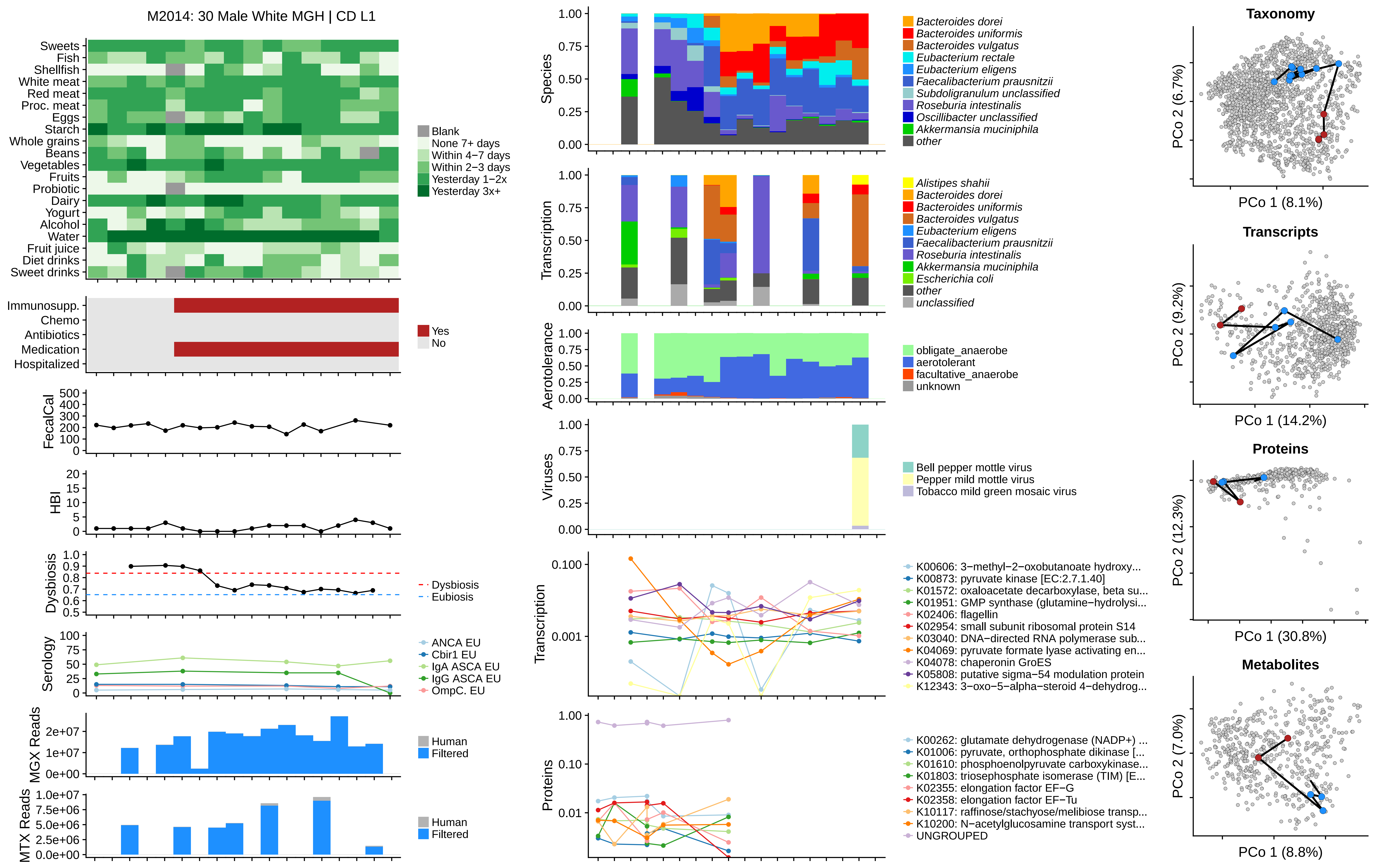

M2014: 30 Male White MGH | CD L1
Sweets
Fish
Shellfish
White meat
Red meat
Proc. meat
Eggs
Starch
Whole grains
Beans
Vegetables
Fruits
Probiotic
Dairy
Yogurt
Alcohol
Water
Fruit juice
Diet drinks
Sweet drinks
Blank
None 7+ days
Within 4−7 days
Within 2−3 days
Yesterday 1−2x
Yesterday 3x+
Immunosupp.
Chemo
Antibiotics
Medication
Hospitalized
Yes
No
FecalCal
HBI
Dysbiosis
Dysbiosis
Eubiosis
Serology
ANCA EU
Cbir1 EU
IgA ASCA EU
IgG ASCA EU
OmpC. EU
MGX Reads
Human
Filtered
MTX Reads
Human
Filtered
Species
Bacteroides dorei
Bacteroides uniformis
Bacteroides vulgatus
Eubacterium rectale
Eubacterium eligens
Faecalibacterium prausnitzii
Subdoligranulum unclassified
Roseburia intestinalis
Oscillibacter unclassified
Akkermansia muciniphila
other
Transcription
Alistipes shahii
Bacteroides dorei
Bacteroides uniformis
Bacteroides vulgatus
Eubacterium eligens
Faecalibacterium prausnitzii
Roseburia intestinalis
Akkermansia muciniphila
Escherichia coli
other
unclassified
Aerotolerance
obligate_anaerobe
aerotolerant
facultative_anaerobe
unknown
Viruses
Bell pepper mottle virus
Pepper mild mottle virus
Tobacco mild green mosaic virus
Transcription
K00606: 3−methyl−2−oxobutanoate hydroxy...
K00873: pyruvate kinase [EC:2.7.1.40]
K01572: oxaloacetate decarboxylase, beta su...
K01951: GMP synthase (glutamine−hydrolysi...
K02406: flagellin
K02954: small subunit ribosomal protein S14
K03040: DNA−directed RNA polymerase sub...
K04069: pyruvate formate lyase activating en...
K04078: chaperonin GroES
K05808: putative sigma−54 modulation protein
K12343: 3−oxo−5−alpha−steroid 4−dehydrog...
Proteins
K00262: glutamate dehydrogenase (NADP+) ...
K01006: pyruvate, orthophosphate dikinase [...
K01610: phosphoenolpyruvate carboxykinase...
K01803: triosephosphate isomerase (TIM) [E...
K02355: elongation factor EF−G
K02358: elongation factor EF−Tu
K10117: raffinose/stachyose/melibiose transp...
K10200: N−acetylglucosamine transport syst...
UNGROUPED
Taxonomy
PCo 1 (8.1%)
PCo 2 (6.7%)
Transcripts
PCo 1 (14.2%)
PCo 2 (9.2%)
Proteins
PCo 1 (30.8%)
PCo 2 (12.3%)
Metabolites
PCo 1 (8.8%)
PCo 2 (7.0%)

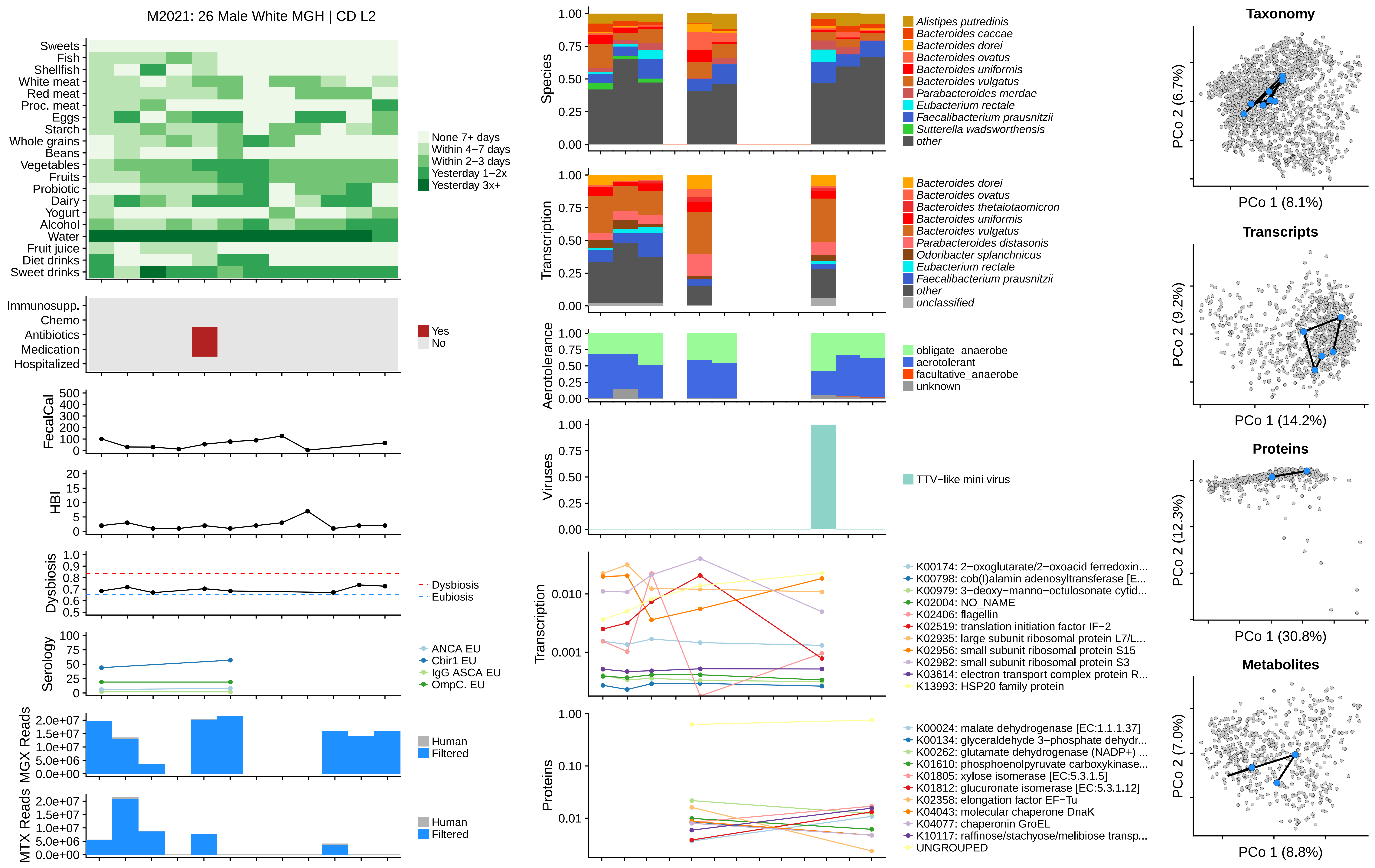
M2021: 26 Male White MGH | CD L2
Sweets
Fish
Shellfish
White meat
Red meat
Proc. meat
Eggs
Starch
Whole grains
Beans
Vegetables
Fruits
Probiotic
Dairy
Yogurt
Alcohol
Water
Fruit juice
Diet drinks
Sweet drinks
None 7+ days
Within 4−7 days
Within 2−3 days
Yesterday 1−2x
Yesterday 3x+
Immunosupp.
Chemo
Antibiotics
Medication
Hospitalized
Yes
No
FecalCal
HBI
Dysbiosis
Dysbiosis
Eubiosis
Serology
ANCA EU
Cbir1 EU
IgG ASCA EU
OmpC. EU
MGX Reads
Human
Filtered
MTX Reads
Human
Filtered
Species
Alistipes putredinis
Bacteroides caccae
Bacteroides dorei
Bacteroides ovatus
Bacteroides uniformis
Bacteroides vulgatus
Parabacteroides merdae
Eubacterium rectale
Faecalibacterium prausnitzii
Sutterella wadsworthensis
other
Transcription
Bacteroides dorei
Bacteroides ovatus
Bacteroides thetaiotaomicron
Bacteroides uniformis
Bacteroides vulgatus
Parabacteroides distasonis
Odoribacter splanchnicus
Eubacterium rectale
Faecalibacterium prausnitzii
other
unclassified
Aerotolerance
obligate_anaerobe
aerotolerant
facultative_anaerobe
unknown
Viruses
TTV−like mini virus
Transcription
K00174: 2−oxoglutarate/2−oxoacid ferredoxin...
K00798: cob(I)alamin adenosyltransferase [E...
K00979: 3−deoxy−manno−octulosonate cytid...
K02004: NO_NAME
K02406: flagellin
K02519: translation initiation factor IF−2
K02935: large subunit ribosomal protein L7/L...
K02956: small subunit ribosomal protein S15
K02982: small subunit ribosomal protein S3
K03614: electron transport complex protein R...
K13993: HSP20 family protein
Proteins
K00024: malate dehydrogenase [EC:1.1.1.37]
K00134: glyceraldehyde 3−phosphate dehydr...
K00262: glutamate dehydrogenase (NADP+) ...
K01610: phosphoenolpyruvate carboxykinase...
K01805: xylose isomerase [EC:5.3.1.5]
K01812: glucuronate isomerase [EC:5.3.1.12]
K02358: elongation factor EF−Tu
K04043: molecular chaperone DnaK
K04077: chaperonin GroEL
K10117: raffinose/stachyose/melibiose transp...
UNGROUPED
Taxonomy
PCo 1 (8.1%)
PCo 2 (6.7%)
Transcripts
PCo 1 (14.2%)
PCo 2 (9.2%)
Proteins
PCo 1 (30.8%)
PCo 2 (12.3%)
Metabolites
PCo 1 (8.8%)
PCo 2 (7.0%)

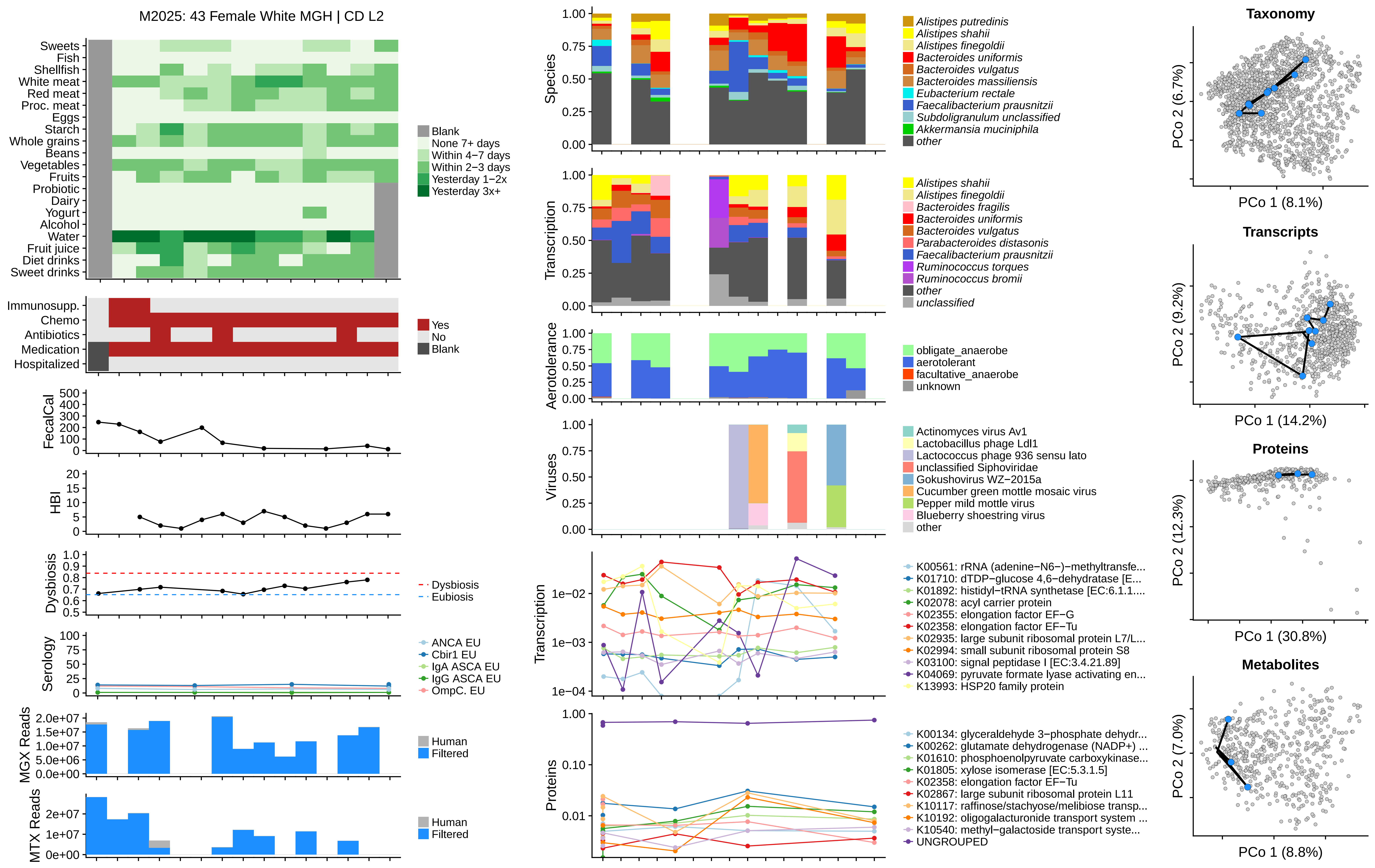
M2025: 43 Female White MGH | CD L2
Sweets
Fish
Shellfish
White meat
Red meat
Proc. meat
Eggs
Starch
Whole grains
Beans
Vegetables
Fruits
Probiotic
Dairy
Yogurt
Alcohol
Water
Fruit juice
Diet drinks
Sweet drinks
Blank
None 7+ days
Within 4−7 days
Within 2−3 days
Yesterday 1−2x
Yesterday 3x+
Immunosupp.
Chemo
Antibiotics
Medication
Hospitalized
Yes
No
Blank
FecalCal
HBI
Dysbiosis
Dysbiosis
Eubiosis
Serology
ANCA EU
Cbir1 EU
IgA ASCA EU
IgG ASCA EU
OmpC. EU
MGX Reads
Human
Filtered
MTX Reads
Human
Filtered
Species
Alistipes putredinis
Alistipes shahii
Alistipes finegoldii
Bacteroides uniformis
Bacteroides vulgatus
Bacteroides massiliensis
Eubacterium rectale
Faecalibacterium prausnitzii
Subdoligranulum unclassified
Akkermansia muciniphila
other
Transcription
Alistipes shahii
Alistipes finegoldii
Bacteroides fragilis
Bacteroides uniformis
Bacteroides vulgatus
Parabacteroides distasonis
Faecalibacterium prausnitzii
Ruminococcus torques
Ruminococcus bromii
other
unclassified
Aerotolerance
obligate_anaerobe
aerotolerant
facultative_anaerobe
unknown
Viruses
Actinomyces virus Av1
Lactobacillus phage Ldl1
Lactococcus phage 936 sensu lato
unclassified Siphoviridae
Gokushovirus WZ−2015a
Cucumber green mottle mosaic virus
Pepper mild mottle virus
Blueberry shoestring virus
other
Transcription
K00561: rRNA (adenine−N6−)−methyltransfe...
K01710: dTDP−glucose 4,6−dehydratase [E...
K01892: histidyl−tRNA synthetase [EC:6.1.1....
K02078: acyl carrier protein
K02355: elongation factor EF−G
K02358: elongation factor EF−Tu
K02935: large subunit ribosomal protein L7/L...
K02994: small subunit ribosomal protein S8
K03100: signal peptidase I [EC:3.4.21.89]
K04069: pyruvate formate lyase activating en...
K13993: HSP20 family protein
Proteins
K00134: glyceraldehyde 3−phosphate dehydr...
K00262: glutamate dehydrogenase (NADP+) ...
K01610: phosphoenolpyruvate carboxykinase...
K01805: xylose isomerase [EC:5.3.1.5]
K02358: elongation factor EF−Tu
K02867: large subunit ribosomal protein L11
K10117: raffinose/stachyose/melibiose transp...
K10192: oligogalacturonide transport system ...
K10540: methyl−galactoside transport syste...
UNGROUPED
Taxonomy
PCo 1 (8.1%)
PCo 2 (6.7%)
Transcripts
PCo 1 (14.2%)
PCo 2 (9.2%)
Proteins
PCo 1 (30.8%)
PCo 2 (12.3%)
Metabolites
PCo 1 (8.8%)
PCo 2 (7.0%)

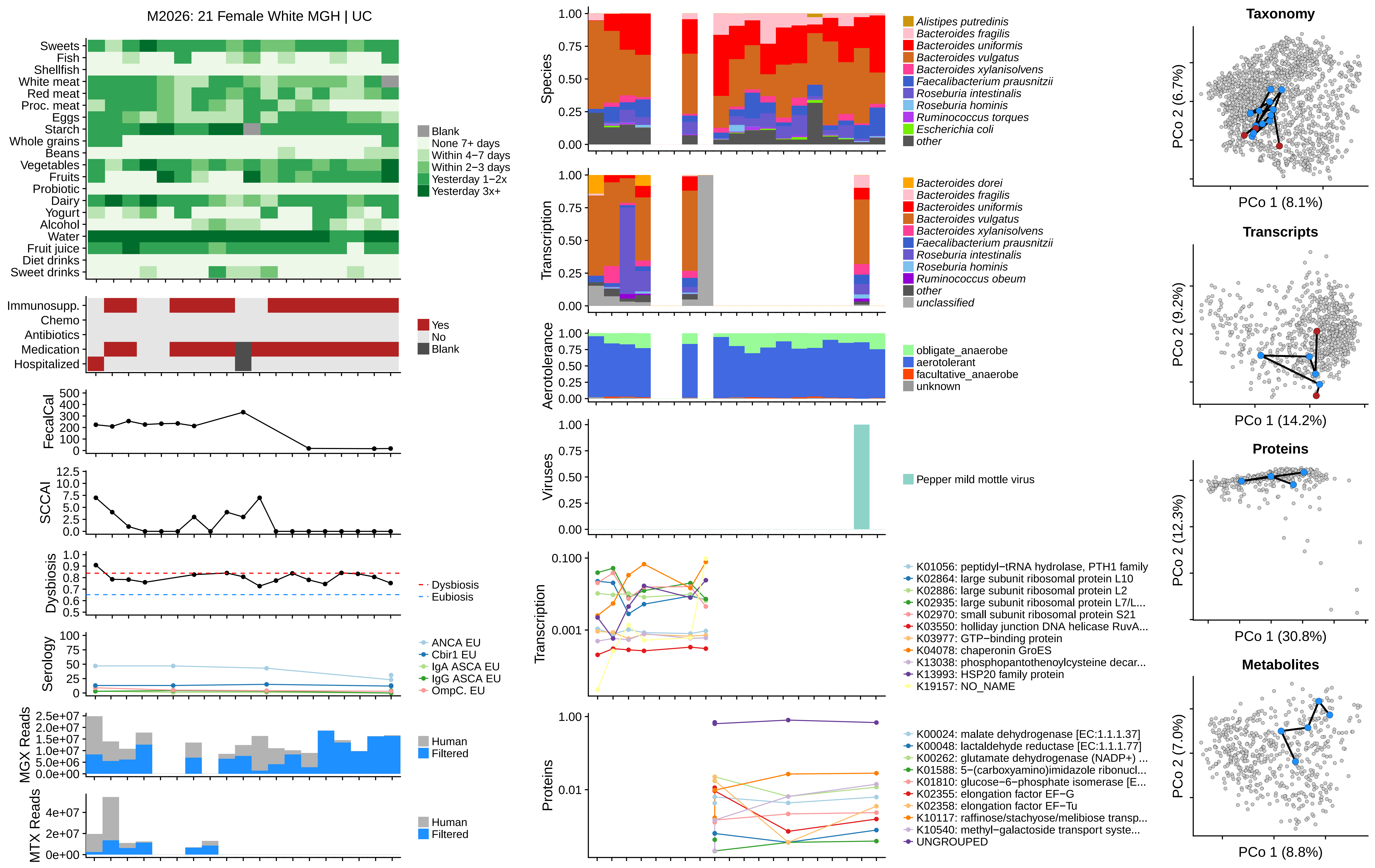

M2026: 21 Female White MGH | UC
Sweets
Fish
Shellfish
White meat
Red meat
Proc. meat
Eggs
Starch
Whole grains
Beans
Vegetables
Fruits
Probiotic
Dairy
Yogurt
Alcohol
Water
Fruit juice
Diet drinks
Sweet drinks
Blank
None 7+ days
Within 4−7 days
Within 2−3 days
Yesterday 1−2x
Yesterday 3x+
Immunosupp.
Chemo
Antibiotics
Medication
Hospitalized
Yes
No
Blank
FecalCal
SCCAI
Dysbiosis
Dysbiosis
Eubiosis
Serology
ANCA EU
Cbir1 EU
IgA ASCA EU
IgG ASCA EU
OmpC. EU
MGX Reads
Human
Filtered
MTX Reads
Human
Filtered
Species
Alistipes putredinis
Bacteroides fragilis
Bacteroides uniformis
Bacteroides vulgatus
Bacteroides xylanisolvens
Faecalibacterium prausnitzii
Roseburia intestinalis
Roseburia hominis
Ruminococcus torques
Escherichia coli
other
Transcription
Bacteroides dorei
Bacteroides fragilis
Bacteroides uniformis
Bacteroides vulgatus
Bacteroides xylanisolvens
Faecalibacterium prausnitzii
Roseburia intestinalis
Roseburia hominis
Ruminococcus obeum
other
unclassified
Aerotolerance
obligate_anaerobe
aerotolerant
facultative_anaerobe
unknown
Viruses
Pepper mild mottle virus
Transcription
K01056: peptidyl−tRNA hydrolase, PTH1 family
K02864: large subunit ribosomal protein L10
K02886: large subunit ribosomal protein L2
K02935: large subunit ribosomal protein L7/L...
K02970: small subunit ribosomal protein S21
K03550: holliday junction DNA helicase RuvA...
K03977: GTP−binding protein
K04078: chaperonin GroES
K13038: phosphopantothenoylcysteine decar...
K13993: HSP20 family protein
K19157: NO_NAME
Proteins
K00024: malate dehydrogenase [EC:1.1.1.37]
K00048: lactaldehyde reductase [EC:1.1.1.77]
K00262: glutamate dehydrogenase (NADP+) ...
K01588: 5−(carboxyamino)imidazole ribonucl...
K01810: glucose−6−phosphate isomerase [E...
K02355: elongation factor EF−G
K02358: elongation factor EF−Tu
K10117: raffinose/stachyose/melibiose transp...
K10540: methyl−galactoside transport syste...
UNGROUPED
Taxonomy
PCo 1 (8.1%)
PCo 2 (6.7%)
Transcripts
PCo 1 (14.2%)
PCo 2 (9.2%)
Proteins
PCo 1 (30.8%)
PCo 2 (12.3%)
Metabolites
PCo 1 (8.8%)
PCo 2 (7.0%)

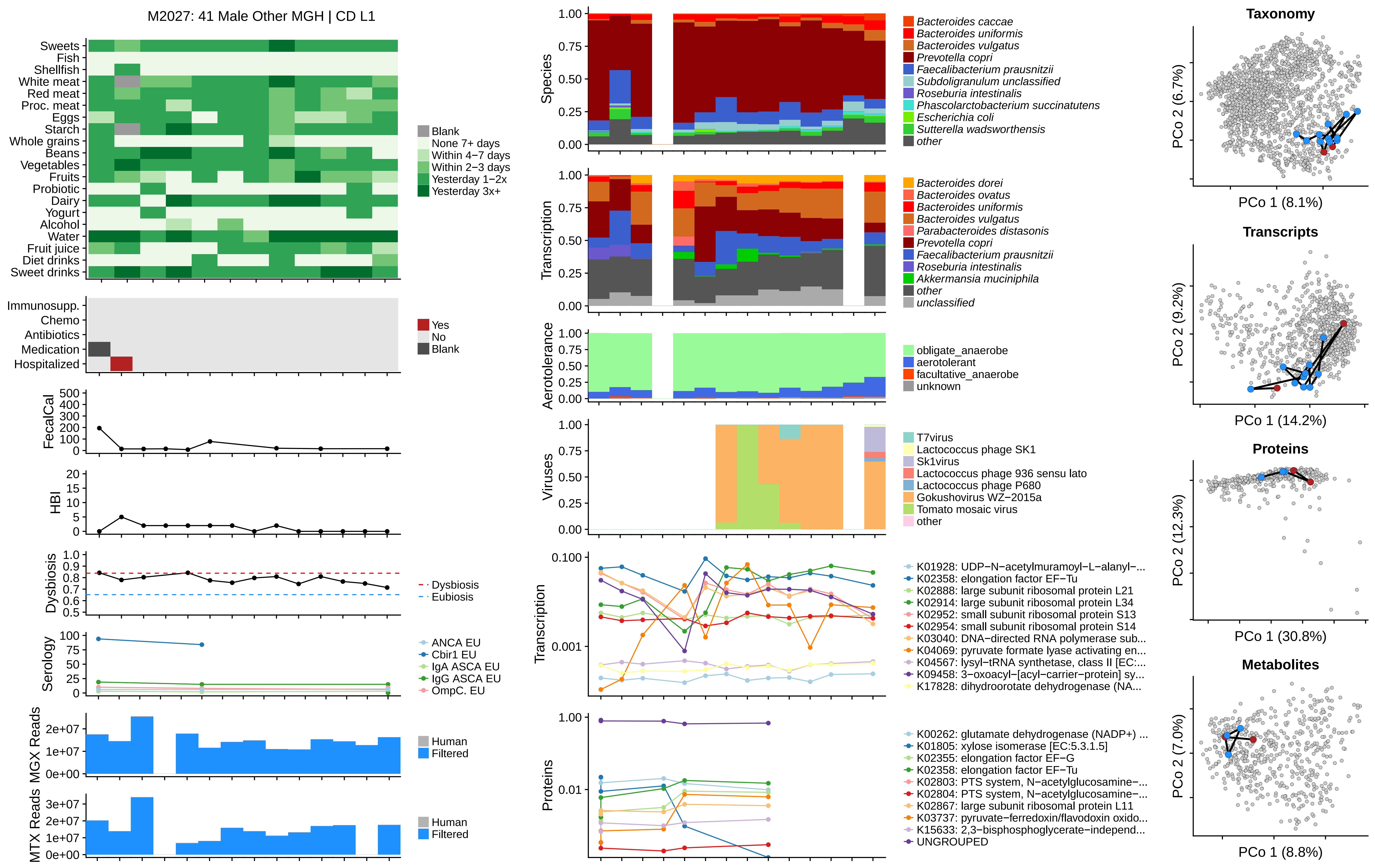

M2027: 41 Male Other MGH | CD L1
Sweets
Fish
Shellfish
White meat
Red meat
Proc. meat
Eggs
Starch
Whole grains
Beans
Vegetables
Fruits
Probiotic
Dairy
Yogurt
Alcohol
Water
Fruit juice
Diet drinks
Sweet drinks
Blank
None 7+ days
Within 4−7 days
Within 2−3 days
Yesterday 1−2x
Yesterday 3x+
Immunosupp.
Chemo
Antibiotics
Medication
Hospitalized
Yes
No
Blank
FecalCal
HBI
Dysbiosis
Dysbiosis
Eubiosis
Serology
ANCA EU
Cbir1 EU
IgA ASCA EU
IgG ASCA EU
OmpC. EU
MGX Reads
Human
Filtered
MTX Reads
Human
Filtered
Species
Bacteroides caccae
Bacteroides uniformis
Bacteroides vulgatus
Prevotella copri
Faecalibacterium prausnitzii
Subdoligranulum unclassified
Roseburia intestinalis
Phascolarctobacterium succinatutens
Escherichia coli
Sutterella wadsworthensis
other
Transcription
Bacteroides dorei
Bacteroides ovatus
Bacteroides uniformis
Bacteroides vulgatus
Parabacteroides distasonis
Prevotella copri
Faecalibacterium prausnitzii
Roseburia intestinalis
Akkermansia muciniphila
other
unclassified
Aerotolerance
obligate_anaerobe
aerotolerant
facultative_anaerobe
unknown
Viruses
T7virus
Lactococcus phage SK1
Sk1virus
Lactococcus phage 936 sensu lato
Lactococcus phage P680
Gokushovirus WZ−2015a
Tomato mosaic virus
other
Transcription
K01928: UDP−N−acetylmuramoyl−L−alanyl−...
K02358: elongation factor EF−Tu
K02888: large subunit ribosomal protein L21
K02914: large subunit ribosomal protein L34
K02952: small subunit ribosomal protein S13
K02954: small subunit ribosomal protein S14
K03040: DNA−directed RNA polymerase sub...
K04069: pyruvate formate lyase activating en...
K04567: lysyl−tRNA synthetase, class II [EC:...
K09458: 3−oxoacyl−[acyl−carrier−protein] sy...
K17828: dihydroorotate dehydrogenase (NA...
Proteins
K00262: glutamate dehydrogenase (NADP+) ...
K01805: xylose isomerase [EC:5.3.1.5]
K02355: elongation factor EF−G
K02358: elongation factor EF−Tu
K02803: PTS system, N−acetylglucosamine−...
K02804: PTS system, N−acetylglucosamine−...
K02867: large subunit ribosomal protein L11
K03737: pyruvate−ferredoxin/flavodoxin oxido...
K15633: 2,3−bisphosphoglycerate−independ...
UNGROUPED
Taxonomy
PCo 1 (8.1%)
PCo 2 (6.7%)
Transcripts
PCo 1 (14.2%)
PCo 2 (9.2%)
Proteins
PCo 1 (30.8%)
PCo 2 (12.3%)
Metabolites
PCo 1 (8.8%)
PCo 2 (7.0%)

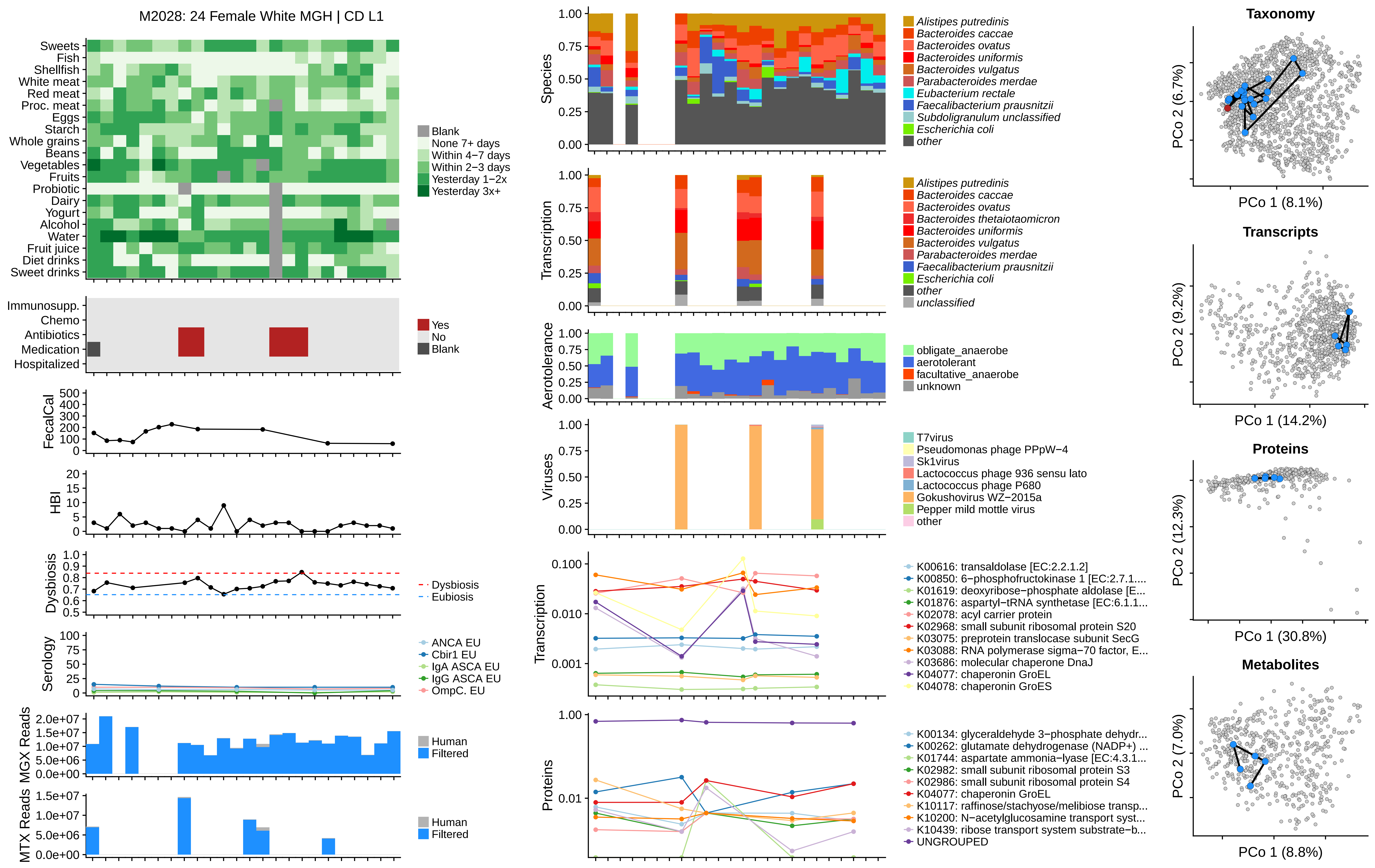

M2028: 24 Female White MGH | CD L1
Sweets
Fish
Shellfish
White meat
Red meat
Proc. meat
Eggs
Starch
Whole grains
Beans
Vegetables
Fruits
Probiotic
Dairy
Yogurt
Alcohol
Water
Fruit juice
Diet drinks
Sweet drinks
Blank
None 7+ days
Within 4−7 days
Within 2−3 days
Yesterday 1−2x
Yesterday 3x+
Immunosupp.
Chemo
Antibiotics
Medication
Hospitalized
Yes
No
Blank
FecalCal
HBI
Dysbiosis
Dysbiosis
Eubiosis
Serology
ANCA EU
Cbir1 EU
IgA ASCA EU
IgG ASCA EU
OmpC. EU
MGX Reads
Human
Filtered
MTX Reads
Human
Filtered
Species
Alistipes putredinis
Bacteroides caccae
Bacteroides ovatus
Bacteroides uniformis
Bacteroides vulgatus
Parabacteroides merdae
Eubacterium rectale
Faecalibacterium prausnitzii
Subdoligranulum unclassified
Escherichia coli
other
Transcription
Alistipes putredinis
Bacteroides caccae
Bacteroides ovatus
Bacteroides thetaiotaomicron
Bacteroides uniformis
Bacteroides vulgatus
Parabacteroides merdae
Faecalibacterium prausnitzii
Escherichia coli
other
unclassified
Aerotolerance
obligate_anaerobe
aerotolerant
facultative_anaerobe
unknown
Viruses
T7virus
Pseudomonas phage PPpW−4
Sk1virus
Lactococcus phage 936 sensu lato
Lactococcus phage P680
Gokushovirus WZ−2015a
Pepper mild mottle virus
other
Transcription
K00616: transaldolase [EC:2.2.1.2]
K00850: 6−phosphofructokinase 1 [EC:2.7.1....
K01619: deoxyribose−phosphate aldolase [E...
K01876: aspartyl−tRNA synthetase [EC:6.1.1...
K02078: acyl carrier protein
K02968: small subunit ribosomal protein S20
K03075: preprotein translocase subunit SecG
K03088: RNA polymerase sigma−70 factor, E...
K03686: molecular chaperone DnaJ
K04077: chaperonin GroEL
K04078: chaperonin GroES
Proteins
K00134: glyceraldehyde 3−phosphate dehydr...
K00262: glutamate dehydrogenase (NADP+) ...
K01744: aspartate ammonia−lyase [EC:4.3.1...
K02982: small subunit ribosomal protein S3
K02986: small subunit ribosomal protein S4
K04077: chaperonin GroEL
K10117: raffinose/stachyose/melibiose transp...
K10200: N−acetylglucosamine transport syst...
K10439: ribose transport system substrate−b...
UNGROUPED
Taxonomy
PCo 1 (8.1%)
PCo 2 (6.7%)
Transcripts
PCo 1 (14.2%)
PCo 2 (9.2%)
Proteins
PCo 1 (30.8%)
PCo 2 (12.3%)
Metabolites
PCo 1 (8.8%)
PCo 2 (7.0%)

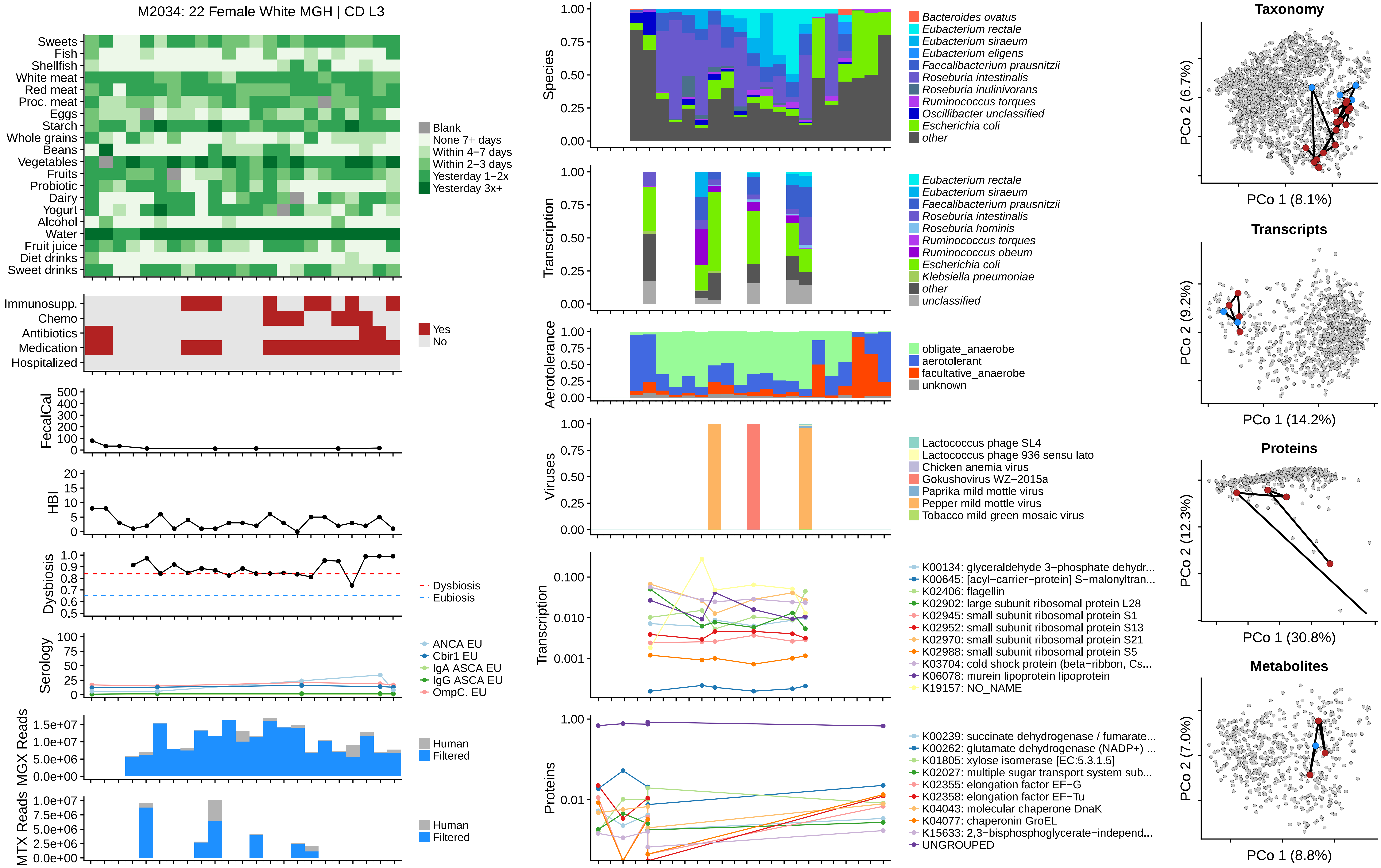

M2034: 22 Female White MGH | CD L3
Sweets
Fish
Shellfish
White meat
Red meat
Proc. meat
Eggs
Starch
Whole grains
Beans
Vegetables
Fruits
Probiotic
Dairy
Yogurt
Alcohol
Water
Fruit juice
Diet drinks
Sweet drinks
Blank
None 7+ days
Within 4−7 days
Within 2−3 days
Yesterday 1−2x
Yesterday 3x+
Immunosupp.
Chemo
Antibiotics
Medication
Hospitalized
Yes
No
FecalCal
HBI
Dysbiosis
Dysbiosis
Eubiosis
Serology
ANCA EU
Cbir1 EU
IgA ASCA EU
IgG ASCA EU
OmpC. EU
MGX Reads
Human
Filtered
MTX Reads
Human
Filtered
Species
Bacteroides ovatus
Eubacterium rectale
Eubacterium siraeum
Eubacterium eligens
Faecalibacterium prausnitzii
Roseburia intestinalis
Roseburia inulinivorans
Ruminococcus torques
Oscillibacter unclassified
Escherichia coli
other
Transcription
Eubacterium rectale
Eubacterium siraeum
Faecalibacterium prausnitzii
Roseburia intestinalis
Roseburia hominis
Ruminococcus torques
Ruminococcus obeum
Escherichia coli
Klebsiella pneumoniae
other
unclassified
Aerotolerance
obligate_anaerobe
aerotolerant
facultative_anaerobe
unknown
Viruses
Lactococcus phage SL4
Lactococcus phage 936 sensu lato
Chicken anemia virus
Gokushovirus WZ−2015a
Paprika mild mottle virus
Pepper mild mottle virus
Tobacco mild green mosaic virus
Transcription
K00134: glyceraldehyde 3−phosphate dehydr...
K00645: [acyl−carrier−protein] S−malonyltran...
K02406: flagellin
K02902: large subunit ribosomal protein L28
K02945: small subunit ribosomal protein S1
K02952: small subunit ribosomal protein S13
K02970: small subunit ribosomal protein S21
K02988: small subunit ribosomal protein S5
K03704: cold shock protein (beta−ribbon, Cs...
K06078: murein lipoprotein lipoprotein
K19157: NO_NAME
Proteins
K00239: succinate dehydrogenase / fumarate...
K00262: glutamate dehydrogenase (NADP+) ...
K01805: xylose isomerase [EC:5.3.1.5]
K02027: multiple sugar transport system sub...
K02355: elongation factor EF−G
K02358: elongation factor EF−Tu
K04043: molecular chaperone DnaK
K04077: chaperonin GroEL
K15633: 2,3−bisphosphoglycerate−independ...
UNGROUPED
Taxonomy
PCo 1 (8.1%)
PCo 2 (6.7%)
Transcripts
PCo 1 (14.2%)
PCo 2 (9.2%)
Proteins
PCo 1 (30.8%)
PCo 2 (12.3%)
Metabolites
PCo 1 (8.8%)
PCo 2 (7.0%)

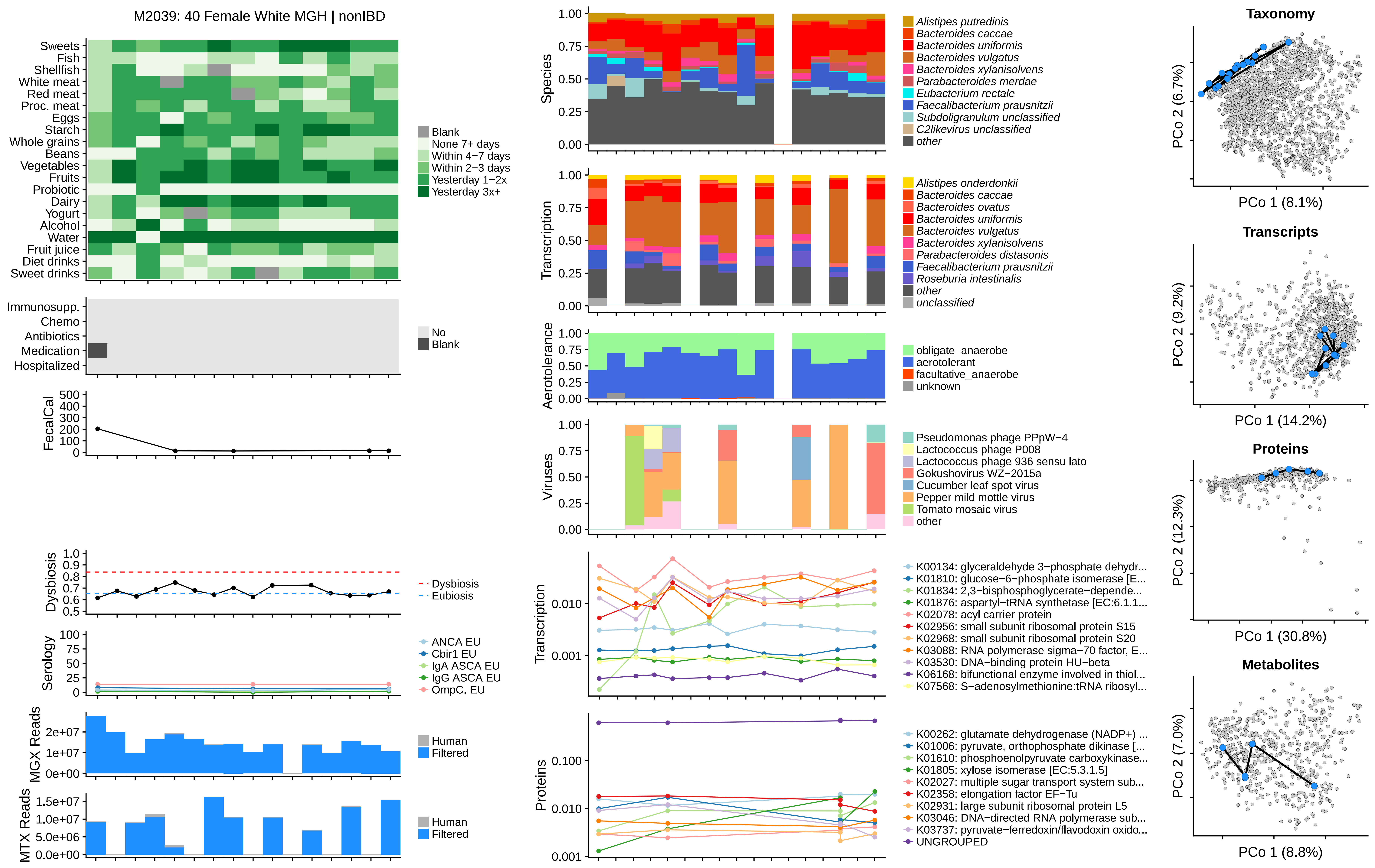
M2039: 40 Female White MGH | nonIBD
Sweets
Fish
Shellfish
White meat
Red meat
Proc. meat
Eggs
Starch
Whole grains
Beans
Vegetables
Fruits
Probiotic
Dairy
Yogurt
Alcohol
Water
Fruit juice
Diet drinks
Sweet drinks
Blank
None 7+ days
Within 4−7 days
Within 2−3 days
Yesterday 1−2x
Yesterday 3x+
Immunosupp.
Chemo
Antibiotics
Medication
Hospitalized
No
Blank
FecalCal
Dysbiosis
Dysbiosis
Eubiosis
Serology
ANCA EU
Cbir1 EU
IgA ASCA EU
IgG ASCA EU
OmpC. EU
MGX Reads
Human
Filtered
MTX Reads
Human
Filtered
Species
Alistipes putredinis
Bacteroides caccae
Bacteroides uniformis
Bacteroides vulgatus
Bacteroides xylanisolvens
Parabacteroides merdae
Eubacterium rectale
Faecalibacterium prausnitzii
Subdoligranulum unclassified
C2likevirus unclassified
other
Transcription
Alistipes onderdonkii
Bacteroides caccae
Bacteroides ovatus
Bacteroides uniformis
Bacteroides vulgatus
Bacteroides xylanisolvens
Parabacteroides distasonis
Faecalibacterium prausnitzii
Roseburia intestinalis
other
unclassified
Aerotolerance
obligate_anaerobe
aerotolerant
facultative_anaerobe
unknown
Viruses
Pseudomonas phage PPpW−4
Lactococcus phage P008
Lactococcus phage 936 sensu lato
Gokushovirus WZ−2015a
Cucumber leaf spot virus
Pepper mild mottle virus
Tomato mosaic virus
other
Transcription
K00134: glyceraldehyde 3−phosphate dehydr...
K01810: glucose−6−phosphate isomerase [E...
K01834: 2,3−bisphosphoglycerate−depende...
K01876: aspartyl−tRNA synthetase [EC:6.1.1...
K02078: acyl carrier protein
K02956: small subunit ribosomal protein S15
K02968: small subunit ribosomal protein S20
K03088: RNA polymerase sigma−70 factor, E...
K03530: DNA−binding protein HU−beta
K06168: bifunctional enzyme involved in thiol...
K07568: S−adenosylmethionine:tRNA ribosyl...
Proteins
K00262: glutamate dehydrogenase (NADP+) ...
K01006: pyruvate, orthophosphate dikinase [...
K01610: phosphoenolpyruvate carboxykinase...
K01805: xylose isomerase [EC:5.3.1.5]
K02027: multiple sugar transport system sub...
K02358: elongation factor EF−Tu
K02931: large subunit ribosomal protein L5
K03046: DNA−directed RNA polymerase sub...
K03737: pyruvate−ferredoxin/flavodoxin oxido...
UNGROUPED
Taxonomy
PCo 1 (8.1%)
PCo 2 (6.7%)
Transcripts
PCo 1 (14.2%)
PCo 2 (9.2%)
Proteins
PCo 1 (30.8%)
PCo 2 (12.3%)
Metabolites
PCo 1 (8.8%)
PCo 2 (7.0%)

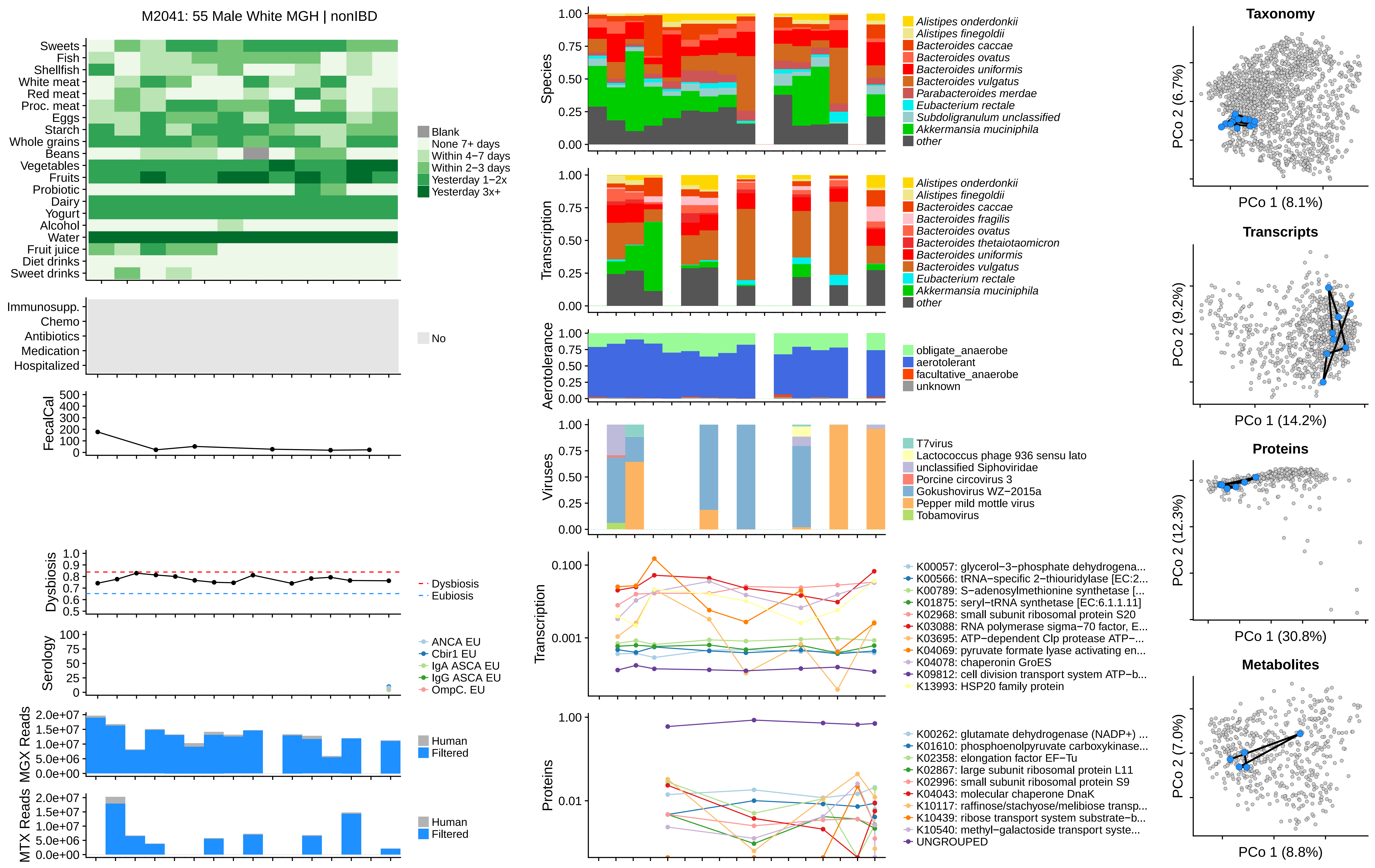
M2041: 55 Male White MGH | nonIBD
Sweets
Fish
Shellfish
White meat
Red meat
Proc. meat
Eggs
Starch
Whole grains
Beans
Vegetables
Fruits
Probiotic
Dairy
Yogurt
Alcohol
Water
Fruit juice
Diet drinks
Sweet drinks
Blank
None 7+ days
Within 4−7 days
Within 2−3 days
Yesterday 1−2x
Yesterday 3x+
Immunosupp.
Chemo
Antibiotics
Medication
Hospitalized
No
FecalCal
Dysbiosis
Eubiosis
Serology
ANCA EU
Cbir1 EU
IgA ASCA EU
IgG ASCA EU
OmpC. EU
MGX Reads
MTX Reads
Human
Filtered
Species
Alistipes onderdonkii
Alistipes finegoldii
Bacteroides caccae
Bacteroides ovatus
Bacteroides uniformis
Bacteroides vulgatus
Parabacteroides merdae
Eubacterium rectale
Subdoligranulum unclassified
Akkermansia muciniphila
other
Transcription
Bacteroides fragilis
Bacteroides thetaiotaomicron
Aerotolerance
obligate_anaerobe
aerotolerant
facultative_anaerobe
unknown
Viruses
T7virus
Lactococcus phage 936 sensu lato
unclassified Siphoviridae
Porcine circovirus 3
Gokushovirus WZ−2015a
Pepper mild mottle virus
Tobamovirus
K00057: glycerol−3−phosphate dehydrogena...
K00566: tRNA−specific 2−thiouridylase [EC:2...
K00789: S−adenosylmethionine synthetase [...
K01875: seryl−tRNA synthetase [EC:6.1.1.11]
K02968: small subunit ribosomal protein S20
K03088: RNA polymerase sigma−70 factor, E...
K03695: ATP−dependent Clp protease ATP−...
K04069: pyruvate formate lyase activating en...
K04078: chaperonin GroES
K09812: cell division transport system ATP−b...
K13993: HSP20 family protein
Proteins
K00262: glutamate dehydrogenase (NADP+) ...
K01610: phosphoenolpyruvate carboxykinase...
K02358: elongation factor EF−Tu
K02867: large subunit ribosomal protein L11
K02996: small subunit ribosomal protein S9
K04043: molecular chaperone DnaK
K10117: raffinose/stachyose/melibiose transp...
K10439: ribose transport system substrate−b...
K10540: methyl−galactoside transport syste...
UNGROUPED
Taxonomy
PCo 1 (8.1%)
PCo 2 (6.7%)
Transcripts
PCo 1 (14.2%)
PCo 2 (9.2%)
Proteins
PCo 1 (30.8%)
PCo 2 (12.3%)
Metabolites
PCo 1 (8.8%)
PCo 2 (7.0%)

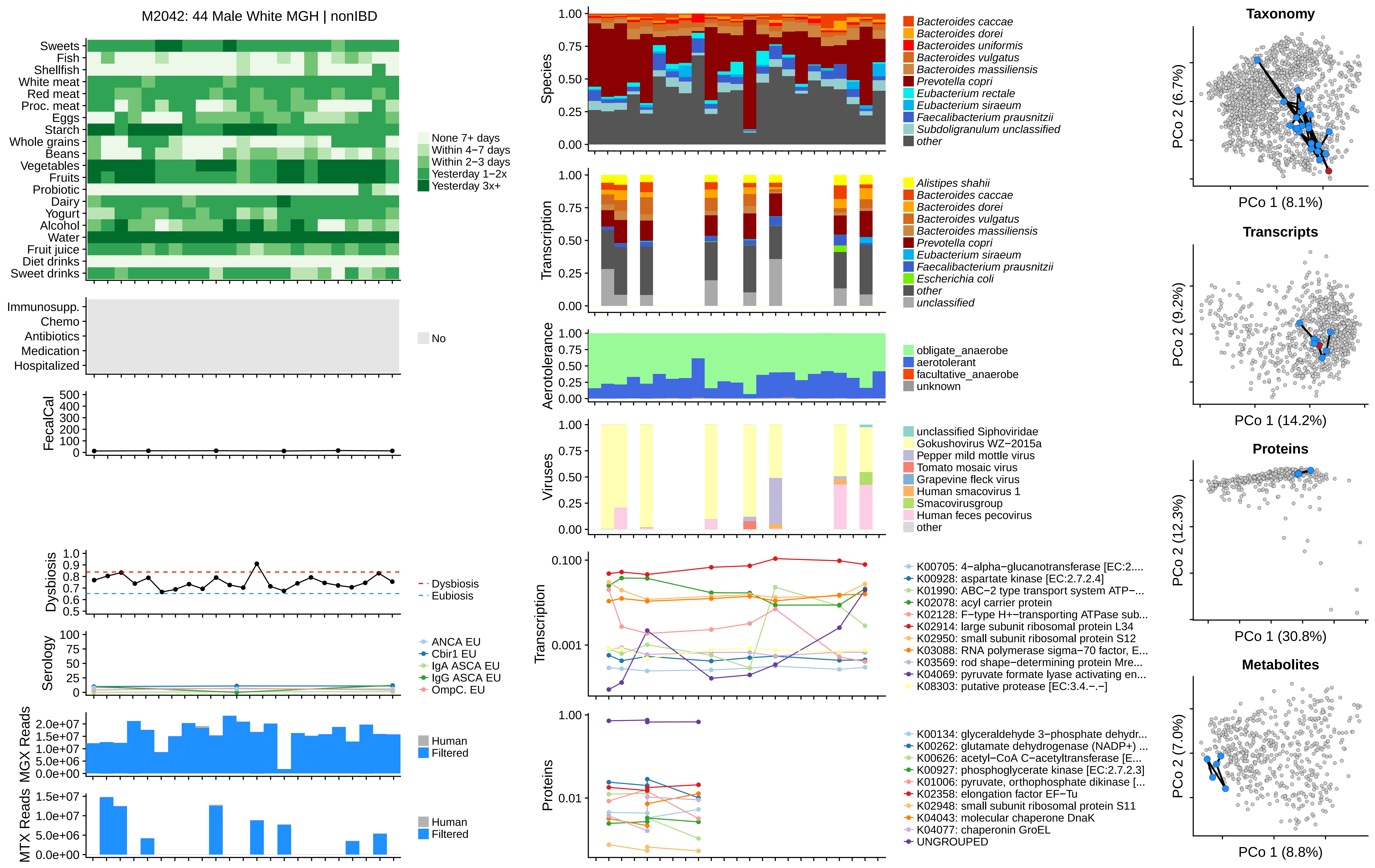
M2042: 44 Male White MGH | nonIBD
Sweets
Fish
Shellfish
White meat
Red meat
Proc. meat
Eggs
Starch
Whole grains
Beans
Vegetables
Fruits
Probiotic
Dairy
Yogurt
Alcohol
Water
Fruit juice
Diet drinks
Sweet drinks
None 7+ days
Within 4−7 days
Within 2−3 days
Yesterday 1−2x
Yesterday 3x+
Immunosupp.
Chemo
Antibiotics
Medication
Hospitalized
No
FecalCal
Dysbiosis
Dysbiosis
Eubiosis
Serology
ANCA EU
Cbir1 EU
IgA ASCA EU
IgG ASCA EU
OmpC. EU
MGX Reads
Human
Filtered
MTX Reads
Human
Filtered
Species
Bacteroides caccae
Bacteroides dorei
Bacteroides uniformis
Bacteroides vulgatus
Bacteroides massiliensis
Prevotella copri
Eubacterium rectale
Eubacterium siraeum
Faecalibacterium prausnitzii
Subdoligranulum unclassified
other
Transcription
Alistipes shahii
Bacteroides caccae
Bacteroides dorei
Bacteroides vulgatus
Bacteroides massiliensis
Prevotella copri
Eubacterium siraeum
Faecalibacterium prausnitzii
Escherichia coli
other
unclassified
Aerotolerance
obligate_anaerobe
aerotolerant
facultative_anaerobe
unknown
Viruses
unclassified Siphoviridae
Gokushovirus WZ−2015a
Pepper mild mottle virus
Tomato mosaic virus
Grapevine fleck virus
Human smacovirus 1
Smacovirusgroup
Human feces pecovirus
other
Transcription
K00705: 4−alpha−glucanotransferase [EC:2....
K00928: aspartate kinase [EC:2.7.2.4]
K01990: ABC−2 type transport system ATP−...
K02078: acyl carrier protein
K02128: F−type H+−transporting ATPase sub...
K02914: large subunit ribosomal protein L34
K02950: small subunit ribosomal protein S12
K03088: RNA polymerase sigma−70 factor, E...
K03569: rod shape−determining protein Mre...
K04069: pyruvate formate lyase activating en...
K08303: putative protease [EC:3.4.−.−]
Proteins
K00134: glyceraldehyde 3−phosphate dehydr...
K00262: glutamate dehydrogenase (NADP+) ...
K00626: acetyl−CoA C−acetyltransferase [E...
K00927: phosphoglycerate kinase [EC:2.7.2.3]
K01006: pyruvate, orthophosphate dikinase [...
K02358: elongation factor EF−Tu
K02948: small subunit ribosomal protein S11
K04043: molecular chaperone DnaK
K04077: chaperonin GroEL
UNGROUPED
Taxonomy
PCo 1 (8.1%)
PCo 2 (6.7%)
Transcripts
PCo 1 (14.2%)
PCo 2 (9.2%)
Proteins
PCo 1 (30.8%)
PCo 2 (12.3%)
Metabolites
PCo 1 (8.8%)
PCo 2 (7.0%)

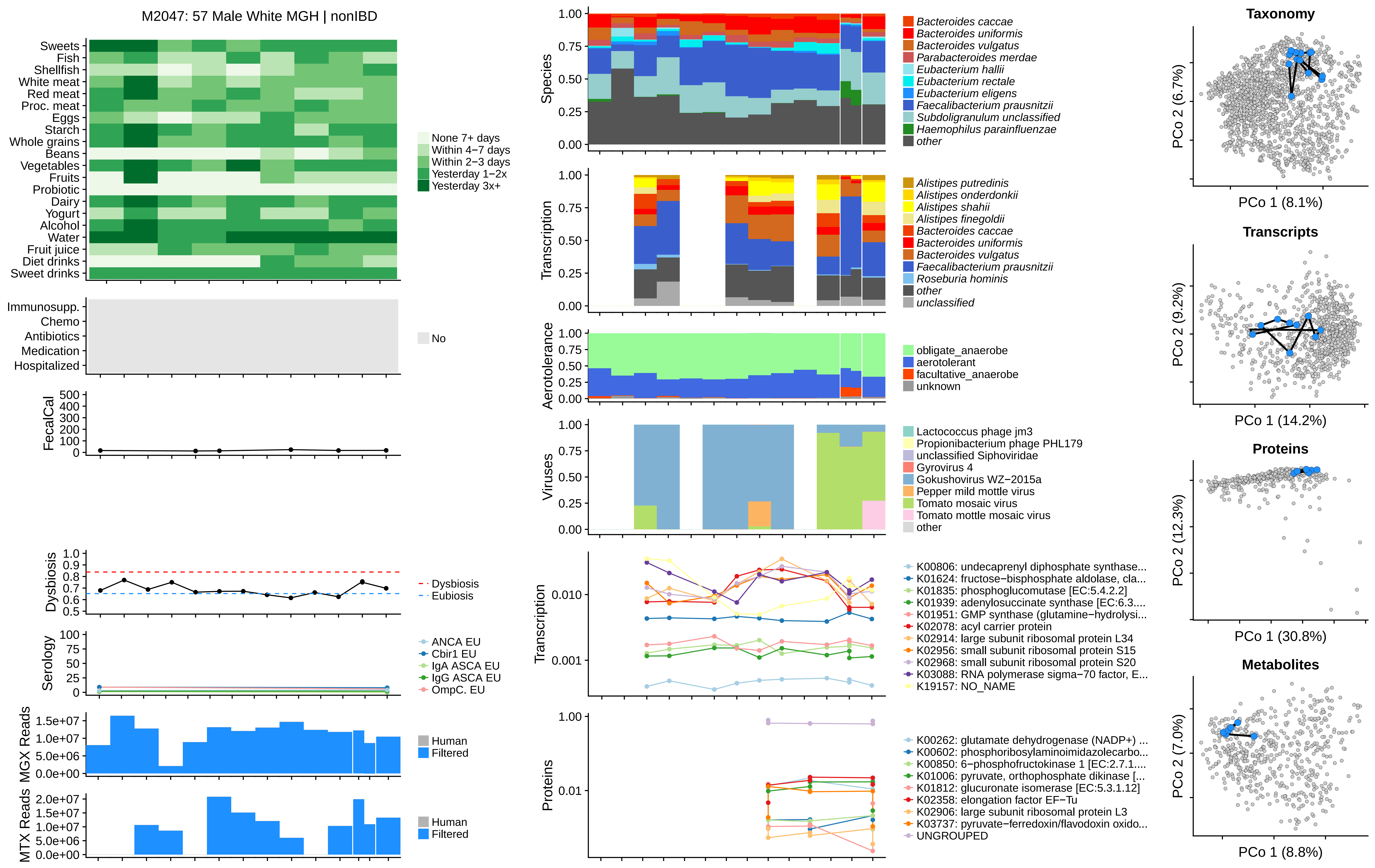

M2047: 57 Male White MGH | nonIBD
Sweets
Fish
Shellfish
White meat
Red meat
Proc. meat
Eggs
Starch
Whole grains
Beans
Vegetables
Fruits
Probiotic
Dairy
Yogurt
Alcohol
Water
Fruit juice
Diet drinks
Sweet drinks
None 7+ days
Within 4−7 days
Within 2−3 days
Yesterday 1−2x
Yesterday 3x+
Immunosupp.
Chemo
Antibiotics
Medication
Hospitalized
No
FecalCal
Dysbiosis
Dysbiosis
Eubiosis
Serology
ANCA EU
Cbir1 EU
IgA ASCA EU
IgG ASCA EU
OmpC. EU
MGX Reads
Human
Filtered
MTX Reads
Human
Filtered
Species
Bacteroides caccae
Bacteroides uniformis
Bacteroides vulgatus
Parabacteroides merdae
Eubacterium hallii
Eubacterium rectale
Eubacterium eligens
Faecalibacterium prausnitzii
Subdoligranulum unclassified
Haemophilus parainfluenzae
other
Transcription
Alistipes putredinis
Alistipes onderdonkii
Alistipes shahii
Alistipes finegoldii
Bacteroides caccae
Bacteroides uniformis
Bacteroides vulgatus
Faecalibacterium prausnitzii
Roseburia hominis
other
unclassified
Aerotolerance
obligate_anaerobe
aerotolerant
facultative_anaerobe
unknown
Viruses
Lactococcus phage jm3
Propionibacterium phage PHL179
unclassified Siphoviridae
Gyrovirus 4
Gokushovirus WZ−2015a
Pepper mild mottle virus
Tomato mosaic virus
Tomato mottle mosaic virus
other
Transcription
K00806: undecaprenyl diphosphate synthase...
K01624: fructose−bisphosphate aldolase, cla...
K01835: phosphoglucomutase [EC:5.4.2.2]
K01939: adenylosuccinate synthase [EC:6.3....
K01951: GMP synthase (glutamine−hydrolysi...
K02078: acyl carrier protein
K02914: large subunit ribosomal protein L34
K02956: small subunit ribosomal protein S15
K02968: small subunit ribosomal protein S20
K03088: RNA polymerase sigma−70 factor, E...
K19157: NO_NAME
Proteins
K00262: glutamate dehydrogenase (NADP+) ...
K00602: phosphoribosylaminoimidazolecarbo...
K00850: 6−phosphofructokinase 1 [EC:2.7.1....
K01006: pyruvate, orthophosphate dikinase [...
K01812: glucuronate isomerase [EC:5.3.1.12]
K02358: elongation factor EF−Tu
K02906: large subunit ribosomal protein L3
K03737: pyruvate−ferredoxin/flavodoxin oxido...
UNGROUPED
Taxonomy
PCo 1 (8.1%)
PCo 2 (6.7%)
Transcripts
PCo 1 (14.2%)
PCo 2 (9.2%)
Proteins
PCo 1 (30.8%)
PCo 2 (12.3%)
Metabolites
PCo 1 (8.8%)
PCo 2 (7.0%)

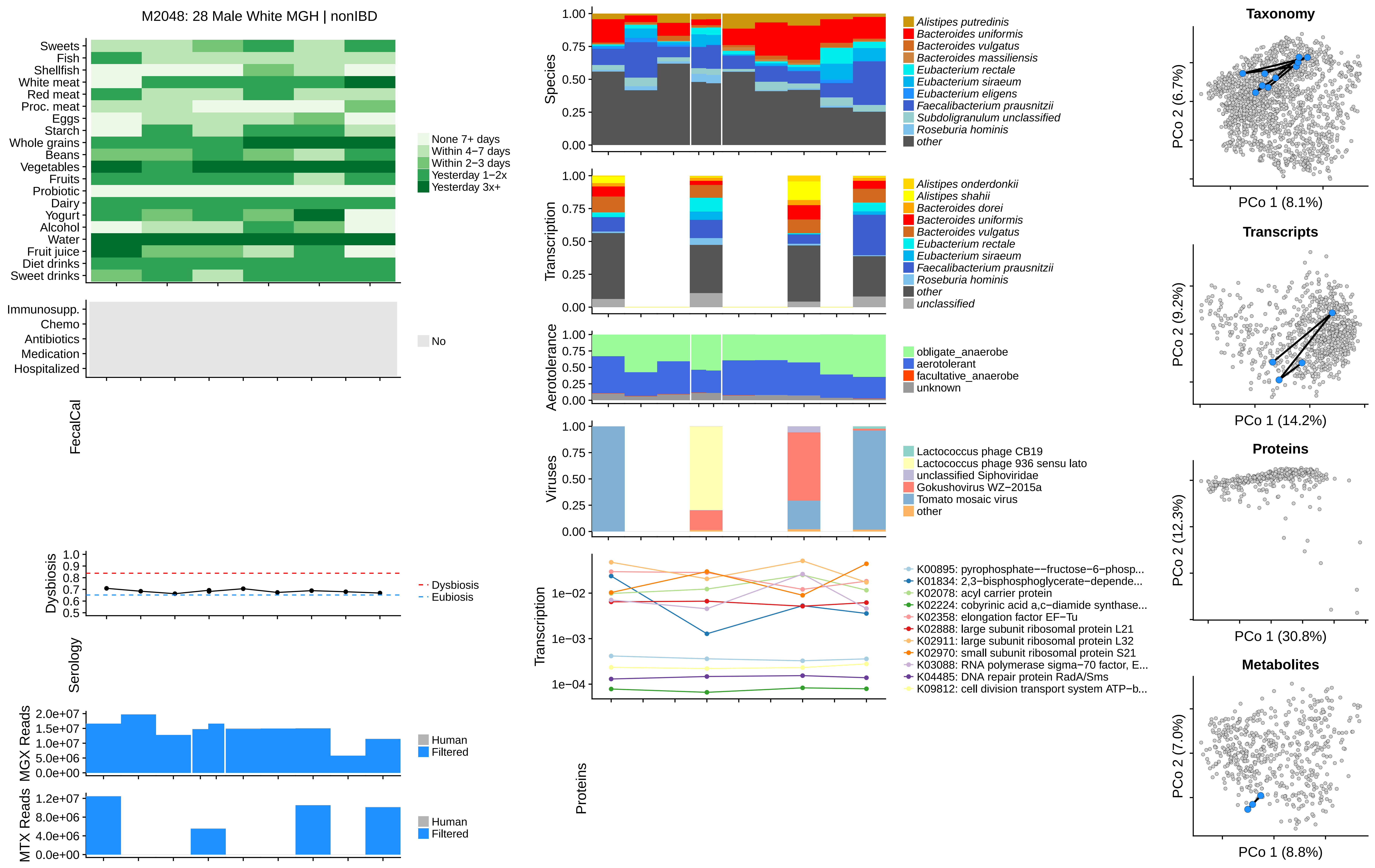

M2048: 28 Male White MGH | nonIBD
Sweets
Fish
Shellfish
White meat
Red meat
Proc. meat
Eggs
Starch
Whole grains
Beans
Vegetables
Fruits
Probiotic
Dairy
Yogurt
Alcohol
Water
Fruit juice
Diet drinks
Sweet drinks
None 7+ days
Within 4−7 days
Within 2−3 days
Yesterday 1−2x
Yesterday 3x+
Immunosupp.
Chemo
Antibiotics
Medication
Hospitalized
No
FecalCal
Dysbiosis
Dysbiosis
Eubiosis
Serology
MGX Reads
Human
Filtered
MTX Reads
Human
Filtered
Species
Alistipes putredinis
Bacteroides uniformis
Bacteroides vulgatus
Bacteroides massiliensis
Eubacterium rectale
Eubacterium siraeum
Eubacterium eligens
Faecalibacterium prausnitzii
Subdoligranulum unclassified
Roseburia hominis
other
Transcription
Alistipes onderdonkii
Alistipes shahii
Bacteroides dorei
Bacteroides uniformis
Bacteroides vulgatus
Eubacterium rectale
Eubacterium siraeum
Faecalibacterium prausnitzii
Roseburia hominis
other
unclassified
Aerotolerance
obligate_anaerobe
aerotolerant
facultative_anaerobe
unknown
Viruses
Lactococcus phage CB19
Lactococcus phage 936 sensu lato
unclassified Siphoviridae
Gokushovirus WZ−2015a
Tomato mosaic virus
other
Transcription
K00895: pyrophosphate−−fructose−6−phosp...
K01834: 2,3−bisphosphoglycerate−depende...
K02078: acyl carrier protein
K02224: cobyrinic acid a,c−diamide synthase...
K02358: elongation factor EF−Tu
K02888: large subunit ribosomal protein L21
K02911: large subunit ribosomal protein L32
K02970: small subunit ribosomal protein S21
K03088: RNA polymerase sigma−70 factor, E...
K04485: DNA repair protein RadA/Sms
K09812: cell division transport system ATP−b...
Proteins
Taxonomy
PCo 1 (8.1%)
PCo 2 (6.7%)
Transcripts
PCo 1 (14.2%)
PCo 2 (9.2%)
Proteins
PCo 1 (30.8%)
PCo 2 (12.3%)
Metabolites
PCo 1 (8.8%)
PCo 2 (7.0%)

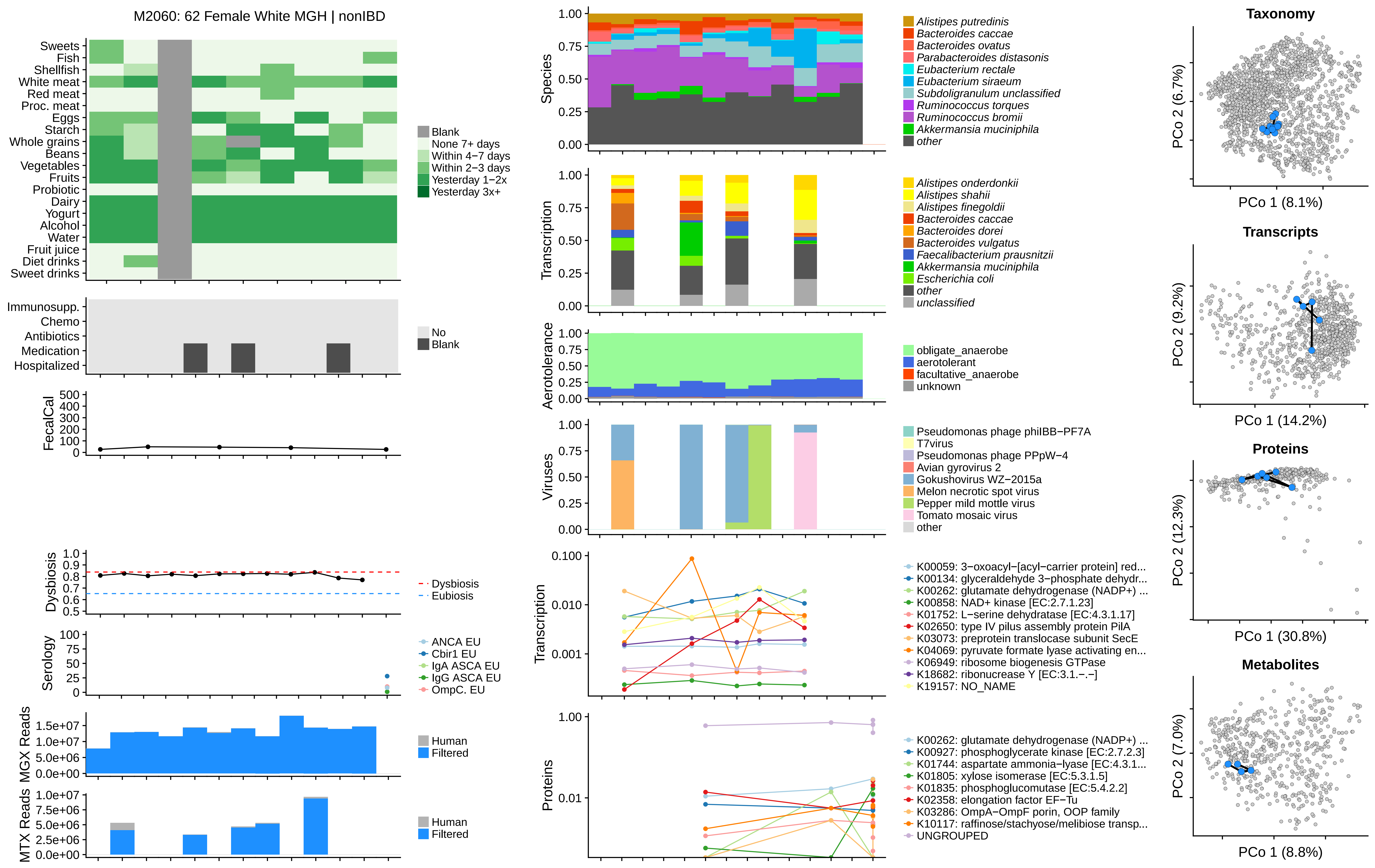

M2060: 62 Female White MGH | nonIBD
Sweets
Fish
Shellfish
White meat
Red meat
Proc. meat
Eggs
Starch
Whole grains
Beans
Vegetables
Fruits
Probiotic
Dairy
Yogurt
Alcohol
Water
Fruit juice
Diet drinks
Sweet drinks
Blank
None 7+ days
Within 4−7 days
Within 2−3 days
Yesterday 1−2x
Yesterday 3x+
Immunosupp.
Chemo
Antibiotics
Medication
Hospitalized
No
Blank
FecalCal
Dysbiosis
Dysbiosis
Eubiosis
Serology
ANCA EU
Cbir1 EU
IgA ASCA EU
IgG ASCA EU
OmpC. EU
MGX Reads
Human
Filtered
MTX Reads
Human
Filtered
Species
Alistipes putredinis
Bacteroides caccae
Bacteroides ovatus
Parabacteroides distasonis
Eubacterium rectale
Eubacterium siraeum
Subdoligranulum unclassified
Ruminococcus torques
Ruminococcus bromii
Akkermansia muciniphila
other
Transcription
Alistipes onderdonkii
Alistipes shahii
Alistipes finegoldii
Bacteroides caccae
Bacteroides dorei
Bacteroides vulgatus
Faecalibacterium prausnitzii
Akkermansia muciniphila
Escherichia coli
other
unclassified
Aerotolerance
obligate_anaerobe
aerotolerant
facultative_anaerobe
unknown
Viruses
Pseudomonas phage philBB−PF7A
T7virus
Pseudomonas phage PPpW−4
Avian gyrovirus 2
Gokushovirus WZ−2015a
Melon necrotic spot virus
Pepper mild mottle virus
Tomato mosaic virus
other
Transcription
K00059: 3−oxoacyl−[acyl−carrier protein] red...
K00134: glyceraldehyde 3−phosphate dehydr...
K00262: glutamate dehydrogenase (NADP+) ...
K00858: NAD+ kinase [EC:2.7.1.23]
K01752: L−serine dehydratase [EC:4.3.1.17]
K02650: type IV pilus assembly protein PilA
K03073: preprotein translocase subunit SecE
K04069: pyruvate formate lyase activating en...
K06949: ribosome biogenesis GTPase
K18682: ribonucrease Y [EC:3.1.−.−]
K19157: NO_NAME
Proteins
K00262: glutamate dehydrogenase (NADP+) ...
K00927: phosphoglycerate kinase [EC:2.7.2.3]
K01744: aspartate ammonia−lyase [EC:4.3.1...
K01805: xylose isomerase [EC:5.3.1.5]
K01835: phosphoglucomutase [EC:5.4.2.2]
K02358: elongation factor EF−Tu
K03286: OmpA−OmpF porin, OOP family
K10117: raffinose/stachyose/melibiose transp...
UNGROUPED
Taxonomy
PCo 1 (8.1%)
PCo 2 (6.7%)
Transcripts
PCo 1 (14.2%)
PCo 2 (9.2%)
Proteins
PCo 1 (30.8%)
PCo 2 (12.3%)
Metabolites
PCo 1 (8.8%)
PCo 2 (7.0%)

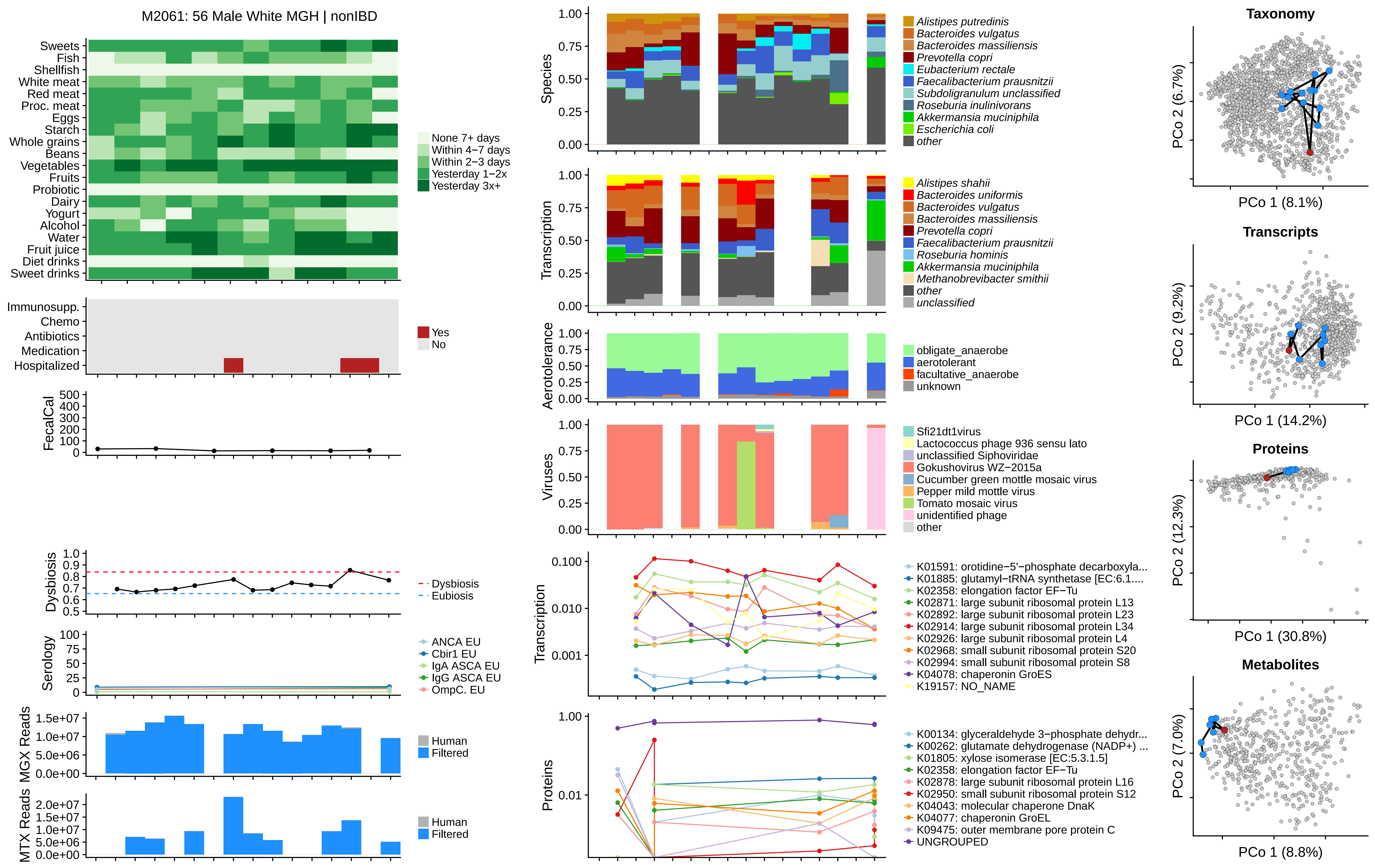
M2061: 56 Male White MGH | nonIBD
Sweets
Fish
Shellfish
White meat
Red meat
Proc. meat
Eggs
Starch
Whole grains
Beans
Vegetables
Fruits
Probiotic
Dairy
Yogurt
Alcohol
Water
Fruit juice
Diet drinks
Sweet drinks
None 7+ days
Within 4−7 days
Within 2−3 days
Yesterday 1−2x
Yesterday 3x+
Immunosupp.
Chemo
Antibiotics
Medication
Hospitalized
Yes
No
FecalCal
Dysbiosis
Dysbiosis
Eubiosis
Serology
ANCA EU
Cbir1 EU
IgA ASCA EU
IgG ASCA EU
OmpC. EU
MGX Reads
Human
Filtered
MTX Reads
Human
Filtered
Species
Alistipes putredinis
Bacteroides vulgatus
Bacteroides massiliensis
Prevotella copri
Eubacterium rectale
Faecalibacterium prausnitzii
Subdoligranulum unclassified
Roseburia inulinivorans
Akkermansia muciniphila
Escherichia coli
other
Transcription
Alistipes shahii
Bacteroides uniformis
Bacteroides vulgatus
Bacteroides massiliensis
Prevotella copri
Faecalibacterium prausnitzii
Roseburia hominis
Akkermansia muciniphila
Methanobrevibacter smithii
other
unclassified
Aerotolerance
obligate_anaerobe
aerotolerant
facultative_anaerobe
unknown
Viruses
Sfi21dt1virus
Lactococcus phage 936 sensu lato
unclassified Siphoviridae
Gokushovirus WZ−2015a
Cucumber green mottle mosaic virus
Pepper mild mottle virus
Tomato mosaic virus
unidentified phage
other
Transcription
K01591: orotidine−5'−phosphate decarboxyla...
K01885: glutamyl−tRNA synthetase [EC:6.1....
K02358: elongation factor EF−Tu
K02871: large subunit ribosomal protein L13
K02892: large subunit ribosomal protein L23
K02914: large subunit ribosomal protein L34
K02926: large subunit ribosomal protein L4
K02968: small subunit ribosomal protein S20
K02994: small subunit ribosomal protein S8
K04078: chaperonin GroES
K19157: NO_NAME
Proteins
K00134: glyceraldehyde 3−phosphate dehydr...
K00262: glutamate dehydrogenase (NADP+) ...
K01805: xylose isomerase [EC:5.3.1.5]
K02358: elongation factor EF−Tu
K02878: large subunit ribosomal protein L16
K02950: small subunit ribosomal protein S12
K04043: molecular chaperone DnaK
K04077: chaperonin GroEL
K09475: outer membrane pore protein C
UNGROUPED
Taxonomy
PCo 1 (8.1%)
PCo 2 (6.7%)
Transcripts
PCo 1 (14.2%)
PCo 2 (9.2%)
Proteins
PCo 1 (30.8%)
PCo 2 (12.3%)
Metabolites
PCo 1 (8.8%)
PCo 2 (7.0%)

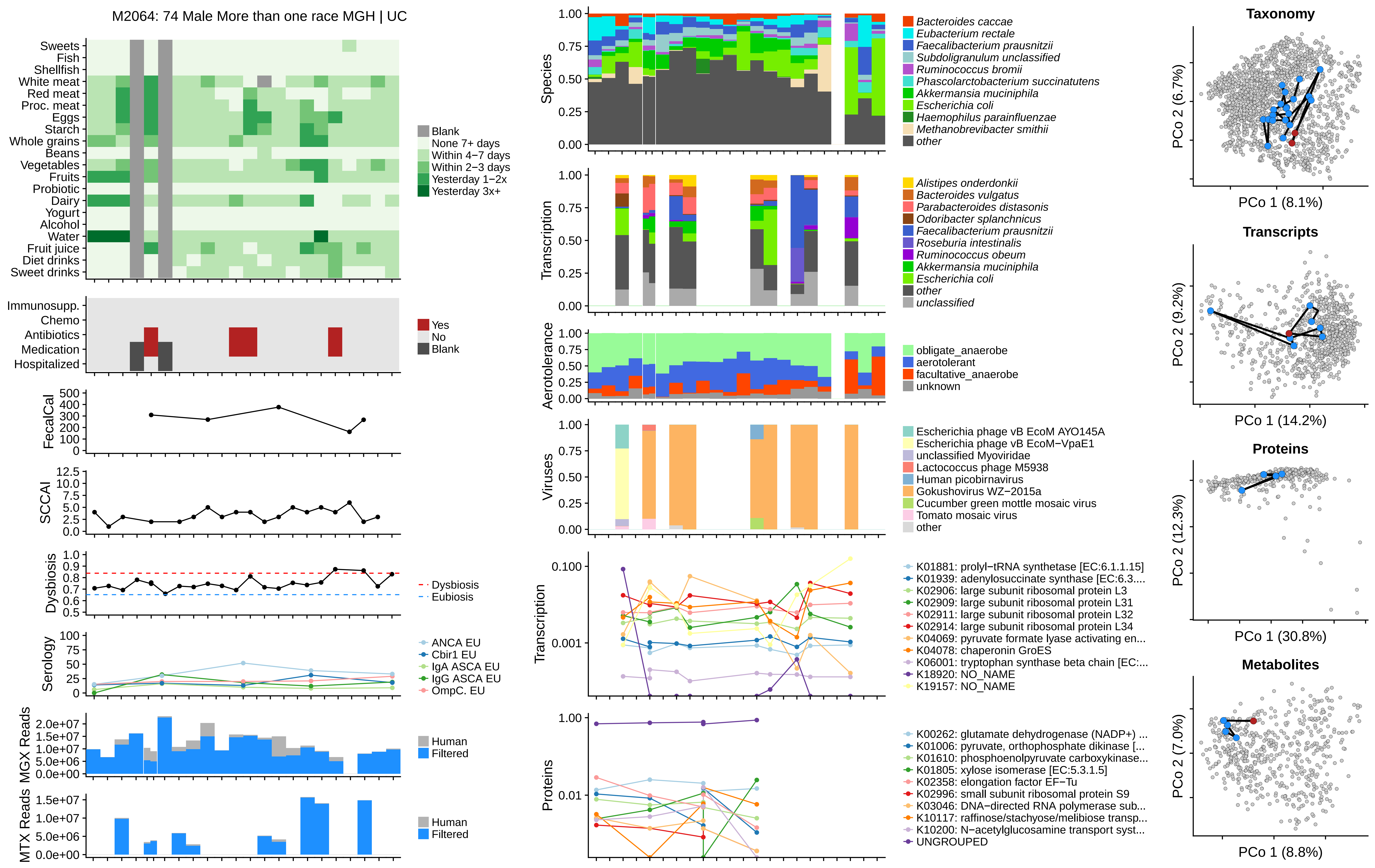
M2064: 74 Male More than one race MGH | UC
Sweets
Fish
Shellfish
White meat
Red meat
Proc. meat
Eggs
Starch
Whole grains
Beans
Vegetables
Fruits
Probiotic
Dairy
Yogurt
Alcohol
Water
Fruit juice
Diet drinks
Sweet drinks
Blank
None 7+ days
Within 4−7 days
Within 2−3 days
Yesterday 1−2x
Yesterday 3x+
Immunosupp.
Chemo
Antibiotics
Medication
Hospitalized
Yes
No
Blank
FecalCal
SCCAI
Dysbiosis
Dysbiosis
Eubiosis
Serology
ANCA EU
Cbir1 EU
IgA ASCA EU
IgG ASCA EU
OmpC. EU
MGX Reads
Human
Filtered
MTX Reads
Human
Filtered
Species
Bacteroides caccae
Eubacterium rectale
Faecalibacterium prausnitzii
Subdoligranulum unclassified
Ruminococcus bromii
Phascolarctobacterium succinatutens
Akkermansia muciniphila
Escherichia coli
Haemophilus parainfluenzae
Methanobrevibacter smithii
other
Transcription
Alistipes onderdonkii
Bacteroides vulgatus
Parabacteroides distasonis
Odoribacter splanchnicus
Faecalibacterium prausnitzii
Roseburia intestinalis
Ruminococcus obeum
Akkermansia muciniphila
Escherichia coli
other
unclassified
Aerotolerance
obligate_anaerobe
aerotolerant
facultative_anaerobe
unknown
Viruses
Escherichia phage vB EcoM AYO145A
Escherichia phage vB EcoM−VpaE1
unclassified Myoviridae
Lactococcus phage M5938
Human picobirnavirus
Gokushovirus WZ−2015a
Cucumber green mottle mosaic virus
Tomato mosaic virus
other
Transcription
K01881: prolyl−tRNA synthetase [EC:6.1.1.15]
K01939: adenylosuccinate synthase [EC:6.3....
K02906: large subunit ribosomal protein L3
K02909: large subunit ribosomal protein L31
K02911: large subunit ribosomal protein L32
K02914: large subunit ribosomal protein L34
K04069: pyruvate formate lyase activating en...
K04078: chaperonin GroES
K06001: tryptophan synthase beta chain [EC:...
K18920: NO_NAME
K19157: NO_NAME
Proteins
K00262: glutamate dehydrogenase (NADP+) ...
K01006: pyruvate, orthophosphate dikinase [...
K01610: phosphoenolpyruvate carboxykinase...
K01805: xylose isomerase [EC:5.3.1.5]
K02358: elongation factor EF−Tu
K02996: small subunit ribosomal protein S9
K03046: DNA−directed RNA polymerase sub...
K10117: raffinose/stachyose/melibiose transp...
K10200: N−acetylglucosamine transport syst...
UNGROUPED
Taxonomy
PCo 1 (8.1%)
PCo 2 (6.7%)
Transcripts
PCo 1 (14.2%)
PCo 2 (9.2%)
Proteins
PCo 1 (30.8%)
PCo 2 (12.3%)
Metabolites
PCo 1 (8.8%)
PCo 2 (7.0%)

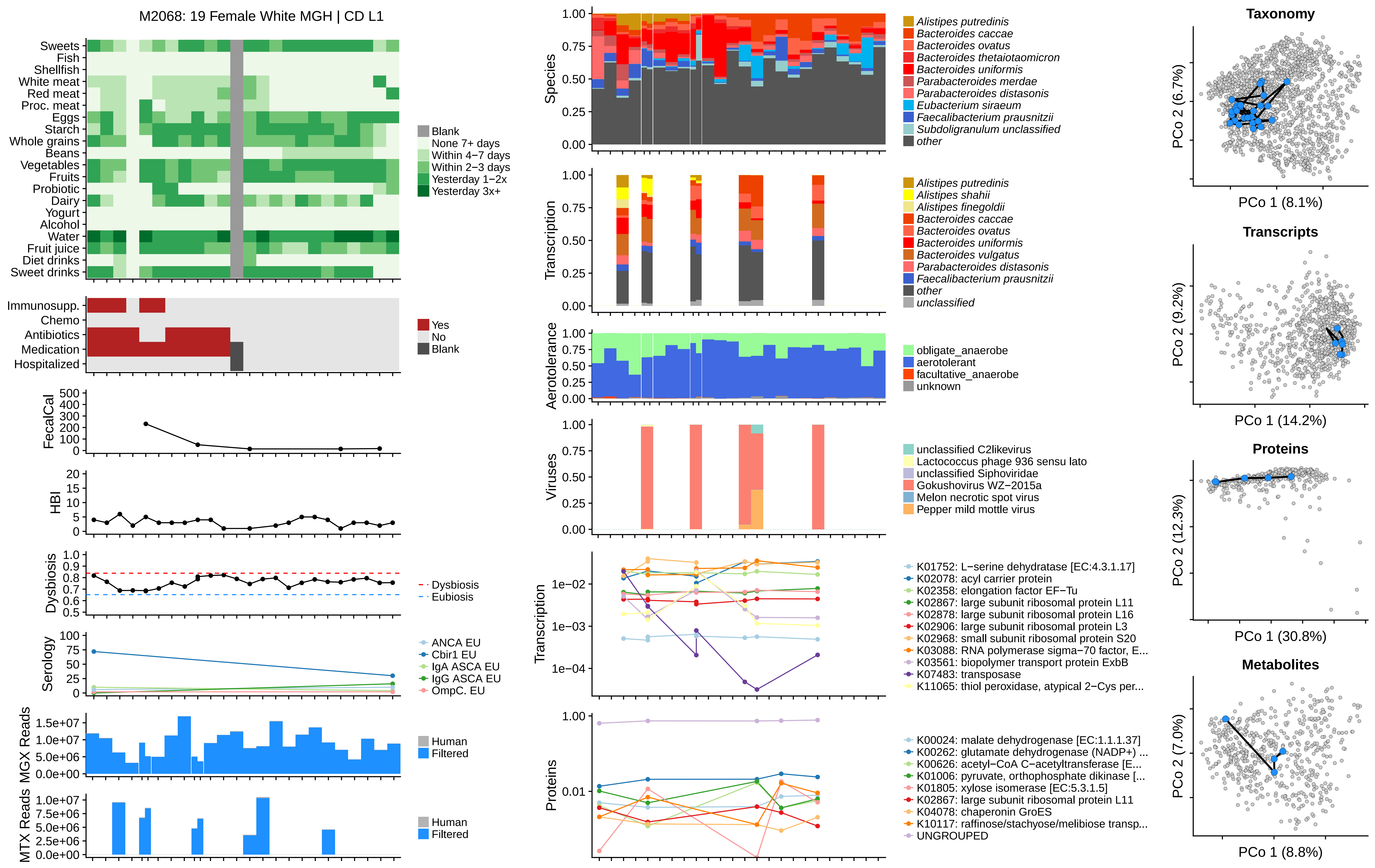
M2068: 19 Female White MGH | CD L1
Sweets
Fish
Shellfish
White meat
Red meat
Proc. meat
Eggs
Starch
Whole grains
Beans
Vegetables
Fruits
Probiotic
Dairy
Yogurt
Alcohol
Water
Fruit juice
Diet drinks
Sweet drinks
Blank
None 7+ days
Within 4−7 days
Within 2−3 days
Yesterday 1−2x
Yesterday 3x+
Immunosupp.
Chemo
Antibiotics
Medication
Hospitalized
Yes
No
Blank
FecalCal
HBI
Dysbiosis
Dysbiosis
Eubiosis
Serology
ANCA EU
Cbir1 EU
IgA ASCA EU
IgG ASCA EU
OmpC. EU
MGX Reads
Human
Filtered
MTX Reads
Human
Filtered
Species
Alistipes putredinis
Bacteroides caccae
Bacteroides ovatus
Bacteroides thetaiotaomicron
Bacteroides uniformis
Parabacteroides merdae
Parabacteroides distasonis
Eubacterium siraeum
Faecalibacterium prausnitzii
Subdoligranulum unclassified
other
Transcription
Alistipes putredinis
Alistipes shahii
Alistipes finegoldii
Bacteroides caccae
Bacteroides ovatus
Bacteroides uniformis
Bacteroides vulgatus
Parabacteroides distasonis
Faecalibacterium prausnitzii
other
unclassified
Aerotolerance
obligate_anaerobe
aerotolerant
facultative_anaerobe
unknown
Viruses
unclassified C2likevirus
Lactococcus phage 936 sensu lato
unclassified Siphoviridae
Gokushovirus WZ−2015a
Melon necrotic spot virus
Pepper mild mottle virus
Transcription
K01752: L−serine dehydratase [EC:4.3.1.17]
K02078: acyl carrier protein
K02358: elongation factor EF−Tu
K02867: large subunit ribosomal protein L11
K02878: large subunit ribosomal protein L16
K02906: large subunit ribosomal protein L3
K02968: small subunit ribosomal protein S20
K03088: RNA polymerase sigma−70 factor, E...
K03561: biopolymer transport protein ExbB
K07483: transposase
K11065: thiol peroxidase, atypical 2−Cys per...
Proteins
K00024: malate dehydrogenase [EC:1.1.1.37]
K00262: glutamate dehydrogenase (NADP+) ...
K00626: acetyl−CoA C−acetyltransferase [E...
K01006: pyruvate, orthophosphate dikinase [...
K01805: xylose isomerase [EC:5.3.1.5]
K02867: large subunit ribosomal protein L11
K04078: chaperonin GroES
K10117: raffinose/stachyose/melibiose transp...
UNGROUPED
Taxonomy
PCo 1 (8.1%)
PCo 2 (6.7%)
Transcripts
PCo 1 (14.2%)
PCo 2 (9.2%)
Proteins
PCo 1 (30.8%)
PCo 2 (12.3%)
Metabolites
PCo 1 (8.8%)
PCo 2 (7.0%)

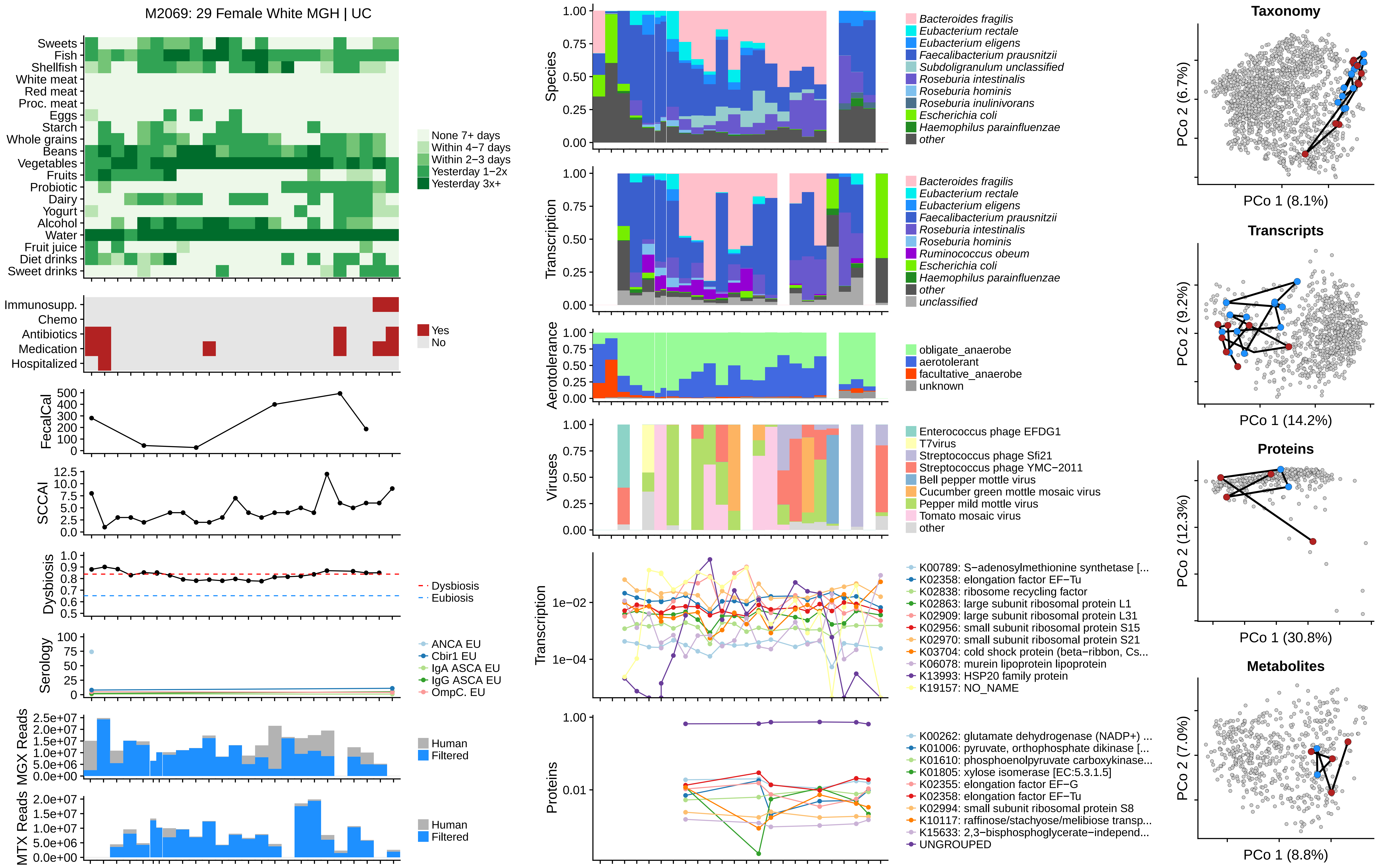
M2069: 29 Female White MGH | UC
Sweets
Fish
Shellfish
White meat
Red meat
Proc. meat
Eggs
Starch
Whole grains
Beans
Vegetables
Fruits
Probiotic
Dairy
Yogurt
Alcohol
Water
Fruit juice
Diet drinks
Sweet drinks
None 7+ days
Within 4−7 days
Within 2−3 days
Yesterday 1−2x
Yesterday 3x+
Immunosupp.
Chemo
Antibiotics
Medication
Hospitalized
Yes
No
FecalCal
SCCAI
Dysbiosis
Dysbiosis
Eubiosis
Serology
ANCA EU
Cbir1 EU
IgA ASCA EU
IgG ASCA EU
OmpC. EU
MGX Reads
Human
Filtered
MTX Reads
Human
Filtered
Species
Bacteroides fragilis
Eubacterium rectale
Eubacterium eligens
Faecalibacterium prausnitzii
Subdoligranulum unclassified
Roseburia intestinalis
Roseburia hominis
Roseburia inulinivorans
Escherichia coli
Haemophilus parainfluenzae
other
Transcription
Bacteroides fragilis
Eubacterium rectale
Eubacterium eligens
Faecalibacterium prausnitzii
Roseburia intestinalis
Roseburia hominis
Ruminococcus obeum
Escherichia coli
Haemophilus parainfluenzae
other
unclassified
Aerotolerance
obligate_anaerobe
aerotolerant
facultative_anaerobe
unknown
Viruses
Enterococcus phage EFDG1
T7virus
Streptococcus phage Sfi21
Streptococcus phage YMC−2011
Bell pepper mottle virus
Cucumber green mottle mosaic virus
Pepper mild mottle virus
Tomato mosaic virus
other
Transcription
K00789: S−adenosylmethionine synthetase [...
K02358: elongation factor EF−Tu
K02838: ribosome recycling factor
K02863: large subunit ribosomal protein L1
K02909: large subunit ribosomal protein L31
K02956: small subunit ribosomal protein S15
K02970: small subunit ribosomal protein S21
K03704: cold shock protein (beta−ribbon, Cs...
K06078: murein lipoprotein lipoprotein
K13993: HSP20 family protein
K19157: NO_NAME
Proteins
K00262: glutamate dehydrogenase (NADP+) ...
K01006: pyruvate, orthophosphate dikinase [...
K01610: phosphoenolpyruvate carboxykinase...
K01805: xylose isomerase [EC:5.3.1.5]
K02355: elongation factor EF−G
K02358: elongation factor EF−Tu
K02994: small subunit ribosomal protein S8
K10117: raffinose/stachyose/melibiose transp...
K15633: 2,3−bisphosphoglycerate−independ...
UNGROUPED
Taxonomy
PCo 1 (8.1%)
PCo 2 (6.7%)
Transcripts
PCo 1 (14.2%)
PCo 2 (9.2%)
Proteins
PCo 1 (30.8%)
PCo 2 (12.3%)
Metabolites
PCo 1 (8.8%)
PCo 2 (7.0%)

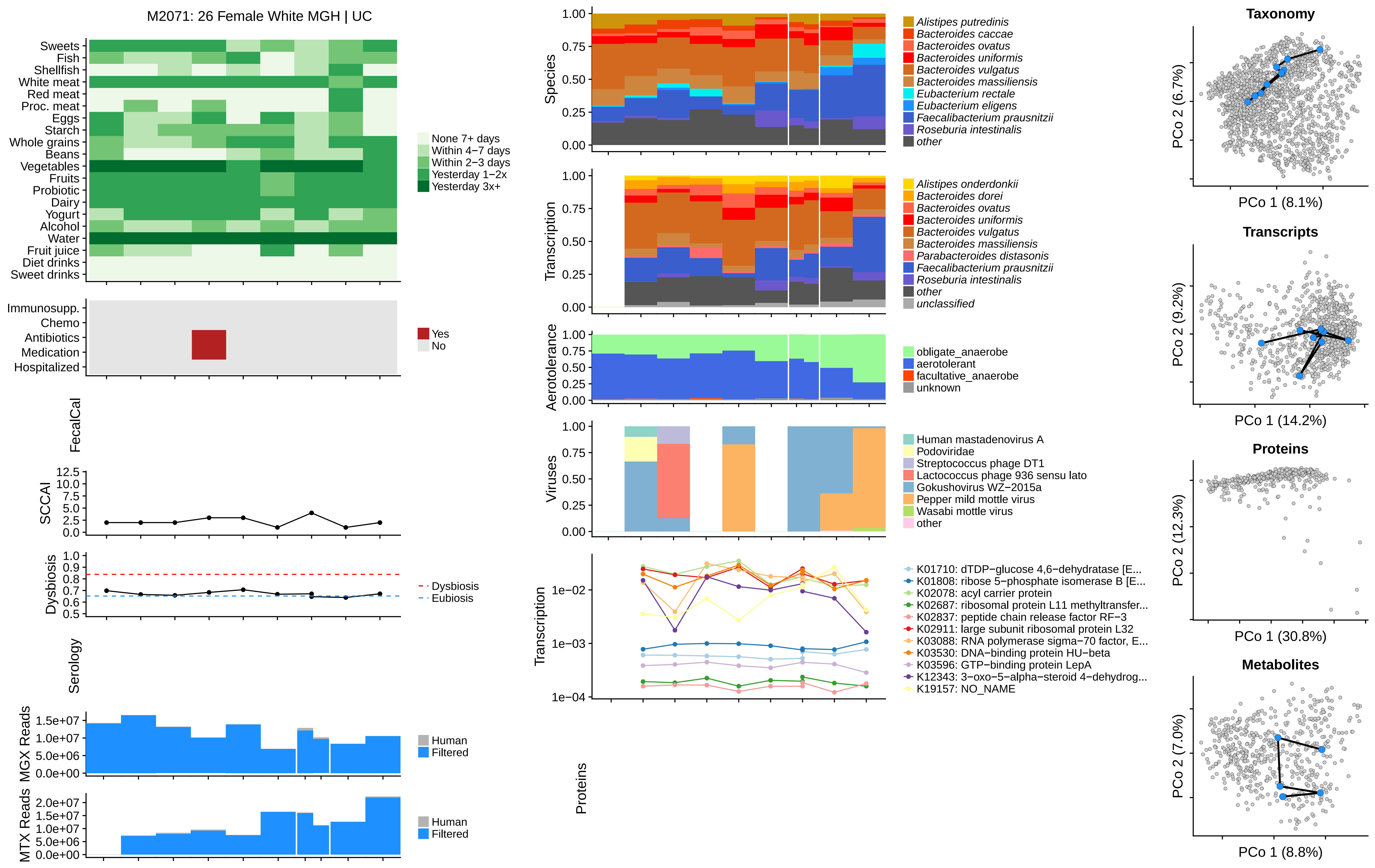

M2071: 26 Female White MGH | UC
Sweets
Fish
Shellfish
White meat
Red meat
Proc. meat
Eggs
Starch
Whole grains
Beans
Vegetables
Fruits
Probiotic
Dairy
Yogurt
Alcohol
Water
Fruit juice
Diet drinks
Sweet drinks
None 7+ days
Within 4−7 days
Within 2−3 days
Yesterday 1−2x
Yesterday 3x+
Immunosupp.
Chemo
Antibiotics
Medication
Hospitalized
Yes
No
FecalCal
SCCAI
Dysbiosis
Dysbiosis
Eubiosis
Serology
MGX Reads
Human
Filtered
MTX Reads
Human
Filtered
Species
Alistipes putredinis
Bacteroides caccae
Bacteroides ovatus
Bacteroides uniformis
Bacteroides vulgatus
Bacteroides massiliensis
Eubacterium rectale
Eubacterium eligens
Faecalibacterium prausnitzii
Roseburia intestinalis
other
Transcription
Alistipes onderdonkii
Bacteroides dorei
Bacteroides ovatus
Bacteroides uniformis
Bacteroides vulgatus
Bacteroides massiliensis
Parabacteroides distasonis
Faecalibacterium prausnitzii
Roseburia intestinalis
other
unclassified
Aerotolerance
obligate_anaerobe
aerotolerant
facultative_anaerobe
unknown
Viruses
Human mastadenovirus A
Podoviridae
Streptococcus phage DT1
Lactococcus phage 936 sensu lato
Gokushovirus WZ−2015a
Pepper mild mottle virus
Wasabi mottle virus
other
Transcription
K01710: dTDP−glucose 4,6−dehydratase [E...
K01808: ribose 5−phosphate isomerase B [E...
K02078: acyl carrier protein
K02687: ribosomal protein L11 methyltransfer...
K02837: peptide chain release factor RF−3
K02911: large subunit ribosomal protein L32
K03088: RNA polymerase sigma−70 factor, E...
K03530: DNA−binding protein HU−beta
K03596: GTP−binding protein LepA
K12343: 3−oxo−5−alpha−steroid 4−dehydrog...
K19157: NO_NAME
Proteins
Taxonomy
PCo 1 (8.1%)
PCo 2 (6.7%)
Transcripts
PCo 1 (14.2%)
PCo 2 (9.2%)
Proteins
PCo 1 (30.8%)
PCo 2 (12.3%)
Metabolites
PCo 1 (8.8%)
PCo 2 (7.0%)

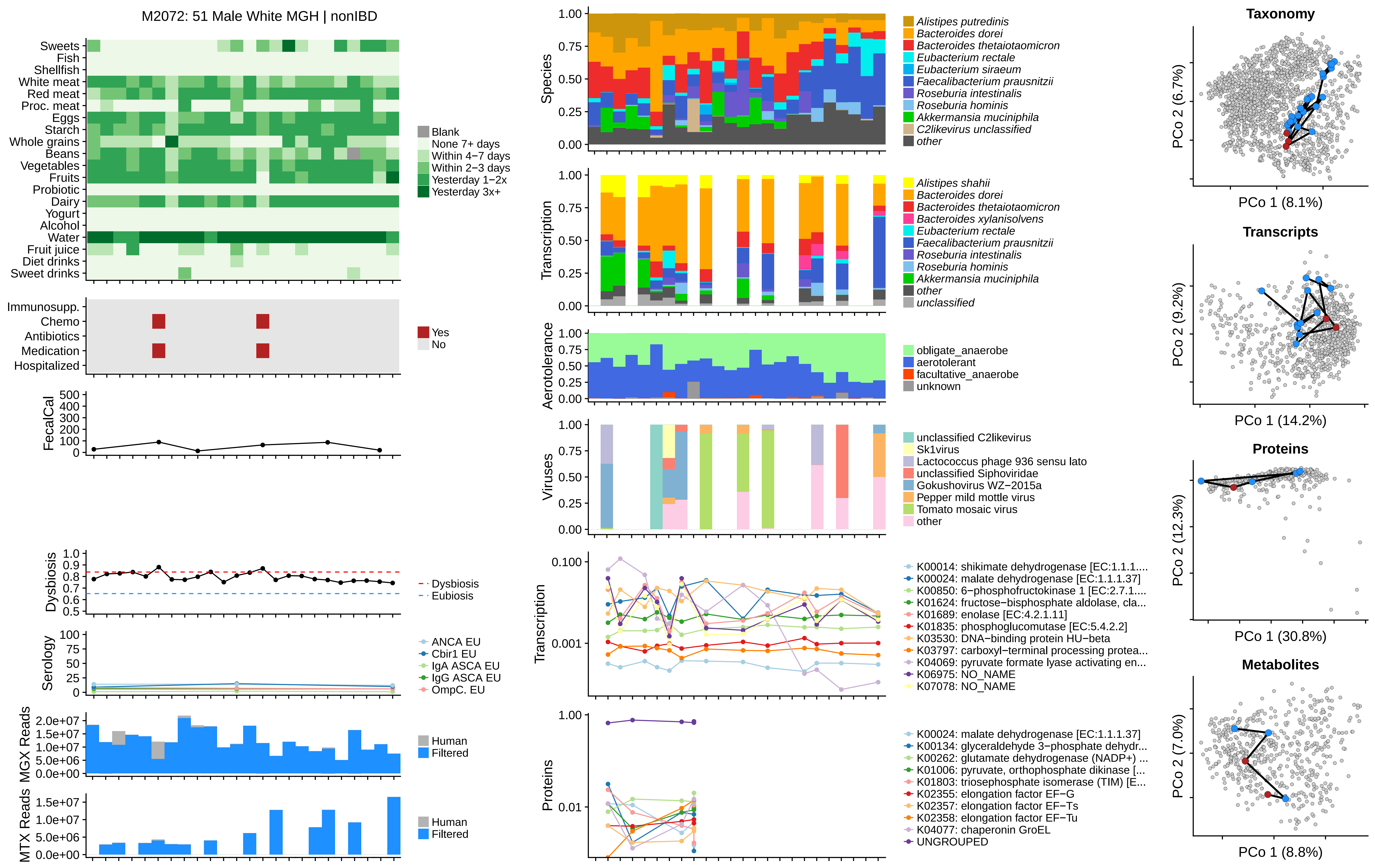

M2072: 51 Male White MGH | nonIBD
Sweets
Fish
Shellfish
White meat
Red meat
Proc. meat
Eggs
Starch
Whole grains
Beans
Vegetables
Fruits
Probiotic
Dairy
Yogurt
Alcohol
Water
Fruit juice
Diet drinks
Sweet drinks
Blank
None 7+ days
Within 4−7 days
Within 2−3 days
Yesterday 1−2x
Yesterday 3x+
Immunosupp.
Chemo
Antibiotics
Medication
Hospitalized
Yes
No
FecalCal
Dysbiosis
Dysbiosis
Eubiosis
Serology
ANCA EU
Cbir1 EU
IgA ASCA EU
IgG ASCA EU
OmpC. EU
MGX Reads
Human
Filtered
MTX Reads
Human
Filtered
Species
Alistipes putredinis
Bacteroides dorei
Bacteroides thetaiotaomicron
Eubacterium rectale
Eubacterium siraeum
Faecalibacterium prausnitzii
Roseburia intestinalis
Roseburia hominis
Akkermansia muciniphila
C2likevirus unclassified
other
Transcription
Alistipes shahii
Bacteroides dorei
Bacteroides thetaiotaomicron
Bacteroides xylanisolvens
Eubacterium rectale
Faecalibacterium prausnitzii
Roseburia intestinalis
Roseburia hominis
Akkermansia muciniphila
other
unclassified
Aerotolerance
obligate_anaerobe
aerotolerant
facultative_anaerobe
unknown
Viruses
unclassified C2likevirus
Sk1virus
Lactococcus phage 936 sensu lato
unclassified Siphoviridae
Gokushovirus WZ−2015a
Pepper mild mottle virus
Tomato mosaic virus
other
Transcription
K00014: shikimate dehydrogenase [EC:1.1.1....
K00024: malate dehydrogenase [EC:1.1.1.37]
K00850: 6−phosphofructokinase 1 [EC:2.7.1....
K01624: fructose−bisphosphate aldolase, cla...
K01689: enolase [EC:4.2.1.11]
K01835: phosphoglucomutase [EC:5.4.2.2]
K03530: DNA−binding protein HU−beta
K03797: carboxyl−terminal processing protea...
K04069: pyruvate formate lyase activating en...
K06975: NO_NAME
K07078: NO_NAME
Proteins
K00024: malate dehydrogenase [EC:1.1.1.37]
K00134: glyceraldehyde 3−phosphate dehydr...
K00262: glutamate dehydrogenase (NADP+) ...
K01006: pyruvate, orthophosphate dikinase [...
K01803: triosephosphate isomerase (TIM) [E...
K02355: elongation factor EF−G
K02357: elongation factor EF−Ts
K02358: elongation factor EF−Tu
K04077: chaperonin GroEL
UNGROUPED
Taxonomy
PCo 1 (8.1%)
PCo 2 (6.7%)
Transcripts
PCo 1 (14.2%)
PCo 2 (9.2%)
Proteins
PCo 1 (30.8%)
PCo 2 (12.3%)
Metabolites
PCo 1 (8.8%)
PCo 2 (7.0%)

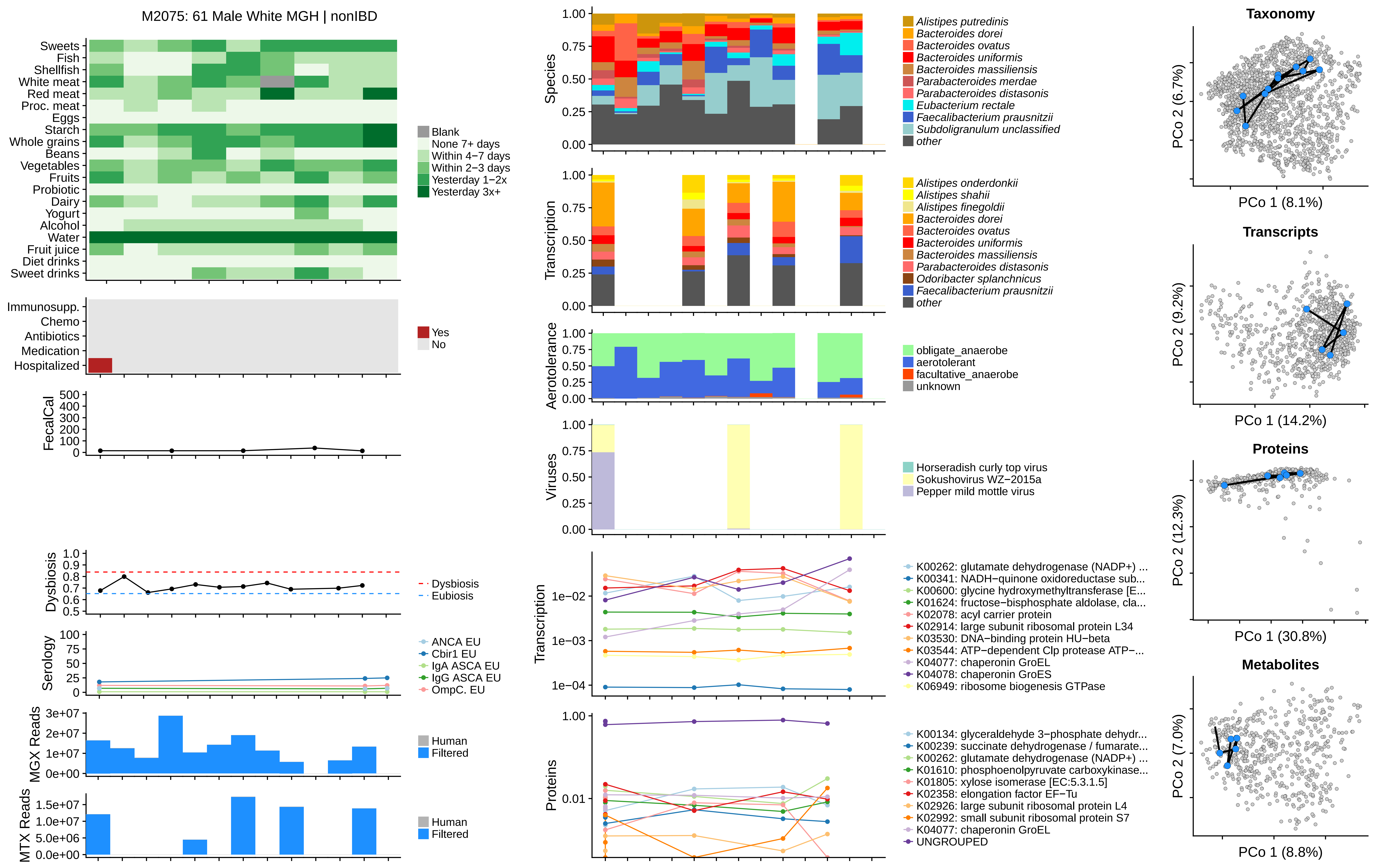
M2075: 61 Male White MGH | nonIBD
Sweets
Fish
Shellfish
White meat
Red meat
Proc. meat
Eggs
Starch
Whole grains
Beans
Vegetables
Fruits
Probiotic
Dairy
Yogurt
Alcohol
Water
Fruit juice
Diet drinks
Sweet drinks
Blank
None 7+ days
Within 4−7 days
Within 2−3 days
Yesterday 1−2x
Yesterday 3x+
Immunosupp.
Chemo
Antibiotics
Medication
Hospitalized
Yes
No
FecalCal
Dysbiosis
Eubiosis
Serology
ANCA EU
Cbir1 EU
IgA ASCA EU
IgG ASCA EU
OmpC. EU
MGX Reads
MTX Reads
Human
Filtered
Species
Alistipes putredinis
Bacteroides dorei
Bacteroides ovatus
Bacteroides uniformis
Bacteroides massiliensis
Parabacteroides merdae
Parabacteroides distasonis
Eubacterium rectale
Faecalibacterium prausnitzii
Subdoligranulum unclassified
other
Transcription
Alistipes onderdonkii
Alistipes shahii
Alistipes finegoldii
Odoribacter splanchnicus
Aerotolerance
obligate_anaerobe
aerotolerant
facultative_anaerobe
unknown
Viruses
Horseradish curly top virus
Gokushovirus WZ−2015a
Pepper mild mottle virus
K00262: glutamate dehydrogenase (NADP+) ...
K00341: NADH−quinone oxidoreductase sub...
K00600: glycine hydroxymethyltransferase [E...
K01624: fructose−bisphosphate aldolase, cla...
K02078: acyl carrier protein
K02914: large subunit ribosomal protein L34
K03530: DNA−binding protein HU−beta
K03544: ATP−dependent Clp protease ATP−...
K04077: chaperonin GroEL
K04078: chaperonin GroES
K06949: ribosome biogenesis GTPase
Proteins
K00134: glyceraldehyde 3−phosphate dehydr...
K00239: succinate dehydrogenase / fumarate...
K01610: phosphoenolpyruvate carboxykinase...
K01805: xylose isomerase [EC:5.3.1.5]
K02358: elongation factor EF−Tu
K02926: large subunit ribosomal protein L4
K02992: small subunit ribosomal protein S7
UNGROUPED
Taxonomy
PCo 1 (8.1%)
PCo 2 (6.7%)
Transcripts
PCo 1 (14.2%)
PCo 2 (9.2%)
Proteins
PCo 1 (30.8%)
PCo 2 (12.3%)
Metabolites
PCo 1 (8.8%)
PCo 2 (7.0%)

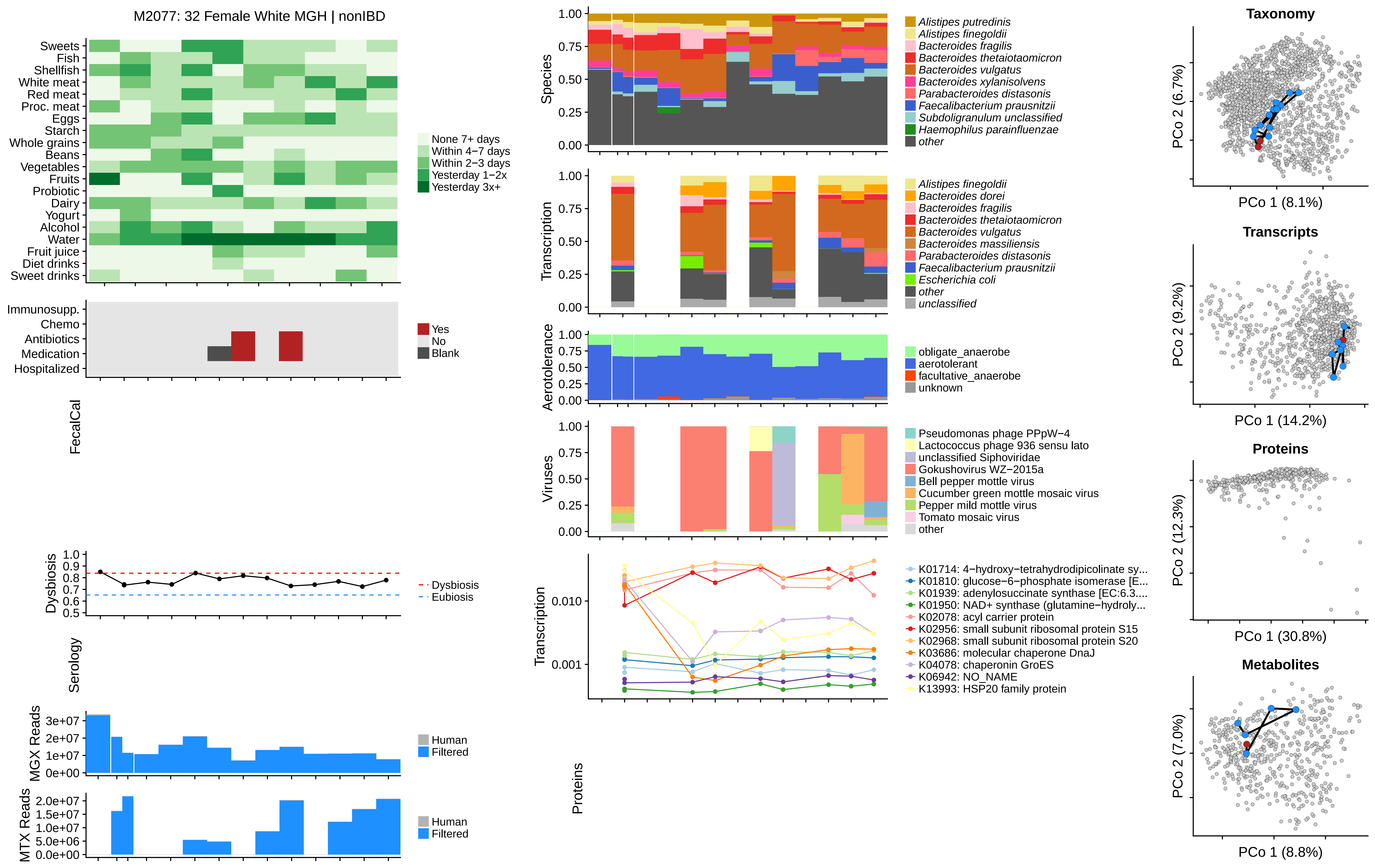
M2077: 32 Female White MGH | nonIBD
Sweets
Fish
Shellfish
White meat
Red meat
Proc. meat
Eggs
Starch
Whole grains
Beans
Vegetables
Fruits
Probiotic
Dairy
Yogurt
Alcohol
Water
Fruit juice
Diet drinks
Sweet drinks
None 7+ days
Within 4−7 days
Within 2−3 days
Yesterday 1−2x
Yesterday 3x+
Immunosupp.
Chemo
Antibiotics
Medication
Hospitalized
Yes
No
Blank
FecalCal
Dysbiosis
Dysbiosis
Eubiosis
Serology
MGX Reads
Human
Filtered
MTX Reads
Human
Filtered
Species
Alistipes putredinis
Alistipes finegoldii
Bacteroides fragilis
Bacteroides thetaiotaomicron
Bacteroides vulgatus
Bacteroides xylanisolvens
Parabacteroides distasonis
Faecalibacterium prausnitzii
Subdoligranulum unclassified
Haemophilus parainfluenzae
other
Transcription
Alistipes finegoldii
Bacteroides dorei
Bacteroides fragilis
Bacteroides thetaiotaomicron
Bacteroides vulgatus
Bacteroides massiliensis
Parabacteroides distasonis
Faecalibacterium prausnitzii
Escherichia coli
other
unclassified
Aerotolerance
obligate_anaerobe
aerotolerant
facultative_anaerobe
unknown
Viruses
Pseudomonas phage PPpW−4
Lactococcus phage 936 sensu lato
unclassified Siphoviridae
Gokushovirus WZ−2015a
Bell pepper mottle virus
Cucumber green mottle mosaic virus
Pepper mild mottle virus
Tomato mosaic virus
other
Transcription
K01714: 4−hydroxy−tetrahydrodipicolinate sy...
K01810: glucose−6−phosphate isomerase [E...
K01939: adenylosuccinate synthase [EC:6.3....
K01950: NAD+ synthase (glutamine−hydroly...
K02078: acyl carrier protein
K02956: small subunit ribosomal protein S15
K02968: small subunit ribosomal protein S20
K03686: molecular chaperone DnaJ
K04078: chaperonin GroES
K06942: NO_NAME
K13993: HSP20 family protein
Proteins
Taxonomy
PCo 1 (8.1%)
PCo 2 (6.7%)
Transcripts
PCo 1 (14.2%)
PCo 2 (9.2%)
Proteins
PCo 1 (30.8%)
PCo 2 (12.3%)
Metabolites
PCo 1 (8.8%)
PCo 2 (7.0%)

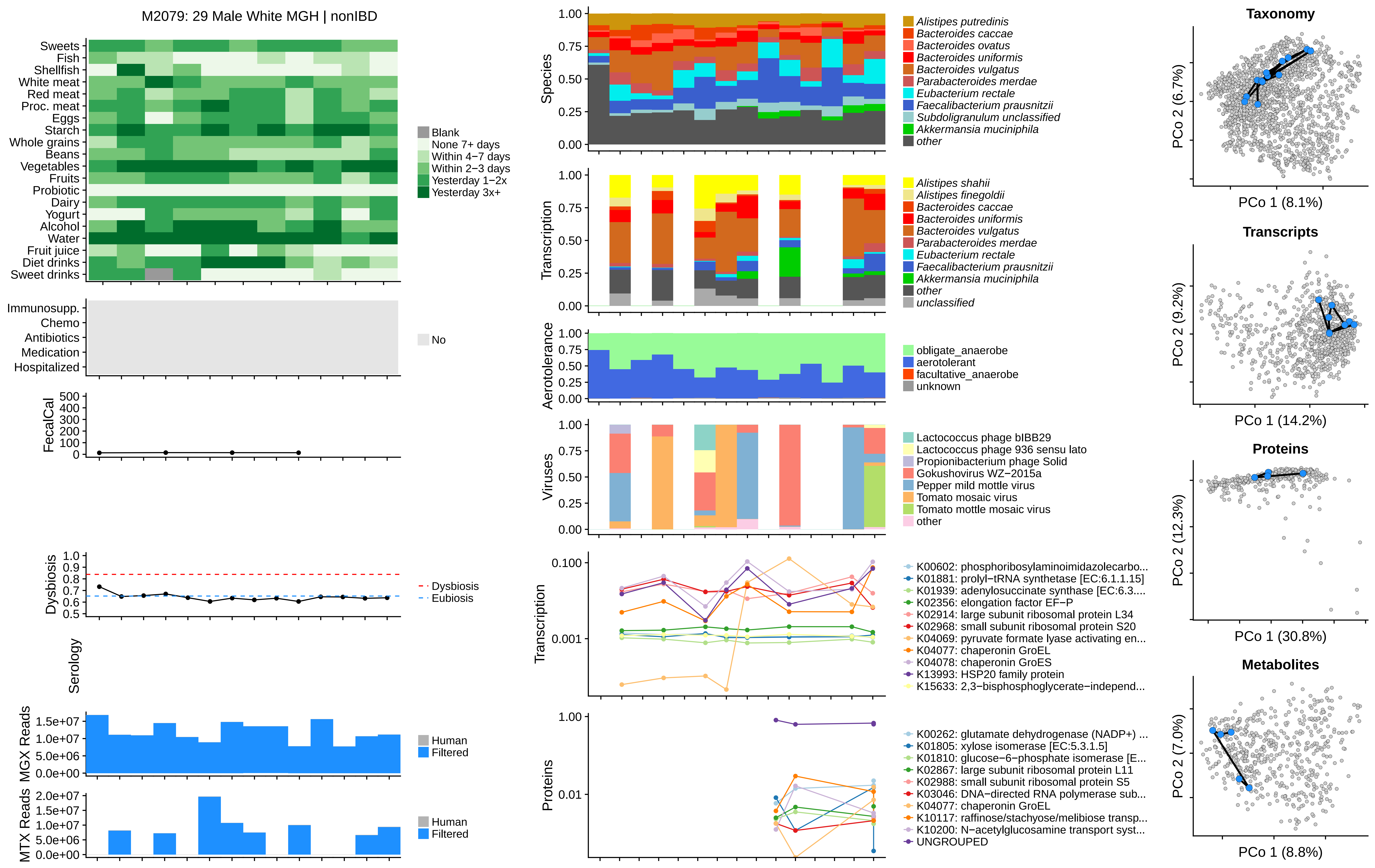
M2079: 29 Male White MGH | nonIBD
Sweets
Fish
Shellfish
White meat
Red meat
Proc. meat
Eggs
Starch
Whole grains
Beans
Vegetables
Fruits
Probiotic
Dairy
Yogurt
Alcohol
Water
Fruit juice
Diet drinks
Sweet drinks
Blank
None 7+ days
Within 4−7 days
Within 2−3 days
Yesterday 1−2x
Yesterday 3x+
Immunosupp.
Chemo
Antibiotics
Medication
Hospitalized
No
FecalCal
Dysbiosis
Dysbiosis
Eubiosis
Serology
MGX Reads
Human
Filtered
MTX Reads
Human
Filtered
Species
Alistipes putredinis
Bacteroides caccae
Bacteroides ovatus
Bacteroides uniformis
Bacteroides vulgatus
Parabacteroides merdae
Eubacterium rectale
Faecalibacterium prausnitzii
Subdoligranulum unclassified
Akkermansia muciniphila
other
Transcription
Alistipes shahii
Alistipes finegoldii
Bacteroides caccae
Bacteroides uniformis
Bacteroides vulgatus
Parabacteroides merdae
Eubacterium rectale
Faecalibacterium prausnitzii
Akkermansia muciniphila
other
unclassified
Aerotolerance
obligate_anaerobe
aerotolerant
facultative_anaerobe
unknown
Viruses
Lactococcus phage bIBB29
Lactococcus phage 936 sensu lato
Propionibacterium phage Solid
Gokushovirus WZ−2015a
Pepper mild mottle virus
Tomato mosaic virus
Tomato mottle mosaic virus
other
Transcription
K00602: phosphoribosylaminoimidazolecarbo...
K01881: prolyl−tRNA synthetase [EC:6.1.1.15]
K01939: adenylosuccinate synthase [EC:6.3....
K02356: elongation factor EF−P
K02914: large subunit ribosomal protein L34
K02968: small subunit ribosomal protein S20
K04069: pyruvate formate lyase activating en...
K04077: chaperonin GroEL
K04078: chaperonin GroES
K13993: HSP20 family protein
K15633: 2,3−bisphosphoglycerate−independ...
Proteins
K00262: glutamate dehydrogenase (NADP+) ...
K01805: xylose isomerase [EC:5.3.1.5]
K01810: glucose−6−phosphate isomerase [E...
K02867: large subunit ribosomal protein L11
K02988: small subunit ribosomal protein S5
K03046: DNA−directed RNA polymerase sub...
K04077: chaperonin GroEL
K10117: raffinose/stachyose/melibiose transp...
K10200: N−acetylglucosamine transport syst...
UNGROUPED
Taxonomy
PCo 1 (8.1%)
PCo 2 (6.7%)
Transcripts
PCo 1 (14.2%)
PCo 2 (9.2%)
Proteins
PCo 1 (30.8%)
PCo 2 (12.3%)
Metabolites
PCo 1 (8.8%)
PCo 2 (7.0%)

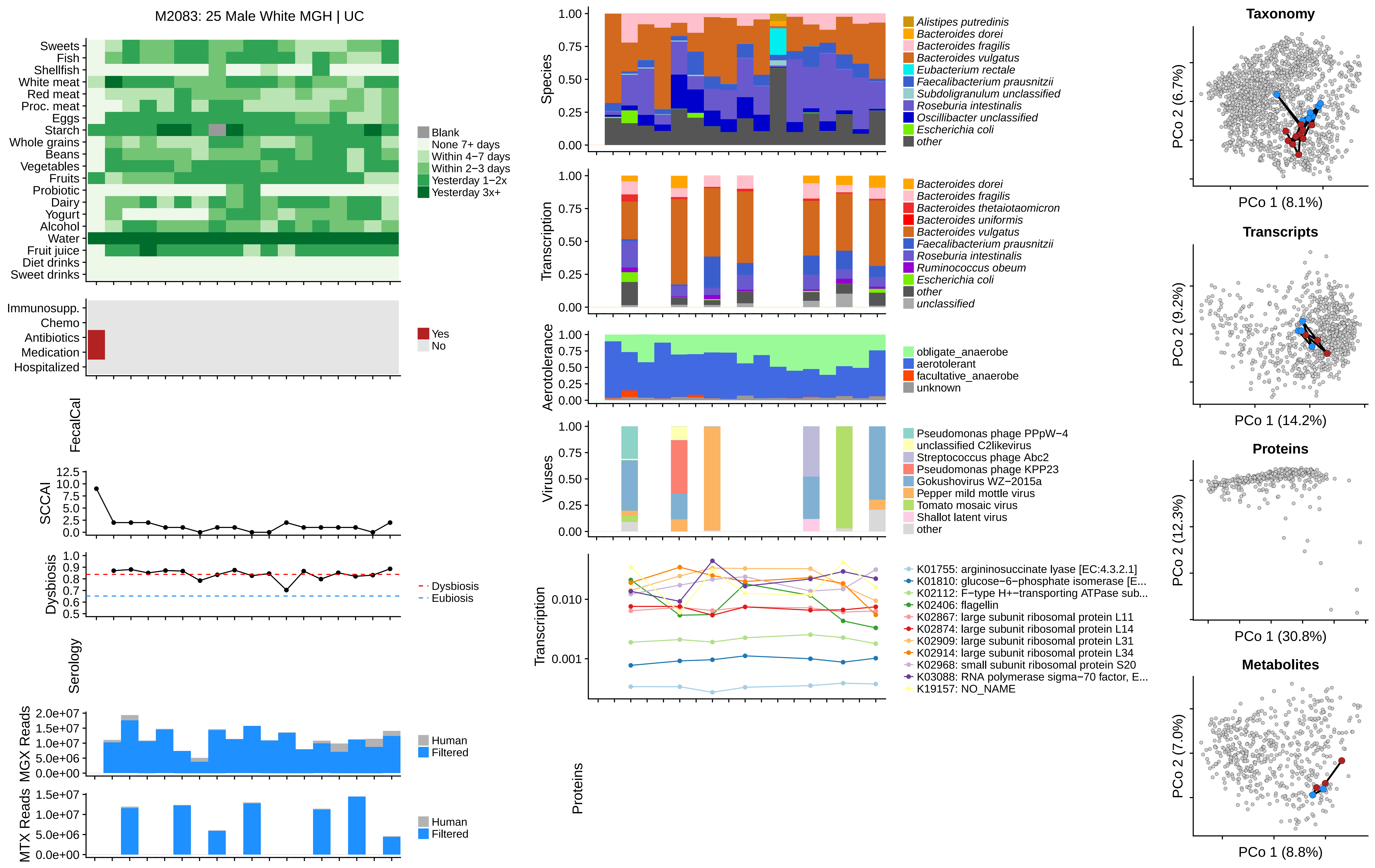
M2083: 25 Male White MGH | UC
Sweets
Fish
Shellfish
White meat
Red meat
Proc. meat
Eggs
Starch
Whole grains
Beans
Vegetables
Fruits
Probiotic
Dairy
Yogurt
Alcohol
Water
Fruit juice
Diet drinks
Sweet drinks
Blank
None 7+ days
Within 4−7 days
Within 2−3 days
Yesterday 1−2x
Yesterday 3x+
Immunosupp.
Chemo
Antibiotics
Medication
Hospitalized
Yes
No
FecalCal
SCCAI
12.5
10.0
7.5
5.0
2.5
0.0
Dysbiosis
1.0
0.9
0.8
0.7
0.6
0.5
Dysbiosis
Eubiosis
Serology
MGX Reads
2.0e+07
1.5e+07
1.0e+07
5.0e+06
0.0e+00
Human
Filtered
MTX Reads
1.5e+07
1.0e+07
5.0e+06
0.0e+00
Human
Filtered
Species
1.00
0.75
0.50
0.25
0.00
Alistipes putredinis
Bacteroides dorei
Bacteroides fragilis
Bacteroides vulgatus
Eubacterium rectale
Faecalibacterium prausnitzii
Subdoligranulum unclassified
Roseburia intestinalis
Oscillibacter unclassified
Escherichia coli
other
Transcription
Bacteroides dorei
Bacteroides fragilis
Bacteroides thetaiotaomicron
Bacteroides uniformis
Bacteroides vulgatus
Faecalibacterium prausnitzii
Roseburia intestinalis
Ruminococcus obeum
Escherichia coli
other
unclassified
Aerotolerance
obligate_anaerobe
aerotolerant
facultative_anaerobe
unknown
Viruses
Pseudomonas phage PPpW−4
unclassified C2likevirus
Streptococcus phage Abc2
Pseudomonas phage KPP23
Gokushovirus WZ−2015a
Pepper mild mottle virus
Tomato mosaic virus
Shallot latent virus
other
Transcription
0.010
0.001
K01755: argininosuccinate lyase [EC:4.3.2.1]
K01810: glucose−6−phosphate isomerase [E...
K02112: F−type H+−transporting ATPase sub...
K02406: flagellin
K02867: large subunit ribosomal protein L11
K02874: large subunit ribosomal protein L14
K02909: large subunit ribosomal protein L31
K02914: large subunit ribosomal protein L34
K02968: small subunit ribosomal protein S20
K03088: RNA polymerase sigma−70 factor, E...
K19157: NO_NAME
Proteins
Taxonomy
PCo 2 (6.7%)
PCo 1 (8.1%)
Transcripts
PCo 2 (9.2%)
PCo 1 (14.2%)
Proteins
PCo 2 (12.3%)
PCo 1 (30.8%)
Metabolites
PCo 2 (7.0%)
PCo 1 (8.8%)

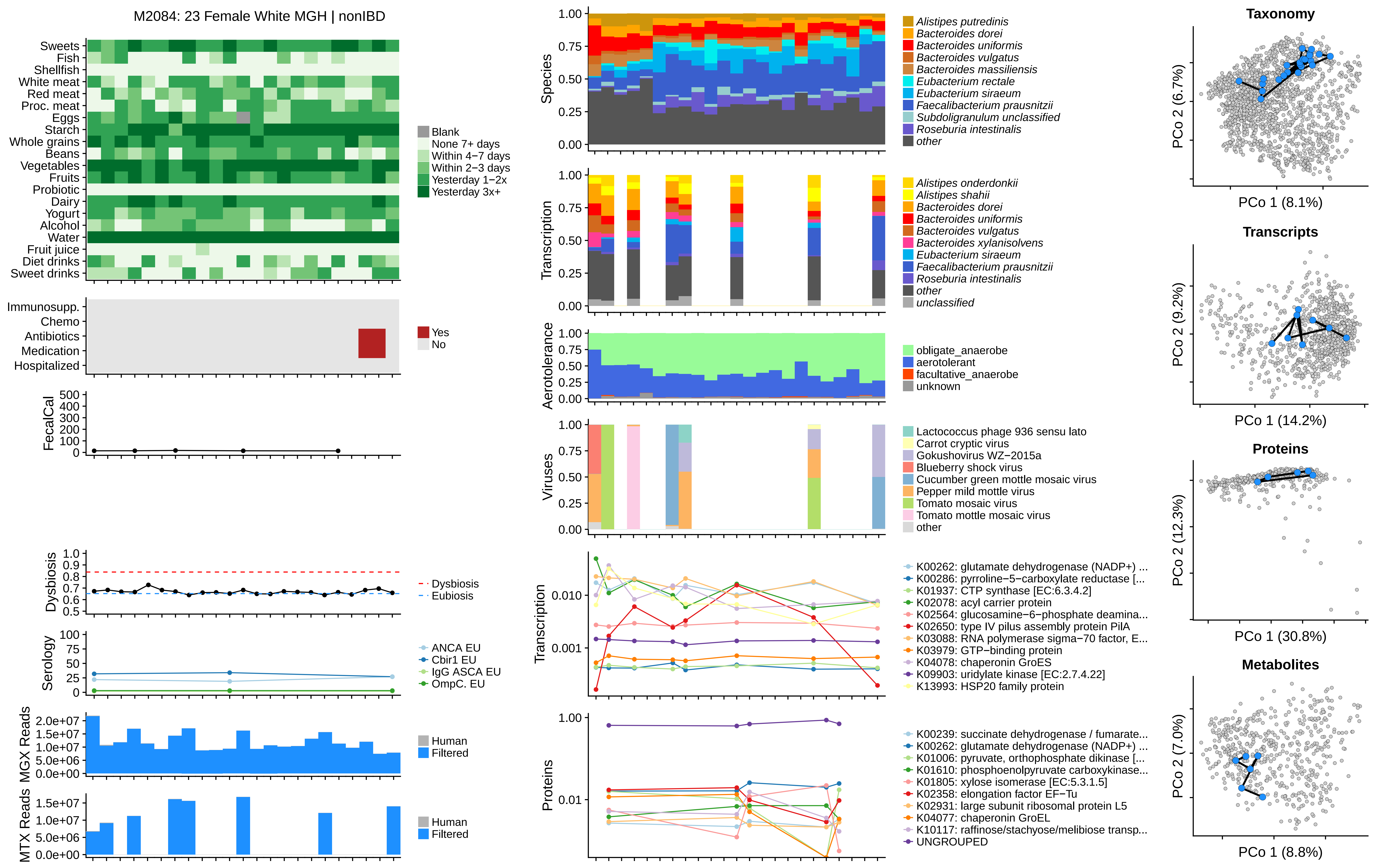
M2084: 23 Female White MGH | nonIBD
Sweets
Fish
Shellfish
White meat
Red meat
Proc. meat
Eggs
Starch
Whole grains
Beans
Vegetables
Fruits
Probiotic
Dairy
Yogurt
Alcohol
Water
Fruit juice
Diet drinks
Sweet drinks
Blank
None 7+ days
Within 4−7 days
Within 2−3 days
Yesterday 1−2x
Yesterday 3x+
Immunosupp.
Chemo
Antibiotics
Medication
Hospitalized
Yes
No
FecalCal
500
400
300
200
100
0
Dysbiosis
1.0
0.9
0.8
0.7
0.6
0.5
Dysbiosis
Eubiosis
Serology
100
75
50
25
0
ANCA EU
Cbir1 EU
IgG ASCA EU
OmpC. EU
MGX Reads
2.0e+07
1.5e+07
1.0e+07
5.0e+06
0.0e+00
Human
Filtered
MTX Reads
1.5e+07
1.0e+07
5.0e+06
0.0e+00
Human
Filtered
Species
1.00
0.75
0.50
0.25
0.00
Alistipes putredinis
Bacteroides dorei
Bacteroides uniformis
Bacteroides vulgatus
Bacteroides massiliensis
Eubacterium rectale
Eubacterium siraeum
Faecalibacterium prausnitzii
Subdoligranulum unclassified
Roseburia intestinalis
other
Transcription
1.00
0.75
0.50
0.25
0.00
Alistipes onderdonkii
Alistipes shahii
Bacteroides dorei
Bacteroides uniformis
Bacteroides vulgatus
Bacteroides xylanisolvens
Eubacterium siraeum
Faecalibacterium prausnitzii
Roseburia intestinalis
other
unclassified
Aerotolerance
1.00
0.75
0.50
0.25
0.00
obligate_anaerobe
aerotolerant
facultative_anaerobe
unknown
Viruses
1.00
0.75
0.50
0.25
0.00
Lactococcus phage 936 sensu lato
Carrot cryptic virus
Gokushovirus WZ−2015a
Blueberry shock virus
Cucumber green mottle mosaic virus
Pepper mild mottle virus
Tomato mosaic virus
Tomato mottle mosaic virus
other
Transcription
0.010
0.001
K00262: glutamate dehydrogenase (NADP+) ...
K00286: pyrroline−5−carboxylate reductase [...
K01937: CTP synthase [EC:6.3.4.2]
K02078: acyl carrier protein
K02564: glucosamine−6−phosphate deamina...
K02650: type IV pilus assembly protein PilA
K03088: RNA polymerase sigma−70 factor, E...
K03979: GTP−binding protein
K04078: chaperonin GroES
K09903: uridylate kinase [EC:2.7.4.22]
K13993: HSP20 family protein
Proteins
1.00
0.01
K00239: succinate dehydrogenase / fumarate...
K00262: glutamate dehydrogenase (NADP+) ...
K01006: pyruvate, orthophosphate dikinase [...
K01610: phosphoenolpyruvate carboxykinase...
K01805: xylose isomerase [EC:5.3.1.5]
K02358: elongation factor EF−Tu
K02931: large subunit ribosomal protein L5
K04077: chaperonin GroEL
K10117: raffinose/stachyose/melibiose transp...
UNGROUPED
Taxonomy
PCo 2 (6.7%)
PCo 1 (8.1%)
Transcripts
PCo 2 (9.2%)
PCo 1 (14.2%)
Proteins
PCo 2 (12.3%)
PCo 1 (30.8%)
Metabolites
PCo 2 (7.0%)
PCo 1 (8.8%)

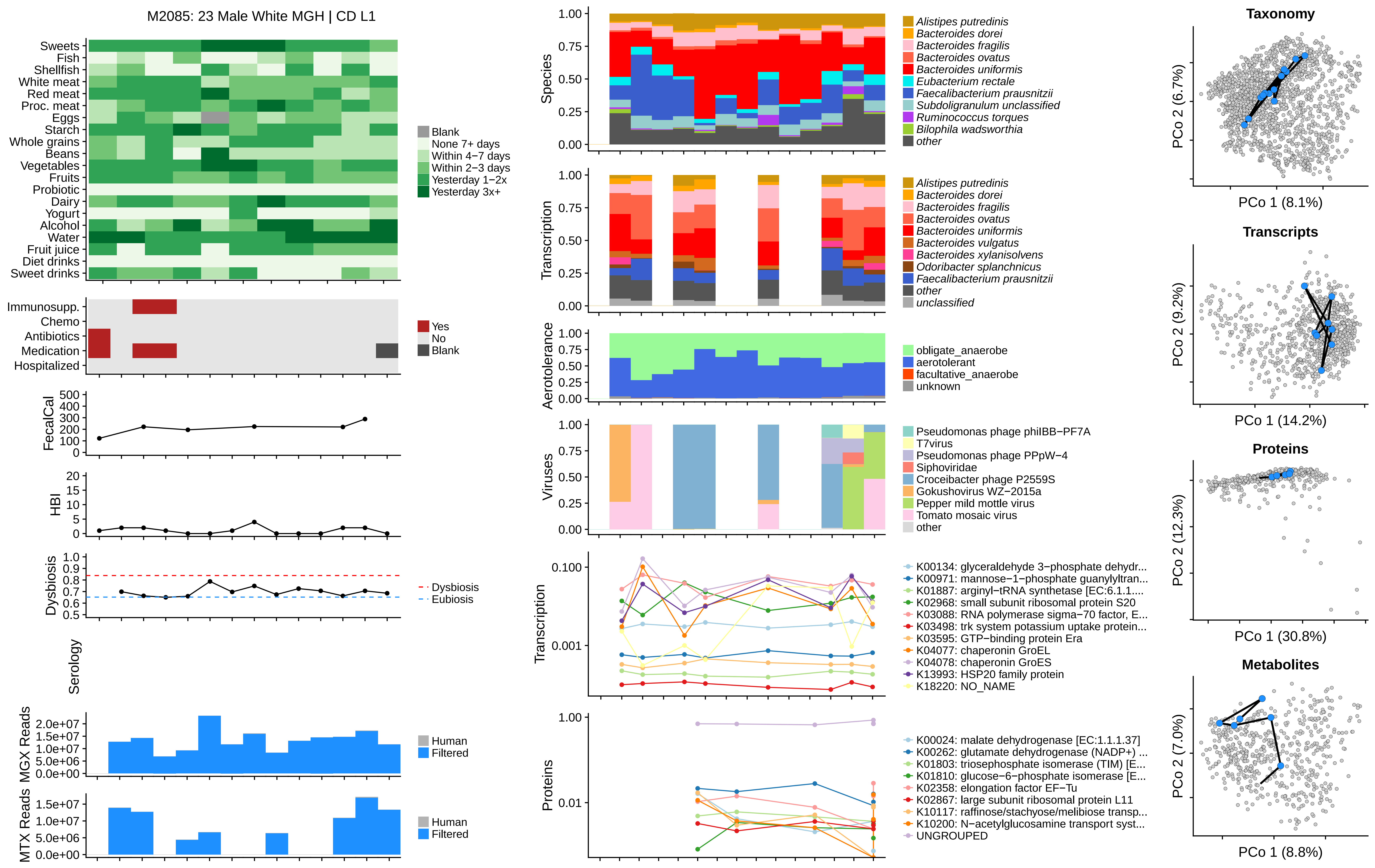

M2085: 23 Male White MGH | CD L1
Sweets
Fish
Shellfish
White meat
Red meat
Proc. meat
Eggs
Starch
Whole grains
Beans
Vegetables
Fruits
Probiotic
Dairy
Yogurt
Alcohol
Water
Fruit juice
Diet drinks
Sweet drinks
Blank
None 7+ days
Within 4−7 days
Within 2−3 days
Yesterday 1−2x
Yesterday 3x+
Immunosupp.
Chemo
Antibiotics
Medication
Hospitalized
Yes
No
Blank
FecalCal
HBI
Dysbiosis
Dysbiosis
Eubiosis
Serology
MGX Reads
Human
Filtered
MTX Reads
Human
Filtered
Species
Alistipes putredinis
Bacteroides dorei
Bacteroides fragilis
Bacteroides ovatus
Bacteroides uniformis
Eubacterium rectale
Faecalibacterium prausnitzii
Subdoligranulum unclassified
Ruminococcus torques
Bilophila wadsworthia
other
Transcription
Alistipes putredinis
Bacteroides dorei
Bacteroides fragilis
Bacteroides ovatus
Bacteroides uniformis
Bacteroides vulgatus
Bacteroides xylanisolvens
Odoribacter splanchnicus
Faecalibacterium prausnitzii
other
unclassified
Aerotolerance
obligate_anaerobe
aerotolerant
facultative_anaerobe
unknown
Viruses
Pseudomonas phage phiIBB−PF7A
T7virus
Pseudomonas phage PPpW−4
Siphoviridae
Croceibacter phage P2559S
Gokushovirus WZ−2015a
Pepper mild mottle virus
Tomato mosaic virus
other
Transcription
K00134: glyceraldehyde 3−phosphate dehydr...
K00971: mannose−1−phosphate guanylyltran...
K01887: arginyl−tRNA synthetase [EC:6.1.1....
K02968: small subunit ribosomal protein S20
K03088: RNA polymerase sigma−70 factor, E...
K03498: trk system potassium uptake protein...
K03595: GTP−binding protein Era
K04077: chaperonin GroEL
K04078: chaperonin GroES
K13993: HSP20 family protein
K18220: NO_NAME
Proteins
K00024: malate dehydrogenase [EC:1.1.1.37]
K00262: glutamate dehydrogenase (NADP+) ...
K01803: triosephosphate isomerase (TIM) [E...
K01810: glucose−6−phosphate isomerase [E...
K02358: elongation factor EF−Tu
K02867: large subunit ribosomal protein L11
K10117: raffinose/stachyose/melibiose transp...
K10200: N−acetylglucosamine transport syst...
UNGROUPED
Taxonomy
PCo 1 (8.1%)
PCo 2 (6.7%)
Transcripts
PCo 1 (14.2%)
PCo 2 (9.2%)
Proteins
PCo 1 (30.8%)
PCo 2 (12.3%)
Metabolites
PCo 1 (8.8%)
PCo 2 (7.0%)

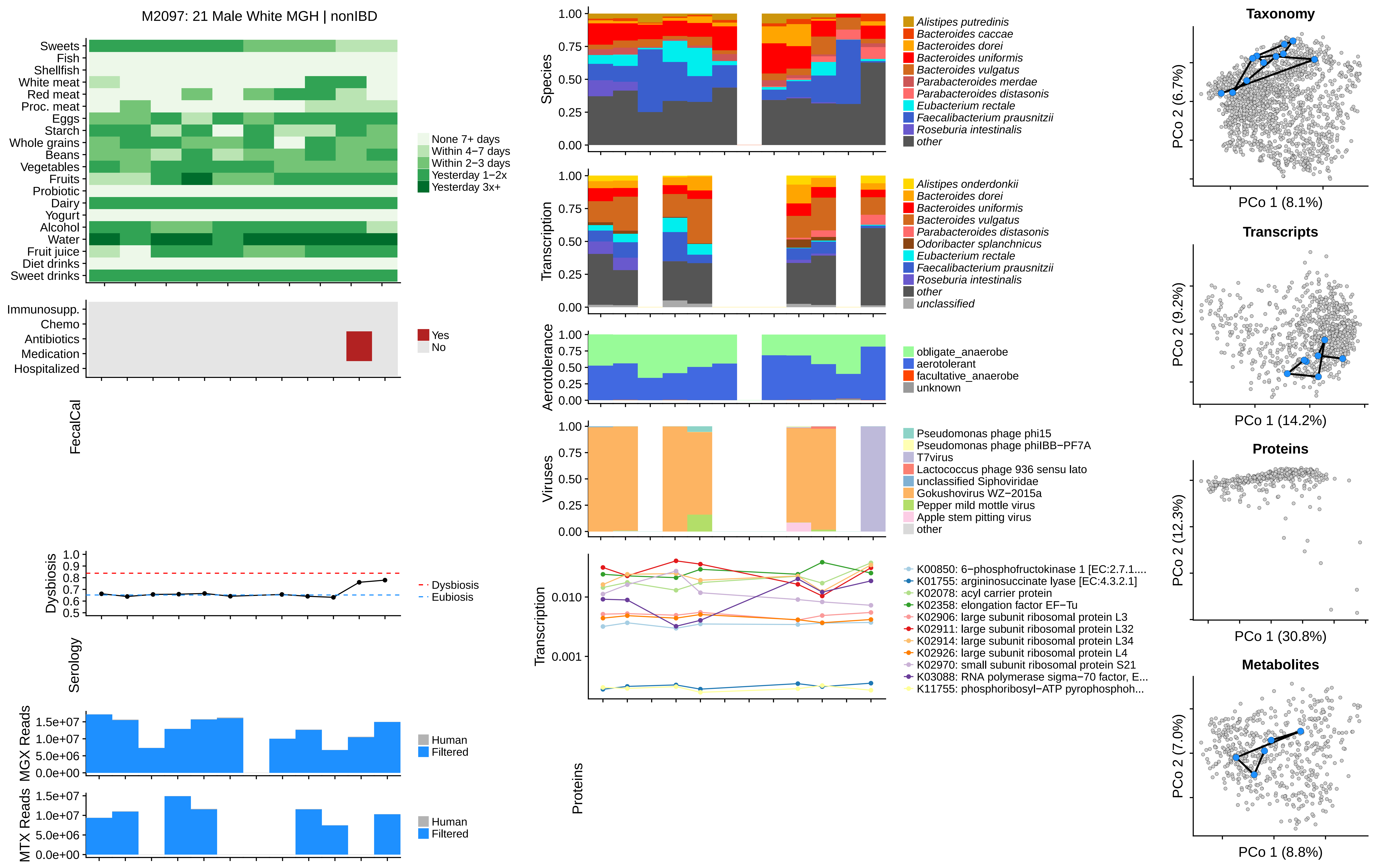

M2097: 21 Male White MGH | nonIBD
Sweets
Fish
Shellfish
White meat
Red meat
Proc. meat
Eggs
Starch
Whole grains
Beans
Vegetables
Fruits
Probiotic
Dairy
Yogurt
Alcohol
Water
Fruit juice
Diet drinks
Sweet drinks
None 7+ days
Within 4−7 days
Within 2−3 days
Yesterday 1−2x
Yesterday 3x+
Immunosupp.
Chemo
Antibiotics
Medication
Hospitalized
Yes
No
FecalCal
Dysbiosis
1.0
0.9
0.8
0.7
0.6
0.5
Dysbiosis
Eubiosis
Serology
MGX Reads
1.5e+07
1.0e+07
5.0e+06
0.0e+00
Human
Filtered
MTX Reads
1.5e+07
1.0e+07
5.0e+06
0.0e+00
Human
Filtered
Species
1.00
0.75
0.50
0.25
0.00
Alistipes putredinis
Bacteroides caccae
Bacteroides dorei
Bacteroides uniformis
Bacteroides vulgatus
Parabacteroides merdae
Parabacteroides distasonis
Eubacterium rectale
Faecalibacterium prausnitzii
Roseburia intestinalis
other
Transcription
1.00
0.75
0.50
0.25
0.00
Alistipes onderdonkii
Bacteroides dorei
Bacteroides uniformis
Bacteroides vulgatus
Parabacteroides distasonis
Odoribacter splanchnicus
Eubacterium rectale
Faecalibacterium prausnitzii
Roseburia intestinalis
other
unclassified
Aerotolerance
1.00
0.75
0.50
0.25
0.00
obligate_anaerobe
aerotolerant
facultative_anaerobe
unknown
Viruses
1.00
0.75
0.50
0.25
0.00
Pseudomonas phage phi15
Pseudomonas phage phiIBB−PF7A
T7virus
Lactococcus phage 936 sensu lato
unclassified Siphoviridae
Gokushovirus WZ−2015a
Pepper mild mottle virus
Apple stem pitting virus
other
Transcription
0.010
0.001
K00850: 6−phosphofructokinase 1 [EC:2.7.1....
K01755: argininosuccinate lyase [EC:4.3.2.1]
K02078: acyl carrier protein
K02358: elongation factor EF−Tu
K02906: large subunit ribosomal protein L3
K02911: large subunit ribosomal protein L32
K02914: large subunit ribosomal protein L34
K02926: large subunit ribosomal protein L4
K02970: small subunit ribosomal protein S21
K03088: RNA polymerase sigma−70 factor, E...
K11755: phosphoribosyl−ATP pyrophosphoh...
Proteins
Taxonomy
PCo 2 (6.7%)
PCo 1 (8.1%)
Transcripts
PCo 2 (9.2%)
PCo 1 (14.2%)
Proteins
PCo 2 (12.3%)
PCo 1 (30.8%)
Metabolites
PCo 2 (7.0%)
PCo 1 (8.8%)

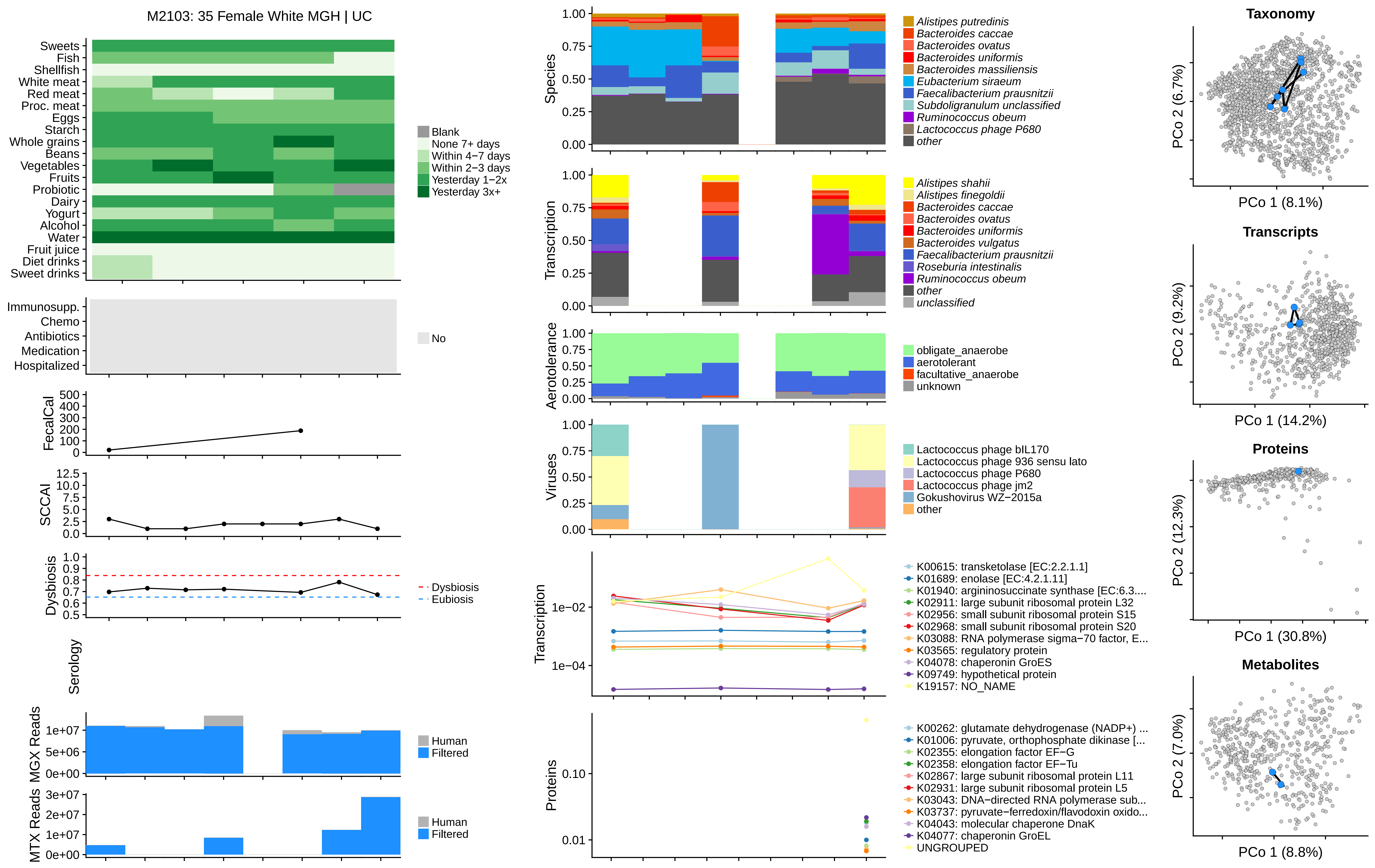
M2103: 35 Female White MGH | UC
Sweets
Fish
Shellfish
White meat
Red meat
Proc. meat
Eggs
Starch
Whole grains
Beans
Vegetables
Fruits
Probiotic
Dairy
Yogurt
Alcohol
Water
Fruit juice
Diet drinks
Sweet drinks
Blank
None 7+ days
Within 4−7 days
Within 2−3 days
Yesterday 1−2x
Yesterday 3x+
Immunosupp.
Chemo
Antibiotics
Medication
Hospitalized
No
FecalCal
500
400
300
200
100
0
SCCAI
12.5
10.0
7.5
5.0
2.5
0.0
Dysbiosis
1.0
0.9
0.8
0.7
0.6
0.5
Dysbiosis
Eubiosis
Serology
MGX Reads
1e+07
5e+06
0e+00
Human
Filtered
MTX Reads
3e+07
2e+07
1e+07
0e+00
Human
Filtered
Species
1.00
0.75
0.50
0.25
0.00
Alistipes putredinis
Bacteroides caccae
Bacteroides ovatus
Bacteroides uniformis
Bacteroides massiliensis
Eubacterium siraeum
Faecalibacterium prausnitzii
Subdoligranulum unclassified
Ruminococcus obeum
Lactococcus phage P680
other
Transcription
Alistipes shahii
Alistipes finegoldii
Bacteroides caccae
Bacteroides ovatus
Bacteroides uniformis
Bacteroides vulgatus
Faecalibacterium prausnitzii
Roseburia intestinalis
Ruminococcus obeum
other
unclassified
Aerotolerance
obligate_anaerobe
aerotolerant
facultative_anaerobe
unknown
Viruses
Lactococcus phage bIL170
Lactococcus phage 936 sensu lato
Lactococcus phage P680
Lactococcus phage jm2
Gokushovirus WZ−2015a
other
Transcription
1e−02
1e−04
K00615: transketolase [EC:2.2.1.1]
K01689: enolase [EC:4.2.1.11]
K01940: argininosuccinate synthase [EC:6.3....
K02911: large subunit ribosomal protein L32
K02956: small subunit ribosomal protein S15
K02968: small subunit ribosomal protein S20
K03088: RNA polymerase sigma−70 factor, E...
K03565: regulatory protein
K04078: chaperonin GroES
K09749: hypothetical protein
K19157: NO_NAME
Proteins
0.10
0.01
K00262: glutamate dehydrogenase (NADP+) ...
K01006: pyruvate, orthophosphate dikinase [...
K02355: elongation factor EF−G
K02358: elongation factor EF−Tu
K02867: large subunit ribosomal protein L11
K02931: large subunit ribosomal protein L5
K03043: DNA−directed RNA polymerase sub...
K03737: pyruvate−ferredoxin/flavodoxin oxido...
K04043: molecular chaperone DnaK
K04077: chaperonin GroEL
UNGROUPED
Taxonomy
PCo 2 (6.7%)
PCo 1 (8.1%)
Transcripts
PCo 2 (9.2%)
PCo 1 (14.2%)
Proteins
PCo 2 (12.3%)
PCo 1 (30.8%)
Metabolites
PCo 2 (7.0%)
PCo 1 (8.8%)

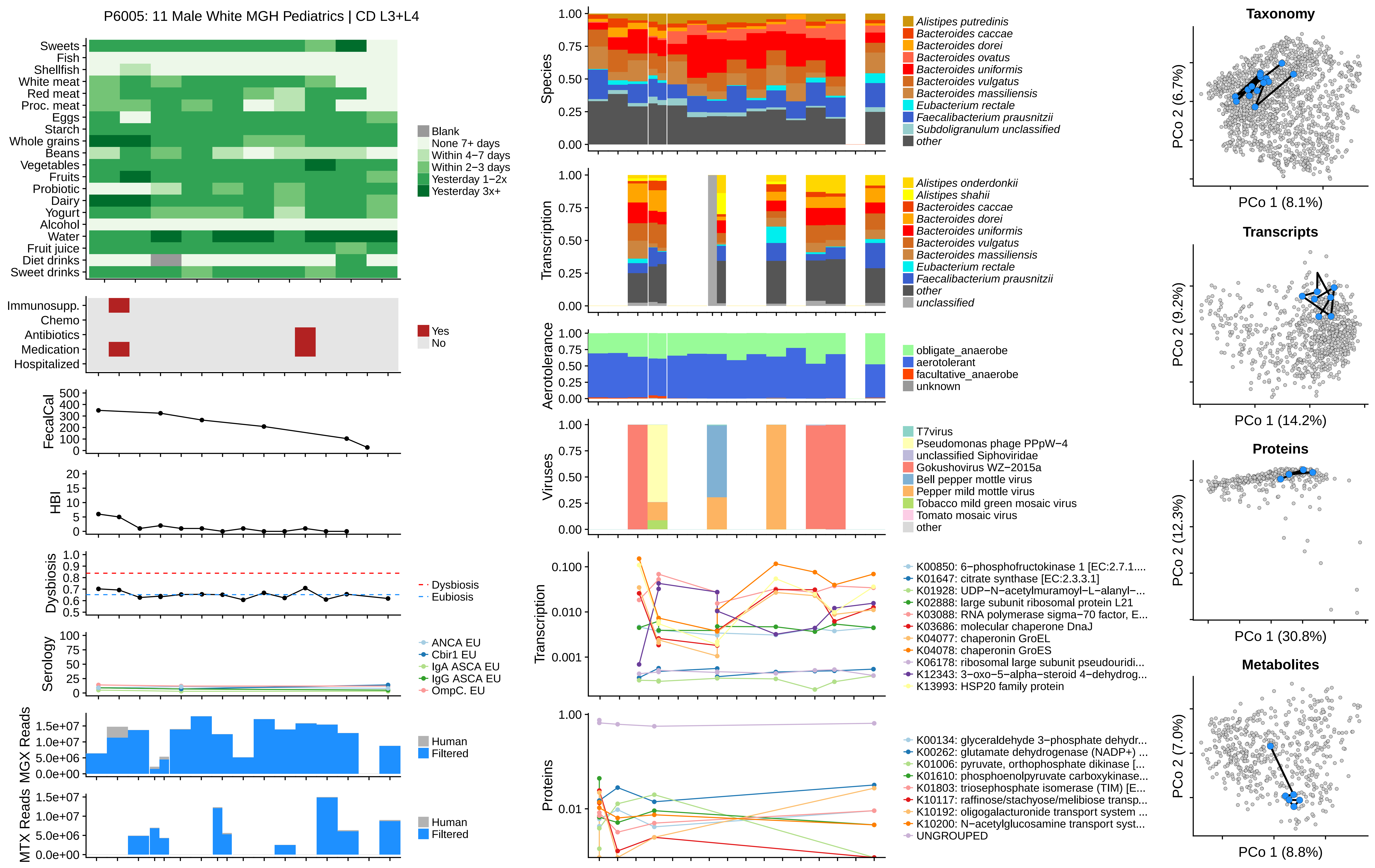

P6005: 11 Male White MGH Pediatrics | CD L3+L4
Sweets
Fish
Shellfish
White meat
Red meat
Proc. meat
Eggs
Starch
Whole grains
Beans
Vegetables
Fruits
Probiotic
Dairy
Yogurt
Alcohol
Water
Fruit juice
Diet drinks
Sweet drinks
Blank
None 7+ days
Within 4−7 days
Within 2−3 days
Yesterday 1−2x
Yesterday 3x+
Immunosupp.
Chemo
Antibiotics
Medication
Hospitalized
Yes
No
FecalCal
HBI
Dysbiosis
Dysbiosis
Eubiosis
Serology
ANCA EU
Cbir1 EU
IgA ASCA EU
IgG ASCA EU
OmpC. EU
MGX Reads
Human
Filtered
MTX Reads
Human
Filtered
Species
Alistipes putredinis
Bacteroides caccae
Bacteroides dorei
Bacteroides ovatus
Bacteroides uniformis
Bacteroides vulgatus
Bacteroides massiliensis
Eubacterium rectale
Faecalibacterium prausnitzii
Subdoligranulum unclassified
other
Transcription
Alistipes onderdonkii
Alistipes shahii
Bacteroides caccae
Bacteroides dorei
Bacteroides uniformis
Bacteroides vulgatus
Bacteroides massiliensis
Eubacterium rectale
Faecalibacterium prausnitzii
other
unclassified
Aerotolerance
obligate_anaerobe
aerotolerant
facultative_anaerobe
unknown
Viruses
T7virus
Pseudomonas phage PPpW−4
unclassified Siphoviridae
Gokushovirus WZ−2015a
Bell pepper mottle virus
Pepper mild mottle virus
Tobacco mild green mosaic virus
Tomato mosaic virus
other
Transcription
K00850: 6−phosphofructokinase 1 [EC:2.7.1....
K01647: citrate synthase [EC:2.3.3.1]
K01928: UDP−N−acetylmuramoyl−L−alanyl−...
K02888: large subunit ribosomal protein L21
K03088: RNA polymerase sigma−70 factor, E...
K03686: molecular chaperone DnaJ
K04077: chaperonin GroEL
K04078: chaperonin GroES
K06178: ribosomal large subunit pseudouridi...
K12343: 3−oxo−5−alpha−steroid 4−dehydrog...
K13993: HSP20 family protein
Proteins
K00134: glyceraldehyde 3−phosphate dehydr...
K00262: glutamate dehydrogenase (NADP+) ...
K01006: pyruvate, orthophosphate dikinase [...
K01610: phosphoenolpyruvate carboxykinase...
K01803: triosephosphate isomerase (TIM) [E...
K10117: raffinose/stachyose/melibiose transp...
K10192: oligogalacturonide transport system ...
K10200: N−acetylglucosamine transport syst...
UNGROUPED
Taxonomy
PCo 1 (8.1%)
PCo 2 (6.7%)
Transcripts
PCo 1 (14.2%)
PCo 2 (9.2%)
Proteins
PCo 1 (30.8%)
PCo 2 (12.3%)
Metabolites
PCo 1 (8.8%)
PCo 2 (7.0%)

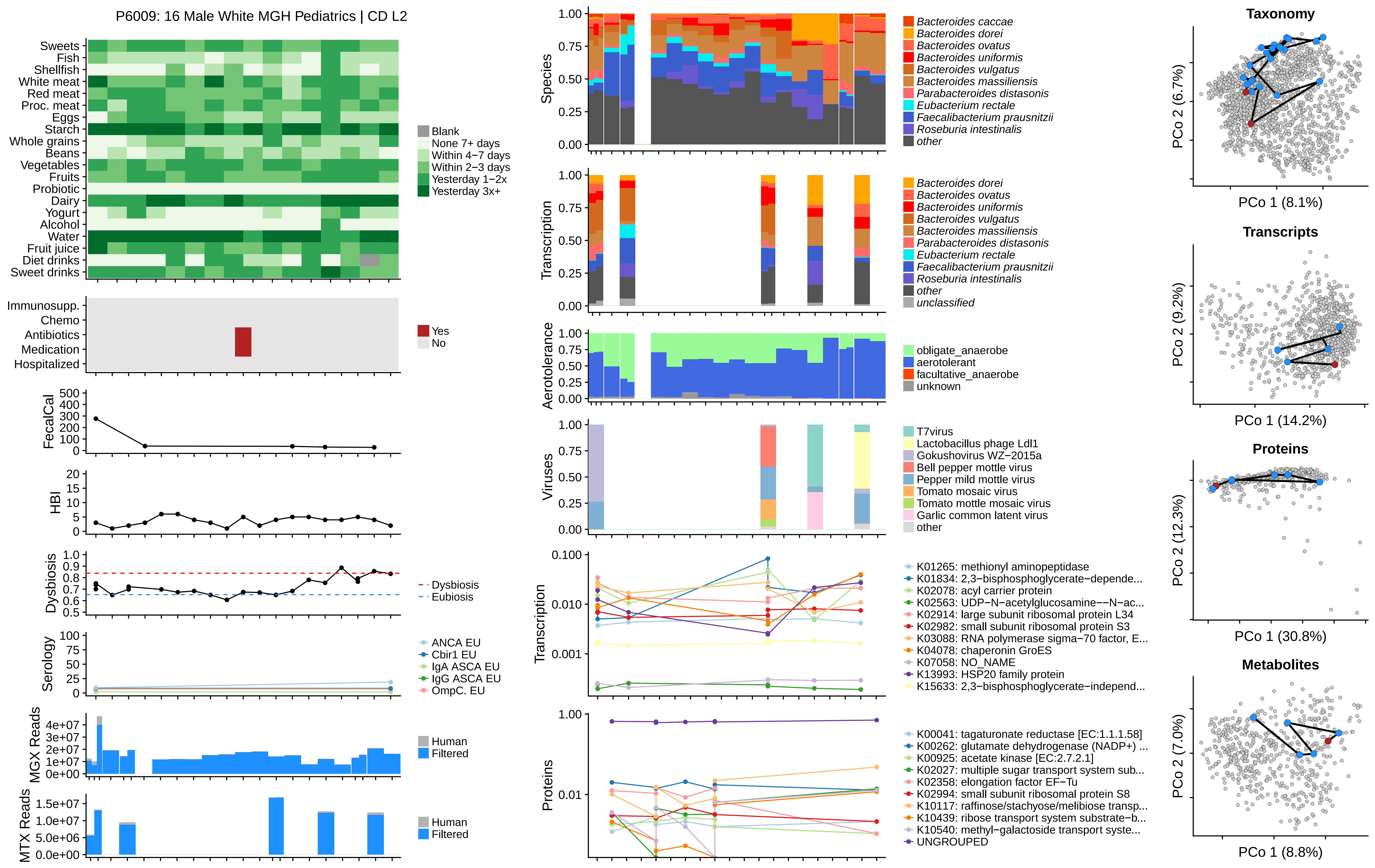
P6009: 16 Male White MGH Pediatrics | CD L2
Sweets
Fish
Shellfish
White meat
Red meat
Proc. meat
Eggs
Starch
Whole grains
Beans
Vegetables
Fruits
Probiotic
Dairy
Yogurt
Alcohol
Water
Fruit juice
Diet drinks
Sweet drinks
Blank
None 7+ days
Within 4−7 days
Within 2−3 days
Yesterday 1−2x
Yesterday 3x+
Immunosupp.
Chemo
Antibiotics
Medication
Hospitalized
Yes
No
FecalCal
500
400
300
200
100
0
HBI
20
15
10
5
0
Dysbiosis
1.0
0.9
0.8
0.7
0.6
0.5
Dysbiosis
Eubiosis
Serology
100
75
50
25
0
ANCA EU
Cbir1 EU
IgA ASCA EU
IgG ASCA EU
OmpC. EU
MGX Reads
4e+07
3e+07
2e+07
1e+07
0e+00
Human
Filtered
MTX Reads
1.5e+07
1.0e+07
5.0e+06
0.0e+00
Human
Filtered
Species
1.00
0.75
0.50
0.25
0.00
Bacteroides caccae
Bacteroides dorei
Bacteroides ovatus
Bacteroides uniformis
Bacteroides vulgatus
Bacteroides massiliensis
Parabacteroides distasonis
Eubacterium rectale
Faecalibacterium prausnitzii
Roseburia intestinalis
other
Transcription
Bacteroides dorei
Bacteroides ovatus
Bacteroides uniformis
Bacteroides vulgatus
Bacteroides massiliensis
Parabacteroides distasonis
Eubacterium rectale
Faecalibacterium prausnitzii
Roseburia intestinalis
other
unclassified
Aerotolerance
obligate_anaerobe
aerotolerant
facultative_anaerobe
unknown
Viruses
T7virus
Lactobacillus phage Ldl1
Gokushovirus WZ−2015a
Bell pepper mottle virus
Pepper mild mottle virus
Tomato mosaic virus
Tomato mottle mosaic virus
Garlic common latent virus
other
Transcription
0.100
0.010
0.001
K01265: methionyl aminopeptidase
K01834: 2,3−bisphosphoglycerate−depende...
K02078: acyl carrier protein
K02563: UDP−N−acetylglucosamine−−N−ac...
K02914: large subunit ribosomal protein L34
K02982: small subunit ribosomal protein S3
K03088: RNA polymerase sigma−70 factor, E...
K04078: chaperonin GroES
K07058: NO_NAME
K13993: HSP20 family protein
K15633: 2,3−bisphosphoglycerate−independ...
Proteins
1.00
0.01
K00041: tagaturonate reductase [EC:1.1.1.58]
K00262: glutamate dehydrogenase (NADP+) ...
K00925: acetate kinase [EC:2.7.2.1]
K02027: multiple sugar transport system sub...
K02358: elongation factor EF−Tu
K02994: small subunit ribosomal protein S8
K10117: raffinose/stachyose/melibiose transp...
K10439: ribose transport system substrate−b...
K10540: methyl−galactoside transport syste...
UNGROUPED
Taxonomy
PCo 2 (6.7%)
PCo 1 (8.1%)
Transcripts
PCo 2 (9.2%)
PCo 1 (14.2%)
Proteins
PCo 2 (12.3%)
PCo 1 (30.8%)
Metabolites
PCo 2 (7.0%)
PCo 1 (8.8%)

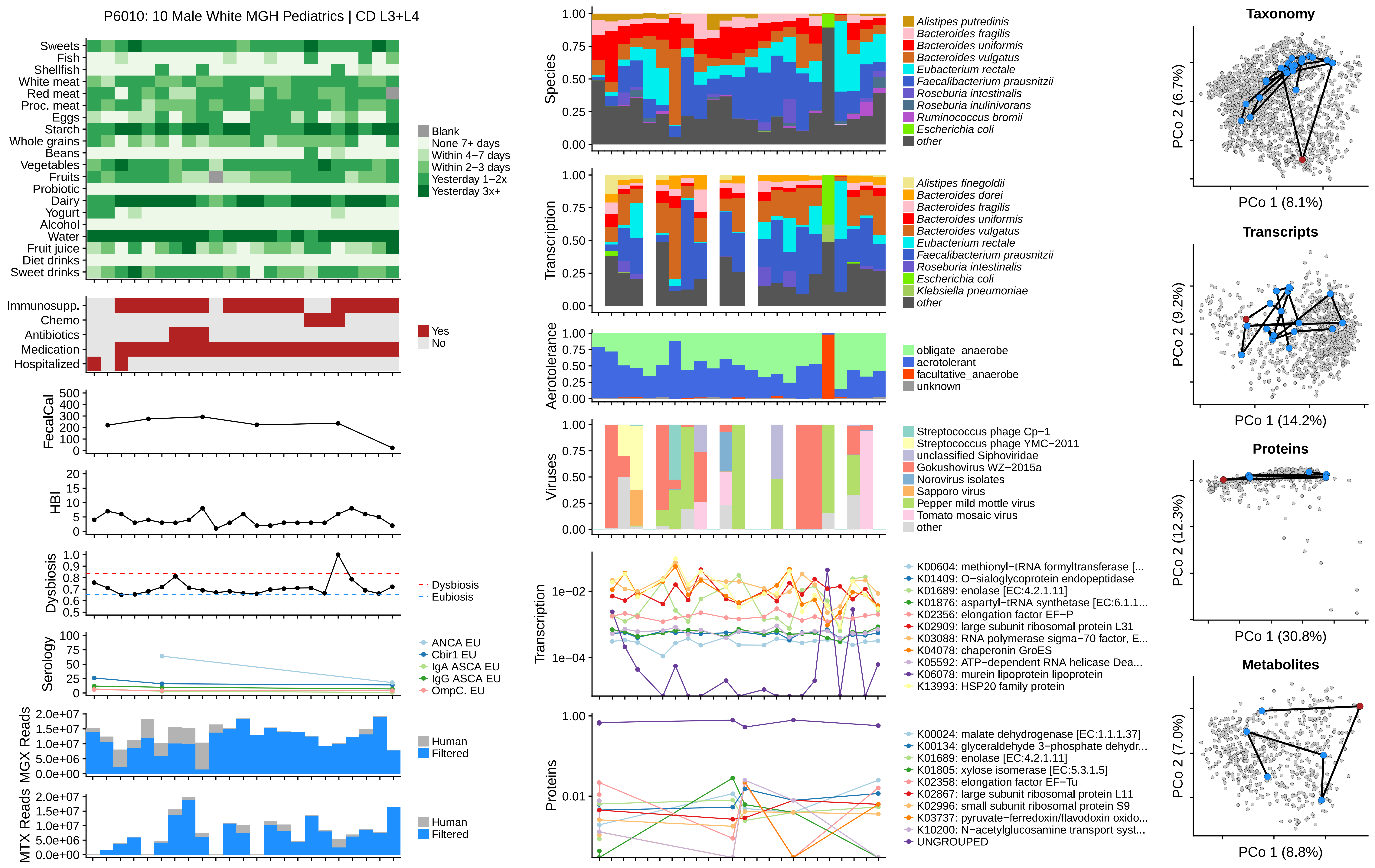
P6010: 10 Male White MGH Pediatrics | CD L3+L4
Sweets
Fish
Shellfish
White meat
Red meat
Proc. meat
Eggs
Starch
Whole grains
Beans
Vegetables
Fruits
Probiotic
Dairy
Yogurt
Alcohol
Water
Fruit juice
Diet drinks
Sweet drinks
Blank
None 7+ days
Within 4−7 days
Within 2−3 days
Yesterday 1−2x
Yesterday 3x+
Immunosupp.
Chemo
Antibiotics
Medication
Hospitalized
Yes
No
FecalCal
HBI
Dysbiosis
Dysbiosis
Eubiosis
Serology
ANCA EU
Cbir1 EU
IgA ASCA EU
IgG ASCA EU
OmpC. EU
MGX Reads
Human
Filtered
MTX Reads
Human
Filtered
Species
Alistipes putredinis
Bacteroides fragilis
Bacteroides uniformis
Bacteroides vulgatus
Eubacterium rectale
Faecalibacterium prausnitzii
Roseburia intestinalis
Roseburia inulinivorans
Ruminococcus bromii
Escherichia coli
other
Transcription
Alistipes finegoldii
Bacteroides dorei
Bacteroides fragilis
Bacteroides uniformis
Bacteroides vulgatus
Eubacterium rectale
Faecalibacterium prausnitzii
Roseburia intestinalis
Escherichia coli
Klebsiella pneumoniae
other
Aerotolerance
obligate_anaerobe
aerotolerant
facultative_anaerobe
unknown
Viruses
Streptococcus phage Cp−1
Streptococcus phage YMC−2011
unclassified Siphoviridae
Gokushovirus WZ−2015a
Norovirus isolates
Sapporo virus
Pepper mild mottle virus
Tomato mosaic virus
other
Transcription
K00604: methionyl−tRNA formyltransferase [...
K01409: O−sialoglycoprotein endopeptidase
K01689: enolase [EC:4.2.1.11]
K01876: aspartyl−tRNA synthetase [EC:6.1.1...
K02356: elongation factor EF−P
K02909: large subunit ribosomal protein L31
K03088: RNA polymerase sigma−70 factor, E...
K04078: chaperonin GroES
K05592: ATP−dependent RNA helicase Dea...
K06078: murein lipoprotein lipoprotein
K13993: HSP20 family protein
Proteins
K00024: malate dehydrogenase [EC:1.1.1.37]
K00134: glyceraldehyde 3−phosphate dehydr...
K01689: enolase [EC:4.2.1.11]
K01805: xylose isomerase [EC:5.3.1.5]
K02358: elongation factor EF−Tu
K02867: large subunit ribosomal protein L11
K02996: small subunit ribosomal protein S9
K03737: pyruvate−ferredoxin/flavodoxin oxido...
K10200: N−acetylglucosamine transport syst...
UNGROUPED
Taxonomy
PCo 1 (8.1%)
PCo 2 (6.7%)
Transcripts
PCo 1 (14.2%)
PCo 2 (9.2%)
Proteins
PCo 1 (30.8%)
PCo 2 (12.3%)
Metabolites
PCo 1 (8.8%)
PCo 2 (7.0%)

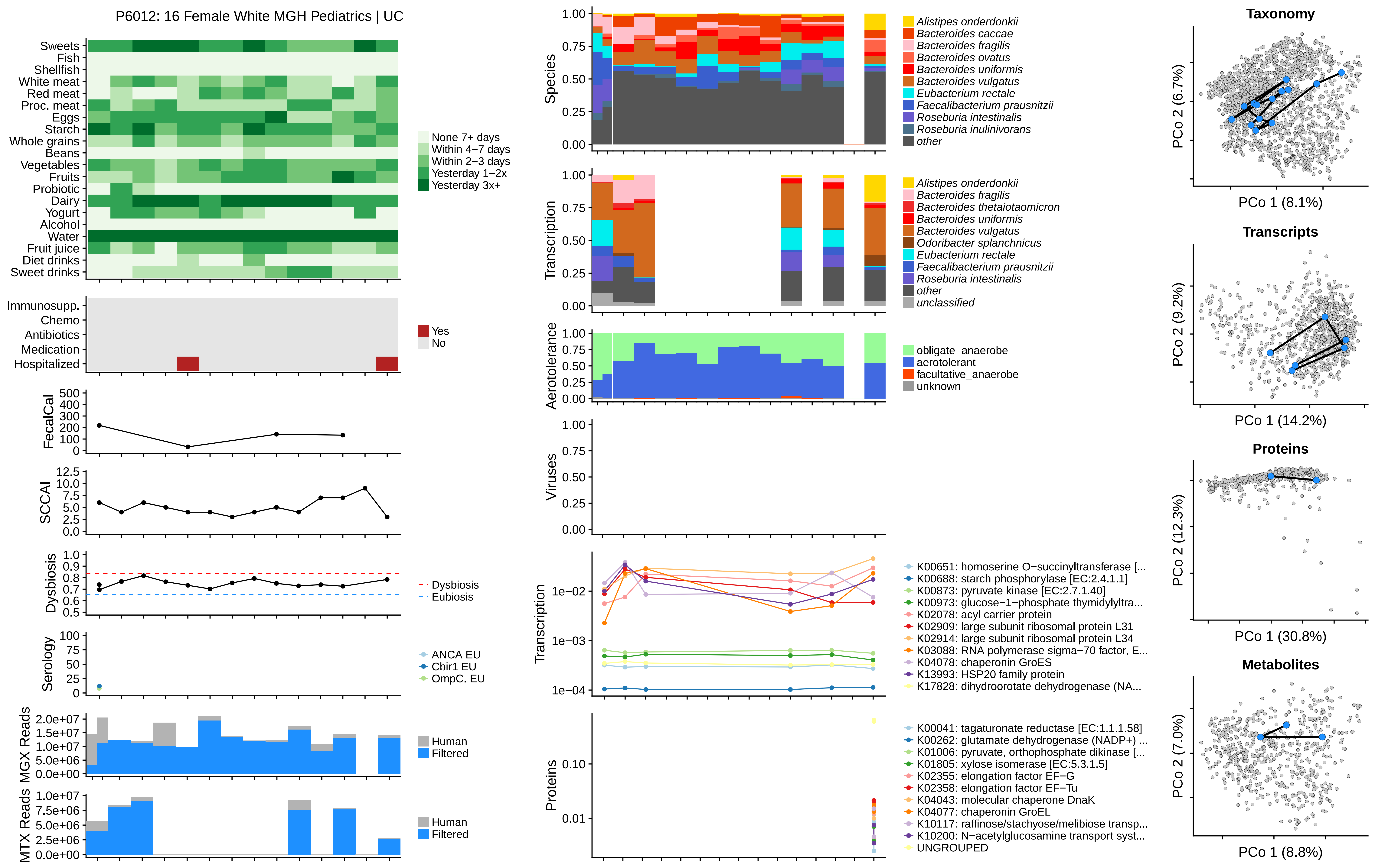
P6012: 16 Female White MGH Pediatrics | UC
Sweets
Fish
Shellfish
White meat
Red meat
Proc. meat
Eggs
Starch
Whole grains
Beans
Vegetables
Fruits
Probiotic
Dairy
Yogurt
Alcohol
Water
Fruit juice
Diet drinks
Sweet drinks
None 7+ days
Within 4−7 days
Within 2−3 days
Yesterday 1−2x
Yesterday 3x+
Immunosupp.
Chemo
Antibiotics
Medication
Hospitalized
Yes
No
FecalCal
SCCAI
Dysbiosis
Dysbiosis
Eubiosis
Serology
ANCA EU
Cbir1 EU
OmpC. EU
MGX Reads
Human
Filtered
MTX Reads
Human
Filtered
Species
Alistipes onderdonkii
Bacteroides caccae
Bacteroides fragilis
Bacteroides ovatus
Bacteroides uniformis
Bacteroides vulgatus
Eubacterium rectale
Faecalibacterium prausnitzii
Roseburia intestinalis
Roseburia inulinivorans
other
Transcription
Alistipes onderdonkii
Bacteroides fragilis
Bacteroides thetaiotaomicron
Bacteroides uniformis
Bacteroides vulgatus
Odoribacter splanchnicus
Eubacterium rectale
Faecalibacterium prausnitzii
Roseburia intestinalis
other
unclassified
Aerotolerance
obligate_anaerobe
aerotolerant
facultative_anaerobe
unknown
Viruses
Transcription
K00651: homoserine O−succinyltransferase [...
K00688: starch phosphorylase [EC:2.4.1.1]
K00873: pyruvate kinase [EC:2.7.1.40]
K00973: glucose−1−phosphate thymidylyltra...
K02078: acyl carrier protein
K02909: large subunit ribosomal protein L31
K02914: large subunit ribosomal protein L34
K03088: RNA polymerase sigma−70 factor, E...
K04078: chaperonin GroES
K13993: HSP20 family protein
K17828: dihydroorotate dehydrogenase (NA...
Proteins
K00041: tagaturonate reductase [EC:1.1.1.58]
K00262: glutamate dehydrogenase (NADP+) ...
K01006: pyruvate, orthophosphate dikinase [...
K01805: xylose isomerase [EC:5.3.1.5]
K02355: elongation factor EF−G
K02358: elongation factor EF−Tu
K04043: molecular chaperone DnaK
K04077: chaperonin GroEL
K10117: raffinose/stachyose/melibiose transp...
K10200: N−acetylglucosamine transport syst...
UNGROUPED
Taxonomy
PCo 1 (8.1%)
PCo 2 (6.7%)
Transcripts
PCo 1 (14.2%)
PCo 2 (9.2%)
Proteins
PCo 1 (30.8%)
PCo 2 (12.3%)
Metabolites
PCo 1 (8.8%)
PCo 2 (7.0%)

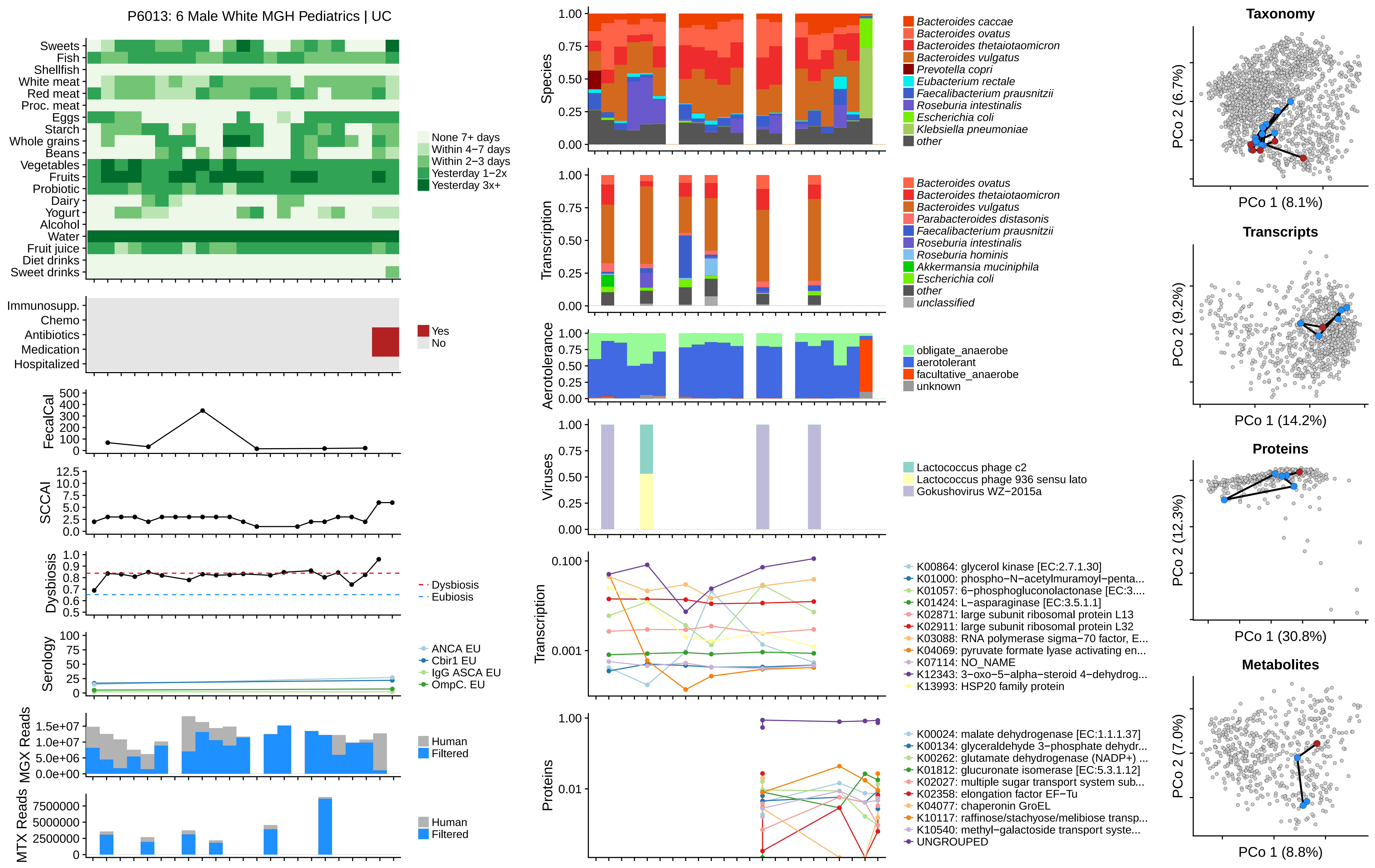
P6013: 6 Male White MGH Pediatrics | UC
Sweets
Fish
Shellfish
White meat
Red meat
Proc. meat
Eggs
Starch
Whole grains
Beans
Vegetables
Fruits
Probiotic
Dairy
Yogurt
Alcohol
Water
Fruit juice
Diet drinks
Sweet drinks
None 7+ days
Within 4−7 days
Within 2−3 days
Yesterday 1−2x
Yesterday 3x+
Immunosupp.
Chemo
Antibiotics
Medication
Hospitalized
Yes
No
FecalCal
SCCAI
Dysbiosis
Dysbiosis
Eubiosis
Serology
ANCA EU
Cbir1 EU
IgG ASCA EU
OmpC. EU
MGX Reads
Human
Filtered
MTX Reads
Human
Filtered
Species
Bacteroides caccae
Bacteroides ovatus
Bacteroides thetaiotaomicron
Bacteroides vulgatus
Prevotella copri
Eubacterium rectale
Faecalibacterium prausnitzii
Roseburia intestinalis
Escherichia coli
Klebsiella pneumoniae
other
Transcription
Bacteroides ovatus
Bacteroides thetaiotaomicron
Bacteroides vulgatus
Parabacteroides distasonis
Faecalibacterium prausnitzii
Roseburia intestinalis
Roseburia hominis
Akkermansia muciniphila
Escherichia coli
other
unclassified
Aerotolerance
obligate_anaerobe
aerotolerant
facultative_anaerobe
unknown
Viruses
Lactococcus phage c2
Lactococcus phage 936 sensu lato
Gokushovirus WZ−2015a
Transcription
K00864: glycerol kinase [EC:2.7.1.30]
K01000: phospho−N−acetylmuramoyl−penta...
K01057: 6−phosphogluconolactonase [EC:3....
K01424: L−asparaginase [EC:3.5.1.1]
K02871: large subunit ribosomal protein L13
K02911: large subunit ribosomal protein L32
K03088: RNA polymerase sigma−70 factor, E...
K04069: pyruvate formate lyase activating en...
K07114: NO_NAME
K12343: 3−oxo−5−alpha−steroid 4−dehydrog...
K13993: HSP20 family protein
Proteins
K00024: malate dehydrogenase [EC:1.1.1.37]
K00134: glyceraldehyde 3−phosphate dehydr...
K00262: glutamate dehydrogenase (NADP+) ...
K01812: glucuronate isomerase [EC:5.3.1.12]
K02027: multiple sugar transport system sub...
K02358: elongation factor EF−Tu
K04077: chaperonin GroEL
K10117: raffinose/stachyose/melibiose transp...
K10540: methyl−galactoside transport syste...
UNGROUPED
Taxonomy
PCo 1 (8.1%)
PCo 2 (6.7%)
Transcripts
PCo 1 (14.2%)
PCo 2 (9.2%)
Proteins
PCo 1 (30.8%)
PCo 2 (12.3%)
Metabolites
PCo 1 (8.8%)
PCo 2 (7.0%)

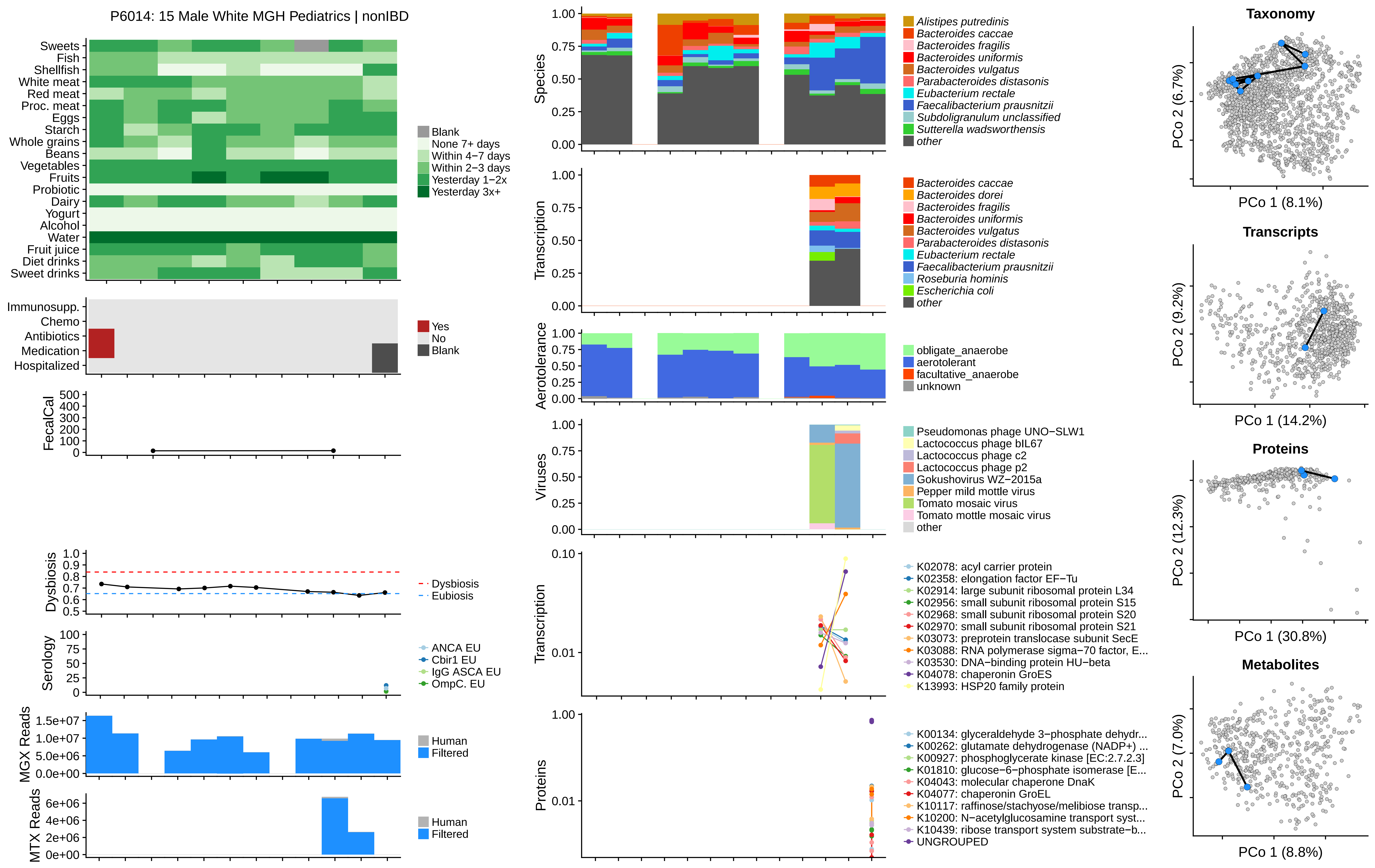
P6014: 15 Male White MGH Pediatrics | nonIBD
Sweets
Fish
Shellfish
White meat
Red meat
Proc. meat
Eggs
Starch
Whole grains
Beans
Vegetables
Fruits
Probiotic
Dairy
Yogurt
Alcohol
Water
Fruit juice
Diet drinks
Sweet drinks
Blank
None 7+ days
Within 4−7 days
Within 2−3 days
Yesterday 1−2x
Yesterday 3x+
Immunosupp.
Chemo
Antibiotics
Medication
Hospitalized
Yes
No
Blank
FecalCal
500
400
300
200
100
0
Dysbiosis
1.0
0.9
0.8
0.7
0.6
0.5
Dysbiosis
Eubiosis
Serology
100
75
50
25
0
ANCA EU
Cbir1 EU
IgG ASCA EU
OmpC. EU
MGX Reads
1.5e+07
1.0e+07
5.0e+06
0.0e+00
Human
Filtered
MTX Reads
6e+06
4e+06
2e+06
0e+00
Human
Filtered
Species
1.00
0.75
0.50
0.25
0.00
Alistipes putredinis
Bacteroides caccae
Bacteroides fragilis
Bacteroides uniformis
Bacteroides vulgatus
Parabacteroides distasonis
Eubacterium rectale
Faecalibacterium prausnitzii
Subdoligranulum unclassified
Sutterella wadsworthensis
other
Transcription
1.00
0.75
0.50
0.25
0.00
Bacteroides caccae
Bacteroides dorei
Bacteroides fragilis
Bacteroides uniformis
Bacteroides vulgatus
Parabacteroides distasonis
Eubacterium rectale
Faecalibacterium prausnitzii
Roseburia hominis
Escherichia coli
other
Aerotolerance
1.00
0.75
0.50
0.25
0.00
obligate_anaerobe
aerotolerant
facultative_anaerobe
unknown
Viruses
1.00
0.75
0.50
0.25
0.00
Pseudomonas phage UNO−SLW1
Lactococcus phage bIL67
Lactococcus phage c2
Lactococcus phage p2
Gokushovirus WZ−2015a
Pepper mild mottle virus
Tomato mosaic virus
Tomato mottle mosaic virus
other
Transcription
0.10
0.01
K02078: acyl carrier protein
K02358: elongation factor EF−Tu
K02914: large subunit ribosomal protein L34
K02956: small subunit ribosomal protein S15
K02968: small subunit ribosomal protein S20
K02970: small subunit ribosomal protein S21
K03073: preprotein translocase subunit SecE
K03088: RNA polymerase sigma−70 factor, E...
K03530: DNA−binding protein HU−beta
K04078: chaperonin GroES
K13993: HSP20 family protein
Proteins
1.00
0.01
K00134: glyceraldehyde 3−phosphate dehydr...
K00262: glutamate dehydrogenase (NADP+) ...
K00927: phosphoglycerate kinase [EC:2.7.2.3]
K01810: glucose−6−phosphate isomerase [E...
K04043: molecular chaperone DnaK
K04077: chaperonin GroEL
K10117: raffinose/stachyose/melibiose transp...
K10200: N−acetylglucosamine transport syst...
K10439: ribose transport system substrate−b...
UNGROUPED
Taxonomy
PCo 2 (6.7%)
PCo 1 (8.1%)
Transcripts
PCo 2 (9.2%)
PCo 1 (14.2%)
Proteins
PCo 2 (12.3%)
PCo 1 (30.8%)
Metabolites
PCo 2 (7.0%)
PCo 1 (8.8%)

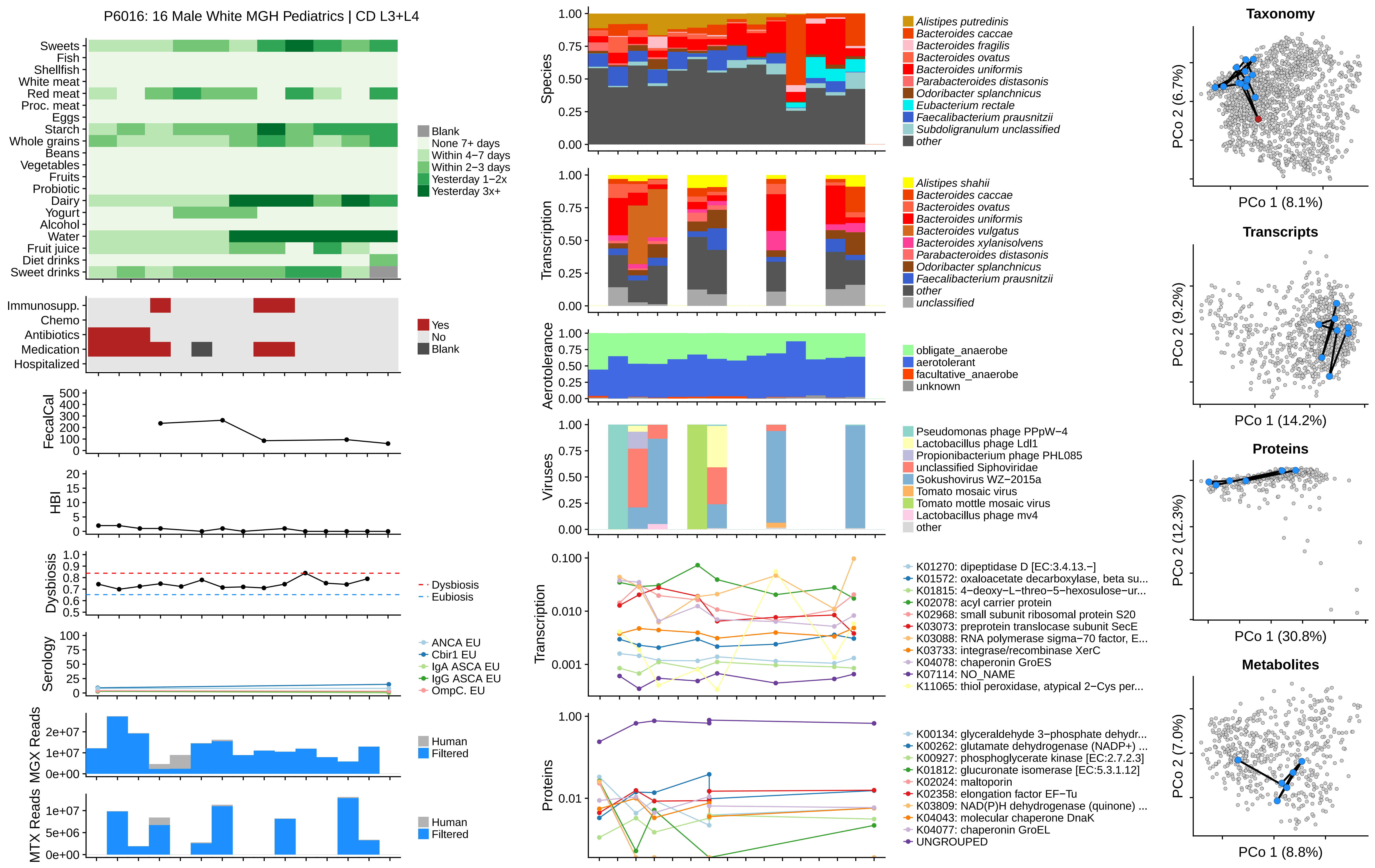
P6016: 16 Male White MGH Pediatrics | CD L3+L4
Sweets
Fish
Shellfish
White meat
Red meat
Proc. meat
Eggs
Starch
Whole grains
Beans
Vegetables
Fruits
Probiotic
Dairy
Yogurt
Alcohol
Water
Fruit juice
Diet drinks
Sweet drinks
Blank
None 7+ days
Within 4−7 days
Within 2−3 days
Yesterday 1−2x
Yesterday 3x+
Immunosupp.
Chemo
Antibiotics
Medication
Hospitalized
Yes
No
Blank
FecalCal
HBI
Dysbiosis
Dysbiosis
Eubiosis
Serology
ANCA EU
Cbir1 EU
IgA ASCA EU
IgG ASCA EU
OmpC. EU
MGX Reads
Human
Filtered
MTX Reads
Human
Filtered
Species
Alistipes putredinis
Bacteroides caccae
Bacteroides fragilis
Bacteroides ovatus
Bacteroides uniformis
Parabacteroides distasonis
Odoribacter splanchnicus
Eubacterium rectale
Faecalibacterium prausnitzii
Subdoligranulum unclassified
other
Transcription
Alistipes shahii
Bacteroides caccae
Bacteroides ovatus
Bacteroides uniformis
Bacteroides vulgatus
Bacteroides xylanisolvens
Parabacteroides distasonis
Odoribacter splanchnicus
Faecalibacterium prausnitzii
other
unclassified
Aerotolerance
obligate_anaerobe
aerotolerant
facultative_anaerobe
unknown
Viruses
Pseudomonas phage PPpW−4
Lactobacillus phage Ldl1
Propionibacterium phage PHL085
unclassified Siphoviridae
Gokushovirus WZ−2015a
Tomato mosaic virus
Tomato mottle mosaic virus
Lactobacillus phage mv4
other
Transcription
K01270: dipeptidase D [EC:3.4.13.−]
K01572: oxaloacetate decarboxylase, beta su...
K01815: 4−deoxy−L−threo−5−hexosulose−ur...
K02078: acyl carrier protein
K02968: small subunit ribosomal protein S20
K03073: preprotein translocase subunit SecE
K03088: RNA polymerase sigma−70 factor, E...
K03733: integrase/recombinase XerC
K04078: chaperonin GroES
K07114: NO_NAME
K11065: thiol peroxidase, atypical 2−Cys per...
Proteins
K00134: glyceraldehyde 3−phosphate dehydr...
K00262: glutamate dehydrogenase (NADP+) ...
K00927: phosphoglycerate kinase [EC:2.7.2.3]
K01812: glucuronate isomerase [EC:5.3.1.12]
K02024: maltoporin
K02358: elongation factor EF−Tu
K03809: NAD(P)H dehydrogenase (quinone) ...
K04043: molecular chaperone DnaK
K04077: chaperonin GroEL
UNGROUPED
Taxonomy
PCo 1 (8.1%)
PCo 2 (6.7%)
Transcripts
PCo 1 (14.2%)
PCo 2 (9.2%)
Proteins
PCo 1 (30.8%)
PCo 2 (12.3%)
Metabolites
PCo 1 (8.8%)
PCo 2 (7.0%)

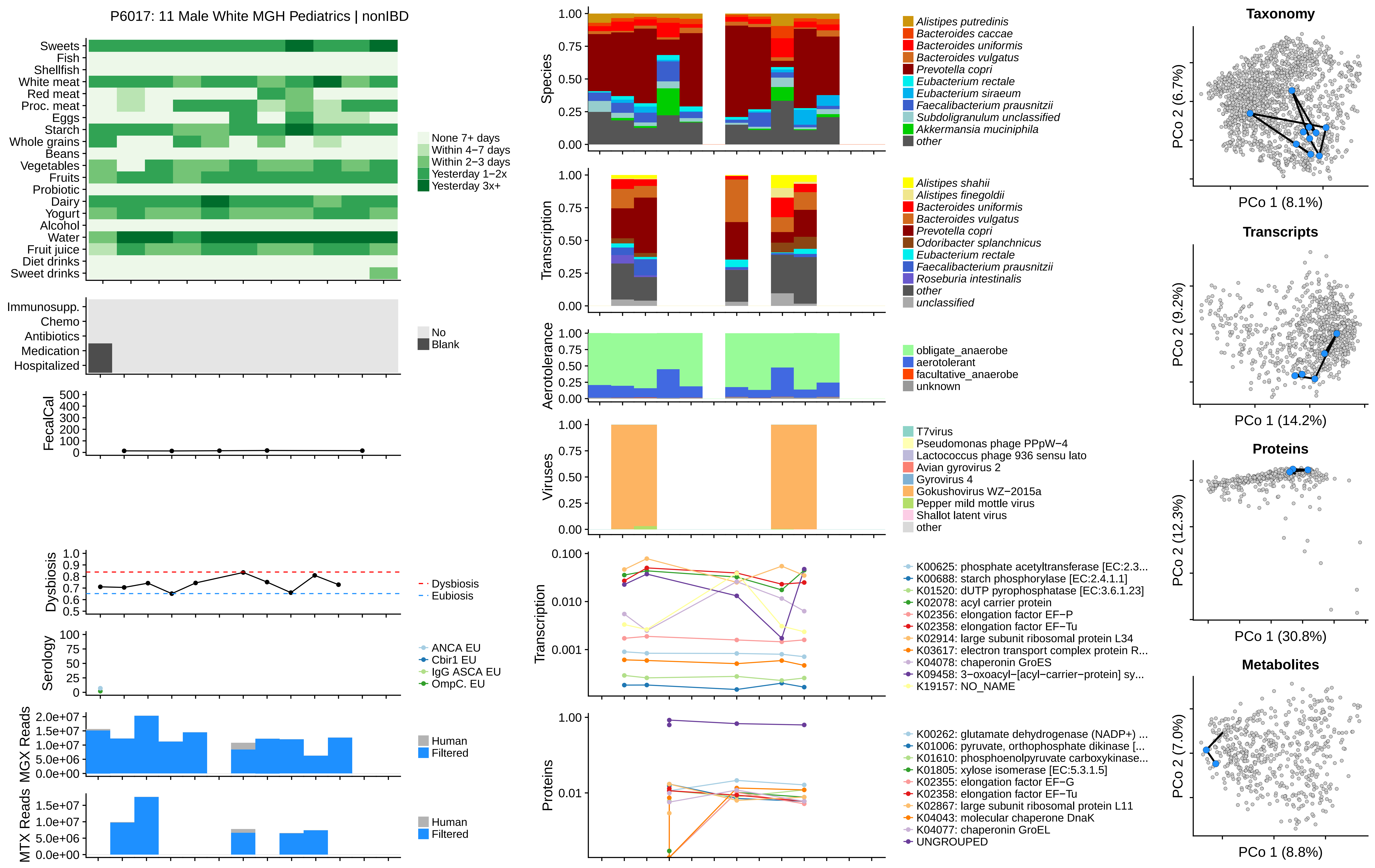
P6017: 11 Male White MGH Pediatrics | nonIBD
Sweets
Fish
Shellfish
White meat
Red meat
Proc. meat
Eggs
Starch
Whole grains
Beans
Vegetables
Fruits
Probiotic
Dairy
Yogurt
Alcohol
Water
Fruit juice
Diet drinks
Sweet drinks
None 7+ days
Within 4−7 days
Within 2−3 days
Yesterday 1−2x
Yesterday 3x+
Immunosupp.
Chemo
Antibiotics
Medication
Hospitalized
No
Blank
FecalCal
Dysbiosis
Eubiosis
Serology
ANCA EU
Cbir1 EU
IgG ASCA EU
OmpC. EU
MGX Reads
MTX Reads
Human
Filtered
Species
Alistipes putredinis
Bacteroides caccae
Bacteroides uniformis
Bacteroides vulgatus
Prevotella copri
Eubacterium rectale
Eubacterium siraeum
Faecalibacterium prausnitzii
Subdoligranulum unclassified
Akkermansia muciniphila
other
Transcription
Alistipes shahii
Alistipes finegoldii
Bacteroides uniformis
Bacteroides vulgatus
Prevotella copri
Odoribacter splanchnicus
Eubacterium rectale
Faecalibacterium prausnitzii
Roseburia intestinalis
other
unclassified
Aerotolerance
obligate_anaerobe
aerotolerant
facultative_anaerobe
unknown
Viruses
T7virus
Pseudomonas phage PPpW−4
Lactococcus phage 936 sensu lato
Avian gyrovirus 2
Gyrovirus 4
Gokushovirus WZ−2015a
Pepper mild mottle virus
Shallot latent virus
other
Transcription
K00625: phosphate acetyltransferase [EC:2.3...
K00688: starch phosphorylase [EC:2.4.1.1]
K01520: dUTP pyrophosphatase [EC:3.6.1.23]
K02078: acyl carrier protein
K02356: elongation factor EF−P
K02358: elongation factor EF−Tu
K02914: large subunit ribosomal protein L34
K03617: electron transport complex protein R...
K04078: chaperonin GroES
K09458: 3−oxoacyl−[acyl−carrier−protein] sy...
K19157: NO_NAME
Proteins
K00262: glutamate dehydrogenase (NADP+) ...
K01006: pyruvate, orthophosphate dikinase [...
K01610: phosphoenolpyruvate carboxykinase...
K01805: xylose isomerase [EC:5.3.1.5]
K02355: elongation factor EF−G
K02358: elongation factor EF−Tu
K02867: large subunit ribosomal protein L11
K04043: molecular chaperone DnaK
K04077: chaperonin GroEL
UNGROUPED
Taxonomy
PCo 1 (8.1%)
PCo 2 (6.7%)
Transcripts
PCo 1 (14.2%)
PCo 2 (9.2%)
Proteins
PCo 1 (30.8%)
PCo 2 (12.3%)
Metabolites
PCo 1 (8.8%)
PCo 2 (7.0%)

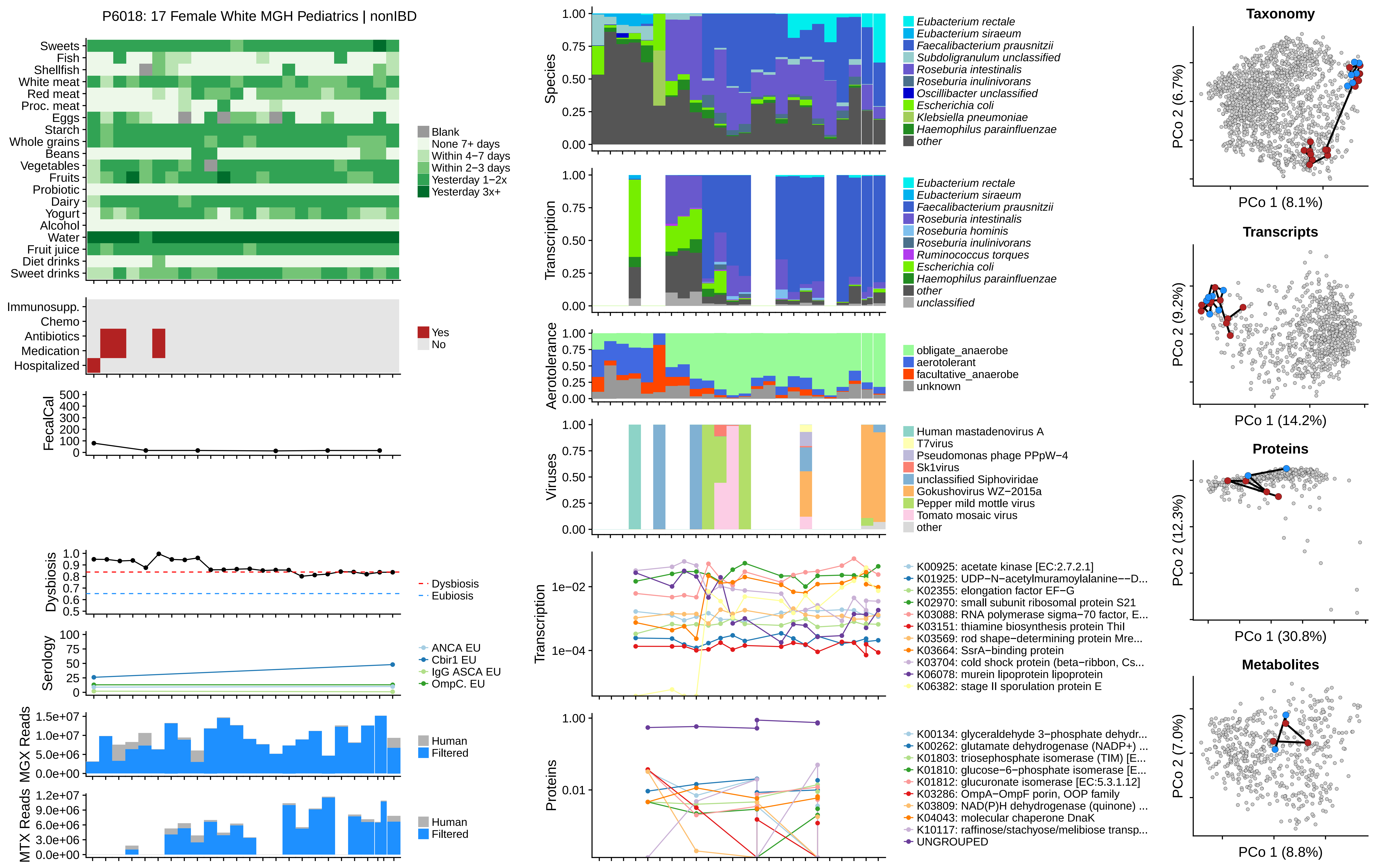
P6018: 17 Female White MGH Pediatrics | nonIBD
Sweets
Fish
Shellfish
White meat
Red meat
Proc. meat
Eggs
Starch
Whole grains
Beans
Vegetables
Fruits
Probiotic
Dairy
Yogurt
Alcohol
Water
Fruit juice
Diet drinks
Sweet drinks
Blank
None 7+ days
Within 4−7 days
Within 2−3 days
Yesterday 1−2x
Yesterday 3x+
Immunosupp.
Chemo
Antibiotics
Medication
Hospitalized
Yes
No
FecalCal
Dysbiosis
Dysbiosis
Eubiosis
Serology
ANCA EU
Cbir1 EU
IgG ASCA EU
OmpC. EU
MGX Reads
Human
Filtered
MTX Reads
Human
Filtered
Species
Eubacterium rectale
Eubacterium siraeum
Faecalibacterium prausnitzii
Subdoligranulum unclassified
Roseburia intestinalis
Roseburia inulinivorans
Oscillibacter unclassified
Escherichia coli
Klebsiella pneumoniae
Haemophilus parainfluenzae
other
Transcription
Eubacterium rectale
Eubacterium siraeum
Faecalibacterium prausnitzii
Roseburia intestinalis
Roseburia hominis
Roseburia inulinivorans
Ruminococcus torques
Escherichia coli
Haemophilus parainfluenzae
other
unclassified
Aerotolerance
obligate_anaerobe
aerotolerant
facultative_anaerobe
unknown
Viruses
Human mastadenovirus A
T7virus
Pseudomonas phage PPpW−4
Sk1virus
unclassified Siphoviridae
Gokushovirus WZ−2015a
Pepper mild mottle virus
Tomato mosaic virus
other
Transcription
K00925: acetate kinase [EC:2.7.2.1]
K01925: UDP−N−acetylmuramoylalanine−−D...
K02355: elongation factor EF−G
K02970: small subunit ribosomal protein S21
K03088: RNA polymerase sigma−70 factor, E...
K03151: thiamine biosynthesis protein ThiI
K03569: rod shape−determining protein Mre...
K03664: SsrA−binding protein
K03704: cold shock protein (beta−ribbon, Cs...
K06078: murein lipoprotein lipoprotein
K06382: stage II sporulation protein E
Proteins
K00134: glyceraldehyde 3−phosphate dehydr...
K00262: glutamate dehydrogenase (NADP+) ...
K01803: triosephosphate isomerase (TIM) [E...
K01810: glucose−6−phosphate isomerase [E...
K01812: glucuronate isomerase [EC:5.3.1.12]
K03286: OmpA−OmpF porin, OOP family
K03809: NAD(P)H dehydrogenase (quinone) ...
K04043: molecular chaperone DnaK
K10117: raffinose/stachyose/melibiose transp...
UNGROUPED
Taxonomy
PCo 1 (8.1%)
PCo 2 (6.7%)
Transcripts
PCo 1 (14.2%)
PCo 2 (9.2%)
Proteins
PCo 1 (30.8%)
PCo 2 (12.3%)
Metabolites
PCo 1 (8.8%)
PCo 2 (7.0%)

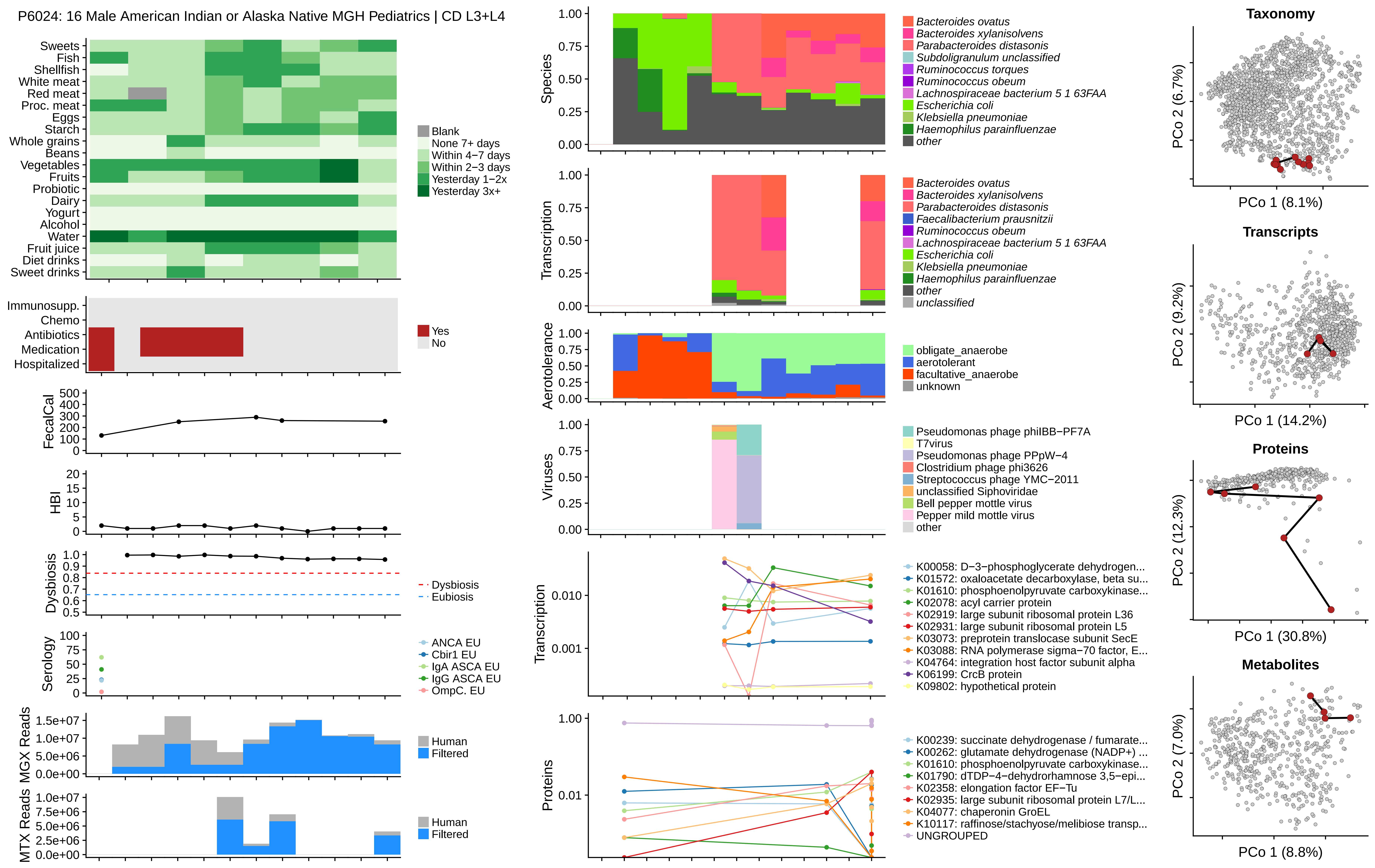
P6024: 16 Male American Indian or Alaska Native MGH Pediatrics | CD L3+L4
Sweets
Fish
Shellfish
White meat
Red meat
Proc. meat
Eggs
Starch
Whole grains
Beans
Vegetables
Fruits
Probiotic
Dairy
Yogurt
Alcohol
Water
Fruit juice
Diet drinks
Sweet drinks
Blank
None 7+ days
Within 4−7 days
Within 2−3 days
Yesterday 1−2x
Yesterday 3x+
Immunosupp.
Chemo
Antibiotics
Medication
Hospitalized
Yes
No
FecalCal
HBI
Dysbiosis
Dysbiosis
Eubiosis
Serology
ANCA EU
Cbir1 EU
IgA ASCA EU
IgG ASCA EU
OmpC. EU
MGX Reads
Human
Filtered
MTX Reads
Human
Filtered
Species
Bacteroides ovatus
Bacteroides xylanisolvens
Parabacteroides distasonis
Subdoligranulum unclassified
Ruminococcus torques
Ruminococcus obeum
Lachnospiraceae bacterium 5 1 63FAA
Escherichia coli
Klebsiella pneumoniae
Haemophilus parainfluenzae
other
Transcription
Bacteroides ovatus
Bacteroides xylanisolvens
Parabacteroides distasonis
Faecalibacterium prausnitzii
Ruminococcus obeum
Lachnospiraceae bacterium 5 1 63FAA
Escherichia coli
Klebsiella pneumoniae
Haemophilus parainfluenzae
other
unclassified
Aerotolerance
obligate_anaerobe
aerotolerant
facultative_anaerobe
unknown
Viruses
Pseudomonas phage phiIBB−PF7A
T7virus
Pseudomonas phage PPpW−4
Clostridium phage phi3626
Streptococcus phage YMC−2011
unclassified Siphoviridae
Bell pepper mottle virus
Pepper mild mottle virus
other
Transcription
K00058: D−3−phosphoglycerate dehydrogen...
K01572: oxaloacetate decarboxylase, beta su...
K01610: phosphoenolpyruvate carboxykinase...
K02078: acyl carrier protein
K02919: large subunit ribosomal protein L36
K02931: large subunit ribosomal protein L5
K03073: preprotein translocase subunit SecE
K03088: RNA polymerase sigma−70 factor, E...
K04764: integration host factor subunit alpha
K06199: CrcB protein
K09802: hypothetical protein
Proteins
K00239: succinate dehydrogenase / fumarate...
K00262: glutamate dehydrogenase (NADP+) ...
K01610: phosphoenolpyruvate carboxykinase...
K01790: dTDP−4−dehydrorhamnose 3,5−epi...
K02358: elongation factor EF−Tu
K02935: large subunit ribosomal protein L7/L...
K04077: chaperonin GroEL
K10117: raffinose/stachyose/melibiose transp...
UNGROUPED
Taxonomy
PCo 1 (8.1%)
PCo 2 (6.7%)
Transcripts
PCo 1 (14.2%)
PCo 2 (9.2%)
Proteins
PCo 1 (30.8%)
PCo 2 (12.3%)
Metabolites
PCo 1 (8.8%)
PCo 2 (7.0%)

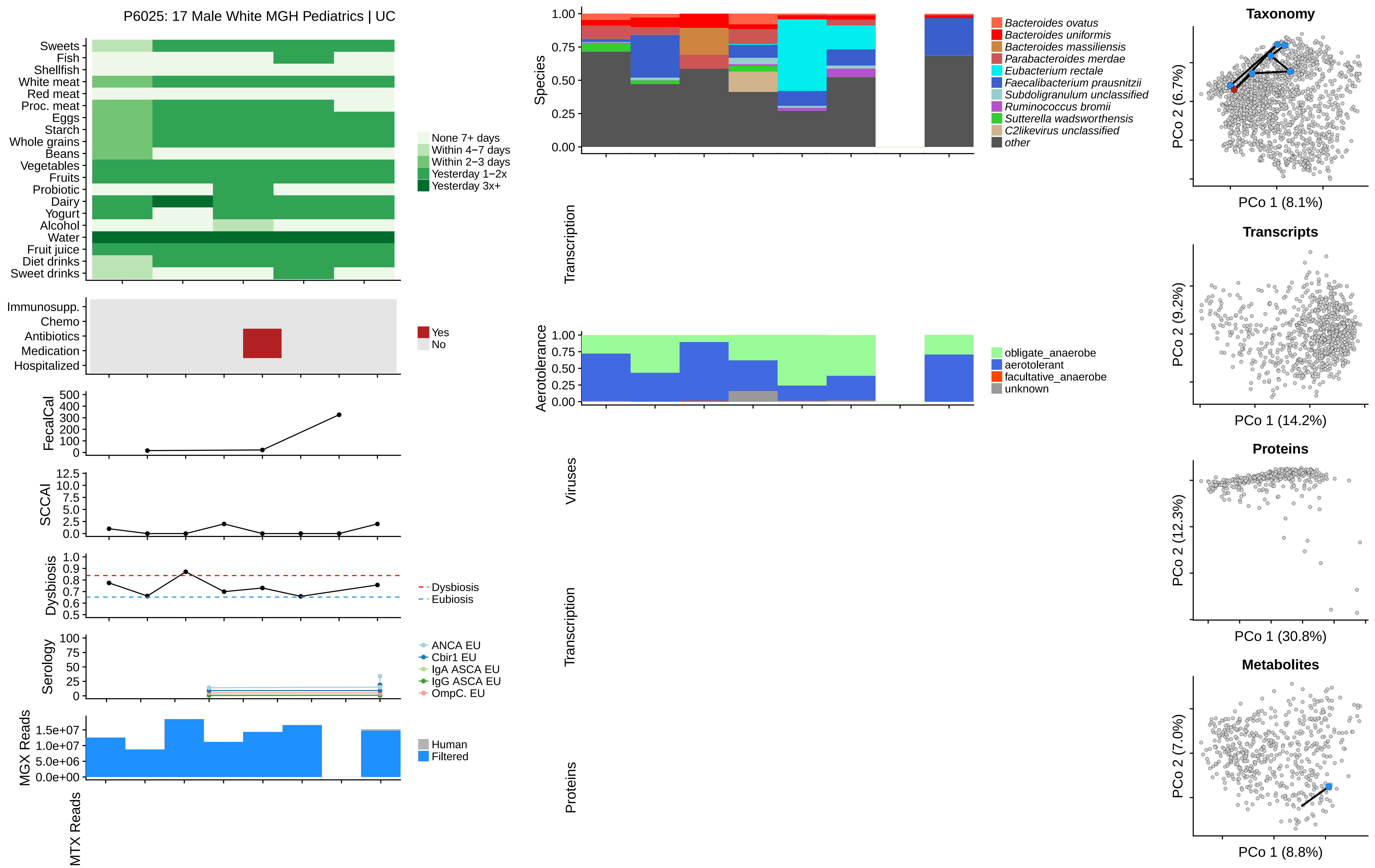
P6025: 17 Male White MGH Pediatrics | UC
Sweets
Fish
Shellfish
White meat
Red meat
Proc. meat
Eggs
Starch
Whole grains
Beans
Vegetables
Fruits
Probiotic
Dairy
Yogurt
Alcohol
Water
Fruit juice
Diet drinks
Sweet drinks
None 7+ days
Within 4−7 days
Within 2−3 days
Yesterday 1−2x
Yesterday 3x+
Immunosupp.
Chemo
Antibiotics
Medication
Hospitalized
Yes
No
FecalCal
SCCAI
Dysbiosis
Dysbiosis
Eubiosis
Serology
ANCA EU
Cbir1 EU
IgA ASCA EU
IgG ASCA EU
OmpC. EU
MGX Reads
Human
Filtered
MTX Reads
Species
Bacteroides ovatus
Bacteroides uniformis
Bacteroides massiliensis
Parabacteroides merdae
Eubacterium rectale
Faecalibacterium prausnitzii
Subdoligranulum unclassified
Ruminococcus bromii
Sutterella wadsworthensis
C2likevirus unclassified
other
Transcription
Aerotolerance
obligate_anaerobe
aerotolerant
facultative_anaerobe
unknown
Viruses
Transcription
Proteins
Taxonomy
PCo 1 (8.1%)
PCo 2 (6.7%)
Transcripts
PCo 1 (14.2%)
PCo 2 (9.2%)
Proteins
PCo 1 (30.8%)
PCo 2 (12.3%)
Metabolites
PCo 1 (8.8%)
PCo 2 (7.0%)

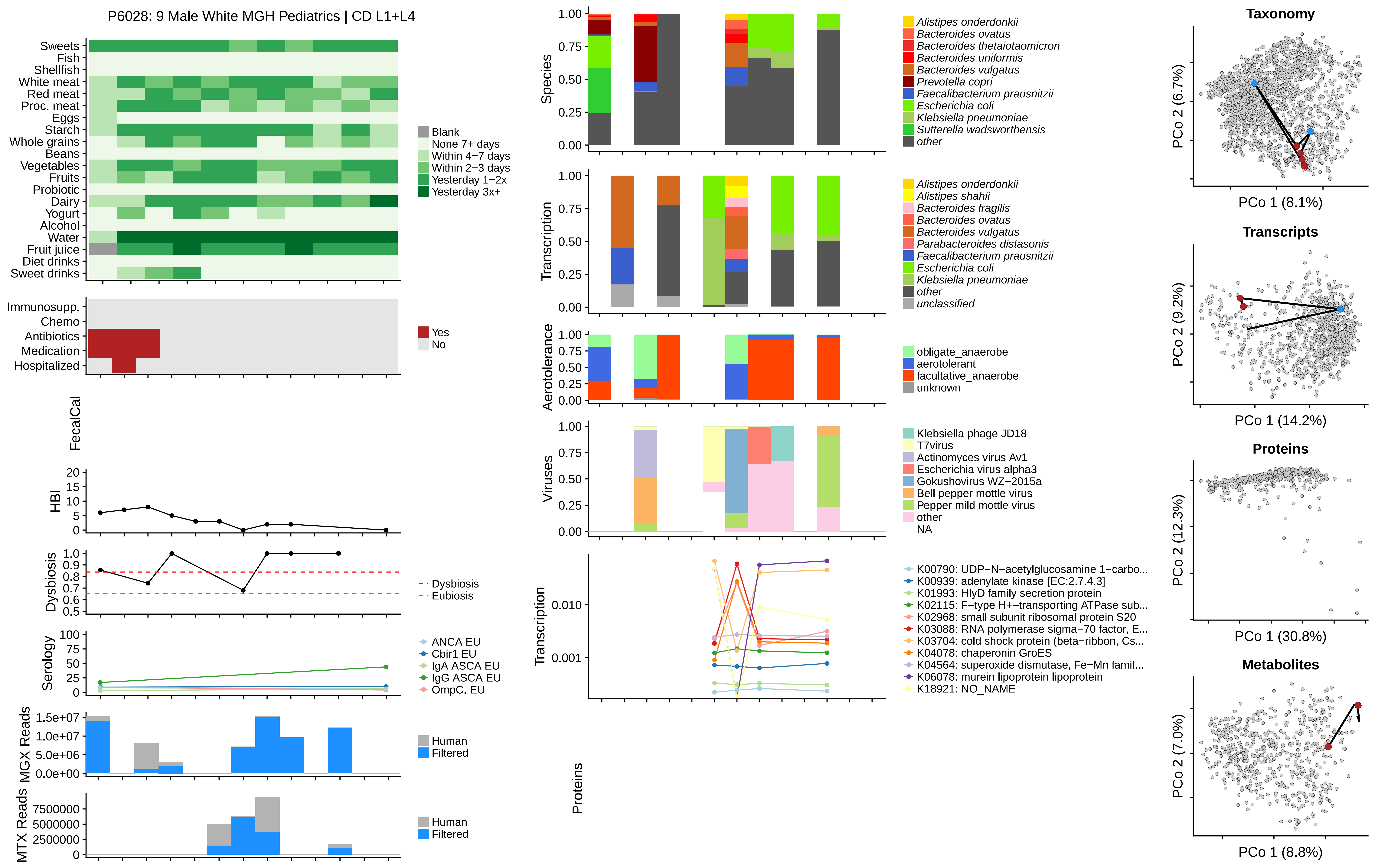

P6028: 9 Male White MGH Pediatrics | CD L1+L4
Sweets
Fish
Shellfish
White meat
Red meat
Proc. meat
Eggs
Starch
Whole grains
Beans
Vegetables
Fruits
Probiotic
Dairy
Yogurt
Alcohol
Water
Fruit juice
Diet drinks
Sweet drinks
Blank
None 7+ days
Within 4−7 days
Within 2−3 days
Yesterday 1−2x
Yesterday 3x+
Immunosupp.
Chemo
Antibiotics
Medication
Hospitalized
Yes
No
FecalCal
HBI
Dysbiosis
Dysbiosis
Eubiosis
Serology
ANCA EU
Cbir1 EU
IgA ASCA EU
IgG ASCA EU
OmpC. EU
MGX Reads
Human
Filtered
MTX Reads
Human
Filtered
Species
Alistipes onderdonkii
Bacteroides ovatus
Bacteroides thetaiotaomicron
Bacteroides uniformis
Bacteroides vulgatus
Prevotella copri
Faecalibacterium prausnitzii
Escherichia coli
Klebsiella pneumoniae
Sutterella wadsworthensis
other
Transcription
Alistipes onderdonkii
Alistipes shahii
Bacteroides fragilis
Bacteroides ovatus
Bacteroides vulgatus
Parabacteroides distasonis
Faecalibacterium prausnitzii
Escherichia coli
Klebsiella pneumoniae
other
unclassified
Aerotolerance
obligate_anaerobe
aerotolerant
facultative_anaerobe
unknown
Viruses
Klebsiella phage JD18
T7virus
Actinomyces virus Av1
Escherichia virus alpha3
Gokushovirus WZ−2015a
Bell pepper mottle virus
Pepper mild mottle virus
other
NA
Transcription
K00790: UDP−N−acetylglucosamine 1−carbo...
K00939: adenylate kinase [EC:2.7.4.3]
K01993: HlyD family secretion protein
K02115: F−type H+−transporting ATPase sub...
K02968: small subunit ribosomal protein S20
K03088: RNA polymerase sigma−70 factor, E...
K03704: cold shock protein (beta−ribbon, Cs...
K04078: chaperonin GroES
K04564: superoxide dismutase, Fe−Mn famil...
K06078: murein lipoprotein lipoprotein
K18921: NO_NAME
Proteins
Taxonomy
PCo 1 (8.1%)
PCo 2 (6.7%)
Transcripts
PCo 1 (14.2%)
PCo 2 (9.2%)
Proteins
PCo 1 (30.8%)
PCo 2 (12.3%)
Metabolites
PCo 1 (8.8%)
PCo 2 (7.0%)

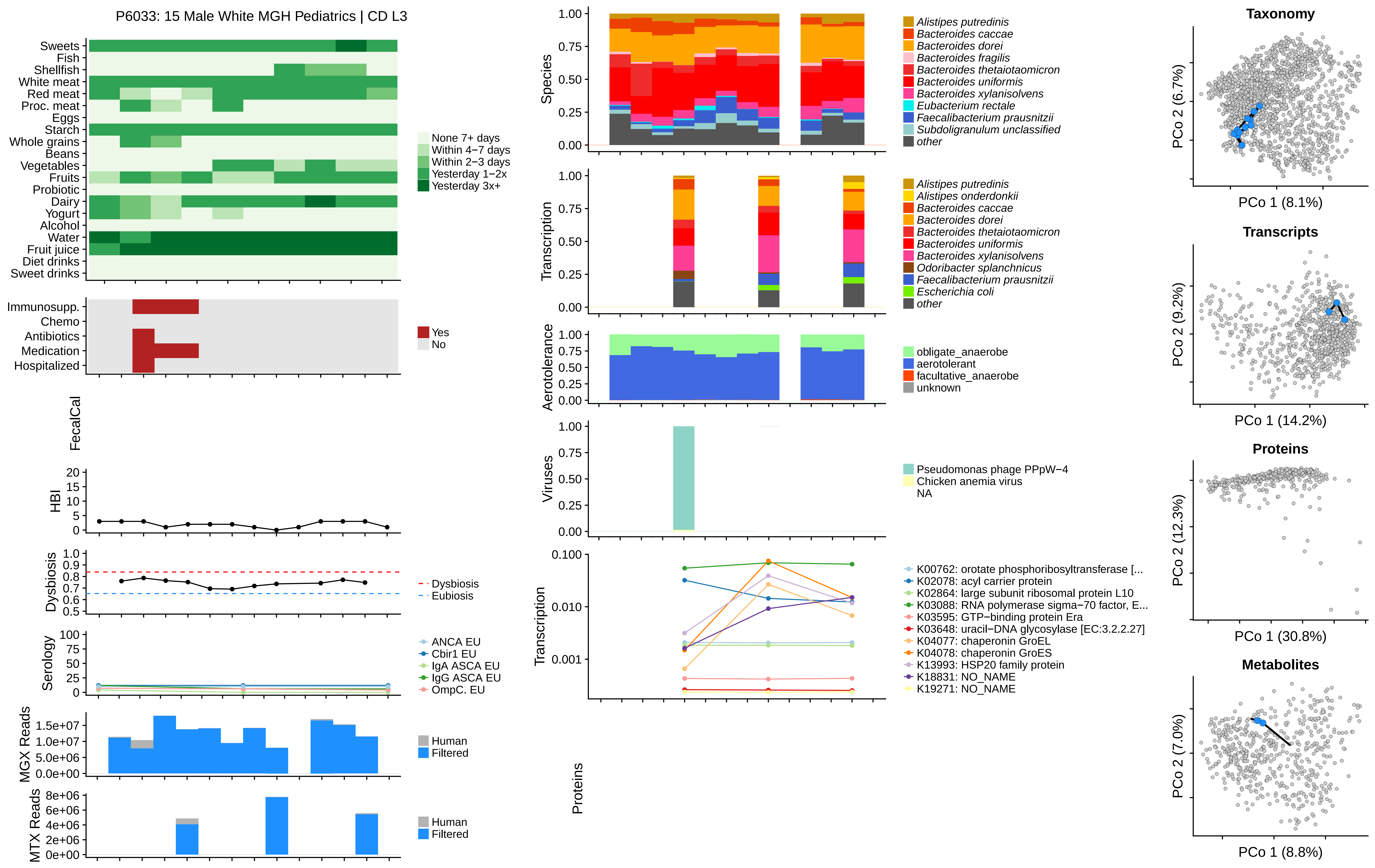
P6033: 15 Male White MGH Pediatrics | CD L3
Sweets
Fish
Shellfish
White meat
Red meat
Proc. meat
Eggs
Starch
Whole grains
Beans
Vegetables
Fruits
Probiotic
Dairy
Yogurt
Alcohol
Water
Fruit juice
Diet drinks
Sweet drinks
None 7+ days
Within 4−7 days
Within 2−3 days
Yesterday 1−2x
Yesterday 3x+
Immunosupp.
Chemo
Antibiotics
Medication
Hospitalized
Yes
No
FecalCal
HBI
Dysbiosis
Dysbiosis
Eubiosis
Serology
ANCA EU
Cbir1 EU
IgA ASCA EU
IgG ASCA EU
OmpC. EU
MGX Reads
Human
Filtered
MTX Reads
Human
Filtered
Species
Alistipes putredinis
Bacteroides caccae
Bacteroides dorei
Bacteroides fragilis
Bacteroides thetaiotaomicron
Bacteroides uniformis
Bacteroides xylanisolvens
Eubacterium rectale
Faecalibacterium prausnitzii
Subdoligranulum unclassified
other
Transcription
Alistipes putredinis
Alistipes onderdonkii
Bacteroides caccae
Bacteroides dorei
Bacteroides thetaiotaomicron
Bacteroides uniformis
Bacteroides xylanisolvens
Odoribacter splanchnicus
Faecalibacterium prausnitzii
Escherichia coli
other
Aerotolerance
obligate_anaerobe
aerotolerant
facultative_anaerobe
unknown
Viruses
Pseudomonas phage PPpW−4
Chicken anemia virus
NA
Transcription
K00762: orotate phosphoribosyltransferase [...
K02078: acyl carrier protein
K02864: large subunit ribosomal protein L10
K03088: RNA polymerase sigma−70 factor, E...
K03595: GTP−binding protein Era
K03648: uracil−DNA glycosylase [EC:3.2.2.27]
K04077: chaperonin GroEL
K04078: chaperonin GroES
K13993: HSP20 family protein
K18831: NO_NAME
K19271: NO_NAME
Proteins
Taxonomy
PCo 1 (8.1%)
PCo 2 (6.7%)
Transcripts
PCo 1 (14.2%)
PCo 2 (9.2%)
Proteins
PCo 1 (30.8%)
PCo 2 (12.3%)
Metabolites
PCo 1 (8.8%)
PCo 2 (7.0%)

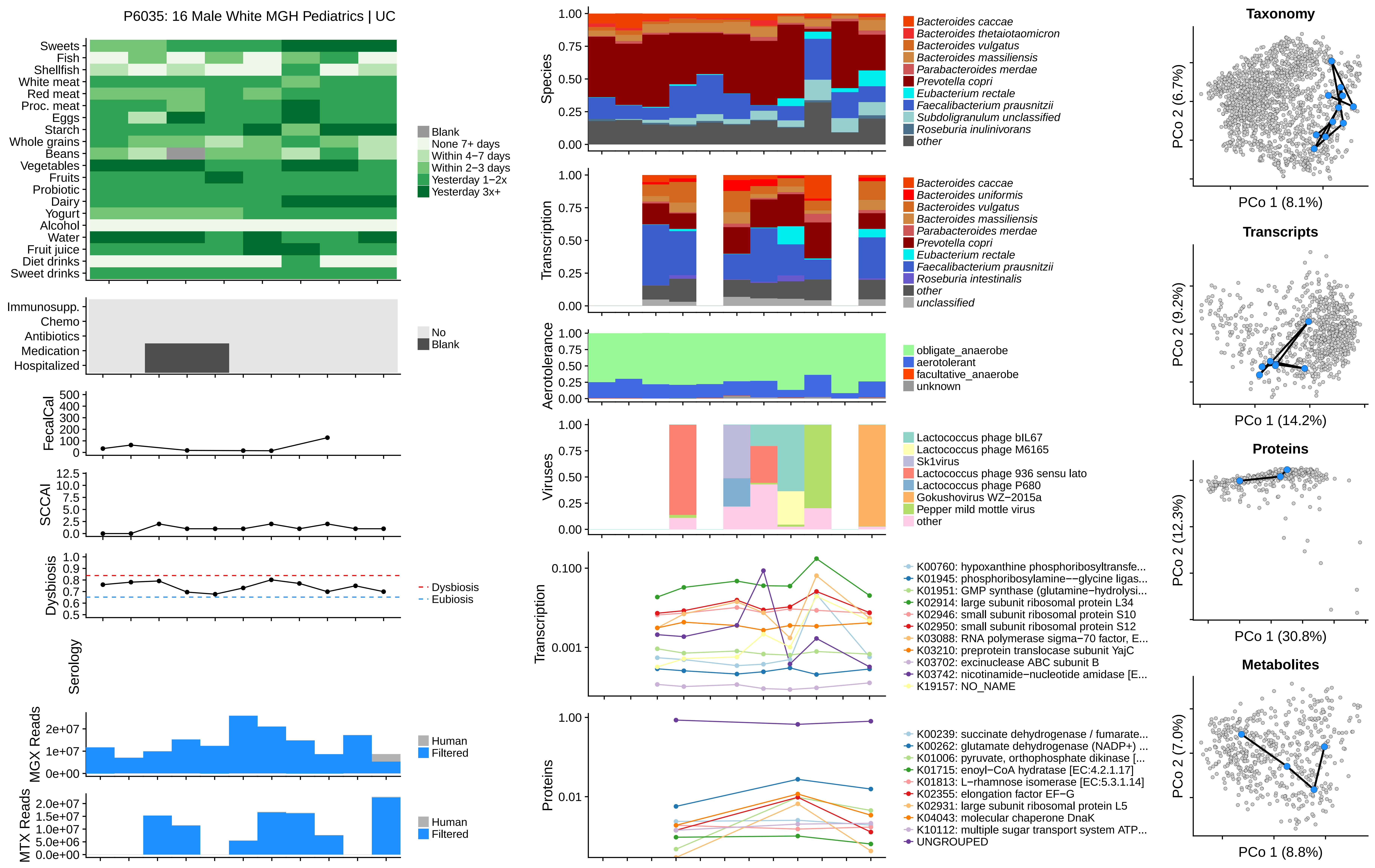

P6035: 16 Male White MGH Pediatrics | UC
Sweets
Fish
Shellfish
White meat
Red meat
Proc. meat
Eggs
Starch
Whole grains
Beans
Vegetables
Fruits
Probiotic
Dairy
Yogurt
Alcohol
Water
Fruit juice
Diet drinks
Sweet drinks
Blank
None 7+ days
Within 4−7 days
Within 2−3 days
Yesterday 1−2x
Yesterday 3x+
Immunosupp.
Chemo
Antibiotics
Medication
Hospitalized
No
Blank
FecalCal
500
400
300
200
100
0
SCCAI
12.5
10.0
7.5
5.0
2.5
0.0
Dysbiosis
1.0
0.9
0.8
0.7
0.6
0.5
Dysbiosis
Eubiosis
Serology
MGX Reads
2e+07
1e+07
0e+00
Human
Filtered
MTX Reads
2.0e+07
1.5e+07
1.0e+07
5.0e+06
0.0e+00
Human
Filtered
Species
1.00
0.75
0.50
0.25
0.00
Bacteroides caccae
Bacteroides thetaiotaomicron
Bacteroides vulgatus
Bacteroides massiliensis
Parabacteroides merdae
Prevotella copri
Eubacterium rectale
Faecalibacterium prausnitzii
Subdoligranulum unclassified
Roseburia inulinivorans
other
Transcription
Bacteroides caccae
Bacteroides uniformis
Bacteroides vulgatus
Bacteroides massiliensis
Parabacteroides merdae
Prevotella copri
Eubacterium rectale
Faecalibacterium prausnitzii
Roseburia intestinalis
other
unclassified
Aerotolerance
obligate_anaerobe
aerotolerant
facultative_anaerobe
unknown
Viruses
Lactococcus phage bIL67
Lactococcus phage M6165
Sk1virus
Lactococcus phage 936 sensu lato
Lactococcus phage P680
Gokushovirus WZ−2015a
Pepper mild mottle virus
other
Transcription
0.100
0.001
K00760: hypoxanthine phosphoribosyltransfe...
K01945: phosphoribosylamine−−glycine ligas...
K01951: GMP synthase (glutamine−hydrolysi...
K02914: large subunit ribosomal protein L34
K02946: small subunit ribosomal protein S10
K02950: small subunit ribosomal protein S12
K03088: RNA polymerase sigma−70 factor, E...
K03210: preprotein translocase subunit YajC
K03702: excinuclease ABC subunit B
K03742: nicotinamide−nucleotide amidase [E...
K19157: NO_NAME
Proteins
1.00
0.01
K00239: succinate dehydrogenase / fumarate...
K00262: glutamate dehydrogenase (NADP+) ...
K01006: pyruvate, orthophosphate dikinase [...
K01715: enoyl−CoA hydratase [EC:4.2.1.17]
K01813: L−rhamnose isomerase [EC:5.3.1.14]
K02355: elongation factor EF−G
K02931: large subunit ribosomal protein L5
K04043: molecular chaperone DnaK
K10112: multiple sugar transport system ATP...
UNGROUPED
Taxonomy
PCo 2 (6.7%)
PCo 1 (8.1%)
Transcripts
PCo 2 (9.2%)
PCo 1 (14.2%)
Proteins
PCo 2 (12.3%)
PCo 1 (30.8%)
Metabolites
PCo 2 (7.0%)
PCo 1 (8.8%)

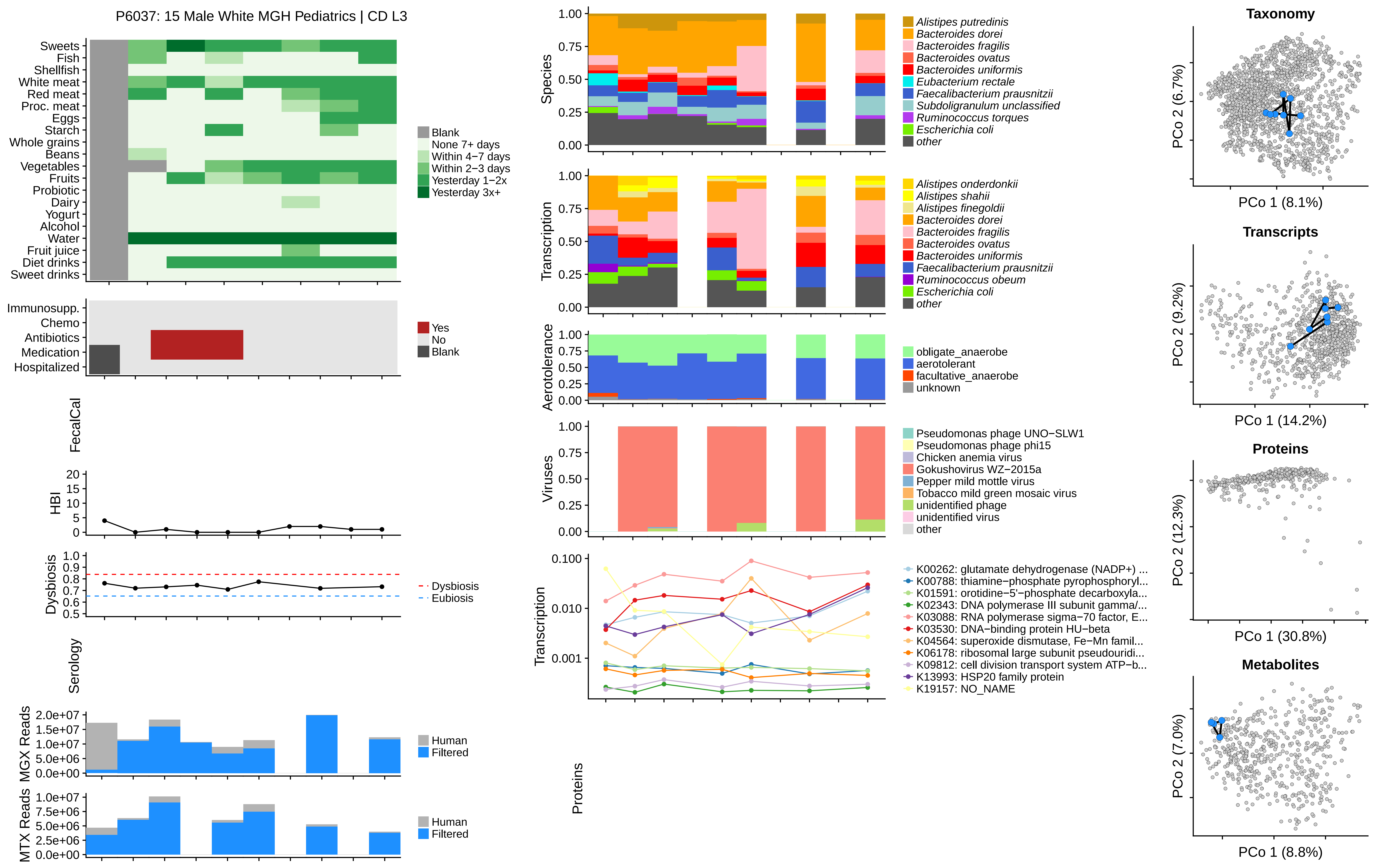
P6037: 15 Male White MGH Pediatrics | CD L3
Sweets
Fish
Shellfish
White meat
Red meat
Proc. meat
Eggs
Starch
Whole grains
Beans
Vegetables
Fruits
Probiotic
Dairy
Yogurt
Alcohol
Water
Fruit juice
Diet drinks
Sweet drinks
Blank
None 7+ days
Within 4−7 days
Within 2−3 days
Yesterday 1−2x
Yesterday 3x+
Immunosupp.
Chemo
Antibiotics
Medication
Hospitalized
Yes
No
Blank
FecalCal
HBI
Dysbiosis
Dysbiosis
Eubiosis
Serology
MGX Reads
Human
Filtered
MTX Reads
Human
Filtered
Species
Alistipes putredinis
Bacteroides dorei
Bacteroides fragilis
Bacteroides ovatus
Bacteroides uniformis
Eubacterium rectale
Faecalibacterium prausnitzii
Subdoligranulum unclassified
Ruminococcus torques
Escherichia coli
other
Transcription
Alistipes onderdonkii
Alistipes shahii
Alistipes finegoldii
Bacteroides dorei
Bacteroides fragilis
Bacteroides ovatus
Bacteroides uniformis
Faecalibacterium prausnitzii
Ruminococcus obeum
Escherichia coli
other
Aerotolerance
obligate_anaerobe
aerotolerant
facultative_anaerobe
unknown
Viruses
Pseudomonas phage UNO−SLW1
Pseudomonas phage phi15
Chicken anemia virus
Gokushovirus WZ−2015a
Pepper mild mottle virus
Tobacco mild green mosaic virus
unidentified phage
unidentified virus
other
Transcription
K00262: glutamate dehydrogenase (NADP+) ...
K00788: thiamine−phosphate pyrophosphoryl...
K01591: orotidine−5'−phosphate decarboxyla...
K02343: DNA polymerase III subunit gamma/...
K03088: RNA polymerase sigma−70 factor, E...
K03530: DNA−binding protein HU−beta
K04564: superoxide dismutase, Fe−Mn famil...
K06178: ribosomal large subunit pseudouridi...
K09812: cell division transport system ATP−b...
K13993: HSP20 family protein
K19157: NO_NAME
Proteins
Taxonomy
PCo 1 (8.1%)
PCo 2 (6.7%)
Transcripts
PCo 1 (14.2%)
PCo 2 (9.2%)
Proteins
PCo 1 (30.8%)
PCo 2 (12.3%)
Metabolites
PCo 1 (8.8%)
PCo 2 (7.0%)

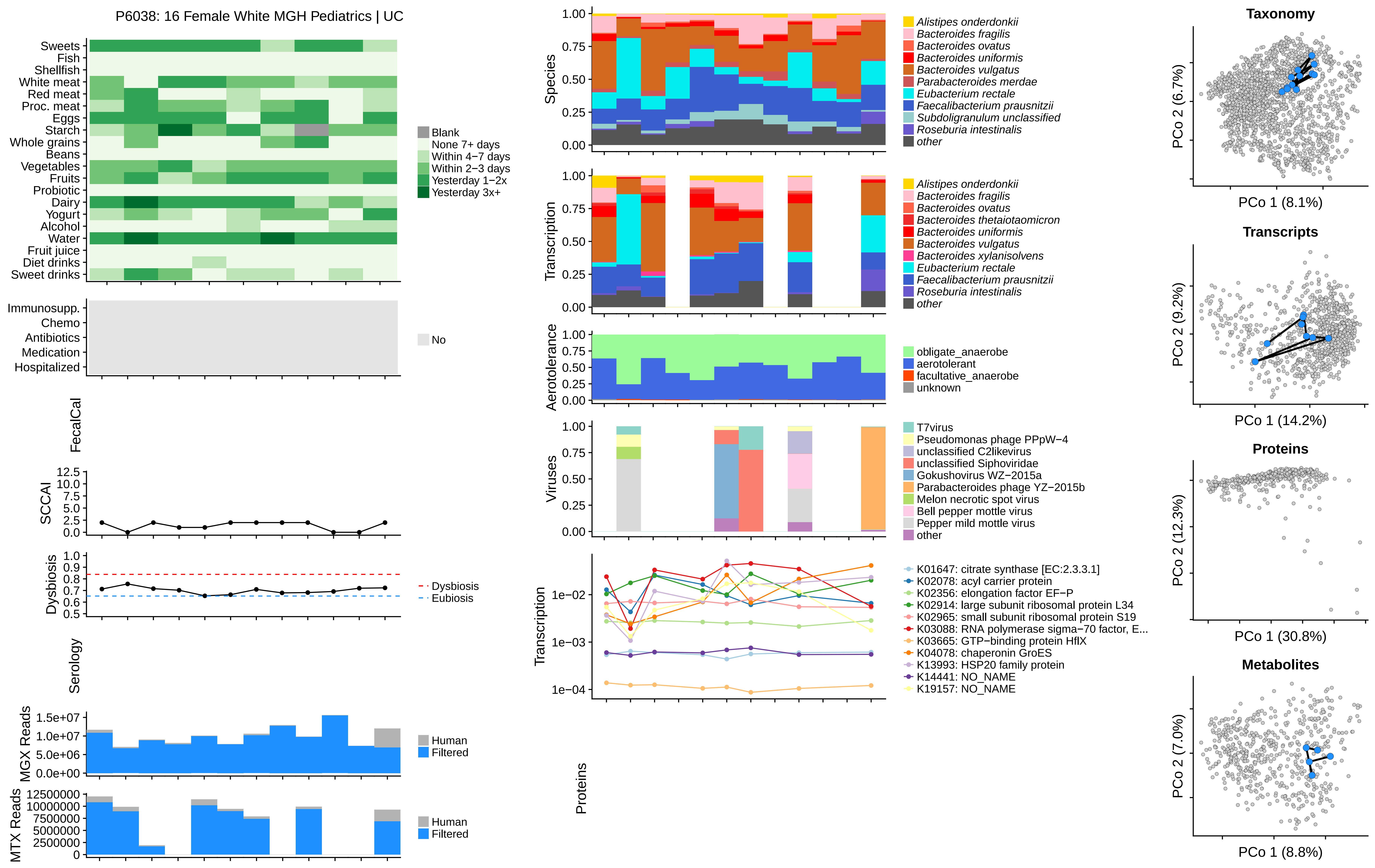

P6038: 16 Female White MGH Pediatrics | UC
Sweets
Fish
Shellfish
White meat
Red meat
Proc. meat
Eggs
Starch
Whole grains
Beans
Vegetables
Fruits
Probiotic
Dairy
Yogurt
Alcohol
Water
Fruit juice
Diet drinks
Sweet drinks
Blank
None 7+ days
Within 4−7 days
Within 2−3 days
Yesterday 1−2x
Yesterday 3x+
Immunosupp.
Chemo
Antibiotics
Medication
Hospitalized
No
FecalCal
SCCAI
Dysbiosis
Dysbiosis
Eubiosis
Serology
MGX Reads
MTX Reads
Human
Filtered
Species
Alistipes onderdonkii
Bacteroides fragilis
Bacteroides ovatus
Bacteroides uniformis
Bacteroides vulgatus
Parabacteroides merdae
Eubacterium rectale
Faecalibacterium prausnitzii
Subdoligranulum unclassified
Roseburia intestinalis
other
Transcription
Bacteroides thetaiotaomicron
Bacteroides xylanisolvens
Aerotolerance
obligate_anaerobe
aerotolerant
facultative_anaerobe
unknown
Viruses
T7virus
Pseudomonas phage PPpW−4
unclassified C2likevirus
unclassified Siphoviridae
Gokushovirus WZ−2015a
Parabacteroides phage YZ−2015b
Melon necrotic spot virus
Bell pepper mottle virus
Pepper mild mottle virus
K01647: citrate synthase [EC:2.3.3.1]
K02078: acyl carrier protein
K02356: elongation factor EF−P
K02914: large subunit ribosomal protein L34
K02965: small subunit ribosomal protein S19
K03088: RNA polymerase sigma−70 factor, E...
K03665: GTP−binding protein HflX
K04078: chaperonin GroES
K13993: HSP20 family protein
K14441: NO_NAME
K19157: NO_NAME
Proteins
Taxonomy
PCo 1 (8.1%)
PCo 2 (6.7%)
Transcripts
PCo 1 (14.2%)
PCo 2 (9.2%)
Proteins
PCo 1 (30.8%)
PCo 2 (12.3%)
Metabolites
PCo 1 (8.8%)
PCo 2 (7.0%)
