# Supplementary material for: Multi-omics of the gut microbial ecosystem in inflammatory bowel diseases
Source: Nature. 2019 May 29;569(7758):655–62. doi: 10.1038/s41586-019-1237-9 (PMC6650278; doi:10.1038/s41586-019-1237-9)
Supplement: Supplementary file 5 — The distribution of each associated metadatum is shown across subjects (“per Participant ID”), biosamples (“per site_sub_coll”), and generated profiles (“per row”). Numeric fields are displayed as histograms. [file 41586_2019_1237_MOESM5_ESM.pdf]

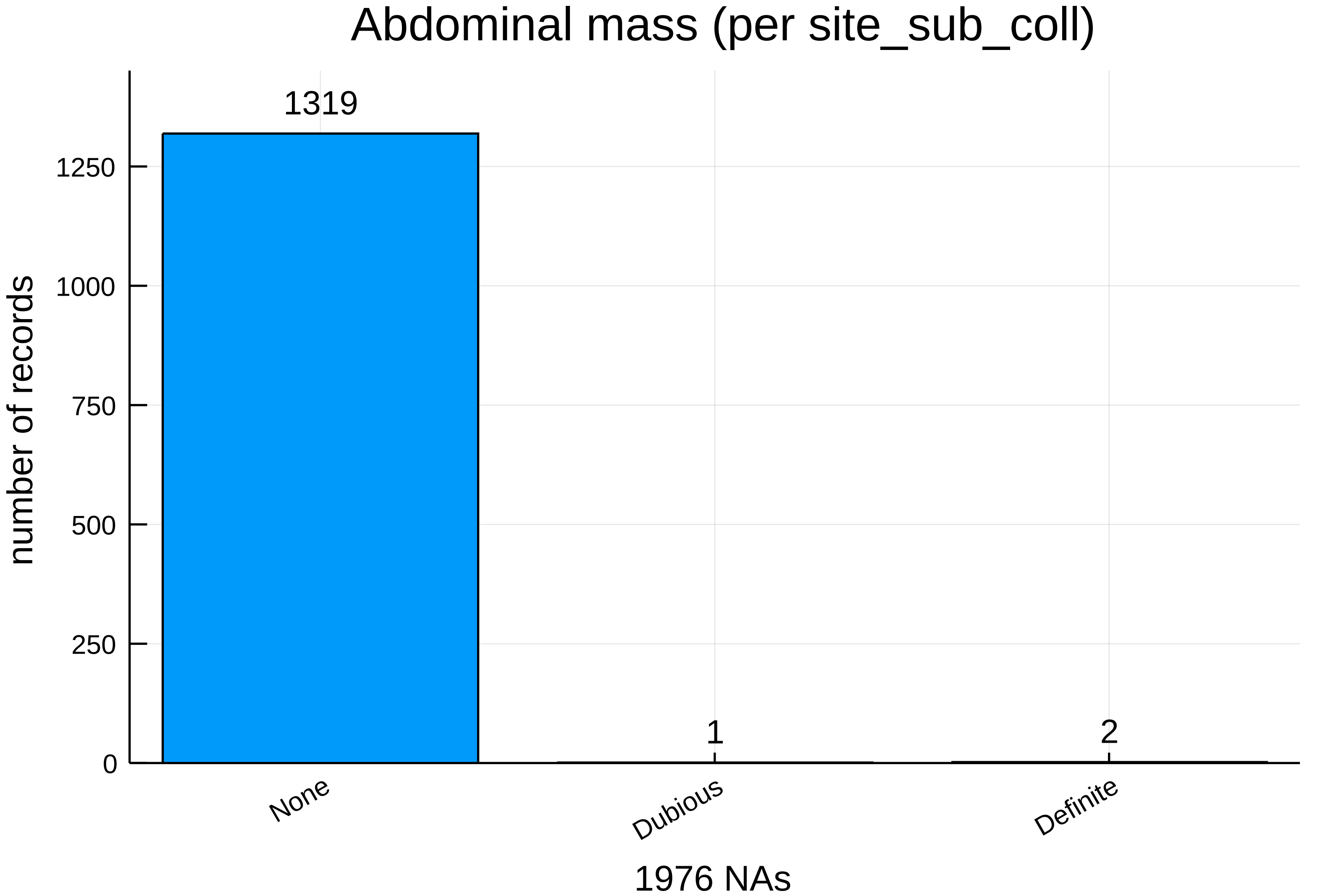
Abdominal mass (per site_sub_coll)
number of records
1319
1
2
0
250
500
750
1000
1250
None
Dubious
Definite
1976 NAs

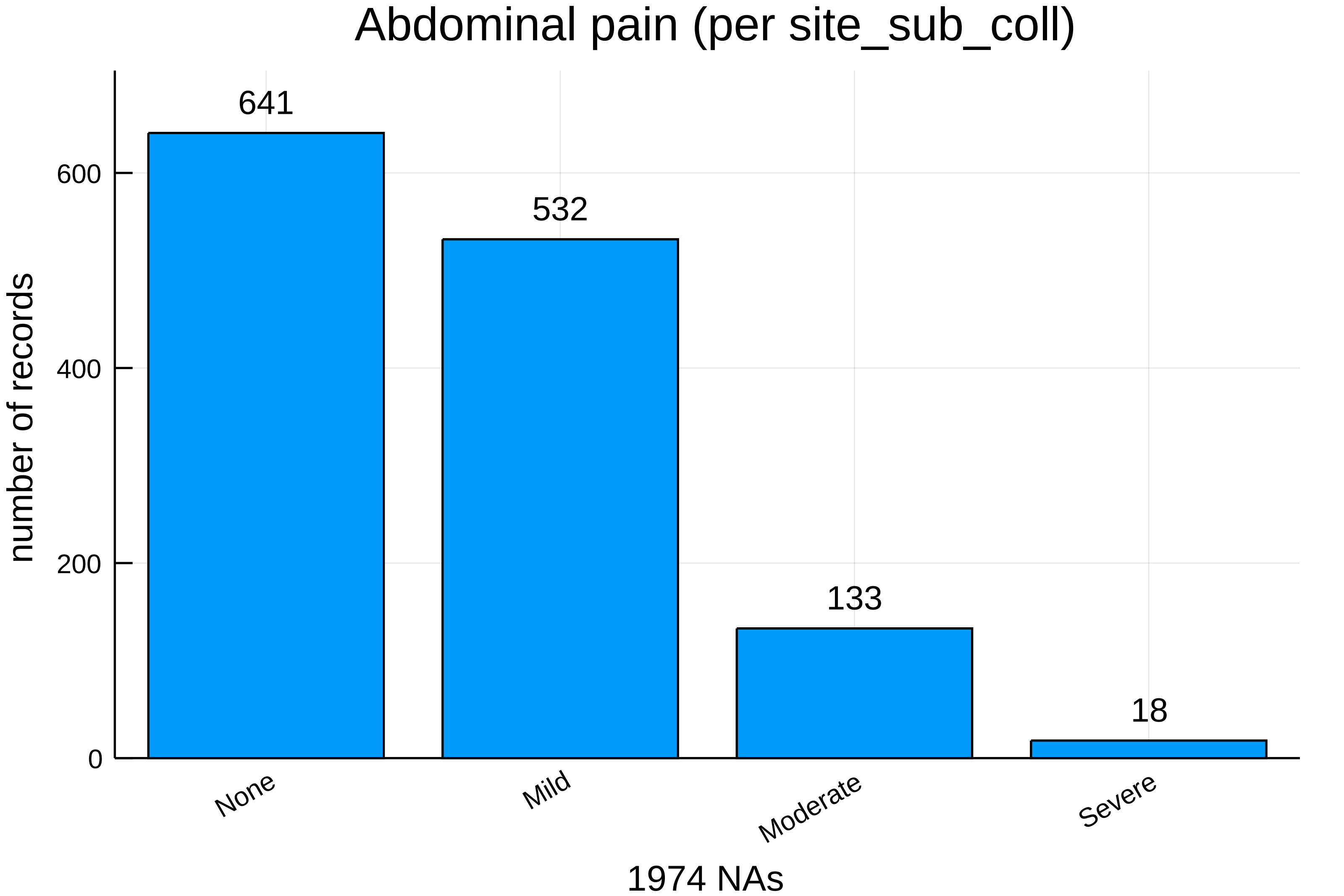
Abdominal pain (per site_sub_coll)
number of records
0
200
400
600
641
532
133
18
None
Mild
Moderate
Severe
1974 NAs

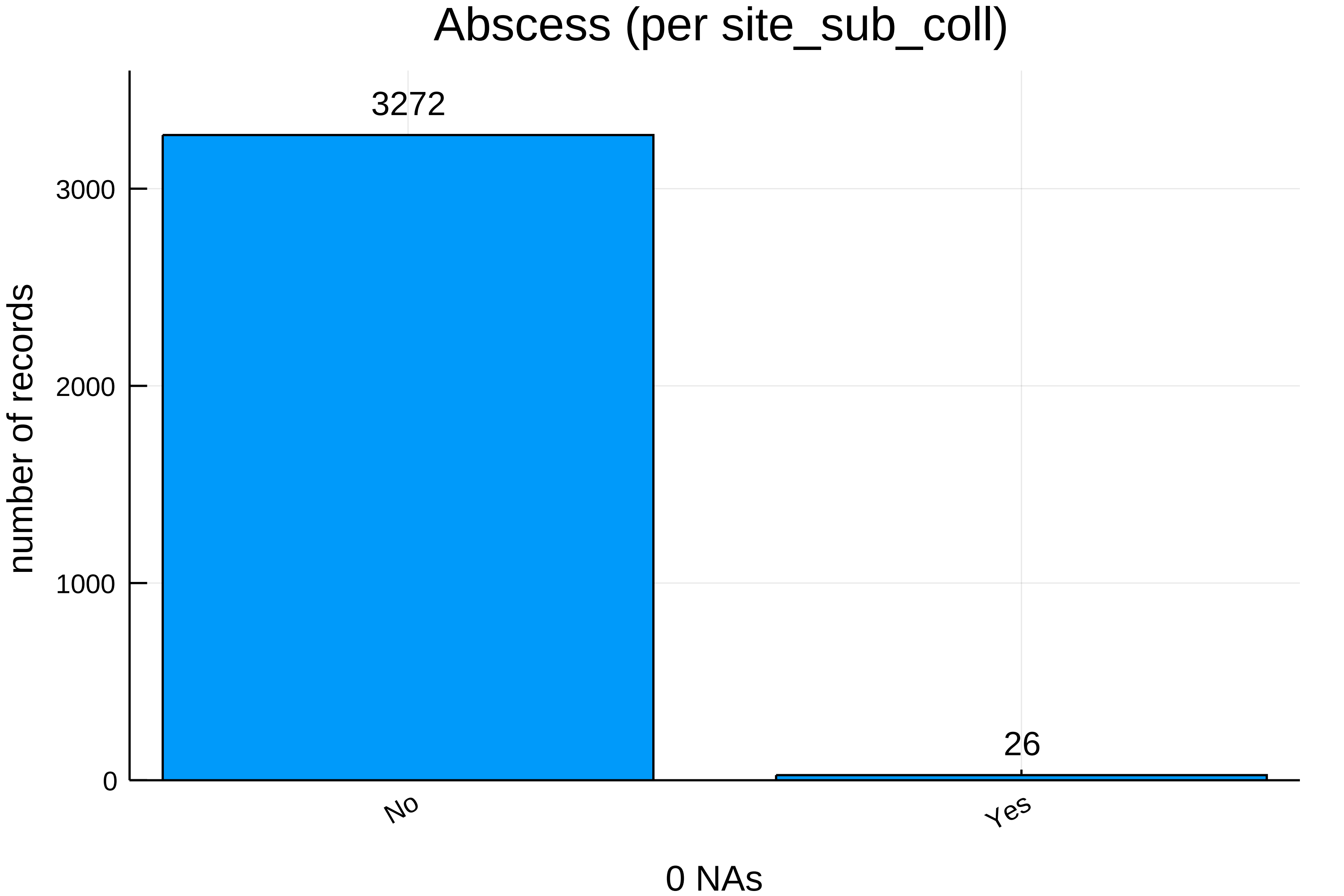
Abscess (per site_sub_coll)
number of records
3272
26
3000
2000
1000
0
No
Yes
0 NAs

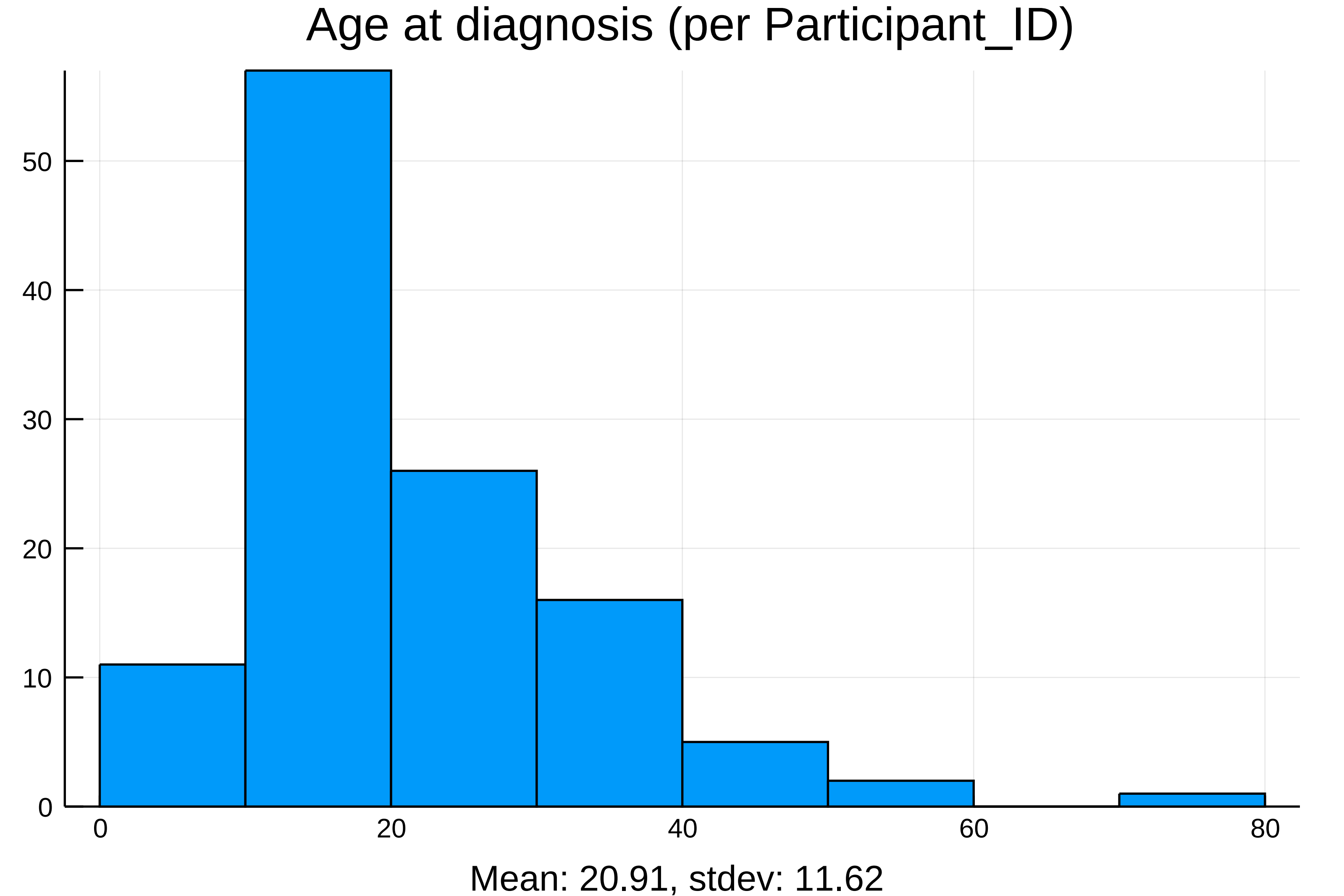
Age at diagnosis (per Participant_ID)
50
40
30
20
10
0
0
20
40
60
80
Mean: 20.91, stdev: 11.62

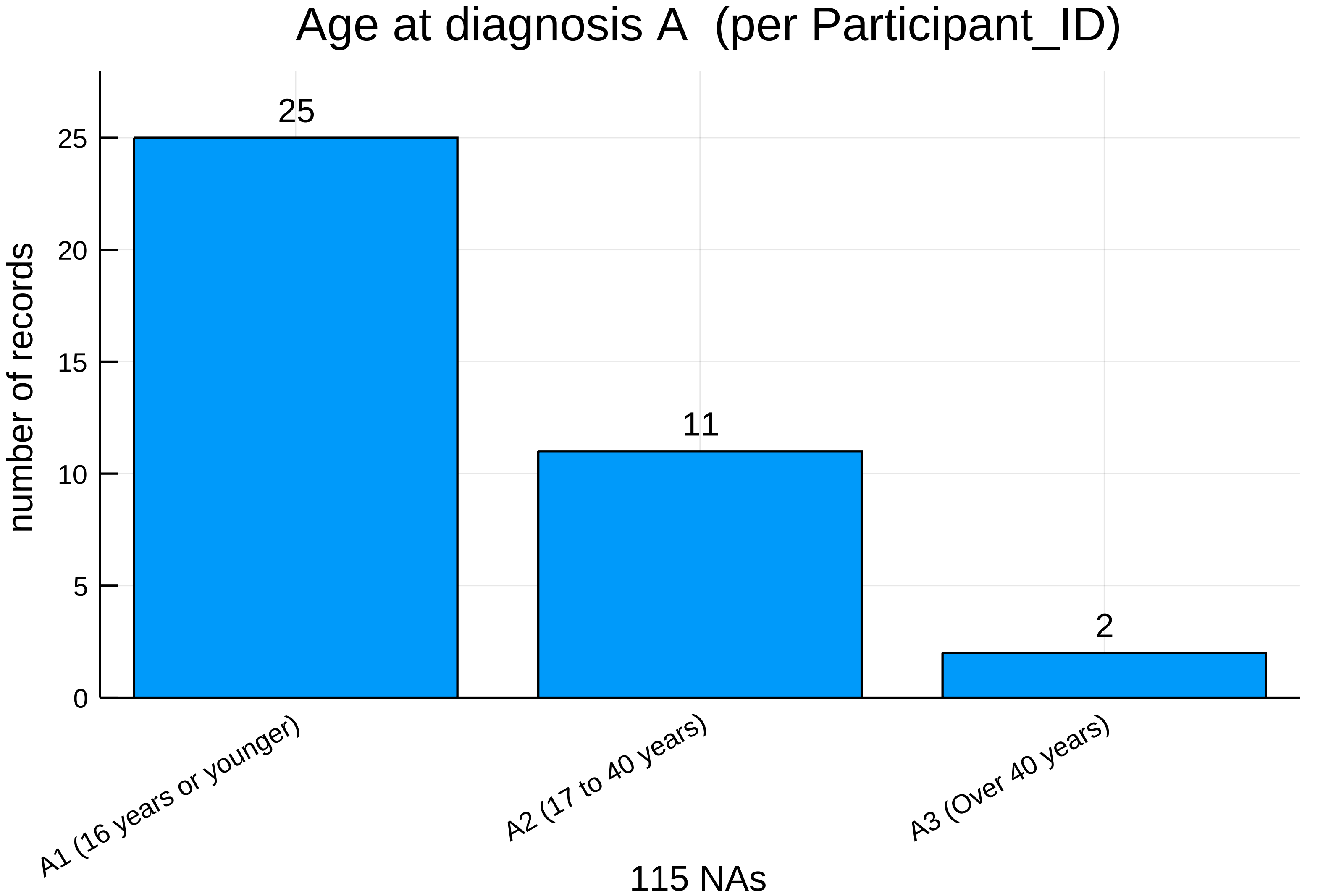
Age at diagnosis A (per Participant_ID)
number of records
25
11
2
0
5
10
15
20
25
A1 (16 years or younger)
A2 (17 to 40 years)
A3 (Over 40 years)
115 NAs

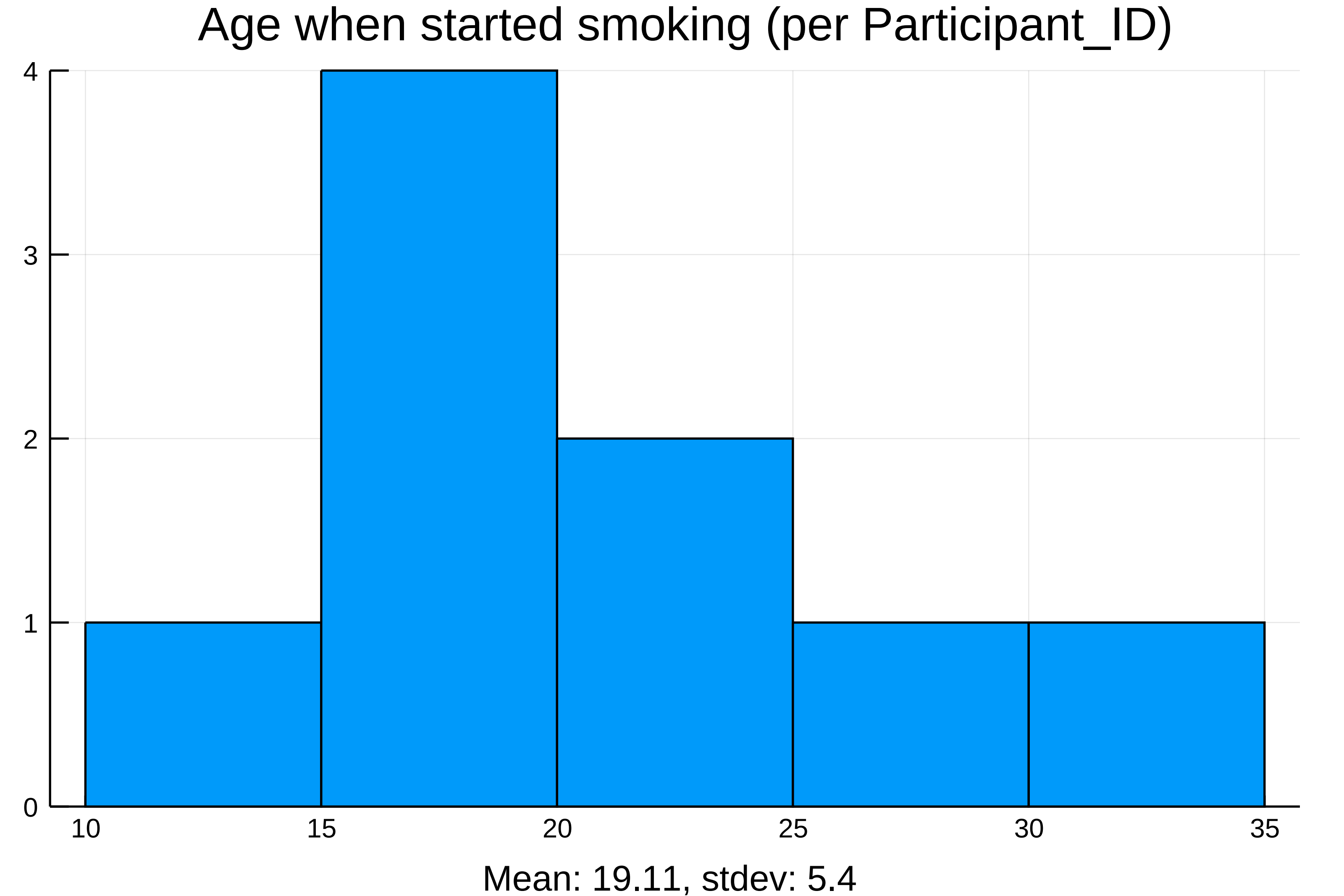
Age when started smoking (per Participant_ID)
0
1
2
3
4
10
15
20
25
30
35
Mean: 19.11, stdev: 5.4

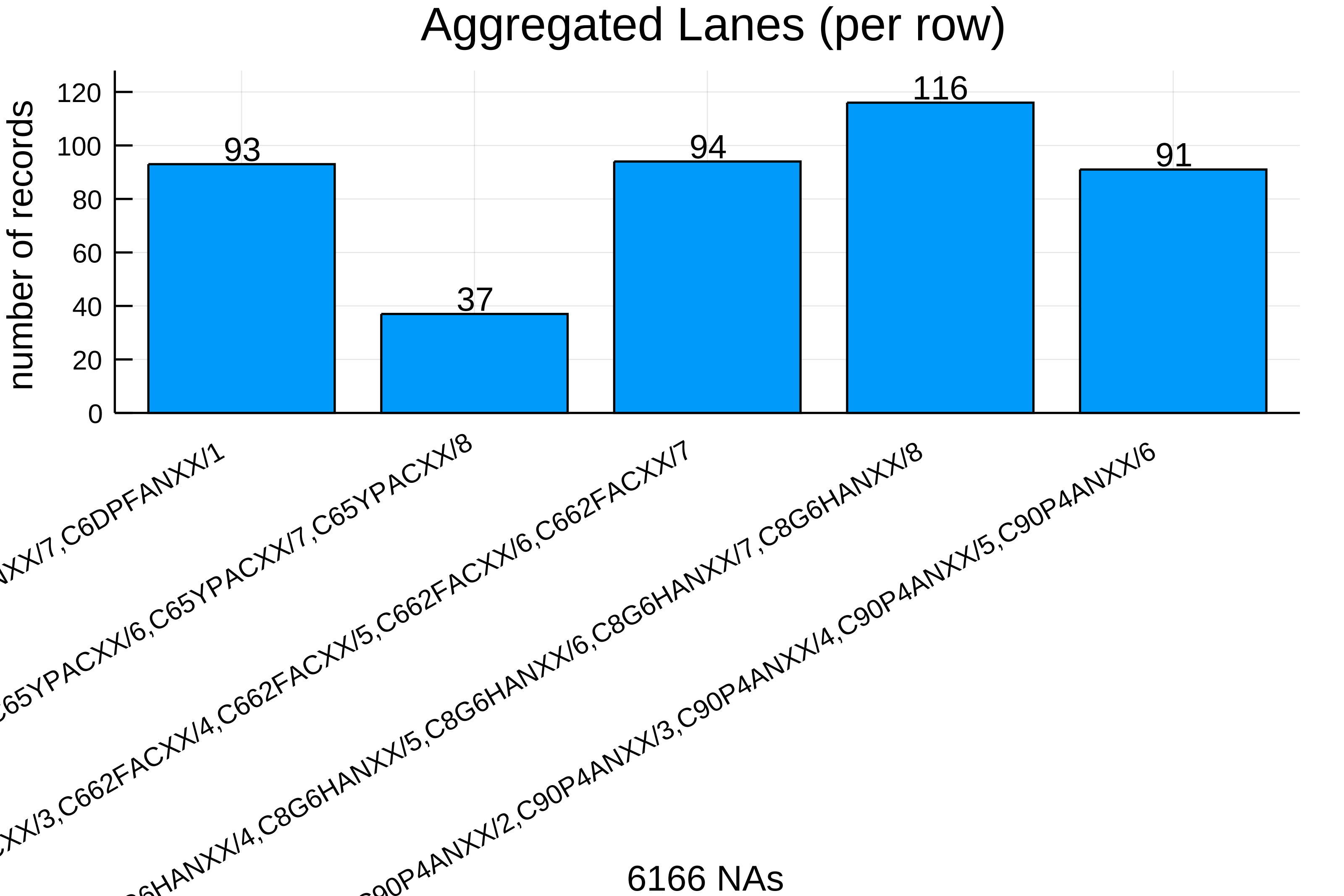

Aggregated Lanes (per row)
number of records
0
20
40
60
80
100
120
93
37
94
116
91
6166 NAs

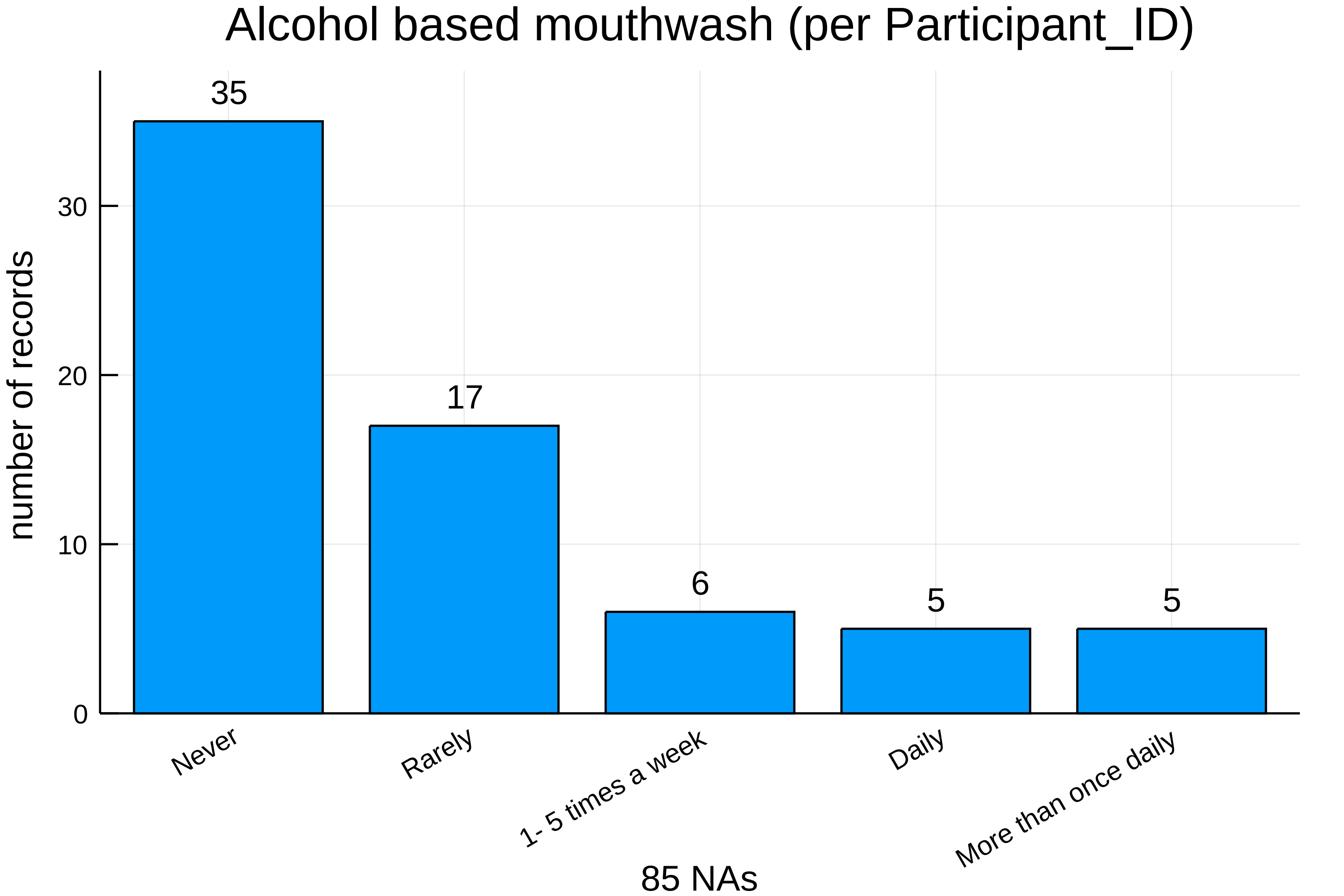
Alcohol based mouthwash (per Participant_ID)
number of records
0
10
20
30
35
17
6
5
5
Never
Rarely
1- 5 times a week
Daily
More than once daily
85 NAs

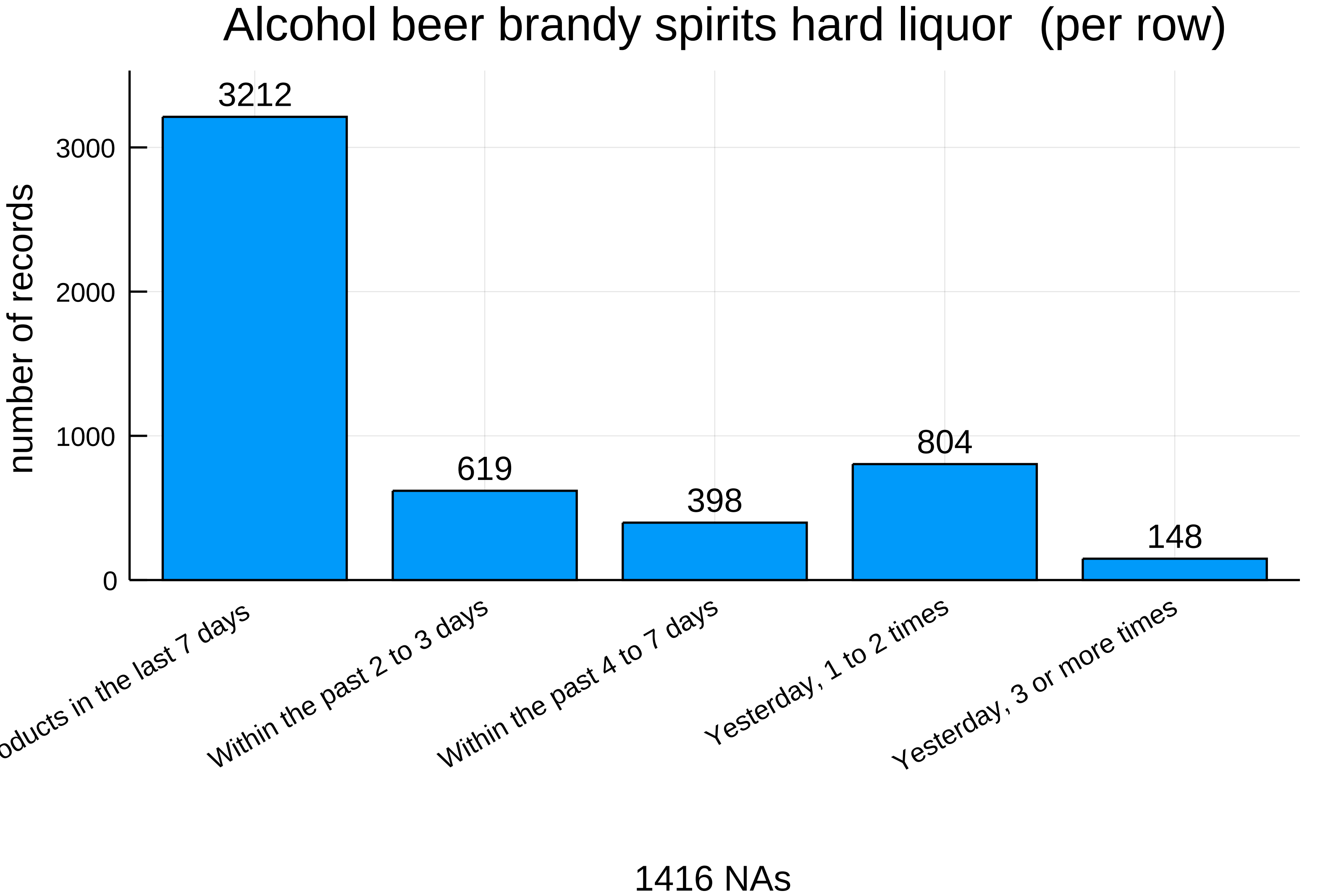

Alcohol beer brandy spirits hard liquor (per row)
number of records
0
1000
2000
3000
3212
619
398
804
148
oducts in the last 7 days
Within the past 2 to 3 days
Within the past 4 to 7 days
Yesterday, 1 to 2 times
Yesterday, 3 or more times
1416 NAs

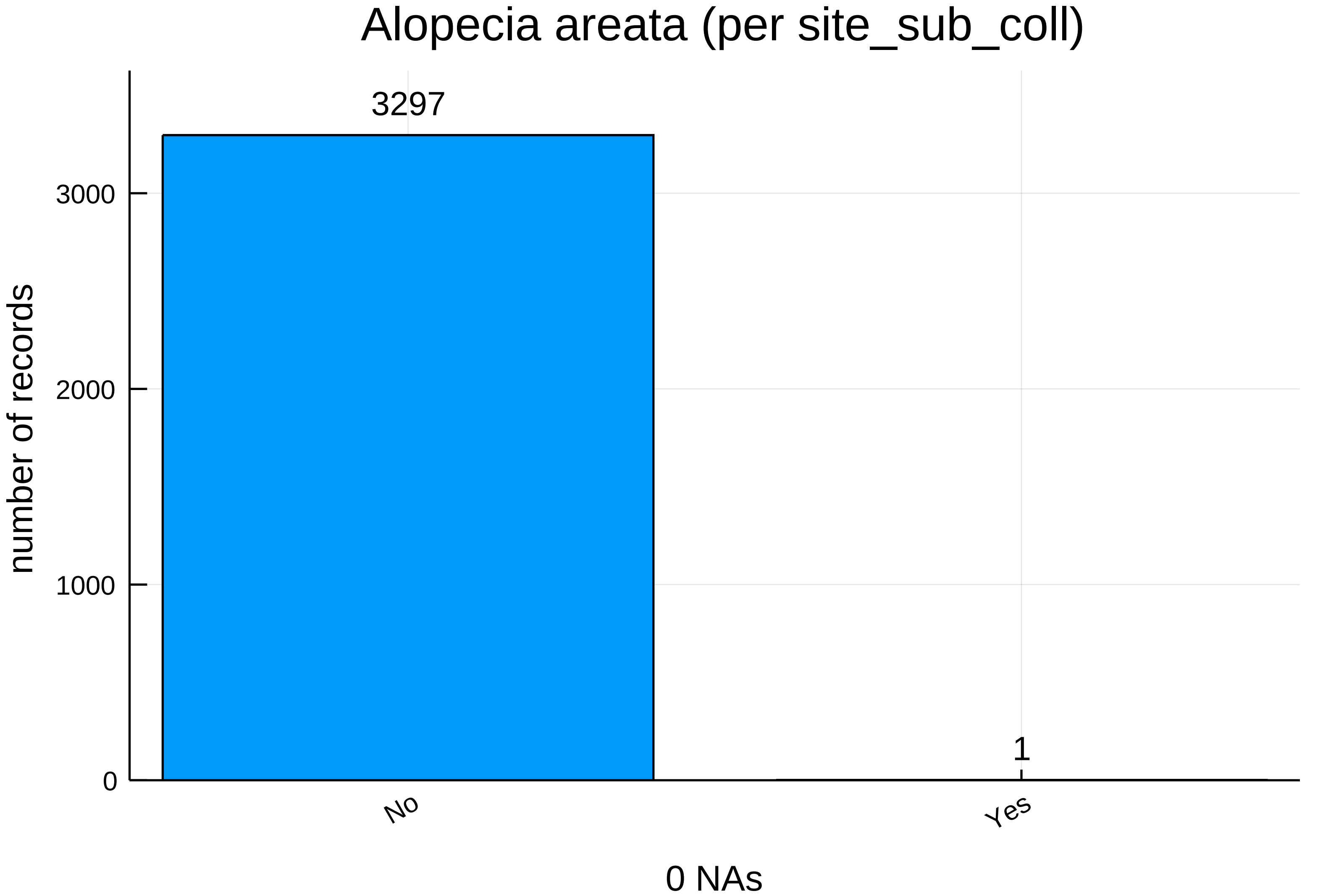
Alopecia areata (per site_sub_coll)
3297
1
3000
2000
1000
0
number of records
No
Yes
0 NAs

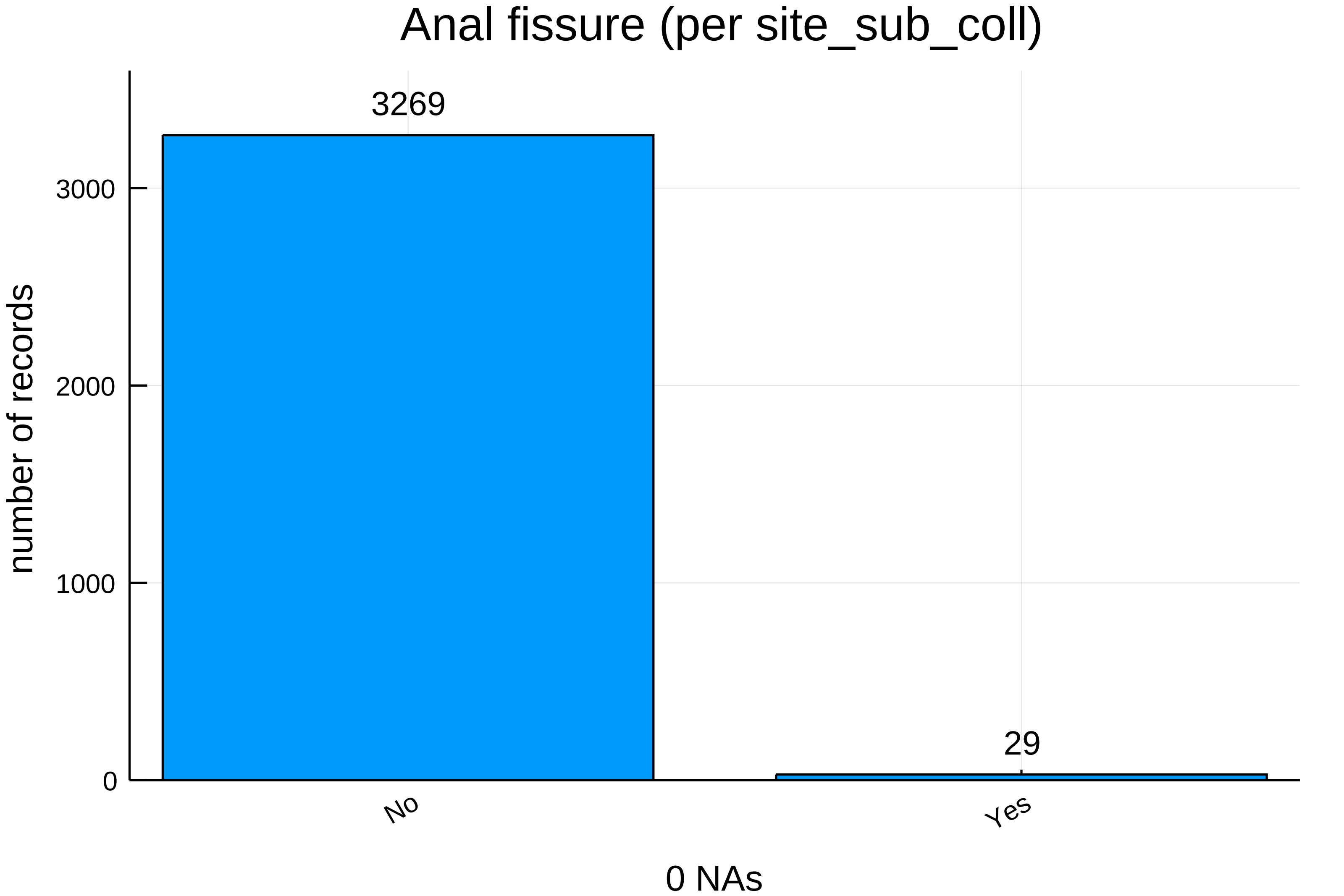
Anal fissure (per site_sub_coll)
number of records
3269
29
3000
2000
1000
0
No
Yes
0 NAs

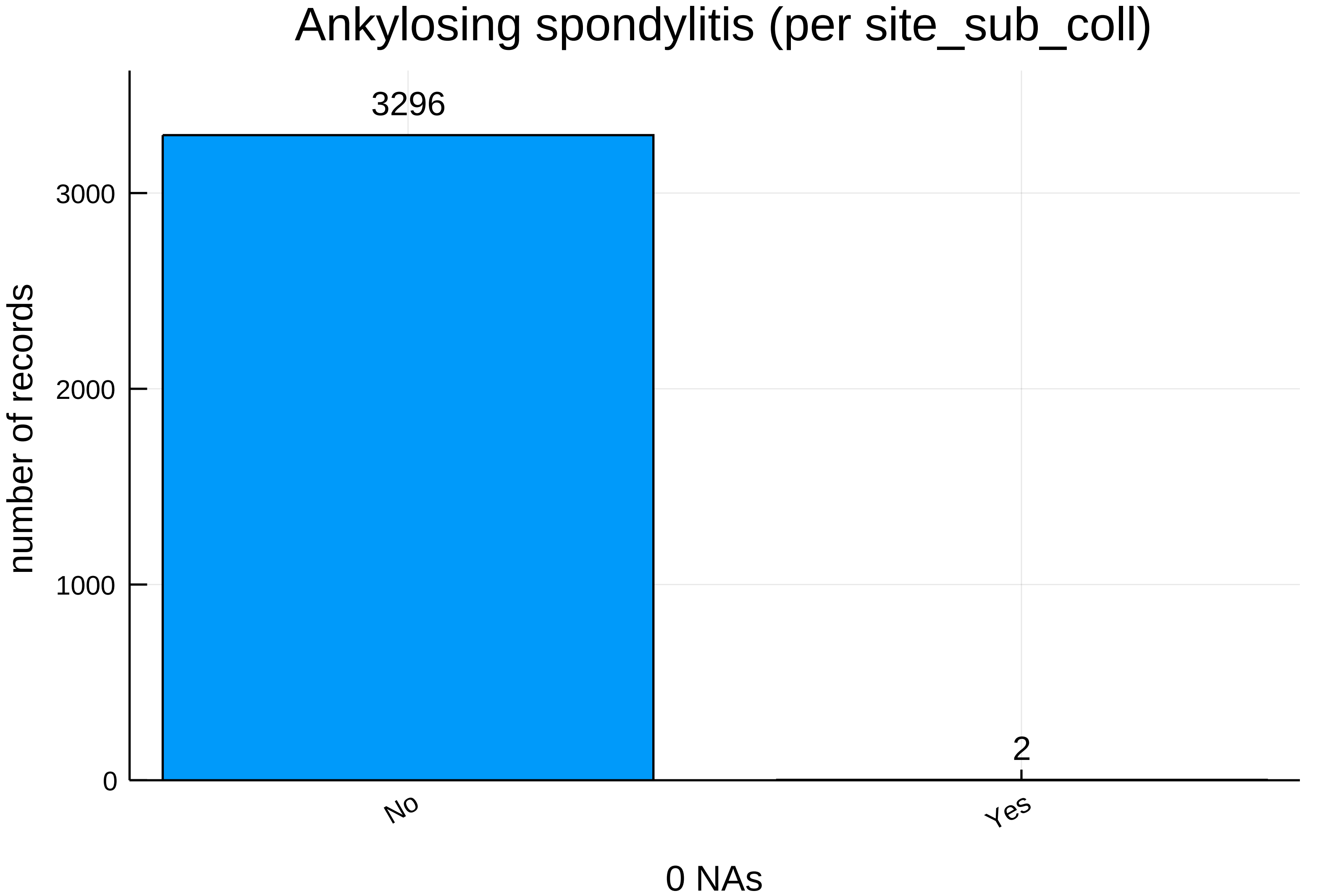
Ankylosing spondylitis (per site_sub_coll)
number of records
3000
2000
1000
0
3296
2
No
Yes
0 NAs

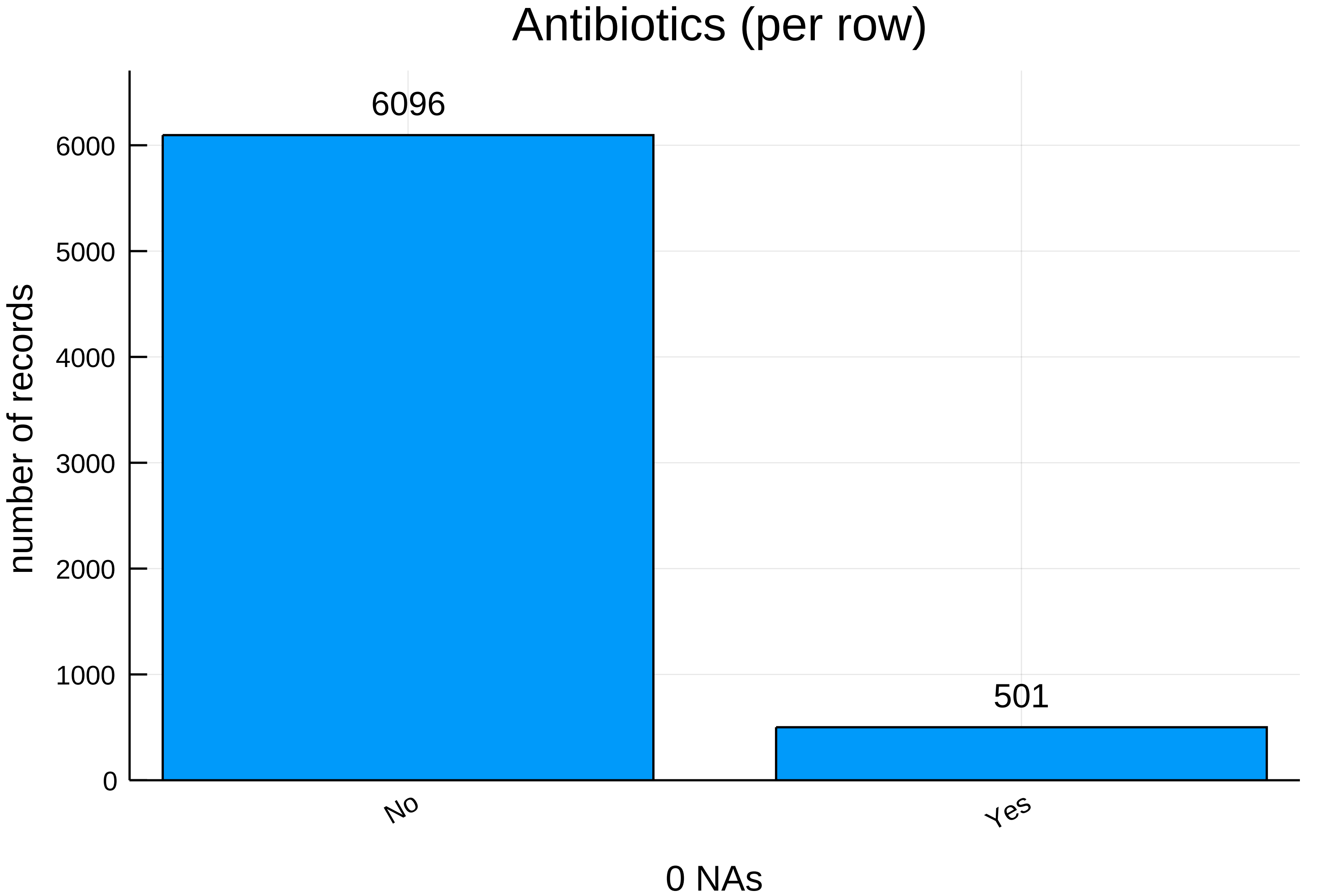
Antibiotics (per row)
number of records
6096
501
6000
5000
4000
3000
2000
1000
0
No
Yes
0 NAs

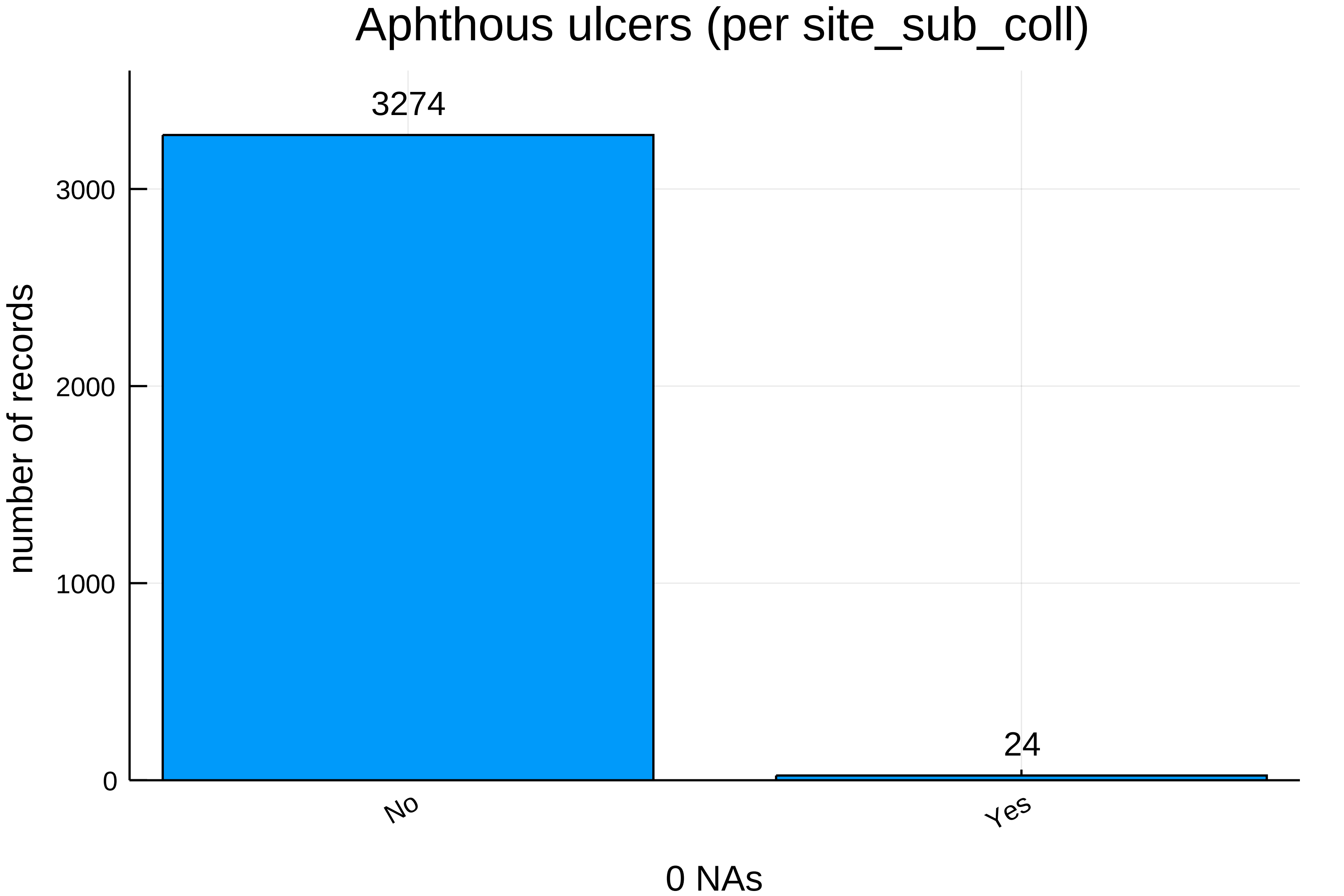
Aphthous ulcers (per site_sub_coll)
number of records
3000
2000
1000
0
3274
24
No
Yes
0 NAs

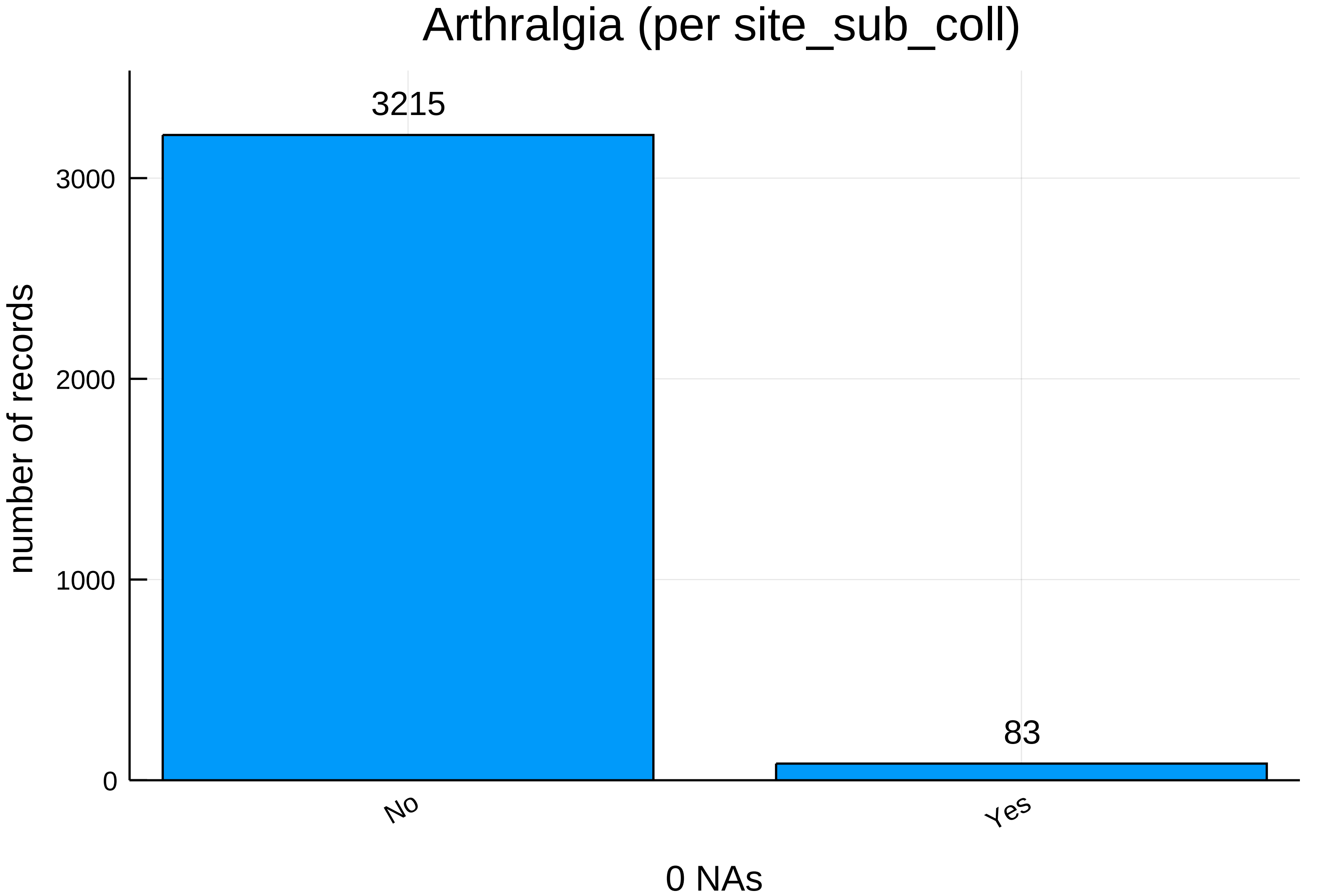
Arthralgia (per site_sub_coll)
number of records
3000
2000
1000
0
3215
83
No
Yes
0 NAs

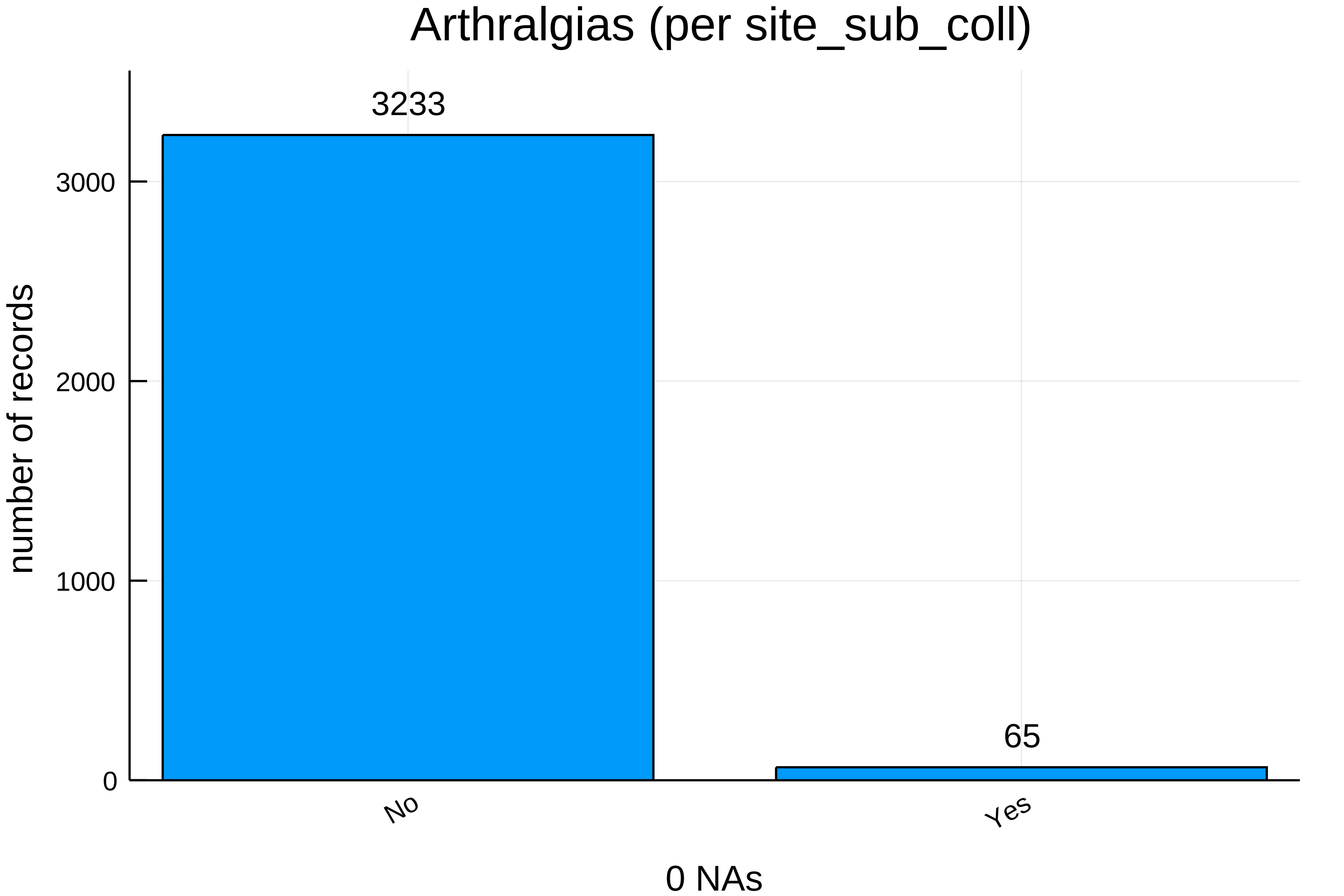
Arthralgias (per site_sub_coll)
number of records
3233
65
3000
2000
1000
0
No
Yes
0 NAs

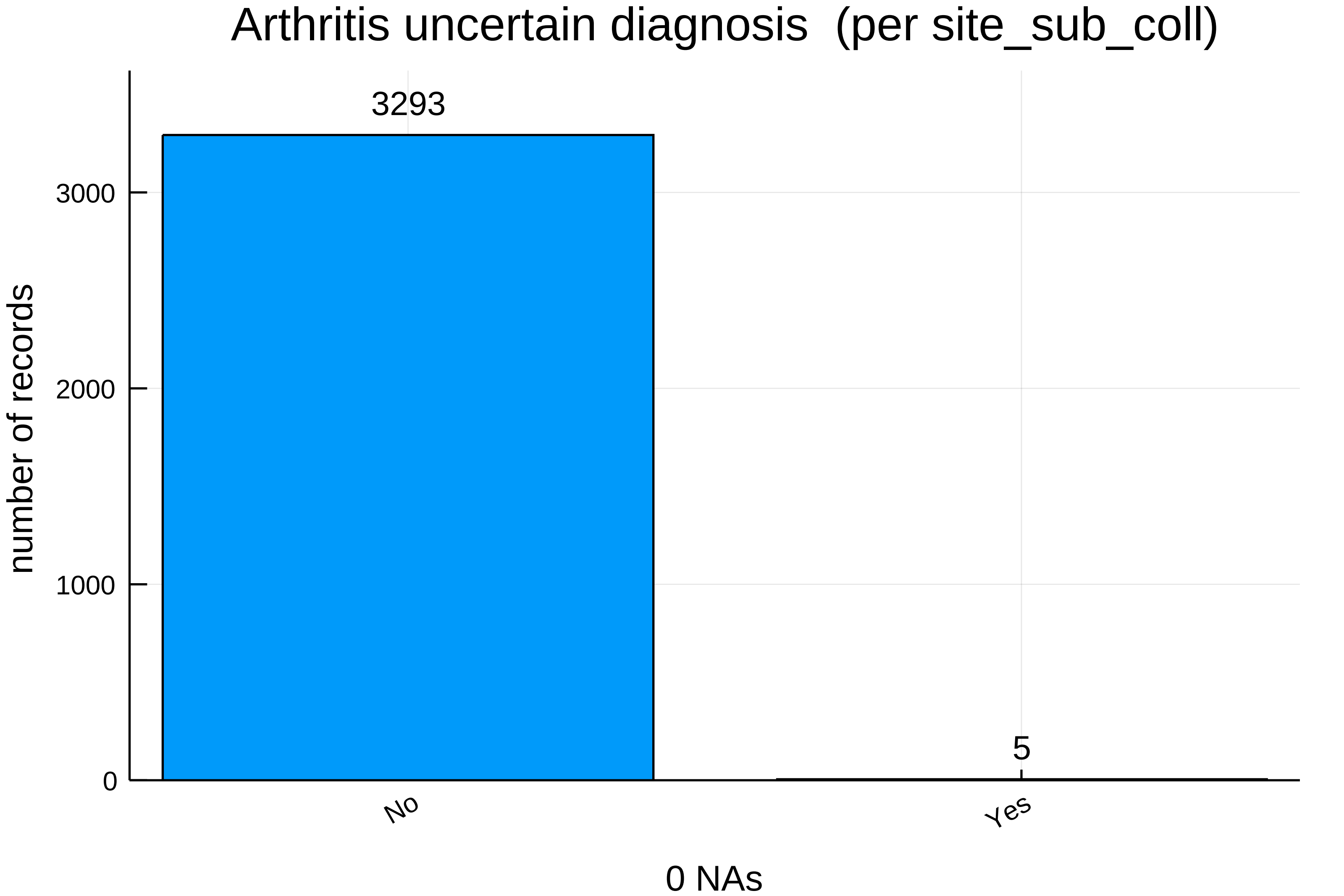
Arthritis uncertain diagnosis (per site_sub_coll)
number of records
3293
5
0
1000
2000
3000
No
Yes
0 NAs

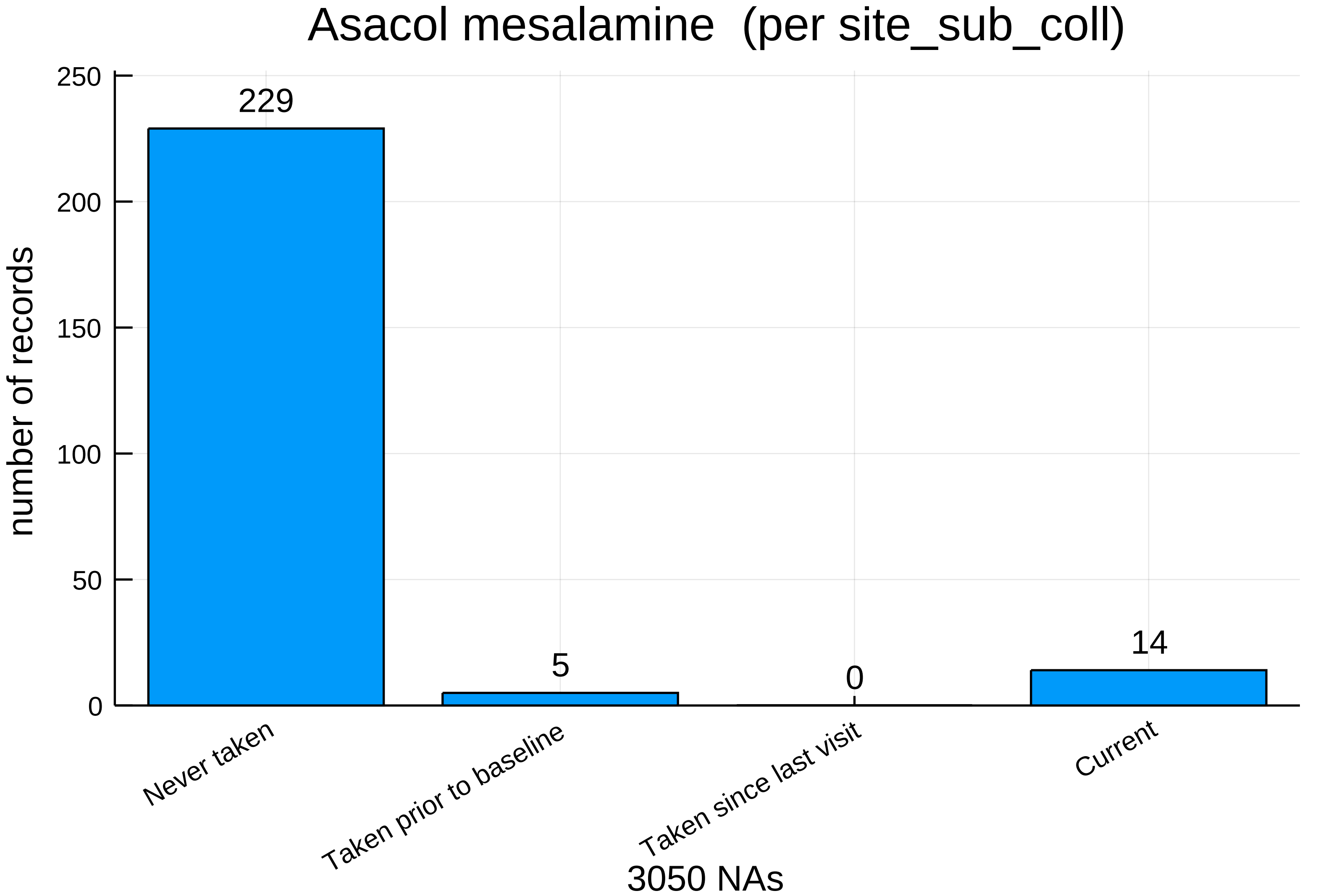
Asacol mesalamine (per site_sub_coll)
number of records
250
200
150
100
50
0
229
5
0
14
Never taken
Taken prior to baseline
Taken since last visit
Current
3050 NAs

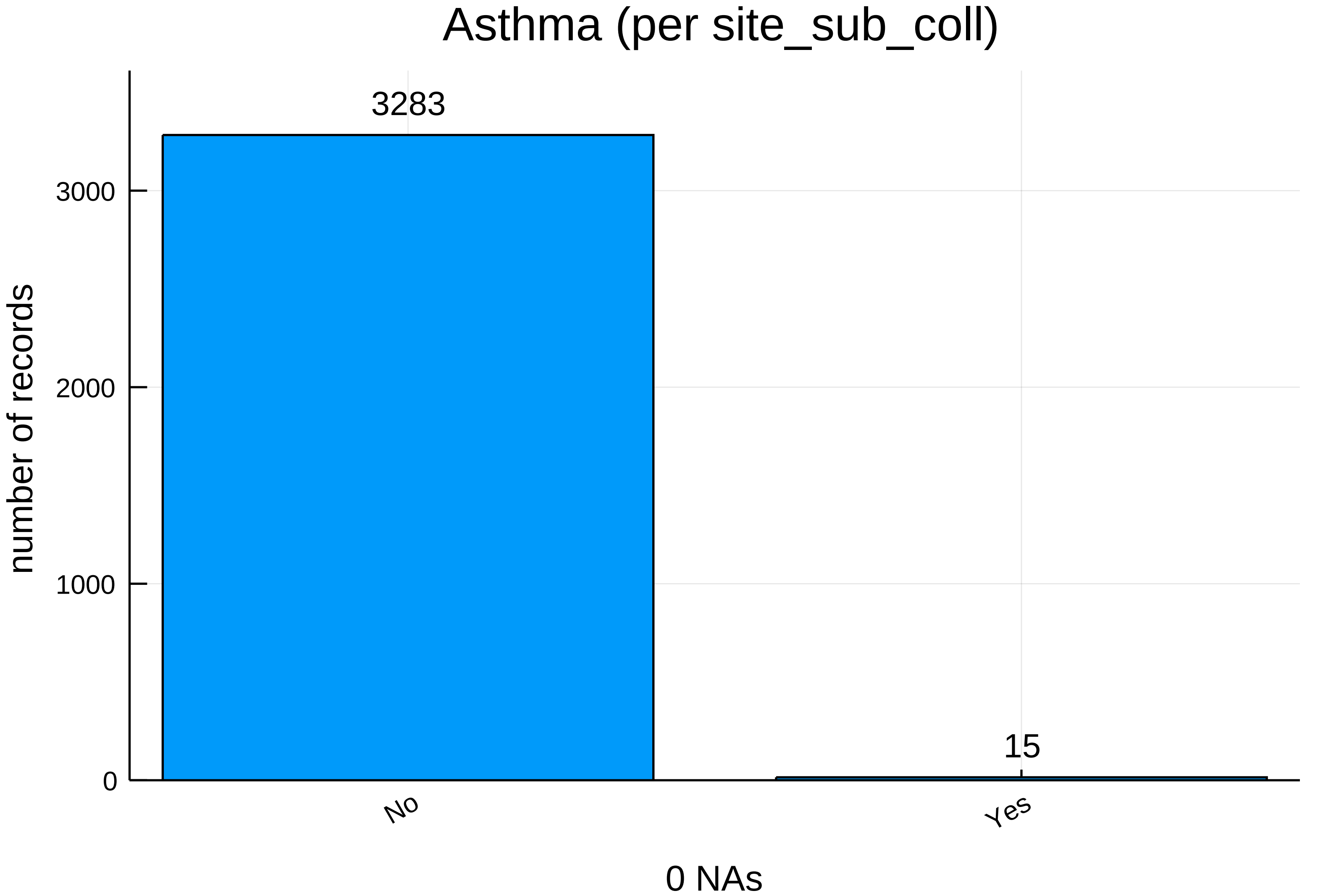

Asthma (per site_sub_coll)
number of records
3283
15
3000
2000
1000
0
No
Yes
0 NAs

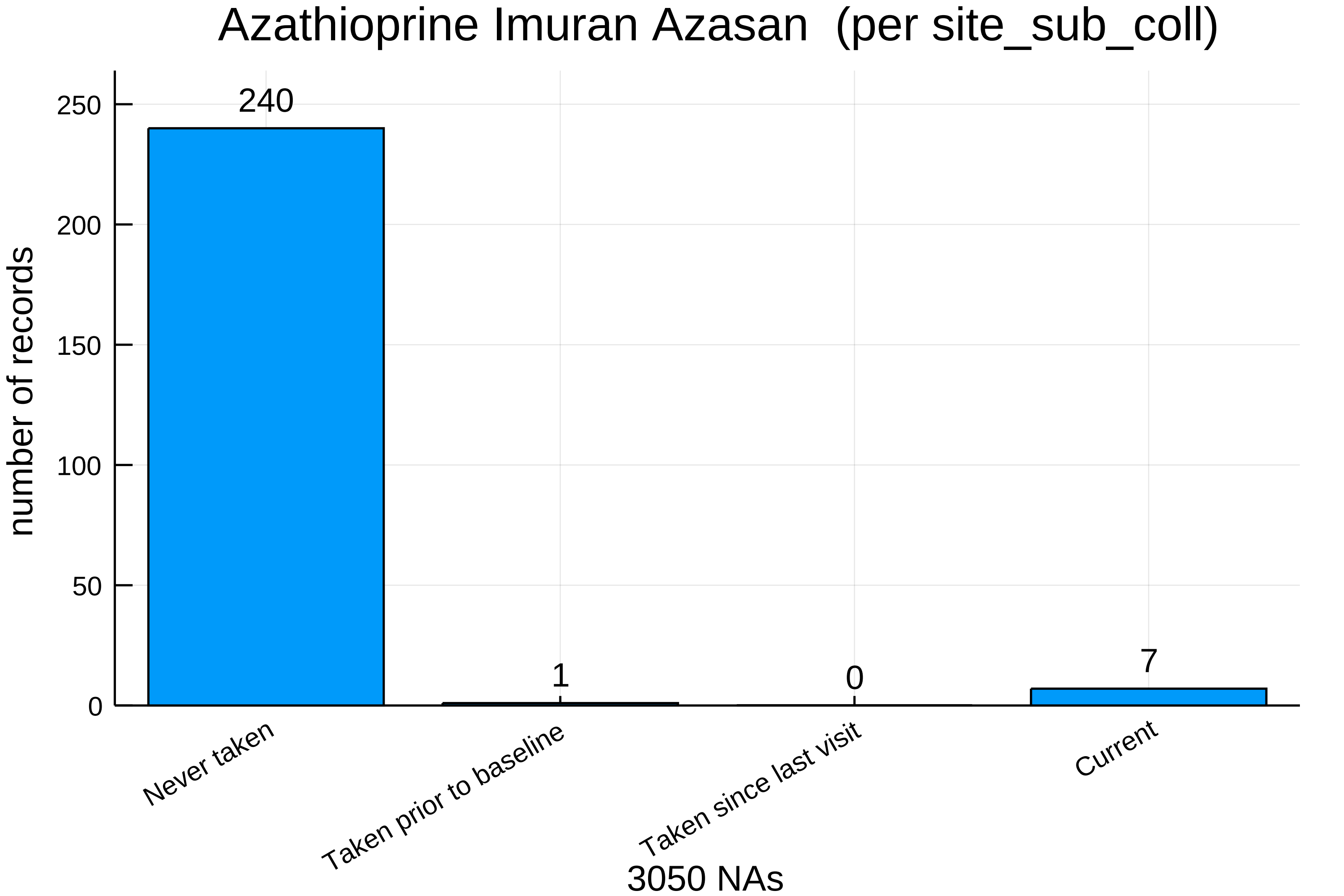

Azathioprine Imuran Azasan (per site_sub_coll)
number of records
250
200
150
100
50
0
240
1
0
7
Never taken
Taken prior to baseline
Taken since last visit
Current
3050 NAs

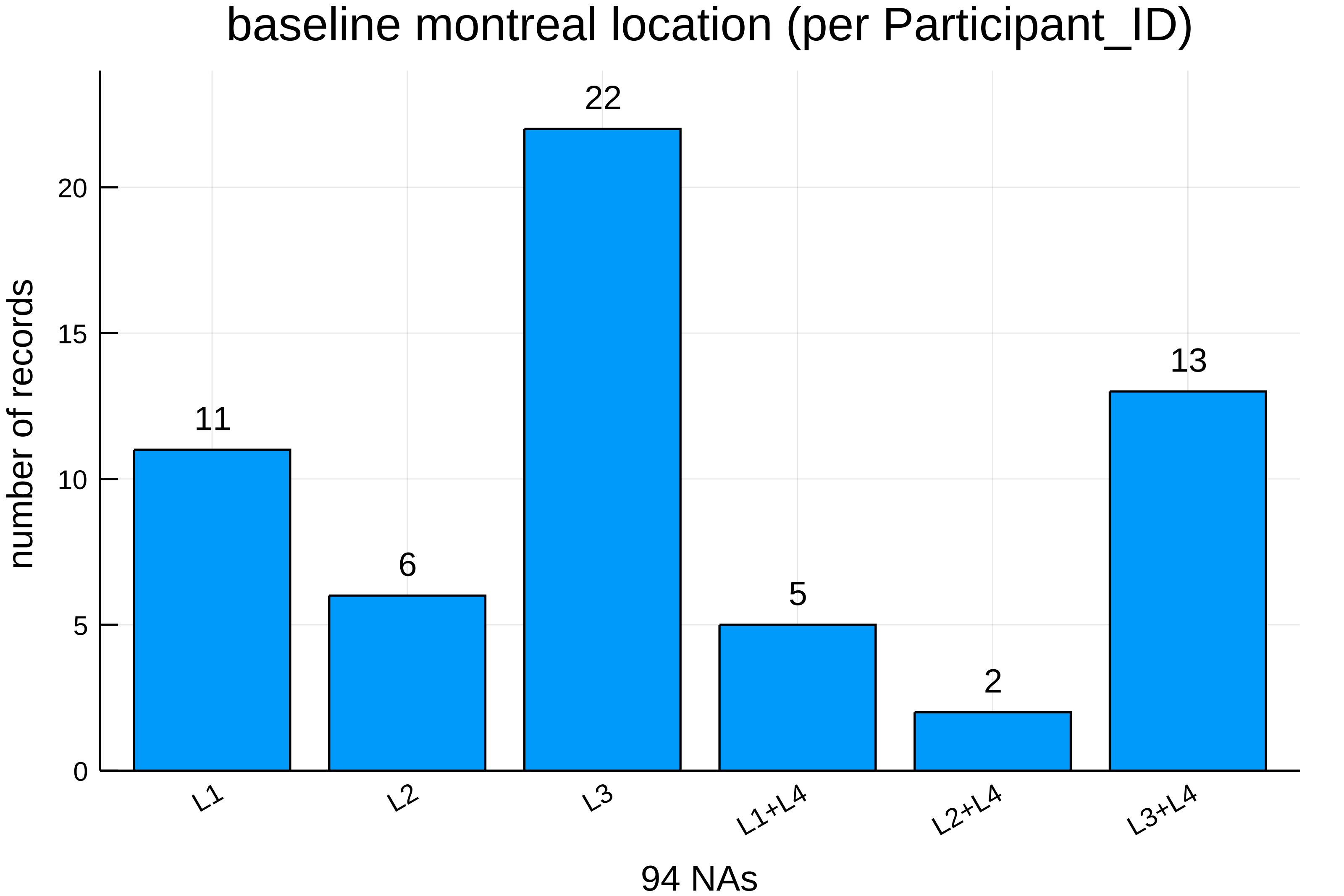
baseline montreal location (per Participant_ID)
number of records
0
5
10
15
20
11
6
22
5
2
13
L1
L2
L3
L1+L4
L2+L4
L3+L4
94 NAs

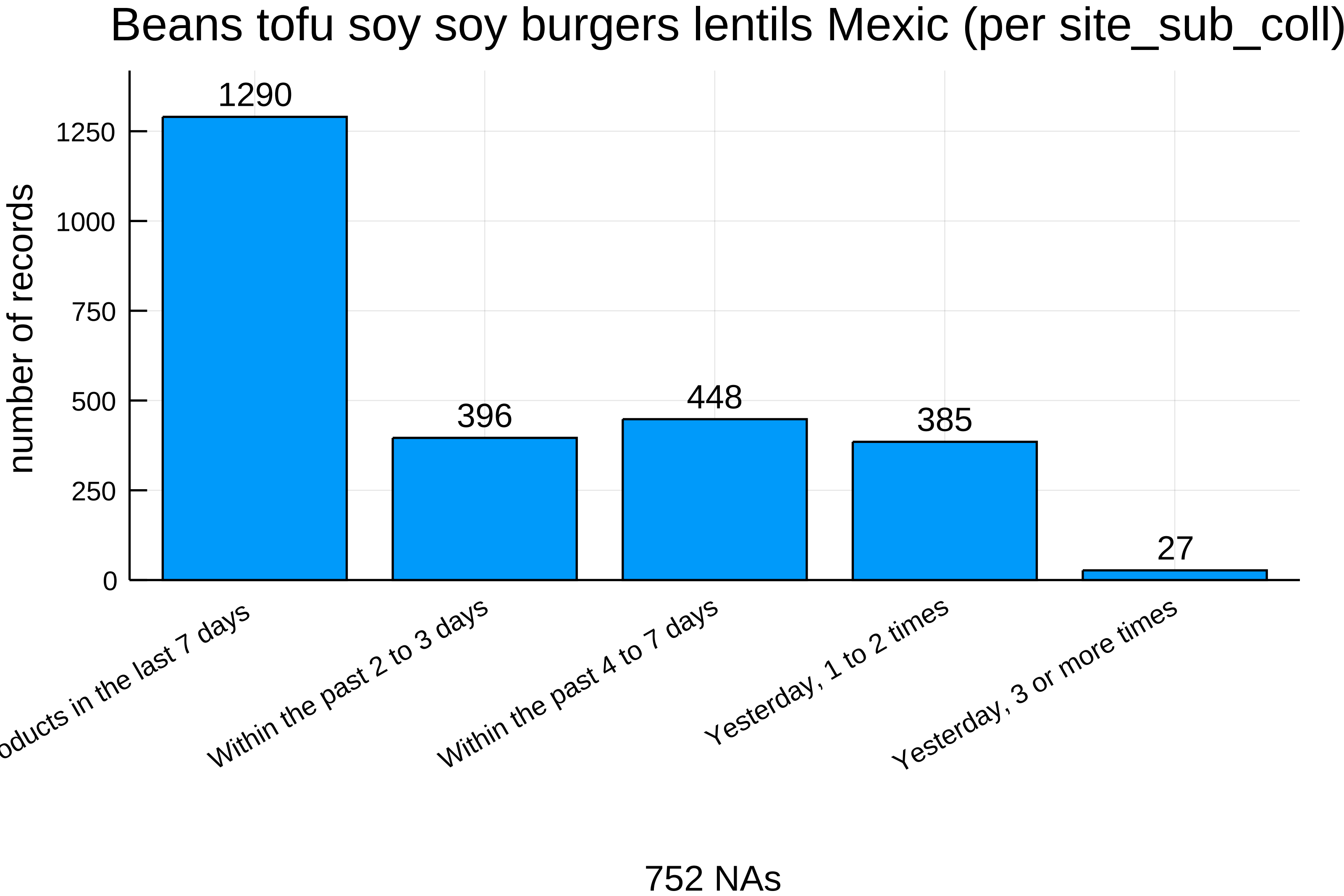
Beans tofu soy soy burgers lentils Mexic (per site_sub_coll)
number of records
1250
1000
750
500
250
0
1290
396
448
385
27
oducts in the last 7 days
Within the past 2 to 3 days
Within the past 4 to 7 days
Yesterday, 1 to 2 times
Yesterday, 3 or more times
752 NAs

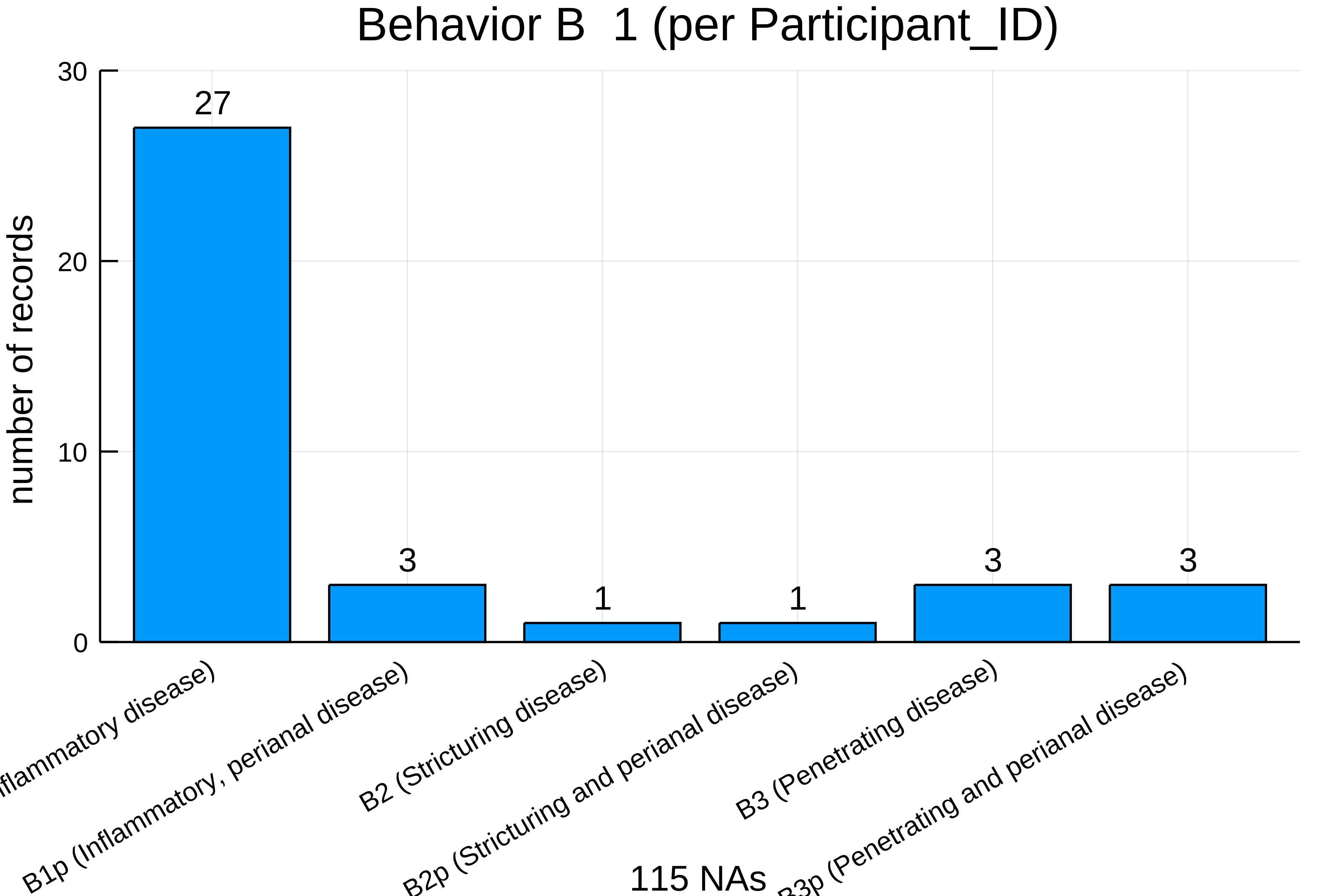

Behavior B 1 (per Participant_ID)
number of records
30
20
10
0
27
3
1
1
3
3
nflammatory disease)
B1p (Inflammatory, perianal disease)
B2 (Stricturing disease)
B2p (Stricturing and perianal disease)
B3 (Penetrating disease)
B3p (Penetrating and perianal disease)
115 NAs

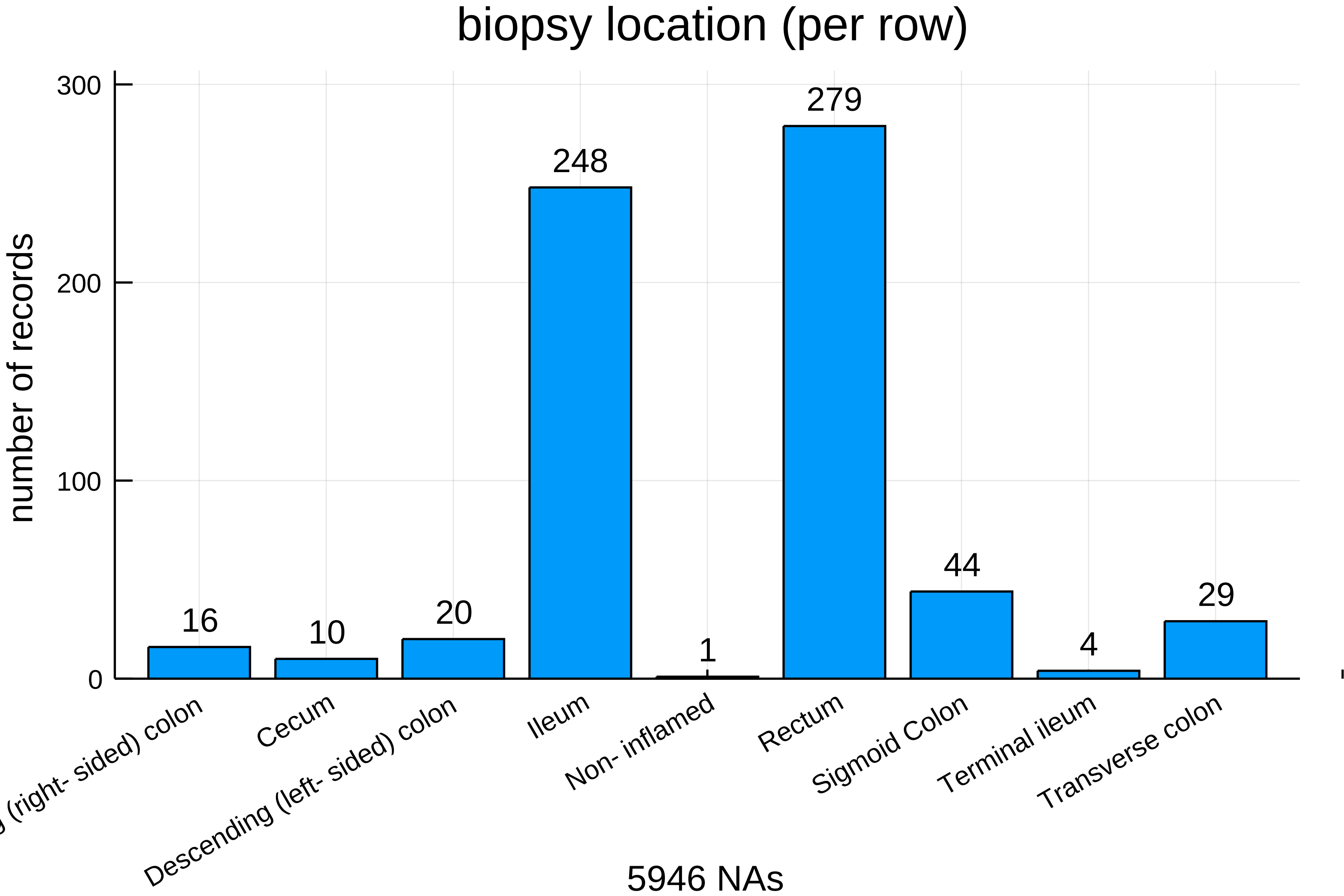
biopsy location (per row)
number of records
300
200
100
0
16
10
20
248
1
279
44
4
29
g (right- sided) colon
Cecum
Descending (left- sided) colon
Ileum
Non- inflamed
Rectum
Sigmoid Colon
Terminal ileum
Transverse colon
5946 NAs

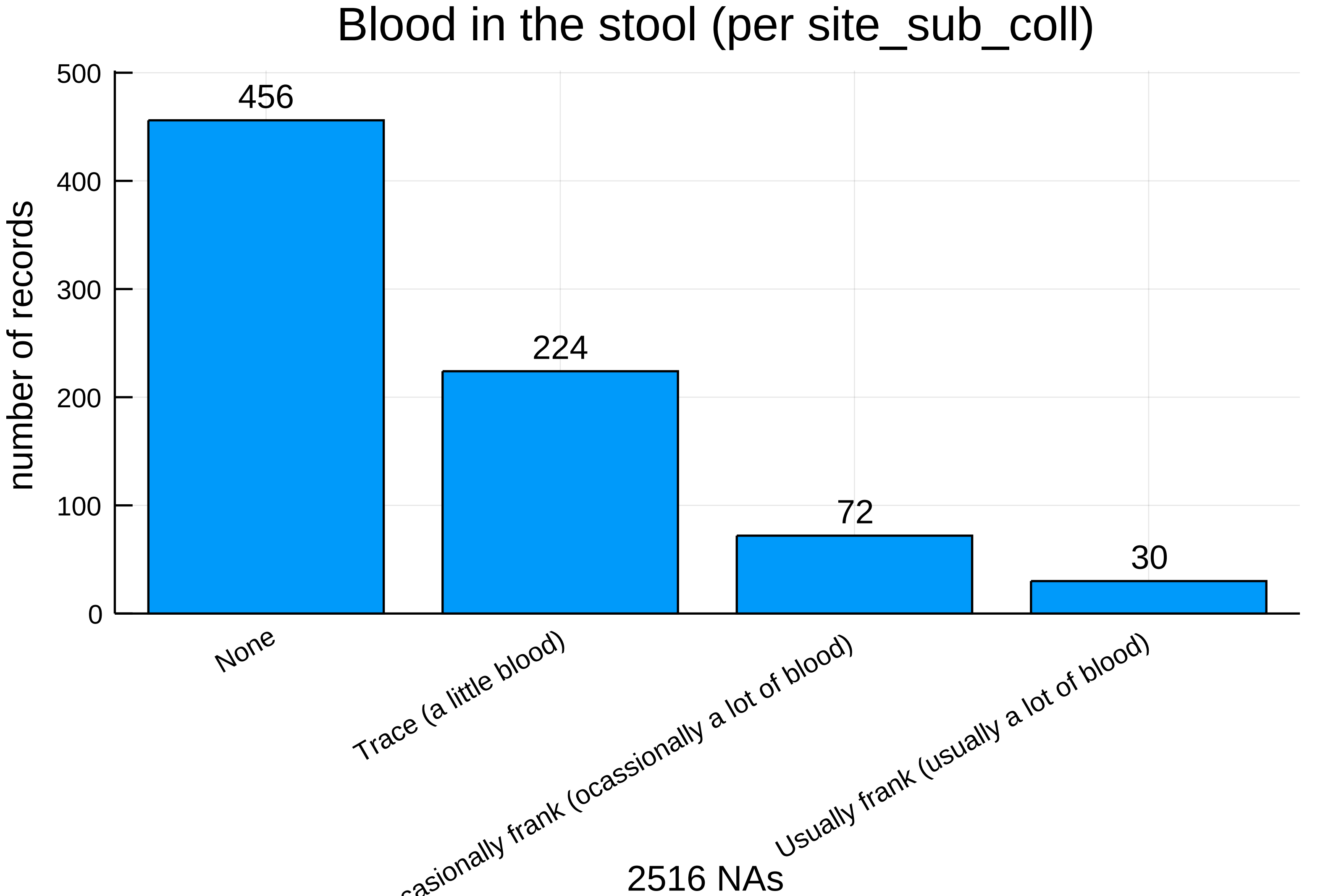
Blood in the stool (per site_sub_coll)
number of records
500
400
300
200
100
0
456
224
72
30
None
Trace (a little blood)
casionally frank (ocassionally a lot of blood)
Usually frank (usually a lot of blood)
2516 NAs

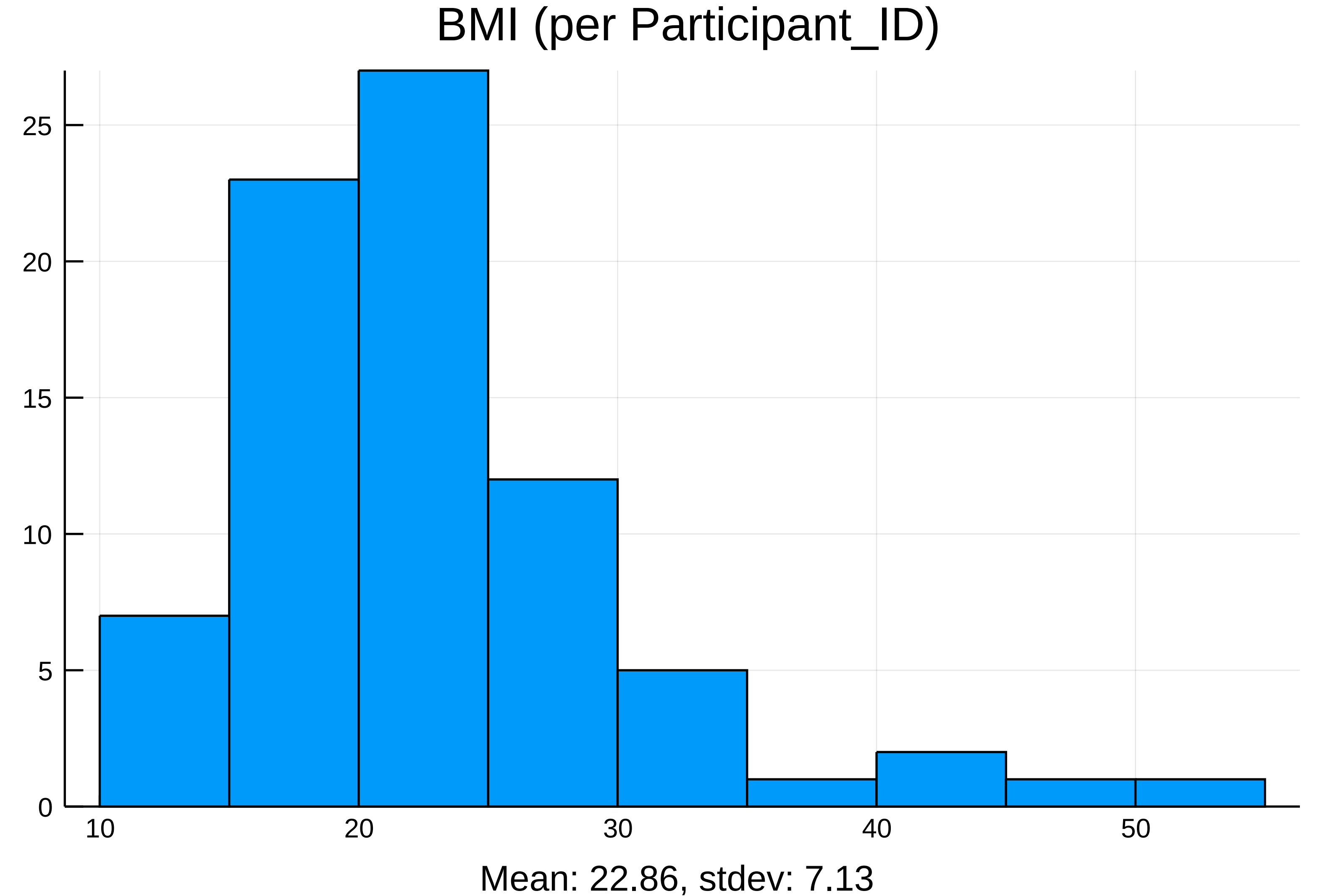
BMI (per Participant_ID)
25
20
15
10
5
0
10
20
30
40
50
Mean: 22.86, stdev: 7.13

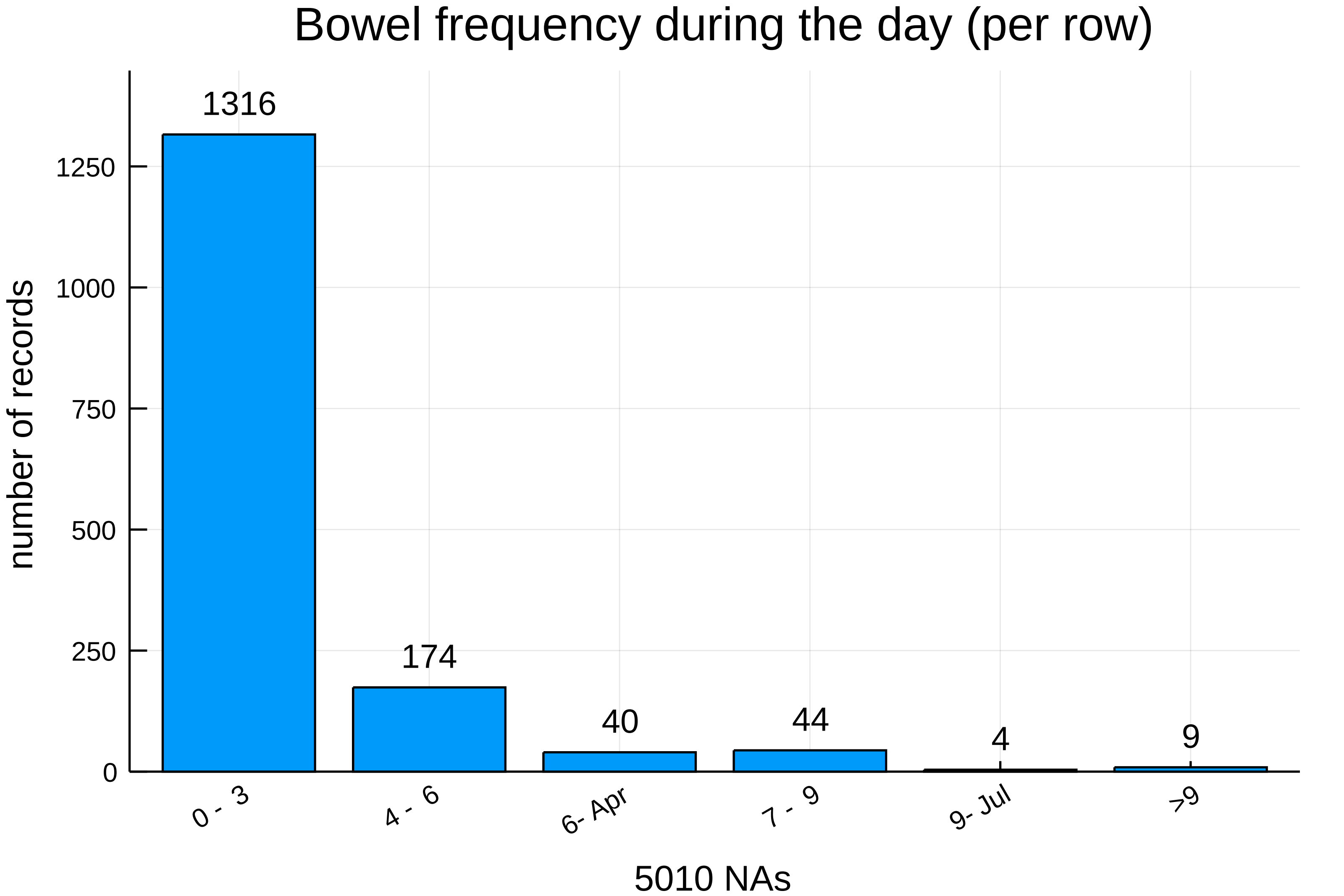
Bowel frequency during the day (per row)
number of records
1316
174
40
44
4
9
0
250
500
750
1000
1250
0 - 3
4 - 6
6- Apr
7 - 9
9- Jul
>9
5010 NAs

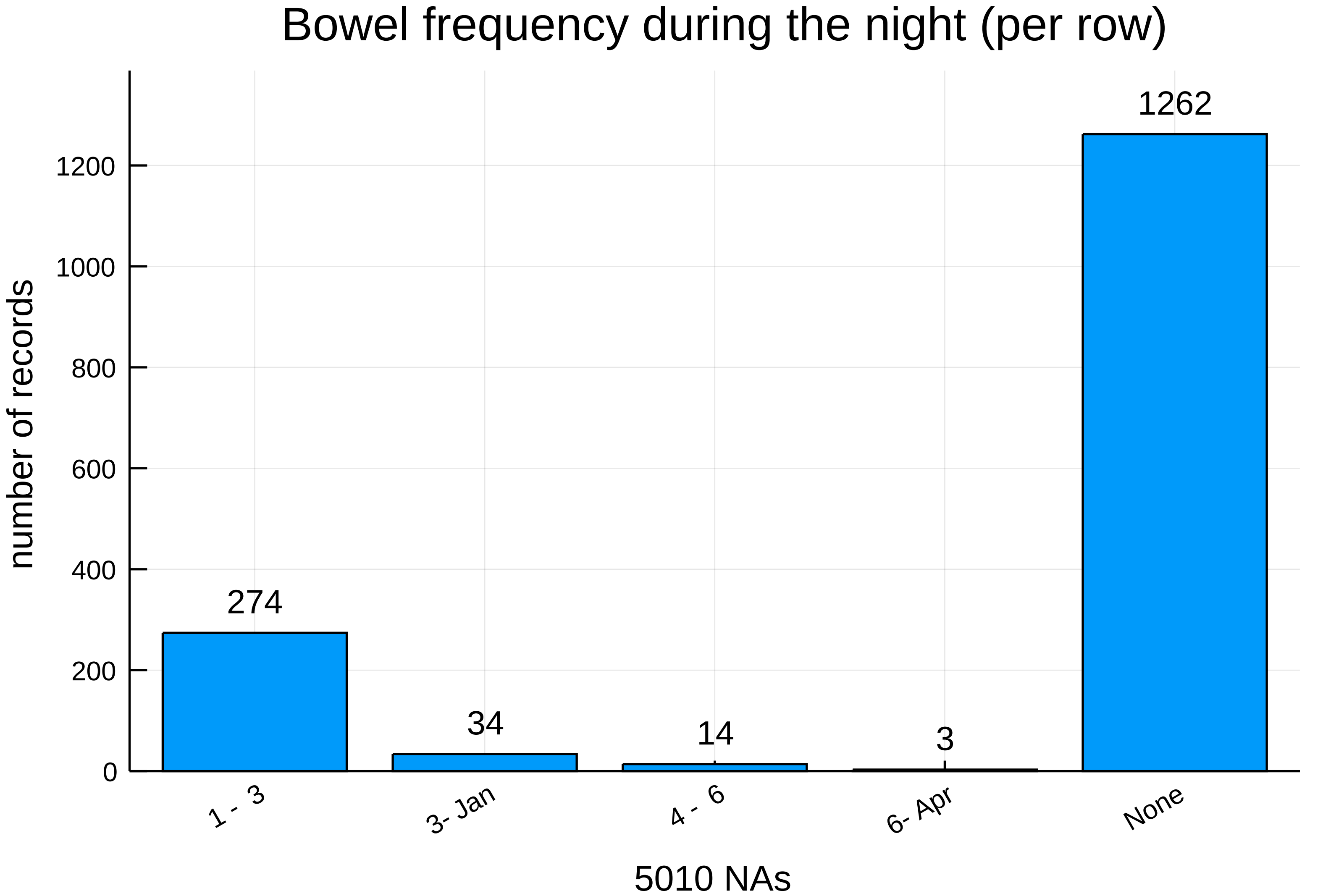
Bowel frequency during the night (per row)
number of records
0
200
400
600
800
1000
1200
274
34
14
3
1262
1 - 3
3- Jan
4 - 6
6- Apr
None
5010 NAs

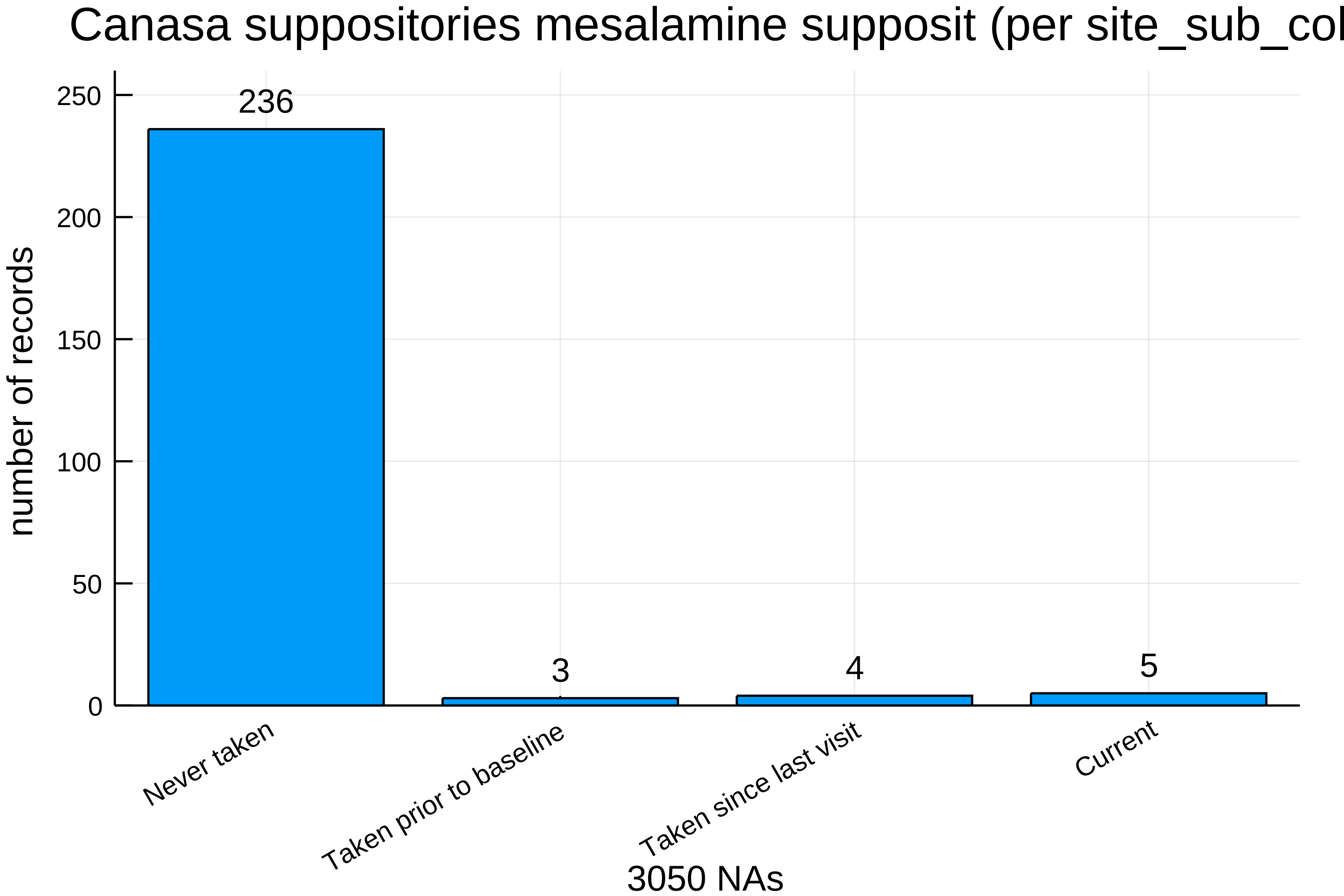
Canasa suppositories mesalamine supposit (per site_sub_col
number of records
250
200
150
100
50
0
236
3
4
5
Never taken
Taken prior to baseline
Taken since last visit
Current
3050 NAs

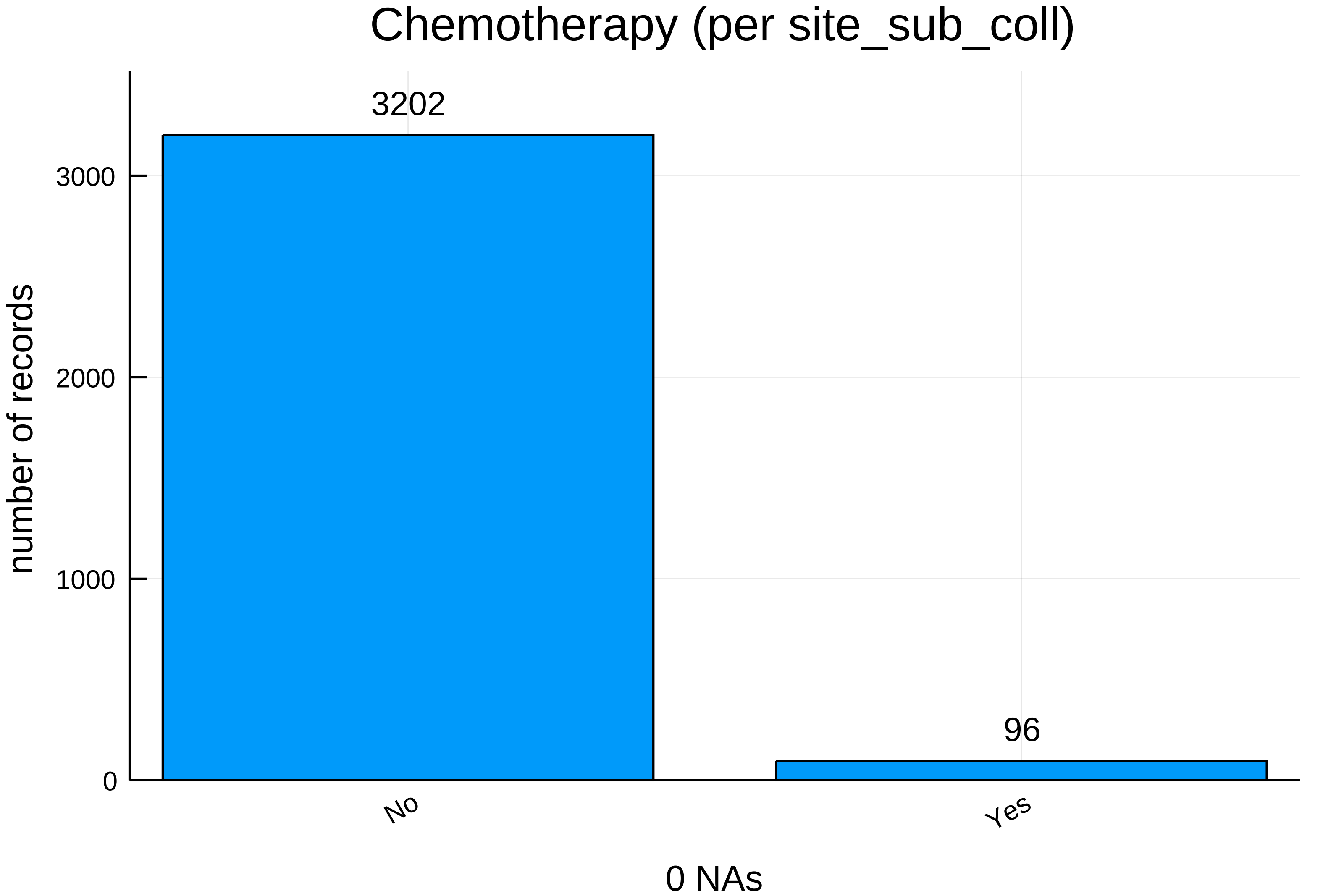
Chemotherapy (per site_sub_coll)
number of records
3202
96
3000
2000
1000
0
No
Yes
0 NAs

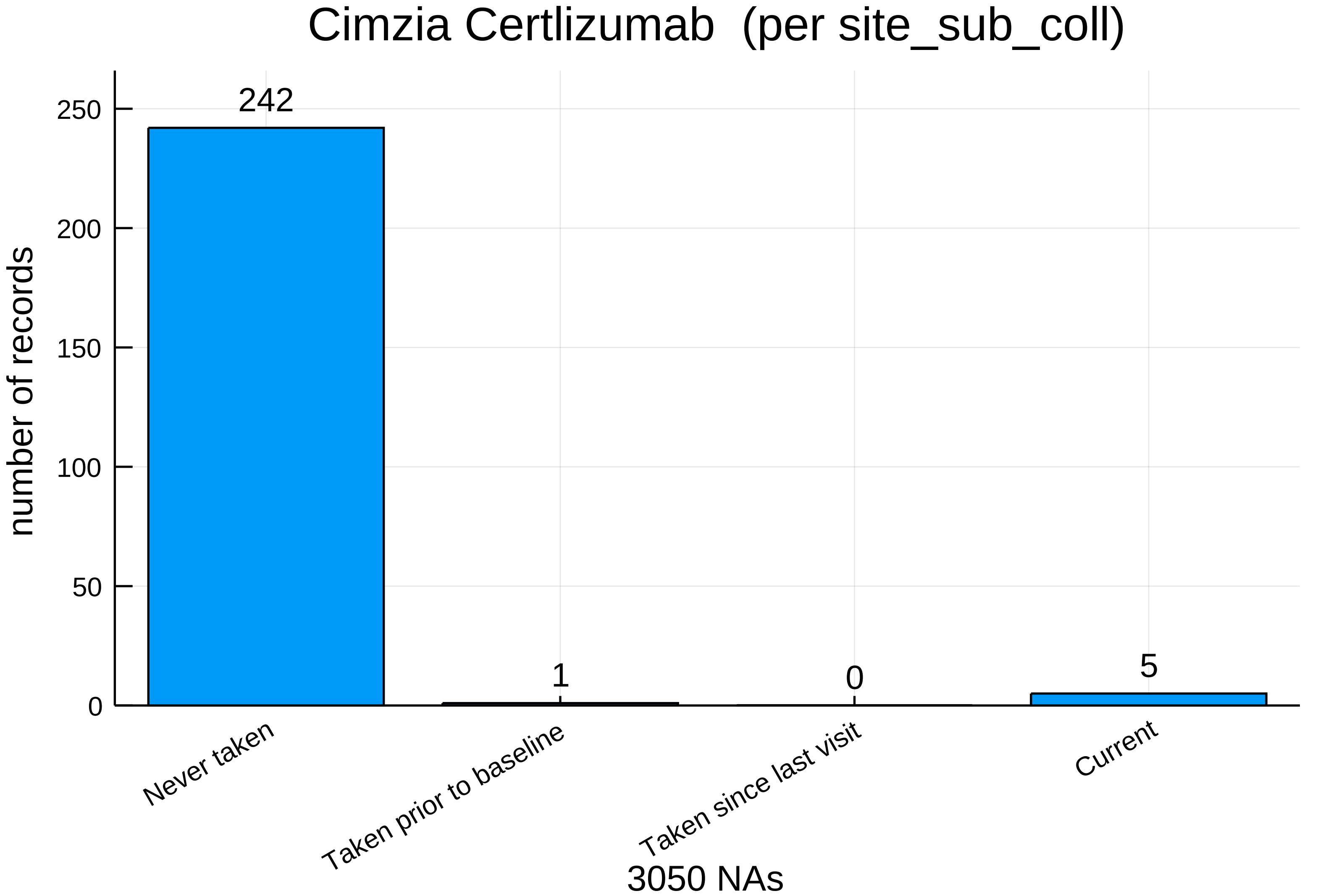
Cimzia Certlizumab (per site_sub_coll)
number of records
250
200
150
100
50
0
242
1
0
5
Never taken
Taken prior to baseline
Taken since last visit
Current
3050 NAs

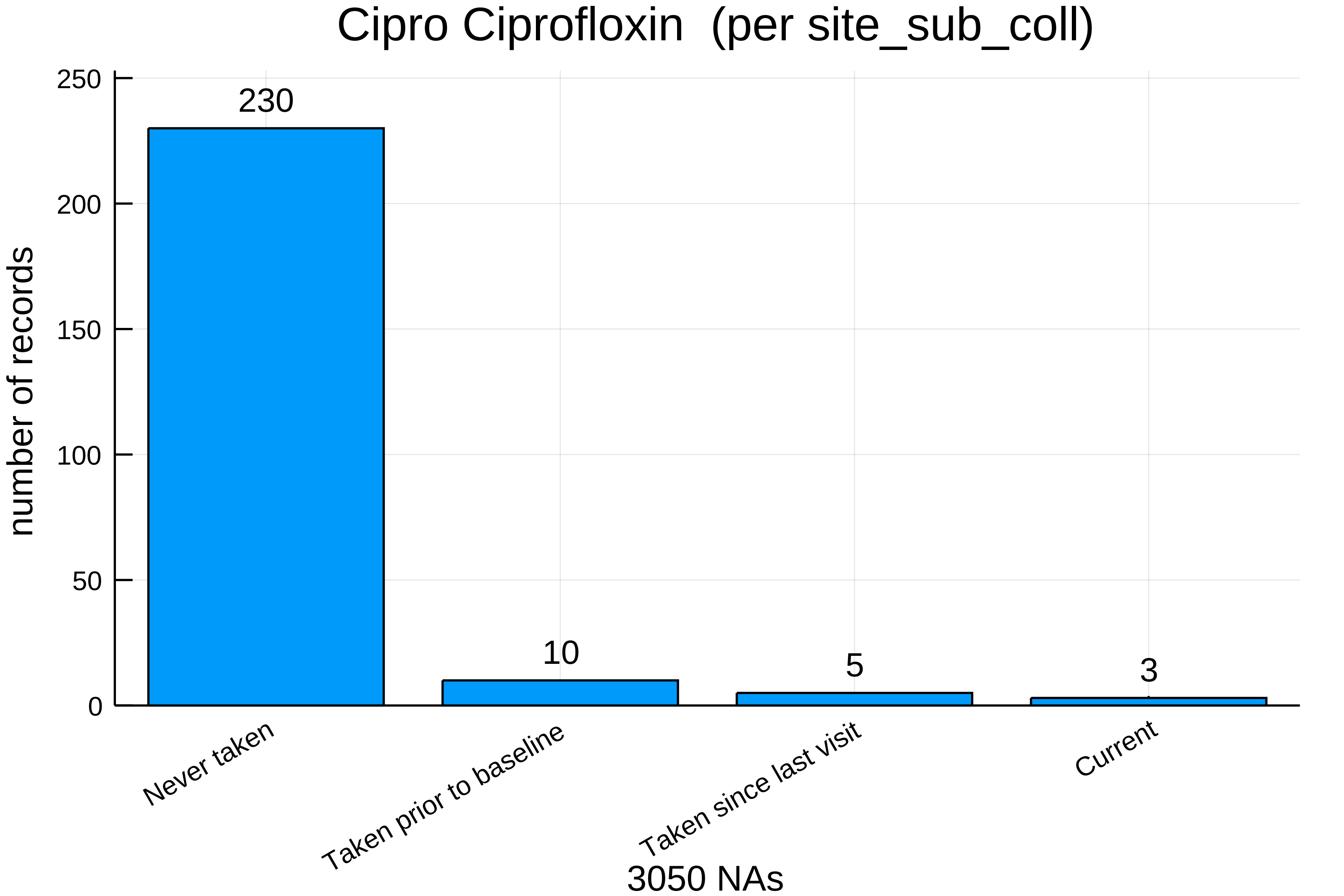
Cipro Ciprofloxin (per site_sub_coll)
number of records
250
200
150
100
50
0
230
10
5
3
Never taken
Taken prior to baseline
Taken since last visit
Current
3050 NAs

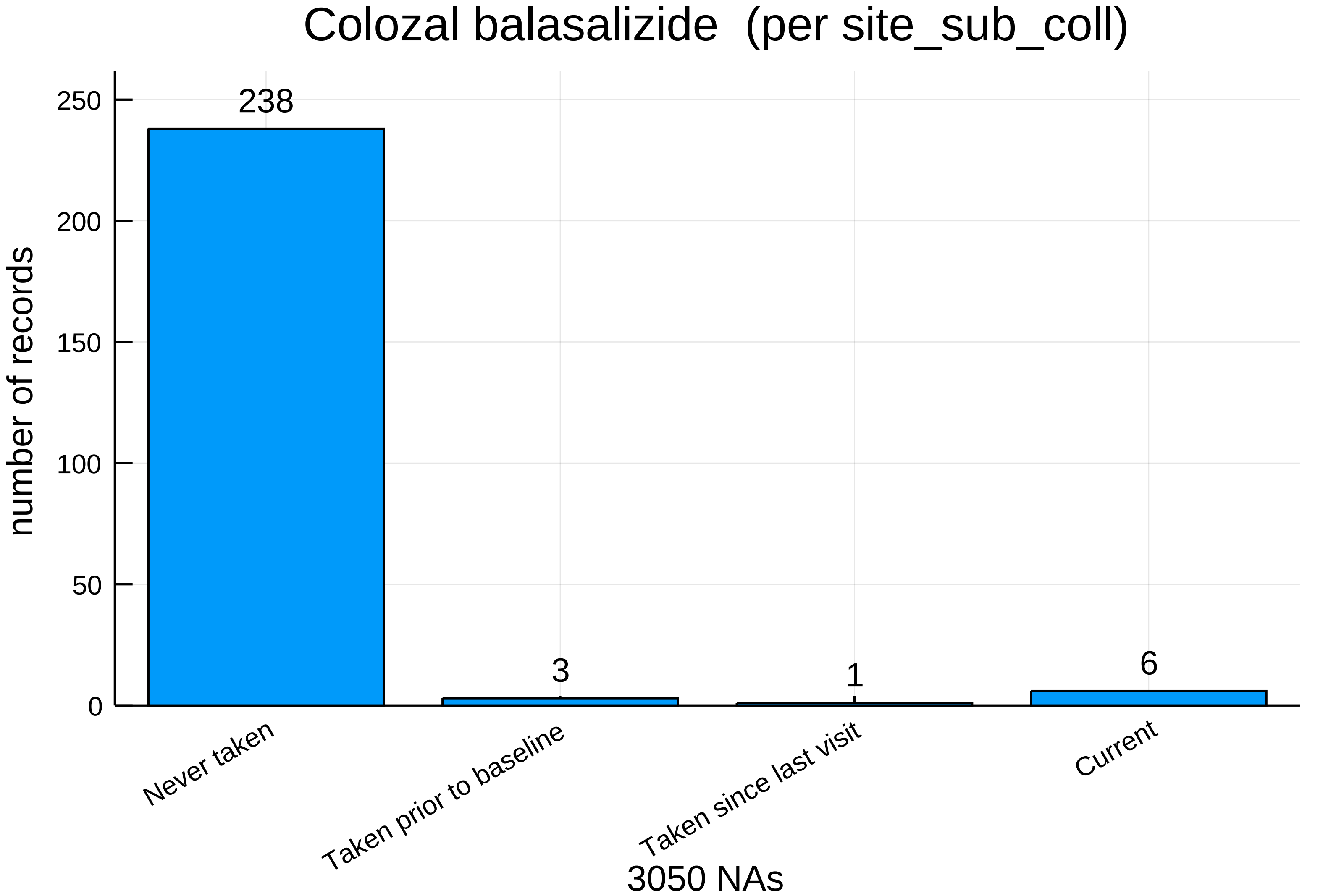
Colozal balasalizide (per site_sub_coll)
number of records
250
200
150
100
50
0
238
3
1
6
Never taken
Taken prior to baseline
Taken since last visit
Current
3050 NAs

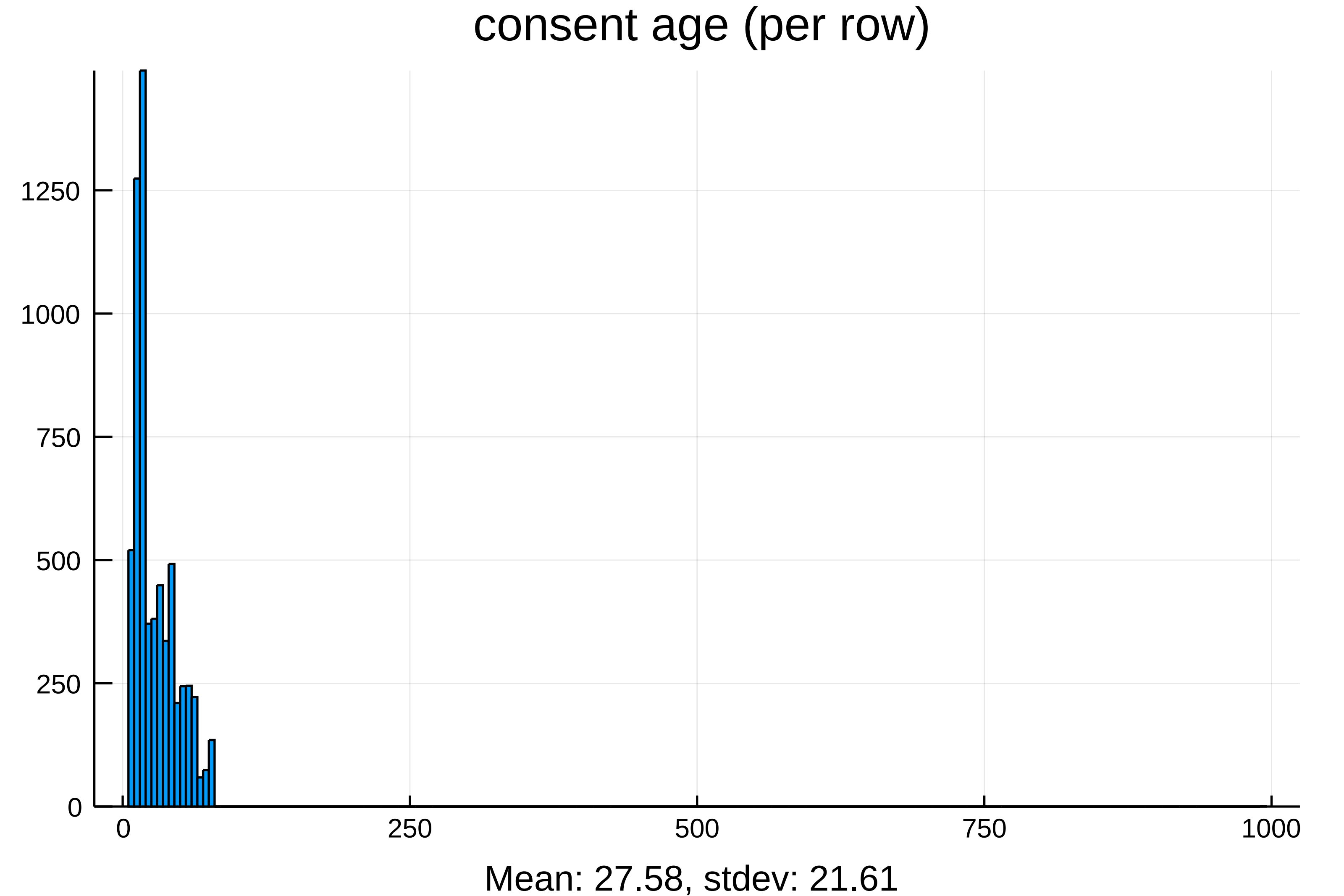
consent age (per row)
0
250
500
750
1000
1250
0
250
500
750
1000
Mean: 27.58, stdev: 21.61

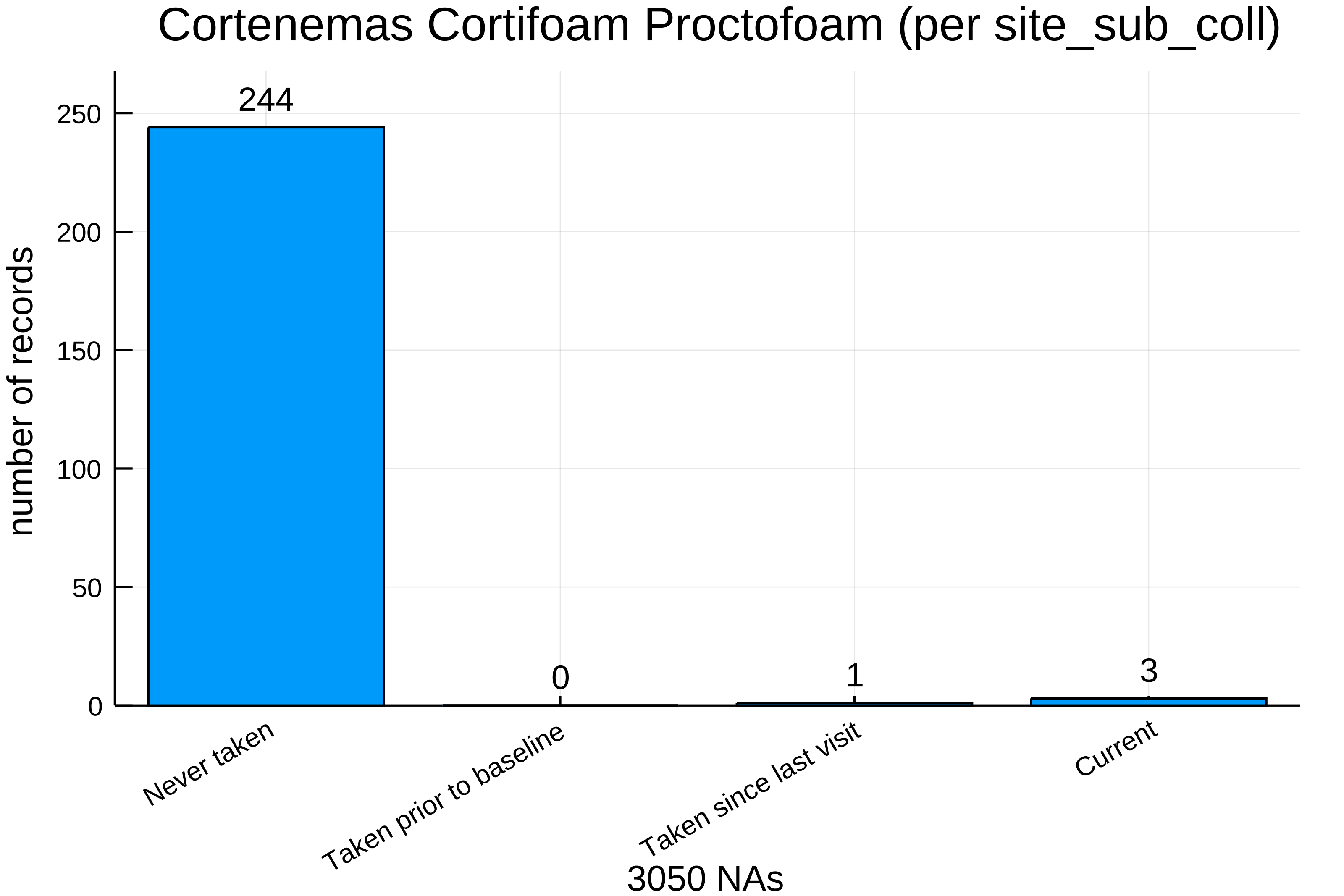

Cortenemas Cortifoam Proctofoam (per site_sub_coll)
number of records
250
200
150
100
50
0
244
0
1
3
Never taken
Taken prior to baseline
Taken since last visit
Current
3050 NAs

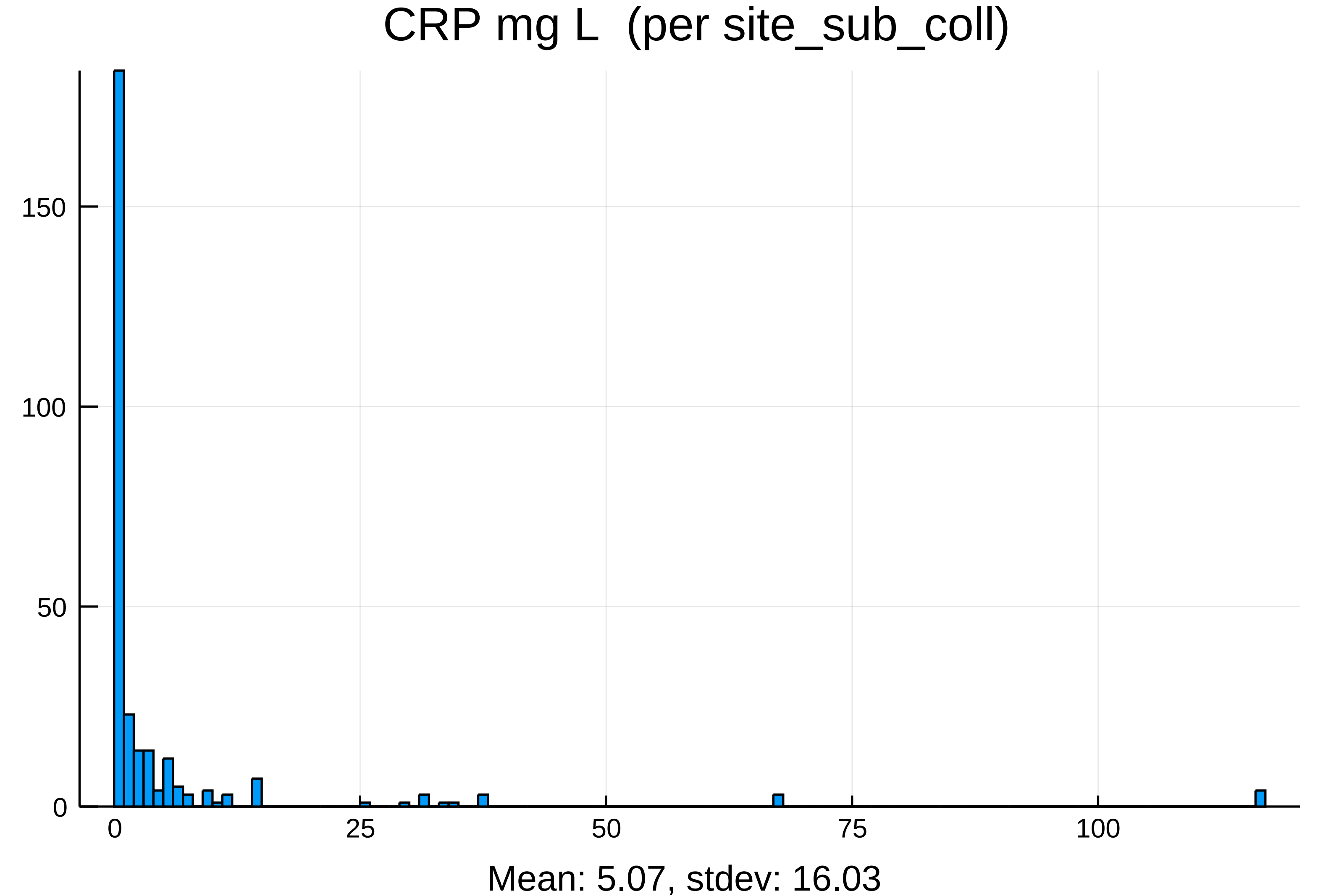
CRP mg L (per site_sub_coll)
150
100
50
0
0
25
50
75
100
Mean: 5.07, stdev: 16.03

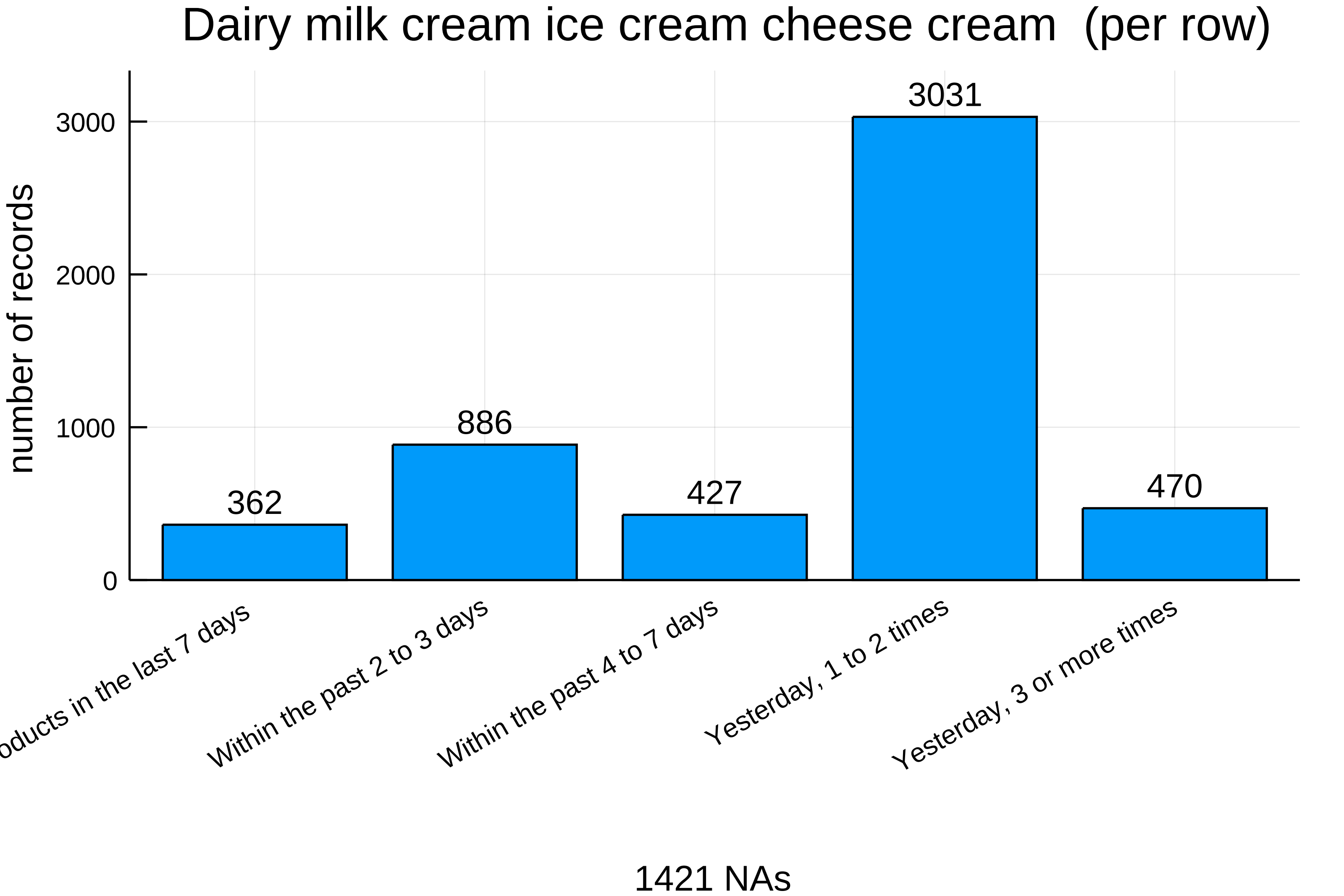
Dairy milk cream ice cream cheese cream (per row)
number of records
3000
2000
1000
0
362
886
427
3031
470
oducts in the last 7 days
Within the past 2 to 3 days
Within the past 4 to 7 days
Yesterday, 1 to 2 times
Yesterday, 3 or more times
1421 NAs

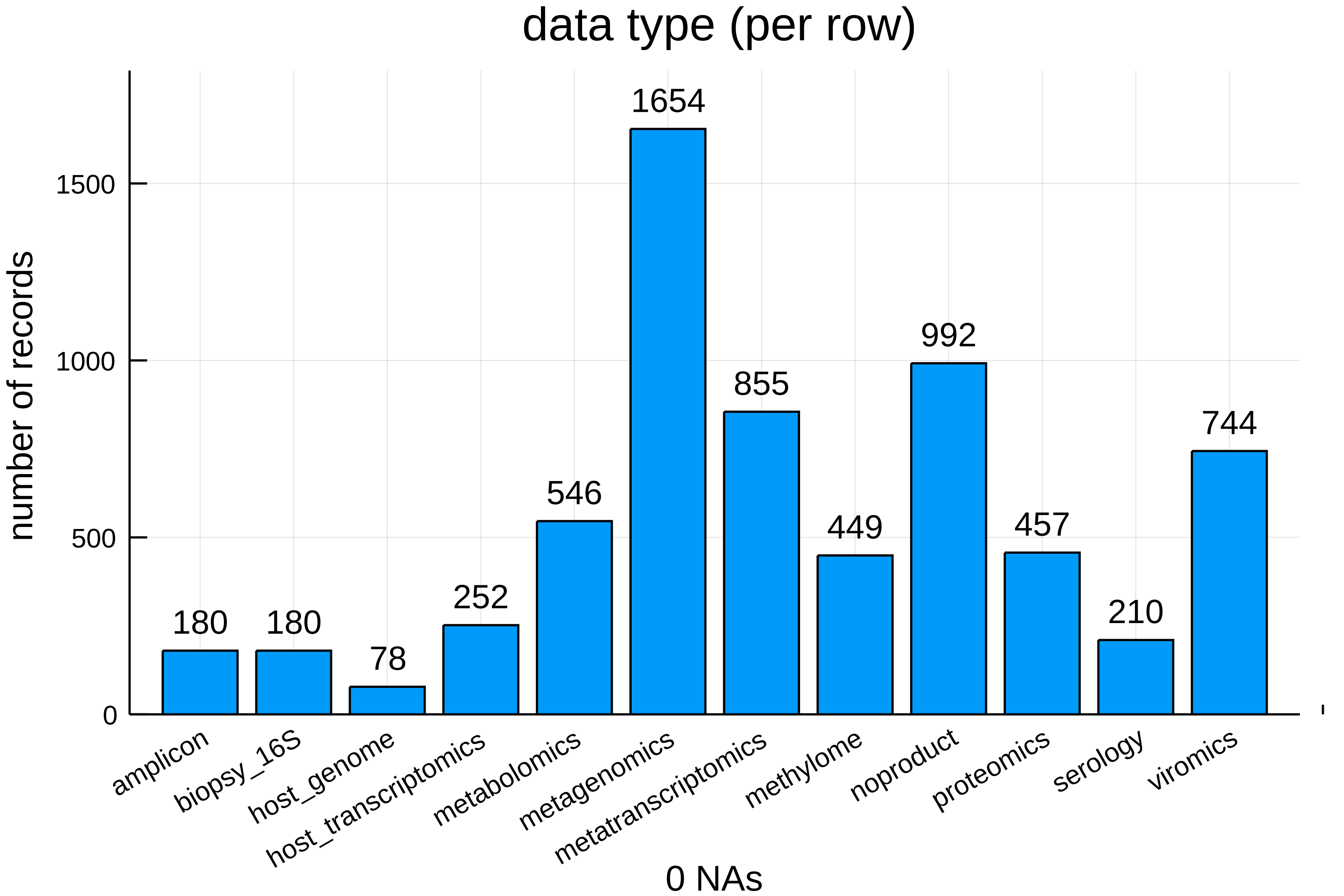
data type (per row)
number of records
0
500
1000
1500
180
180
78
252
546
1654
855
449
992
457
210
744
amplicon
biopsy_16S
host_genome
host_transcriptomics
metabolomics
metagenomics
metatranscriptomics
methylome
noproduct
proteomics
serology
viromics
0 NAs

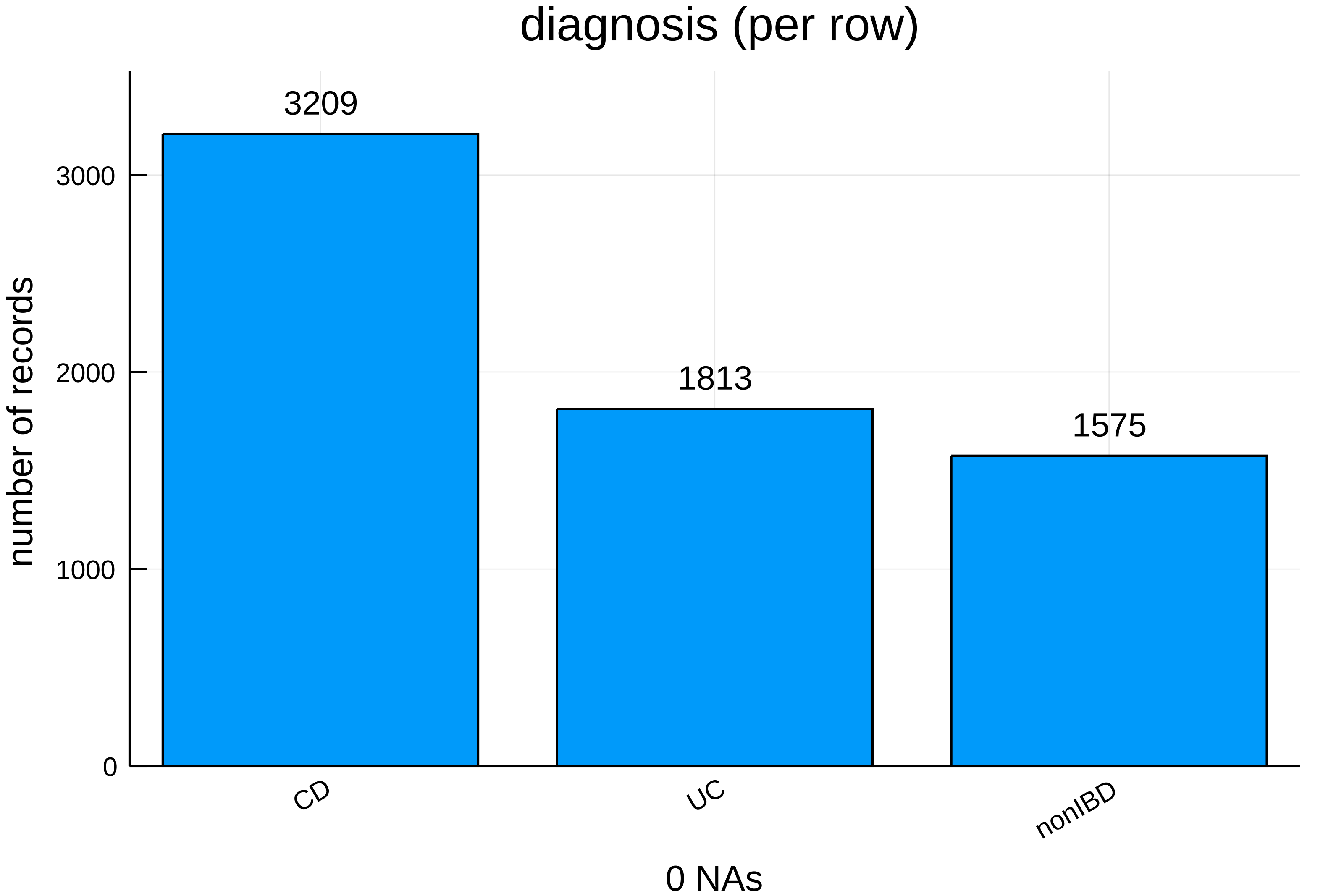
diagnosis (per row)
number of records
3000
2000
1000
0
3209
1813
1575
CD
UC
nonIBD
0 NAs

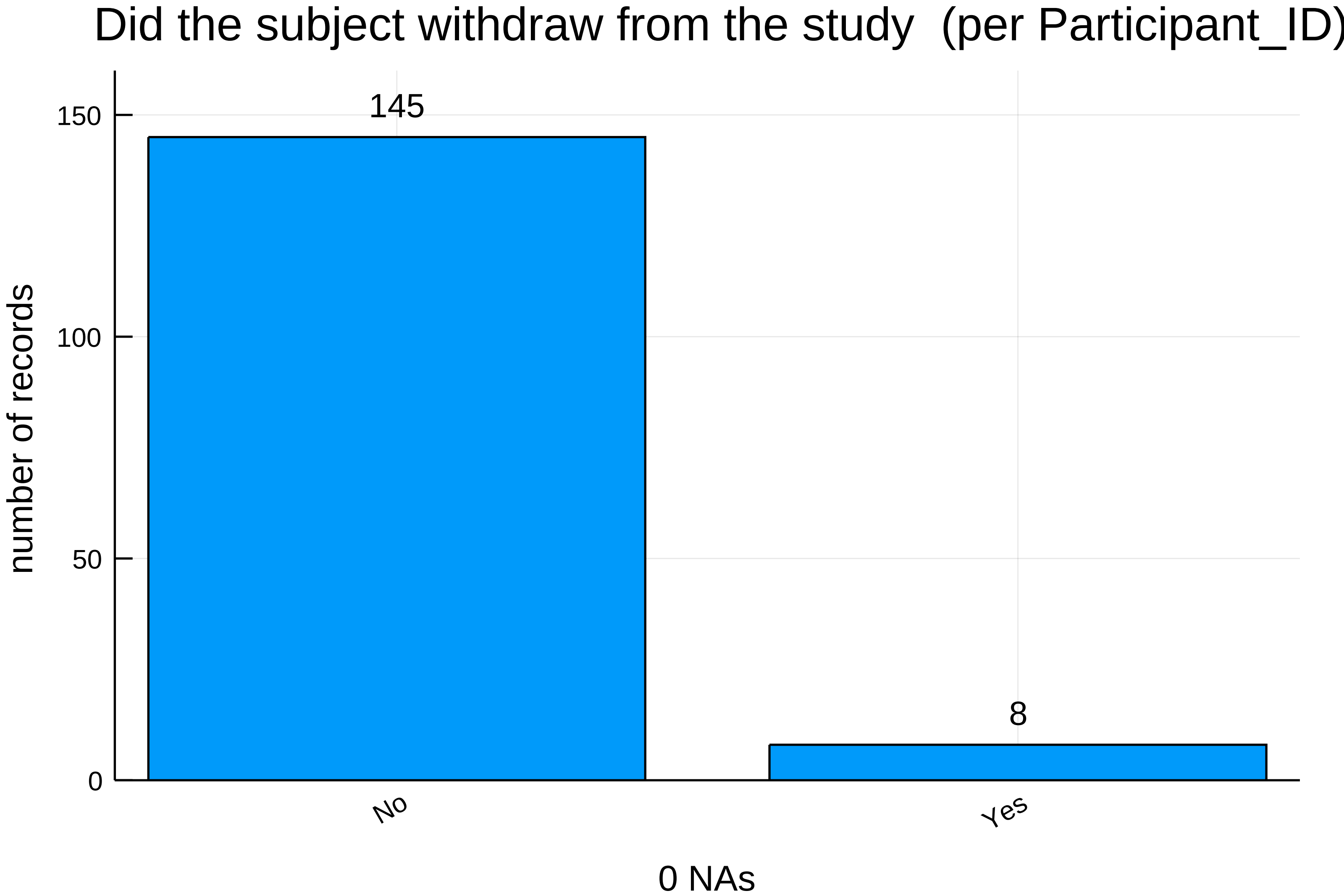
Did the subject withdraw from the study (per Participant_ID)
number of records
150
100
50
0
145
8
No
Yes
0 NAs

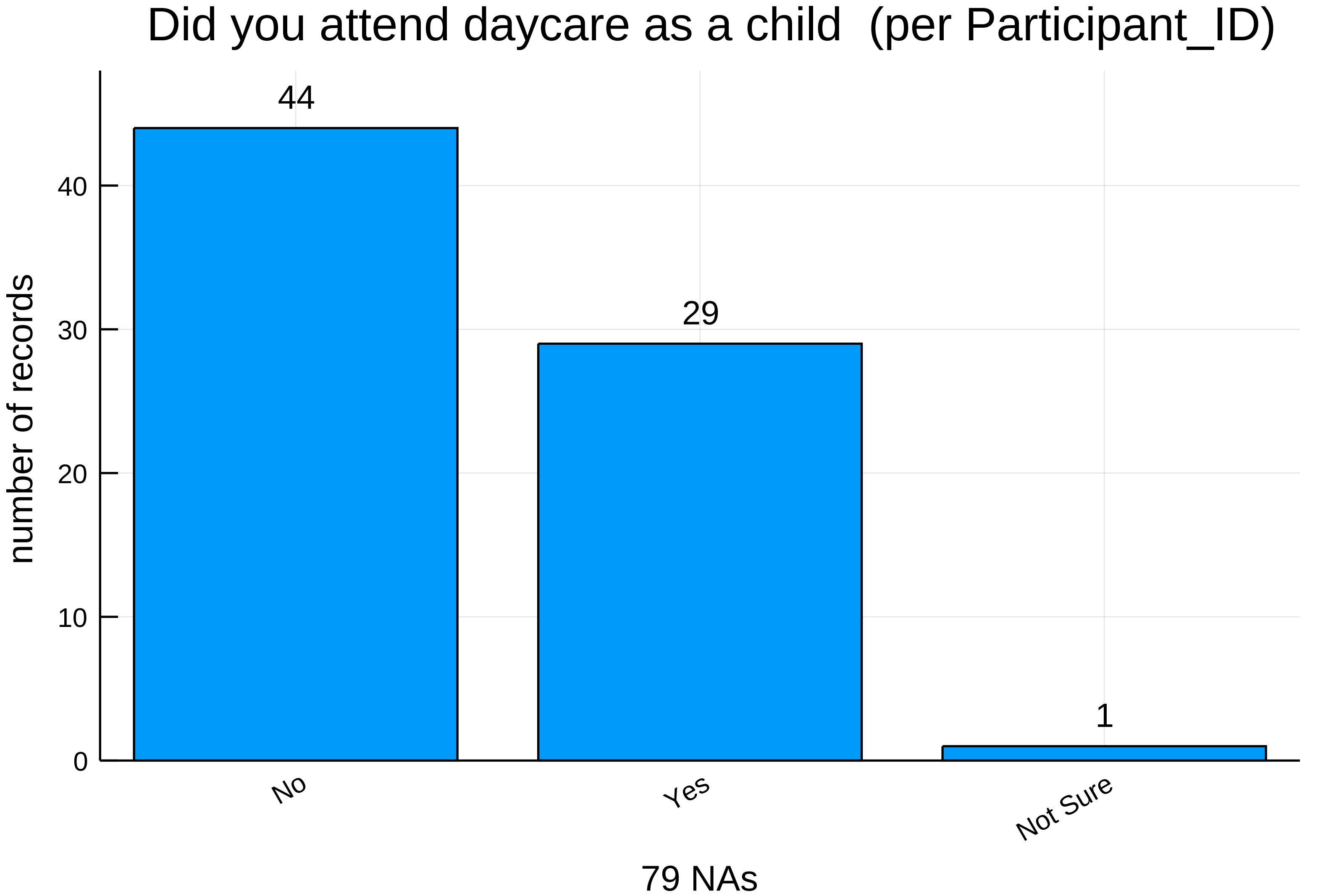
Did you attend daycare as a child (per Participant_ID)
number of records
44
29
1
0
10
20
30
40
No
Yes
Not Sure
79 NAs

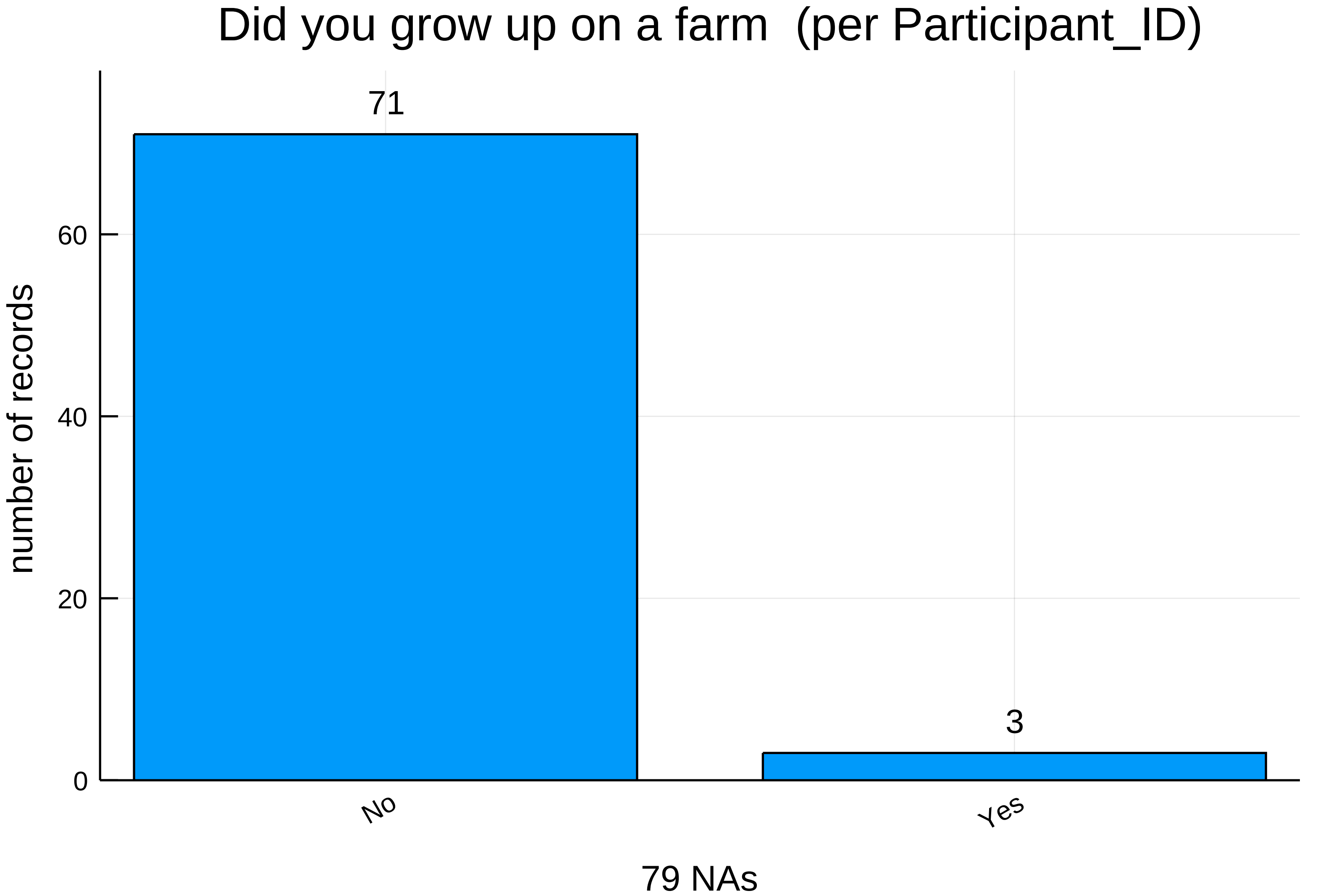

Did you grow up on a farm (per Participant_ID)
number of records
0
20
40
60
71
3
No
Yes
79 NAs

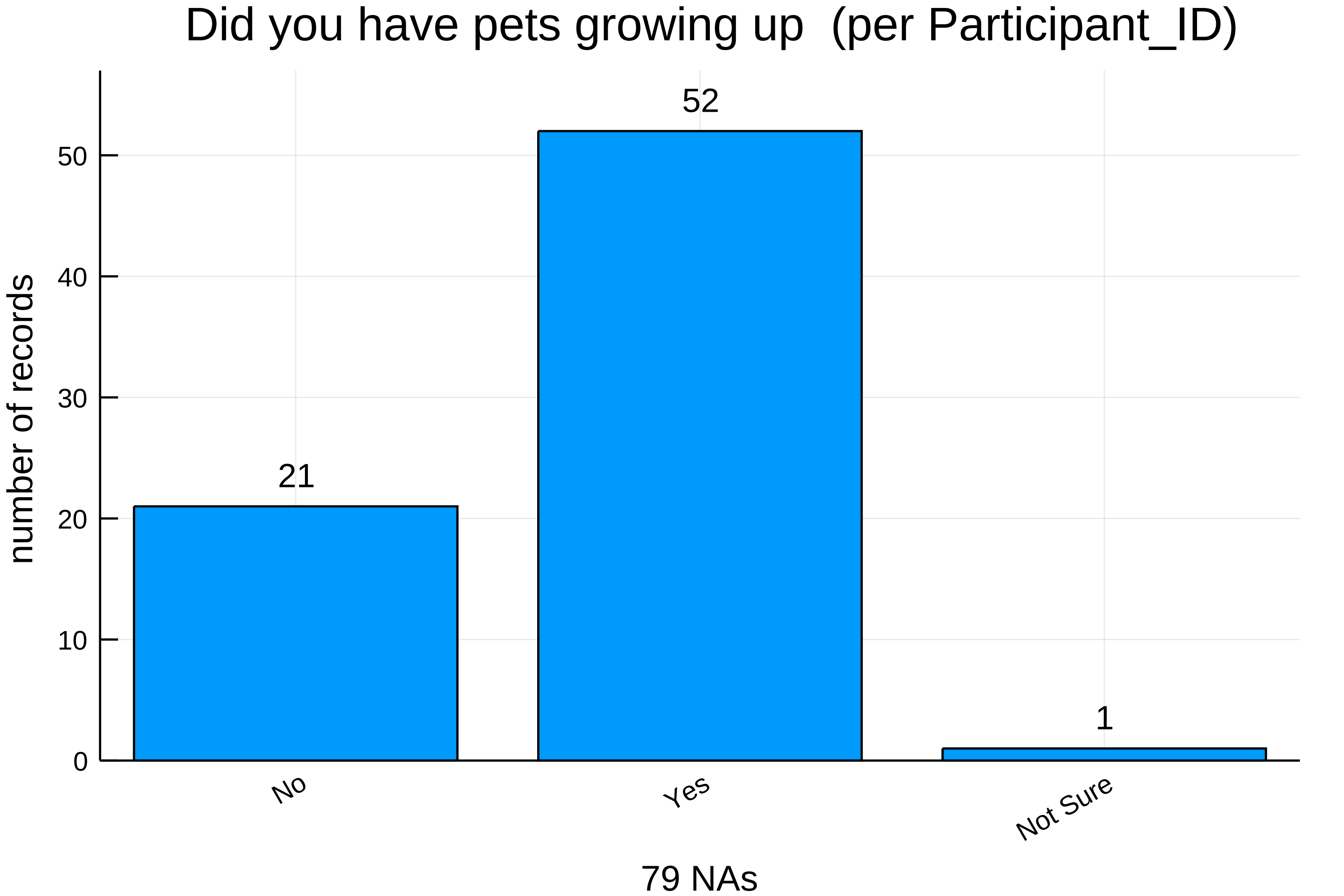

Did you have pets growing up (per Participant_ID)
number of records
0
10
20
30
40
50
21
52
1
No
Yes
Not Sure
79 NAs

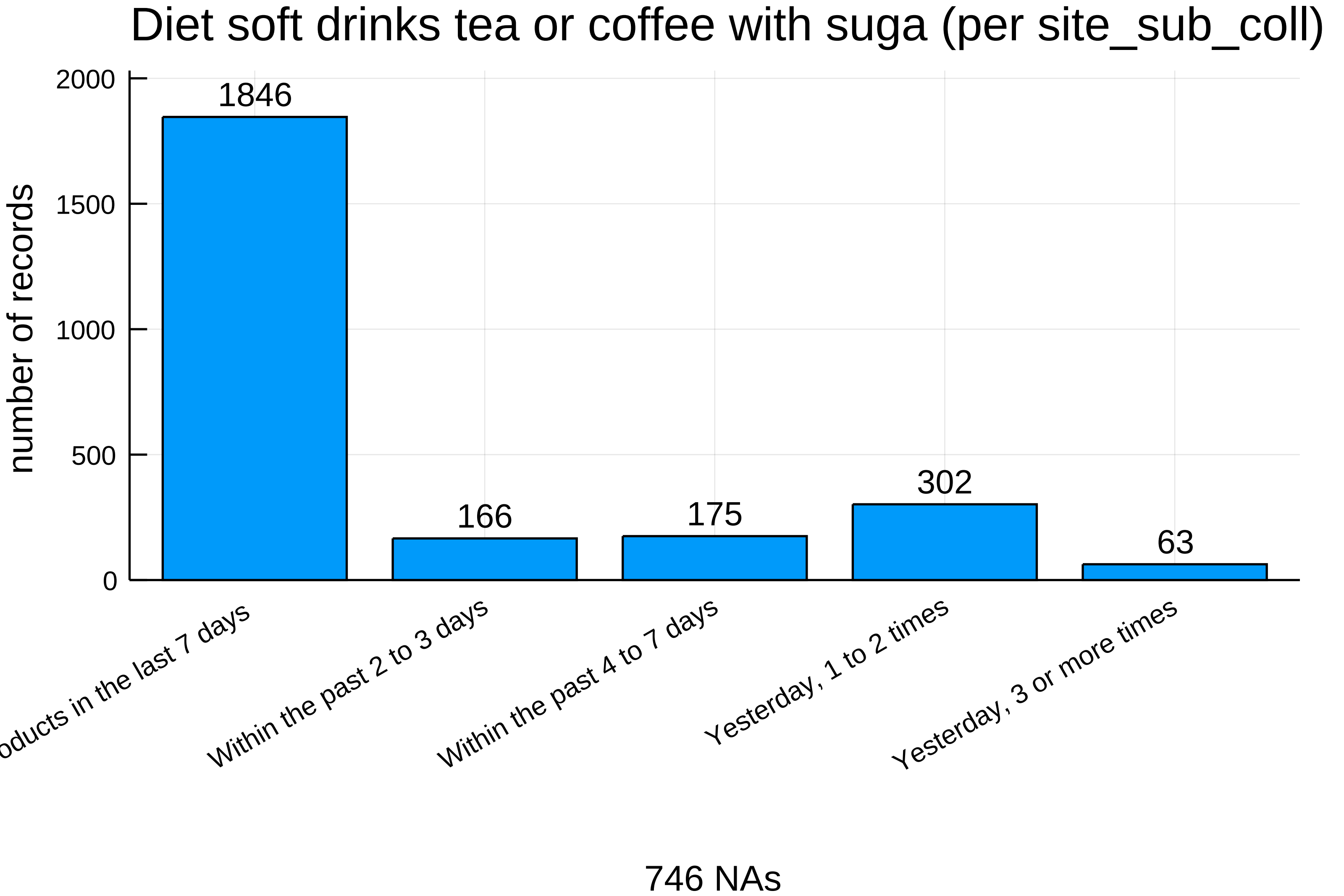
Diet soft drinks tea or coffee with suga (per site_sub_coll)
number of records
2000
1500
1000
500
0
1846
166
175
302
63
oducts in the last 7 days
Within the past 2 to 3 days
Within the past 4 to 7 days
Yesterday, 1 to 2 times
Yesterday, 3 or more times
746 NAs

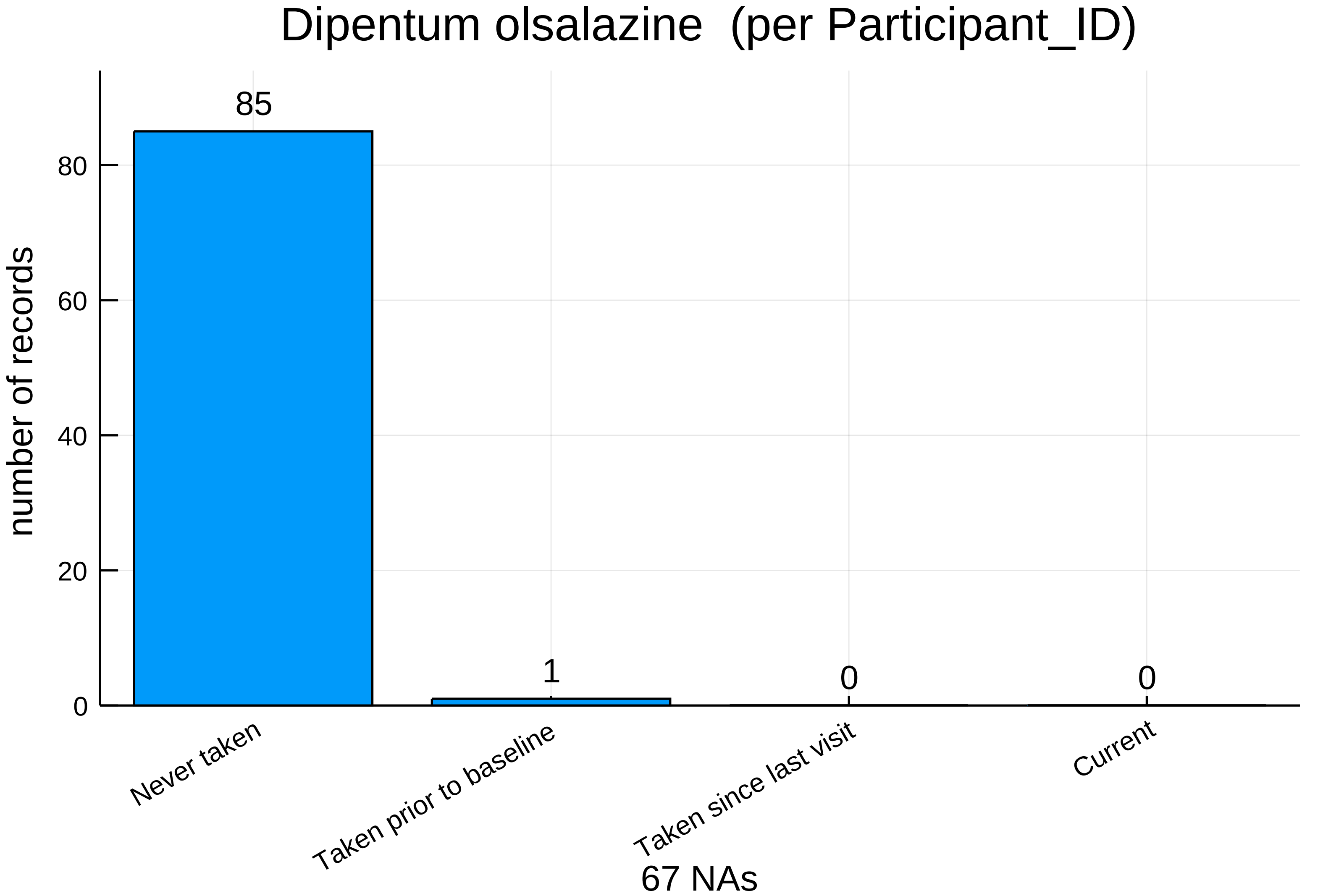
Dipentum olsalazine (per Participant_ID)
number of records
85
1
0
0
0
20
40
60
80
Never taken
Taken prior to baseline
Taken since last visit
Current
67 NAs

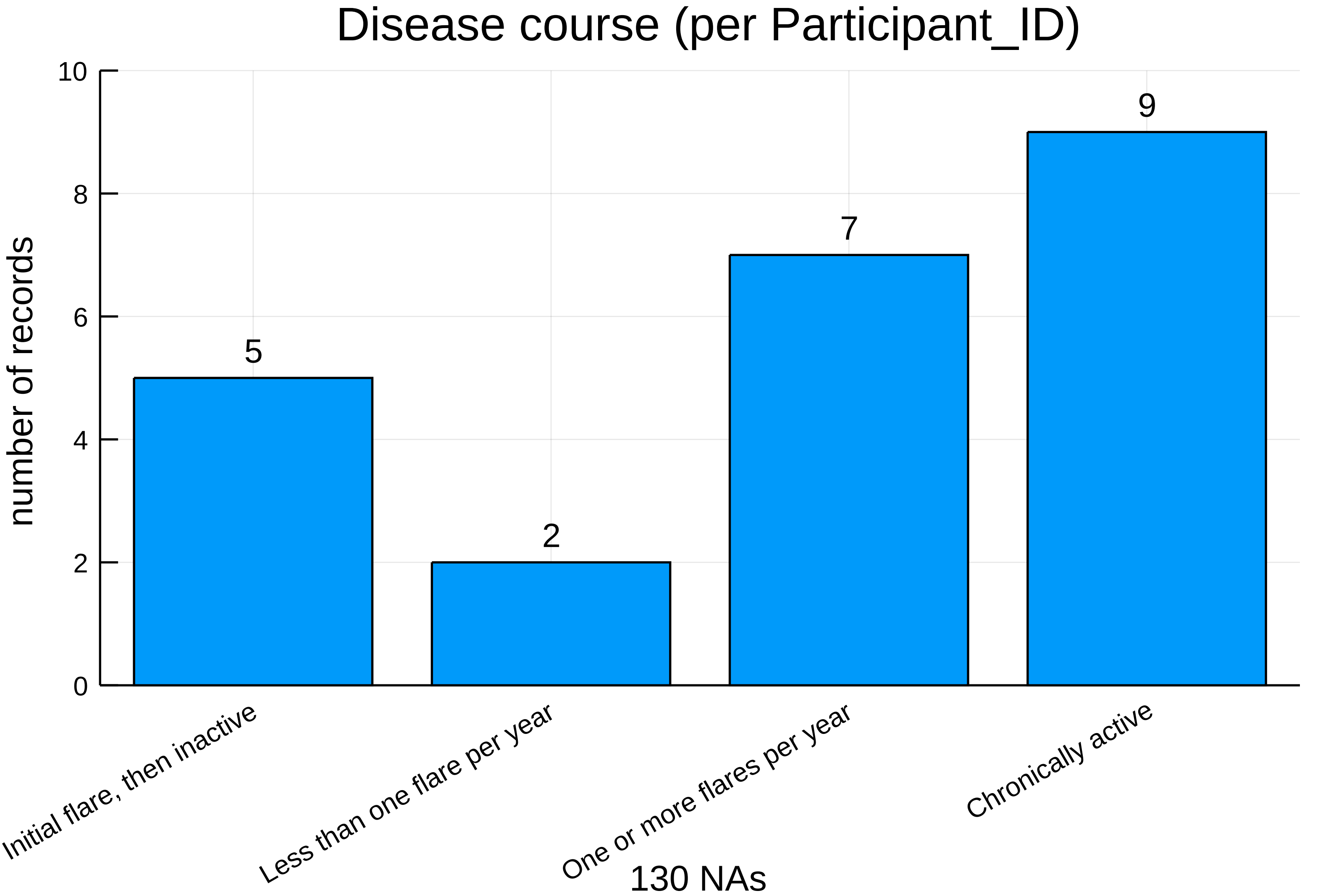
Disease course (per Participant_ID)
number of records
10
8
6
4
2
0
5
2
7
9
Initial flare, then inactive
Less than one flare per year
One or more flares per year
Chronically active
130 NAs

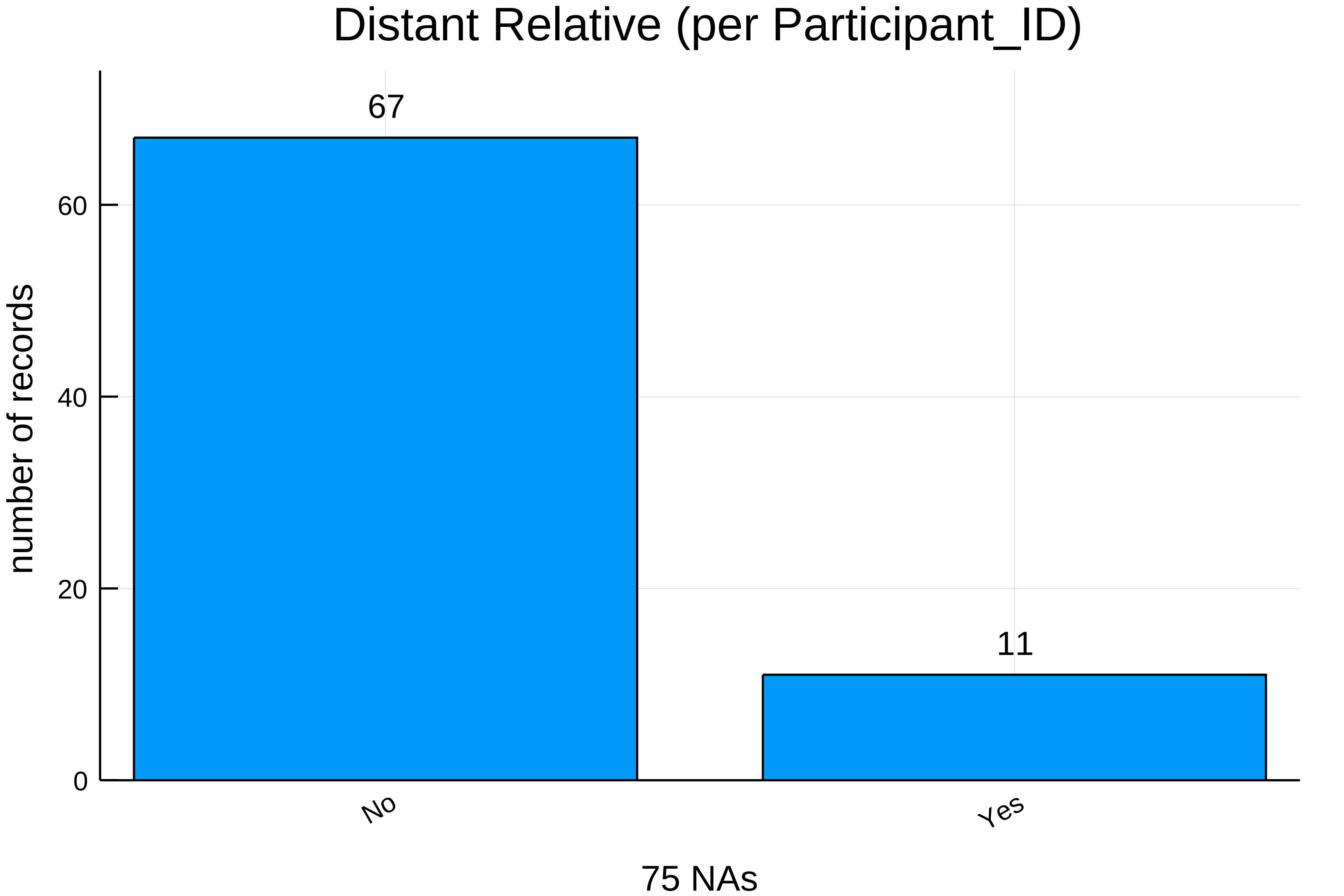
Distant Relative (per Participant_ID)
number of records
67
11
0
20
40
60
No
Yes
75 NAs

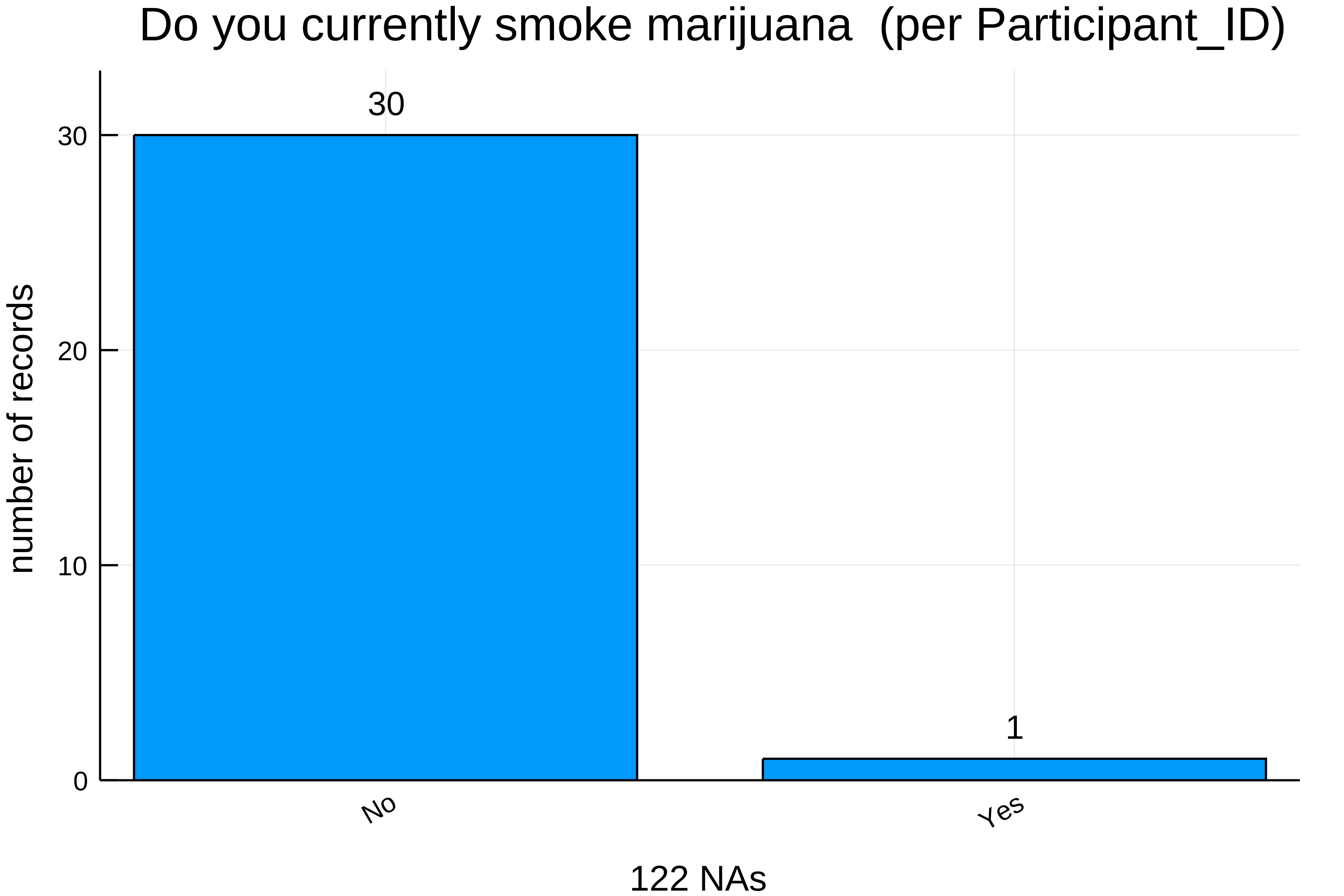
Do you currently smoke marijuana (per Participant_ID)
number of records
30
20
10
0
30
1
No
Yes
122 NAs

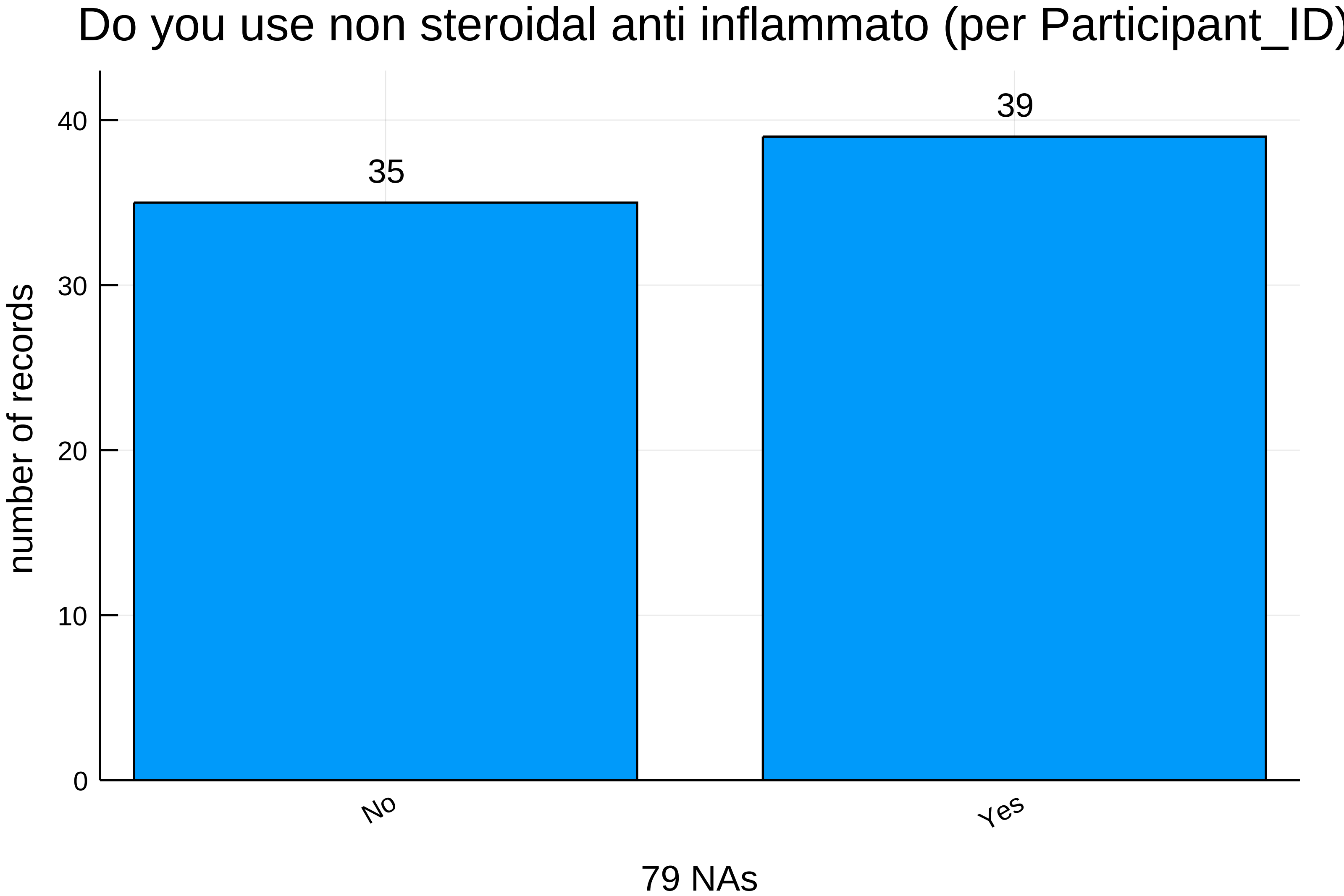
Do you use non steroidal anti inflammato (per Participant_ID)
number of records
40
30
20
10
0
35
39
No
Yes
79 NAs

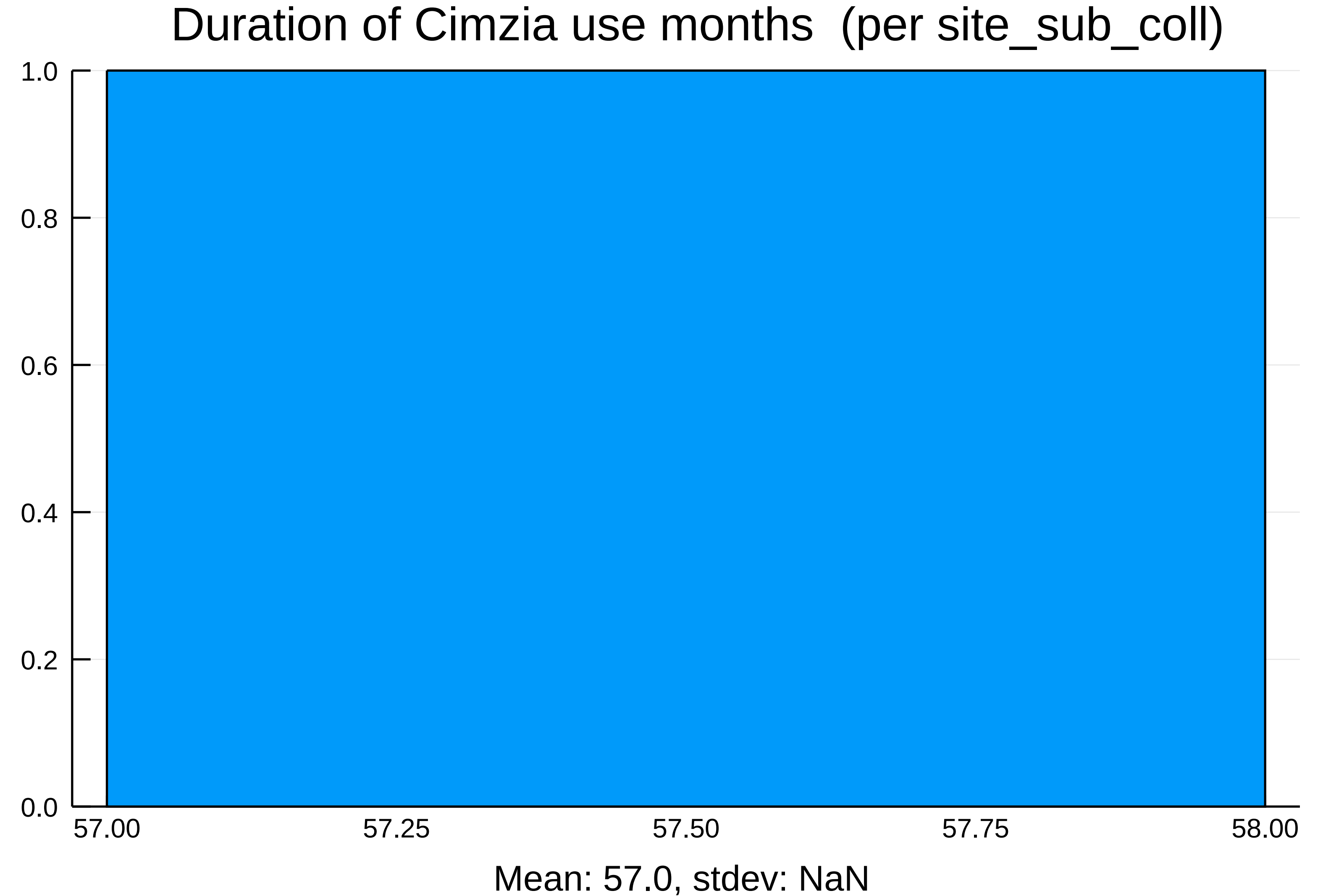

Duration of Cimzia use months (per site_sub_coll)
1.0
0.8
0.6
0.4
0.2
0.0
57.00
57.25
57.50
57.75
58.00
Mean: 57.0, stdev: NaN

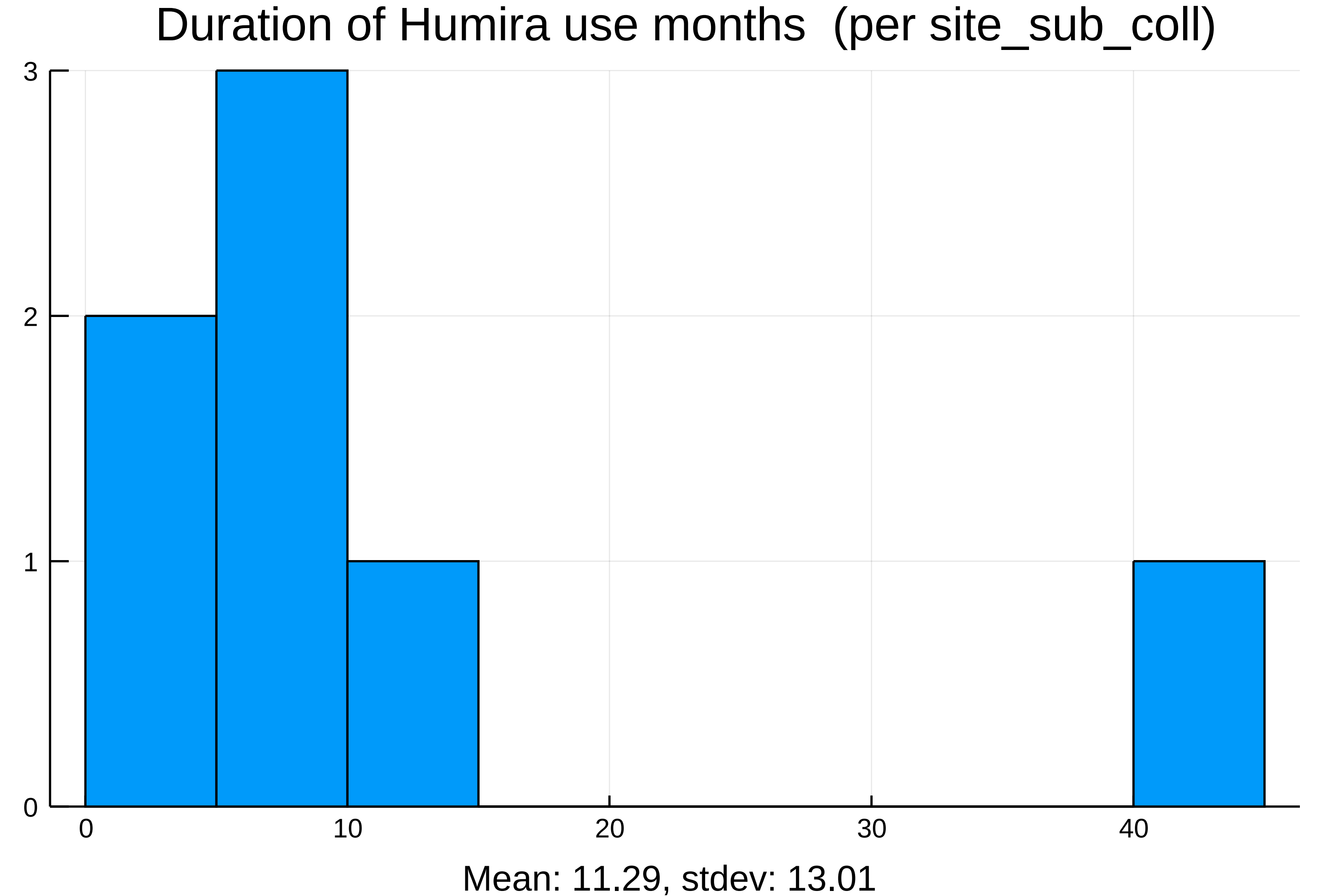
Duration of Humira use months (per site_sub_coll)
3
2
1
0
0
10
20
30
40
Mean: 11.29, stdev: 13.01

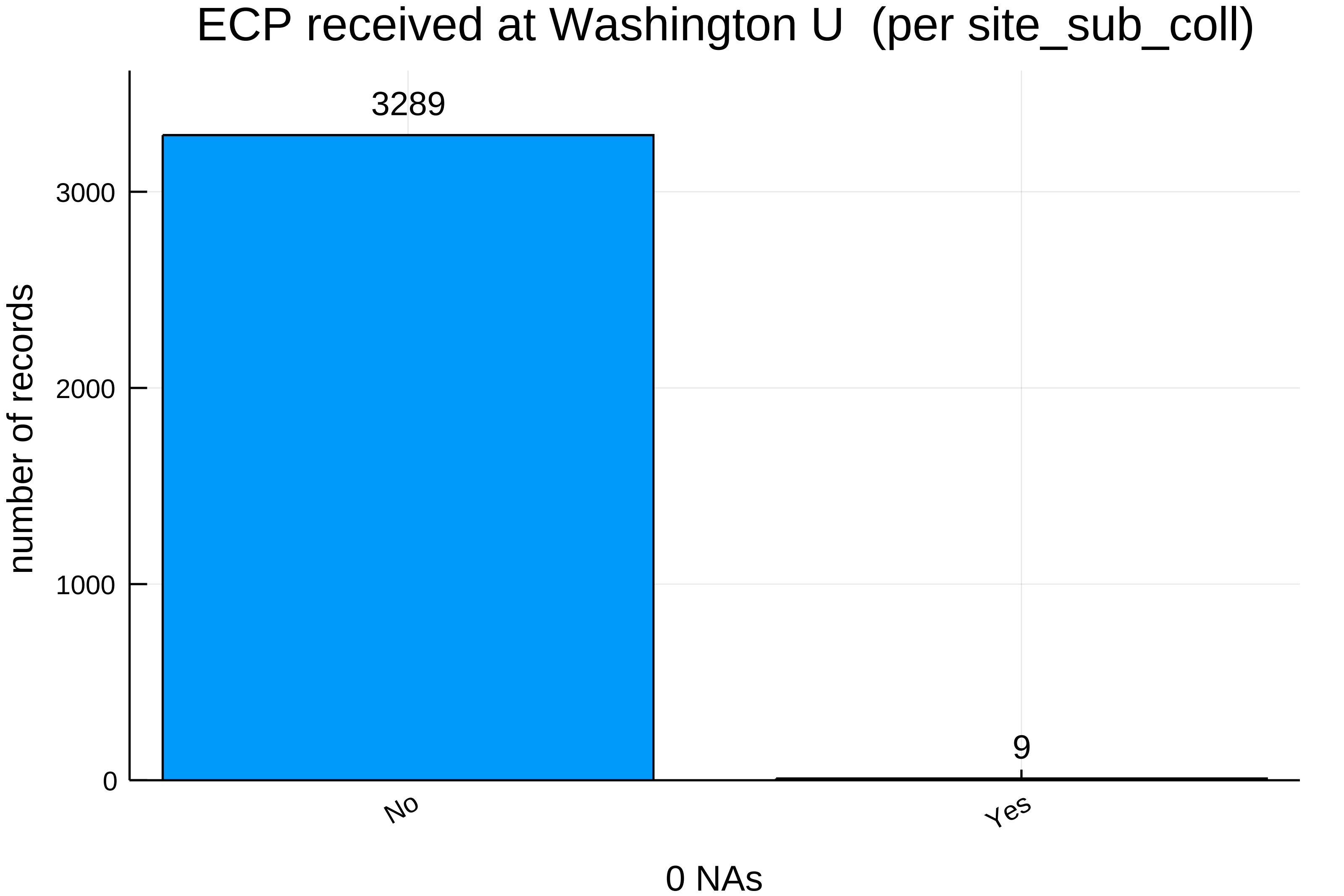
ECP received at Washington U (per site_sub_coll)
number of records
3289
9
3000
2000
1000
0
No
Yes
0 NAs

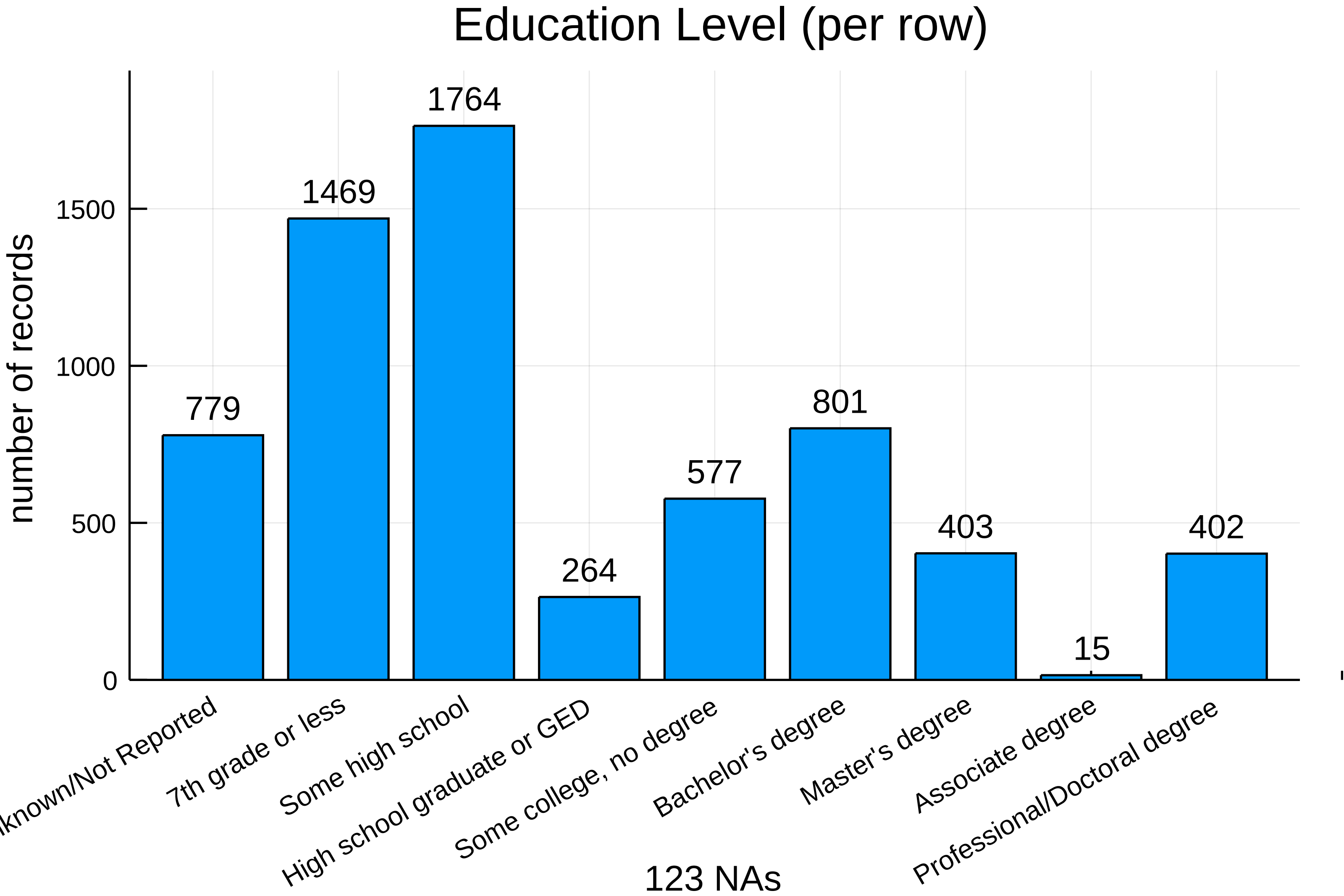

Education Level (per row)
number of records
0
500
1000
1500
779
1469
1764
264
577
801
403
15
402
known/Not Reported
7th grade or less
Some high school
High school graduate or GED
Some college, no degree
Bachelor's degree
Master's degree
Associate degree
Professional/Doctoral degree
123 NAs

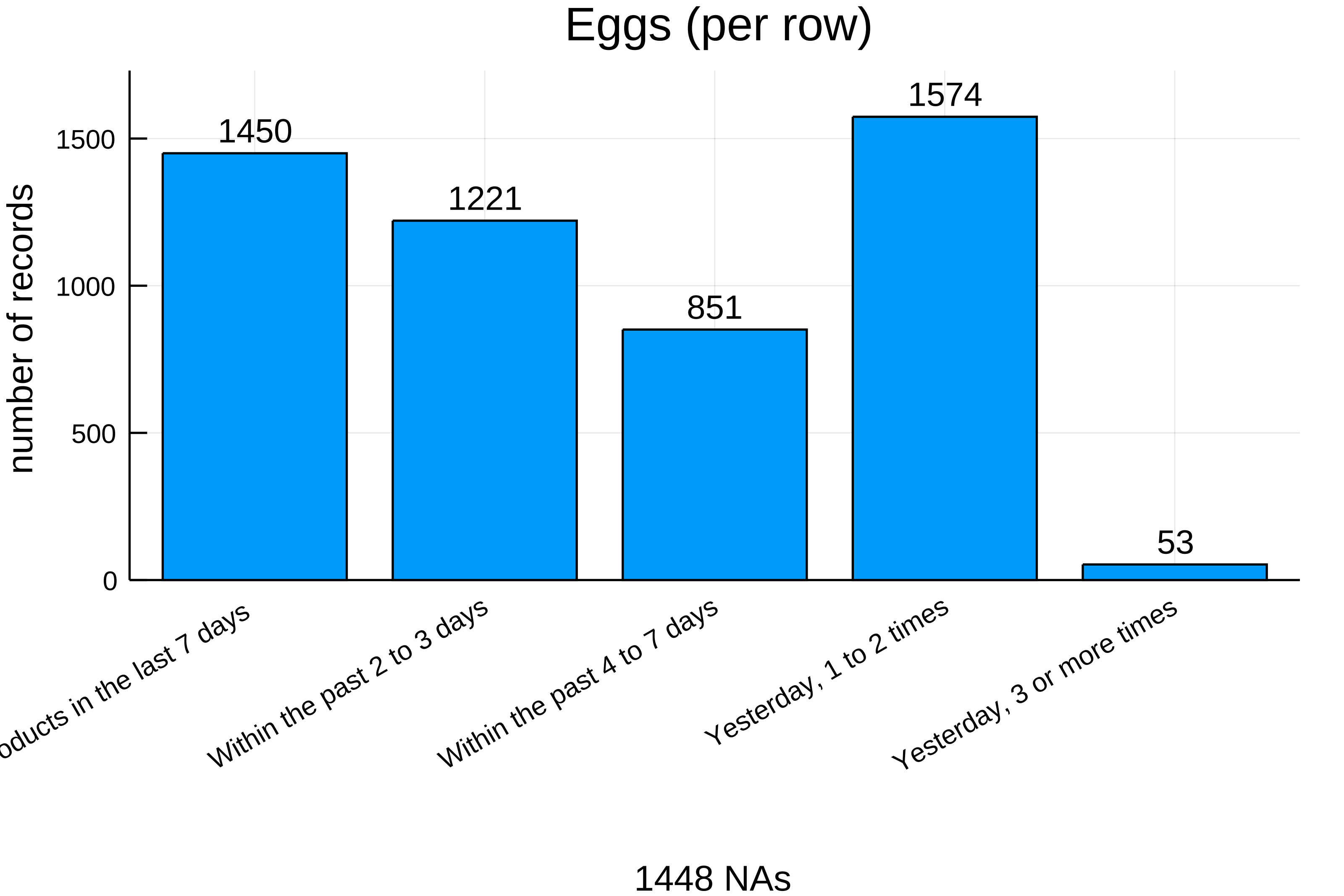
Eggs (per row)
number of records
1500
1000
500
0
1450
1221
851
1574
53
oducts in the last 7 days
Within the past 2 to 3 days
Within the past 4 to 7 days
Yesterday, 1 to 2 times
Yesterday, 3 or more times
1448 NAs

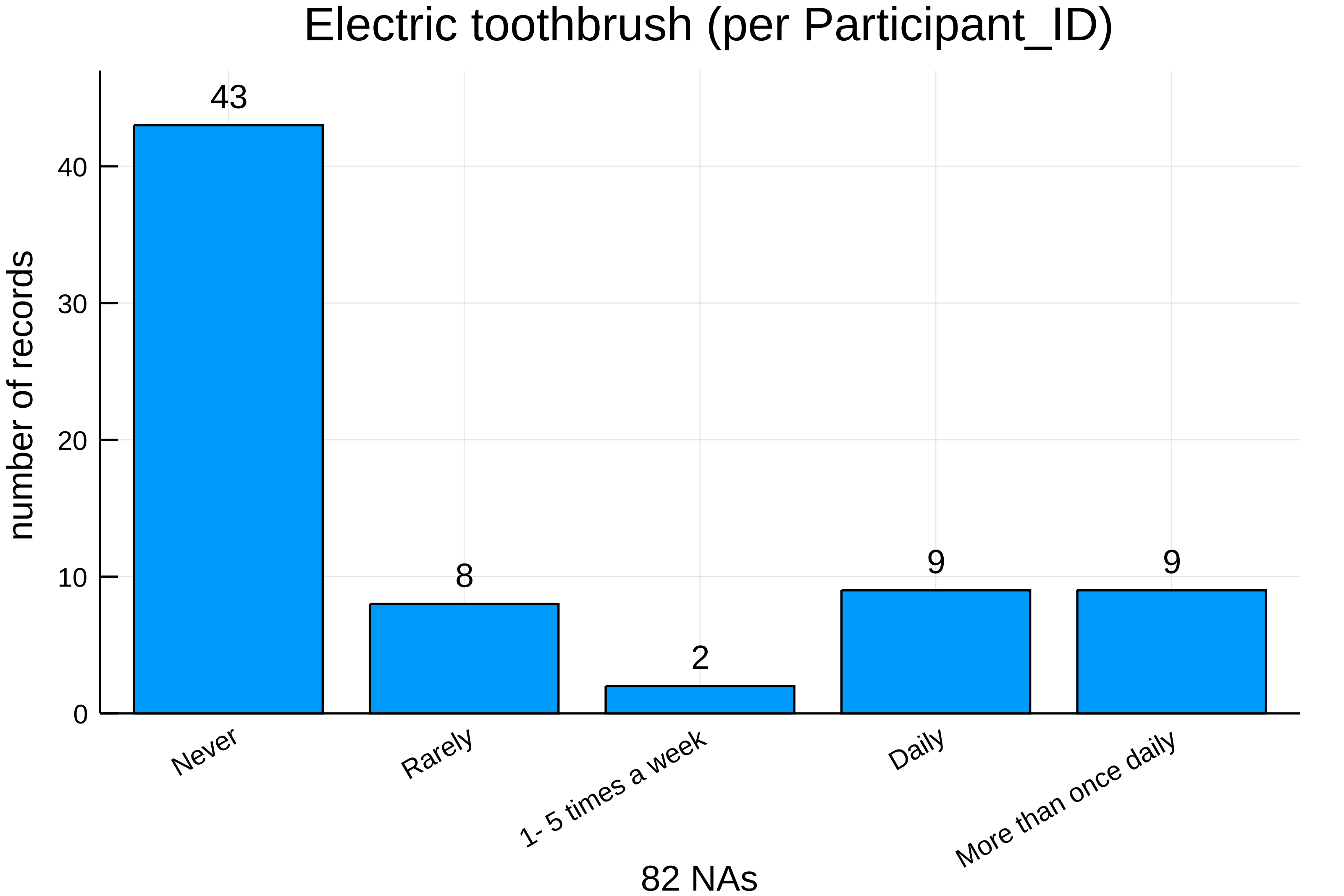
Electric toothbrush (per Participant_ID)
number of records
0
10
20
30
40
43
8
2
9
9
Never
Rarely
1- 5 times a week
Daily
More than once daily
82 NAs

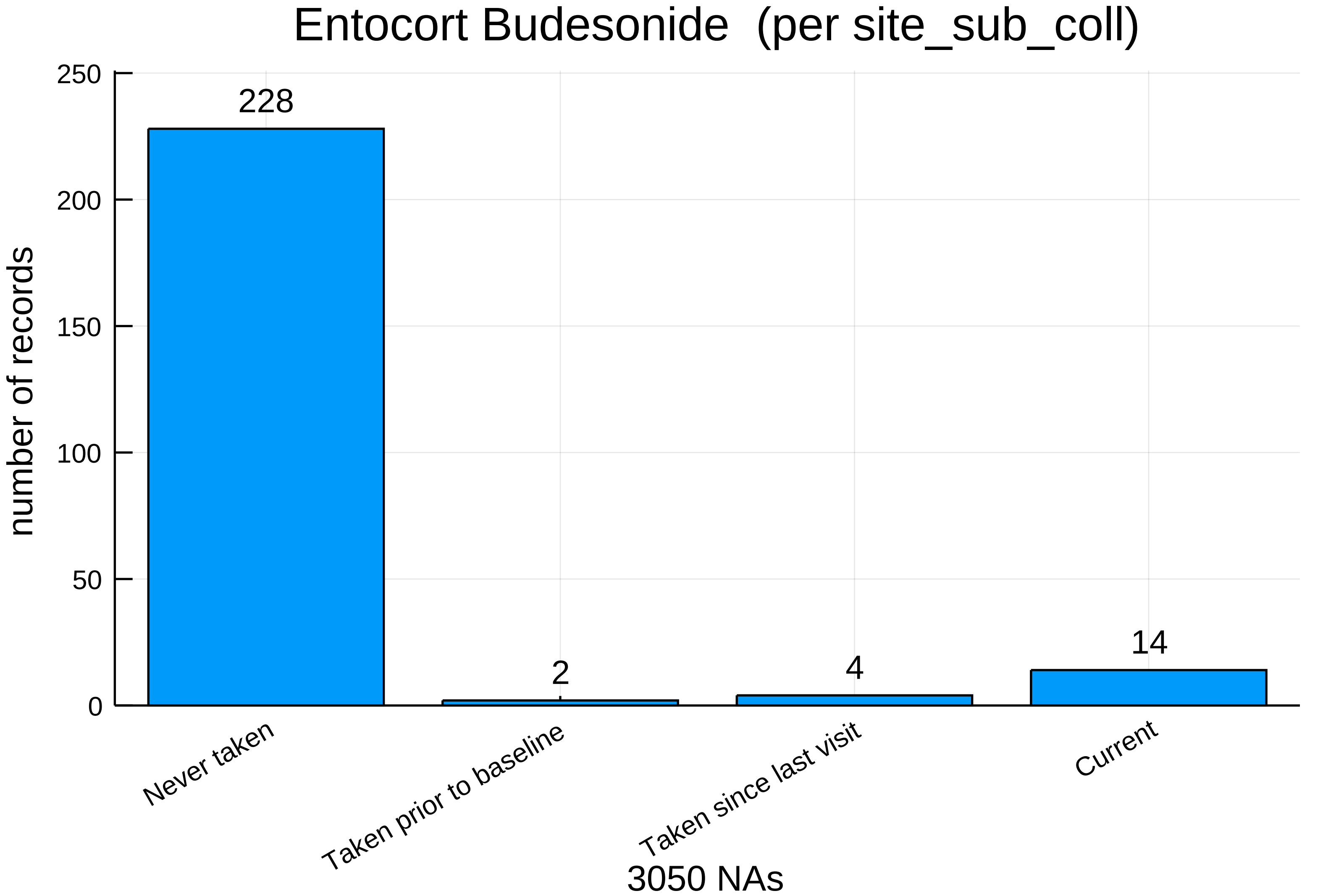

Entocort Budesonide (per site_sub_coll)
number of records
250
200
150
100
50
0
228
2
4
14
Never taken
Taken prior to baseline
Taken since last visit
Current
3050 NAs

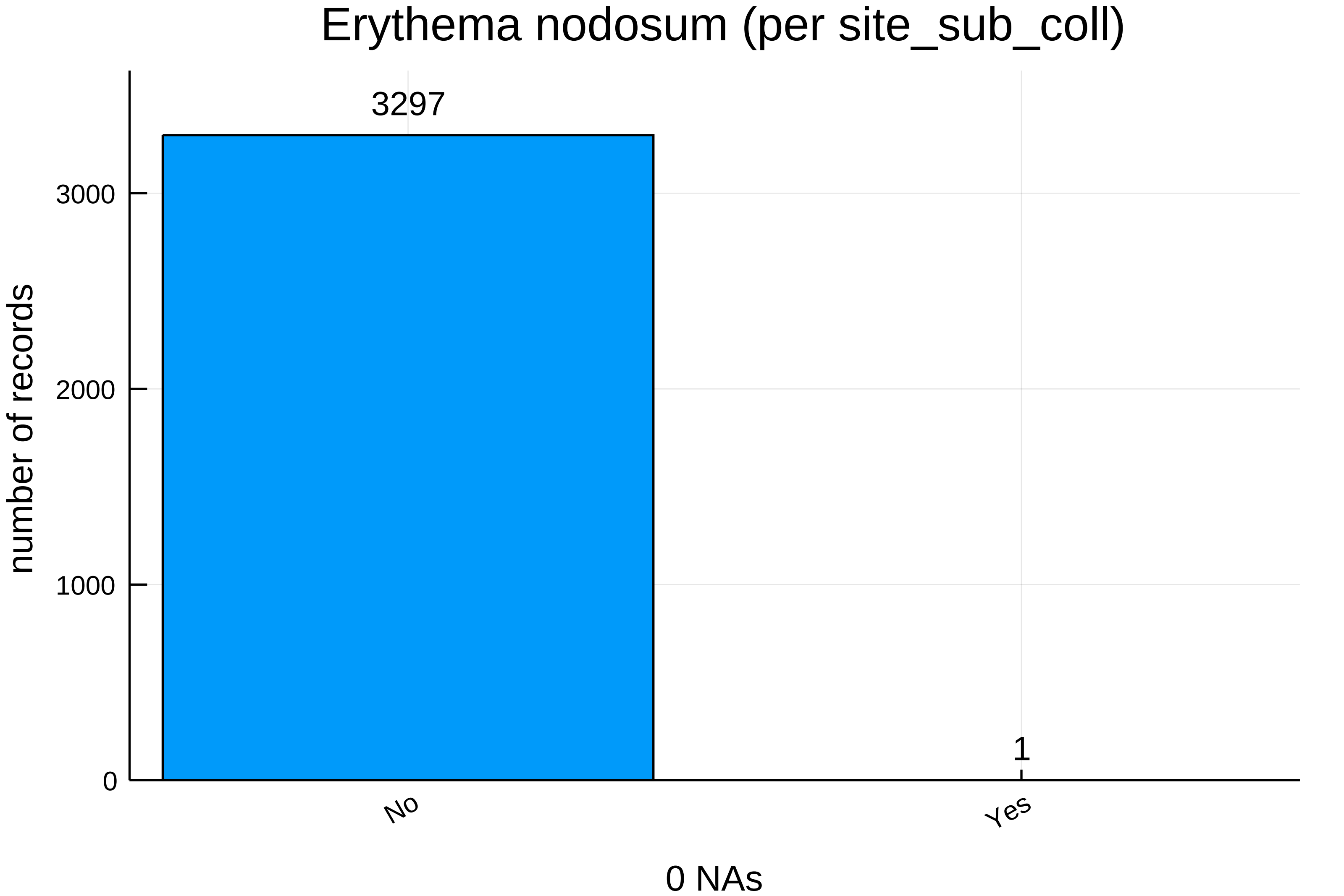
Erythema nodosum (per site_sub_coll)
number of records
3297
1
0
1000
2000
3000
No
Yes
0 NAs

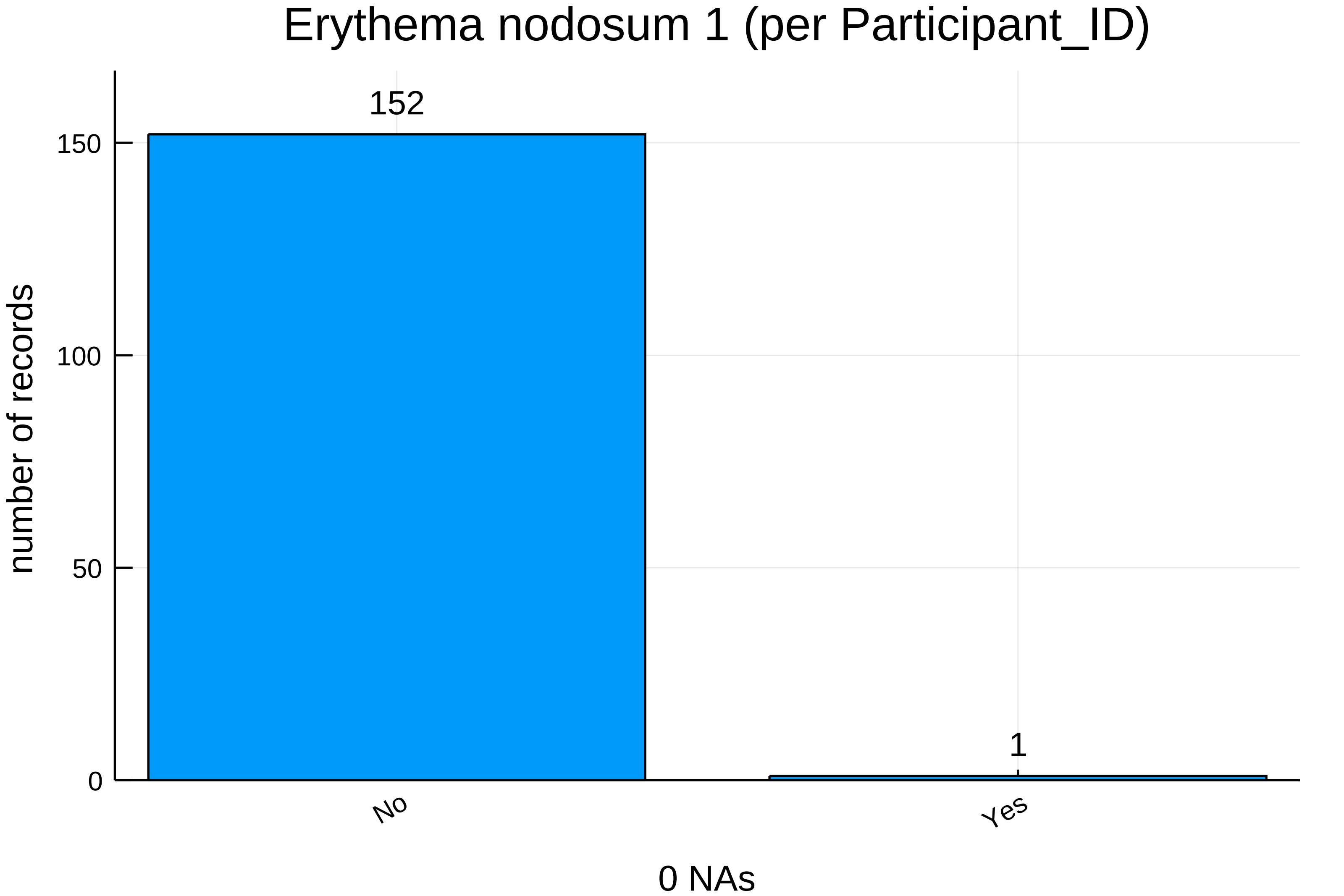
Erythema nodosum 1 (per Participant_ID)
152
1
150
100
50
0
number of records
No
Yes
0 NAs

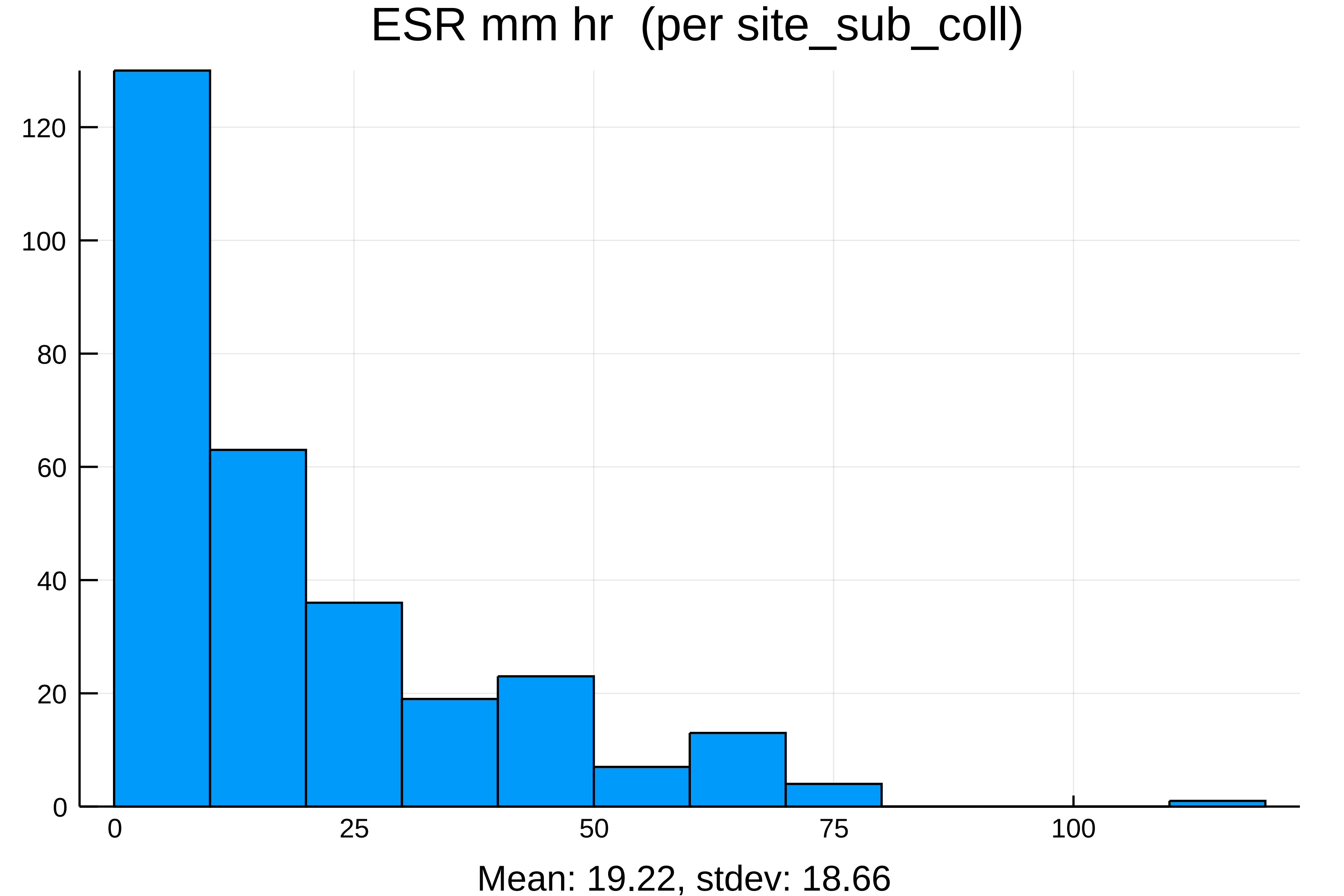
ESR mm hr (per site_sub_coll)
0
20
40
60
80
100
120
0
25
50
75
100
Mean: 19.22, stdev: 18.66

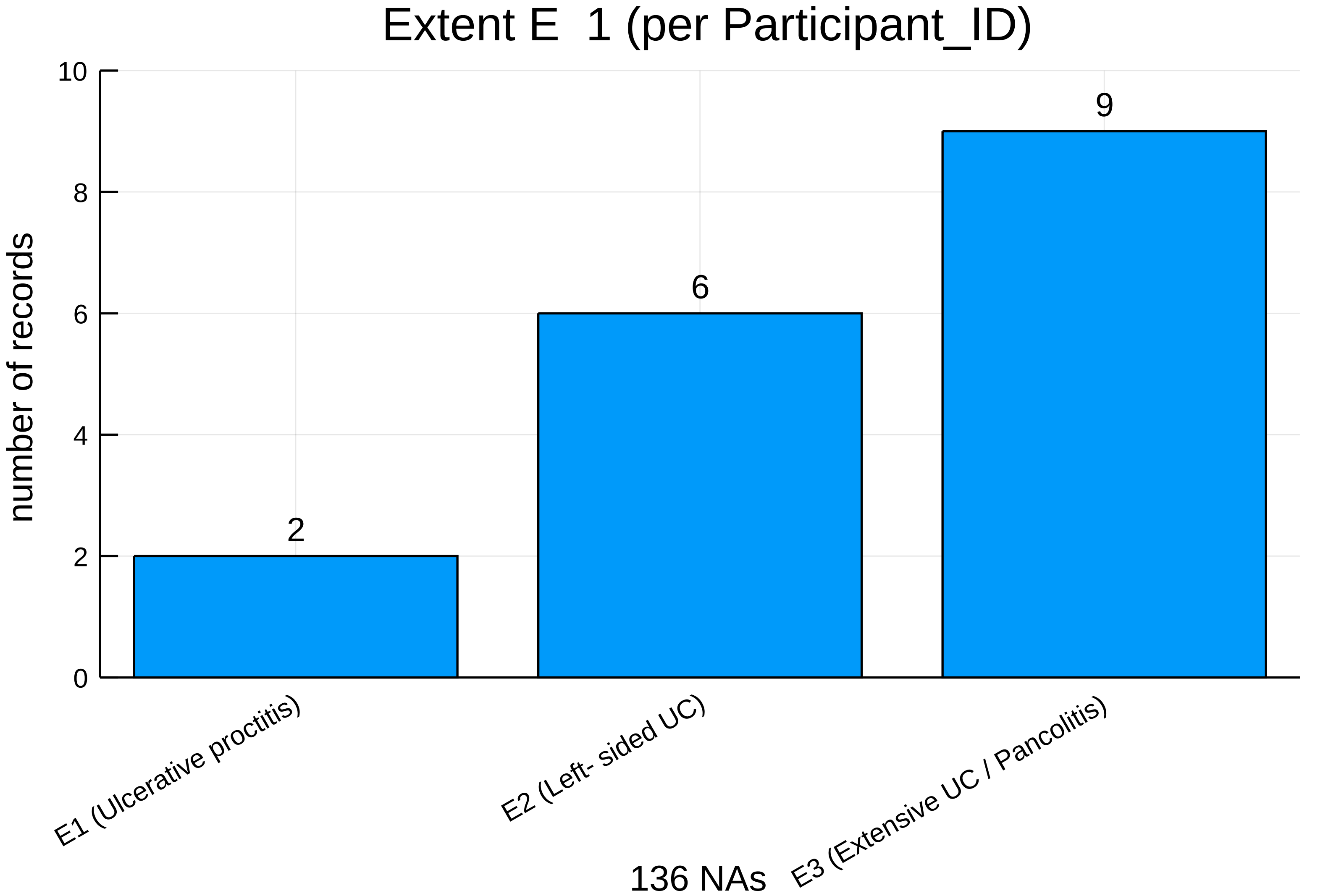
Extent E 1 (per Participant_ID)
number of records
0
2
4
6
8
10
2
6
9
E1 (Ulcerative proctitis)
E2 (Left- sided UC)
E3 (Extensive UC / Pancolitis)
136 NAs

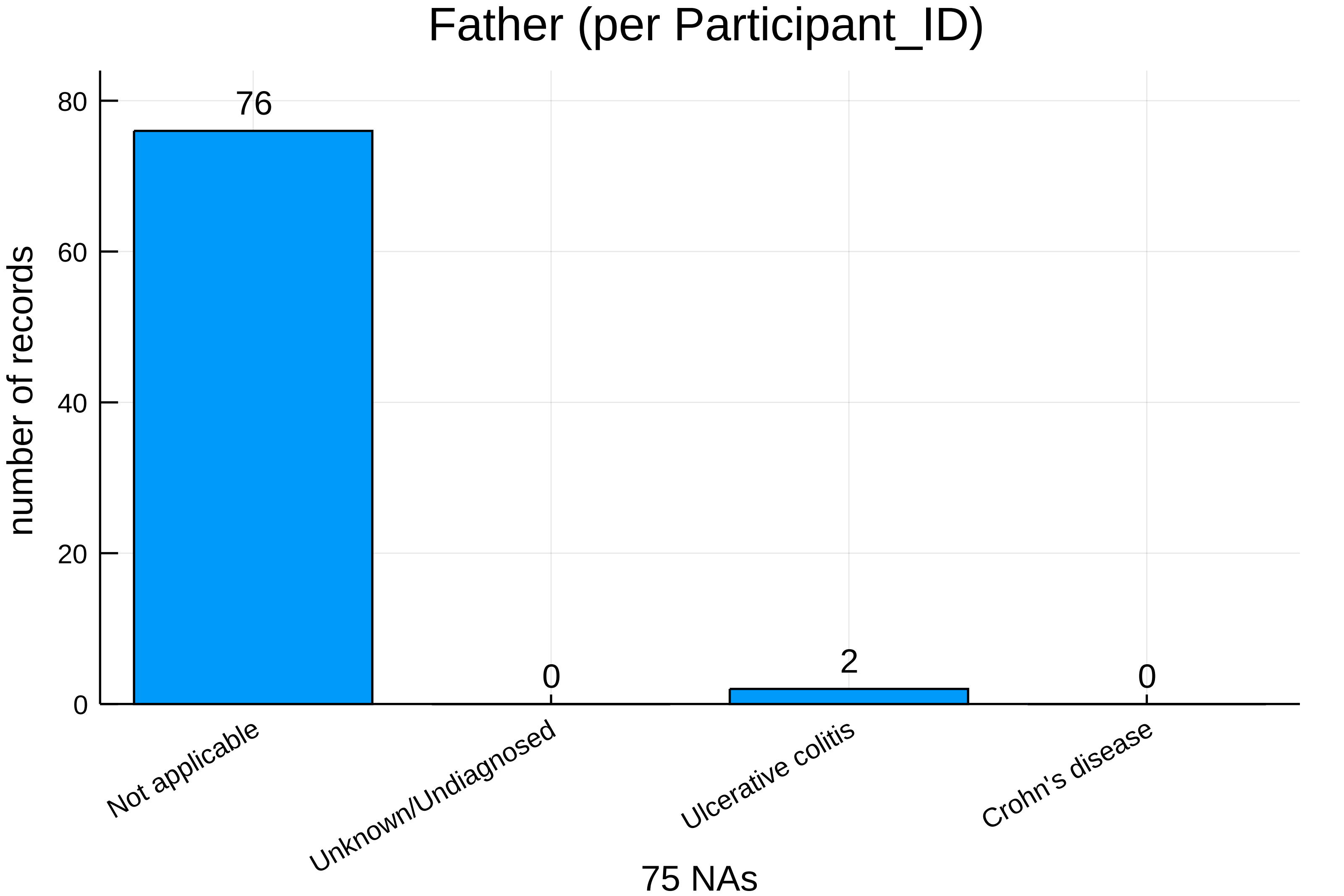
Father (per Participant_ID)
number of records
0
20
40
60
80
76
0
2
0
Not applicable
Unknown/Undiagnosed
Ulcerative colitis
Crohn's disease
75 NAs

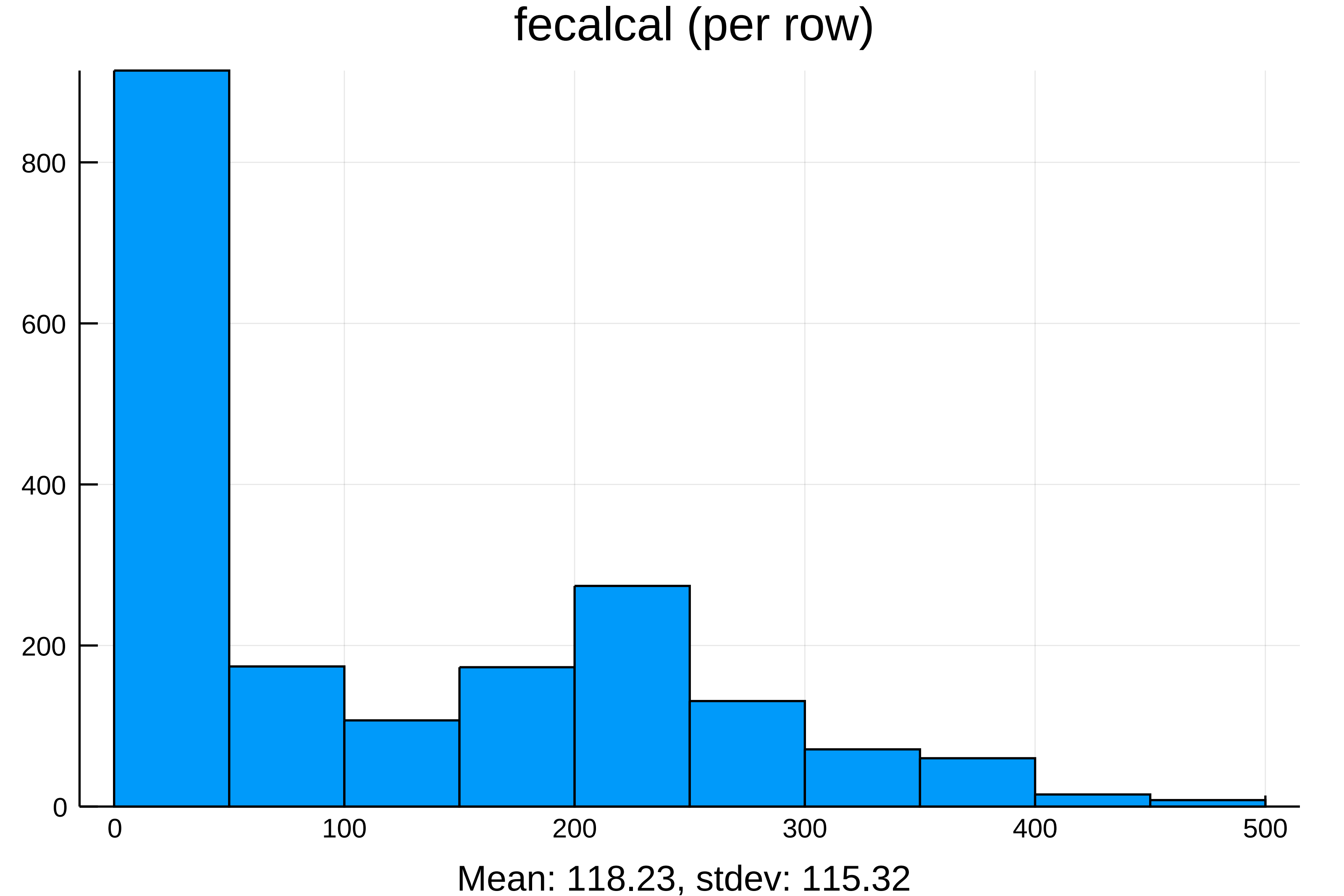
fecalcal (per row)
800
600
400
200
0
0
100
200
300
400
500
Mean: 118.23, stdev: 115.32

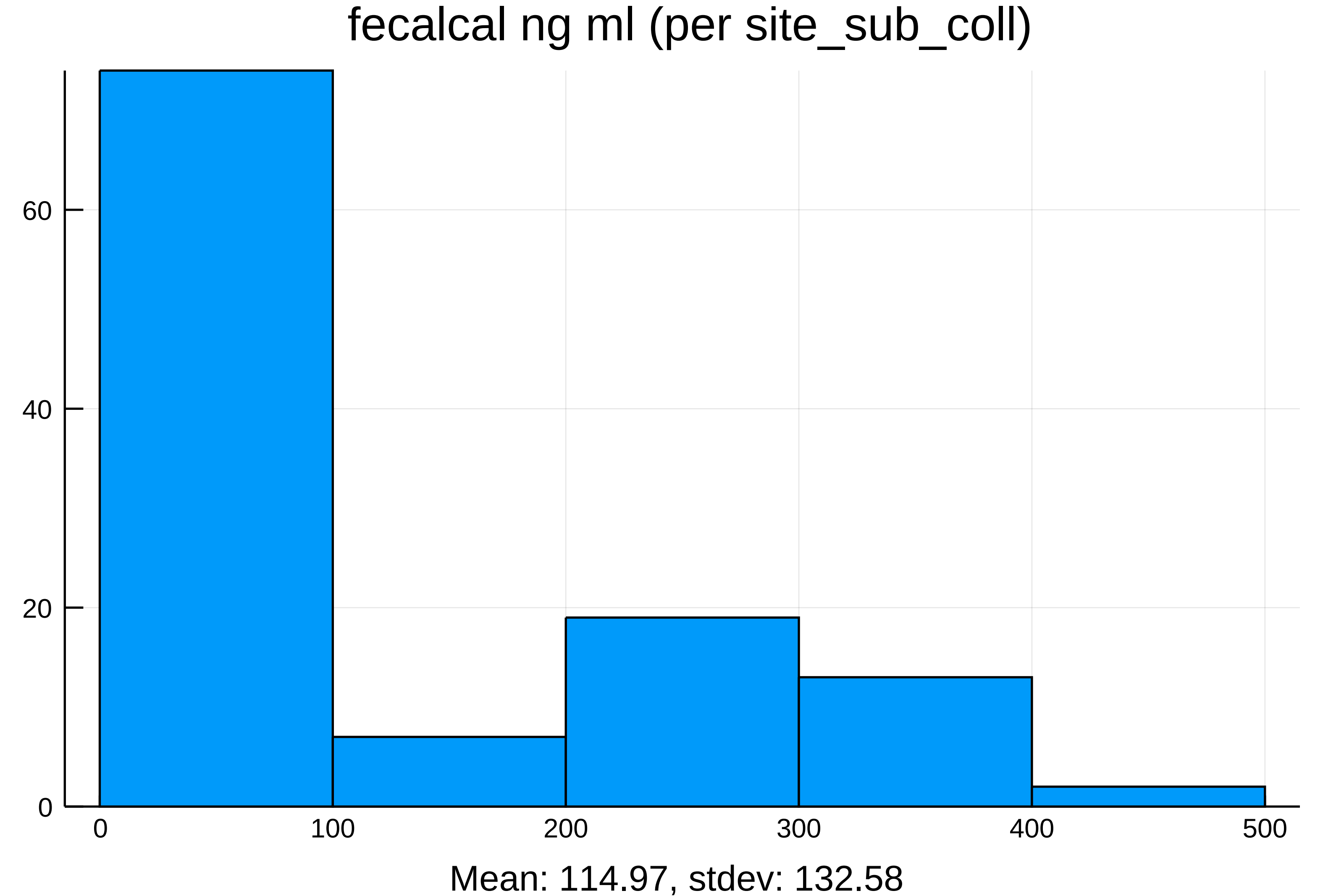
fecalcal ng ml (per site_sub_coll)
60
40
20
0
0
100
200
300
400
500
Mean: 114.97, stdev: 132.58

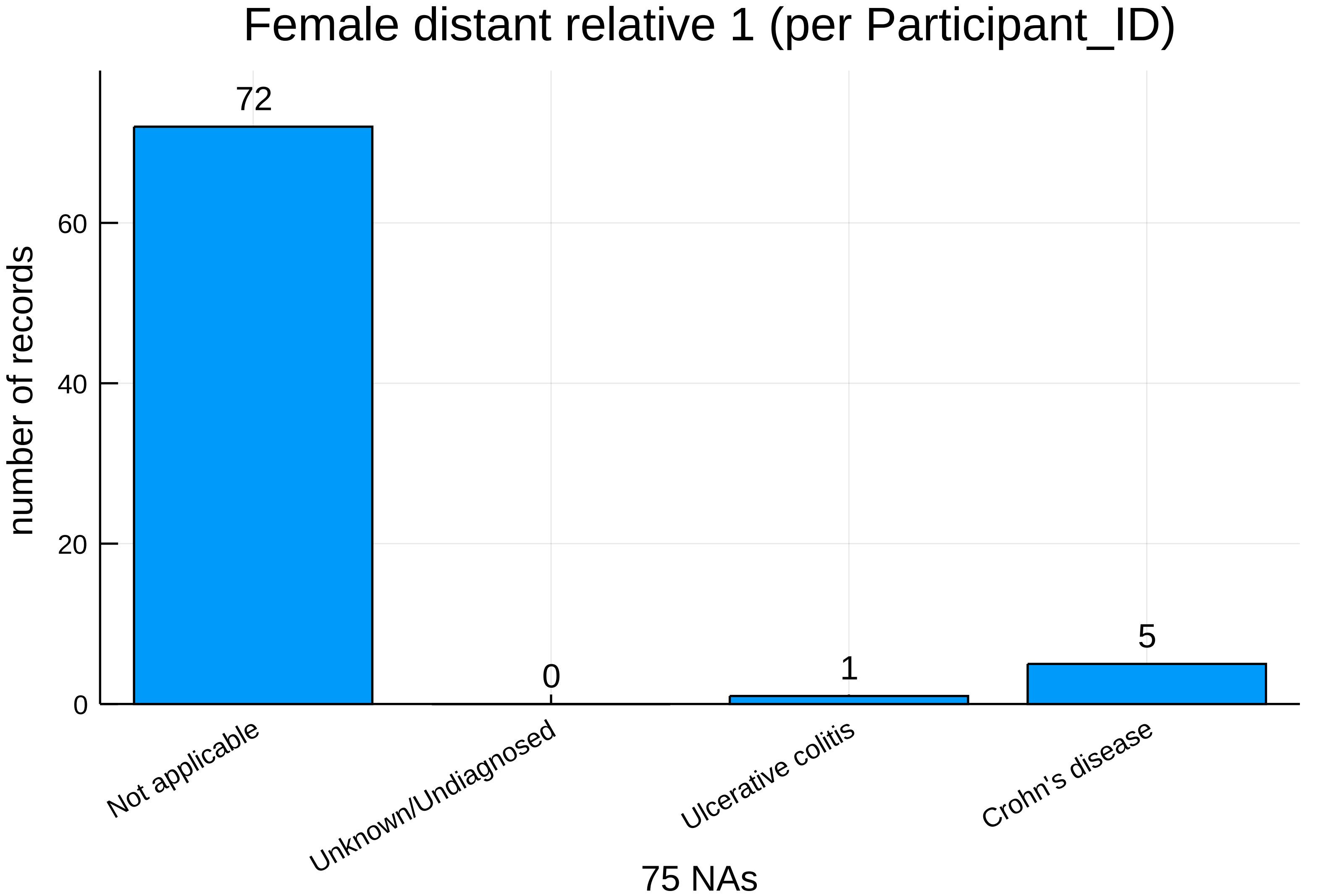

Female distant relative 1 (per Participant_ID)
number of records
0
20
40
60
72
0
1
5
Not applicable
Unknown/Undiagnosed
Ulcerative colitis
Crohn's disease
75 NAs

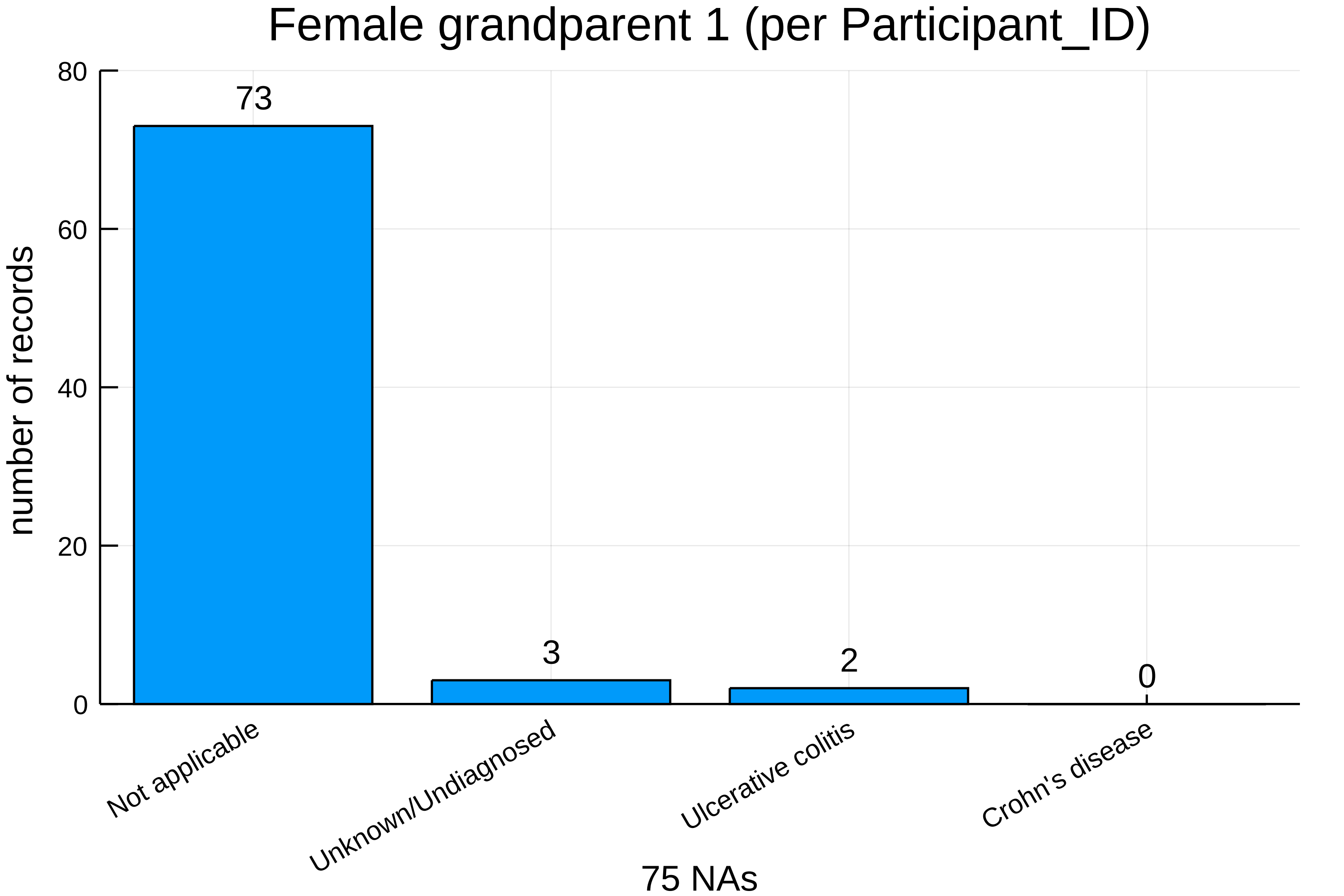
Female grandparent 1 (per Participant_ID)
number of records
0
20
40
60
80
73
3
2
0
Not applicable
Unknown/Undiagnosed
Ulcerative colitis
Crohn's disease
75 NAs

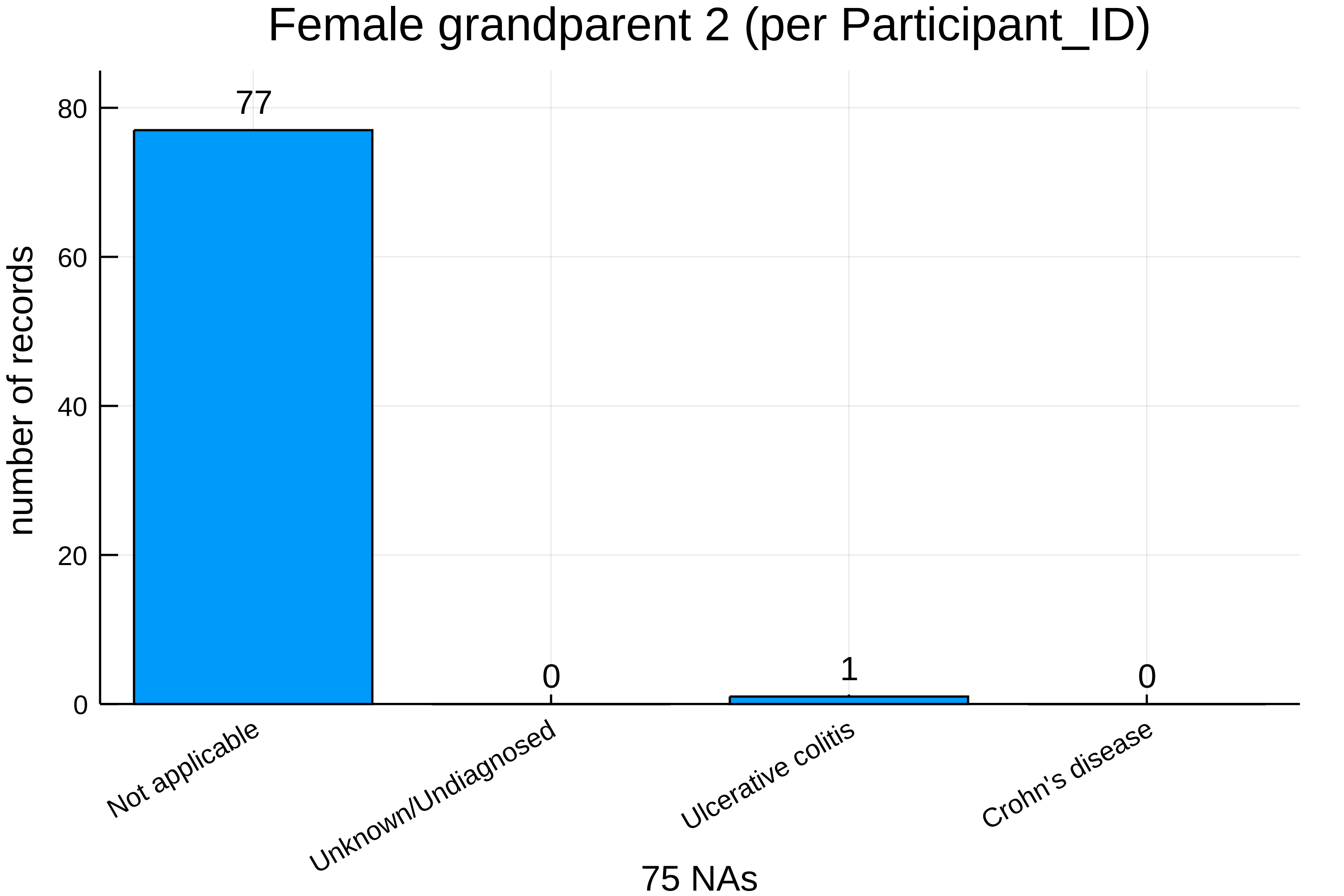
Female grandparent 2 (per Participant_ID)
number of records
0
20
40
60
80
77
0
1
0
Not applicable
Unknown/Undiagnosed
Ulcerative colitis
Crohn's disease
75 NAs

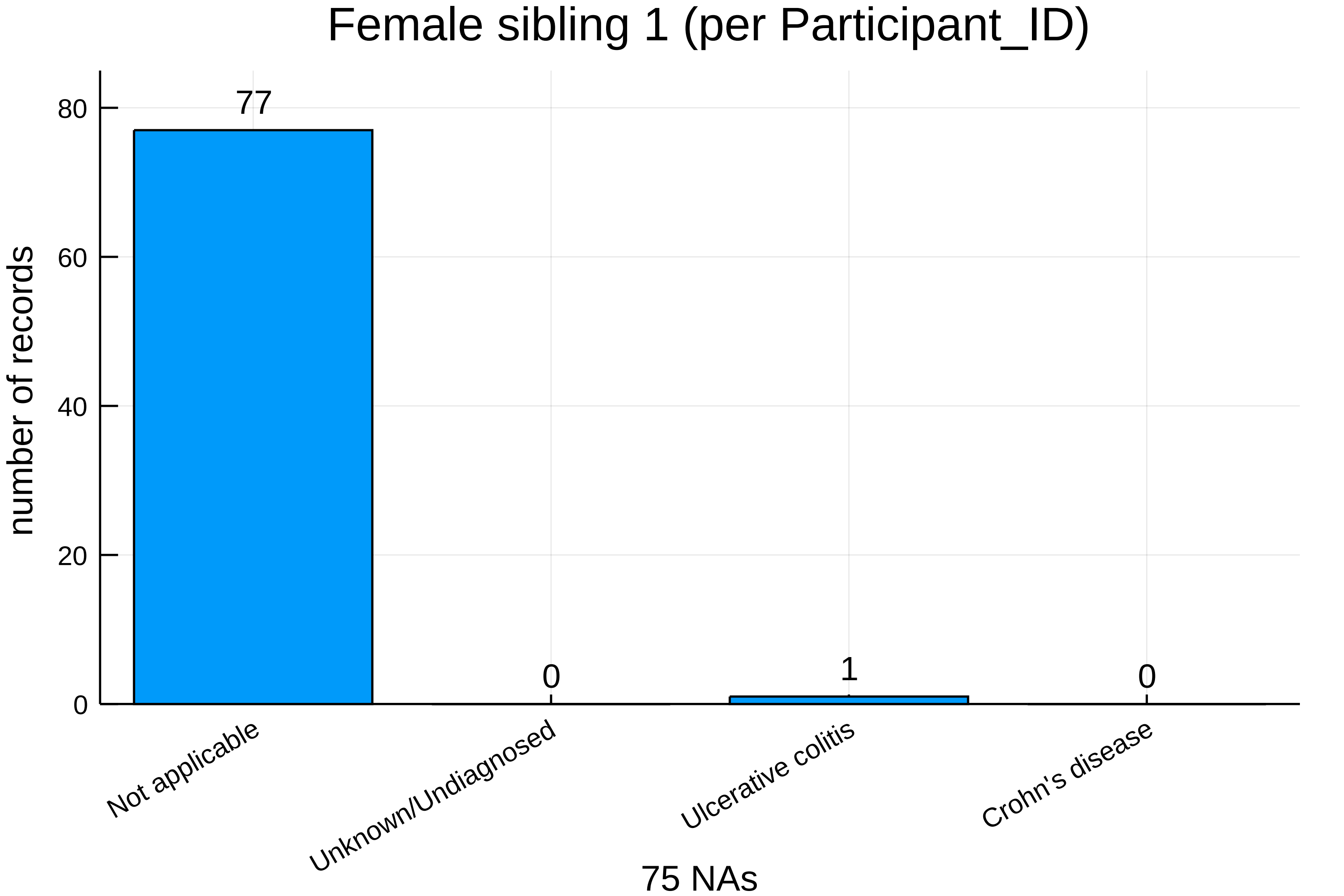
Female sibling 1 (per Participant_ID)
number of records
0
20
40
60
80
77
0
1
0
Not applicable
Unknown/Undiagnosed
Ulcerative colitis
Crohn's disease
75 NAs

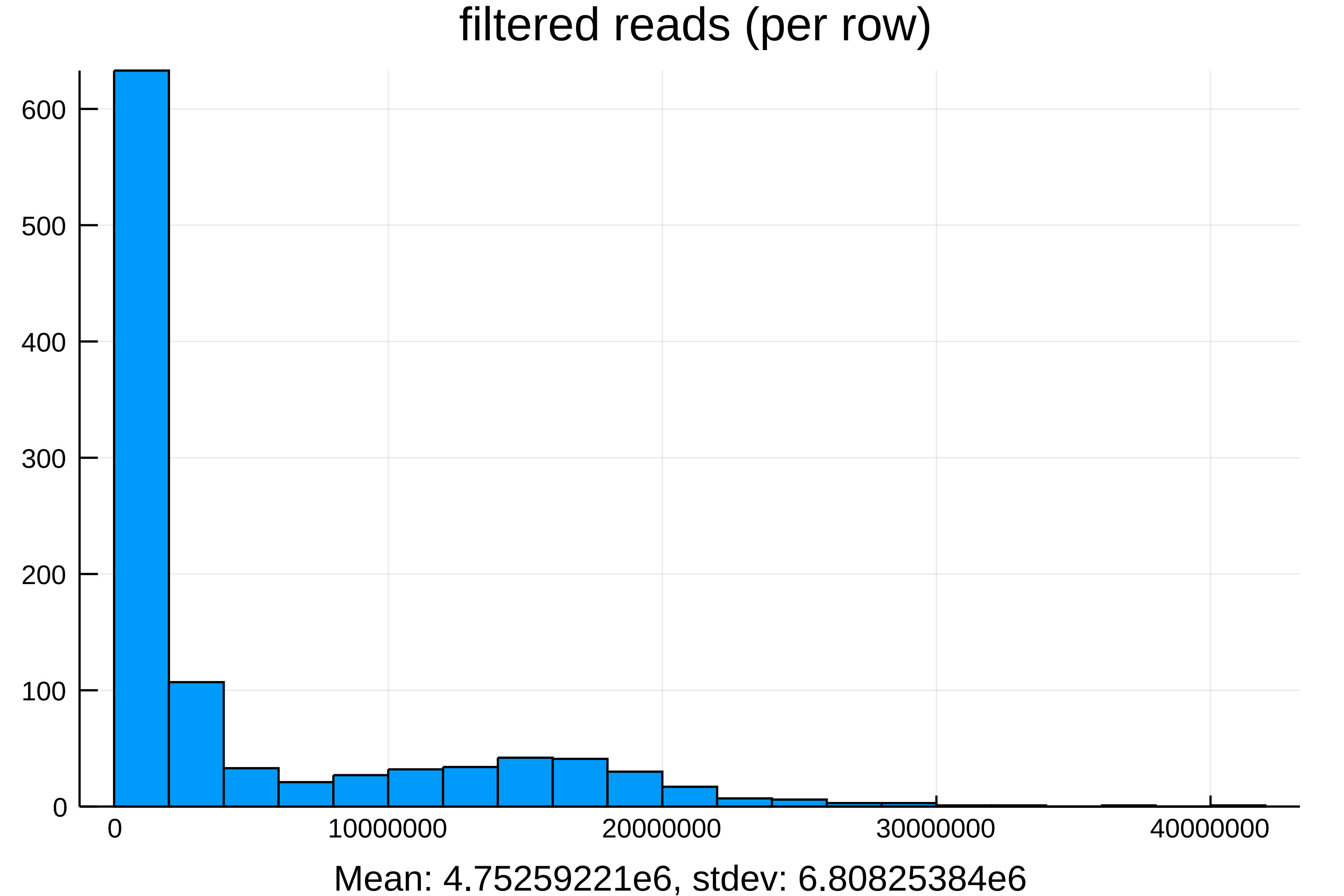
filtered reads (per row)
0
100
200
300
400
500
600
0
10000000
20000000
30000000
40000000
Mean: 4.75259221e6, stdev: 6.80825384e6

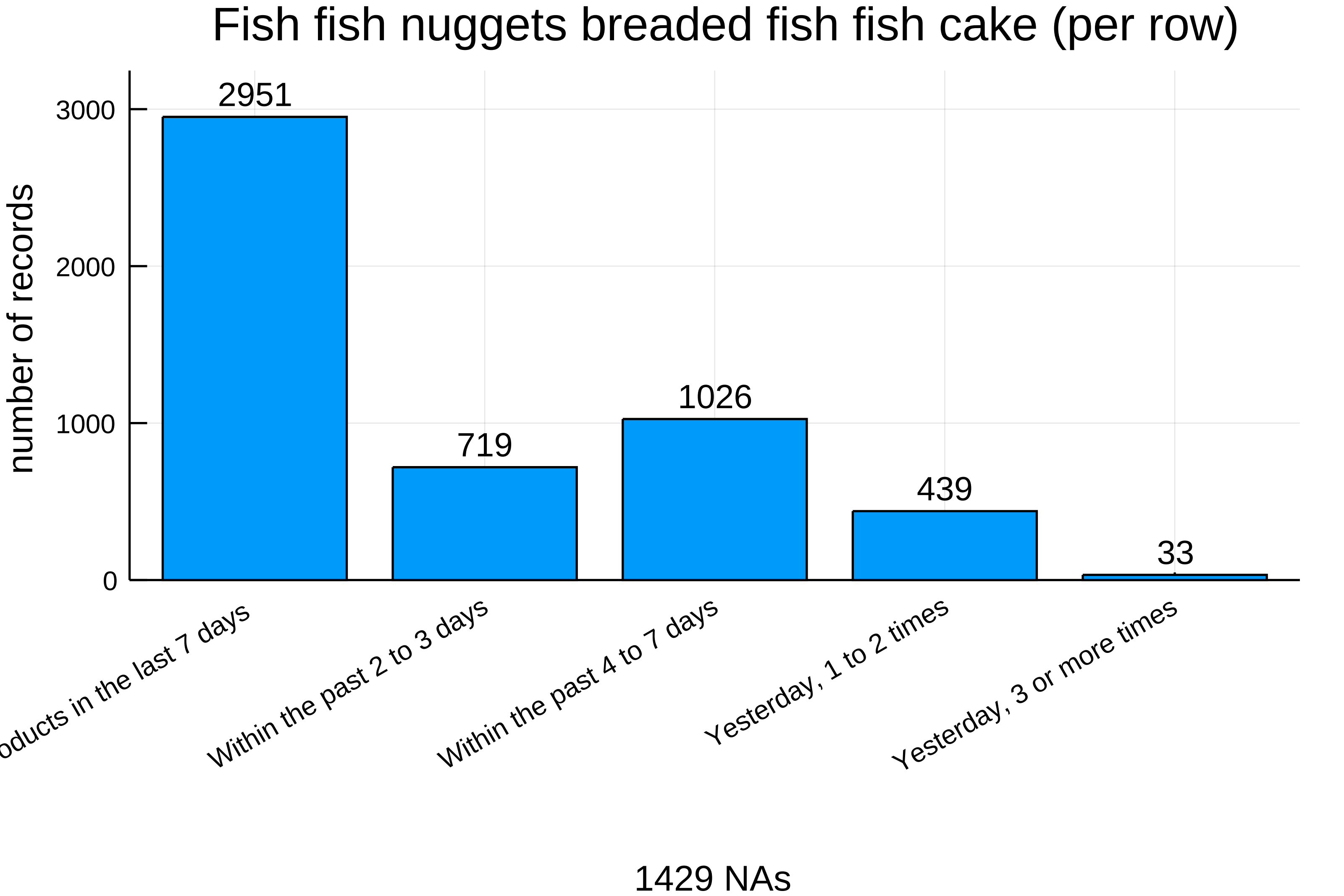

Fish fish nuggets breaded fish fish cake (per row)
number of records
3000
2000
1000
0
2951
719
1026
439
33
oducts in the last 7 days
Within the past 2 to 3 days
Within the past 4 to 7 days
Yesterday, 1 to 2 times
Yesterday, 3 or more times
1429 NAs

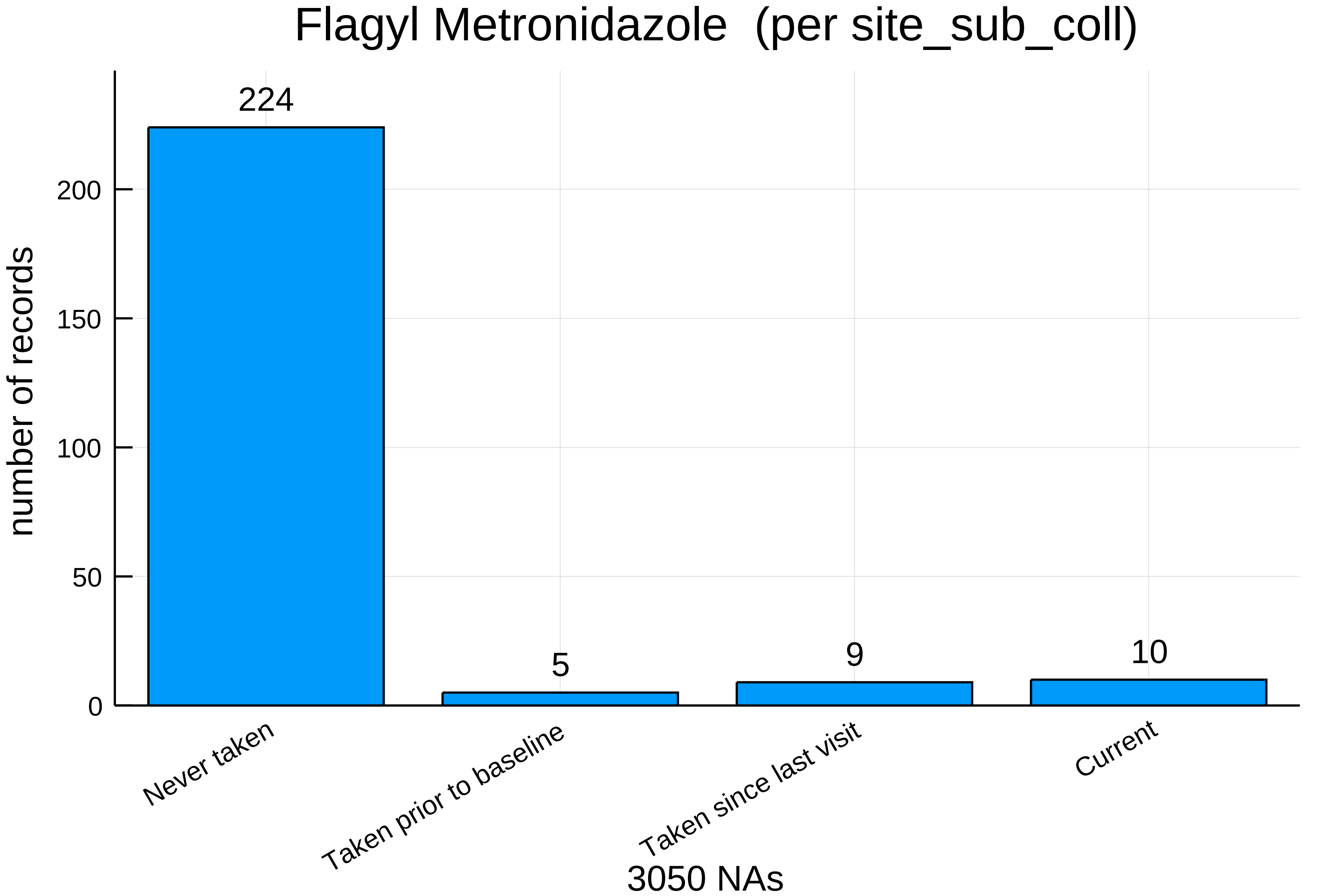
Flagyl Metronidazole (per site_sub_coll)
number of records
224
5
9
10
0
50
100
150
200
Never taken
Taken prior to baseline
Taken since last visit
Current
3050 NAs

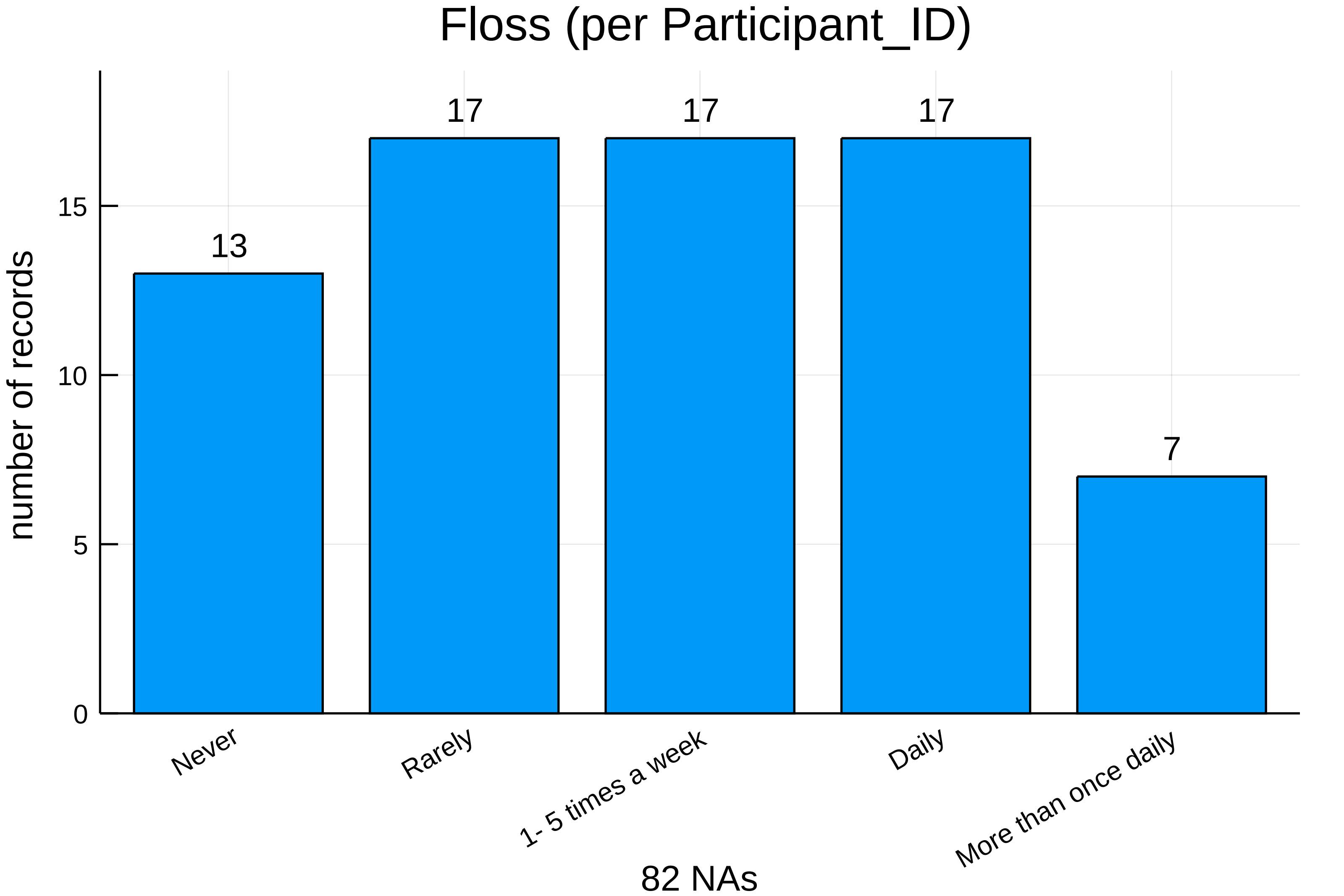
Floss (per Participant_ID)
number of records
0
5
10
15
13
17
17
17
7
Never
Rarely
1- 5 times a week
Daily
More than once daily
82 NAs

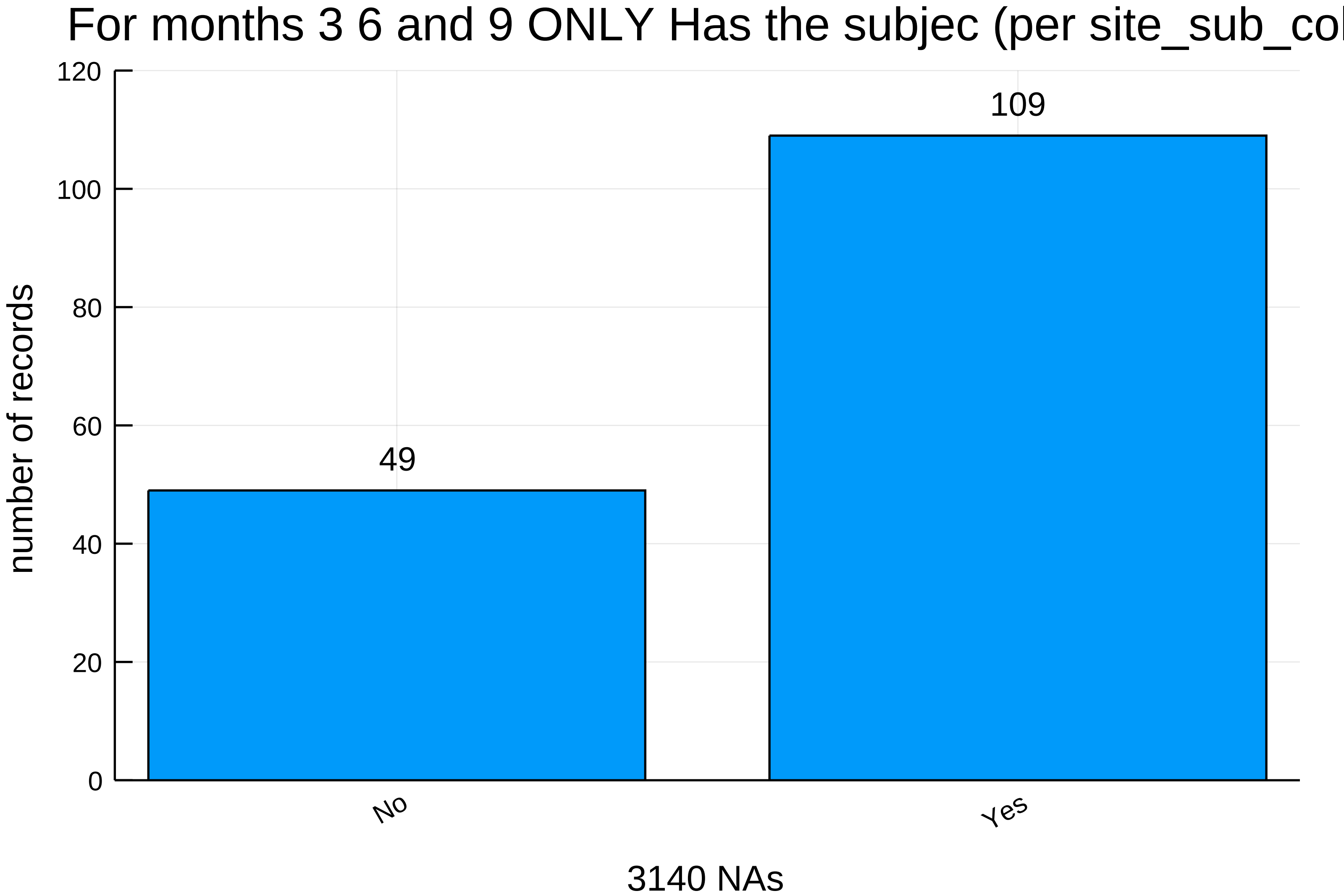
For months 3 6 and 9 ONLY Has the subjec (per site_sub_co
number of records
120
100
80
60
40
20
0
49
109
No
Yes
3140 NAs

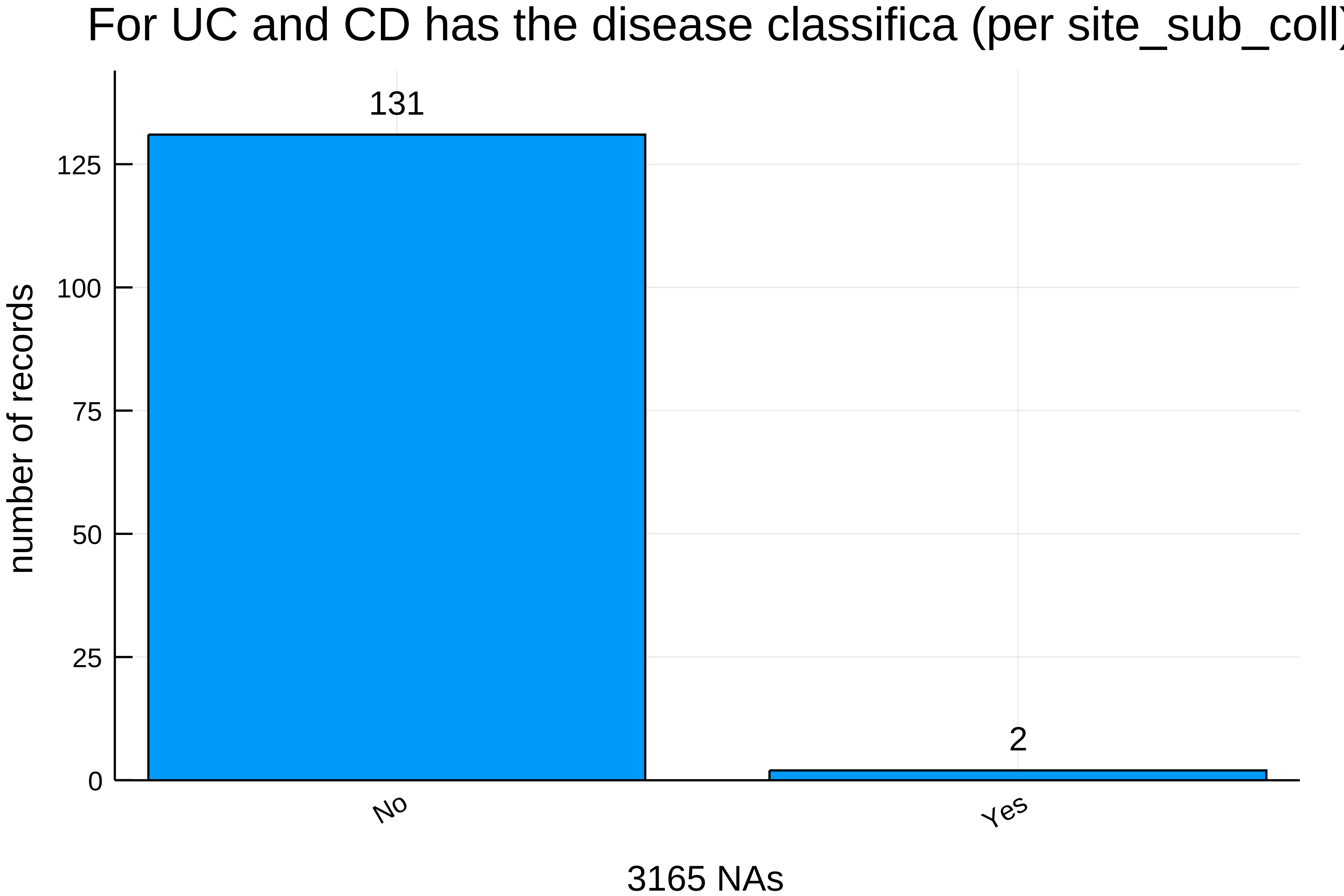
For UC and CD has the disease classifica (per site_sub_coll)
number of records
131
2
125
100
75
50
25
0
No
Yes
3165 NAs

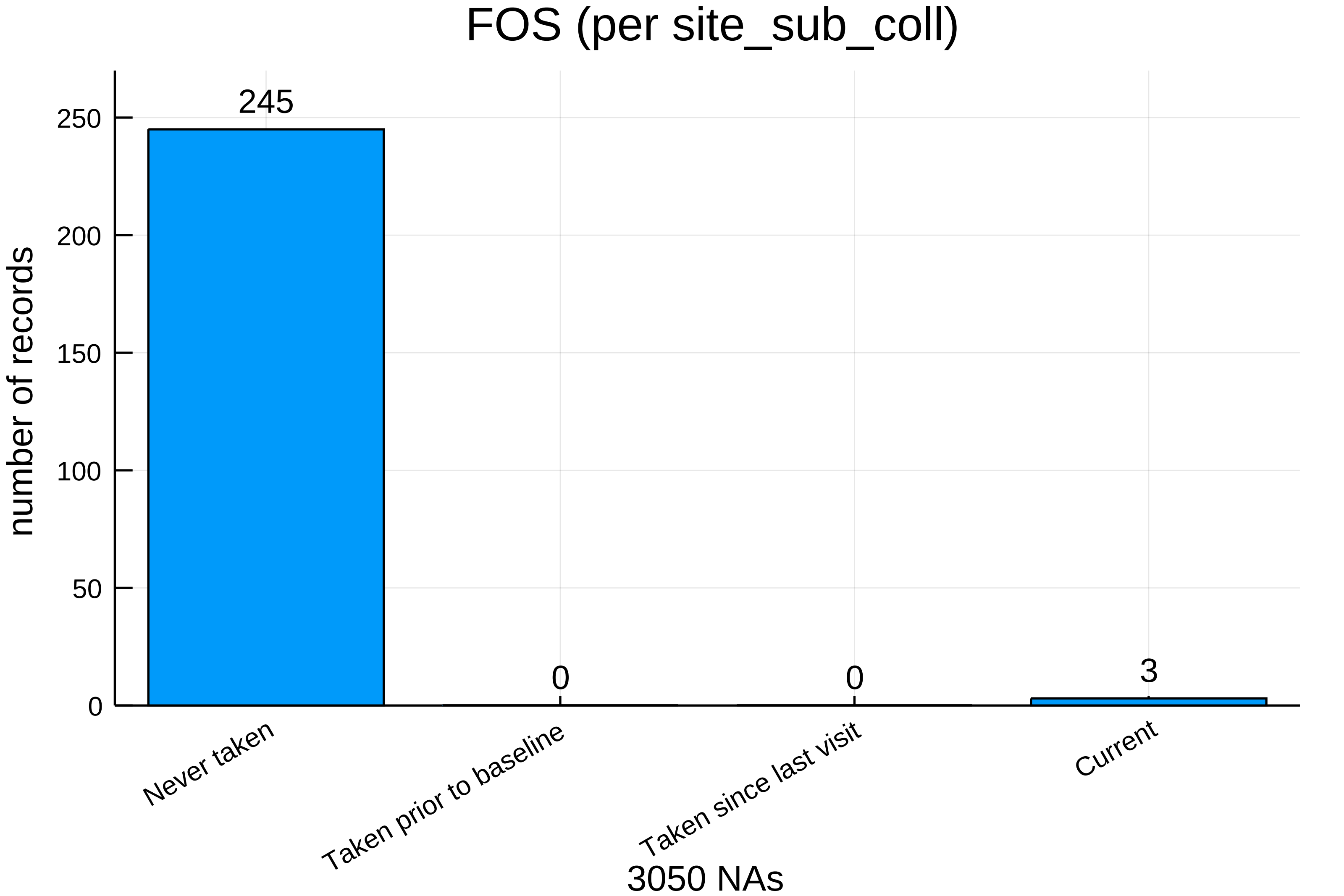
FOS (per site_sub_coll)
number of records
245
0
0
3
0
50
100
150
200
250
Never taken
Taken prior to baseline
Taken since last visit
Current
3050 NAs

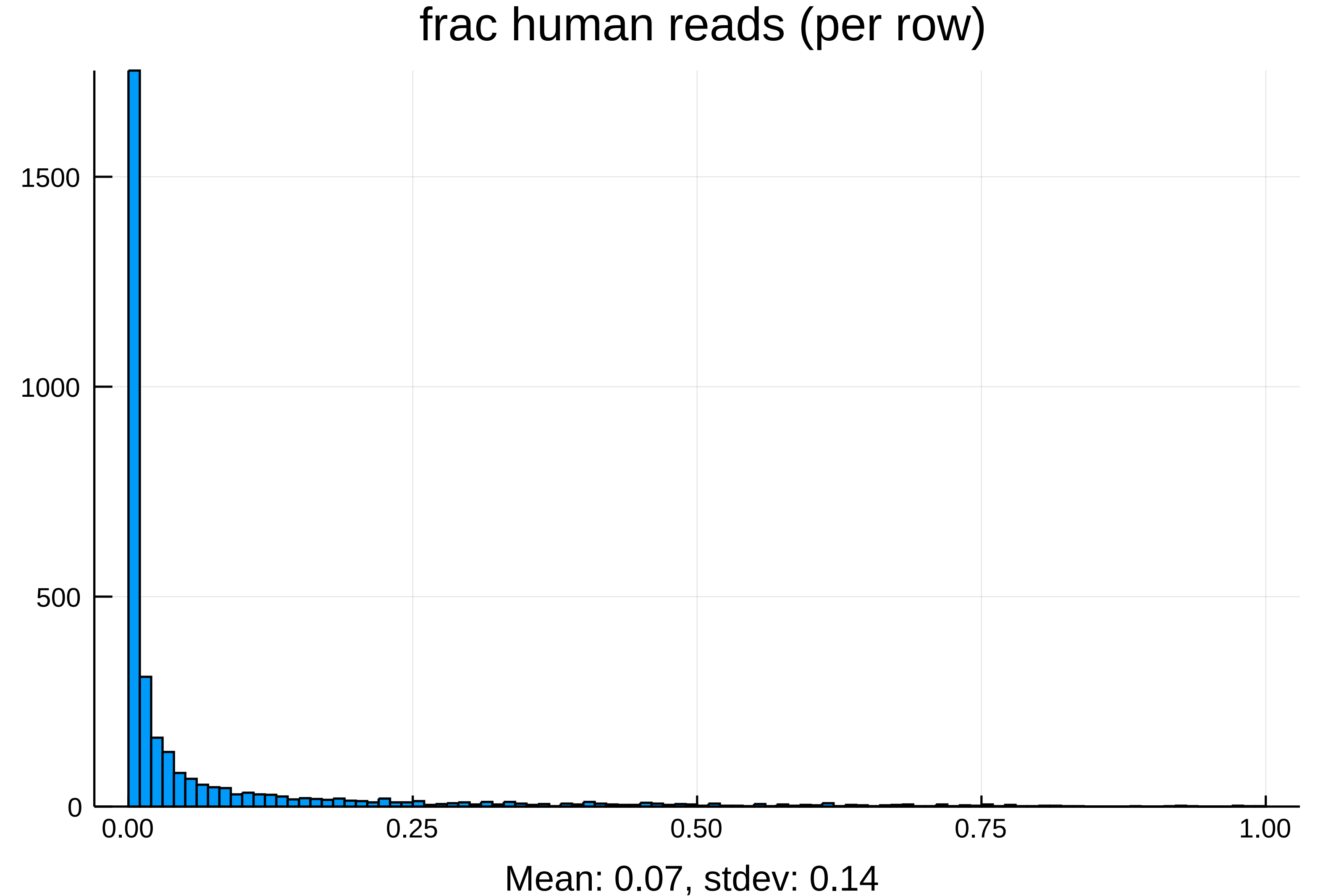

frac human reads (per row)
1500
1000
500
0
0.00
0.25
0.50
0.75
1.00
Mean: 0.07, stdev: 0.14

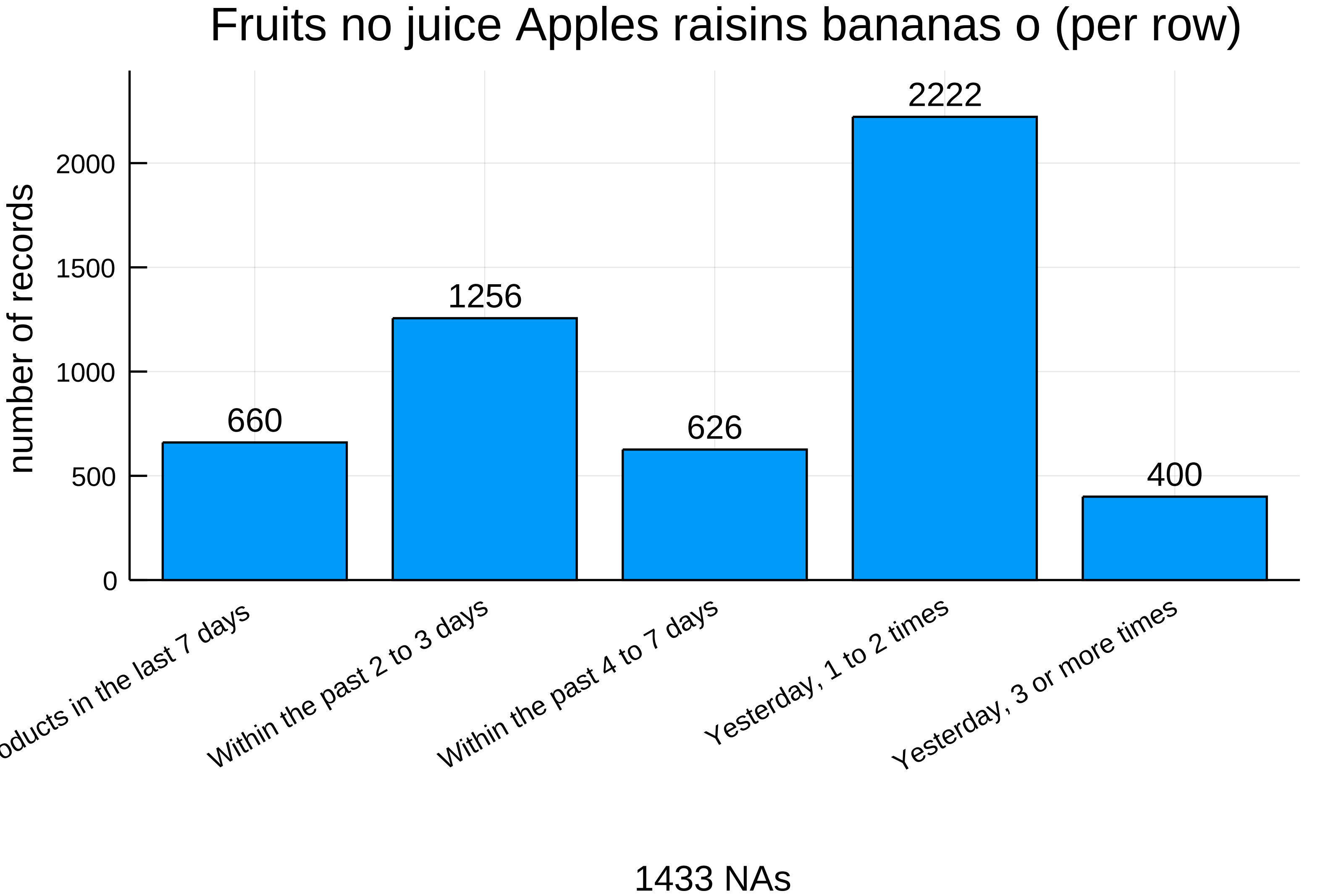
Fruits no juice Apples raisins bananas o (per row)
number of records
0
500
1000
1500
2000
660
1256
626
2222
400
oducts in the last 7 days
Within the past 2 to 3 days
Within the past 4 to 7 days
Yesterday, 1 to 2 times
Yesterday, 3 or more times
1433 NAs

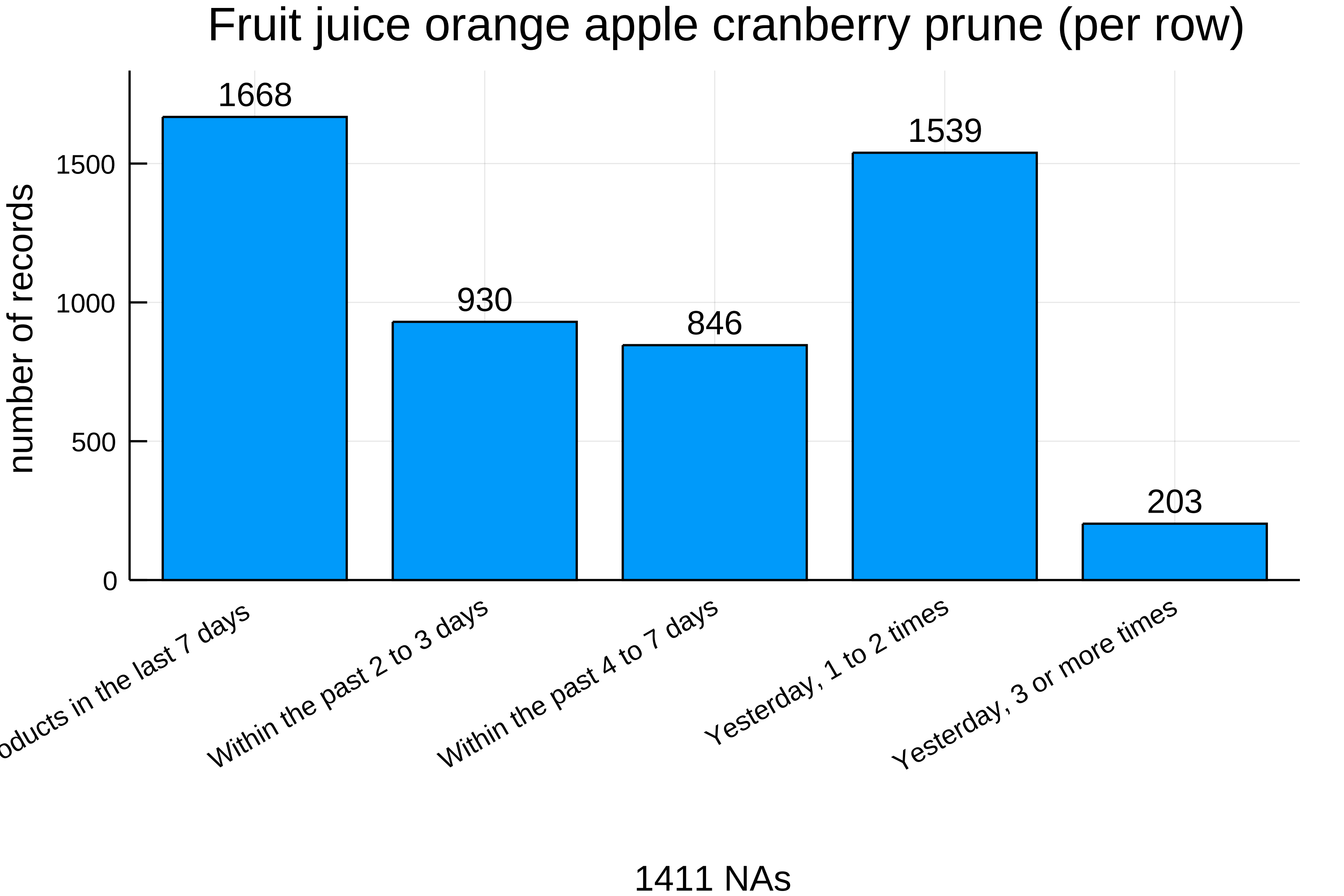
Fruit juice orange apple cranberry prune (per row)
number of records
0
500
1000
1500
1668
930
846
1539
203
oducts in the last 7 days
Within the past 2 to 3 days
Within the past 4 to 7 days
Yesterday, 1 to 2 times
Yesterday, 3 or more times
1411 NAs

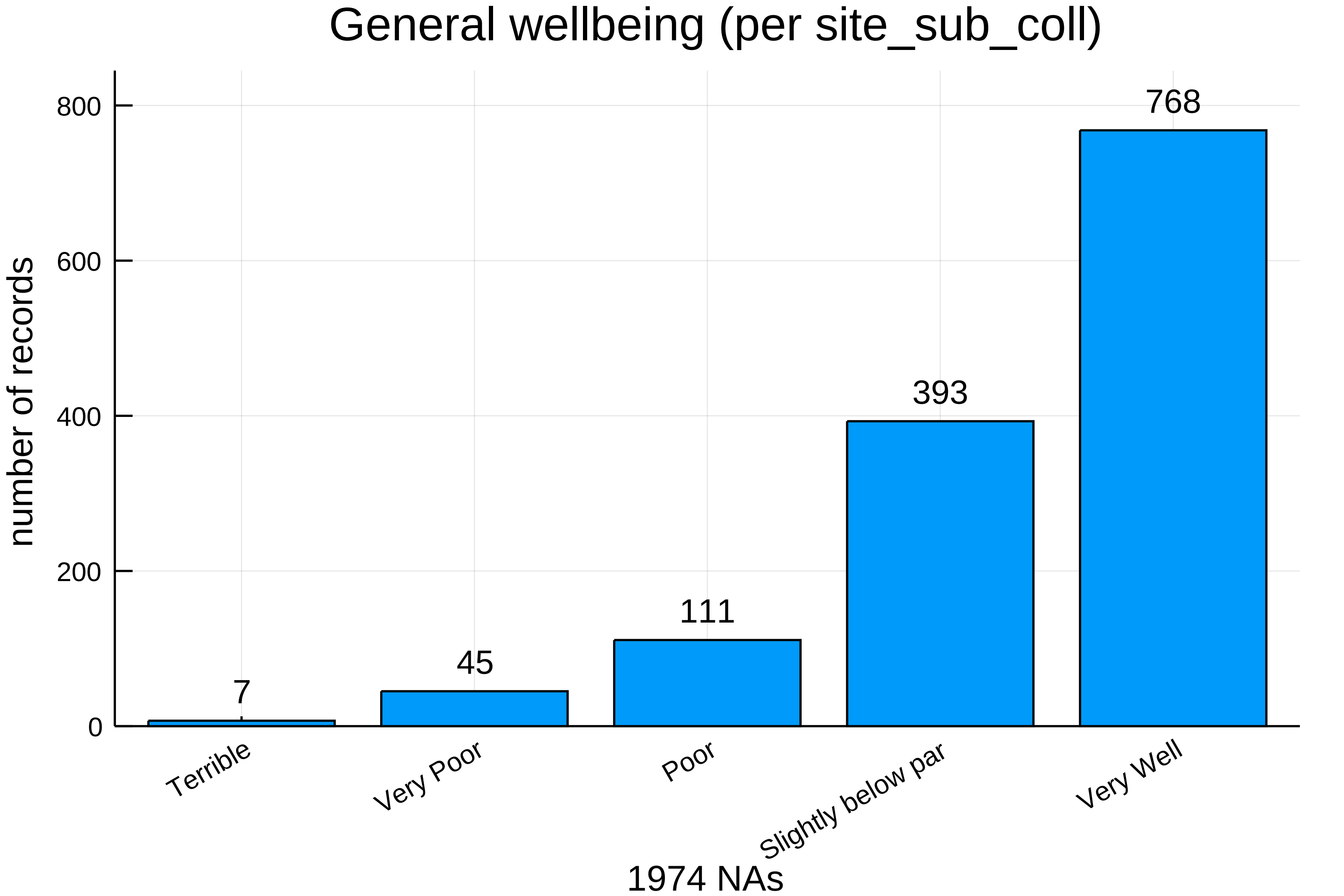
General wellbeing (per site_sub_coll)
number of records
800
600
400
200
0
7
45
111
393
768
Terrible
Very Poor
Poor
Slightly below par
Very Well
1974 NAs

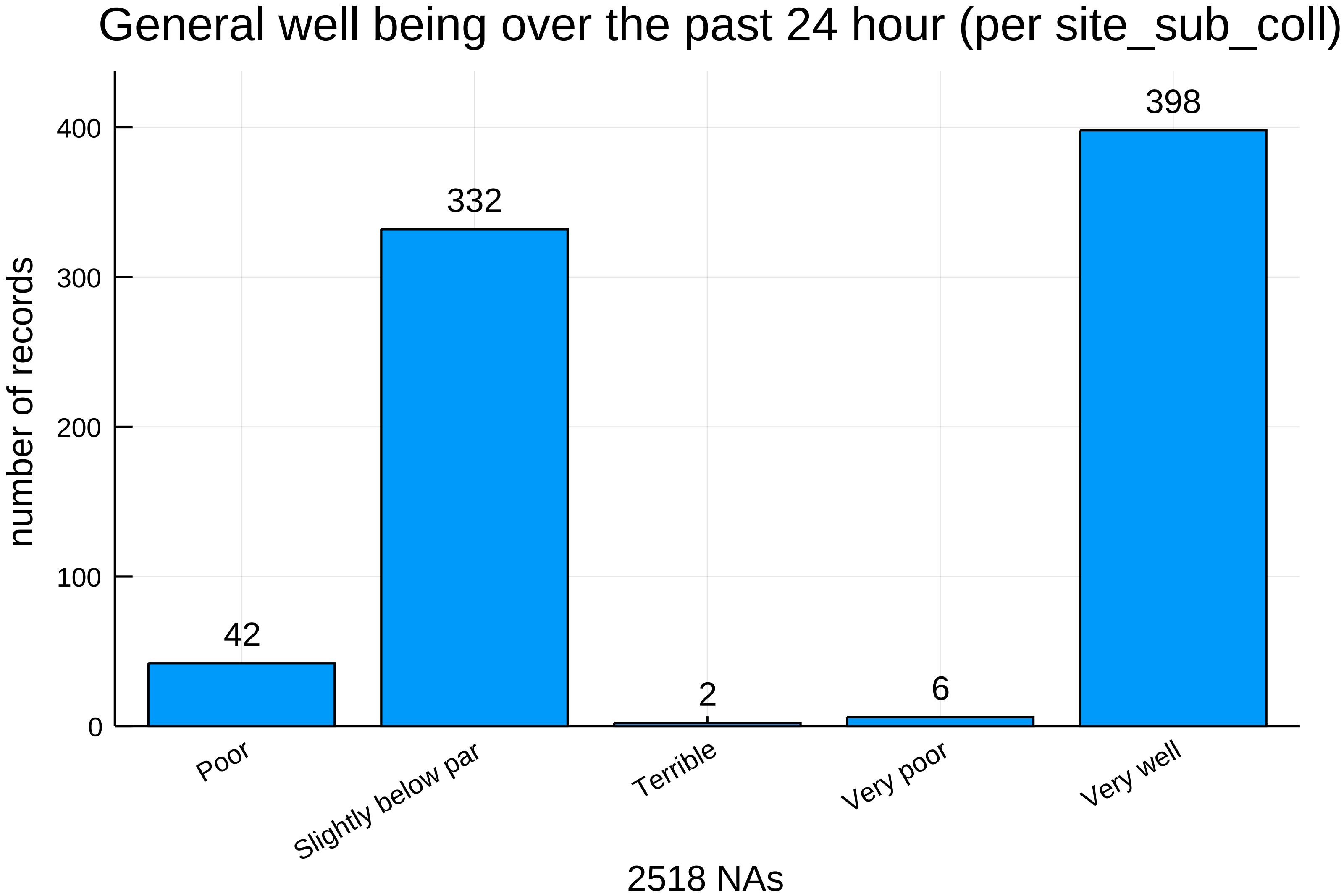
General well being over the past 24 hour (per site_sub_coll)
number of records
400
300
200
100
0
42
332
2
6
398
Poor
Slightly below par
Terrible
Very poor
Very well
2518 NAs

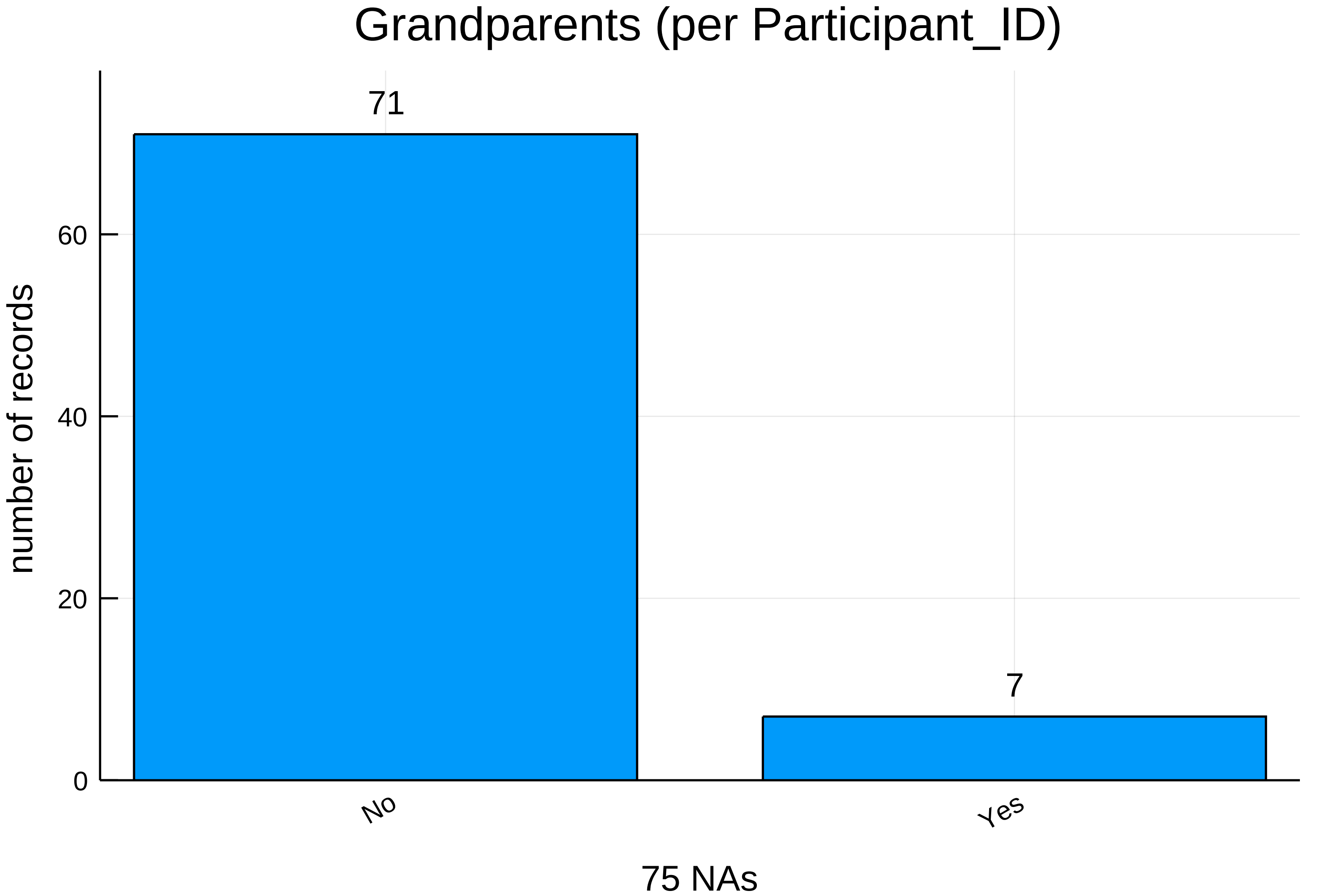
Grandparents (per Participant_ID)
71
7
60
40
20
0
number of records
No
Yes
75 NAs

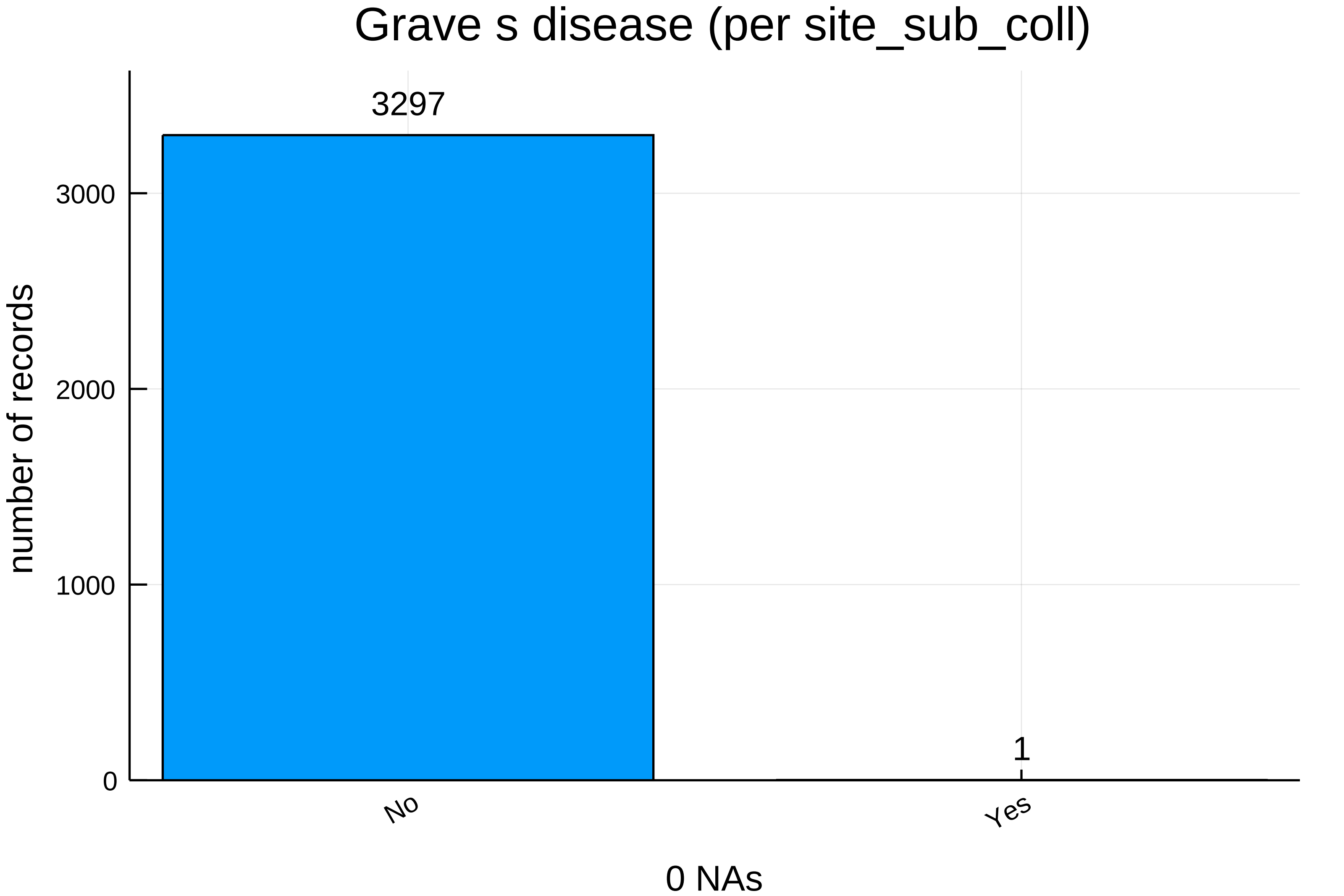
Grave s disease (per site_sub_coll)
number of records
3297
1
3000
2000
1000
0
No
Yes
0 NAs

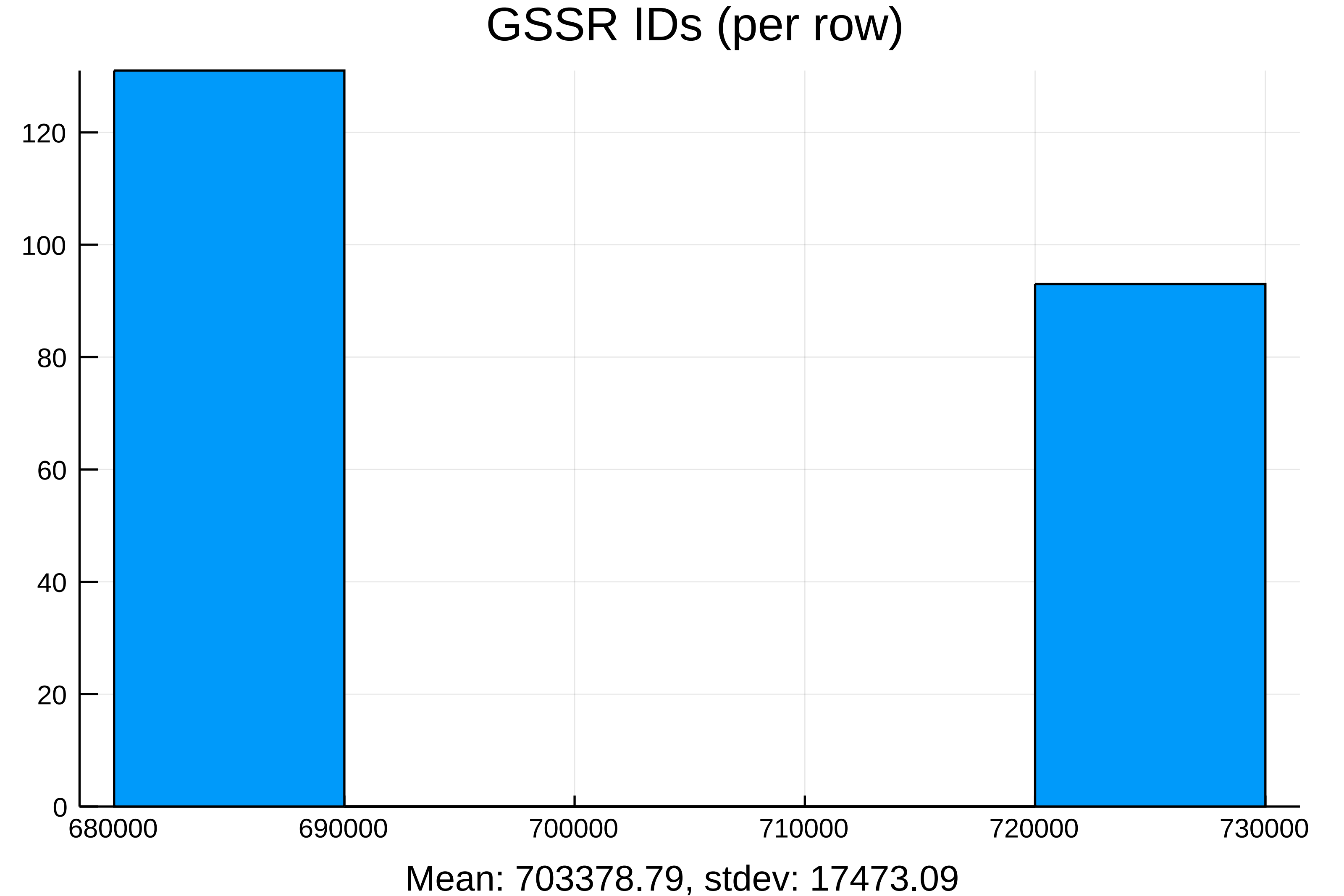
GSSR IDs (per row)
0
20
40
60
80
100
120
680000
690000
700000
710000
720000
730000
Mean: 703378.79, stdev: 17473.09

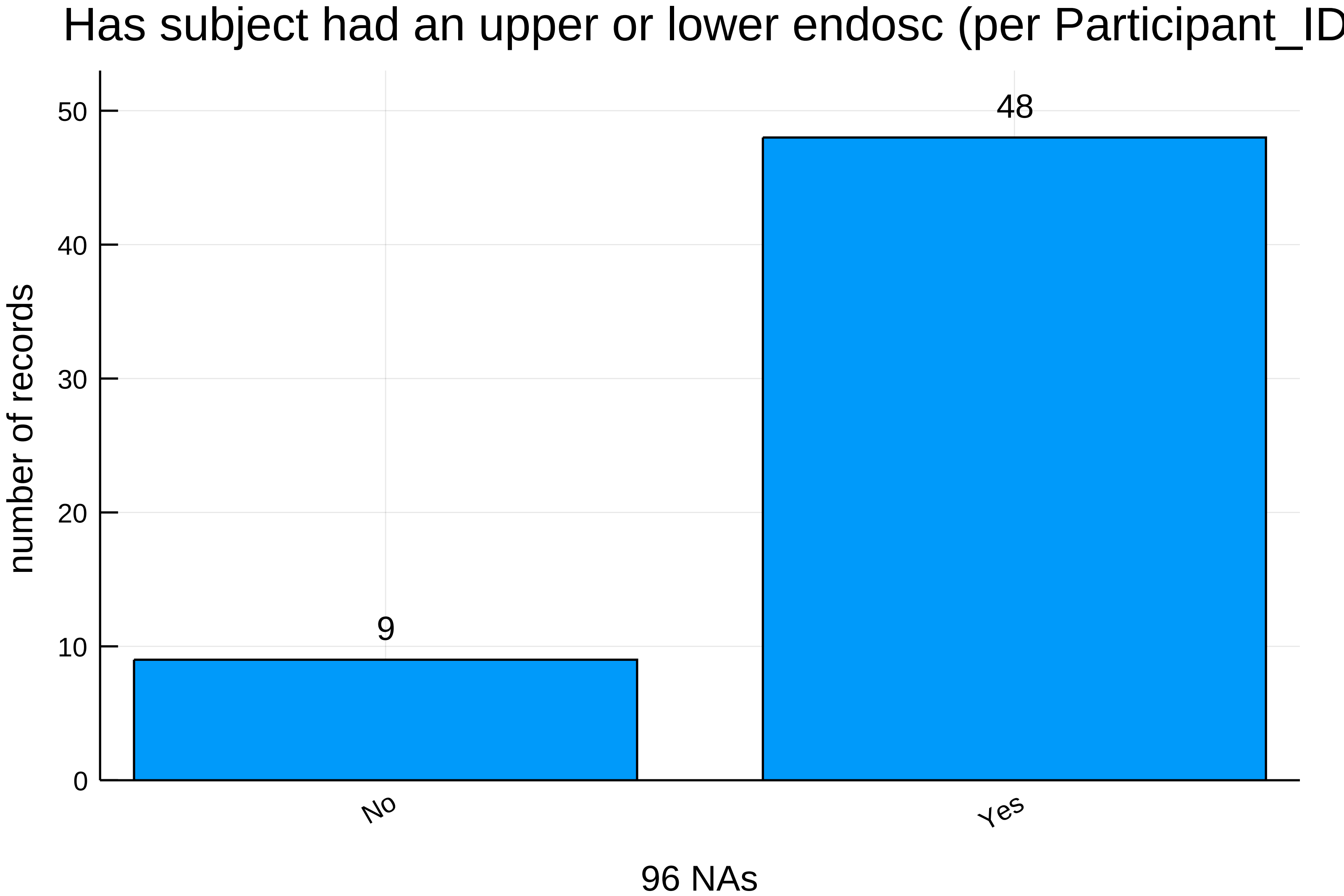

Has subject had an upper or lower endosc (per Participant_ID
number of records
0
10
20
30
40
50
9
48
No
Yes
96 NAs

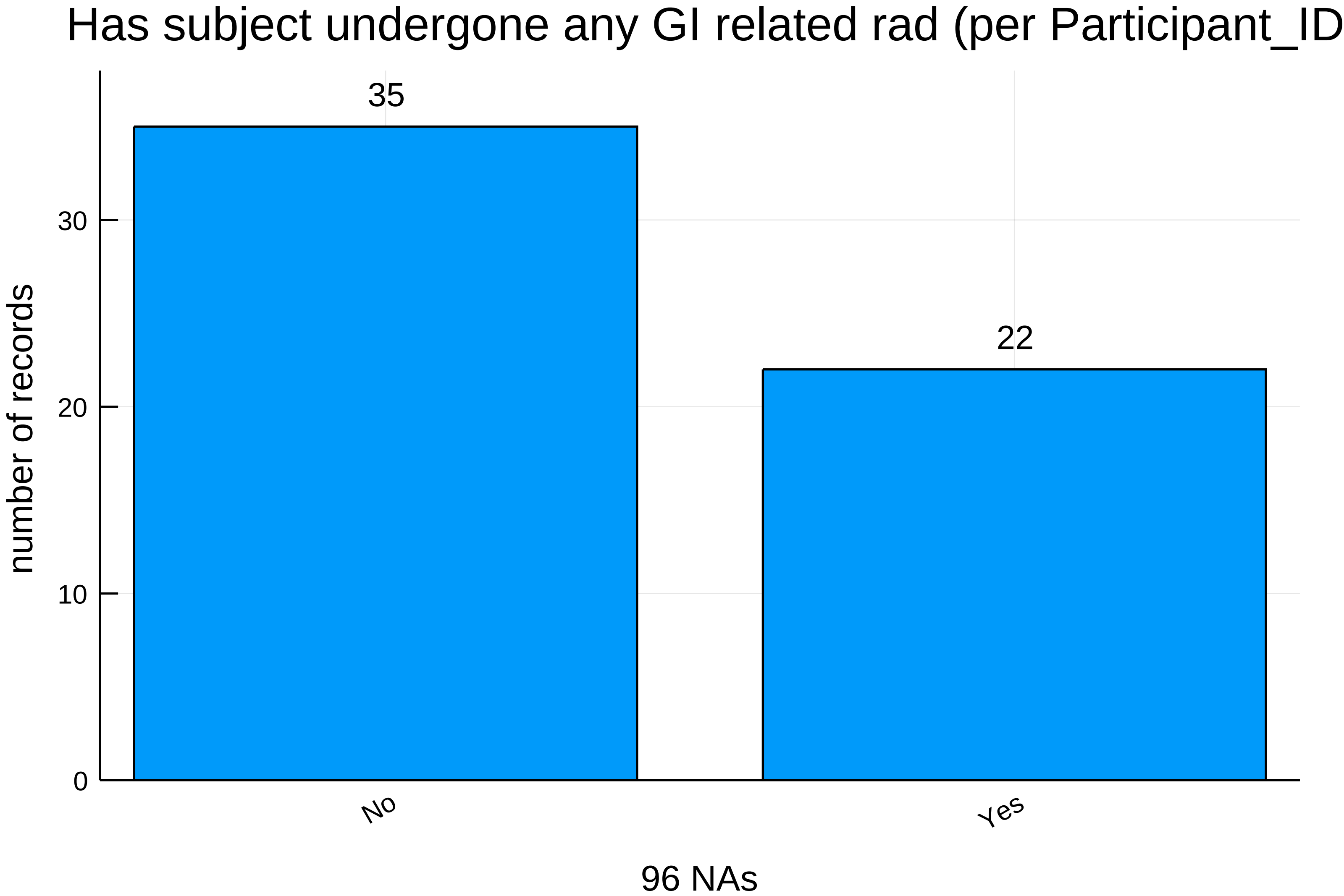
Has subject undergone any GI related rad (per Participant_ID
number of records
35
22
0
10
20
30
No
Yes
96 NAs

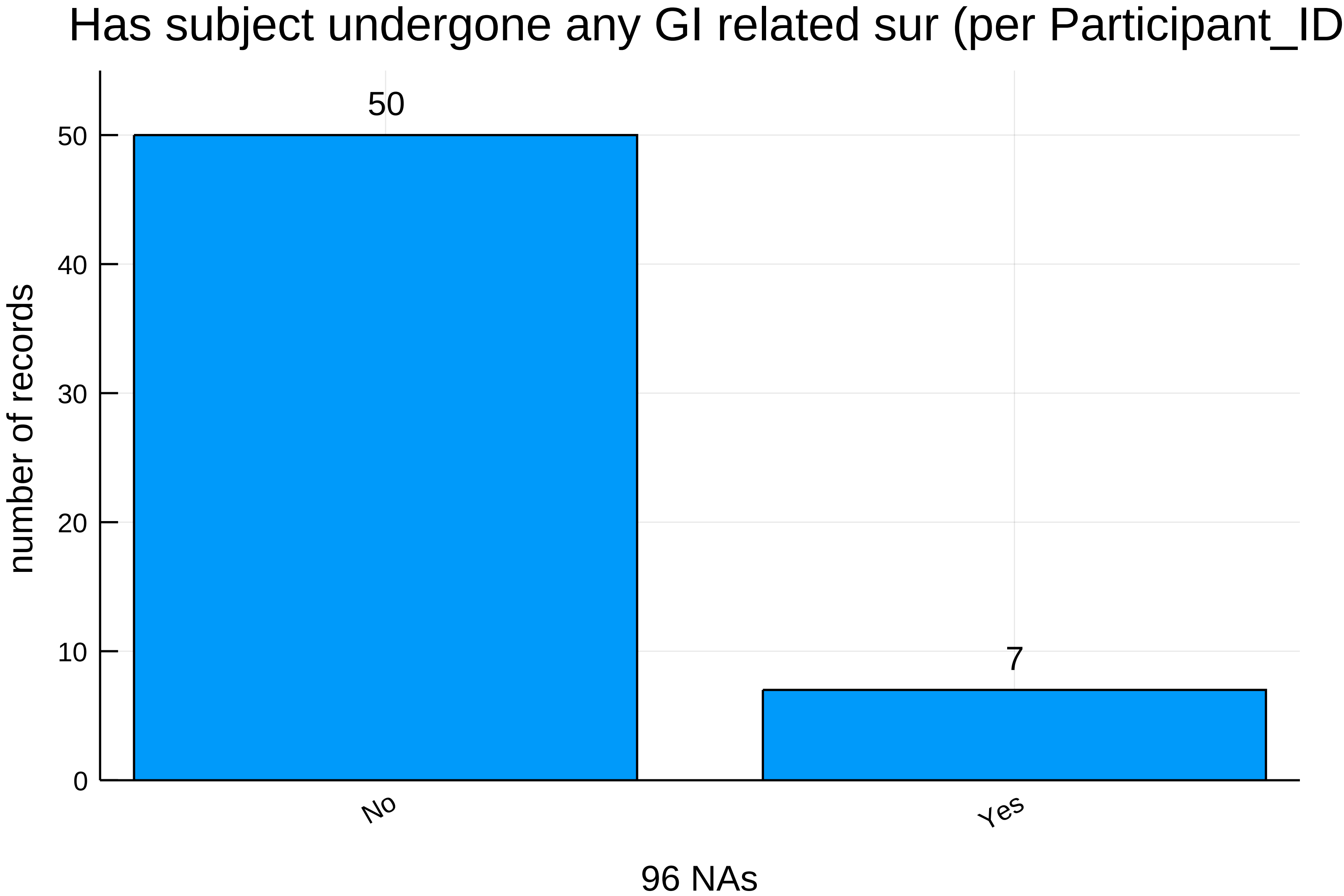
Has subject undergone any GI related sur (per Participant_ID
number of records
0
10
20
30
40
50
50
7
No
Yes
96 NAs

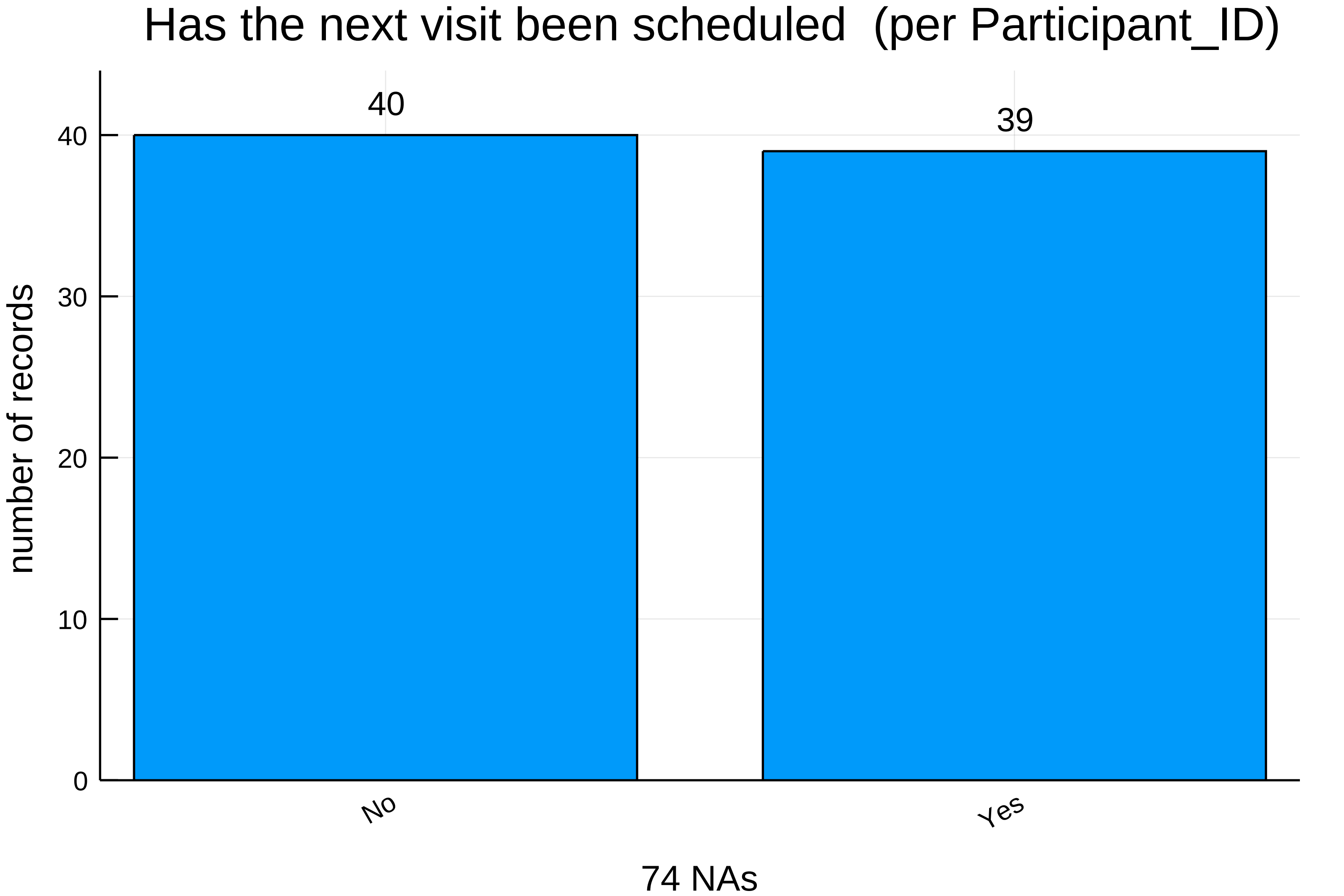
Has the next visit been scheduled (per Participant_ID)
number of records
40
39
0
10
20
30
40
No
Yes
74 NAs

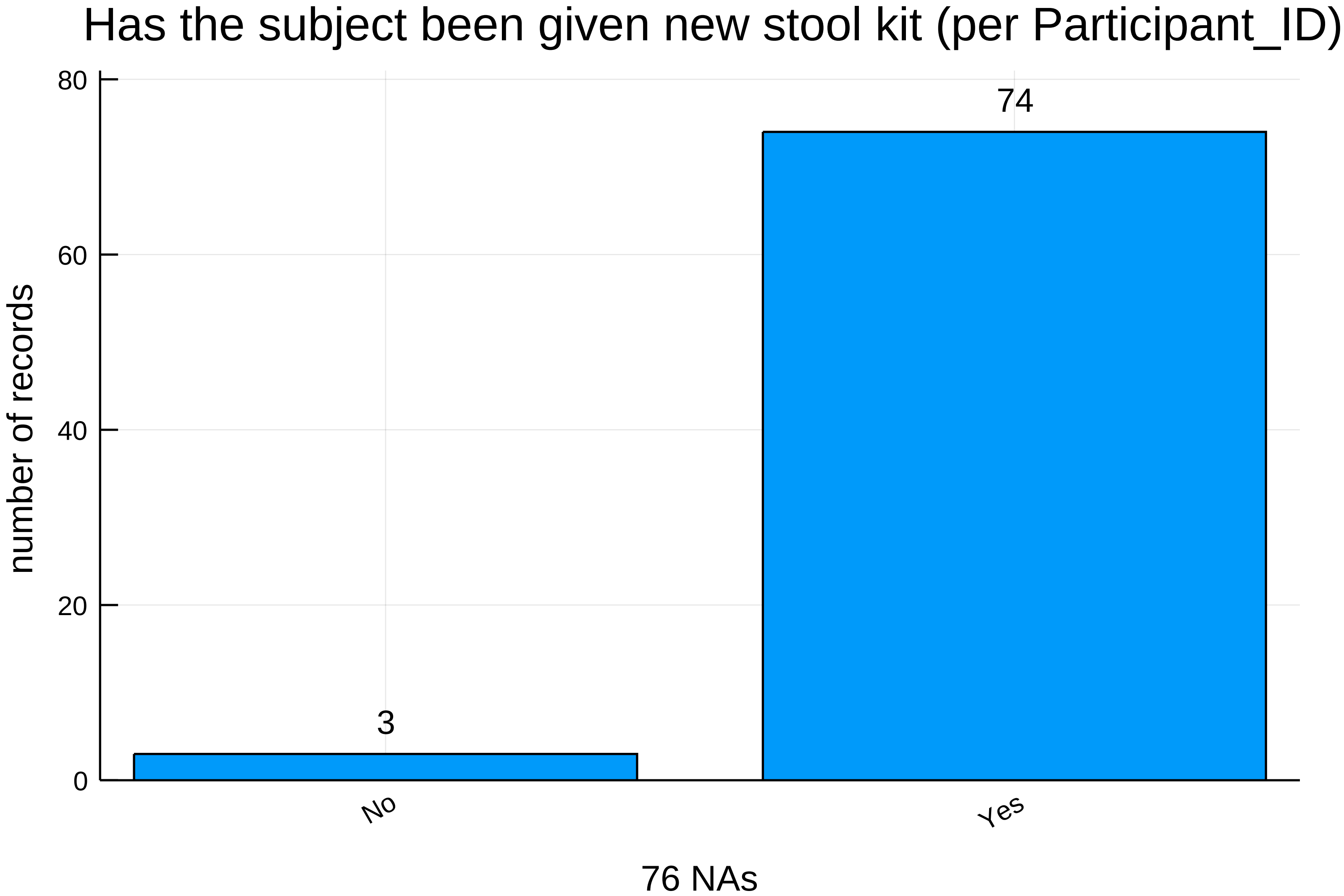
Has the subject been given new stool kit (per Participant_ID)
number of records
0
20
40
60
80
3
74
No
Yes
76 NAs

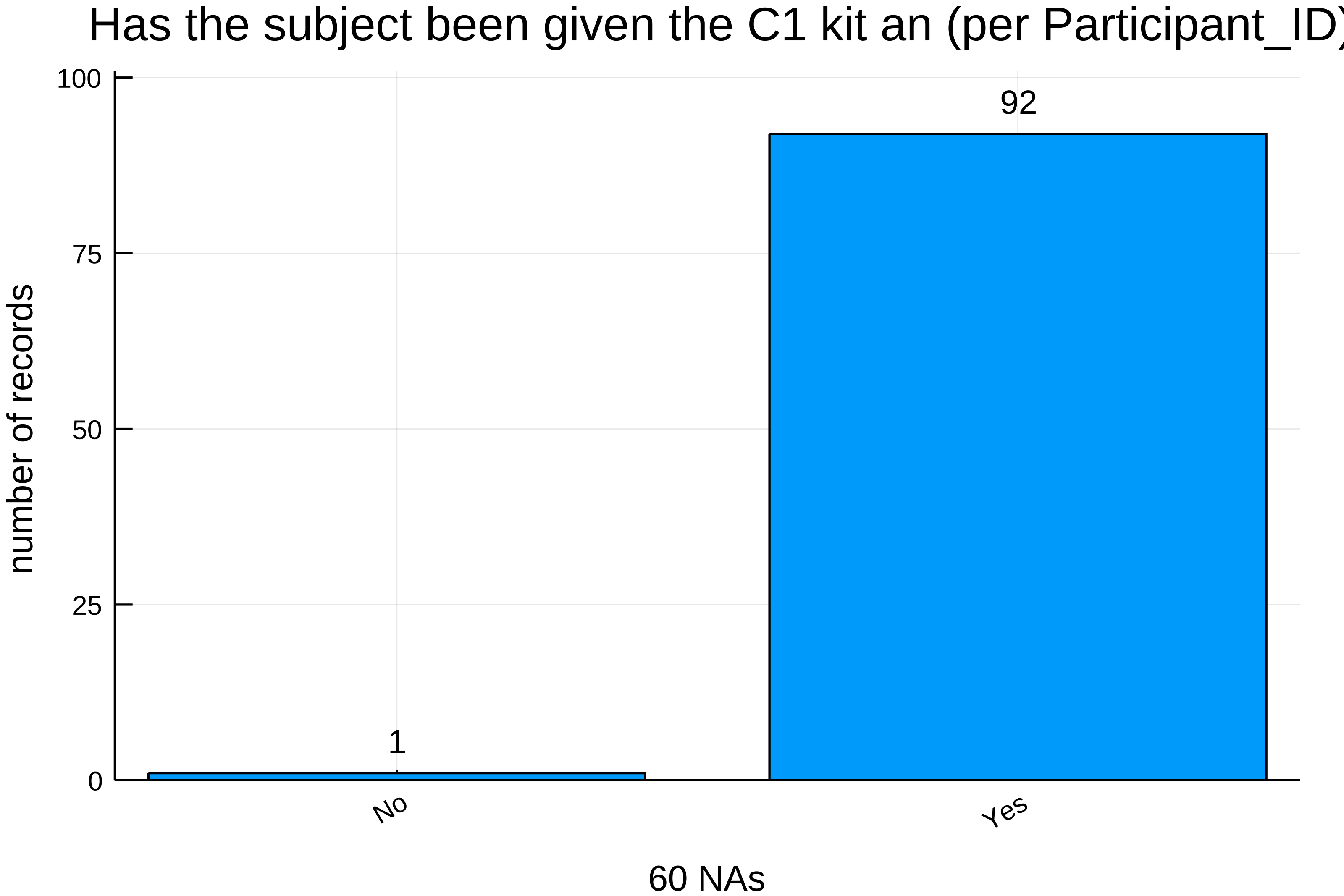

Has the subject been given the C1 kit an (per Participant_ID)
number of records
100
75
50
25
0
92
1
No
Yes
60 NAs

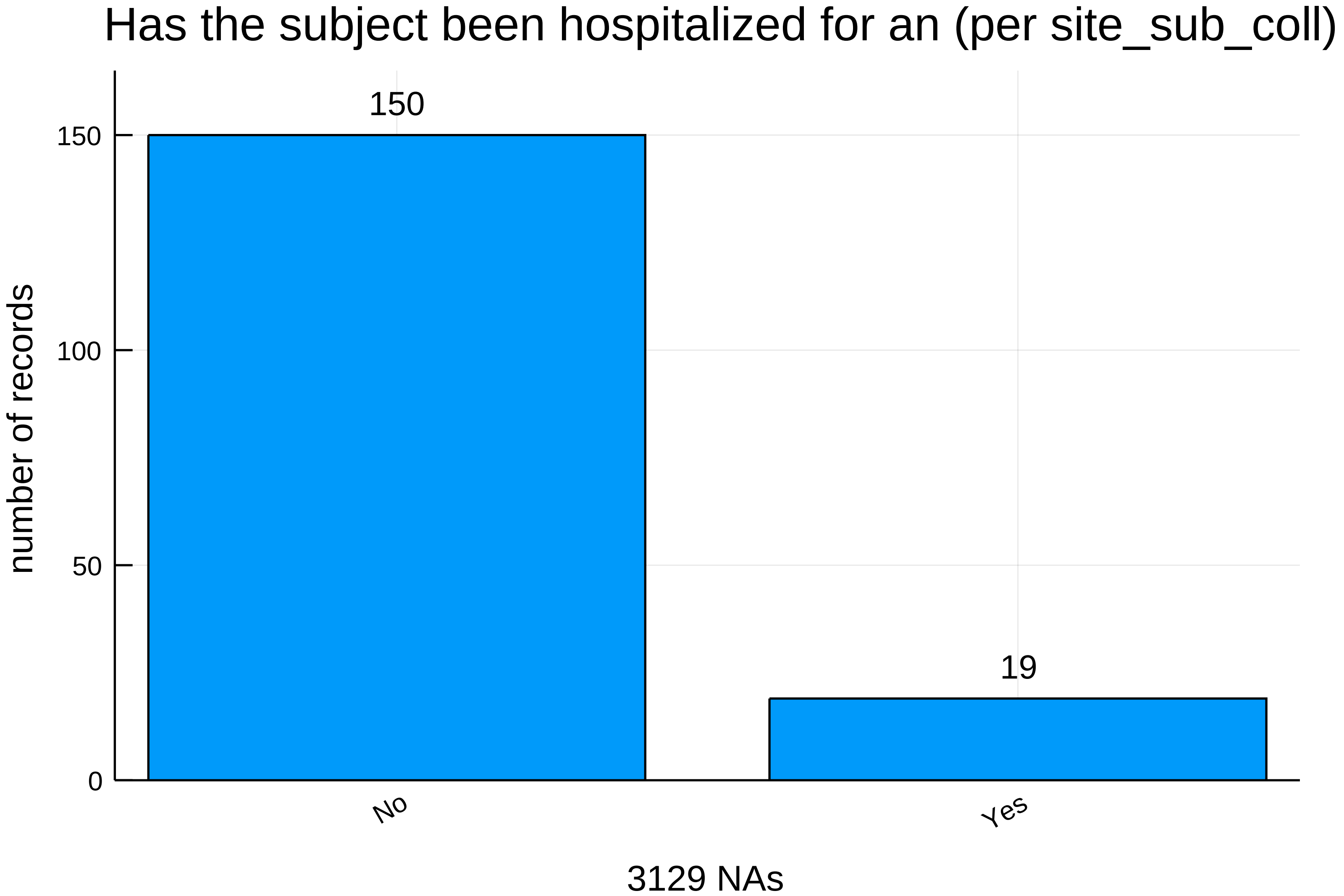
Has the subject been hospitalized for an (per site_sub_coll)
number of records
150
19
150
100
50
0
No
Yes
3129 NAs

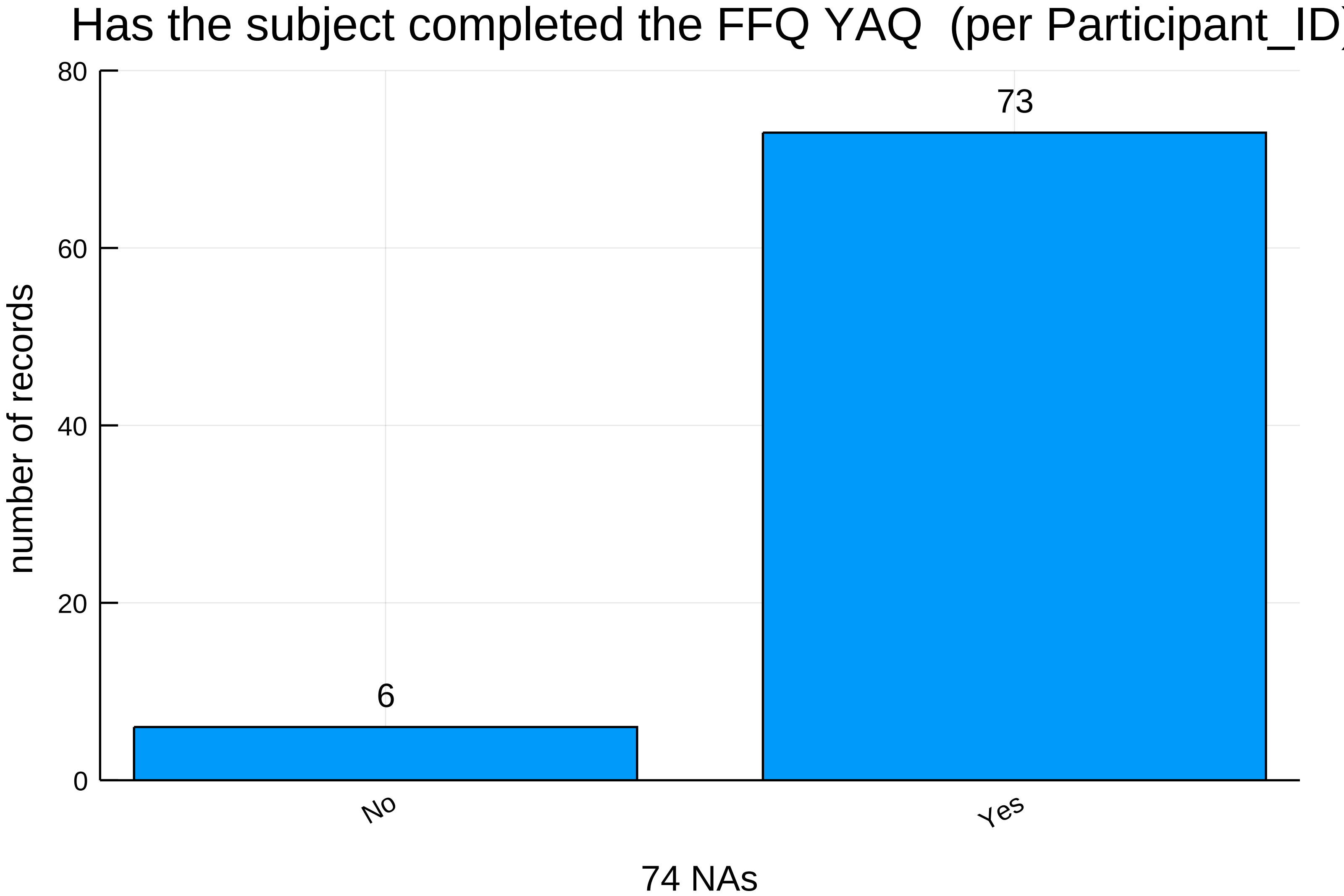
Has the subject completed the FFQ YAQ (per Participant_ID)
number of records
80
60
40
20
0
73
6
No
Yes
74 NAs

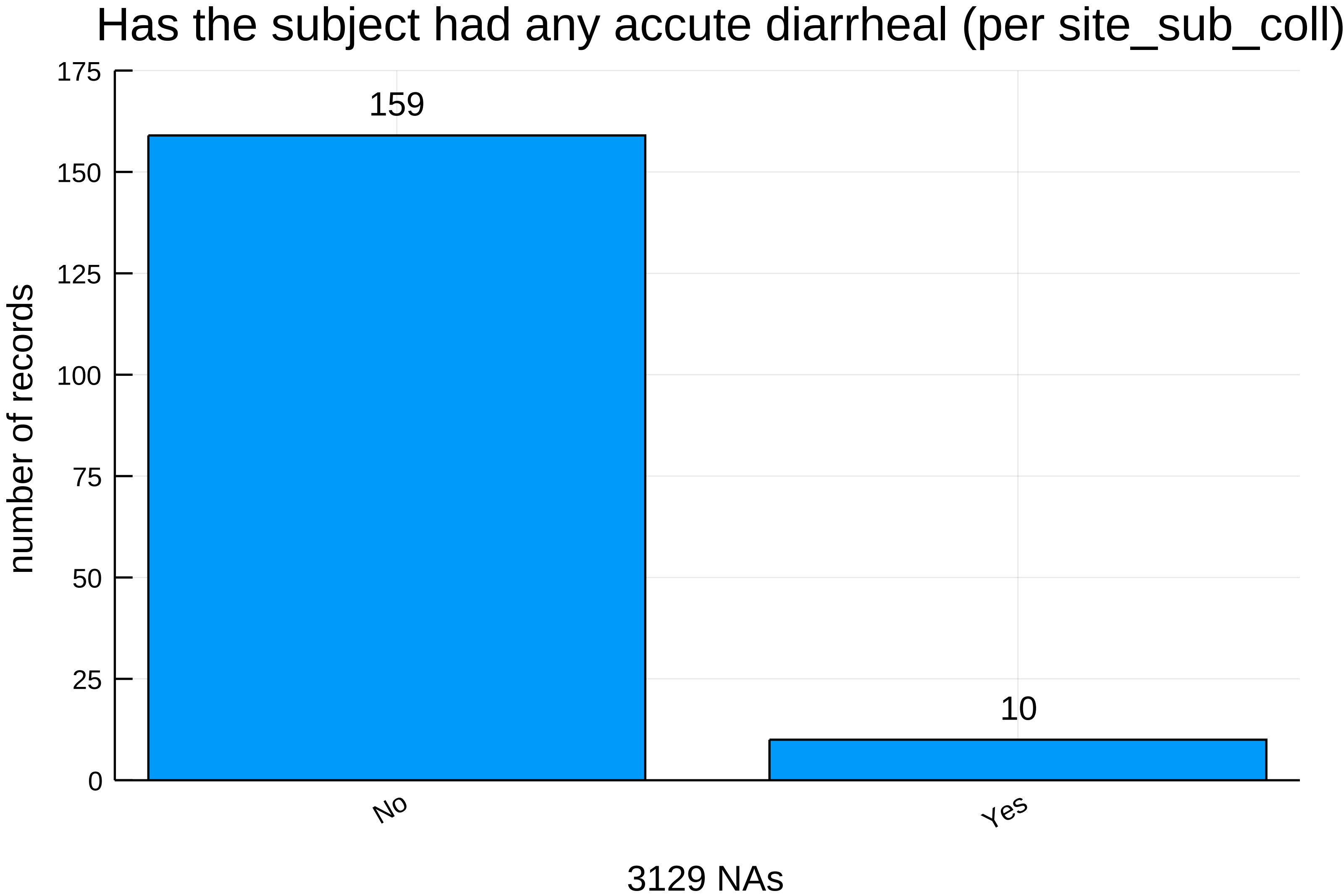
Has the subject had any accute diarrheal (per site_sub_coll)
number of records
175
150
125
100
75
50
25
0
159
10
No
Yes
3129 NAs

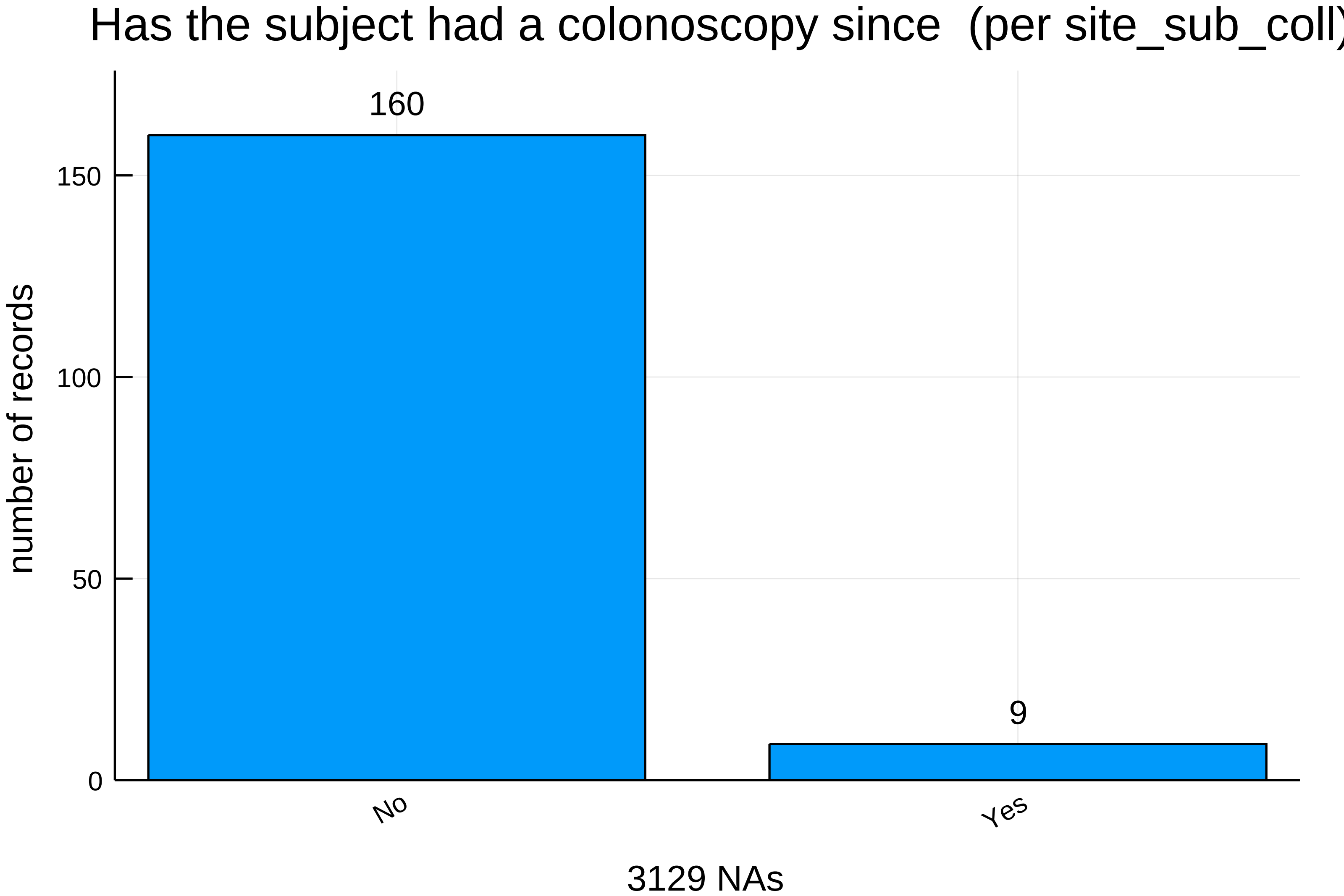
Has the subject had a colonoscopy since (per site_sub_coll)
number of records
160
9
150
100
50
0
No
Yes
3129 NAs

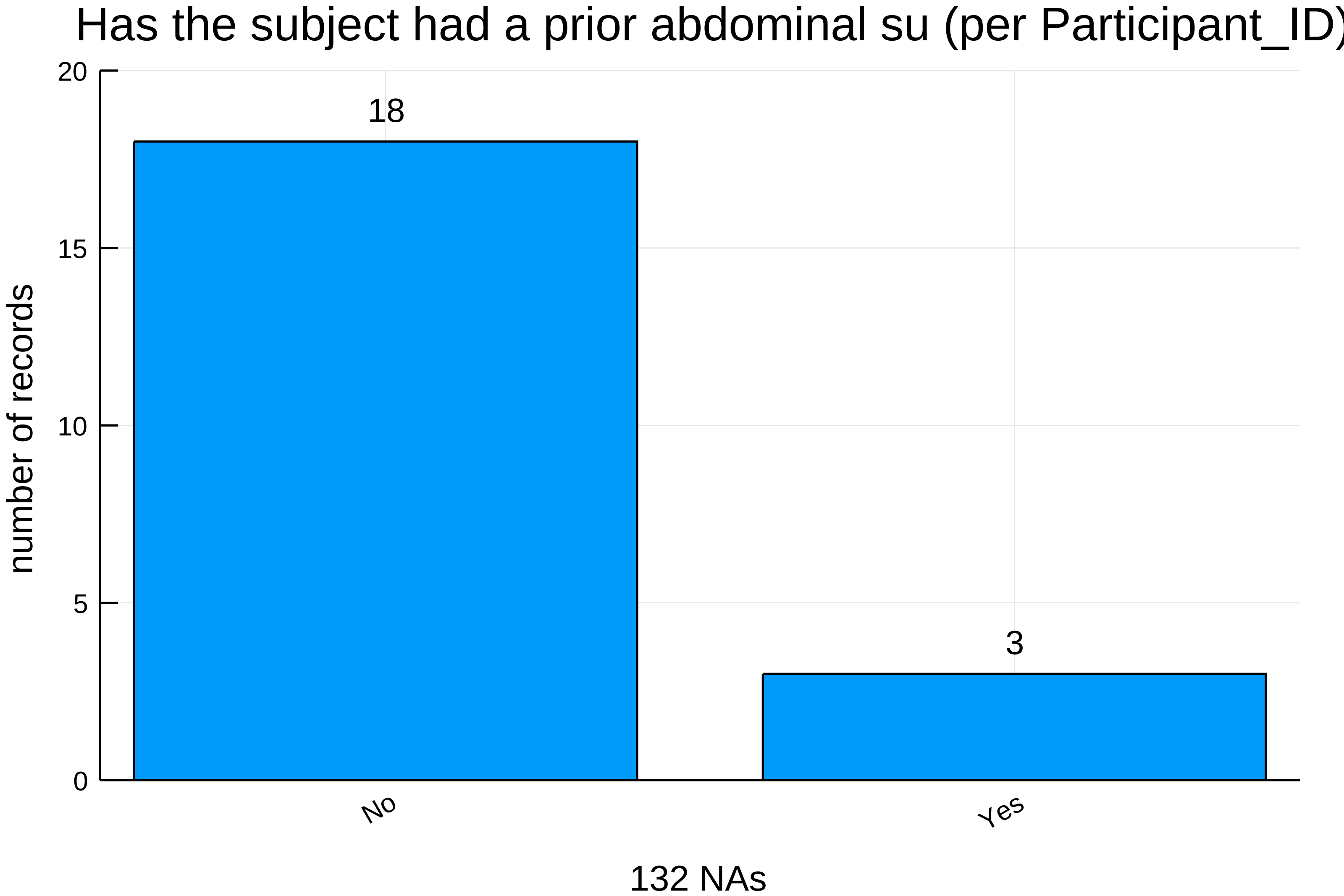
Has the subject had a prior abdominal su (per Participant_ID)
number of records
0
5
10
15
20
18
3
No
Yes
132 NAs

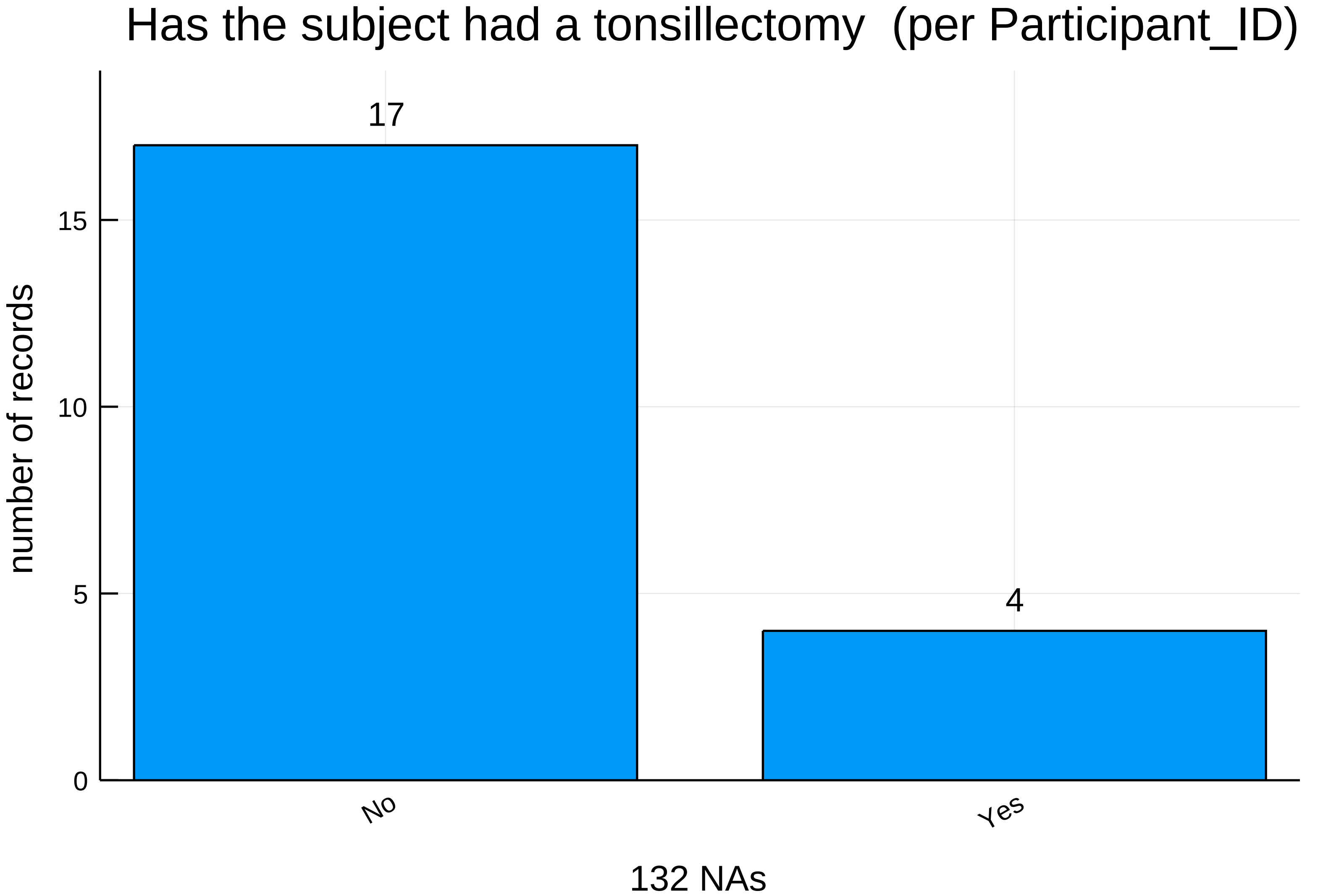
Has the subject had a tonsillectomy (per Participant_ID)
number of records
17
4
0
5
10
15
No
Yes
132 NAs

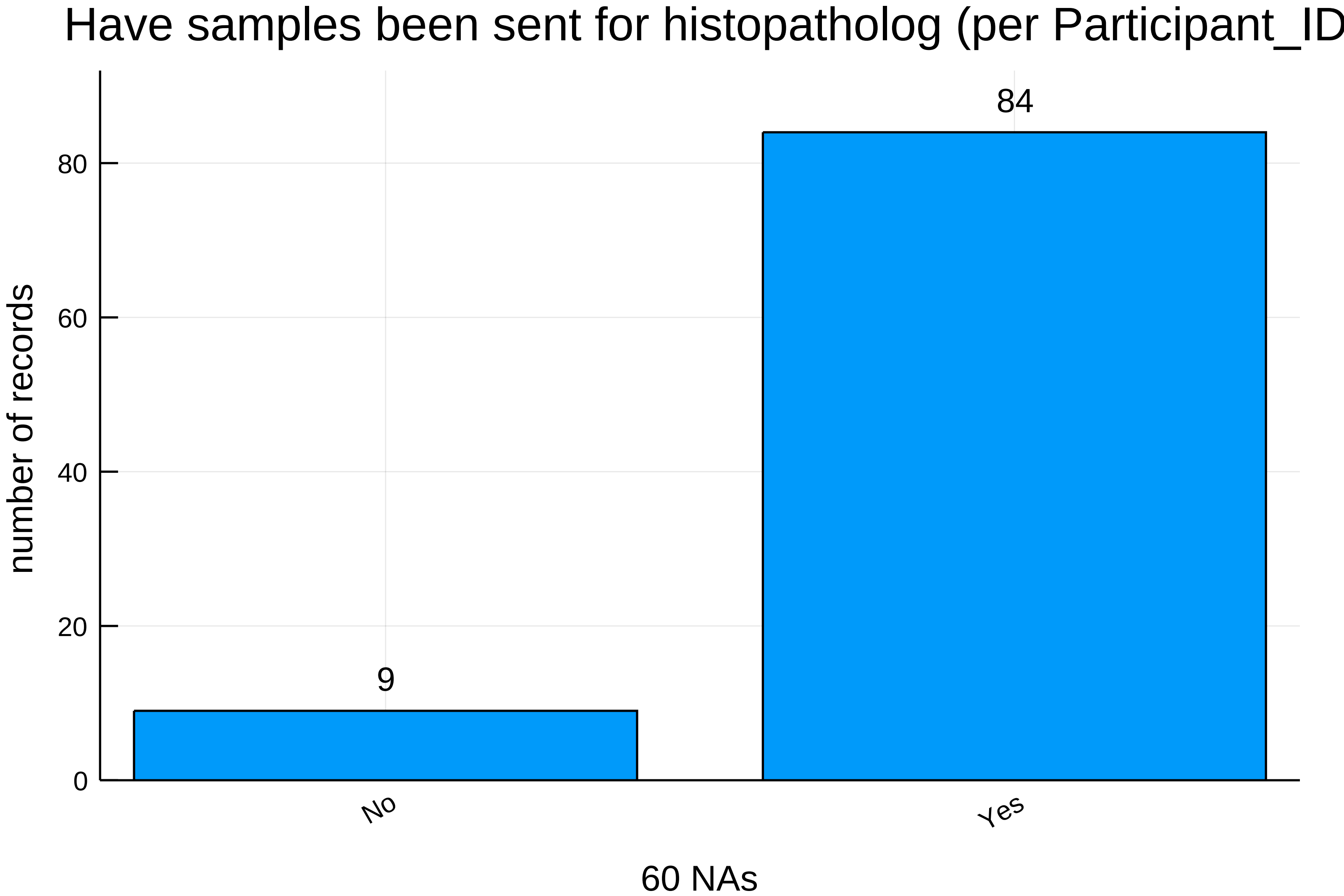

Have samples been sent for histopatholog (per Participant_ID
number of records
0
20
40
60
80
9
84
No
Yes
60 NAs

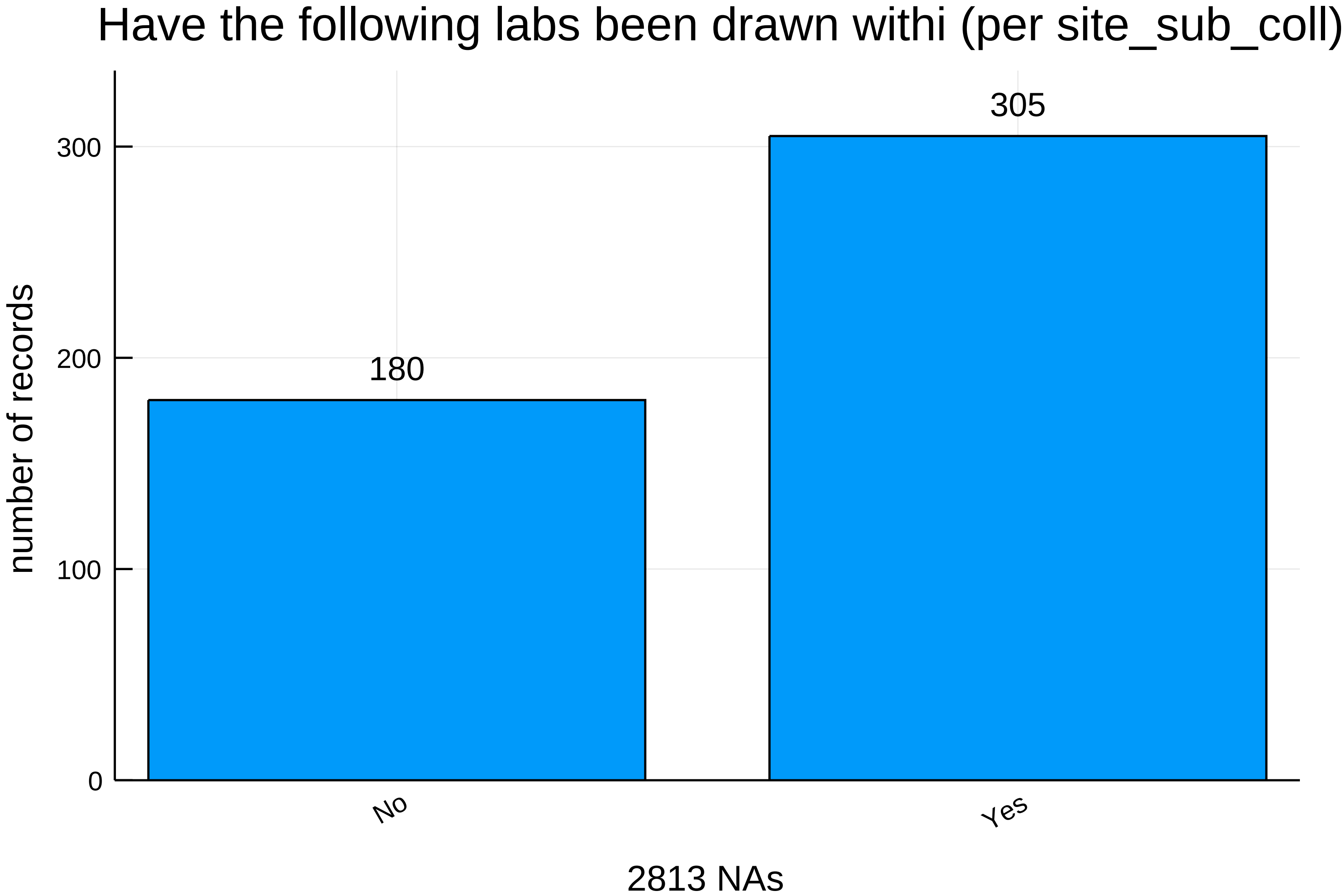

Have the following labs been drawn withi (per site_sub_coll)
number of records
300
200
100
0
180
305
No
Yes
2813 NAs

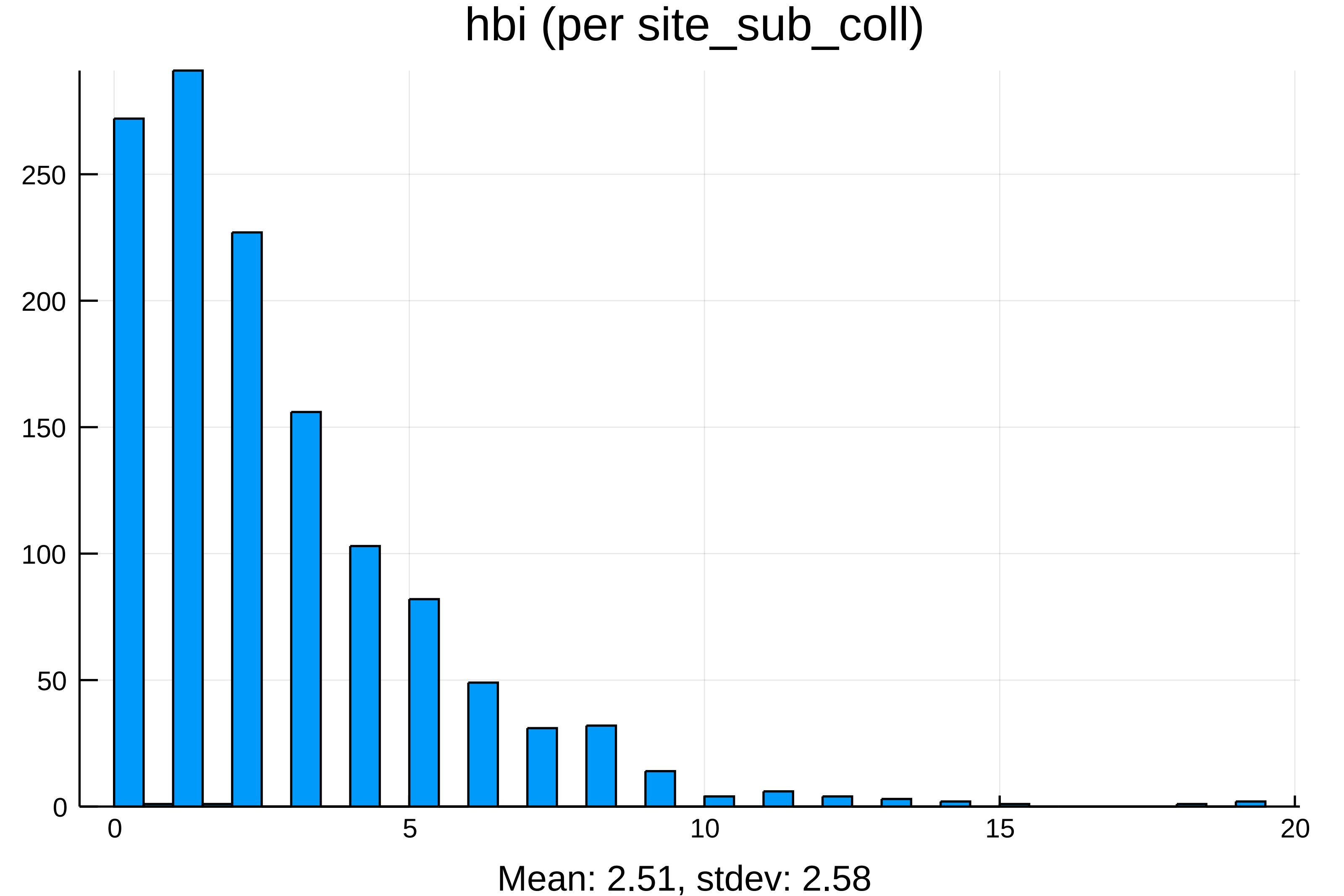
hbi (per site_sub_coll)
0
50
100
150
200
250
0
5
10
15
20
Mean: 2.51, stdev: 2.58

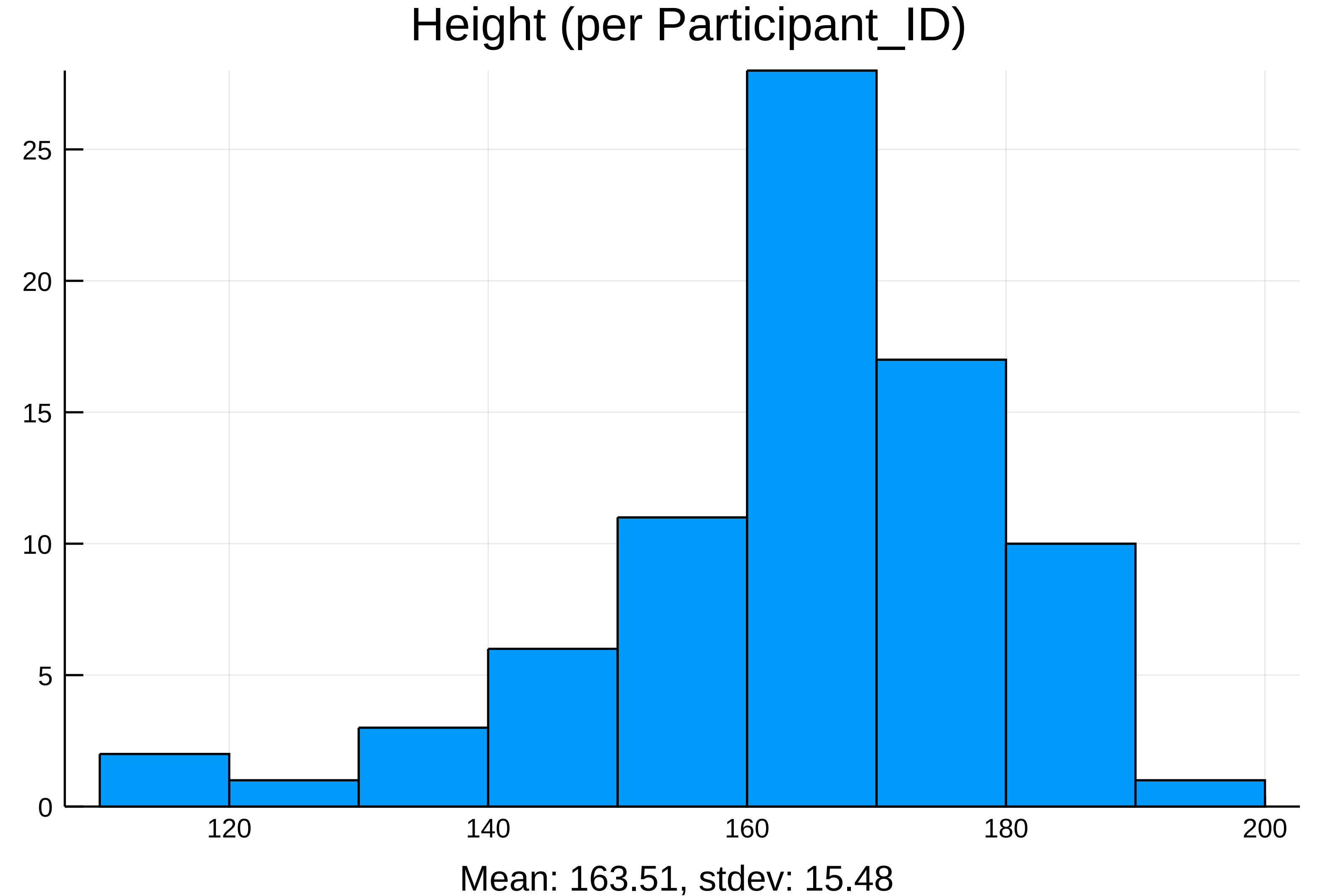
Height (per Participant_ID)
0
5
10
15
20
25
120
140
160
180
200
Mean: 163.51, stdev: 15.48

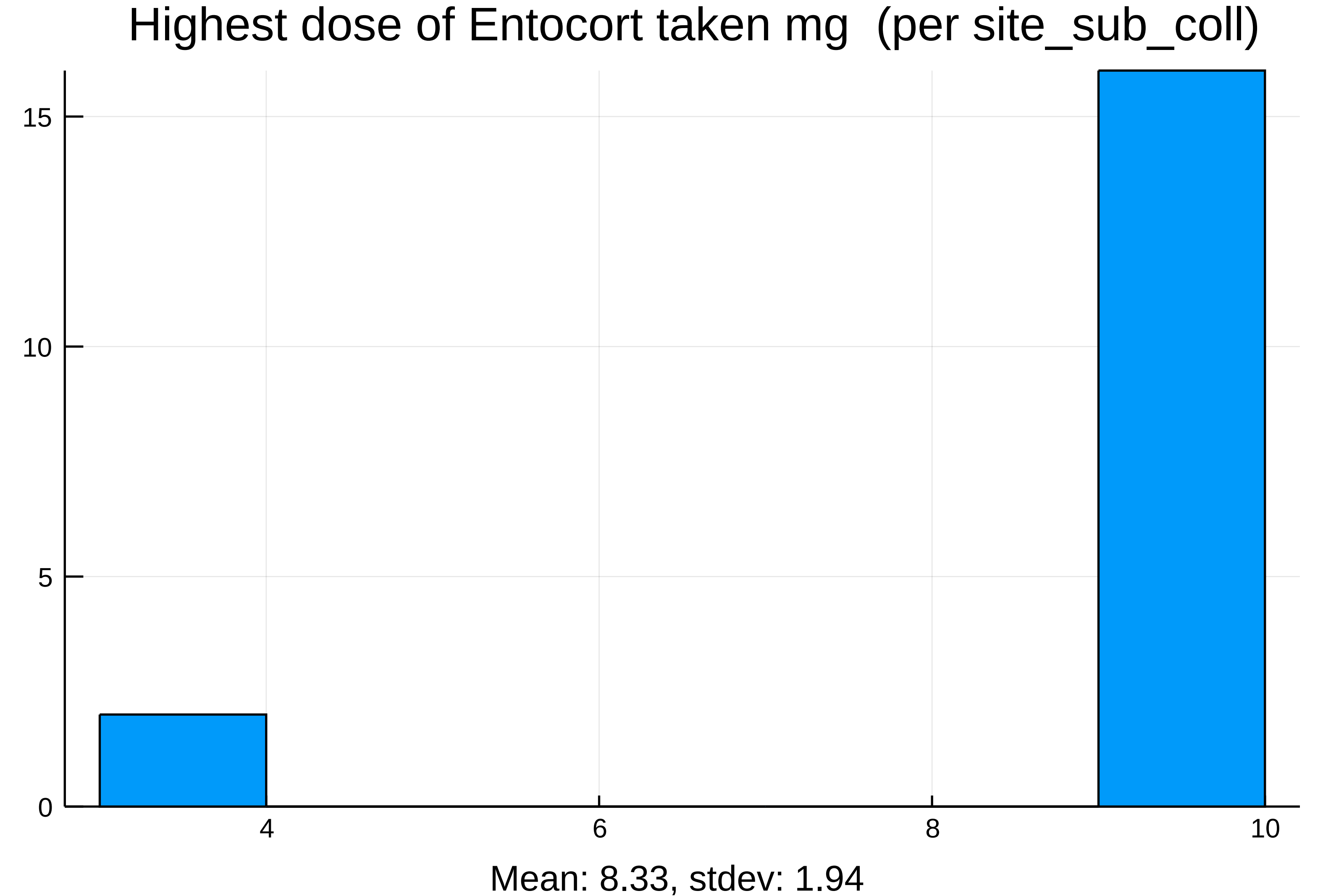
Highest dose of Entocort taken mg (per site_sub_coll)
0
5
10
15
4
6
8
10
Mean: 8.33, stdev: 1.94

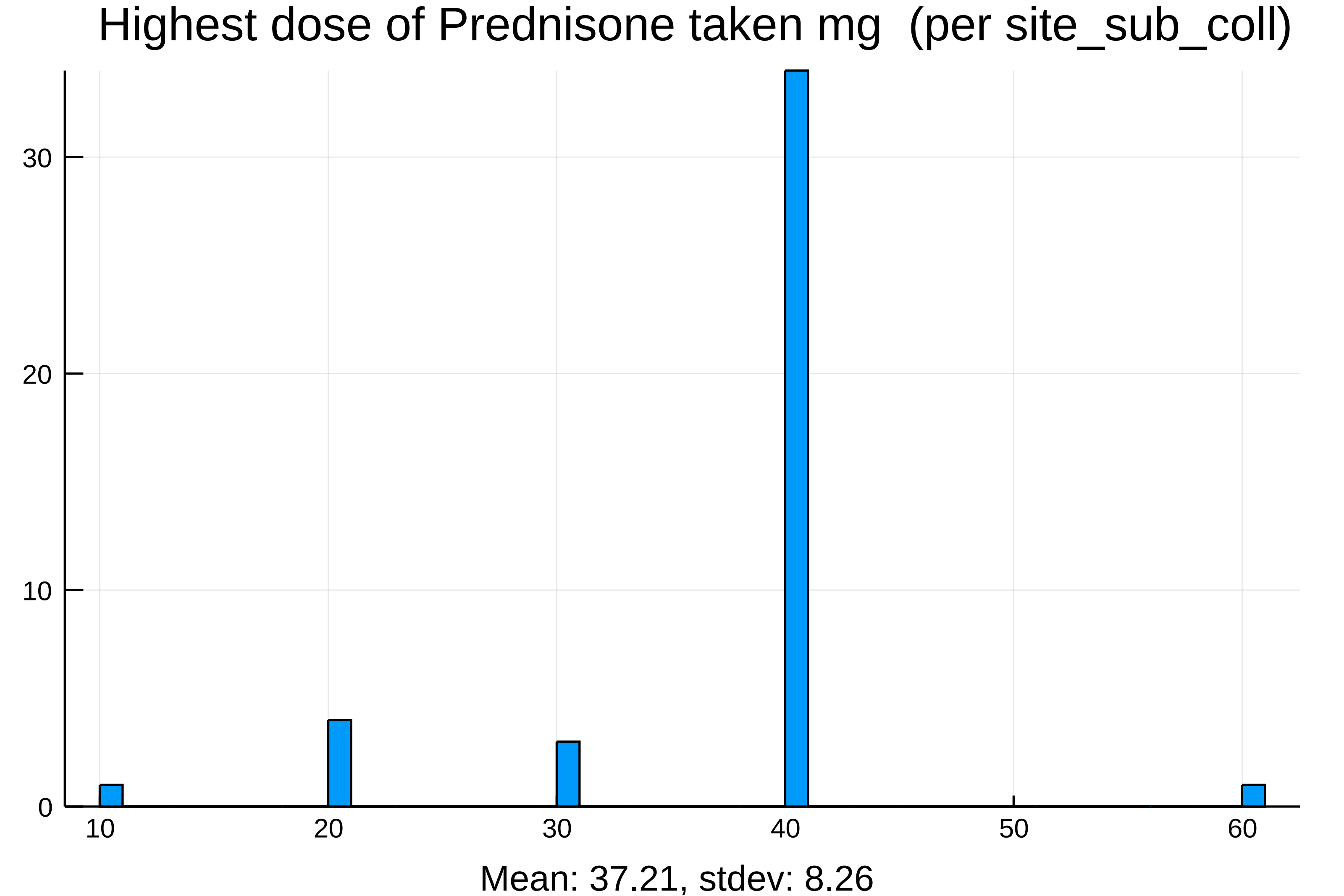
Highest dose of Prednisone taken mg (per site_sub_coll)
30
20
10
0
10
20
30
40
50
60
Mean: 37.21, stdev: 8.26

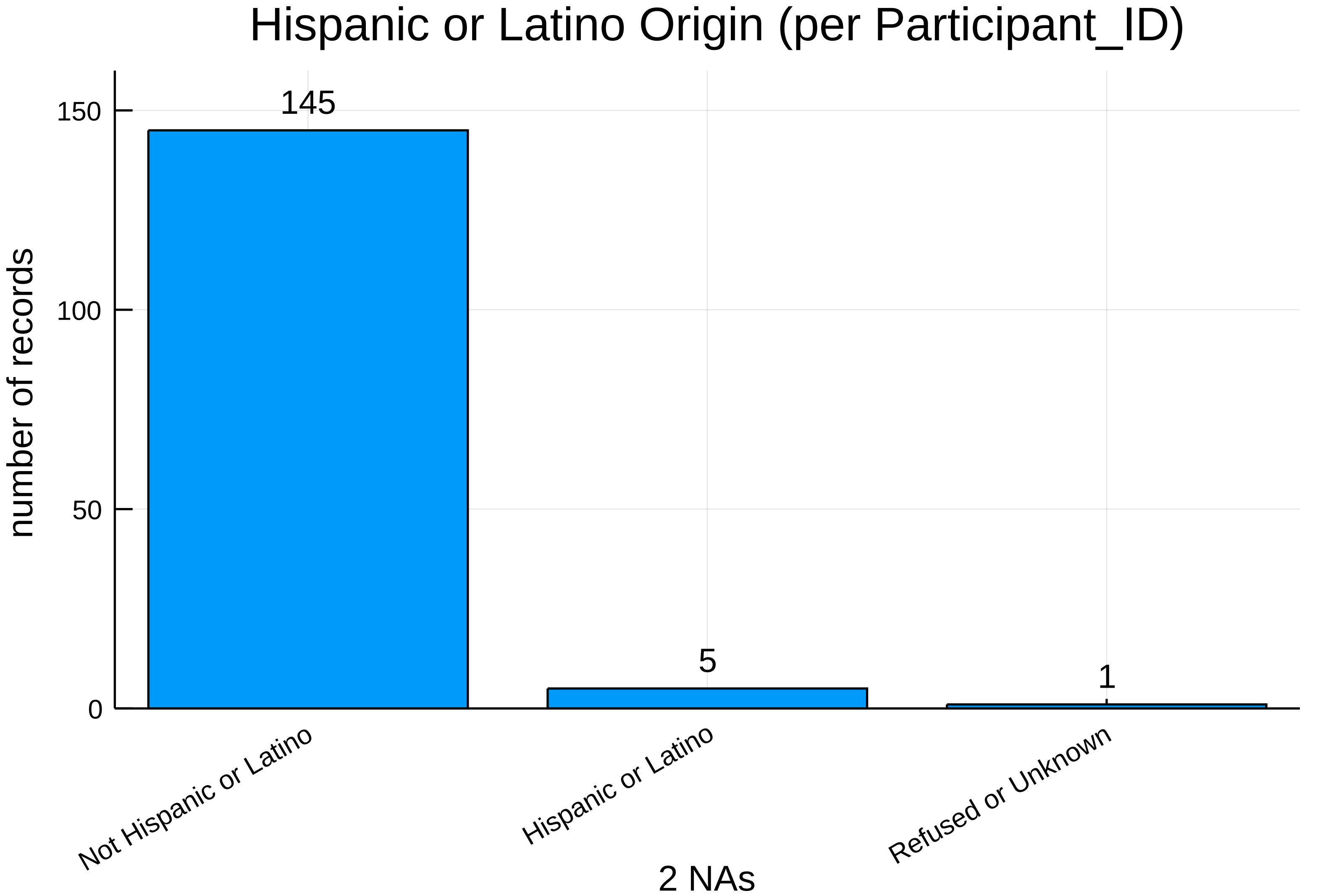
Hispanic or Latino Origin (per Participant_ID)
number of records
150
100
50
0
145
5
1
Not Hispanic or Latino
Hispanic or Latino
Refused or Unknown
2 NAs

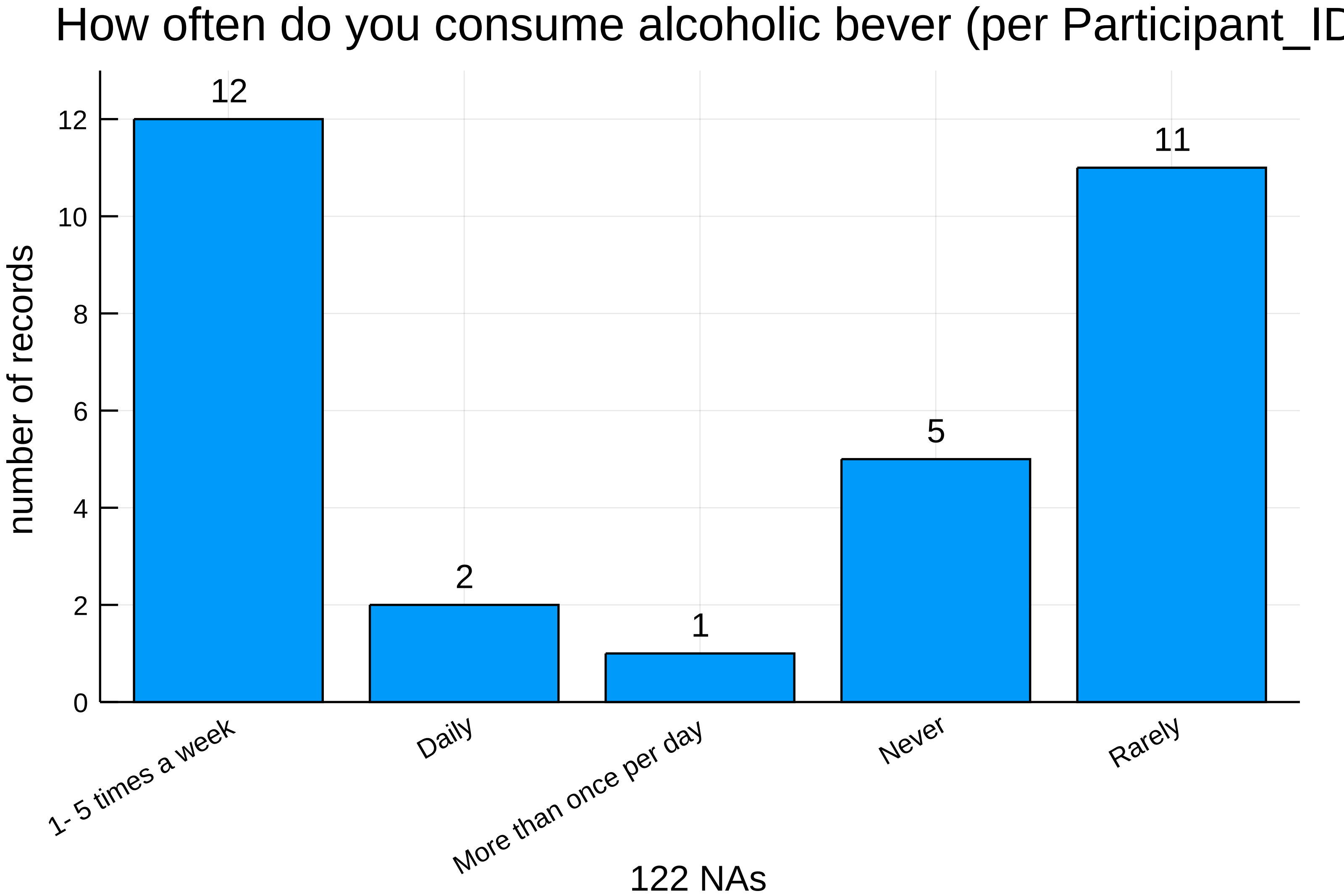
How often do you consume alcoholic bever (per Participant_ID
number of records
0
2
4
6
8
10
12
12
2
1
5
11
1- 5 times a week
Daily
More than once per day
Never
Rarely
122 NAs

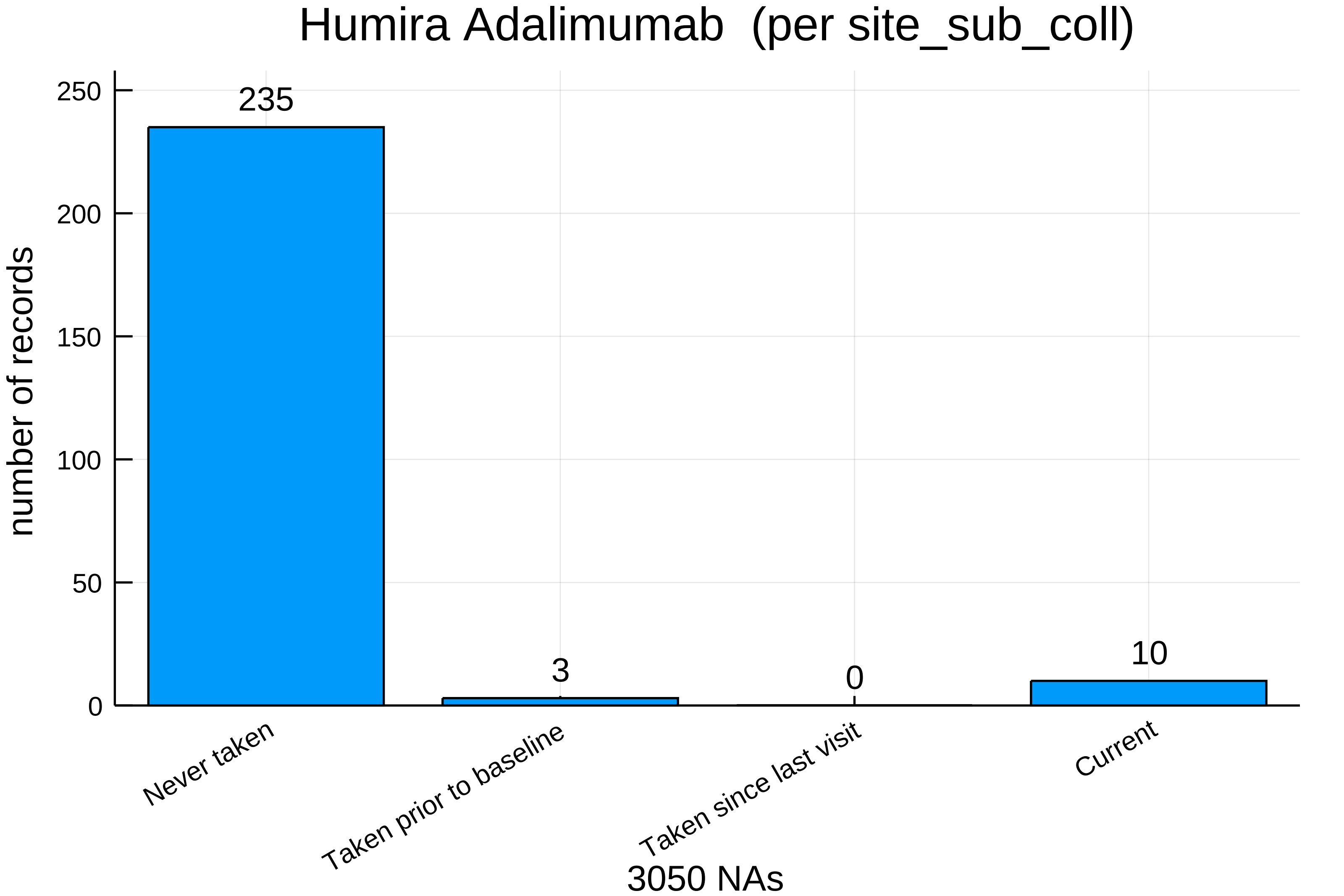
Humira Adalimumab (per site_sub_coll)
number of records
250
200
150
100
50
0
235
3
0
10
Never taken
Taken prior to baseline
Taken since last visit
Current
3050 NAs

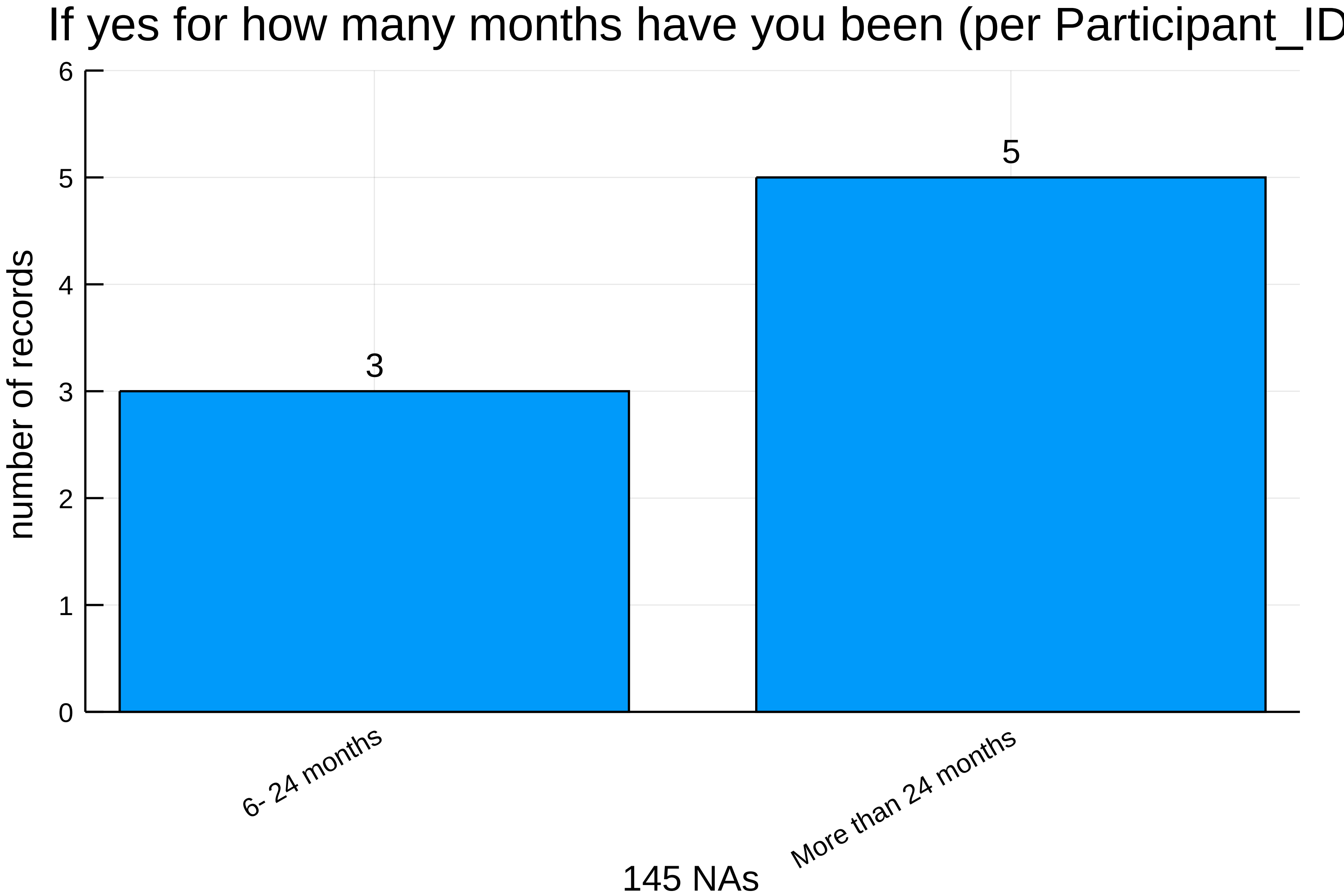
If yes for how many months have you been (per Participant_ID
number of records
6
5
4
3
2
1
0
3
5
6- 24 months
More than 24 months
145 NAs

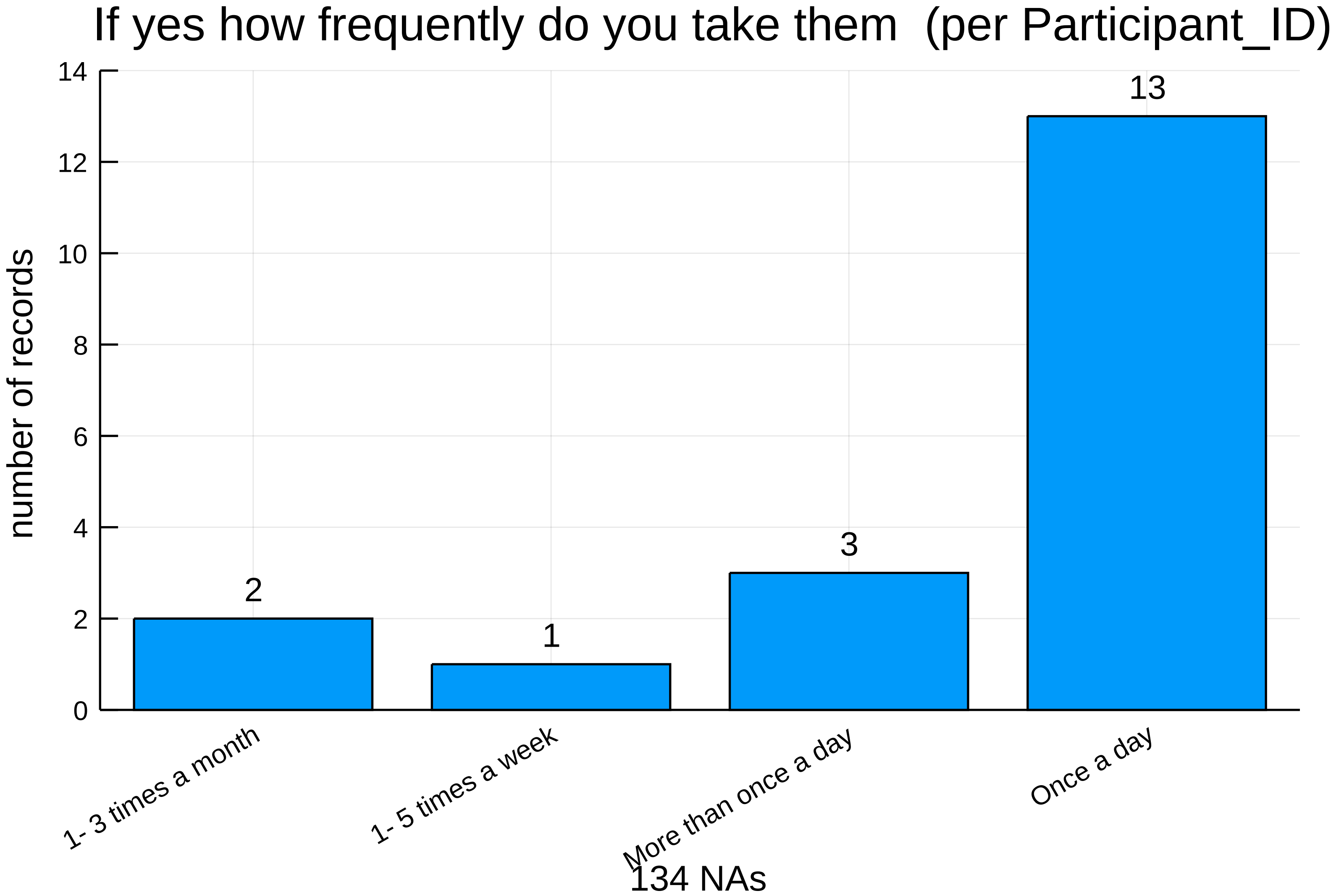
If yes how frequently do you take them (per Participant_ID)
number of records
14
12
10
8
6
4
2
0
2
1
3
13
1- 3 times a month
1- 5 times a week
More than once a day
Once a day
134 NAs

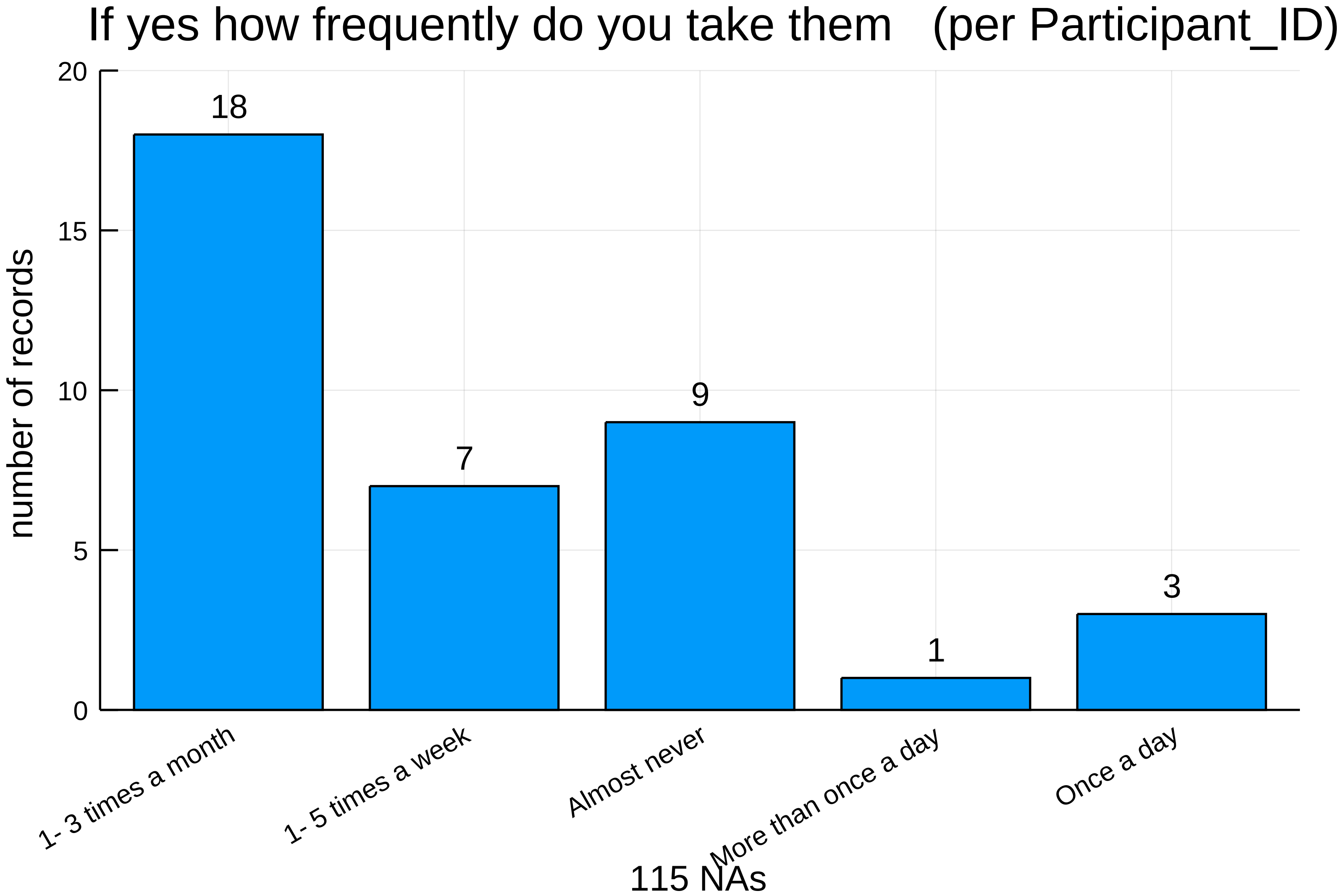
If yes how frequently do you take them (per Participant_ID)
number of records
0
5
10
15
20
18
7
9
1
3
1- 3 times a month
1- 5 times a week
Almost never
More than once a day
Once a day
115 NAs

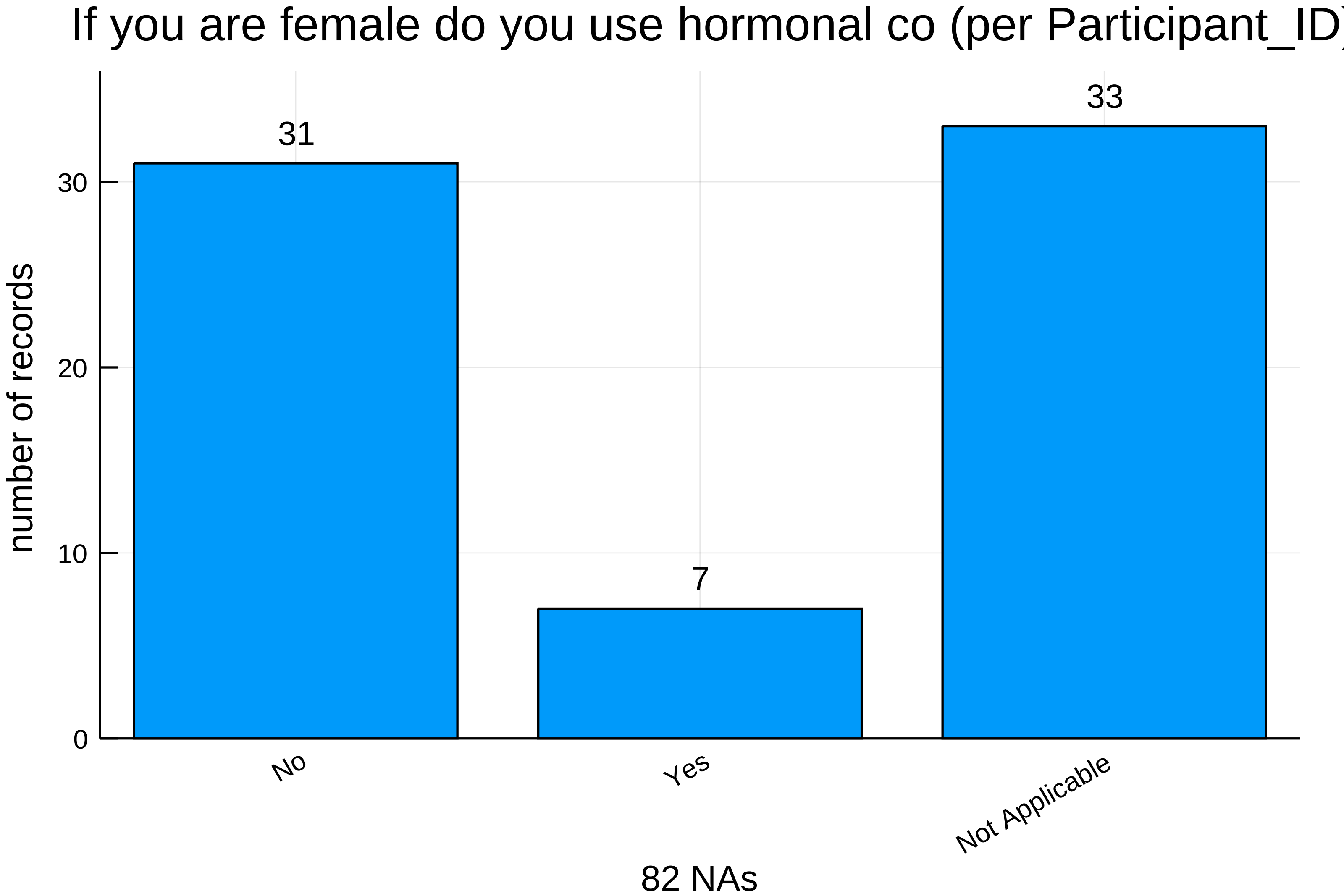
If you are female do you use hormonal co (per Participant_ID)
number of records
0
10
20
30
31
7
33
No
Yes
Not Applicable
82 NAs

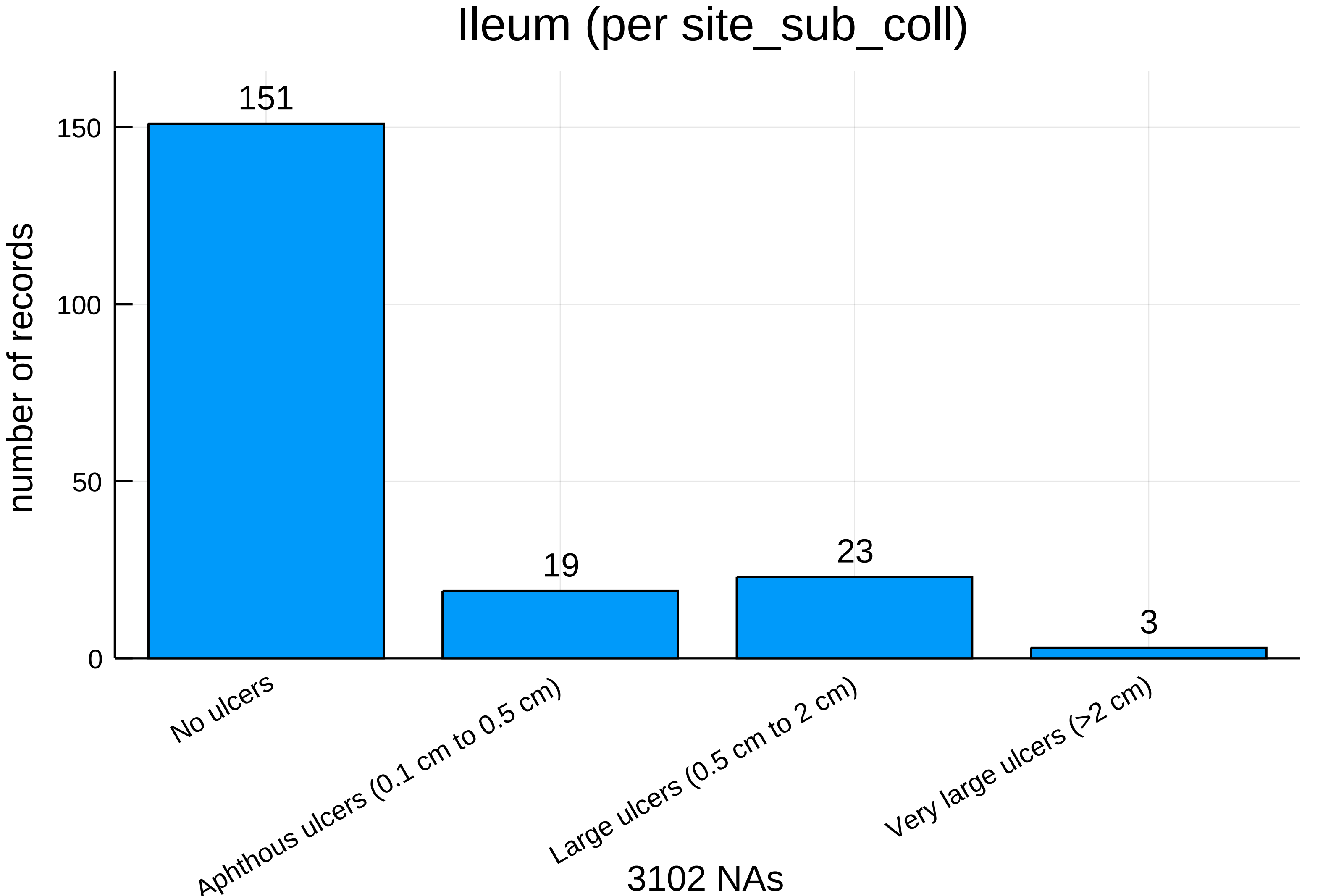
Ileum (per site_sub_coll)
number of records
150
100
50
0
151
19
23
3
No ulcers
Aphthous ulcers (0.1 cm to 0.5 cm)
Large ulcers (0.5 cm to 2 cm)
Very large ulcers (>2 cm)
3102 NAs

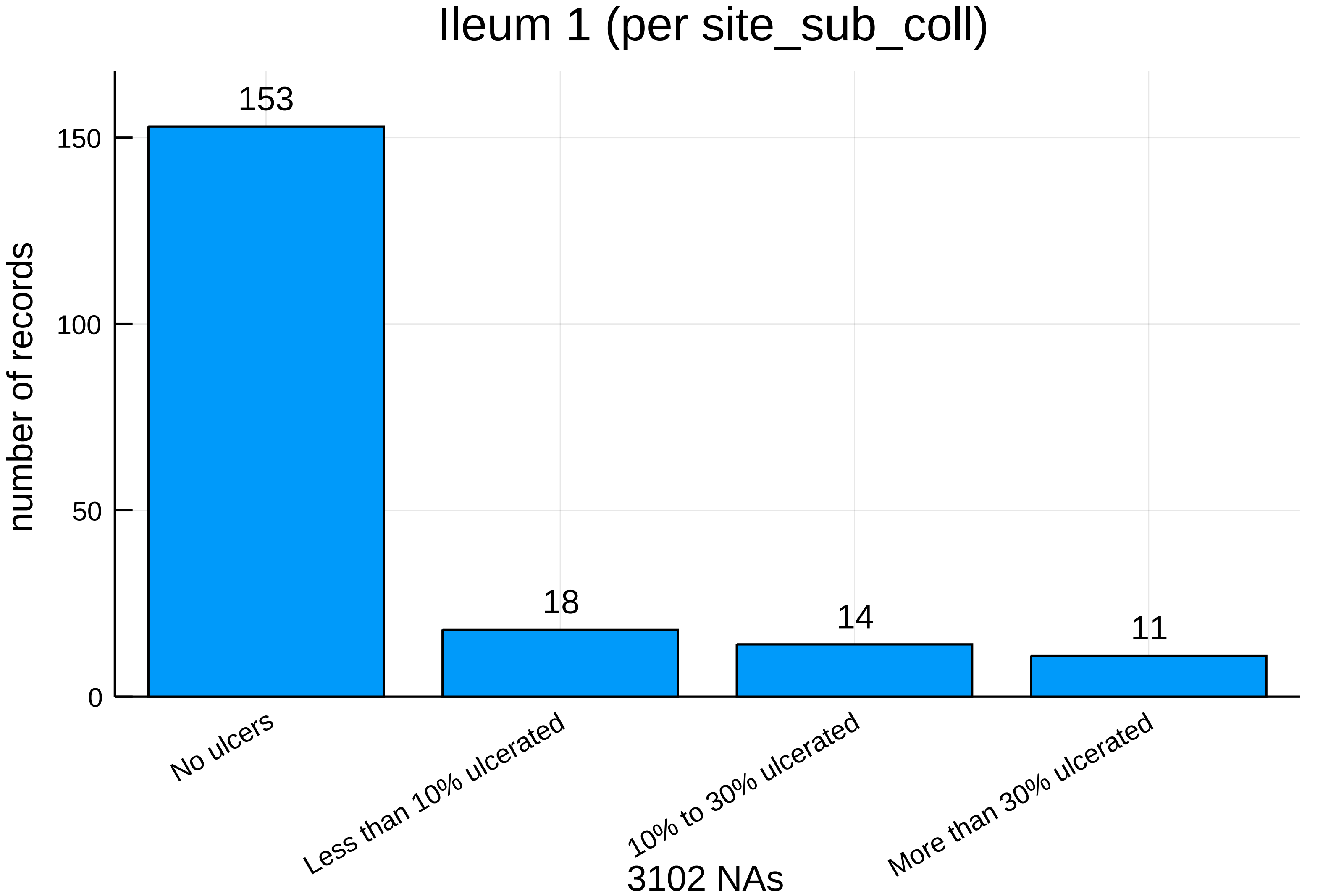
Ileum 1 (per site_sub_coll)
number of records
0
50
100
150
153
18
14
11
No ulcers
Less than 10% ulcerated
10% to 30% ulcerated
More than 30% ulcerated
3102 NAs

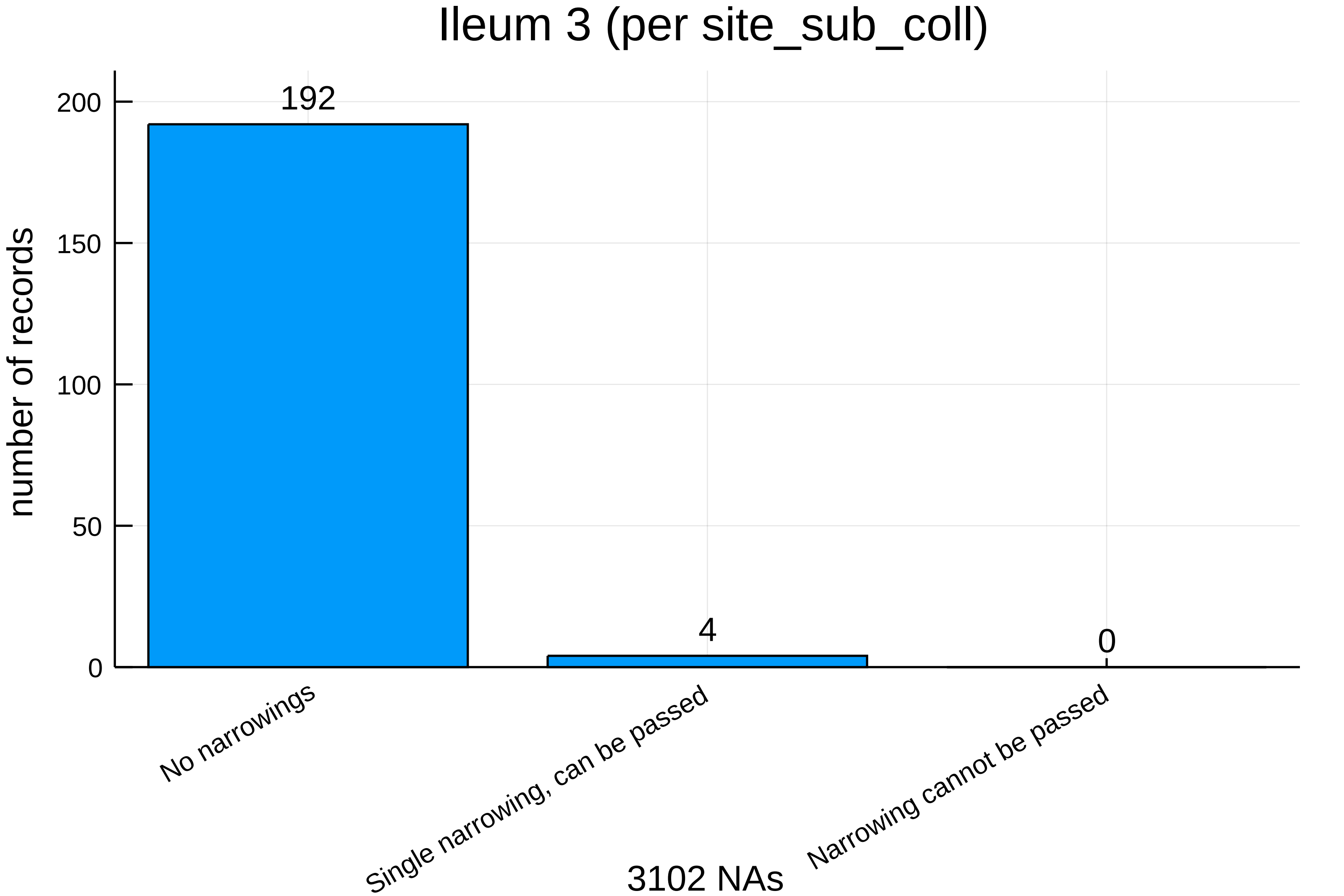
Ileum 3 (per site_sub_coll)
number of records
200
150
100
50
0
192
4
0
No narrowings
Single narrowing, can be passed
Narrowing cannot be passed
3102 NAs

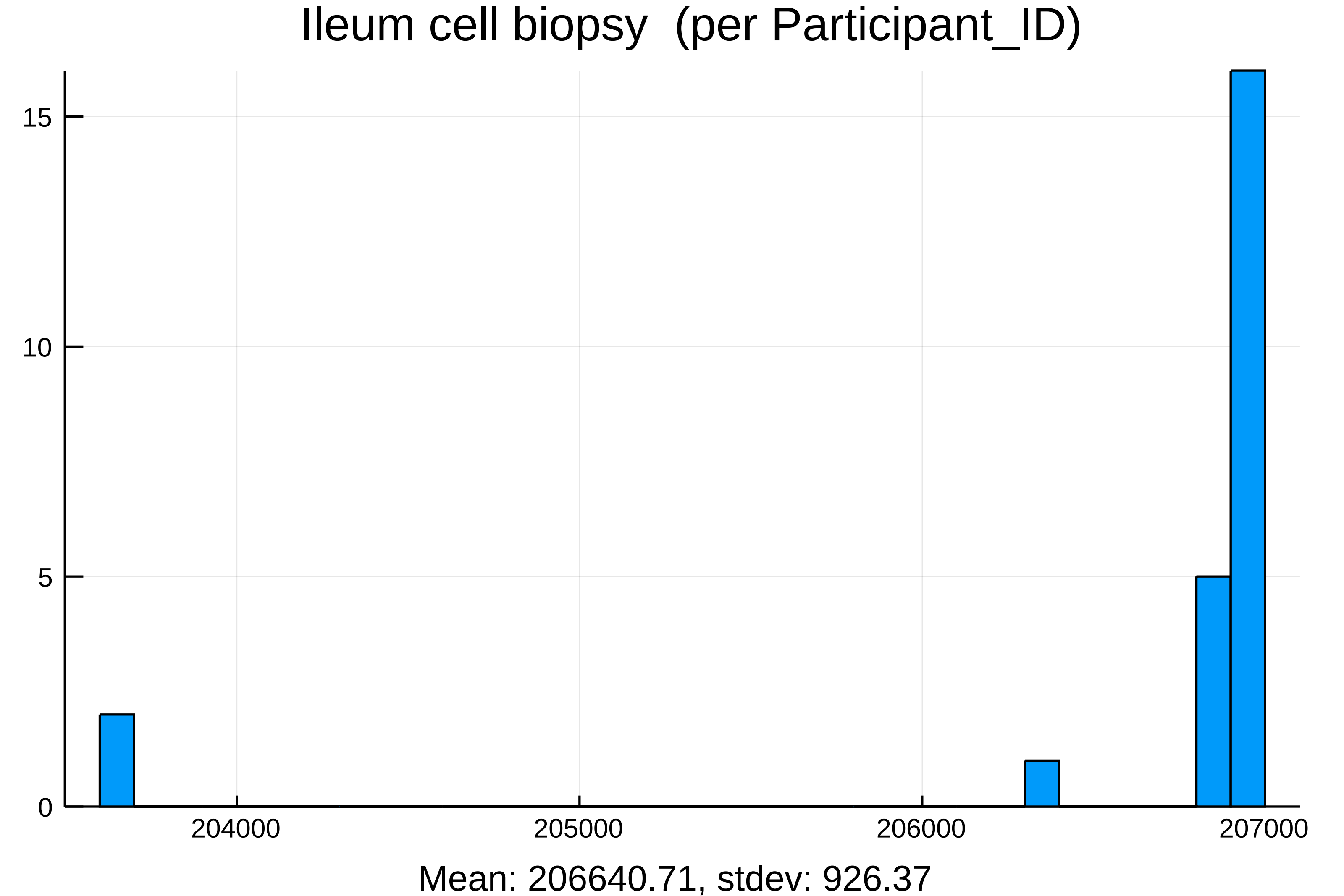
Ileum cell biopsy (per Participant_ID)
15
10
5
0
204000
205000
206000
207000
Mean: 206640.71, stdev: 926.37

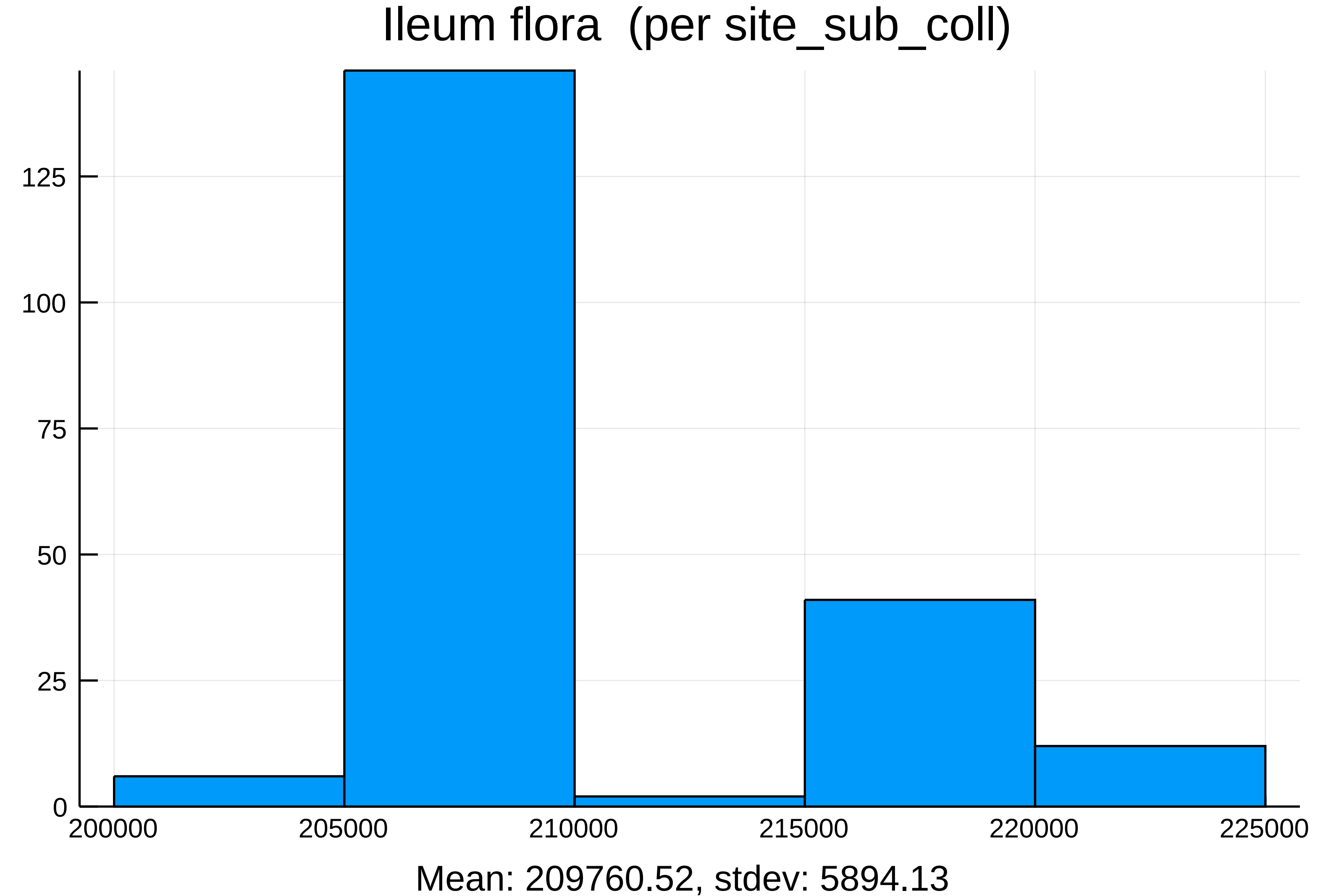
Ileum flora (per site_sub_coll)
125
100
75
50
25
0
200000
205000
210000
215000
220000
225000
Mean: 209760.52, stdev: 5894.13

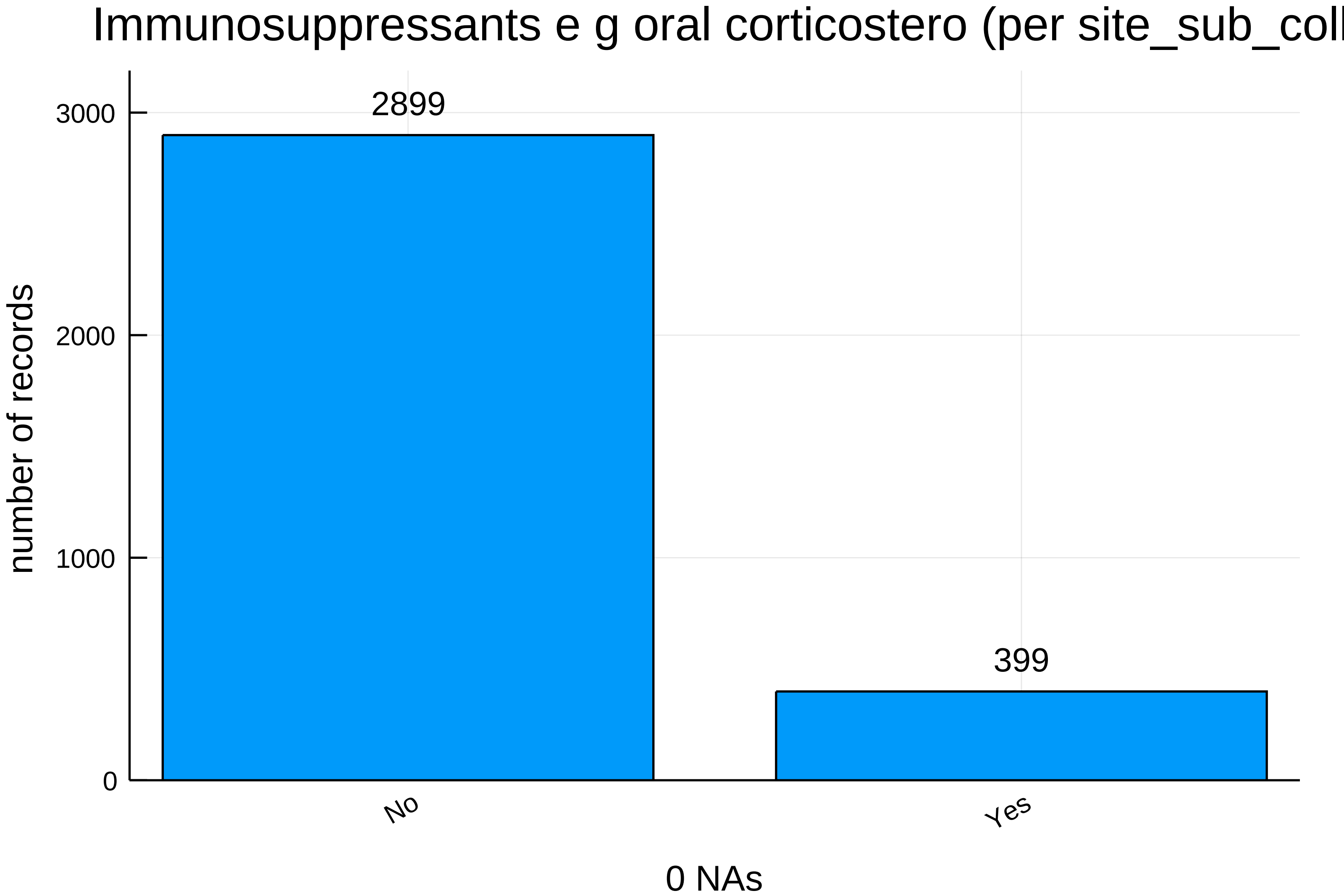
Immunosuppressants e g oral corticostero (per site_sub_coll
number of records
3000
2000
1000
0
2899
399
No
Yes
0 NAs

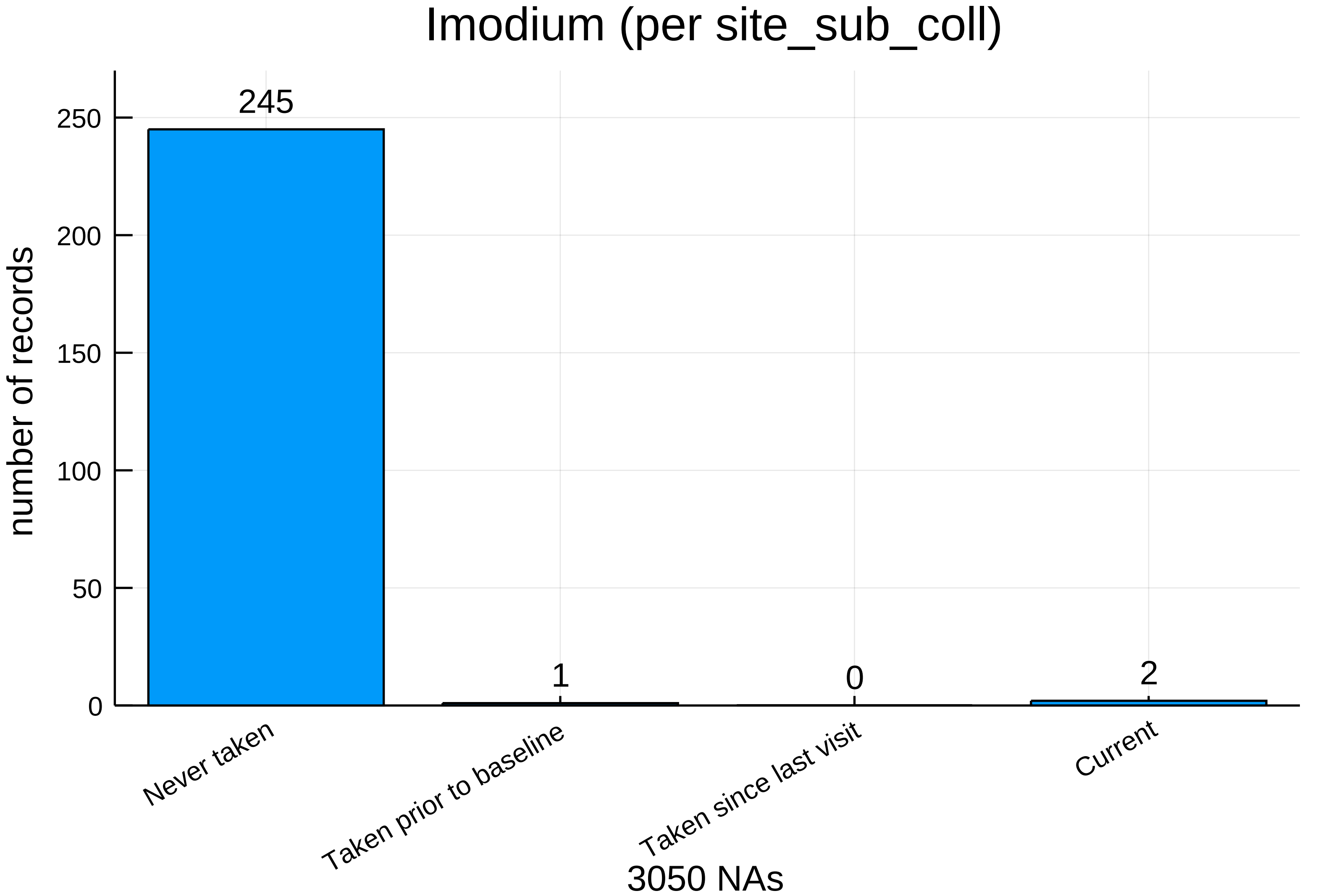
Imodium (per site_sub_coll)
number of records
0
50
100
150
200
250
245
1
0
2
Never taken
Taken prior to baseline
Taken since last visit
Current
3050 NAs

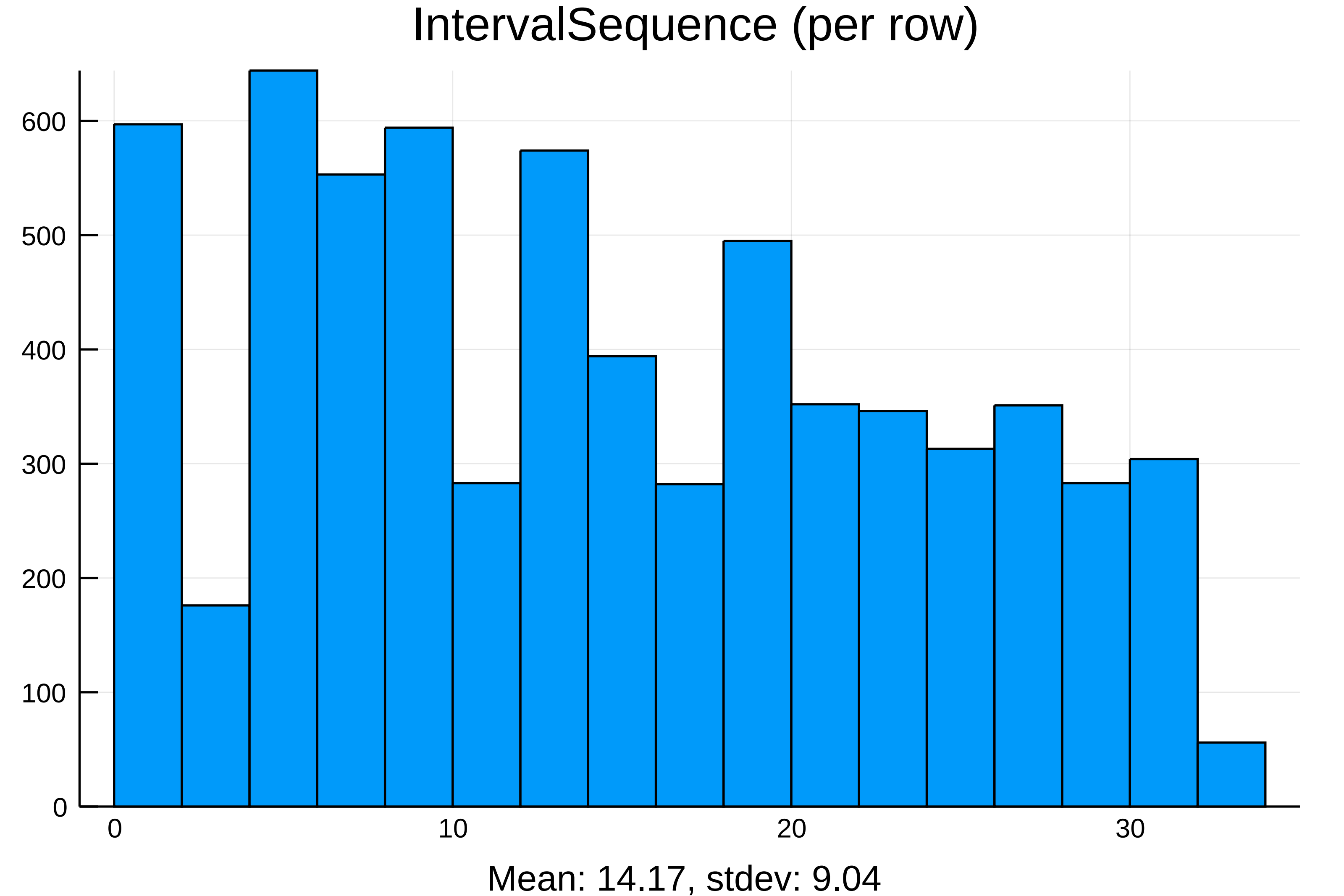
IntervalSequence (per row)
600
500
400
300
200
100
0
0
10
20
30
Mean: 14.17, stdev: 9.04

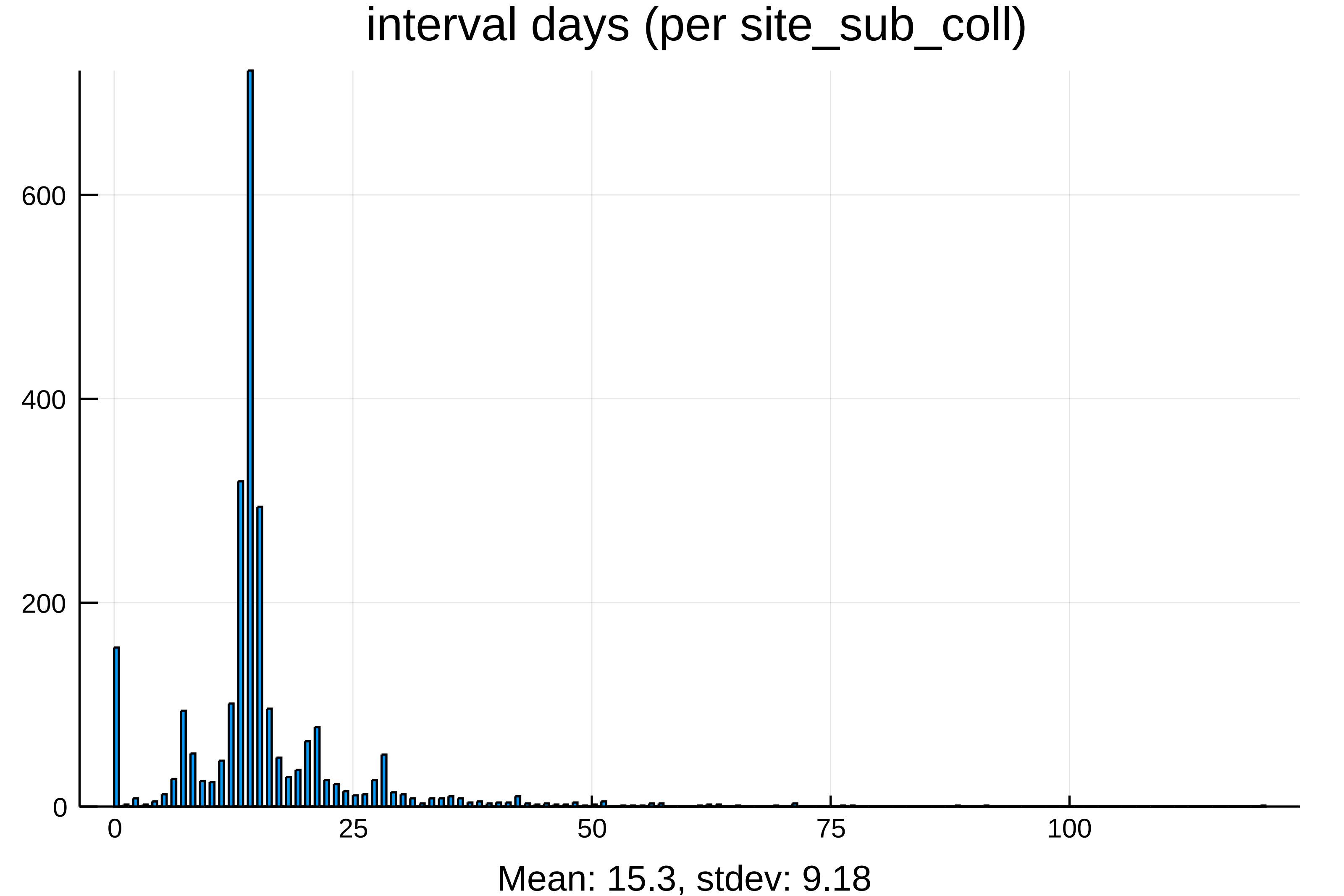
interval days (per site_sub_coll)
600
400
200
0
0
25
50
75
100
Mean: 15.3, stdev: 9.18

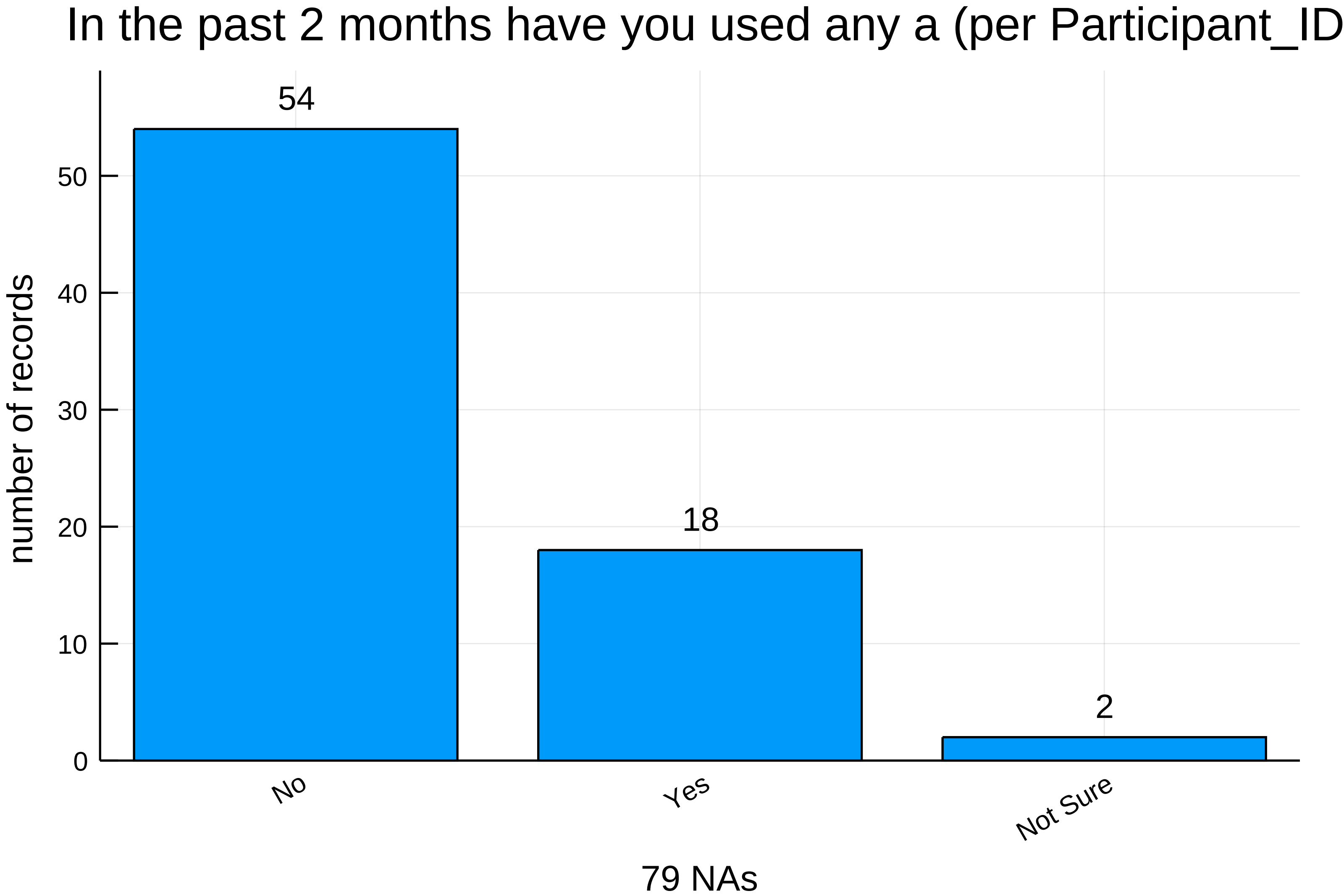
In the past 2 months have you used any a (per Participant_ID
number of records
0
10
20
30
40
50
54
18
2
No
Yes
Not Sure
79 NAs

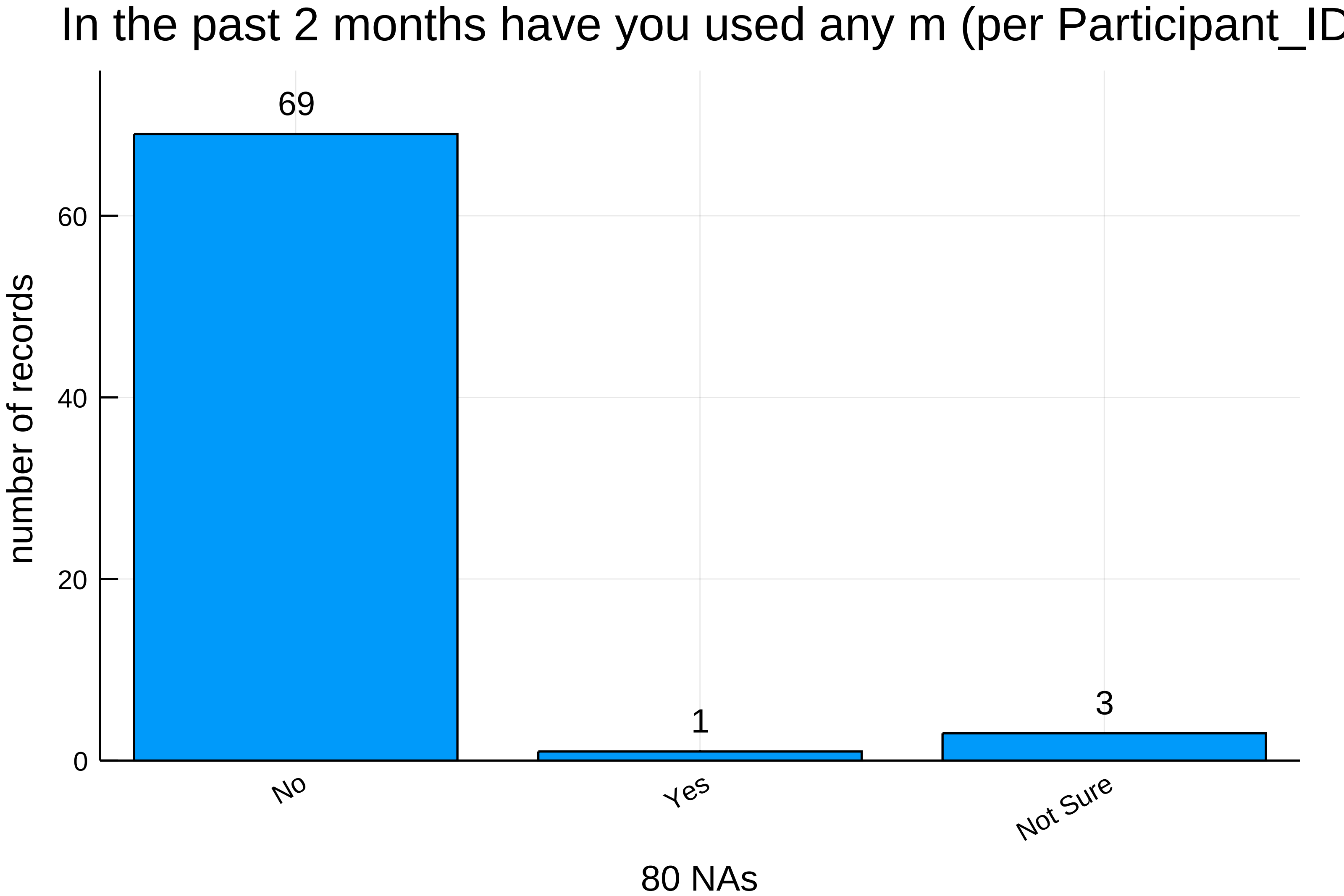
In the past 2 months have you used any m (per Participant_ID
number of records
69
1
3
60
40
20
0
No
Yes
Not Sure
80 NAs

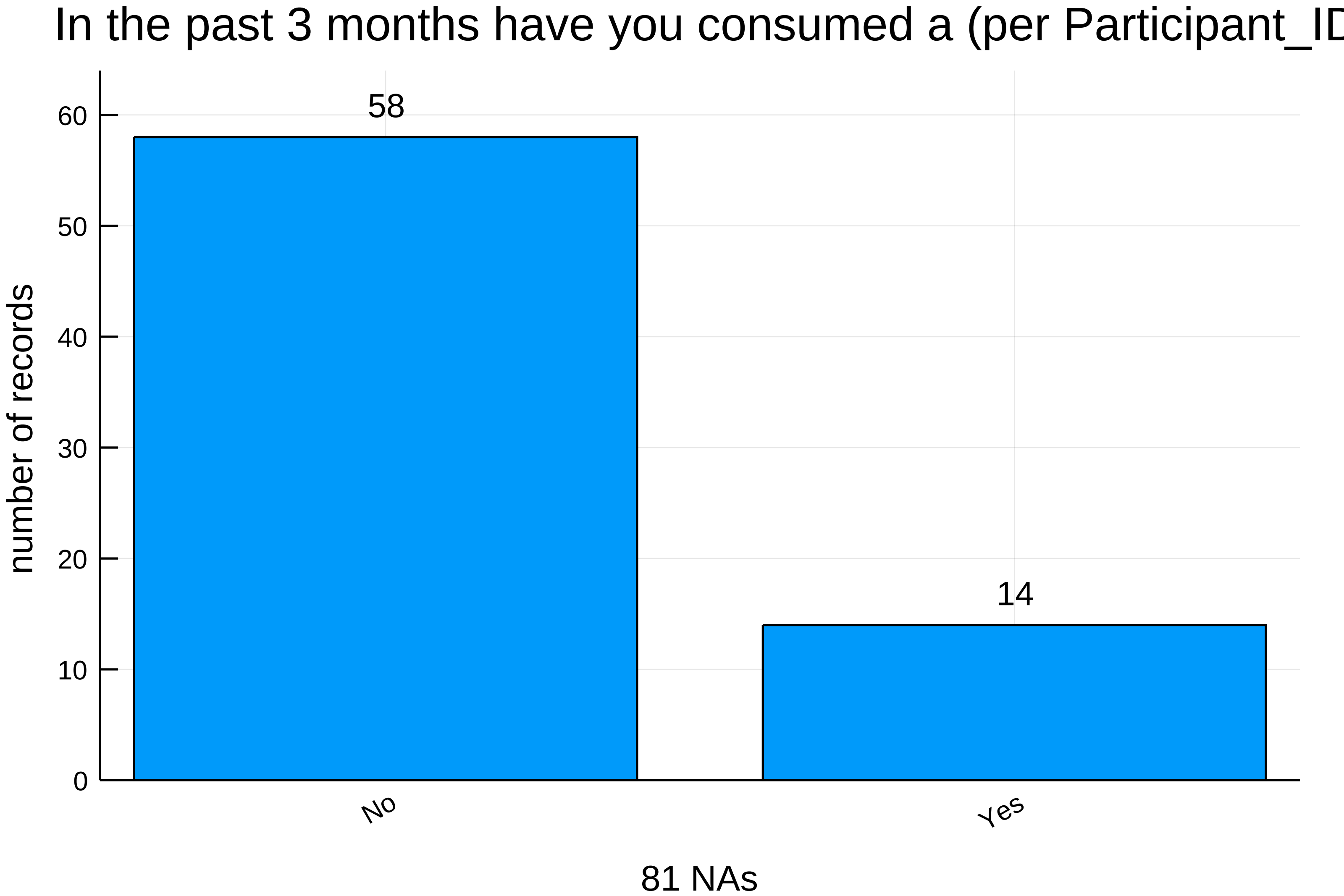
In the past 3 months have you consumed a (per Participant_ID
number of records
60
50
40
30
20
10
0
58
14
No
Yes
81 NAs

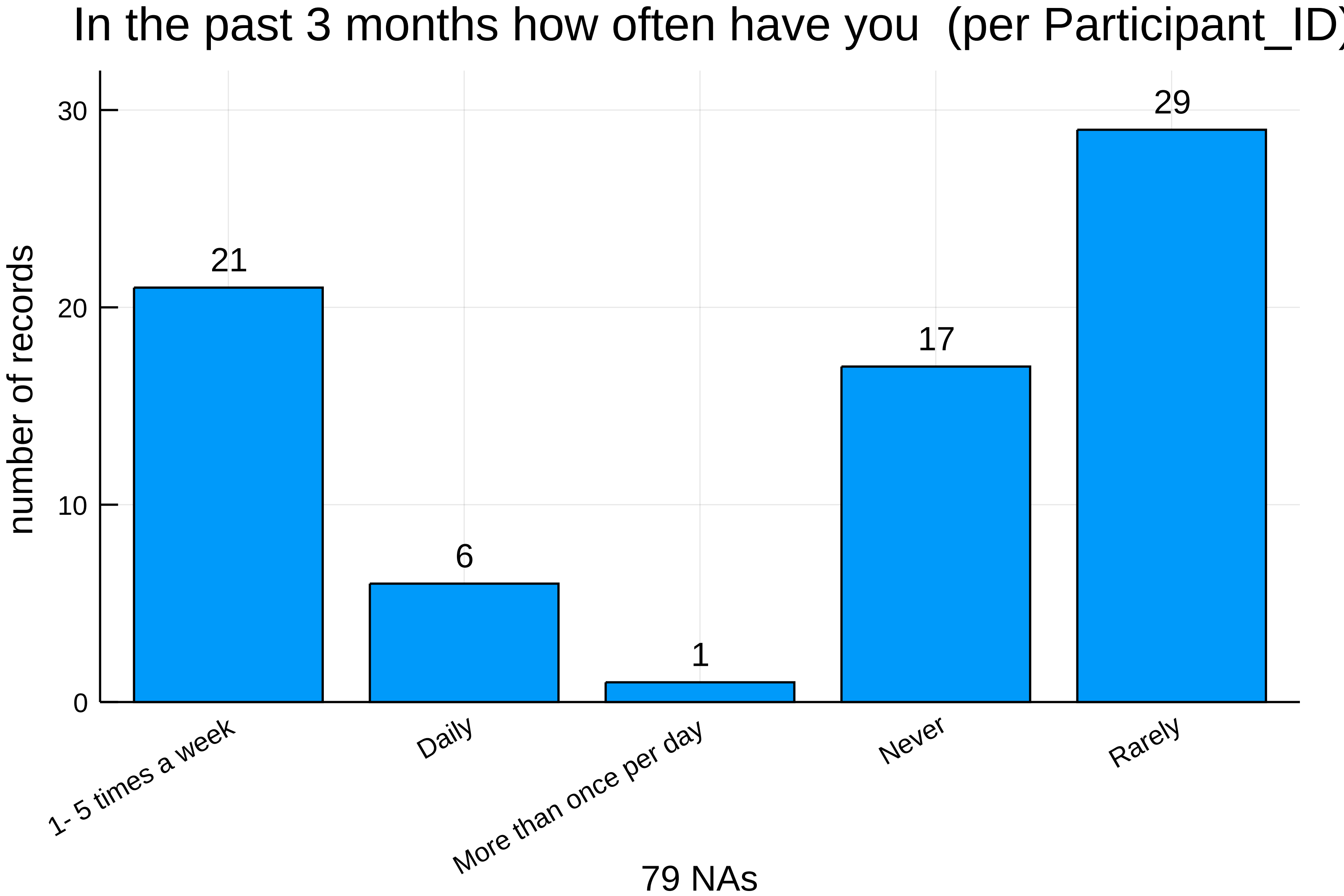
In the past 3 months how often have you (per Participant_ID)
number of records
30
20
10
0
21
6
1
17
29
1- 5 times a week
Daily
More than once per day
Never
Rarely
79 NAs

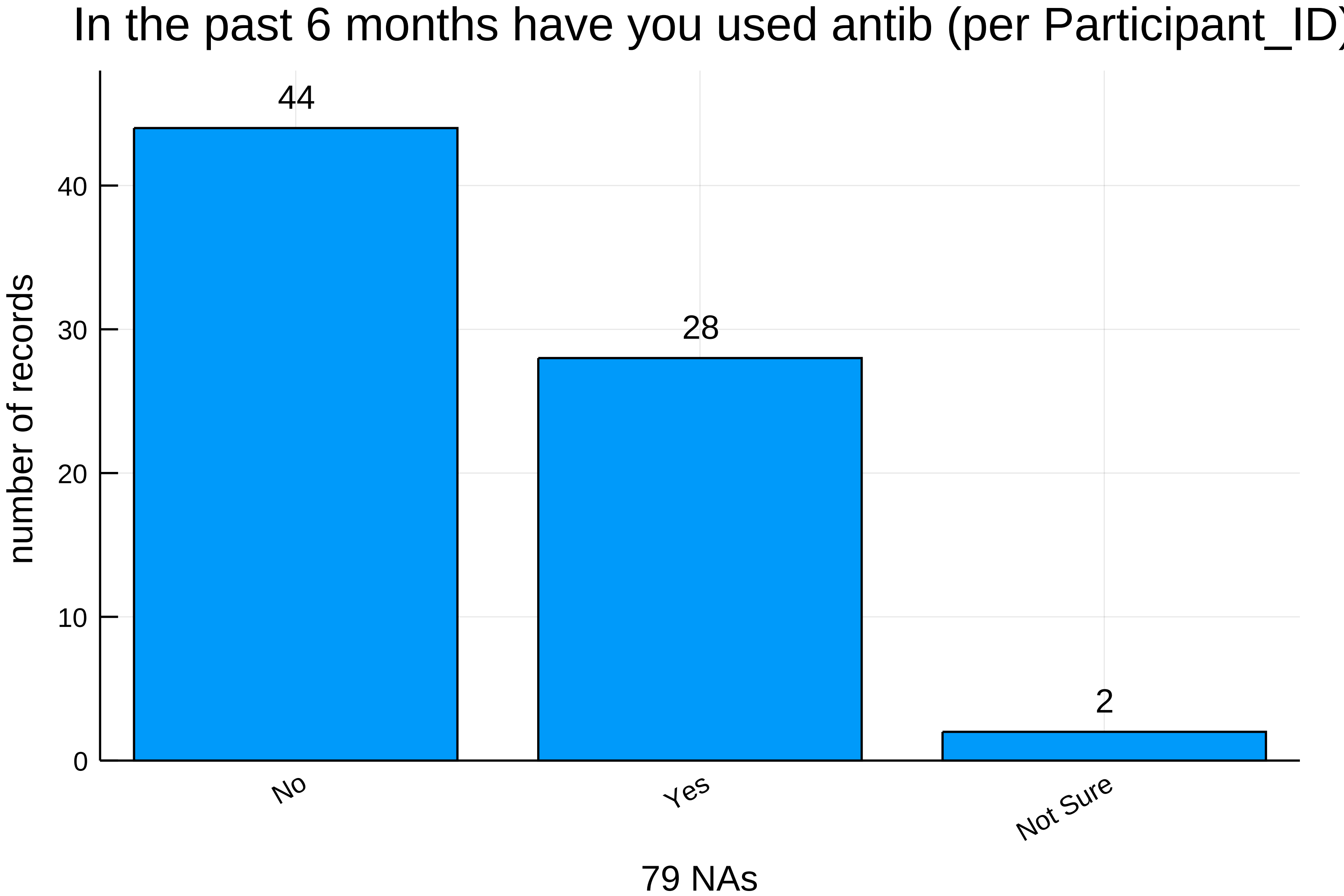
In the past 6 months have you used antib (per Participant_ID)
number of records
0
10
20
30
40
44
28
2
No
Yes
Not Sure
79 NAs

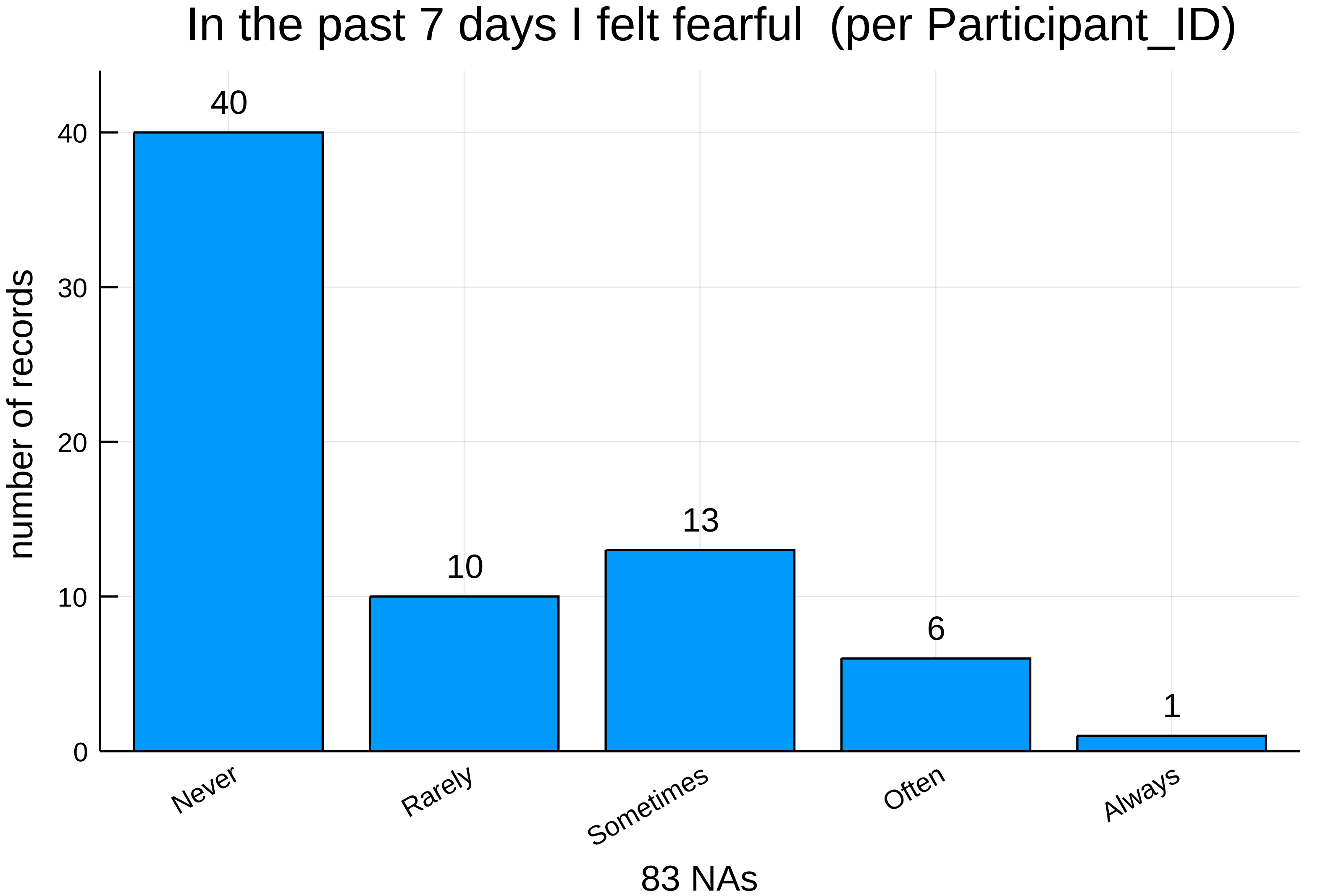
In the past 7 days I felt fearful (per Participant_ID)
number of records
0
10
20
30
40
40
10
13
6
1
Never
Rarely
Sometimes
Often
Always
83 NAs

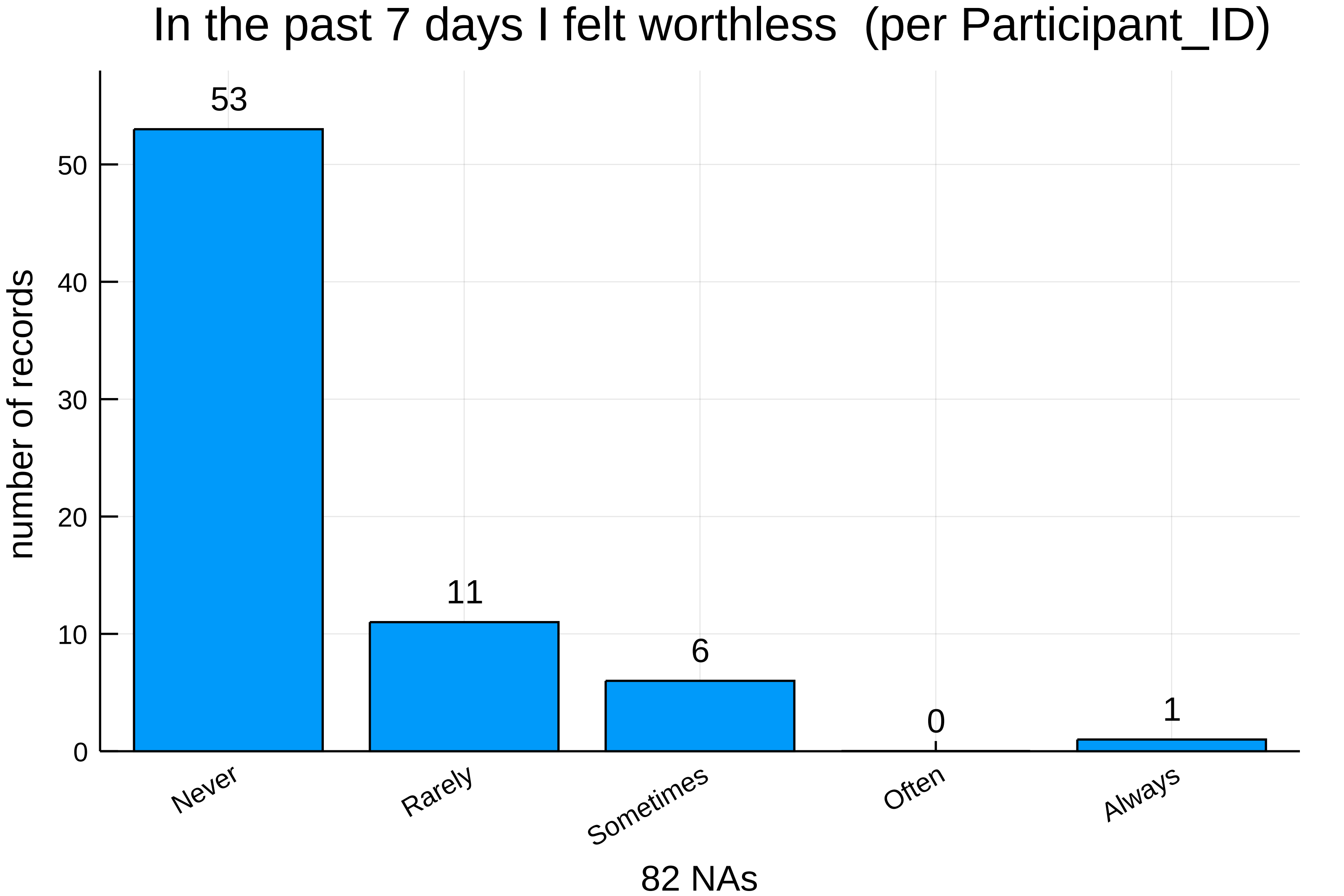
In the past 7 days I felt worthless (per Participant_ID)
number of records
0
10
20
30
40
50
53
11
6
0
1
Never
Rarely
Sometimes
Often
Always
82 NAs

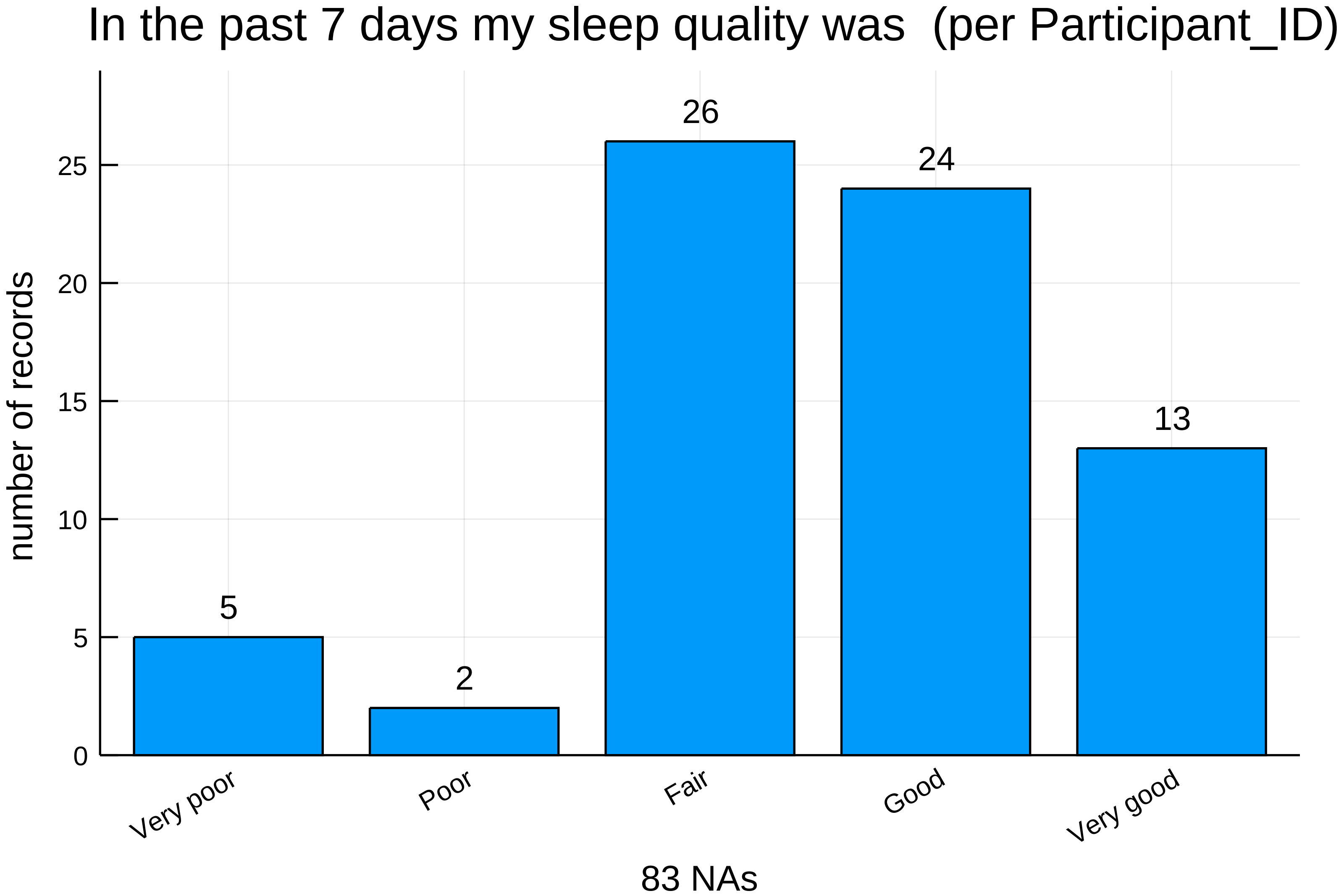
In the past 7 days my sleep quality was (per Participant_ID)
number of records
0
5
10
15
20
25
5
2
26
24
13
Very poor
Poor
Fair
Good
Very good
83 NAs

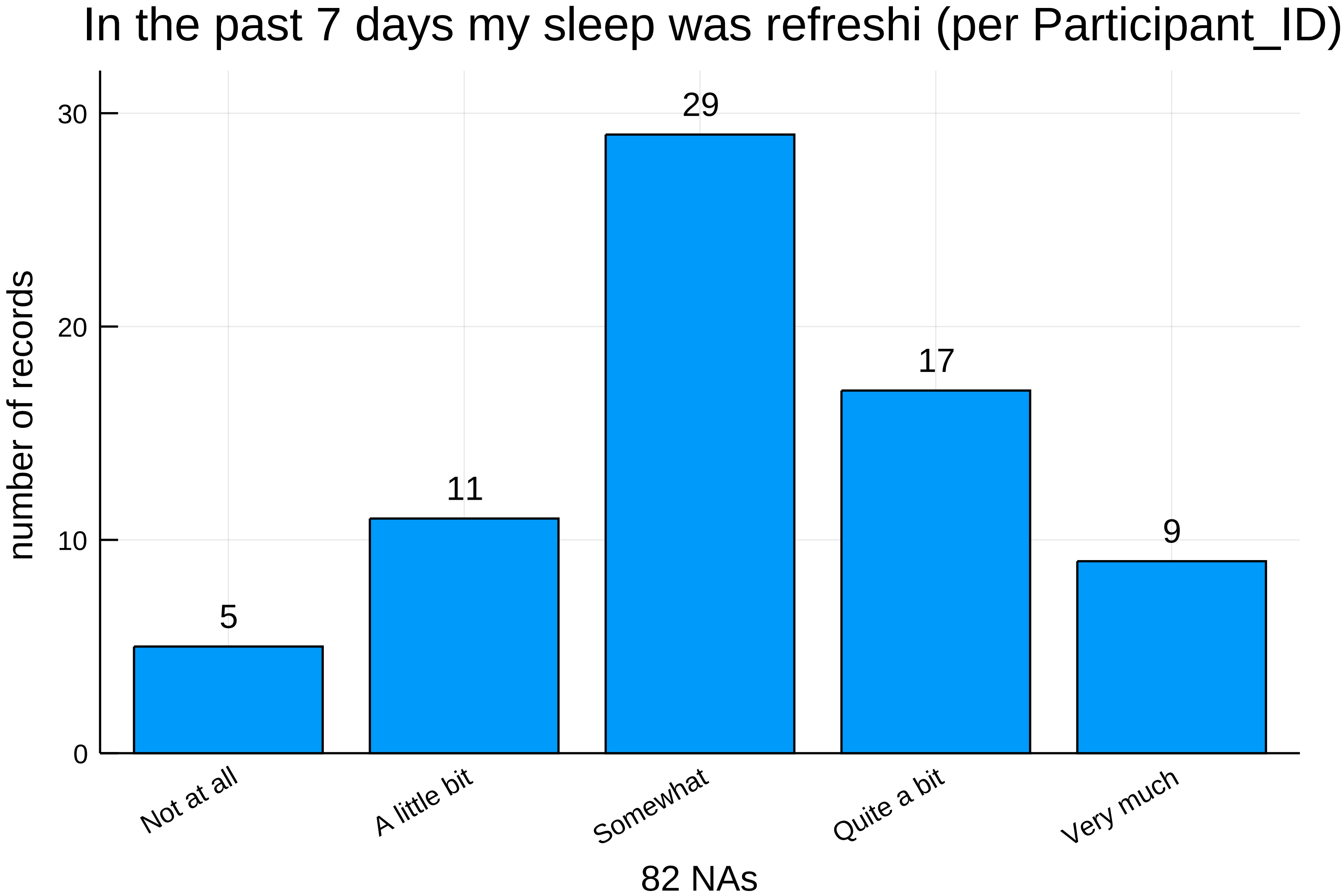

In the past 7 days my sleep was refreshi (per Participant_ID)
number of records
0
10
20
30
5
11
29
17
9
Not at all
A little bit
Somewhat
Quite a bit
Very much
82 NAs

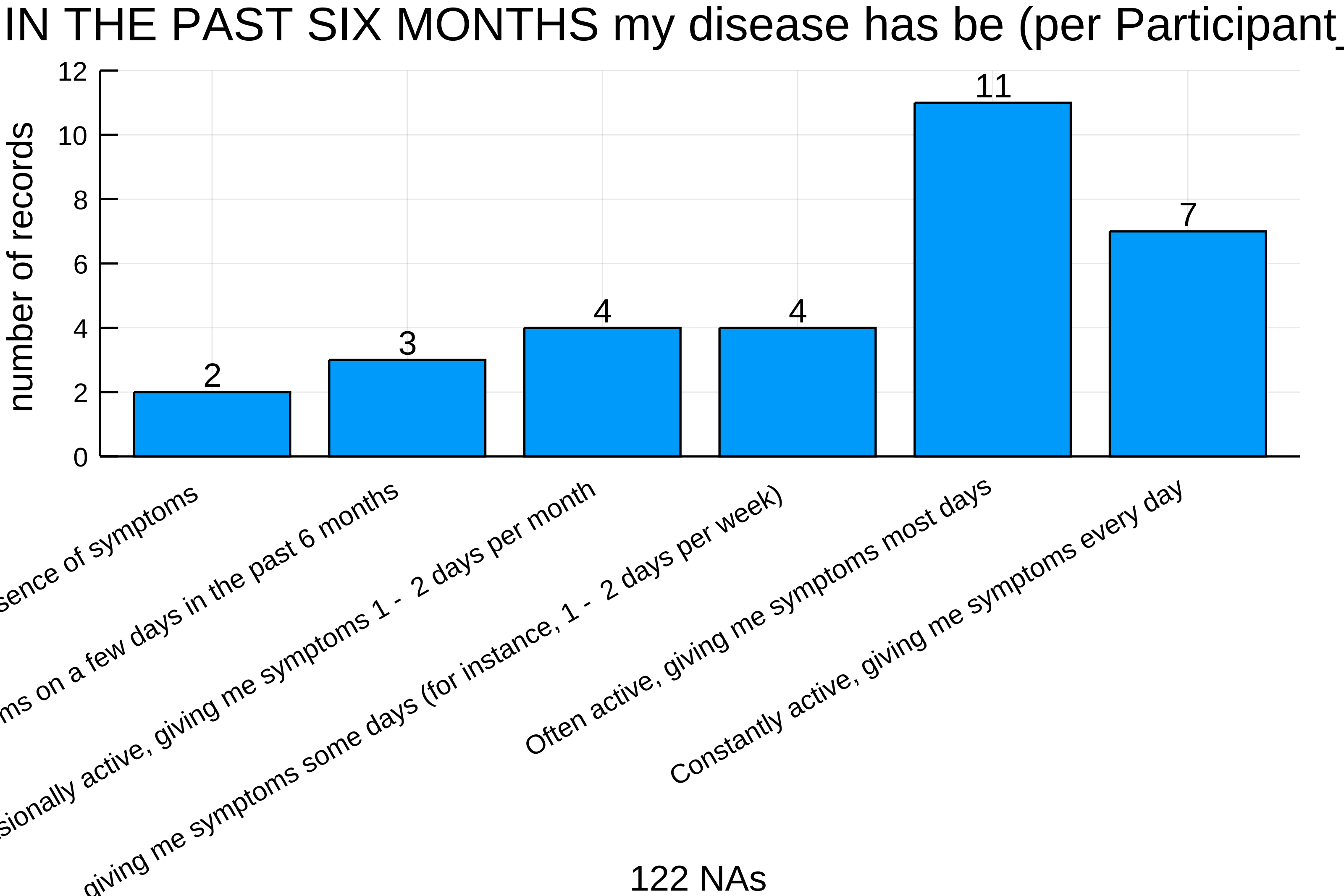
IN THE PAST SIX MONTHS my disease has be (per Participant_
number of records
0
2
4
6
8
10
12
2
3
4
4
11
7
sence of symptoms
ms on a few days in the past 6 months
sionally active, giving me symptoms 1 - 2 days per month
giving me symptoms some days (for instance, 1 - 2 days per week)
Often active, giving me symptoms most days
Constantly active, giving me symptoms every day
122 NAs

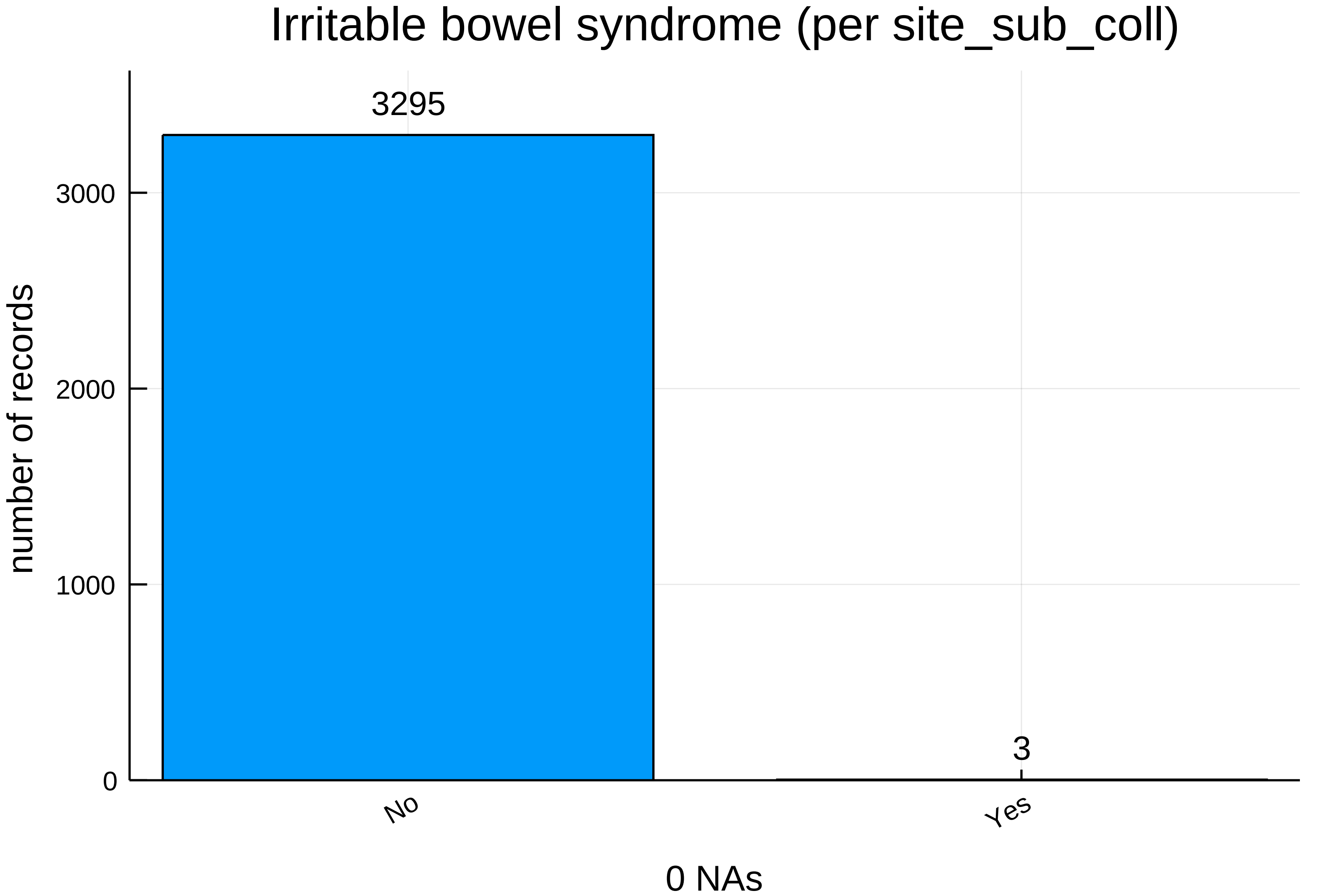

Irritable bowel syndrome (per site_sub_coll)
number of records
3295
3
3000
2000
1000
0
No
Yes
0 NAs

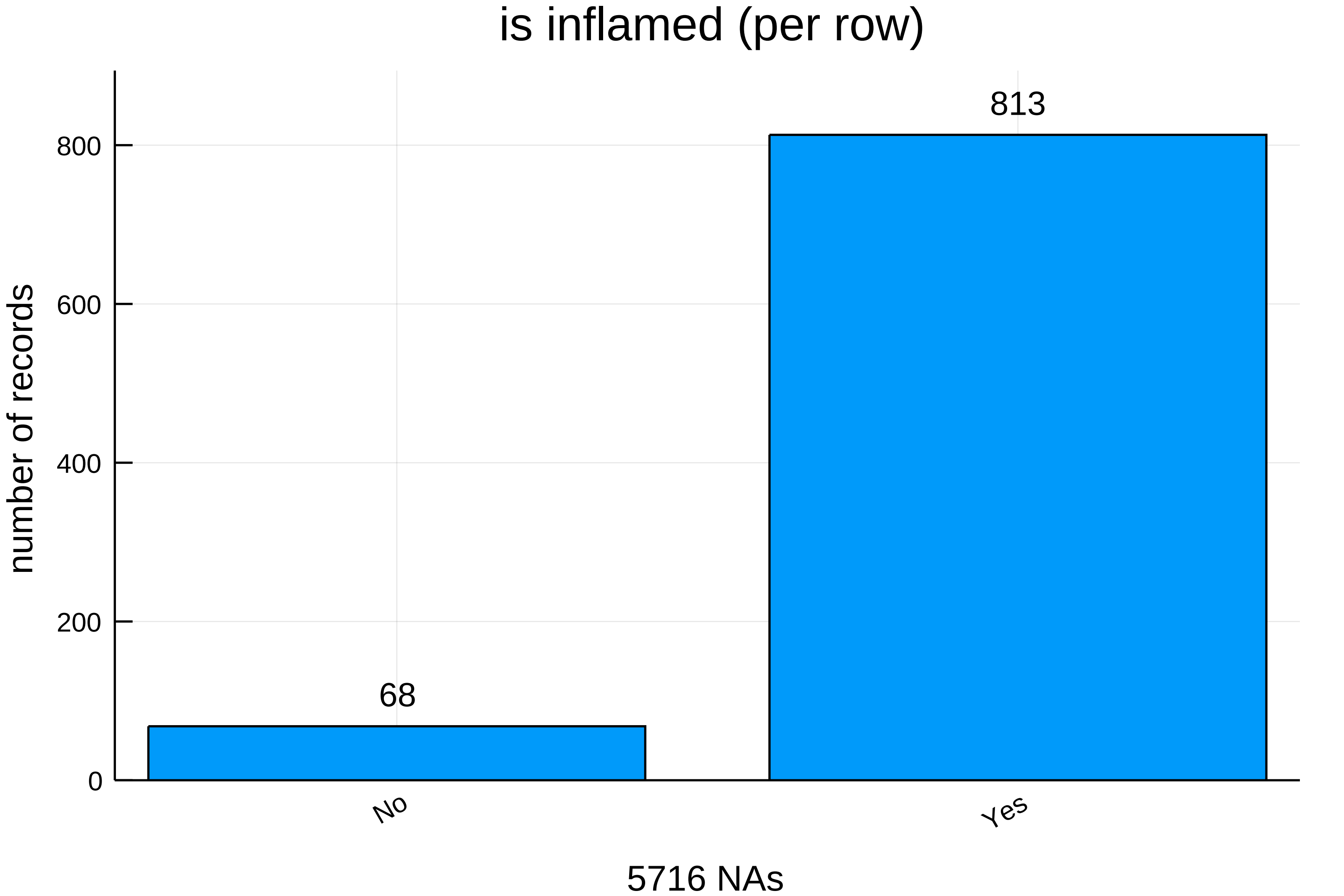
is inflamed (per row)
number of records
800
600
400
200
0
68
813
No
Yes
5716 NAs

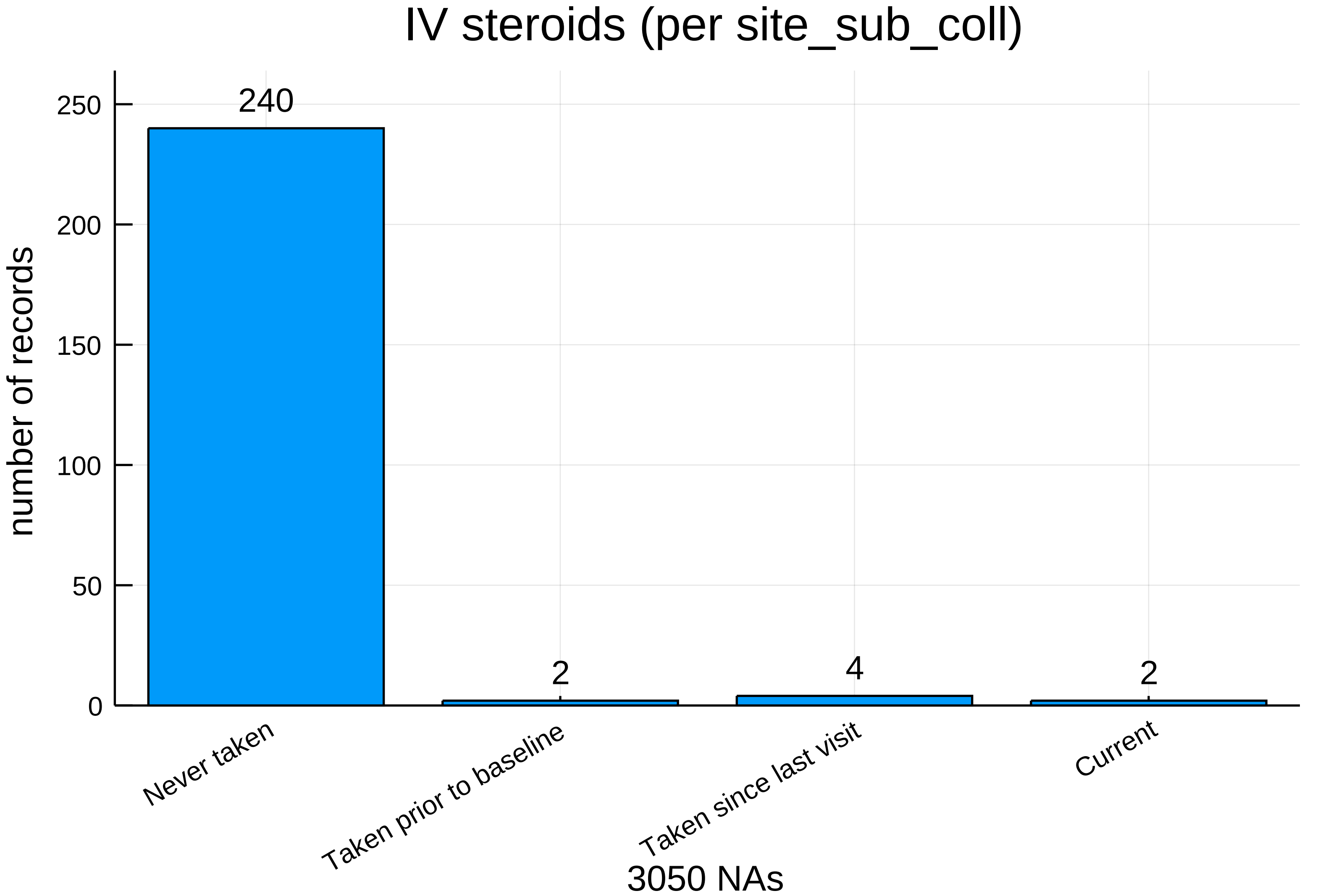
IV steroids (per site_sub_coll)
number of records
250
200
150
100
50
0
240
2
4
2
Never taken
Taken prior to baseline
Taken since last visit
Current
3050 NAs

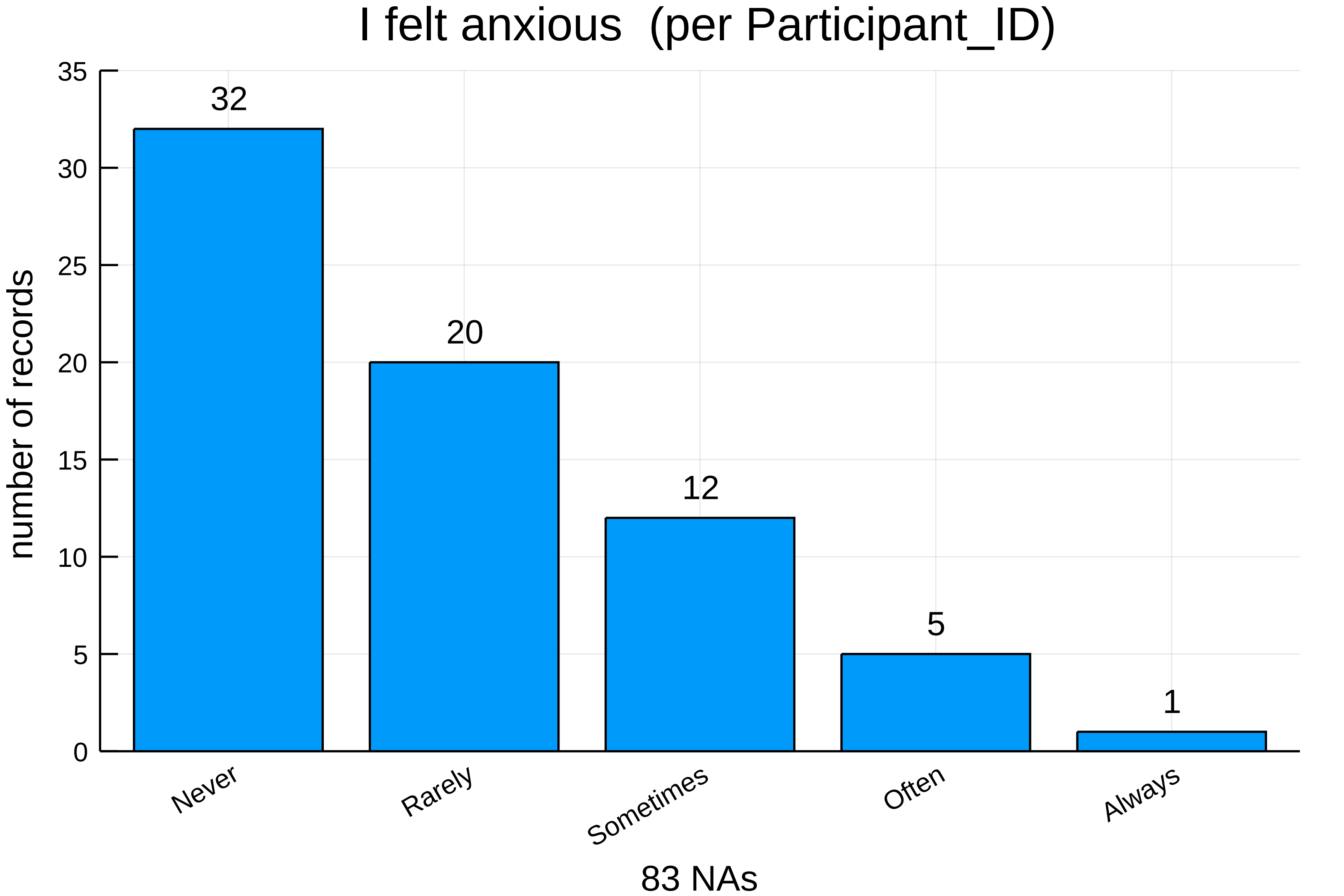
I felt anxious (per Participant_ID)
number of records
0
5
10
15
20
25
30
35
32
20
12
5
1
Never
Rarely
Sometimes
Often
Always
83 NAs

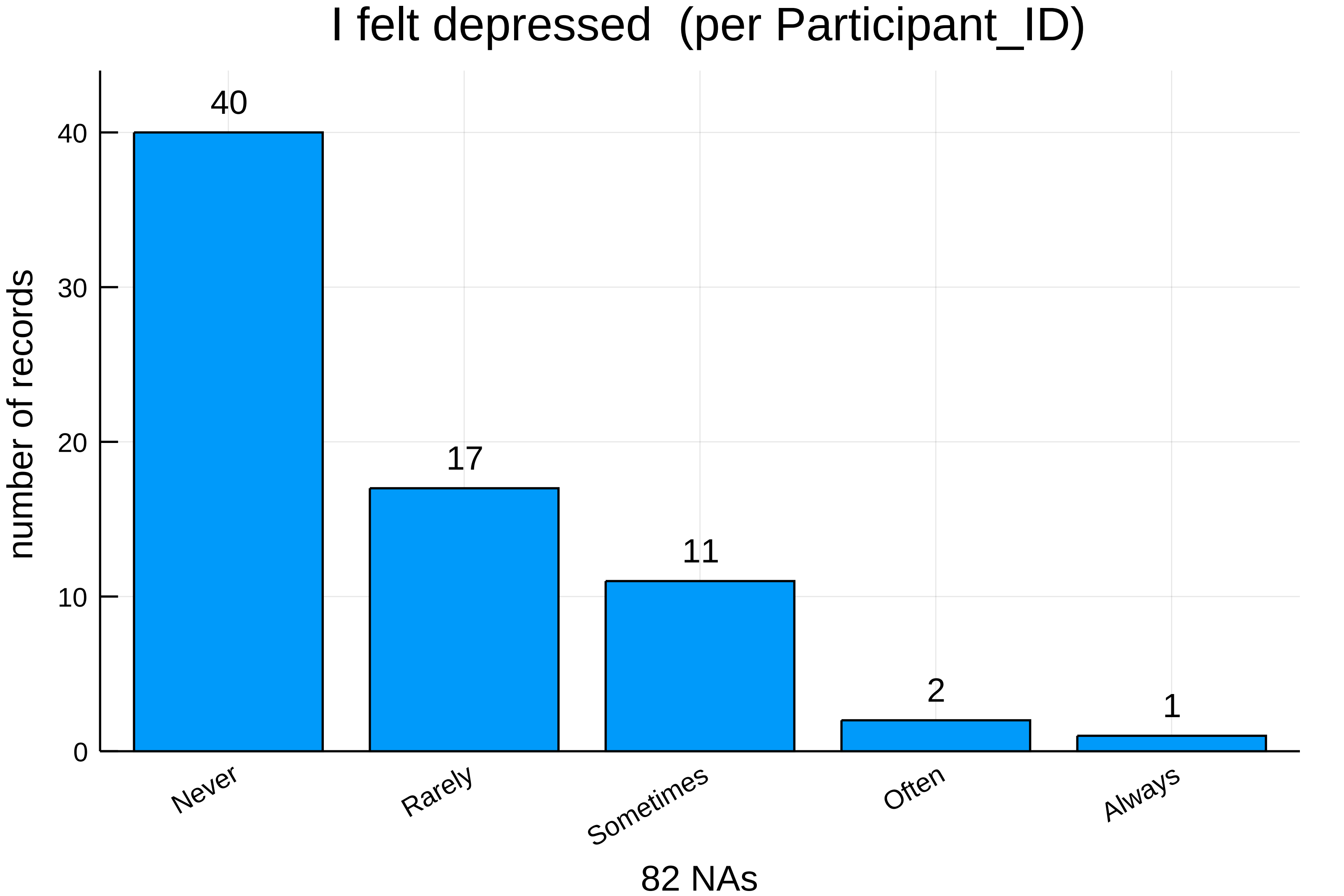
I felt depressed (per Participant_ID)
number of records
0
10
20
30
40
40
17
11
2
1
Never
Rarely
Sometimes
Often
Always
82 NAs

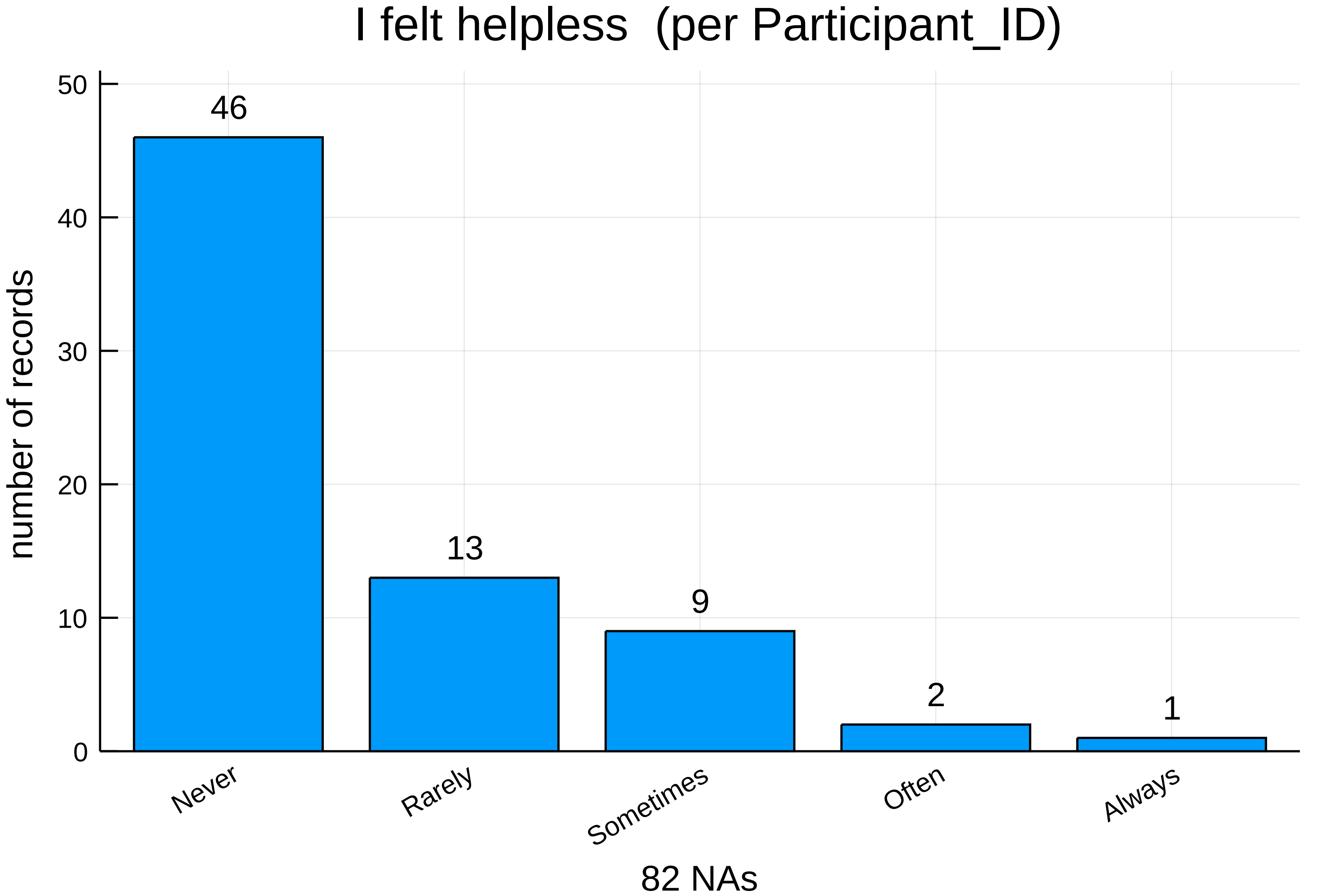
I felt helpless (per Participant_ID)
number of records
0
10
20
30
40
50
46
13
9
2
1
Never
Rarely
Sometimes
Often
Always
82 NAs

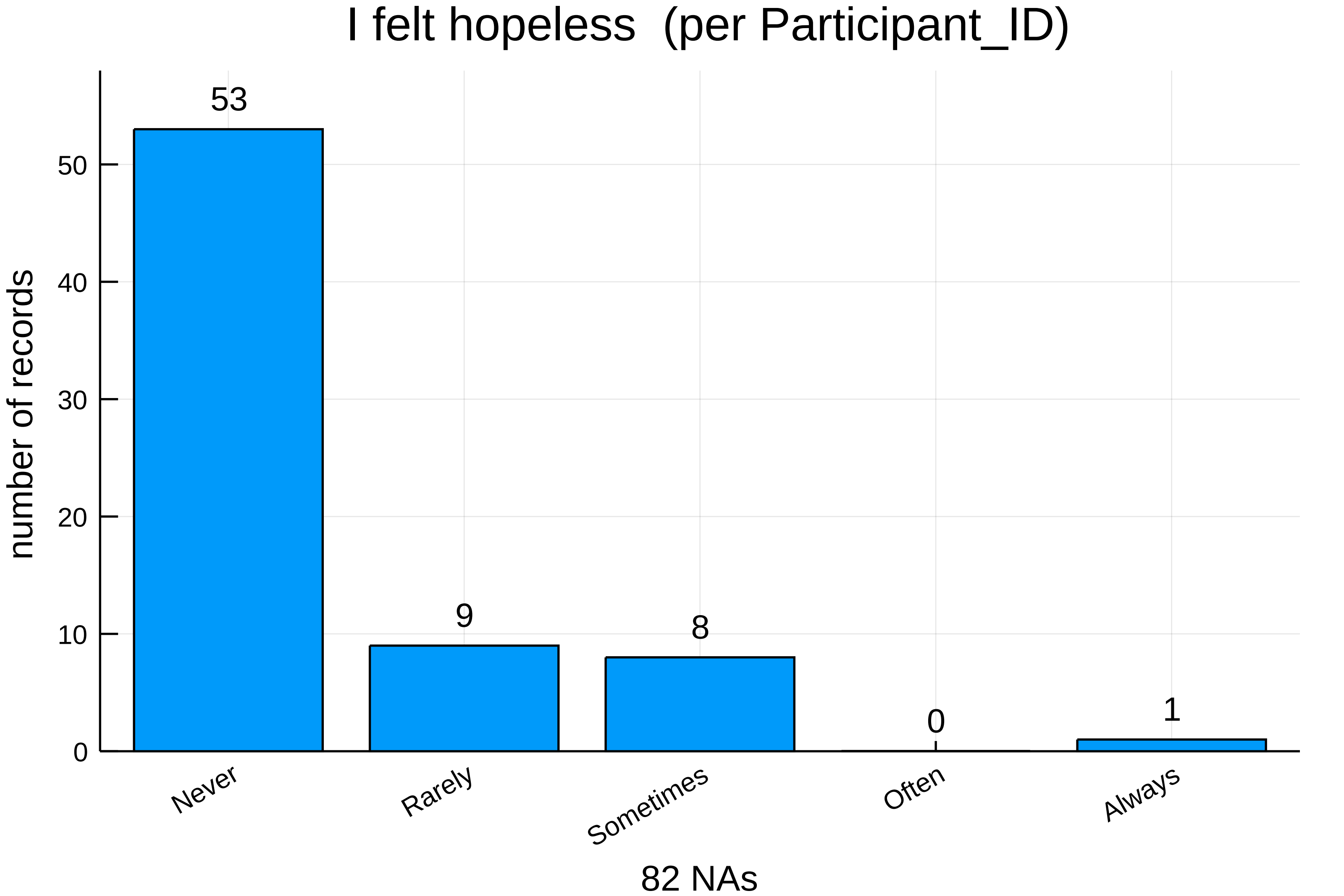
I felt hopeless (per Participant_ID)
number of records
0
10
20
30
40
50
53
9
8
0
1
Never
Rarely
Sometimes
Often
Always
82 NAs

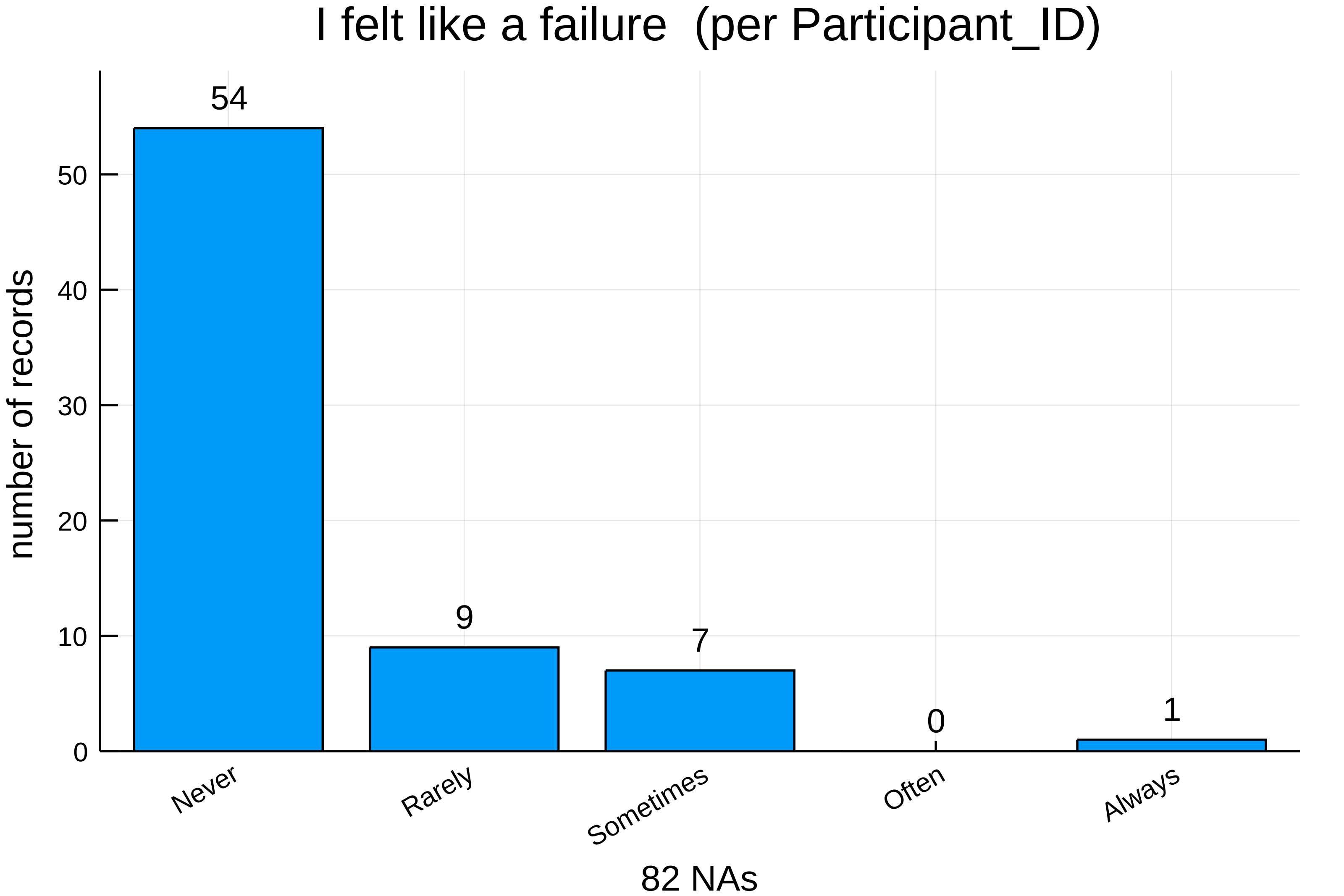
I felt like a failure (per Participant_ID)
number of records
0
10
20
30
40
50
54
9
7
0
1
Never
Rarely
Sometimes
Often
Always
82 NAs

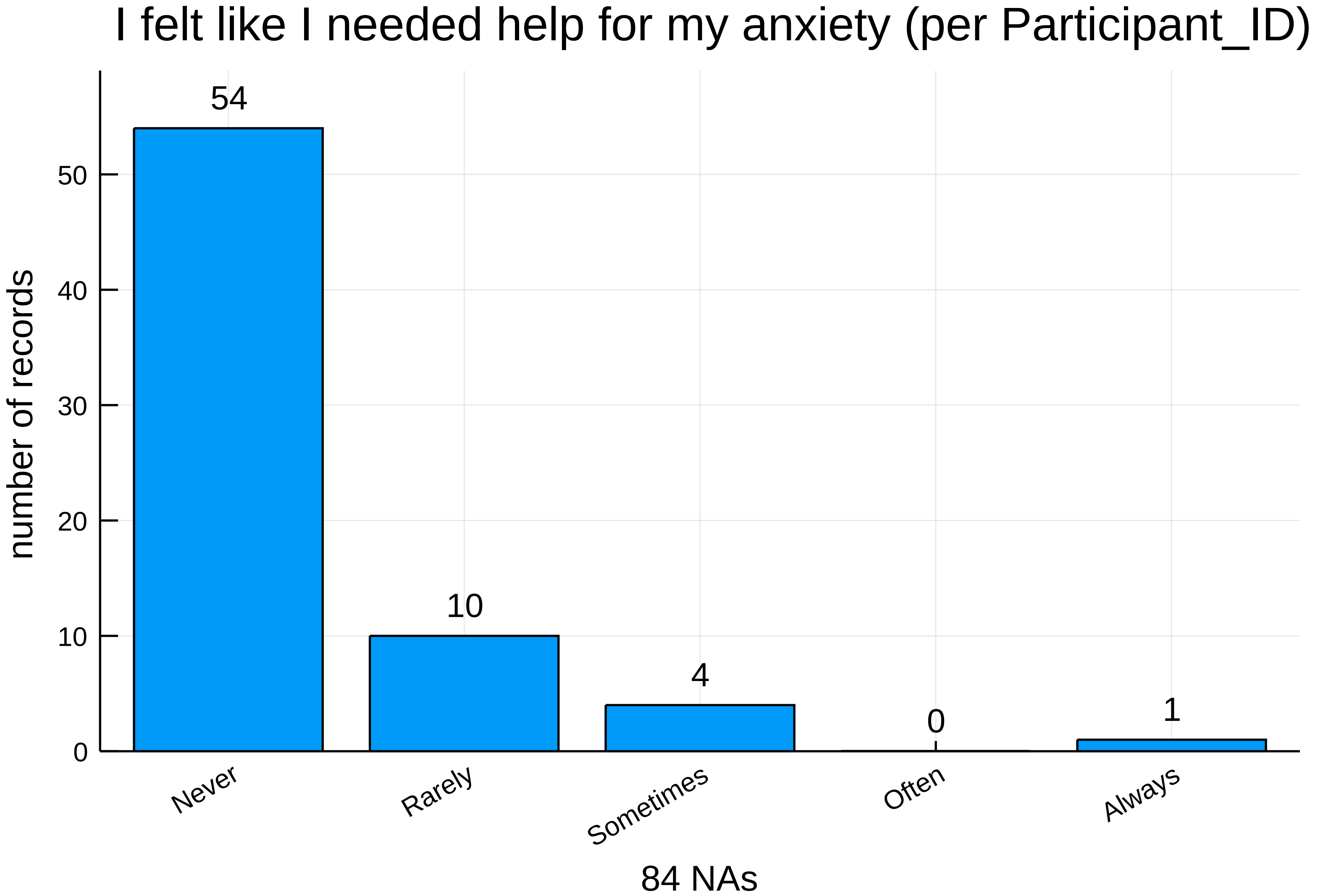
I felt like I needed help for my anxiety (per Participant_ID)
number of records
0
10
20
30
40
50
54
10
4
0
1
Never
Rarely
Sometimes
Often
Always
84 NAs

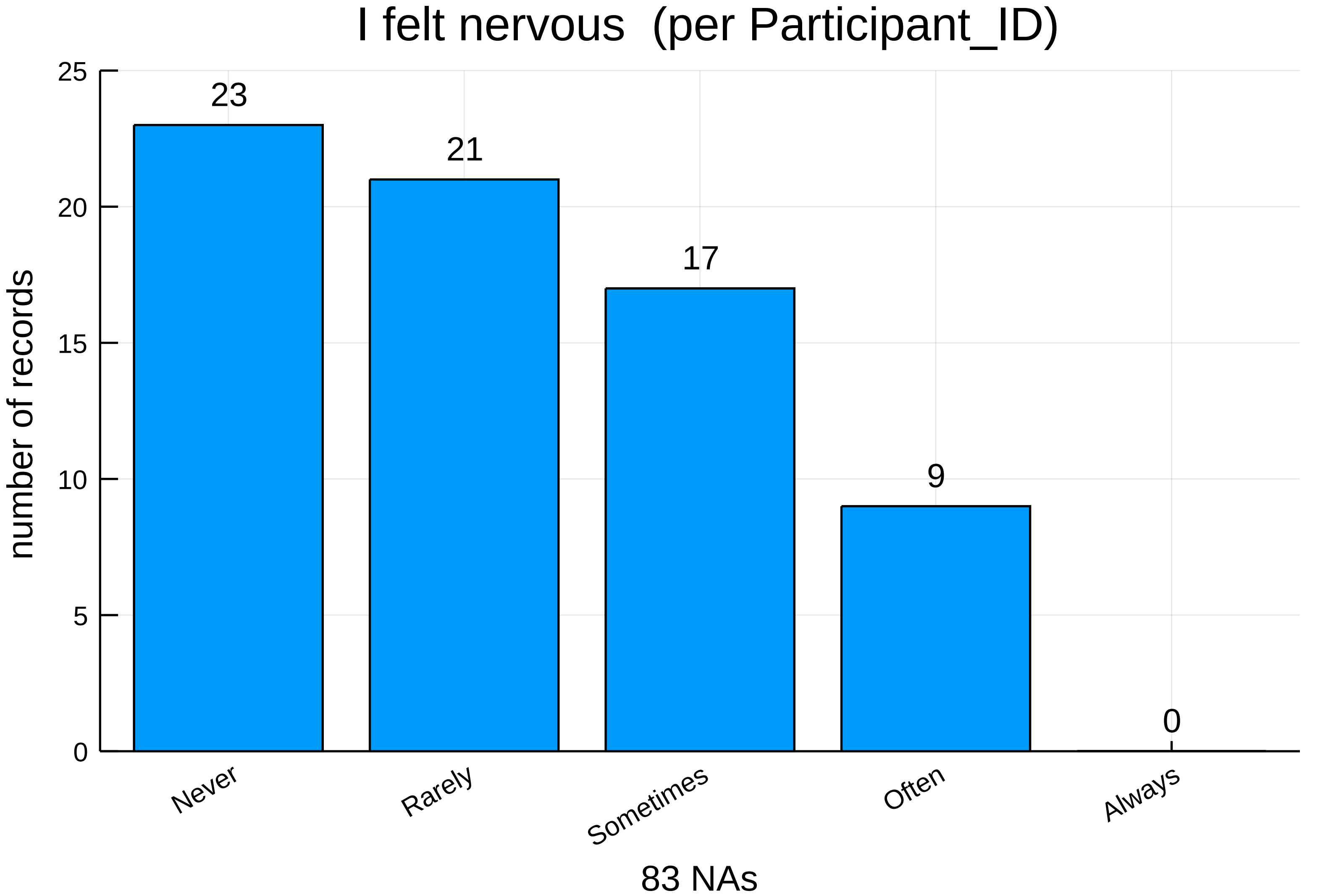
I felt nervous (per Participant_ID)
number of records
25
20
15
10
5
0
23
21
17
9
0
Never
Rarely
Sometimes
Often
Always
83 NAs

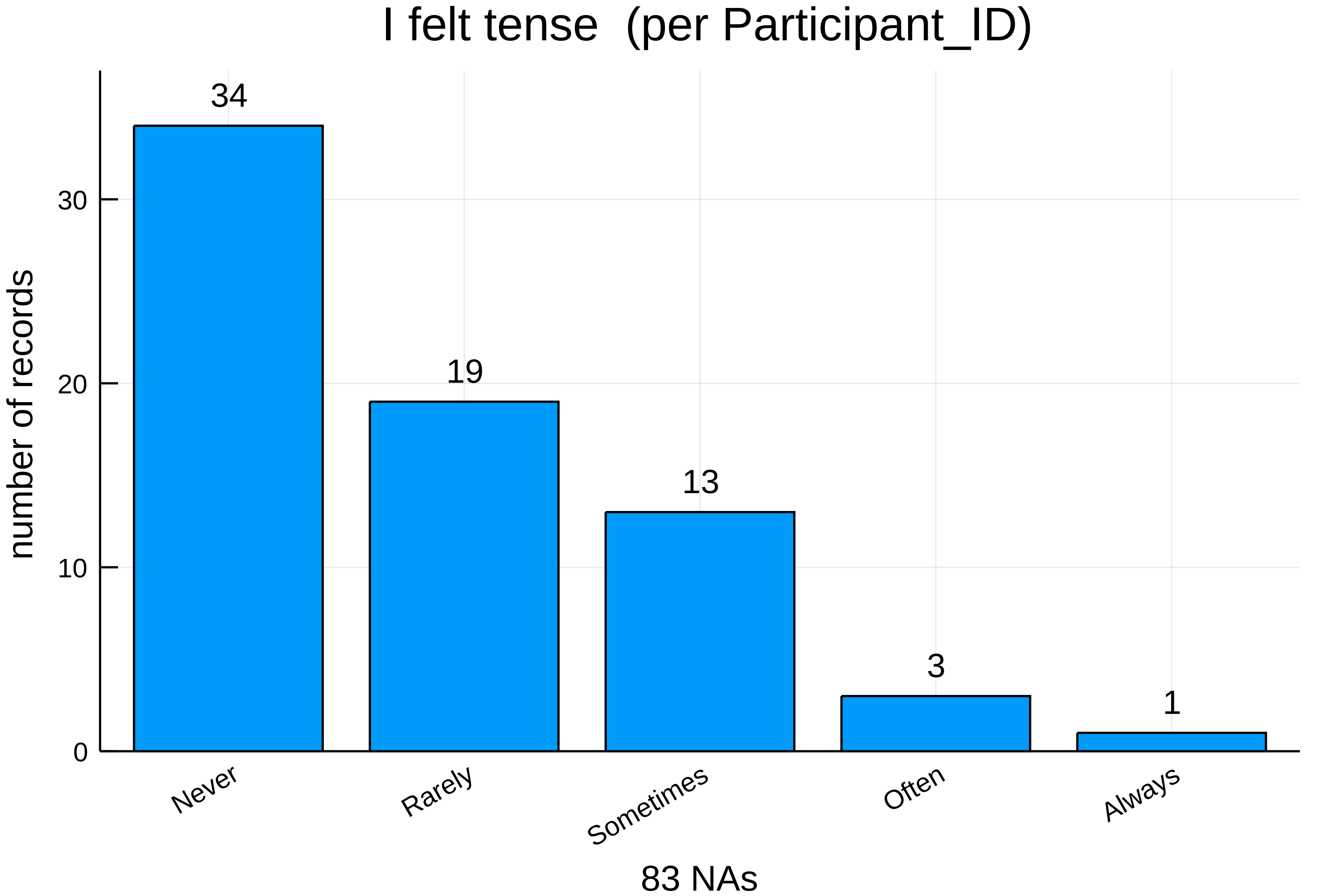
I felt tense (per Participant_ID)
number of records
0
10
20
30
34
19
13
3
1
Never
Rarely
Sometimes
Often
Always
83 NAs

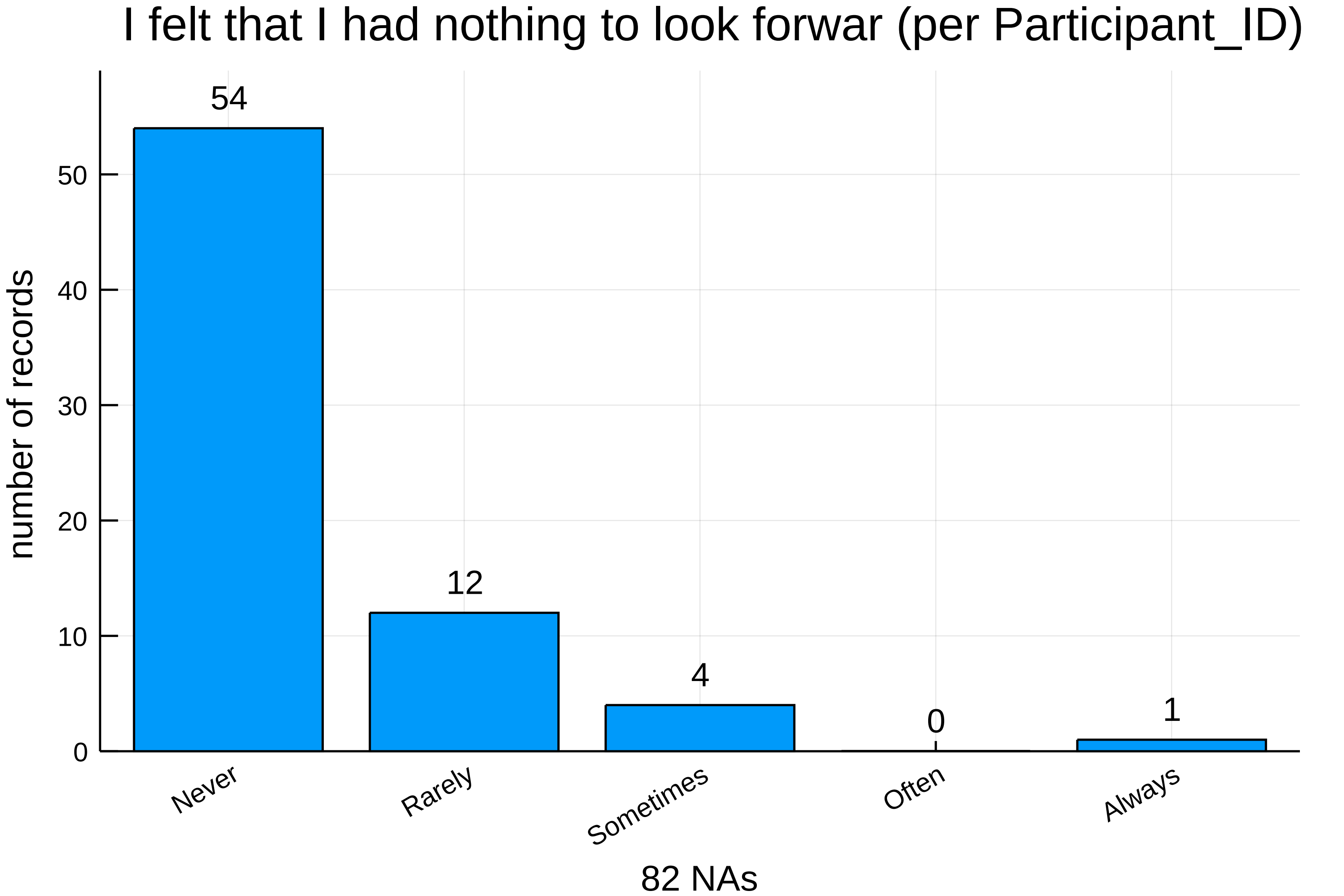
I felt that I had nothing to look forwar (per Participant_ID)
number of records
54
12
4
0
1
0
10
20
30
40
50
Never
Rarely
Sometimes
Often
Always
82 NAs

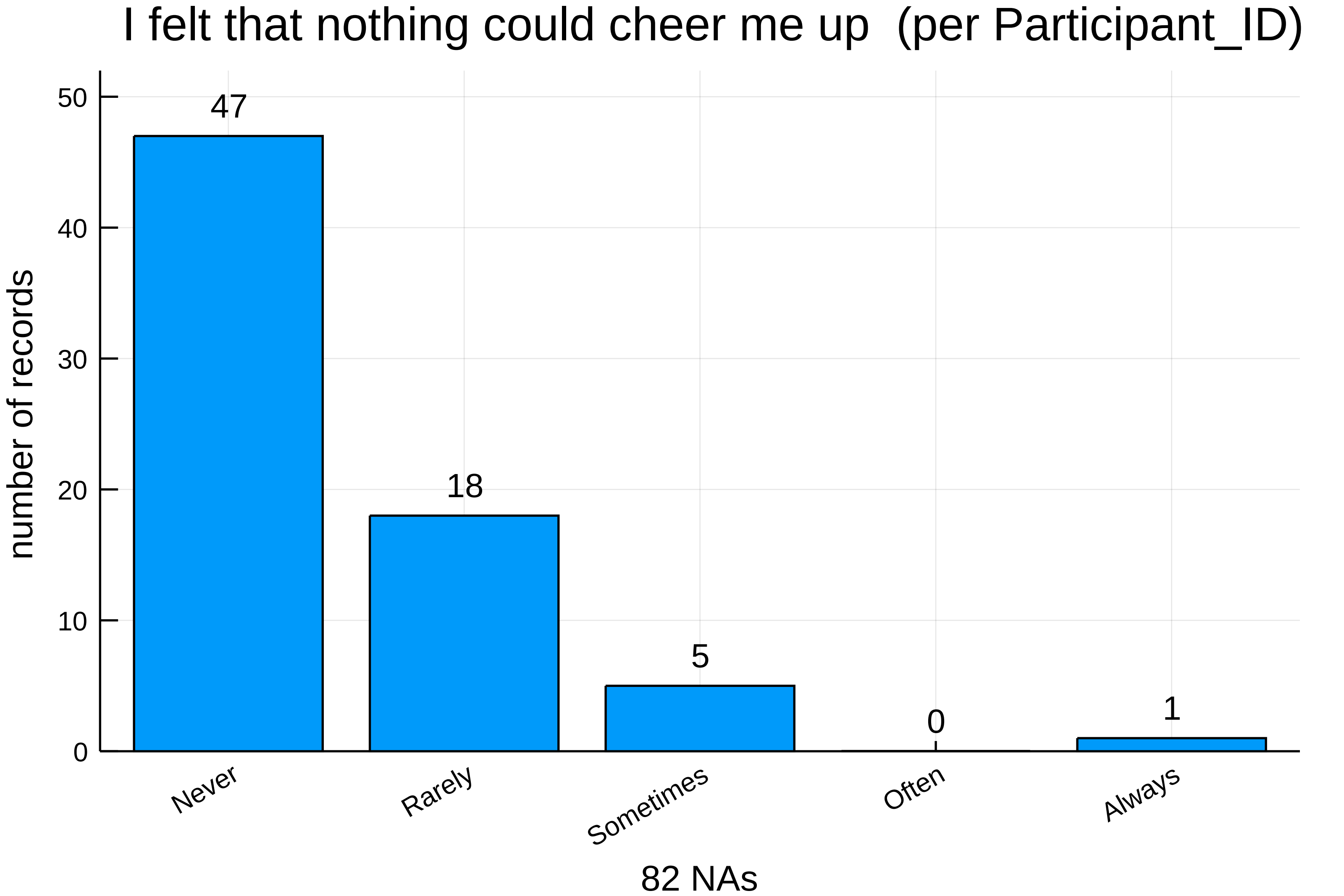
I felt that nothing could cheer me up (per Participant_ID)
number of records
50
40
30
20
10
0
47
18
5
0
1
Never
Rarely
Sometimes
Often
Always
82 NAs

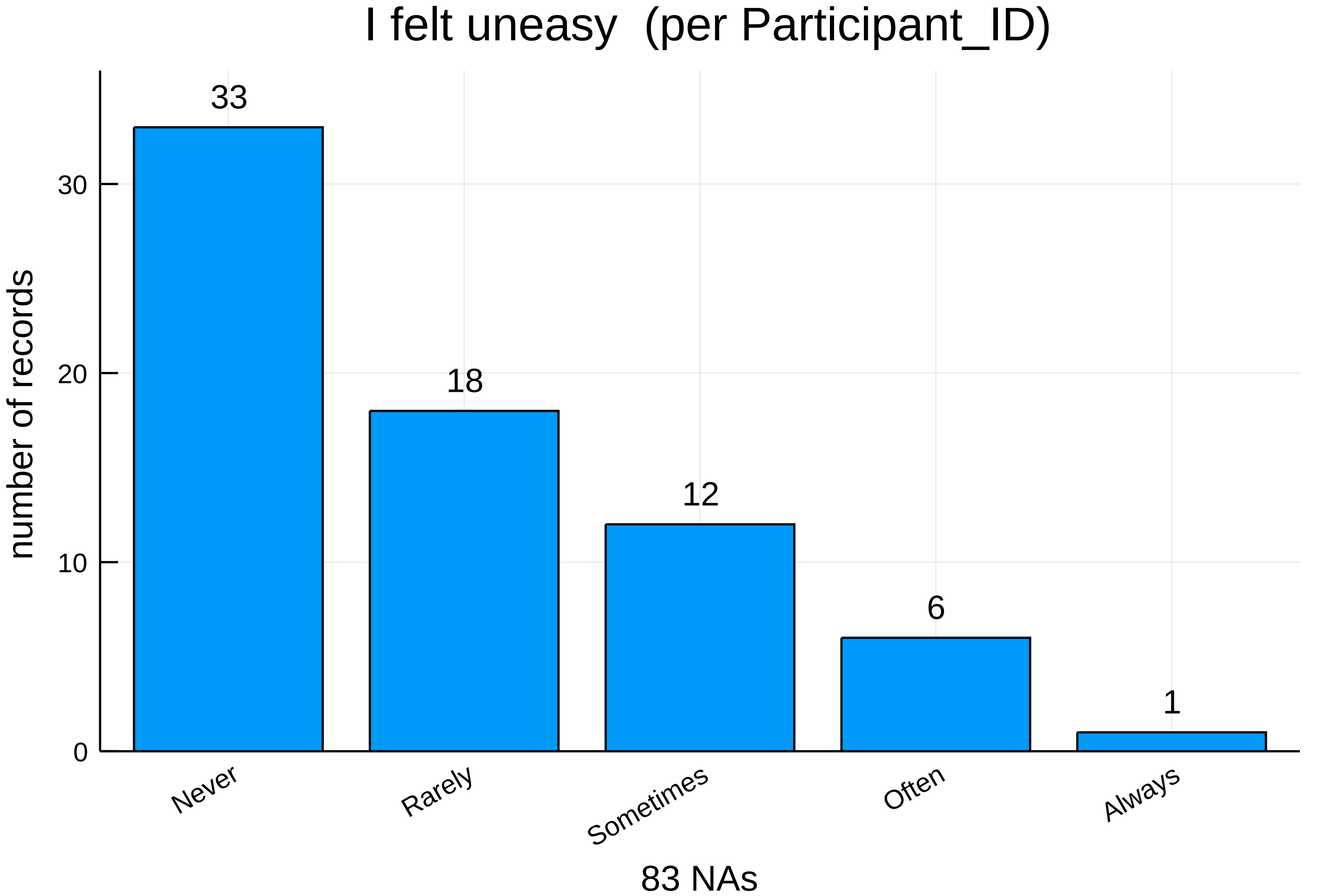
I felt uneasy (per Participant_ID)
number of records
33
18
12
6
1
0
10
20
30
Never
Rarely
Sometimes
Often
Always
83 NAs

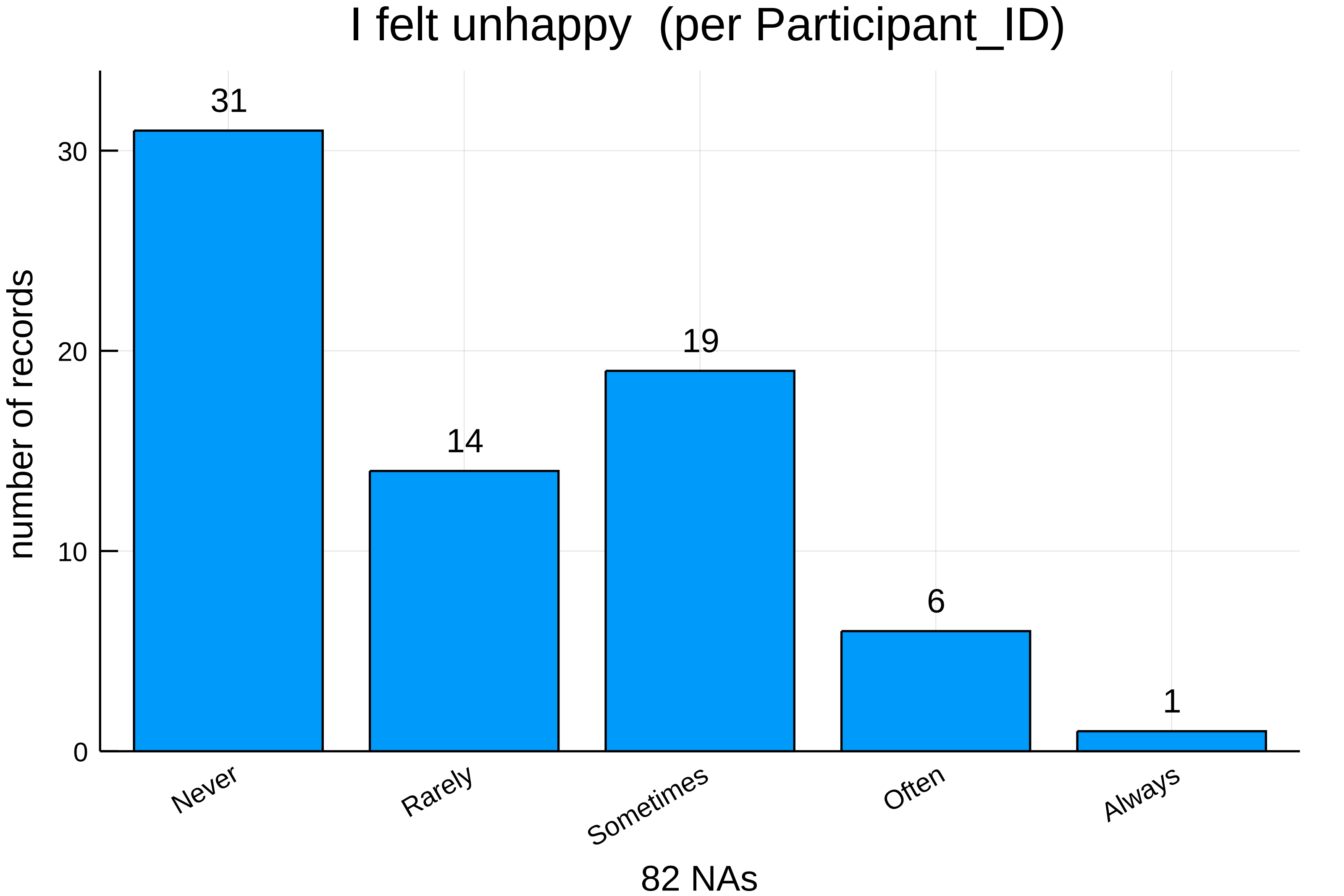
I felt unhappy (per Participant_ID)
number of records
0
10
20
30
31
14
19
6
1
Never
Rarely
Sometimes
Often
Always
82 NAs

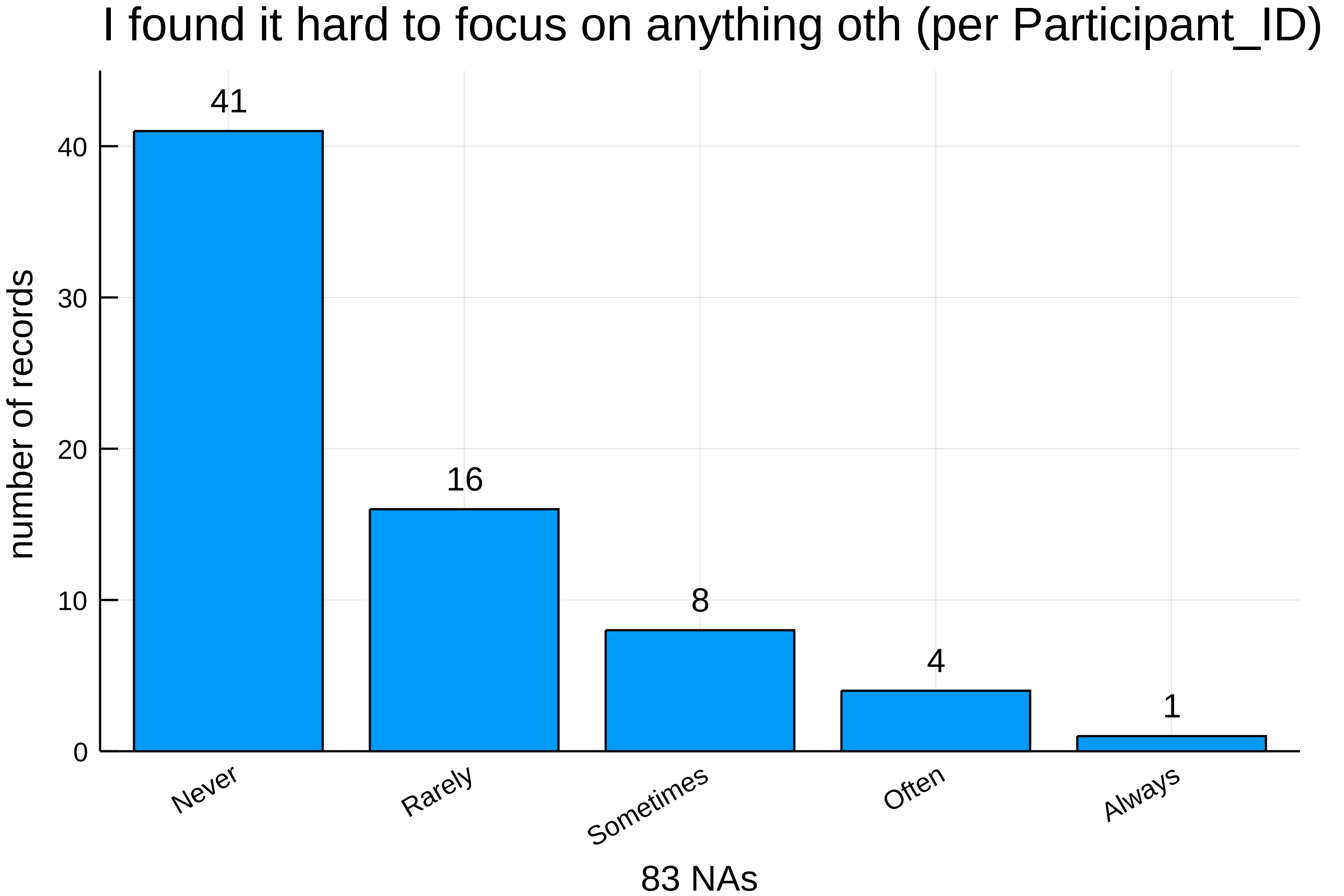
I found it hard to focus on anything oth (per Participant_ID)
number of records
0
10
20
30
40
41
16
8
4
1
Never
Rarely
Sometimes
Often
Always
83 NAs

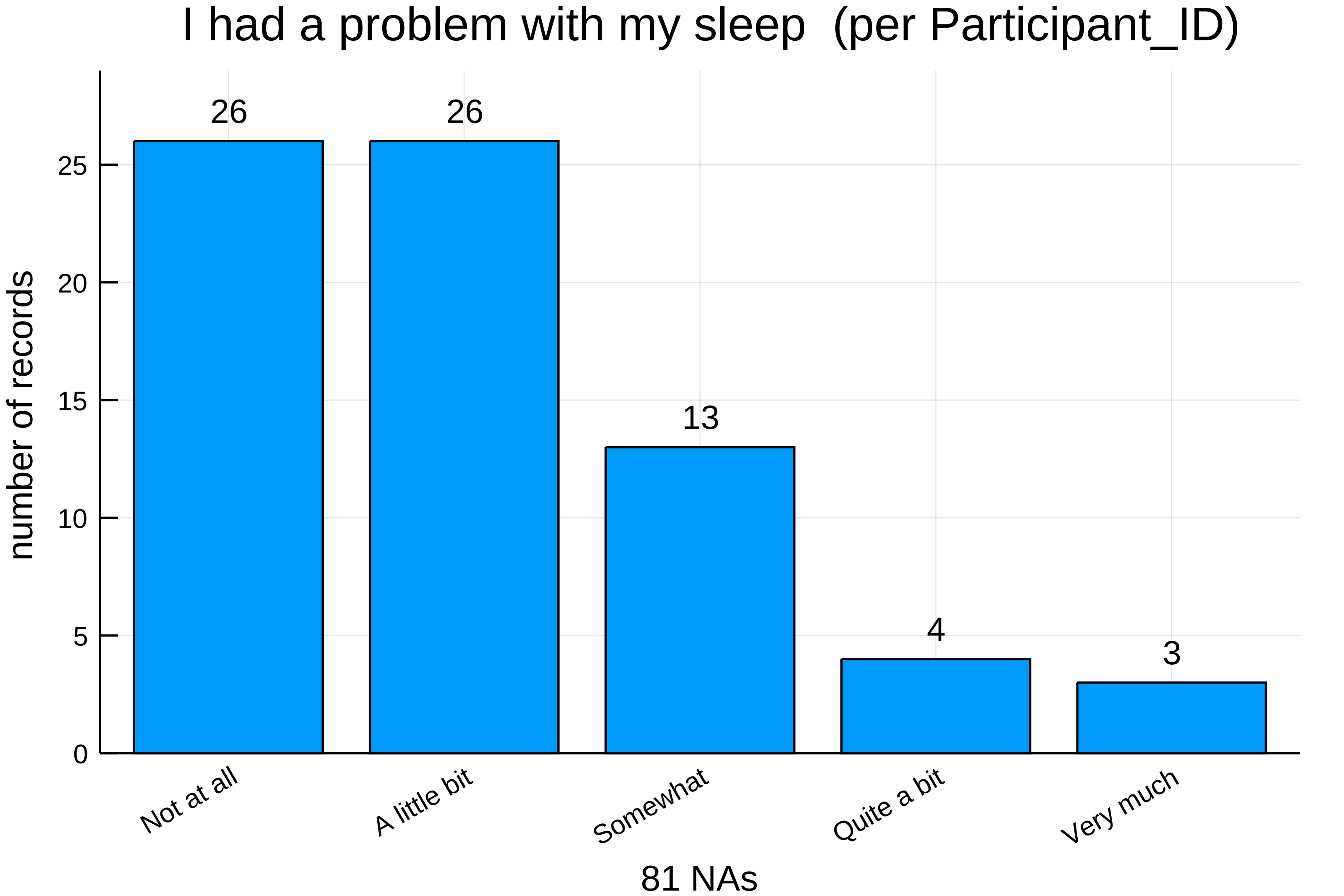
I had a problem with my sleep (per Participant_ID)
number of records
0
5
10
15
20
25
26
26
13
4
3
Not at all
A little bit
Somewhat
Quite a bit
Very much
81 NAs

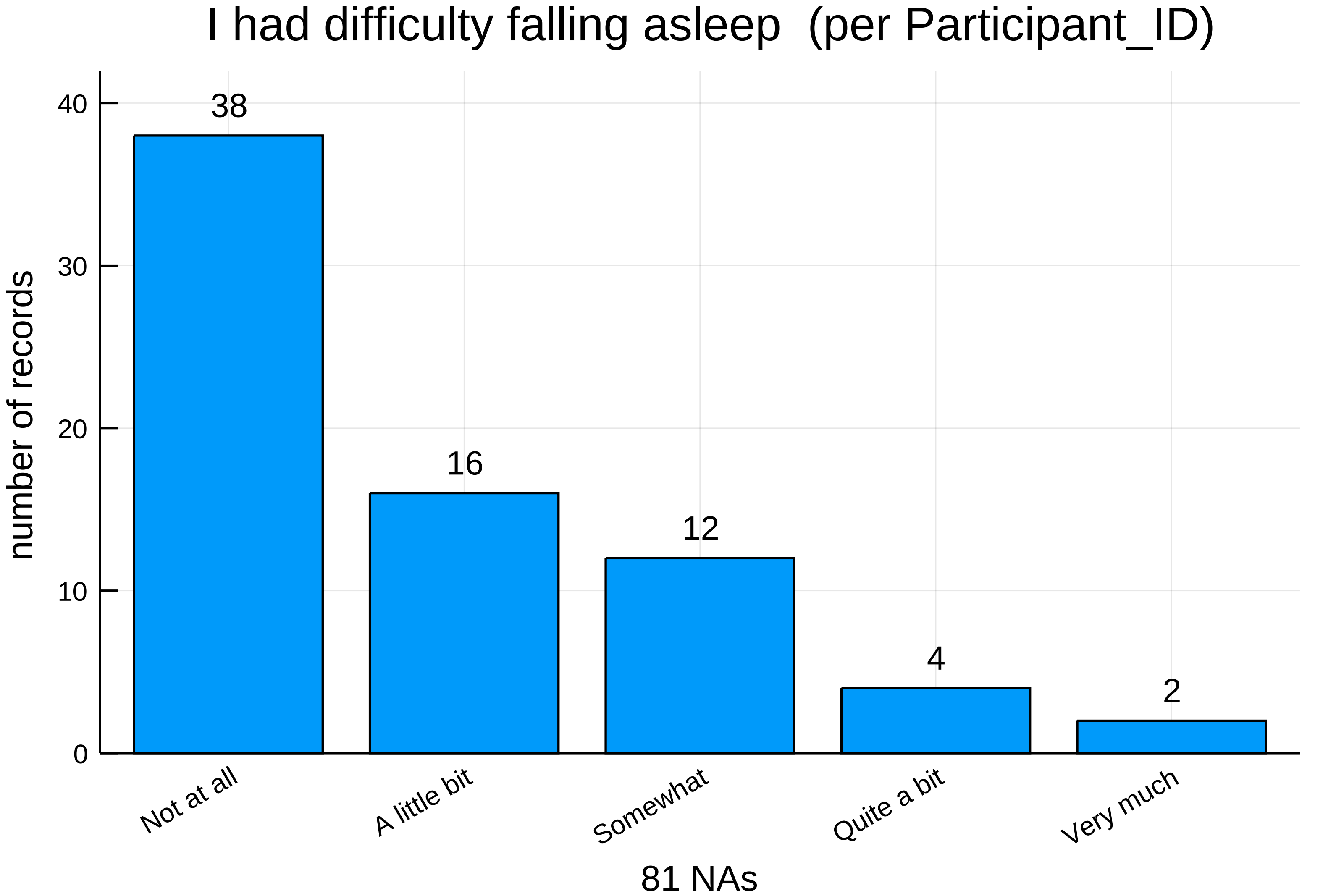

I had difficulty falling asleep (per Participant_ID)
number of records
0
10
20
30
40
38
16
12
4
2
Not at all
A little bit
Somewhat
Quite a bit
Very much
81 NAs

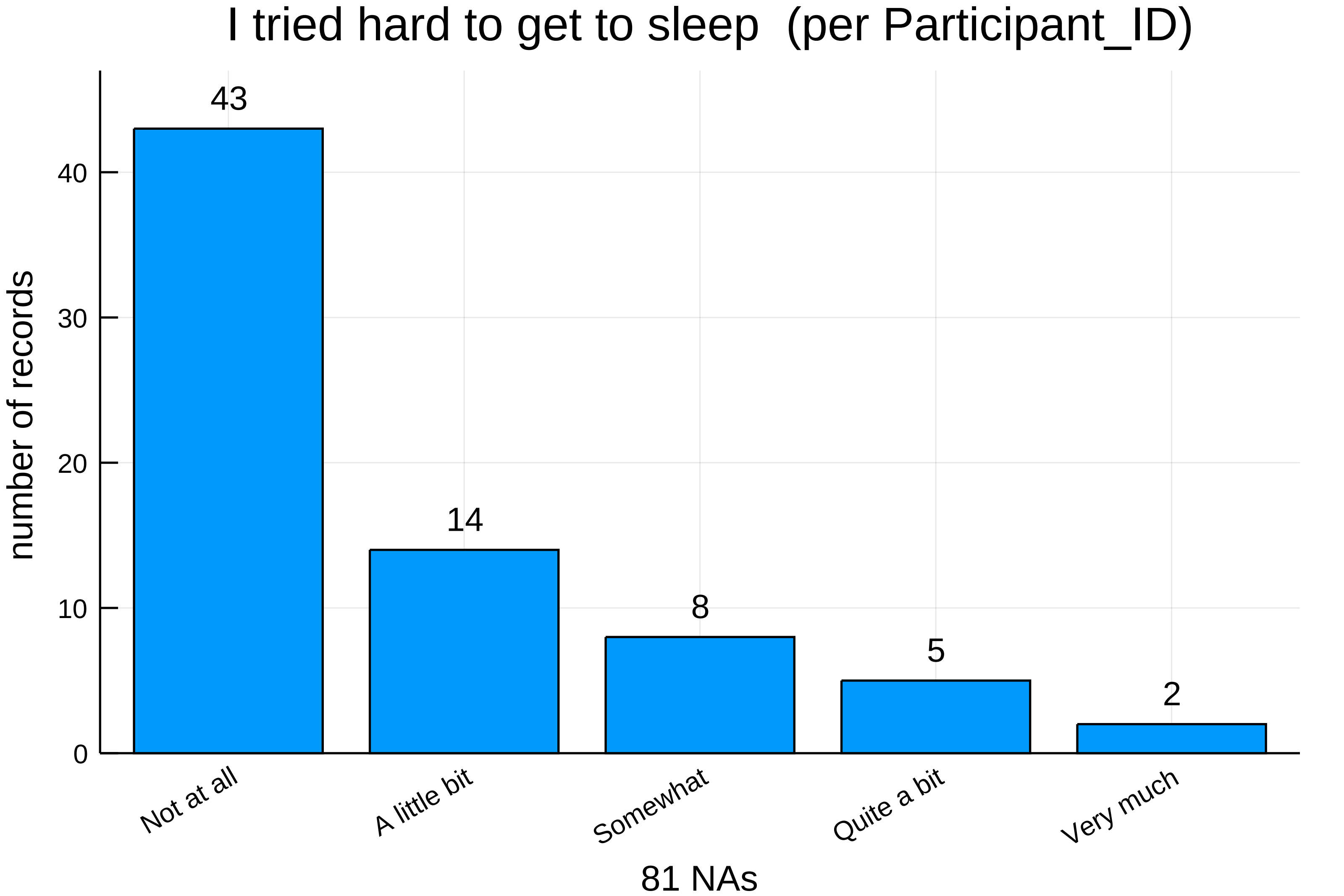

I tried hard to get to sleep (per Participant_ID)
number of records
0
10
20
30
40
43
14
8
5
2
Not at all
A little bit
Somewhat
Quite a bit
Very much
81 NAs

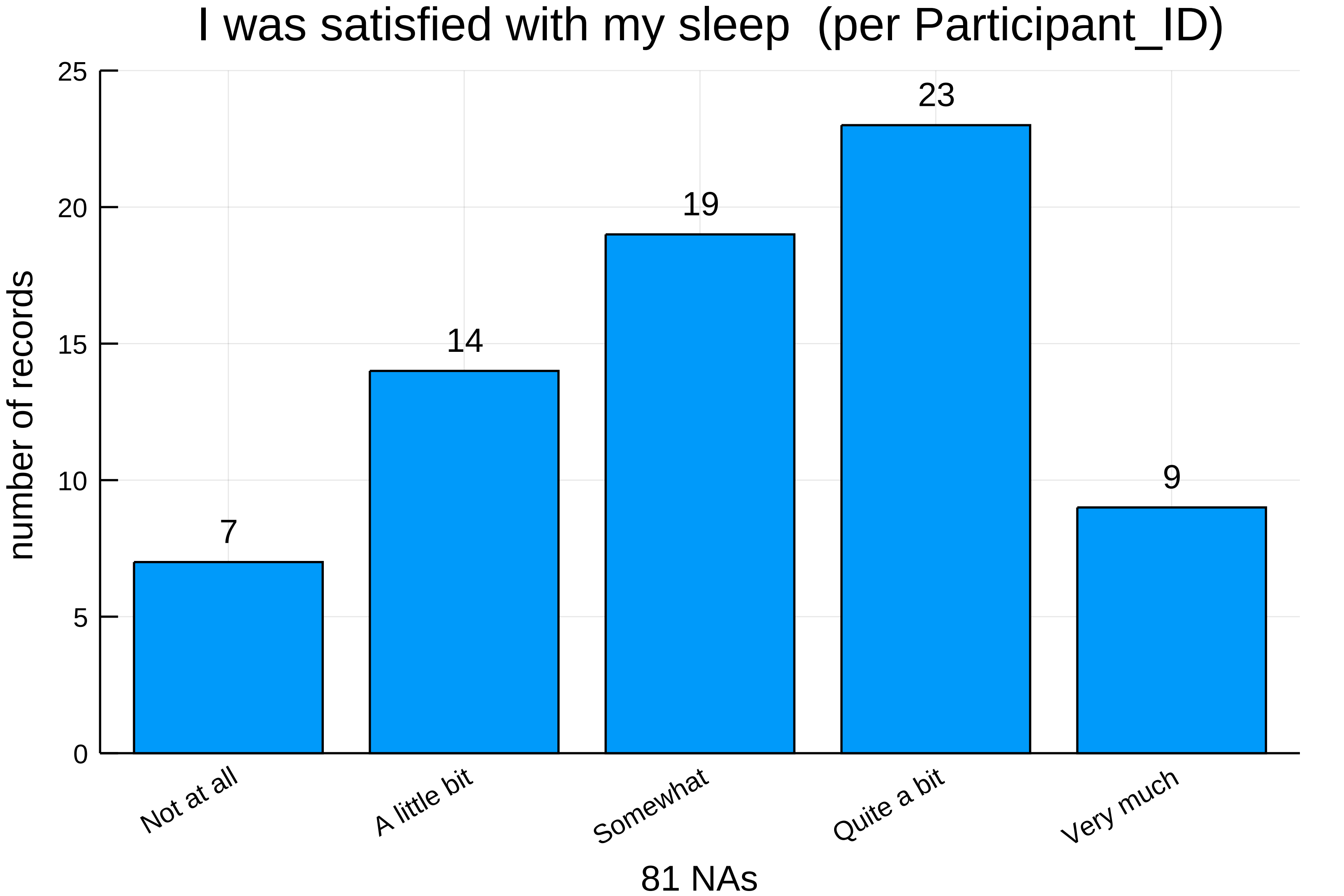
I was satisfied with my sleep (per Participant_ID)
number of records
0
5
10
15
20
25
7
14
19
23
9
Not at all
A little bit
Somewhat
Quite a bit
Very much
81 NAs

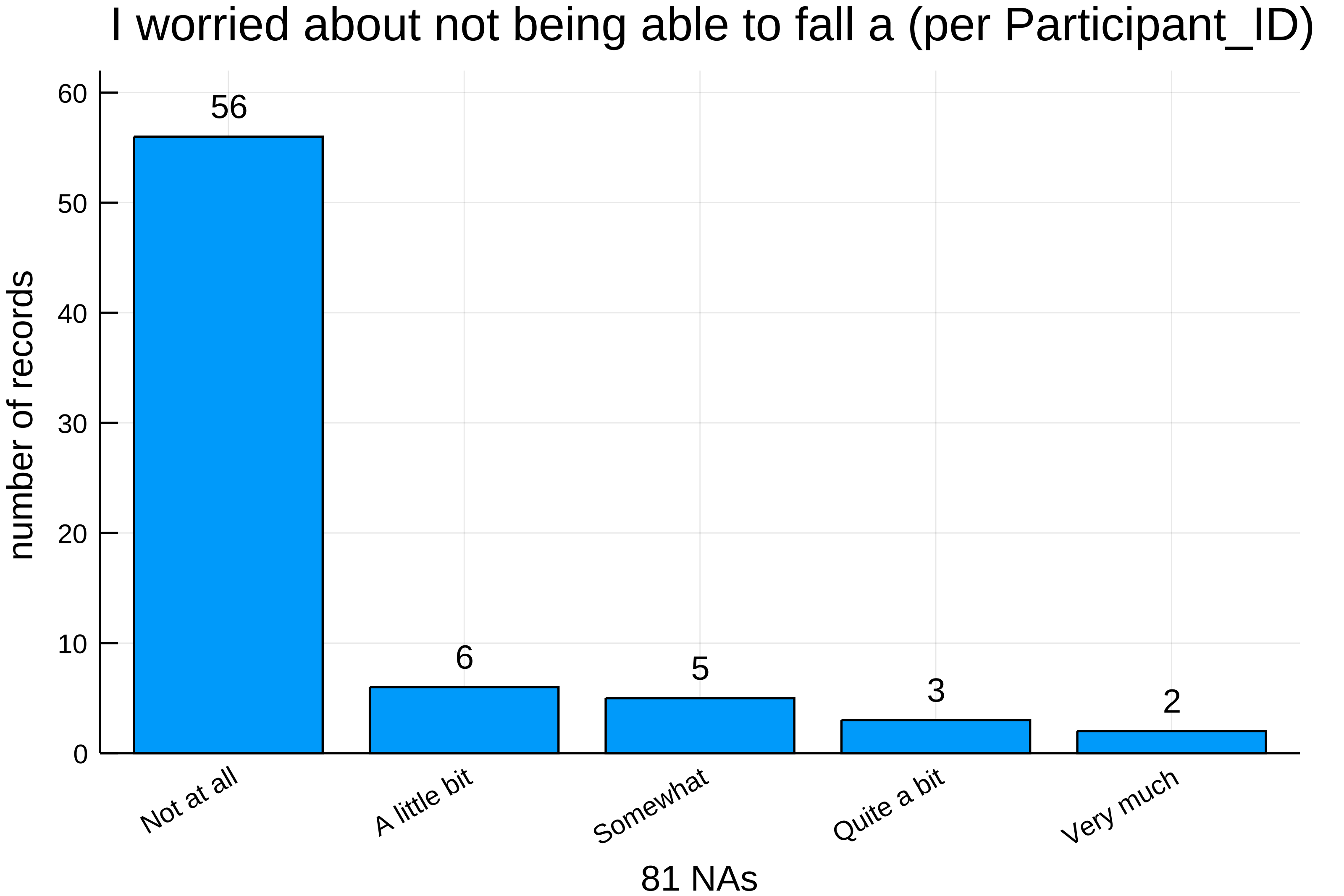
I worried about not being able to fall a (per Participant_ID)
number of records
0
10
20
30
40
50
60
56
6
5
3
2
Not at all
A little bit
Somewhat
Quite a bit
Very much
81 NAs

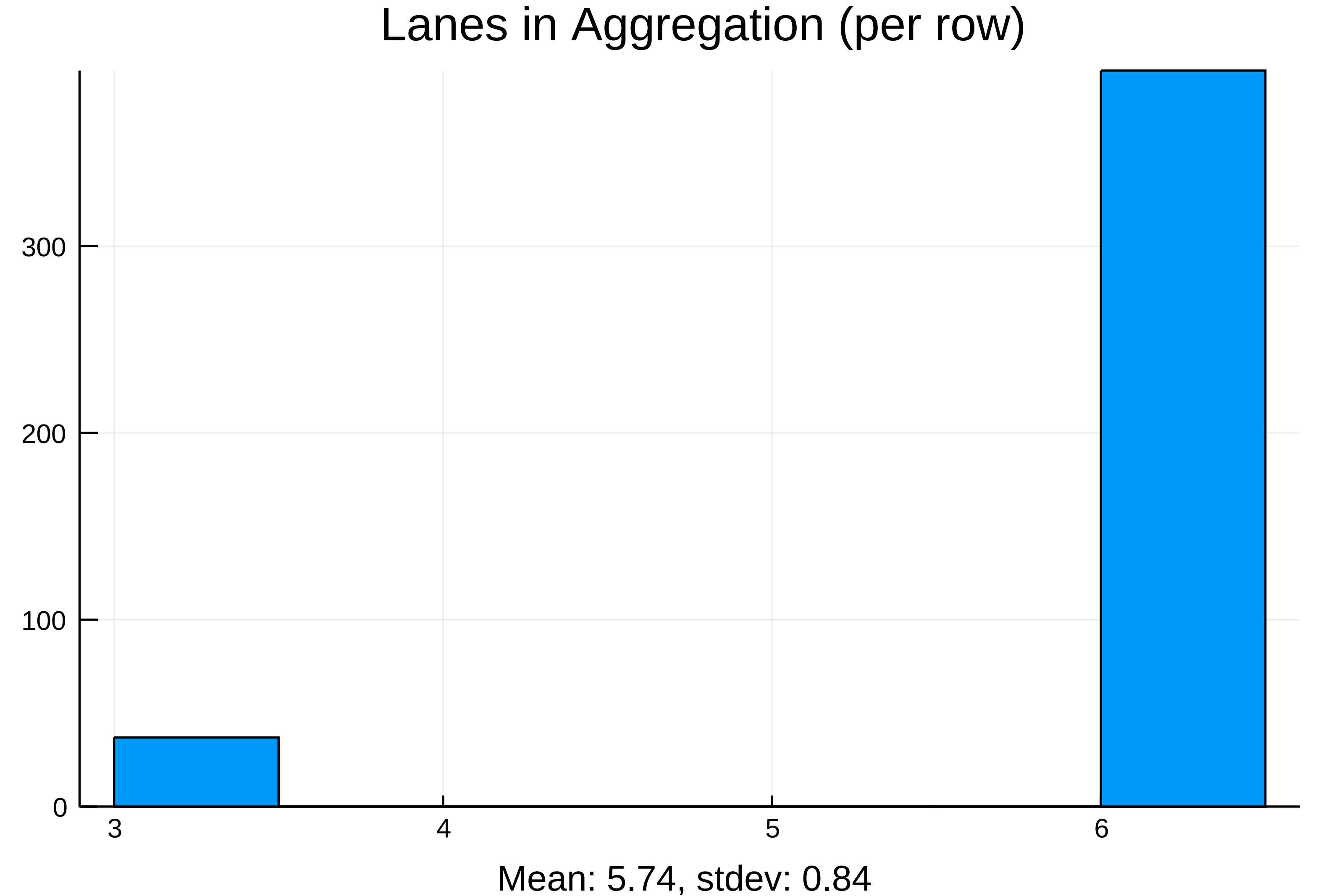
Lanes in Aggregation (per row)
300
200
100
0
3
4
5
6
Mean: 5.74, stdev: 0.84

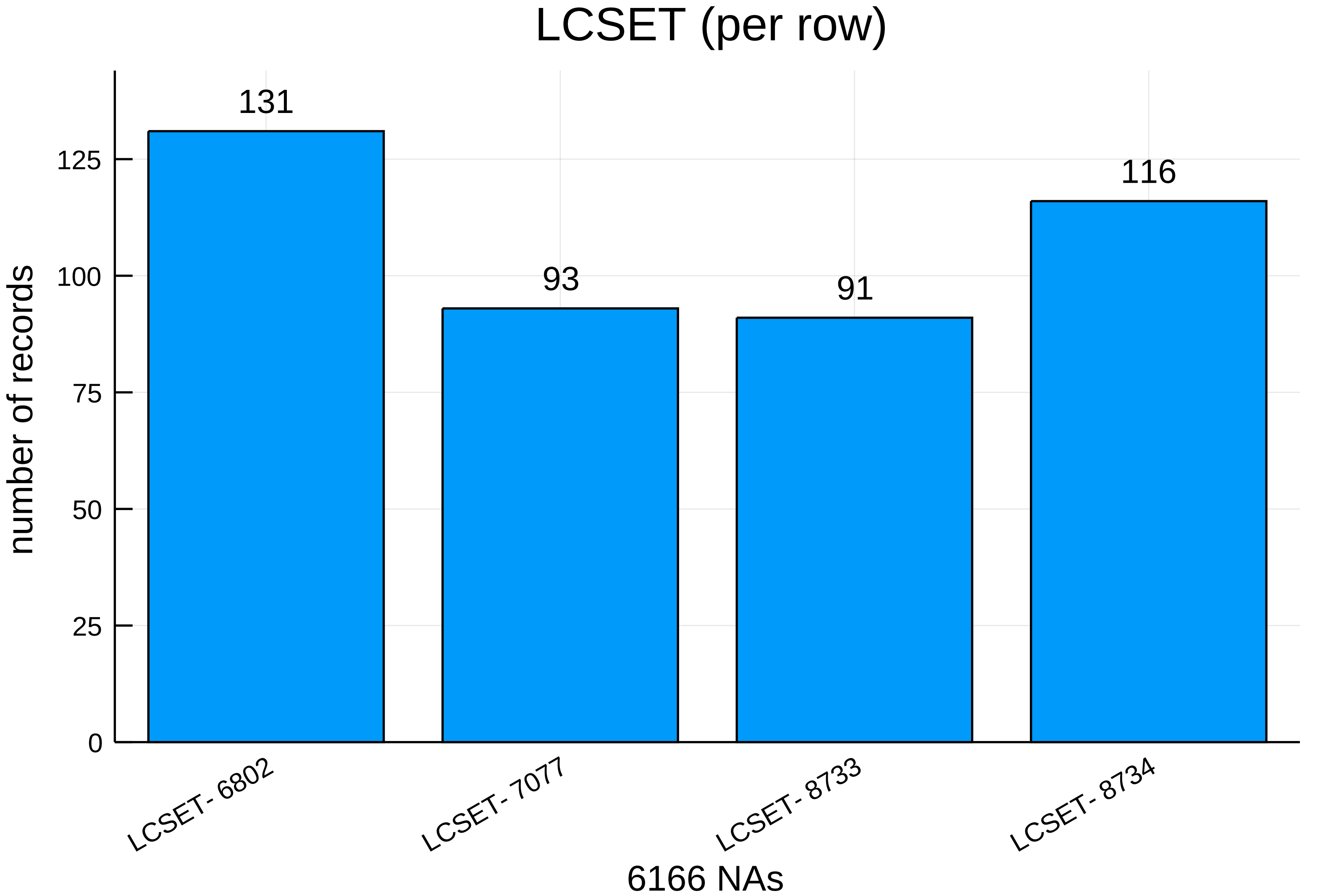
LCSET (per row)
number of records
0
25
50
75
100
125
131
93
91
116
LCSET- 6802
LCSET- 7077
LCSET- 8733
LCSET- 8734
6166 NAs

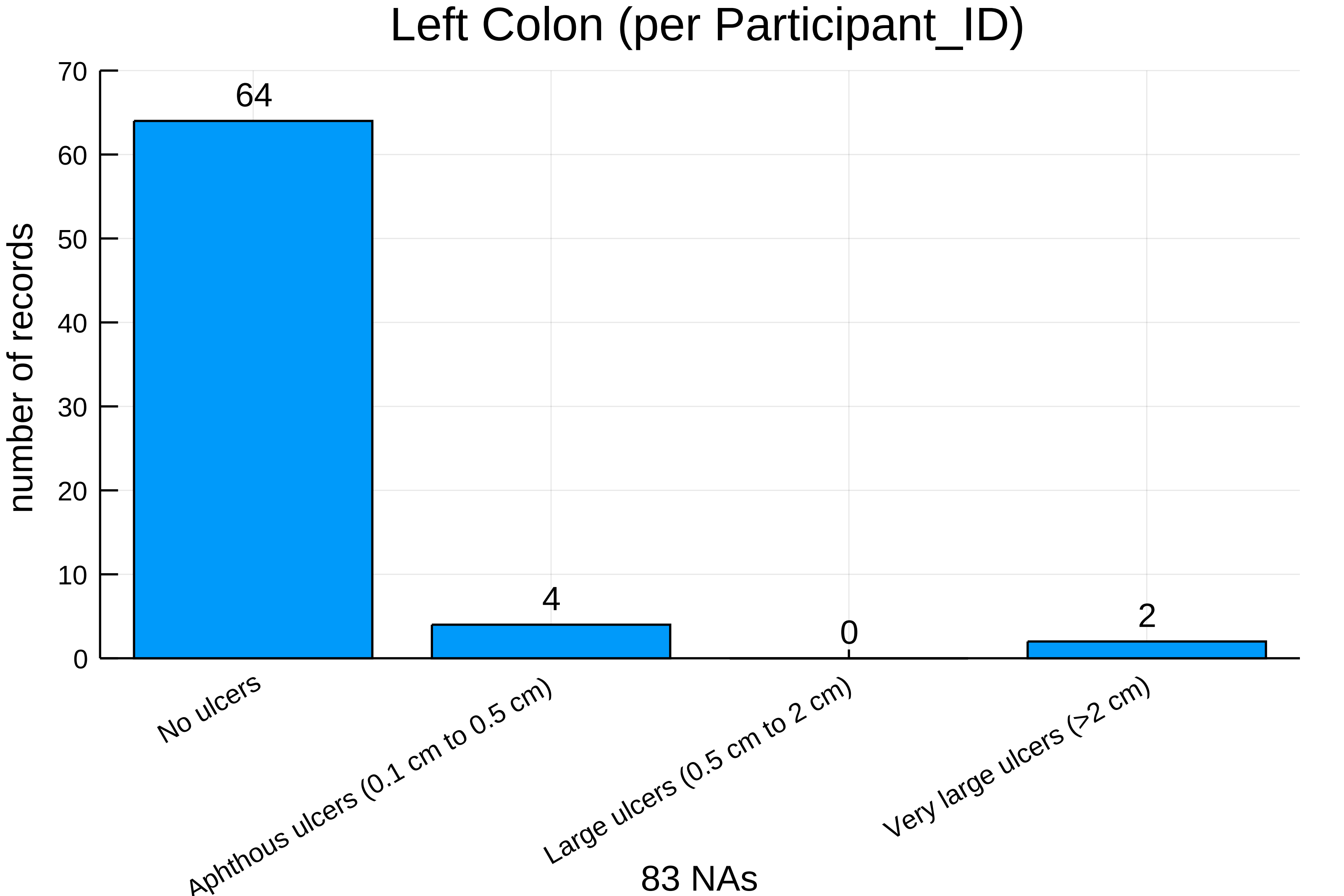
Left Colon (per Participant_ID)
number of records
70
60
50
40
30
20
10
0
64
4
0
2
No ulcers
Aphthous ulcers (0.1 cm to 0.5 cm)
Large ulcers (0.5 cm to 2 cm)
Very large ulcers (>2 cm)
83 NAs

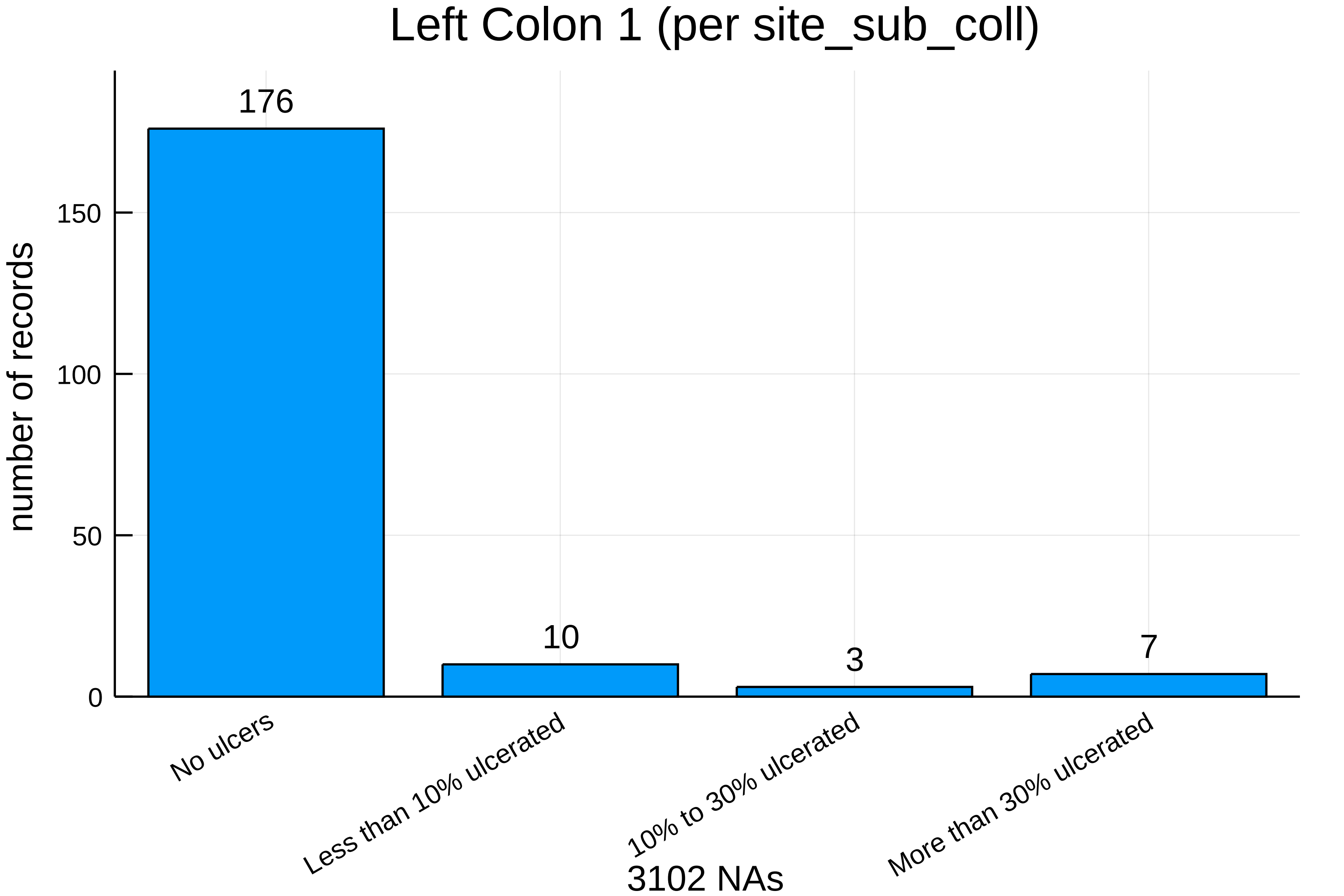

Left Colon 1 (per site_sub_coll)
number of records
176
10
3
7
0
50
100
150
No ulcers
Less than 10% ulcerated
10% to 30% ulcerated
More than 30% ulcerated
3102 NAs

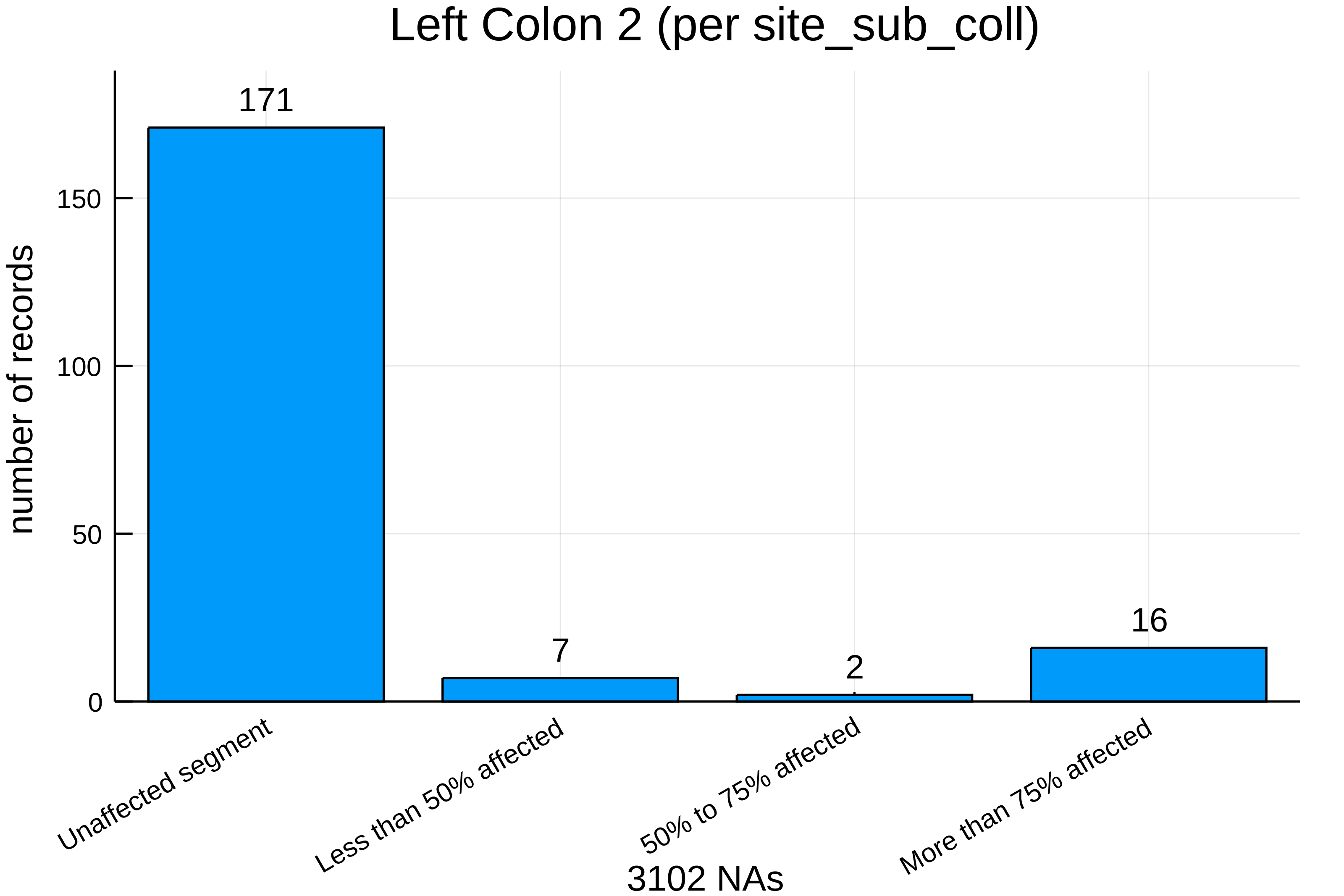

Left Colon 2 (per site_sub_coll)
number of records
0
50
100
150
171
7
2
16
Unaffected segment
Less than 50% affected
50% to 75% affected
More than 75% affected
3102 NAs

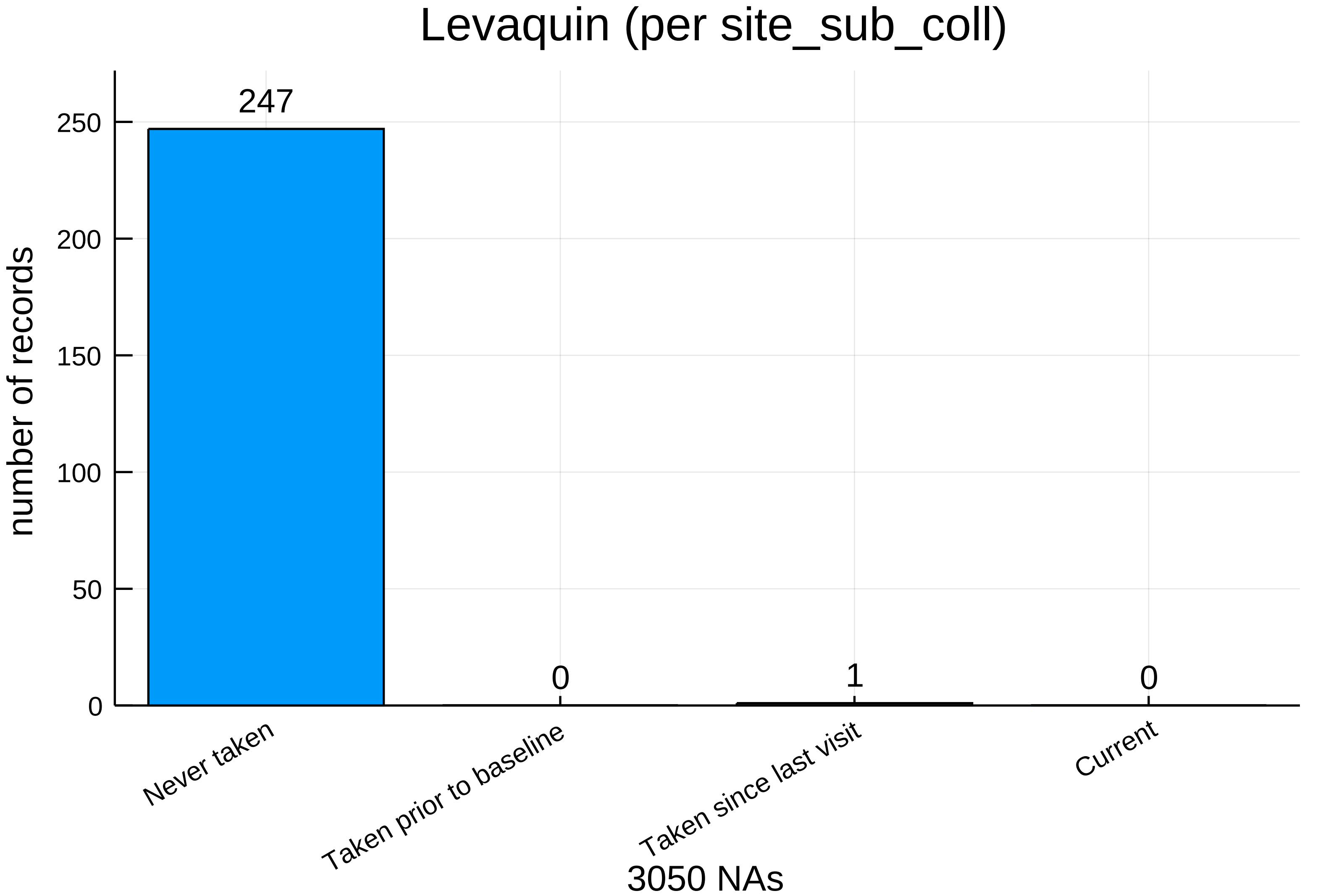

Levaquin (per site_sub_coll)
number of records
250
200
150
100
50
0
247
0
1
0
Never taken
Taken prior to baseline
Taken since last visit
Current
3050 NAs

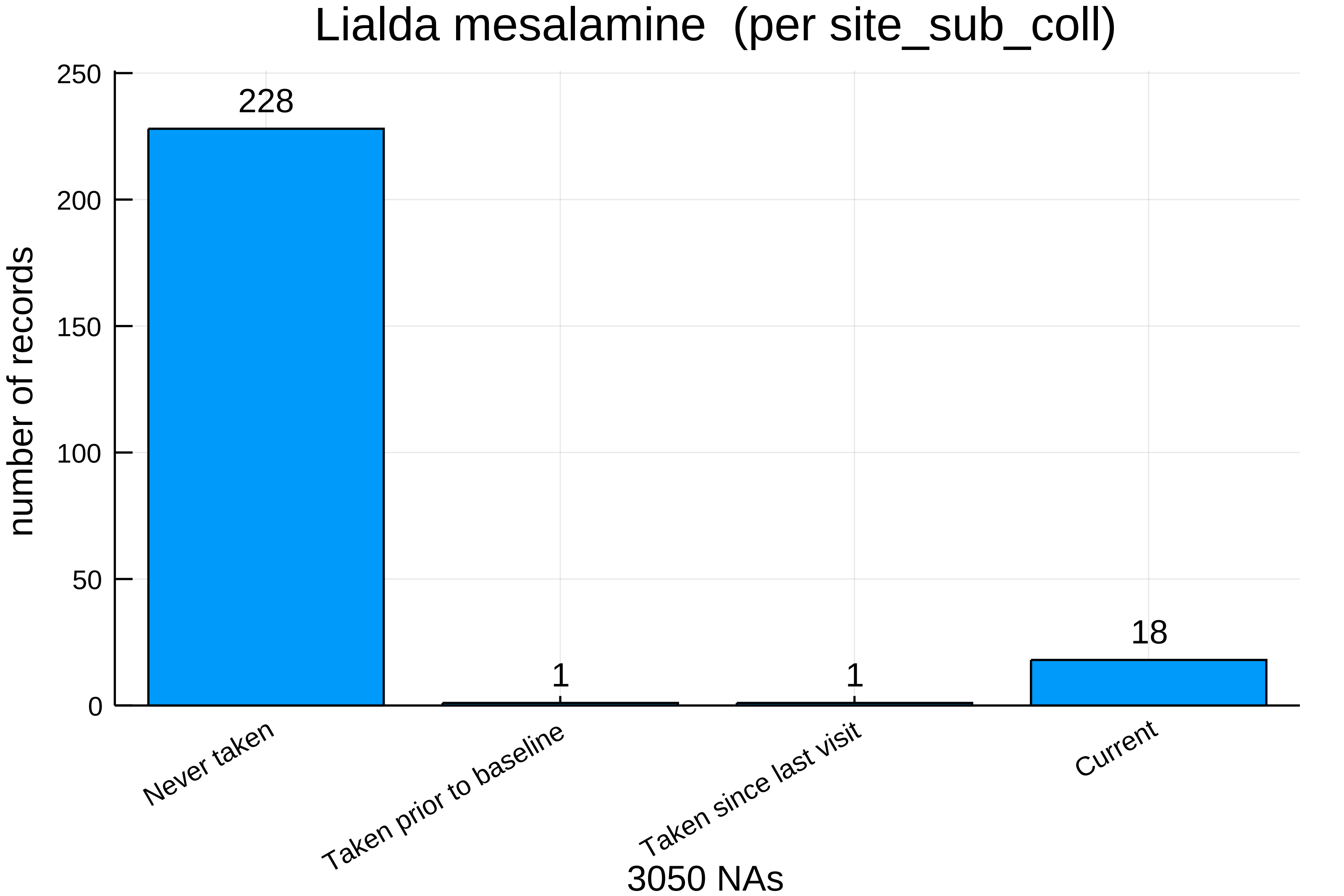

Lialda mesalamine (per site_sub_coll)
number of records
250
200
150
100
50
0
228
1
1
18
Never taken
Taken prior to baseline
Taken since last visit
Current
3050 NAs

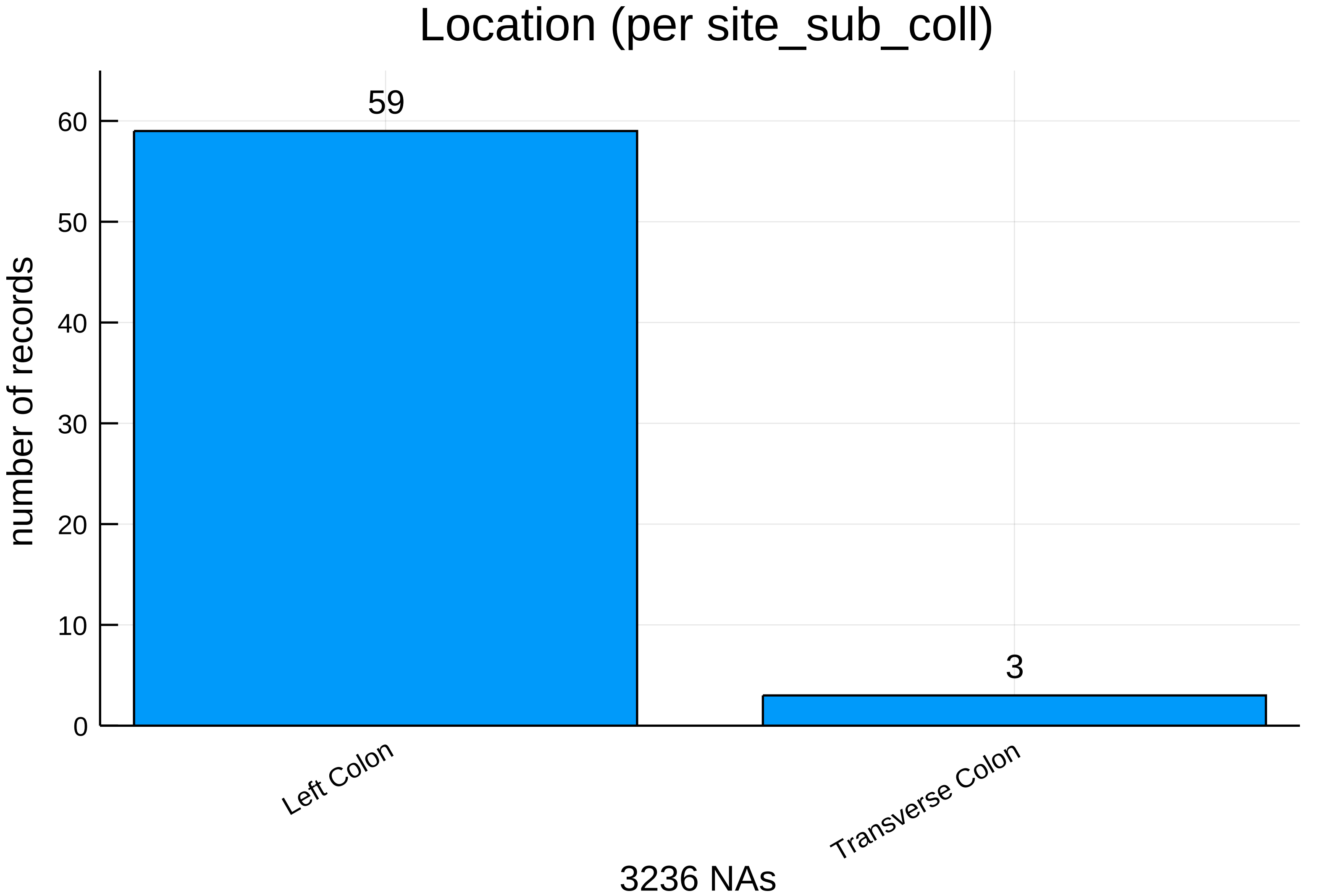
Location (per site_sub_coll)
number of records
0
10
20
30
40
50
60
59
3
Left Colon
Transverse Colon
3236 NAs

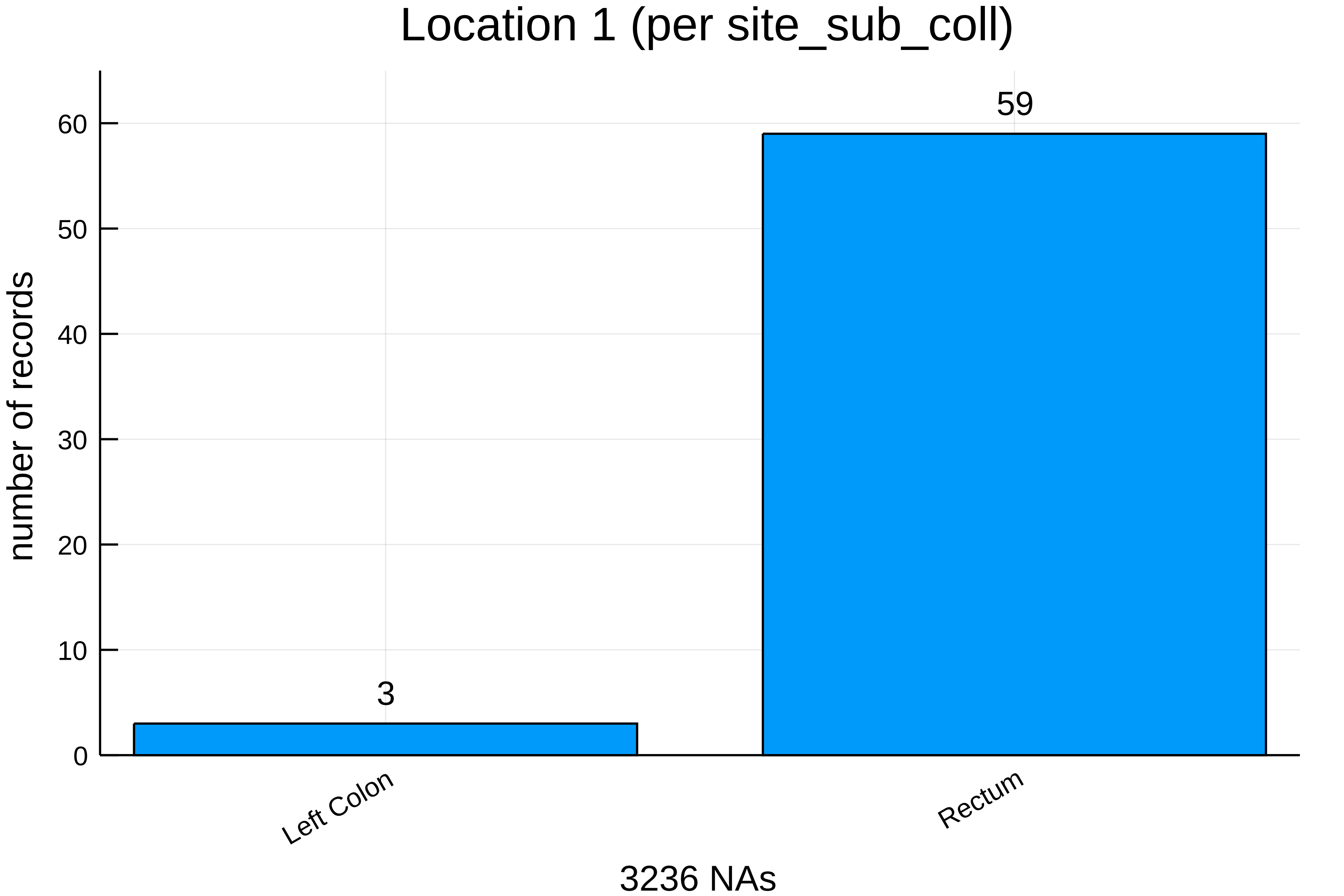
Location 1 (per site_sub_coll)
number of records
0
10
20
30
40
50
60
3
59
Left Colon
Rectum
3236 NAs

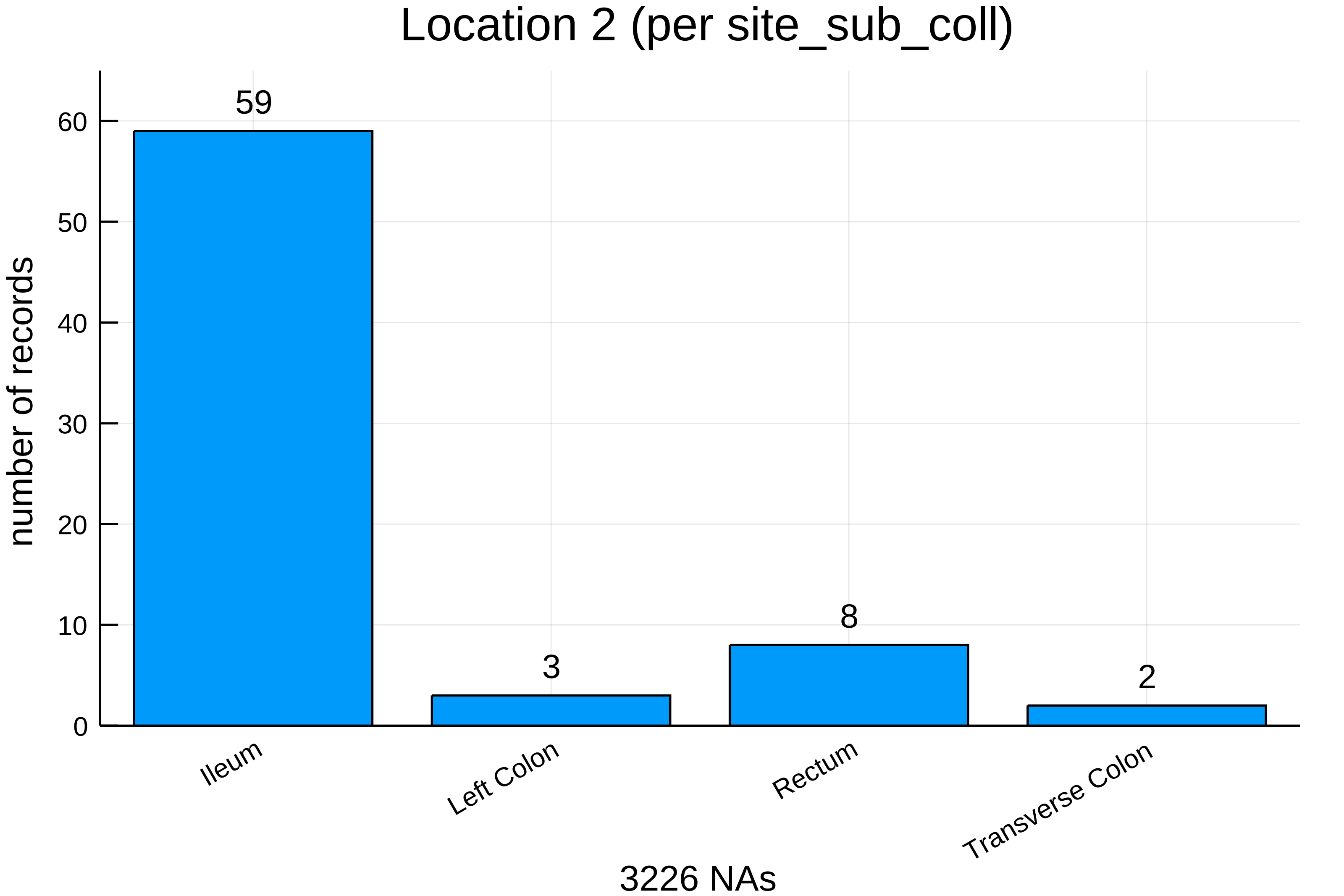
Location 2 (per site_sub_coll)
number of records
0
10
20
30
40
50
60
59
3
8
2
Ileum
Left Colon
Rectum
Transverse Colon
3226 NAs

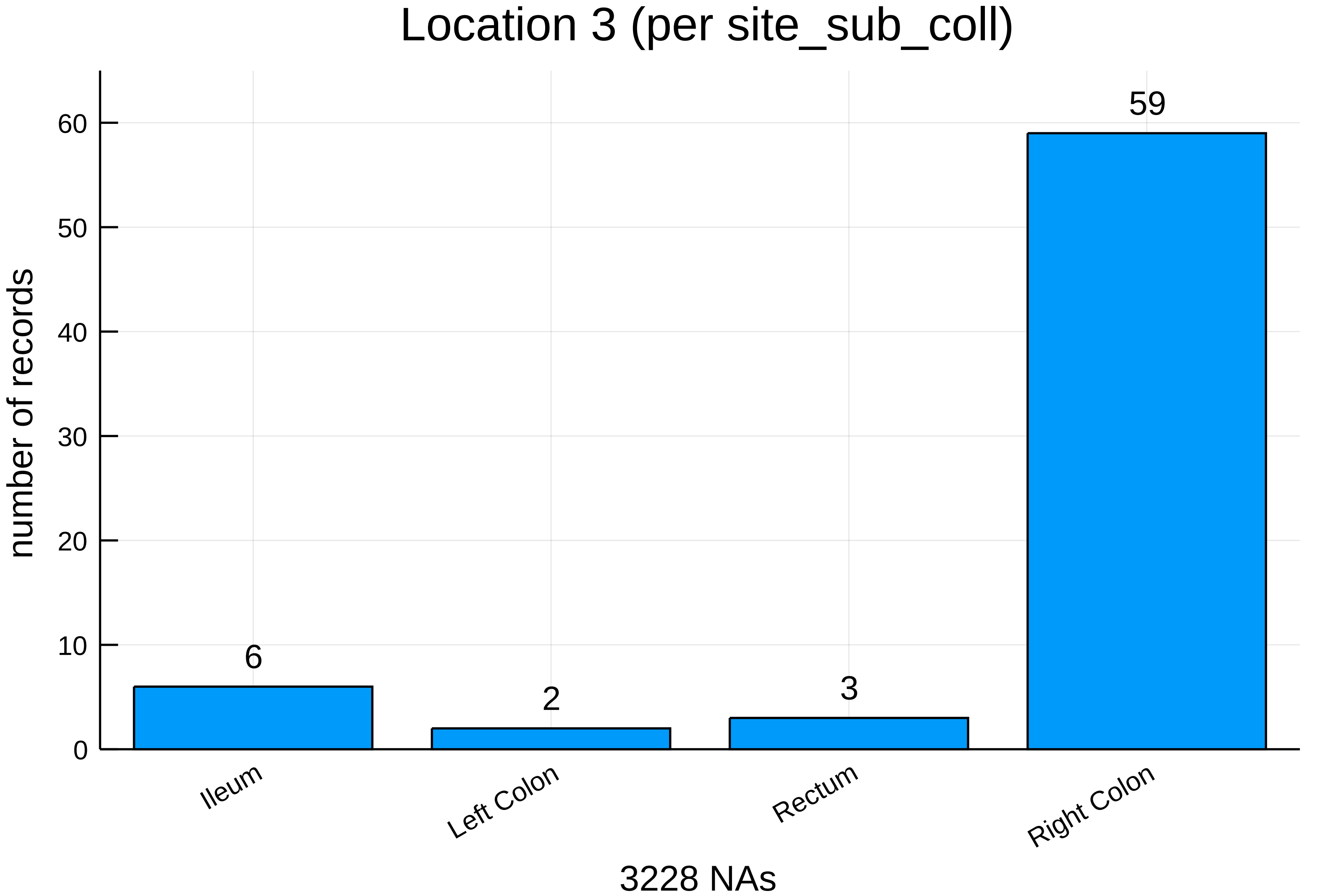
Location 3 (per site_sub_coll)
number of records
0
10
20
30
40
50
60
6
2
3
59
Ileum
Left Colon
Rectum
Right Colon
3228 NAs

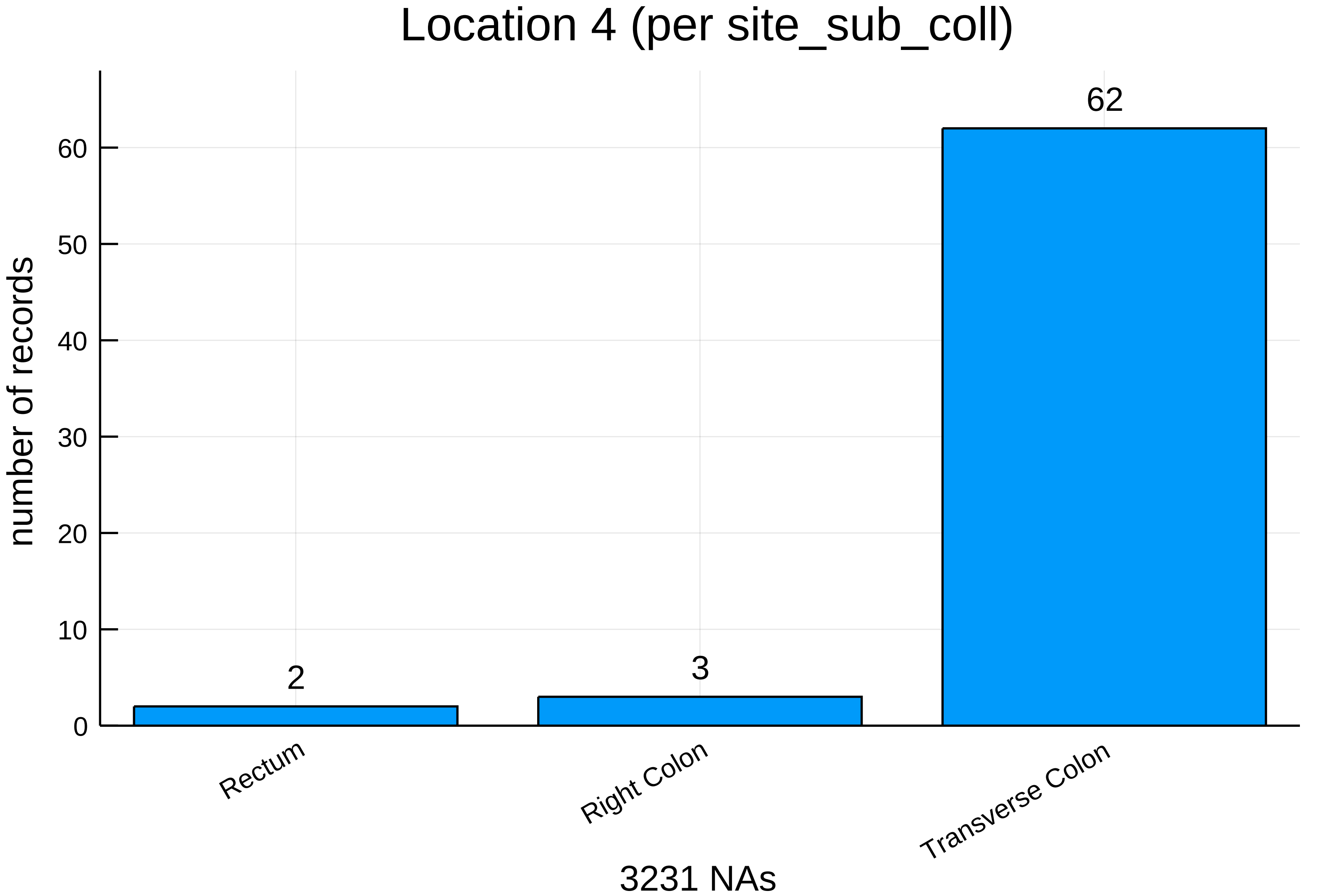
Location 4 (per site_sub_coll)
number of records
0
10
20
30
40
50
60
2
3
62
Rectum
Right Colon
Transverse Colon
3231 NAs

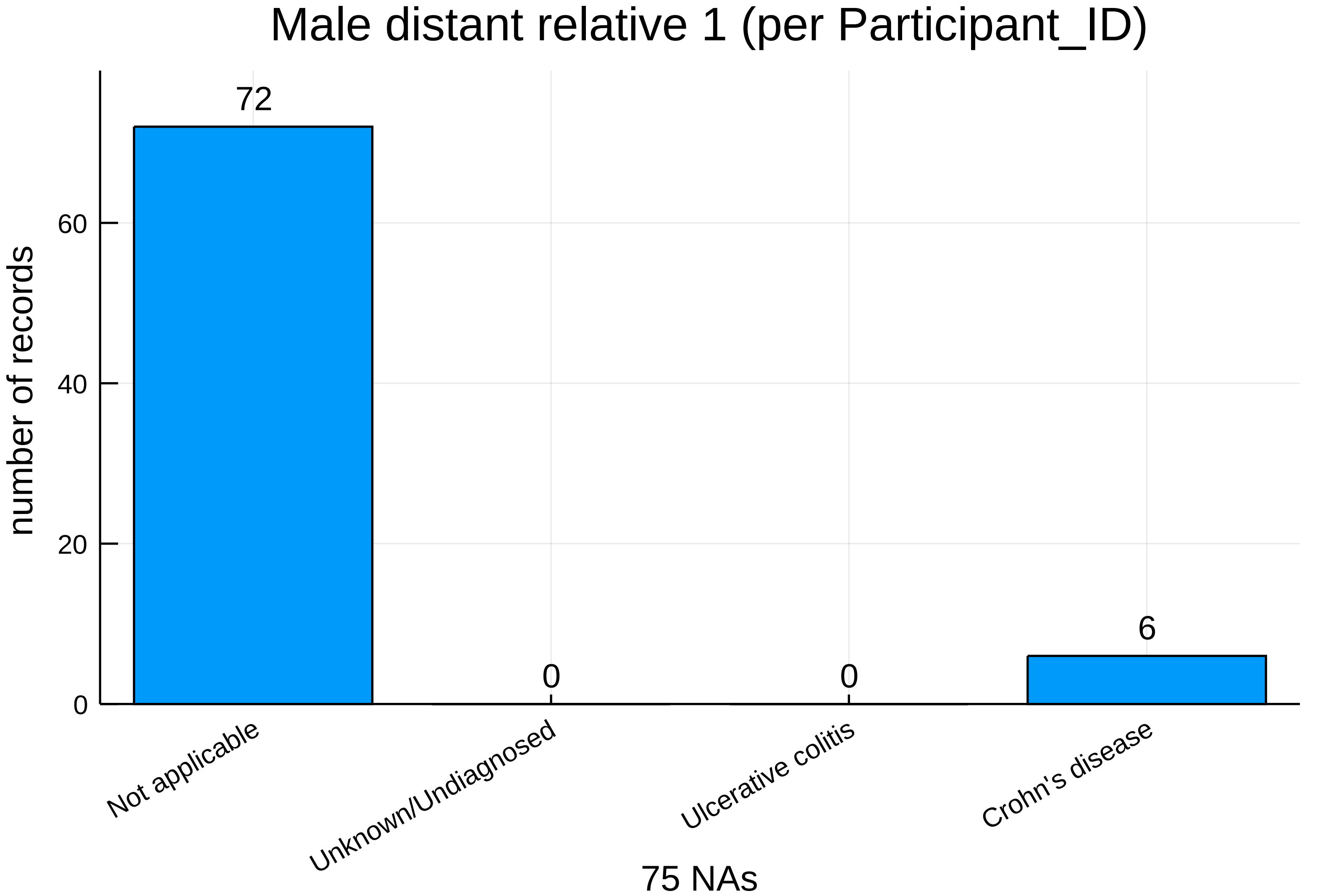
Male distant relative 1 (per Participant_ID)
72
0
0
6
60
40
20
0
number of records
Not applicable
Unknown/Undiagnosed
Ulcerative colitis
Crohn's disease
75 NAs

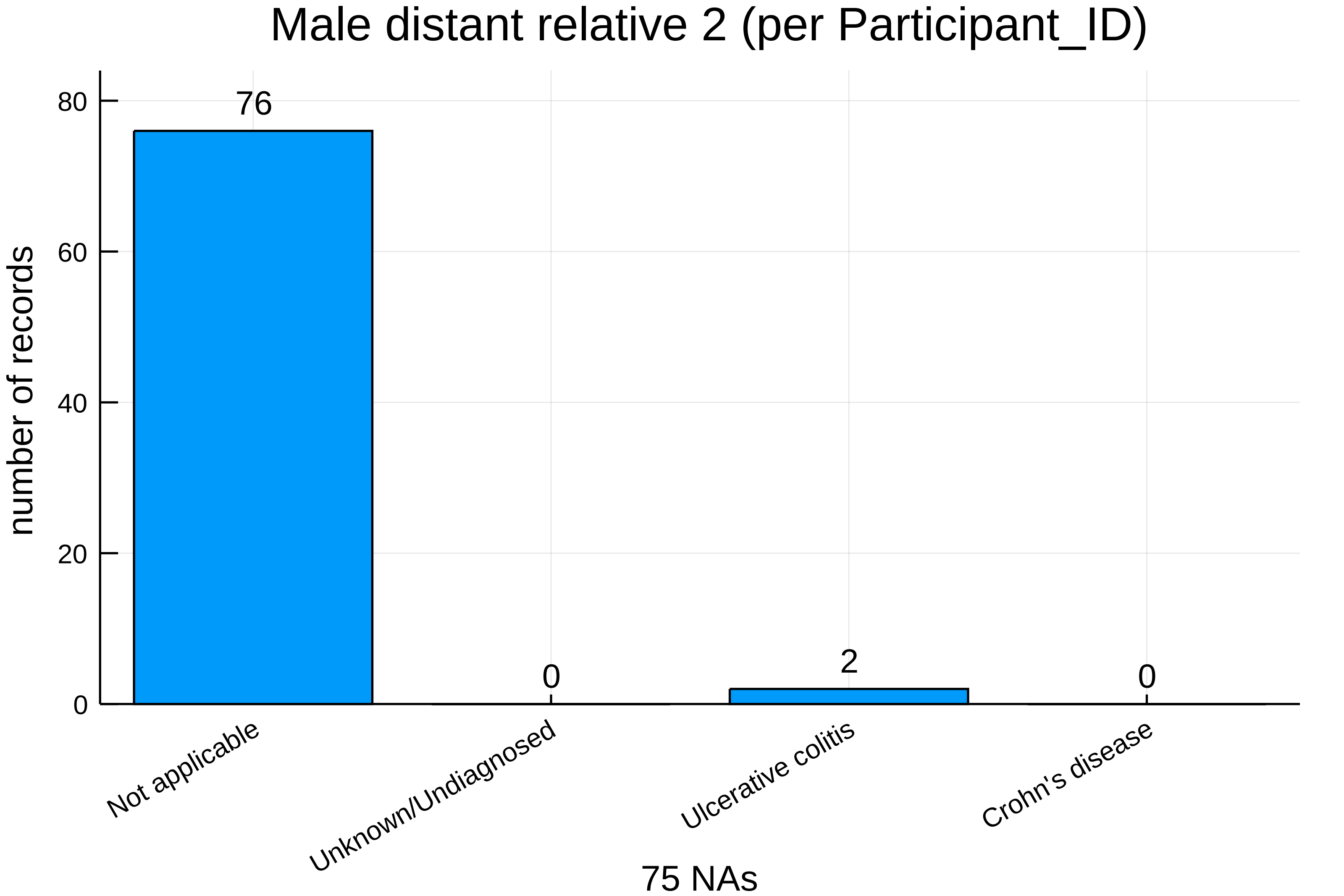
Male distant relative 2 (per Participant_ID)
number of records
0
20
40
60
80
76
0
2
0
Not applicable
Unknown/Undiagnosed
Ulcerative colitis
Crohn's disease
75 NAs

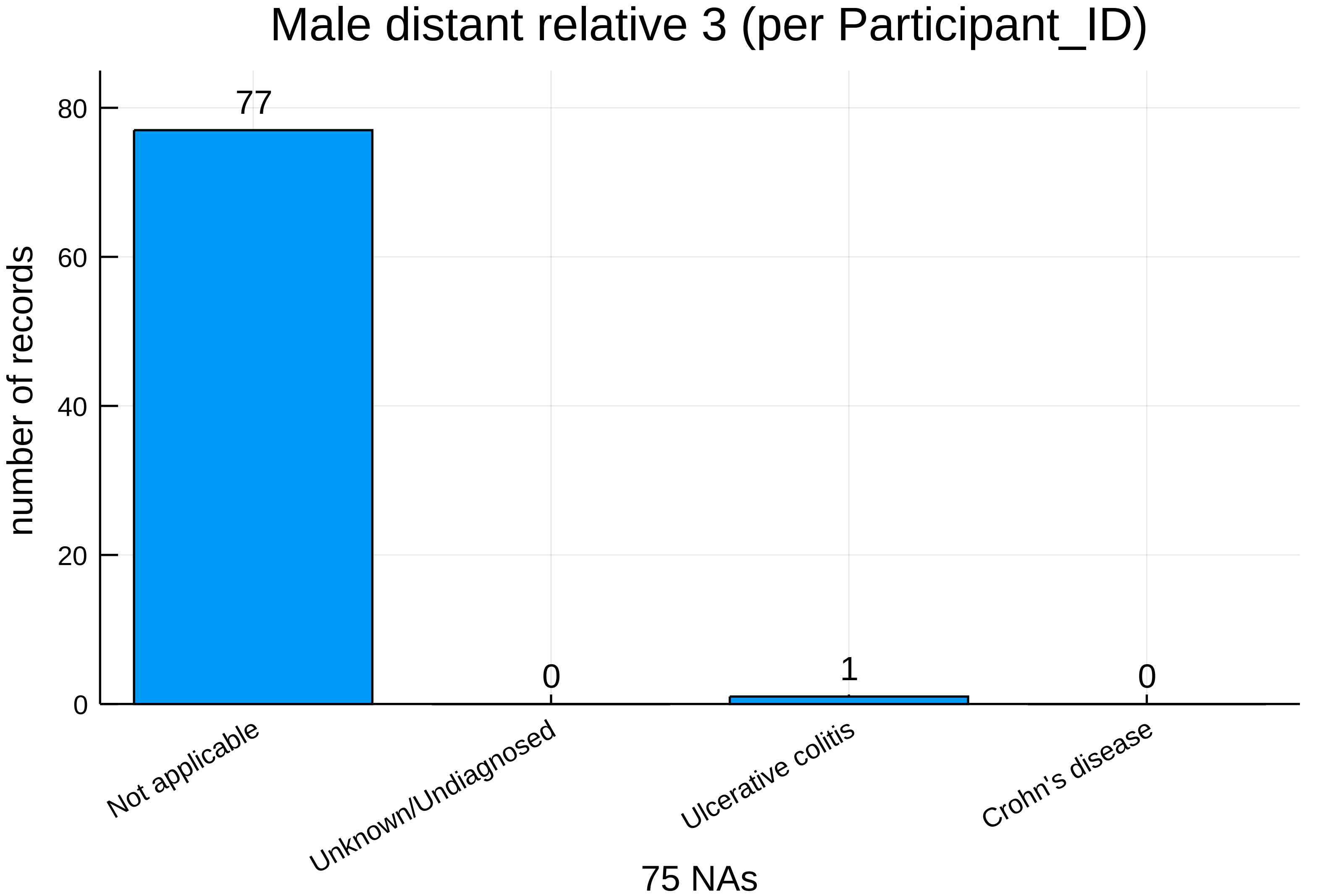

Male distant relative 3 (per Participant_ID)
number of records
80
60
40
20
0
77
0
1
0
Not applicable
Unknown/Undiagnosed
Ulcerative colitis
Crohn's disease
75 NAs

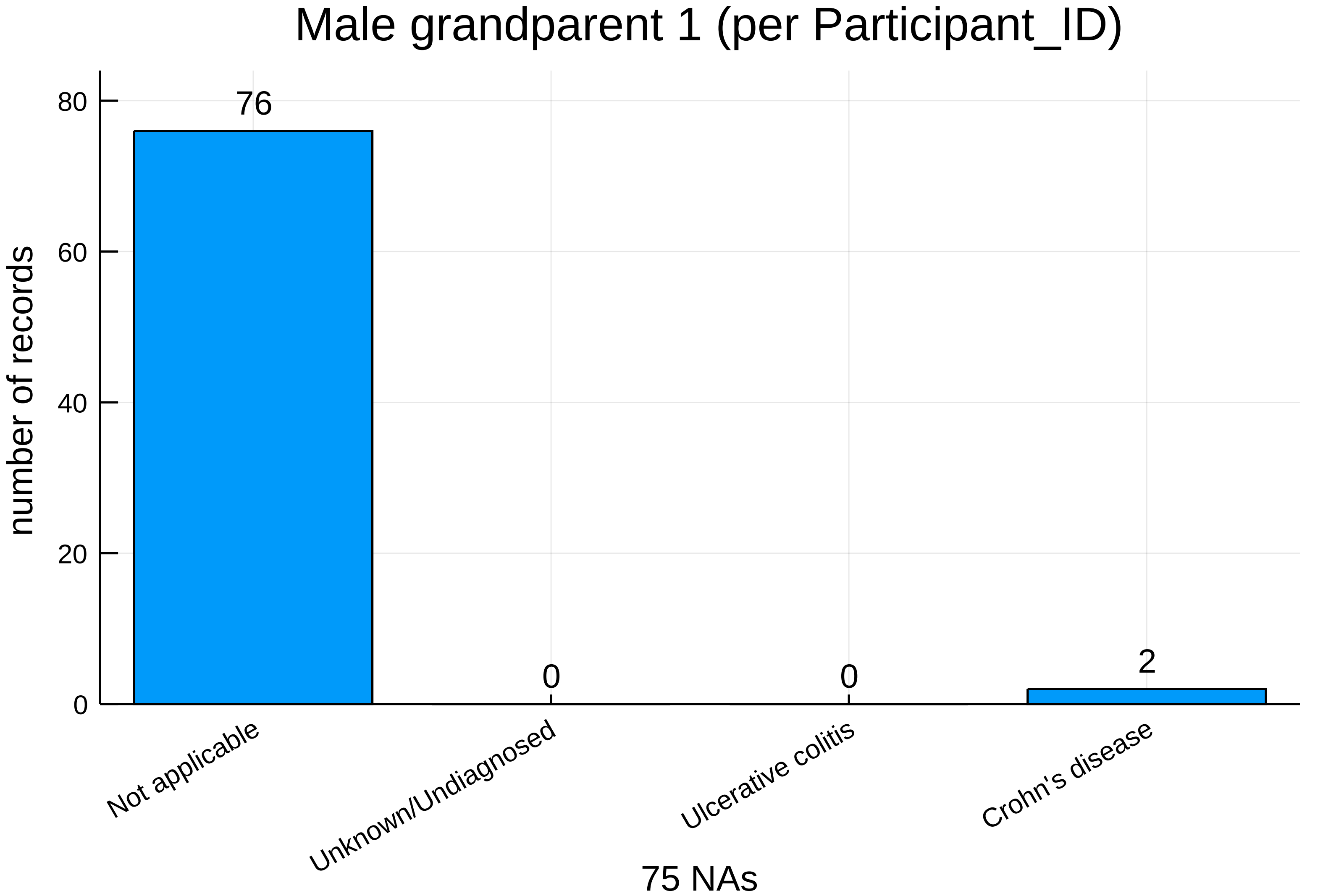
Male grandparent 1 (per Participant_ID)
number of records
0
20
40
60
80
76
0
0
2
Not applicable
Unknown/Undiagnosed
Ulcerative colitis
Crohn's disease
75 NAs

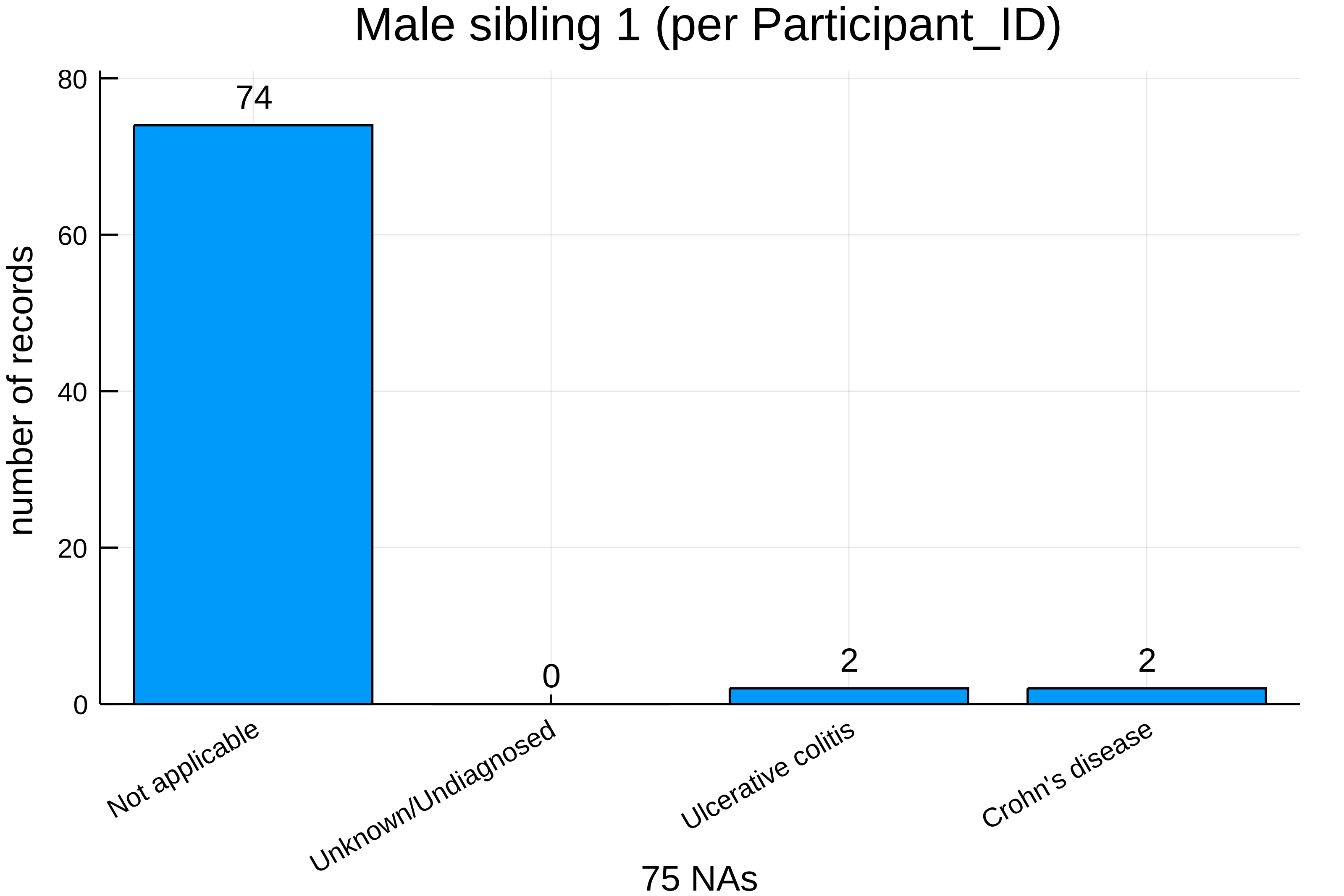
Male sibling 1 (per Participant_ID)
number of records
0
20
40
60
80
74
0
2
2
Not applicable
Unknown/Undiagnosed
Ulcerative colitis
Crohn's disease
75 NAs

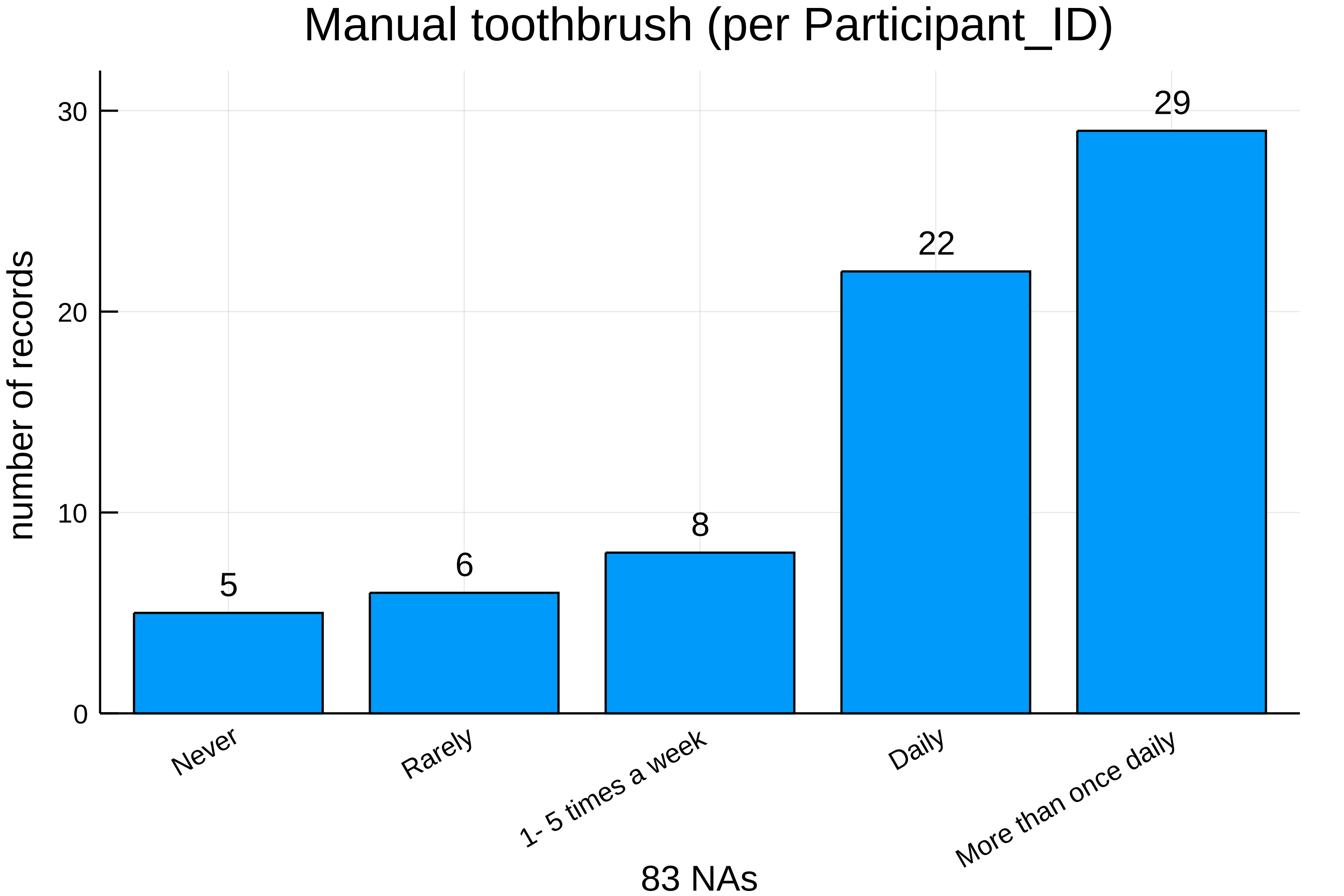
Manual toothbrush (per Participant_ID)
number of records
30
20
10
0
5
6
8
22
29
Never
Rarely
1- 5 times a week
Daily
More than once daily
83 NAs

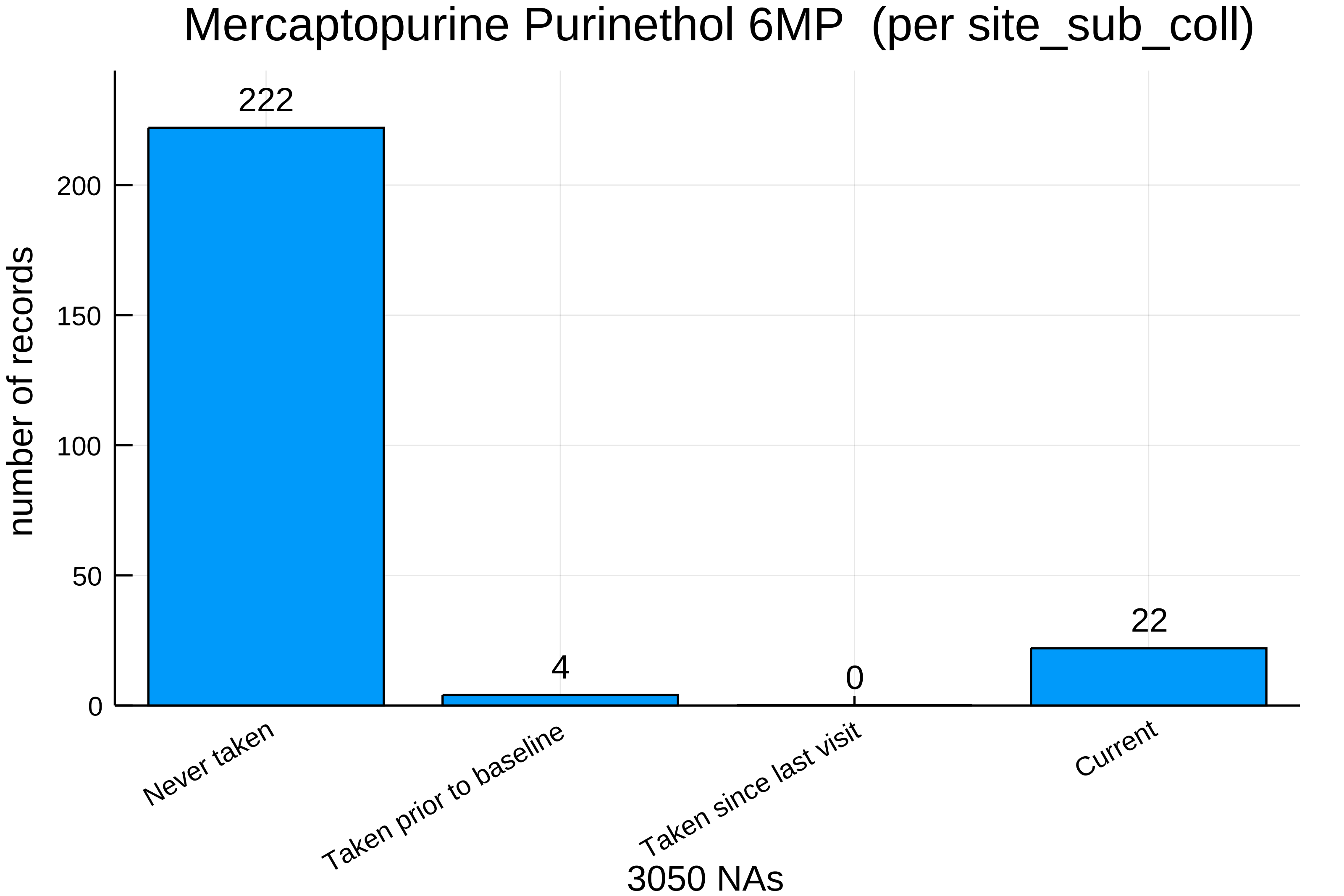
Mercaptopurine Purinethol 6MP (per site_sub_coll)
number of records
222
4
0
22
0
50
100
150
200
Never taken
Taken prior to baseline
Taken since last visit
Current
3050 NAs

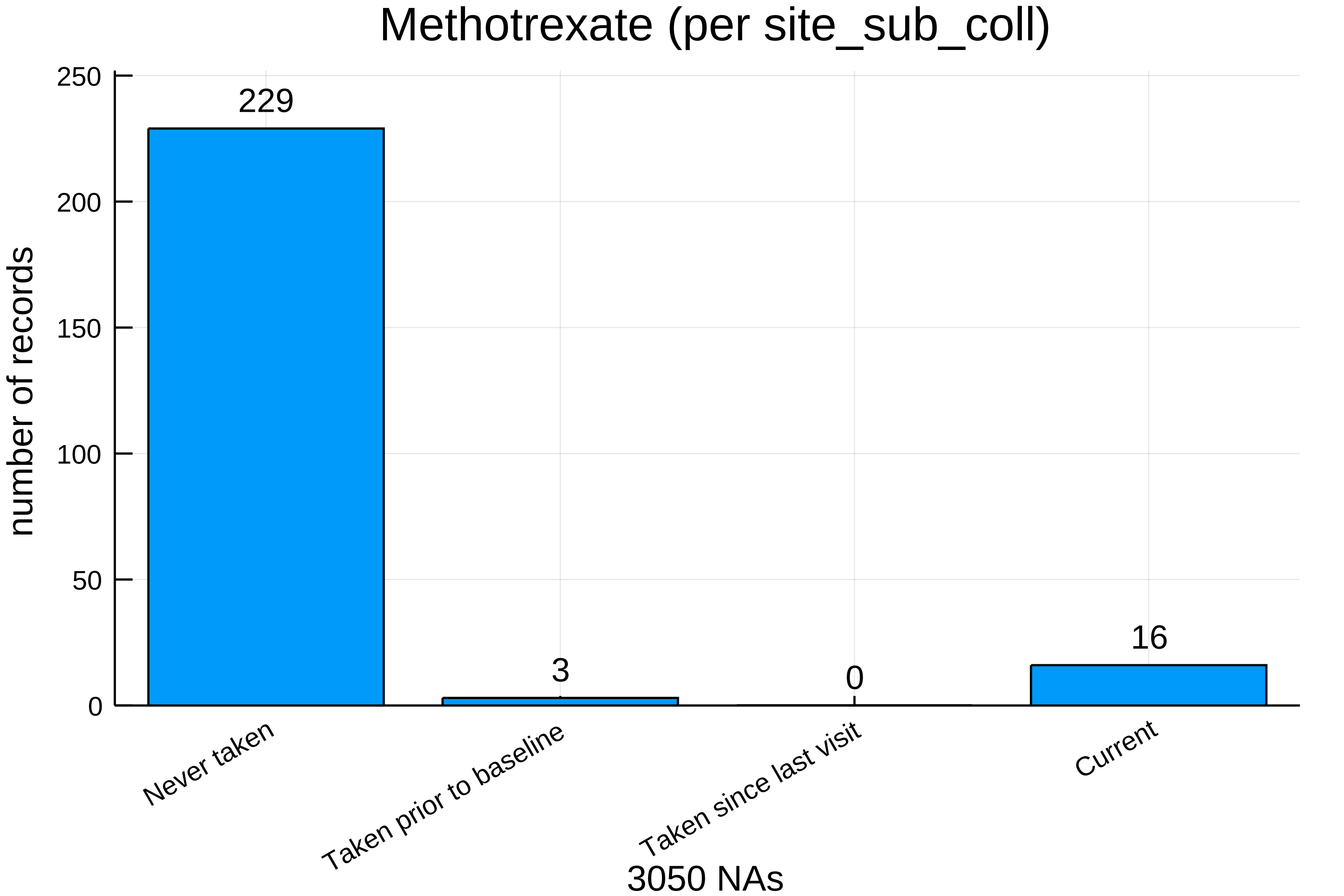
Methotrexate (per site_sub_coll)
number of records
250
200
150
100
50
0
229
3
0
16
Never taken
Taken prior to baseline
Taken since last visit
Current
3050 NAs

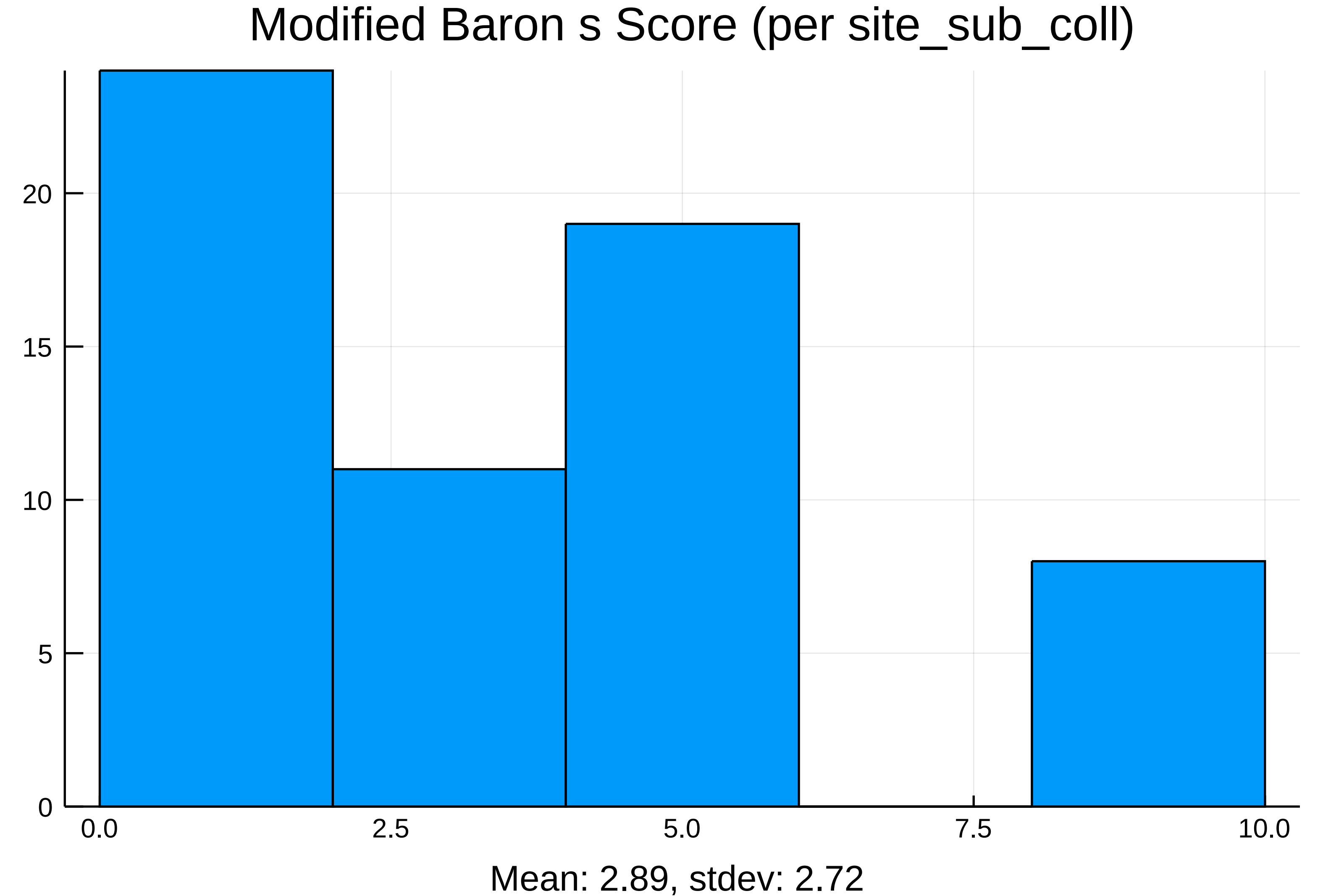
Modified Baron s Score (per site_sub_coll)
0
5
10
15
20
0.0
2.5
5.0
7.5
10.0
Mean: 2.89, stdev: 2.72

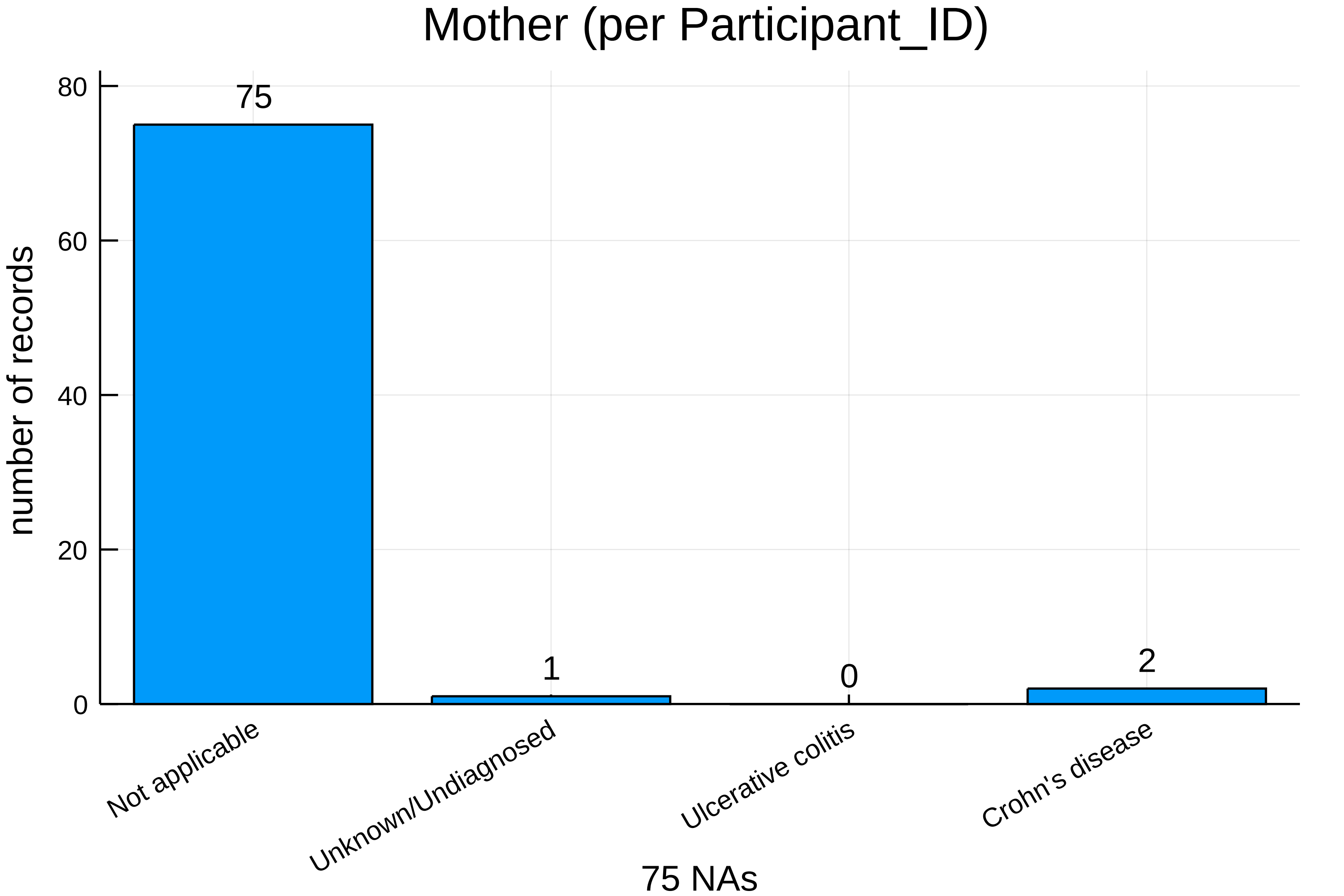
Mother (per Participant_ID)
number of records
80
60
40
20
0
75
1
0
2
Not applicable
Unknown/Undiagnosed
Ulcerative colitis
Crohn's disease
75 NAs

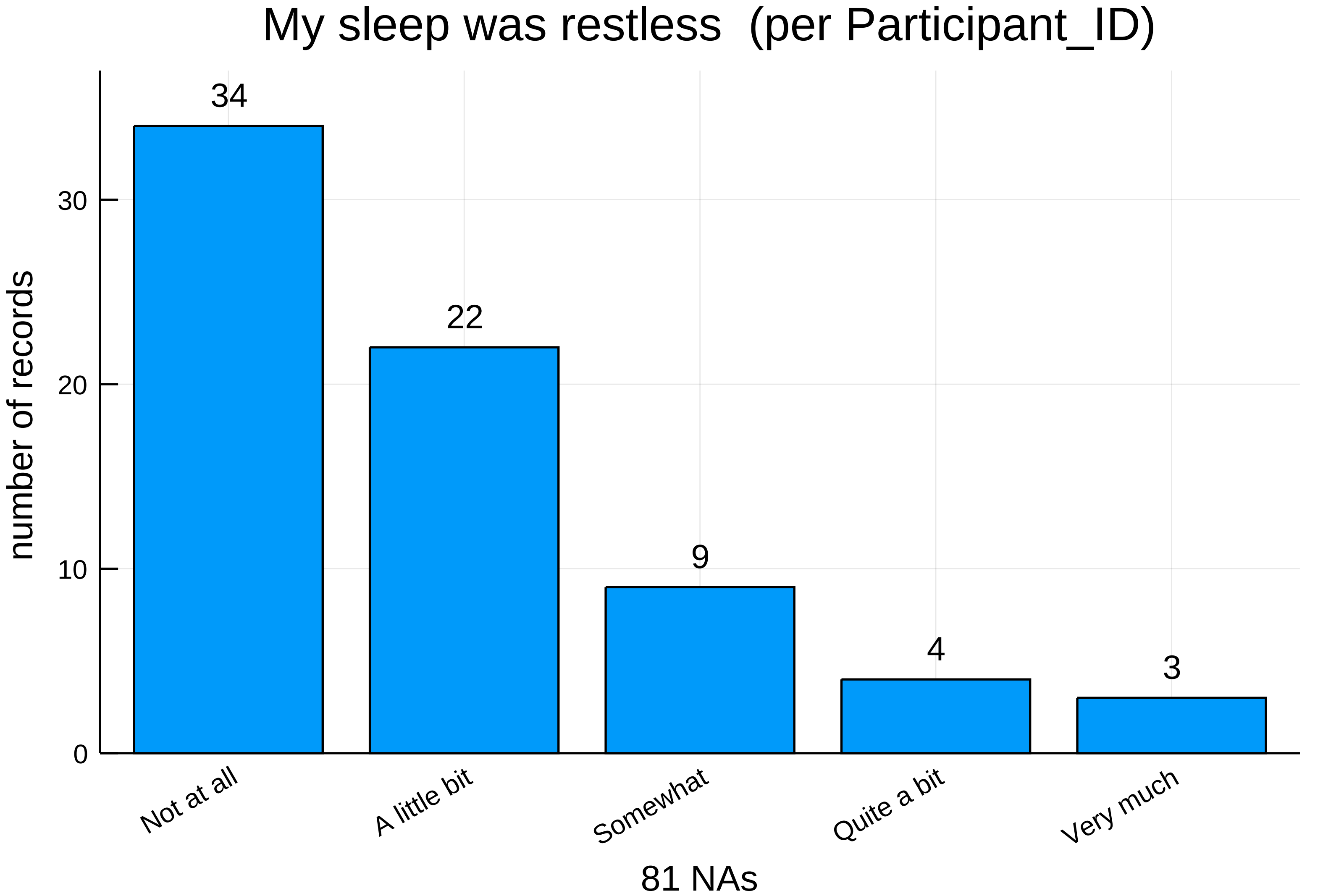

My sleep was restless (per Participant_ID)
number of records
0
10
20
30
34
22
9
4
3
Not at all
A little bit
Somewhat
Quite a bit
Very much
81 NAs

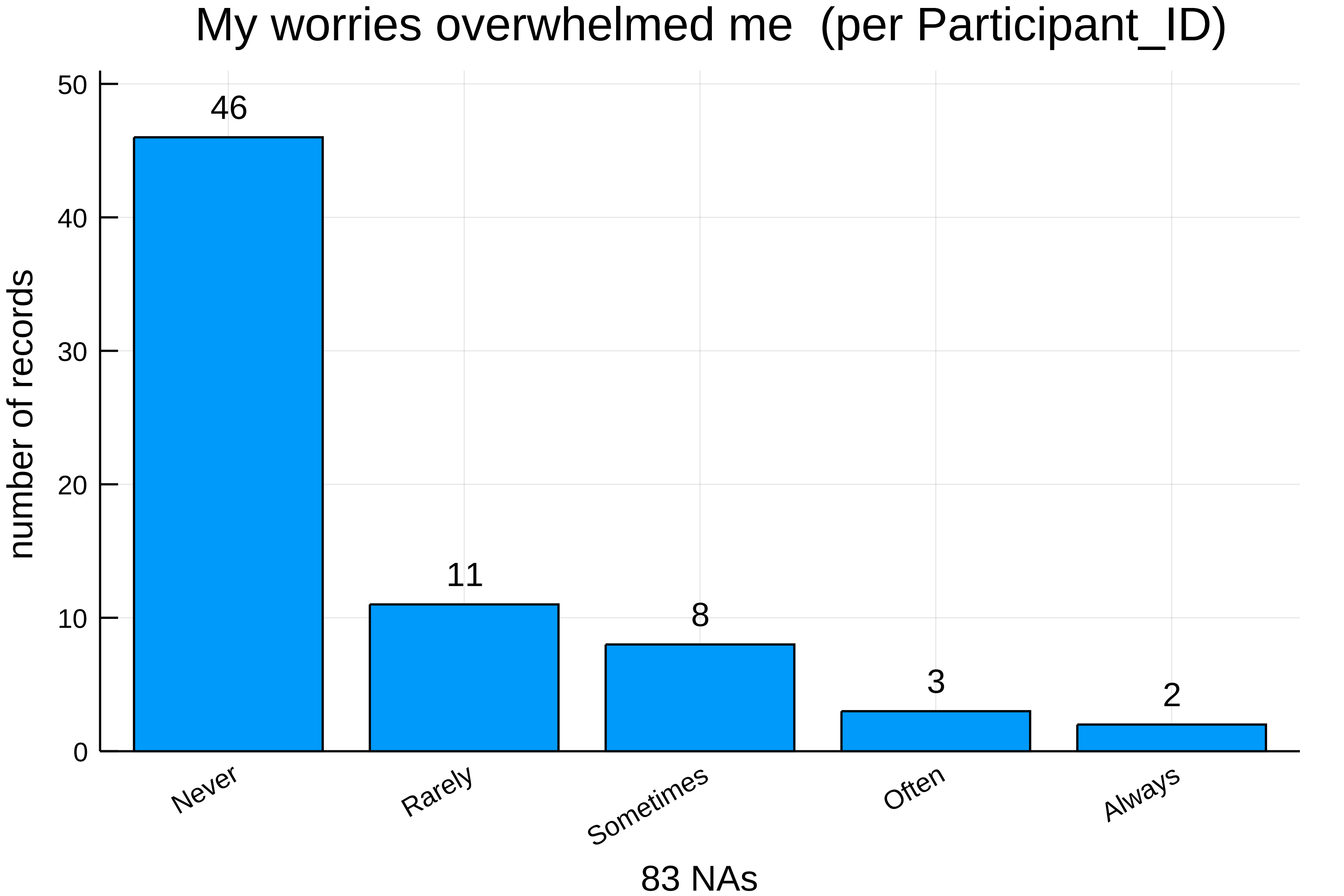
My worries overwhelmed me (per Participant_ID)
number of records
50
40
30
20
10
0
46
11
8
3
2
Never
Rarely
Sometimes
Often
Always
83 NAs

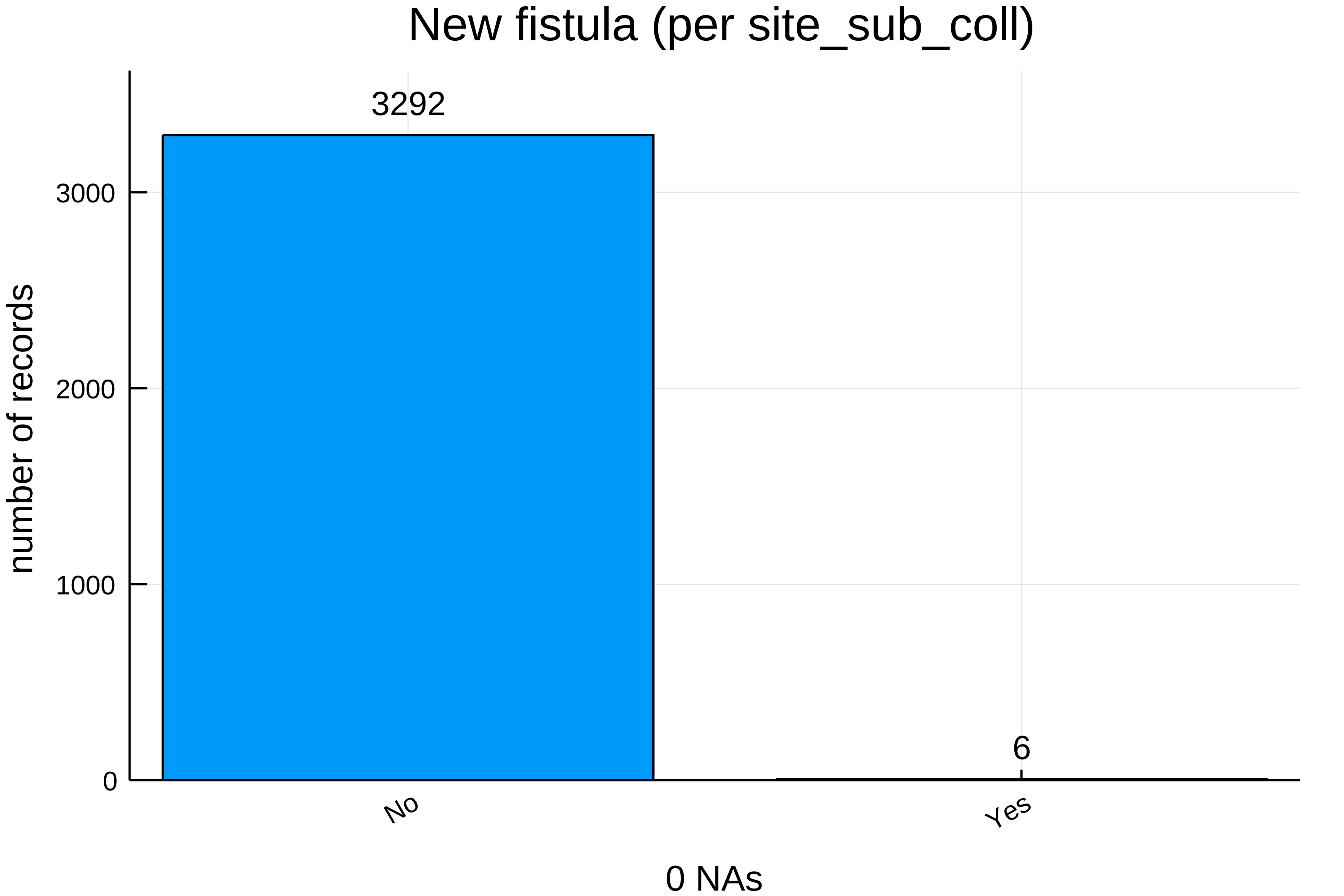
New fistula (per site_sub_coll)
number of records
3292
6
3000
2000
1000
0
No
Yes
0 NAs

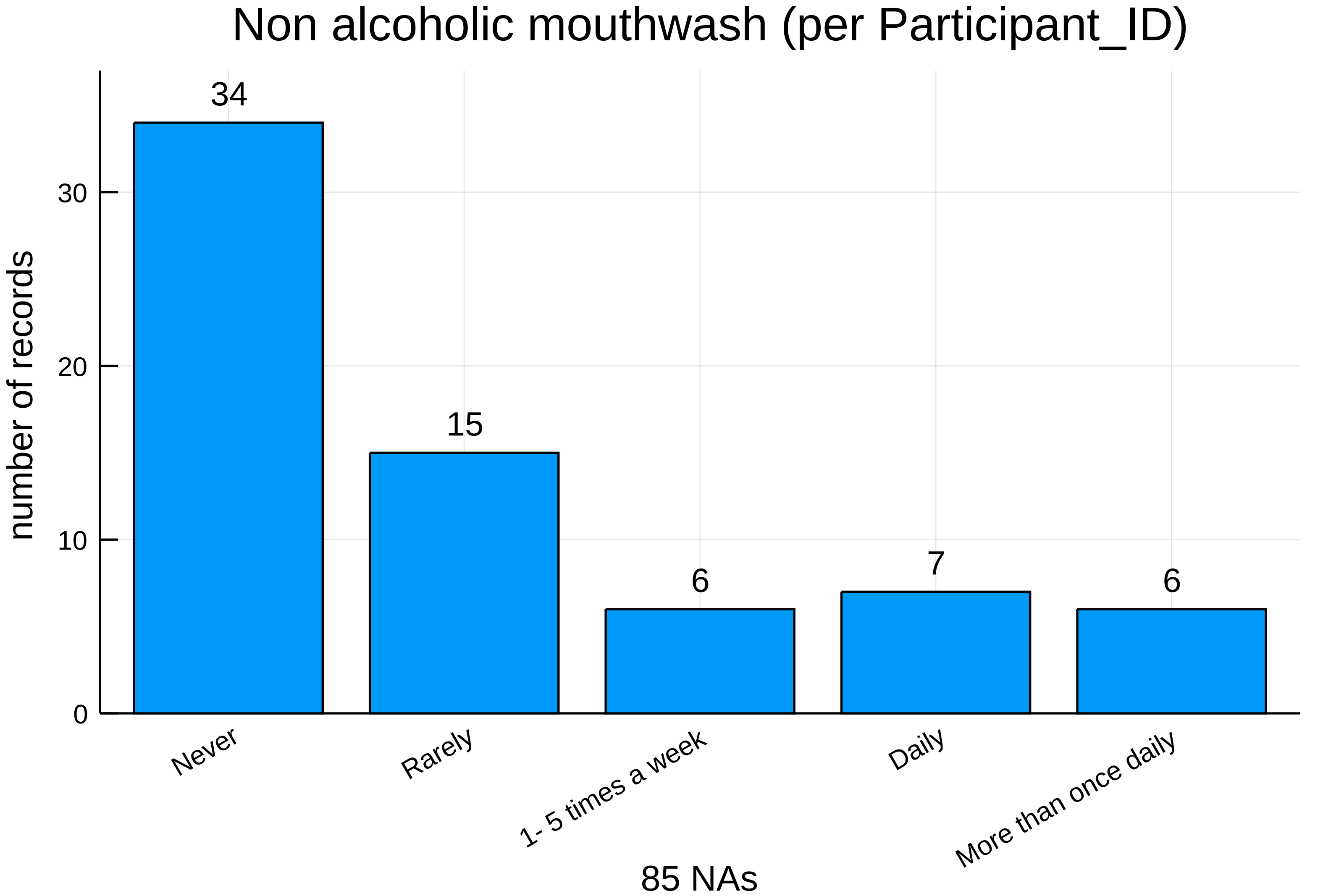
Non alcoholic mouthwash (per Participant_ID)
number of records
0
10
20
30
34
15
6
7
6
Never
Rarely
1- 5 times a week
Daily
More than once daily
85 NAs

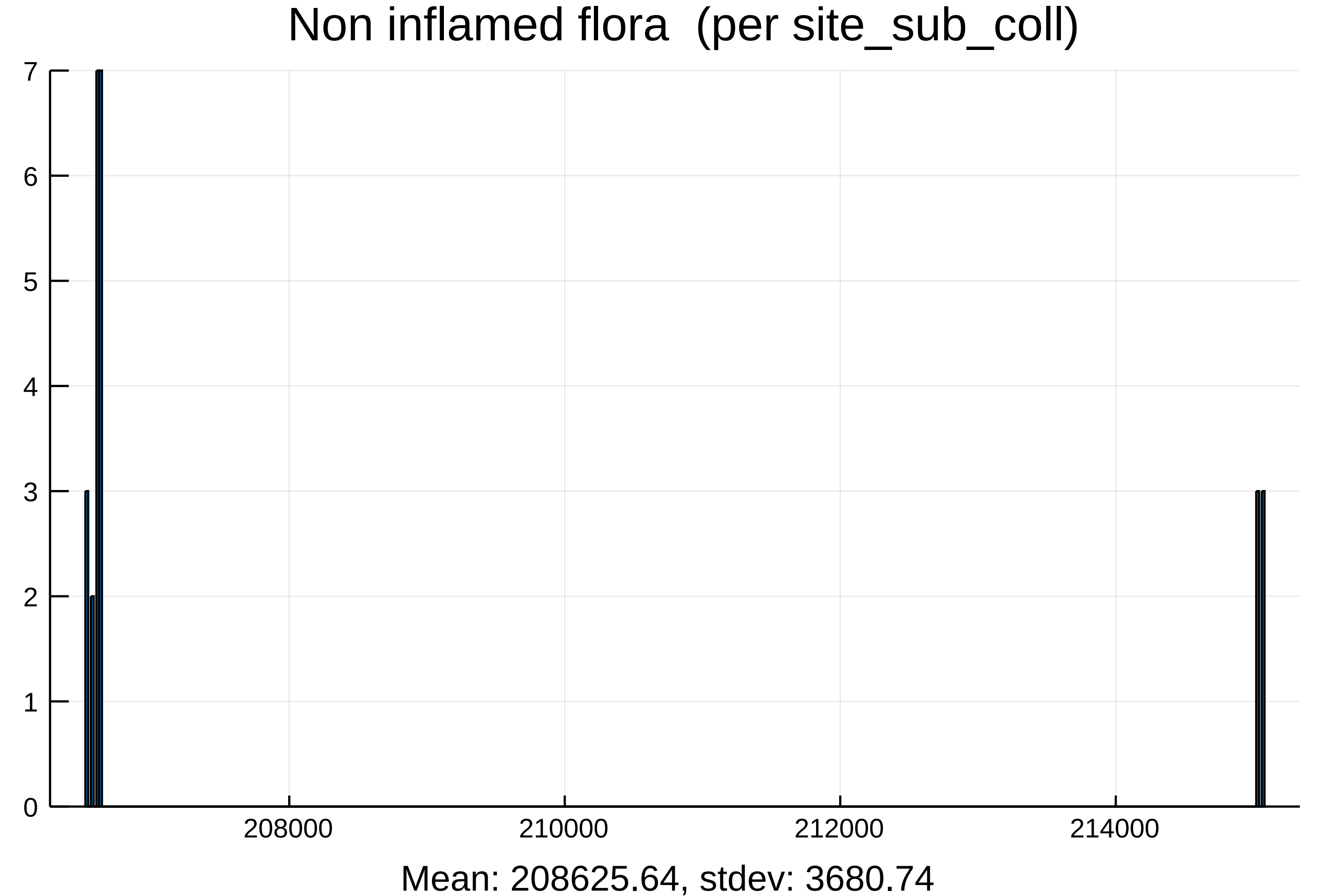
Non inflamed flora (per site_sub_coll)
7
6
5
4
3
2
1
0
208000
210000
212000
214000
Mean: 208625.64, stdev: 3680.74

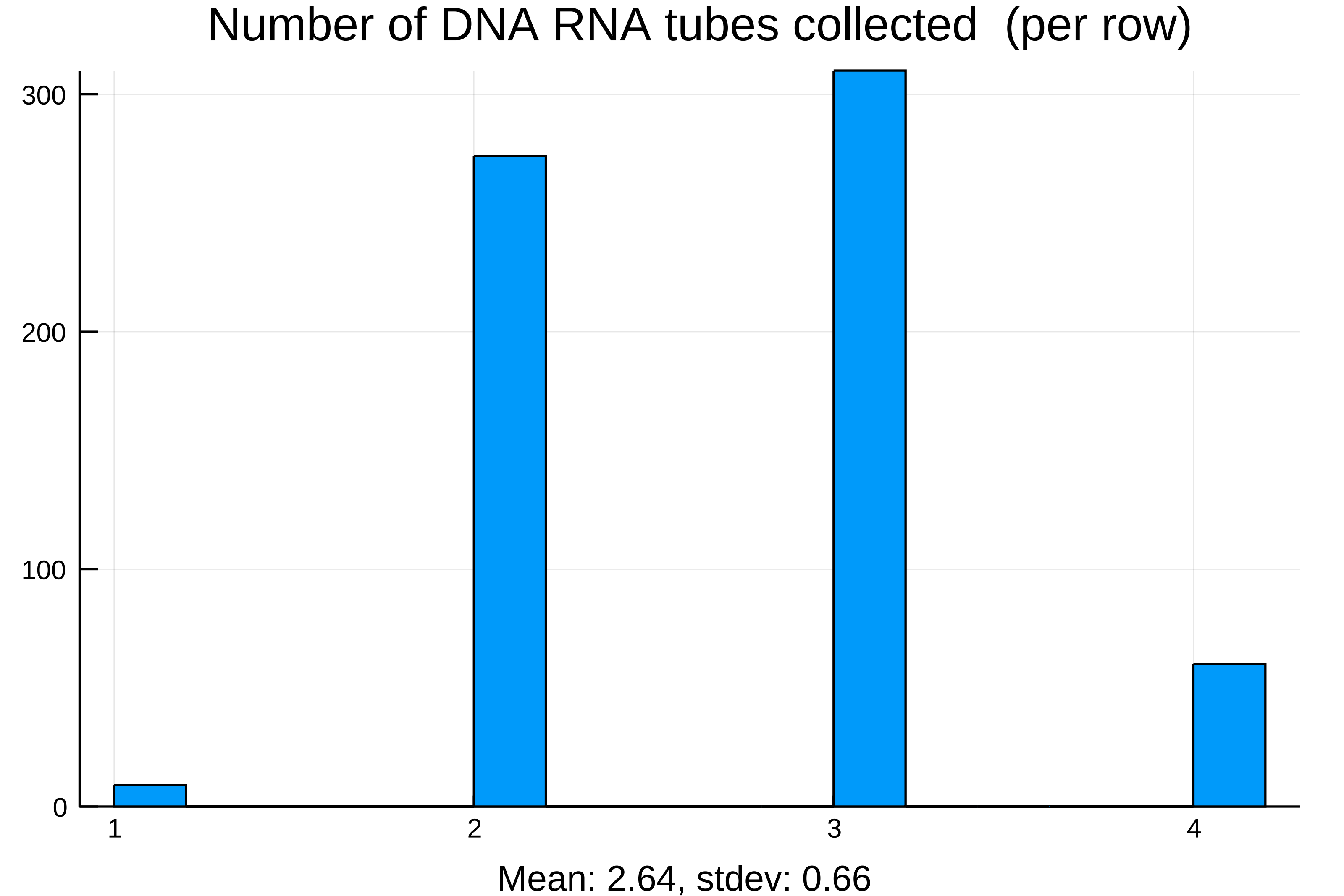
Number of DNA RNA tubes collected (per row)
300
200
100
0
1
2
3
4
Mean: 2.64, stdev: 0.66

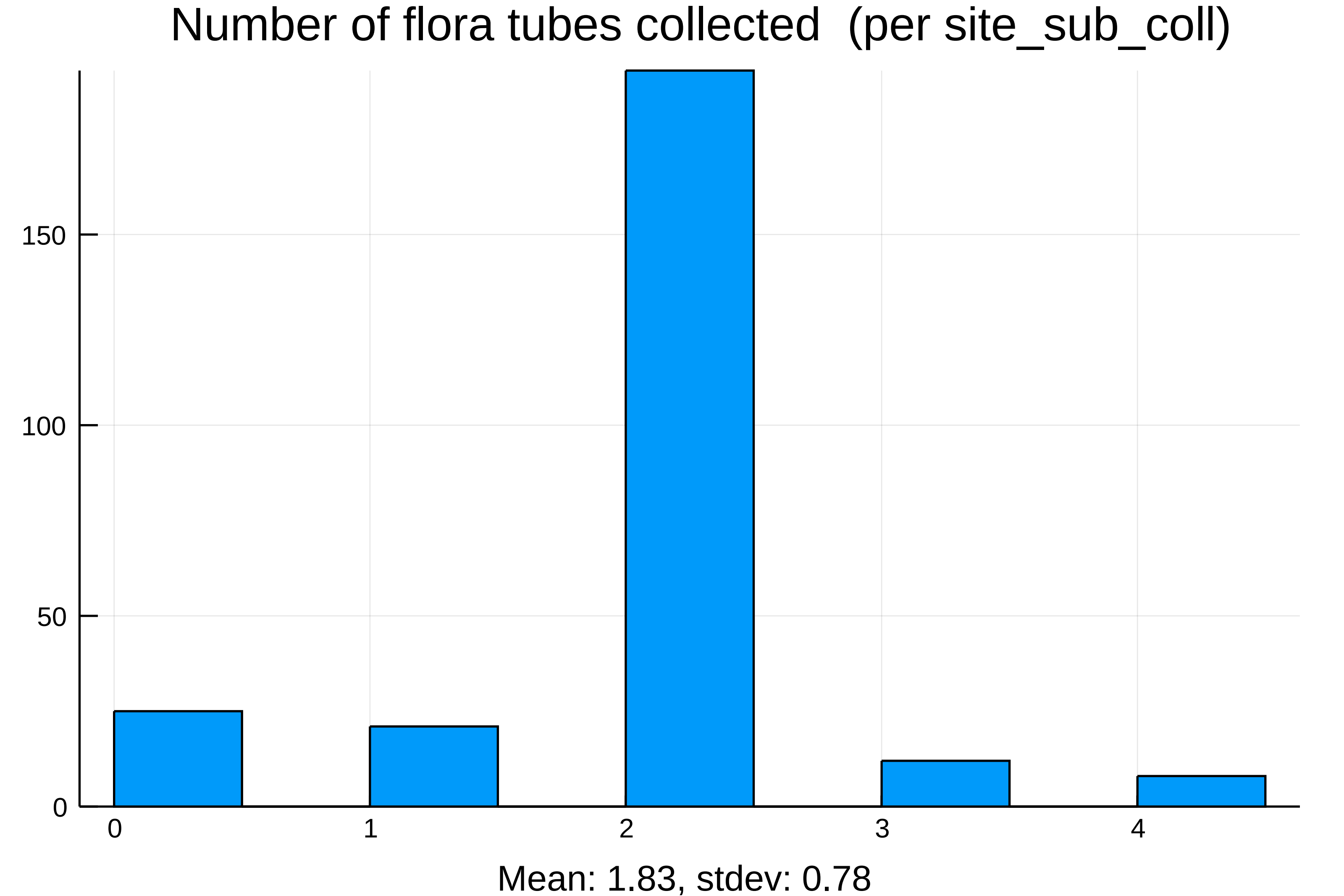
Number of flora tubes collected (per site_sub_coll)
150
100
50
0
0
1
2
3
4
Mean: 1.83, stdev: 0.78

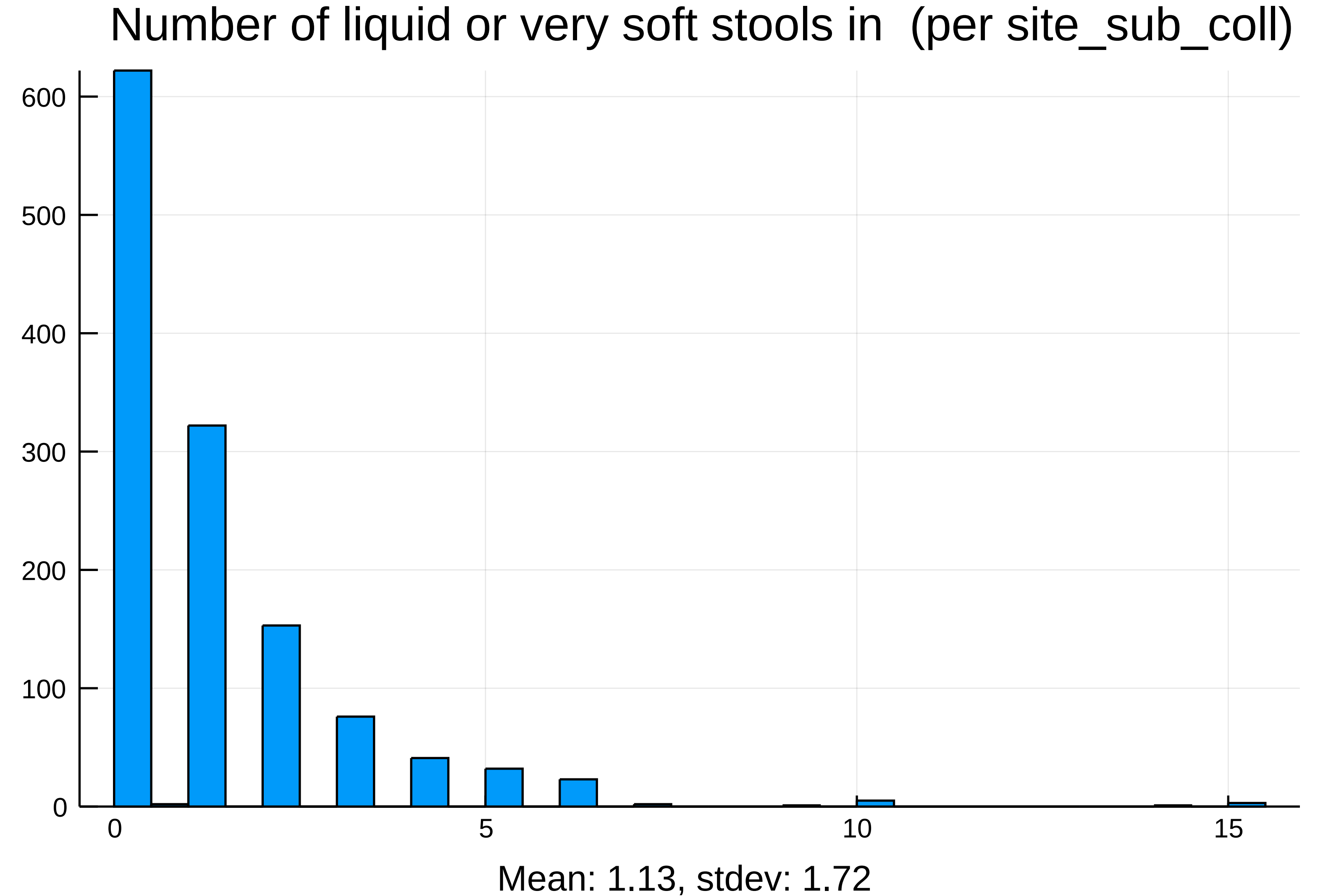
Number of liquid or very soft stools in (per site_sub_coll)
600
500
400
300
200
100
0
0
5
10
15
Mean: 1.13, stdev: 1.72

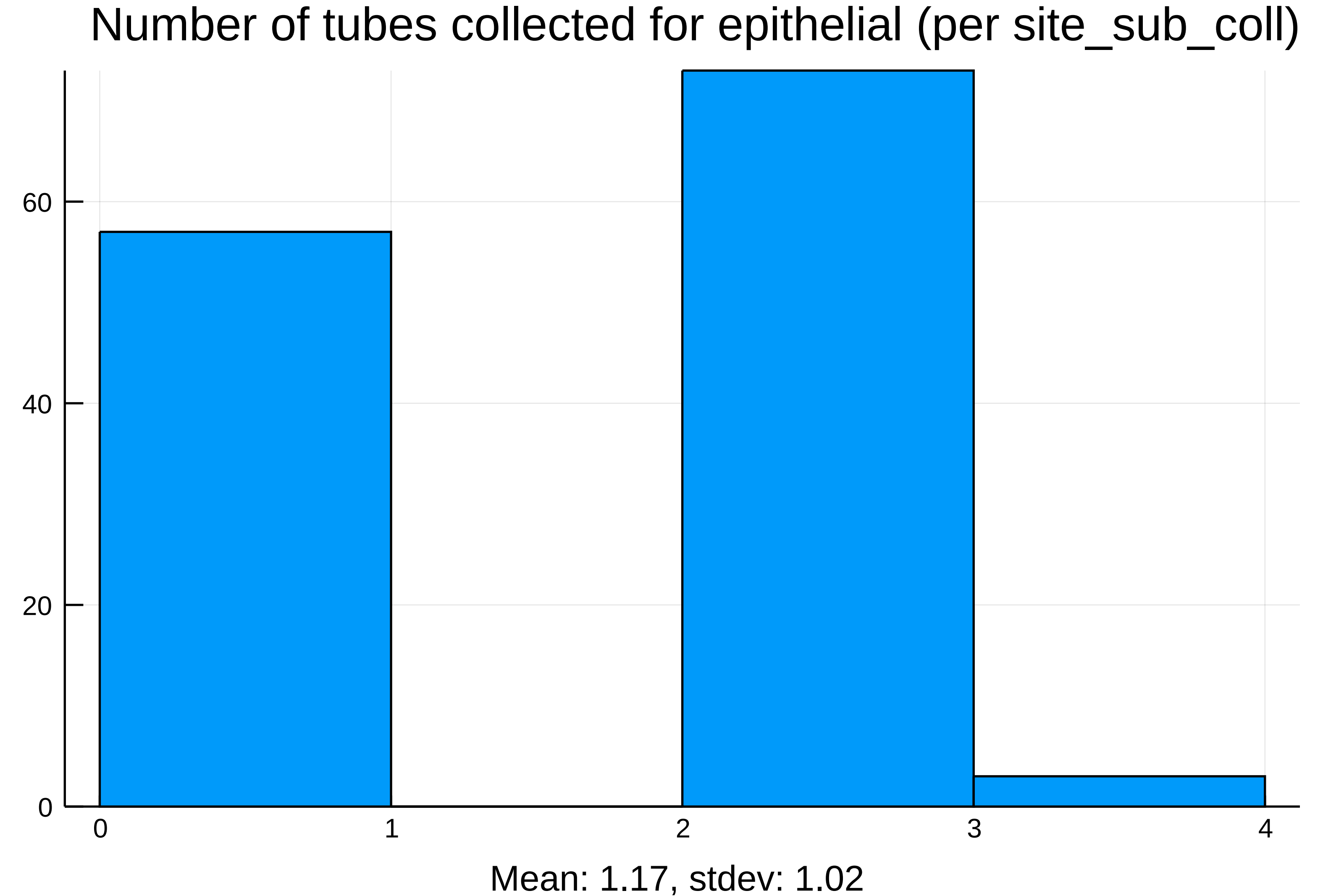
Number of tubes collected for epithelial (per site_sub_coll)
60
40
20
0
0
1
2
3
4
Mean: 1.17, stdev: 1.02

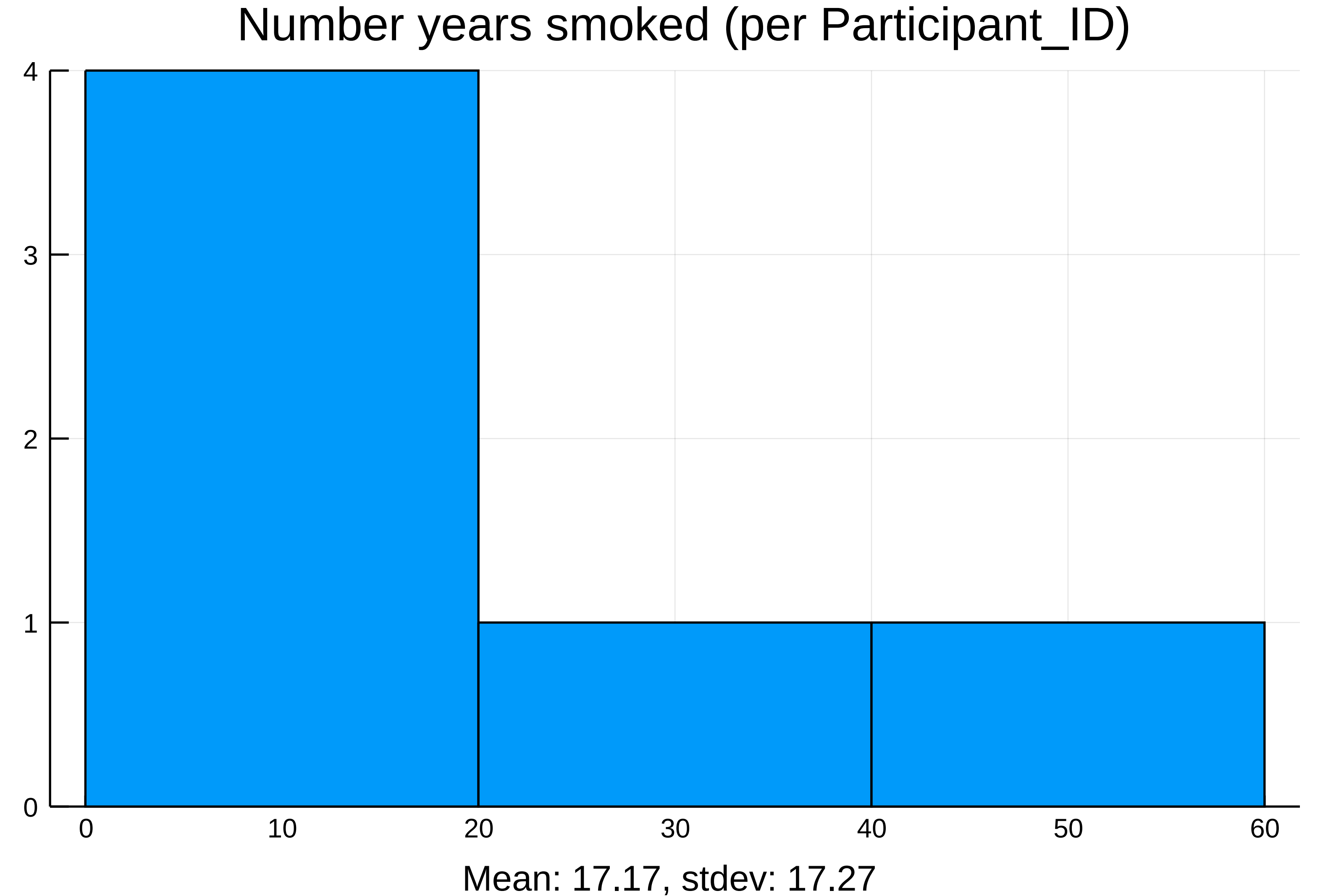
Number years smoked (per Participant_ID)
0
1
2
3
4
0
10
20
30
40
50
60
Mean: 17.17, stdev: 17.27

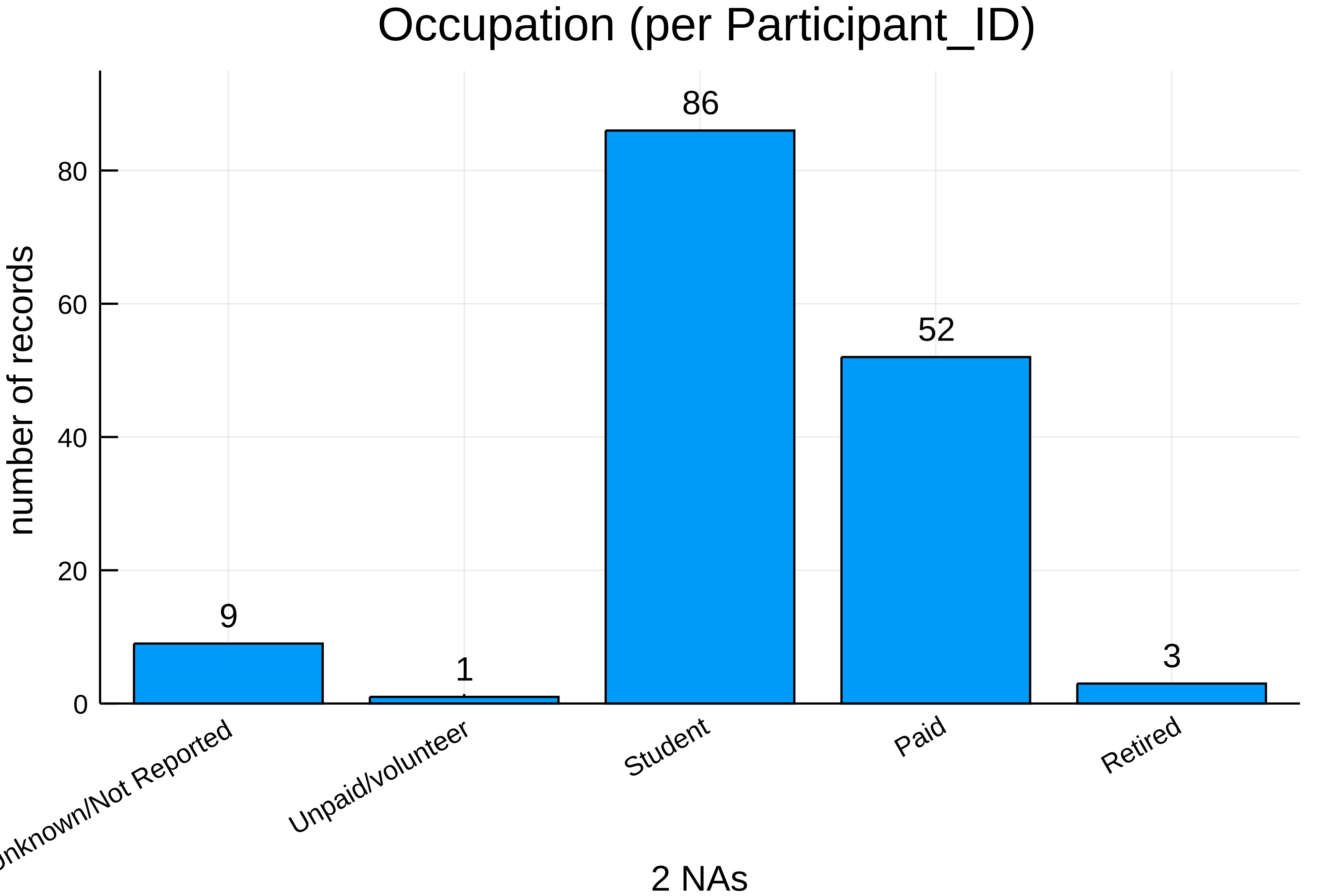

Occupation (per Participant_ID)
number of records
0
20
40
60
80
9
1
86
52
3
Unknown/Not Reported
Unpaid/volunteer
Student
Paid
Retired
2 NAs

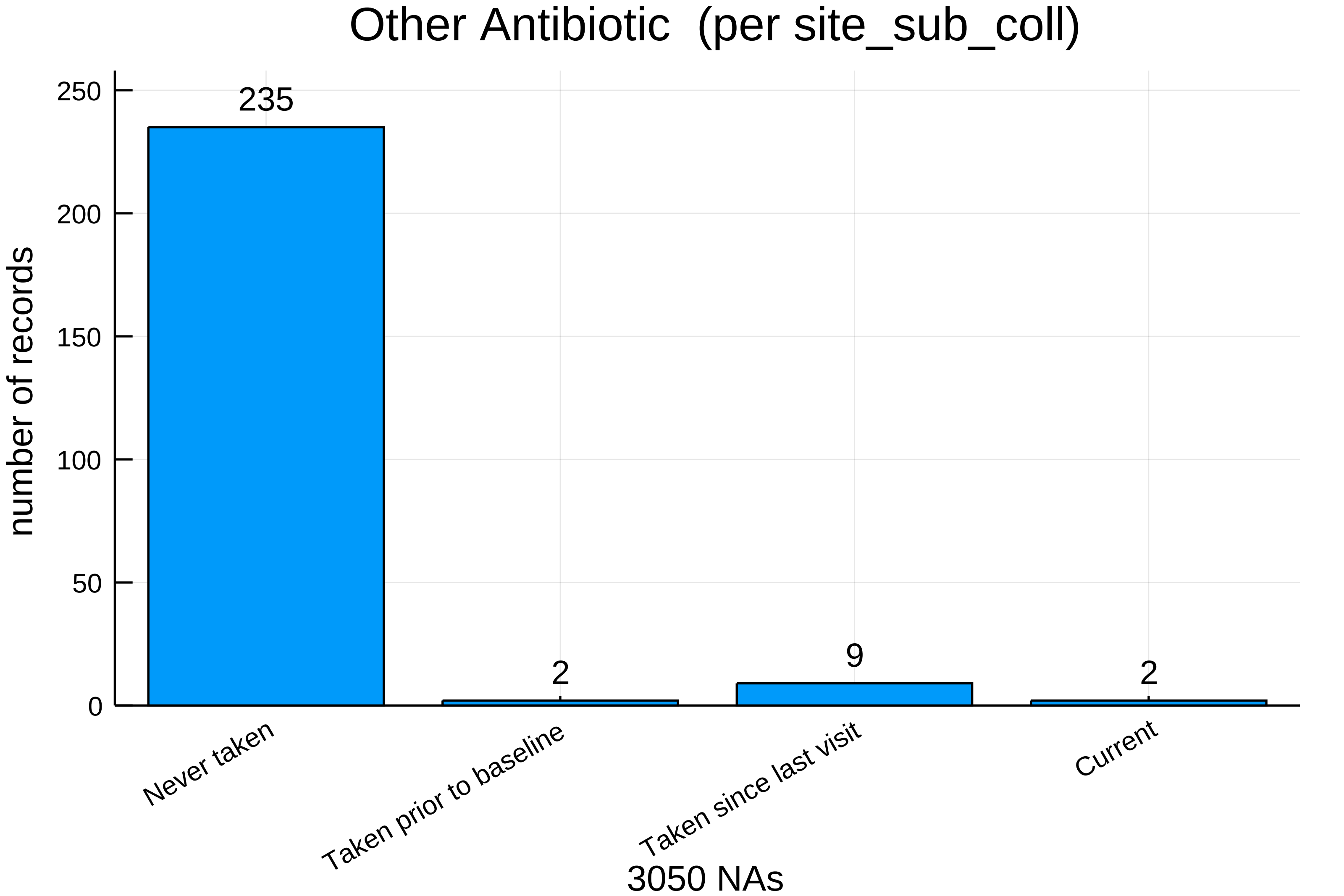
Other Antibiotic (per site_sub_coll)
number of records
250
200
150
100
50
0
235
2
9
2
Never taken
Taken prior to baseline
Taken since last visit
Current
3050 NAs

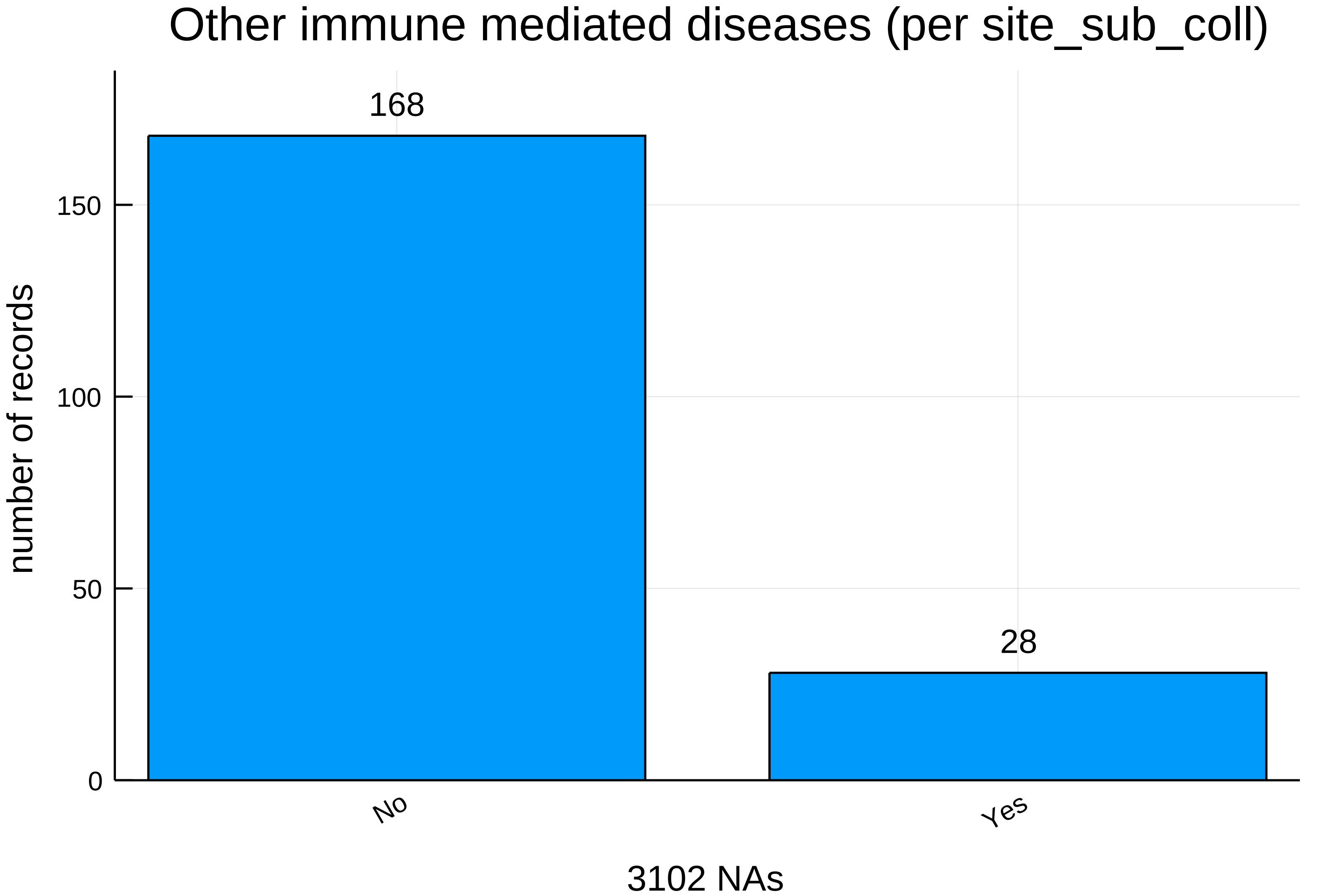
Other immune mediated diseases (per site_sub_coll)
number of records
168
28
150
100
50
0
No
Yes
3102 NAs

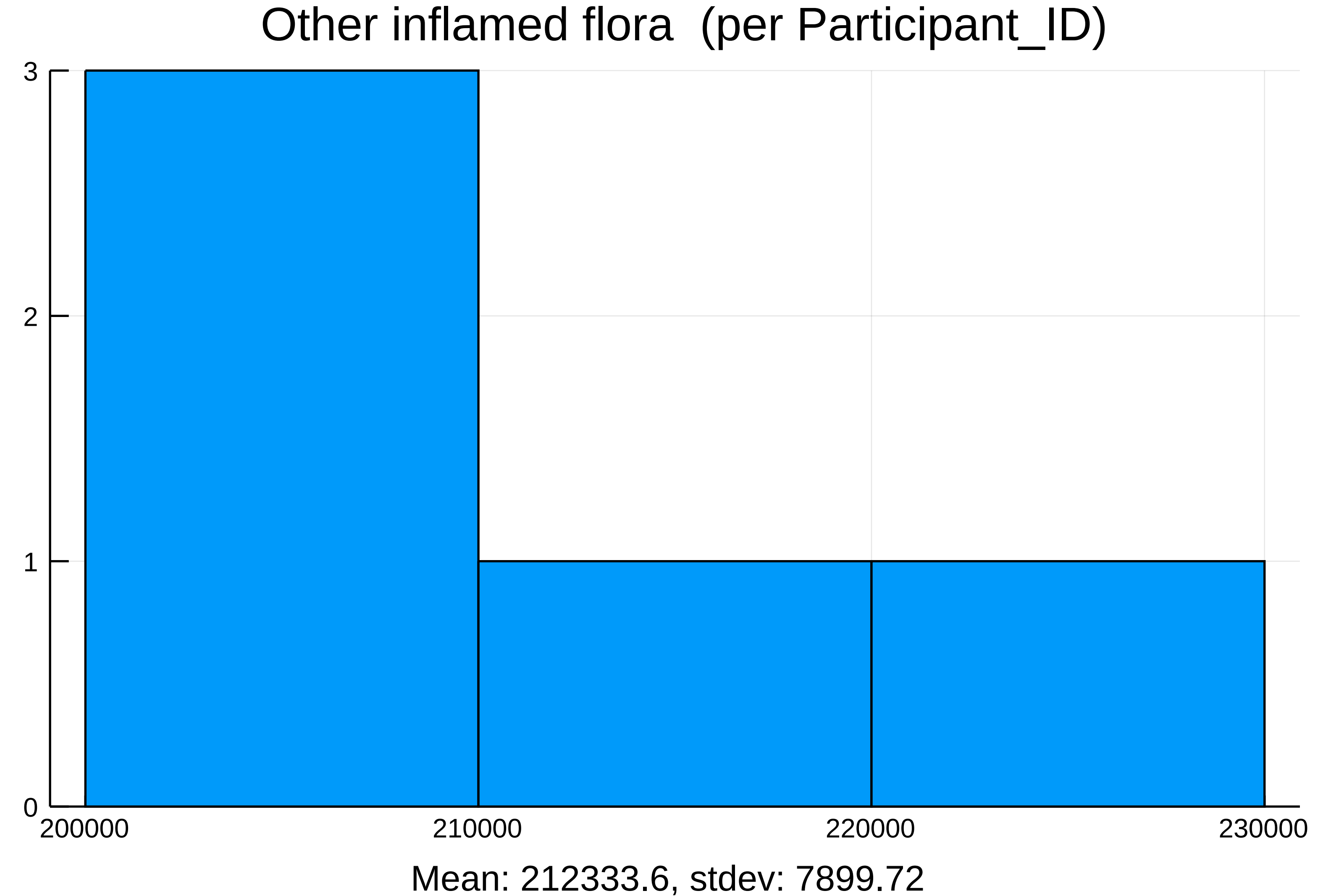
Other inflamed flora (per Participant_ID)
3
2
1
0
200000
210000
220000
230000
Mean: 212333.6, stdev: 7899.72

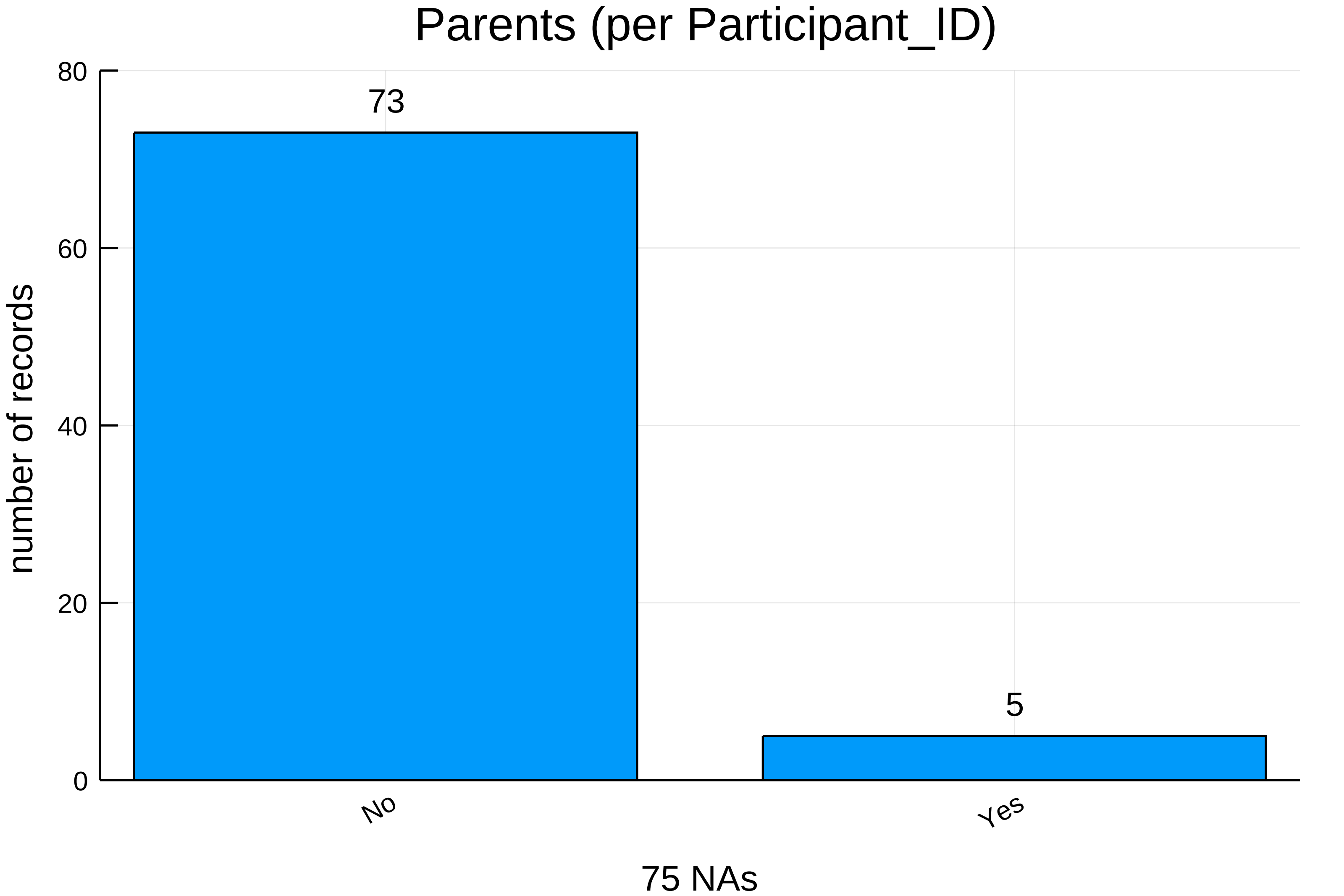
Parents (per Participant_ID)
number of records
80
60
40
20
0
73
5
No
Yes
75 NAs

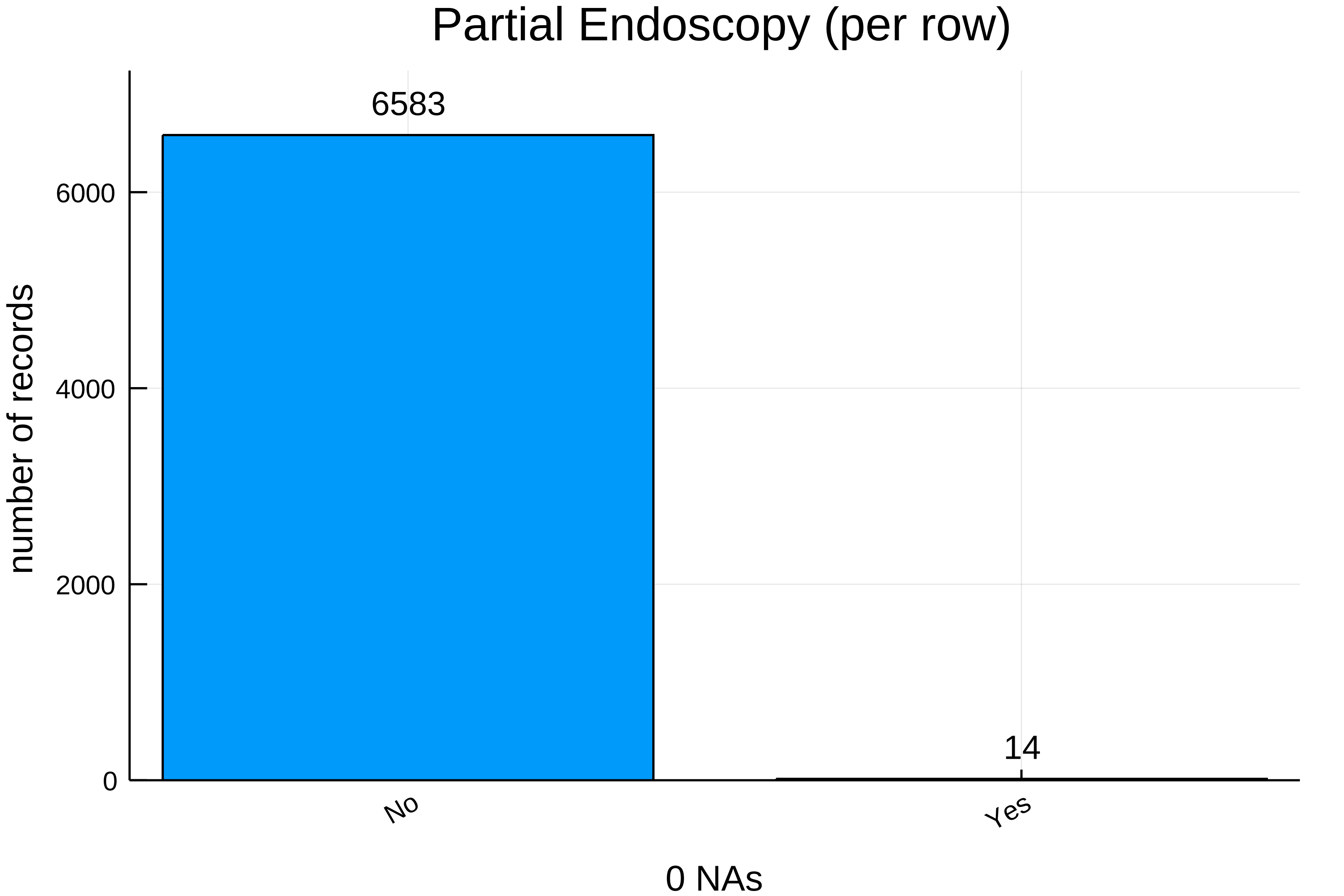
Partial Endoscopy (per row)
number of records
6583
14
0
2000
4000
6000
No
Yes
0 NAs

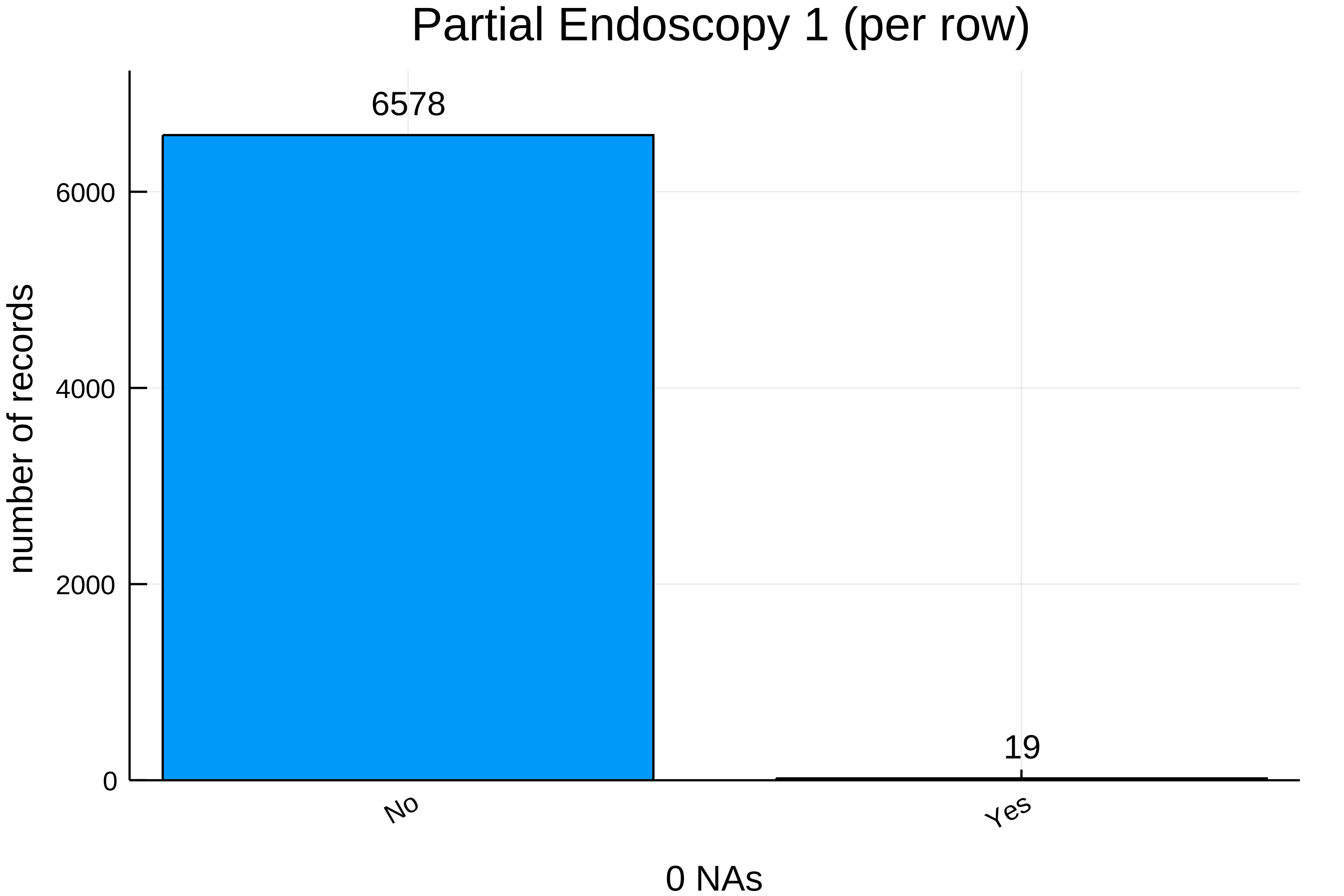
Partial Endoscopy 1 (per row)
number of records
6578
19
6000
4000
2000
0
No
Yes
0 NAs

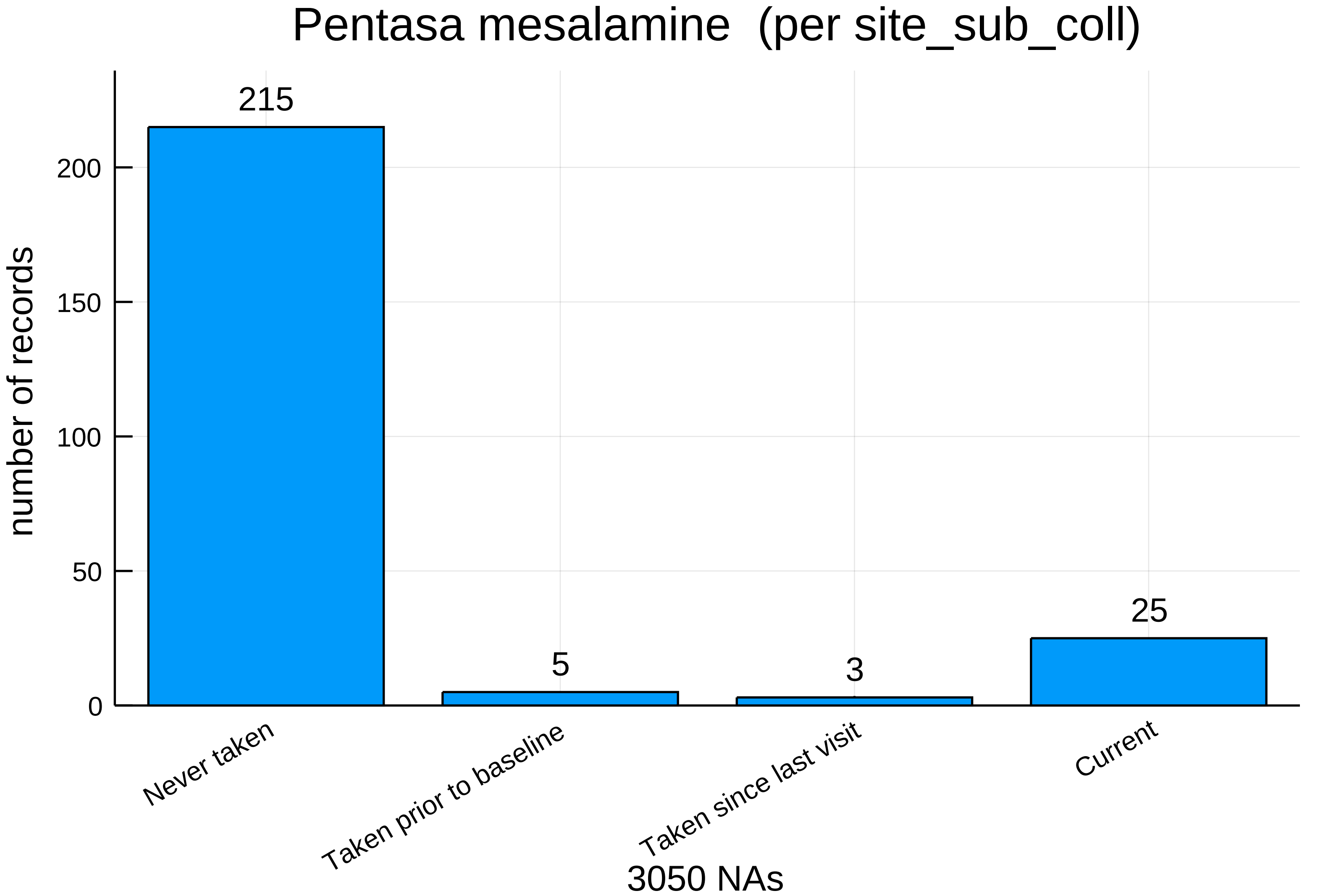
Pentasa mesalamine (per site_sub_coll)
number of records
215
5
3
25
0
50
100
150
200
Never taken
Taken prior to baseline
Taken since last visit
Current
3050 NAs

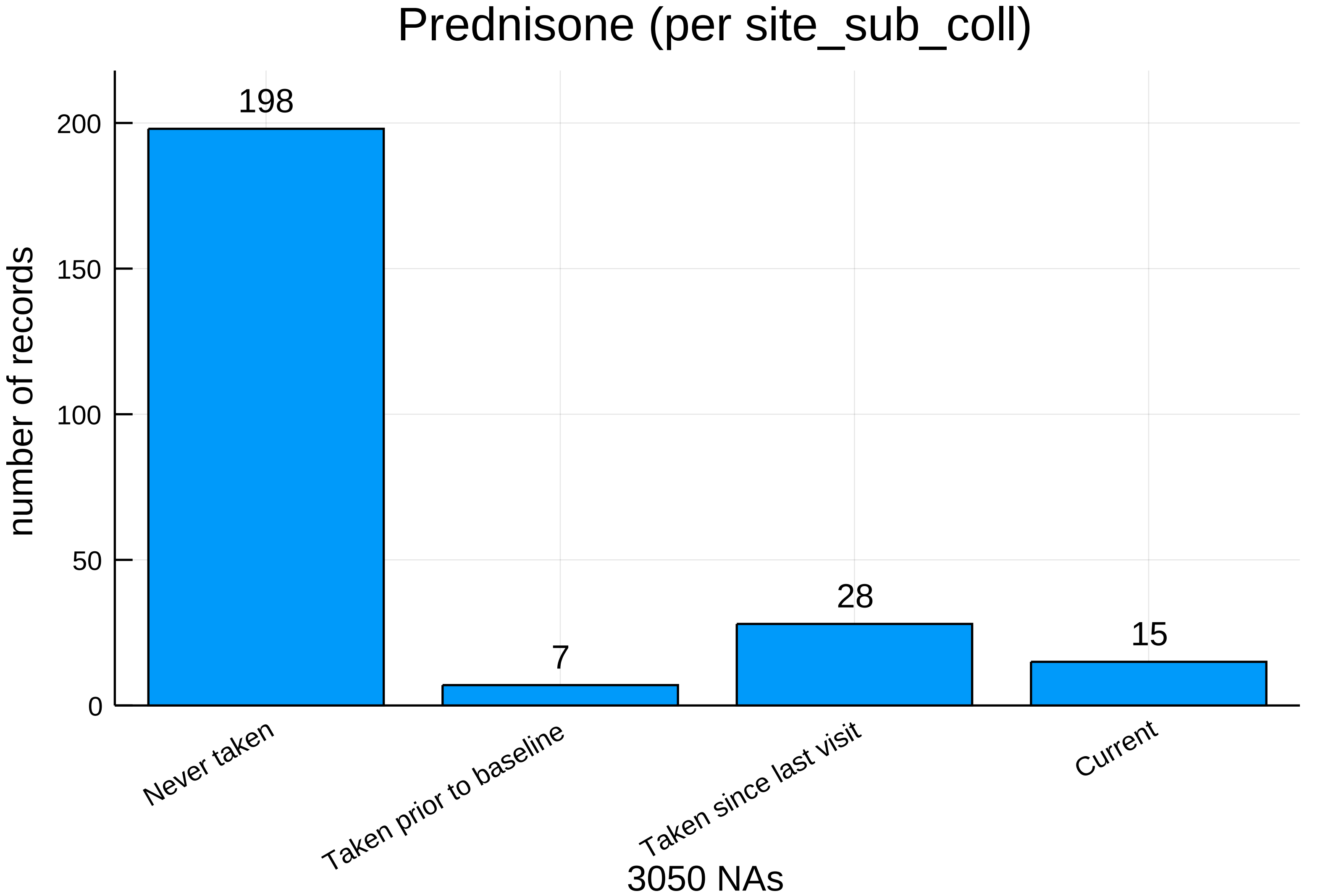

Prednisone (per site_sub_coll)
number of records
200
150
100
50
0
198
7
28
15
Never taken
Taken prior to baseline
Taken since last visit
Current
3050 NAs

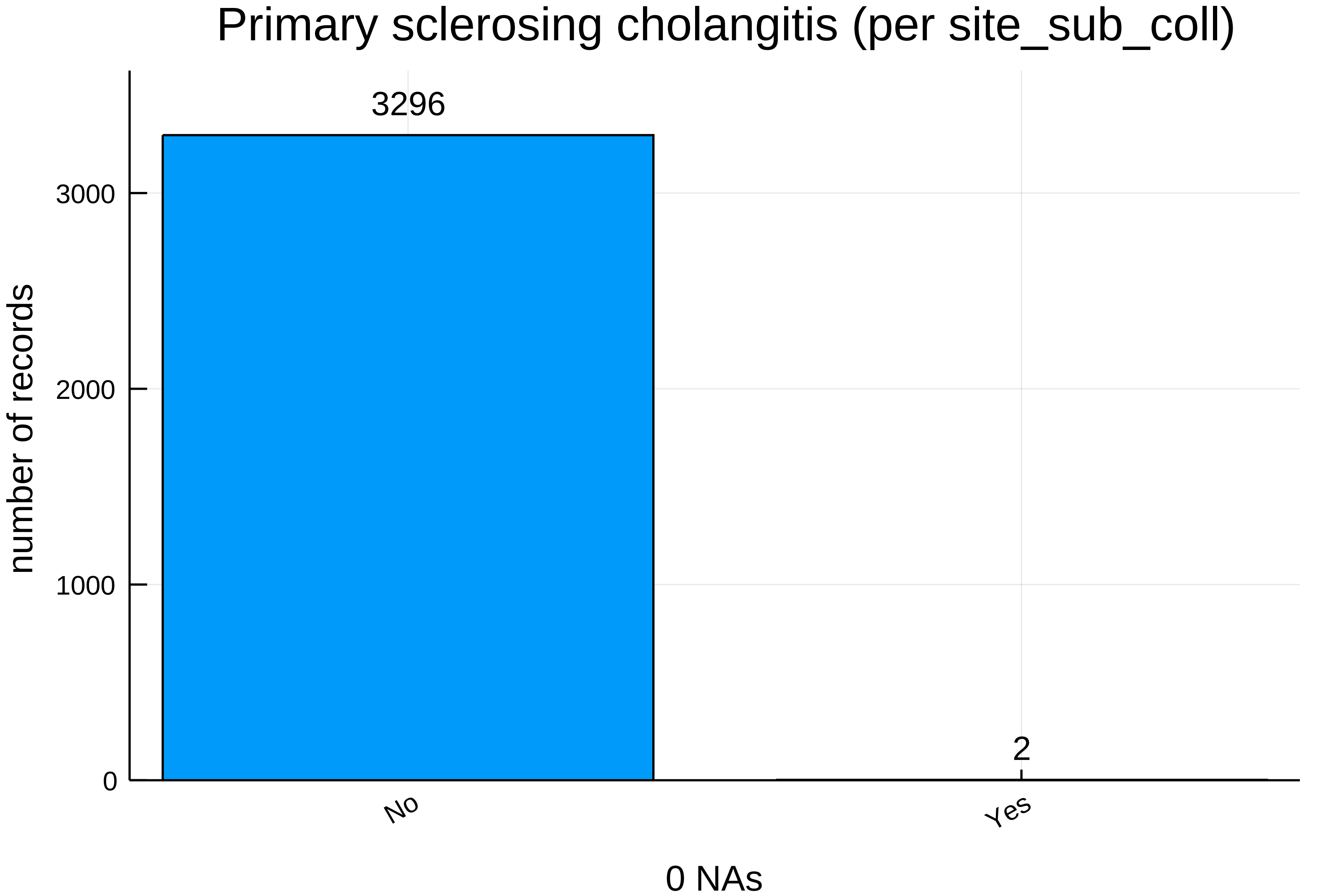

Primary sclerosing cholangitis (per site_sub_coll)
number of records
3296
2
3000
2000
1000
0
No
Yes
0 NAs

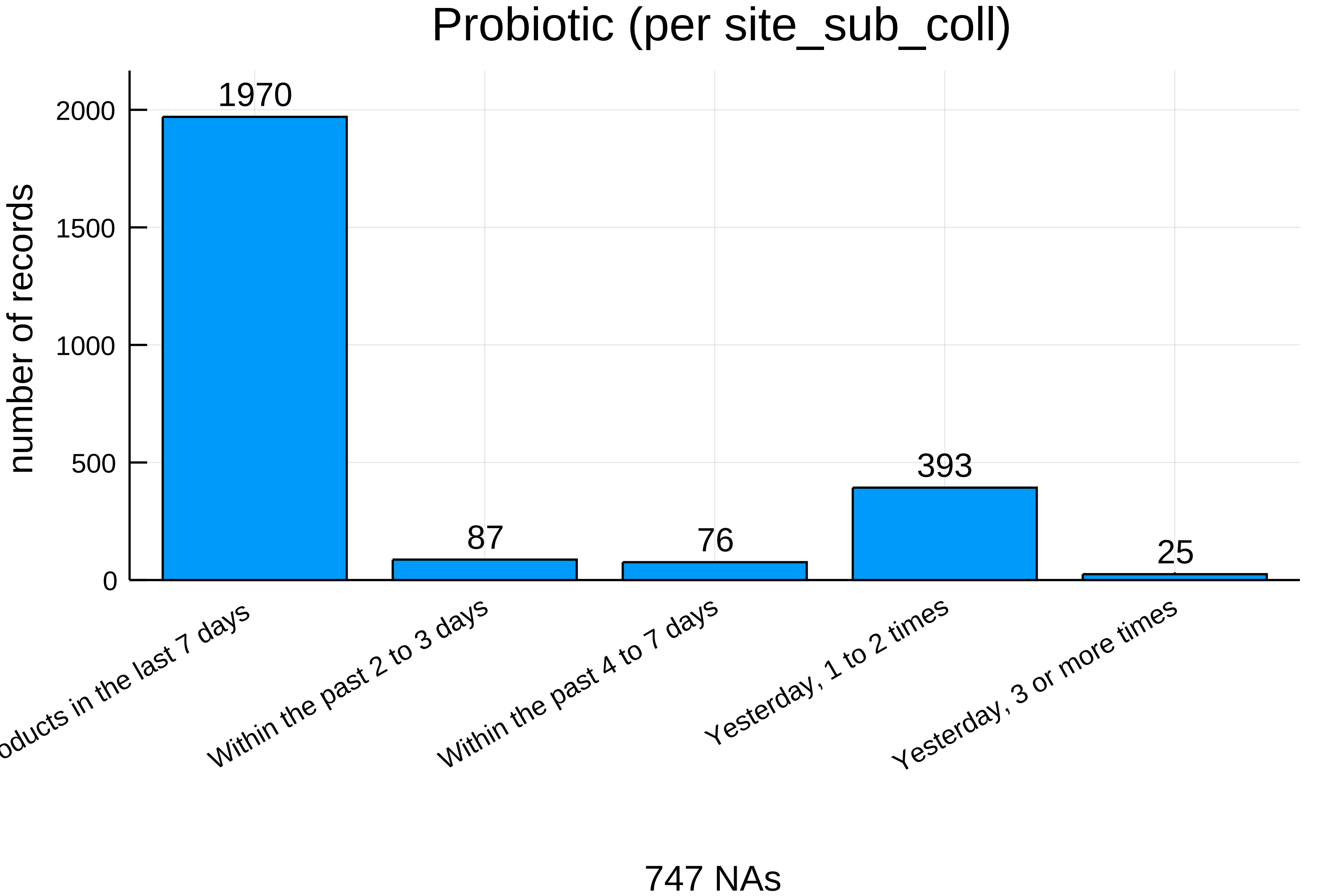
Probiotic (per site_sub_coll)
number of records
2000
1500
1000
500
0
1970
87
76
393
25
oducts in the last 7 days
Within the past 2 to 3 days
Within the past 4 to 7 days
Yesterday, 1 to 2 times
Yesterday, 3 or more times
747 NAs

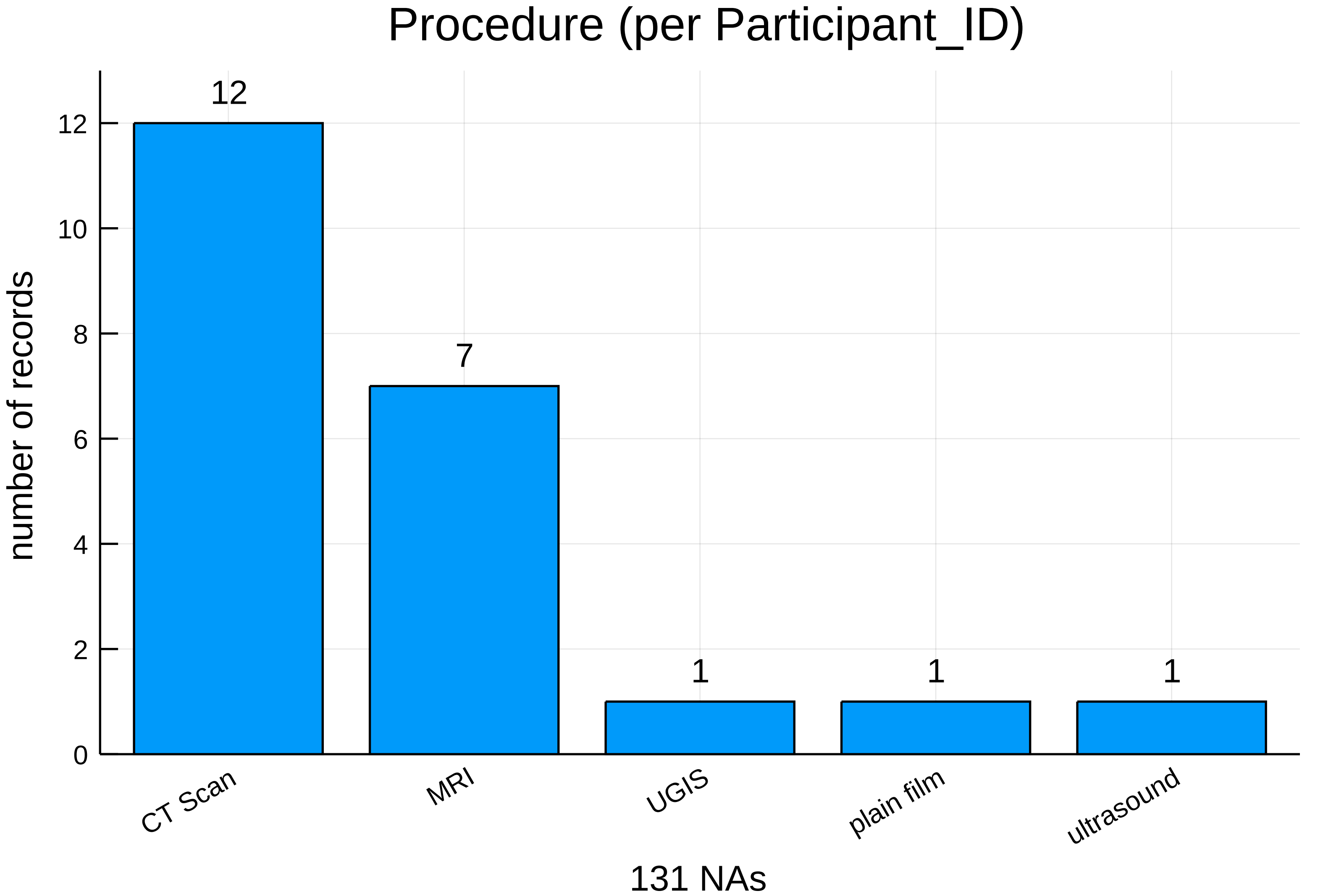

Procedure (per Participant_ID)
number of records
0
2
4
6
8
10
12
12
7
1
1
1
CT Scan
MRI
UGIS
plain film
ultrasound
131 NAs

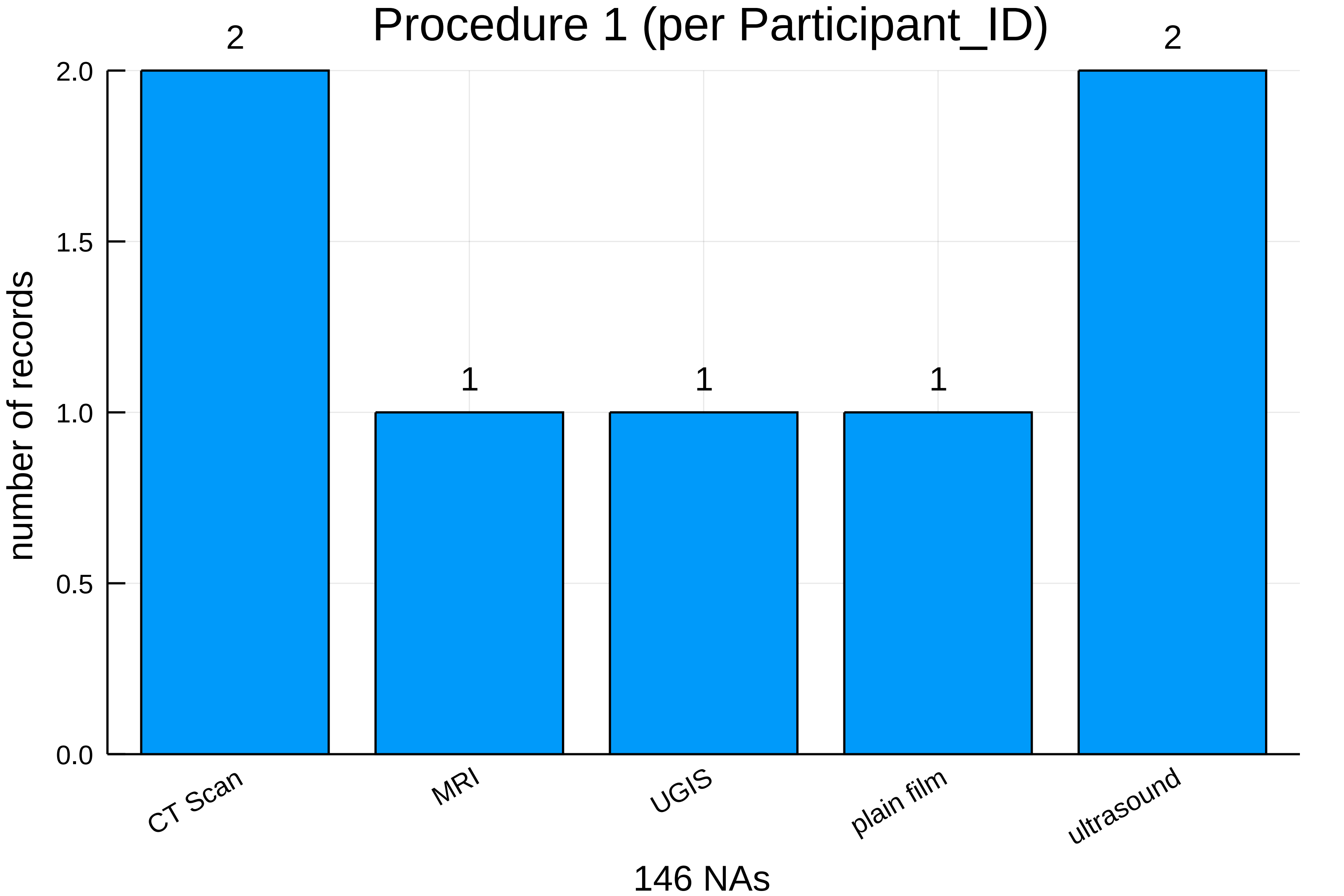
Procedure 1 (per Participant_ID)
2
1
1
1
2
2.0
1.5
1.0
0.5
0.0
number of records
CT Scan
MRI
UGIS
plain film
ultrasound
146 NAs

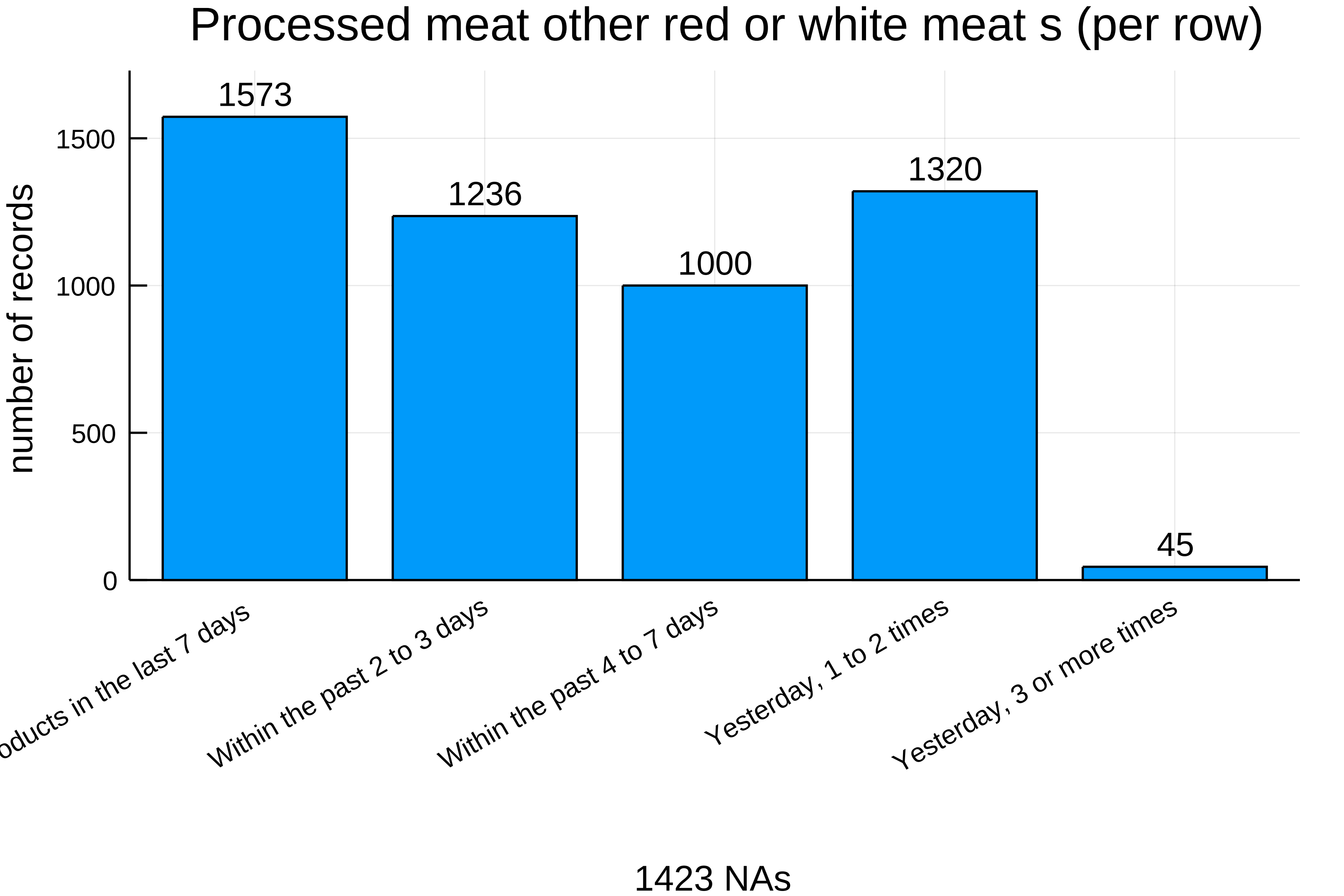

Processed meat other red or white meat s (per row)
number of records
0
500
1000
1500
1573
1236
1000
1320
45
oducts in the last 7 days
Within the past 2 to 3 days
Within the past 4 to 7 days
Yesterday, 1 to 2 times
Yesterday, 3 or more times
1423 NAs

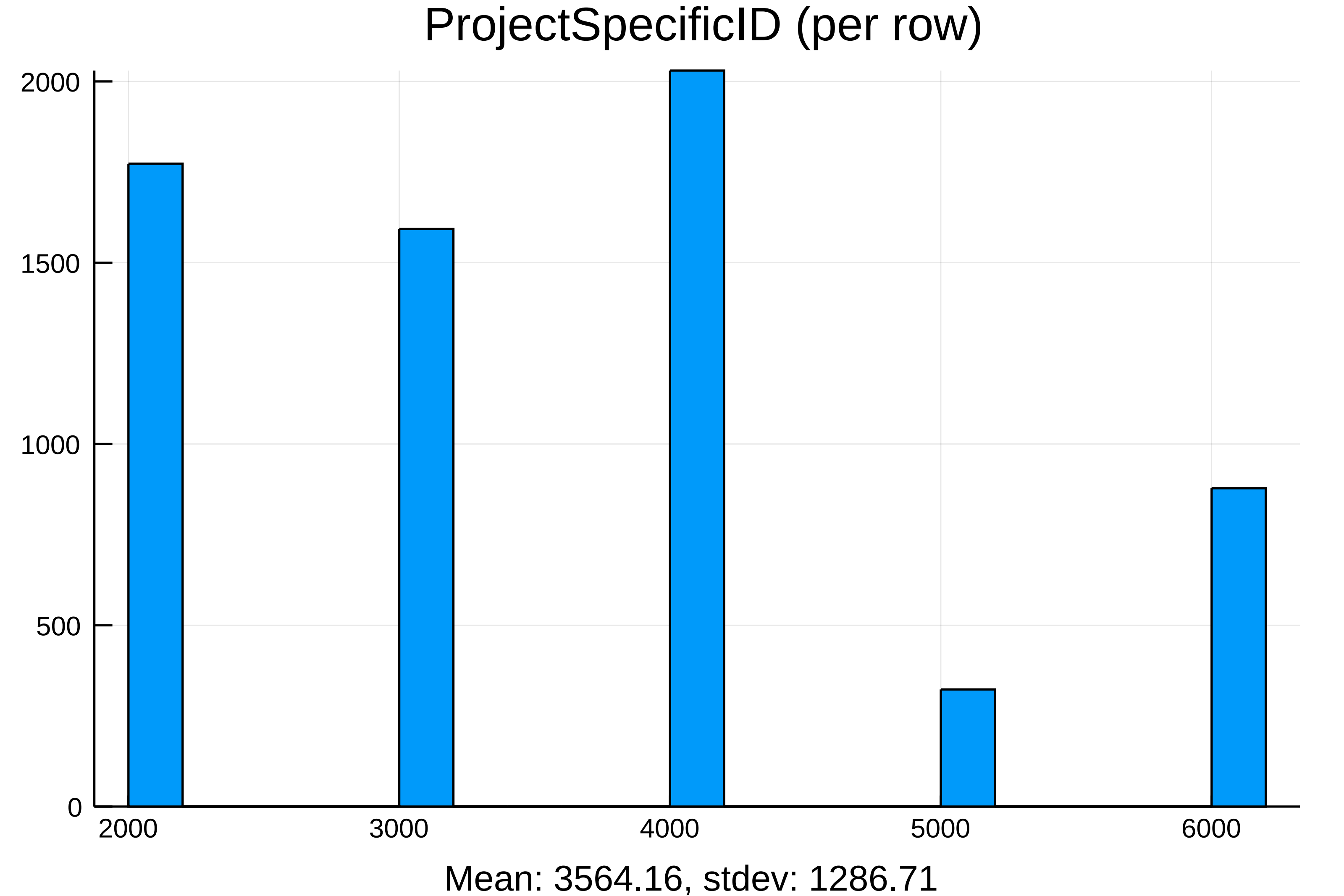
ProjectSpecificID (per row)
2000
1500
1000
500
0
2000
3000
4000
5000
6000
Mean: 3564.16, stdev: 1286.71

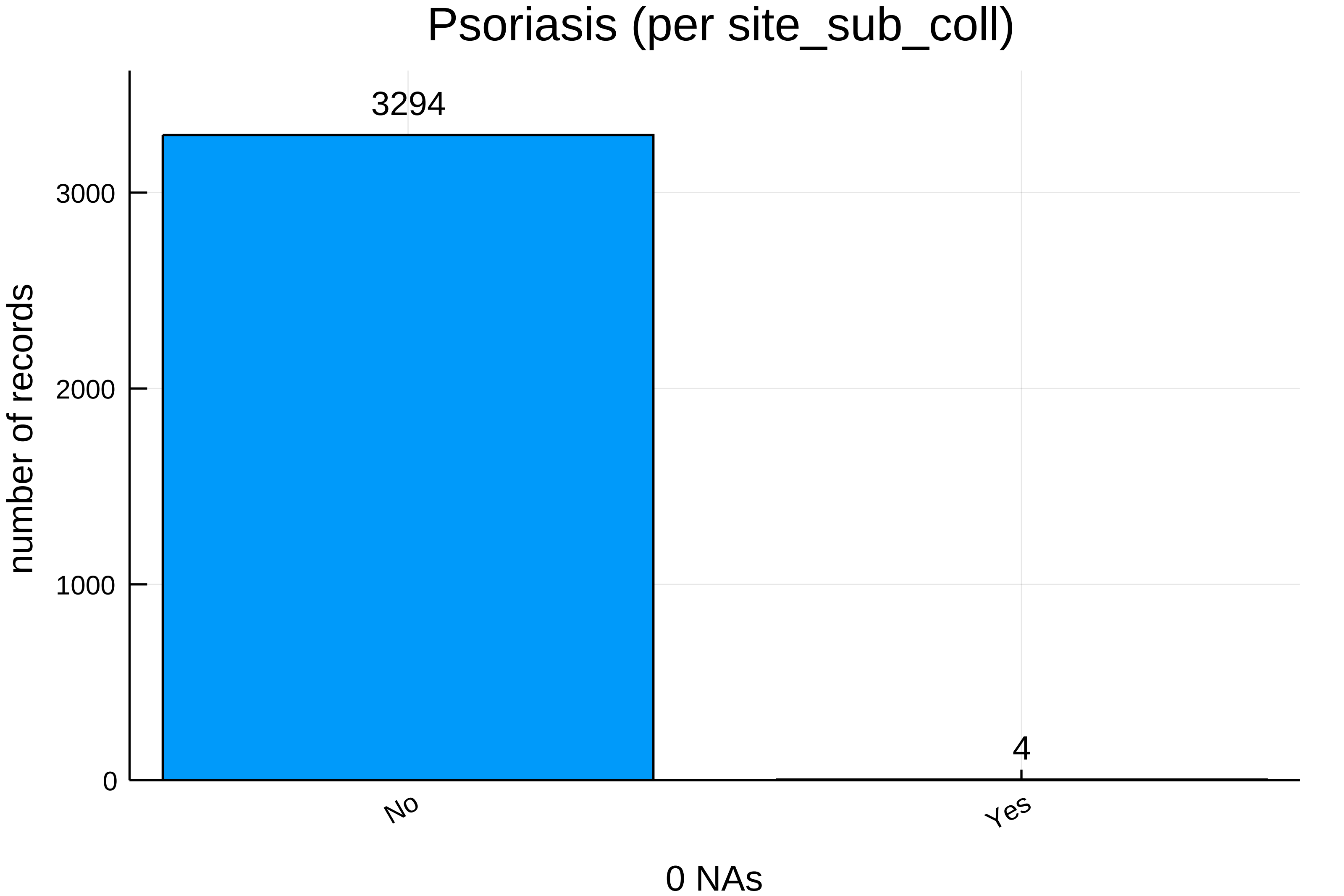
Psoriasis (per site_sub_coll)
number of records
3294
4
3000
2000
1000
0
No
Yes
0 NAs

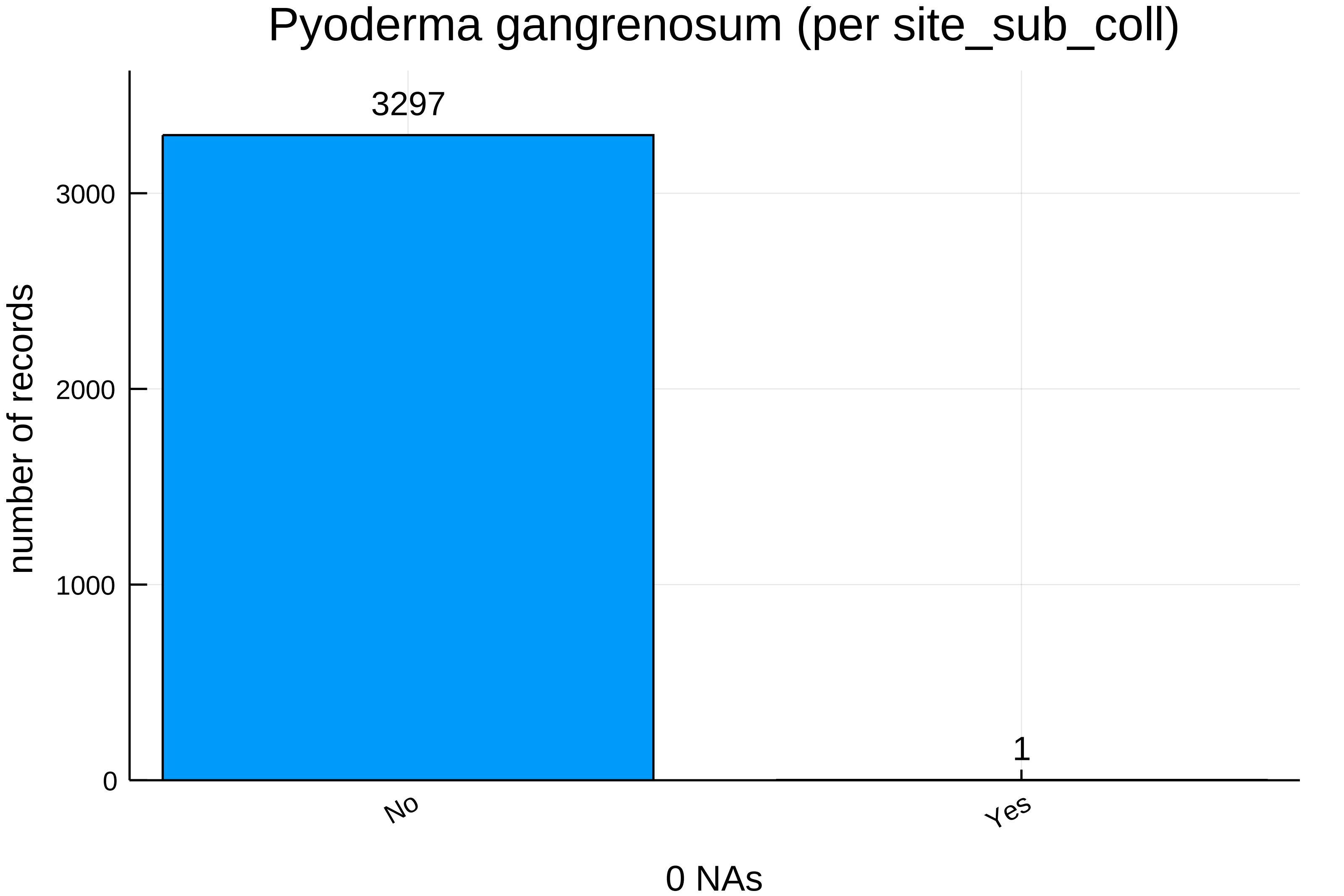
Pyoderma gangrenosum (per site_sub_coll)
number of records
0
1000
2000
3000
3297
1
No
Yes
0 NAs

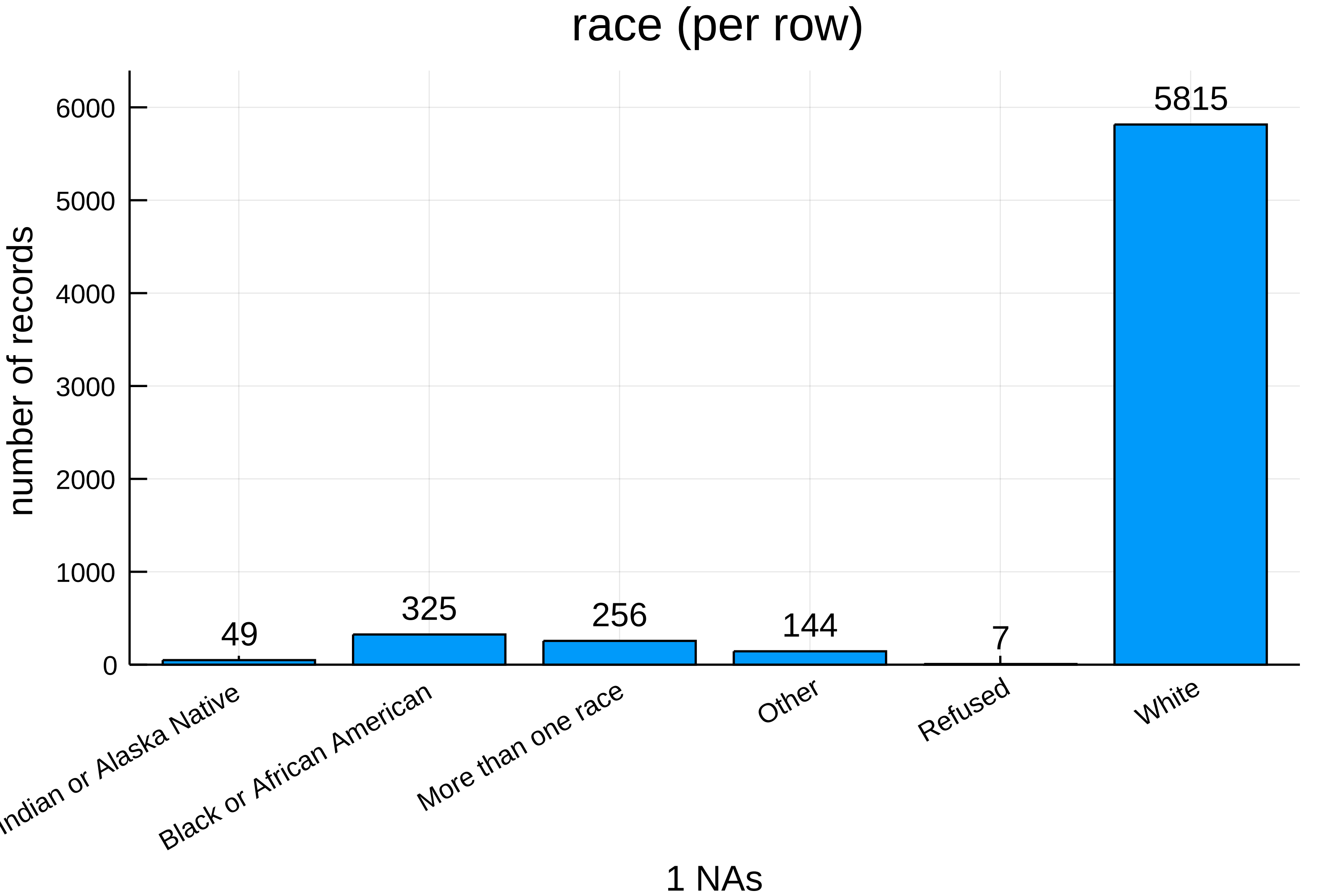
race (per row)
number of records
6000
5000
4000
3000
2000
1000
0
49
325
256
144
7
5815
Indian or Alaska Native
Black or African American
More than one race
Other
Refused
White
1 NAs

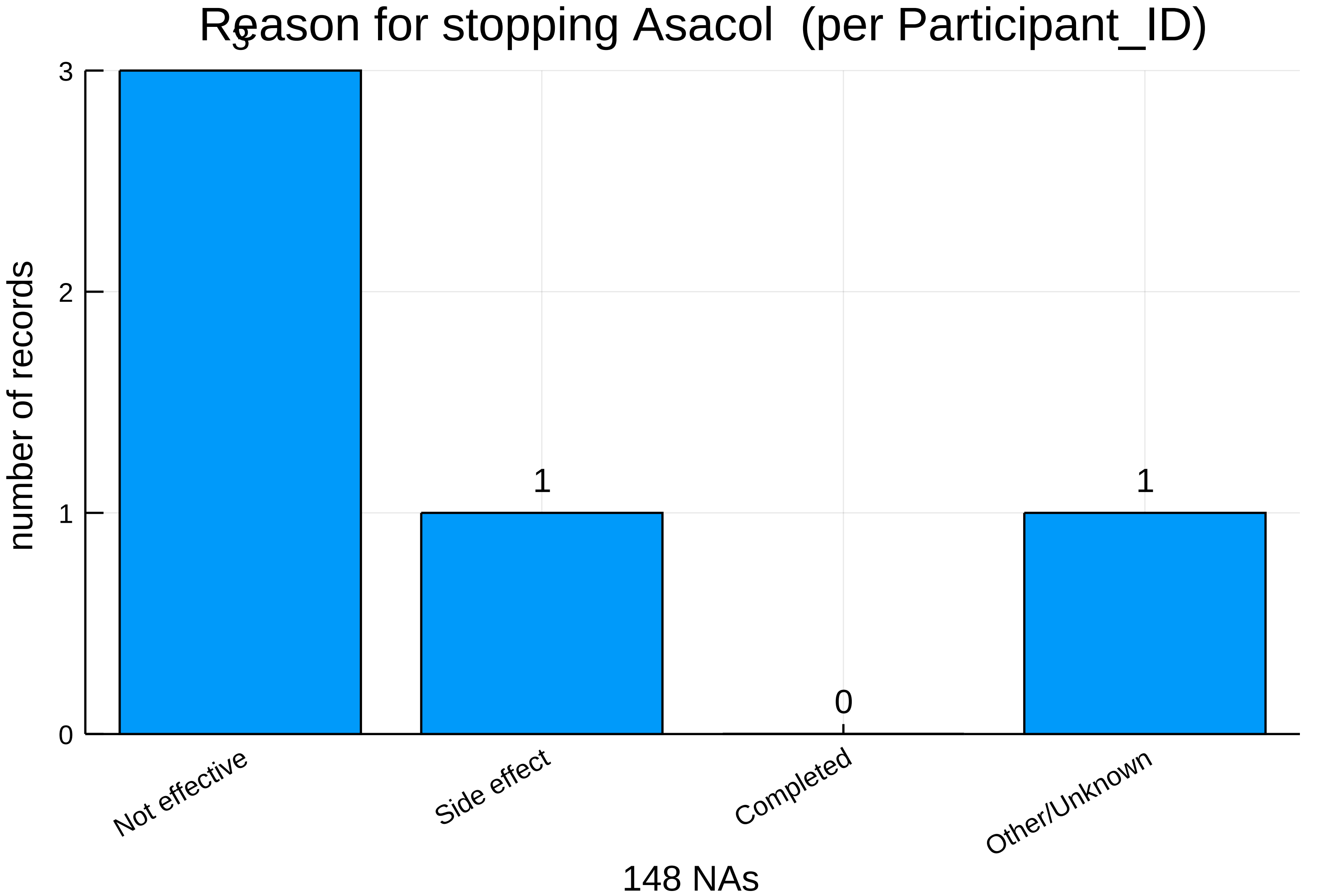
Reason for stopping Asacol (per Participant_ID)
number of records
3
1
0
1
3
2
1
0
Not effective
Side effect
Completed
Other/Unknown
148 NAs

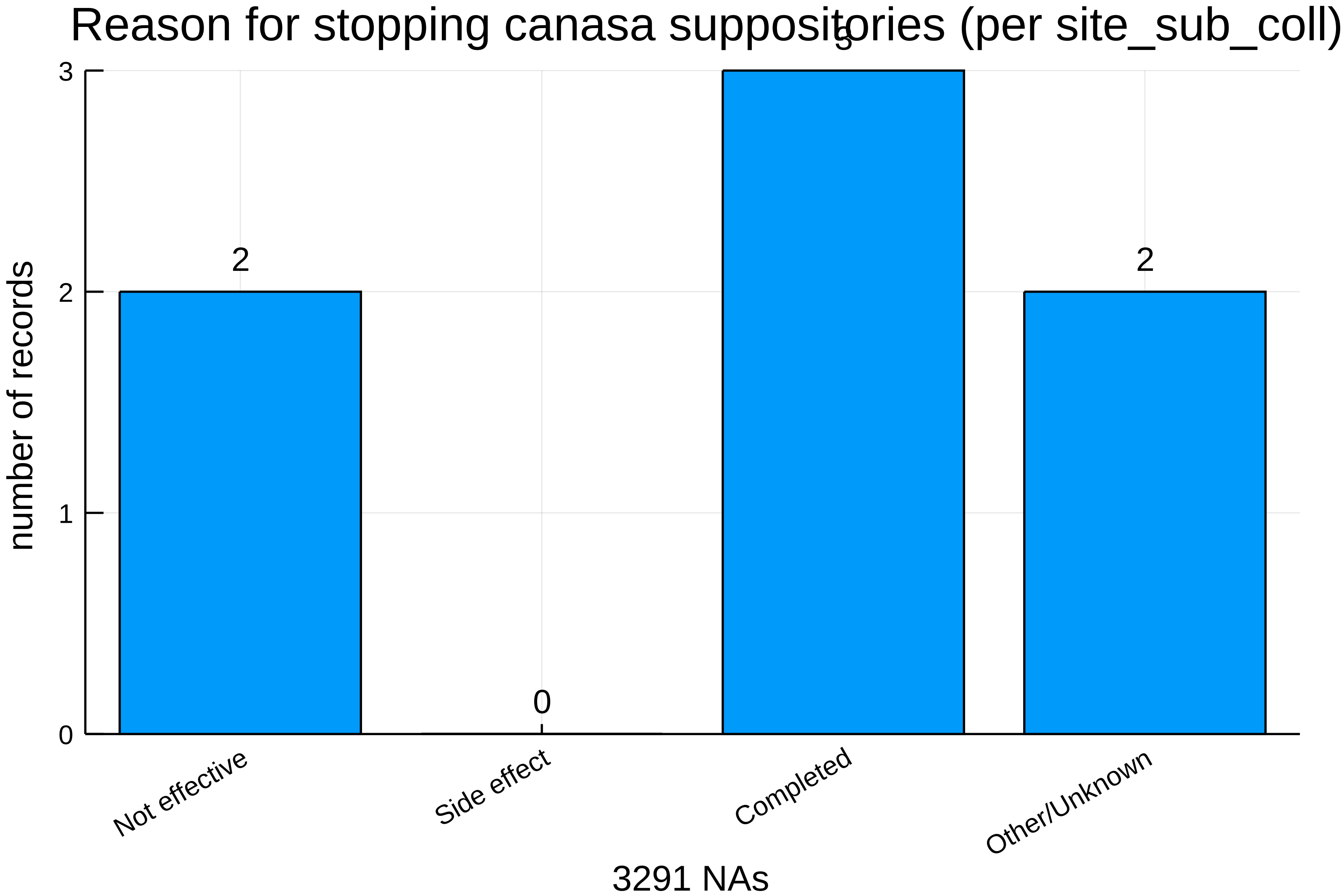
Reason for stopping canasa suppositories (per site_sub_coll)
number of records
3
2
1
0
2
0
3
2
Not effective
Side effect
Completed
Other/Unknown
3291 NAs

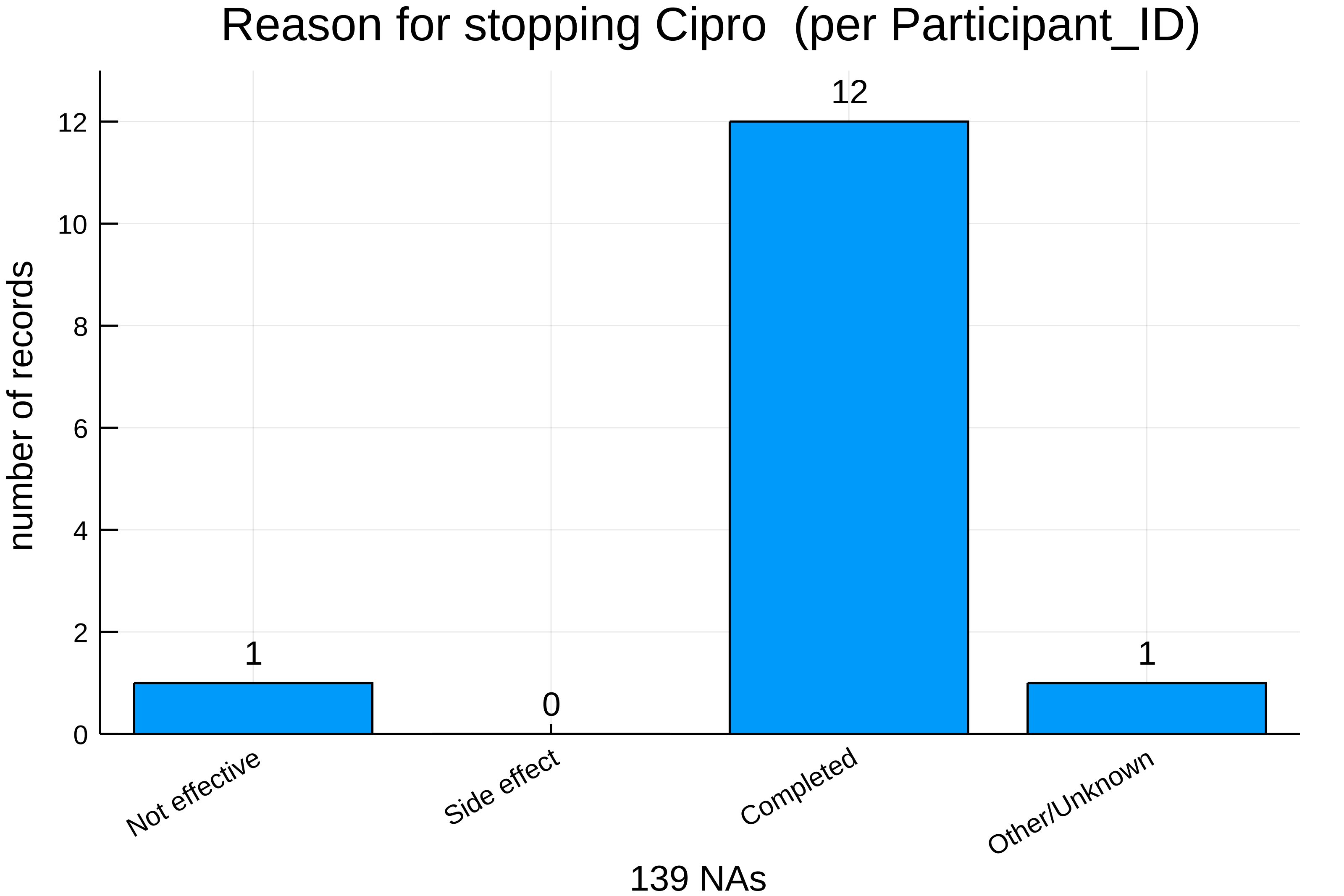
Reason for stopping Cipro (per Participant_ID)
number of records
0
2
4
6
8
10
12
1
0
12
1
Not effective
Side effect
Completed
Other/Unknown
139 NAs

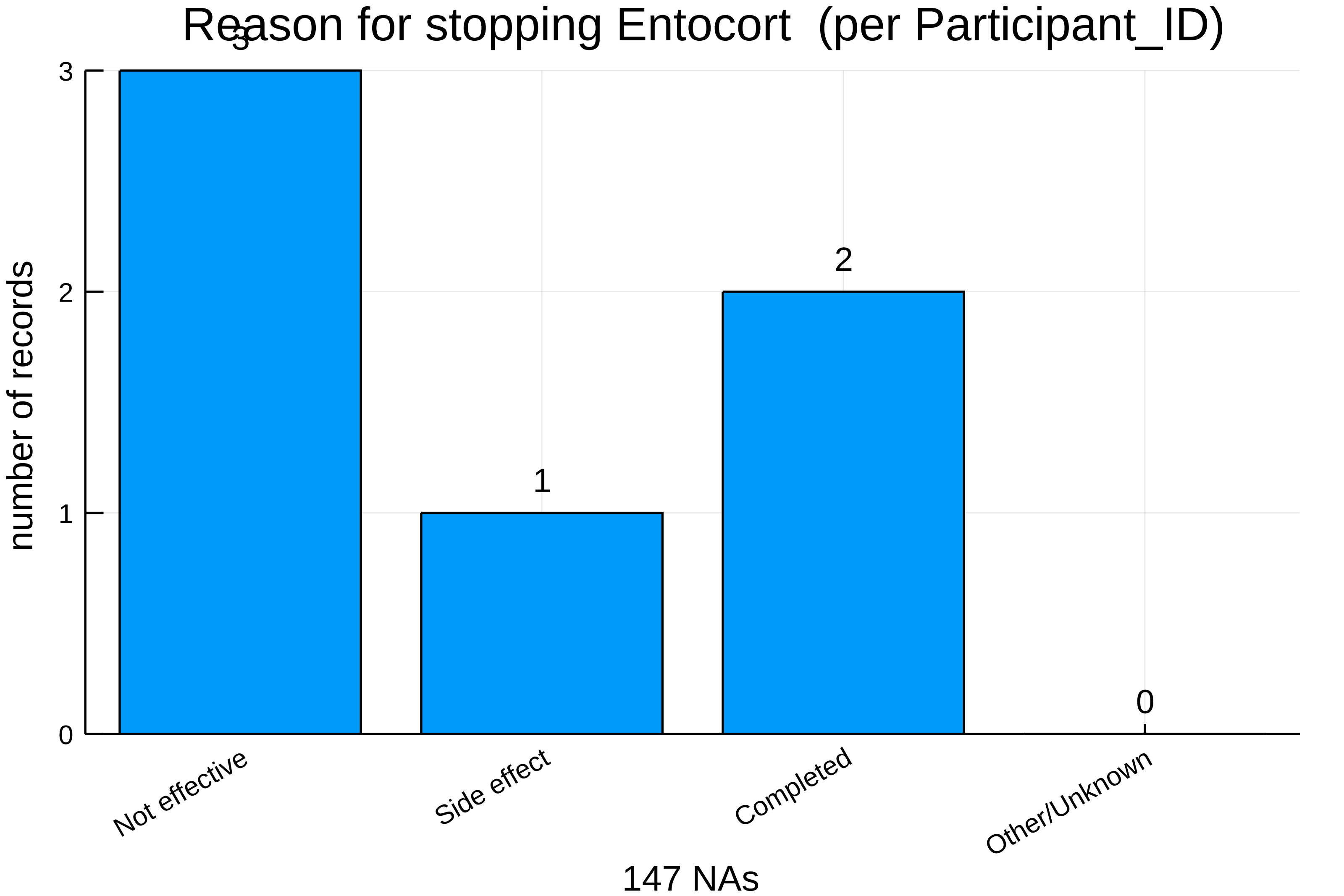
Reason for stopping Entocort (per Participant_ID)
number of records
3
2
1
0
3
1
2
0
Not effective
Side effect
Completed
Other/Unknown
147 NAs

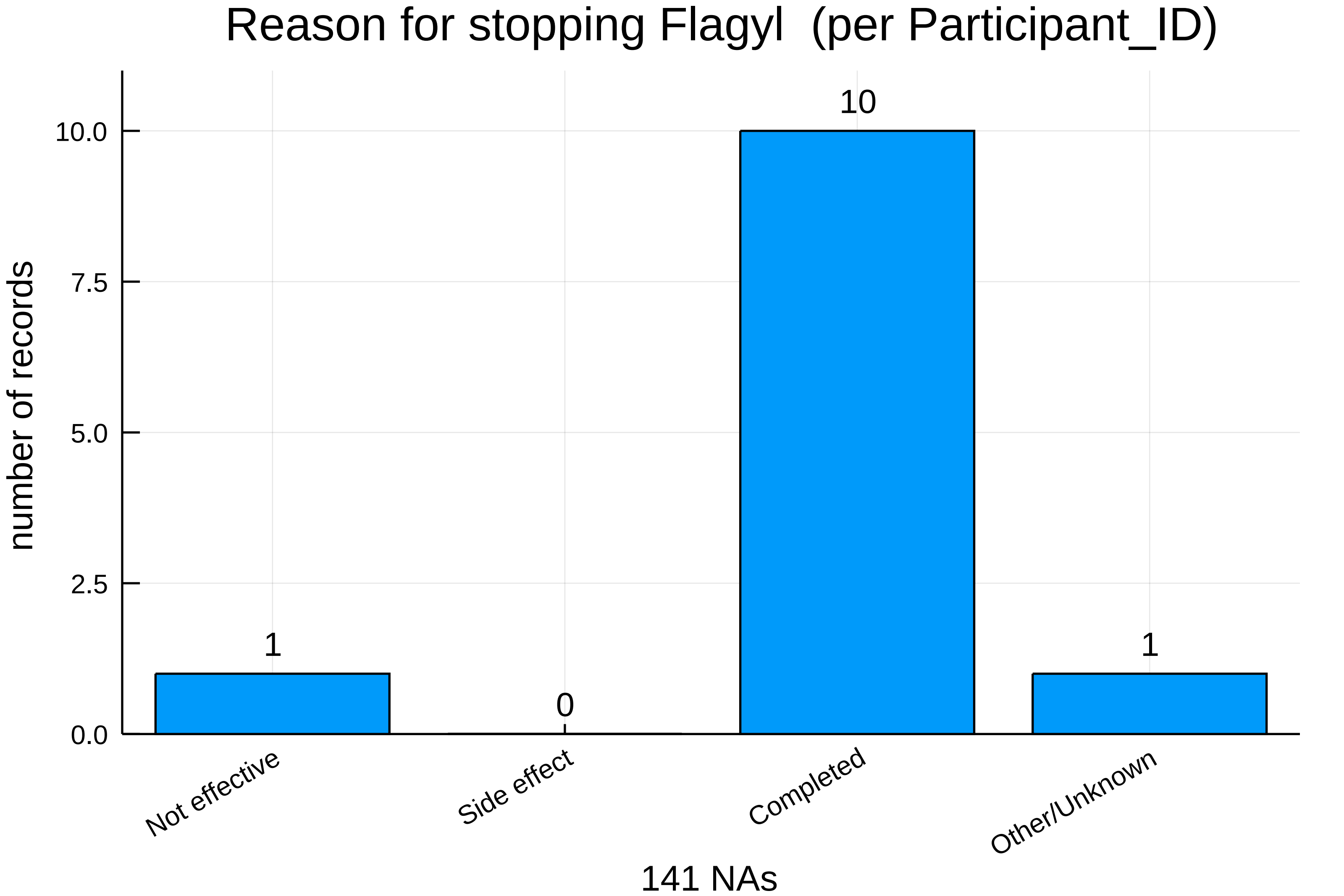
Reason for stopping Flagyl (per Participant_ID)
number of records
10.0
7.5
5.0
2.5
0.0
1
0
10
1
Not effective
Side effect
Completed
Other/Unknown
141 NAs

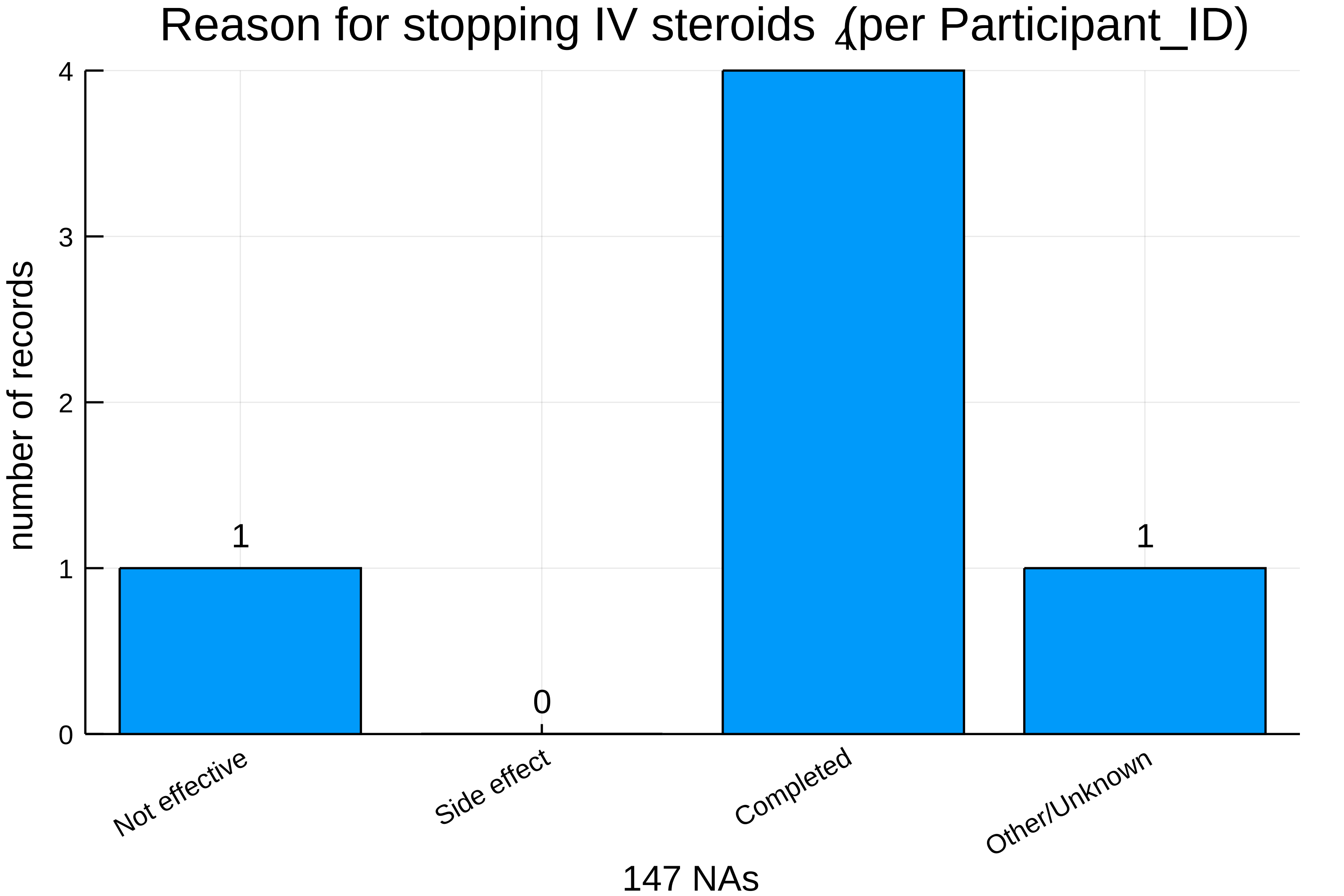
Reason for stopping IV steroids (per Participant_ID)
number of records
0
1
2
3
4
1
0
4
1
Not effective
Side effect
Completed
Other/Unknown
147 NAs

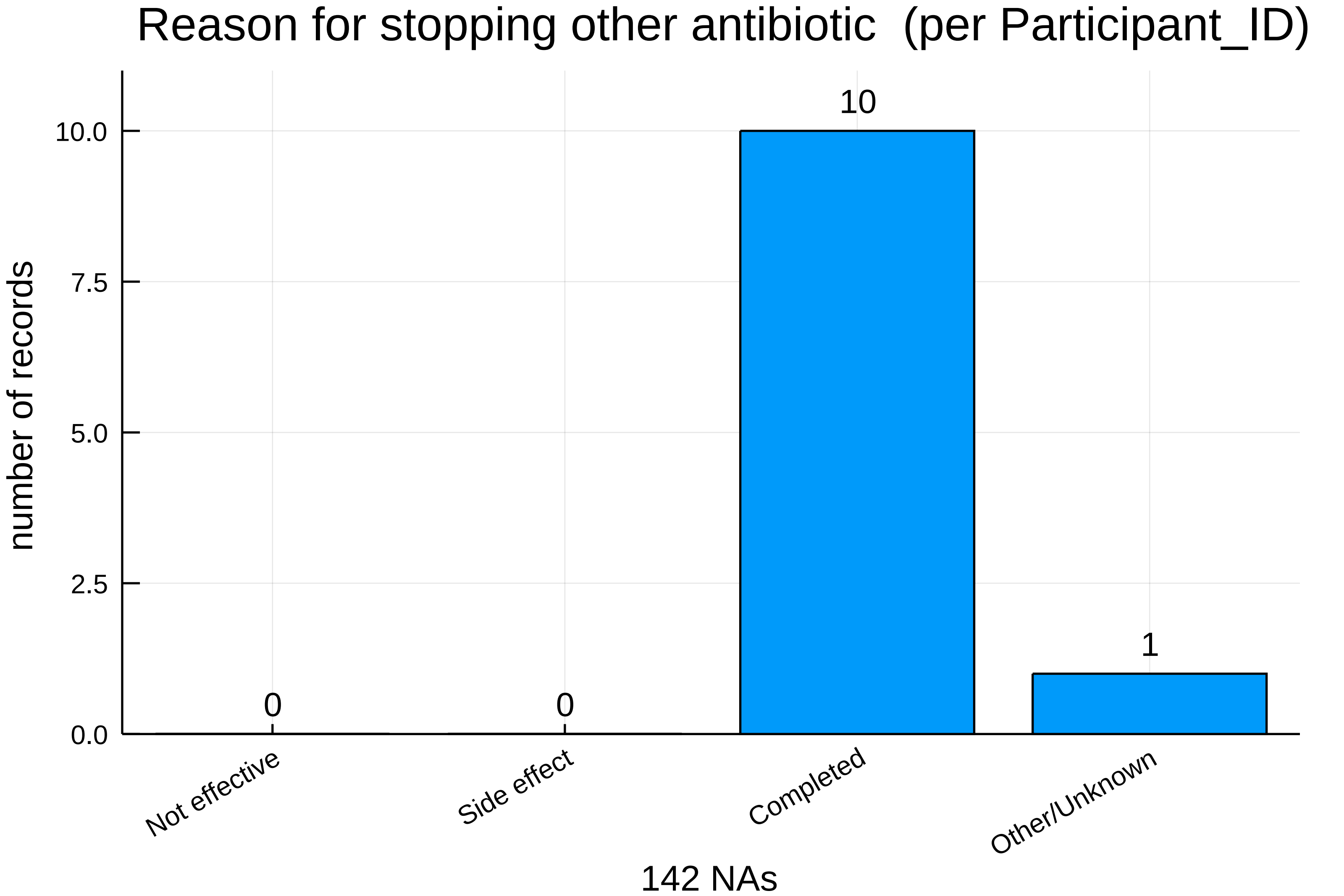
Reason for stopping other antibiotic (per Participant_ID)
number of records
0.0
2.5
5.0
7.5
10.0
0
0
10
1
Not effective
Side effect
Completed
Other/Unknown
142 NAs

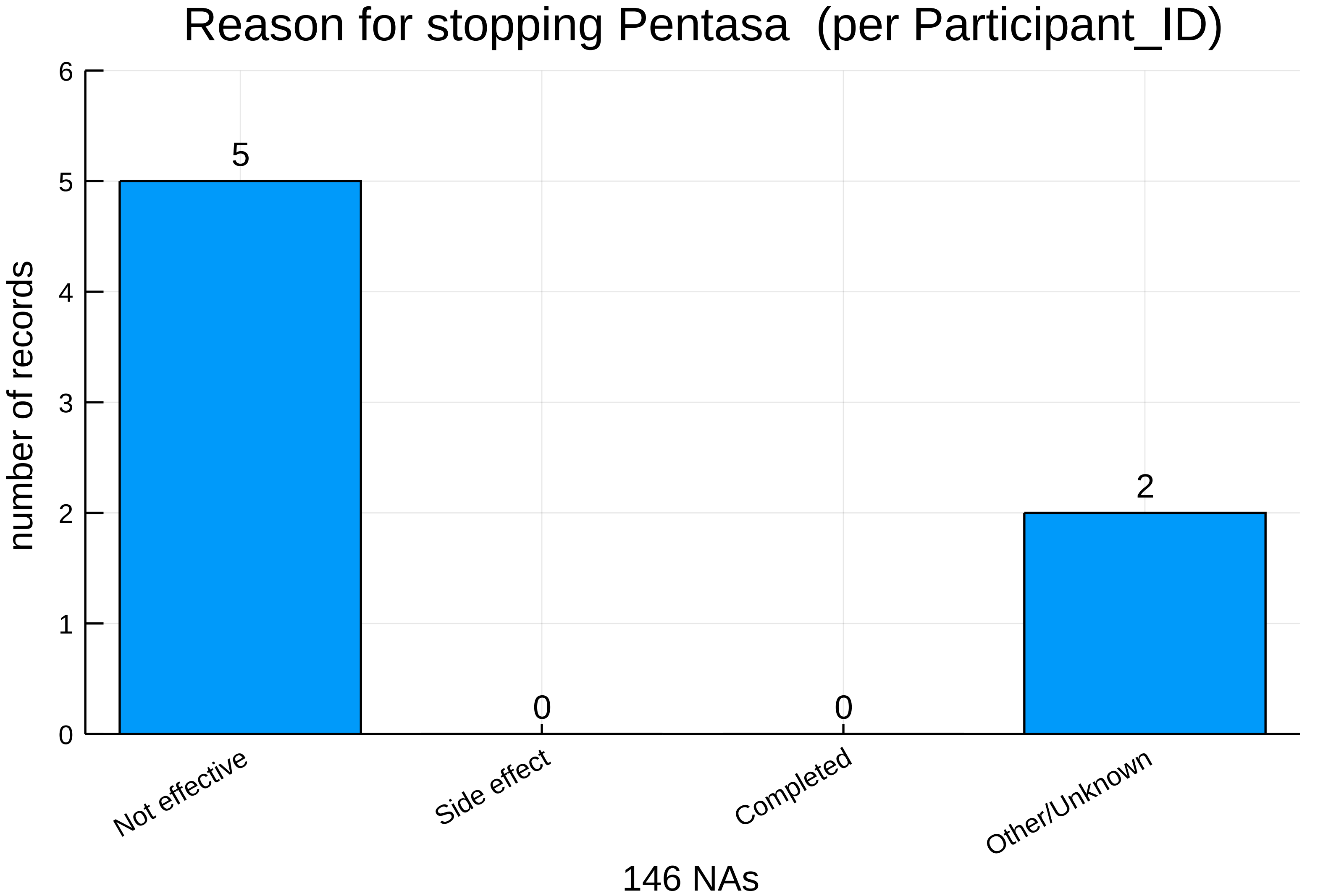
Reason for stopping Pentasa (per Participant_ID)
number of records
0
1
2
3
4
5
6
5
0
0
2
Not effective
Side effect
Completed
Other/Unknown
146 NAs

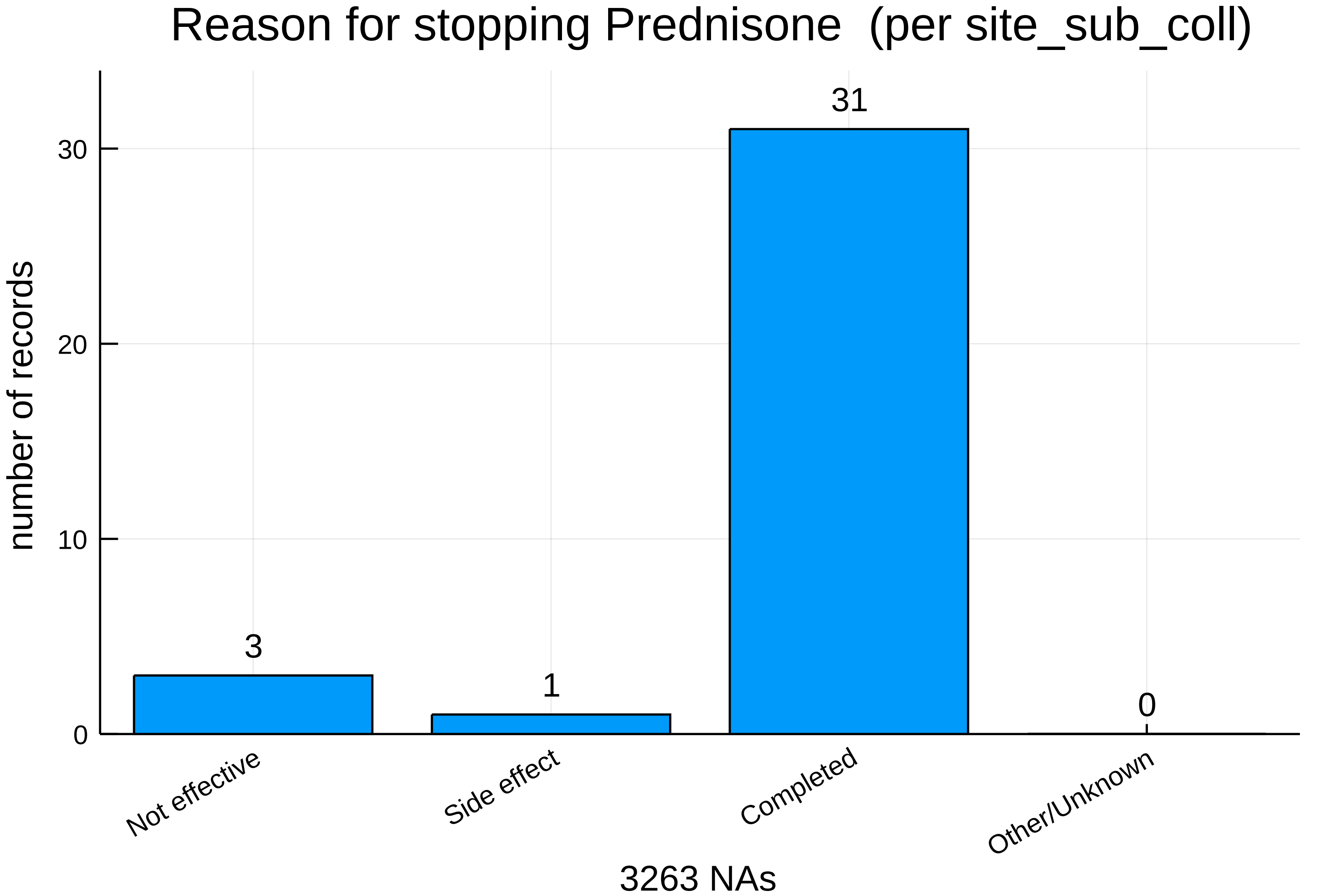

Reason for stopping Prednisone (per site_sub_coll)
number of records
0
10
20
30
3
1
31
0
Not effective
Side effect
Completed
Other/Unknown
3263 NAs

Reason for withdrawal or termination (per Participant_ID)

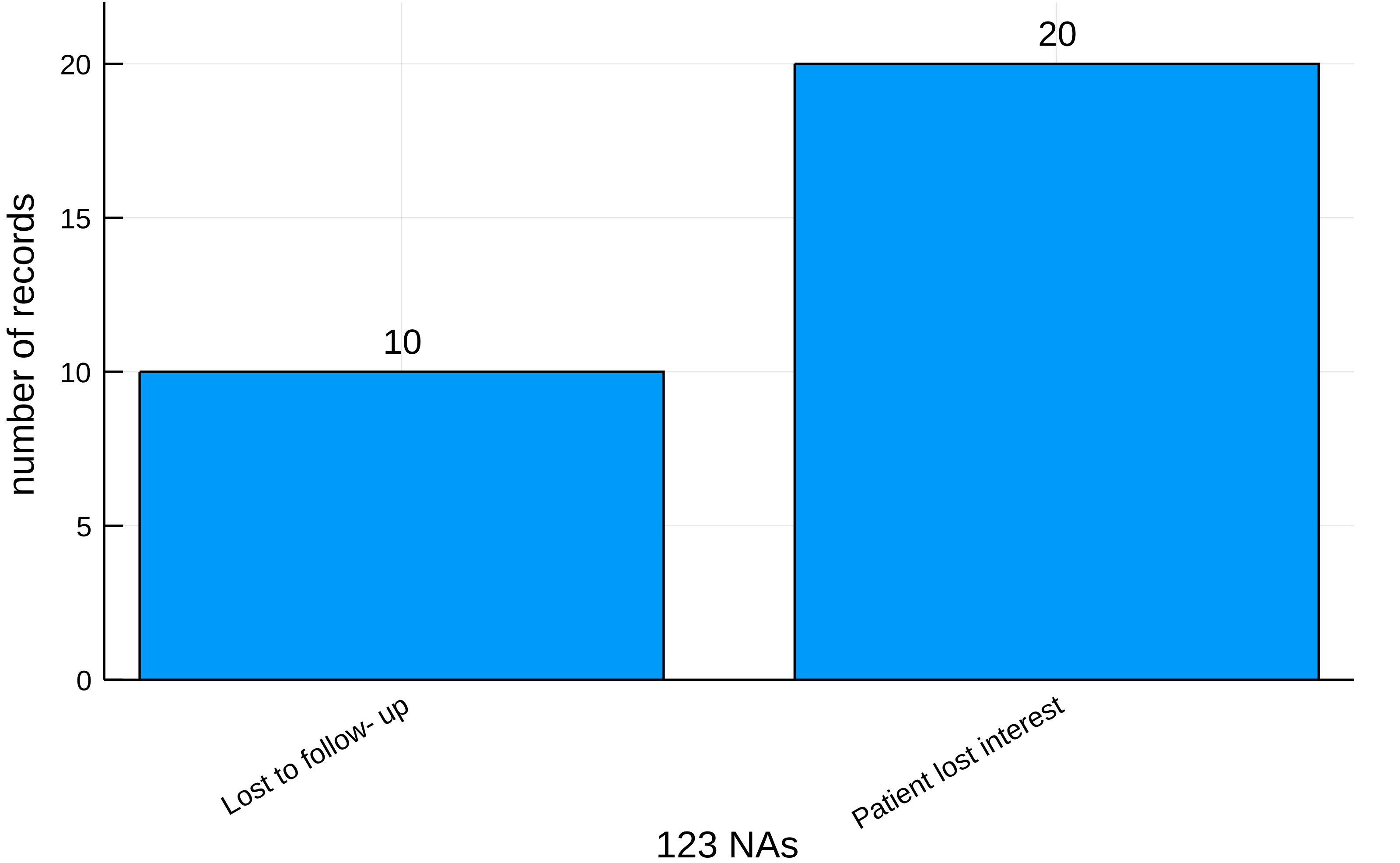

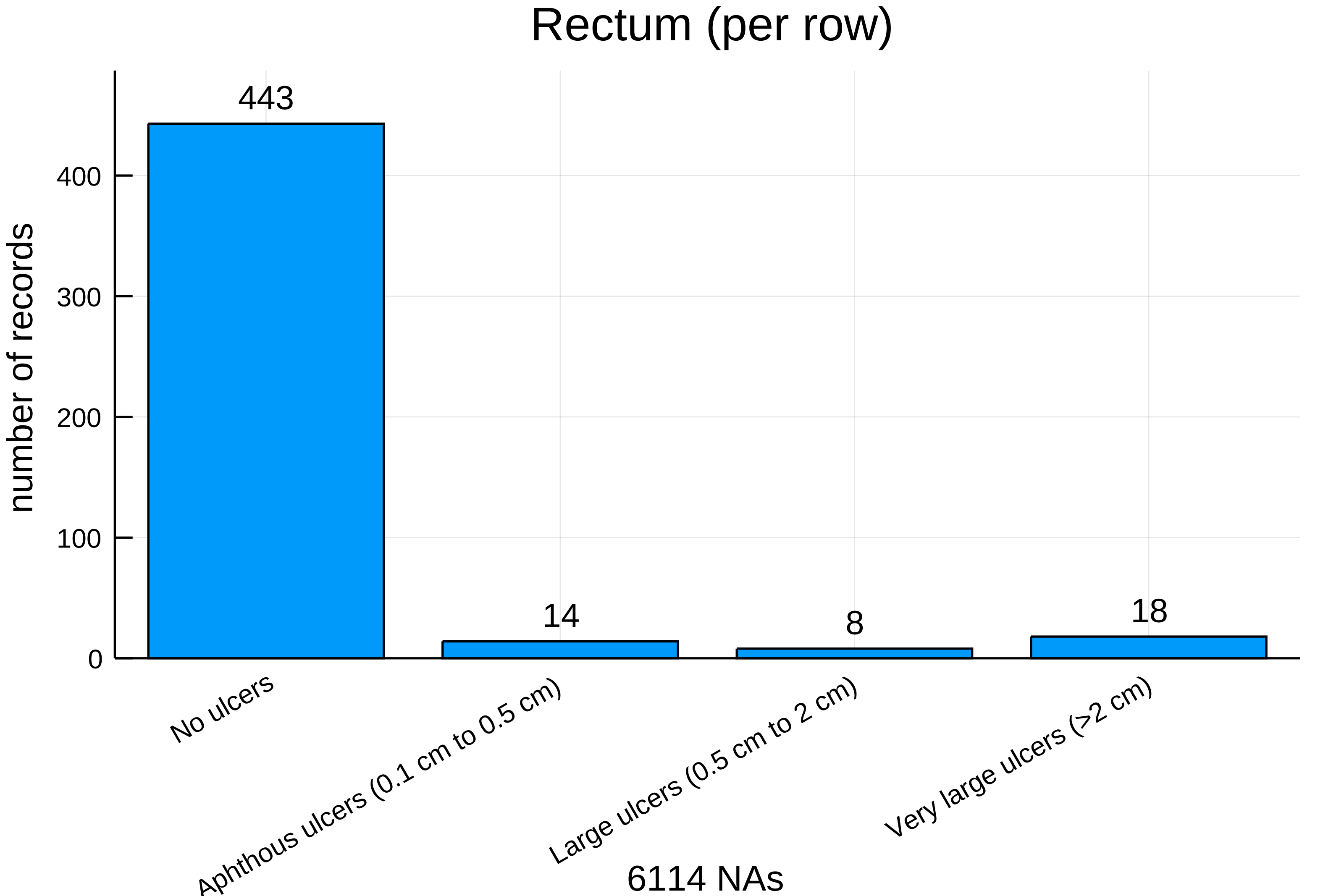
Rectum (per row)
number of records
0
100
200
300
400
443
14
8
18
No ulcers
Aphthous ulcers (0.1 cm to 0.5 cm)
Large ulcers (0.5 cm to 2 cm)
Very large ulcers (>2 cm)
6114 NAs

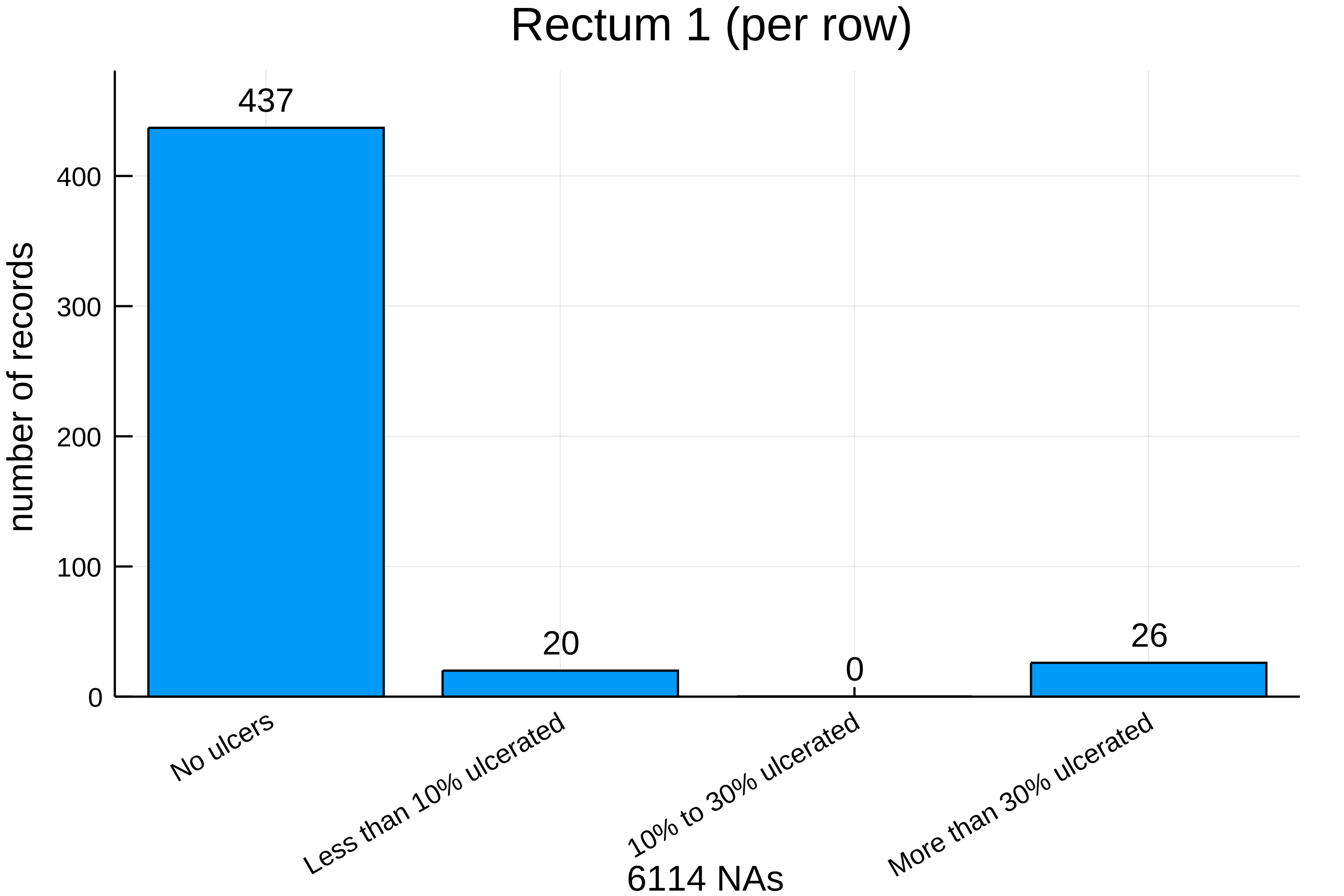
Rectum 1 (per row)
number of records
0
100
200
300
400
437
20
0
26
No ulcers
Less than 10% ulcerated
10% to 30% ulcerated
More than 30% ulcerated
6114 NAs

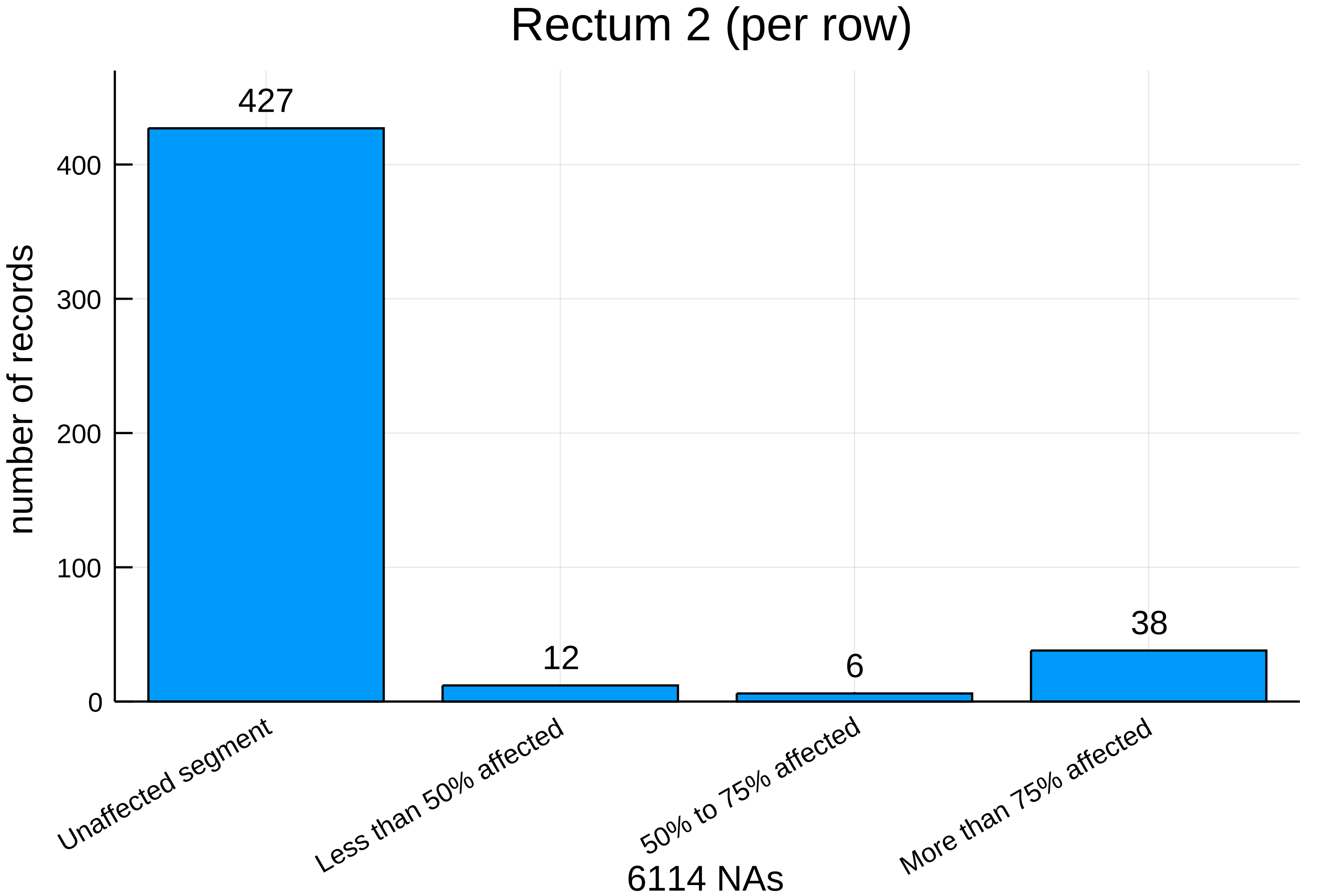
Rectum 2 (per row)
number of records
0
100
200
300
400
427
12
6
38
Unaffected segment
Less than 50% affected
50% to 75% affected
More than 75% affected
6114 NAs

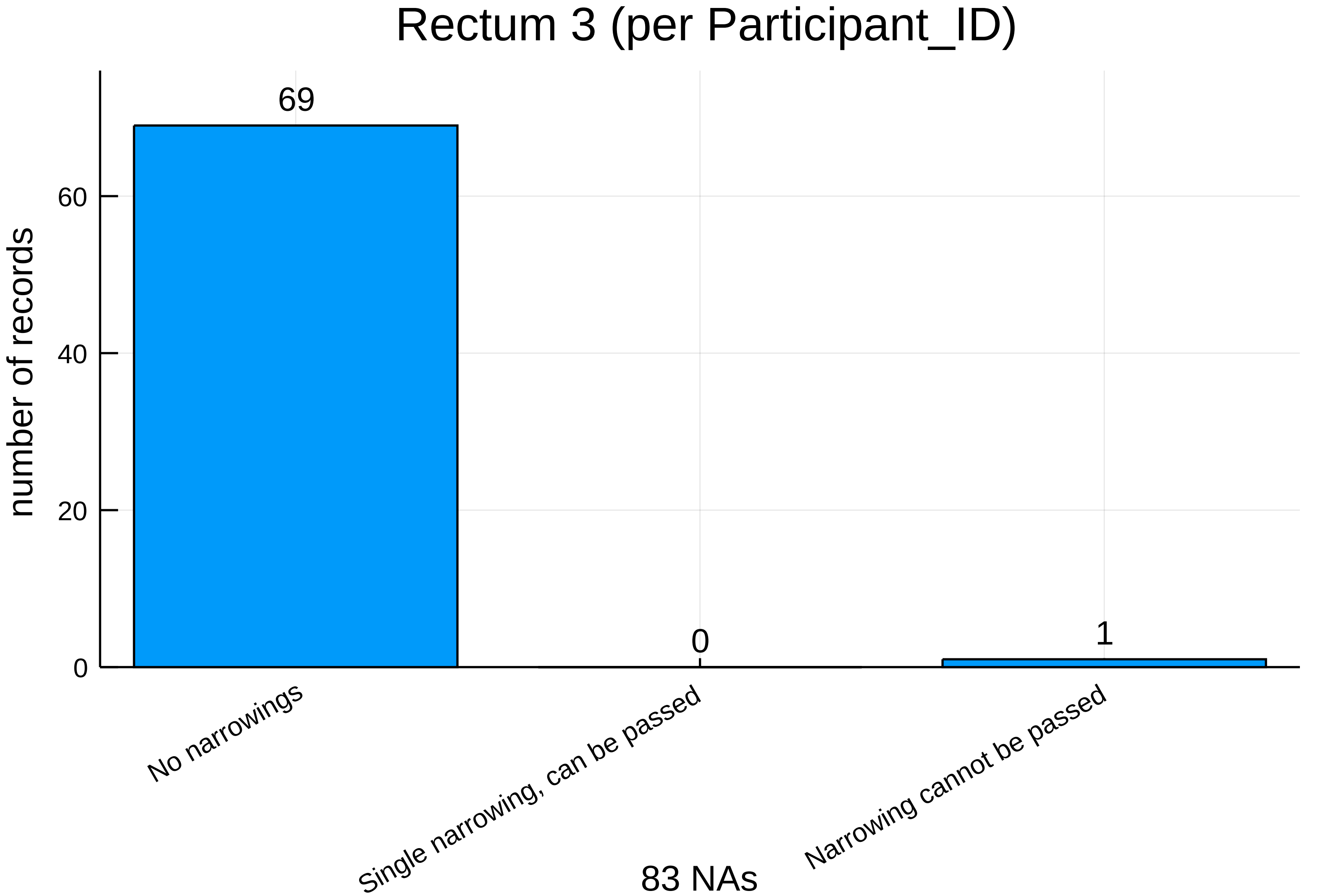
Rectum 3 (per Participant_ID)
number of records
69
0
1
60
40
20
0
No narrowings
Single narrowing, can be passed
Narrowing cannot be passed
83 NAs

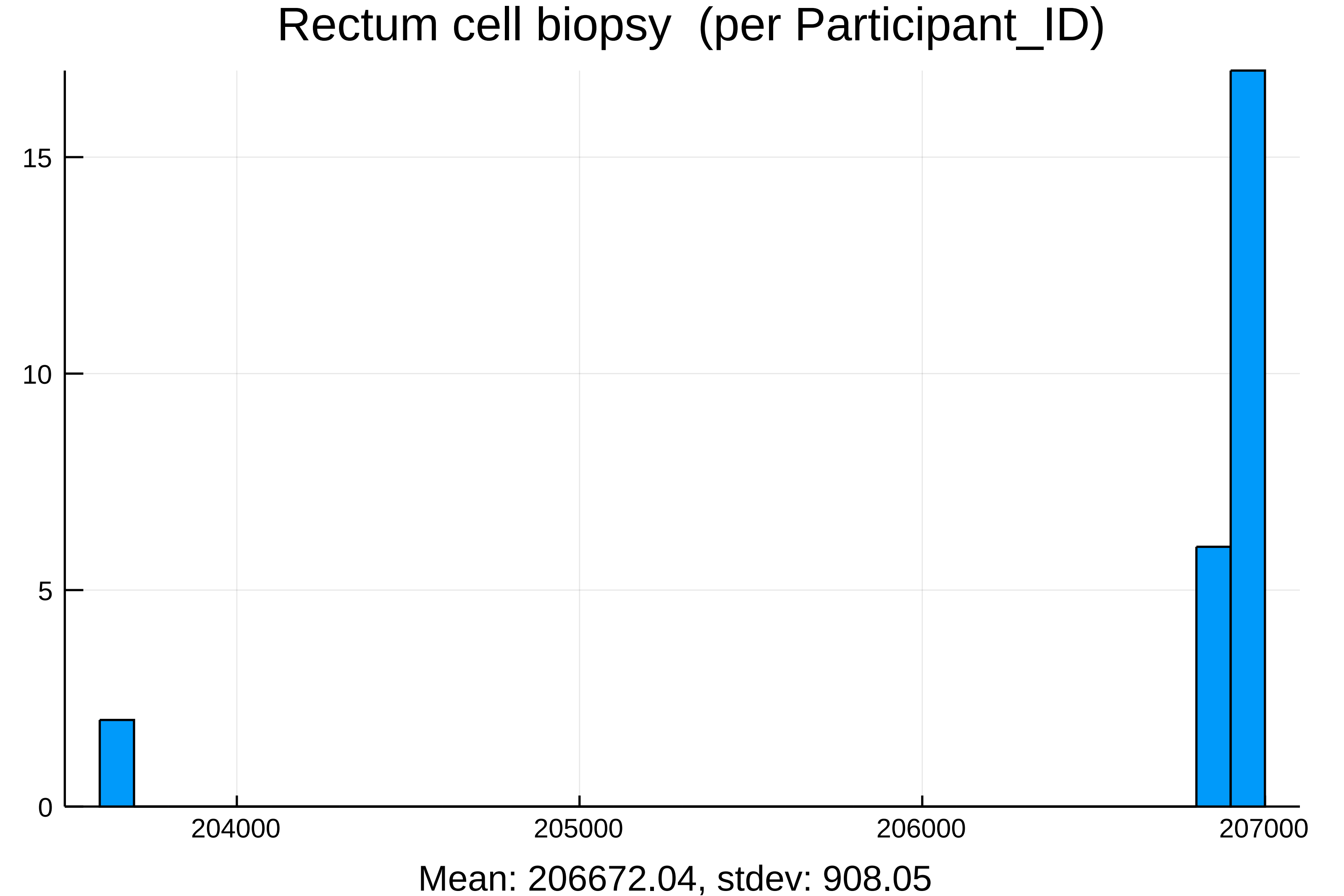
Rectum cell biopsy (per Participant_ID)
15
10
5
0
204000
205000
206000
207000
Mean: 206672.04, stdev: 908.05

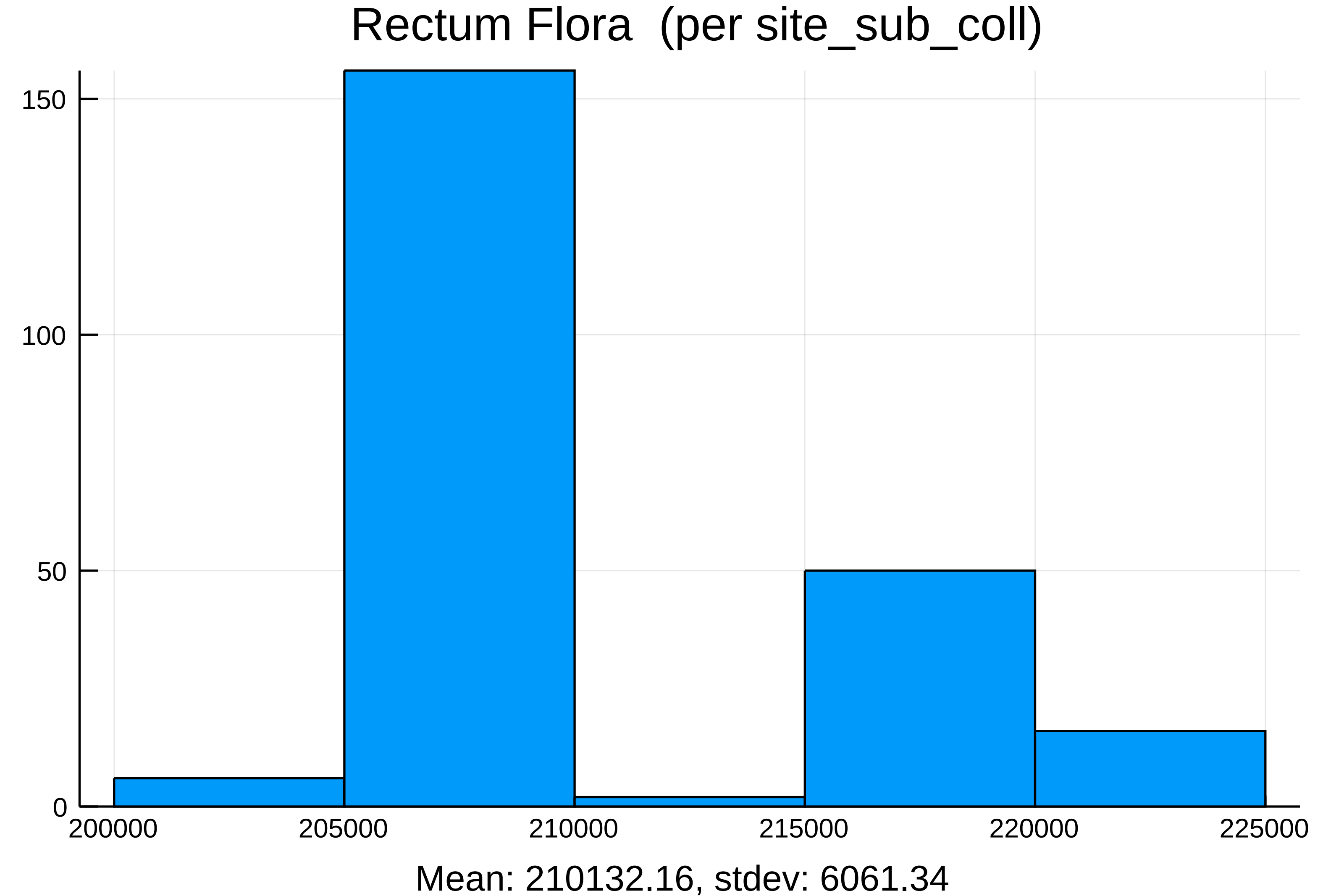
Rectum Flora (per site_sub_coll)
150
100
50
0
200000
205000
210000
215000
220000
225000
Mean: 210132.16, stdev: 6061.34

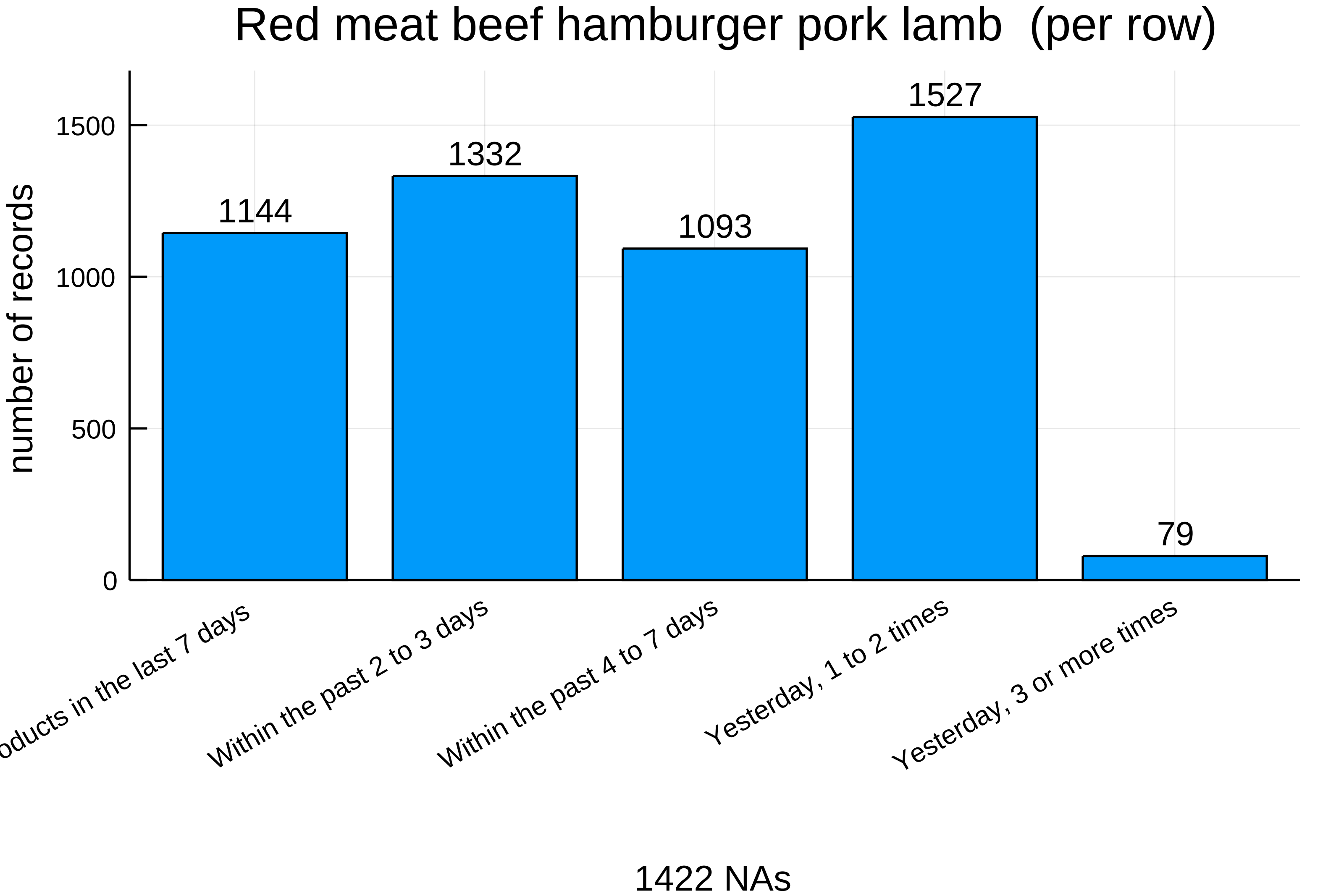
Red meat beef hamburger pork lamb (per row)
number of records
1500
1000
500
0
1144
1332
1093
1527
79
oducts in the last 7 days
Within the past 2 to 3 days
Within the past 4 to 7 days
Yesterday, 1 to 2 times
Yesterday, 3 or more times
1422 NAs

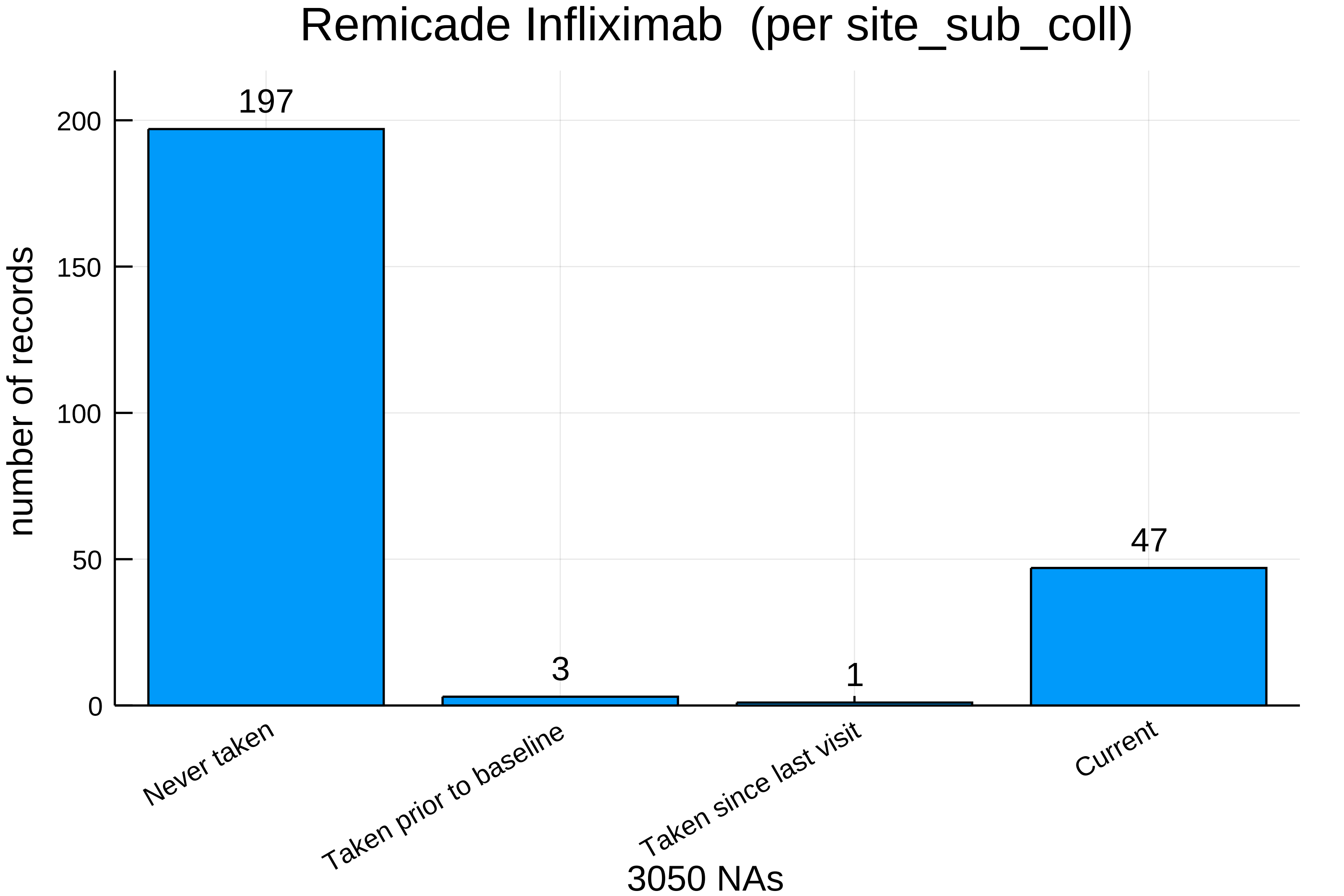

Remicade Infliximab (per site_sub_coll)
number of records
0
50
100
150
200
197
3
1
47
Never taken
Taken prior to baseline
Taken since last visit
Current
3050 NAs

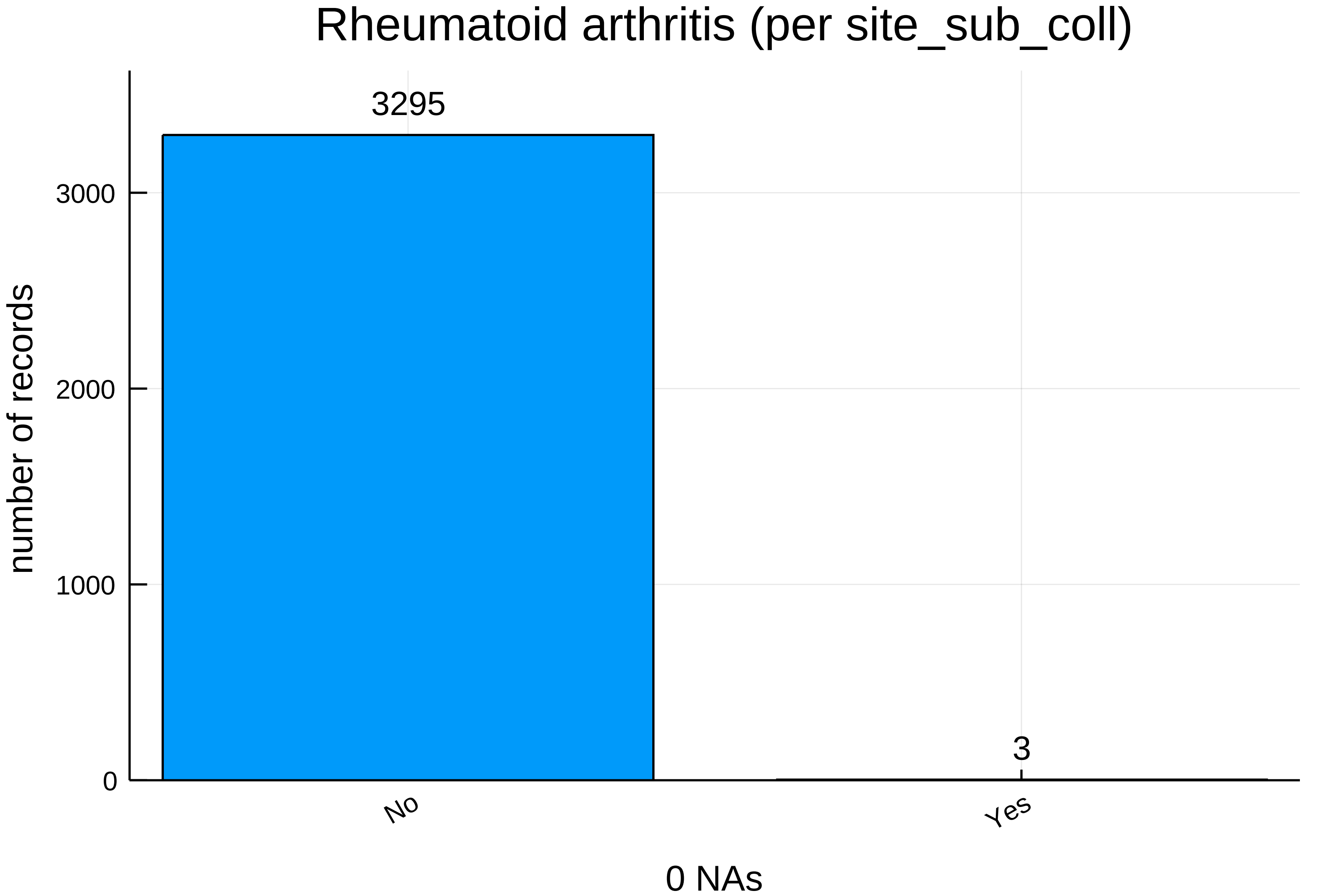
Rheumatoid arthritis (per site_sub_coll)
3295
3
3000
2000
1000
0
number of records
No
Yes
0 NAs

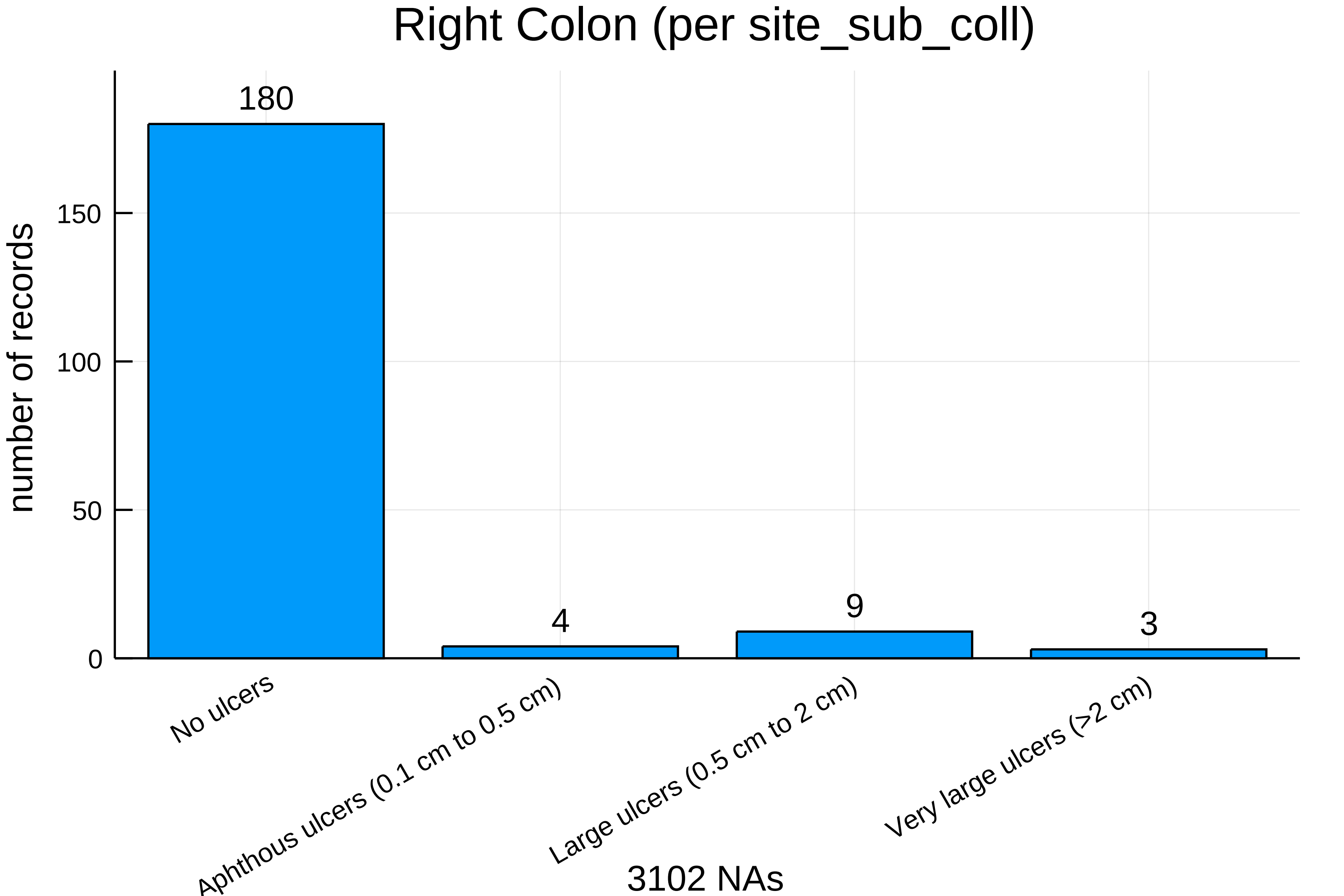

Right Colon (per site_sub_coll)
number of records
180
4
9
3
0
50
100
150
No ulcers
Aphthous ulcers (0.1 cm to 0.5 cm)
Large ulcers (0.5 cm to 2 cm)
Very large ulcers (>2 cm)
3102 NAs

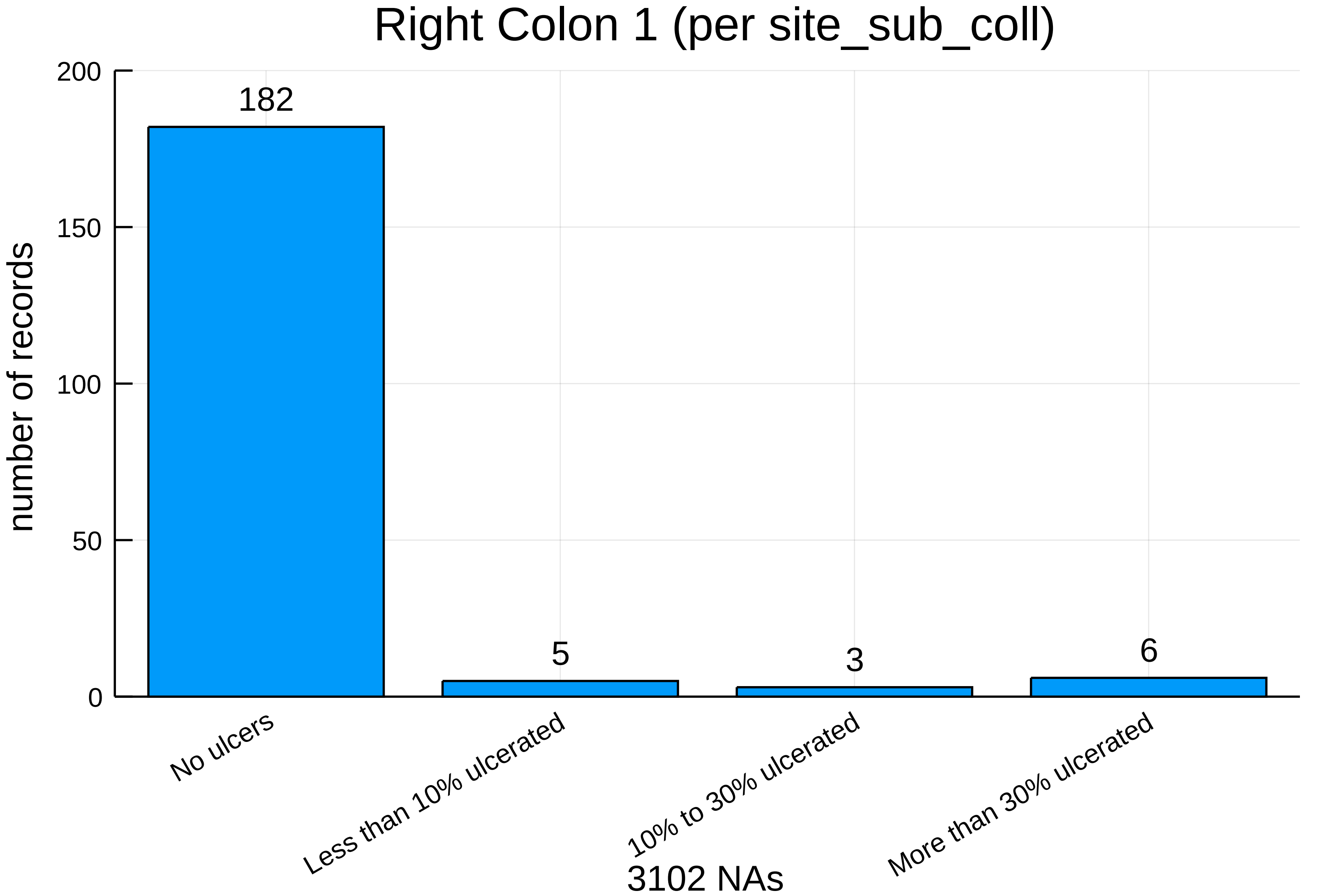
Right Colon 1 (per site_sub_coll)
number of records
200
150
100
50
0
182
5
3
6
No ulcers
Less than 10% ulcerated
10% to 30% ulcerated
More than 30% ulcerated
3102 NAs

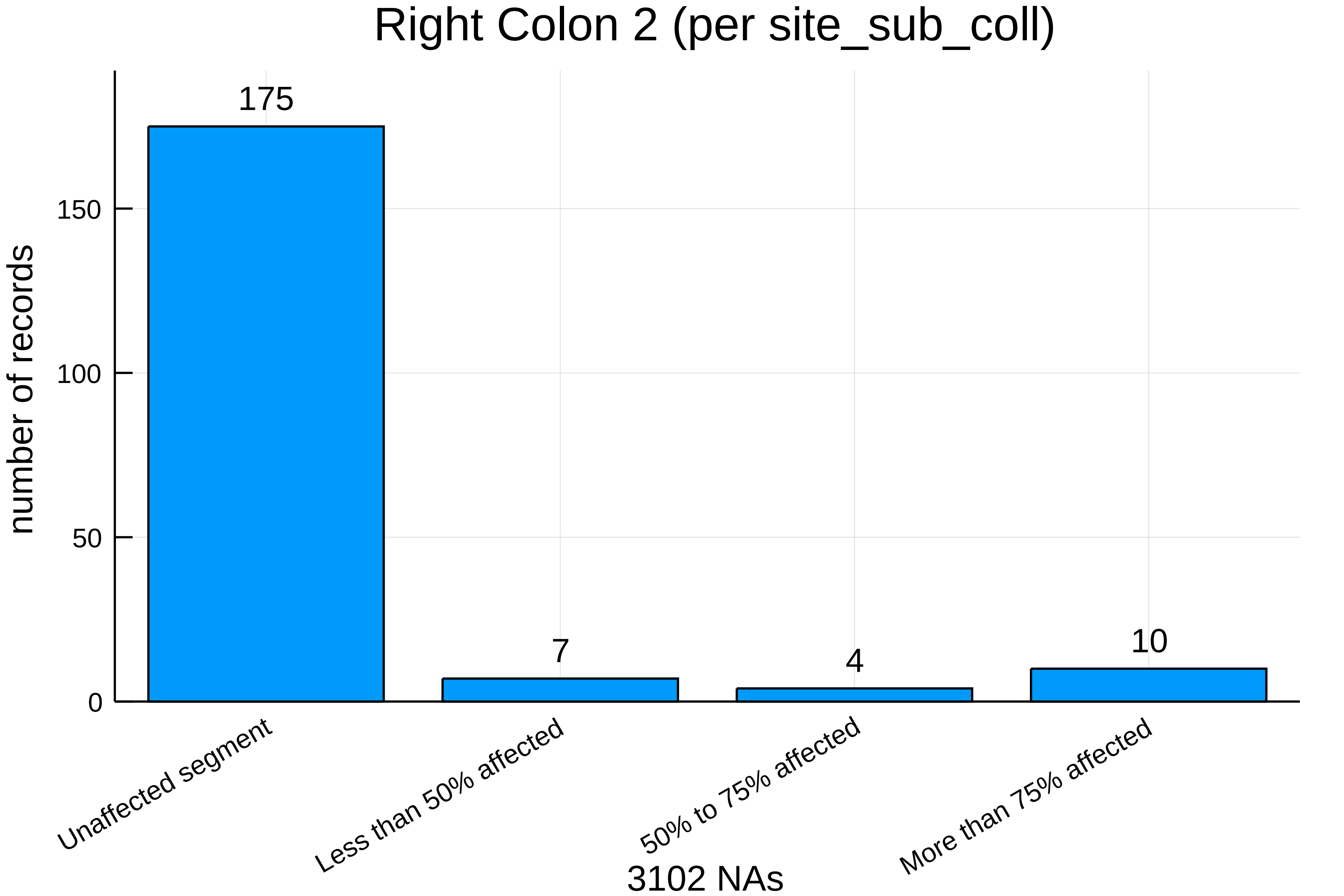
Right Colon 2 (per site_sub_coll)
number of records
175
7
4
10
0
50
100
150
Unaffected segment
Less than 50% affected
50% to 75% affected
More than 75% affected
3102 NAs

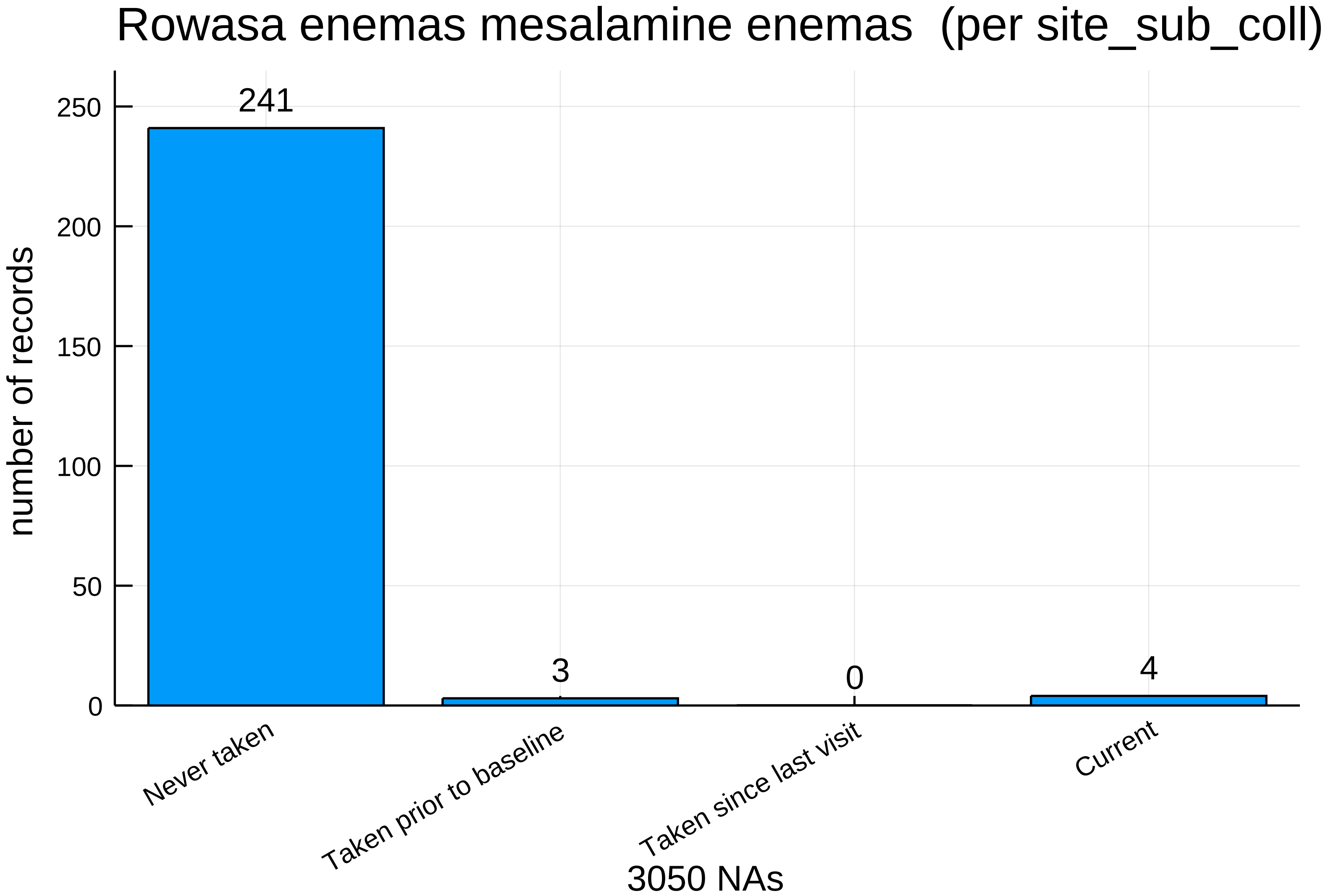
Rowasa enemas mesalamine enemas (per site_sub_coll)
number of records
250
200
150
100
50
0
241
3
0
4
Never taken
Taken prior to baseline
Taken since last visit
Current
3050 NAs

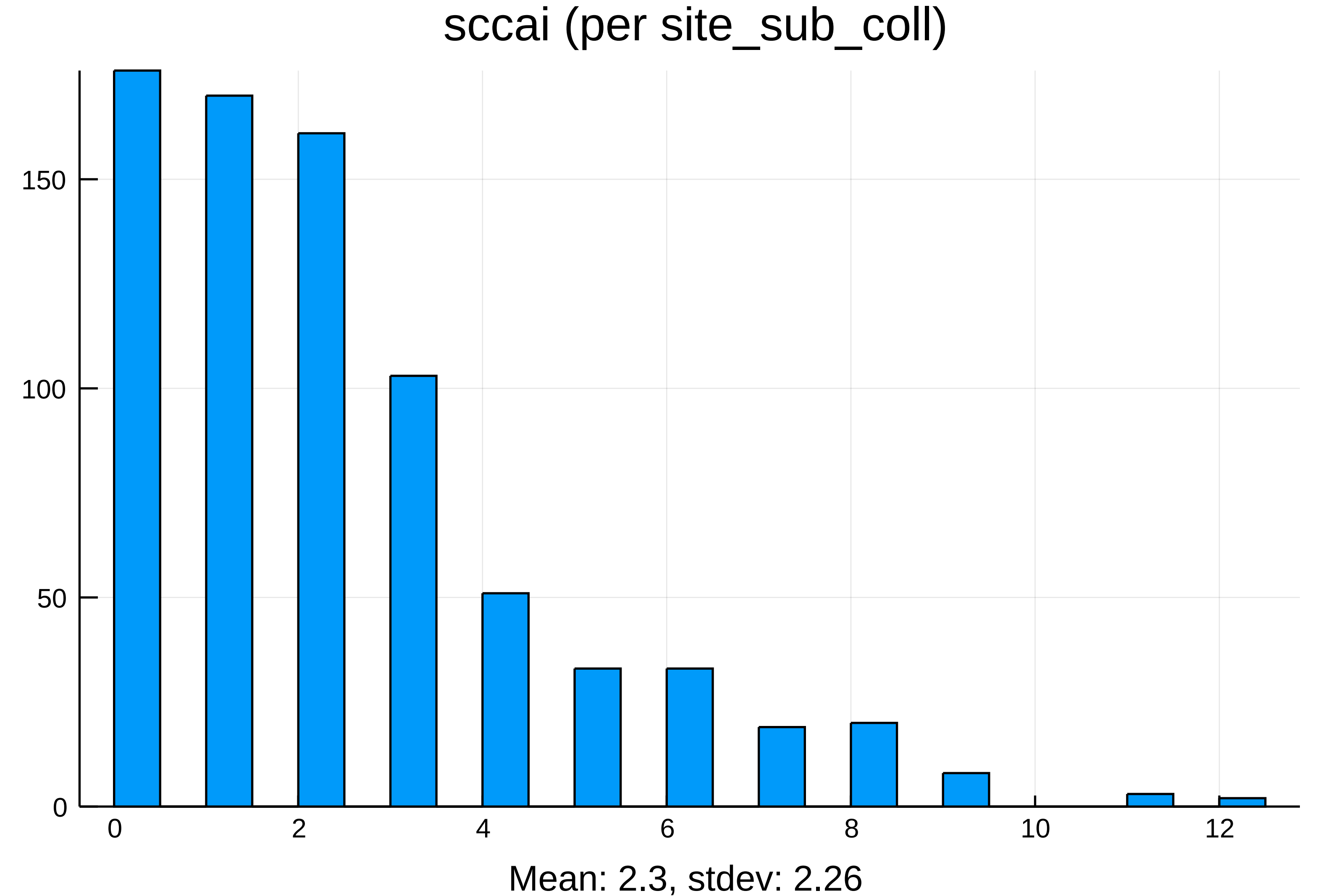
sccai (per site_sub_coll)
150
100
50
0
0
2
4
6
8
10
12
Mean: 2.3, stdev: 2.26

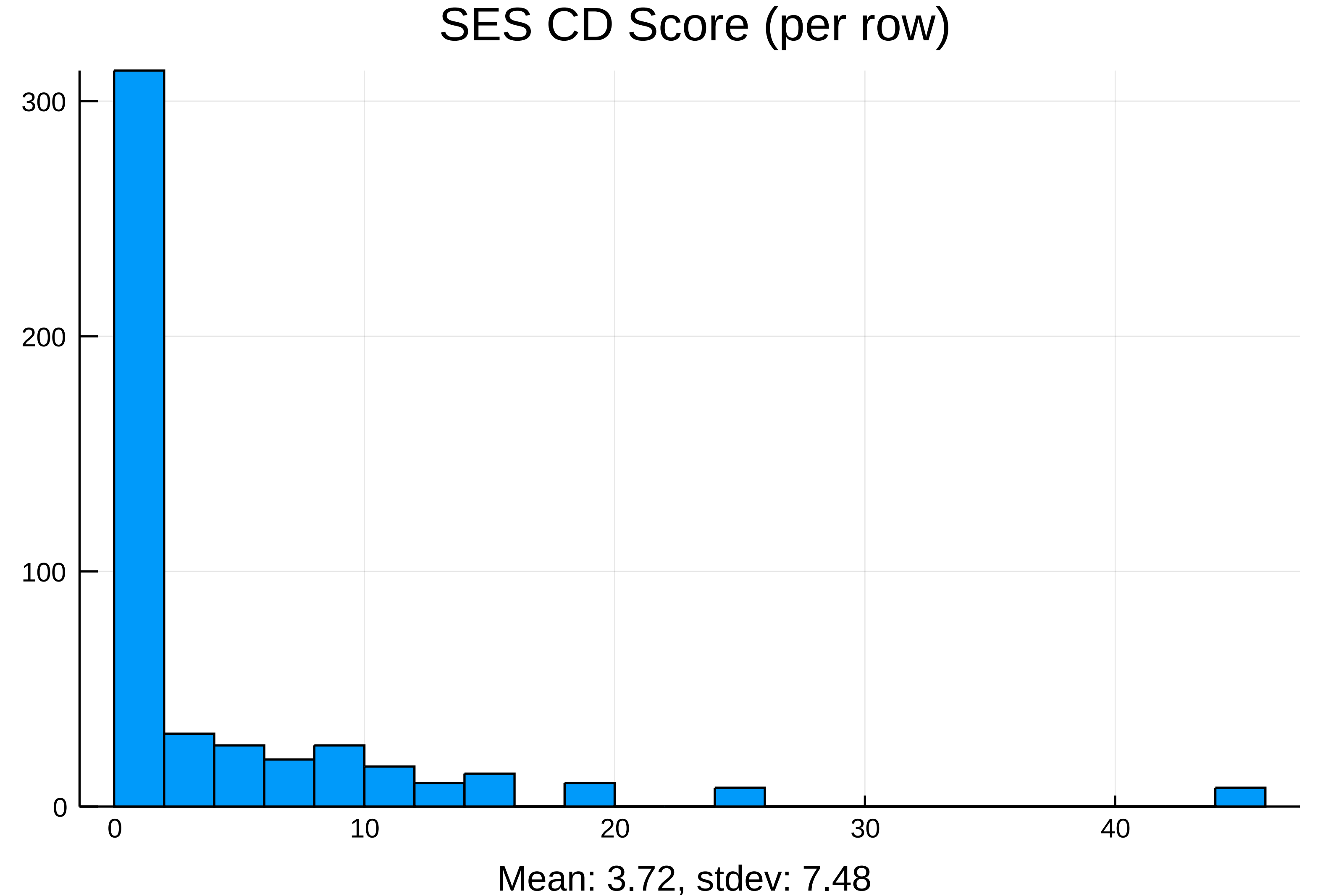
SES CD Score (per row)
300
200
100
0
0
10
20
30
40
Mean: 3.72, stdev: 7.48

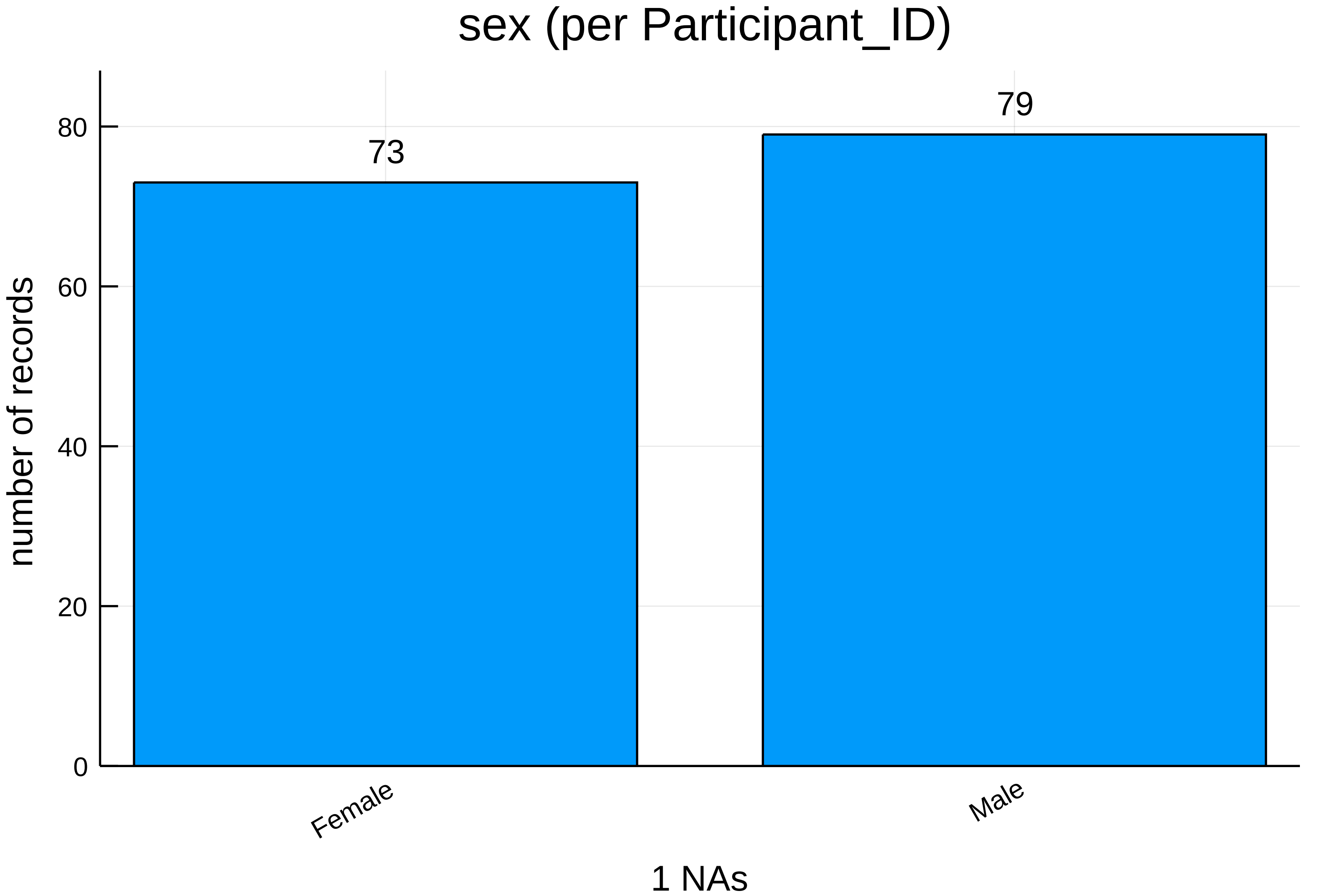

sex (per Participant_ID)
number of records
80
60
40
20
0
73
79
Female
Male
1 NAs

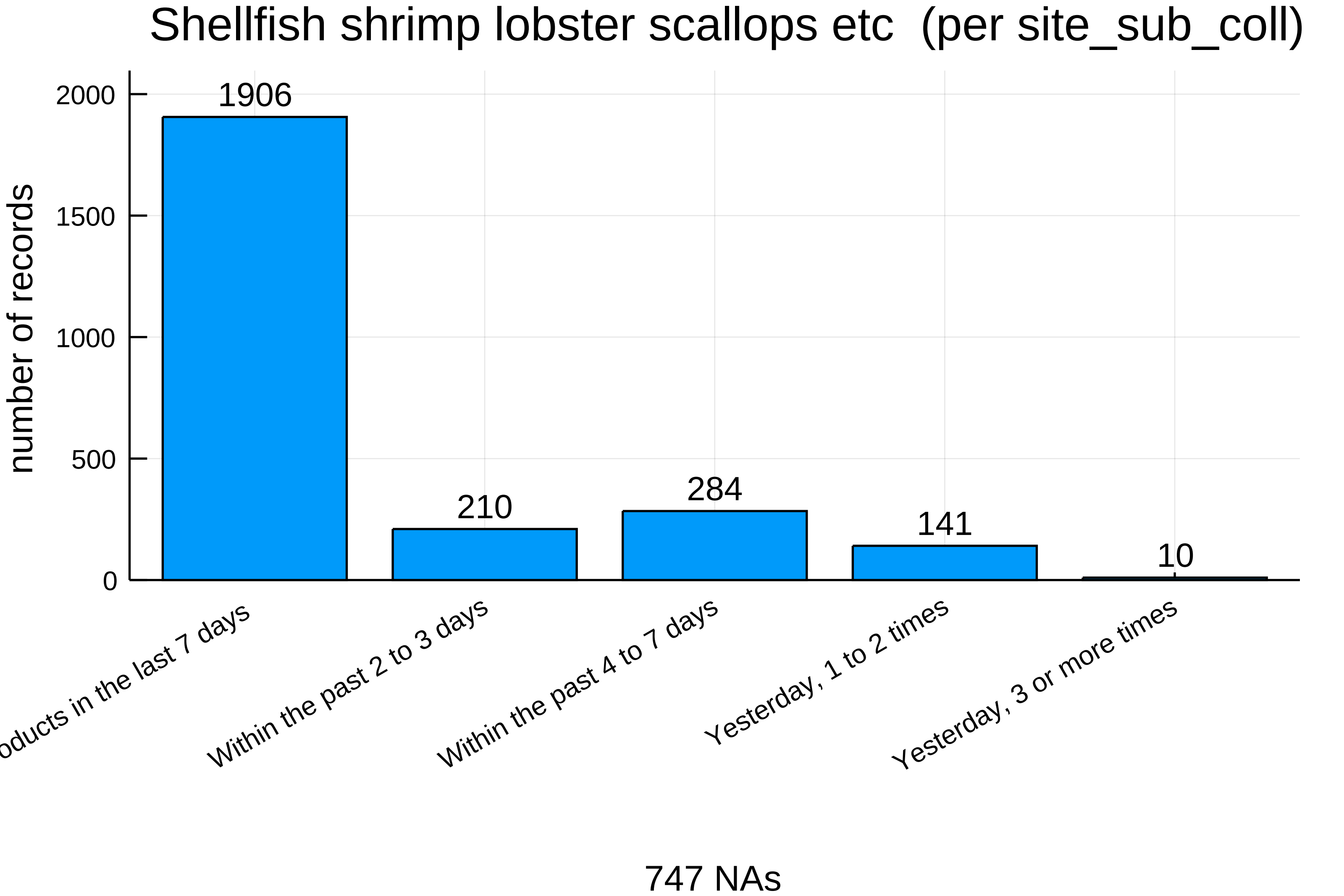
Shellfish shrimp lobster scallops etc (per site_sub_coll)
number of records
2000
1500
1000
500
0
1906
210
284
141
10
oducts in the last 7 days
Within the past 2 to 3 days
Within the past 4 to 7 days
Yesterday, 1 to 2 times
Yesterday, 3 or more times
747 NAs

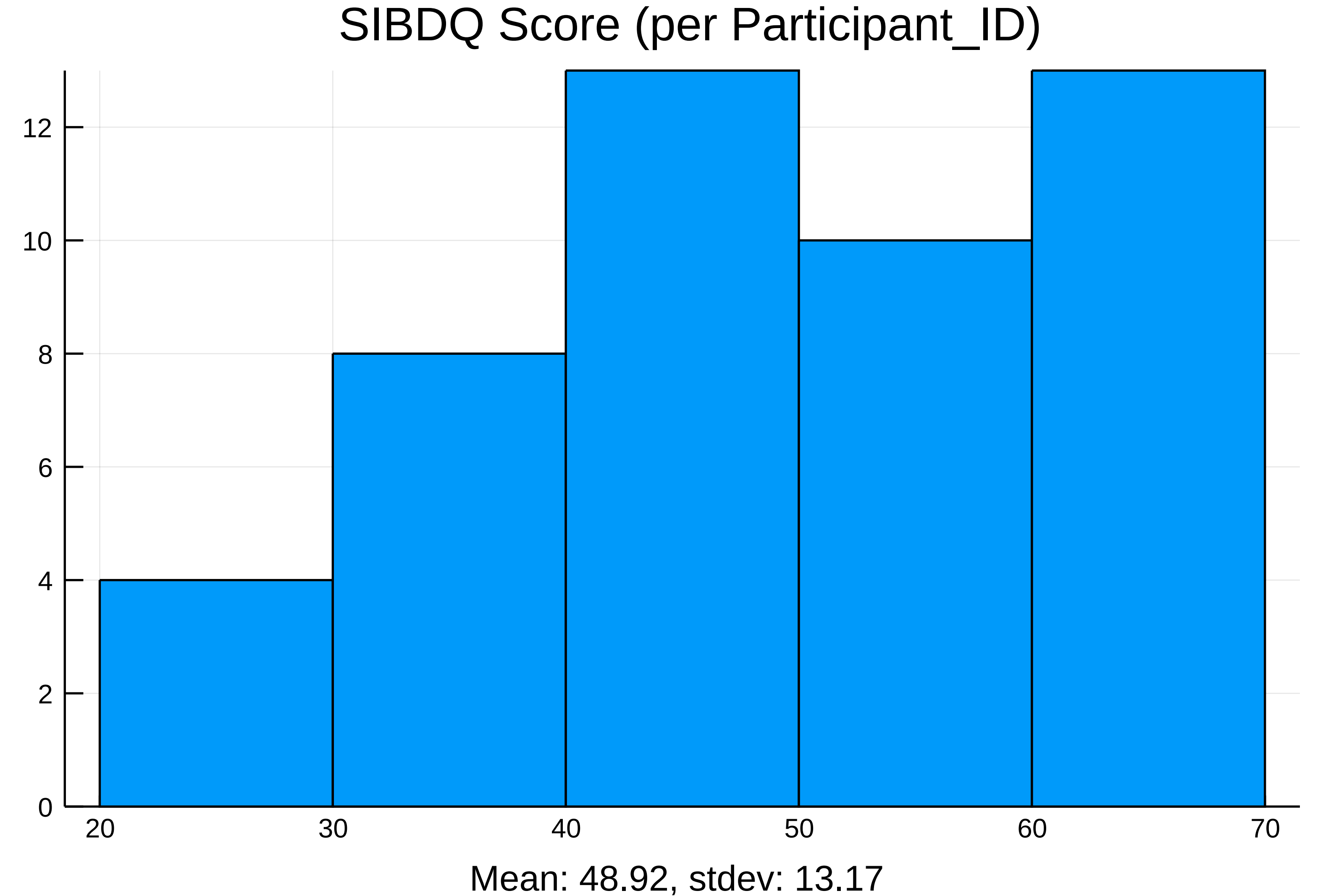
SIBDQ Score (per Participant_ID)
0
2
4
6
8
10
12
20
30
40
50
60
70
Mean: 48.92, stdev: 13.17

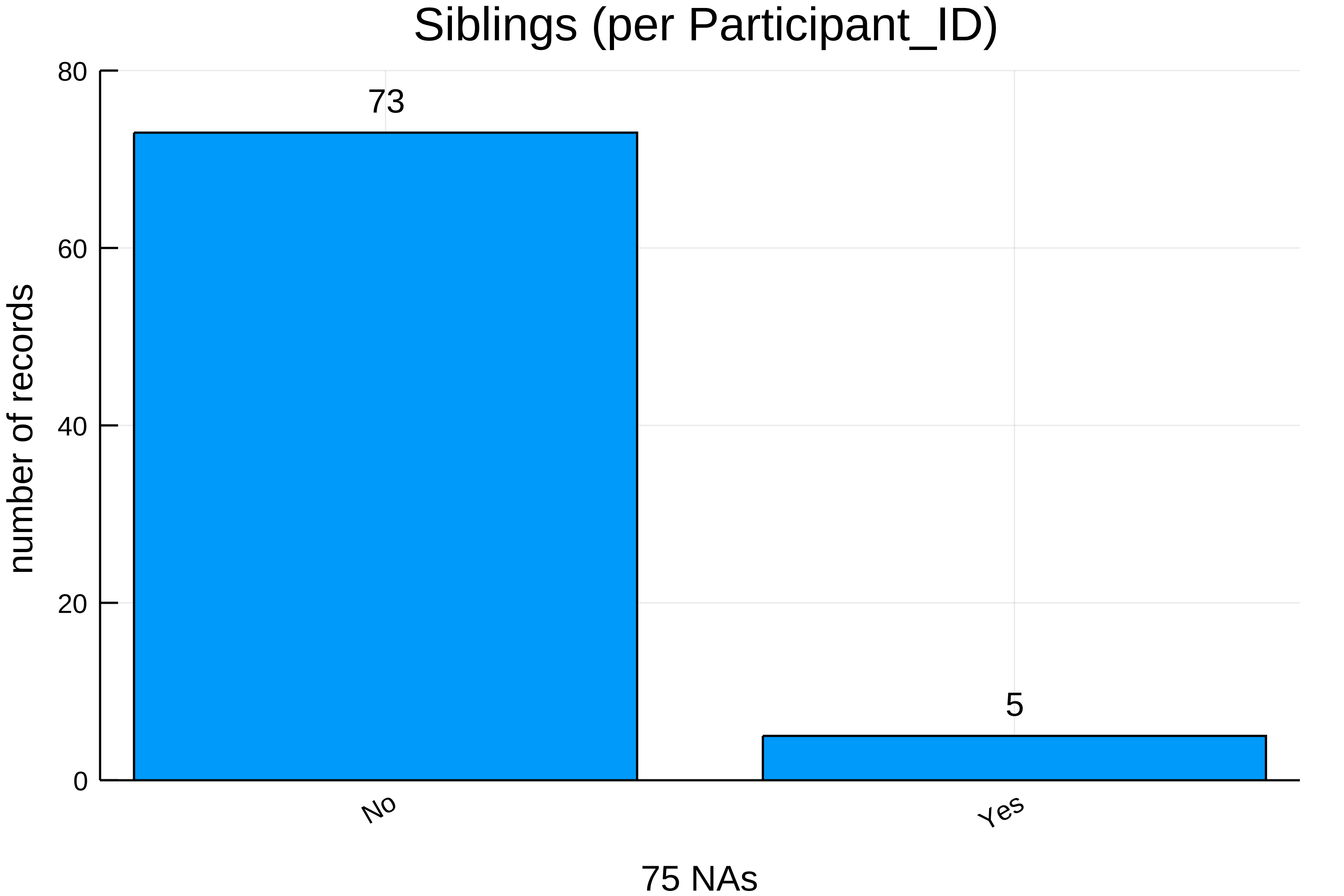
Siblings (per Participant_ID)
number of records
80
60
40
20
0
73
5
No
Yes
75 NAs

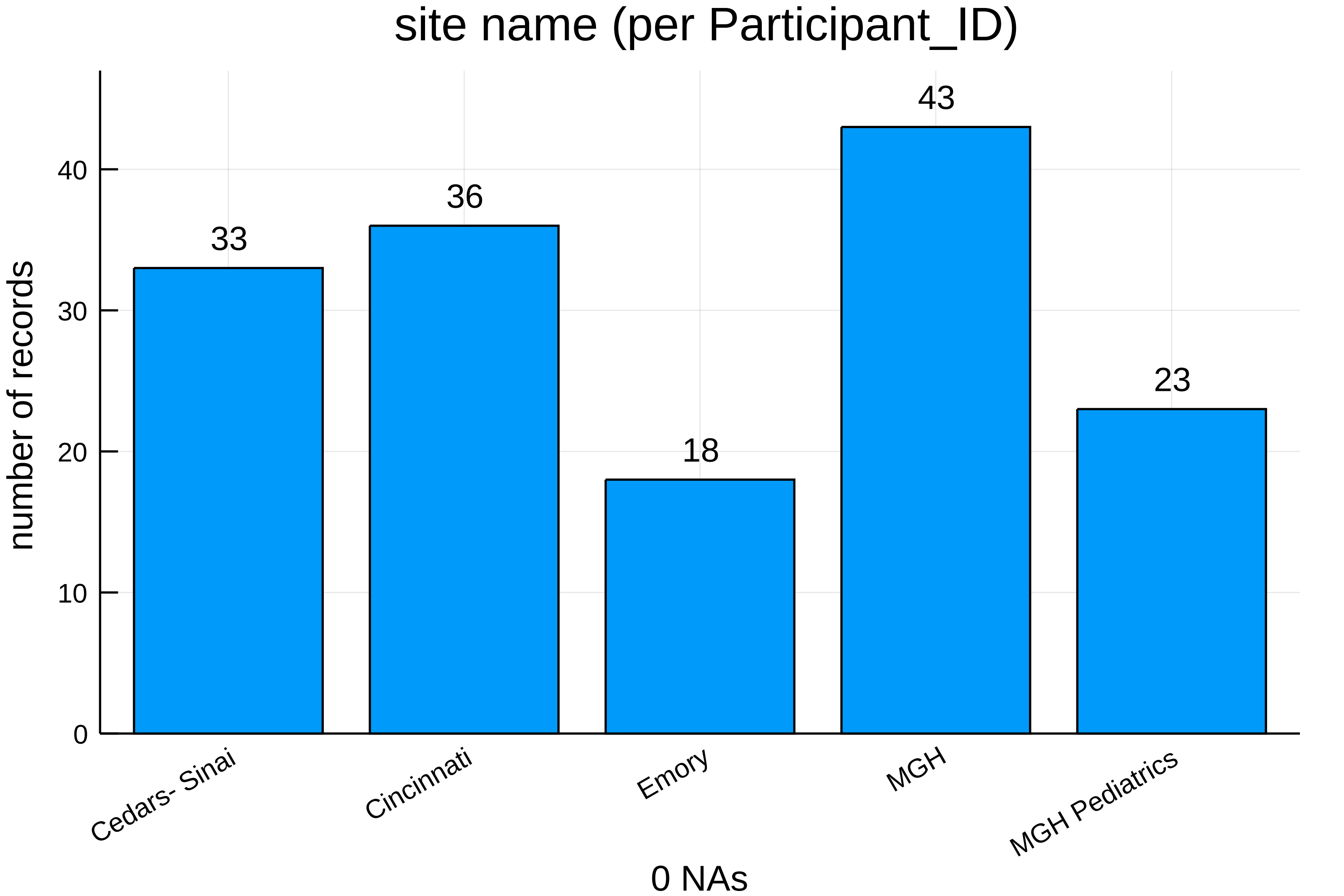
site name (per Participant_ID)
number of records
0
10
20
30
40
33
36
18
43
23
Cedars- Sinai
Cincinnati
Emory
MGH
MGH Pediatrics
0 NAs

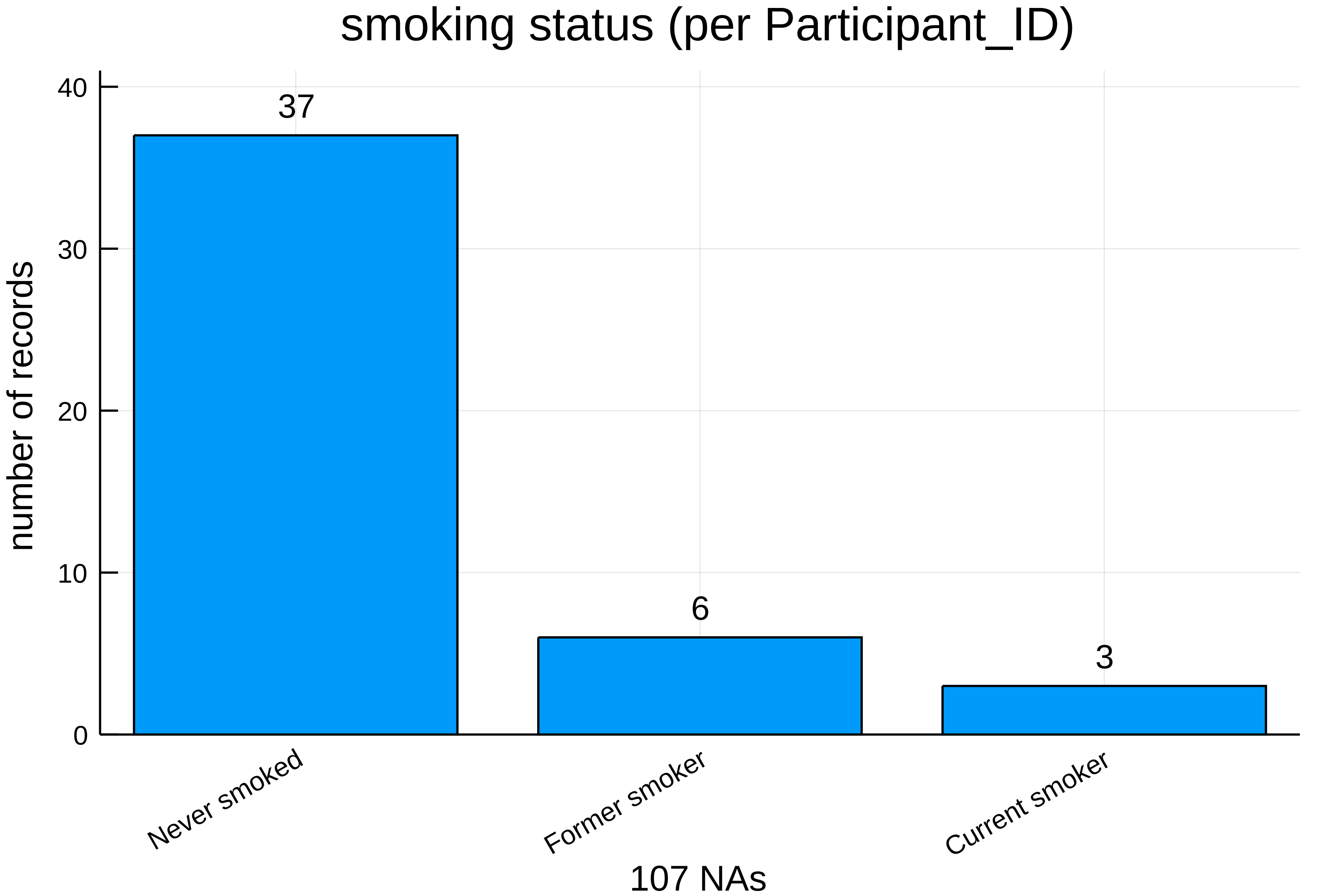
smoking status (per Participant_ID)
number of records
0
10
20
30
40
37
6
3
Never smoked
Former smoker
Current smoker
107 NAs

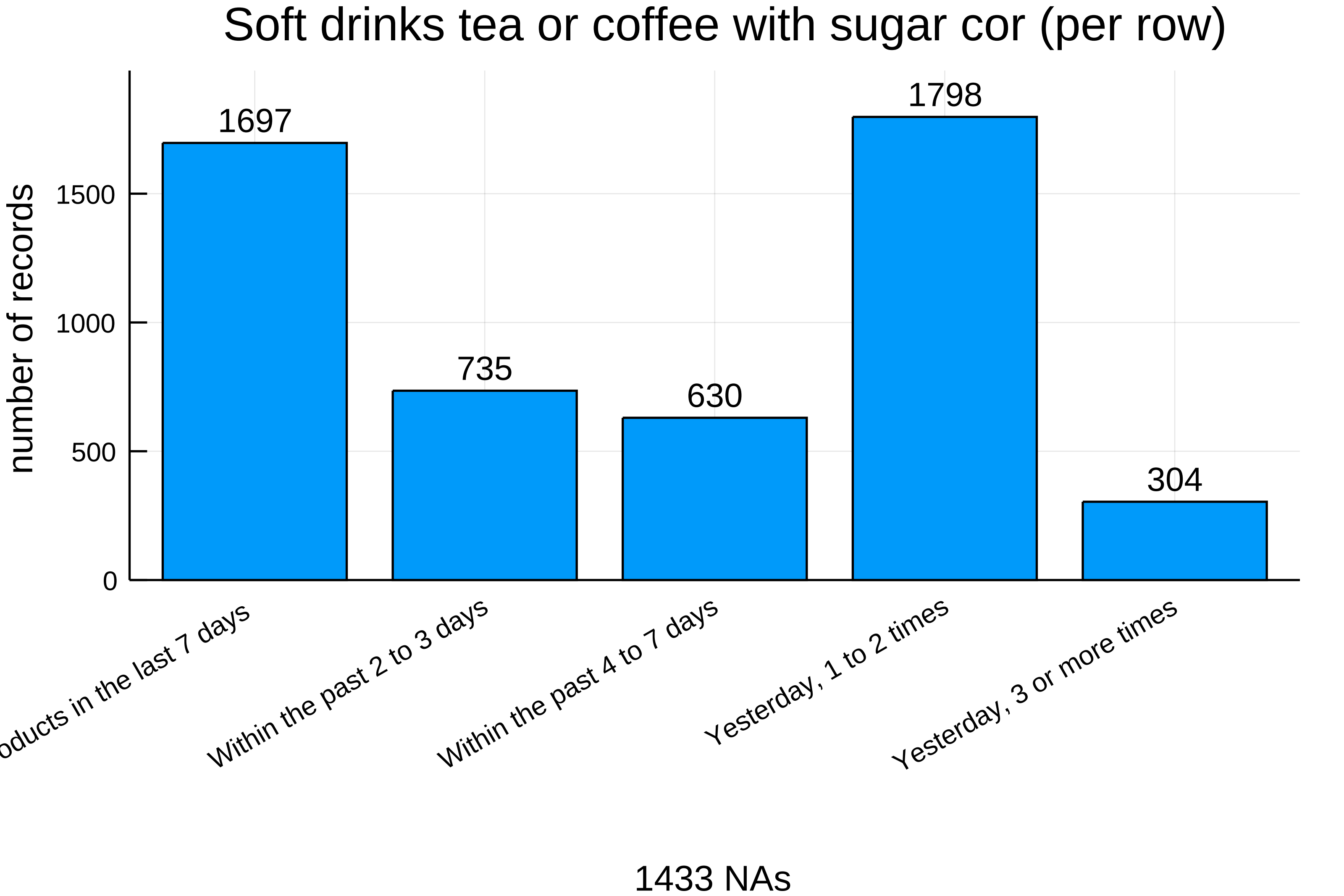

Soft drinks tea or coffee with sugar cor (per row)
number of records
1500
1000
500
0
1697
735
630
1798
304
oducts in the last 7 days
Within the past 2 to 3 days
Within the past 4 to 7 days
Yesterday, 1 to 2 times
Yesterday, 3 or more times
1433 NAs

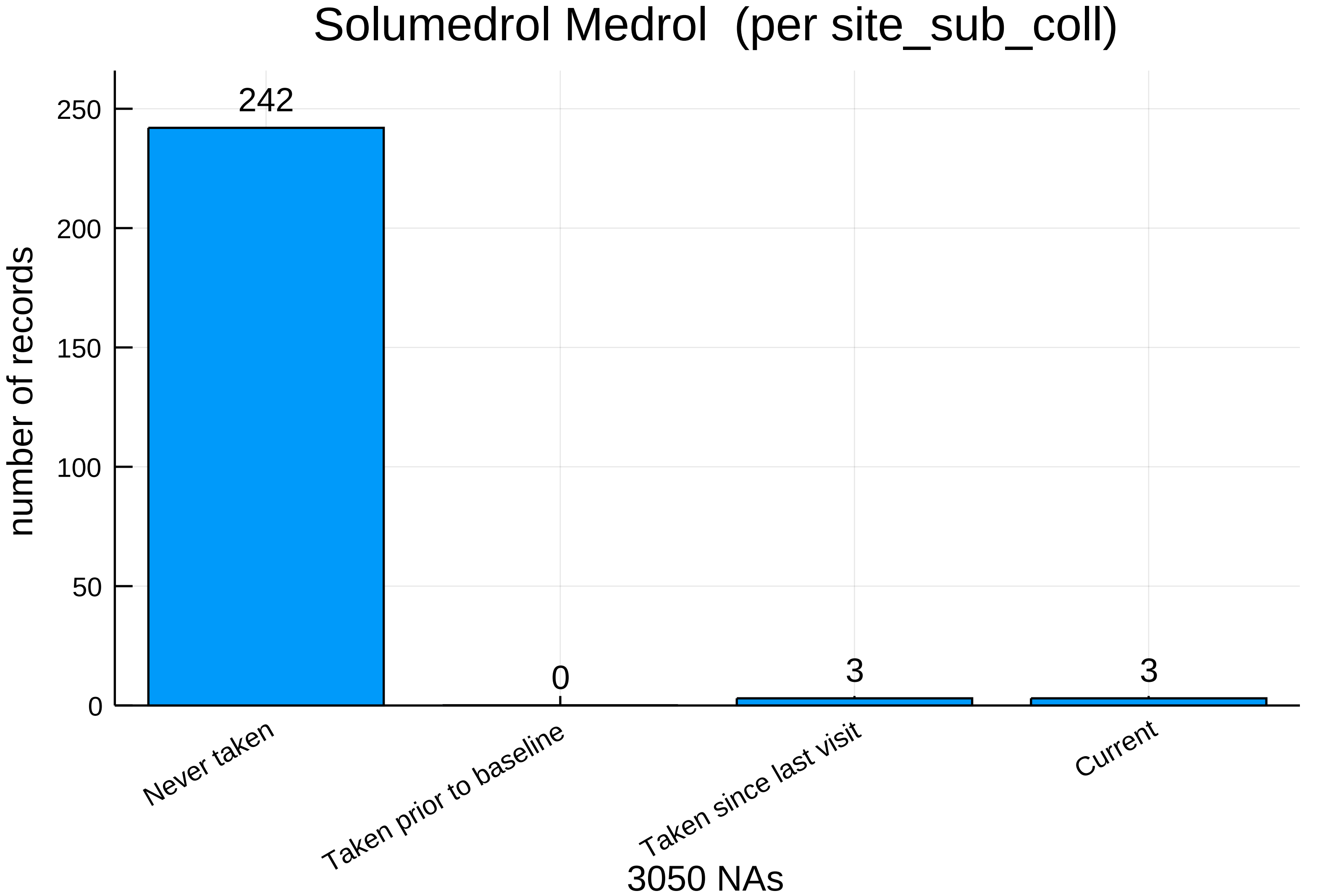

Solumedrol Medrol (per site_sub_coll)
number of records
242
0
3
3
0
50
100
150
200
250
Never taken
Taken prior to baseline
Taken since last visit
Current
3050 NAs

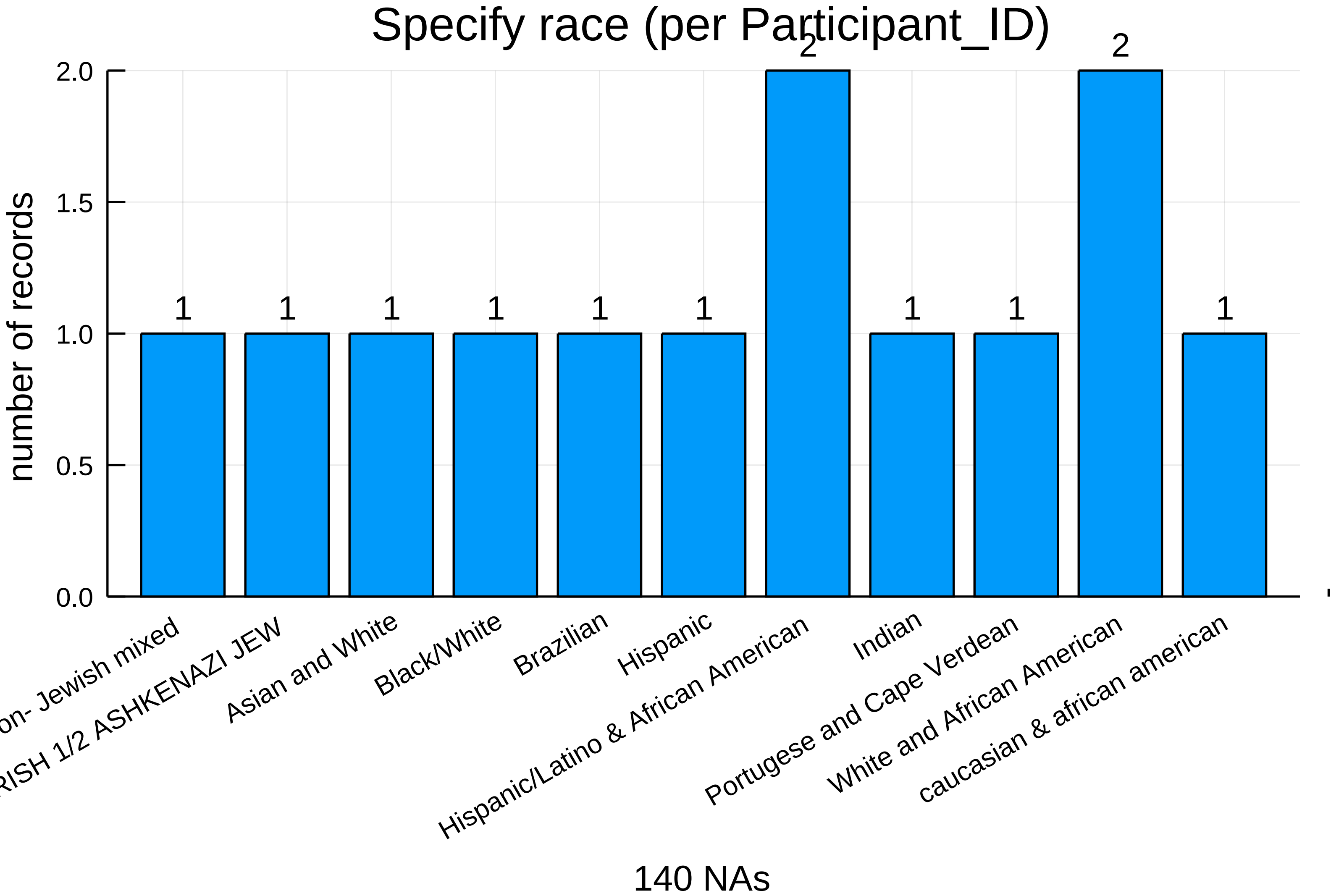

Specify race (per Participant_ID)
number of records
2.0
1.5
1.0
0.5
0.0
1
1
1
1
1
1
2
1
1
2
1
on- Jewish mixed
RISH 1/2 ASHKENAZI JEW
Asian and White
Black/White
Brazilian
Hispanic
Hispanic/Latino & African American
Indian
Portugese and Cape Verdean
White and African American
caucasian & african american
140 NAs

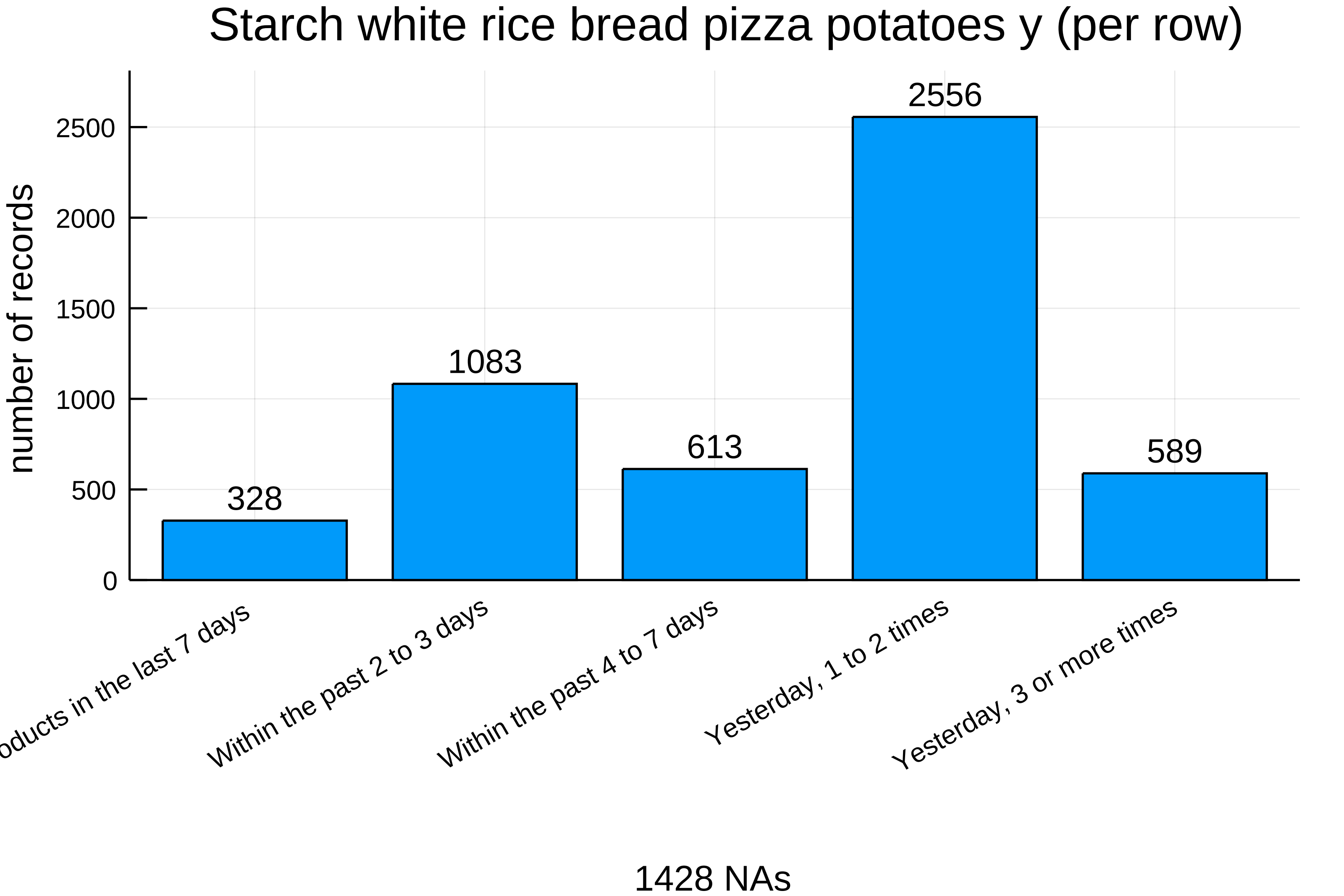

Starch white rice bread pizza potatoes y (per row)
number of records
2500
2000
1500
1000
500
0
328
1083
613
2556
589
oducts in the last 7 days
Within the past 2 to 3 days
Within the past 4 to 7 days
Yesterday, 1 to 2 times
Yesterday, 3 or more times
1428 NAs

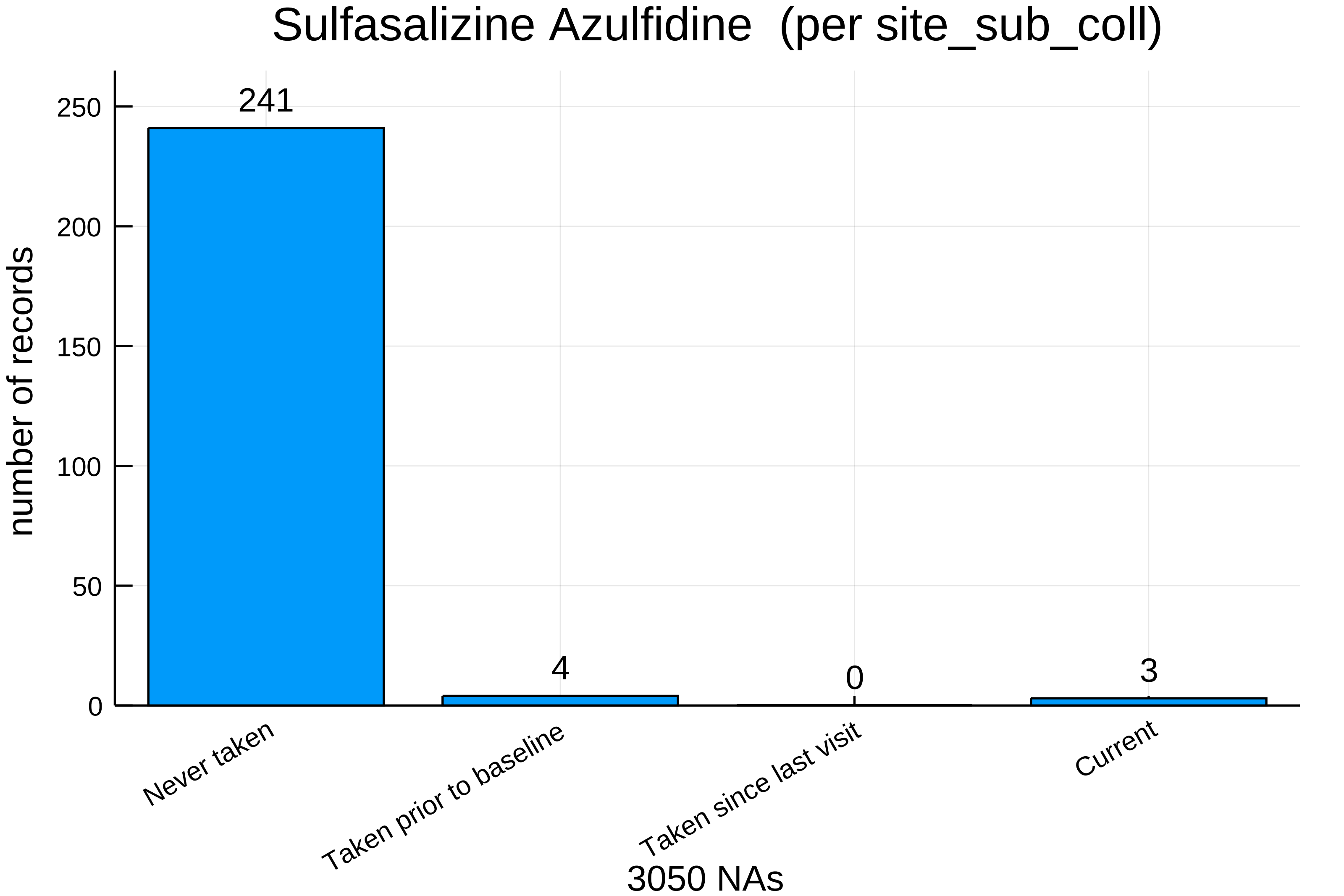
Sulfasalizine Azulfidine (per site_sub_coll)
number of records
250
200
150
100
50
0
241
4
0
3
Never taken
Taken prior to baseline
Taken since last visit
Current
3050 NAs

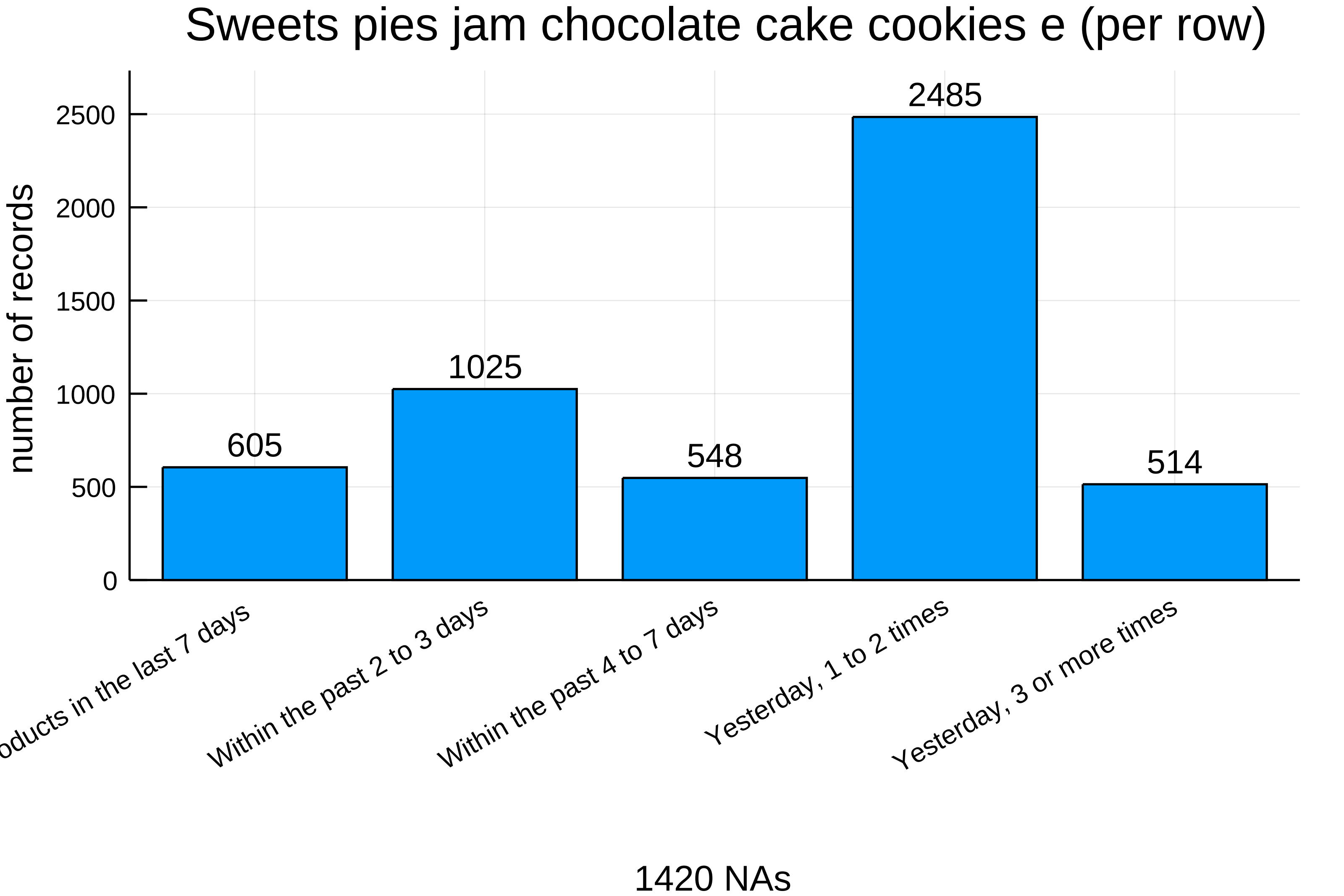

Sweets pies jam chocolate cake cookies e (per row)
number of records
2500
2000
1500
1000
500
0
605
1025
548
2485
514
oducts in the last 7 days
Within the past 2 to 3 days
Within the past 4 to 7 days
Yesterday, 1 to 2 times
Yesterday, 3 or more times
1420 NAs

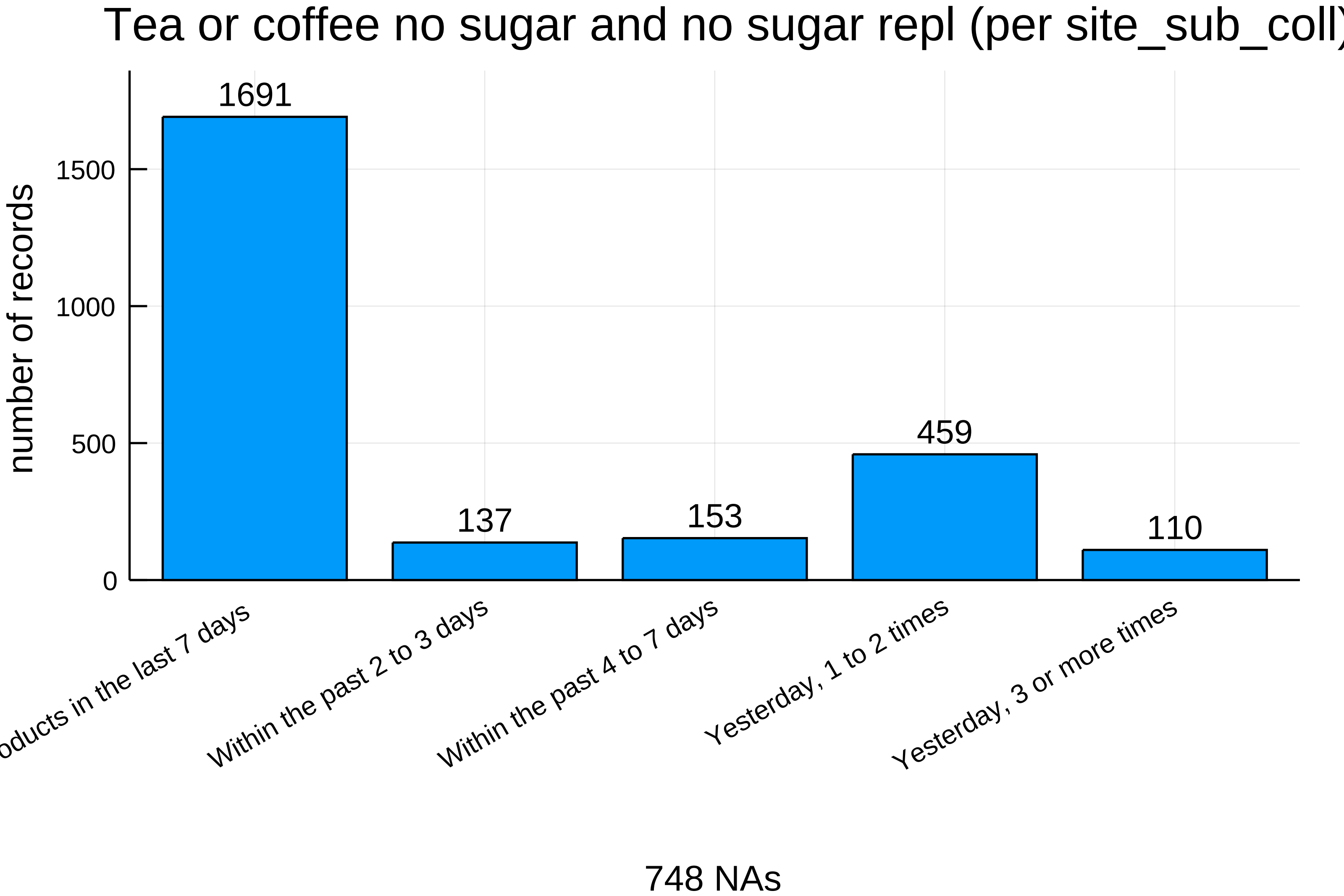
Tea or coffee no sugar and no sugar repl (per site_sub_coll)
number of records
0
500
1000
1500
1691
137
153
459
110
oducts in the last 7 days
Within the past 2 to 3 days
Within the past 4 to 7 days
Yesterday, 1 to 2 times
Yesterday, 3 or more times
748 NAs

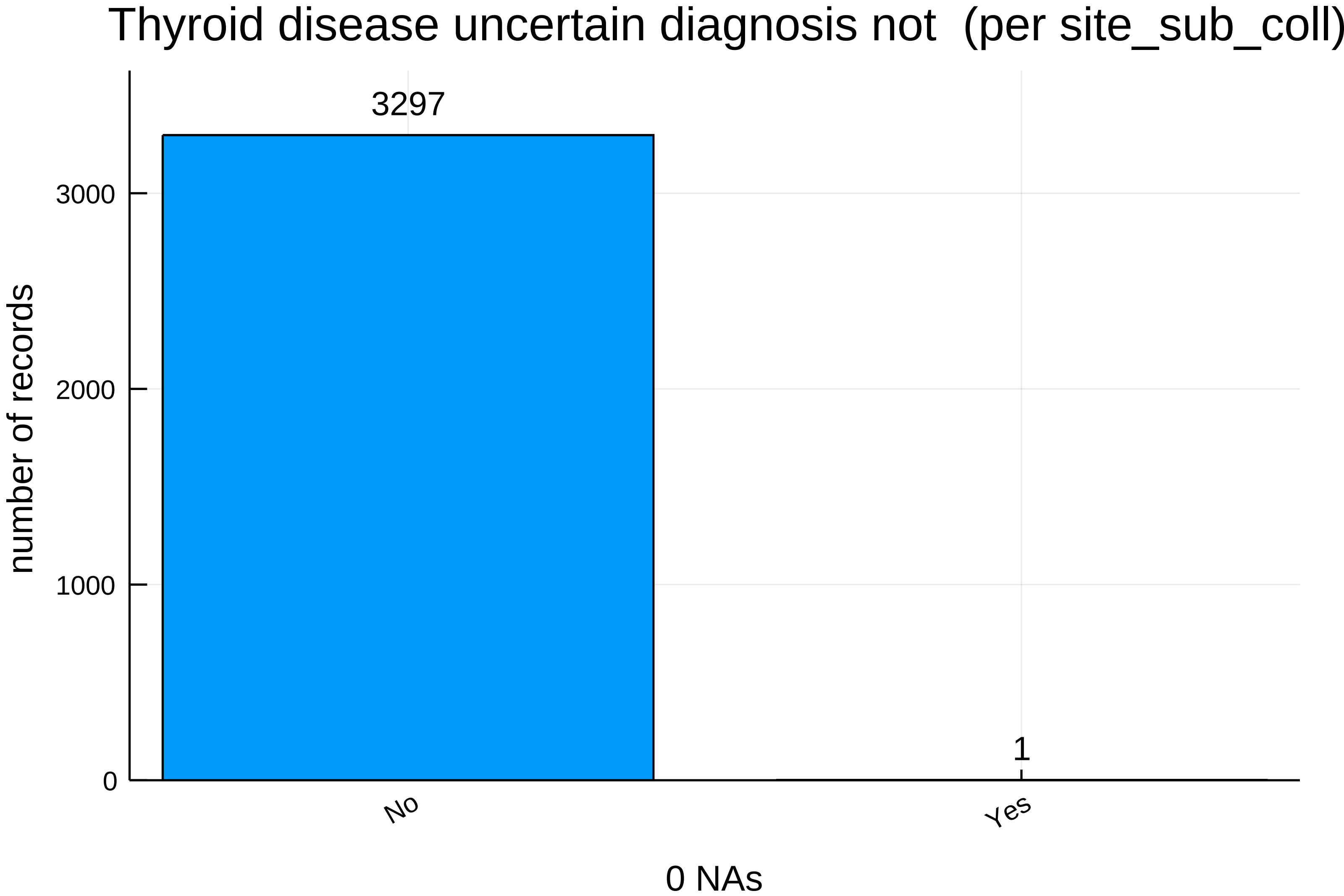
Thyroid disease uncertain diagnosis not (per site_sub_coll)
number of records
3297
1
3000
2000
1000
0
No
Yes
0 NAs

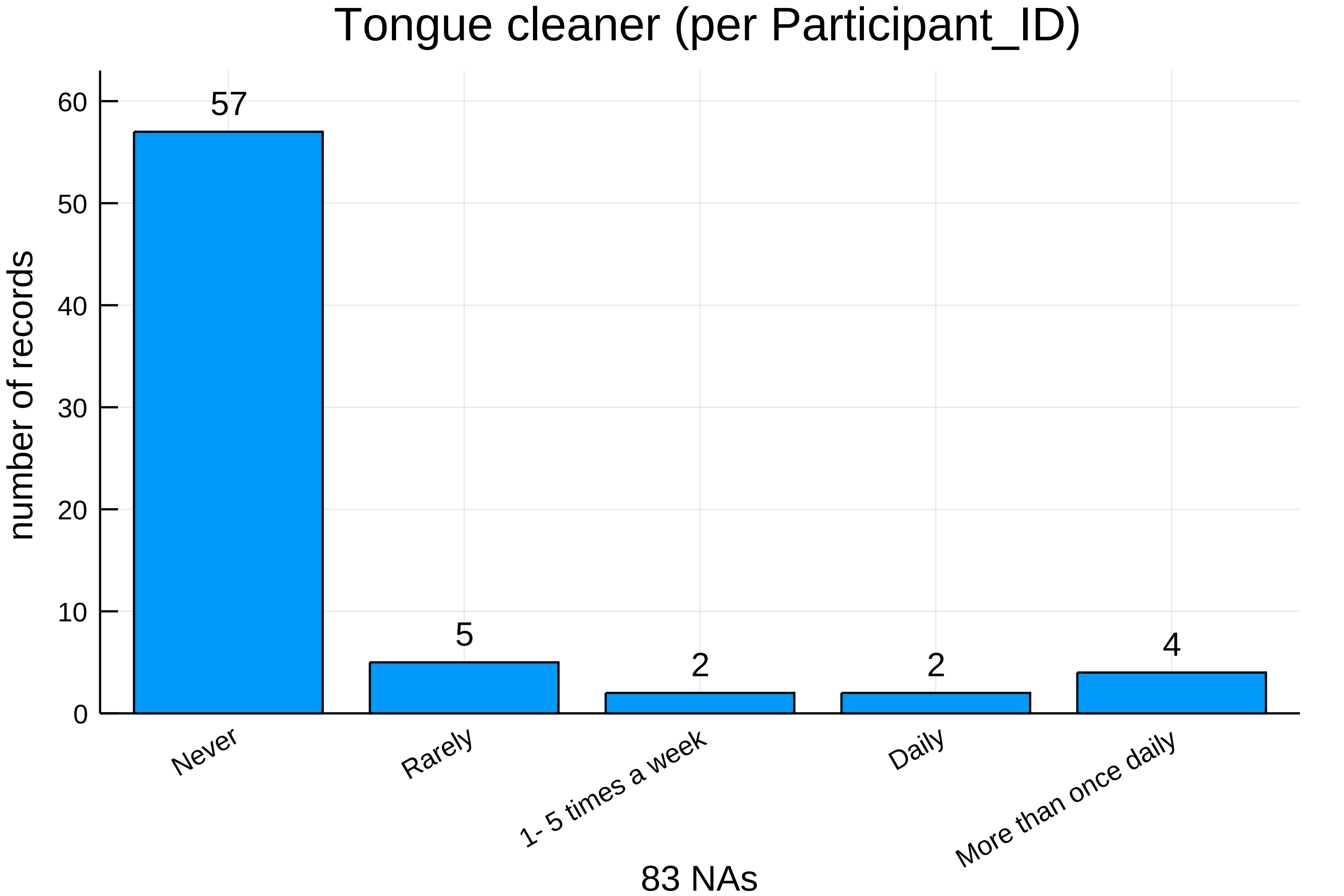
Tongue cleaner (per Participant_ID)
number of records
60
50
40
30
20
10
0
57
5
2
2
4
Never
Rarely
1- 5 times a week
Daily
More than once daily
83 NAs

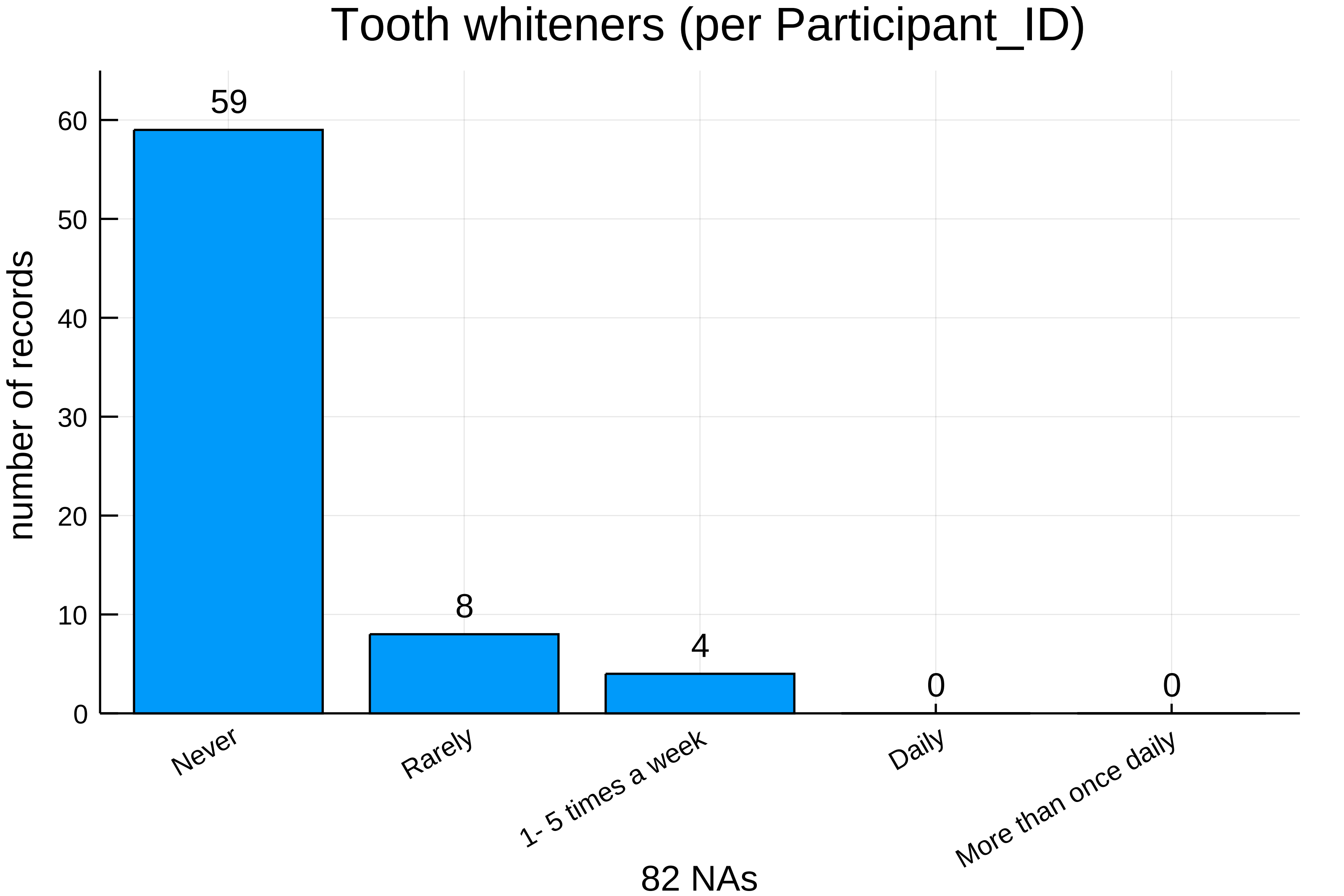
Tooth whiteners (per Participant_ID)
number of records
0
10
20
30
40
50
60
59
8
4
0
0
Never
Rarely
1- 5 times a week
Daily
More than once daily
82 NAs

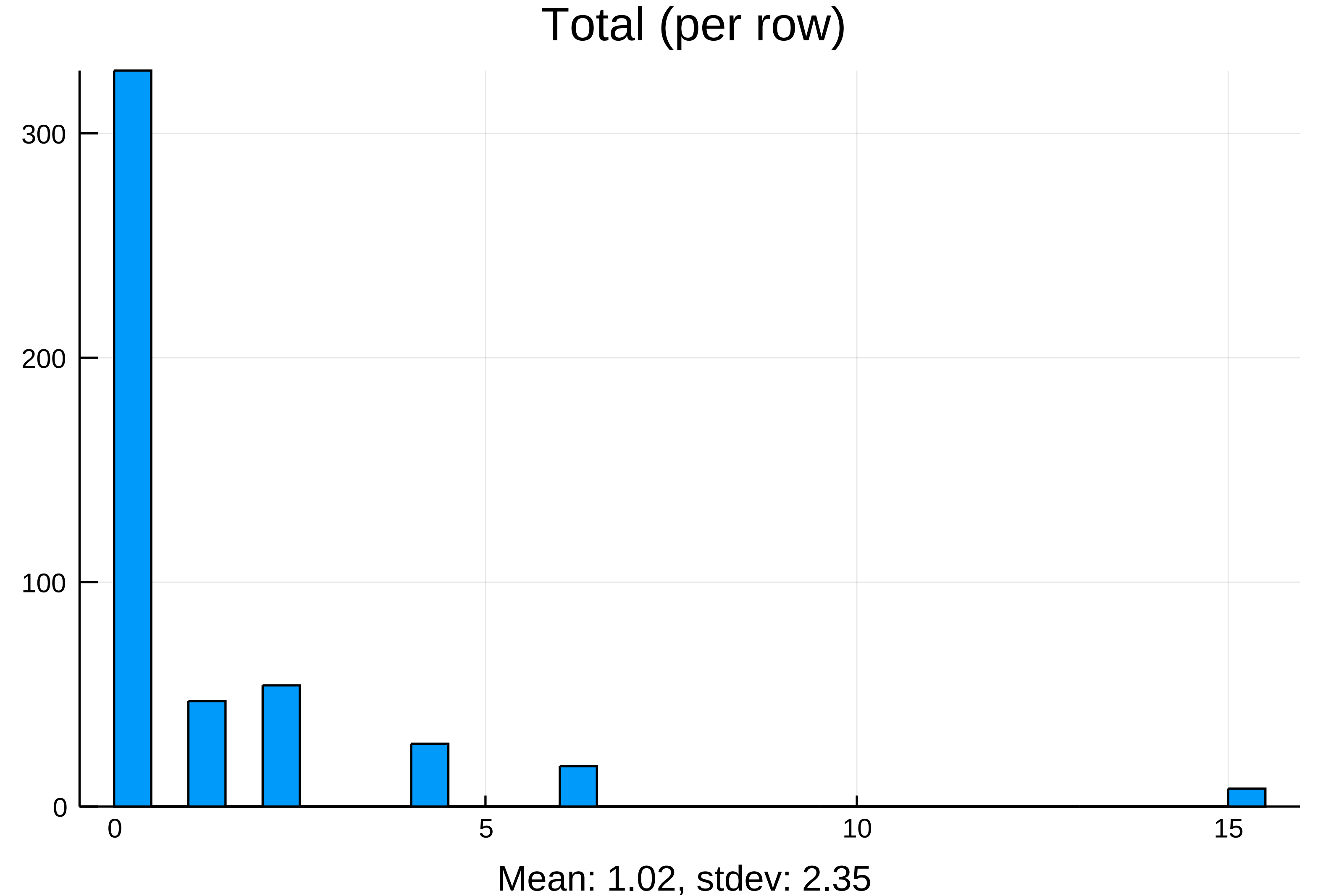
Total (per row)
300
200
100
0
0
5
10
15
Mean: 1.02, stdev: 2.35

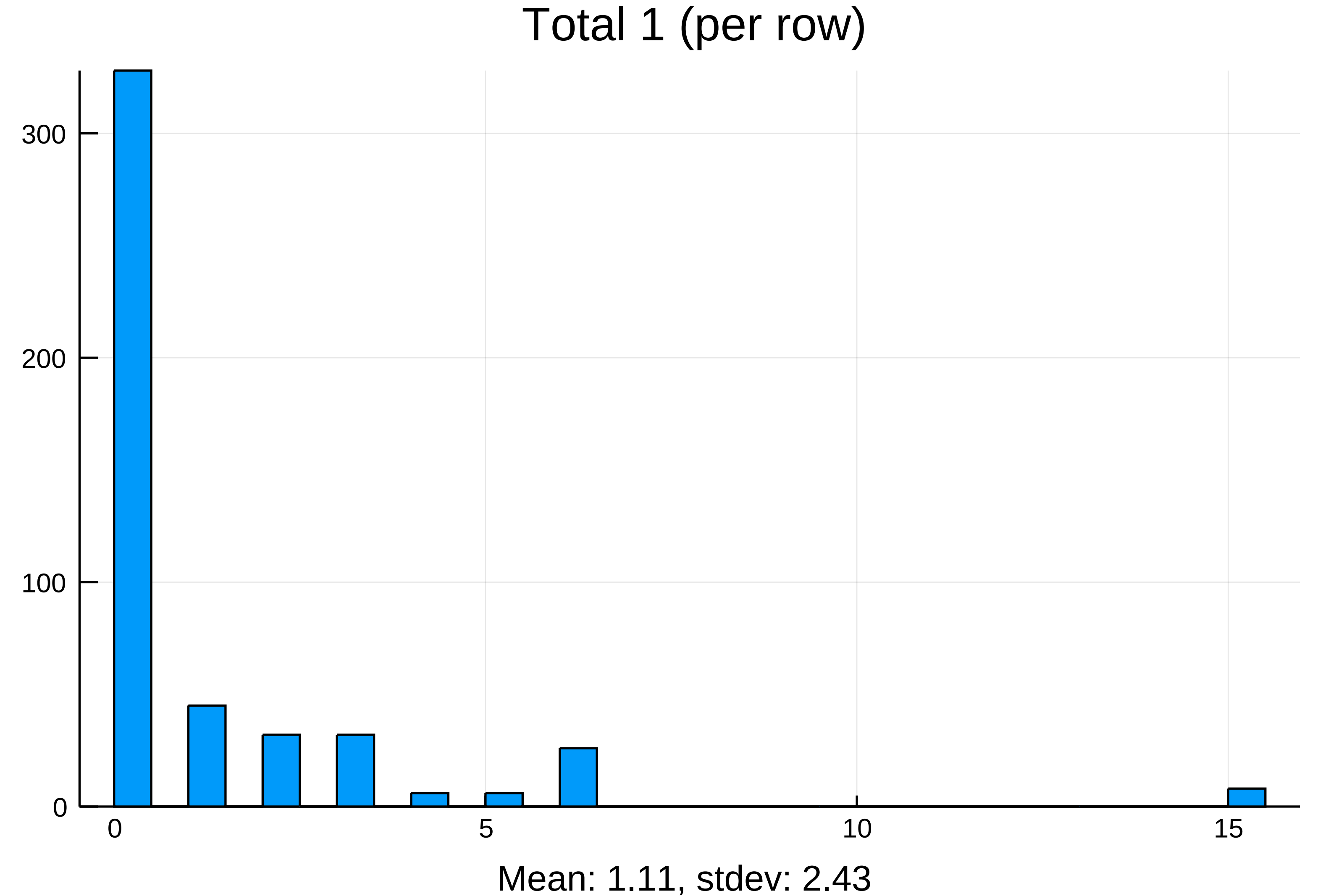
Total 1 (per row)
300
200
100
0
0
5
10
15
Mean: 1.11, stdev: 2.43

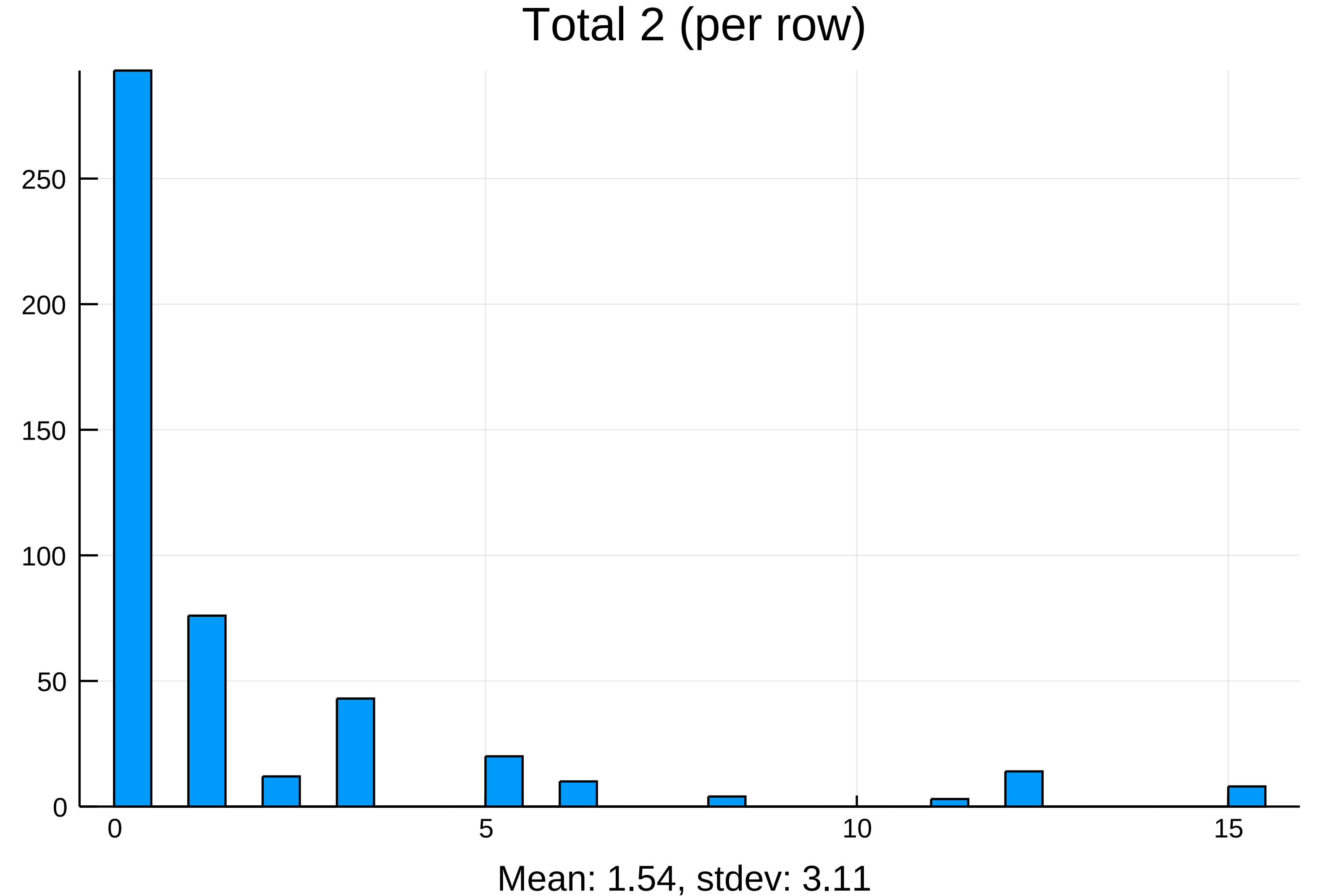
Total 2 (per row)
250
200
150
100
50
0
0
5
10
15
Mean: 1.54, stdev: 3.11

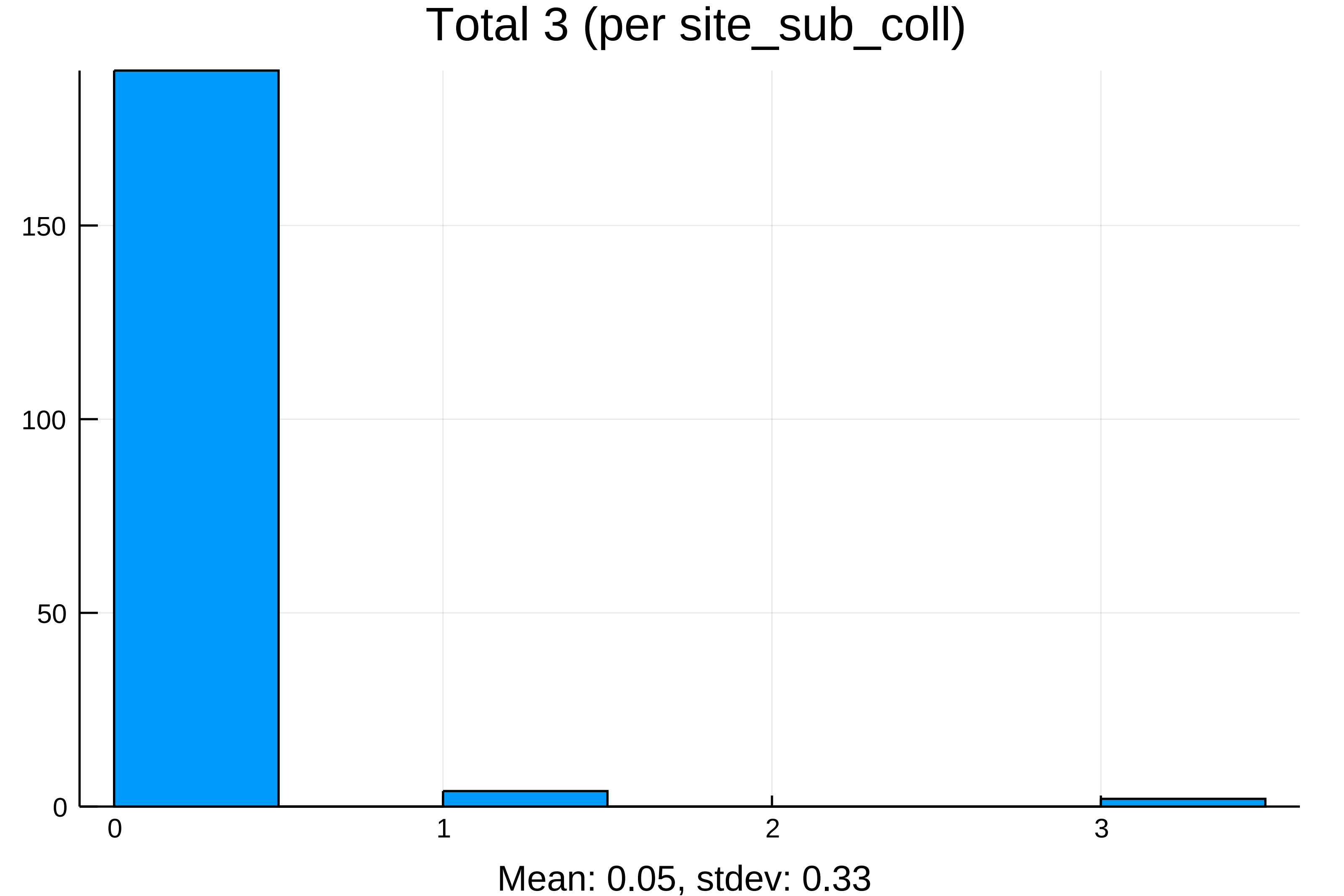
Total 3 (per site_sub_coll)
150
100
50
0
0
1
2
3
Mean: 0.05, stdev: 0.33

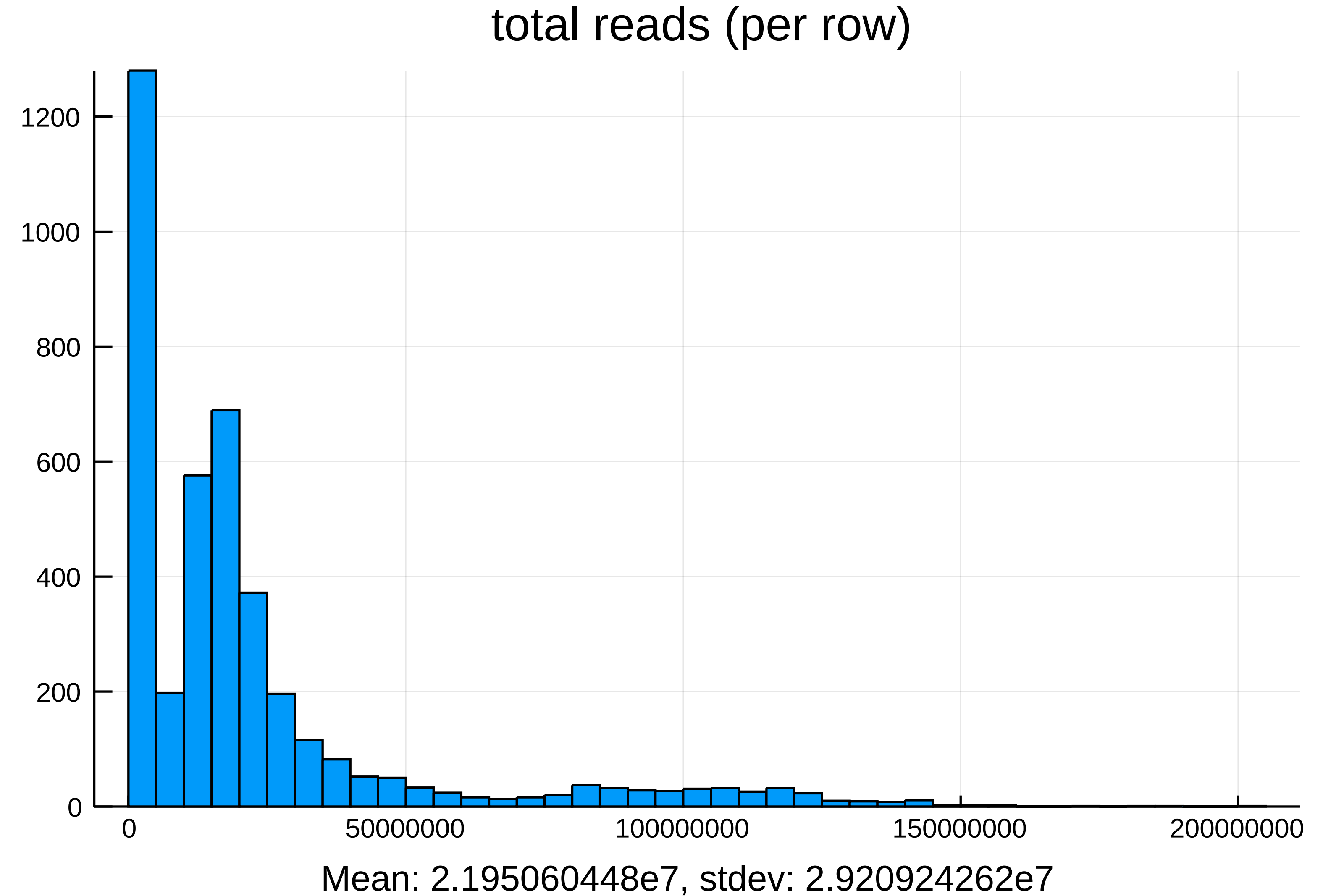
total reads (per row)
1200
1000
800
600
400
200
0
0
50000000
100000000
150000000
200000000
Mean: 2.195060448e7, stdev: 2.920924262e7

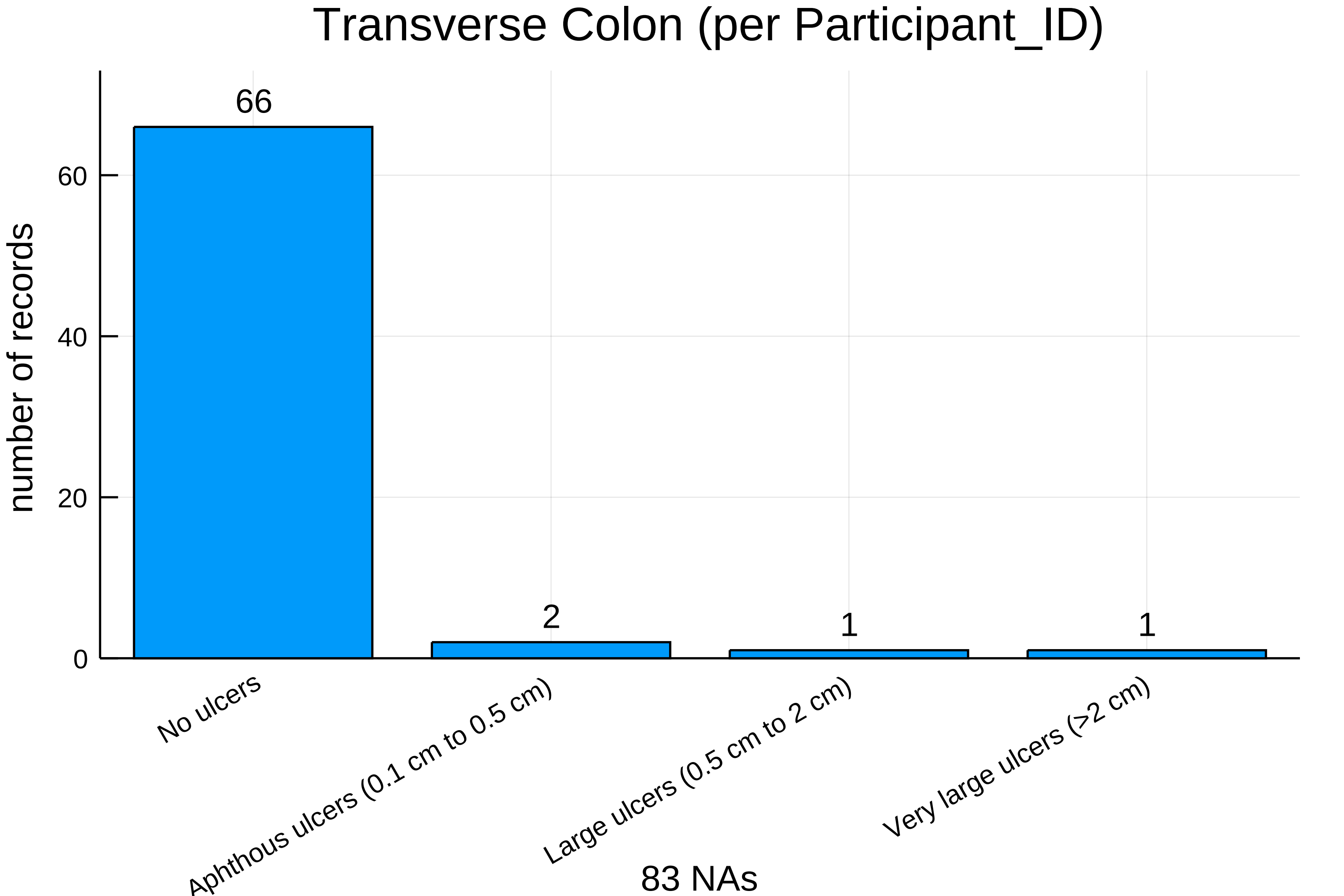
Transverse Colon (per Participant_ID)
number of records
0
20
40
60
66
2
1
1
No ulcers
Aphthous ulcers (0.1 cm to 0.5 cm)
Large ulcers (0.5 cm to 2 cm)
Very large ulcers (>2 cm)
83 NAs

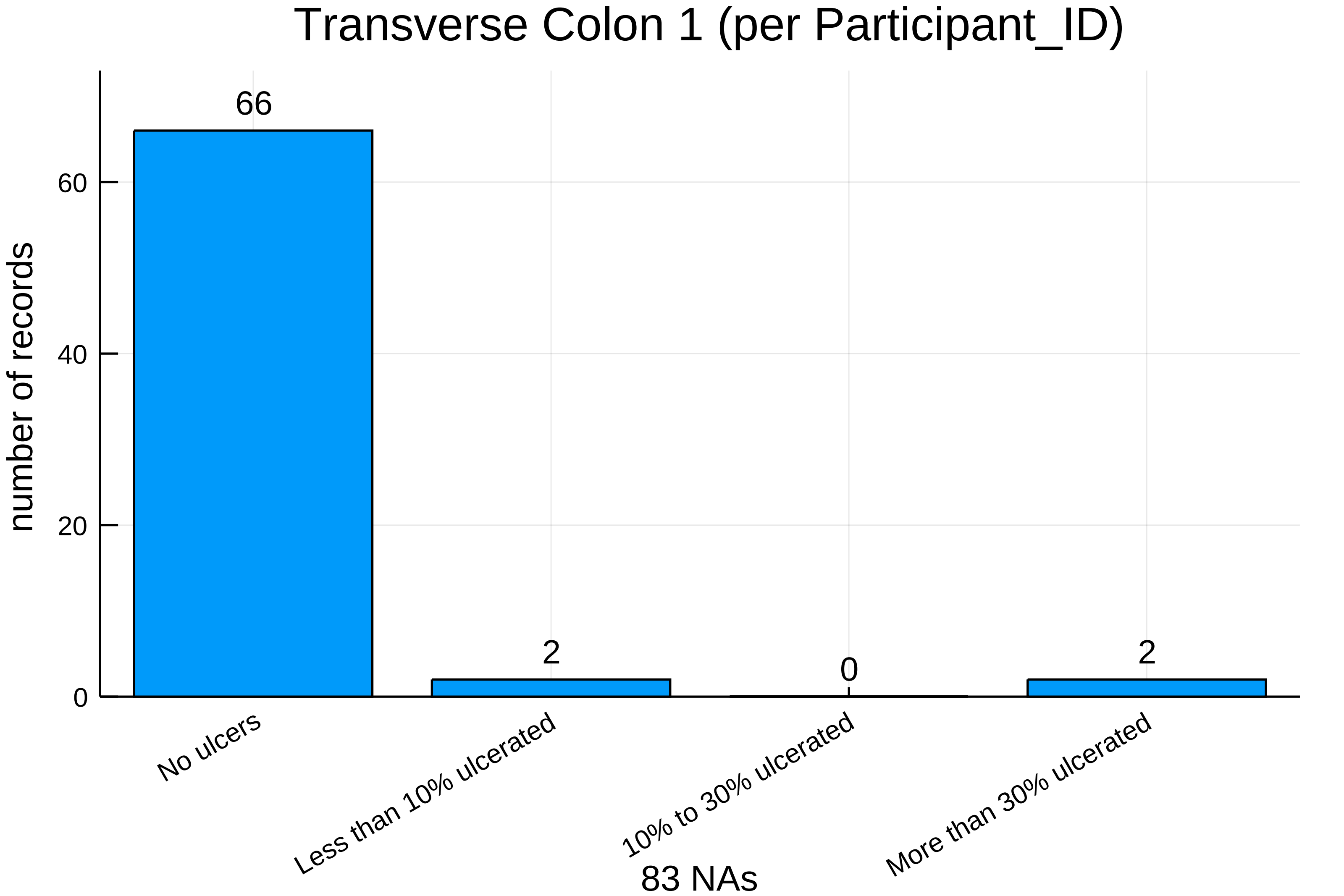

Transverse Colon 1 (per Participant_ID)
number of records
0
20
40
60
66
2
0
2
No ulcers
Less than 10% ulcerated
10% to 30% ulcerated
More than 30% ulcerated
83 NAs

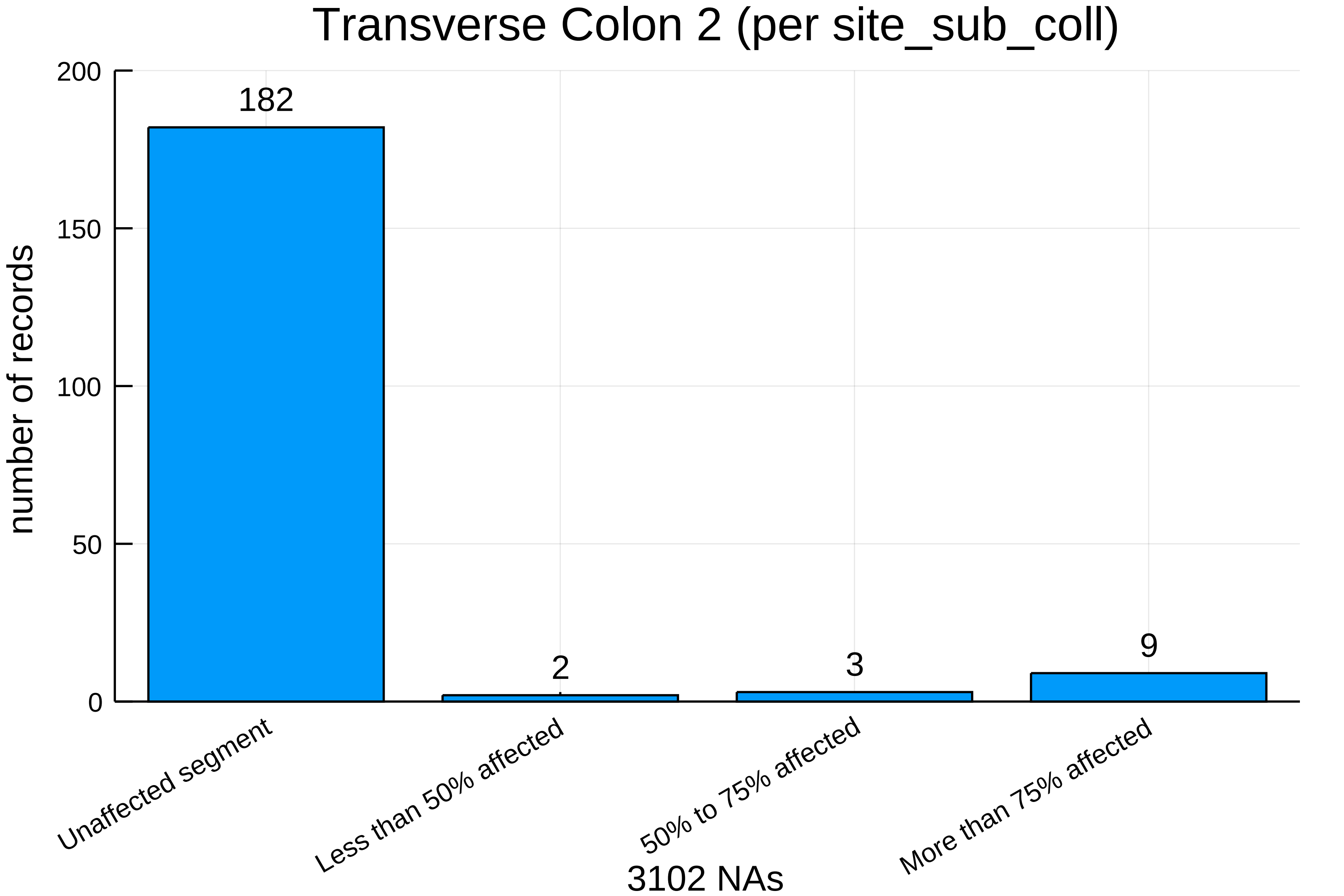

Transverse Colon 2 (per site_sub_coll)
number of records
200
150
100
50
0
182
2
3
9
Unaffected segment
Less than 50% affected
50% to 75% affected
More than 75% affected
3102 NAs

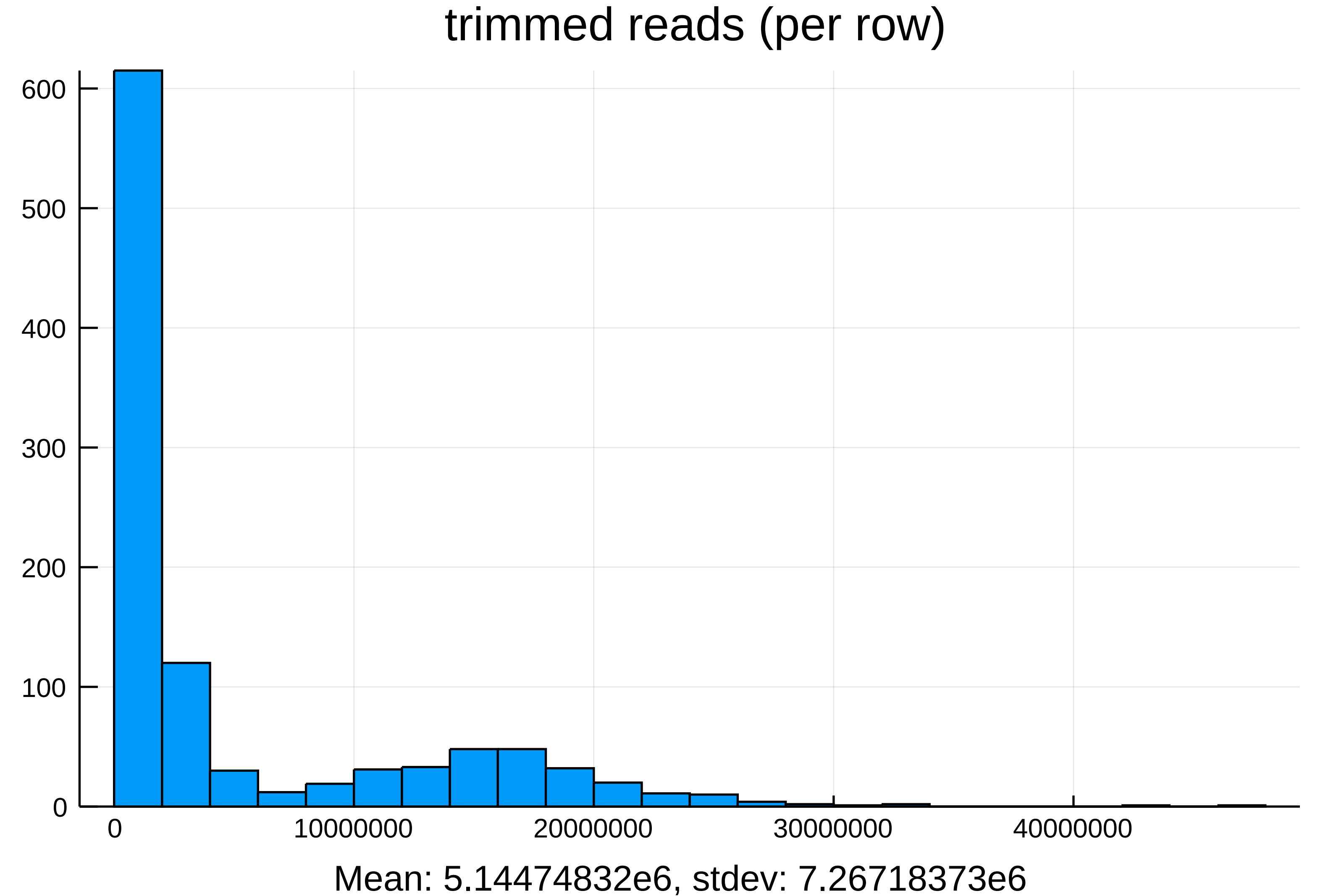
trimmed reads (per row)
600
500
400
300
200
100
0
0
10000000
20000000
30000000
40000000
Mean: 5.14474832e6, stdev: 7.26718373e6

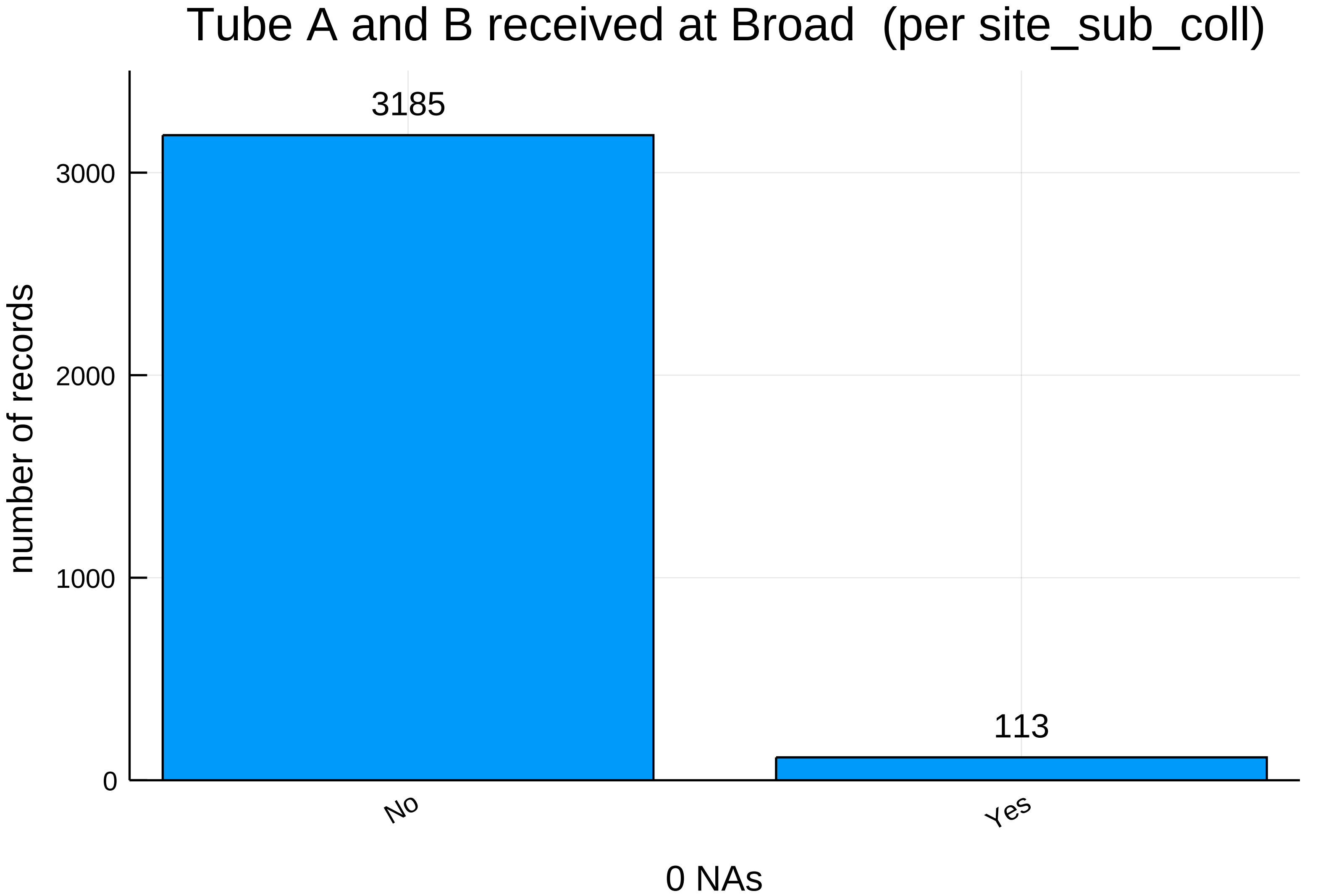
Tube A and B received at Broad (per site_sub_coll)
number of records
3000
2000
1000
0
3185
113
No
Yes
0 NAs

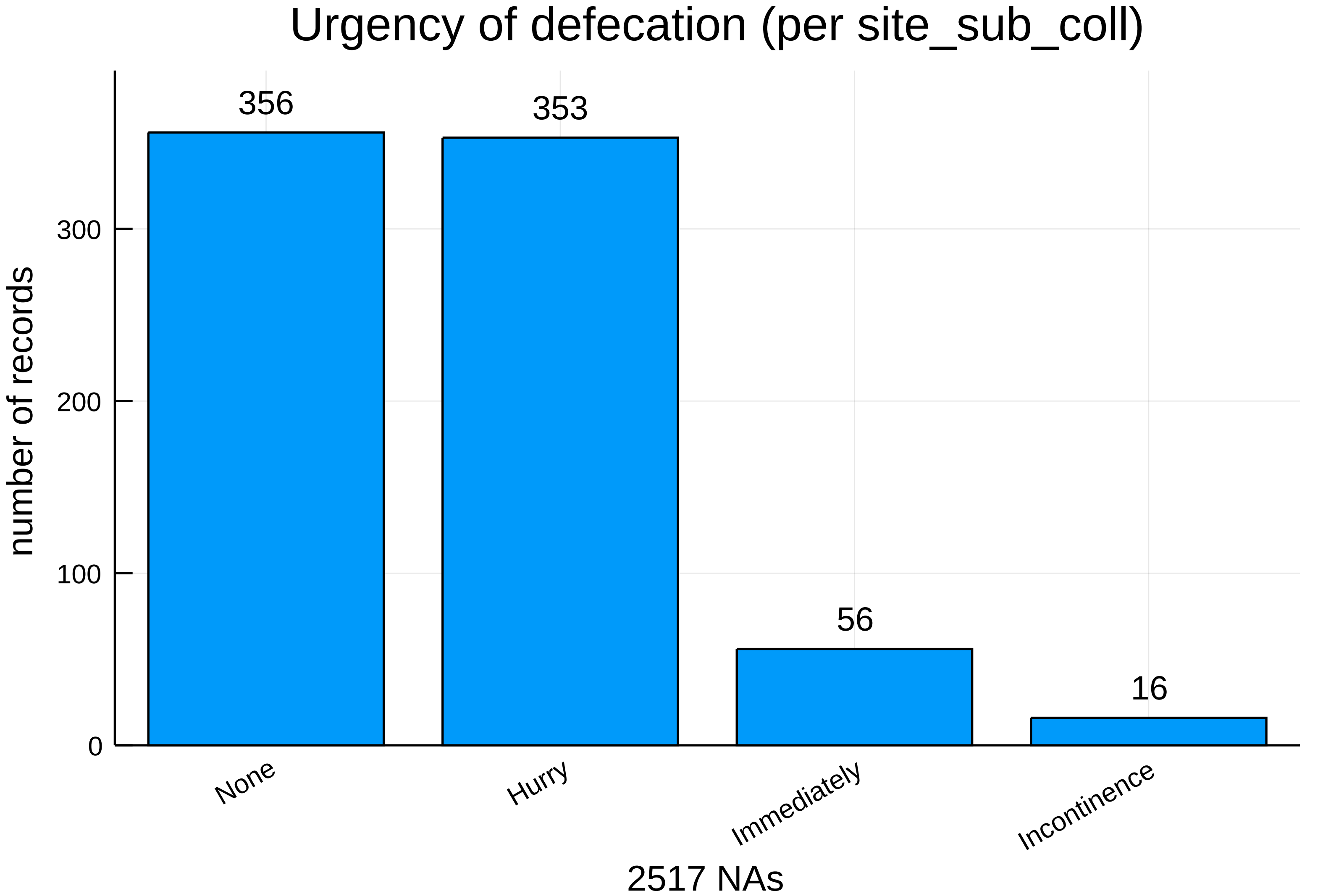

Urgency of defecation (per site_sub_coll)
number of records
0
100
200
300
356
353
56
16
None
Hurry
Immediately
Incontinence
2517 NAs

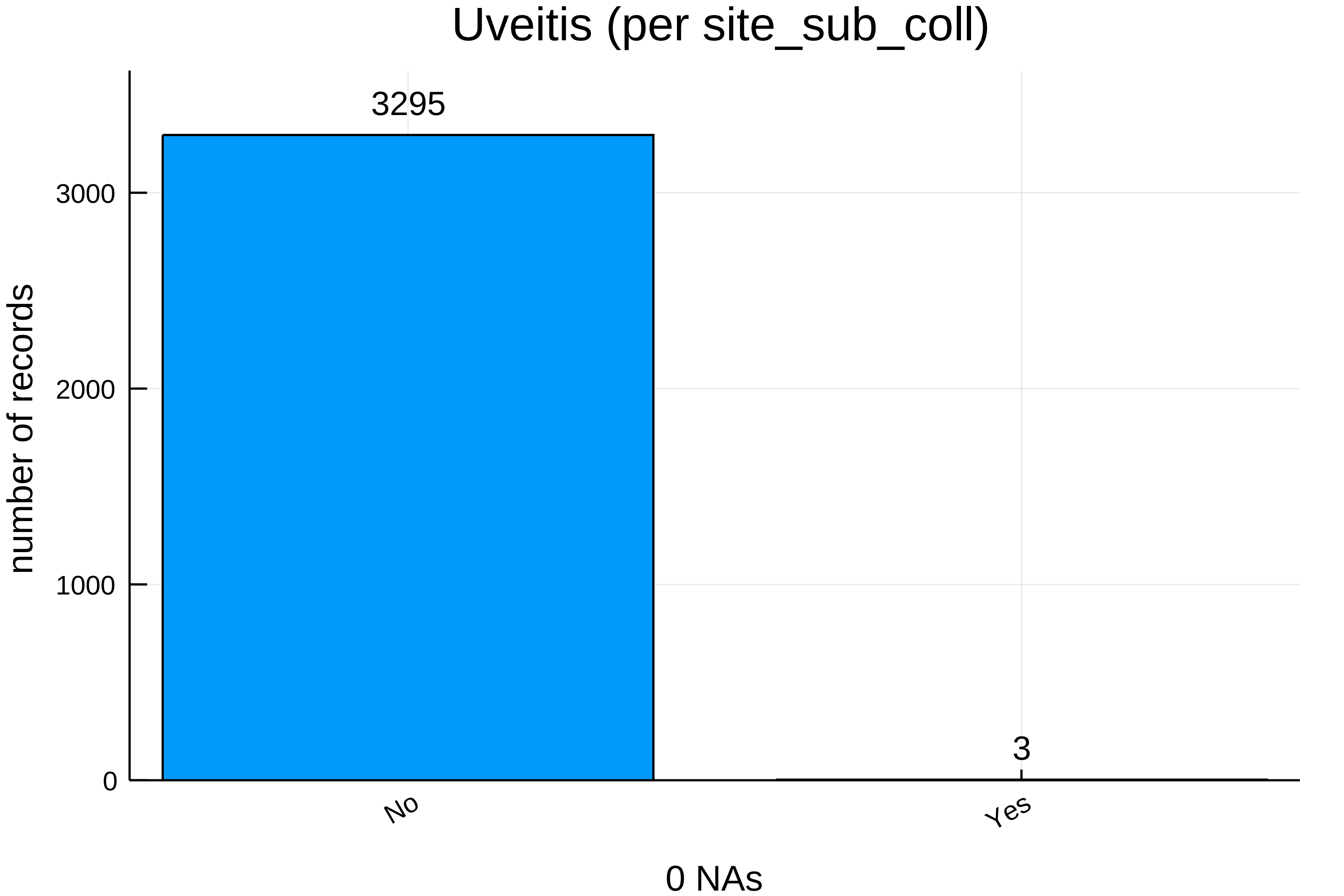

Uveitis (per site_sub_coll)
number of records
3295
3
0
1000
2000
3000
No
Yes
0 NAs

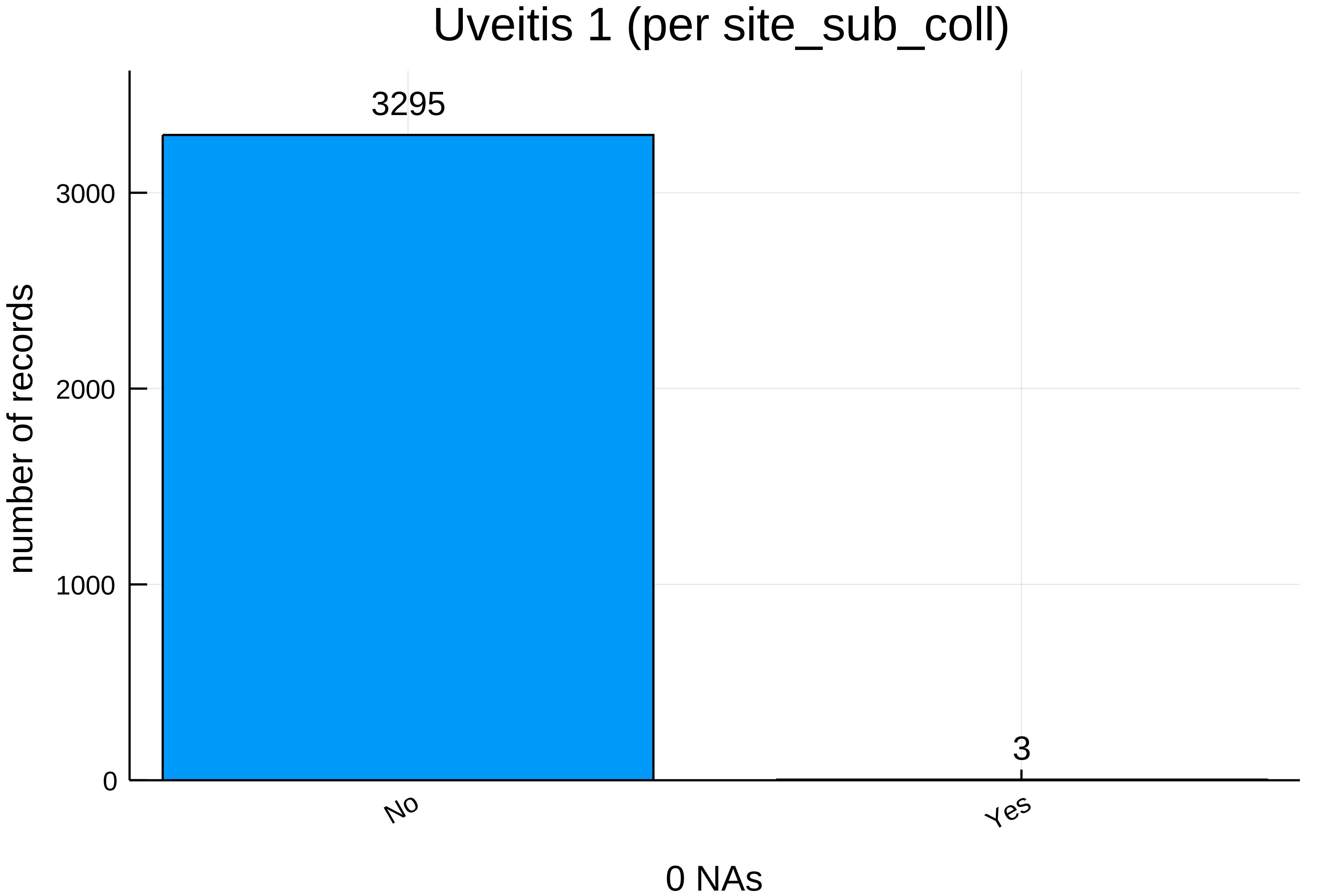
Uveitis 1 (per site_sub_coll)
3295
3
3000
2000
1000
0
number of records
No
Yes
0 NAs

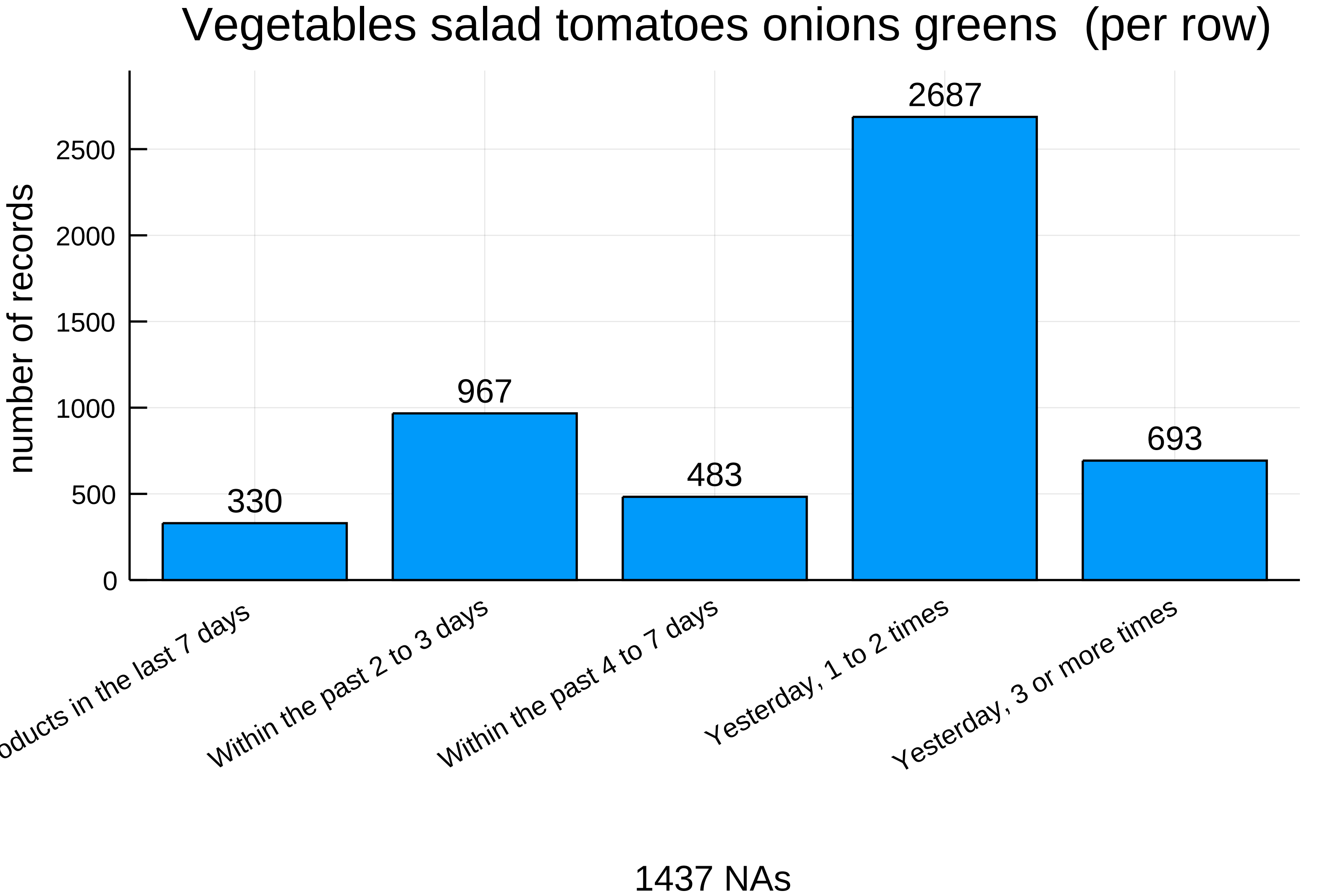

Vegetables salad tomatoes onions greens (per row)
number of records
0
500
1000
1500
2000
2500
330
967
483
2687
693
oducts in the last 7 days
Within the past 2 to 3 days
Within the past 4 to 7 days
Yesterday, 1 to 2 times
Yesterday, 3 or more times
1437 NAs

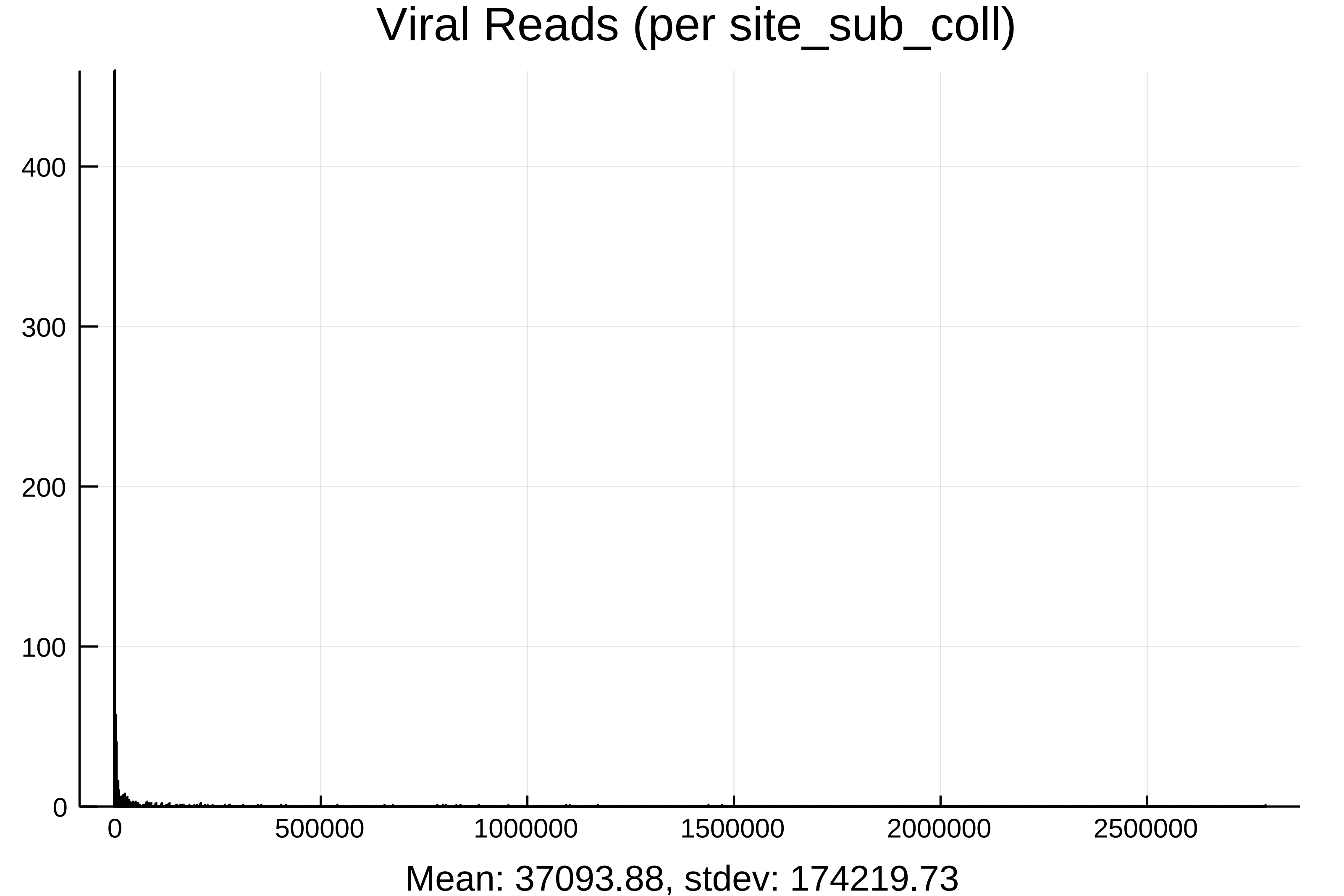
Viral Reads (per site_sub_coll)
400
300
200
100
0
0
500000
1000000
1500000
2000000
2500000
Mean: 37093.88, stdev: 174219.73

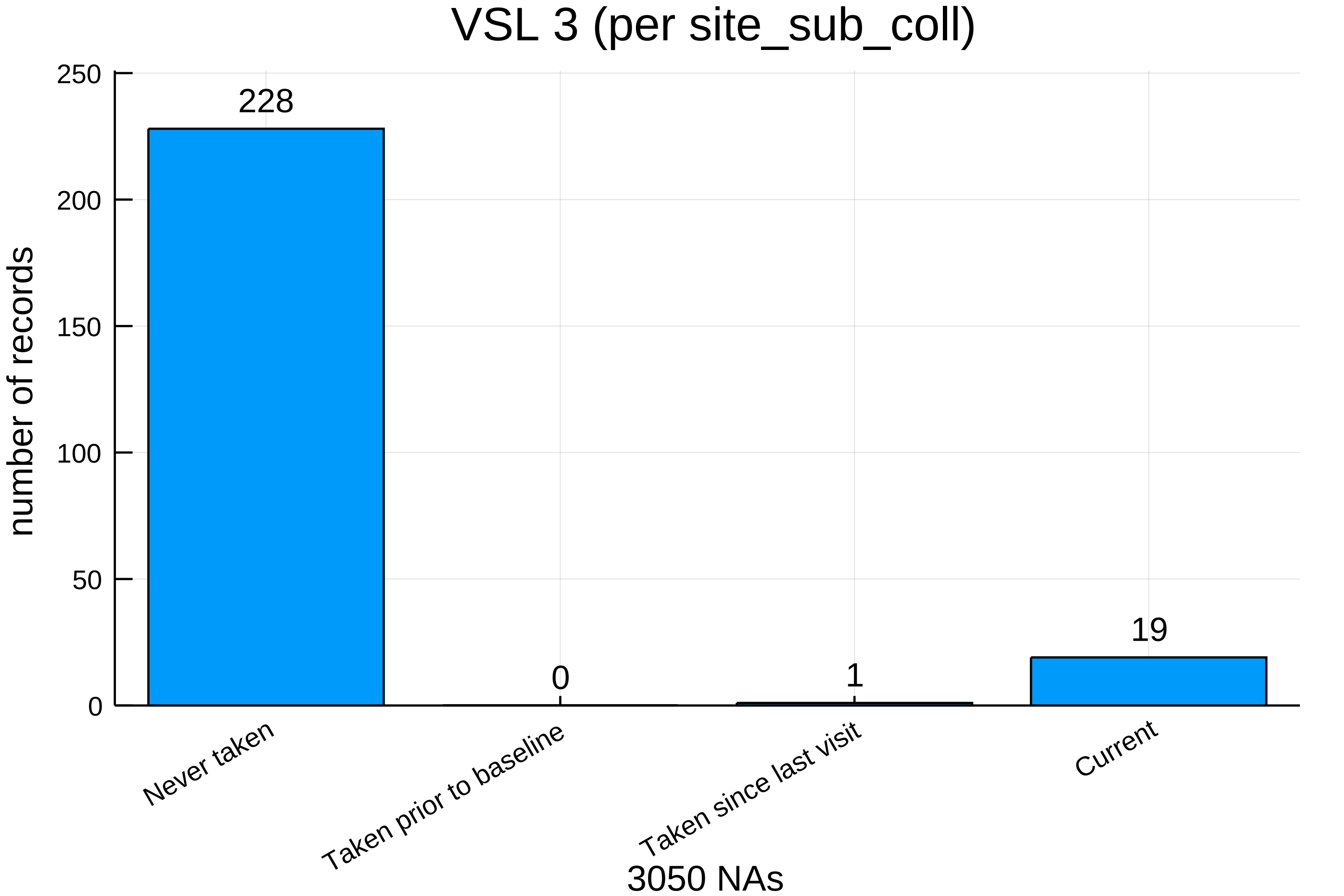
VSL 3 (per site_sub_coll)
number of records
250
200
150
100
50
0
228
0
1
19
Never taken
Taken prior to baseline
Taken since last visit
Current
3050 NAs

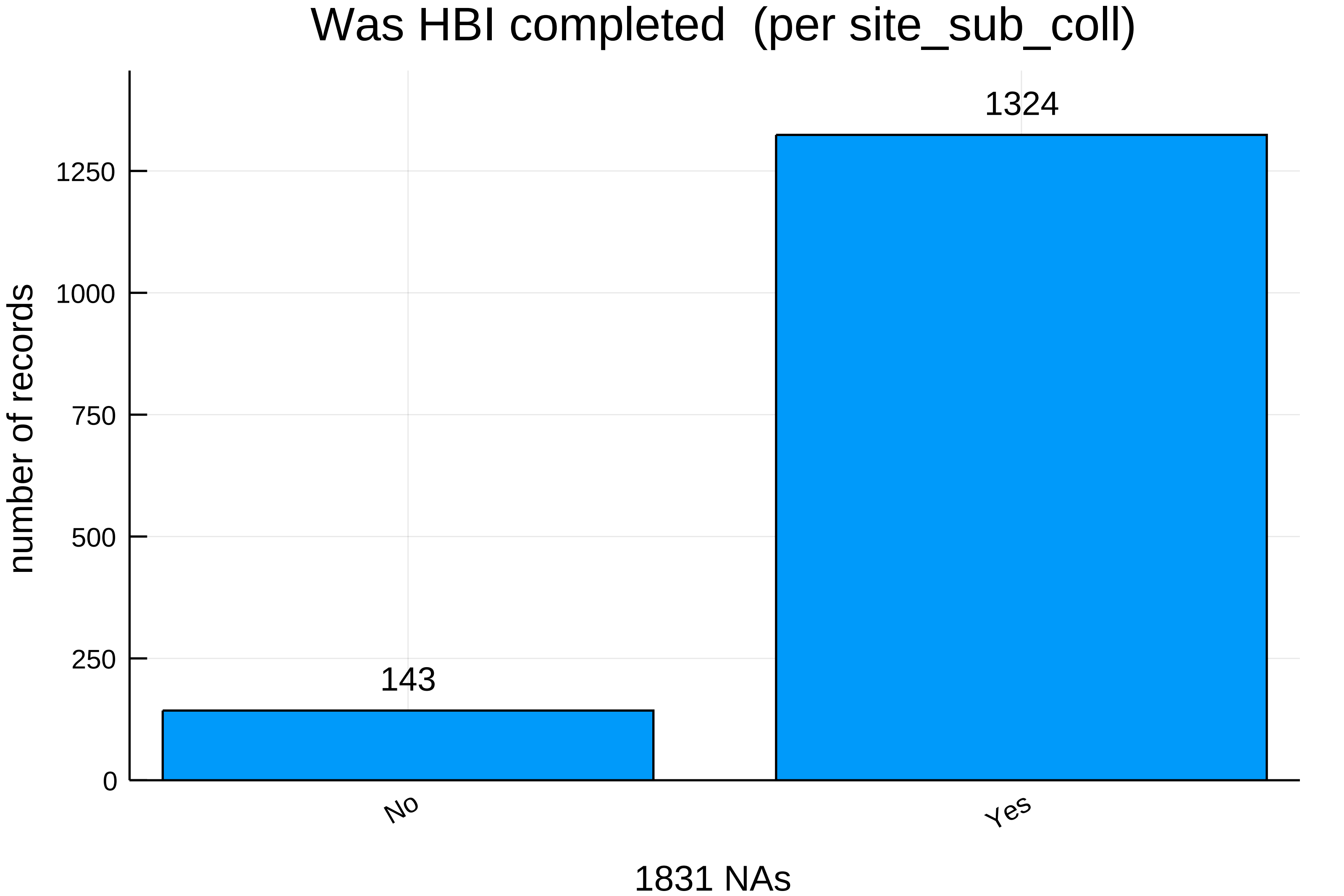
Was HBI completed (per site_sub_coll)
number of records
1250
1000
750
500
250
0
143
1324
No
Yes
1831 NAs

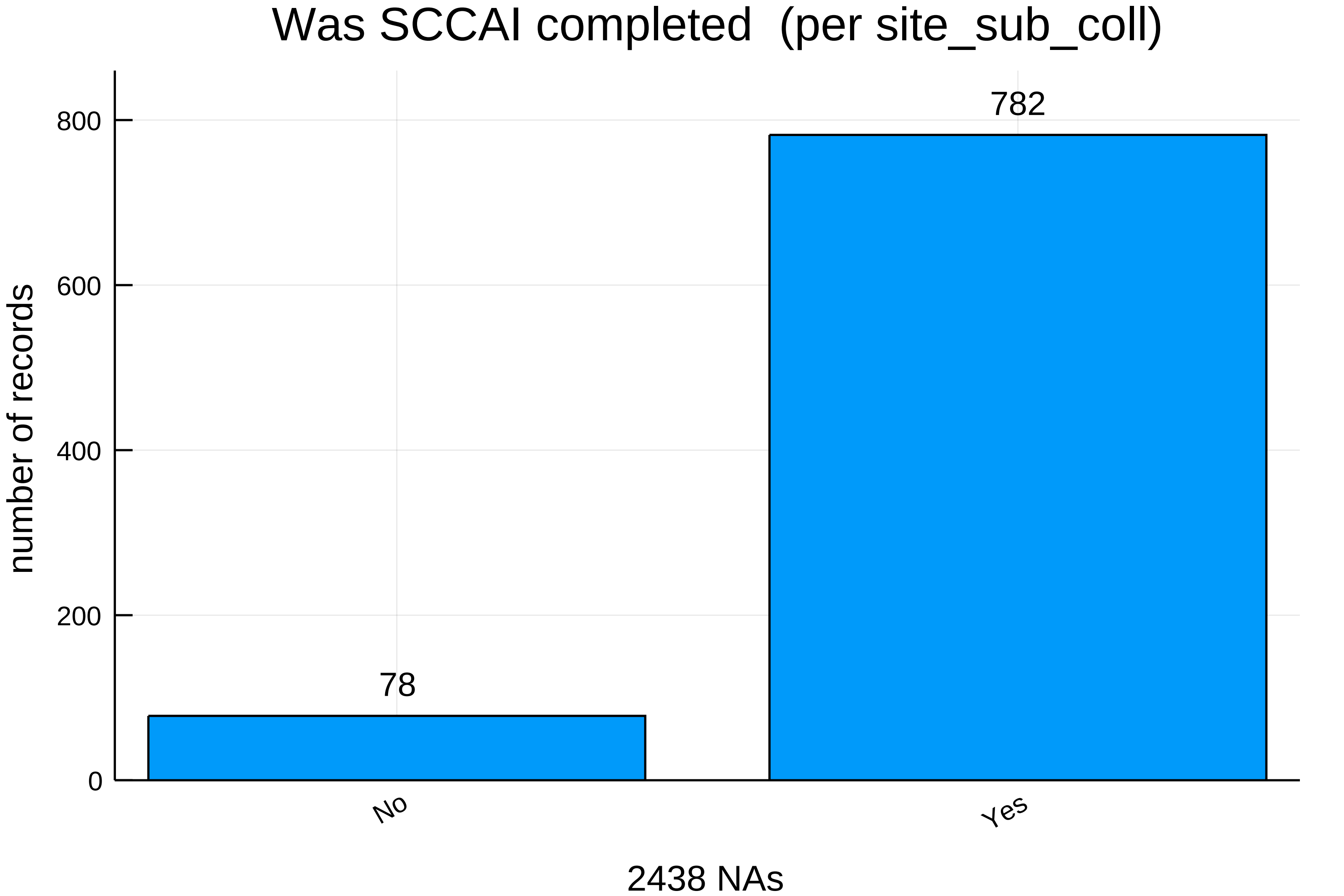
Was SCCAI completed (per site_sub_coll)
number of records
800
600
400
200
0
782
78
No
Yes
2438 NAs

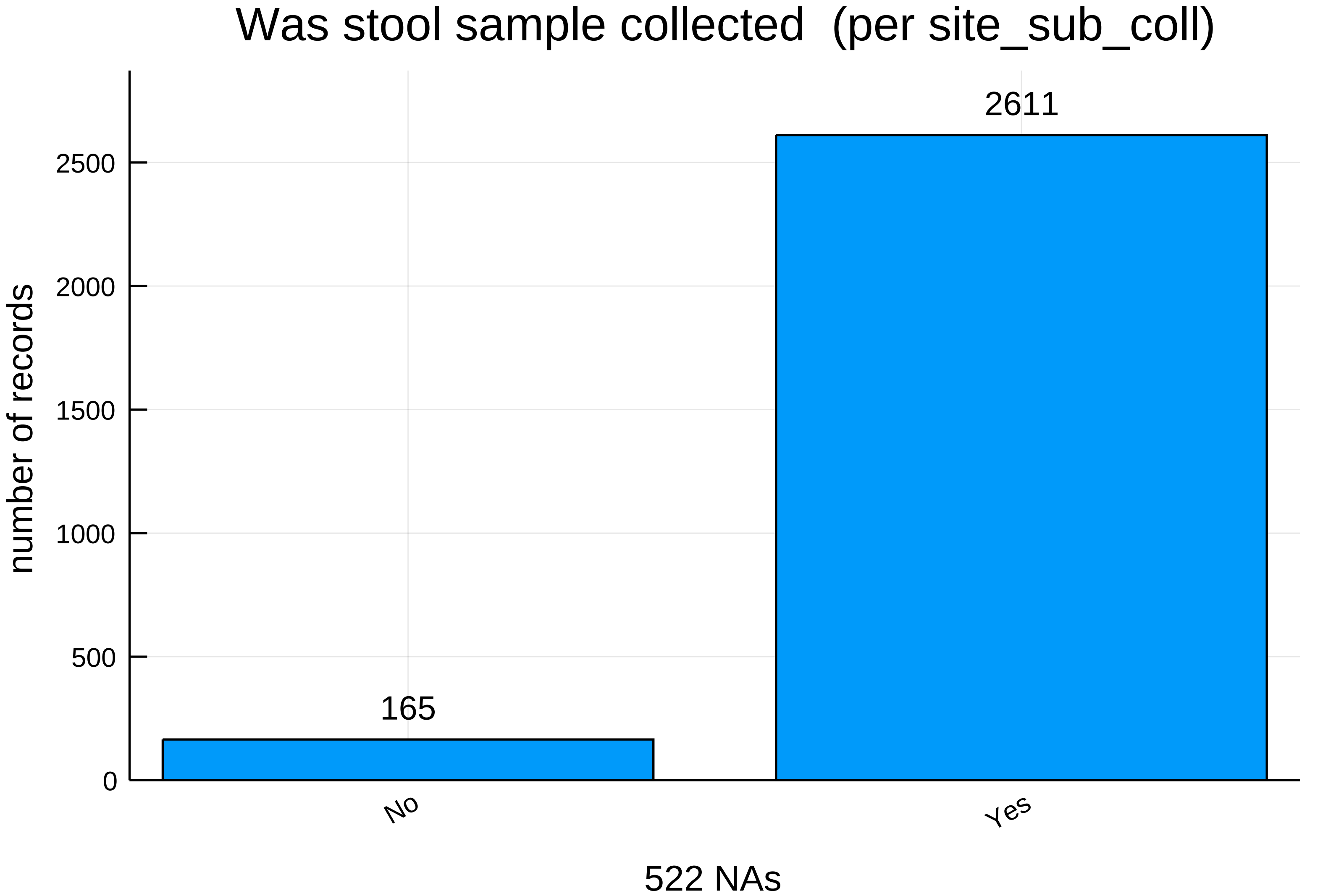
Was stool sample collected (per site_sub_coll)
number of records
2500
2000
1500
1000
500
0
165
2611
No
Yes
522 NAs

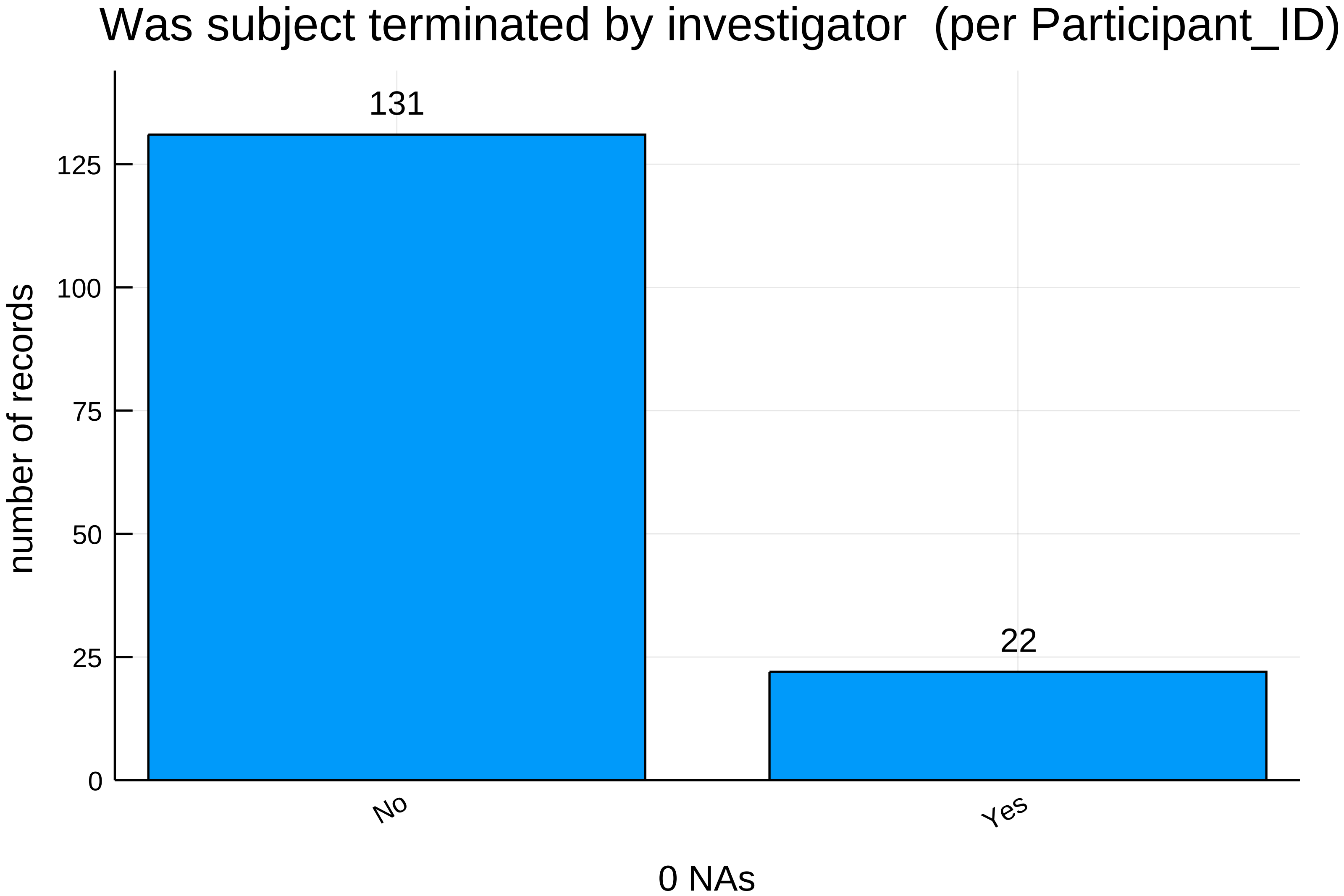
Was subject terminated by investigator (per Participant_ID)
number of records
131
22
125
100
75
50
25
0
No
Yes
0 NAs

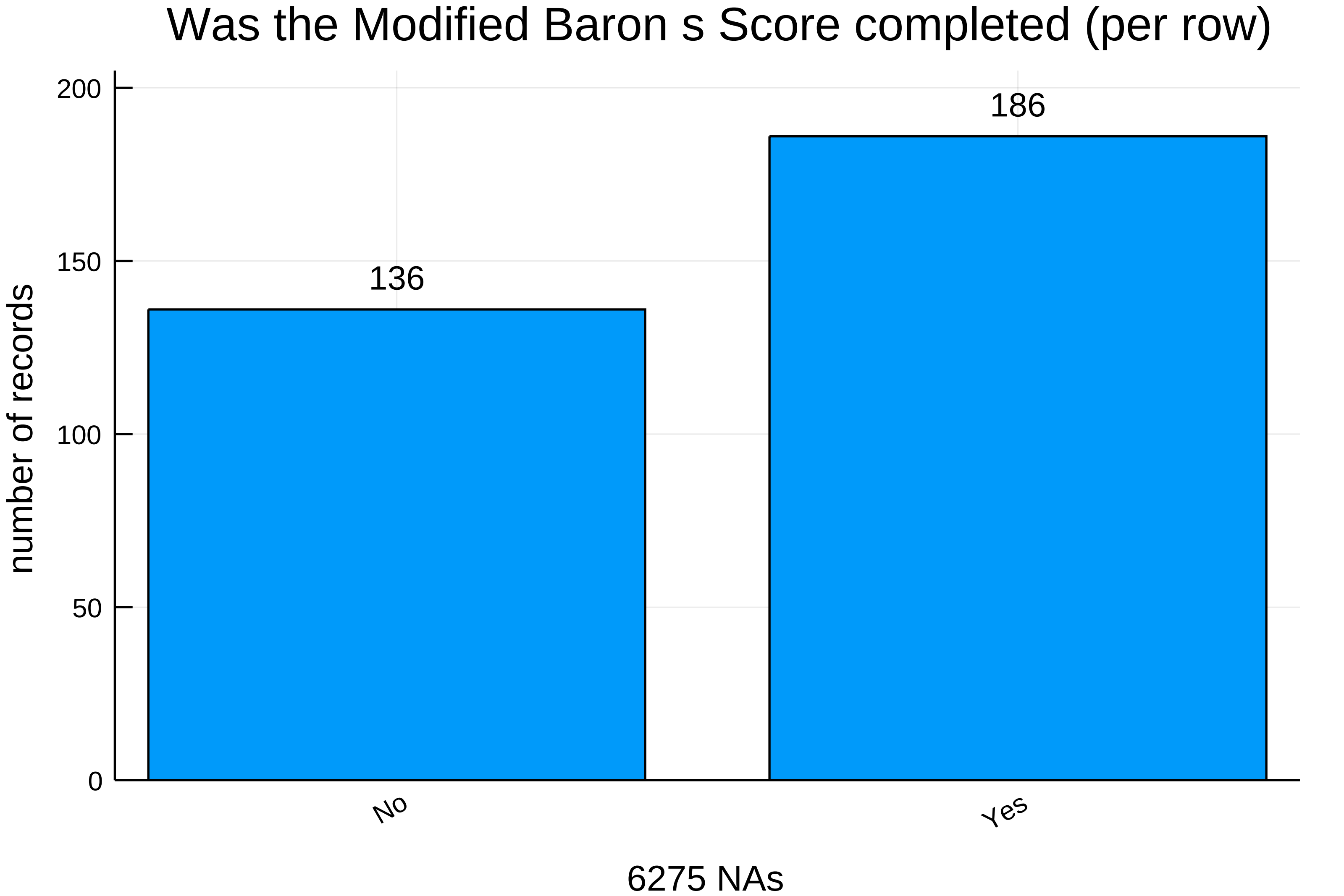
Was the Modified Baron s Score completed (per row)
number of records
200
150
100
50
0
136
186
No
Yes
6275 NAs

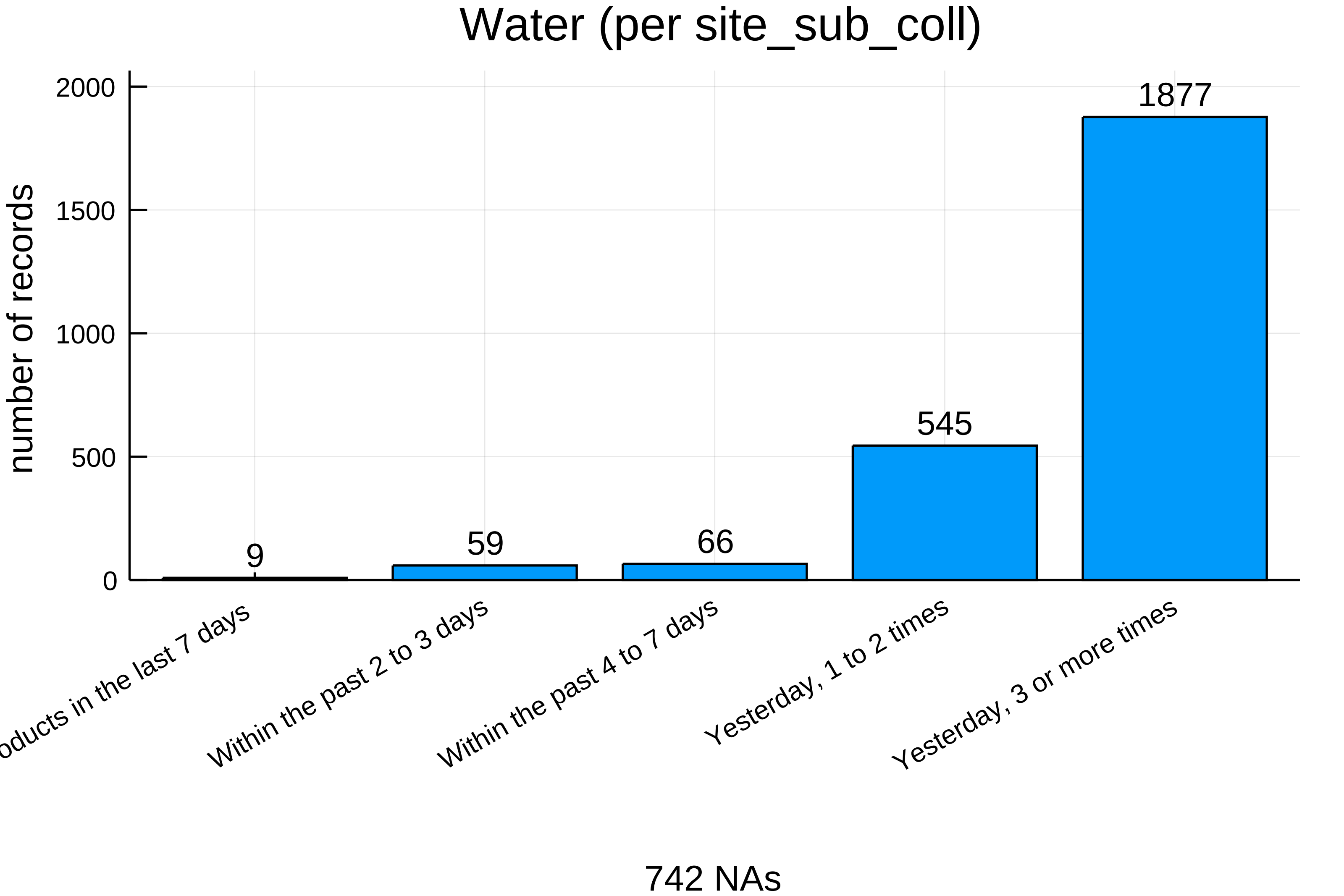
Water (per site_sub_coll)
number of records
2000
1500
1000
500
0
9
59
66
545
1877
oducts in the last 7 days
Within the past 2 to 3 days
Within the past 4 to 7 days
Yesterday, 1 to 2 times
Yesterday, 3 or more times
742 NAs

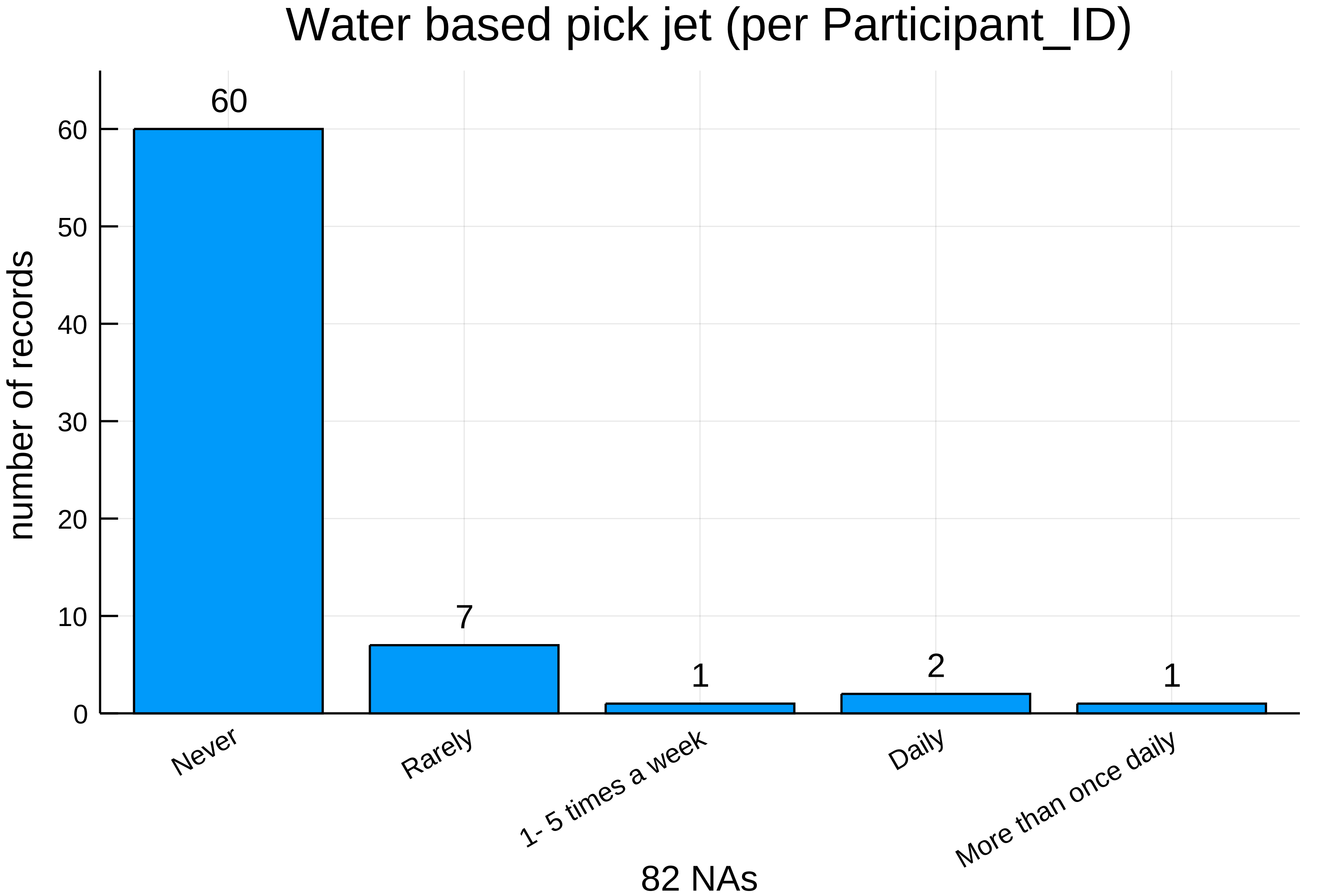
Water based pick jet (per Participant_ID)
number of records
0
10
20
30
40
50
60
60
7
1
2
1
Never
Rarely
1- 5 times a week
Daily
More than once daily
82 NAs

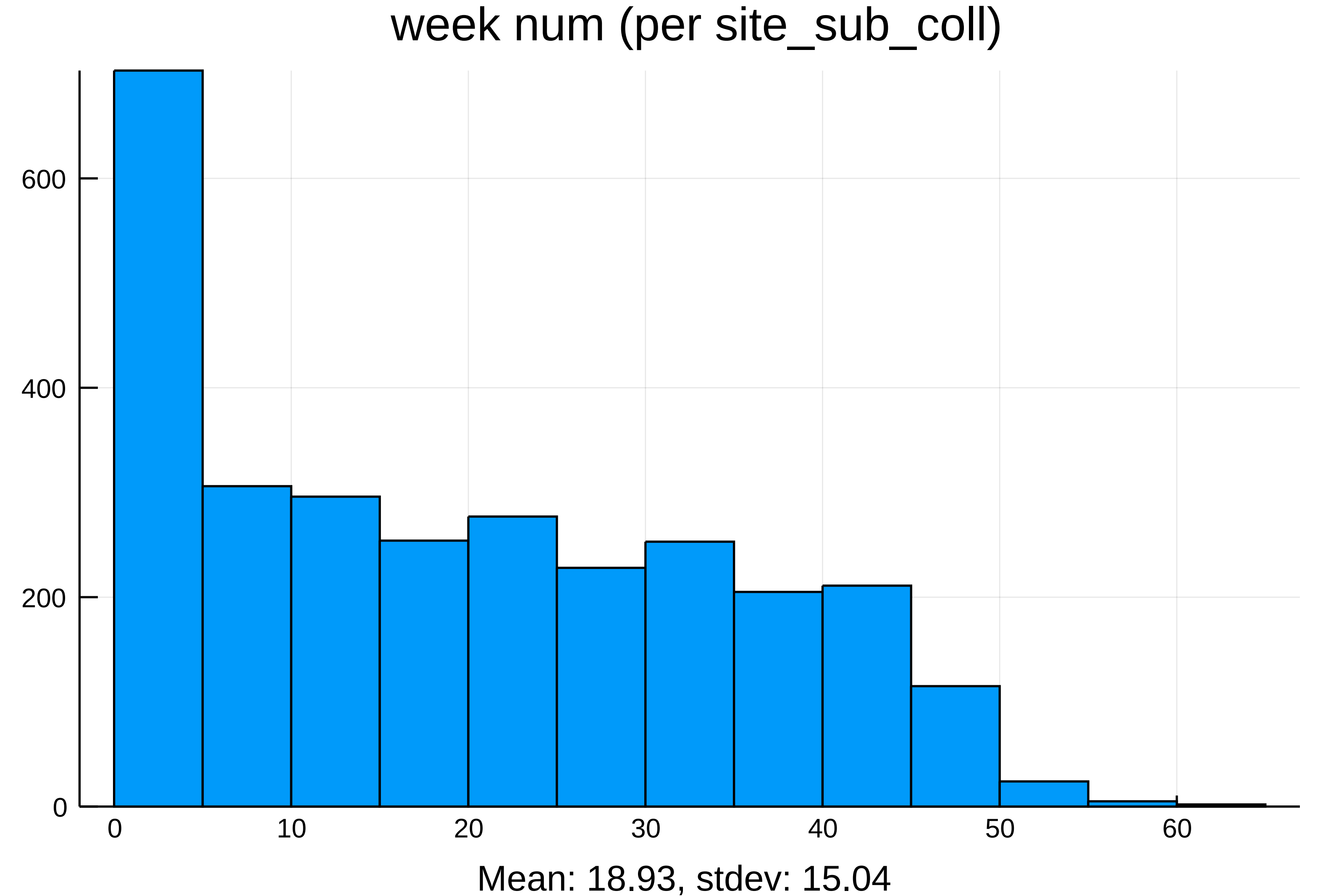
week num (per site_sub_coll)
600
400
200
0
0
10
20
30
40
50
60
Mean: 18.93, stdev: 15.04

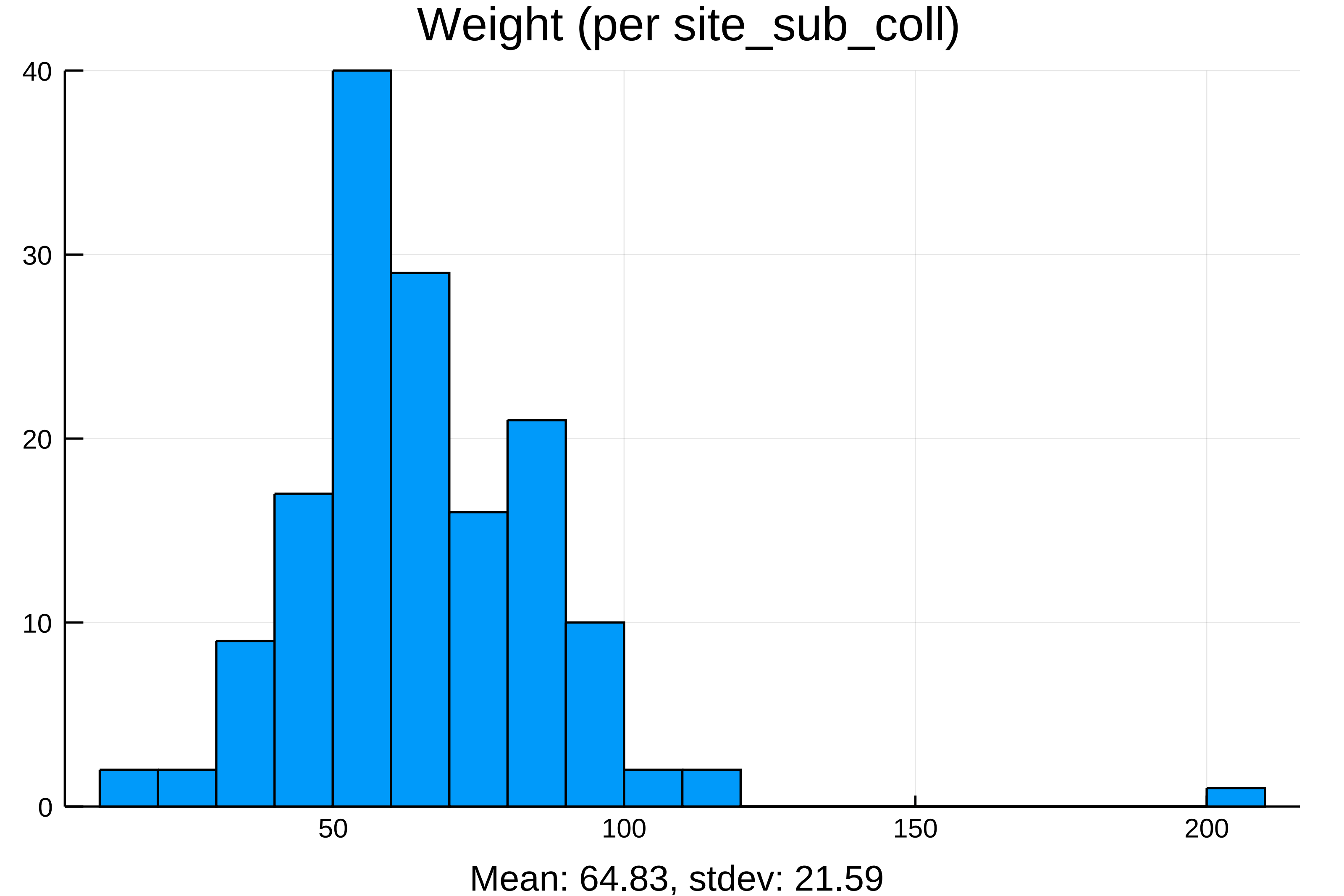
Weight (per site_sub_coll)
40
30
20
10
0
50
100
150
200
Mean: 64.83, stdev: 21.59

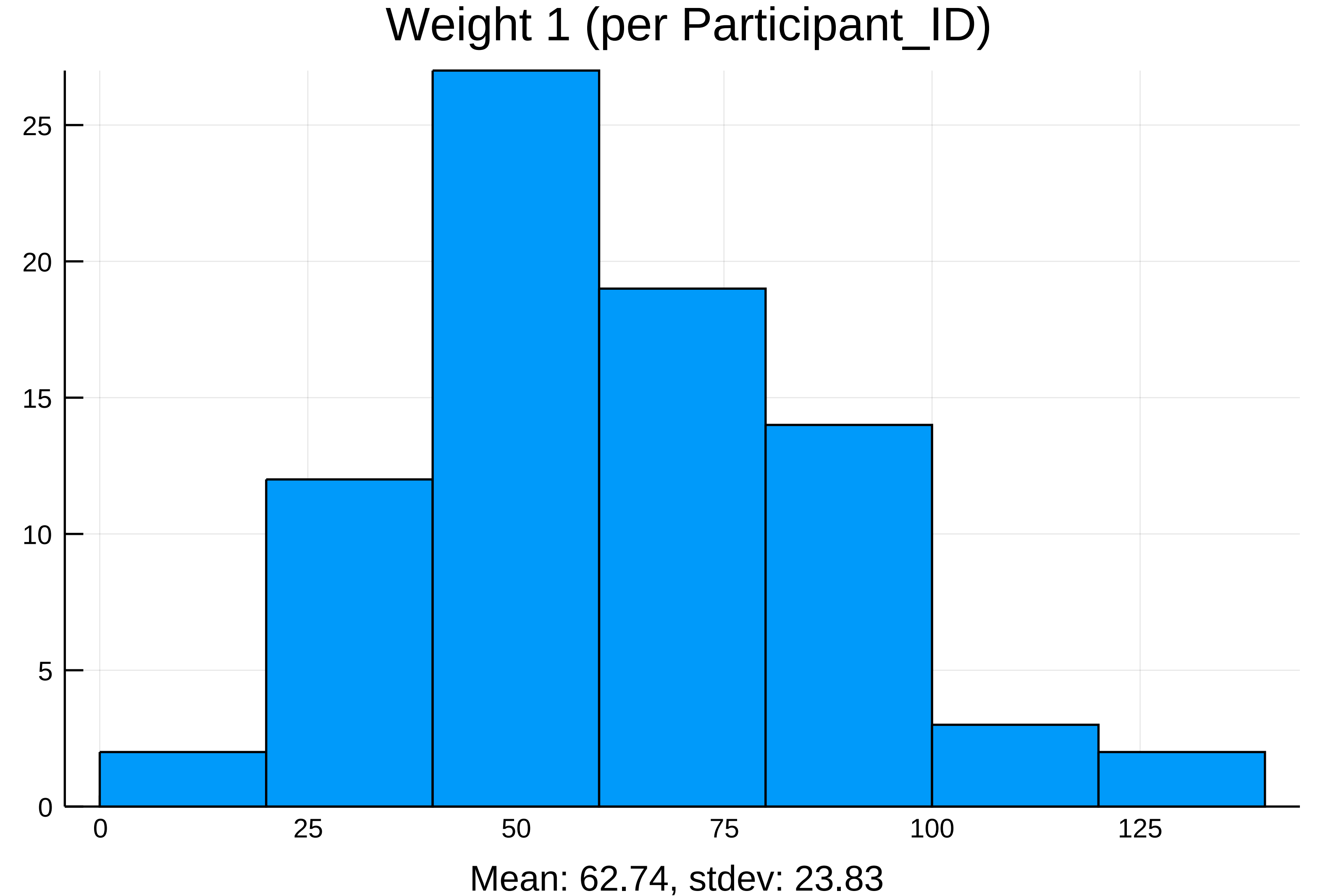
Weight 1 (per Participant_ID)
0
5
10
15
20
25
0
25
50
75
100
125
Mean: 62.74, stdev: 23.83

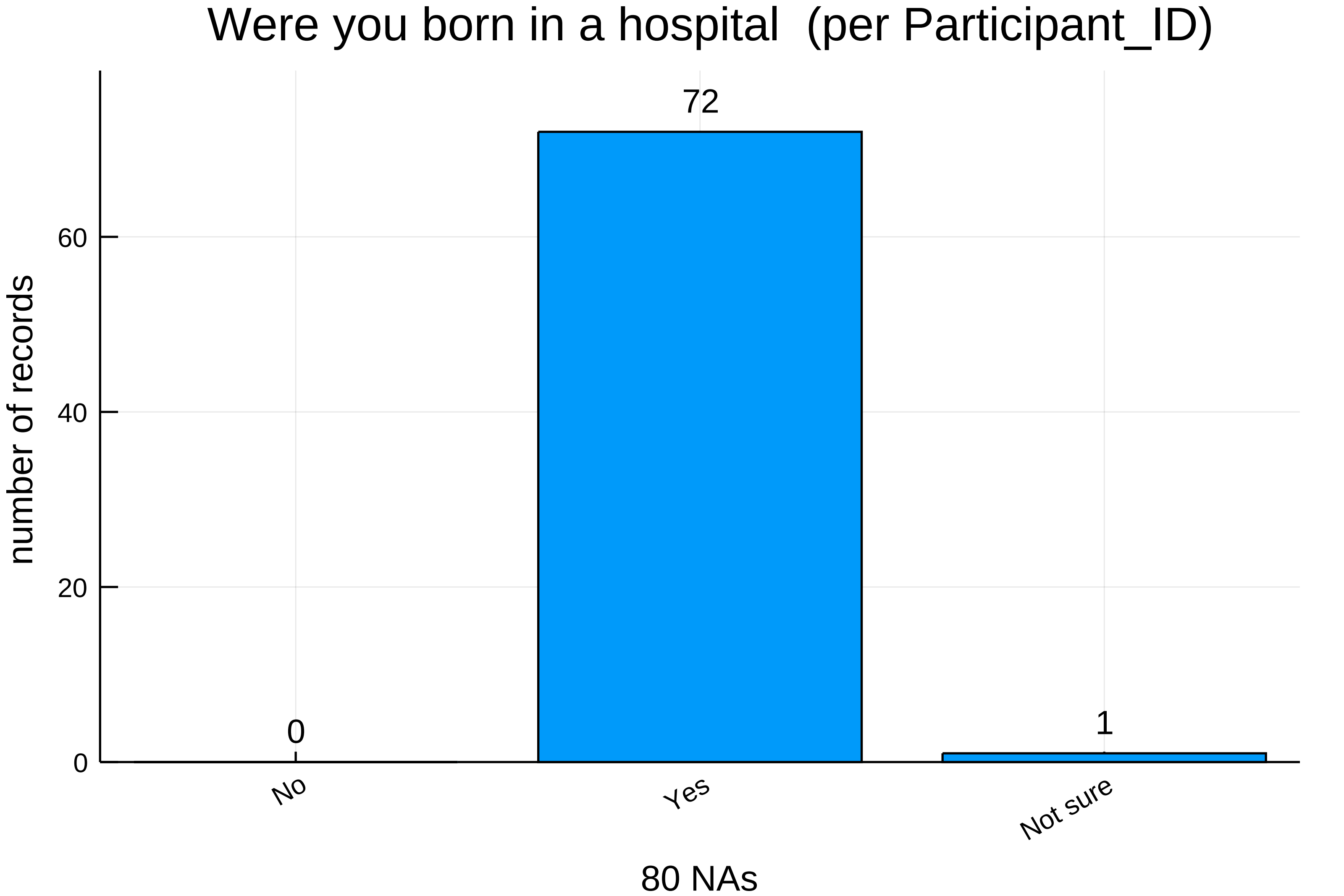
Were you born in a hospital (per Participant_ID)
number of records
0
20
40
60
0
72
1
No
Yes
Not sure
80 NAs

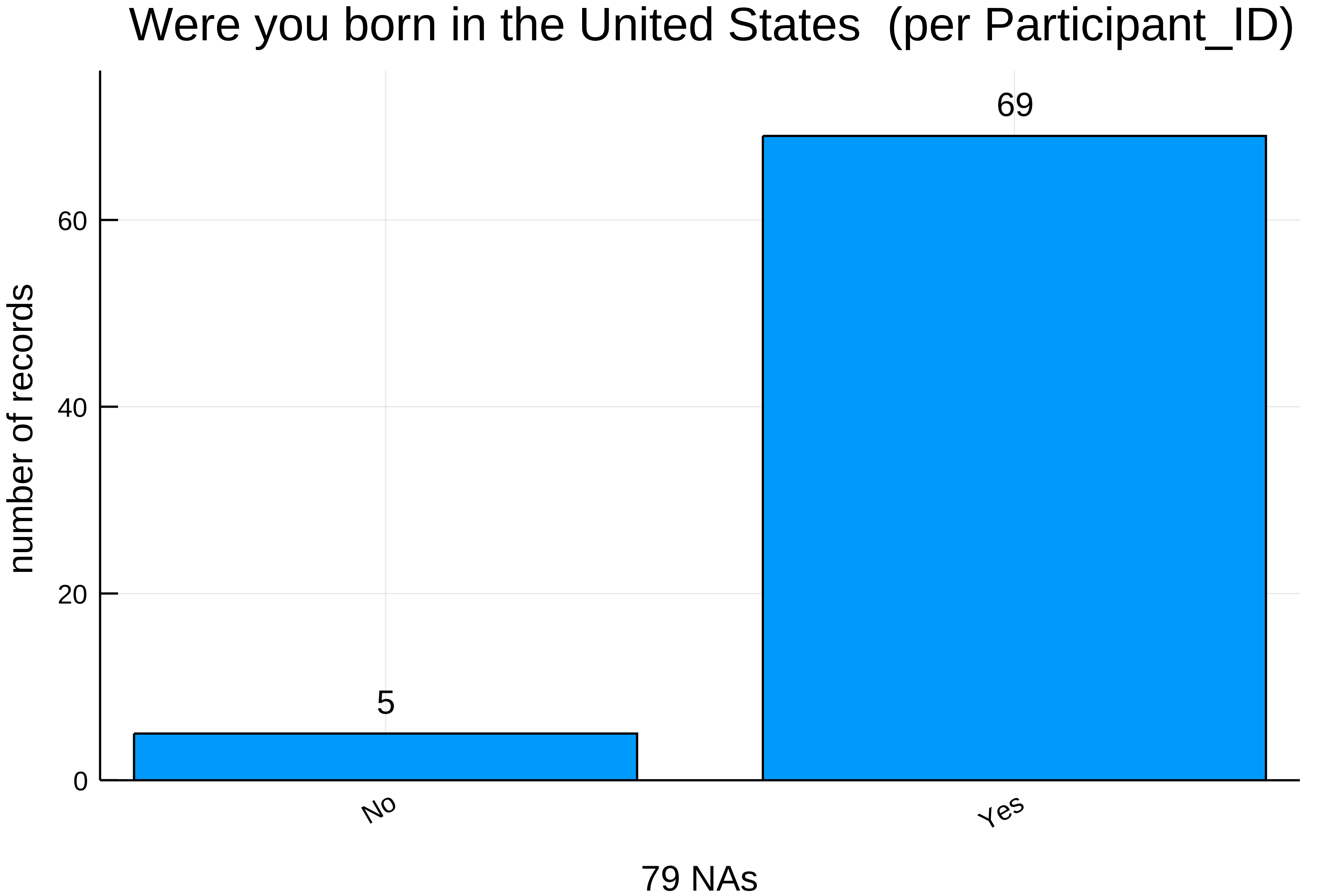
Were you born in the United States (per Participant_ID)
number of records
0
20
40
60
5
69
No
Yes
79 NAs

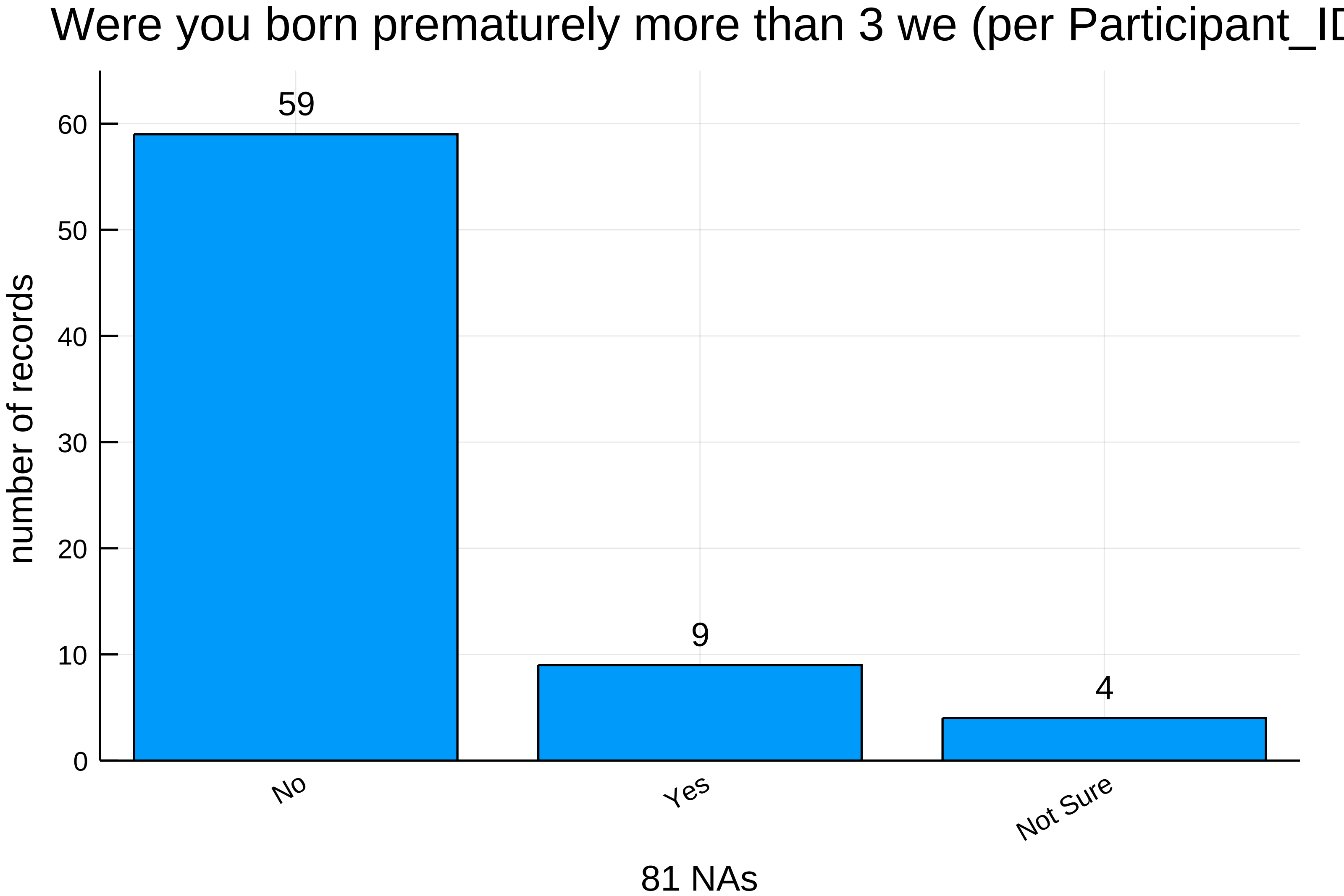
Were you born prematurely more than 3 we (per Participant_ID
number of records
59
9
4
60
50
40
30
20
10
0
No
Yes
Not Sure
81 NAs

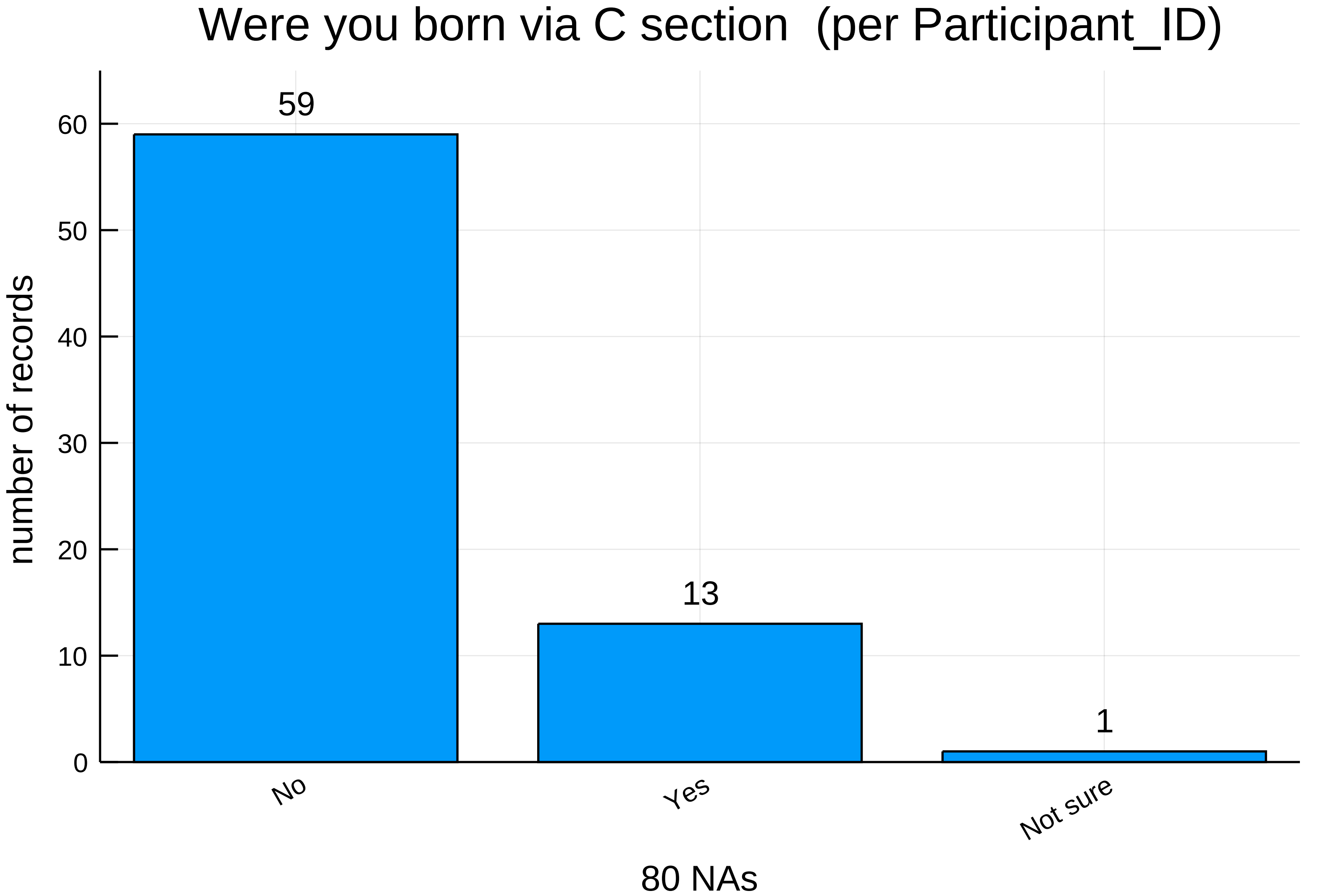

Were you born via C section (per Participant_ID)
number of records
59
13
1
0
10
20
30
40
50
60
No
Yes
Not sure
80 NAs

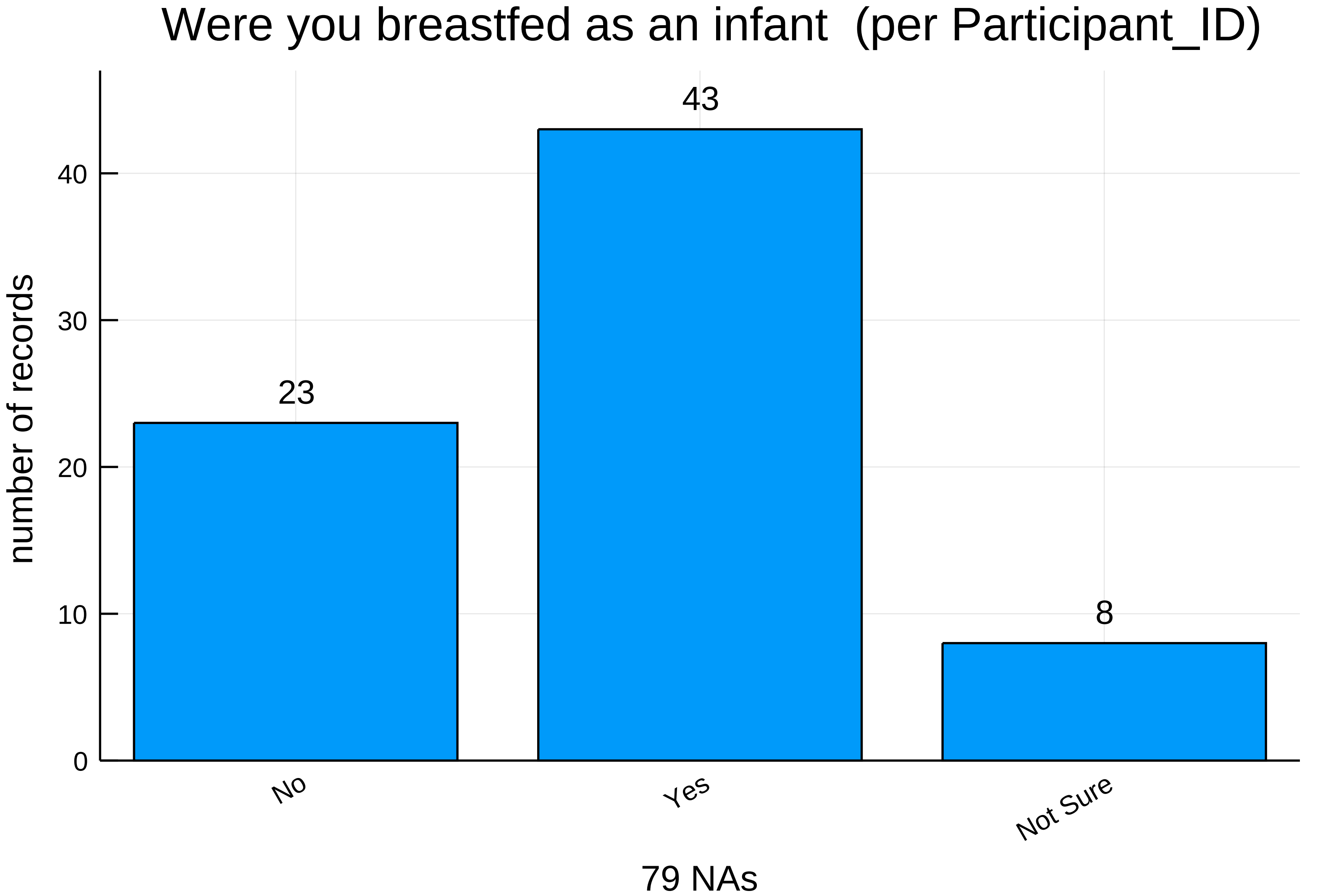

Were you breastfed as an infant (per Participant_ID)
number of records
0
10
20
30
40
23
43
8
No
Yes
Not Sure
79 NAs

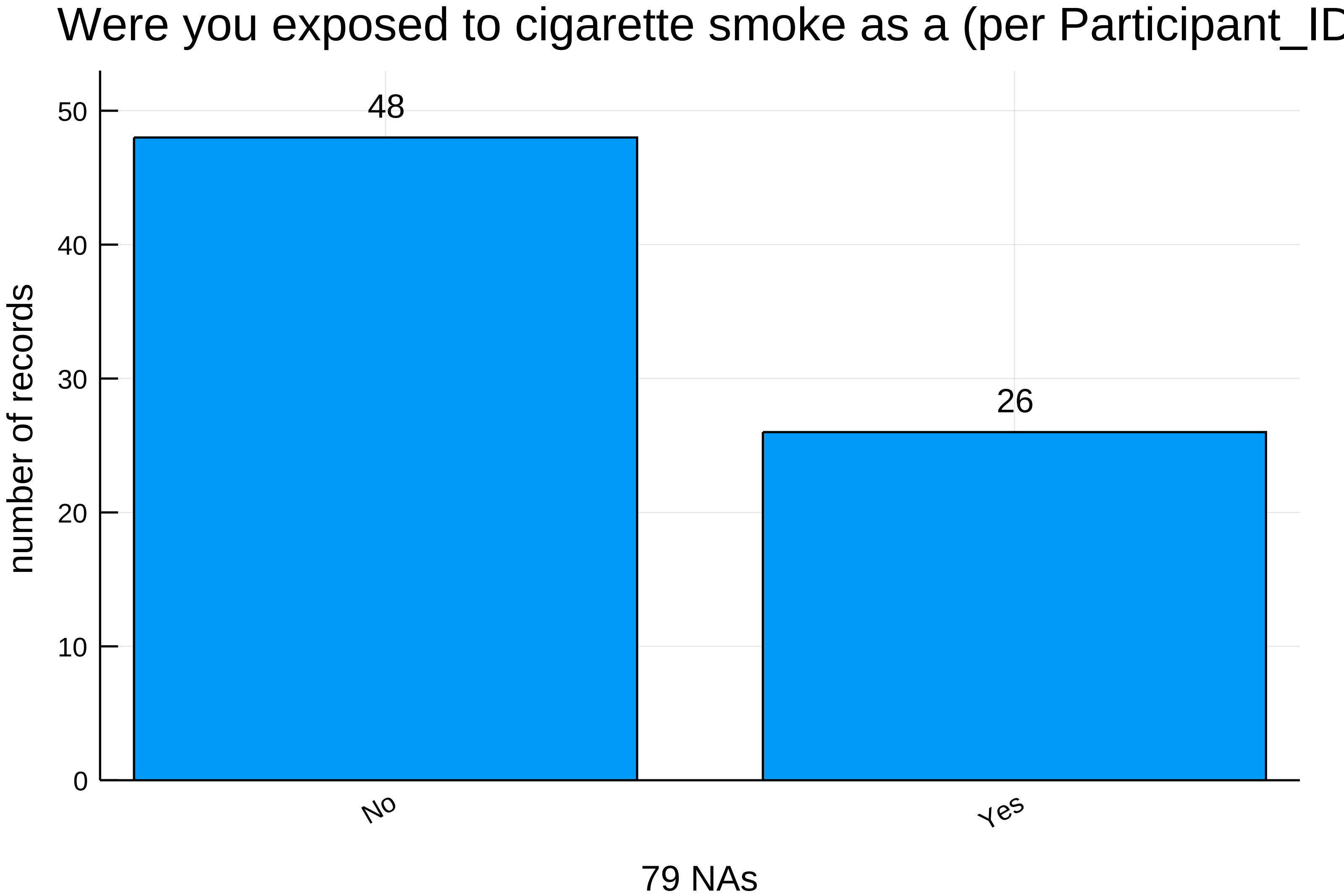
Were you exposed to cigarette smoke as a (per Participant_ID
number of records
50
40
30
20
10
0
48
26
No
Yes
79 NAs

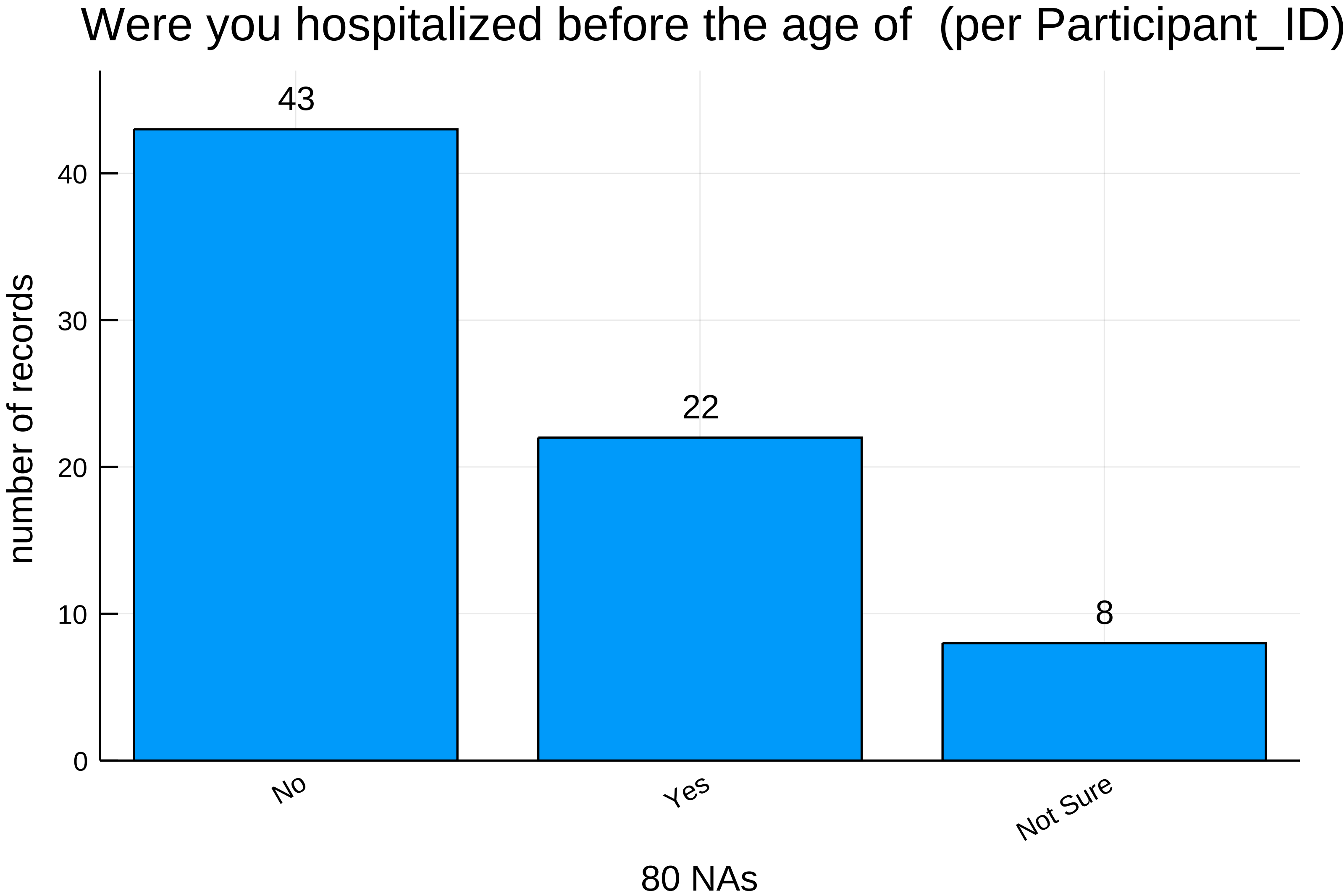
Were you hospitalized before the age of (per Participant_ID)
number of records
43
22
8
0
10
20
30
40
No
Yes
Not Sure
80 NAs

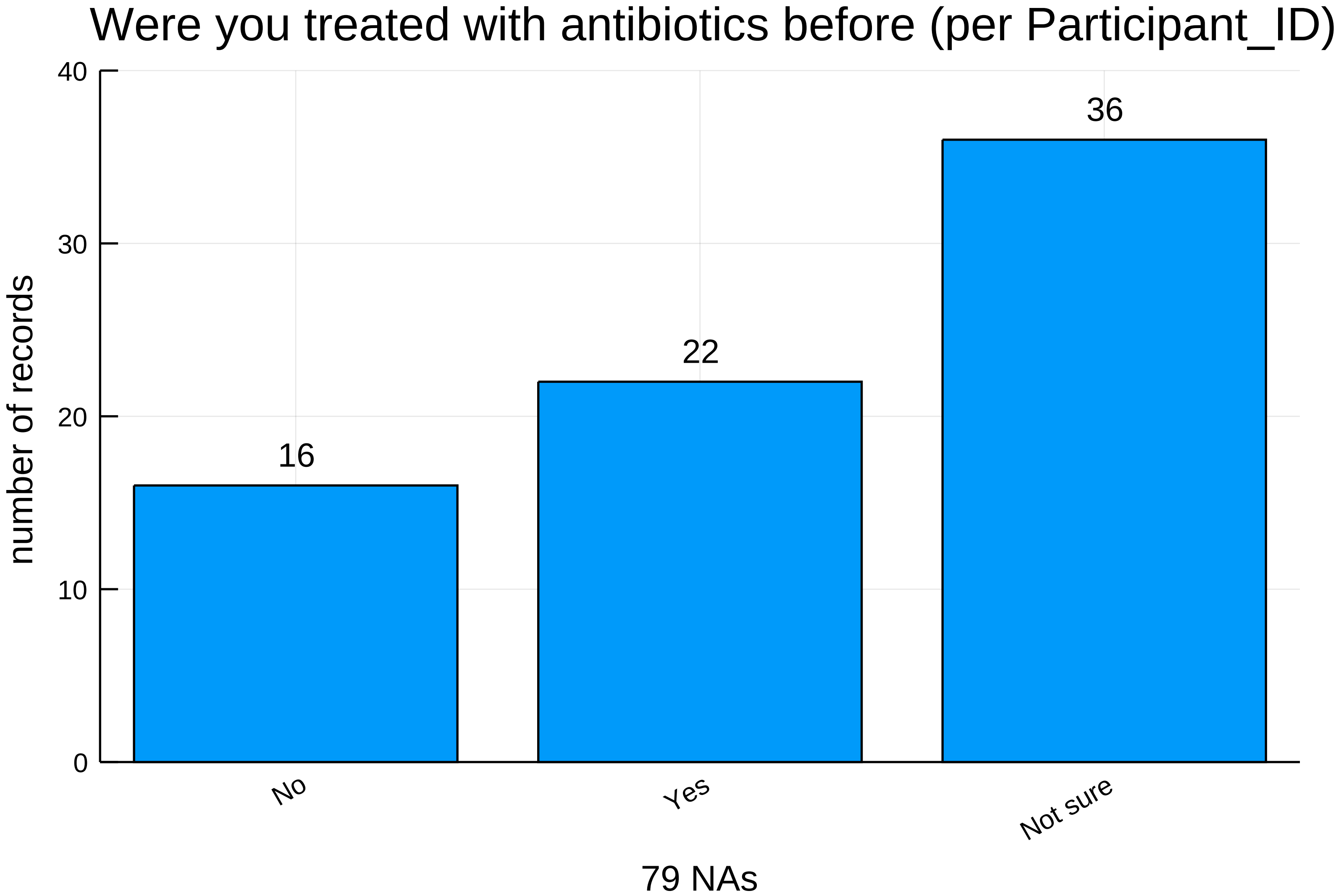
Were you treated with antibiotics before (per Participant_ID)
number of records
40
30
20
10
0
16
22
36
No
Yes
Not sure
79 NAs

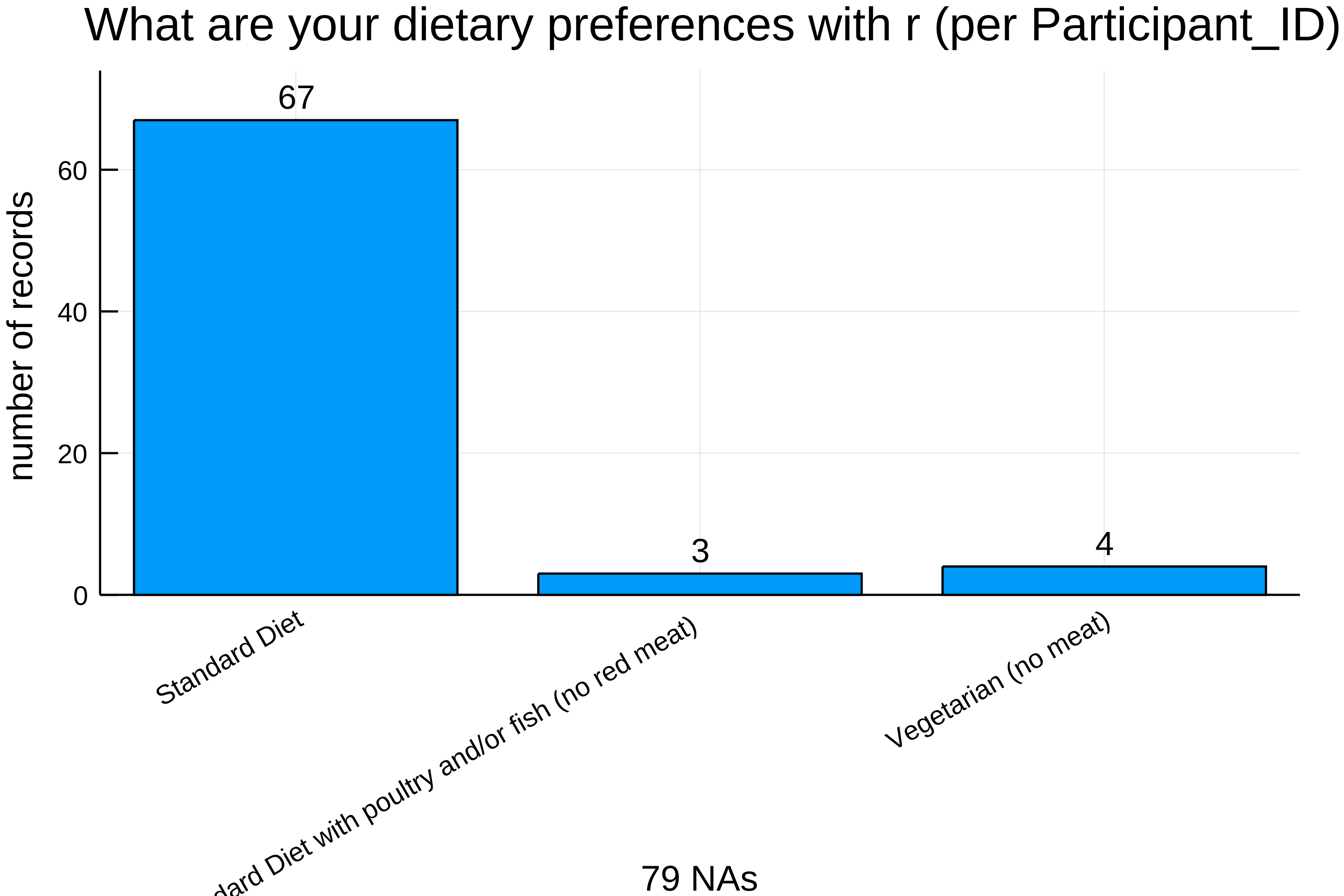
What are your dietary preferences with r (per Participant_ID)
number of records
0
20
40
60
67
3
4
Standard Diet
dard Diet with poultry and/or fish (no red meat)
Vegetarian (no meat)
79 NAs

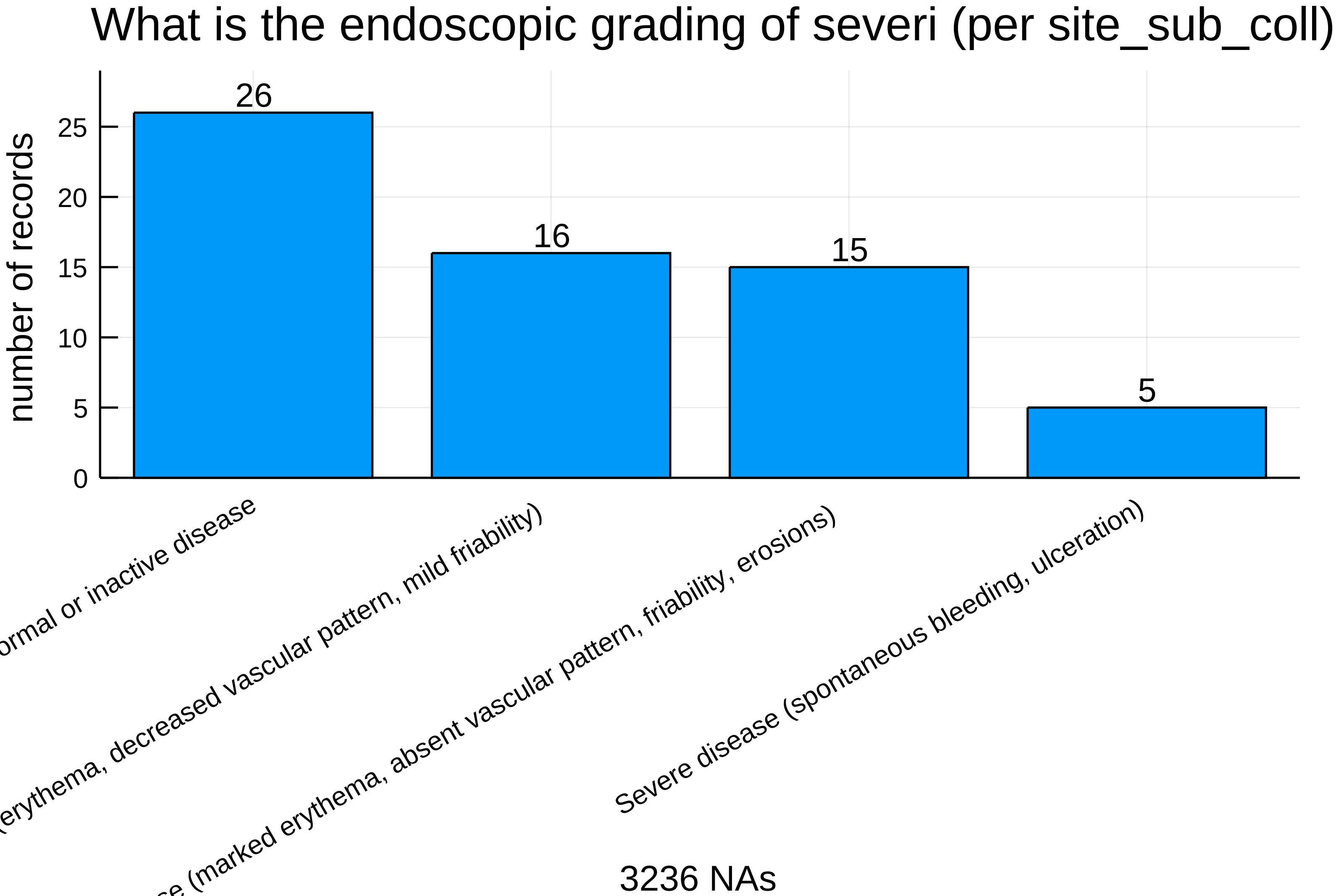
What is the endoscopic grading of severi (per site_sub_coll)
number of records
0
5
10
15
20
25
26
16
15
5
ormal or inactive disease
(erythema, decreased vascular pattern, mild friability)
(marked erythema, absent vascular pattern, friability, erosions)
Severe disease (spontaneous bleeding, ulceration)
3236 NAs

What is the endoscopic grading of severi (per site_sub_coll)

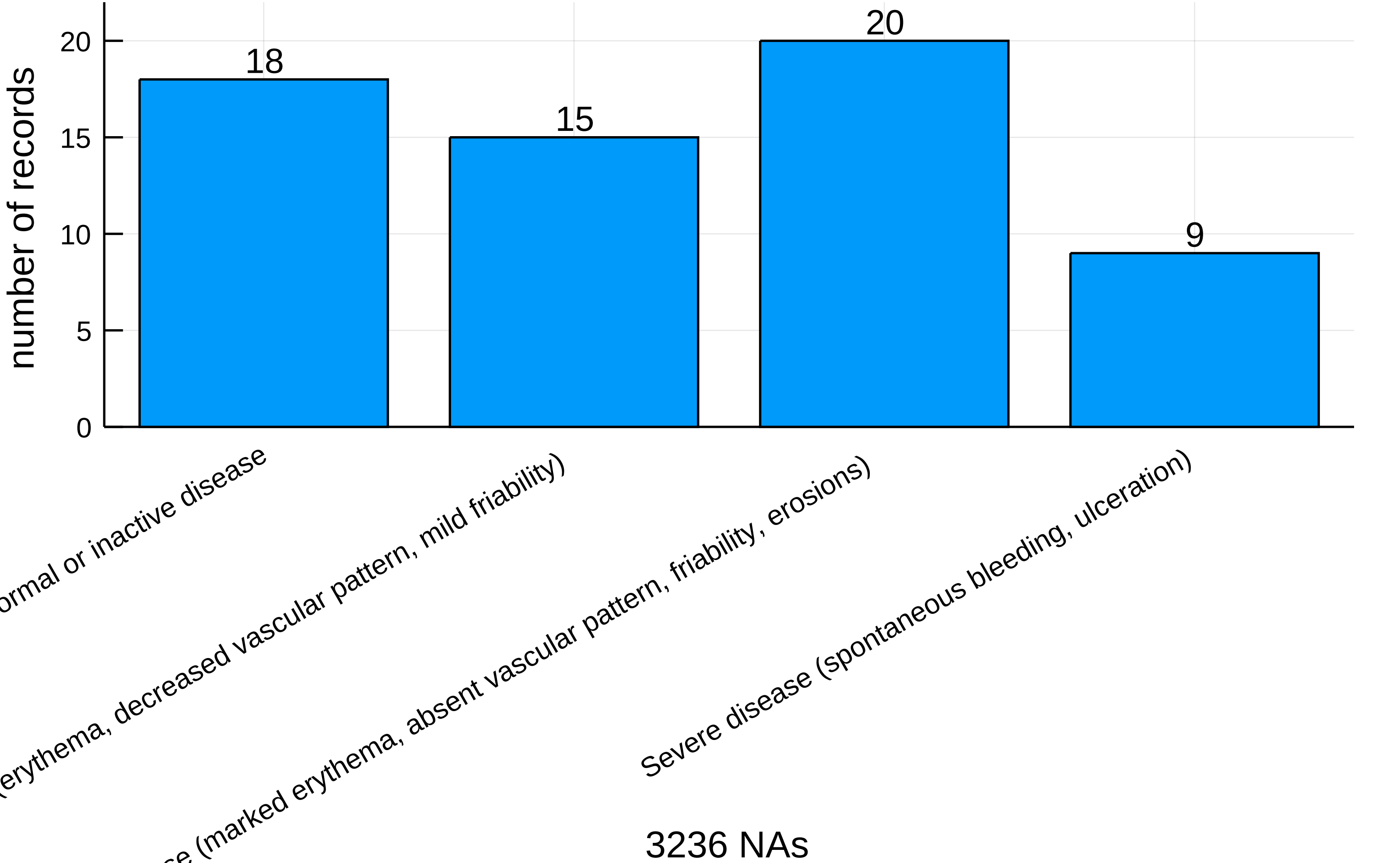

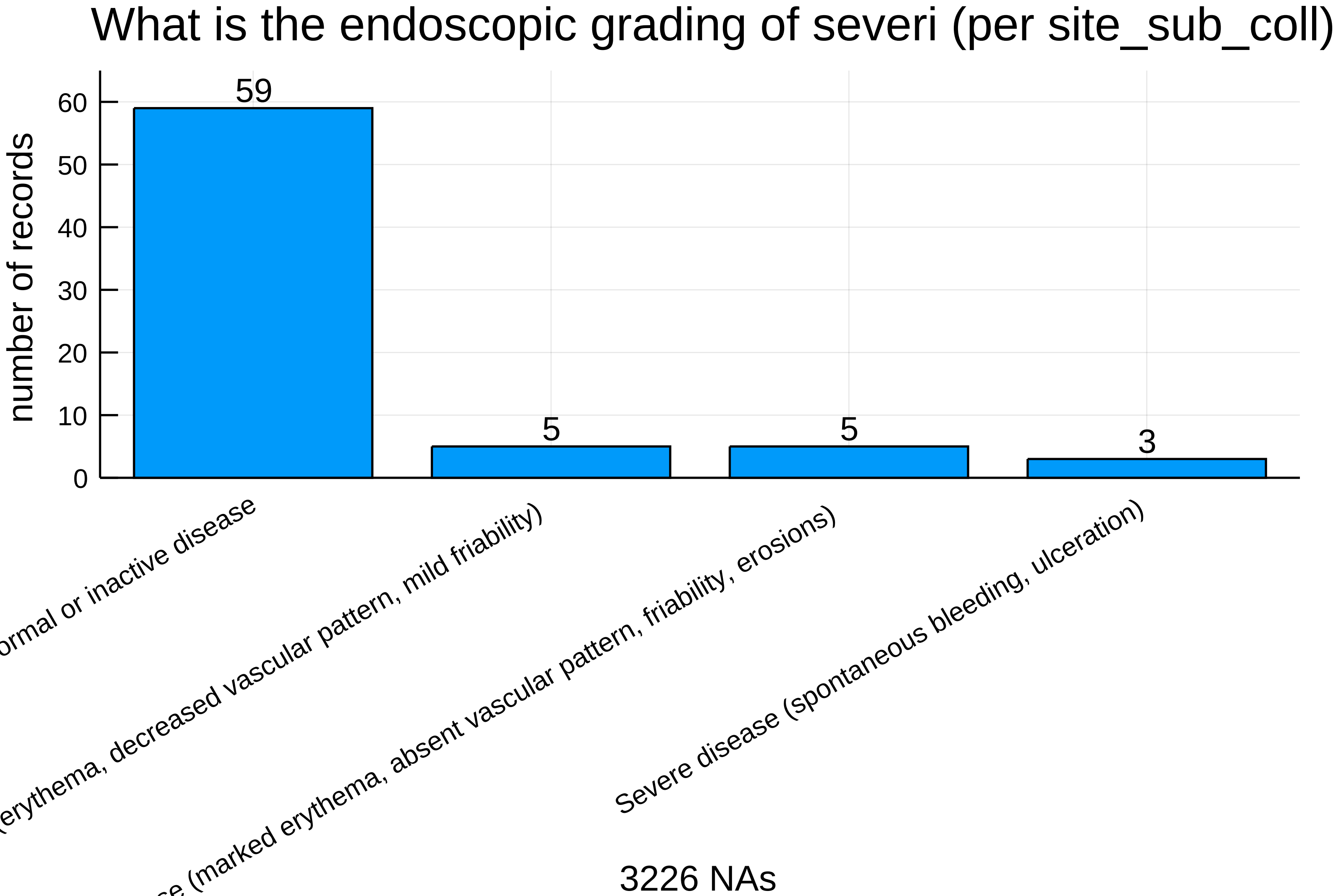
What is the endoscopic grading of severi (per site_sub_coll)
number of records
0
10
20
30
40
50
60
59
5
5
3
ormal or inactive disease
(erythema, decreased vascular pattern, mild friability)
se (marked erythema, absent vascular pattern, friability, erosions)
Severe disease (spontaneous bleeding, ulceration)
3226 NAs

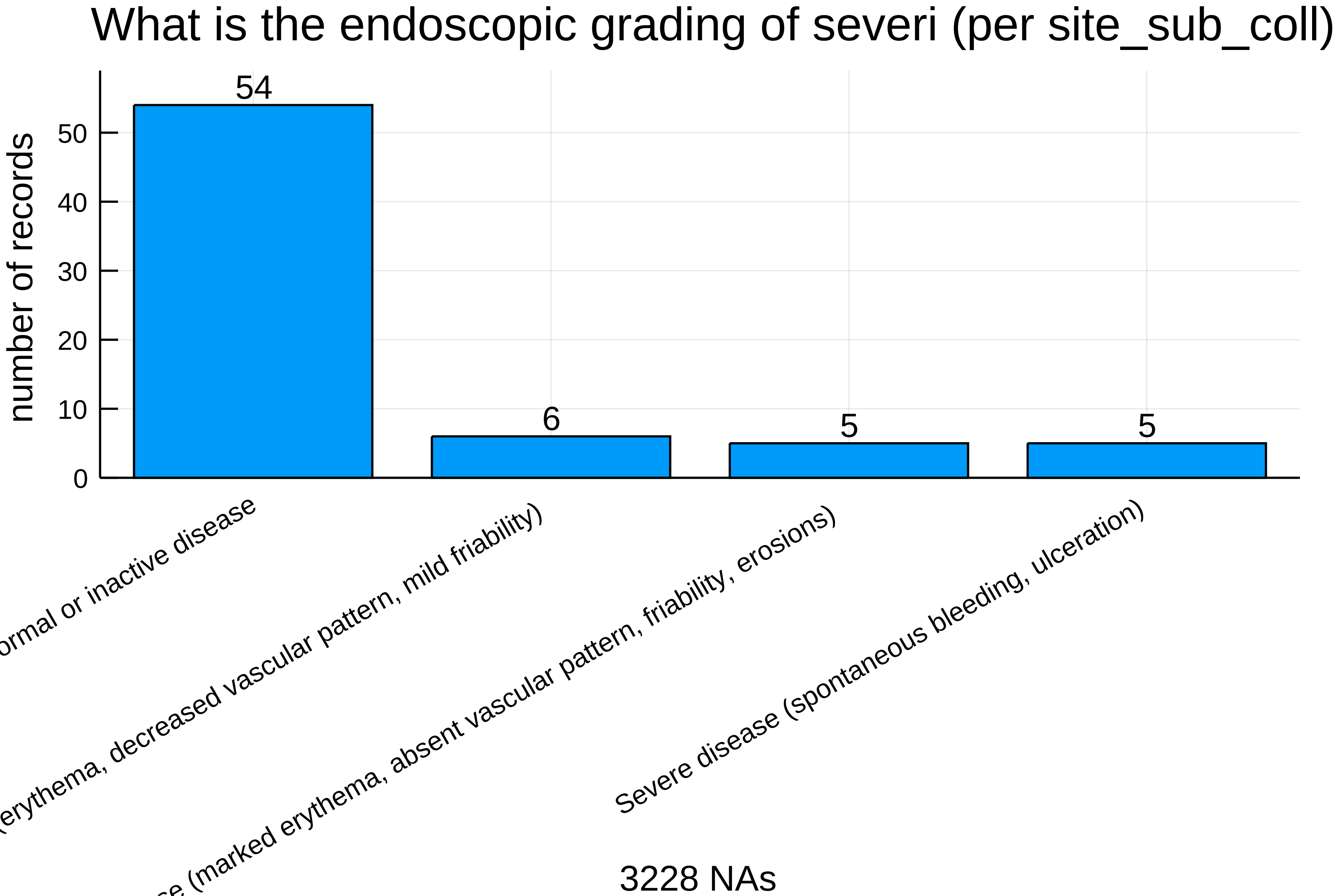

What is the endoscopic grading of severi (per site_sub_coll)
number of records
0
10
20
30
40
50
54
6
5
5
ormal or inactive disease
erythema, decreased vascular pattern, mild friability)
se (marked erythema, absent vascular pattern, friability, erosions)
Severe disease (spontaneous bleeding, ulceration)
3228 NAs

What is the endoscopic grading of severi (per site_sub_coll)

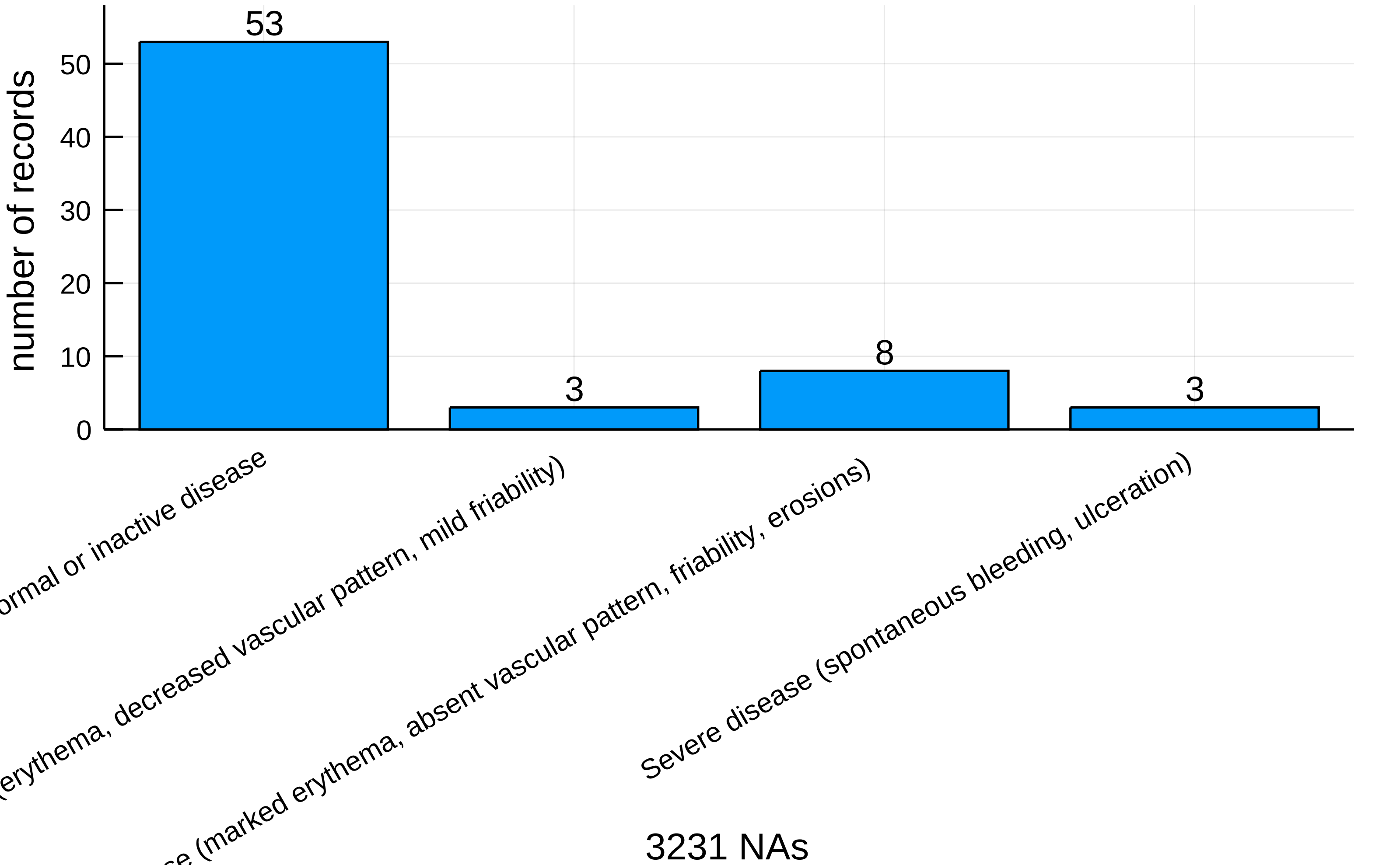

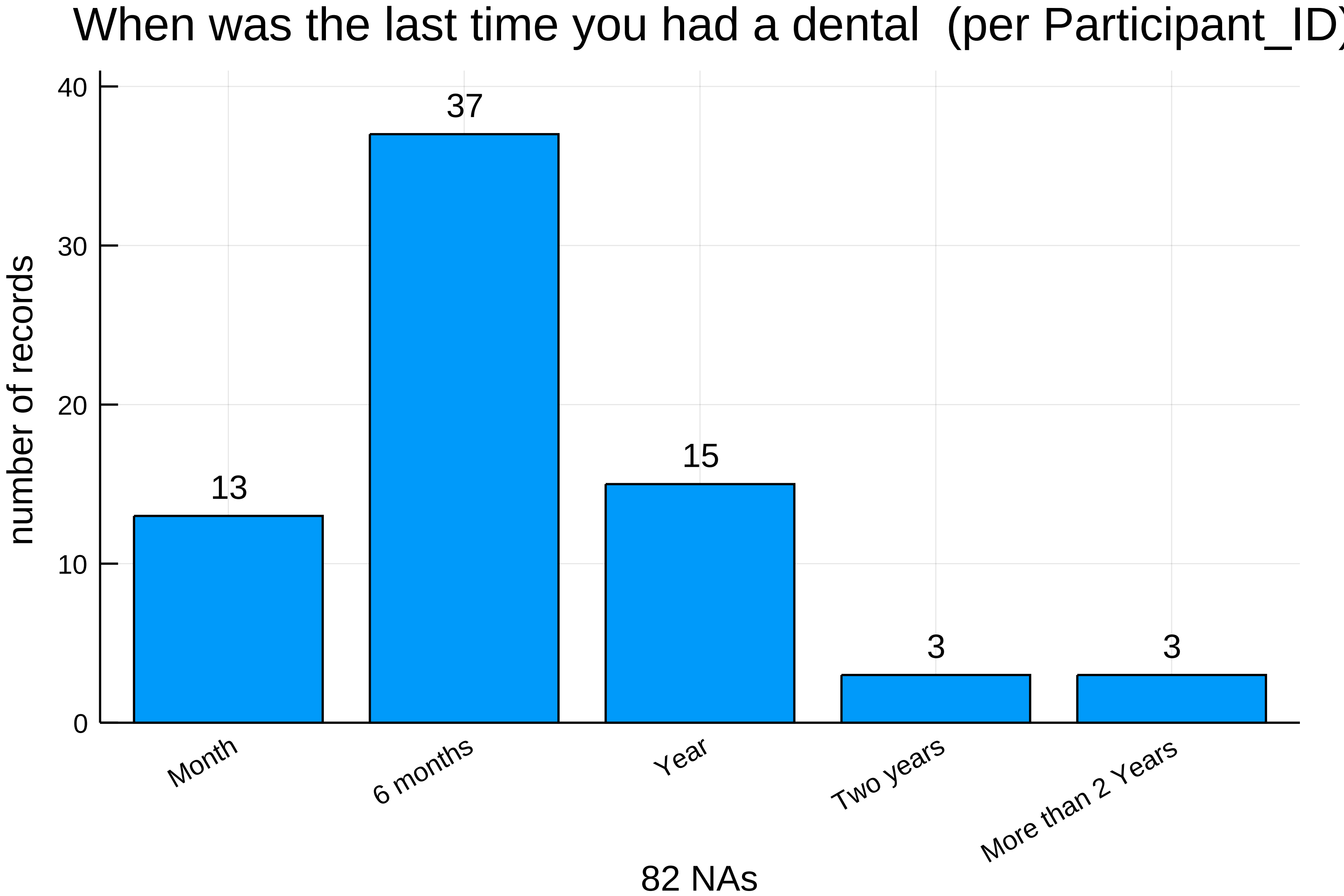
When was the last time you had a dental (per Participant_ID)
number of records
0
10
20
30
40
13
37
15
3
3
Month
6 months
Year
Two years
More than 2 Years
82 NAs

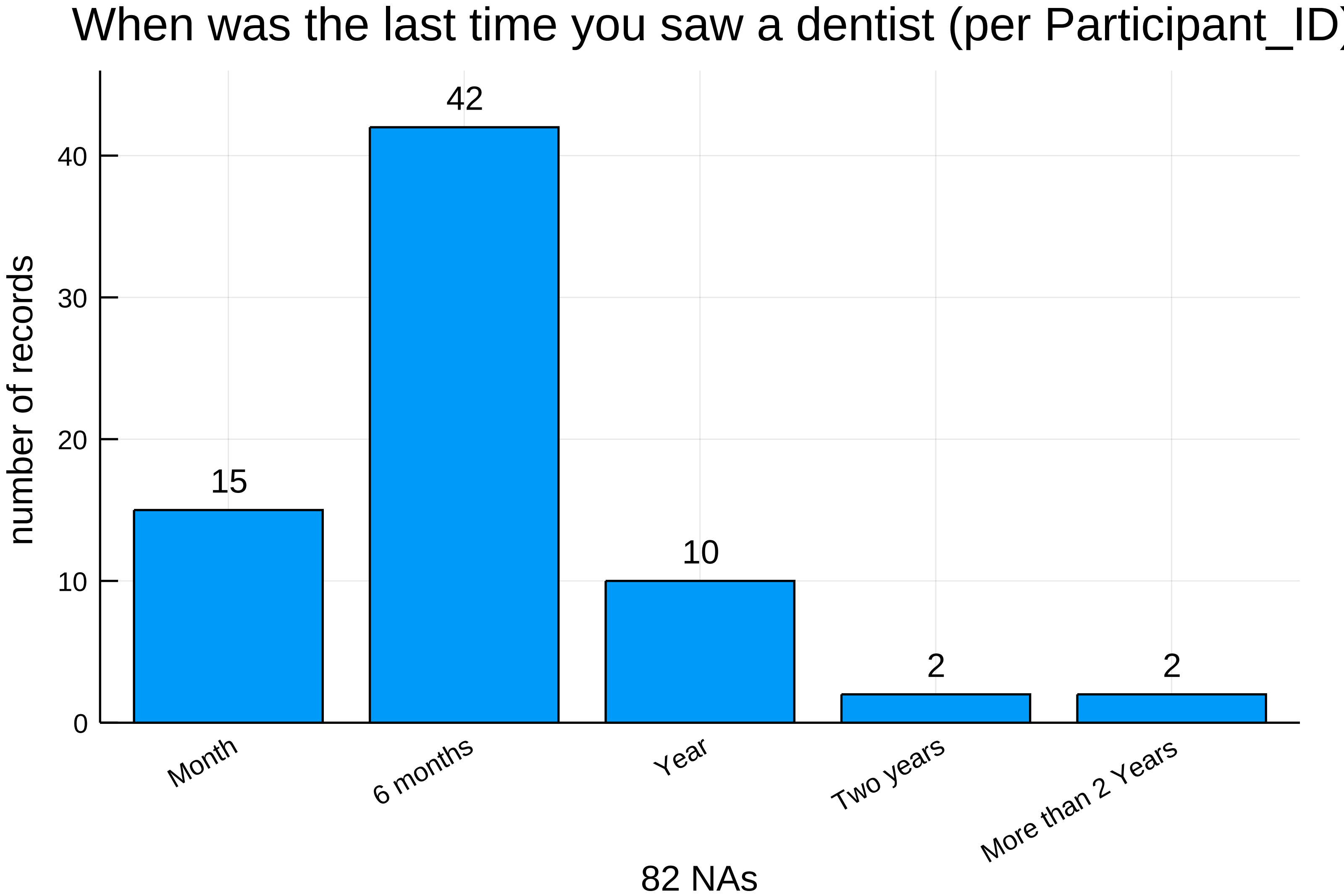
When was the last time you saw a dentist (per Participant_ID)
number of records
0
10
20
30
40
15
42
10
2
2
Month
6 months
Year
Two years
More than 2 Years
82 NAs

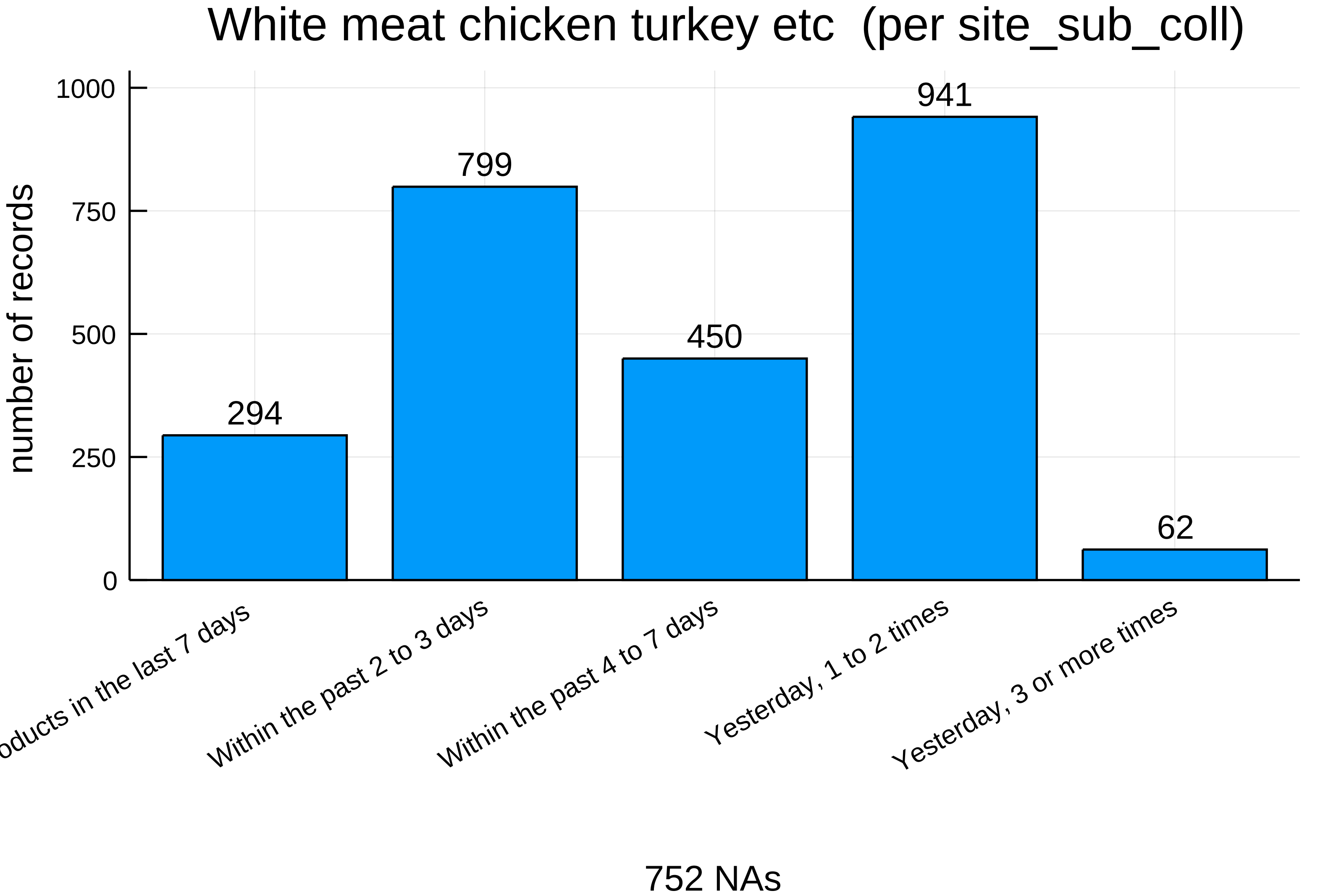
White meat chicken turkey etc (per site_sub_coll)
number of records
1000
750
500
250
0
294
799
450
941
62
oducts in the last 7 days
Within the past 2 to 3 days
Within the past 4 to 7 days
Yesterday, 1 to 2 times
Yesterday, 3 or more times
752 NAs

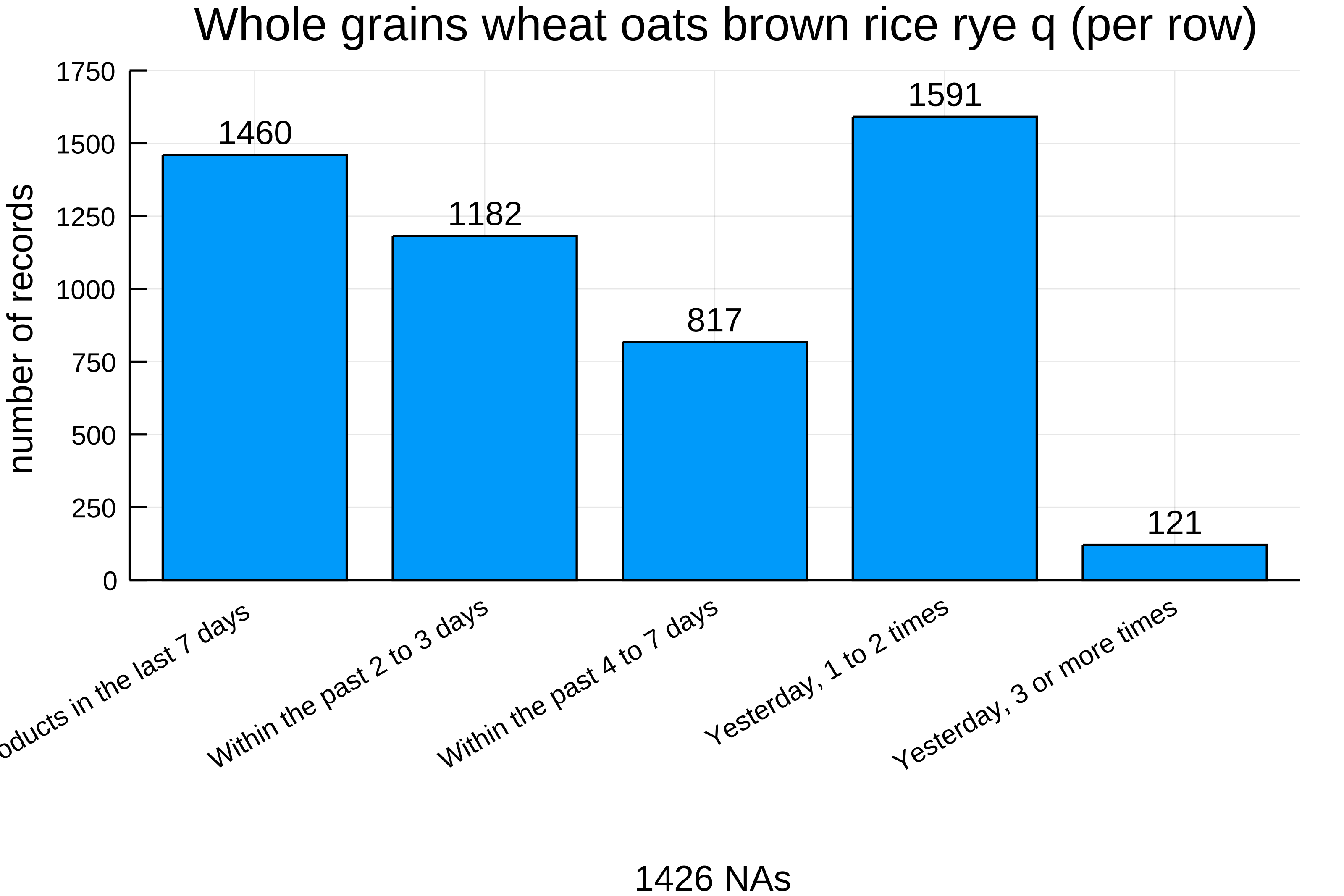
Whole grains wheat oats brown rice rye q (per row)
number of records
1750
1500
1250
1000
750
500
250
0
1460
1182
817
1591
121
oducts in the last 7 days
Within the past 2 to 3 days
Within the past 4 to 7 days
Yesterday, 1 to 2 times
Yesterday, 3 or more times
1426 NAs

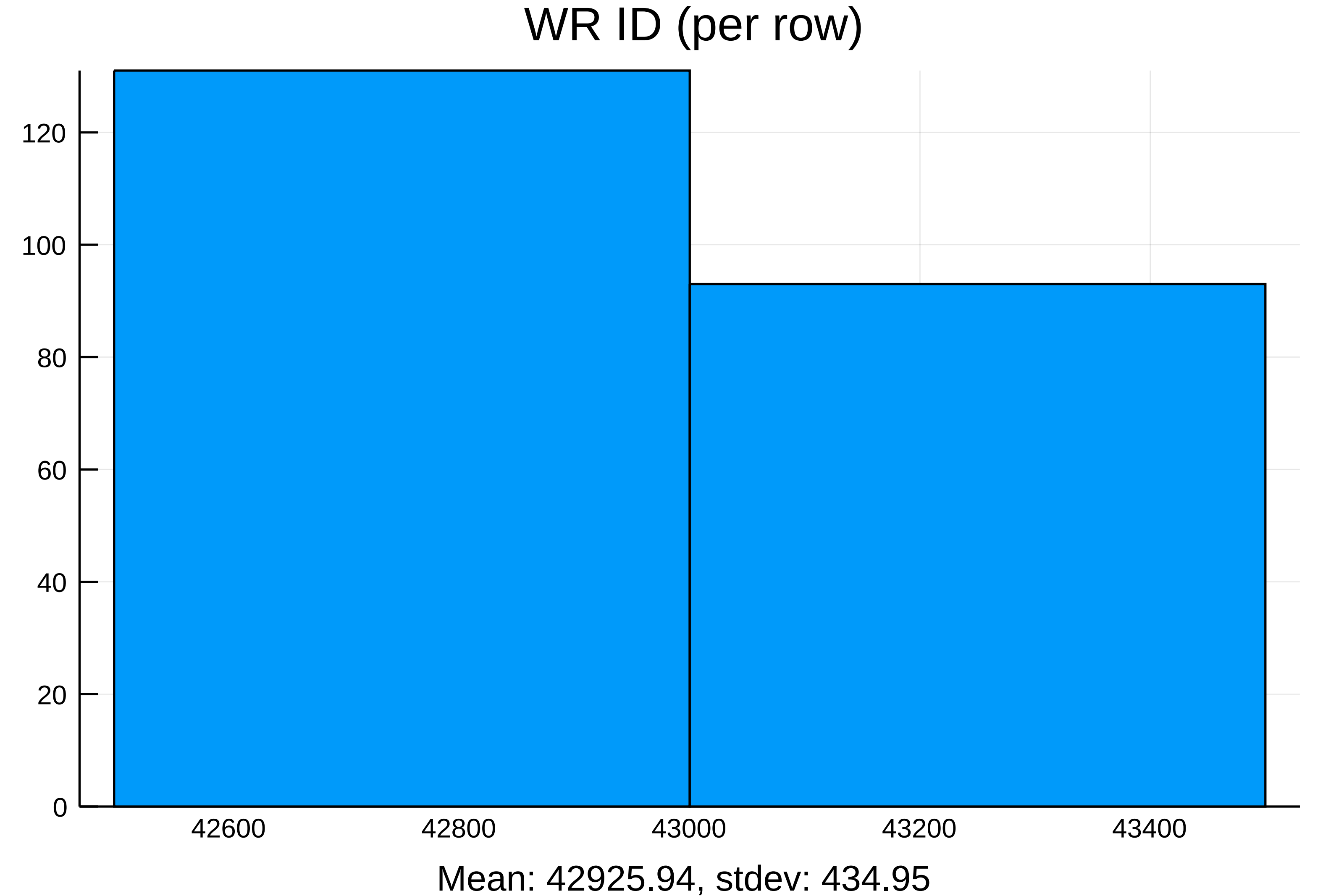
WR ID (per row)
120
100
80
60
40
20
0
42600
42800
43000
43200
43400
Mean: 42925.94, stdev: 434.95

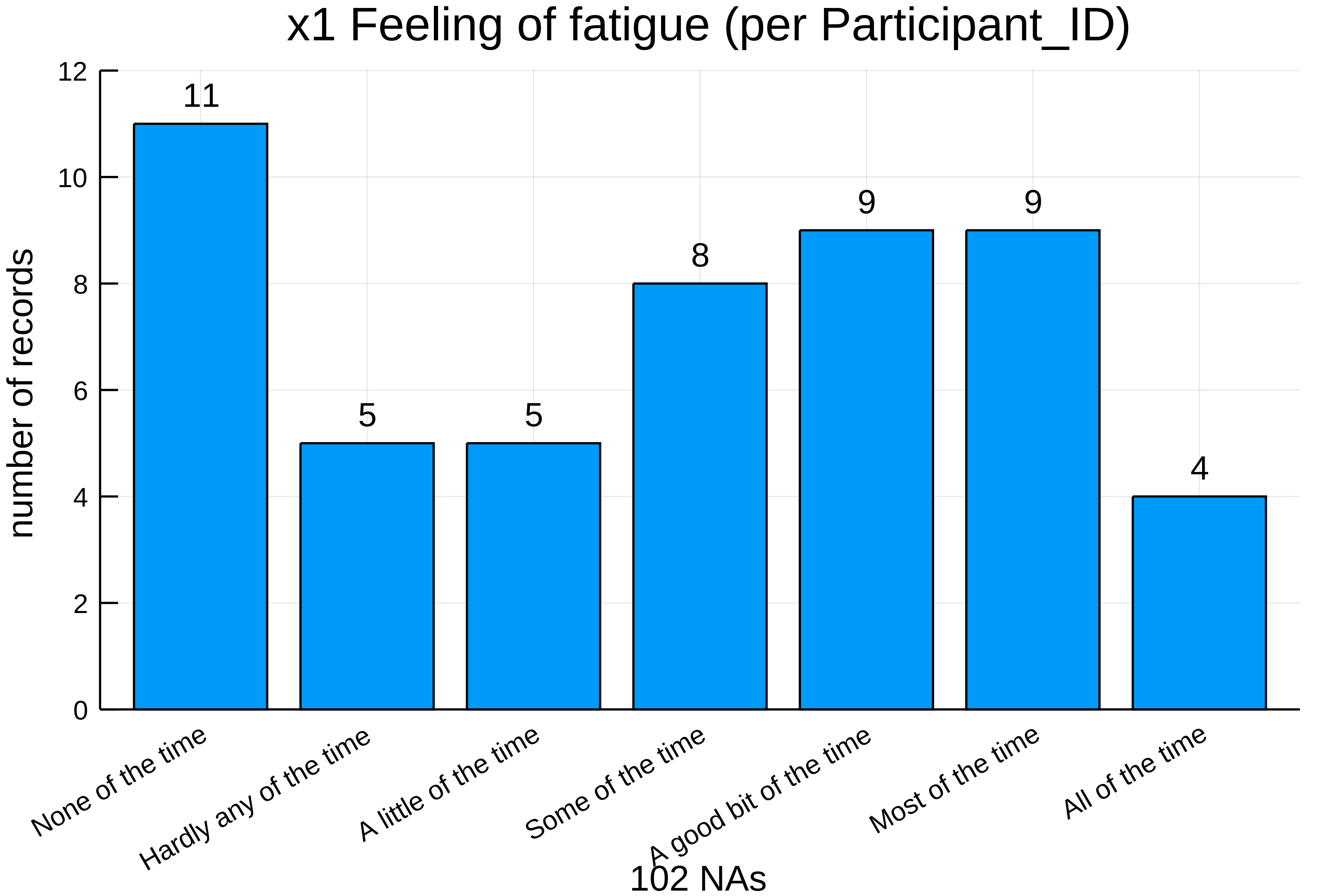
x1 Feeling of fatigue (per Participant_ID)
number of records
0
2
4
6
8
10
12
11
5
5
8
9
9
4
None of the time
Hardly any of the time
A little of the time
Some of the time
A good bit of the time
Most of the time
All of the time
102 NAs

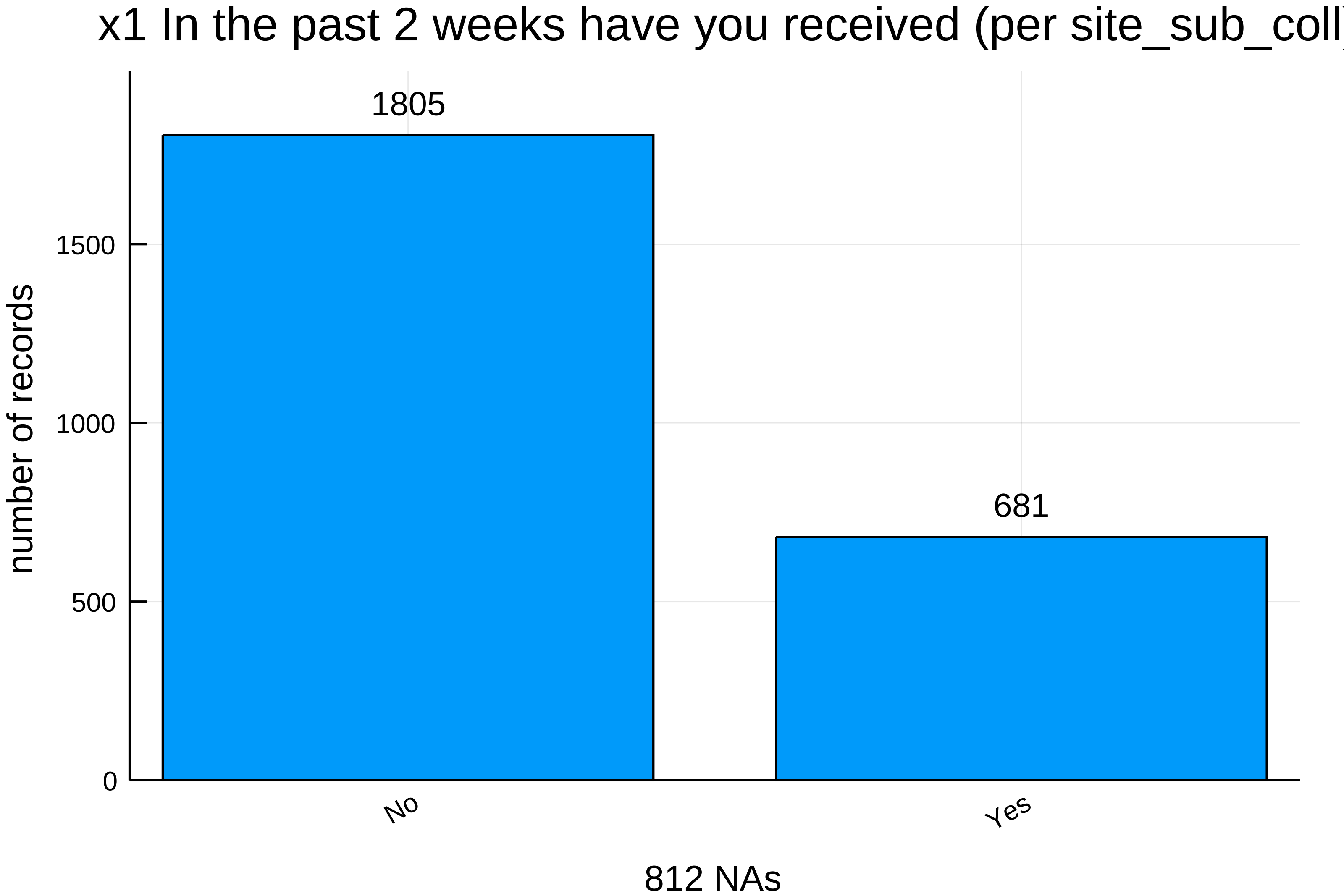
x1 In the past 2 weeks have you received (per site_sub_coll
number of records
1805
681
1500
1000
500
0
No
Yes
812 NAs

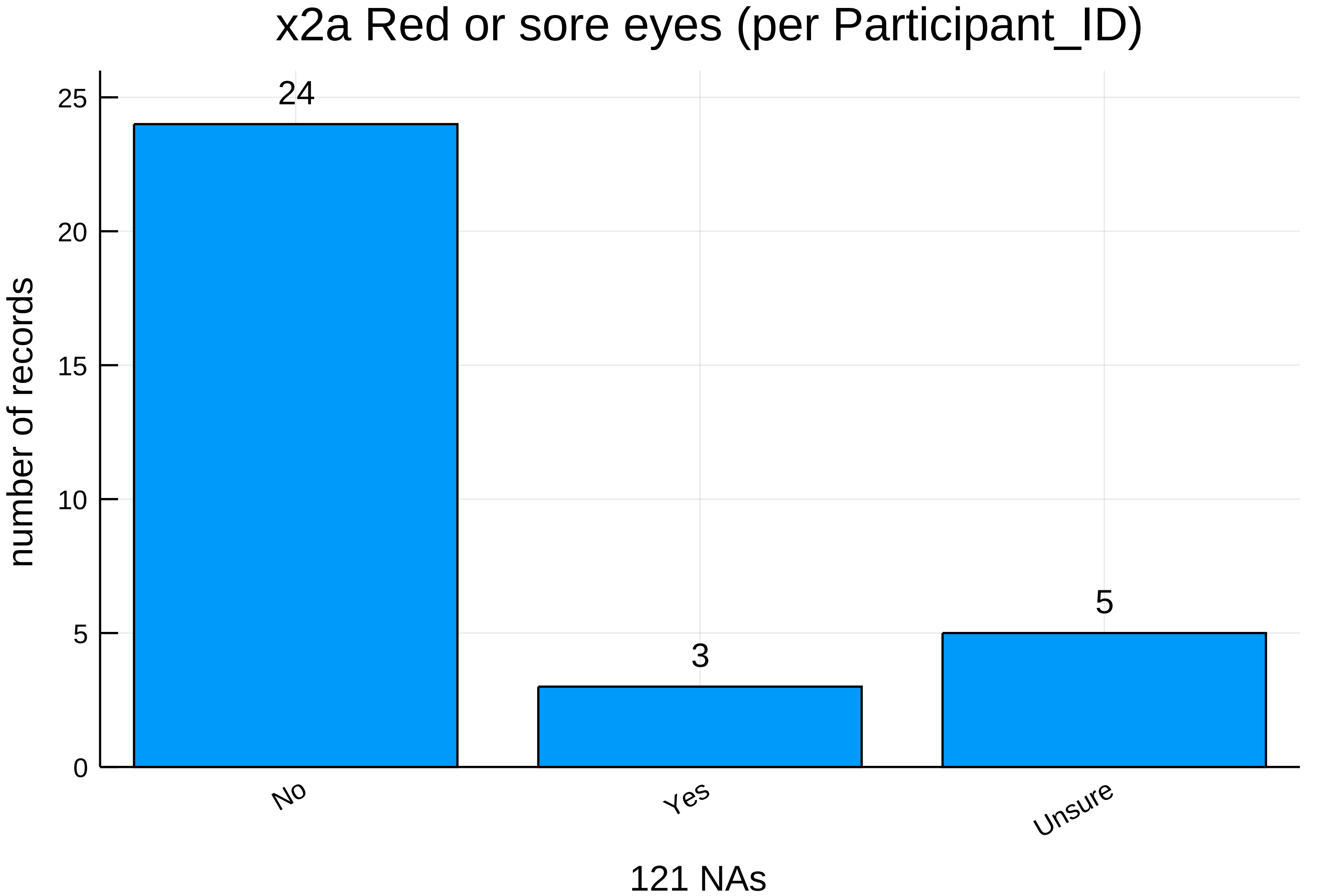
x2a Red or sore eyes (per Participant_ID)
number of records
0
5
10
15
20
25
24
3
5
No
Yes
Unsure
121 NAs

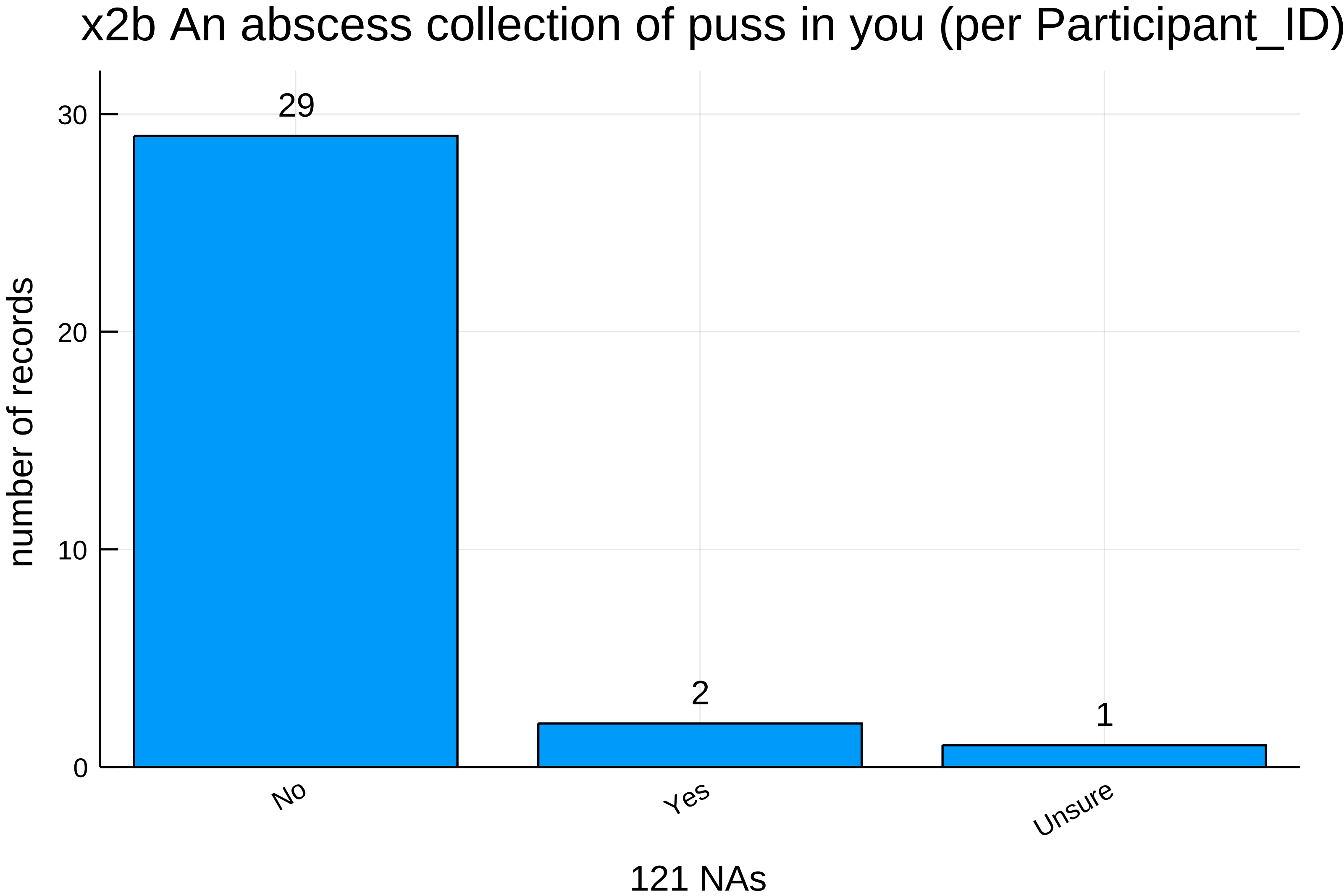

x2b An abscess collection of puss in you (per Participant_ID)
number of records
0
10
20
30
29
2
1
No
Yes
Unsure
121 NAs

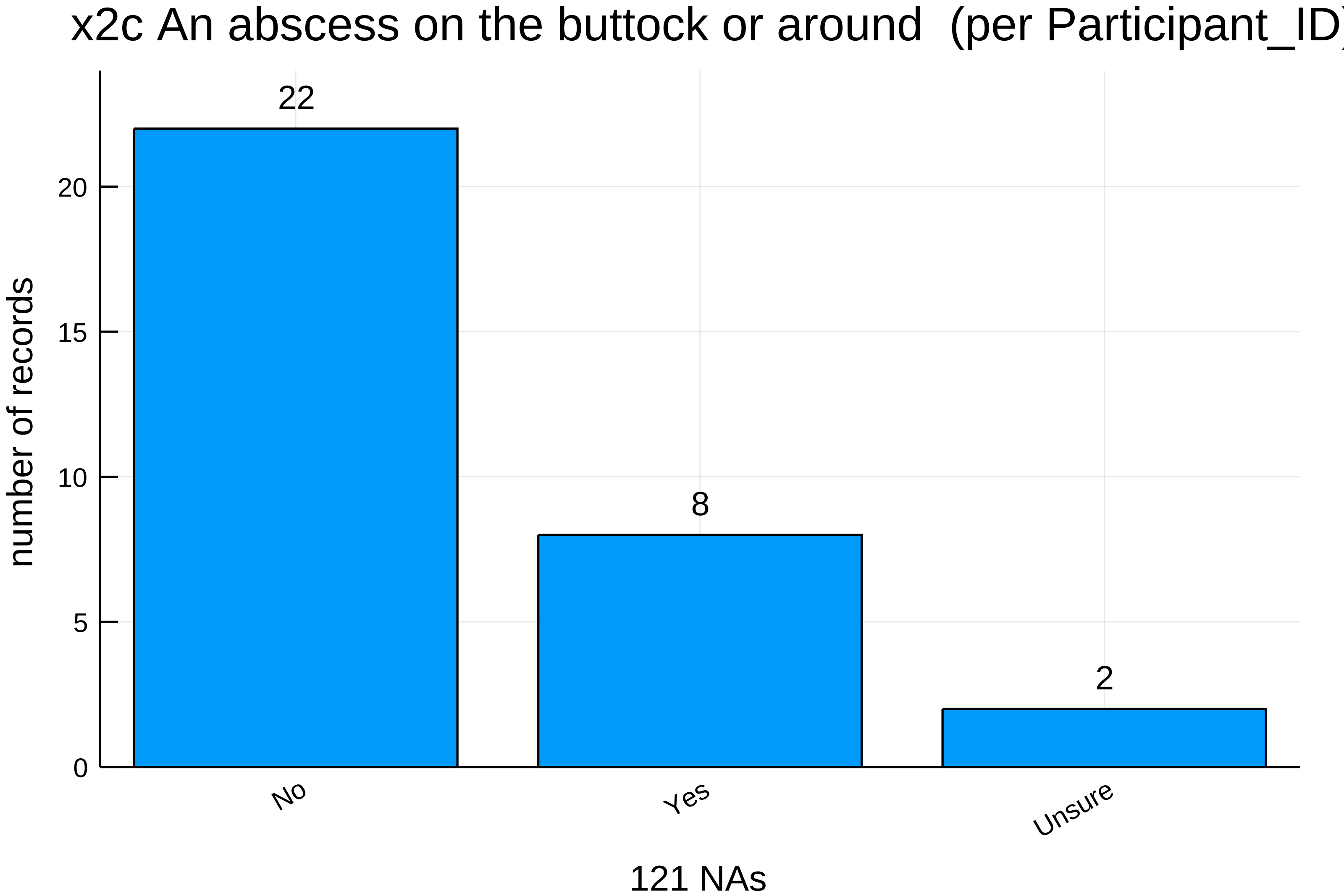
x2c An abscess on the buttock or around (per Participant_ID)
number of records
0
5
10
15
20
22
8
2
No
Yes
Unsure
121 NAs

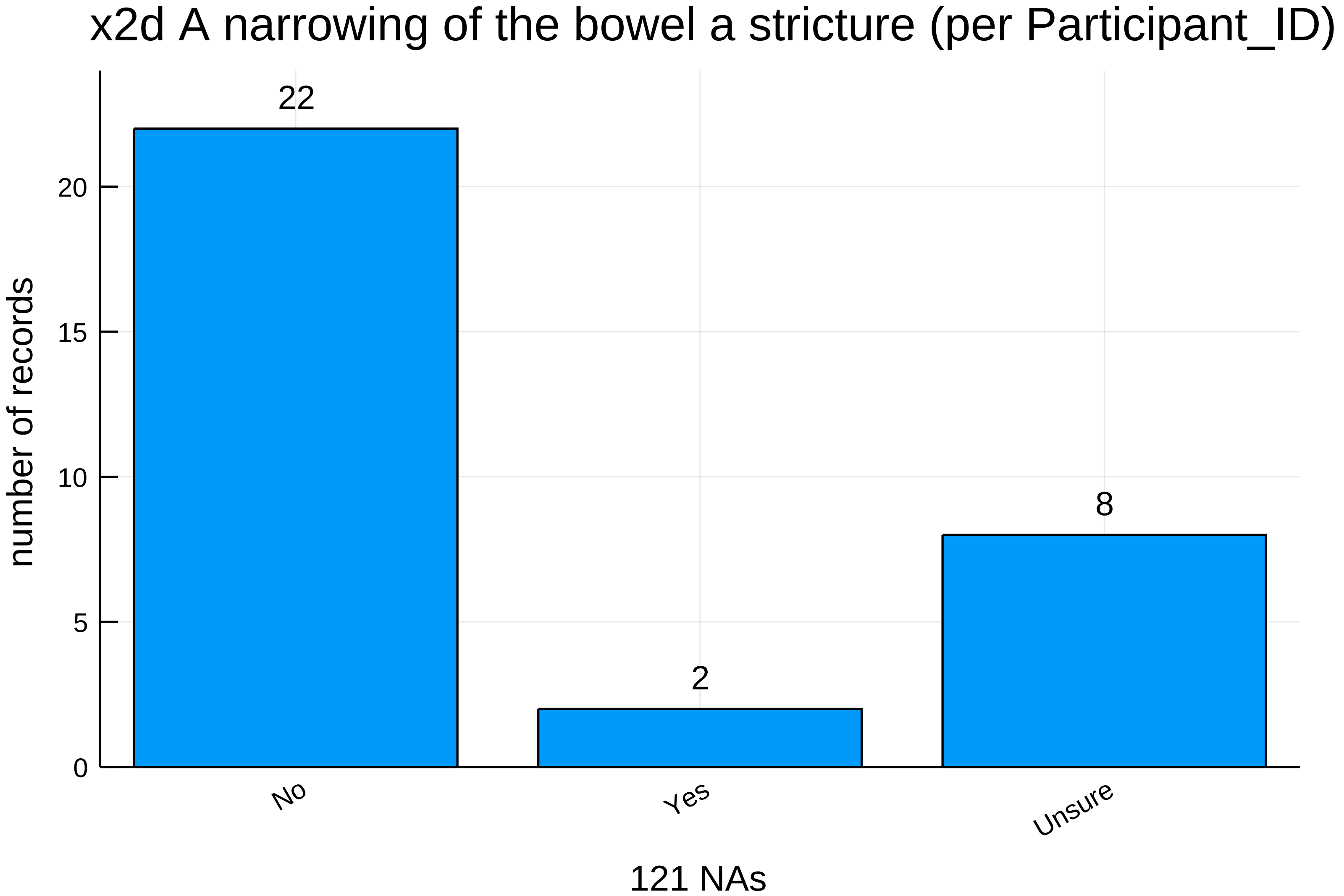
x2d A narrowing of the bowel a stricture (per Participant_ID)
number of records
22
2
8
0
5
10
15
20
No
Yes
Unsure
121 NAs

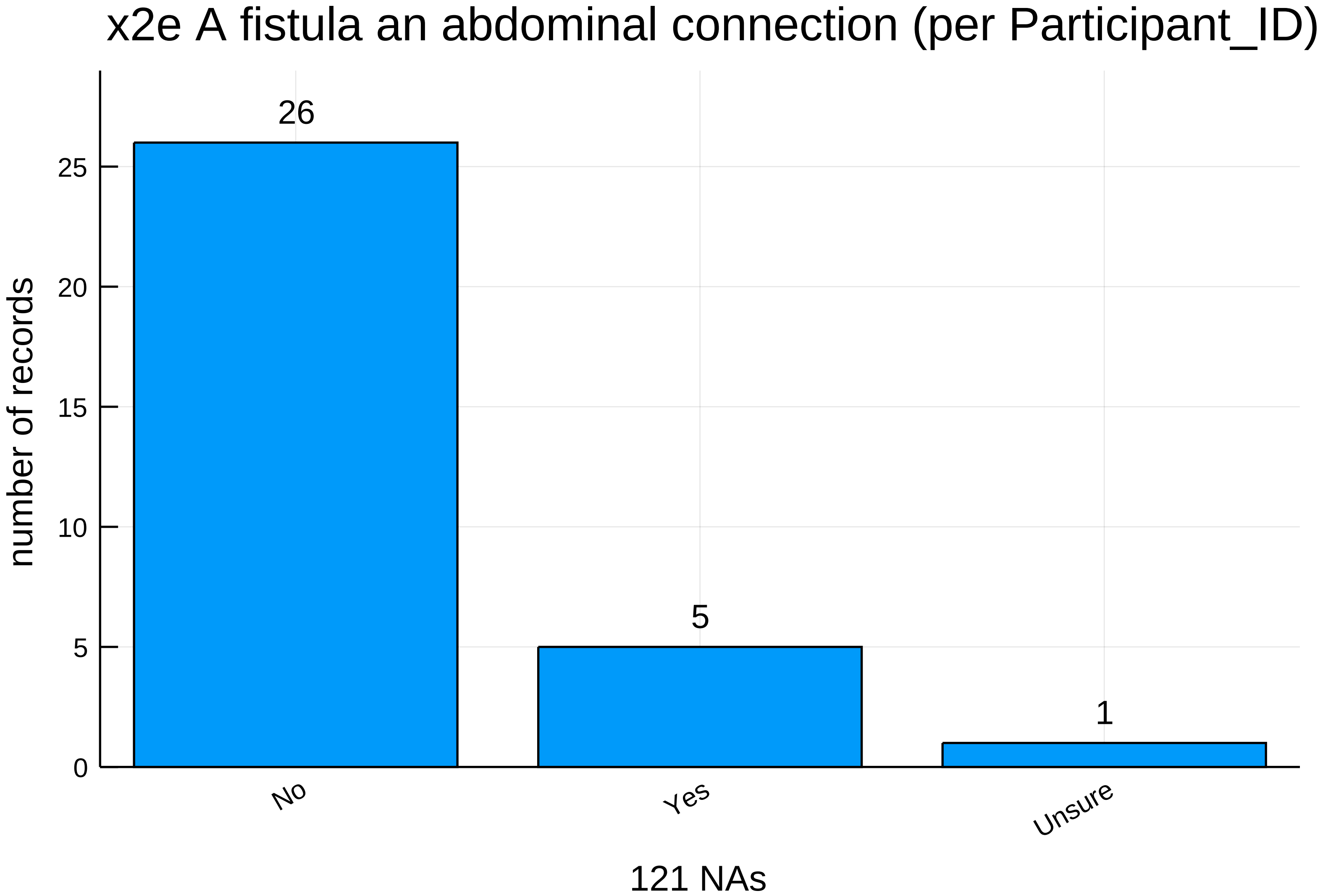
x2e A fistula an abdominal connection (per Participant_ID)
number of records
26
5
1
0
5
10
15
20
25
No
Yes
Unsure
121 NAs

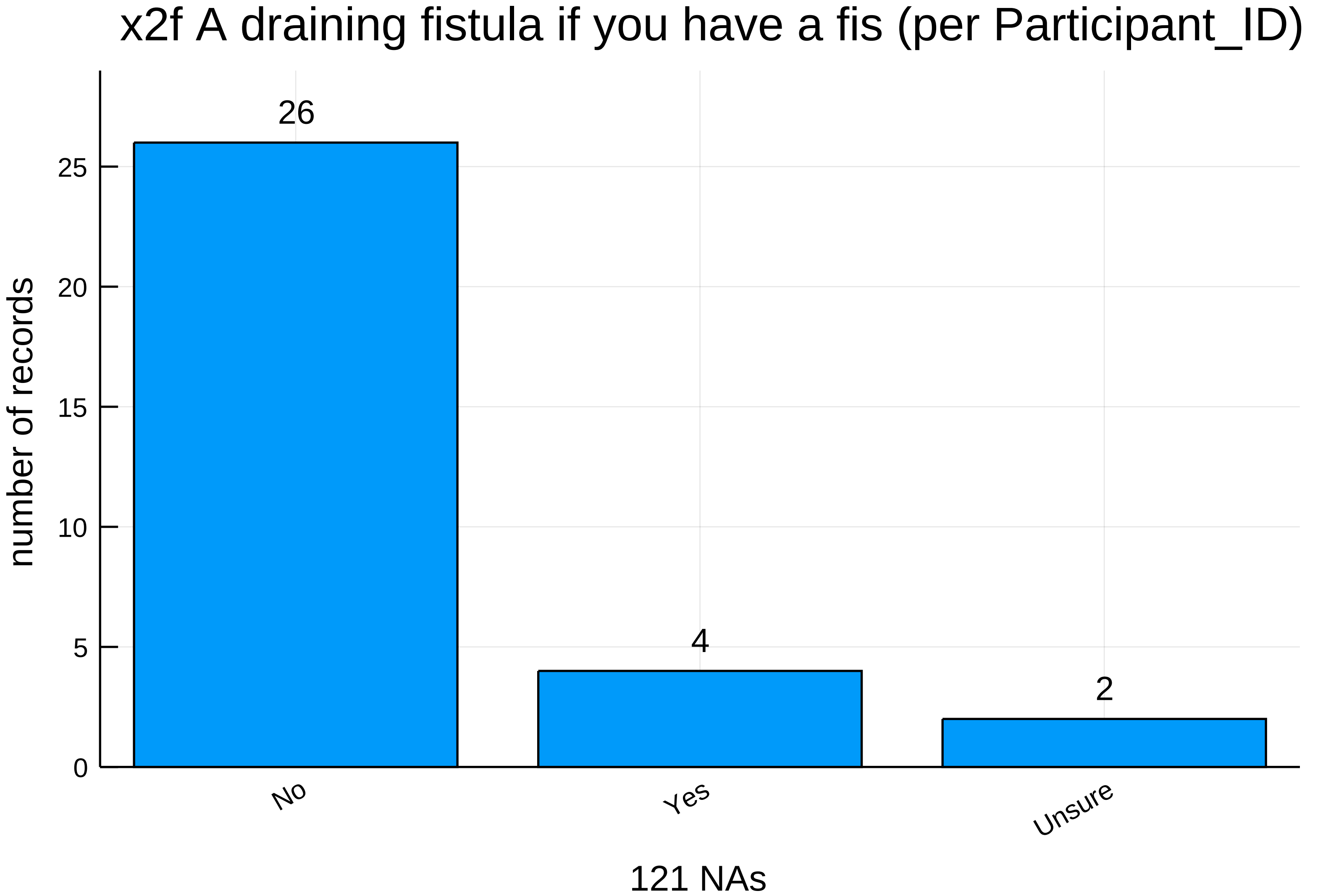
x2f A draining fistula if you have a fis (per Participant_ID)
number of records
0
5
10
15
20
25
26
4
2
No
Yes
Unsure
121 NAs

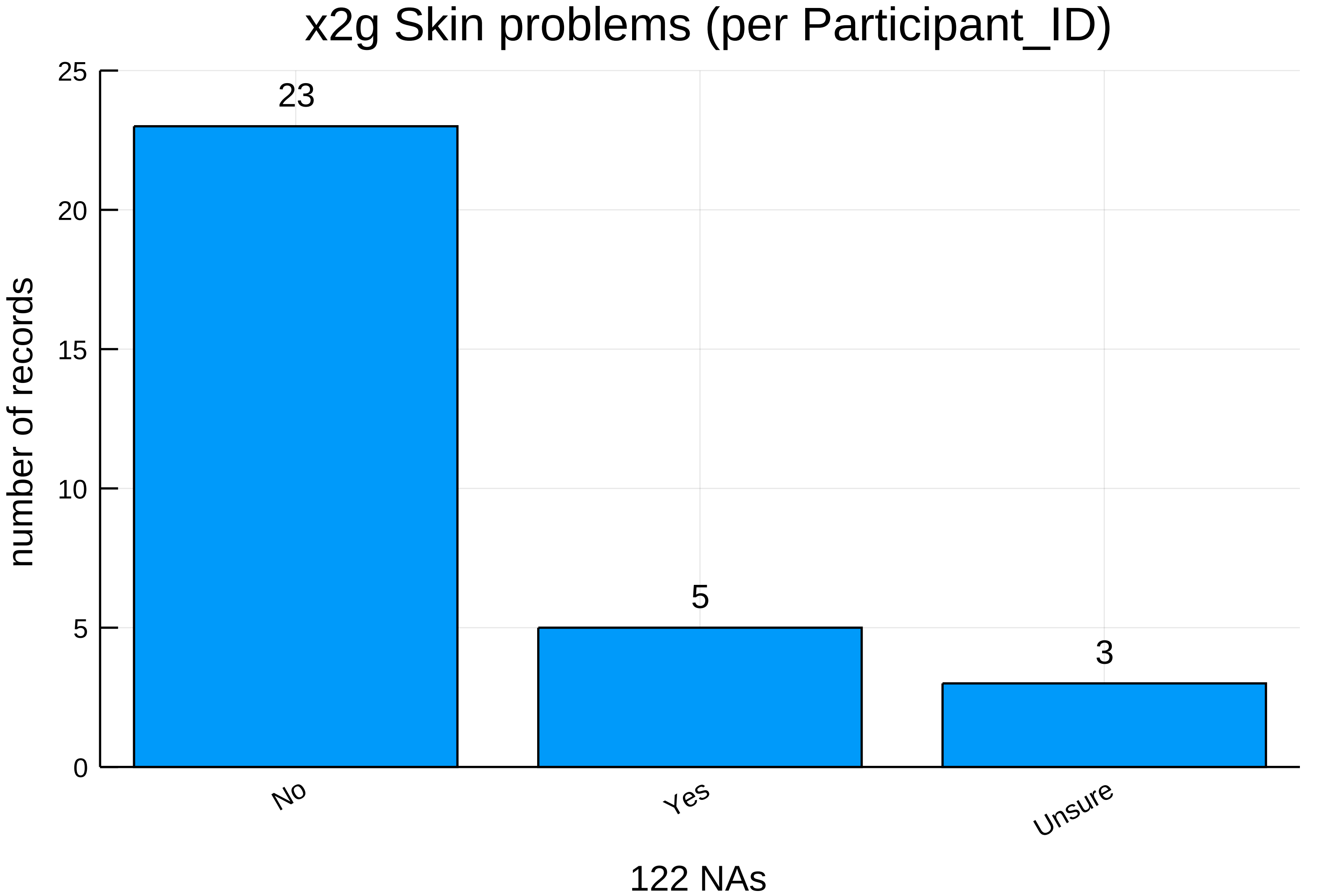
x2g Skin problems (per Participant_ID)
number of records
25
20
15
10
5
0
23
5
3
No
Yes
Unsure
122 NAs

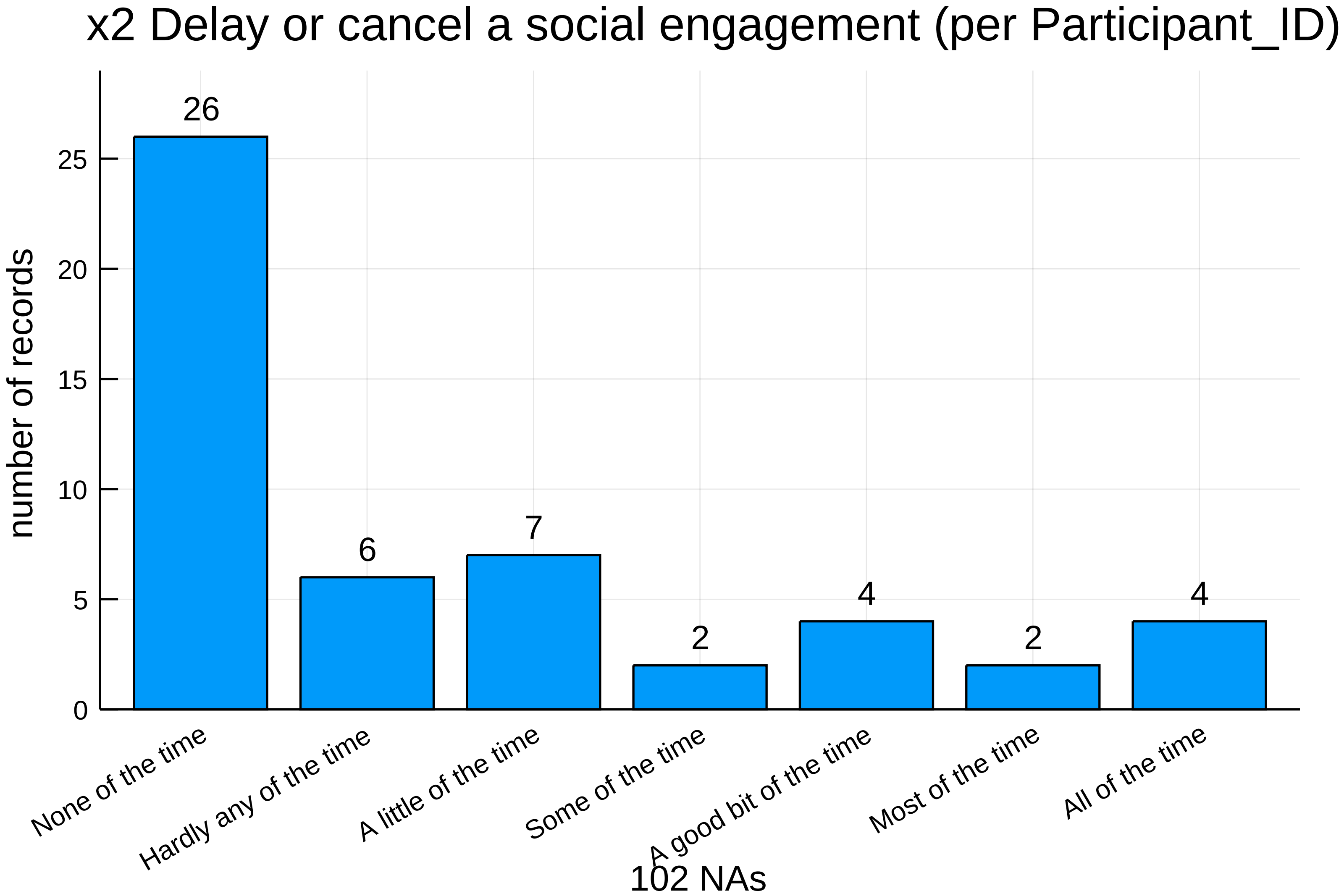
x2 Delay or cancel a social engagement (per Participant_ID)
number of records
0
5
10
15
20
25
26
6
7
2
4
2
4
None of the time
Hardly any of the time
A little of the time
Some of the time
A good bit of the time
Most of the time
All of the time
102 NAs

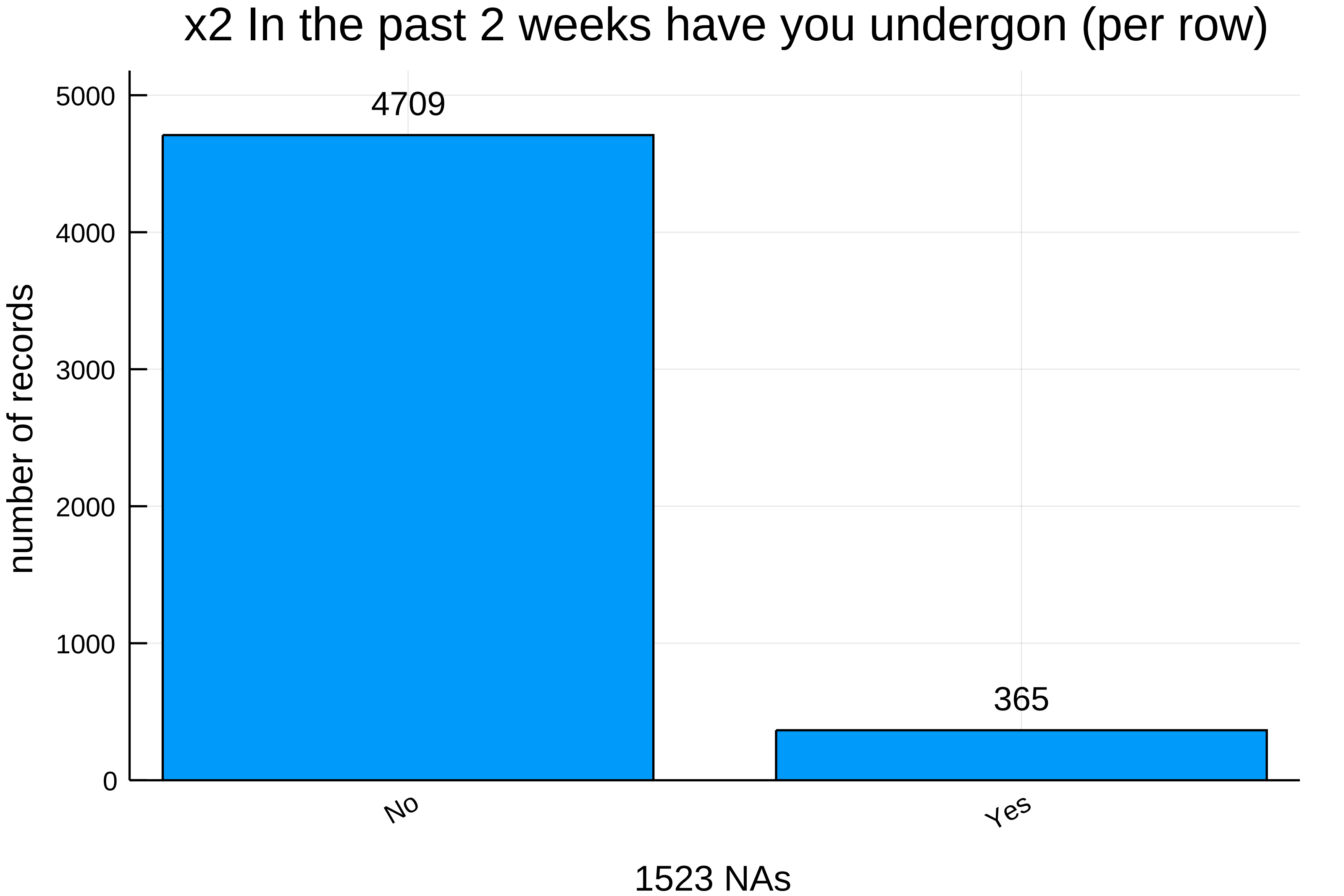
x2 In the past 2 weeks have you undergon (per row)
number of records
5000
4000
3000
2000
1000
0
4709
365
No
Yes
1523 NAs

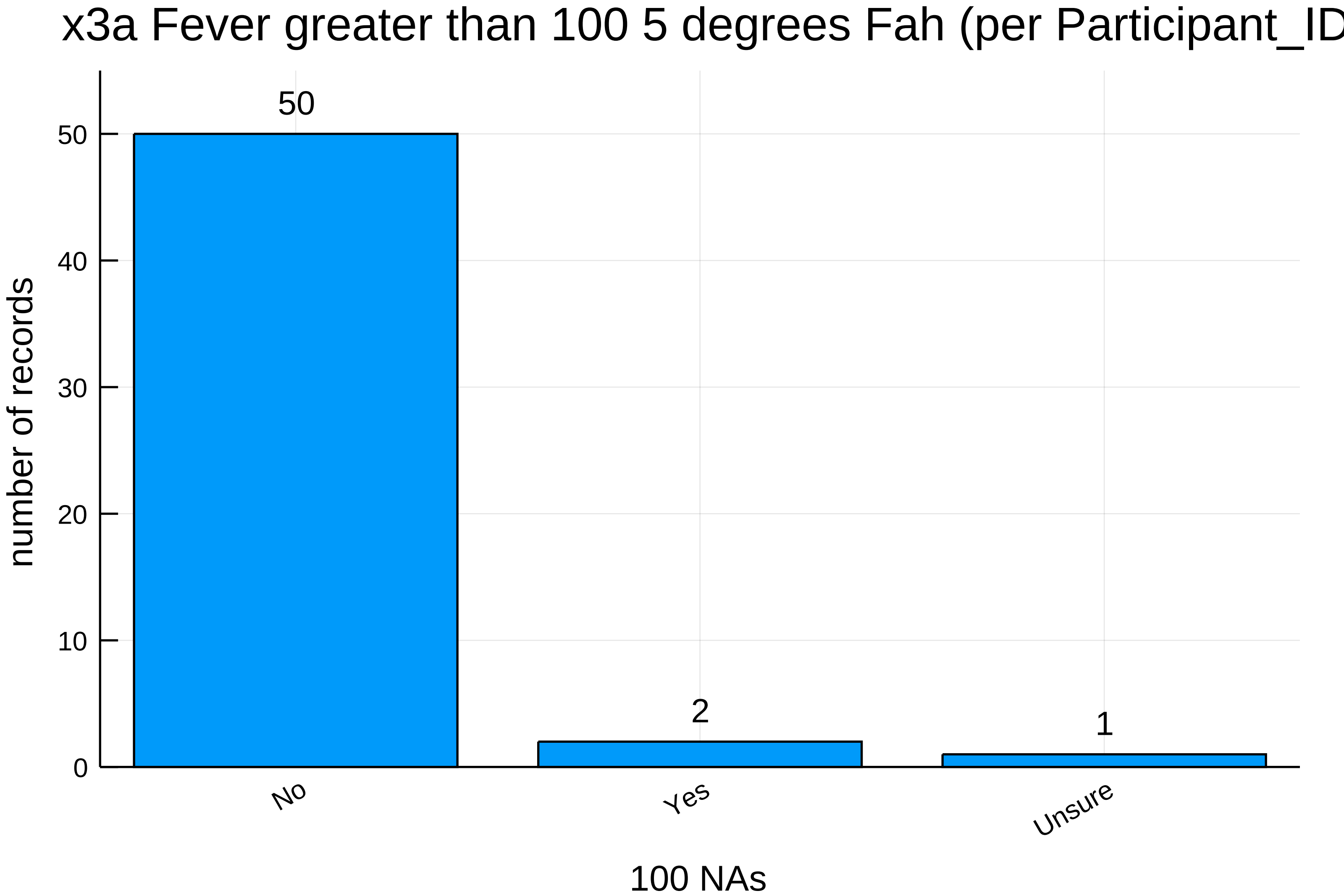
x3a Fever greater than 100 5 degrees Fah (per Participant_ID
number of records
50
40
30
20
10
0
50
2
1
No
Yes
Unsure
100 NAs

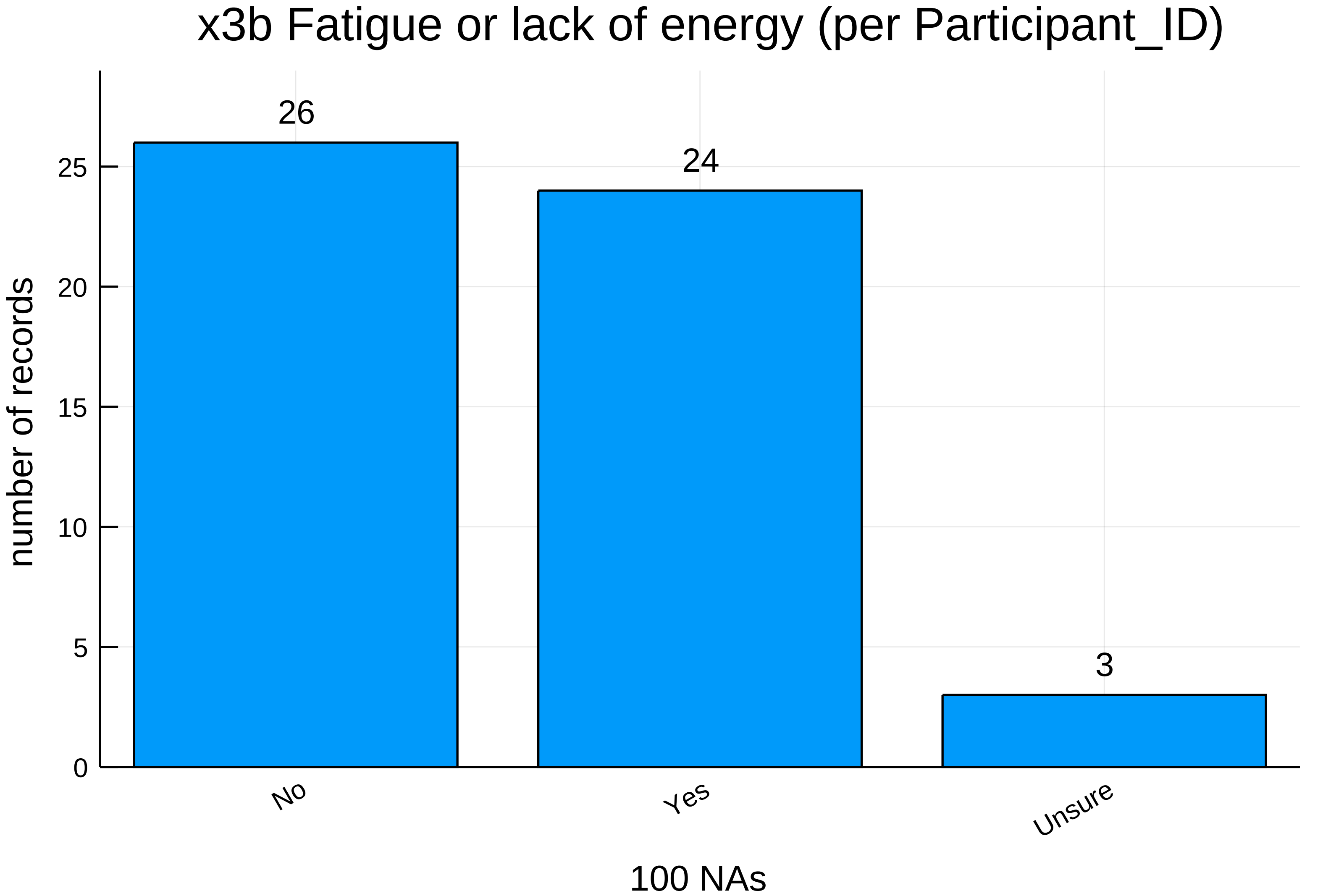
x3b Fatigue or lack of energy (per Participant_ID)
number of records
0
5
10
15
20
25
26
24
3
No
Yes
Unsure
100 NAs

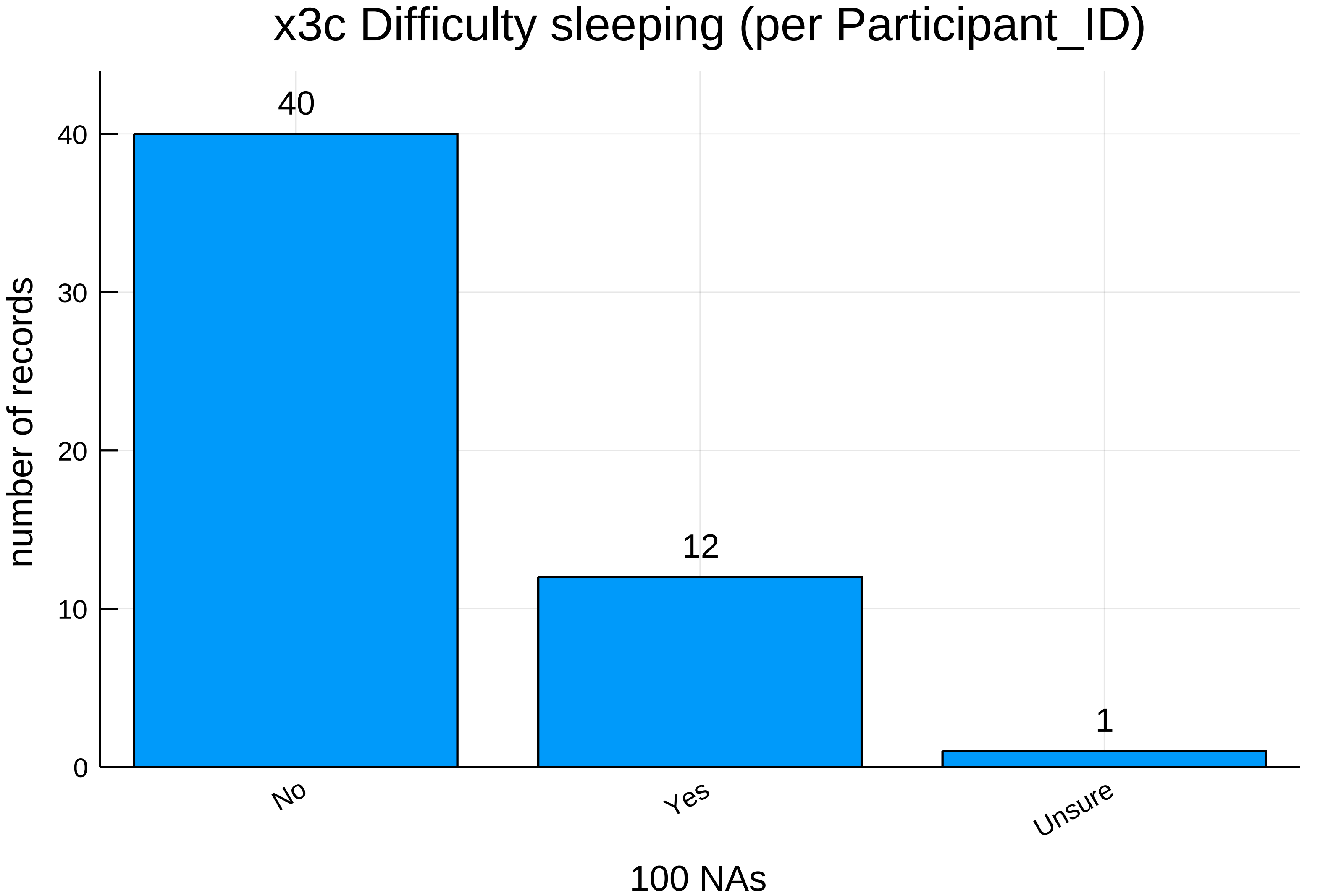
x3c Difficulty sleeping (per Participant_ID)
number of records
0
10
20
30
40
40
12
1
No
Yes
Unsure
100 NAs

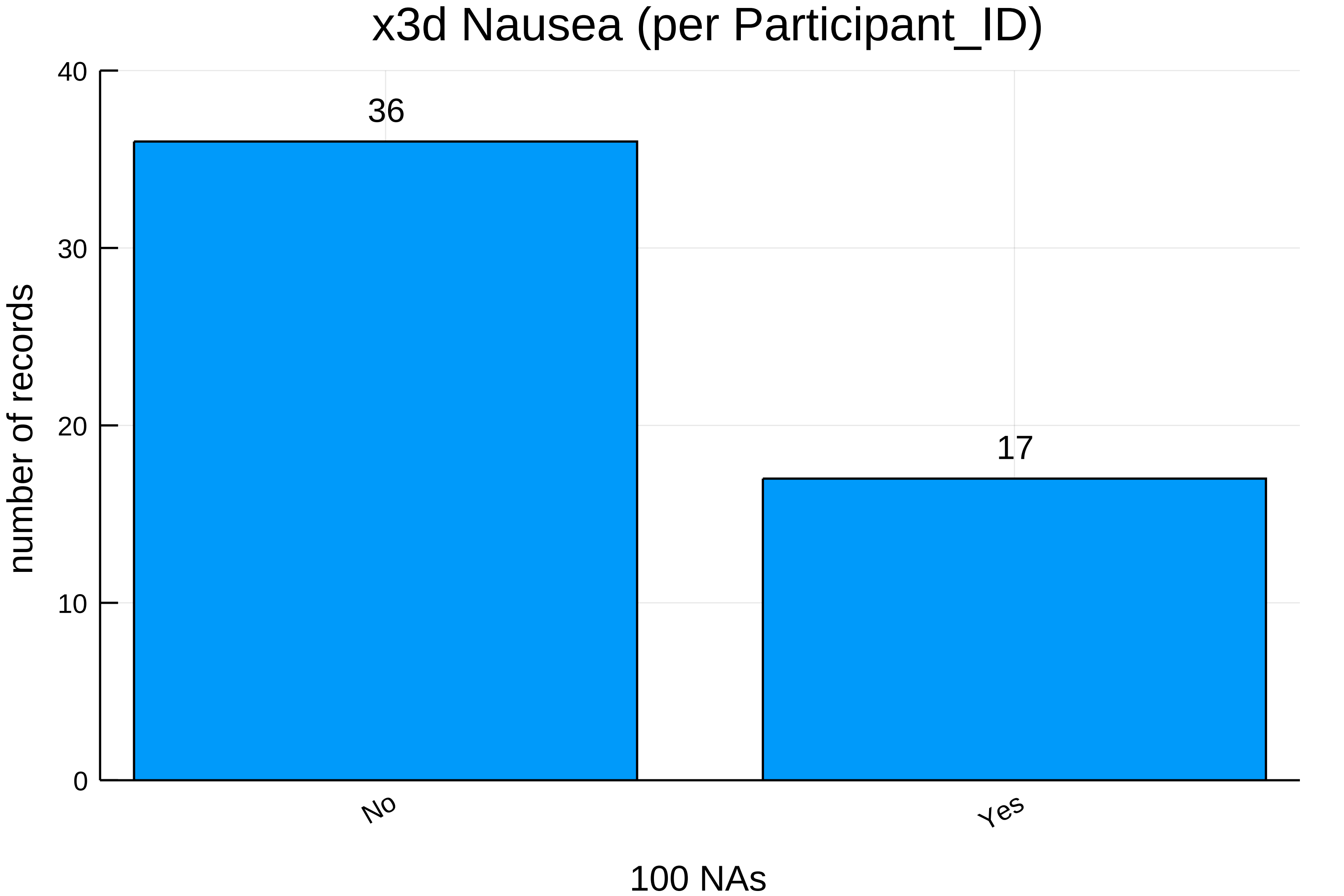

x3d Nausea (per Participant_ID)
number of records
40
30
20
10
0
36
17
No
Yes
100 NAs

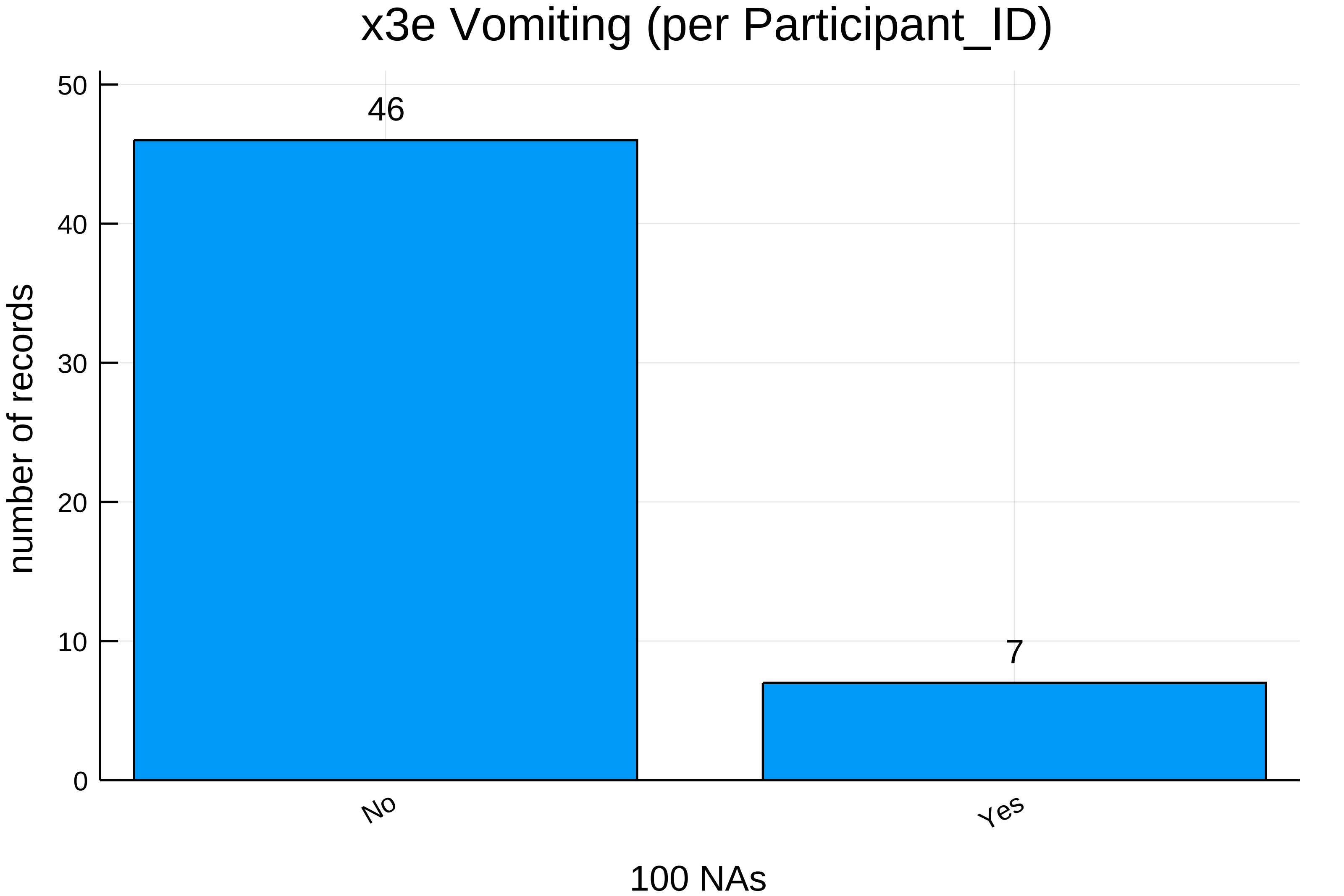
x3e Vomiting (per Participant_ID)
number of records
0
10
20
30
40
50
46
7
No
Yes
100 NAs

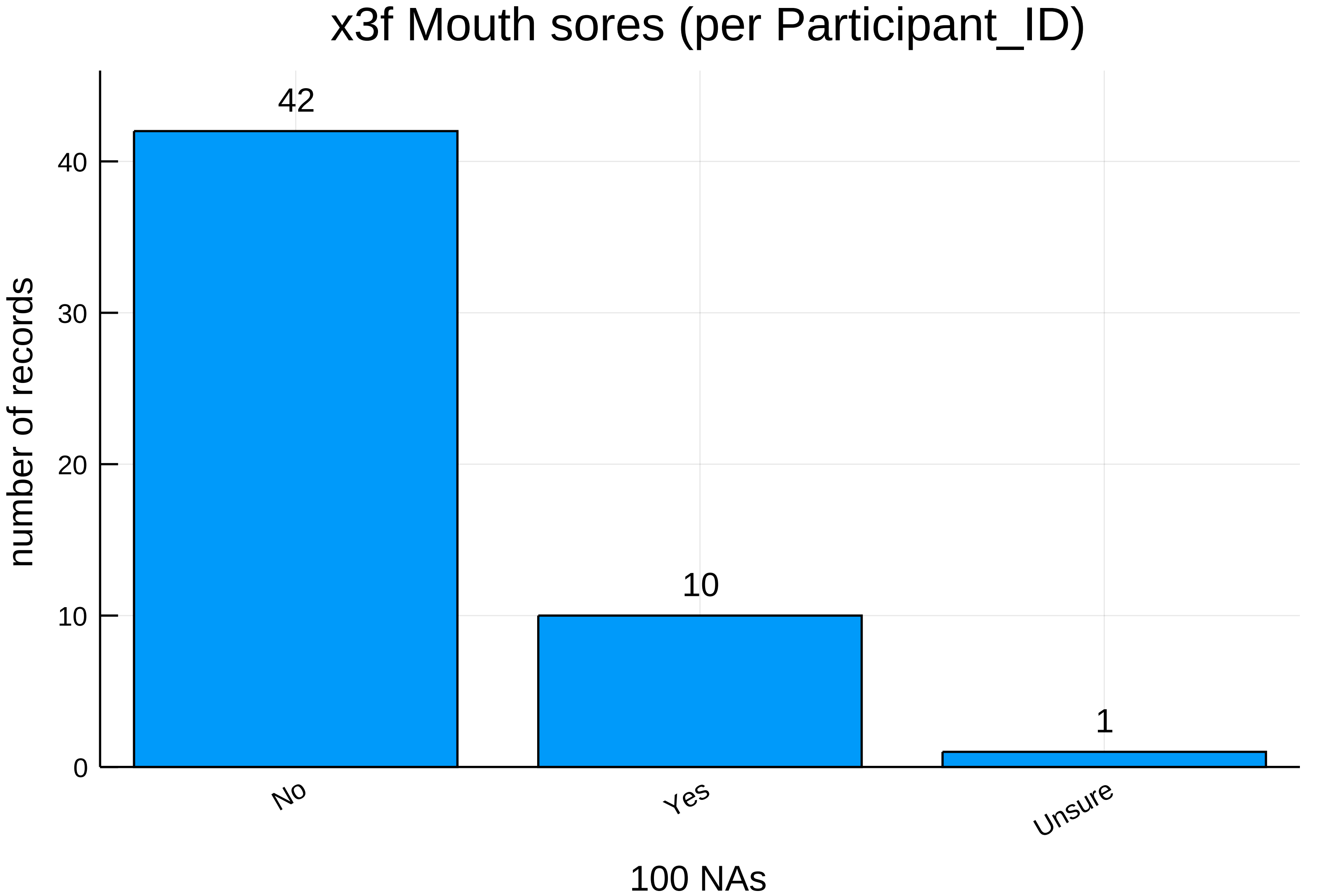
x3f Mouth sores (per Participant_ID)
number of records
0
10
20
30
40
42
10
1
No
Yes
Unsure
100 NAs

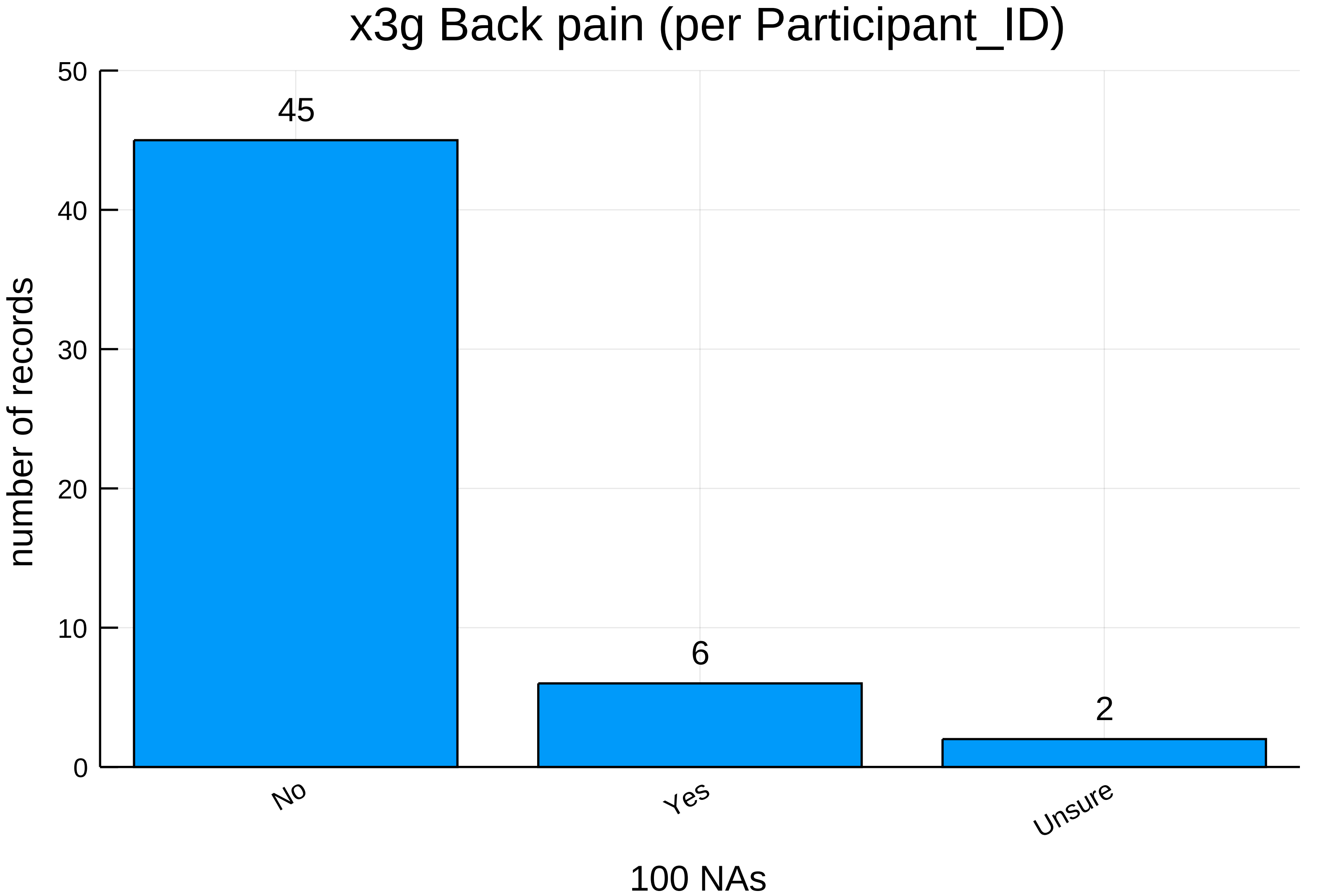
x3g Back pain (per Participant_ID)
number of records
50
40
30
20
10
0
45
6
2
No
Yes
Unsure
100 NAs

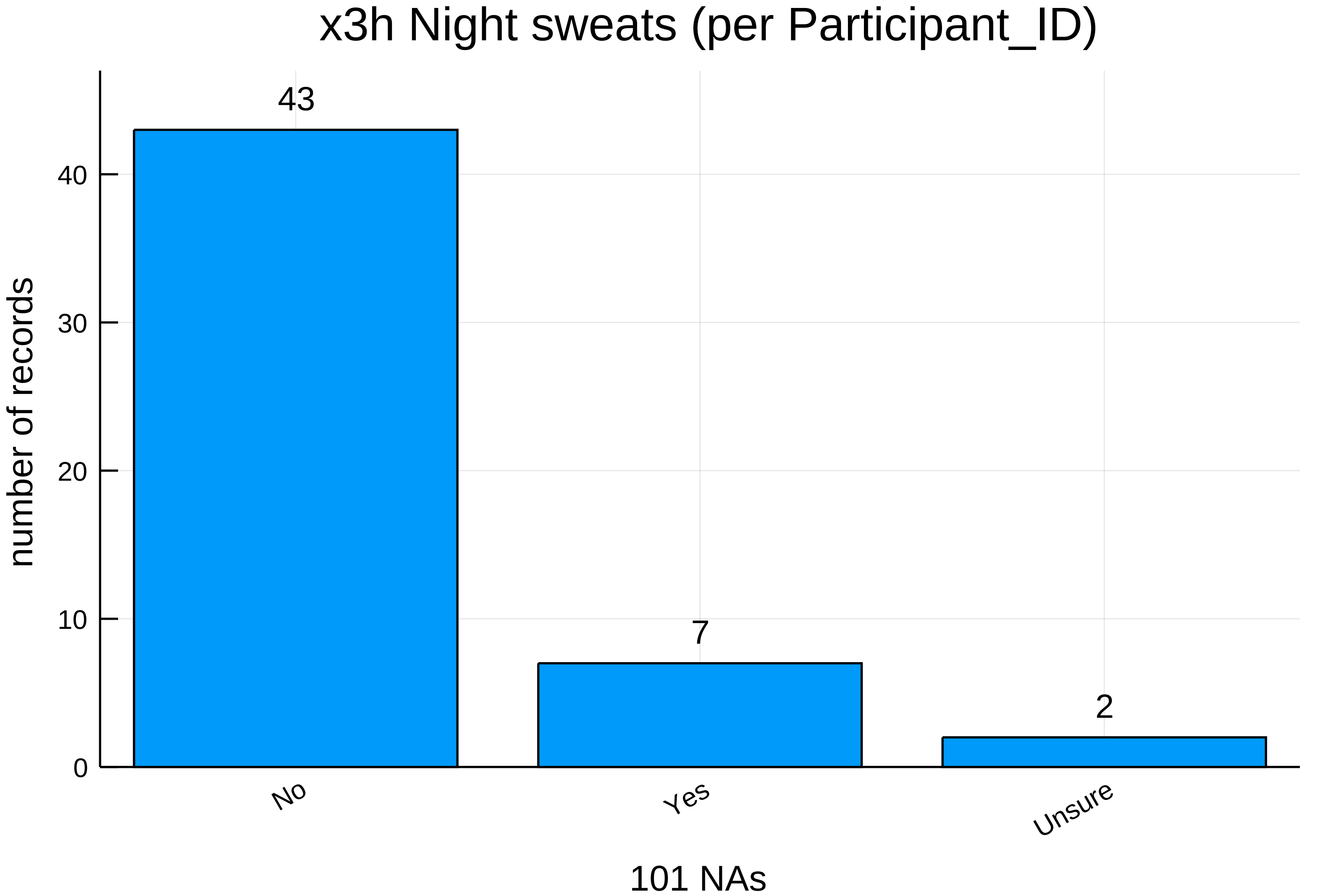
x3h Night sweats (per Participant_ID)
number of records
0
10
20
30
40
43
7
2
No
Yes
Unsure
101 NAs

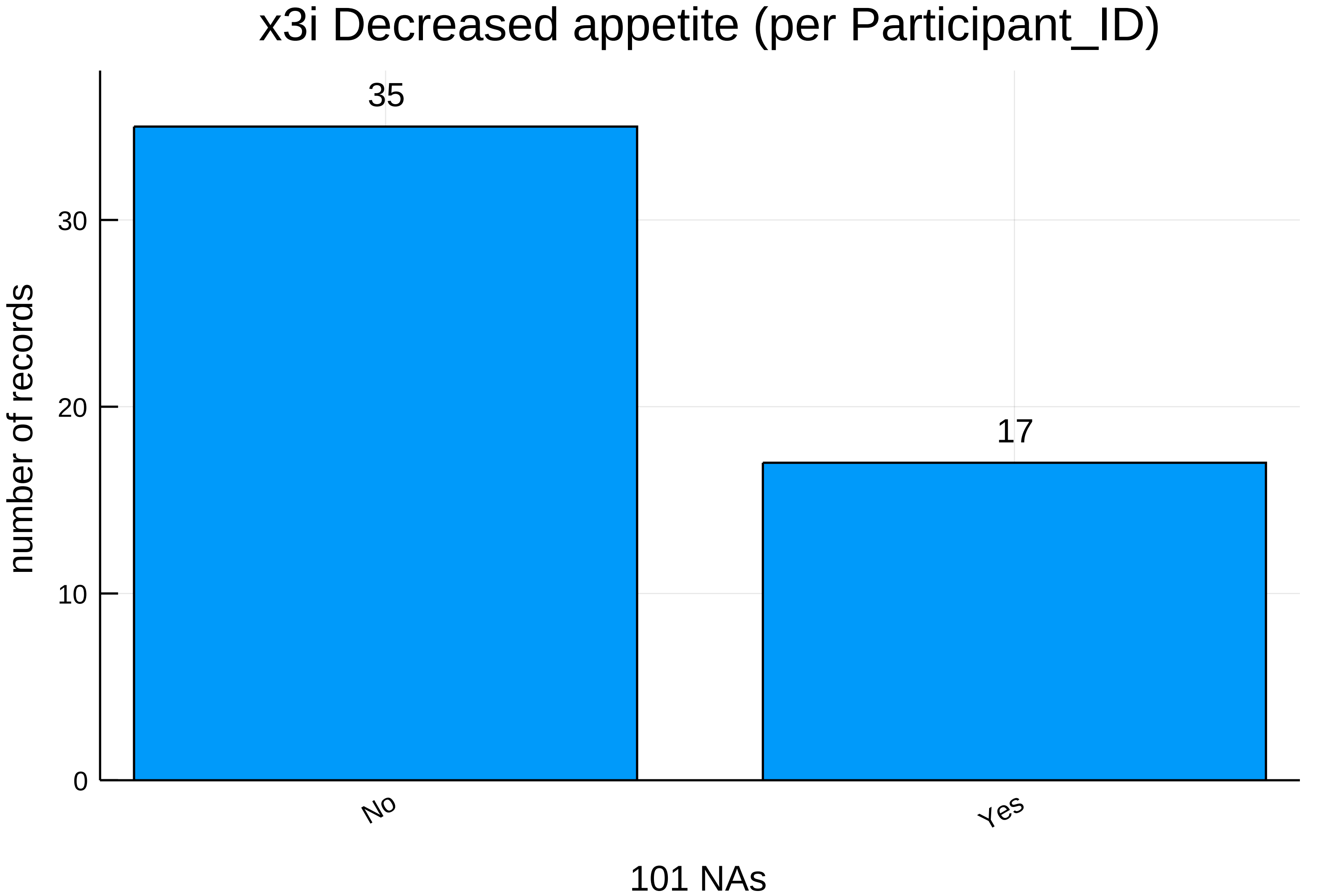

x3i Decreased appetite (per Participant_ID)
number of records
35
17
0
10
20
30
No
Yes
101 NAs

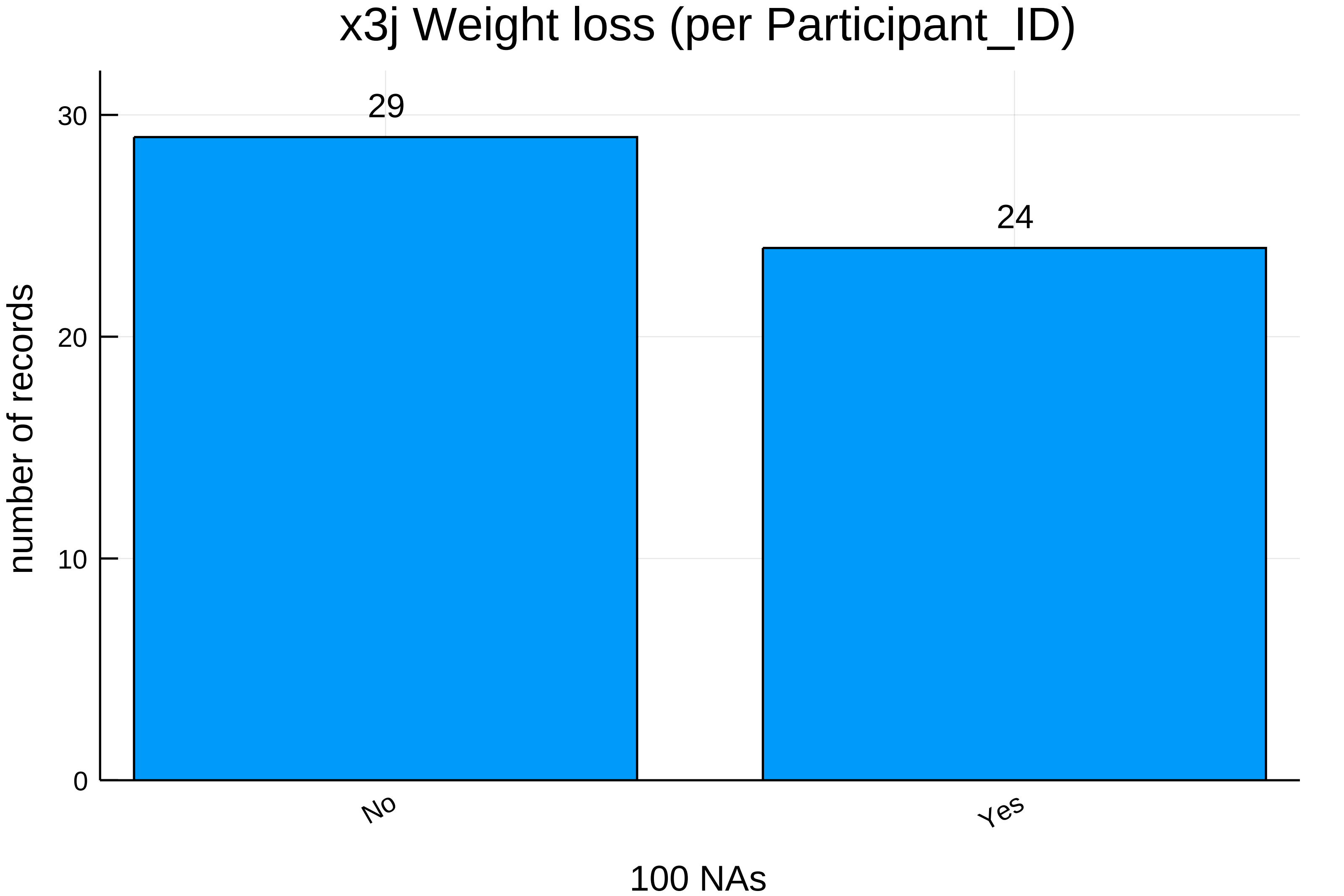

x3j Weight loss (per Participant_ID)
number of records
30
20
10
0
29
24
No
Yes
100 NAs

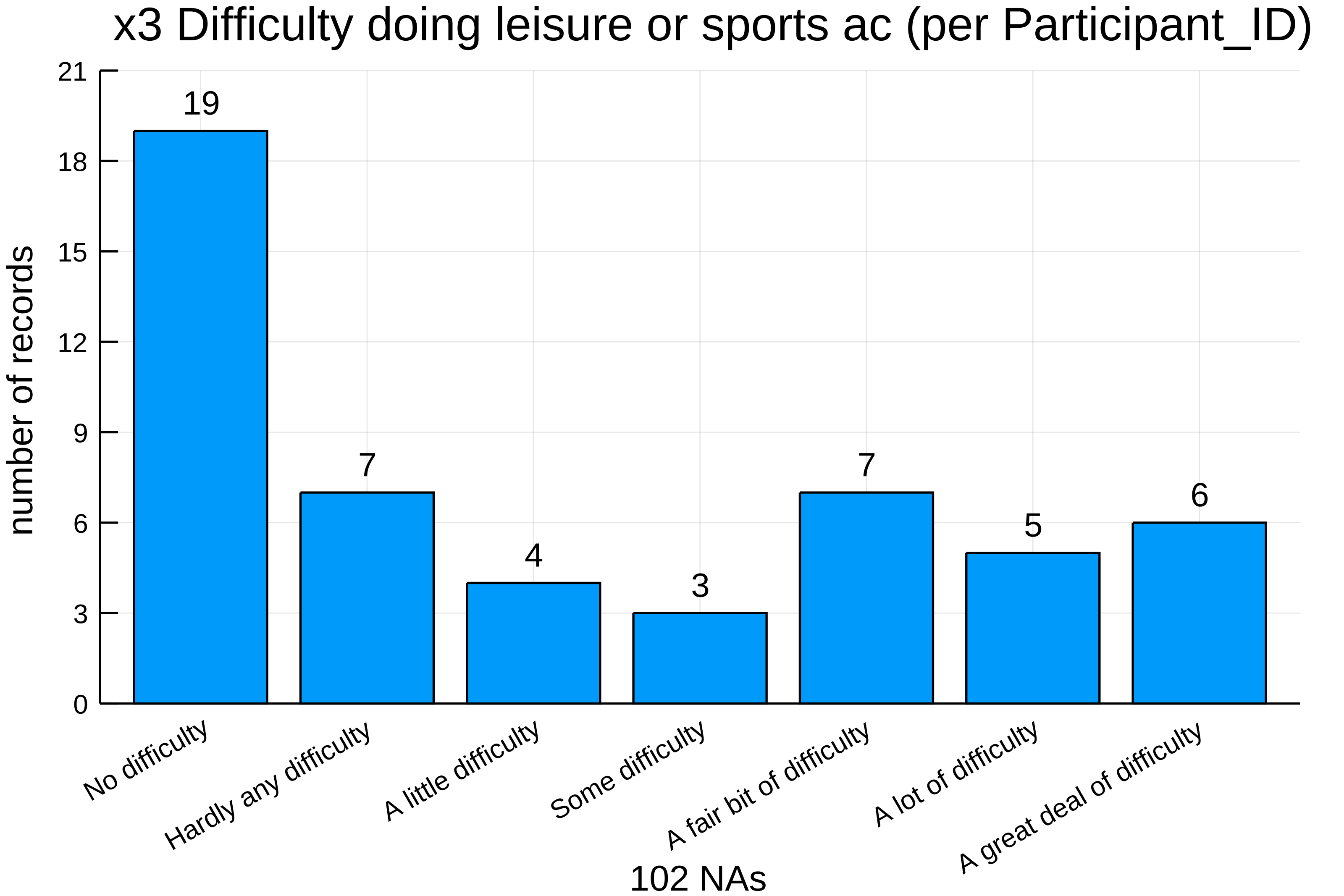
x3 Difficulty doing leisure or sports ac (per Participant_ID)
number of records
0
3
6
9
12
15
18
21
19
7
4
3
7
5
6
No difficulty
Hardly any difficulty
A little difficulty
Some difficulty
A fair bit of difficulty
A lot of difficulty
A great deal of difficulty
102 NAs

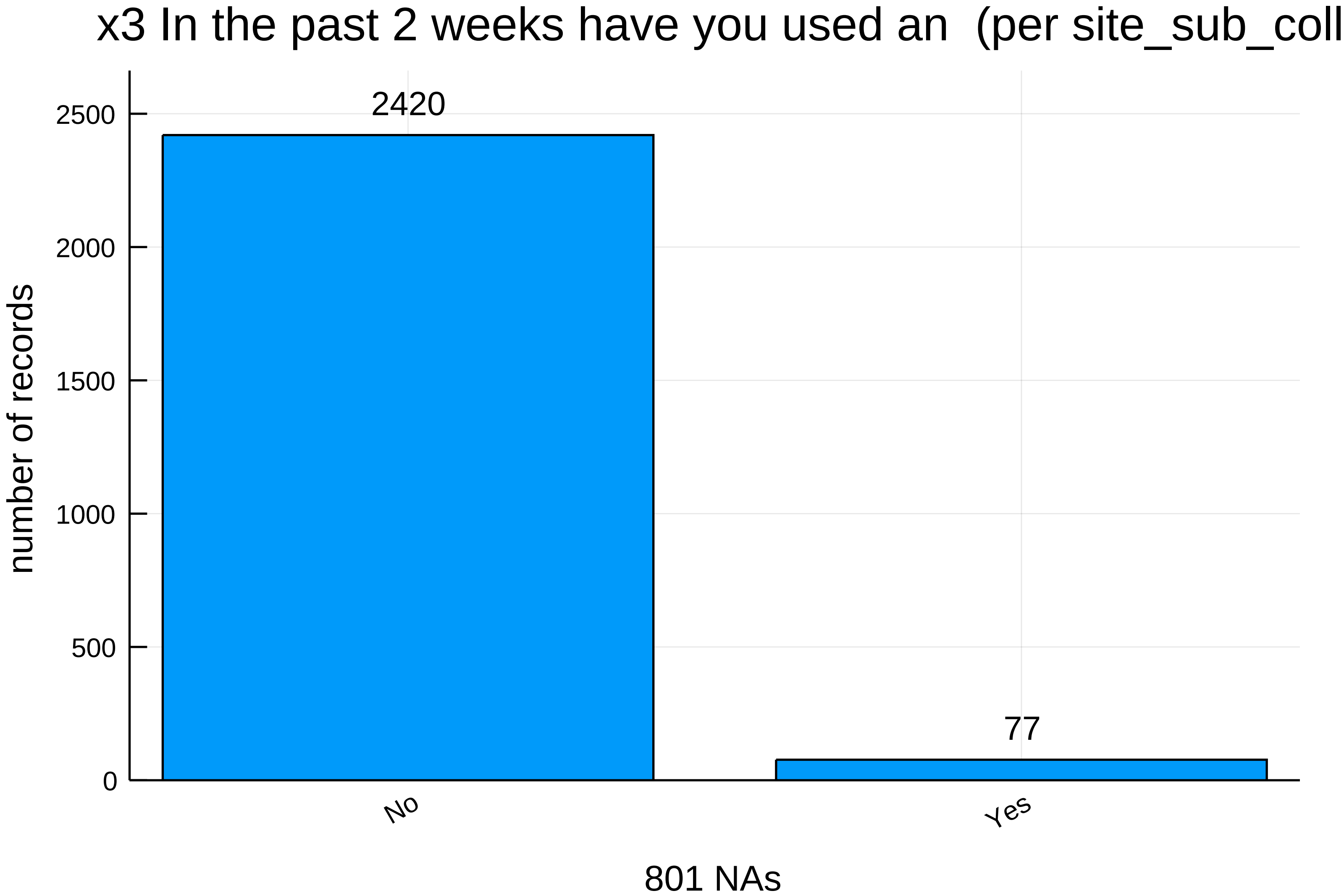
x3 In the past 2 weeks have you used an (per site_sub_coll
number of records
2500
2000
1500
1000
500
0
2420
77
No
Yes
801 NAs

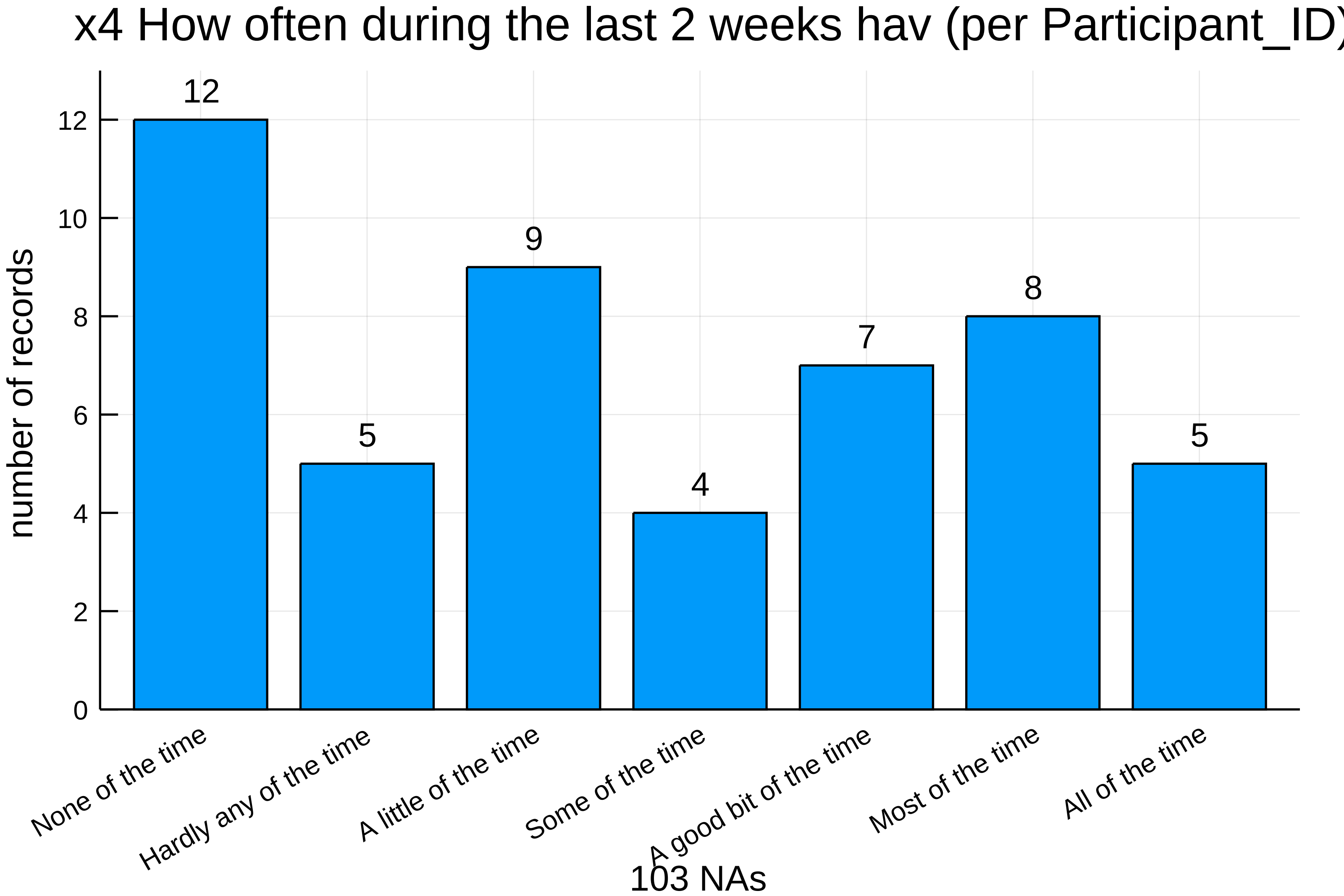
x4 How often during the last 2 weeks hav (per Participant_ID)
number of records
0
2
4
6
8
10
12
12
5
9
4
7
8
5
None of the time
Hardly any of the time
A little of the time
Some of the time
A good bit of the time
Most of the time
All of the time
103 NAs

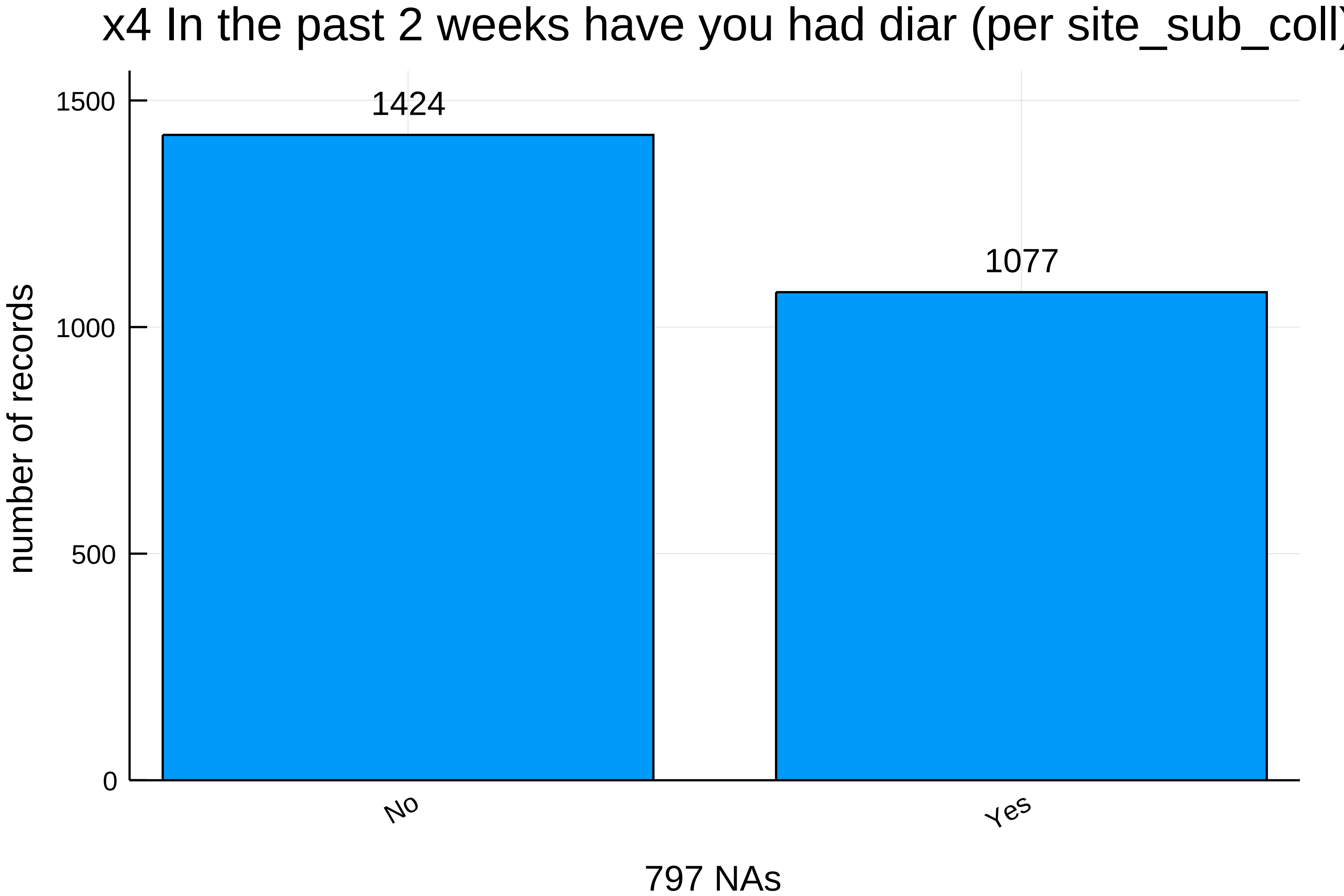
x4 In the past 2 weeks have you had diar (per site_sub_coll)
number of records
1500
1000
500
0
1424
1077
No
Yes
797 NAs

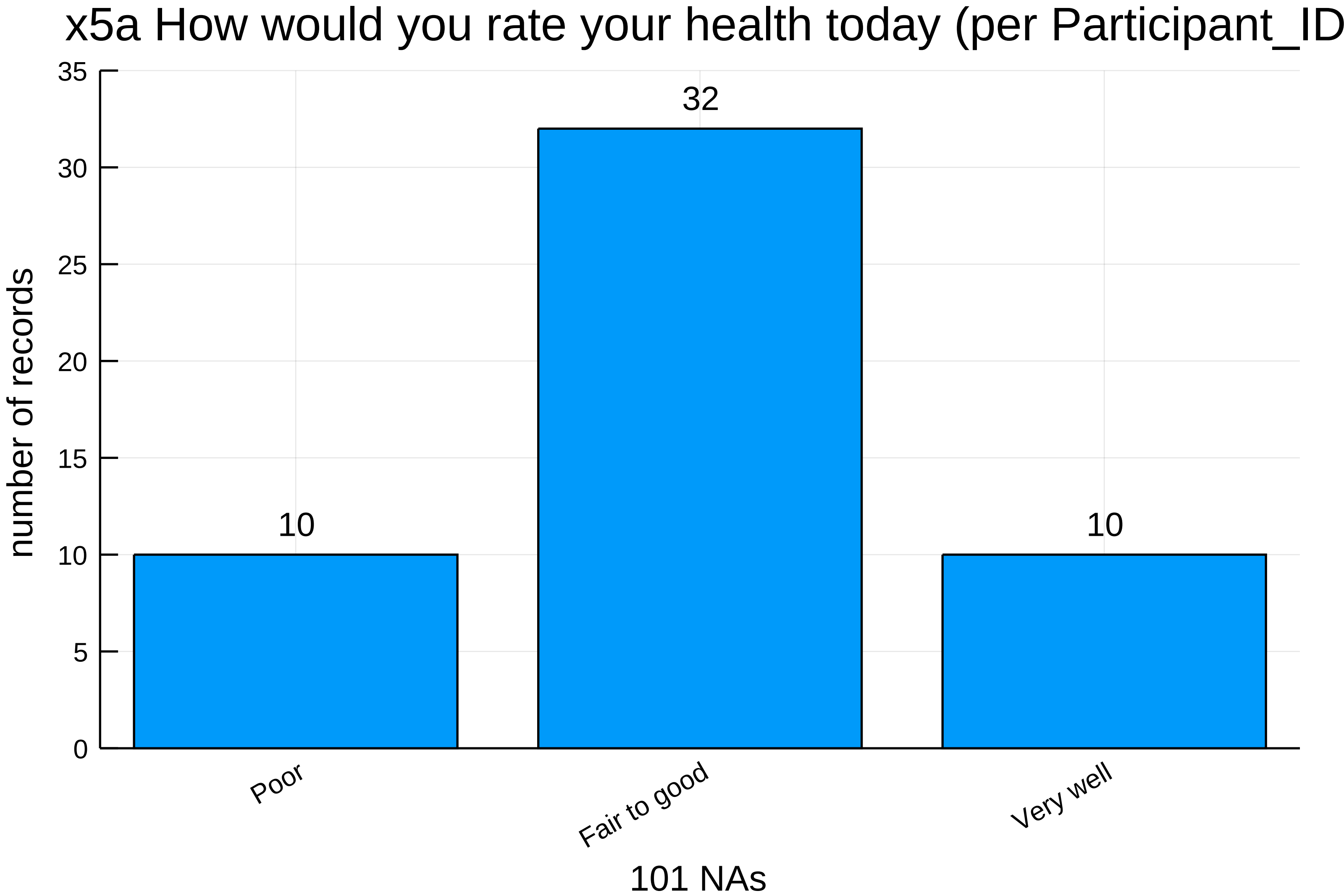
x5a How would you rate your health today (per Participant_ID
number of records
35
30
25
20
15
10
5
0
10
32
10
Poor
Fair to good
Very well
101 NAs

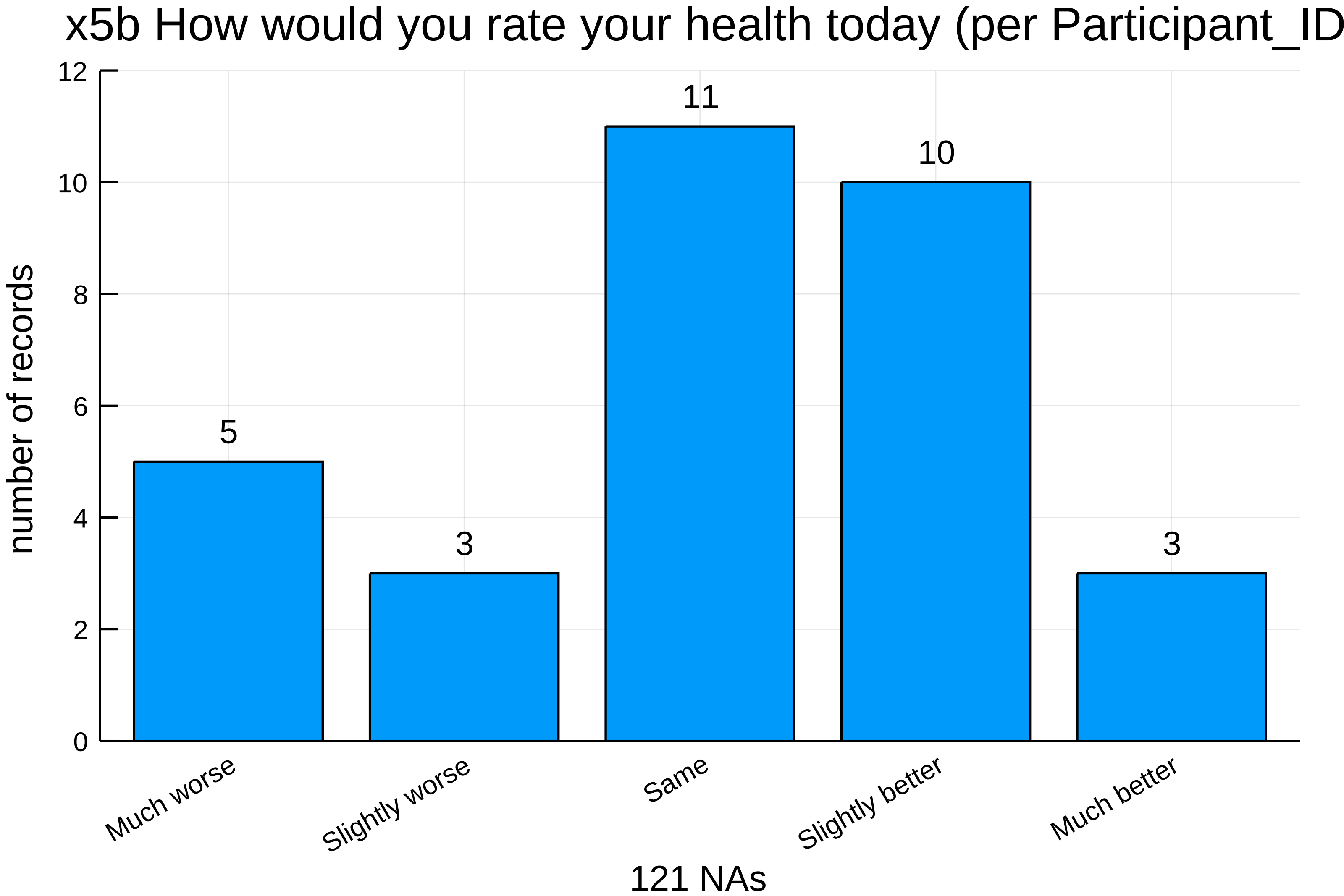
x5b How would you rate your health today (per Participant_ID
12
10
8
6
4
2
0
number of records
5
3
11
10
3
Much worse
Slightly worse
Same
Slightly better
Much better
121 NAs

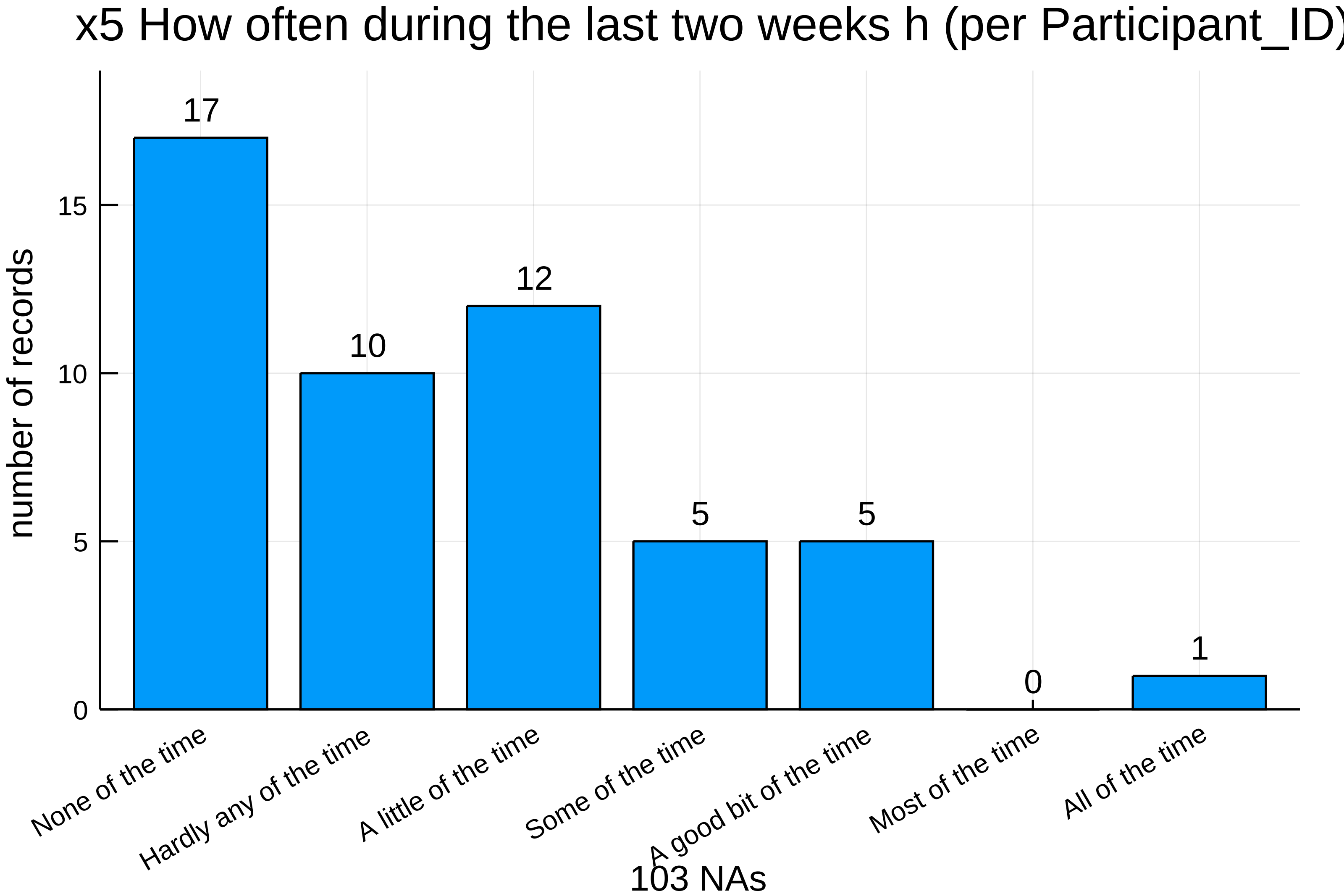
x5 How often during the last two weeks h (per Participant_ID)
number of records
0
5
10
15
17
10
12
5
5
0
1
None of the time
Hardly any of the time
A little of the time
Some of the time
A good bit of the time
Most of the time
All of the time
103 NAs

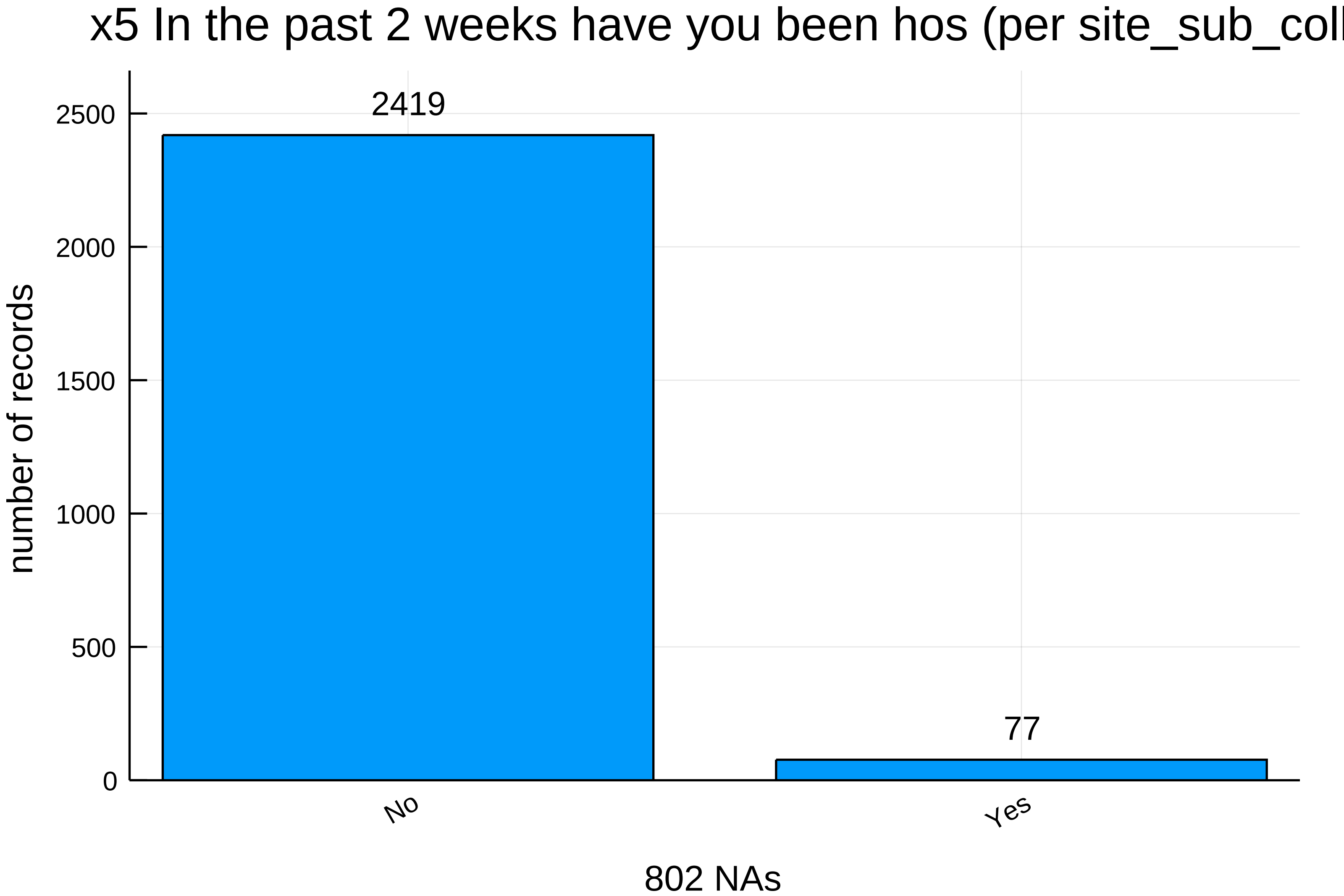
x5 In the past 2 weeks have you been hos (per site_sub_coll
number of records
2500
2000
1500
1000
500
0
2419
77
No
Yes
802 NAs

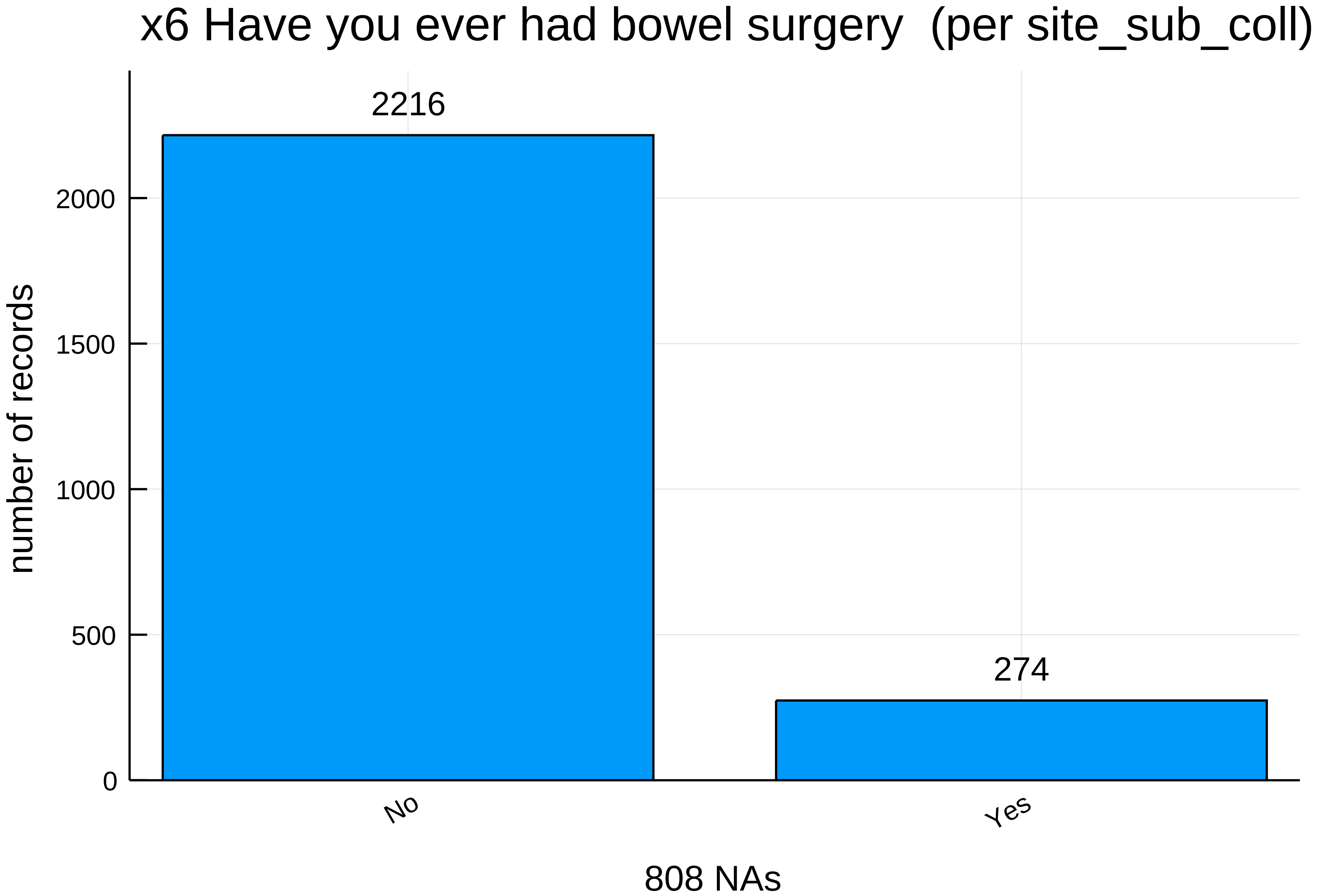
x6 Have you ever had bowel surgery (per site_sub_coll)
number of records
2216
274
0
500
1000
1500
2000
No
Yes
808 NAs

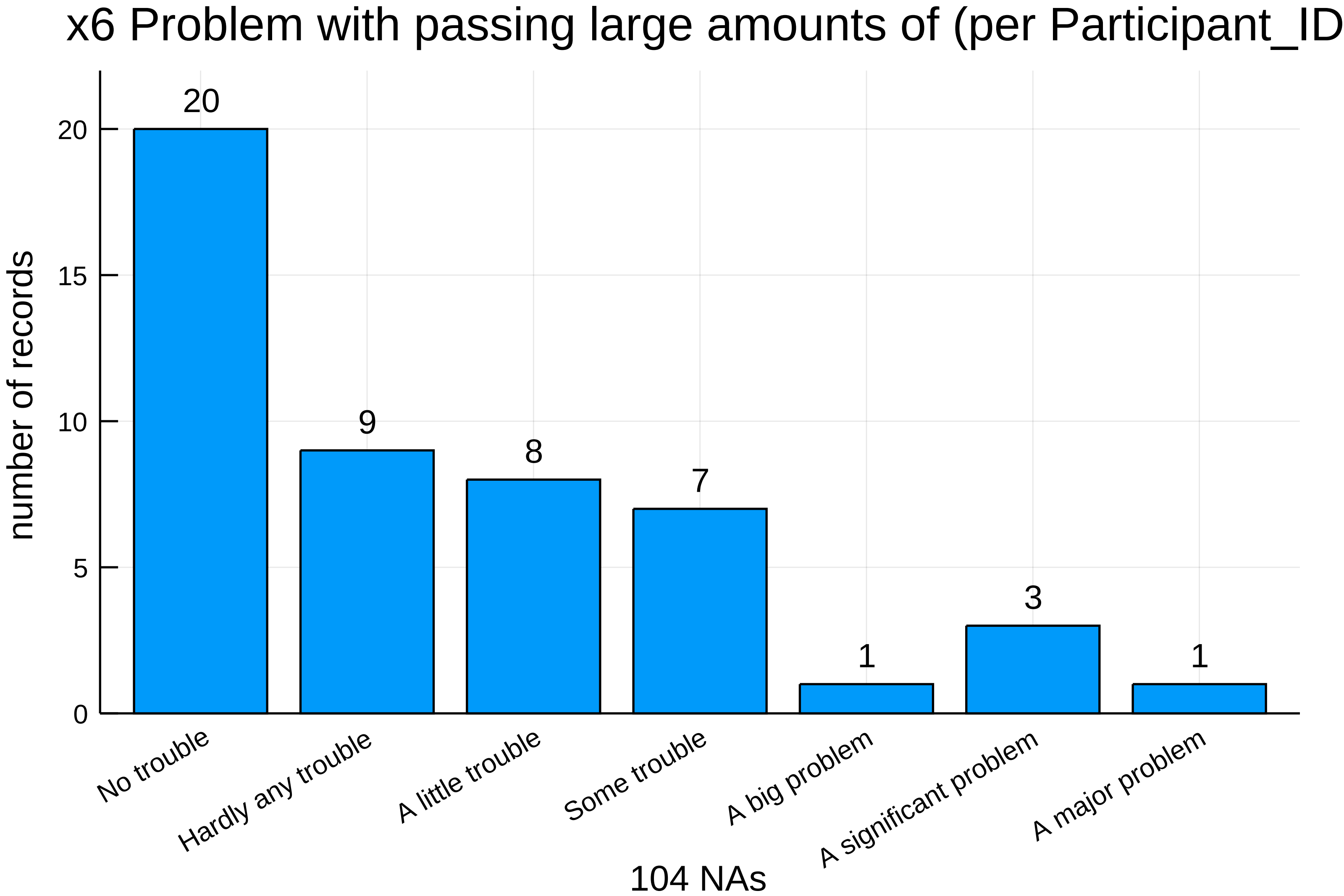
x6 Problem with passing large amounts of (per Participant_ID
number of records
20
9
8
7
1
3
1
0
5
10
15
20
No trouble
Hardly any trouble
A little trouble
Some trouble
A big problem
A significant problem
A major problem
104 NAs

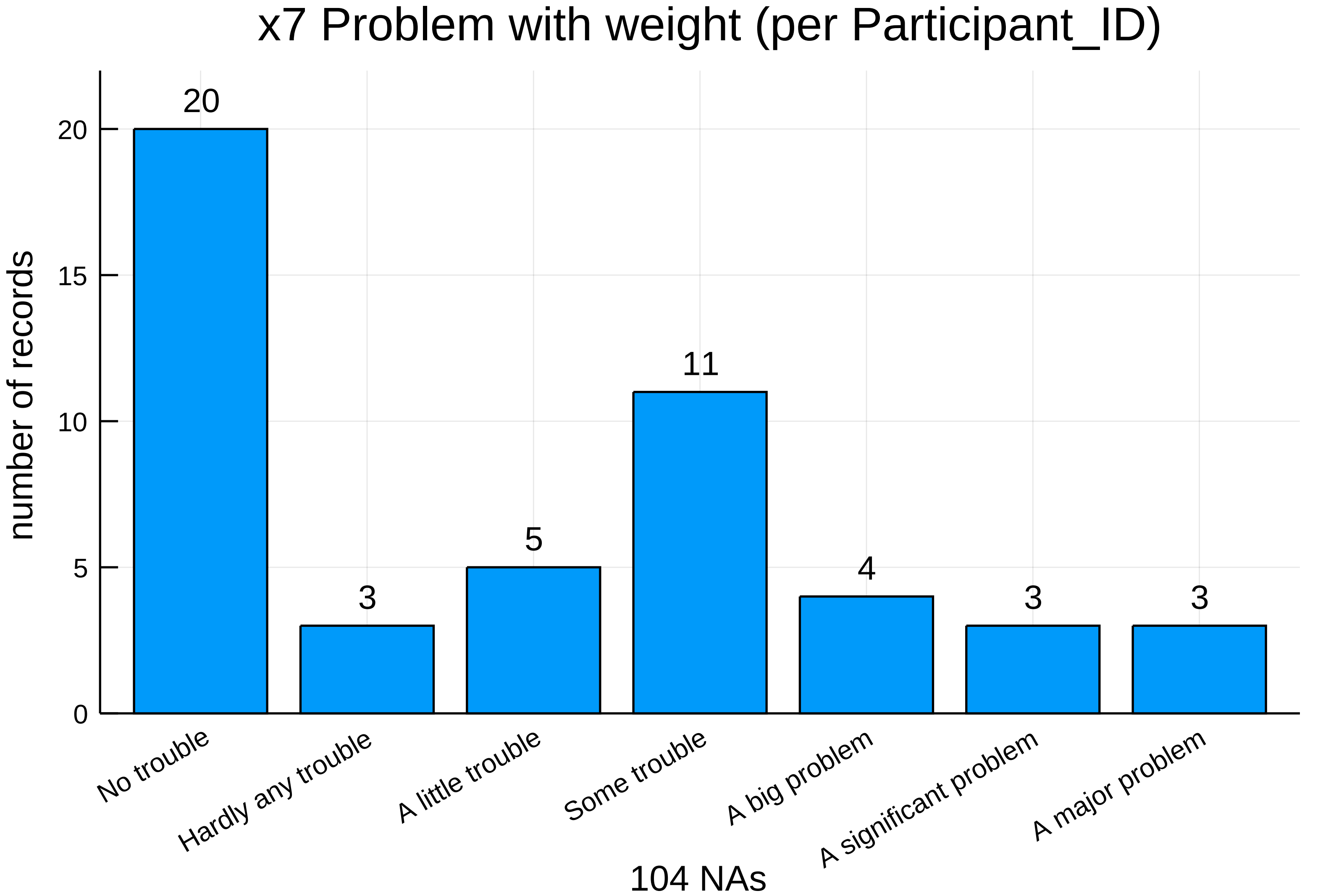
x7 Problem with weight (per Participant_ID)
number of records
20
15
10
5
0
20
3
5
11
4
3
3
No trouble
Hardly any trouble
A little trouble
Some trouble
A big problem
A significant problem
A major problem
104 NAs

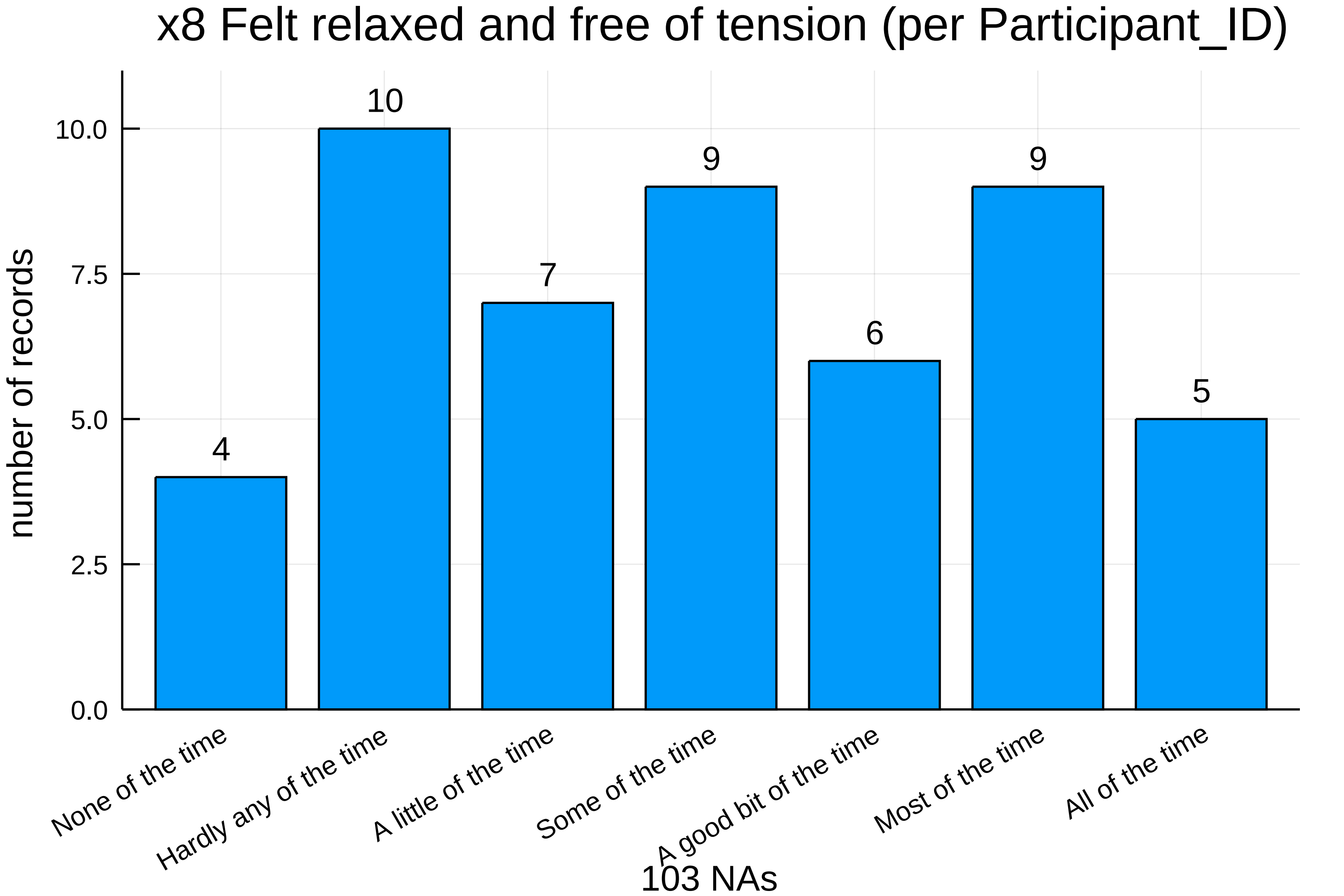
x8 Felt relaxed and free of tension (per Participant_ID)
number of records
10.0
7.5
5.0
2.5
0.0
4
10
7
9
6
9
5
None of the time
Hardly any of the time
A little of the time
Some of the time
A good bit of the time
Most of the time
All of the time
103 NAs

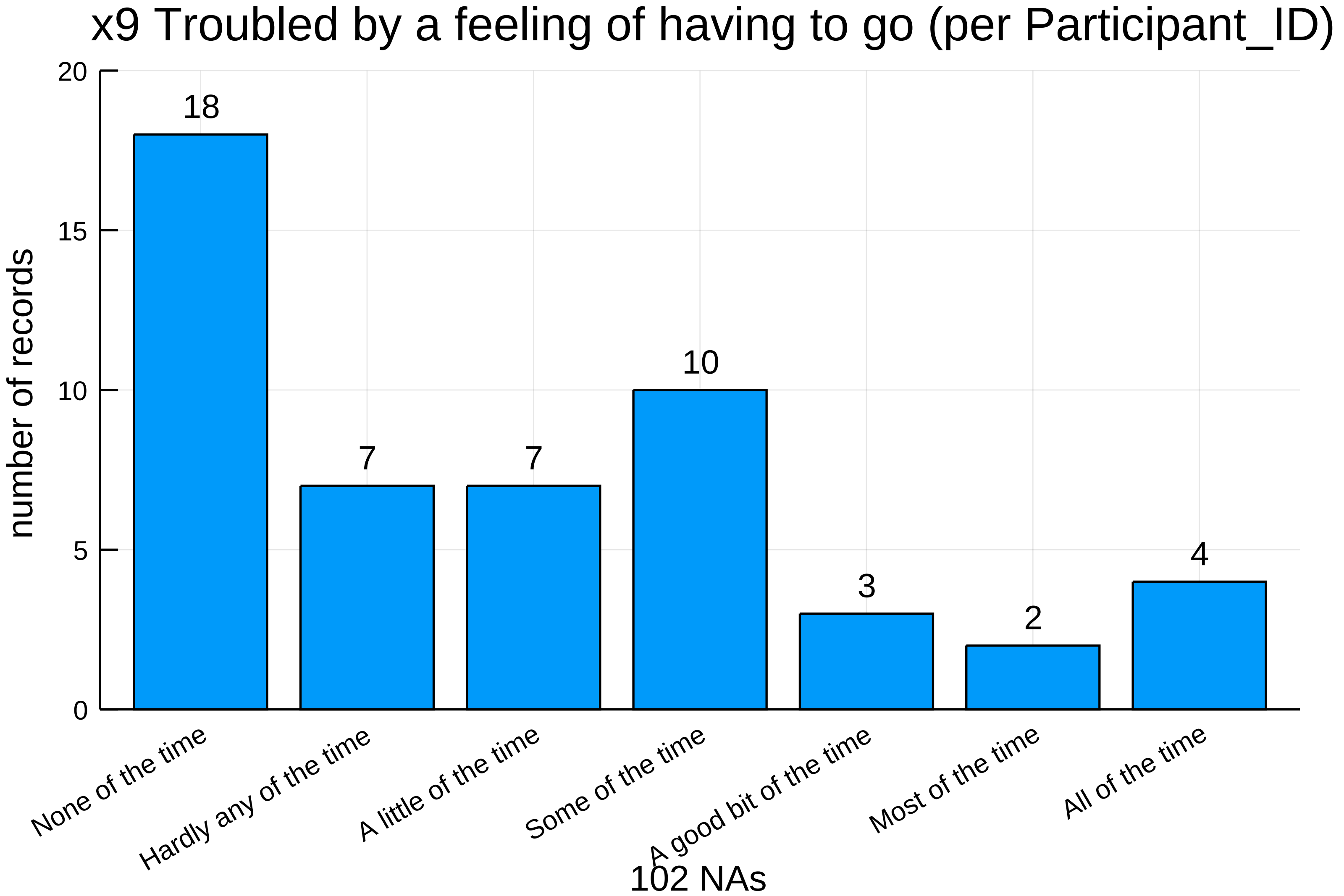
x9 Troubled by a feeling of having to go (per Participant_ID)
number of records
0
5
10
15
20
18
7
7
10
3
2
4
None of the time
Hardly any of the time
A little of the time
Some of the time
A good bit of the time
Most of the time
All of the time
102 NAs

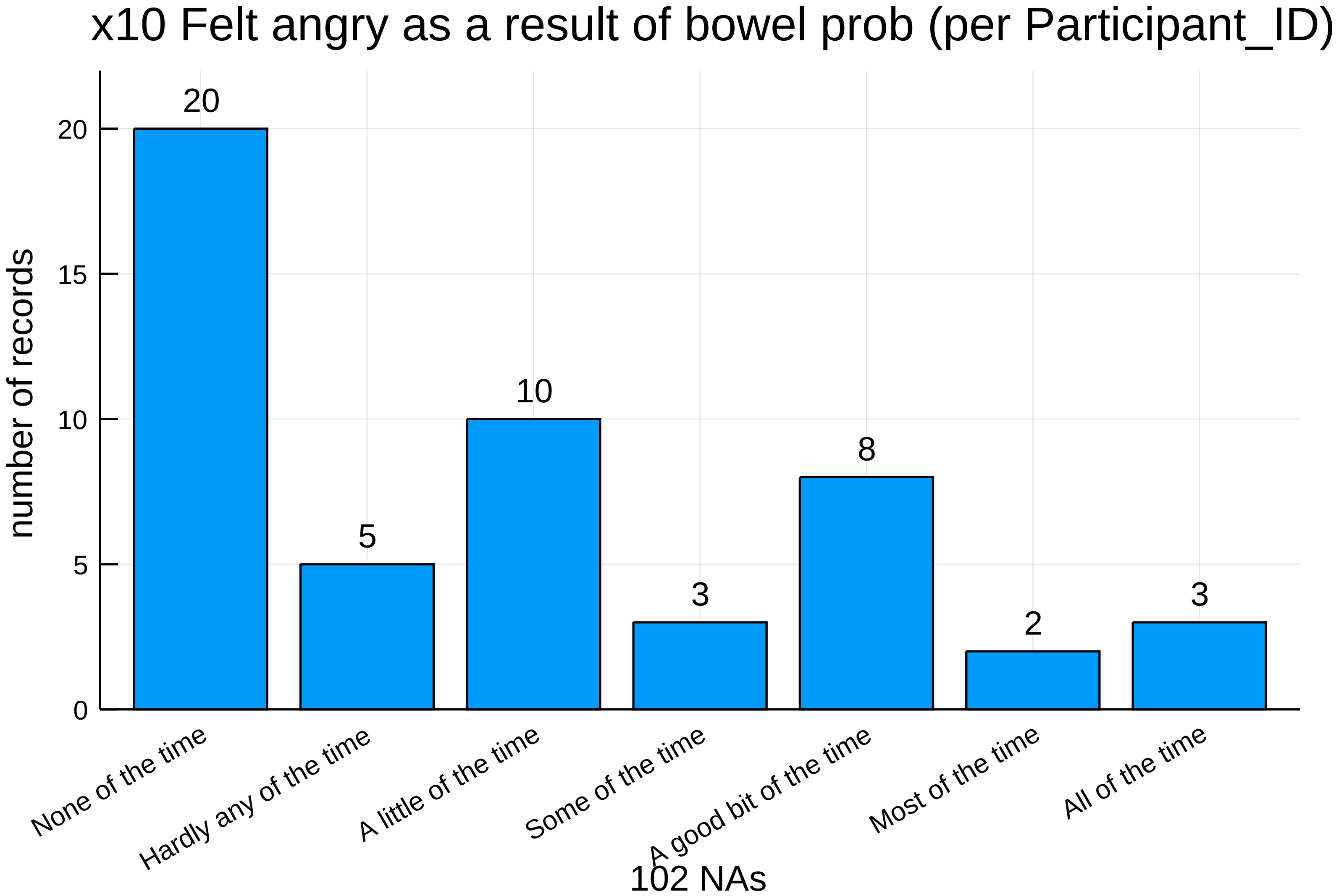
x10 Felt angry as a result of bowel prob (per Participant_ID)
number of records
0
5
10
15
20
20
5
10
3
8
2
3
None of the time
Hardly any of the time
A little of the time
Some of the time
A good bit of the time
Most of the time
All of the time
102 NAs

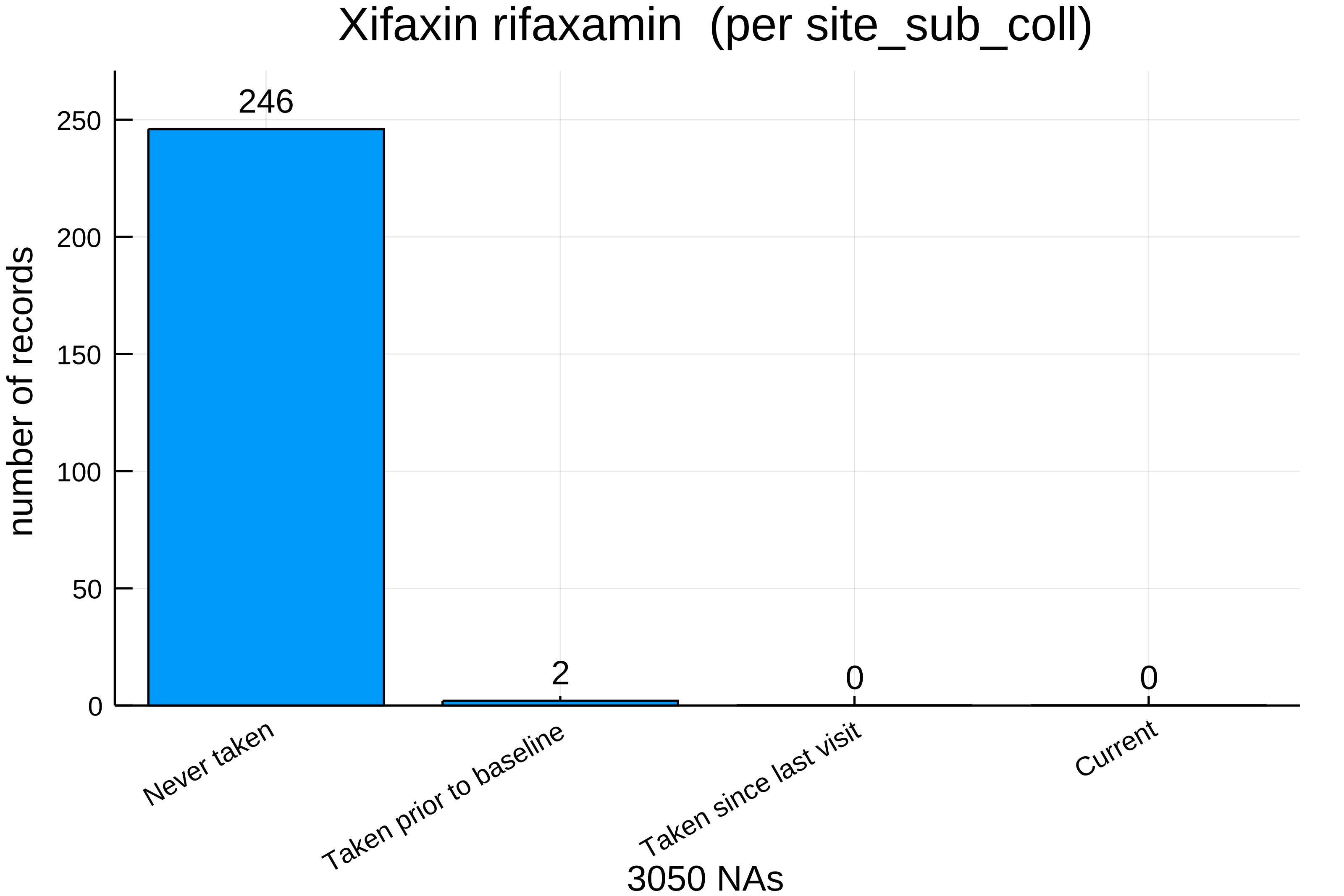
Xifaxin rifaxamin (per site_sub_coll)
number of records
250
200
150
100
50
0
246
2
0
0
Never taken
Taken prior to baseline
Taken since last visit
Current
3050 NAs

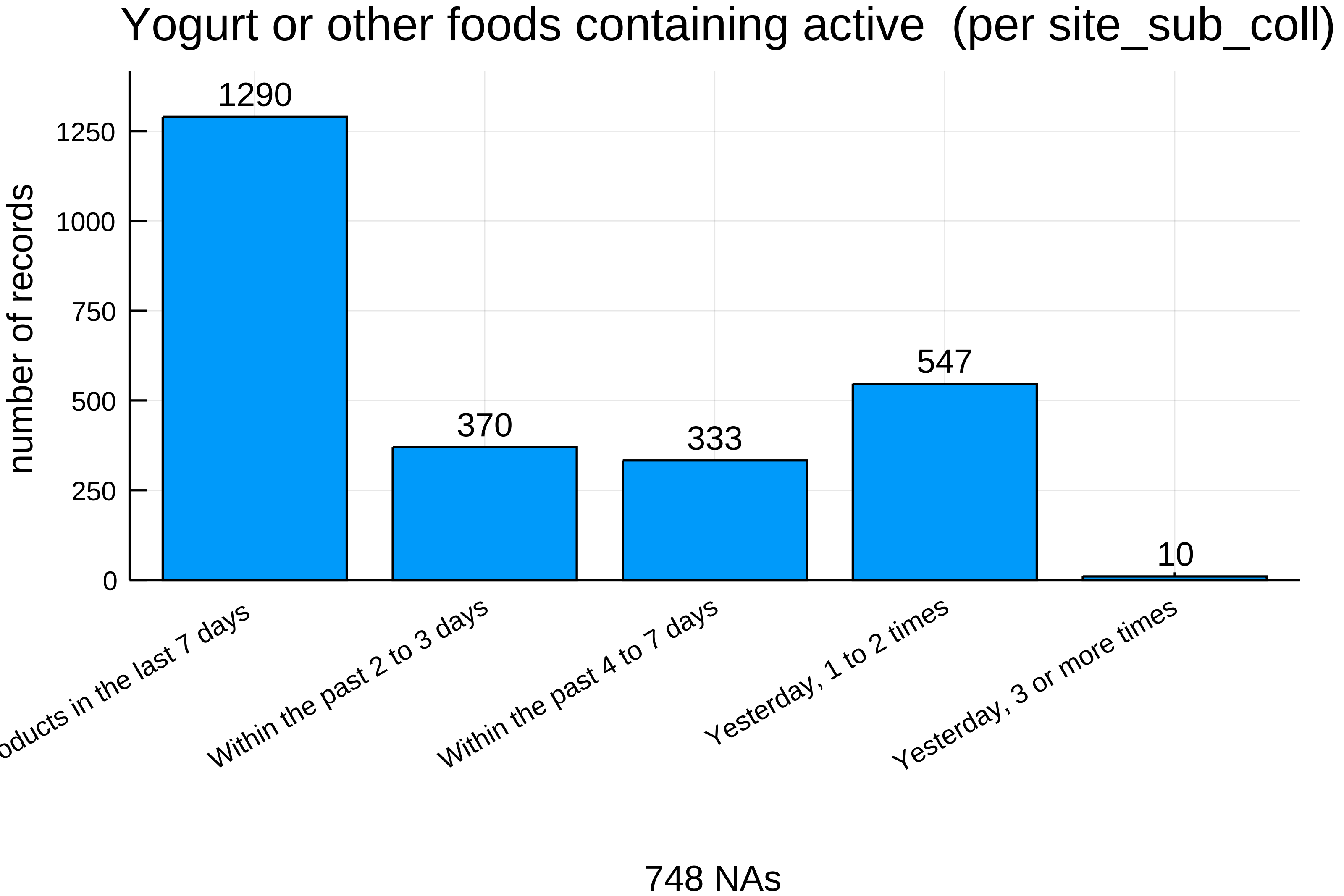
Yogurt or other foods containing active (per site_sub_coll)
number of records
1250
1000
750
500
250
0
1290
370
333
547
10
oducts in the last 7 days
Within the past 2 to 3 days
Within the past 4 to 7 days
Yesterday, 1 to 2 times
Yesterday, 3 or more times
748 NAs
