# Supplementary material for: Multi-omics of the gut microbial ecosystem in inflammatory bowel diseases
Source: Nature. 2019 May 29;569(7758):655–62. doi: 10.1038/s41586-019-1237-9 (PMC6650278; doi:10.1038/s41586-019-1237-9)
Supplement: Supplementary file 6 — This zipped folder contains Supplementary Tables 1-36 and legends. [file 41586_2019_1237_MOESM6_ESM.zip › 2018-01-01078D-s6/Legends for Supplementary Tables.docx]

**Legends for Supplementary Tables 1-36**

**Supplementary Tables 1-14: Associations between multi’omic features and IBD phenotypes.** List of all associations between disease phenotype (CD/UC/non-IBD) with multiple data feature types, using multivariate association testing with a linear mixed effects model (**Methods**). Features are sorted by minimum FDR-adjusted p-values between UC- and CD-specific comparisons (non-IBD as reference). For each feature, coefficient estimates and test statistics (CD vs. non-IBD and UC vs. non-IBD) and the associated two-tailed p-values are also reported. Feature spaces tested are metagenomic species relative abundances (**Supplementary Table 1**, N=1,595 samples from 130 subjects), metabolites (**Supplementary Table 2**, N=461 samples from 106 subjects), viruses (**Supplementary Table 3**, N=592 samples from 104 subjects), species-level transcription ratios (**Supplementary Table 4**, N=785 samples from 109 subjects), metagenomic pathway functional profiles (**Supplementary Table 5**, N=1,595 samples from 130 subjects), pathway transcription (**Supplementary Table 6**, N=785 samples from 109 subjects), pathway transcription ratios (**Supplementary Table 7**, N=785 samples from 109 subjects), metagenomic EC profiles (**Supplementary Table 8**, N=1,595 samples from 130 subjects), EC transcription (**Supplementary Table 9**, N=785 samples from 109 subjects), metagenomic KO profiles (**Supplementary Table 10**, N=1,595 samples from 130 subjects), KO transcription (**Supplementary Table 11**, N=785 samples from 109 subjects), protein abundances (**Supplementary Table 12**, N=449 samples from 89 subjects), EC-level protein abundances (**Supplementary Table 13**, N=449 samples from 89 subjects), and KO-level protein abundances (**Supplementary Table 14**, N=449 samples from 89 subjects).

**Supplementary Tables 15-28: Associations between multi’omic features and dysbiosis status.** As in **Supplementary Tables 1-14**, but associating multi’omic features with dysbiosis status rather than IBD phenotype, still using the same linear mixed effects model (**Methods**). Features are sorted by minimum FDR-adjusted p-values between UC-, CD-, and non-IBD-specific comparisons (non-dysbiotic state as reference in each disease group). For each feature, coefficient estimates, and test statistics (CD dysbiosis vs. CD non-dysbiosis, UC dysbiosis vs. UC non-dysbiosis, and non-IBD dysbiosis vs. non-IBD non-dysbiosis) and the associated two-tailed p-values are also reported. Feature spaces tested are metagenomic species relative abundances (**Supplementary Table 15**, N=1,595 samples from 130 subjects), metabolites (**Supplementary Table 16**, N=461 samples from 106 subjects), viruses (**Supplementary Table 17**, N=592 samples from 104 subjects), species-level transcription ratios (**Supplementary Table 18**, N=785 samples from 109 subjects), metagenomic pathway profiles (**Supplementary Table 19**, N=1,595 samples from 130 subjects), pathway transcription (**Supplementary Table 20**, N=785 samples from 109 subjects), pathway transcription ratios (**Supplementary Table 21**, N=785 samples from 109 subjects), metagenomic EC profiles (**Supplementary Table 22**, N=1,595 samples from 130 subjects), EC transcription (**Supplementary Table 23**, N=785 samples from 109 subjects), metagenomic KO profiles (**Supplementary Table 24**, N=1,595 samples from 130 subjects), KO transcription (**Supplementary Table 25**, N=785 samples from 109 subjects), protein abundances (**Supplementary Table 26**, N=449 samples from 89 subjects), EC-level protein abundances (**Supplementary Table 27**, N=449 samples from 89 subjects), and KO-level protein abundances (**Supplementary Table 28**, N=449 samples from 89 subjects).

**Supplementary Table 29: List and characteristics of shifts in metagenomic species abundances.** For each shift (definition in **Methods**), the Bray-Curtis dissimilarity between the two adjacent samples is given, along with the subject identifier, the collection number and number of weeks between the first stool collection for the subject and the second time point in the shift. The primary contributor is the feature with the largest absolute difference in abundance. Finally, the number of weeks left in the time series (weeks_left) is given, along with the change in dysbiosis state, with 0 indicating no change and 1/-1 indicating that the shift marked the entry/exit into/from dysbiosis, respectively.

**Supplementary Table 30: List and characteristics of shifts in metabolite abundances.** For each shift (definition in **Methods**), the Bray-Curtis dissimilarity between the two adjacent samples is given, along with the subject identifier and time point at which the shift occurred. The primary contributor (the feature with the largest absolute difference) is also given, with its abundance change and the number of weeks left in the time series (weeks_left). Finally, the change in dysbiosis state is given, with 0 indicating no change, and 1/-1 indicating that the shift marked the entry/exit into/from dysbiosis, respectively. Dysbiosis was defined here based on an approximate time point matching to the metagenomic profiles with up to 4 weeks lenience.

**Supplementary Table 31: Differentially expressed genes in host biopsies.** Significant differentially expressed genes, their logCPM (average expression represented as read count per million in log scale) and logFC (log fold change of expression between compared groups in log scale) are reported for comparisons between biopsies from CD and non-IBD subjects in ileum (Ileum.CD.Inf.vs.nonIBD comparisons), CD and UC subjects in ileum (Ileum.CD.Inf.vs.UC), CD and non-IBD subjects in rectum (Rectum.CD.Inf.vs.nonIBD), CD and UC subjects in rectum (Rectum.CD.Inf.vs.UC.Inf), and UC and non-IBD subjects in rectum (Rectum.UC.Inf.vs.nonIDB). Tests were performed separately for ileal (N=20, 23, 39 independent samples for non-IBD, UC, CD respectively) and rectal biopsies (N=22, 25, 41 independent samples for non-IBD, UC, CD respectively). Comparisons were limited to inflamed biopsies from CD subjects in both ileum and rectum, non-inflamed biopsies from UC subjects in ileum, and inflamed biopsies from UC subjects in rectum (quasi-likelihood negative binomial generalized log-linear model with Fisher's exact test adapted for overdispersed data, FDR p < 0.05 and log-fold change > 1.5).

**Supplementary Table 32: Enrichment analysis of differentially expressed genes in IBD.** Significantly enriched KEGG pathways (one-sided hypergeometric test, FDR p<0.05) among differentially expressed genes in comparisons between inflamed CD and UC biopsies and biopsies from non-IBD subjects. Tests were performed separately for ileal and rectal biopsies.

**Supplementary Table 33: Associations between host gene expression and OTU abundances from biopsies.** Significant associations between OTU relative abundance and host gene expression in biopsies from the ileum and rectum (partial spearman correlation with BMI, age at consent, sex and diagnosis as covariates, FDR p<0.05). Tests were performed for paired biopsy 16S and biopsy host expression data with ileal N=54 and rectal N=52. Only biopsies where the OTU was present (at least one read) were tested (given by column N).

**Supplementary Table 34: Genetic associations with metagenomic taxa.** For each known IBD locus, association results with microbe abundances are reported (mixed effect model with SNP used as predictor coded using additive genetic model, relative abundance of microbial taxa as outcome and age, sex, antibiotic and immunosuppressant use, and first 20 genetic principal components as covariates while specifying subjects as random effect). Only microbes with prevalence over 40% (>0 abundance) were tested. Effect allele frequencies (eaf) were calculated from IBDMDB data.

**Supplementary Tables 35-36: Cross-data type associations.** List of pairwise associations between features across data sets using HAllA^46^ (**Methods**). Datasets were first residualized using a linear mixed effect model with subjects as random effects and age, sex, diagnosis, dysbiosis status, antibiotic and immunosuppressant use, and bowel surgery status as covariates (or a simple linear model without the random effects when only baseline samples were used) to remove both within-subject correlations in the time series and spurious effect of covariates in the data (**Methods**; **Supplementary Table 35**). Associations were also tested between residuals of an unadjusted model without covariates (**Supplementary Table 36**). Appropriate normalization and/or transformation for each measurement type was performed independently before the model fitting (**Methods**). Datasets were then approximately matched with a maximum time difference between samples of 4 weeks (**Methods**). Paired datasets were compared using HAllA’s hierarchical testing procedure using Spearman correlation, and significant associations are reported (FDR p<0.05, serology associations are reported to FDR p<0.25). All pairs of the following datasets were tested: metagenomic species abundances (MGX|Species), metagenomics (summarized to ECs, MGX|EC), metatranscriptomes (summarized to ECs, MTX|EC, and to the species transcription ratios, MTX|Species), metabolomics (MBX), proteomics (summarized to the EC level, MPX|EC), fecal calprotectin (Calprotectin), and gene expression from ileal (HTX|Ileum) and rectal (HTX|Rectum) biopsies. Datasets were not tested against themselves. Sample counts in **Fig. 1B-C**. The following pairs were excluded from the list due to a clear mechanistic connection between the datasets: MGX|EC-MTX|EC, MTX|EC-MPX|EC, MGX|EC-MPX|EC, MGX|Species-MGX|EC.
